# Type of *in vitro* cultivation influences cytoadhesion, knob structure, protein localization and transcriptome profile of *Plasmodium falciparum*

**DOI:** 10.1038/srep16766

**Published:** 2015-11-16

**Authors:** Ann-Kathrin Tilly, Jenny Thiede, Nahla Metwally, Pedro Lubiana, Anna Bachmann, Thomas Roeder, Nichola Rockliffe, Stephan Lorenzen, Egbert Tannich, Thomas Gutsmann, Iris Bruchhaus

**Affiliations:** 1 Bernhard Nocht Institute for Tropical Medicine, Bernhard-Nocht-Str. 74, 20359 Hamburg, Germany; 2 Zoological Institute, Molecular Physiology, Christian-Albrechts University Kiel, Olshausenstraße 40, 24098 Kiel, Germany; 3 The Centre for Genomic Research, University of Liverpool, The Biosciences Building, Crown Street, Liverpool, L69 7ZB, UK; 4 Division of Biophysics, Research Center Borstel, Leibniz-Center for Medicine and Biosciences, Parkallee 10, 23845 Borstel, Germany

## Abstract

*In vitro* cultivation *of Plasmodium falciparum* is critical for studying the biology of this parasite. However, it is likely that different *in vitro* cultivation conditions influence various aspects of the parasite’s life cycle. In the present study two *P. falciparum* isolates were cultivated using the two most common methods, in which AlbuMAX or human serum as additives are used, and the results were compared. The type of cultivation influenced the knob structure of *P. falciparum*-infected erythrocytes (IEs). IEs cultivated with AlbuMAX had fewer knobs than those cultivated with human serum. Furthermore, knob size varied between isolates and is also depended on the culture medium. In addition, there was a greater reduction in the cytoadhesion of IEs to various endothelial receptors in the presence of AlbuMAX than in the presence of human serum. Surprisingly, cytoadhesion did not correlate with the presence or absence of knobs. Greater numbers of the variant surface antigen families RIFIN, STEVOR, and *Pf*MC-2TM were found at the IE membrane when cultivated in the presence of AlbuMAX. Moreover, the type of cultivation had a marked influence on the transcriptome profile. Compared with cultivation with human serum, cultivation with AlbuMAX increased the expression of approximately 500–870 genes.

The parasite *Plasmodium falciparum* causes the most virulent form of malaria in humans. About 200 million clinical cases were reported in 2013, resulting in about 600,000 deaths (WHO-2014). *P. falciparum* avoids spleen-dependent killing due to the adherence of infected erythrocytes (IEs) to human endothelial receptors that line microvascular venules^1^. This cytoadhesion or sequestration of IEs is the major underlying cause of the most severe pathological phenotypes of malaria, which include blood flow obstruction, hypoxia, tissue damage and, ultimately, organ failure^2^. In addition to physical adhesion, IEs may also induce intracellular signaling events, and endothelial cells can present parasite-derived antigens to immune cells; this is thought to be responsible for various pathophysiological mechanisms relevant to severe malaria^3^,^4^,^5^. The interaction of IEs with host endothelial receptors is mediated by binding of *P. falciparum*-derived proteins, which are exposed on the surface of IEs. The major adhesion molecules involved in these receptor-ligand interactions belong to the *P. falciparum* erythrocyte membrane protein 1 (*Pf*EMP1) family. *Pf*EMP1 proteins are encoded by about 60 different *va*r genes per parasite genome, although only a single *var* gene is expressed at a particular time. Moreover, *var* gene sequences vary greatly between isolates^6^,^7^,^8^. *Pf*EMP1 molecules are localized on the IE surface in characteristic knob-like, electron-dense protrusions^9^,^10^,^11^, which are formed by several parasite-encoded proteins exported to the IE membrane^12^,^13^. However, the relationship between the presence or density of these knobs and cytoadhesion of IE is not straightforward. Several studies show a positive correlation between cytoadherence and the presence of knobs^14^,^15^,^16^; however, a comprehensive study that analyzed 60 *ex vivo P. falciparum* isolates, all of which contained knobby IEs, found that 25 isolates also contained knobless IEs. This led to the hypothesis that both knobby and knobless phenotypes are present during natural infections^17^. Furthermore, *ex vivo*-isolated IEs had a higher knob density than erythrocytes infected by *in vitro*-cultured parasites^18^. However, there was a marked variation in the observed knob density between different *ex vivo* isolates, although it is not known whether this variation correlated with different binding characteristics^18^. In addition, low knob density on long-term *in vitro*-cultivated parasites^19^ and a complete loss of knobs after prolonged *in vitro* culture^20^ have been reported; however, some of the knobless isolates showed strong cytoadherence^16^,^21^. By contrast, other studies reveal that knobless isolates show reduced adhesion^22^,^23^. Thus, it is postulated that the reduced adhesion of knobless IEs depends on a reduction (by roughly 50%) in the amount of *Pf*EMP1 present on the IE surface^22^. The picture is further confused by the observation that the distribution of *Pf*EMP1 in knobless IEs seems to be comparable with that in knobby IEs. In knobless IEs, *Pf*EMP1 is localized within discrete spots on the erythrocyte surface^22^. Moreover, long-term cultivation led to reduced adhesion, although knob morphology did not change. Therefore, knobs are considered necessary but not sufficient for cytoadherence^14^.

The original protocol for the *in vitro* culture of *P. falciparum* was based on RPMI 1640 medium supplemented with human serum^24^. However, human serum has several disadvantages, such as the necessity for serum-erythrocyte compatibility, variations in growth-promoting factors, and high cost; therefore, several laboratories use serum-free medium supplemented with lipid-rich bovine serum (AlbuMAX)^25^. Supplementation with AlbuMAX improves growth profiles and has several additional advantages over human serum^26^. Nevertheless, it is unclear whether cultivating *P. falciparum* in AlbuMAX-supplemented medium has an effect on knob formation/size, cytoadhesion to human endothelial receptors, localization of variant surface antigens and, last but not least, the transcript profile (particularly that of *var* genes).

In the present study, we cultivated two *P. falciparum* isolates (3D7 and FCR3) either in the presence of AlbuMAX or human serum and subjected them to a comprehensive comparative analysis. For this, 1) the physical appearance of IEs was analysed by transmission electron microscopy and atomic force microscopy, 2) the cytoadhesion of IEs to seven different human endothelial receptors was investigated using transgenic CHO cells expressing the respective receptors on their surface, 3) the localization of members of the variant surface antigen families RIFIN, STEVOR and *Pf*MC-2TM was visualized by immunofluorescence microscopy using family specific, cross-reactive antibodies, and 4) the transcriptome of ring-stage FCR3 parasites was analyzed by mRNA sequencing.

## Results

### Physical appearance of the knobs

**Figure 1:**
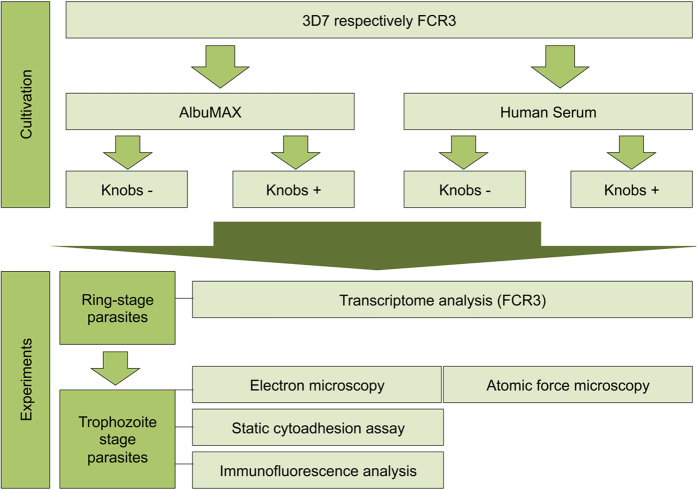
Flow chart summarizing the procedure of the comparative study of isolates 3D7 and FCR3 cultivated under different conditions.

**Figure 2:**
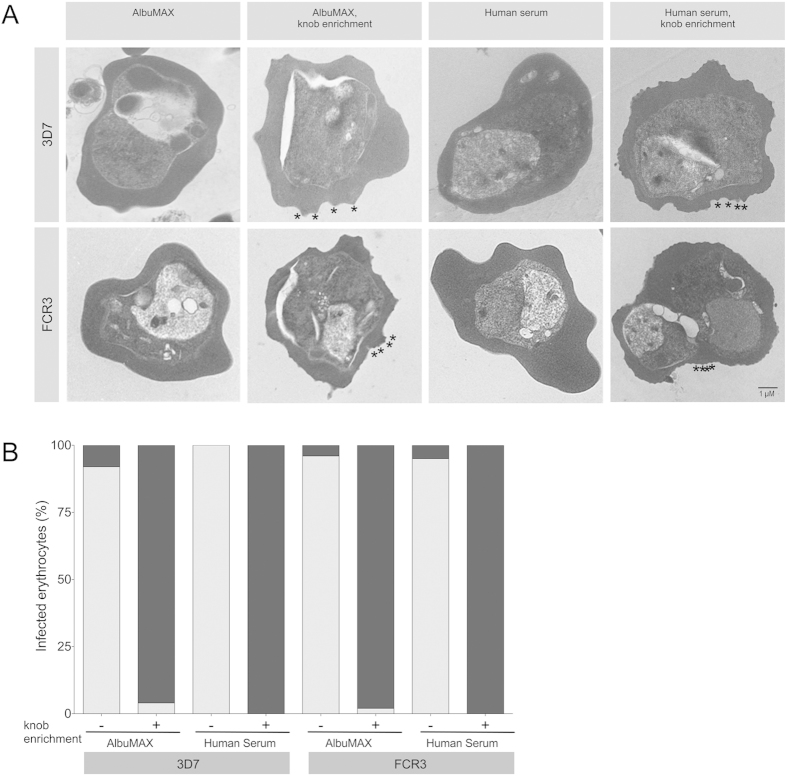
Transmission electron micrographs showing *P. falciparum* infected erythrocytes (IEs). The isolates 3D7 and FCR3 were cultivated in the presence of AlbuMAX (0.5%) or human serum (10%). In addition, isolates were subjected to gelatin sedimentation to enrich knobby IEs. A. Morphology was analyzed by transmission electron microscopy. For each type of cultivation one IE was shown as an example. Knobs are marked exemplarily by stars. B. Quantification of knobby IEs. Between 25 and 40 IEs per culture were analyzed for the presence of knobs. Gray column, knobless IEs; black column, knobby IEs.

Laboratory isolates 3D7 and FCR3 were cultivated in the presence of 0.5% AlbuMAX or 10% human serum for at least three months (Fig. 1). Electron microscopic examination of highly synchronized trophozoite-stage parasite cultures revealed that nearly all IEs infected with long-term cultivated parasites showed a smooth surface (between 92%–100% of the investigated IEs) (Fig. 2A,B). Nevertheless, it was possible to enrich knobby IEs to nearly 100% (between 96%–100% of the investigated IEs) after three rounds of gelatin sedimentation (Fig. 2A,B).

**Figure 3:**
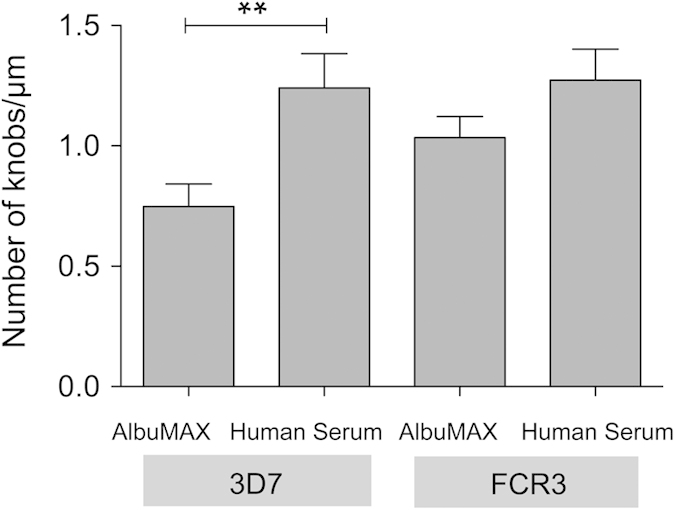
Determination of knob density on the surface of IEs infected by 3D7 and FCR3 cultivated in the presence of AlbuMAX or human serum. The number of knobs was calculated from electron microscopy images of isolates cultivated under different culture conditions after enrichment for knob carrying IEs. **p < 0.01.

We found it interesting that cultivation of isolate 3D7 in the presence of AlbuMAX rather than human serum resulted in fewer knobs on the surface of IEs. Fewer knobs were also observed on FCR3, but the difference between AlbuMAX- and human serum-containing media was not significant (Fig. 3).

**Figure 4:**
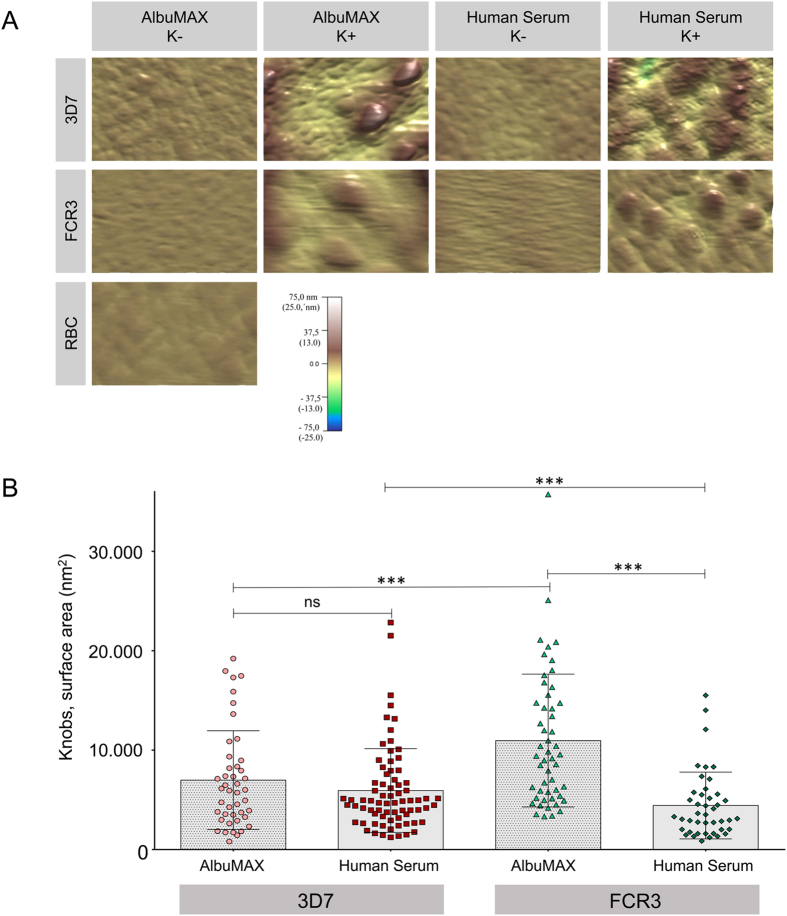
Atomic force microscopy of *P. falciparum* IEs. Isolates 3D7 and FCR3 were cultivated in the presence of AlbuMAX or human serum (K−). In addition, the isolates were enriched for the presence of knobs by gelatin sedimentation (K+). In each case, highly synchronized trophozoite-stage parasites (28–32 hpi) were examined. A. Scanning measurements from atomic force micrographs of 3D7 and FCR3 IEs, as well as uninfected erythrocytes (control). B. Surface area of the knobs on 3D7 and FCR3 IEs enriched for the presence of knobs. (*p ≤ 0.05, **p ≤ 0.01, ***p ≤ 0.001).

We next compared the physical appearance of knob-containing IEs in the presence of AlbuMAX or human serum using atomic force microscopy, which allows very high resolution imaging (Fig. 1, Fig. 4A). Although the knob density/μm for isolate 3D7 was lower in the presence of AlbuMAX than in the presence of human serum, there were no differences in the size measured as surface area of the knobs (Fig. 4B). However, when FCR3 was cultivated in the presence of AlbuMAX, the knobs varied greatly in terms of size. The smallest knobs covered a surface area of approximately 3,300 nm^2^, whereas the largest covered a surface area between 20,000 and 36,000 nm^2^. The size of the knobs on IEs cultivated in the presence of human serum was more uniform; in this case the knobs covered a surface area between 900 and 14,000 nm^2^, the average being about 8,000 nm^2^. Therefore, cultivating FCR3 IEs in the presence of human serum resulted in the formation of smaller, more uniform knobs when compared with cultivation in the presence of AlbuMAX (Fig. 4B, Table 1). The knobs on erythrocytes infected with isolate FCR3 were larger than those on cells infected with isolate 3D7 when cultivated in AlbuMAX. By contrast, the knobs of FCR3 IEs were smaller than those of 3D7 IEs when cultivated in human serum (Fig. 4B). When the height and width of the knobs were analyzed separately, it was clear that the knobs of 3D7 IEs cultivated with AlbuMAX were wider (13 ± 9 nm) than those of FCR3 IEs; however, those of FCR3 IEs were higher (115 ± 40 nm) (Tables 1 and 2). The dried samples were imaged in air. Thus, the measured heights appear lower as they appear in fixed EM sections. In summary, sizes of knobs differed between the two isolates 3D7 and FCR3. While for FCR3 the type of cultivation has a strong influence on knob sizes, the influence of cultivation on knob sizes in 3D7 was not significant.

### Binding capacity of IE cultivated under different culture conditions

To analyze whether the cultivation conditions and/or the presence of knobs influences the binding of IEs to various endothelial receptors, we performed a series of static binding experiments (Fig. 1). In these experiments, we examined the cytoadhesion of IEs to the human endothelial receptors CD36, P-selectin, E-selectin, ICAM-1, CD9, CD151, and MDR1.

**Figure 5:**
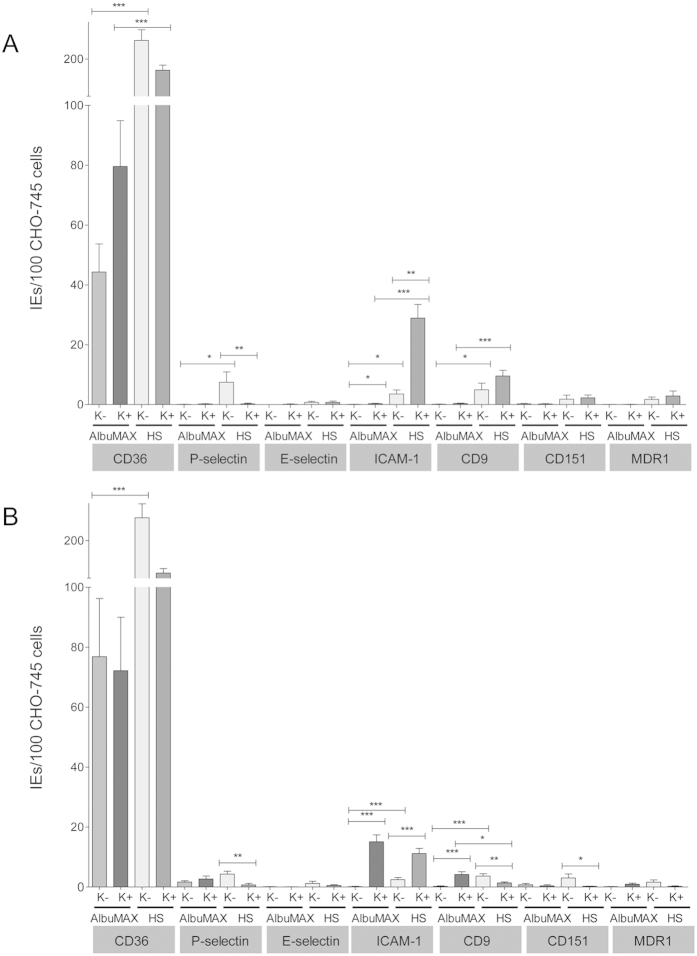
Binding of *P. falciparum* IEs to endothelial receptors expressed on CHO-745 cells. Binding of erythrocytes infected with isolate 3D7 (A) or isolate FCR3 (B) to CHO-745 cells expressing CD36, P-selectin, E-selectin, ICAM-1, CD9, CD151, or MDR1. Bars indicate the median number of IEs specifically bound to 100 CHO cells (as determined by microscopic inspection of 500 CHO cells in three independent experiments, each performed in triplicate). K−, cultivation in the presence of AlbuMAX or human serum; K+, cultivation in the presence of AlbuMAX or human serum plus gelatin sedimentation to enrich knobby IEs. (*p ≥ 0.05, **p ≤ 0.01, ***p ≤ 0.001).

The results showed that the culture conditions did influence the binding of IEs to endothelial receptors. 3D7 IEs cultivated in the presence of AlbuMAX only interacted with CD36. Only negligible binding to the other receptors was observed. Also, the presence of knobs did not increase the binding capacity. However, the presence of human serum clearly increased the amount of IEs that bound to the different receptors. In addition, the knobs had a detectable influence on binding capacity. Binding to ICAM-1 increased significantly when IEs enriched for the presence of knobs were used in the experiment (AlbuMAX cultivated: K− vs K+ *p* < 0.03; HS cultivated K- vs K+ *p* < 0.002). By contrast, a significant increase in binding to P-selectin was observed for knobless IEs. IEs with or without knobs bound to CD36, E-selectin, CD9, CD151, and MDR1 to similar extents (Fig. 5A, Table 1).

The effect of the culture medium was much smaller for isolate FCR3 than for isolate 3D7. Although FCR3 IEs bound to most of the receptors when cultivated in the presence of AlbuMAX or human serum, the combination of culture medium and the presence or absence of knobs correlated with binding to individual receptors. Knobby IEs showed stronger binding to CD9 when cultivated in the presence of AlbuMAX. By contrast, more knobless IEs bound to this receptor when cultivated in the presence of human serum. As observed for 3D7, the ICAM-1 binding capacity of FCR3 IEs increased markedly when knobby IEs were used. Binding of knobless IEs to P-selectin and CD151 was significantly improved when the parasites were cultivated in the presence of human serum (Fig. 5B, Table 1). Taken together, the results show that parasites cultivated in the presence of human serum show enhanced binding to the human endothelial receptors. Moreover, no direct correlation was observed between cytoadhesion and the presence of knobs. For example, knobby IEs bind better to ICAM-1 in comparison to knobless IEs, whereas knobless IEs bind better to P-selectin in comparison to knobby IEs (Table 1).

### Localization of members of the small variant surface antigen families RIFIN, STEVOR, and *Pf*MC-2TM

**Figure 6:**
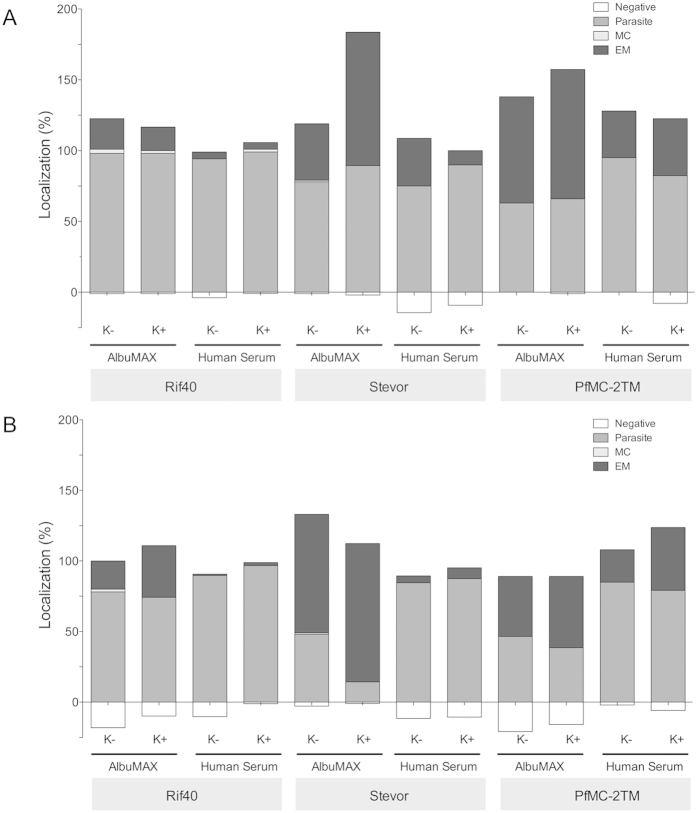
Localization of small VSA within infected erythrocytes. The localization of RIFIN, STEVOR, and *Pf*MC-2TM proteins was quantified in trophozoite-stage parasites (28-32 hpi). Shown are percentages of proteins inside the parasitic boundaries (parasite, dark grey), associated within the Maurer’s clefts (MC, light grey), and at the erythrocyte membrane (E, black) as well as without specific fluorescence signal (negative, white). One hundred IEs were counted for each staining; however, the overall percentage is greater than 100 because some proteins localized to multiple sites within a single cell. A. Immunofluorescence microscopy of 3D7 IEs. B. Immunofluorescence microscopy of FCR3 IEs. K-, cultivation in the presence of AlbuMAX or human serum; K+, cultivation in the presence of AlbuMAX or human serum plus gelatin sedimentation to enrich knobby IEs. HS, human serum.

We next investigated whether cultivation in the presence of AlbuMAX or human serum, as well as the presence or absence of knobs, influenced the localization of the small variant surface antigen families RIFIN, STEVOR, and *Pf*MC-2TM (Fig. 1). The localization studies were based on immunofluorescence microscopy using three different antisera (*a*-RIF40 (RIFIN), *a*-PFC0025c (STEVOR), and *a-Pf*MC2TM-CT (*Pf*MC-2TM)). The three antisera were selected, because it is known that they are cross-reactive recognizing semi-conserved portions of RIFIN, STEVOR or *Pf*MC-2TM respectively in the isolates 3D7 and FCR3^27^. For RIFIN, between 75% and 100% of the 3D7 and FCR3 parasites within the IEs were stained. Occasionally RIFIN was detected in the Maurer’s clefts. Staining of the erythrocyte membrane was detected more frequently when the parasites were cultivated in the presence of AlbuMAX (17%–37%) rather than in human serum (1%–5%). The presence or absence of knobs had no influence on localization (Fig. 6A,B, Table 1). The localization pattern of STEVOR was not as uniform as that of RIFIN. Between 75% and 89% of 3D7 parasites within IEs were stained, with little staining of Maurer’s clefts observed. In addition, 40% of knobless IEs cultivated in the presence of AlbuMAX showed staining of the erythrocyte membrane. This increased 94% if the IEs were enriched for the presence of knobs. Cultivation with human serum yielded contrasting results. Knobless IEs showed 34% erythrocyte membrane localization with anti-STEVOR serum, whereas only 10% of knobby IEs exported STEVOR to the host cell membrane. When FCR3 were cultivated in the presence of AlbuMAX, STEVOR antiserum stained the parasites in 48% of knobless and 14% of knobby IEs; however, 84% of knobless IEs and 98% of knobby IEs showed staining of the erythrocyte membrane. Host cell membrane staining was markedly reduced in the presence of human serum (5% of knobless and 8% of knobby IEs), whereas approximately 85% of the parasites within knobless and knobby IEs were stained (Fig. 6A,B, Table 1).

The number of 3D7 parasites stained with anti-*Pf*MC-2TM was higher in the presence of human serum (95% of knobless IEs and 82% of knobby IEs, respectively) than in the presence of AlbuMAX (63% of knobless IEs and 66% of knobby IEs). Nevertheless, 75% of knobless IEs and 91% of knobby IEs showed staining of the erythrocyte membrane when cultivated with AlbuMAX, whereas cultivation in the presence of human serum revealed that 33% of knobless IEs and 44% knobby IEs expressed *Pf*MC-2TM at the membrane (Fig. 6A, Table 1).

In case of FCR3 cultivated in the presence of AlbuMAX, 46% of the knobless IEs and 38% of the knobby IEs showed a staining of the parasites, and about 38% to 46% of the parasites as well as 43% of knobless IEs and 50% of knobby IEs showed a staining at the erythrocyte membrane using the *Pf*MC-2TM antiserum. Membrane staining was less prevalent in the presence of human serum (23% of knobless IEs and 45% of knobby IEs), although the number of stained parasites was higher (approximately 80% in both knobless and knobby IEs) than that after cultivation in the presence of AlbuMAX (Fig. 6B, Table 1). Therefore, cultivation in the presence of AlbuMAX apparently recruits more members of the RIFIN, STEVOR and *Pf*MC2-TM family to the erythrocyte membrane than cultivation with human serum does. Only for localization of *Pf*MC2-TM members in the FCR3 strain no differences were observed. In addition, no correlation between the presence of knobs and the localization of these protein family members at the erythrocyte membrane could be detected (Table 1).

### Transcriptomes of ring-stage FCR3 parasites after cultivation under different conditions

To examine whether *P. falciparum* parasites show different gene expression profiles when grown in medium supplemented with human serum or AlbuMAX, we performed mRNA sequencing using a next generation sequencing approach (Fig. 1). Since most of the genes for the proteins involved in knob formation or cytoadhesion are transcribed at early points in the asexual cycle, we examined highly synchronized ring-stage parasites (6 to 8 hours post invasion (hpi). For the purpose of this study, differential expression was defined as a threshold of ≥ 3 fold and a p-value adjusted (padj) of  < 0.05.

**Figure 7:**
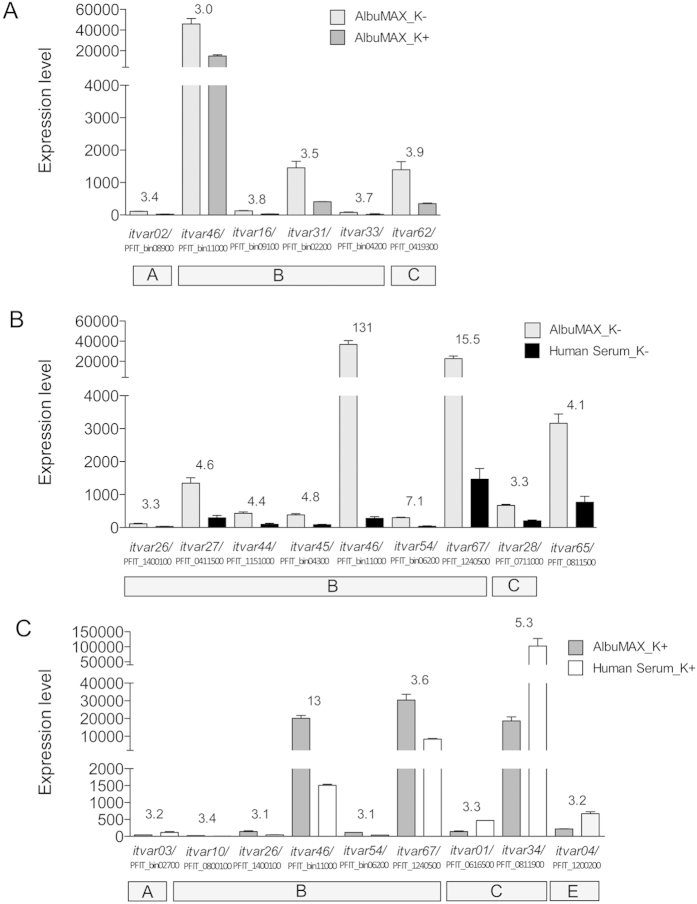
Expression of *var* genes differentially expressed by isolate FCR3 under different culture conditions. (**A**) Differential expression of *var* genes by FCR3 parasites in knobby (K+) and knobless (K−) IEs cultivated in the presence of AlbuMAX. (**B**) Differential expression of *var* genes by FCR3 parasites in knobless IEs cultivated in the presence of AlbuMAX or human serum. (**C**) Differential expression of *var* genes by FCR3 parasites in knobby IEs cultivated in the presence of AlbuMAX or human serum. Numbers above columns indicate x-fold expression with padj of ≤0.05.

There were no significant differences in the transcriptome profiles of ring-stage parasites in knobless and knobby IEs when cultivated in the presence of human serum (Table 1, Table S1). In addition, only minor differences were observed when knobby and knobless IEs were cultivated in the presence of AlbuMAX. In the latter case, the expression of 28 genes was upregulated and that of eight genes was downregulated in knobless IEs compared with knobby IEs. In the majority of cases, the degree of differential expression was between 3- and 6-fold. The 28 genes that were upregulated included six *var* genes; no *var* genes were downregulated (Fig. 7A, Table 1, Table S2).

Nevertheless, major differences were observed if the expression profiles of parasites cultivated in the two different media were compared: 873 genes in parasites within knobby IEs and 496 genes in parasites within knobless IEs showed ≥3-fold higher expression when cultivated with AlbuMAX compared with human serum (Table 1). We observed an overlap of 420 upregulated genes between parasites in knobby IEs and knobless IEs (Table S3 and S4). To characterize the differentially expressed genes, we performed gene ontology (GO) assignments to elucidate the functional categories. This analysis was based on the three main GO categories: biological process (BP), cellular component (CC), and molecular function (MF). In the BP category, the most prominent terms were metal ion transport, signaling, DNA catabolic processes, protein phosphorylation, and related GO terms. In the CC category, the most prominent terms were cAMP-dependent protein kinases, actin cytoskeleton components, protein-DNA complexes, and related GO terms. Upregulated molecular functions included divalent inorganic transmembrane transport activity, protein kinase activity, oxidoreductase activity, cyclic nucleotide phosphodiesterase activity, and related GO terms (Tables S5–S13). By contrast, only 24 genes isolated from parasites within knobby IEs, and eight genes isolated from parasites within knobless IEs, were expressed at higher levels in the presence of human serum; in this case, there was an overlap of six genes (Table 1, Tables S3 and S4). The majority of these proteins were *P. falciparum* exported proteins of unknown function, or genes belonging to the PHIST protein family, which is implicated in alterations to the IE membrane and *Pf*EMP1 transport^28^,^29^.

The gene that showed the greatest increase in expression in the presence of AlbuMAX was the *var* gene, *itvar46*, which encodes a member of the *Pf*EMP1 family. A 131-fold increase was observed in parasites within knobless IEs and a 13-fold increase in parasites within knobby IEs. Furthermore, *itvar46* was three fold higher expressed in knobless FCR3-IEs in comparison to knobby FCR3-IEs both cultivated in AlbuMAX (Fig. 7A–C). Two other genes, *itvar67* (in both knobless and knobby IEs) and *itvar65* (only in knobless IEs), were expressed at very high levels in parasites cultivated in the presence of AlbuMAX. In addition, we observed significant differences in the levels of expression of a few more *var* genes, but all were expressed at low levels (Fig. 7A–C). An increase in *var* gene expression was observed only in knobby IEs cultivated in the presence of human serum. Here, three *var* genes showed differential expression, whereby *itvar34* showed the highest expression (Fig. 7C). Taken together, the expression of several hundred genes was increased in FCR3 in response to cultivation in the presence of AlbuMAX in comparison to cultivation in human serum. Among these differentially expressed genes were also some *var* genes. Nevertheless, no correlation between the presence or absence of knobs and the *var* genes expression pattern could be observed. In addition, the transcriptomic study could not decipher which *var* gene is responsible for specific binding to the human endothelial receptors investigated in the present study (Table 1).

## Discussion

Cultivating *P. falciparum* in the presence of AlbuMAX or human serum revealed marked differences in knob structure, surface localization of variant surface antigens, cytoadhesion to endothelial receptors, and transcriptome profiles, indicating that the type of cultivation is of great and previously underestimated importance for various aspects of the parasite’s biology. Previous reports show that *in vitro*-cultivated parasites lose their knobs during long-term culture^19^,^20^; this was observed for isolates 3D7 and FCR3 in the present study. Nevertheless, knobby IEs were easily enriched by gelatin sedimentation, regardless of whether the parasites were cultivated in the presence of AlbuMAX or human serum.

We could show, that the size of the knobs differs between the two isolates 3D7 and FCR3. It was already described by Quadt and colleagues that the sizes of knobs varied significantly between individual isolates^18^. However, while the type of cultivation has a strong influence on the size of the knobs of FCR3, the influence on 3D7 was not significant.

A major finding of the present study is that the amount of IEs that bind specifically to endothelial receptors depends on the receptor type, the isolate, the presence or absence of knobs, and the culture medium. We found that both isolates bound most strongly to CD36, which is in agreement with earlier studies that report exceptionally strong binding of IEs to this receptor^30^,^31^. Binding to CD36 was independent of the presence of knobs, but was significantly reduced if the parasites were cultivated in the presence of AlbuMAX rather than human serum. Furthermore, 3D7 IE showed almost no binding to the receptors investigated when cultivated in the presence of AlbuMAX, but did bind to these receptors in the presence of human serum. In concordance with our data and as shown previously, IEs show reduced adhesion to CD36 and ICAM-1 when cultivated in the presence of AlbuMAX rather than human serum^32^. This correlates nicely with the presentation of *Pf*EMP1 on the IE membrane. The presentation of *Pf*EMP1 depends on the presence of human serum in the culture medium; much less *Pf*EMP1 is found on the IE membrane in the presence of AlbuMAX^32^. This may be due to a defective *Pf*EMP1 membrane transfer or to an altered surface conformation rather than to switching or downregulation of the respective *var* genes^32^. Our results support this hypothesis. Although some *var* genes were expressed at higher levels in AlbuMAX-cultivated IEs, they showed a lower binding to endothelial receptors than IEs cultivated in human serum. ICAM-1 showed higher binding of 3D7 IEs if knobby IEs were used, whereas we observed higher binding of knobless IEs to P-selectin. Binding to CD9, CD151, and MDR1 was independent of the presence of knobs. For the isolate FCR3, binding of IEs to the various receptors was observed if the parasites were cultivated in the presence of AlbuMAX. As observed for 3D7, more knobby IEs bound to ICAM-1 regardless of the cultivation method, whereas more knobless IEs bound to P-selectin when cultivated in the presence of human serum. Based on these results, it can be assumed that knobs are not essential for receptor binding (with the exception of ICAM-1) under static conditions. Exactly as Crabb and colleagues, we found no significant differences in cytoadhesion of knobby or knobless IEs to CD36 under static conditions; however, the adhesion of knobless IEs was significantly reduced under flow conditions^33^. By contrast, Horrocks and colleagues showed that the adhesion of knobless IEs is reduced under both static and flow conditions. They were also able to show that this reduced cytoadherence correlated with a 50% reduction in the amount of *Pf*EMP1 localized on the surface of knobless IEs^22^. Currently, we are not able to explain these diverse results that might come from subtle differences in the cultivation regimes or from the type of selection of knobless parasites.

Surprisingly, when we compared the gene expression profiles of parasites cultivated in AlbuMAX with those of parasites cultivated in human serum, we found that several hundred genes were differentially regulated. This observation is in strong contrast to those reported by Singh and colleagues, who identified only minor differences in gene expression profiles between parasites cultivated in AlbuMAX and those cultivated in human serum^26^. However, results from the present study cannot directly be compared with those of Singh and colleagues, because they studied trophozoite-stage parasites^26^; whereas we examined ring-stage parasites. It has to be kept in mind that for analysis of the protein composition required for binding in the trophozoite state, transcript in the ring-state are decisive, making this developmental stage much more important for this type of analysis than the trophozoite stage. Furthermore, nearly all differentially expressed genes were upregulated in AlbuMAX-cultivated parasites, regardless of the presence of knobs. It may also be of interest to mention that the majority of upregulated genes encoded proteins are mainly involved in metal ion transport and DNA metabolism.

Another difference between IEs cultivated in the presence of AlbuMAX or human serum is the increased export of RIFIN, STEVOR, and *Pf*MC-2TM family proteins to the erythrocyte membrane. We cannot rule out the possibility that human serum proteins coat the surface of IEs; therefore, RIFIN, STEVOR, and PfMC2-TM proteins were not detectable using the specific antibodies used in our experiments.

In summary, we did not observe a correlation between the presence and absence of knobs or gene expression profiles with cytoadhesion to various human endothelial receptors (at least under static conditions). Nevertheless, it is important to mention that cultivation in the presence of AlbuMAX or human serum induced marked differences in protein export and gene expression, but also on knob structure. Therefore, the cultivation medium used in a study, and whether or not IEs should be enriched for knobs, depends on the research question being asked.

## Methods

### Parasite culture

*P. falciparum* clones 3D7 and FCR3S1.2 were cultivated in human 0+ erythrocytes at a hematocrit of 5% in the presence of 10% human serum or 0.5% AlbuMAX II (Life Technologies), according to standard procedures^24^. Parasite growth was synchronized using 5% sorbitol^34^. Gelatin sedimentation assays were performed to enrich knobby IEs; assays were performed in RPMI 1640 medium containing 3% Gelafundin® (B. Braun Melsungen AG, Germany). Flotation was conducted as previously described^35^. Originally isolates 3D7 and FCR3 were cultivated in the presence of AlbuMAX. Three months before gelatin enrichment and transmission electron microscopy, the cells were transferred to culture medium supplemented with human serum. All other experiments (atomic force microscopy, static cytoadhesion assay, localization studies, and transcriptome analysis) were started at least six months after continuously cultivation in the presence of human serumor AlbuMAX. Knobs were continuously enriched every second week.

### Electron microscopy

Highly synchronized parasites at the trophozoite-stage were enriched by MACS (Magnet Activated Cell Sorting; Miltenyi Biotech) and pre-fixed in 2% glutaraldehyde in 0.1 M sodium-cacodylate buffer, pH 7.2. The pre-fixed cells were resuspended in liquid agar (2%), cut into small pieces, post-fixed in 2% osmium tetroxide, contrasted with 2% uranylacetate, dehydrated, and impregnated with a 50% propylene oxide/50% Epon (epoxy resin) mixture. After embedding with Epon, thin sections (80–100 nm) were cut and post-stained with lead citrate before examination in a Leo 910 transmission electron microscope (Zeiss, Oberkocken, Germany). The knobs of at least 20 IEs containing each of the *P. falciparum* isolates were counted at 10,000 × magnification.

### Atomic force microscopy

Highly synchronized parasites at the trophozoite-stage (28–32 hpi) were enriched by MACS (Miltenyi Biotech) and added to 0.1% glutaraldehyde/3% formaldehyde in phosphate-buffered saline (PBS; pH = 7.4) in a mixing ratio 1/15. After 4 h fixation time, the IEs were washed with PBS. Briefly, 4 μl of the fixed IE suspension were deposited onto an ethanol cleaned glass slide and treated as previously described^36^. At least ten single IEs containing each *P. falciparum* isolate were measured with a Molecular Force Probe (MFP)-3D AFM (Asylum Research Oxford Instruments) using an Au-coated CSG11 (NT-MDT) cantilever (spring constant of 0.03 N/m, resonance frequency in air of 10 kHz), contact mode, and scan rates of 0.5–1.0 kHz (depending on the scan size; 0.5–10.0 μm). All scanning measurements were recorded and analyzed using the MFP-3D software (Asylum Research) based on Igor Pro 6.0 (Wavemetrix). The surface area of each knob (nm^2^) was calculated using following formula: π * (width^2^ + height^2^). The resulting data are shown as single dots (median value ± standard deviation).

### Static cytoadhesion assay

Stably transfected Chinese hamster ovary (CHO) cells defective in glycosaminoglycan biosynthesis (CHO-745; American Type Culture Collection No. CRL-2242) were used for the cytoadhesion assays. The transfectants expressed human CD36, ICAM-1, E-Selectin, P-selectin, CD9, CD151, or MDR1 fused to GFP on the cell membrane. The transfectants were kindly provided by Rolf Horstmann (Bernhard Nocht Institute for Tropical Medicine, Hamburg) and generated as previously described^37^,^38^.

The static cytoadhesion assays were performed as previously described^37^,^38^, with some modifications. Briefly, the transfectants were grown in Ham’s F-12 medium (PAN Biotech) supplemented with 10% fetal calf serum and penicillin-streptomycin; selection was achieved by addition of G418 (0.7 mg/ml; Biochrom AG). CHO-745 cells expressing CD36, ICAM-1, P-selectin, E-selectin, CD151, or MDR1, as well as control CHO-745 cells expressing GFP alone, were seeded onto coverslips (13 mm) at a density of 30,000 cells per ml and maintained in 24-well plates for 48 h. Trophozoite-stage parasites cultured (parasitemia, 5%; hematocrit, 1%) in binding medium (RPMI 1640 medium supplemented with 2% glucose, pH 7.2) were firstly added to GFP-expressing CHO-745 cells for 1 h at 37 °C with gentle agitation at 15 min intervals to allow pre-absorption. Afterwards, the pre-absorbed parasite suspension was incubated with receptor-expressing CHO-745 cells for 1 h at 37 °C with gentle agitation at 15 min intervals. Unbound erythrocytes were gently removed by washing with binding medium. The cells adhered to the coverslips were fixed with 1% glutaraldehyde in PBS for 30 min at room temperature. Finally the cells were stained with Giemsa solution, and the number of adherent IEs was determined by counting 500 CHO-745 under a light microscope. All cytoadhesion assays were conducted three times in triplicate. The number of IEs adhered to the GFP-expressing CHO-745 control was subtracted from the number of IEs adhered to endothelial receptor-expressing CHO-745 cells. Negative values were presented as zero. Data were expressed as the median value and standard deviation.

### Immunofluorescence analysis

Blood smears were air-dried and fixed in 100% methanol at −20 °C for 5 min. After rehydrating in PBS for 10 min, the slides were incubated with rat α-RIF40.2 (AF483820, AA35-215; 1:300), mouse α-*Pf*MC-2TM-CT (PFF1525c/PF3D7_0631400, AA54-159; 1:200), or rabbit α-PFC0025c (PF3D7_0300400, AA33-251; 1:200)^27^,^39^ for 2 h at room temperature. All antibodies were diluted in PBS/1% BSA. After washing three times with PBS, the slides were incubated with Alexa Fluor488- or Alexa Fluor594-conjugated α-mouse, α-rat, or α-rabbit, secondary antibodies (1:400; Invitrogen) and Hoechst-33342 (1:500; Life Technologies). After repeated washing in PBS, the slides were mounted with MOWIOL 4-88 (Calbiochem) and then viewed under a 100× oil immersion lens fitted to a UV-equipped Nikon Eclipse TS100 microscope and an Olympus FV1000 FL confocal microscope. Data were analyzed with FLUOVIEW software v4.1a. The proportion of positively-stained cells and the localization of variant surface antigen (VSA) were quantified by counting at least 100 IEs. No fluorescence signals were observed in the control group (stained with secondary antibody alone and pre-immune sera).

### RNA purification, Illumina library preparation for transcriptome analysis, and bioinformatics

Ring-stage parasites were harvested, rapidly lysed in a 20-fold volume of pre-warmed (37 °C) TRIzol® (Invitrogen), and stored at −80 °C. RNA was isolated using the PureLink^TM^ RNA Mini Kit (Ambion), according to the manufacturer’s instructions. For the present study, transcriptome libraries were constructed by the Centre of Genomic Research, University of Liverpool, UK. Briefly, RNA quantity and integrity were assessed using the Qubit RNA BR kit (Life Technologies) and the Bioanalyzer RNA Pico kit (Agilent). Samples were DNase treated using the TURBO DNA-free™ Kit (Ambion, Life Technologies), followed by enzyme removal and purification using Agencourt RNAClean XP beads (Beckman). After quality control, the rRNA was depleted using the RiboZero Magnetic Gold kit (Human/Mouse/Rat; Epicentre), according to the manufacturer’s protocol. Afterwards, RNA-Seq libraries were generated using the ScriptSeq v2 kit (Epicentre). The individual libraries were assessed using a Qubit dsDNA high sensitivity kit and Bioanalyzer DNA HS chips to ascertain the concentration and fragment size distribution, respectively, prior to multiplexing the libraries into three pools of 6–7 libraries. The multiplexed libraries were subjected to quality control by Qubit and Bioanalyzer as for individual libraries, before final qPCR assessment, using an Illumina Library Quantification Kit (KK4854; KAPA) on a Roche Light Cycler LC480II according to manufacturer’s instructions, to obtain the concentration with the Illumina adaptors added. In preparation for sequencing, template DNA was denatured according to the protocol described in the Illumina cBot User guide. The libraries were paired-end sequenced (2 × 100 bp read length) using Illumina HiSeq 2000.

Reads were aligned to the *P. falciparum* transcriptome using bwa^40^ version 0.7.10 and differential expression was analyzed using DESeq^41^ version 1.18. Gene ontology^42^ terms were obtained from PlasmoDB^43^ version 24 and parsed by an in-house Perl script.

### Statistical analysis

The non-parametric Mann-Whitney U test was used for statistical evaluation of the electron microscopy and atomic force microscopy data, as well as the static cytoadhesion assay, whereas two-tailed p-values ≤ 0.05 were considered significant. For statistical evaluation of the transcriptome the statistics of GO terms were calculated using the Gostats^44^ package version 2.32 for the R statistical programming environment (http://www.R-project.org/).

## Additional Information

**How to cite this article**: Tilly, A.-K. *et al.* Type of *in vitro* cultivation influences cytoadhesion, knob structure, protein localization and transcriptome profile of *Plasmodium falciparum*. *Sci. Rep.* **5**, 16766; doi: 10.1038/srep16766 (2015).

## Supplementary Material

Supplementary Information

## Figures and Tables

**Table 1:** Summary of the results of the comparative study of isolates 3D7 and FCR3 cultivated under different conditions.

	Isolate 3D7				Isolate FCR3			
	AlbuMAX		Human Serum		AlbuMAX		Human Serum	
	K–*	K+†	K–	K+	K–	K+	K–	K+
Number of knobs/μm	—	0.75 ± 0.09	—	1.24 ± 0.14	—	1.03 ± 0.09	—	1.27 ± 0.13
Knobs surface area (nm^2^)	—	6986 ± 4970	—	5940 ± 4211	—	10961 ± 6681	—	4433 ± 3356
Diameter/height of knobs (nm)	—	87 ± 38/13 ± 9	—	82 ± 27/4 ± 3	—	115 ± 40	—	7 ± 5
Cytoadhesion (IE/100 CHO cells)								
CD36	44.3 ± 9.4	79.6 ± 15.3	250 ± 28.5	170 ± 13.9	76.9 ± 19.4	72.2 ± 17.8	261.2 ± 37.2	113.7 ± 12
P-selectin	0	0.2 ± 0.1	7.5 ± 3.4	0.2 ± 0.2	1.7 ± 0.4	2.7 ± 1	4.3 ± 1	0.7 ± 0.5
E-selectin	0	0.1 ± 0.1	0.7 ± 0.4	0.7 ± 0.4	0	0	1.2 ± 0.7	0.5 ± 0.3
ICAM-1	0	0.3 ± 0.1	3.5 ± 1.3	28.9 ± 4.5	0.1 ± 0.1	15 ± 2.3	2.5 ± 0.7	11 ± 1.7
CD9	0.1 ± 0.1	0.4 ± 0.1	5 ± 2.2	10 ± 2.2	0.2 ± 0.2	4.2 ± 1	3.7 ± 0.8	1.4 ± 0.3
CD151	0.3 ± 0.1	0.2 ± 0.1	1.8 ± 1.4	2.3 ± 0.9	0.8 ± 0.4	0.4 ± 0.3	3 ± 1.3	0
MDR1	0	0	1.7 ± 0.8	2.9 ± 1.6	0.1 ± 0.1	0.9 ± 0.4	1.6 ± 0.8	0.2 ± 0.2
Localization (%), Negative/Parasit/MC/EM‡								
RIFIN	1/98/3/22	1/98/2/17	4/94/0/5	1/99/2/5	18/78/2/20	10/74/0/37	10/90/0/1	1/97/0/2
STEVOR	1/78/1/40	2/89/0/94	14/75/0/34	9/89/0/10	3/48/1/84	1/14/0/98	11/85/0/5	11/88/0/8
*Pf*MC-2TM	0/63/0/75	1/66/0/91	0/95/0/33	8/82/0/40	21/47/0/43	16/39/0/51	2/85/0/23	6/80/0/45
Differential gene expression (threshold ≥3 fold, padj <0.05)					K− > K+ = 28 genesK− < K+ = 8 genes		No differential expression	
					AlbuMAX_K− > Human Serum_K− = 873AlbuMAX_K+ > Human Serum_K+ = 496AlbuMAX_K− < Human Serum_K− = 8AlbuMAX_K+ < Human Serum_K+ = 24			

^*^K− (knobless infected erythrocytes).

^†^K+ (knobby infected erythrocytes).

^‡^Negative (without specific fluorescence signal), Parasite (inside parasitic bounderies), MC (Maurer’s clefts), EM (Erythrocyte membrane).

**Table 2:** Height and width of the knobs on the surface erythrocytes infected with *P. falciparum* isolates 3D7 and FCR3 and cultivated in the presence of AlbuMAX or human serum after enrichment for knob carrying IEs (n ≥ 43).

Isolate	Cultivation	Diameter (nm)	Height (nm)
**3D7**	AlbuMAX	87 ± 38	13 ± 9
**3D7**	Human serum	82 ± 27	4 ± 3
**FCR3**	AlbuMAX	115 ± 40	7 ± 4
**FCR3**	Human serum	68 ± 26	6 ± 5
