# Supplementary material for: Type of in vitro cultivation influences cytoadhesion, knob structure, protein localization and transcriptome profile of Plasmodium falciparum
Source: Sci Rep. 2015 Nov 16;5:16766. doi: 10.1038/srep16766 (PMC4645185; doi:10.1038/srep16766)
Supplement: Supplementary Information [file srep16766-s1.pdf]

Type of *in vitro* cultivation influences cytoadhesion, knob structure, protein localization and transcriptome profile of *Plasmodium falciparum*

Ann-Kathrin Tilly[1], Jenny Thiede[1], Nahla Metwally[1], Pedro Lubiana[1], Anna Bachmann[1], Thomas Roeder[2], Nichola Rockliffe[3], Stephan Lorenzen[1], Egbert Tannich[1], Thomas Gutsmann[4], Iris Bruchhaus[1*]

[1]Bernhard Nocht Institute for Tropical Medicine, Bernhard-Nocht-Str. 74, 20359 Hamburg, Germany

[2]Zoological Institute, Molecular Physiology, Christian-Albrechts University Kiel, Olshausenstraße 40, 24098 Kiel, Germany

[3]The Centre for Genomic Research, University of Liverpool, The Biosciences Building, Crown Street, Liverpool, L69 7ZB, UK

[4]Division of Biophysics, Research Center Borstel, Leibniz-Center for Medicine and Biosciences, Parkallee 10, 23845 Borstel, Germany

*Corresponding author:
Iris Bruchhaus, Bernhard Nocht Institute for Tropical Medicine, Bernhard-Nocht-Str. 74, 20359 Hamburg, Germany
Tel.: 00494042818472, Fax: 00494042818512, email: bruchhaus@bnitm.de

Table S1. Differentially expressed genes (threshold, ≥ 3-fold; padj ≤ 0.05) in FCR3 knobless and knobby IEs cultivated in the presence of human serum.

	HS_K-_1	HS_K-_2	HS_K-_3	HS_K-_4	HS_K-_5	HS_K-_6	HS_K+_1	HS_K+_2	baseMeanA	baseMeanB	foldChange	log2FoldChange	pval	padj	name
PFIT_1240500	2136,60654	1493,994474	1243,955867	854,1931167	1107,065146	3360,822019	5869,638185	5432,341483	1699,439527	5650,989834	3,325207955	1,733444568	0,0016236	1	erythrocyte membrane protein 1, PfEMP1
PFIT_bin11000	492,9655659	234,3950275	244,9883689	210,880887	265,4952612	501,0556154	1032,053908	999,5456201	324,9634543	1015,799764	3,125889236	1,644266658	0,00434	1	erythrocyte membrane protein 1, PfEMP1, putative
PFIT_1452100	27,87980064	91,27532223	118,4719874	107,3233086	115,2149247	153,384372	216,2462823	345,9965608	102,2582859	281,1214215	2,749130978	1,458975643	0,028388	1	acetyl-CoA acetyltransferase, putative
PFIT_1231500	65,89771061	92,73572738	252,3014546	160,671152	50,09344552	204,5124961	323,3589268	461,3287477	137,7019977	392,3438373	2,849224004	1,51056905	0,0349546	1	conserved Plasmodium protein, unknown function
PFIT_0411500	693,1932251	266,5239409	299,8365112	219,6675906	131,9127399	429,4762417	885,8687265	691,3415273	340,1017082	788,6051269	2,318733214	1,213336837	0,040949	1	BC_ITvar27 - erythrocyte membrane protein 1,, putative (VAR)
PFIT_1452000	48,15601929	102,2283609	141,8738614	101,0470917	93,50776496	98,84770643	232,4142286	306,9009042	97,61013411	269,6575664	2,762598053	1,466025671	0,0462235	1	conserved Plasmodium protein, unknown function
PFIT_bin06200	115,3209936	30,66850827	35,83411963	19,45627231	11,68847062	68,17083202	119,9122687	91,87479298	46,85653274	105,8935308	2,259952341	1,176292348	0,0583651	1	B_ITvar54 - erythrocyte membrane protein 1, PfEMP1, putative
PFIT_1442000	454,9476559	143,1197053	206,9603236	161,2987737	260,4859167	518,0983234	68,04010751	76,88812462	290,8184498	72,46411607	0,249173036	-2,004780137	0,0592019	1	major facilitator superfamily, putative
PFIT_0800100	163,4770129	78,8618784	111,1589017	82,84606275	61,78191614	95,43916483	253,9714904	224,8000254	98,92748945	239,3857579	2,4198103	1,274893952	0,0627285	1	B_ITvar13 - erythrocyte membrane protein 1, PfEMP1, putative
PFIT_1358700	1364,842968	579,7808468	968,2525387	976,5793457	1830,080543	2133,747042	387,3570477	374,0151147	1308,880547	380,6860812	0,290848605	-1,781659711	0,0665204	1	conserved Plasmodium protein, unknown function
PFIT_1200200	290,2033794	168,6767955	206,229015	192,0522364	138,5918659	136,341664	487,0593835	418,3235255	188,6824927	452,6914545	2,399223415	1,262567507	0,0720207	1	erythrocyte membrane protein 1, PfEMP1
PFIT_0732100	245,8491511	107,3397789	342,2524079	288,0783546	227,0902863	736,2449858	61,3034632	73,63015324	324,4758275	67,46680822	0,207925529	-2,265861189	0,0809557	1	Plasmodium exported protein, unknown function
PFIT_1302100	686,8569067	537,4290973	1023,83199	798,3347865	318,9282698	2014,448086	1447,704862	1924,157898	896,6381893	1685,93138	1,880280586	0,910947965	0,0830033	1	conserved Plasmodium protein, unknown function
PFIT_0702300	373,8427813	298,6528543	498,7524406	373,4349041	165,3083702	875,9951915	781,4507397	904,4128557	430,997757	842,9317977	1,95576841	0,967735545	0,0833552	1	conserved Plasmodium protein, unknown function
PFIT_1219100	352,299299	164,29558	134,5607758	111,0890387	140,2616474	405,6164505	466,8494505	305,5977157	218,0204652	386,2235831	1,771501509	0,824972694	0,0853564	1	erythrocyte membrane protein 1 (PfEMP1), pseudogene
PFIT_0401700	0	0,730202578	21,93925692	8,786703625	23,37694124	61,35374882	47,83017459	29,97333672	19,36447553	38,90175565	2,008923794	1,006422838	0,09512	1	tRNA Asparagine
PFIT_0700100	27,87980064	2,190607733	16,08878841	22,59438075	8,348907586	57,94520722	2,694657723	1,954782829	22,50794872	2,324720276	0,103284413	-3,275305543	0,0988076	1	stevor
PFIT_0811500	1338,230431	632,3554324	568,9580627	561,0937886	515,9624888	1700,862259	1568,290795	1430,901031	886,2437437	1499,595913	1,692080676	0,758798356	0,0998129	1	erythrocyte membrane protein 1, PfEMP1
PFIT_1452200	1946,51699	1044,189686	1260,044656	921,3486372	479,2272954	1844,021006	2134,842581	2673,491316	1249,224712	2404,166949	1,924527209	0,944504069	0,0999206	1	conserved Plasmodium protein, unknown function
PFIT_0727300.2	0	5,841620623	16,08878841	15,0629205	6,679126069	0	40,41986585	24,76058251	7,2787426	32,59022418	4,47745249	2,162678124	0,1042426	1	conserved Plasmodium protein, unknown function
PFIT_1327800	2402,73191	1300,490791	737,890341	1196,874558	1015,227162	1670,185385	595,5193568	454,812805	1387,233358	525,1660809	0,378570828	-1,401364851	0,1068298	1	alpha/beta-hydrolase, putative
PFIT_1333600	447,3440739	99,30755058	144,0677871	119,8757423	136,9220844	422,6591585	42,44085914	52,77913639	228,3627328	47,60999777	0,208484095	-2,261990766	0,1179053	1	MSP7-like protein
PFIT_0828000	0	2,920810311	42,41589671	50,209735	13,35825214	0	65,34544979	65,15942765	18,15078236	65,25243872	3,595020723	1,846000087	0,1179748	1	conserved Plasmodium protein, unknown function
PFIT_0536400	107,7174116	321,2891342	370,7734419	329,5013859	151,9501181	214,7381209	107,1126445	131,6220438	249,3282688	119,3673442	0,478755757	-1,062638258	0,1182072	1	stevor, pseudogene
PFIT_0400700	7,603581993	24,82688765	202,5724722	173,2235857	63,45169765	316,9943689	22,90459065	16,94145119	131,4454324	19,92302092	0,151568758	-2,721955686	0,1318895	1	Plasmodium exported protein (PHISTa), unknown function
PFIT_bin05500	10,13810932	6,5718232	145,5304042	111,7166604	118,5544877	115,8904144	149,5535036	194,1750944	84,73364988	171,864299	2,028288634	1,020262969	0,1358044	1	Plasmodium exported protein (hyp15), unknown function
PFIT_1248800	396,6535273	381,1657456	756,1730551	632,642661	340,6354295	681,7083202	1021,275277	1103,14911	531,4964565	1062,212194	1,998531092	0,998940018	0,1429856	1	conserved Plasmodium protein, unknown function
PFIT_0510900	39,28517363	113,9116021	150,6495642	137,4491496	45,08410096	238,5979121	188,6260406	281,4887274	120,8295838	235,057384	1,945362855	0,960039276	0,1455	1	conserved Plasmodium protein, unknown function
PFIT_1315900	35,48338264	41,62154694	21,20794835	28,24297594	20,03737821	34,08541601	66,01911422	69,72058758	30,11310801	67,8698509	2,253830819	1,172379226	0,1463073	1	conserved Plasmodium protein, unknown function
PFIT_1322200	225,5729325	300,1132595	190,1402266	164,4368821	88,49842041	201,1039545	438,5555444	365,5443891	194,9776126	402,0499668	2,062031437	1,044066328	0,147317	1	beta-hydroxyacyl-ACP dehydratase
PFIT_0727300.1	0	11,68324125	20,47663979	11,92481206	20,03737821	10,2256248	45,80918129	23,45739395	12,39128268	34,63328762	2,794971958	1,482833808	0,1478559	1	conserved Plasmodium protein, unknown function
PFIT_0902000	16,47442765	19,7154696	29,98365112	25,1048675	60,11213462	44,31104081	10,77863089	6,515942765	32,61693188	8,647286829	0,2651165	-1,915301632	0,1485768	1	Plasmodium exported protein, unknown function
PFIT_0313700	88,70845659	90,54511965	87,75702767	75,3146025	263,8254797	170,4270801	46,48284572	39,09565659	129,4296277	42,78925116	0,33059858	-1,596847566	0,1493715	1	conserved Plasmodium protein, unknown function
PFIT_0517200	419,4642733	393,5791894	378,0865276	319,4594389	173,6572778	231,7808289	677,7064174	665,9293505	319,3379227	671,817884	2,103783598	1,072986312	0,1494803	1	conserved Plasmodium protein, unknown function
PFIT_0205900	57,02686495	189,8526702	272,7780944	187,6588846	121,8940508	286,3174945	348,9581751	379,2278689	185,9213432	364,093022	1,958317511	0,969614694	0,1593243	1	transcription factor, putative
PFIT_0536500	25,34527331	13,87384898	44,6098224	30,125841	16,69781517	68,17083202	10,77863089	8,470725594	33,13723881	9,624678243	0,290449011	-1,783643177	0,1652027	1	rifin
PFIT_0110800	191,3568135	192,043278	174,7827468	183,8931544	242,11832	514,6897818	93,63935588	87,31363305	249,8140157	90,47649446	0,362175414	-1,465239483	0,1655669	1	multidrug resistance-associated protein 1
PFIT_0209700	35,48338264	25,55709022	38,75935389	41,42303137	76,80994979	68,17083202	13,47328862	16,28985691	47,70060666	14,88157276	0,31197869	-1,680480608	0,1679906	1	50S ribosomal protein L33, putative
PFIT_0834700	750,22009	246,8084713	434,397287	470,0886439	48,42364	334,0370769	492,4486989	1002,803591	380,6625389	747,6261452	1,964012922	0,973804422	0,1689828	1	Plasmodium exported protein (PHISTa), unknown function, pseudogene
PFIT_0535500	124,1918392	70,09944747	21,20794835	39,54016631	148,610555	44,31104081	20,20993292	24,10898823	74,6601662	22,15946058	0,29680433	-1,752415958	0,1710339	1	conserved Plasmodium protein, unknown function
PFIT_1245600	219,2366141	115,3720073	111,8902103	123,0138507	232,0996309	299,9516609	66,69277865	64,50783337	183,5939957	65,60030601	0,357311827	-1,484744428	0,1719161	1	mitochondrial ribosomal protein L3 precursor, putative
PFIT_1238200	347,2302444	273,8259667	199,647238	210,880887	512,6229258	647,6229042	146,1851815	115,9837812	365,3050277	131,0844813	0,358835689	-1,47860471	0,1728018	1	conserved Plasmodium protein, unknown function
PFIT_0403200	676,7187974	552,0331488	892,9277566	717,9992105	470,8783879	1523,618096	1244,258204	1402,882477	805,6958995	1323,57034	1,642766634	0,71612755	0,1742176	1	6-cysteine protein
PFIT_1350500	102,6483569	94,19613254	151,3808727	92,88800975	50,09344552	51,12812402	187,9523762	209,1617627	90,38915691	198,5570695	2,196691243	1,135332105	0,1766223	1	conserved Plasmodium membrane protein, unknown function
PFIT_0500900	282,5997974	185,4714548	230,3621976	268,6220822	447,5014466	841,9097755	98,35500689	109,4678384	376,0777924	103,9114227	0,276303001	-1,855676863	0,1773552	1	erythrocyte membrane protein 2
PFIT_0422200	74,76855627	76,67127067	158,6939584	143,7253664	113,5451432	289,7260361	63,32445649	48,21797646	142,8550552	55,77121648	0,390404221	-1,356959445	0,1787761	1	Plasmodium exported protein (PHISTc), unknown function
PFIT_1369100	0	0	0	0,627621687	3,339563034	0	4,041986585	6,515942765	0,661197454	5,278964675	7,983945863	2,997101938	0,1803113	1	tRNA Cysteine
PFIT_0833200	230,6419871	118,2928176	187,9463009	191,4246147	30,05606731	153,384372	226,3512487	381,1826517	151,9576933	303,7669502	1,999023173	0,999295196	0,1807663	1	sporozoite invasion-associated protein 2 (SIAP-2)
PFIT_bin09200	43,08696463	18,98526702	14,62617128	16,94578556	26,71650428	30,67687441	49,85116788	44,96000508	25,17292786	47,40558648	1,883197169	0,913184056	0,1816938	1	erythrocyte membrane protein 1, PfEMP1 (VAR)
PFIT_1436900.1	76,03581993	77,40147325	87,75702767	91,63276637	158,6292441	259,0491617	30,31489939	54,08232495	125,0842488	42,19861217	0,337361519	-1,567632673	0,184383	1	ataxin-2 like protein, putative
PFIT_0805700	50,69054662	40,89134436	220,1238777	143,7253664	343,9749925	129,5245808	280,9180676	254,1217678	154,8217848	267,5199177	1,727921676	0,789037823	0,1861851	1	conserved Plasmodium protein, unknown function
PFIT_1132600	2587,752405	2408,208102	1026,757224	1798,763756	1769,968408	2754,101614	938,4145521	908,9740157	2057,591918	923,6942839	0,448920058	-1,155469536	0,18654	1	oxysterol-binding protein-related protein 2
PFIT_0727700	81,1048746	62,79742169	89,95095337	83,47368444	272,1743873	184,0612465	45,13551686	34,53449665	128,9270946	39,83500676	0,308973121	-1,69444676	0,187349	1	conserved Plasmodium protein, unknown function
PFIT_1448500	27,87980064	48,19337014	57,77337655	42,67827475	40,07475641	71,57937362	108,4599734	76,88812462	48,02982535	92,67404899	1,92951043	0,948234843	0,187815	1	mitochondrial ribosomal protein S14 precursor, putative
PFIT_1452500	86,17392926	75,21086552	108,964976	98,53660494	212,0622527	109,0733312	154,9428191	237,1803166	115,0036599	196,0615679	1,704828942	0,76962699	0,1886948	1	conserved Plasmodium protein, unknown function
PFIT_bin02000	1,267263666	4,381215467	2,193925692	1,255243375	0	0	11,45229532	8,470725594	1,5162747	9,961510459	6,569726752	2,715833367	0,1891108	1	rifin
PFIT_0316400	505,6382026	92,73572738	119,9346045	138,0767712	253,8067906	224,9637457	77,47140954	57,34029633	222,5259737	67,40585293	0,302912293	-1,723027967	0,1897072	1	conserved Plasmodium protein, unknown function
PFIT_0200700	0	0,730202578	15,35747984	5,0209735	0	0	14,14695305	19,54782829	3,51810932	16,84739067	4,78876695	2,259652945	0,1965796	1	Plasmodium exported protein (hyp10), unknown function
PFIT_0732500	43496,29079	29475,35726	28354,29564	18423,83464	23363,58299	70430,6951	35914,39813	64926,1569	35590,67607	50420,27751	1,416670968	0,502504721	0,2018707	1	histidine-rich protein III
PFIT_1341300	2383,722955	2738,989869	3539,533449	2888,315006	2289,27046	5750,209681	5113,11303	5396,503798	3265,006903	5254,808414	1,609432558	0,686552123	0,2024528	1	aconitate hydratase
PFIT_1140000	12595,33357	5797,808468	6229,286348	4400,255651	5022,702804	16695,03676	11636,87938	11753,45756	8456,737267	11695,16847	1,382940973	0,467739581	0,2063429	1	transcription factor with AP2 domain(s)
PFIT_0315200	1208,969537	853,6068135	308,612214	457,5362102	470,8783879	262,4577033	307,8646449	235,8771281	593,676811	271,8708865	0,457944258	-1,126756095	0,2064248	1	circumsporozoite- and TRAP-related protein
PFIT_0936600	1994,67301	5951,881212	4675,986958	3134,342707	2501,332713	2266,680165	7499,232443	6335,45115	3420,816127	6917,341797	2,022132012	1,015877184	0,2068141	1	ring-exported protein 2
PFIT_1348000	262,3235788	170,1372006	419,7711157	287,4507329	275,5139503	187,4697881	602,2560011	410,5043942	267,1110611	506,3801976	1,895766486	0,922781269	0,2083726	1	E1-E2 ATPase, putative
PFIT_0411300	17,74169132	16,79465929	19,01402266	23,84962412	16,69781517	17,04270801	45,13551686	39,09565659	18,5234201	42,11558673	2,273639884	1,185003768	0,2094382	1	erythrocyte membrane protein 1, PfEMP1
PFIT_0822400	65,89771061	71,55985263	111,8902103	89,12227962	130,2429583	303,3602025	41,09353028	33,2313081	128,678869	37,16241919	0,288799703	-1,791858835	0,2106894	1	conserved protein, unknown function
PFIT_0400500	25260,36665	6160,719149	9678,868844	9025,827488	7206,777028	30567,80108	3582,547443	4443,221371	14650,06004	4012,884407	0,273915902	-1,868195073	0,2115271	1	Plasmodium exported protein (PHISTb), unknown function
PFIT_bin07200	6,336318328	15,33425413	14,62617128	26,98773256	8,348907586	44,31104081	5,389315446	5,212754212	19,32407078	5,301034829	0,274322884	-1,866053118	0,2146891	1	rifin
PFIT_1403400	1,267263666	42,35174951	29,25234256	25,73248919	6,679126069	3,408541601	53,89315446	51,47594784	18,1152521	52,68455115	2,908297984	1,540175095	0,2159937	1	small nucleolar RNA snoR22
PFIT_0834900	65,89771061	97,11694285	119,9346045	126,1519592	85,15885738	109,0733312	162,3531278	221,542054	100,5555676	191,9475909	1,908870841	0,932719489	0,2166485	1	Plasmodium exported protein, unknown function, pseudogene
PFIT_0829500	84,90666559	61,33701654	40,22197102	20,083894	53,43300855	115,8904144	102,3969935	82,75247311	62,64549502	92,57473329	1,477755635	0,563407722	0,2173047	1	SNARE protein, putative
PFIT_bin04600	7,603581993	31,39871085	55,57945086	42,67827475	101,8566725	34,08541601	86,90271157	80,79769028	45,5336845	83,8502093	1,841498263	0,880880037	0,2174324	1	rifin (RIF)
PFIT_1207900	48,15601929	29,20810311	60,69861081	50,83735669	108,5357986	167,0185385	20,88359735	24,76058251	77,40907116	22,82208993	0,29482449	-1,762071726	0,2177468	1	mitochondrial ACP precursor
PFIT_0815200	98,84656592	68,63904232	201,8411636	134,9386628	105,1962356	34,08541601	230,3932353	219,5872712	107,2578477	224,9902532	2,097657729	1,068779295	0,2182219	1	conserved Plasmodium protein, unknown function
PFIT_0830900	5,069054662	18,25506445	128,7103073	125,5243375	55,10279007	146,5672888	154,9428191	138,1379866	79,8714738	146,5404028	1,834702628	0,875546247	0,220625	1	rhomboid protease ROM3
PFIT_0707800	262,3235788	362,1804786	578,4650741	431,1760993	409,0964717	691,933945	776,7350887	735,6499381	455,8626079	756,1925134	1,658816714	0,730154489	0,2214822	1	Cg3 protein
PFIT_1307900	147,0025852	62,06721911	29,98365112	38,91254462	156,9594626	122,7074976	35,0305504	28,01855389	92,93882672	31,52455214	0,339196795	-1,559805557	0,2235951	1	conserved Plasmodium protein, unknown function
PFIT_0820200	832,5922283	1223,089318	1856,792444	1943,116744	2745,120814	6527,357166	580,0250749	533,6557124	2521,344786	556,8403937	0,220850554	-2,178857641	0,2245122	1	asparagine-rich antigen
PFIT_1365000	36,7506463	175,2486187	222,3178034	170,713099	100,186891	354,4883265	302,4753294	284,7466988	176,6175642	293,6110141	1,66241185	0,733277266	0,2256259	1	conserved Plasmodium protein, unknown function
PFIT_1007300	803,445164	1059,52394	1222,747919	1215,075587	2815,251638	3895,96305	627,1815851	484,1345474	1835,33455	555,6580662	0,302755738	-1,723773792	0,2290595	1	transcription factor with AP2 domain(s)
PFIT_1015800	13440,59844	1542,187844	5971,134425	7690,876159	4984,297829	17536,94654	1521,134285	3665,869399	8527,673538	2593,501842	0,304127712	-1,717250815	0,2297033	1	glycophorin binding protein
PFIT_0625900	0	11,68324125	22,67056548	18,20102894	8,348907586	10,2256248	30,31489939	33,88290238	11,85489468	32,09890088	2,707649605	1,437041053	0,2297747	1	conserved Plasmodium protein, unknown function
PFIT_1218900	25,34527331	157,7237568	189,4089181	136,8215279	85,15885738	109,0733312	198,0573427	276,9275675	117,2552774	237,4924551	2,02543084	1,018228824	0,2314379	1	rifin, pseudogene
PFIT_0606800	935,2405852	791,5395944	278,6285629	728,6687792	480,897077	173,8356217	347,6108463	302,3397463	564,8017034	324,9752953	0,575379453	-0,797414391	0,2324163	1	conserved Plasmodium protein, unknown function
PFIT_1441400	32,94885531	40,89134436	68,01169645	51,46497837	108,5357986	143,1587472	30,31489939	23,45739395	74,16857006	26,88616467	0,36250054	-1,463944952	0,233469	1	delta-aminolevulinic acid dehydratase
PFIT_1222600	10,13810932	21,90607733	39,49066245	42,05065306	86,82863889	57,94520722	17,5152752	13,68347981	43,05989138	15,5993775	0,362271641	-1,464856221	0,2362647	1	conserved Plasmodium protein, unknown function
PFIT_1438100	44,3542283	354,8784528	653,0585476	684,1076394	322,2678328	681,7083202	784,8190619	869,2267648	456,7291702	827,0229133	1,810751245	0,856588367	0,2377742	1	histidine triad protein, putative
PFIT_0512200	62,09591961	9,492633512	24,86449117	23,22200244	33,39563034	61,35374882	13,47328862	8,470725594	35,73740432	10,9720071	0,30701746	-1,703607391	0,2382994	1	conserved Plasmodium protein, unknown function
PFIT_1000800	36,7506463	70,09944747	143,3364785	96,65373987	263,8254797	207,9210377	63,99812092	37,14087376	136,4311383	50,56949734	0,370659499	-1,431833611	0,2383958	1	acyl-CoA binding protein, isoform 2, ACBP2
PFIT_1419800	11601,79886	24261,71085	12487,09373	12708,08393	44831,96396	16395,0851	30300,75243	31167,05743	20380,95607	30733,90493	1,507971698	0,592609352	0,2396963	1	signal recognition particle RNA
PFIT_1469000	2442,017084	2529,42173	745,2034267	1292,273055	2135,650561	702,1595698	950,5405118	809,9316856	1641,120904	880,2360987	0,536362736	-0,898719085	0,2402621	1	rab GTPase activator, putative
PFIT_1145100	141,9335305	231,4742172	334,9393223	332,0118727	76,80994979	119,298956	401,5040007	476,9670104	206,0779748	439,2355056	2,131404417	1,091804359	0,2404076	1	conserved Plasmodium protein, unknown function
PFIT_0529200	451,1458649	449,8047879	1136,453508	1044,362488	898,3424563	3596,011389	214,8989534	245,6510422	1262,686749	230,2749978	0,182369062	-2,455067092	0,2411305	1	zinc finger protein, putative
PFIT_0221300	32,94885531	92,73572738	123,5911473	76,56984587	41,74453793	102,256248	129,3435707	168,1113233	78,30772697	148,727467	1,899269111	0,925444338	0,2413755	1	erythrocyte membrane protein 1 (PfEMP1), pseudogene
PFIT_1345400	92,51024759	200,8057089	384,6683046	377,8282559	148,610555	398,7993673	413,6299605	546,687598	267,2037399	480,1587792	1,796976268	0,845571356	0,242166	1	mog1 homolog, putative
PFIT_1149600	158,4079582	95,65653769	502,4089834	537,8717862	322,2678328	1823,569757	65,34544979	65,15942765	573,3638091	65,25243872	0,113806344	-3,135347118	0,2425672	1	Plasmodium exported protein, unknown function
PFIT_1443300	106,4501479	48,19337014	62,8925365	47,69924825	262,1556982	207,9210377	30,98856382	29,97333672	122,5520064	30,48095027	0,248718492	-2,007414319	0,2426026	1	replication factor A protein 3, putative
PFIT_0411350	216,7020868	53,30478818	51,19159948	42,67827475	33,39563034	132,9331224	158,3111412	107,5130556	88,36758367	132,9120984	1,504082073	0,588883292	0,2434288	1	erythrocyte membrane protein 1, PfEMP1
PFIT_1225900	148,2698489	151,8821362	348,1028764	296,2374365	56,77257158	139,7502056	344,2425241	441,1293252	190,1691792	392,6859246	2,064929377	1,04609244	0,2447486	1	conserved Plasmodium protein, unknown function
[illegible]	[illegible]	[illegible]	[illegible]	[illegible]	[illegible]	[illegible]	[illegible]	[illegible]	[illegible]	[illegible]	[illegible]	[illegible]	[illegible]	[illegible]	[illegible]

PFIT_1128600	8,870845659	27,74769796	27,05841687	21,96675906	6,679126069	10,2256248	33,68322154	33,88290238	17,09141174	33,78306196	1,976610387	0,983028527	0,4489298	1	conserved Plasmodium protein, unknown function
PFIT_0416400	984,6638682	267,9843461	204,7663979	174,4788291	447,5014466	875,9951915	169,7634366	218,2840826	492,5650132	194,0237596	0,393904874	-1,344080826	0,4491409	1	cdc2-related protein kinase 1
PFIT_0827600	46,88875563	102,9585635	236,9439747	262,3458654	170,3177148	27,26833281	210,1833024	288,6562645	141,1205345	249,4197834	1,767423744	0,821647971	0,4495076	1	conserved Plasmodium membrane protein, unknown function
PFIT_1307400	20093,73268	26055,08838	32320,18198	28278,12275	21303,0726	27472,8453	38144,90106	36802,04473	25920,50728	37473,4729	1,445707543	0,531775734	0,4500017	1	carbamoyl phosphate synthetase
PFIT_1408700	68,43223794	230,7440146	171,8575125	182,6379111	51,76322703	129,5245808	166,3951144	296,4753958	139,159914	231,4352551	1,663088518	0,733864958	0,4501416	1	conserved Plasmodium protein, unknown function
PFIT_0904100	3519,191199	7487,497233	9262,754271	8144,019017	7422,178844	18569,73464	10604,1518	11378,13926	9067,562534	10991,14553	1,212138928	0,277555061	0,450873	1	60S ribosomal protein L32, putative
PFIT_0207900	1064,501479	282,5883976	329,0888538	393,5187981	601,1213462	436,2933249	322,0115979	276,9275675	517,8520333	299,4695827	0,578291797	-0,790130458	0,4509472	1	ribosome-recycling factor, putative
PFIT_1462400	439,740492	684,1998154	778,112312	800,8452732	707,9873633	899,8549827	917,5309547	1075,130556	718,4567064	996,3307554	1,386765196	0,471723535	0,4509966	1	U3 small nucleolar ribonucleoprotein protein IMP3, putative (IMP3)
PFIT_0730900	1,267263666	8,032228356	3,65654282	3,765730125	1,669781517	6,817083202	6,736644308	11,0771027	4,201438281	8,906873504	2,119958192	1,084035814	0,4510677	1	rifin, pseudogene
PFIT_0321600	46,88875563	242,4272558	259,6145402	299,3755449	906,6913638	524,9154066	152,2481614	162,8985691	379,9854778	157,5733652	0,414682598	-1,269920588	0,4510807	1	peptidase, putative
PFIT_1465200	646,3044694	631,6252298	515,5725376	442,4732897	315,5887068	286,3174945	747,0938537	688,0835559	472,980288	717,5887048	1,517164083	0,601377123	0,4511077	1	conserved Plasmodium protein, unknown function
PFIT_1127200	1397,791823	1641,495395	2336,530862	1966,338747	1831,750324	3524,432015	2743,161562	2644,821168	2116,389861	2693,991365	1,272918291	0,348139815	0,4512839	1	DnaJ protein, putative
PFIT_1110600	0	13,1436464	5,850468512	5,0209735	328,9469589	248,8235369	8,083973169	5,864348488	100,2975974	6,974160829	0,069534675	-3,846123602	0,4513341	1	unspecified product
PFIT_0936700	1368,644759	1409,290975	3059,795032	2060,482	1056,9717	1680,411009	2264,186152	3146,548761	1772,599246	2705,367456	1,526214943	0,609958157	0,4513768	1	early transcribed membrane protein
PFIT_1315800	1876,817489	2762,356352	1515,271344	2059,854378	3468,136211	4915,116989	2848,926878	3501,667642	2766,258794	3175,29726	1,147867028	0,198955526	0,4516319	1	choline-phosphate cytidylyltransferase
PFIT_bin02200	694,4604887	440,3121544	624,5375136	399,1673932	258,8161352	773,7389434	768,6511155	622,272534	531,8387714	695,4618248	1,307655369	0,38698237	0,4519715	1	stII_A4varTres - erythrocyte membrane protein 1,, putative
PFIT_0825400	1,267263666	40,89134436	84,83179342	84,72892781	20,03737821	47,71958241	77,47140954	89,92001015	46,57938165	83,69570985	1,796840295	0,845462186	0,4519907	1	dynein light chain 1
PFIT_0305600	470,1548199	685,6602206	721,8015526	543,5203814	729,694523	538,549573	924,267599	820,3571941	614,8968451	872,3123966	1,41863209	0,504500488	0,4523801	1	ubiquitin conjugating enzyme E2, putative
PFIT_1218500	2,534527331	20,44567218	29,98365112	23,84962412	50,09344552	54,53666562	11,45229532	15,63826264	30,24059765	13,54527898	0,44791704	-1,158696544	0,4524144	1	secreted ookinete protein, putative (PSOP17)
PFIT_1104600	1200,098691	1098,224677	617,224428	1168,00396	1566,255063	814,6414426	737,6625517	652,2458707	1077,408044	694,9542112	0,645024153	-0,632574911	0,4528473	1	CCR4-NOT transcription complex subunit 1, putative
PFIT_0423600	173,6151222	130,7062614	108,964976	111,7166604	83,48907586	85,21354003	140,795866	199,3878486	115,617606	170,0918573	1,47115879	0,556952973	0,4531184	1	Plasmodium exported protein (PHISTa), unknown function
PFIT_1362800	112,7864662	46,73296498	27,05841687	18,82865062	35,06541186	34,08541601	19,53626849	25,41217678	45,75955443	22,47422264	0,49113727	-1,025801789	0,4534781	1	conserved Plasmodium membrane protein, unknown function
PFIT_0620500	0	0	0,731308564	0,627621687	8,348907586	6,817083202	3,368322154	3,909565659	2,754153507	3,638943906	1,321256748	0,40191084	0,4535047	1	tRNA Serine
PFIT_1225800	119,1227846	70,09944747	65,81777075	67,78314225	61,78191614	40,90249921	87,576376	122,499724	70,91792673	105,03805	1,481121274	0,566689773	0,4536564	1	hydrolase/phosphatase, putative
PFIT_1204000	2768,971109	8348,406072	7396,454816	5556,962421	7193,418776	11766,28561	9784,302193	8601,696044	7171,7498	9192,999118	1,28183489	0,358210444	0,453689	1	eukaryotic translation initiation factor 5A
PFIT_0628300	76,03581993	159,184162	198,9159294	172,5959641	482,5668585	726,019361	113,1756244	91,87479298	302,5530158	102,5252087	0,33886692	-1,561209285	0,4538617	1	RNA binding protein, putative
PFIT_1149300.2	2509,182058	2313,281767	2580,787922	2064,875352	1382,579096	3633,505347	2890,694072	3435,205026	2414,035257	3162,949549	1,31023337	0,389823797	0,4539862	1	Plasmodium exported protein, unknown function
PFIT_1212100	3335,437968	3643,710863	4455,86308	3564,263563	3842,167271	4073,207213	5048,441244	5468,179168	3819,108326	5258,310206	1,376842382	0,461363412	0,4540334	1	glycerol-3-phosphate acyltransferase
PFIT_1431300	60,82865595	42,35174951	48,99767378	88,49465794	120,2242692	64,7629042	38,39887255	43,00522225	70,94321614	40,7020474	0,573727125	-0,801563368	0,4541043	1	phosphotyrosyl phosphatase activator, putative
PFIT_1407900	31,68159164	51,84438303	24,86449117	52,09260006	65,12147917	54,53666562	28,96757052	22,80579968	46,69020178	25,8866851	0,554435066	-0,85090959	0,4542662	1	radical SAM protein, putative
PFIT_0528100	325,6867621	1327,508286	1363,890472	1057,542543	1001,86891	1083,916229	1566,269802	1477,164225	1026,735534	1521,717013	1,482092479	0,567635472	0,4547579	1	conserved Plasmodium membrane protein, unknown function
PFIT_1231100	120,3900482	473,901473	57,04206799	104,8128218	198,7040005	64,7629042	88,25004043	87,96522732	169,9354503	88,10763388	0,518477067	-0,947647917	0,4554288	1	conserved Plasmodium protein, unknown function
PFIT_0530900	824,9886463	365,1012889	696,2057529	588,7091429	619,4889429	889,6293579	742,3782027	927,2186554	664,0205219	834,7984291	1,257187694	0,330200056	0,4563964	1	conserved Plasmodium protein, unknown function
PFIT_0533300	57,02686495	34,31952116	27,05841687	23,22200244	120,2242692	64,7629042	21,55726179	26,71536533	54,43556085	24,13631356	0,443392392	-1,173344078	0,45734	1	conserved Plasmodium protein, unknown function
PFIT_1112600	26,61253698	83,97329645	86,29441055	81,59081937	176,9968408	47,71958241	118,5649398	121,1965354	83,8645811	119,8807376	1,429456107	0,515466321	0,4574685	1	peptidyl-prolyl cis-trans isomerase, putative
PFIT_1308300	13567,3248	13554,02025	12891,50737	10704,7155	12082,53906	16991,57988	18307,50457	16384,98968	13298,61448	17346,24712	1,304364989	0,383347623	0,4574788	1	60S ribosomal protein L24, putative
PFIT_1231400	4790,256656	2944,906996	3086,12214	2337,263164	1835,089887	3330,145144	3583,894772	4545,521673	3053,963998	4064,708222	1,330961408	0,41246874	0,4577113	1	pre-mRNA-splicing factor ATP-dependent RNA helicase PRP2, putative
PFIT_0103000	278,7980064	495,8075503	486,320195	358,3719836	470,8783879	640,805821	562,5097997	630,0916653	455,1636574	596,3007325	1,310079842	0,389654739	0,4577971	1	conserved protein, unknown function
PFIT_0516500	391,5844727	267,2541435	223,049112	261,090622	370,6914968	224,9637457	166,3951144	197,4330658	289,7722654	181,9140901	0,627782958	-0,671662228	0,4581127	1	phosducin-like protein, putative (PhLP3)
PFIT_1300900	17402,06466	16146,9696	14299,27635	10807,01784	13309,82847	44103,11978	17175,74833	24609,41263	19344,71278	20892,58048	1,080015026	0,111051385	0,4584061	1	Plasmodium exported protein (hypB), unknown function
PFIT_0722200	3017,354788	2425,002761	1859,717678	1332,440843	1208,921818	3739,170136	2567,335146	2925,006707	2263,768004	2746,170926	1,213097332	0,278695309	0,4586087	1	Rab GTPase activator and protein kinase, putative
PFIT_1322800	92,51024759	92,73572738	96,53273044	94,14325312	340,6354295	231,7808289	70,0611008	72,97855896	158,0563695	71,51982988	0,452495715	-1,144023965	0,4590566	1	conserved Plasmodium protein, unknown function
PFIT_1311900	572,8031768	186,9318599	392,7126988	372,1796607	305,5700176	674,891237	224,3302555	251,5153907	417,5147752	237,9228231	0,569854858	-0,811333582	0,4590999	1	probable protein, unknown function
PFIT_1416000	62,09591961	59,87661138	50,46029091	87,23941456	272,1743873	51,12812402	37,05154369	41,05043942	97,16245796	39,05099156	0,401914406	-1,315039806	0,4594718	1	uracil-DNA glycosylase, putative
PFIT_0728500	12,67263666	23,36648249	43,14720527	60,251682	145,270992	61,35374882	20,88359735	28,67014816	57,67712454	24,77687276	0,429578849	-1,219005134	0,4596622	1	conserved Plasmodium protein, unknown function
PFIT_0303200	579,1394952	908,3720068	1069,904429	875,5322541	756,4110273	933,9403987	1329,139922	1112,27143	853,8832685	1220,705676	1,429593155	0,515604632	0,4601345	1	DNA-directed RNA polymerase subunit I, putative
PFIT_1473800	297,8069614	186,9318599	127,2476901	185,7760195	432,473413	204,5124961	169,7634366	103,60349	239,12474	136,6834633	0,571599004	-0,806924692	0,4605344	1	DNA-directed RNA polymerase, alpha subunit, putative
PFIT_0613000	266,1253698	873,3222831	266,1963173	687,8733695	733,0340861	218,1466625	387,3570477	368,8023605	507,4496814	378,0797041	0,745058511	-0,424574367	0,4609547	1	thiamin-phosphate pyrophosphorylase, putative
PFIT_1114500	639,9681511	985,7734801	1029,682458	914,4447987	776,4484055	1421,361848	1110,198982	1441,32654	961,2798568	1275,762761	1,327150207	0,408331664	0,4613597	1	glyoxalase I
PFIT_1221700	363,704672	181,0902393	330,5514709	261,7182437	227,0902863	504,464157	313,9276247	451,5548336	311,4365115	382,7412292	1,228954265	0,297431228	0,4617846	1	conserved protein, unknown function
PFIT_0822700	377,6445723	170,1372006	67,28038788	109,2061736	185,3457484	64,7629042	91,61836259	84,05566166	162,3960622	87,83701213	0,540881416	-0,886615765	0,4619774	1	conserved Plasmodium protein, unknown function
PFIT_1466400	528,4489485	960,9465924	1372,666175	1311,729327	869,9561705	1227,074976	1458,483493	1576,206555	1045,137032	1517,345024	1,45181443	0,537857061	0,4621431	1	tRNA 3'-trailer sequence RNase, putative
PFIT_0618900	102,6483569	72,2900552	146,9930214	145,6082315	145,270992	109,0733312	83,53438942	72,32696469	120,313998	77,93067705	0,647727433	-0,626541248	0,462518	1	conserved Plasmodium protein, unknown function
PFIT_1430300	732,4783987	198,6151012	199,647238	188,2865062	515,9624888	661,2570706	179,868403	206,5553856	416,0411339	193,2118943	0,464405749	-1,106542264	0,46266	1	apicoplast ribosomal protein L15 precursor, putative
PFIT_1027000	92,51024759	239,5064455	179,1705982	121,7586074	96,847328	119,298956	229,0459065	212,4197341	141,5153638	220,7328203	1,559779902	0,641342467	0,4633219	1	topoisomerase, putative
PFIT_0818200	345,9629807	149,6915285	128,7103073	142,4701231	238,778757	132,9331224	109,1336378	117,938564	189,7578031	113,5361009	0,598321118	-0,741008111	0,4645419	1	FAD-dependent monooxygenase, putative
PFIT_0211300	73,5012926	62,06721911	86,29441055	70,92125069	232,0996309	95,43916483	61,97712763	48,86957073	103,3871614	55,42334918	0,536075741	-0,899491244	0,4656101	1	conserved Plasmodium protein, unknown function
PFIT_0624400	117,8555209	169,4069981	179,9019067	169,4578556	88,49842041	231,7808289	245,2138528	202,64582	159,4835884	223,9298364	1,40409329	0,489638794	0,46572	1	conserved Plasmodium membrane protein, unknown function
PFIT_0414400	1036,621678	748,4576423	193,7967694	482,013456	472,5481694	163,6099968	343,5688597	330,3582982	516,1746187	336,9635789	0,652809276	-0,615266539	0,4658602	1	RING zinc finger protein, putative
PFIT_0707900	1880,61928	6804,02762	6916,716398	6015,126253	4630,304147	2842,723695	8678,818862	6916,02165	4848,252899	7797,420256	1,608294868	0,685531937	0,4666378	1	chloroquine resistance transporter
PFIT_0711600	784,436209	909,832412	948,5072074	1082,647411	495,9251106	668,0741538	1203,164673	1288,853479	814,9037506	1246,009076	1,529026066	0,612613001	0,4670946	1	conserved Plasmodium protein, unknown function
PFIT_1225100	729,9438714	442,5027622	471,6940237	604,3996851	362,3425892	364,7139513	347,6108463	347,2997494	495,9328138	347,4552978	0,700609615	-0,513317307	0,4672503	1	conserved Plasmodium protein, unknown function
PFIT_1303300	421,9988006	354,1482502	261,0771573	419,8789089	1145,470121	484,0129073	291,0230341	257,3797192	514,4310242	274,2013867	0,53301876	-0,907741784	0,467389	1	DNA ligase I
PFIT_0522900	1,267263666	10,22283609	2,925234256	2,51048675	8,348907586	10,2256248	10,77863089	8,470725594	5,916725525	9,624678243	1,62669	0,701939341	0,4676741	1	iron-sulfur assembly protein, putative, putative
PFIT_0423400	201,4949228	273,8259667	310,8061397	319,4594389	253,8067906	180,6527049	402,8513296	377,2730861	256,6743273	390,0622078	1,519677531	0,603765272	0,4677342	1	Plasmodium exported protein (hyp15), unknown function
PFIT_1105700	3244,194984	2069,394106	931,6871105	1600,435303	1270,703735	470,3787409	878,4584177	1316,220438	1597,798996	1097,339428	0,686781898	-0,54207608	0,4683465	1	calcium/calmodulin-dependent protein kinase, putative
PFIT_1032200	22,81074598	35,77992631	24,86449117	32,63632775	118,5544877	37,49395761	16,16794634	23,45739395	45,35665609	19,81267015	0,436819463	-1,194890957	0,468743	1	leucine-rich repeat protein
PFIT_1320300	823,7213826	1445,801104	573,3459141	993,5251313	2354,391939	541,9581146	715,4316255	686,1287731	1122,123931	700,7801993	0,62451302	-0,679198104	0,4688437	1	conserved Plasmodium protein, unknown function
PFIT_0506500	3061,709016	1486,692448	1415,082071	1929,936689	1390,928004	3101,772857	1192,386042	1270,608839	2064,353514	1231,497441	0,596553561	-0,745276419	0,4690961	1	conserved Plasmodium protein, unknown function
PFIT_1125800	1682,926148	1904,368323	3214,101139	2919,69609	1823,401417	3371,047643	2580,808434	4252,304248	2485,92346	3416,556341	1,374361036	0,458761041	0,4693795	1	60S ribosomal protein L35, putative
PFIT_0729400	1130,39919	1069,746777	340,7897908	685,9905044	1218,940508	330,6285353	547,0155178	508,8951299	796,0825507	527,9553239	0,66319168	-0,592502188	0,4695516	1	conserved Plasmodium protein, unknown function
PFIT_1013200	5,069054662	41,61254694	24,86449117	37,65730125	135,2523029	61,35374882	18,86260406	19,54782829	50,96974096	19,20521618	0,376796425	-1,408142818	0,4702057	1	conserved Plasmodium protein, unknown function
PFIT_1035700	813,5832733	1477,199815	501,6776749	1275,954891	771,4390609	548,7751978	772,6931021	597,5119515	898,1049854	685,1025268	0,762831226	-0,390564195	0,4702659	1	probable protein, unknown function
PFIT_1132300	5,069054662	17,52486187	8,775702767	8,159081937	41,74453793	6,817083202	23,57825508	18,89623402	14,68172039	21,23724455	1,446509263	0,532575562	0,4702948	1	conserved Plasmodium protein, unknown function
PF_apicoplast_ge	6,336318328	18,98526702	19,74533123	11,92481206	16,69781517	6,817083202	30,98856382	17,59304546	13,41777117	24,29080464	1,810345722	0,856265236	0,4703954	1	probable protein, unknown function
PFIT_1023500	508,1727299	473,1712704	430,7407442	825,3225191	494,2553291	654,4399874	334,8112221	430,7038167	564,35043	382,7575194	0,678226682	-0,560160553	0,4705618	1	conserved Plasmodium protein, unknown function
PFIT_0403400	1769,100077	3637,13904	715,2197755	1859,015438	4027,513019	957,8001899	1514,39764	1233,467965	2160,96459	1373,932803	0,635796074	-0,653363986	0,4706533	1	dipeptidyl peptidase 3
PFIT_1004800	0	0	0	0	1,669781517	0	0,673664431	1,954782829	0,27829692	1,31422363	4,722379365	2,239513943	0,4709052	1	conserved Plasmodium protein, unknown function
PFIT_1461500	822,454119	1594,76243	1853,86721	1941,233879	1050,292574	2130,338501	1989,331064	2441,523754	1565,491452	2215,427409	1,415164168	0,500969425	0,4709542	1	ribosomal protein L29, putative
PFIT_1003900	14624,2227	4382,675872	6202,959239	5669,934325	4995,986299	9404,166277	5049,788573	3976,679869	7546,657452	4513,234221	0,598044134	-0,741676139	0,4717258	1	zinc finger, C3HC4 type, putative
PFIT_1440700	19670,46662	9558,351744	4492,428508	9359,722226	5114,540787	1857,655173	4383,534451	5801,143843	8342,194176	5092,339147	0,610431625	-0,712098388	0,4717462	1	CCAAT-binding transcription factor, putative
PFIT_1312000	1954,120572	1963,514732	579,9276912	1146,037201	2105,594493	623,763113	989,6130488	867,271982	1395,492967	928,4425154	0,66531508	-0,58789036	0,4722514	1	conserved Plasmodium protein, unknown function
PFIT_1250500	49,42328296	109,5303867	73,86216496	77,82508925	188,6853114	160,2014552	70,0611008	49,52116501	109,9212818	59,79113291	0,543945012	-0,87846728	0,4725534	1	Hbeta58/Vps26 protein homolog, putative
PFIT_0109000	1628,43381	1014,981583	1247,61241	1732,235857	806,5044728	1867,880797	883,8477332	941,5537295	1382,941489	912,7007313	0,659970605	-0,599526326	0,4726587	1	bromodomain protein, putative
PFIT_1343500	3090,85608	4847,084712	1453,841425	3650,875356	4869,082904	2665,479532	2274,291118	2350,952149	3429,536668	2312,621634	0,674324802	-0,568484434	0,4732784	1	conserved protein, unknown function
PFIT_1443100	10,13810932	71,55985263	48,26636522	50,209735	46,75388248	40,90249921	70,0611008	72,32696469	44,63840731	71,19403274	1,594905308	0,673470771	0,4733697	1	transcription factor TFIIH complex subunit Tfb5, putative
PFIT_0602900	253,4527331	102,2283609	136,7547015	124,8967158	130,2429583	74,98791522	101,0496646	77,5397189	137,0938975	89,29469176	0,651339654	-0,618518033	0,4733923	1	conserved Plasmodium protein, unknown function
PFIT_0216100.2	669,1152154	557,8747695	218,6612606	672,1828273	245,457883	122,7074976	400,1566719	246,9542308	414,3332422	323,5554513	0,780906329	-0,356778589	0,4736175	1	conserved Plasmodium protein, unknown function
PFIT_1427000	3095,925135	5759,107731	7798,674526	6148,182051	5977,817832	8644,0615	8604,04211	8078,46584	6237,294796	8341,253975	1,337319182	0,419343839	0,4737075	1	60S ribosomal protein L21e, putative
PFIT_0623700	13,93990032	17,52486187	24,86449117	23,22200244	30,05606731	10,2256248	24,92558394	40,39884514	19,97215799	32,66221454	1,635387351	0,709632387	0,4739608	1	conserved Plasmodium protein, unknown function
PFIT_1443600	1235,582074	1106,256905	764,9487579	893,1056613	1786,666223	2024,673711	666,9277865	776,7003775	1301,872222	721,814082	0,554443109	-0,850888662	0,4746231	1	conserved Plasmodium protein, unknown function
PFIT_1328400	1269,798193	2349,791895	1905,058809	2249,396128	3785,3947	4669,701993	1500,924352	1581,419309	2704,856953	1541,17183	0,569779422	-0,811524577	0,4747614	1	myosin C
PFIT_1410800	81,1048746	88,35451192	175,5140553	137,4491496	325,6073959	736,2449858	74,10308739	63,2046482	257,3791622	68,6538661	0,266742131	-1,906482385	0,4748132	1	RNA binding protein Bruno, putative
PFIT_1350300	103,9156206	343,9254142	476,8131837	357,7443619	382,3799674	265,8662449	542,2988668	420,9299026	321,7741321	481,6148847	1,496748298	0,58183163	0,4752196	1	conserved Plasmodium protein, unknown function
PFIT_1030700	1757,694704	295,732044	299,8365112	491,4277813	424,1245054	654,4399874	227,0249132	329,0551096	653,8759222	278,0400114	0,425218305	-1,233724391	0,4754386	1	conserved Plasmodium protein, unknown function
PFIT_1128800	1300,212521	1006,949355	372,236059	734,3173744	572,7350604	391,9822841	549,0365111	475,6638218	729,7387756	512,3501665	0,702100784	-0,510249956	0,4760653	1	conserved Plasmodium protein, unknown function
PFIT_0525400	0	4,381215467	8,044394203	3,765730125	8,348907586	0	12,12595975	6,515942765	4,09004123	9,320951259	2,278938215	1,188361812	0,4762789	1	conserved Plasmodium protein, unknown function
PFIT_0103100	358,6356174	260,6823203	155,7687241	151,8844484	187,0155299	231,7808289	136,7538794	140,7443637	224,2945782	138,7491216	0,618602209	-0,692916109	0,4764996	1	conserved Plasmodium protein, unknown function
PFIT_1402800	209,0985048	592,1942906	100,1892733	201,4665617	287,202421	132,9331224	160,3321345	175,9304546	253,8473623	168,1312946	0,66233225	-0,594372988	0,4768245	1	conserved Plasmodium protein, unknown function
PFIT_0508400	263,5908424	63,52762427	70,20562214	74,05939512	98,51710952	109,0733312	130,6908996	142,6991465	113,1623148	136,6950231	1,207955346	0,272567124	0,4774626	1	transcription factor IIb, putative
PFIT_bin05840	17,74169132	2,190607733	3,65654282	2,51048675	10,0186891	17,04270801	2,694657723	1,303188553	8,860120955	1,998923138	0,225609012	-2,148103395	0,4781329	1	rifin (RIF)
PFIT_1029300	1924,973508	2194,988949	548,481423	669,6723406	4682,067374	668,0741538	747,7675182	712,8441384	1781,376291	730,3058283	0,409967188	-1,286410649	0,4782957	1	conserved Plasmodium protein, unknown function
PFIT_0104200	566,4668585	448,3443828	410,2641044	412,3474487	728,0247415	610,1289466	345,589853	291,9142359	529,2627471	318,7520444	0,602256717	-0,731549516	0,4783174	1	asparagine-rich antigen Pfa35-2
PFIT_0503800	3672,530103	5222,408837	6053,772292	5907,802944	4995,986299	8241,853591	6497,493435	8367,122104	5682,392344	7432,307769	1,307953995	0,387311798	0,4783694	1	60S ribosomal protein L31, putative
PFIT_1326600	428,335119	210,2983424	93,60749618	145,6082315	651,2147917	143,1587472	122,6069264	126,4092896	278,703788	124,508108	0,44673992	-1,162492917	0,4786042	1	conserved Plasmodium protein, unknown function
[illegible]															

PFIT_0934000	1410,46446	773,2845299	608,4487252	573,0186007	1193,893785	1070,282063	528,8265782	629,4400711	938,2320272	579,1333246	0,617260238	-0,696049234	0,5592667	1 gamma-tubulin complex component, putative
PFIT_0517400	12055,47925	22236,8591	27114,72763	26420,36256	18958,69935	28185,2305	30478,59984	28831,74354	22495,2264	29655,17169	1,318287319	0,398664838	0,5592779	1 pre-mRNA-splicing factor
PFIT_0612600	865,5410836	1231,851749	328,3575452	746,8698081	963,4639354	245,4149953	544,9945245	581,8736889	730,2498527	563,4341067	0,771563465	-0,374143264	0,5594848	1 conserved Plasmodium protein, unknown function
PFIT_0708300	557,5960129	129,9760589	134,5607758	112,9719037	519,3020519	105,6647896	136,080215	111,4226213	260,0119321	123,7514181	0,47594515	-1,071132773	0,5595754	1 Cg7 protein
PFIT_1346200	21,54348231	21,17587476	5,850468512	12,55243375	41,74453793	17,04270801	9,431302031	9,773914147	19,98491754	9,602608089	0,480492755	-1,057413417	0,5595904	1 G-beta repeat protein, putative
PFIT_0703400	2007,345646	1105,526703	346,6402593	436,8246945	3124,161219	545,3666562	633,2445649	492,605273	1260,97753	562,924919	0,446419469	-1,163528149	0,5597108	1 serine/threonine protein kinase, putative
PFIT_1247000	92,51024759	128,5156537	122,1285302	123,6414724	462,5294803	381,7566593	105,0916512	93,82957581	218,5136739	99,46061351	0,455168831	-1,135526327	0,5598971	1 signal recognition particle receptor, beta subunit
PFIT_0209100	5823,076543	13405,78913	12686,00966	12135,06533	11397,92864	14145,44764	15646,53007	14464,08975	11598,88616	15055,30991	1,297996179	0,376286136	0,5600699	1 ATP-dependent RNA helicase UAP56
PFIT_1305800	771,7635723	2584,917125	3061,257649	2413,205388	2290,940242	2246,228915	2918,987979	3183,689635	2228,052149	3051,338807	1,3695096	0,453659379	0,5600962	1 vacuolar ATP synthase subunit h, putative
PF_apicoplast_ge	7,603581993	10,95303867	25,59579974	16,94578556	10,0186891	3,408541601	24,25191951	20,85101685	12,42090611	22,55146818	1,81560572	0,860450938	0,5601216	1 probable protein, unknown function
PFIT_1412400	329,4885531	251,9198893	267,6589344	296,8650582	759,7505903	190,8783297	231,0668998	226,1032139	349,4268925	228,5850568	0,654171335	-0,612259551	0,5601953	1 plastid replication-repair enzyme
PFIT_1146400	44,3542283	18,98526702	13,16355415	16,31816387	88,49842041	10,2256248	16,16794634	10,42550842	31,92420976	13,29672738	0,41650921	-1,263579698	0,560275	1 bin3, bicoid-interacting 3, putative
PFIT_0200500	3,801790997	14,60405156	3,65654282	4,393351812	5,009344552	0	11,45229532	9,773914147	5,244180289	10,61310474	2,02378716	1,017057571	0,560658	1 rifin (RIF)
PFIT_1249500	2156,882759	1456,754143	1564,269018	1327,419869	1833,420106	2086,02746	1199,796351	1095,981573	1737,462226	1147,888962	0,660669881	-0,597998519	0,5607852	1 protein phosphatase, putative
PFIT_0706800	466,3530289	1038,348066	1144,497903	1083,902654	743,0527752	732,8364442	1287,372727	1198,933469	868,1651451	1243,153098	1,431931591	0,517962572	0,5607986	1 large ribosomal subunit processing protein, putative
PFIT_0704100	5812,938434	10094,32044	8123,375528	6357,807694	1501,133584	1711,087884	10830,50305	7247,683137	5600,110593	9039,093095	1,614091891	0,690722714	0,560858	1 conserved Plasmodium protein, unknown function
PFIT_0917700	2434,413502	4047,512889	5549,169383	5686,252489	3746,989725	6397,832585	5605,561729	6407,778115	4643,695095	6006,669922	1,293510835	0,371292139	0,5609849	1 pre-mRNA-splicing factor ATP-dependent RNA helicase PRP43, putative
PFIT_0805500	0	13,87384898	7,313085639	9,414325312	21,70715972	6,817083202	12,79962418	16,94145119	9,854250476	14,87053769	1,509048072	0,593638765	0,5611555	1 conserved Plasmodium protein, unknown function
PFIT_0805000	129,2608939	194,2338857	229,6308891	183,2655327	656,2241363	279,5004113	169,7634366	139,4411752	278,6859582	154,6023059	0,554754559	-0,850078476	0,5612047	1 1-cys peroxiredoxin
PFIT_1426000	0	5,841620623	3,65654282	4,393351812	11,68847062	6,817083202	11,45229532	4,561159935	5,399511513	8,006727629	1,482861479	0,568383835	0,5613504	1 unspecified product
PFIT_1409600	21675,27774	15486,86647	22051,87844	19446,85799	16367,19843	40790,01734	22703,83865	27813,30169	22636,3494	25258,57017	1,115841151	0,158131662	0,5613968	1 40S ribosomal protein S8e, putative
PFIT_1354800	3378,524932	5515,950273	9242,277631	9928,347474	5662,229125	8027,11547	9007,567104	10036,50664	6959,074151	9522,036872	1,368290762	0,452374836	0,5613976	1 protein phosphatase, putative
PFIT_1136800	68,43223794	20,44567218	19,74533123	13,80767712	16,69781517	6,817083202	12,79962418	13,03188553	24,32430281	12,91575486	0,530981503	-0,913266491	0,5615686	1 conserved Plasmodium protein, unknown function
PFIT_1303200	145,7353215	500,1887658	106,7710503	139,9596363	734,7038676	102,256248	161,005799	130,3188553	288,2691483	145,6623271	0,505299745	-0,984788643	0,5617472	1 chromosome condensation protein, putative
PFIT_1431200	367,506463	329,3213626	399,2944759	370,2967956	357,3332447	289,7260361	462,8074639	471,7542562	352,2463963	467,2808601	1,326573856	0,407704998	0,5620545	1 conserved Plasmodium protein, unknown function
PFIT_0612400	181,2187042	308,1454878	122,8598387	197,0732099	798,1555652	197,6954129	160,3321345	138,7895809	300,8580365	149,5608577	0,497114385	-1,008350244	0,5621428	1 apicoplast ribosomal protein L18 precursor, putative
PFIT_1462500	964,3876495	1116,479741	692,5492101	466,9505355	1195,563566	2495,052452	1497,55603	763,668492	1155,163859	1130,612261	0,97874622	-0,030993264	0,5624027	1 40S ribosomal protein S28e, putative
PFIT_1319700	1438,34426	182,5506445	151,3808727	156,2778002	265,4952612	548,7751978	113,8492888	182,4463974	457,1373395	148,1478431	0,324077318	-1,625590042	0,5626112	1 SNARE protein, putative
PFIT_0911300	3217,582447	5264,760586	1472,124139	5127,041565	2471,276645	797,5987346	2535,672917	2816,842057	3058,397353	2676,257487	0,875052251	-0,19255893	0,5626219	1 conserved Plasmodium protein, unknown function
PFIT_1132900	74,76855627	139,4686924	88,48833624	143,0977447	61,78191614	54,53666562	148,8798392	141,395958	93,69031856	145,1378986	1,54912376	0,631452406	0,5627568	1 conserved Plasmodium protein, unknown function
PFIT_1036900	120,3900482	408,183241	398,5631673	357,1167402	233,7694124	170,4270801	439,2292089	418,9751198	281,4082815	429,1021643	1,524838437	0,608656392	0,5627723	1 enhancer of rudimentary homolog, putative
PFIT_1345300	3215,04792	7735,766109	10876,75227	8962,437697	9180,458782	14261,33806	10474,13457	11418,5381	9038,633473	10946,33634	1,211060983	0,276271513	0,5628915	1 Sec61 alpha subunit, PfSec61
PFIT_0612000.1	0	3,651012889	9,507011331	5,0209735	33,39563034	10,2256248	1,347328862	4,561159935	10,30004214	2,954244398	0,286818671	-1,801789153	0,5630103	1 conserved Plasmodium protein, unknown function
PFIT_1303400.2	140,6662669	267,2541435	97,264039	167,5749906	343,9749925	129,5245808	125,3015841	137,4863923	191,0431689	131,3939882	0,687771193	-0,539999404	0,5632605	1 CorA-like Mg2 transporter protein, putative
PFIT_1432900	4641,986807	7632,077343	10481,84565	9354,07363	7265,219381	20069,49295	9569,403239	12755,60956	9907,449293	11162,5064	1,126678126	0,172075419	0,5633831	1 60S ribosomal protein L14, putative
PFIT_1031300	95,04477492	33,58931858	28,52103399	33,26394944	33,39563034	40,90249921	29,64123495	22,80579968	44,11953441	26,22351732	0,594374298	-0,750556362	0,5634574	1 conserved Plasmodium protein, unknown function
PFIT_1322700	1472,560379	2664,509206	2400,886015	1796,25327	2028,784543	1864,472256	2729,688274	2704,767842	2037,910945	2717,228058	1,3333994	0,415044648	0,5634608	1 PEXEL protease
PFIT_1118800	139,3990032	211,028545	285,9416485	283,6850027	419,1151608	630,5801962	179,868403	208,5101685	328,2915927	194,1892857	0,591514647	-0,757514201	0,5636002	1 conserved Plasmodium protein, unknown function
PFIT_1037100	45,62149196	98,57734801	230,3621976	233,4752677	153,6198996	156,7929136	206,8149803	227,4064025	153,0748531	217,1106914	1,418330228	0,504193473	0,5636931	1 conserved Plasmodium protein, unknown function
PFIT_0110900	0	2,190607733	0	0,627621687	215,4018157	105,6647896	3,368322154	4,561159935	53,9808058	3,964741045	0,073447237	-3,767147968	0,5637179	1 5.8S ribosomal RNA
PFIT_0316000	691,9259614	995,2661136	474,619258	1625,540171	641,1961026	409,0249921	504,5746587	887,4714045	806,2620997	696,0230316	0,863271425	-0,21211386	0,5637752	1 conserved Plasmodium protein, unknown function
PFIT_1228500	8536,288051	3782,449353	3663,124597	3224,72023	3842,167271	5136,672193	3265,92516	2899,59453	4697,570283	3082,759845	0,656245604	-0,607692242	0,5639342	1 conserved Plasmodium protein, unknown function
PFIT_1115000	3462,164334	4713,45764	5329,045505	6519,73409	3823,799674	3688,042012	5846,05993	7146,686024	4589,373876	6496,372977	1,415524896	0,501337123	0,5644646	1 GTPase activator, putative
PFIT_1017100	3752,367714	1143,497237	1344,876449	1674,494662	834,8907586	1680,411009	1363,496808	996,939243	1738,422972	1180,218025	0,678901536	-0,558725746	0,5645398	1 conserved Plasmodium protein, unknown function
PFIT_1351000.1	381,4463633	667,4051561	883,4207452	890,5951746	998,5293473	957,8001899	980,8554112	1021,048231	796,5328294	1000,951821	1,256635991	0,329566805	0,5651343	1 conserved Plasmodium protein, unknown function
PFIT_0624300	3109,865035	4265,84346	3760,388636	3686,649792	3648,472615	4823,086365	5173,742828	4519,457902	3882,384317	4846,600365	1,248356672	0,32003019	0,5657146	1 conserved Plasmodium protein, unknown function
PFIT_1459900	86,17392926	232,2044197	74,59347352	112,9719037	347,3145556	81,80499842	92,29202702	102,3003014	155,84388	97,29616421	0,624318159	-0,679646667	0,5657147	1 exonuclease V, mitochondrial, putative
PFIT_0710800	12671,36939	5431,246774	6703,905606	4226,404444	2442,89036	10304,02126	4990,506103	4026,852629	6963,306306	4508,679366	0,647491173	-0,62706757	0,5659477	1 erythrocyte membrane protein 1, PfEMP1
PFIT_1413400	5000,622424	2138,76335	1933,579843	1816,337164	1160,498154	1837,203923	1564,248808	1694,145119	2314,50081	1629,196964	0,703908574	-0,506540037	0,566129	1 conserved Plasmodium protein, unknown function
PFIT_1144100	906,0935209	1957,673111	2044,007436	2021,569455	1840,099232	3197,212022	2533,651924	2291,005476	1994,442463	2412,3287	1,209525341	0,274440996	0,5661818	1 translation initiation factor eIF-1A, putative
PFIT_1030300	7,603581993	85,43370161	34,37150251	55,2307085	60,11213462	17,04270801	72,08209409	63,85623909	43,2990562	67,96916659	1,569760927	0,650544855	0,56638	1 28 kDa ookinete surface protein
PFIT_0610100	5,069054662	18,98526702	26,3271083	22,59438075	83,48907586	17,04270801	16,16794634	10,42550842	28,91793243	13,29672738	0,459809062	-1,120893196	0,5664216	1 transcription factor with AP2 domain(s), putative
PFIT_1439900	46,88875563	86,89410676	88,48833624	75,3146025	268,8348243	64,76229042	54,56681889	61,90145626	105,1971526	58,23413758	0,553571424	-0,853158624	0,5665218	1 small subunit DNA primase
PFIT_0422100	504,3709389	535,9686921	317,3879168	498,9592416	385,7195305	211,3295793	328,7482422	316,0232241	408,9559832	322,3857332	0,788314015	-0,343157671	0,5665307	1 probable protein, unknown function
PFIT_0923800	5,069054662	5,111418045	8,044394203	3,138108437	11,68847062	0	6,736644308	11,72869698	5,508574328	9,232670642	1,676054473	0,745069038	0,5666074	1 conserved protein, unknown function
PFIT_1341900	107,7174116	441,7725596	531,661326	495,8211331	581,083968	518,0983234	617,75083	537,5652781	446,0257869	577,6577806	1,295121936	0,373087934	0,5667009	1 transcription activator, putative
PFIT_0604000	3890,499453	3005,51381	4825,905213	4864,6957	2092,236241	4127,743879	4764,154855	5468,830762	3801,099049	5116,492808	1,346056165	0,428738608	0,5668029	1 RNA binding protein, putative
PFIT_0111500	74,76855627	23,36648249	29,25234256	29,49821931	21,70715972	37,49395761	24,25191951	18,24463974	36,01445299	21,24827962	0,589993124	-0,761229954	0,567398	1 surface-associated interspersed protein 1.1 (SURFIN 1.1) (SURF1.1)
PFIT_0103500	264,8581061	342,465009	258,1519231	277,4087859	621,1587244	259,0491617	443,2711955	370,105549	337,1819517	406,6883722	1,206139208	0,270396428	0,5674165	1 unspecified product
PFIT_0209500	1449,749633	471,7108653	375,8926019	562,349032	1070,329953	688,5254034	357,0421483	591,647603	769,7595814	474,3448757	0,616224711	-0,698471558	0,5675958	1 conserved Plasmodium protein, unknown function
PFIT_1313200	108,9846752	52,5745856	62,8925365	49,58211331	118,5544877	61,35374882	52,5458256	39,74725086	75,65702453	46,14653823	0,609943869	-0,713251613	0,5678599	1 conserved Plasmodium protein, unknown function
PFIT_0209300	781,9016817	576,1298339	741,5468838	429,2932342	652,8845732	1083,916229	815,8076257	775,397189	710,945406	795,6024073	1,119076656	0,162308863	0,5679831	1 secretory complex protein 61 gamma subunit
PFIT_1143800	4406,275765	6823,012887	1769,766725	5157,167406	3748,659506	1267,977476	3313,755335	3145,245572	3862,143294	3229,500454	0,836193846	-0,25809067	0,5684084	1 transcription factor with AP2 domain(s)
PFIT_1351000.2	296,5396977	606,0681396	723,2641697	753,146025	701,3082372	678,2997786	799,6396793	850,3305308	626,4376746	824,9851051	1,316946822	0,397197092	0,5684241	1 conserved Plasmodium protein, unknown function
PFIT_0208500	245,8491511	654,2615097	751,0538952	592,474873	541,0092116	579,4520722	775,3877598	747,3786351	560,6834521	761,3831975	1,357955535	0,441436241	0,5684663	1 3'exoribonuclease, putative
PFIT_0606100	45,62149196	37,97053405	27,78972543	29,49821931	61,78191614	17,04270801	23,57825508	22,1542054	36,61743248	22,86623024	0,624462959	-0,679312096	0,5685472	1 RING zinc finger protein, putative
PFIT_0707000	88,70845659	205,9171269	191,6028438	175,7340725	166,9781517	92,03062323	238,4772085	207,2069799	153,4952125	222,8420942	1,451785307	0,537828121	0,5689365	1 DNA-directed RNA polymerase 2 8.2 kDa polypeptide, putative
PFIT_0710000	529,7162122	1002,568139	1214,703525	1388,299173	863,2770464	429,4762417	1390,443385	1346,845369	904,6733892	1368,644377	1,512860214	0,597278691	0,569089	1 conserved Plasmodium membrane protein, unknown function
PFIT_0712400	12969,17635	2208,862798	2262,668697	2870,113977	2441,220578	2754,101614	1747,485533	2023,851823	4251,024003	1885,668678	0,443579871	-1,172734196	0,5691117	1 zinc finger protein, putative
PFIT_1119600	10,13810932	44,54235725	21,20794835	26,36011087	36,73519338	13,6341664	33,68322154	43,00522225	25,43631426	38,34422189	1,507459827	0,592119555	0,5691514	1 conserved Plasmodium protein, unknown function
PFIT_0802800	254,7199968	964,5976053	1240,299324	1322,398896	766,4297164	402,2079089	1158,029157	1410,050014	825,1089079	1284,039585	1,556206185	0,638033218	0,569311	1 Plasmodium exported protein (hyp13), unknown function
PFIT_1228800	43185,8112	47040,38027	56797,07962	54004,33572	31734,19773	47920,68637	56573,66523	68964,08663	46780,41515	62768,87593	1,341776804	0,424144708	0,5693179	1 ABC transporter, (CT family)
PFIT_0929100	320,6177074	385,5469611	456,3365439	385,9873378	312,2491437	460,1531161	444,6185243	550,5971636	386,815135	497,607844	1,28642289	0,363364983	0,5696493	1 guanylate kinase
PFIT_0409800	157,1406945	100,7679557	87,75702767	87,22941456	88,49842041	279,5004113	81,51339612	67,11421048	133,4839874	74,3138033	0,556724479	-0,844964576	0,5697344	1 conserved Plasmodium protein, unknown function
PFIT_1447600	86,17392926	165,7559852	205,4977065	146,8634749	103,5264541	242,0064537	169,0897721	233,9223452	158,3040006	201,5060587	1,272905662	0,348125502	0,5697603	1 pyruvate dehydrogenase E1 beta subunit
PFIT_0727400	3,801790997	5,111418045	7,313085639	6,903838562	5,009344552	3,408541601	5,389315446	13,68347981	5,258003233	9,536397626	1,813691853	0,858929362	0,5699036	1 conserved Plasmodium protein, unknown function
PFIT_0611000	0	11,68324125	10,2383199	10,041947	15,02803365	10,2256248	10,10496646	18,89623402	9,536194433	14,50060024	1,520585632	0,604627064	0,5702147	1 conserved Plasmodium protein, unknown function
PFIT_1131000	26887,53319	22312,06997	32750,92273	27606,56755	18968,71804	43997,45499	30734,59233	38218,61069	28753,87774	34476,60151	1,199024417	0,261861039	0,5704247	1 60S ribosomal protein P0
PFIT_0924700	793,3070547	374,5939224	212,0794835	273,0154341	731,3643045	259,0491617	233,0878931	323,8423554	440,5682268	278,4651242	0,632059026	-0,661868802	0,5705296	1 conserved Plasmodium protein, unknown function
PFIT_1364600	453,6803923	224,1721914	95,07011331	124,2690941	272,1743873	92,03062323	157,6374768	106,8614613	210,2328003	132,2494691	0,629062016	-0,668725844	0,5705586	1 DNA topoisomerase VI, b subunit, putative
PFIT_1218100	1259,660084	2229,30847	2506,194449	2379,313817	1658,093047	1677,002468	2898,104381	2567,933044	1951,595389	2733,018712	1,400402321	0,485841358	0,570583	1 triose or hexose phosphate/phosphate translocator, putative
PFIT_0608000	2002,276592	3758,352668	5344,402985	5510,518416	3852,18596	5150,306359	5112,439365	6184,281278	4269,67383	5648,360322	1,322902064	0,403706261	0,5708264	1 Zn2 or Fe2 permease
PFIT_0810000	4109,736067	2549,867402	1776,348502	1671,356554	1678,130425	2011,039545	1564,248808	1674,597291	2299,413082	1619,423049	0,704276696	-0,505785748	0,5709163	1 RNA helicase, putative
PFIT_1142000	214,1675595	229,2836094	101,6518904	153,7673134	861,6072629	149,9758304	113,1756244	114,0289984	285,0755777	113,6023114	0,398498925	-1,327352261	0,5709428	1 conserved Plasmodium protein, unknown function
PFIT_1148900	5256,609685	3291,753221	2472,554255	1801,274243	2479,625553	7464,706106	3242,346905	4348,088607	3794,42051	3795,217756	1,0002101	0,000303093	0,5711207	1 Plasmodium exported protein (PHISTc), unknown function
PFIT_1431500	5869,965299	3527,608653	4314,720527	3558,614968	2315,986964	3493,755141	2812,548999	2895,684965	3846,775259	2854,116982	0,74195054	-0,430605077	0,5711944	1 acid phosphatase, putative
PFIT_0526200	259,7890514	280,3977899	163,8131183	139,9596363	41,74453793	54,53666562	216,9199467	253,4701735	156,7067999	235,1950601	1,50086059	0,585789976	0,571565	1 conserved Plasmodium protein, unknown function
PFIT_0209000	5025,967698	4306,004601	4572,87245	4045,649398	3449,768615	5037,824486	6339,855958	4677,143716	4406,347875	5508,499837	1,250128223	0,322076076	0,5719343	1 RING zinc finger protein, putative
PFIT_0218600	1591,683164	1815,283608	1325,131118	1141,016228	2528,049217	2597,3087	2148,989534	1848,572962	1833,078673	1998,781248	1,09039578	0,124851883	0,5719352	1 40S ribosomal protein S30, putative
PFIT_1221400	596,8811865	261,4125229	294,7173513	293,7269497	230,4298494	432,8847833	198,0573427	266,5020591	351,6754405	232,2797009	0,660494519	-0,598381506	0,5719518	1 FbpA domain protein, putative
PFIT_1419700	0	0	0	1,255243375	50,09344552	47,71958241	1,347328862	1,303188553	16,51137855	1,325258707	0,080263359	-3,639114653	0,5730274	1 5S ribosomal RNA
PFIT_1434500	181,2187042	511,8720071	266,9276258	308,7898702	702,9780187	456,7445745	249,9295038	285,3982931	404,7551334	267,6638985	0,661298342	-0,596626811	0,5731962	1 chromatin assembly factor 1 P55 subunit, putative
PFIT_0201800	28315,73934	22785,97144	26590,37939	24893,98661	21610,3124	39658,38153	31004,73176	33281,48086	27309,12845	32143,10631	1,177009598	0,235126085	0,5733045	1 early transcribed membrane protein 2
PFIT_0219000	204,0294502	320,5589317	208,4229407	315,6937088	861,6072629	214,7381209	224,3302555	198,08466	354,1750692	211,2074577	0,596336321	-0,745801885	0,5734125	1 replication factor C subunit 1, putative
PFIT_0926100	2818,394392	3290,292816	4798,115488	3179,531469	1930,267434	1966,728504	4326,946639	4292,051499	2997,221684	4309,499069	1,437831273	0,523894388	0,5736754	1 protein kinase, putative
PFIT_0213600	91,24298392	242,4272558	427,8155099	355,2338751	232,0996309	385,1652009	340,2005375	430,7038167	288,9974094	385,4521771	1,333756513	0,415495316	0,5738292	1 mitochondrial ribosomal protein L13 precursor, putative
PFIT_0533700	231,9092508	492,1565375	687,4300501	512,7669187	679,6010775	1489,53268	668,9487798	754,5461721	682,2327524	711,747476	1,043261956	0,061101455	0,5739619	1 cell cycle regulator protein, putative
PFIT_1426400	1134,200981	1201,183241	454,1426182	1000,42897	1357,532373	518,0983234	652,7808334	734,9983438	944,2644177	693,8895886	0,734846697	-0,444484787	0,5740984	1 DNA-directed DNA polymerase, putative
PFIT_0212000	762,8927267	2618,506444	538,2431031	1646,251686	1051,962356	374,9395761	928,9832501	985,210546	1165,465982	957,096898	0,821213929	-0,284169997	0,5748022	1 secreted protein altered thrombospondin repeat protein
PFIT_1234100	155,8734309	82,51289129	50,46029091	47,69924825	30,05606731	64,76229042	102,3969935	78,84290745	71,89403651	90,61995047	1,260465469	0,333956596	0,5749156	1 aspartyl protease, putative
PFIT_1310600	754,021881	1062,444751	1337,563363	1180,556394	1210,5916	1114,593104	1432,2108	1449,797265	1109,961849	1441,003922	1,298263453	0,376564173	0,5751829	1 clathrin-adaptor medium chain, putative
PFIT_0526400	84,90666559	200,0755063	242,0631347	237,8686196	227,0902863	398,7993673	232,4142286	315,3716298	231,8005966	273,8929292	1,18158543	0,240727742	0,5753168	1 conserved Plasmodium protein, unknown function, putative
PFIT_1239500	1202,633219	1694,800183	1192,032959	1245,82905	3334,55369	2743,875989	1215,290633	1080,34331	1902,287515	1147,816972	0,603387744	-0,728842702	0,5754546	1 conserved Plasmodium protein, unknown function
PFIT_1252400	271,1944244	1247,186003	425,6215842	1153,568662	702,9780187	224,9637457	629,2025784	541,4748437	670,9187396	585,338711	0,872443526	-0,196866347	0,5756202	1 chitinase
PFIT_1026200	21368,59993	24702,02301	27462,8305	26473,08278	20888,96678	42245,4646	28551,91957	36155,66321	27190,16127	32353,79139	1,189908036	0,250850077	0,575683	1 40S ribosomal protein S28, putative
PFIT_1135500	738,814717	1499,836095	2369,439747	2274,500995	1509,482492	2130,338501	2066,128809	2605,073917	1753,735424	2335,601363	1,331786614	0,413362945	0,5757324	1 coatomer delta subunit, putative
PFIT_1033600	24,07800965	27,01749538	21,93925692	24,47724581	105,1962356	44,31104081	13,47328862	25,41217678	41,16988069	19,4427327	0,472256231	-1,082358262	0,5757324	1 apicoplast ribosomal protein L27 precursor, putative
PFIT_0928900	337,092135	185,4714548	189,4089181	245,4000798	255,4765721	115,8904144	157,6374768	167,4597291	221,4565957	162,5486029	0,733997569	-0,44615281	0,5759091	1 conserved Plasmodium protein, unknown function
PFIT_0730700	2472,431412	2071,584713	1570,850795	2052,322918	1225,619634	415,8420753	1321,055949	1428,294654	1634,775258	1374,675301	0,840895588	-0,25000142	0,5759559	1 alpha/beta hydrolase, putative
PFIT_1475200	3,801790997	4,381215467	13,16355415	8,786703625	46,75388248	13,6341664	5,389315446	5,212754212	15,08688552	5,301034829	0,351367105	-1,508949088	0,5760573	1 mitochondrial ribosomal protein L20 precursor, putative
PFIT_0104000	16,47442765	16,06445671	24,13318261	21,33913737	58,4423531	47,71958241	15,49428191	14,98666836	30,69552331	15,24047513	0,496504815	-1,010120385	0,5761861	1 conserved Plasmodium protein, unknown function
PFIT_0901200	158,4079582	4,381215467	2,193925692	1,882865062	0	0	1,347328862	0	27,81099407	0,673664431	0,02422959	-5,367481375	0,5761956	1 rifin (RIF)
PFIT_0902100	19,00895498	138,7384898	171,126204	135,5662845	133,5825214	98,84770643	94,31302031	87,96522732	116,1450268	91,13912382	0,784701044	-0,349784975	0,5763138	1 probable protein, unknown function
PFIT_0615000	39,28517363	95,65653769	68,74300501	56,48595187	141,931429	272,6833281	44,46185243	55,3855135	112,4642375	49,92368297	0,443907184	-1,171670039	0,5763657	1 pyridoxine kinase
PFIT_1301340	1529,587244	5790,506442	5929,449836	5669,934325	5366,677796	5484,343436	6213,207045	6941,433827	4961,749847	6577,320436	1,325605006	0,406650956	0,5768682	1 gametocyte associated protein, pseudogene (GAP)
PFIT_0419200	96,31203858	223,4419888	65,08646219	105,4404435	499,2646736	132,9331224	101,0496646	81,44928456	187,0797882	91,24947459	0,487756991	-1,035765543	0,5771699	1 conserved Plasmodium protein, unknown function
PFIT_0308200	1703,202367	767,4429093	208,4229407	525,3193524	520,9718334	136,341664	380,6204034	363,5896063	643,6168444	372,1050048	0,578146778	-0,790492289	0,5772397	1 conserved Plasmodium protein, unknown function
PFIT_1111900	91,24298392	41,62154694	51,19159948	58,36881694	227,0902863	71,57937362	47,83017459	42,35362797	90,18243454	45,09190128	0,500007585	-0,999978115	0,577629	1 replication factor C subunit 5, putative
PFIT_1314300	13,93990032	89,08471649	69,47431357	91,00514469	51,76322703	30,67687441	98,35500689	86,66203877	57,65736242	92,50852283	1,604452909	0,682081446	0,5778934	1 serine/threonine protein kinase
PFIT_0516300	6563,158524	10076,06537	13957,02394	11518,74083	12548,4081	21027,29314	13709,74483	15366,99999	12615,11498	14528,37241	1,151663891	0,203719733	0,5783336	1 40S ribosomal protein S14, putative
PFIT_1105300	86,17392926	60,60681396	55,57945086	73,43173744	50,09344552	13,6341664	69,38743637	97,73914147	56,56859057	83,56328892	1,476733058	0,56240906	0,578341	1 conserved Plasmodium protein, unknown function
PFIT_bin05800	0	7,302025778	8,775702767	6,276216875	0	3,408541601	9,431302031	9,12231987	4,293747837	9,276810951	2,160539301	1,111391474	0,5785616	1 stevor
PFIT_0217000	1392,722768	1223,089318	818,3342831	766,3260804	1108,734927	1312,288516	736,9888873	744,772258	1103,582649	740,8805726	0,67134126	-0,57488177	0,5785864	1 conserved Plasmodium protein, unknown function
PFIT_1122500	2383,722955	3637,869243	4846,381853	4362,59835	3554,96485	4379,975957	5202,710399	4864,151274	3960,918868	5033,430836	1,303687285	0,382597852	0,5790024	1 protein GCN20
PFIT_1106400	10187,53261	18915,16758	21942,18215	22374,08558	18046,99864	26141,51408	25219,30163	24369,62594	19601,5801	24794,46379	1,264921688	0,33904807	0,5790161	1 translocon component PTEX88
PFIT_1127000	604,4847685	510,4116019	356,1472706	439,3351812	694,6291112	610,1289466	363,1051282	344,047178	535,8561467	353,5734531	0,659829052	-0,599835795	0,5791933	1 autophagy-related protein 7, putative (ATG7)
PFIT_1454400	211,6330322	240,9668507	110,4275932	140,587258	586,0933125	122,7074976	149,5535036	110,771027	235,4025907	130,1622653	0,552934719	-0,854818933	0,5803071	1 conserved Plasmodium protein, unknown function
PFIT_0806200	247,1164148	311,7965007	131,6355415	225,3161858	848,2490107	231,7808289	201,4256648	150,5182779	332,6490804	175,9719713	0,529001827	-0,91865539	0,58033	1 DNA repair protein RAD54, putative
PFIT_0220600	73,5012926	82,51289129	108,964976	98,53660494	76,80994979	211,3295793	63,32445649	71,02377613	108,6092157	67,17411631	0,618493706	-0,693169181	0,5803334	1 Plasmodium exported protein (hyp15), unknown function
PFIT_0835400	1,267263666	8,032228356	15,35747984	21,33913737	8,348907586	23,85079121	3,368322154	10,42550842	13,03413467	6,896915289	0,529142552	-0,918271654	0,5811224	1 stevor
PFIT_0316800	5511,329682	7331,233881	9752,731009	9595,70798	8290,465233	13085,39121	9597,697145	11960,66454	8927,809832	10779,18084	1,207371242	0,271869343	0,5816258	1 40S ribosomal protein S15A, putative
PFIT_1232300	1231,780283	2203,021177	628,925365	1635,582118	2331,014998	443,1104081	1112,89364	1103,800704	1412,239058	1108,347172	0,784815549	-0,34957447	0,5817729	1 CG2-related protein, putative
PFIT_1351400	1311,617894	2662,318599	3061,988957	2952,96004	2302,628712	1091,966213	3349,45955	3244,939497	2365,580069	3297,199523	1,393822837	0,479047198	0,5821397	1 conserved Plasmodium protein, unknown function
PFIT_1253800	0	2,190607733	1,462617128	0	1,669781517	0	2,694657723	1,954782829	0,88716773	2,324720276	2,620384171	1,389778339	0,5824527	1 Plasmodium exported protein, unknown function, pseudogene
PFIT_0907000	759,0909357	660,8333329	929,4931848	692,2667213	711,3269263	1141,861436	897,9946862	1031,47374	815,8120896	964,7342129	1,182544639	0,241894644	0,5824748	1 probable protein, unknown function
PFIT_0317500	2050,432611	1607,906076	2949,367438	3042,08219	1768,298627	2546,180576	3131,192274	2985,606471	2327,377941	3058,398625	1,31409625	0,394070948	0,5825356	1 kinesin-5, putative
PFIT_1030800.1	196,4258682	394,309392	97,264039	208,3704002	497,5948921	136,341664	199,4046715	156,3826264	255,0510426	177,8936489	0,69748254	-0,519770993	0,5828539	1 OTU-like cysteine protease, putative
PFIT_1321000	370,0409903	1417,323204	1660,07044	1769,265537	864,9468259	1407,727681	1630,267922	1879,849488	1248,229113	1755,058705	1,406038913	0,491636522	0,5829574	1 SFT2-like protein, putative
PFIT_1021900	11390,16583	2215,434621	2070,334545	1692,695691	1304,099365	4751,506992	1900,407359	1376,167112	3904,039507	1638,287236	0,41963	-1,25277933	0,5831773	1 pre-mRNA splicing factor, putative
PFIT_0103600	6,336318328	5,841620623	4,387851384	9,414325312	30,05606731	3,408541601	16,16794634	9,773914147	9,907454093	12,97093024	1,309208152	0,388695667	0,5835607	1 conserved Plasmodium protein, unknown function
PFIT_0724400	760,3581993	497,2679555	1126,946497	666,5342321	758,6292441	351,0797849	1206,532996	555,1583235	593,4693188	880,8456595	1,484231167	0,569715807	0,5837481	1 unspecified product
PFIT_1330300	647,5717331	422,7872926	129,4416158	202,7218051	258,8161352	109,0733312	241,1718662	158,0890035	295,0686522	200,0804348	0,678087096	-0,56047056	0,5837496	1 protein kinase, putative
PFIT_0201700	538,5870579	212,4889501	208,4229407	194,5627231	180,3364039	354,4883265	184,584054	176,5820489	281,481067	180,5830515	0,641545996	-0,640375391	0,583871	1 conserved Plasmodium protein, unknown function
PFIT_1478500	2,534527331	8,762430934	11,70093702	12,55243375	5,009344552	0	9,431302031	18,24463974	6,759945598	13,83797089	2,047053587	1,033548869	0,5839598	1 probable protein, unknown function
PFIT_0400600	873,1446656	198,6151012	253,0327631	370,9244173	60,11213462	109,0733312	222,3092622	142,0475523	310,8170688	182,1784072	0,586127422	-0,77071376	0,5839988	1 acyl-CoA synthetase
PFIT_1125750	686,8569067	430,8195209	401,4884016	440,5904246	1023,57607	541,9581146	368,4944436	385,0922174	587,5482397	376,7933305	0,641297693	-0,640938778	0,5840887	1 hypothetical protein
PFIT_1404900	1064,501479	624,323204	381,7430704	260,4630003	541,0092116	824,8670675	388,7043766	372,0603319	616,1511721	380,3823542	0,617352318	-0,695834037	0,5846044	1 phosphatase, putative
PFIT_0101100	301,6087524	119,7532228	358,3411963	430,5484776	182,0061854	1438,404556	164,3741211	158,0890035	471,777065	161,6815623	0,34270755	-1,544950118	0,5846205	1 serine/threonine protein kinase, FIKK family
PFIT_1238100	10,13810932	10,22283609	19,74533123	15,0629205	23,37694124	10,2256248	9,431302031	7,167537041	14,79529386	8,299419536	0,560949962	-0,834056009	0,5846601	1 conserved Plasmodium protein, unknown function
PFIT_1029000	2300,083553	6258,566295	4663,554712	4114,687783	4406,553424	6312,619045	5573,225836	6031,156623	4676,010802	5802,191229	1,240842136	0,311319582	0,5848253	1 adenosine deaminase (ADA)
PFIT_1426900	1229,245756	2476,847144	2616,622042	2117,595574	2536,398125	3302,876811	2941,892569	2803,158577	2379,930909	2872,525573	1,206978557	0,271400045	0,5848821	1 conserved Plasmodium protein, unknown function
PFIT_1124900	716,0039711	1567,744935	1744,902224	1617,381089	1664,772173	930,5318571	2016,951306	1780,155563	1373,556043	1898,553435	1,382217671	0,466984828	0,5849449	1 endoplasmic reticulum oxidoreductin, putative
PFIT_1245700	127,9936302	100,0377532	48,99767378	51,46497837	447,5014466	78,39645682	55,24048332	46,26319363	142,3986565	50,75183848	0,356406723	-1,488403545	0,5850086	1 conserved Plasmodium protein, unknown function
PFIT_0726600	262,3235788	219,7909759	149,186947	161,9263954	240,4485385	224,9637457	139,4485372	140,7443637	209,7733635	140,0964504	0,667846709	-0,582411097	0,5850332	1 conserved Plasmodium protein, unknown function
PFIT_0410500	2665,055489	387,0073662	318,8505339	418,6236656	983,5013136	330,6285353	303,8226583	364,8927948	850,6111506	334,3577266	0,393079407	-1,347107311	0,585318	1 DNA polymerase alpha
PFIT_1453600	2875,421257	2541,104971	2092,273801	1834,538193	1726,554089	2198,509333	1690,224057	1550,142784	2211,400274	1620,18342	0,732650457	-0,448803034	0,5857573	1 conserved Plasmodium protein, unknown function
PFIT_1430400	269,9271608	121,9438305	93,60749618	97,90898325	126,9033953	228,3722873	101,0496646	86,66203877	156,4438589	93,85585169	0,599933116	-0,737126426	0,5857962	1 transcription factor with AP2 domain(s), putative
PFIT_0725300	6554,287678	4754,348084	4319,83687	4397,117543	3698,566061	4427,6954	3358,217187	3540,763298	4691,975915	3449,490243	0,735189248	-0,443812428	0,5859941	1 conserved Plasmodium membrane protein, unknown function
PFIT_0702000	30,41432797	101,4981583	134,5607758	122,3862291	110,2055801	102,256248	152,9218258	122,499724	100,2202199	137,7107749	1,374081748	0,458467836	0,5860163	1 conserved Plasmodium protein, unknown function
PFIT_bin05200	15,20716399	5,841620623	4,387851384	3,138108437	1,669781517	0	6,736644308	10,42550842	5,040754325	8,58107636	1,702339732	0,767518981	0,5861401	1 rifin (RIF)
PFIT_0733200	2,534527331	14,60405156	15,35747984	12,55243375	10,0186891	40,90249921	7,410308739	7,167537041	15,5949468	7,28892289	0,455701603	-1,133838651	0,5865823	1 rifin
PFIT_0929500	473,9566109	132,1666666	152,8434899	168,8302339	136,9220844	98,84770643	113,1756244	139,4411752	193,9277987	126,3083998	0,651316627	-0,618569038	0,5866191	1 conserved Plasmodium protein, unknown function
PFIT_0712800	0	0	0	0	0	0	0,673664431	0	0	0,336832215	#DIV/0!	#DIV/0!	0,5867001	1 tRNA Asparagine
PFIT_1338300	0	0	0	0	0	0	0,673664431	0	0	0,336832215	#DIV/0!	#DIV/0!	0,5867001	1 tRNA Methionine
PF_apicoplast_ge	0	0	0	0	0	0	0,673664431	0	0	0,336832215	#DIV/0!	#DIV/0!	0,5867001	1 unspecified product
PF_apicoplast_ge	0	0	0	0	0	0	0,673664431	0	0	0,336832215	#DIV/0!	#DIV/0!	0,5867001	1 unspecified product
PF_M7661102500	0	0	0	0	0	0	0,673664431	0	0	0,336832215	#DIV/0!	#DIV/0!	0,5867001	1 unspecified product
PFIT_0718200	221,7711415	242,4272558	492,9019721	425,5275041	282,1930764	453,3360329	460,1128062	450,9032393	353,0261638	455,5080228	1,290295365	0,367701354	0,5867245	1 3'-5' exoribonuclease Csl4 homolog, putative
PFIT_0411800	31,68159164	3,651012889	20,47663979	15,69054219	10,0186891	51,12812402	8,083973169	11,72869698	22,1077666	9,906335073	0,448092962	-1,158129962	0,5868369	1 rifin (RIF)
PFIT_1011500	5541,74401	2099,332411	1745,633542	2159,018605	1908,560274	1857,655173	1466,567466	2102,043136	2551,990669	1784,305301	0,69918175	-0,516260568	0,5869028	1 ubiquitin-protein ligase e3, putative
PFIT_0723500	4089,459849	1531,234806	1351,458226	1165,493474	1066,990389	2198,509333	1446,357533	980,6493861	1900,524346	1213,503459	0,638509821	-0,647219285	0,586906	1 conserved Plasmodium protein, unknown function
PFIT_0700400	55,75960129	16,06445671	21,20794835	20,71151569	8,348907586	13,6341664	10,10496646	17,59304546	22,62109934	13,84900596	0,61221631	-0,707886615	0,5869553	1 rifin (RIF)
PFIT_1328700	432,13691	929,5478816	914,1357049	636,4083911	626,168069	671,4826954	932,3515722	946,7664837	701,6466087	939,559028	1,339077274	0,421239216	0,5871993	1 AMP deaminase, putative
PFIT_1416600	769,229045	733,1233881	410,2641044	301,25841	836,5605401	204,673711	362,4314638	402,0336686	542,8515331	382,2325662	0,704122453	-0,506104105	0,5876662	1 conserved Plasmodium protein, unknown function
PFIT_0520200	15522,71264	18493,11049	26359,28588	22800,24066	18744,96731	43370,28333	24774,68311	30277,63124	24215,10005	27526,15718	1,136735224	0,184862551	0,5879027	1 40S ribosomal protein S9, putative
PFIT_0803500	276,2634791	300,8434621	241,3318261	204,6046701	227,0902863	115,8904144	328,7482422	291,2626416	227,6706897	310,0054419	1,361639666	0,44534497	0,5881892	1 Plasmodium exported protein, unknown function, pseudogene

PFIT_1304200	401,722582	414,0248616	280,8224886	377,8282559	874,965515	245,4149953	310,5593026	305,5977157	432,4631164	308,0785091	0,712381004	-0,489279048	0,634251	1	conserved Plasmodium protein, unknown function
PFIT_1360200	9866,9149	2930,302945	4186,01022	4489,377931	2831,949453	4771,958241	3452,530208	3553,14359	4846,085615	3502,836899	0,722817791	-0,468296079	0,6342908	1	conserved Plasmodium protein, unknown function
PFIT_0508300	67,16497428	382,6261508	413,1893386	269,8773256	480,897077	484,0129073	421,7139337	387,0470002	349,6279623	404,3804669	1,15660219	0,209892739	0,634328	1	triose phosphate transporter
PFIT_0829600	6,336318328	86,16390418	49,72898235	25,1048675	76,80994979	44,31104081	72,75575852	49,52116501	48,07584383	61,13846177	1,271708553	0,346768075	0,6345169	1	small nucleolar RNA snoR05
PFIT_1230900	229,3747235	581,9714545	115,5467531	246,0277015	287,202421	105,6647896	239,1508729	191,5687173	260,9646405	215,3597951	0,825245116	-0,277105399	0,6347529	1	conserved Plasmodium protein, unknown function
PFIT_1249800	173,6151222	117,562615	83,36917629	89,12227962	275,5139503	92,03062323	74,77675182	105,5582728	138,5356278	90,1675123	0,650861542	-0,619577424	0,6348318	1	leucine-rich repeat protein
PFIT_1135900	434,6714373	428,6289132	489,2454293	420,5065306	367,3519338	439,7018665	486,385719	577,3125289	430,0176851	531,849124	1,23680756	0,306621043	0,6352259	1	conserved Plasmodium protein, unknown function, pseudogene
PFIT_1138500	64,63044694	187,6620625	110,4275932	168,2026122	135,2523029	61,35374882	182,5630607	163,5501634	121,2547944	173,0566121	1,427214593	0,513202272	0,6352286	1	conserved Plasmodium protein, unknown function
PFIT_0613500	6185,513952	12265,2127	15009,37697	14902,87697	10905,34309	24197,23683	13295,44121	18531,34122	13910,92675	15913,39121	1,143949034	0,194022777	0,6352801	1	60S ribosomal protein L19, putative
PFIT_1222100	1234,31481	472,4410679	665,4907932	830,9711142	746,3923382	674,891237	435,8608867	694,5994987	770,7502268	565,2301927	0,733350667	-0,447424877	0,6353589	1	transcription factor with AP2 domain(s)
PFIT_1331700	1495,371125	1703,562614	2493,762203	2889,570249	2018,765854	3316,510978	2632,006931	2893,078587	2319,590504	2762,542759	1,190961402	0,252126658	0,6353621	1	nucleotidyltransferase, putative
PFIT_1108000	437,2059646	544,0009205	835,15438	664,0237454	721,3456154	1353,191016	718,7999476	920,7027126	759,1536069	819,7513301	1,079822743	0,110794508	0,6354101	1	conserved Plasmodium protein, unknown function
PFIT_1400500	0	4,381215467	3,65654282	1,255243375	0	6,817083202	0,673664431	0	2,685014144	0,336832215	0,125448954	-2,994827655	0,635419	1	rifin (RIF)
PFIT_0712300	8936,74337	4944,201654	4825,905213	5605,289291	4423,251239	4444,738248	4079,038128	4400,867743	5530,021503	4239,952936	0,766715452	-0,383236839	0,6357095	1	conserved Plasmodium protein, unknown function
PFIT_0925800	5232,531675	5076,368321	4708,164535	3674,72498	4394,864953	7870,322557	5655,412896	5624,561794	5159,49617	5639,987345	1,093127538	0,128461734	0,6359237	1	histone deacetylase
PFIT_1103800	6,336318328	64,98802943	61,42991937	48,32686994	43,41431945	44,31104081	56,58781219	68,41739903	44,80108289	62,50260561	1,395113724	0,48038273	0,6360361	1	conserved Plasmodium protein, unknown function
PFIT_1241500	29,14706431	18,98526702	19,01402266	7,53146025	40,07475641	23,85979121	13,47328862	11,0771027	23,10206031	12,27519566	0,53134636	-0,912275501	0,6361149	1	conserved Plasmodium protein, unknown function
PFIT_1368200	2800,652701	2514,087475	3533,682981	3205,89158	2731,762562	3268,791395	3652,608544	3703,010273	3009,144782	3677,809408	1,22210852	0,289493196	0,6361383	1	conserved Plasmodium protein, unknown function
PFIT_1359500	191,3568135	165,0257826	240,6005175	133,6834194	176,9968408	286,3174945	245,8875172	201,9942257	198,9968114	223,9408715	1,125349044	0,170372545	0,6366555	1	conserved Plasmodium protein, unknown function
PFIT_0506000	159,6752219	270,1749538	386,8622303	301,8860317	308,9095807	371,5310345	382,6413967	349,2545322	299,8398421	365,9479644	1,220478112	0,287446422	0,6368966	1	conserved Plasmodium protein, unknown function
PFIT_0903000	173,6151222	272,3655615	268,390243	330,1290076	858,2676998	313,5858273	240,4982018	233,9223452	369,3922436	237,2102735	0,64216365	-0,638987079	0,6369858	1	serine repeat antigen 9
PFIT_0628100	1091,114016	1352,335174	1647,638195	1312,356949	1626,367198	2140,564125	1746,811869	1720,860484	1528,395943	1733,836177	1,134415584	0,181949257	0,6370205	1	DnaJ protein, putative
PFIT_1356700	7,603581993	11,68324125	10,96962846	7,53146025	13,35825214	3,408541601	12,79962418	14,33507408	9,092450948	13,56734913	1,492155329	0,577397724	0,6372258	1	conserved Plasmodium protein, unknown function
PFIT_1102800	78,57034727	1,460405156	2,193925692	3,138108437	0	0	0	1,303188553	14,22713109	0,651594276	0,045799415	-4,448527029	0,6375484	1	Plasmodium exported protein, unknown function, pseudogene
PFIT_1023300	566,4668585	470,9806627	212,0794835	348,9576582	230,4298494	78,39645682	259,3608059	278,230756	317,8851615	268,795781	0,845575112	-0,241995181	0,6377662	1	conserved Plasmodium protein, unknown function
PFIT_0317800	680,5205884	280,3977899	314,4626825	228,4542942	237,1089754	555,592281	251,2768327	247,6058251	382,7561019	249,4413289	0,65169785	-0,617724858	0,6378193	1	proteasome regulatory protein, putative
PFIT_1339600	0	8,032228356	5,119159948	4,393351812	1,669781517	0	8,7576376	5,212754212	3,202420272	6,985195906	2,181223984	1,125137924	0,6378591	1	conserved Plasmodium protein, unknown function
PFIT_1345200	155,8734309	91,27532223	184,2897581	208,9980219	310,5793622	388,5737425	211,5306313	237,8319109	223,2649396	224,6812711	1,006343725	0,009123154	0,6380494	1	dynactin subunit 2, putative
PFIT_1453300	143,2007942	170,8674032	166,007044	141,8425014	118,5544877	68,17083202	118,5649398	106,2098671	134,7738438	112,3874034	0,833896254	-0,262060188	0,6380786	1	leucine-rich repeat protein
PFIT_0922400	13,93990032	45,27255982	12,43224559	26,98773256	28,38628579	10,2256248	18,86260406	14,98666836	22,87405815	16,92463621	0,739905272	-0,434587517	0,6383	1	conserved Plasmodium protein, unknown function
PFIT_1131200	2488,905839	3437,793736	2719,736549	2292,074403	3738,640817	3991,402215	3589,284087	3380,471106	3111,425593	3484,877597	1,120026011	0,163532238	0,6383848	1	26S proteasome regulatory subunit 6a, putative
PFIT_0614500	1074,639588	332,9723755	350,2968021	382,2216077	930,0683051	187,4697881	318,6432758	389,6533773	542,9447445	354,1483265	0,652271238	-0,616451657	0,6385966	1	cdc2-related protein kinase 5
PFIT_1241900	27,87980064	100,0377532	108,2336675	107,3233086	325,6073959	81,80499842	74,10308739	74,93334179	125,1478207	74,51821459	0,595441568	-0,747968155	0,6389222	1	replication factor C subunit 4, putative
PFIT_1143100	751,4873537	1505,677715	1576,701264	1247,084293	1379,239533	1179,355394	1767,695466	1475,209442	1273,257592	1621,452454	1,273467729	0,3487624	0,6390635	1	coproporphyrinogen III oxidase
PFIT_1454500	7,603581993	25,55709022	25,59579974	37,65730125	48,423664	13,6341664	16,84161077	20,85101685	26,41193393	18,84631381	0,713552966	-0,486907571	0,6390636	1	conserved protein, unknown function
PFIT_0917600	27,87980064	122,6740331	65,08646219	74,05935912	465,8690433	102,256248	56,58781219	54,73391922	142,9708244	55,6608657	0,389316253	-1,360985518	0,639116	1	conserved Plasmodium protein, unknown function
PFIT_0720400	1591,683164	1328,238489	541,8996459	1374,491496	2721,743873	1100,958937	1073,147438	927,2186554	1443,169267	1000,183047	0,693046249	-0,528976465	0,6391562	1	rhoptry-associated leucine zipper-like protein 1
PFIT_1425300	31397,72458	21508,84713	32462,05584	30818,73534	21765,60208	43567,97874	30572,2392	38727,50582	30253,49062	34649,87251	1,14531817	0,195748435	0,6392619	1	60S ribosomal protein L5, putative
PFIT_0308800	4219,988006	6200,880291	8769,852299	8685,028912	5298,216754	7846,462766	8569,01156	8944,434633	6836,738171	8756,723096	1,280833473	0,357082917	0,6393578	1	splicing factor 3B subunit 1, putative
PFIT_1404300	20,27621865	52,5745856	40,95327958	52,09260006	258,8161352	68,17083202	35,70421483	31,27652527	82,14727518	33,49037005	0,407686925	-1,294466405	0,6395148	1	conserved Plasmodium protein, unknown function
PFIT_1449600	255,9872604	335,8931858	259,6145402	246,0277015	712,9967078	610,1289466	214,8989534	278,230756	403,4413904	246,5648547	0,611154087	-0,710391929	0,6396233	1	ubiquitin-protein ligase, putative
PFIT_0406600	424,533328	718,5193366	467,3061724	938,2944228	505,9437997	265,8662449	450,0078398	546,687598	553,4105507	498,3477189	0,900502743	-0,151197424	0,6397435	1	conserved Plasmodium protein, unknown function
PFIT_1150900	29,14706431	58,41620623	85,56310198	69,66600731	81,81929434	78,39645682	86,90271157	80,146096	67,16802183	83,52440379	1,243514421	0,314423239	0,6397457	1	A_ITvarII - erythrocyte membrane protein 1, PfEMP1, putative
PFIT_1460400	633,6318328	489,2357271	514,841229	743,104078	255,4765721	289,7260361	472,9124304	381,834246	487,6692459	427,3733382	0,876359011	-0,190406086	0,6397546	1	conserved Plasmodium protein, unknown function
PFIT_0913600	125,4591029	172,3278084	211,348175	174,4788291	275,5139503	187,4697881	240,4982018	215,0261112	191,099609	227,7621565	1,191850458	0,253203232	0,6398824	1	protease, putative
PFIT_1430800	87,44119292	164,29558	202,5724722	184,5207761	267,1650428	245,4149953	187,2787118	254,7733621	191,9016765	221,0260369	1,151767097	0,203849014	0,6401525	1	conserved Plasmodium protein, unknown function
PFIT_1236200	2,534527331	10,22283609	6,581777075	8,159081937	11,68847062	0	7,410308739	14,33507408	6,531115509	10,87269141	1,664752583	0,735307778	0,6401552	1	conserved Plasmodium protein, unknown function
PFIT_1335300	13,93990032	29,93830569	38,75935389	70,92125069	121,8940508	44,31104081	57,26147662	68,41739903	53,29398369	62,83943782	1,179109413	0,237697596	0,6401756	1	conserved Plasmodium protein, unknown function
PFIT_0105500	196,4258682	101,4981583	69,47431357	82,84606275	642,8658841	64,76229042	74,10308739	65,15942765	192,9787629	69,63125752	0,360823422	-1,470635106	0,640709	1	centrin-1
PFIT_0205000.2	135,5972122	319,8287291	449,7547668	459,4190752	270,5046058	340,8541601	396,7883497	486,0893302	329,3264249	441,43884	1,340429454	0,422695293	0,6407476	1	conserved Plasmodium protein, unknown function
PFIT_1437800	605,7520321	1293,918968	269,1215515	701,0534249	1721,544744	320,4029105	649,4125113	502,3791872	818,6322719	575,8958492	0,703485398	-0,507407618	0,6407663	1	cGMP-dependent protein kinase
PFIT_0926400	324,4194984	242,4272558	211,348175	325,7356558	161,9688072	190,8783297	305,1699871	329,0551096	242,796287	317,1125484	1,306084835	0,385248608	0,6409354	1	protein kinase, putative
PFIT_1036700	309,2123344	880,6243089	193,7967694	418,6236656	1115,414053	259,0491617	402,1776652	332,313081	529,4533822	367,2453731	0,693631178	-0,527759348	0,6409936	1	dynamin-like protein
PFIT_0611500	168,5460675	44,54235725	42,41589671	31,3810437	60,11213462	112,4818728	47,83017459	35,83768521	76,57990222	41,8339299	0,546278184	-0,872292286	0,6410092	1	conserved Plasmodium protein, unknown function
PFIT_1108200	206,5639775	335,8931858	530,9300174	583,0605477	450,8410096	524,9154066	472,238766	622,272534	438,7006908	547,25565	1,24744652	0,318977966	0,6410729	1	Rad51 homolog
PFIT_0720300	411,8606913	581,2412519	628,1940564	460,0466969	497,5948921	736,2449858	692,5270348	606,6342714	552,5304291	649,5806531	1,17564684	0,233454745	0,6411677	1	PelOta protein homologue, putative
PFIT_1469700	263,5908424	267,2541435	302,0304369	252,3039184	230,4298494	163,6099968	297,7596784	346,6481551	246,5365312	322,2039167	1,306921595	0,386172594	0,6412623	1	aminophospholipid transporter, putative
PFIT_1113900	1720,944058	4929,597603	6380,66722	7641,294045	4531,787038	6779,589244	5979,445488	7674,477388	5330,646535	6826,961438	1,280700454	0,35693308	0,6417092	1	protein tyrosine phosphatase
PFIT_0504200	163,4770129	214,6795579	113,3528274	227,8266726	374,0310599	156,7929136	150,2271681	151,8214664	208,3600074	151,0243172	0,72482392	-0,464297529	0,6417455	1	ATP-dependent helicase, putative
PFIT_0919600	6,336318328	38,70073662	28,52103399	37,65730125	58,4423531	44,31104081	43,788188	42,35362797	35,66146402	43,07090799	1,207771727	0,272347806	0,6418036	1	sugar transporter, putative
PFIT_1132800	2763,902055	2111,015652	1892,626563	2289,563916	2245,856141	2512,09516	1717,844299	1684,371205	2302,509915	1701,107752	0,738805831	-0,436732841	0,6418161	1	ubiquitin-like protein, putative
PFIT_1211500	9351,138588	15673,79833	12887,11951	12386,114	14258,26438	18378,85631	16258,21737	15807,02555	13822,54852	16032,62146	1,159888962	0,213986701	0,6418664	1	non-SERCA-type Ca2 -transporting P-ATPase
PFIT_0809200	356,10109	403,8020255	109,6962846	192,6798581	950,1056833	109,0733312	185,9313829	192,8719058	353,5763788	189,4016444	0,535673919	-0,90057304	0,6418853	1	ferlin like protein, putative
PFIT_1203100	732,4783987	409,6436462	345,1776422	416,7408005	616,1493798	265,8662449	338,1795443	349,2545322	464,3426854	343,7170382	0,7402279	-0,43396854	0,6420205	1	transporter, putative
PFIT_1244600	18954,46265	4498,047879	4462,444857	4456,741603	2497,99315	1820,161215	3255,14653	3392,851398	6114,975225	3323,998964	0,54358339	-0,879426721	0,6420682	1	conserved Plasmodium protein, unknown function
PFIT_1231800	6,336318328	23,36648249	48,26636522	48,32686994	170,3177148	119,298956	28,96757052	31,27652527	69,31878446	30,1220479	0,434543798	-1,202426409	0,6421567	1	phenylalanyl-tRNA synthetase alpha chain, putative
PFIT_1309000	126,7263666	35,77992631	46,07343953	23,84962412	55,10279007	129,5245808	37,72520812	35,18609093	69,50928791	36,45564953	0,52447163	-0,931063359	0,6423905	1	conserved Plasmodium protein, unknown function
PFIT_1140600	193,8913408	694,4226515	694,0118272	546,0308681	679,6010775	1066,873521	676,3590885	777,3519718	645,8052144	726,8555302	1,125502727	0,170569553	0,6424209	1	conserved protein, unknown function
PFIT_0625600	1252,056502	2728,036831	2456,465466	1845,835383	1784,996442	1847,429548	2707,457347	2429,795057	1985,803362	2568,626202	1,293494739	0,371274187	0,6424557	1	conserved protein, unknown function
PFIT_1449300	152,0716399	147,5009207	123,5913473	129,9176893	138,5918659	102,256248	171,1107654	160,9437863	132,3215852	166,0272759	1,254725566	0,327371852	0,6427814	1	conserved Plasmodium protein, unknown function
PFIT_1446900	3107,330508	3363,313073	4482,921497	4596,073617	3044,011706	4332,256375	4376,797807	5148,246378	3820,984463	4762,522093	1,246412316	0,317781394	0,6428301	1	conserved Plasmodium protein, unknown function
PFIT_0314000.1	354,8338264	1209,215469	644,2828448	920,0933939	686,2802036	364,7139513	1065,063465	916,1415527	696,5699481	990,6025089	1,42211491	0,508038042	0,6429554	1	vesicle transport v-SNARE protein, putative
PFIT_1423000	25,34527331	7,302025778	10,96962846	8,159081937	30,05606731	10,2256248	8,083973169	7,167537041	15,34295027	7,625755105	0,497020128	-1,008623817	0,6430145	1	conserved Plasmodium protein, unknown function
PFIT_bin00600	0	1,460405156	5,850468512	6,276216875	3,339563034	10,2256248	1,347328862	1,303188553	4,52537973	1,325258707	0,292850277	-1,771764836	0,6430218	1	stevor
PFIT_1447300	769,229045	1110,638121	642,8202277	508,3735669	392,3986565	589,677697	851,5118405	865,3171991	668,856219	858,4145198	1,283406651	0,359978365	0,6430598	1	conserved Plasmodium protein, unknown function
PFIT_0910300	1918,63719	3236,257825	4305,213516	3720,541363	3853,855742	4182,280544	4204,339713	4359,817304	3536,13103	4282,078508	1,21095018	0,276139512	0,6433569	1	Ran-binding protein, putative
PFIT_1023000	1,267263666	5,111418045	4,387851384	8,159081937	13,35825214	0	5,389315446	11,72869698	5,380644528	8,559006211	1,59070278	0,669664296	0,643445	1	conserved Plasmodium protein, unknown function
PFIT_1314000	19,00895498	29,93830569	46,80374809	47,69924825	11,68847062	51,12812402	49,85116788	43,00522225	34,37780861	46,42819506	1,350528057	0,433523612	0,6436393	1	ubiquitin-like protein, putative
PFIT_0910000	420,731537	659,3729278	652,327239	512,7669187	676,2615145	593,0862386	790,2083773	632,6980424	585,7577293	711,4532099	1,214586124	0,280464792	0,6439022	1	small nuclear ribonucleoprotein Sm D3, putative
PFIT_1456600	362,4374084	1641,495395	1567,925561	1371,981009	1082,018423	484,0129073	1711,107654	1454,358425	1084,978451	1582,73304	1,458769102	0,544751547	0,6442004	1	hemolysin, putative
PFIT_1443900	2619,433997	318,3683239	259,6145402	316,9489522	627,8378505	245,4149953	226,3512487	298,4301786	731,2697765	262,3907137	0,358815204	-1,478687074	0,6444869	1	conserved Plasmodium protein, unknown function
PFIT_0621300	0	10,95303867	10,96962846	10,041947	33,39563034	6,817083202	9,431302031	3,257971382	12,02955461	6,344636707	0,527420749	-0,922973766	0,6446126	1	conserved Apicomplexan protein, unknown function
PFIT_1110100	43,08696463	64,25782685	40,22197102	237,8686196	530,9905225	201,1039545	62,65079206	339,480618	186,2549765	201,065705	1,079518566	0,110388055	0,6446475	1	small nucleolar RNA snR61/Z1/Z11
PFIT_0204200	5,069054662	48,19337014	45,34113096	32,00870606	133,5825214	95,43916483	30,98856382	31,27652527	59,93899134	31,13254454	0,519403878	-0,945071309	0,644924	1	ubiE/COQ5 methyltransferase, putative
PFIT_0218800.2	31,68159164	136,5478821	54,8481423	55,2307085	80,14951283	88,62208163	121,2595975	64,50783337	74,51331982	92,88371546	1,246538413	0,317927341	0,645184	1	ribosome associated membrane protein RAMP4, putative
PFIT_1337700	16,47442765	37,97053405	40,22197102	32,63632775	53,43300855	44,31104081	54,56681889	36,48927948	37,50788497	45,52804919	1,213826085	0,27956173	0,6453435	1	conserved Plasmodium protein, unknown function
PFIT_0530700	4840,947203	1605,715469	1910,909278	1936,840528	1389,258222	1987,179753	1810,80999	1533,201333	2278,475075	1672,005661	0,733826619	-0,446488857	0,6453437	1	conserved Plasmodium protein, unknown function
PFIT_1140400	29,14706431	318,3683239	296,911277	795,8242997	353,9936816	105,6647896	289,0020408	265,1988705	316,6515727	277,1004557	0,875095782	-0,192487162	0,6454198	1	adrenodoxin reductase, putative
PFIT_0821600	10655,1529	6725,895944	8959,261217	8073,097766	5358,328889	8517,945461	9731,756367	9718,528633	8048,280363	9725,1425	1,208350363	0,273038827	0,645517	1	DNA helicase, putative
PFIT_0616100	5138,754164	5617,448431	7784,048355	8645,488745	4927,525257	5763,843847	7737,035988	8822,586503	6312,851467	8279,811245	1,311580241	0,391306072	0,6455518	1	alpha adaptin-like protein, putative
PFIT_0713600	7,603581993	1,460405156	2,193925692	2,51048675	31,72584883	3,408541601	3,368322154	0	8,150465003	1,684161077	0,206633741	-2,274852243	0,6457179	1	mitochondrial ATP synthase F1, epsilon subunit, putative
PFIT_1451000	1049,294315	490,6961323	412,4580301	441,845668	505,9437997	640,805821	394,093692	424,187874	590,173961	409,140783	0,693254549	-0,528542918	0,6458702	1	conserved Plasmodium protein, unknown function
PFIT_0920800	676,7187974	1219,438305	334,9393223	834,1092227	1168,847062	381,7566593	629,2025784	605,9826771	769,3015615	617,5926277	0,802796535	-0,316893705	0,6461471	1	conserved Plasmodium protein, unknown function
PFIT_1114900.1	182,4859678	502,3793735	372,236059	402,9331234	465,8690433	456,7445745	505,2483231	461,980342	397,1080236	483,6143325	1,217840748	0,28432549	0,6462607	1	rhomboid protease ROM1
PFIT_1303800	344,695717	119,0230202	131,6355415	151,8844484	170,3177148	204,5124961	104,4179868	146,6087122	187,0114897	125,5133495	0,671153145	-0,575286093	0,6465609	1	conserved Plasmodium protein, unknown function
PFIT_1369000	0	0	1,462617128	0,627621687	1,669781517	0	1,347328862	1,303188553	0,626670055	1,325258707	2,114763097	1,080496057	0,6465911	1	tRNA Arginine
PFIT_1360700	105,1828842	85,43370161	33,64019394	46,44400487	305,5700176	64,76229042	49,85116788	48,86957073	106,8388488	49,36036931	0,462007686	-1,114011242	0,6466743	1	cytochrome c oxidase subunit 2, putative
PFIT_1207400	110,2519389	103,6887661	115,5467531	123,6414724	28,38628579	61,35374882	134,7328862	123,8029125	90,47816085	129,2678993	1,428719352	0,514722551	0,6469736	1	conserved Plasmodium protein, unknown function
PFIT_1428900	1,267263666	8,762430934	7,313085639	6,276216875	21,71650428	0	3,368322154	4,561159935	8,389250232	3,964741045	0,472597781	-1,081315239	0,647012	1	conserved Plasmodium protein, unknown function
PFIT_1433200	225,5729325	180,3600367	207,6916322	177,6169376	293,881547	255,6406201	157,6374768	151,8214664	223,4606177	154,7294716	0,692423897	-0,53027258	0,6471553	1	syntaxin, Qa-SNARE family
PFIT_1360500.1	283,8670611	890,1169424	212,8107921	415,4855571	734,7038676	149,9758304	363,7787926	351,8609093	447,8266751	357,819851	0,799014152	-0,323707039	0,647237	1	conserved Plasmodium protein, unknown function
PFIT_1476000	36,7506463	69,36924489	62,16122794	63,38979044	297,2211101	129,5245808	48,50383902	55,3855135	109,7361001	51,94467626	0,473359963	-1,078990408	0,6475604	1	trailer hitch homolog, putative
PFIT_1424800	4084,390794	2616,315836	4549,470576	4316,781967	2900,410495	3913,005758	4253,517216	4898,68577	3730,062571	4576,101493	1,226816281	0,294919218	0,6476748	1	conserved Plasmodium protein, unknown function
PFIT_0222000	2,534527331	32,859116	87,75702767	72,17649406	116,8847062	637,3972794	26,2729128	22,80579968	158,2681918	24,53935624	0,155049198	-2,689202029	0,6477687	1	Pfmc-2TM Maurer's cleft two transmembrane protein
PFIT_1368600	933,9733215	321,2891342	223,049112	211,5085087	469,2086063	599,9033218	298,4333428	254,1217678	459,8220008	276,2775553	0,600835878	-0,734957132	0,6480253	1	conserved Plasmodium protein, unknown function
PFIT_0318000	11,40537299	15,33425413	16,08878841	8,159081937	6,679126069	6,817083202	12,12595975	20,19942257	10,74728446	16,16269116	1,503886049	0,588695257	0,648333	1	conserved Plasmodium protein, unknown function
PFIT_1016500	173,6151222	360,7200734	247,1822946	350,2129016	632,847195	286,3174945	267,444779	235,8771281	341,8158469	251,6609536	0,736247181	-0,441737891	0,6483718	1	DNA polymerase delta catalytic subunit
PFIT_1469400	108,9846752	95,65653769	92,14487906	77,82508925	98,51710952	119,298956	103,0706579	123,8029125	98,73787447	113,4367852	1,148868009	0,200213059	0,6484211	1	leucine-rich repeat protein
PFIT_0204600	304,1432797	383,3563534	209,8855579	284,3126244	105,1962356	47,71958241	356,3684839	301,0365557	222,4356056	328,7025198	1,477742374	0,563394775	0,6484273	1	conserved Plasmodium protein, unknown function
PFIT_1246500	6779,860611	3274,958562	2584,444465	2181,612986	2047,15214	1561,112053	2427,212944	2236,923151	3071,523469	2332,068048	0,759252451	-0,397344521	0,6484555	1	conserved Plasmodium protein, unknown function
PFIT_0700200	25,34527331	5,841620623	5,119159948	1,882865062	0	13,6341664	3,368322154	3,257971382	8,637180891	3,313146768	0,383591221	-1,38235839	0,6486035	1	rifin
PFIT_1408000	86,17392926	112,451197	119,9346045	139,3320146	80,14951283	61,35374882	92,96569145	77,5397189	99,89916783	85,25270517	0,853387541	-0,228727047	0,6486158	1	LCCL domain-containing protein
PFIT_0109100	21875,5054	7275,008283	8497,805513	9868,095792	4947,562635	7004,55299	8122,372042	6923,189187	9911,421768	7522,780615	0,75900116	-0,397826005	0,6486753	1	phosphatidylinositol-4-phosphate 5-kinase
PFIT_1332100	1367,377495	2683,494473	2675,858035	2696,890391	2259,214393	3838,017843	3018,01665	3046,203242	2586,808772	3032,109946	1,17213059	0,22914866	0,6488765	1	translation initiation factor 6, putative
PFIT_1472200	8807,482476	18336,11693	21163,33853	26151,74047	18985,41585	26388,92908	22934,90555	25900,87249	19972,17056	24417,88902	1,222595659	0,289947349	0,648918	1	exported protein 2
PFIT_1471600	96,31203858	72,2900552	51,19159948	55,2307085	165,3083702	54,53666562	107,1126445	74,28174752	82,4782396	90,69719601	1,099649998	0,137044409	0,6489684	1	phosphodiesterase delta, putative
PF_apicoplast_ge[illegible]	446,0768103	525,0156535	334,2080137	434,9418294	783,1275316	269,2747865	361,0841349	344,041778	465,4407708	352,5629564	0,75748189	-0,400716691	0,6490275	1	molecular chaperone
PFIT_0816600	1870,48117	614,1003679	455,6052353	510,8840536	494,2553291	968,0258147	514,6796251	510,1983185	818,8919952	512,4389718	0,625771133	-0,676292986	0,6490897	1	ABC transporter, putative
PFIT_1121800	280,0652701	87,62430934	85,56310198	129,2900676	282,1930764	85,21354003	94,98668474	115,9837812	158,3248942	105,485233	0,666258035	-0,585847608	0,6491925	1	zinc finger, DHHC-type, putative
PFIT_0312000	58,29412862	51,11418045	54,11683373	55,2307085	175,3270593	27,26832281	47,83017459	41,05043942	70,22520723	44,440307	0,632825573	-0,660120194	0,6491942	1	RING zinc finger protein, putative
PFIT_1310900	933,9733215	660,1031304	520,6916975	470,7162656	420,7849423	541,9581146	671,6434375	736,3015324	591,3712453	703,9724849	1,190407025	0,251454946	0,649343	1	cytochrome c2 precursor, putative
PFIT_1343400	8693,428746	7802,944747	9711,777729	8339,209362	8591,025906	15031,66846	9925,098059	11267,36823	9695,009158	10596,23314	1,092957518	0,128237326	0,6493609	1	heat shock protein 110, putative (HSP110)
PFIT_0530000	6328,714746	9995,743088	4475,608411	15202,25251	6537,19464	2873,40057	6521,07169	7721,392176	7568,818995	7121,231933	0,940864346	-0,087941365	0,6494745	1	membrane skeletal protein IMC1-related
PFIT_0806100	164,7442765	259,2219151	234,750049	132,4281761	312,2491437	129,5245808	334,1375577	170,7177004	205,4863569	252,4276291	1,228439848	0,296827216	0,6494992	1	mitogen-activated protein kinase organizer 1, putative
PFIT_1243800	10243,29221	12890,26611	10361,91104	16294,31425	9389,181471	6513,723	13772,39562	16103,50095	10948,78135	14937,94829	1,364348032	0,448211709	0,6495562	1	translation initiation factor SUI1, putative
PFIT_0932600	1565,070627	1784,6151	884,8833624	1371,981009	1776,647534	507,8726986	983,5500689	1158,534624	1315,178389	1071,042346	0,814370397	-0,296242976	0,6500152	1	Protein MAM3, putative
PFIT_0731500	1,267263666	1,460405156	8,044392203	5,0209735	0	10,2256248	1,347328862	1,303188553	4,336443555	1,325258707	0,305609583	-1,710238311	0,6500504	1	rifin
PFIT_0306500	65,89771061	13,1436464	16,08878841	16,31816387	153,6198996	23,85979121	15,49428191	17,59304546	48,15466688	16,54366369	0,343552657	-1,541396854	0,6500738	1	conserved Plasmodium protein, unknown function
PFIT_1354400	373,8427813	1191,690607	1480,168533	1378,884847	943,4265572	695,3424866	1458,483493	1374,863923	1010,559302	1416,673708	1,401870929	0,487353526	0,650128	1	histone-lysine N-methyltransferase, putative
PFIT_0804800	5142,555955	2713,432779	2993,245952	2846,891974	5186,341392	17335,84258	2467,63281	2039,490085	6036,385106	2253,561448	0,373329635	-1,421478061	0,6501916	1	glutamate dehydrogenase, putative (GDH3)
PFIT_0916600	64,63044694	61,33701654	58,50468512	71,54887237	148,610555	109,0733312	55,24048332	52,77913639	85,61748454	54,00980986	0,630826871	-0,66468398	0,6503775	1	apicoplast Ufd1 precursor
PFIT_1359200	108,9846752	451,2651931	425,6215842	459,4190752	392,3986565	412,4335337	479,6490747	482,8313589	375,020453	481,2402168	1,283227255	0,359787932	0,6504067	1	RNA binding protein, putative
PFIT_0615700	163,4770129	78,13167583	65,81777075	56,48595187	85,15885738	167,0185385	62,65079206	61,24986199	102,6816345	61,95032703	0,603324317	-0,728994363	0,6506538	1	ras GTPase, putative
PFIT_1205600	93,77751125	270,9051564	326,8949281	332,6394944	514,2927073	456,7445745	372,5364302	369,4539448	332,5423953	370,9951925	1,115632767	0,157862213	0,6507877	1	shewanella-like protein phosphatase 2, putative (SHLP2)
PFIT_1338200	10,13810932	7,302025778	7,313085639	7,53146025	3,339563034	3,408541601	8,7576376	11,72869698	6,505464271	10,24316729	1,574548235	0,654937954	0,6508215	1	probable protein, unknown function
PFIT_0109800	188,8222862	106,6095764	100,9205818	129,2900676	632,847195	160,2014552	111,8282955	92,52638726	219,7818604	102,1773414	0,464903433	-1,104997016	0,6509942	1	replication factor c protein, putative
PFIT_0503600	172,3478585	190,5828728	105,3084332	110,4614617	198,7040005	286,3174945	127,9962418	98,39073575	177,2870128	113,1934888	0,638475921	-0,647295882	0,6511202	1	protein kinase, putative
PFIT_0816100	263,5908424	257,76151	343,7150251	424,8998824	627,8378505	402,2079089	368,4944436	506,9403471	386,6688365	437,7173954	1,132021394	0,178901224	0,6511906	1	CS domain protein, putative
PFIT_0303900	55,75960129	78,13167583	27,78972543	41,42303137	40,07475641	6,817083202	38,39887255	29,32174244	41,66597892	33,8603075	0,812660794	-0,299274801	0,6511995	1	inner membrane complex protein 1a, putative
PFIT_1314800	167,2788039	441,042357	468,0374809	463,1848054	719,6758339	623,763113	465,5021217	617,7113741	480,4970657	541,6067479	1,127180136	0,172718093	0,6512334	1	phosphatidylinositol synthase
PFIT_0703800	216,7020868	110,9907918	194,528078	184,5207761	385,7195305	422,6591585	143,4905238	159,6405977	252,5200703	151,5655607	0,600211938	-0,736456081	0,6512931	1	peptide chain release factor, putative
PFIT_1341100	1843,868633	2476,847144	2999,096421	2480,988531	2554,765721	5095,769694	2955,365858	3317,266461	2908,556024	3136,3116	1,078306945	0,108767906	0,6516542	1	vacuolar ATP synthase subunit d, putative
Pf_M7661102100	0	1,460405156	0	0	0	13,35825214	6,817083202	0,673664431	0	3,605956749	0,336832215	0,093409943	-3,42028006	0,6517243	1 unspecified product
PFIT_0313400	2909,637376	648,4198891	680,1169645	501,4697183	589,4328756	933,9403487	542,2998668	588,3896316	1043,836205	565,3447492	0,541602932	-0,884692546	0,6517698	1	GTP-binding protein EngA, putative
PFIT_1112700	0	17,52486187	11,70093702	13,18005544	26,71650428	13,6341664	10,77863089	5,212754212	13,79275417	7,995692552	0,57970239	-0,786615662	0,65195	1	conserved Plasmodium protein, unknown function
PFIT_1109500	3778,980251	1539,997237	1362,427855	1085,785519	1319,164777	2198,509333	1692,24505	878,3490847	1884,144162	1285,297067	0,682164928	-0,55180751	0,6519634	1	heat shock protein DnaJ homologue Pfj2
PFIT_0102100	2407,800965	520,634438	664,7594846	641,4293646	519,3020519	1015,745397	522,0899339	630,7432596	961,6119501	576,4165967	0,599427447	-0,738342949	0,6520509	1	ATP-dependent RNA helicase, putative
PFIT_1472100	9466,459582	958,0257821	1124,752571	1135,367633	794,8160022	1363,41664	718,7999476	891,3809702	2473,806368	805,0904589	0,325446029	-1,619509784	0,6525041	1	RNA 3'-terminal phosphate cyclase-like protein, putative
PFIT_1364800	371,308254	970,4392259	1228,598387	1232,648994	928,3985236	927,1233155	1136,471895	1328,60073	943,0861168	1232,536312	1,306918096	0,386168731	0,6525353	1	conserved Plasmodium protein, unknown function
PFIT_1412800	3017,354788	758,6804784	623,0748965	794,5690648	374,0310599	374,9395761	559,815142	557,7647007	990,4416425	558,7899213	0,56418258	-0,825765973	0,6526637	1	conserved Plasmodium protein, unknown function
PFIT_0721500	3645,917566	2356,363719	1230,792313	2120,733682	1414,304945	453,3360329	1331,160915	1830,328323	1870,241376	1580,744619	0,84520888	-0,24262017	0,6527707	1	conserved Plasmodium protein, unknown function
PFIT_0323800	2298,816289	2007,326886	1765,378873	1869,685007	1365,881281	2512,09516	1447,704862	1499,970024	1969,863916	1473,837443	0,748192518	-0,418518557	0,6527811	1	U4/U6.U5 tri-snRNP-associated protein 1, putative
PFIT_0935800	21227,93366	3120,155615	3160,715613	3386,010004	2334,354561	4717,421576	2590,239736	2799,249012	6324,433339	2694,744374	0,426084715	-1,230787798	0,6529283	1	sec-1 family protein
PFIT_1476200	2583,950614	2921,540514	2206,357937	1502,52632	2464,597519	4867,397406	2716,214985	2892,426993	2757,728385	2804,320989	1,016895284	0,024171123	0,6529325	1	conserved Plasmodium protein, unknown function
PFIT_1019700	206,5639775	90,54511965	22,67056548	50,209735	156,9594626	54,53666562	66,69277865	48,86957073	96,91425431	57,78117469	0,596209248	-0,746109341	0,6529753	1	conserved Plasmodium protein, unknown function
PFIT_1312600	181,2187042	132,1666666	125,785073	123,6414724	369,0217153	463,5616577	112,5019599	132,9252324	232,5658815	122,7135962	0,527650898	-0,92234436	0,6530631	1	DEAD box helicase, putative
PFIT_0923400	1459,887743	665,944751	712,2945413	718,6268322	355,6634632	1083,916229	913,4889681	955,2372093	832,7222599	934,3630887	1,122058499	0,166147893	0,6531153	1	perforin like protein 3
PFIT_1332400	153,3389035	119,0230202	76,78739921	75,94222419	105,1962356	47,71958241	69,38743637	76,23653035	96,33456085	72,81198336	0,755824106	-0,403877562	0,6532289	1	conserved Plasmodium protein, unknown function
PFIT_0617800.1	89,97572026	82,51289129	35,83411963	52,09260006	208,7226897	54,53666562	50,52483231	54,08232495	87,27911442	52,30357863	0,59926798	-0,738726804	0,6534379	1	N-acetylglucosamine transferase
PFIT_0312300	176,1496495	281,1335173	250,8388374	303,7688967	1143,800339	528,3239482	251,2768327	258,0313335	462,6691981	254,6540831	0,550402067	-0,861442206	0,6537019	1	glycogen synthase kinase 3
PFIT_0414700	479,0256656	617,0211783	423,4276585	409,8369619	1087,027768	1857,655173	425,7559309	391,6081602	812,3324008	408,6820402	0,503097057	-0,991091344	0,6537711	1	glutamyl-tRNA(Gln) amidotransferase subunit A, putative
PFIT_0823700	714,7367074	593,6546958	495,8272064	429,9208559	490,9157661	334,0370769	691,179706	569,4933976	509,8487181	630,3365518	1,236320755	0,306053089	0,6543895	1	conserved Plasmodium protein, unknown function
PFIT_1204400	3,801790997	33,58931858	36,5654282	32,63632775	128,5731768	57,94520722	28,29390669	21,50261112	48,85187493	24,89825861	0,509668434	-0,97236909	0,6544324	1	conserved Plasmodium protein, unknown function
PFIT_0937200	1249,521974	4014,653773	2711,692155	1813,199055	2823,600546	3691,450554	2890,694072	3401,973717	2717,353009	3146,333895	1,157867191	0,211469783	0,6544979	1	Plasmodium exported protein (PHISTb), unknown function
PFIT_0615900	3337,972495	1614,4779	1624,967629	1513,82351	1197,233348	1363,41664	1430,189587	1299,278987	1775,315254	1364,734287	0,768727855	-0,379455149	0,6548509	1	conserved Plasmodium protein, unknown function
PFIT_0902600	59,56139228	173,7882135	266,9276258	313,1832221	113,5451432	262,4577033	218,2672756	308,2040928	198,2438834	263,2356842	1,327837609	0,40907872	0,6548789	1	serine/threonine protein kinase, FIKK family
PFIT_1246700	345,9629807	231,4742172	214,2734092	210,880887	150,2803365	201,1039545	152,9218258	194,1750944	225,6626309	173,5484601	0,769061583	-0,378828968	0,6551194	1	conserved Plasmodium protein, unknown function
PFIT_1024400	1733,616695	281,858195	312,2687568	326,9908992	307,2397992	149,9758304	235,1088863	251,5153907	518,6583625	243,3121385	0,469118318	-1,09197626	0,6552159	1	conserved Plasmodium protein, unknown function
PFIT_0713700	34,21611897	81,05248614	67,28038788	80,96319769	402,4173456	81,80499842	53,89315446	50,82435356	124,6224225	52,35875401	0,420139113	-1,251069995	0,6553949	1	GTP-binding translation elongation factor tu family protein, putative
PFIT_bin07800	0	0	0,731308564	0,627621687	0	0	0,673664431	0,651594276	0,226488375	0,662629354	2,925666065	1,54876511	0,655508	1	rifin, pseudogene
PFIT_1239800	1239,383865	1862,746776	2301,428051	3318,235862	2022,105417	1216,849352	2709,478341	2796,642635	1993,45822	2753,060488	1,381047498	0,465762939	0,6555669	1	early transcribed membrane protein 12
PFIT_1414300	0	0,730202578	0	0,627621687	0	0	1,347328862	0	0,226304044	0,673664431	2,976811277	1,573767761	0,6557281	1	conserved Plasmodium protein, unknown function
PFIT_0927000	240,7800965	310,3360956	275,7033286	254,8144051	322,2678328	211,3295793	190,6470339	231,3159681	269,205223	210,981501	0,783719939	-0,351589892	0,6559248	1	replication termination factor, putative
PFIT_1236700	414,3952186	368,7523018	403,6823273	297,4926799	422,4547239	821,4585259	331,4428999	257,3797392	454,7059629	294,4113196	0,647476267	-0,627100782	0,6559718	1	ubiquitin-activating enzyme, putative
PFIT_1419400	0	0	0,731308564	0	0	0	0,673664431	0	0,121884761	0,336832215	2,763530187	1,466512372	0,6559772	1	tRNA Proline
PFIT_1454200	1080,975907	786,4281763	963,8646873	947,0811264	844,9094477	1073,690604	745,0728604	686,1287731	949,4916581	715,6008168	0,753667303	-0,408000291	0,6560972	1	conserved Plasmodium protein, unknown function
PFIT_0513400	1390,188241	4990,934619	2522,283237	2131,403251	3718,603439	2512,09516	3707,175363	3401,322123	2877,584658	3554,248743	1,235150018	0,304686279	0,6562373	1	purine nucleoside phosphorylase (PNP)
PFIT_1337800	1624,632019	725,0911598	584,3155426	1457,96518	1392,597785	623,763113	741,0308739	908,3224214	1068,0608	824,6766476	0,77212519	-0,373093315	0,6563895	1	conserved Plasmodium protein, unknown function
PFIT_0218800.1	101,3810932	311,0662982	199,647238	193,3074797	260,4859167	354,4883265	257,3398126	278,8823503	236,729392	268,1110476	1,132563553	0,179520209	0,6565072	1	ribosome associated membrane protein RAMP4, putative
PFIT_1130900	7604,849257	4916,453956	7457,153427	6792,749524	5147,936418	7420,395065	6669,277865	8821,934909	6556,589608	7745,606387	1,181346636	0,240432593	0,656508	1	60S ribosomal protein L38e, putative
PFIT_1455000	1854,006743	3301,976057	3754,538167	3849,203809	2933,806126	2133,747042	4110,700357	3843,103043	2971,212991	3976,9017	1,338477488	0,42059287	0,6565509	1	glucose-6-phosphate dehydrogenase-6-phosphogluconolactonase
PFIT_0722300	1390,188241	426,4383054	796,3950261	942,6877746	337,2958665	766,9218602	666,254122	546,0360037	776,6545123	606,1450629	0,780456501	-0,35760987	0,6566228	1	conserved Plasmodium protein, unknown function
PFIT_1139800	15337,69214	6057,760586	10317,30122	8698,836589	6161,493798	14067,05119	11288,59487	10832,75485	10106,68925	11060,67486	1,094391504	0,130128936	0,6567771	1	RNA binding protein, putative
PFIT_1418000	63,36318328	29,20810311	35,10281107	37,02967956	20,03737821	10,2256248	20,88359735	28,67014816	32,49446334	24,77687276	0,762495213	-0,391199814	0,6568424	1	conserved Plasmodium protein, unknown function
PFIT_0519900	13362,02809	8348,406072	12400,06801	13911,86232	9379,162782	18504,97253	11825,50542	16605,22854	12651,08327	14215,36698	1,123648203	0,16819042	0,6568966	1	conserved Plasmodium protein, unknown function
PFIT_1475400	419,4642733	333,7025781	419,0398071	354,6062534	238,778757	262,4577033	394,7673564	471,1026619	338,0082287	432,9350092	1,280841626	0,3570921	0,657094	1	DNA repair metallo-beta-lactamase protein, putative
PFIT_0715800	3564,812691	3250,131674	4162,608346	3441,877334	3099,114496	6343,29592	4163,919847	4554,643997	3976,97341	4359,28192	1,096130517	0,132419591	0,6571374	1	serine--tRNA ligase, putative
PFIT_0316500	58,29412862	116,1022099	62,16122794	84,10130612	434,1431945	167,0185385	78,14507397	63,20464482	153,6367676	70,67485939	0,460012668	-1,120254503	0,6572049	1	conserved Plasmodium protein, unknown function
PFIT_1354300	210,3657685	295,0018414	356,8785792	367,7863089	187,0155299	204,5124961	391,3990343	350,5577207	270,2600873	370,9783775	1,372671715	0,456986635	0,6572108	1	RAP protein, putative
PFIT_0708800	1084,777698	315,4475136	254,4953803	325,7356558	384,049749	149,9758304	265,4237857	278,8823503	419,0803045	272,153068	0,649405532	-0,622808421	0,6572336	1	conserved Plasmodium membrane protein, unknown function
PFIT_0804600	44083,03387	11236,35727	13263,01212	14182,99489	8749,65515	10709,63771	11066,2856	11453,0726	17037,4485	11259,6791	0,660878247	-0,597543585	0,657241	1	mannose-6-phosphate isomerase, putative
PFIT_0410400	136,8644759	181,0902393	103,8458161	116,7376339	405,7569087	122,7074976	127,9962418	106,2098671	177,8337619	117,1030545	0,658497313	-0,60275054	0,657305	1	conserved Plasmodium protein, unknown function
PFIT_1441300	257,2545241	566,6372004	171,8575125	262,3458654	1531,189651	245,4149953	262,0554636	215,6777055	505,7832915	238,8665845	0,472270612	-1,082314332	0,6574225	1	conserved Plasmodium protein, unknown function
PFIT_1466100	5,069054662	42,35174951	27,05841687	30,75346269	85,15885738	6,817083202	46,48284572	38,44406231	32,86810405	42,46345402	1,29193	0,369533487	0,6575517	1	conserved Plasmodium protein, unknown function
PFIT_0700700	8,870845659	6,5718232	8,044394203	8,159081937	3,339563034	3,408541601	0,673664431	5,864348488	6,399041606	3,269006459	0,51085876	-0,969003619	0,657552	1	rifin
PFIT_1447100	3746,031395	9635,023014	13391,72242	13926,92525	9335,748463	18818,55818	12502,53817	14378,0793	11475,66812	13440,30874	1,171200543	0,227988128	0,6576225	1	DEAD/DEAH box ATP-dependent RNA helicase, putative
PFIT_1230200	243,3146238	591,464088	679,3856559	646,4503381	410,7662532	405,6164505	736,9888873	617,7113744	496,1662349	677,3501307	1,365167726	0,449078213	0,6576314	1	WD-repeat protein, putative
PFIT_1240400	267,3926334	124,1344382	99,4579647	84,10130612	230,4298494	293,1345777	181,2157319	159,6405977	183,1084616	170,4281648	0,930749804	-0,103534687	0,657636	1	C_ITvarS - erythrocyte membrane protein 1, PfEMP1, putative
PFIT_0537400	15,20716399	17,52486187	37,29673676	23,84962412	25,04672276	61,35374882	18,18260406	16,28985691	30,04647639	17,57623049	0,584968109	-0,773570119	0,657705	1	rifin
PFIT_1013400	10714,71429	2975,575505	2674,395418	2480,360909	2060,510392	2941,571402	2436,644246	2388,744618	3974,52132	2412,694432	0,607040254	-0,720135907	0,657773	1	initiation factor 2 subunit family, putative
PFIT_0514600	2,534527331	13,1436464	29,25234256	24,47724581	18,36759669	74,98791522	6,062979877	18,89623402	27,12721234	12,47960695	0,460040154	-1,120168304	0,6578593	1	conserved Plasmodium membrane protein, unknown function
PFIT_1116800	1091,114016	1737,151933	632,5819078	1437,881286	7420,509062	2866,583486	1128,387922	1000,848809	2530,972082	1064,618365	0,420636454	-1,249354209	0,6579153	1	rhoptry neck protein 4
PFIT_0217300	67,16497428	261,4125229	237,6752833	232,2200244	519,3020519	170,4270801	320,6642691	267,1536533	248,0336561	293,9086352	1,184959568	0,244833451	0,6580125	1	conserved Plasmodium protein, unknown function
PFIT_1130800	2400,197383	633,085635	572,6146056	618,2073622	504,2740182	712,3851946	1036,095895	1024,957797	906,794033	1030,526846	1,136450846	0,184535285	0,6581409	1	N-acetyl glucosamine phosphate mutase, putative
PFIT_1351200	3231,522347	709,0267031	665,4907932	684,7352611	609,4702538	998,7026891	635,9392227	634,001231	1149,824675	634,9702268	0,55223215	-0,856653044	0,6582782	1	conserved Plasmodium protein, unknown function
PFIT_1114600	111,5192026	181,0902393	159,4252669	124,8967158	422,4547239	224,9637457	251,2768327	172,020889	204,0583157	211,6488608	1,037197921	0,05269122	0,6583669	1	conserved Plasmodium membrane protein, unknown function
PFIT_1120800	4666,064817	2283,343461	2423,556581	2993,127828	1335,825214	4209,548877	2382,077427	1958,692395	2985,244463	2170,384911	0,72703758	-0,459898156	0,6584833	1	conserved Plasmodium protein, unknown function
PFIT_1150100	5,069054662	3,651012889	9,507011331	11,29719037	15,02803365	23,85979121	9,431302031	14,98666836	11,40201569	12,20898519	1,070774285	0,098654398	0,6585045	1	Plasmodium exported protein (PHISTa), unknown, putative
PFIT_1400200	111,5192026	45,27255982	27,05841687	17,57340725	43,41431945	61,35374882	33,00955711	27,36695961	51,0319246	30,18825836	0,591556129	-0,757413031	0,6585973	1	erythrocyte membrane protein 1, PfEMP1
PFIT_1367400	363,704672	571,0184159	697,66837	667,7894755	719,6758339	807,8243594	706,6739879	785,8226974	637,9468545	746,2483427	1,16976569	0,22621958	0,6586087	1	conserved Plasmodium protein, unknown function
PFIT_1205500	4402,473974	2682,764271	2218,790183	1830,144841	2115,613182	3913,005758	2321,447628	1632,895257	2860,465368	1977,171443	0,691206216	-0,532811904	0,6587491	1	conserved protein, unknown function
PFIT_1014300	193,8913408	48,92357271	43,14720527	35,1468145	80,14951283	34,08541601	40,41986585	43,00522225	72,55731036	41,71254405	0,574890991	-0,798639672	0,6589981	1	conserved Plasmodium protein, unknown function
PFIT_0519100	0	16,06445671	19,74533123	29,49821931	10,0186891	10,2256248	20,20993292	26,71536533	14,25872019	23,46264913	1,645494744	0,718521418	0,6591511	1	conserved Plasmodium protein, unknown function
PFIT_1407100	4848,550784	1340,651933	1356,577386	1513,195889	1158,828373	1342,965391	1242,23721	1314,265656	1926,794959	1278,251433	0,663408126	-0,592031411	0,6591991	1	conserved Plasmodium protein, unknown function
PFIT_0727100	17,74169132	37,24033147	43,87851384	37,65730125	83,48907586	40,90249921	45,80918129	55,3855135	43,48490216	50,59734774	1,163561257	0,218547165	0,6593579	1	actin-like protein, putative (ALP3)
PFIT_0505700	3158,021055	1279,314916	2005,248082	1738,512074	1374,230189	2232,594749	1531,293251	1352,058124	1964,653511	1441,648687	0,733792844	-0,446555259	0,6594963	1	conserved Plasmodium protein, unknown function
PFIT_0714700	30,41432797	31,39871085	46,80374809	43,30589644	140,2614474	160,2014552	35,70421483	34,53449665	75,39763101	35,11935574	0,465788583	-1,102252816	0,659608	1	cysteine desulfurase, putative
PFIT_0402700	32,94885531	131,436464	29,25234256	82,21844106	70,13082372	23,85979121	69,38743637	37,79246803	61,64111964	53,5899522	0,869386418	-0,201930538	0,6598668	1	conserved Plasmodium protein, unknown function
PFIT_1469300	1494,103862	1178,546961	1300,266627	803,9833817	1150,479465	2662,07099	1255,036835	1583,374092	1431,575214	1419,205463	0,991359342	-0,012520003	0,6599445	1	conserved protein, unknown function
PFIT_0214700	1451,016897	2955,860035	2729,243561	3916,986952	1738,242559	1114,593104	3469,371819	3299,021822	2317,657185	3384,19682	1,460180066	0,54614629	0,6600605	1	acyl-CoA synthetase
PFIT_1431700	190,0895498	32,859116	20,47663979	31,3810437	43,41431945	10,2256248	21,55726179	24,76058251	54,74105571	23,15892215	0,423063126	-1,241055149	0,6602156	1	conserved Plasmodium protein, unknown function
PFIT_1001500	6133,556141	6917,939222	8281,338178	7019,320953	4990,976955	3285,834103	8372,301546	8124,077439	6104,827592	8248,189492	1,351092945	0,434126925	0,6602673	1	Plasmodium exported protein (hyp16), unknown function

PFIT_0416800	2421,740865	4304,544196	4242,320979	4779,33915	3259,413522	3418,767226	4585,63378	4948,206935	3737,687656	4766,920358	1,275366161	0,350911508	0,6906972	1	golgi organization and biogenesis factor, putative
PFIT_1344700	2,534527331	37,24033147	26,3271083	18,82865062	123,5638323	98,84770643	25,59924837	17,59304546	51,22369274	21,59614692	0,421604647	-1,246037325	0,6907445	1	ubiquitin conjugating enzyme
PFIT_1138100	68,43223794	67,17863716	92,14487906	79,70795431	158,6292441	344,2627017	61,97712763	67,11421048	135,0592757	64,54566905	0,477906228	-1,065200525	0,6909594	1	UVB-resistance protein UVR8 homologue
PFIT_0318400	27,87980064	59,1464088	46,07243953	41,42303137	20,03737821	17,04270801	56,58781219	46,91478791	35,26696109	51,75130005	1,467415917	0,553277839	0,6909732	1	conserved Plasmodium protein, unknown function
PFIT_0922800	2696,73708	1293,918968	1423,126465	1476,793831	1322,466962	2437,107245	1166,786794	1354,664501	1775,025092	1260,725647	0,71025793	-0,493585061	0,6912367	1	pre-mRNA splicing factor, putative
PFIT_0823100	718,5384984	1322,396868	1068,441812	820,3015456	1118,753617	865,7695667	1254,36317	1125,303315	985,7003179	1189,833243	1,207094308	0,271538395	0,6914782	1	phosphatidylglycerophosphate synthase
PFIT_0112000	496,7673569	181,8204419	191,6028438	158,7882869	88,49842041	92,03062323	182,5630607	118,5901583	201,5846622	150,5766095	0,746964615	-0,420888194	0,6915079	1	surface-associated interspersed protein 1.2 (SURFIN 1.2) (SURF1.2)
PFIT_0935200	5655,797739	2054,059851	2213,671023	1925,543337	2227,488544	3050,644733	1863,355816	2192,61474	2854,534205	2027,985278	0,710443502	-0,49320817	0,6915661	1	UBX domain, putative
PFIT_0703900	30775,49812	5602,84438	9470,445903	8137,7428	5662,229125	10941,41854	7687,18482	7016,367169	11765,02981	7351,775994	0,624883754	-0,678340262	0,6916988	1	methyltransferase, putative
PFIT_0503900	120,3900482	120,4834253	77,51870778	56,48595187	153,6198996	197,6954129	72,75575852	78,19131318	121,0322409	75,47353585	0,623582074	-0,681348639	0,691992	1	conserved Plasmodium protein, unknown function
PFIT_0620900	26,61253698	81,78268872	95,07011331	62,13454706	73,47038676	119,298956	103,7443223	71,02377613	76,39487148	87,38404924	1,143847061	0,193894169	0,6920237	1	conserved Plasmodium protein, unknown function
Pf_M7661102200	2,534527331	5,841620623	10,96962846	10,041947	359,0030262	132,9331224	10,77863089	14,33507408	86,88731201	12,55685249	0,144518828	-2,790670636	0,6921055	1	unspecified product
PFIT_0607600	2737,289518	4811,304785	5040,178623	4062,595183	4054,229524	5068,501361	5167,679849	4986,650998	4295,683166	5077,165423	1,181922695	0,241135677	0,6921506	1	chaperone, putative
PFIT_0607500	778,0998907	59,1464088	193,7967694	163,8092604	61,78191614	354,4883265	120,5859331	142,6991465	268,5204287	131,6425398	0,490251488	-1,028406084	0,6924476	1	conserved Plasmodium protein, unknown function
PFIT_0528900	111,5192026	287,6998157	290,3294999	284,3126244	200,3737821	426,0677001	294,3913563	321,2359783	266,7171041	307,8136673	1,154082968	0,206746945	0,6927186	1	mitochondrial import receptor subunit tom22, putative
PFIT_1217600	163,4770129	347,576427	208,4229407	244,1448364	569,3954974	586,2691554	233,7615575	223,4968368	353,2143116	228,6291972	0,647281805	-0,627534145	0,6928834	1	conserved protein, unknown function
PFIT_0517900	19,00895498	49,65377529	15,35747984	16,31816387	11,68847062	20,45124961	32,33589268	27,36695961	22,07968237	29,85142614	1,351986213	0,435080439	0,6929139	1	apicortin, putative
PFIT_1220100	3795,454678	619,211786	596,0164796	507,1183235	424,1245054	395,3908257	447,9868465	476,3154161	1056,219433	462,1511313	0,437552195	-1,192472971	0,6931776	1	debranching enzyme-associated ribonuclease, putative
PFIT_1461000	424,533328	689,3112335	493,6332807	350,8405233	611,1400353	480,6043657	708,6949812	471,7542562	508,3437944	590,2246187	1,161073717	0,215459572	0,693227	1	bax inhibitor 1, putative
PFIT_0107300	296,5396977	364,3710863	469,5000981	400,4226366	527,6509594	1077,099146	285,6337187	389,6533773	522,5972707	337,643548	0,646087469	-0,630198601	0,6932914	1	conserved Plasmodium protein, unknown function
PFIT_0905000	212,9002958	434,4705338	457,0678525	410,4645836	1218,940508	794,190193	377,9257457	377,2730861	588,0056611	377,5994159	0,642169695	-0,638973512	0,6933492	1	replication protein A1, small fragment
PFIT_1422400	357,3683537	630,1648247	620,1496622	522,8088657	504,2740182	739,6535274	660,8648066	642,4719566	562,4032086	651,6683816	1,158720952	0,212533173	0,6934116	1	DNA-directed RNA polymerase III subunit C, putative
PFIT_1354700	243,3146238	51,11418045	24,86449117	30,75346269	31,72584883	57,94520722	29,64123495	36,48927948	73,28630236	33,06525722	0,451179227	-1,148227451	0,6937765	1	conserved Plasmodium protein, unknown function
PFIT_0501500	22477,45564	15914,76518	23809,94422	38428,02068	18833,46573	45821,02474	23420,6176	18251,15568	27547,44603	20835,88664	0,756363643	-0,402848077	0,6938511	1	interspersed repeat antigen
PFIT_1429800	49,42328296	73,02025778	67,28038788	102,3023351	237,1089754	115,8904144	67,36644308	75,58493607	107,5042756	71,47568957	0,664863692	-0,5888695	0,6938996	1	peptide chain release factor 1, putative
PFIT_0911800	124,1918392	46,0027624	62,16122794	58,36881694	207,0529081	20,45124961	47,15651015	60,59826771	86,37146737	53,87738893	0,623786889	-0,680874865	0,6939407	1	conserved Plasmodium protein, unknown function
PFIT_0307300	78,57034727	59,87661138	76,05609065	85,98417119	317,2584883	160,2014552	63,99812092	77,5397189	129,6578607	70,76891991	0,5458128	-0,873521868	0,6939892	1	ATP-dependent Clp protease proteolytic subunit
PFIT_0807000	8522,348151	4838,322281	5503,096944	5435,203814	4725,481694	9318,952737	6878,787503	6586,314946	6390,567603	6732,551225	1,05351381	0,075209226	0,6940996	1	cactin homolog, putative
PFIT_0422600	240,7800965	113,9116021	79,71263347	82,21844106	41,74453793	54,53666562	92,29202702	64,50783337	102,1506628	78,39993019	0,767493113	-0,38177429	0,6941004	1	surface-associated interspersed protein 4.2 (SURFIN 4.2) (SURF4.2)
PFIT_1124800	1518,181871	2084,72836	2679,514578	2393,749116	2082,217552	3742,578678	2612,470663	2745,818281	2416,828359	2679,144472	1,108537336	0,148657362	0,6941519	1	splicing factor, putative
PFIT_0316900	2074,510621	1843,761509	2020,605562	2256,299967	1335,825214	1155,495603	2380,730098	2222,588077	1781,083079	2301,659088	1,292280587	0,36991935	0,6942782	1	RING zinc finger protein, putative
PFIT_0320400	286,4015884	240,9668507	350,2968021	492,055403	253,8067906	231,7808289	254,6451548	288,6562645	309,218044	271,6507097	0,878508596	-0,186871692	0,6943285	1	oocyst capsule protein
PFIT_1248000	11,40537299	135,0874769	60,69861081	97,28136156	141,931429	40,90249921	63,99812092	72,32696469	81,21779174	68,16254281	0,839256293	-0,252816645	0,6943402	1	dipeptidyl peptidase 2, putative
PFIT_1335700	107,7174116	202,2661141	236,9439747	306,9070052	171,9874963	173,8356217	238,4772085	289,959453	199,9429372	264,2183308	1,321468687	0,40214224	0,6945404	1	RanBPM and CLTH-like protein, putative
PFIT_0729700.1	15532,85075	12888,8057	11072,01166	10131,06928	8160,222275	9513,239608	14527,57345	12373,77531	11216,36655	13450,67438	1,199200679	0,262073106	0,6945813	1	Plasmodium exported protein, unknown function
PFIT_0833400	41469,93619	21224,79833	19393,57181	19934,52004	11907,212	8824,714205	19290,38098	15540,52349	20459,12543	17415,45223	0,85123151	-0,232376539	0,6945978	1	tryptophan/threonine-rich antigen
PFIT_0105400	97,57930225	86,89410676	95,07011331	107,3233086	389,0590935	143,1587472	81,51339612	95,78435864	153,1807786	88,64887738	0,578720634	-0,78906101	0,6947089	1	2-C-methyl-D-erythritol 4-phosphate cytidylyltransferase, putative
PFIT_0409200	353,5665627	711,2173108	455,6052353	412,9750704	719,6758339	824,8670675	646,7178536	588,3896316	579,6511801	617,5537426	1,065388571	0,09137971	0,6947124	1	conserved Plasmodium protein, unknown function
PFIT_0220100	5153,961328	4638,246774	5231,781466	4749,213309	4500,061189	3057,461816	5705,937729	5556,144395	4555,12098	5631,041062	1,236200111	0,3059123	0,6947496	1	Plasmodium exported protein (hyp9), unknown function
PFIT_1439100	6246,342608	6045,347142	6369,697592	5352,985373	5568,72136	8517,945461	7434,560658	6571,979872	6350,173256	7003,270265	1,102847117	0,14123281	0,6947622	1	HSP40, subfamily A, putative
PFIT_1252700	22,81074598	60,60681396	100,9205818	93,51563144	38,4049749	37,49395761	49,85116788	59,29507916	58,95878428	54,57312352	0,925614803	-0,111516157	0,6947757	1	Plasmodium exported protein, unknown function
PFIT_0905600	7319,714932	17681,12522	3777,940041	9338,383088	30962,75867	6708,009871	7516,747719	8759,381858	12631,32197	8138,064789	0,64427657	-0,634247966	0,6948865	1	high molecular weight rhoptry protein 3
PFIT_1367800	1182,357	418,4060771	468,7687895	504,6078367	617,8191614	248,8235369	437,2082156	455,4643992	573,4637336	446,3363074	0,778316537	-0,361571084	0,6949217	1	DNA repair endonuclease, putative
PFIT_1015500	179,9514405	533,0478818	133,8294672	295,6098148	247,1276645	64,76229042	227,6985776	218,9356769	242,3880932	223,3171272	0,921320533	-0,118224929	0,6949836	1	conserved Plasmodium protein, unknown function
PFIT_0416900	6902,785186	4100,087474	5158,65061	7852,802554	2354,391939	5753,618223	4980,401137	4056,174371	5353,722664	4518,287754	0,843952524	-0,244766252	0,6949956	1	conserved Plasmodium protein, unknown function
PFIT_0535700	32,94885531	70,82965005	62,8925365	52,72022175	63,45169765	85,21354003	77,47140954	63,85623909	61,34275021	70,66382432	1,151950704	0,204078981	0,6950148	1	unspecified product
PFIT_0712100	1606,890328	5375,021175	4974,360852	5507,380308	7701,032357	4175,463461	5600,172413	6112,605907	4890,024747	5856,38916	1,197619534	0,260169658	0,6950429	1	histone H2B variant (H2B.Z)
PFIT_0707400	6117,081714	2549,137199	2733,631412	2939,152363	1711,526055	3207,437647	2105,201346	2818,145246	3209,661065	2461,673296	0,766957397	-0,382781654	0,6951346	1	heat shock protein 86 family protein
PFIT_1336700	8799,878894	6377,589315	10153,4881	8874,570661	5643,861528	10873,24771	9300,611131	10190,93448	8453,772701	9745,772808	1,152831186	0,205181268	0,6952031	1	conserved Plasmodium protein, unknown function
PFIT_1035100	1632,235601	1664,131675	739,3529581	1609,849628	2573,133318	1431,587472	1302,193345	1088,814036	1608,381776	1195,50369	0,743295969	-0,427991311	0,6953199	1	duffy binding-like merozoite surface protein
PFIT_0723400	81,1048746	257,0313074	325,432311	247,9105666	272,1743873	351,0797849	299,1070073	295,1722072	255,788872	297,1396073	1,161659634	0,216187421	0,6954392	1	mago nashi protein homolog, putative
PFIT_1235200	634,8990965	495,8075503	588,703394	552,9347067	744,7225567	1162,312686	520,0689406	424,8394683	696,5633317	472,4542044	0,678264535	-0,560080035	0,6954919	1	mRNA methyltransferase, putative
PFIT_0609100	3225,186029	866,0202573	1056,009566	1056,2873	681,270859	1748,581841	1131,756244	808,6284971	1438,892642	970,1923704	0,674263209	-0,568616215	0,6955004	1	conserved Plasmodium protein, unknown function
PFIT_0301200	0	0,730202578	0	1,255243375	1,669781517	3,408541601	0	2,606377106	1,177294845	1,303188553	1,106934731	0,146570158	0,6955973	1	alpha/beta hydrolase, putative
PFIT_1308900	2426,80992	2490,720993	3414,479685	2749,610613	2649,943268	5058,275736	3285,461429	3413,05082	3131,640036	3349,256125	1,069489496	0,096922314	0,6956991	1	conserved Plasmodium protein, unknown function
PFIT_0529500	296,5396977	504,5699813	195,2593866	526,5745958	434,1431945	214,7381209	315,948618	312,1136584	361,9708295	314,0311382	0,867559241	-0,204965819	0,695957	1	conserved Plasmodium protein, unknown function
PFIT_1312400	115,3209936	67,17863716	56,31075942	46,44400487	105,1962356	160,2014552	63,32445649	46,26319363	91,77534764	54,79382506	0,597042958	-0,744093355	0,6959656	1	methionyl-tRNA formyltransferase, putative
PFIT_0916000	980,8620772	411,1040513	570,4206799	576,7843308	375,7008414	678,2997786	396,7883497	508,8951299	598,8619599	452,8417398	0,756170487	-0,403216552	0,6959678	1	conserved Plasmodium protein, unknown function
PFIT_0721800	167,2788039	448,3443828	453,4113096	375,3177691	455,8503542	589,677697	461,4601351	473,0574447	414,9800528	467,2587899	1,125978916	0,171179813	0,6960372	1	conserved Plasmodium protein, unknown function
PFIT_0816000	83,63940193	405,9926333	110,4275932	142,4701231	733,0340861	197,6954129	162,3531278	159,6405977	278,8765417	160,9968628	0,577305147	-0,792594008	0,6960919	1	conserved Plasmodium protein, unknown function
PFIT_1026900	27,87980064	88,35451192	133,8294672	120,503364	93,50776496	204,5124961	103,0706579	144,0023351	111,4312341	123,5364965	1,108634374	0,148783645	0,6961307	1	conserved Plasmodium protein, unknown function
PFIT_1420100	4865,025212	2310,360956	2465,241169	2524,922049	1985,370224	2362,11933	2235,218581	2075,327771	2752,173157	2155,273176	0,783116851	-0,352700503	0,6962375	1	ATP-dependent RNA helicase, putative
PFIT_0805600	494,2328296	871,8618779	207,6916322	410,4645836	1295,750457	252,2320785	411,6089672	416,3687427	588,7055765	413,9888549	0,703218844	-0,507954364	0,6962431	1	serine/threonine protein phosphatase, putative
PFIT_0309300	0	1,460405156	5,119159948	1,255243375	28,38628579	34,08541601	8,083973169	7,819131318	11,71775171	7,951552243	0,678590265	-0,559387361	0,6962586	1	small nucleolar RNA Me18S-Um1356
PFIT_1205900	481,5601929	406,7228358	182,0958324	316,3213305	429,1338499	228,3722873	286,3073831	242,3930708	340,7010548	264,350227	0,775900818	-0,366055849	0,696274	1	conserved Plasmodium protein, unknown function
PFIT_1402300	106037,02	77443,825	88234,57217	88922,69593	71702,08813	103916,2078	91588,72135	111437,6079	89376,06816	101513,1646	1,135798058	0,18370635	0,696341	1	early transcribed membrane protein 14.1
PFIT_1348100	15,20716399	86,89410676	95,07011331	85,98417119	66,79126069	57,94520722	60,62979877	56,68870205	67,98200386	58,65925041	0,862864392	-0,212794252	0,6964373	1	conserved Plasmodium protein, unknown function
PFIT_1007400	319,3504437	195,6942909	481,9323436	492,055403	120,2242692	524,9154066	363,7787926	491,3020845	355,6953595	427,5404385	1,201984865	0,26541873	0,6964626	1	conserved Plasmodium protein, unknown function
PFIT_1473900	1316,686949	261,4125229	227,4369634	259,8353786	435,812976	296,5431193	222,9829266	301,68815	466,2879848	262,3355383	0,562604113	-0,829807994	0,6965099	1	conserved Plasmodium protein, unknown function
PFIT_1016900	299,0742251	682,7394103	532,3926346	406,6988535	576,0746234	845,3183171	681,0747395	531,7009296	557,0496773	606,3878346	1,08857048	0,122434818	0,6967057	1	phosphomannomutase, putative
PFIT_1140800	399,1880547	457,8370163	261,8084659	208,3704002	434,1431945	576,0435306	251,2768327	279,5339446	389,5651104	265,4053886	0,681286341	-0,553666812	0,696866	1	vacuolar ATP synthase subunit f, putative
PFIT_1243000	3296,152794	5244,314914	5097,951999	4157,366058	3746,989725	3459,669725	5321,949003	5128,69855	4167,074202	5225,323777	1,253955059	0,326485644	0,6968734	1	rab specific GDP dissociation inhibitor
PFIT_0213100	335,8248714	473,901473	321,7757681	234,1028894	424,1245054	889,6293579	431,1452357	418,9751198	446,5598109	425,0601777	0,951854975	-0,071186314	0,6970469	1	conserved protein, unknown function
PFIT_0717200	181,2187042	255,5709022	212,0794835	171,3407207	195,3644375	361,3054097	200,7520004	291,9142359	229,479943	246,3331181	1,073440733	0,102242539	0,6974024	1	conserved Plasmodium protein, unknown function
PFIT_0511700	125,4591029	94,92633512	75,32478209	79,08033262	143,6012105	71,57937362	117,8912754	103,60349	98,3285228	110,7473827	1,126299669	0,171590729	0,6974422	1	conserved Plasmodium protein, unknown function
PFIT_0208000.1	68,43223794	226,3627991	247,1822946	182,6379111	171,9874963	272,6833281	200,7520004	258,6829278	194,8810112	229,7174641	1,178757554	0,237267016	0,6976514	1	conserved Plasmodium protein, unknown function
PFIT_1473600	30,41432797	45,27255982	31,44626825	50,83735669	35,06541186	37,49395761	52,5458256	45,61159935	38,42164703	49,07871248	1,277371385	0,353178037	0,6976811	1	conserved Plasmodium protein, unknown function
PFIT_1469900	2358,377682	1182,197973	1221,285302	1253,988132	828,2116325	2730,241822	1237,521559	961,1015578	1595,717091	1099,311559	0,688913821	-0,537604573	0,6977997	1	U2 snRNP auxiliary factor, putative
PFIT_1227100	2694,202553	1475,73941	1612,535383	1695,206178	1290,741113	1567,929136	1324,424271	1401,579289	1722,725629	1363,00178	0,791189123	-0,337905502	0,6978217	1	conserved Plasmodium protein, unknown function
PFIT_0111700	2211,375096	4874,83241	5321,00111	5671,81719	4077,606465	2866,583486	4906,298049	6146,48881	4170,53596	5526,39343	1,325103891	0,406105475	0,6979768	1	Plasmodium exported protein (hyp1), unknown function
PFIT_0814100	1399,059087	2815,66114	3273,337132	2828,063324	2838,628579	3531,249099	3096,161724	3370,065598	2780,999727	3233,103661	1,162568852	0,217316161	0,6980676	1	conserved protein, unknown function
PFIT_1116100	7756,920897	4428,678634	6382,861146	6077,2608	3244,385488	4935,568238	5980,119152	7255,502268	5470,945867	6617,81071	1,209628256	0,274563746	0,6983093	1	cysteine proteinase falcipain 2b
PFIT_0516900	472,6893473	266,5239409	175,5140553	250,4210533	1010,217818	153,384372	241,1718662	211,1165456	388,1250978	226,1440059	0,582658033	-0,779278694	0,6983128	1	transcription factor with AP2 domain(s)
PFIT_0909300	145,7353215	102,2283609	51,92290804	45,81638319	158,6292441	30,67687441	77,47140954	50,17275929	89,16818203	63,82208441	0,71574953	-0,482473276	0,698352	1	conserved Plasmodium membrane protein, unknown function
PFIT_0312200	210,3657685	716,3287288	786,880148	618,8349839	849,9187923	964,6172731	741,0308739	804,0673372	691,1589269	772,5491055	1,117758992	0,160609152	0,6983933	1	26S proteasome regulatory subunit S14, putative
PFIT_0532900	966,9221768	1637,844382	2358,470119	2499,817181	1539,538559	2338,259538	2108,569668	2502,773616	1890,141993	2305,671642	1,219840441	0,286692451	0,6985812	1	peptidyl-prolyl cis-trans isomerase
PFIT_1111700	4417,681138	2028,502761	2588,101008	2947,311444	1379,239533	1203,215185	1934,764245	2279,928373	2427,341845	2107,346309	0,868170387	-0,203949881	0,6987652	1	E52 protein, putative
PFIT_0619900	475,2238746	344,6556167	424,1589671	286,1954895	399,0777826	818,0499842	281,5917321	325,7971382	457,8936191	303,6944351	0,663242339	-0,592391988	0,6988716	1	conserved Plasmodium protein, unknown function
PFIT_1458500	2827,265238	5136,244932	7795,057983	8100,71312	5406,752553	6135,374882	6665,909543	8152,095993	5900,228118	7409,002768	1,25571463	0,328508639	0,6990062	1	conserved Plasmodium protein, unknown function
PFIT_0523500	3450,758961	1081,430018	1615,460618	1971,987342	915,0402714	1295,245808	1171,502445	1612,695834	1721,653836	1392,09914	0,808582486	-0,306533139	0,6990209	1	conserved Plasmodium protein, unknown function, putative
PFIT_0514300	5003,156952	761,6012887	561,6449771	471,971509	532,660304	572,634989	503,9009942	495,8632444	1317,278337	499,8821193	0,379481014	-1,397900386	0,6995259	1	conserved Plasmodium protein, unknown function
PFIT_0932400	24,07800965	175,9788213	68,01169645	131,1729327	116,8847062	37,49395761	86,90271157	82,75247311	92,27002064	84,82759234	0,919340776	-0,121328365	0,6995648	1	adenylate kinase-like protein 2 (AKLP2)
PFIT_1360900	20,27621865	176,7090238	196,7220037	182,0102894	953,4452463	163,6099968	133,3855573	110,1194327	282,1287965	121,752495	0,431549337	-1,21240259	0,6996189	1	proliferating cell nuclear antigen
PFIT_1121300	1630,968338	2138,033148	2292,652348	2410,06728	1896,871804	1820,161215	2555,88285	2474,103468	2031,459022	2514,993159	1,238023082	0,308038213	0,6997439	1	tRNA nucleotidyltransferase, putative
PFIT_0307500	13,93990032	77,40147325	73,13085639	86,61179287	424,1245054	102,256248	54,56681889	57,34029633	129,5774627	55,95355761	0,431815506	-1,211513045	0,6997528	1	conserved Plasmodium protein, unknown function
PFIT_1443500	1551,130727	2937,604971	3763,31387	4164,269897	2960,52263	3384,68181	3781,27845	3953,87407	3126,920651	3867,57626	1,236864216	0,306687129	0,699776	1	tRNA binding protein, putative
PFIT_0501200.1	3823,334479	6473,976055	6899,164992	7788,785142	4857,394434	4008,444923	7588,829813	7333,041987	5641,850004	7460,9359	1,322427199	0,403188303	0,6998138	1	heat shock protein 40, type II
PFIT_0519200	731,211135	181,8204419	311,5374482	269,8773256	248,7974461	286,3174945	297,7596784	438,5229481	338,2602152	368,1413132	1,088337607	0,122126156	0,7001049	1	mitochondrial ribosomal protein L14 precursor, putative
PFIT_1409800	2,534527331	4,381215467	4,387851384	8,786703625	3,339563034	6,817083202	9,431302031	5,212754212	5,041157341	7,322028121	1,45244983	0,538488331	0,7001671	1	conserved Plasmodium protein, unknown function
PFIT_0527700	0	4,381215467	4,387851384	0,627621687	0	0	4,715651015	1,954782829	1,566114756	3,335216922	2,129612092	1,090590668	0,7002714	1	outer arm dynein lc3, putative
PFIT_1131110	17,74169132	10,22283609	13,16355415	5,648595187	155,2896811	122,7074976	9,431302031	16,94145119	54,12897591	13,18637661	0,24361031	-2,037352902	0,7003057	1	small nucleolar RNA snoR14a
PFIT_1017000	3374,723141	4018,304786	1713,455965	3830,375159	2394,466696	923,7147739	2605,734018	2375,712732	2709,17342	2490,723375	0,919366533	-0,121287945	0,7003402	1	conserved Plasmodium protein, unknown function
PFIT_0811800	25,34527331	5,841620623	19,01402266	16,31816387	53,43300855	17,04270801	23,57825508	23,45739395	22,83246617	23,51782452	1,030016834	0,042667916	0,7003615	1	unspecified product
PFIT_0902700	3981,742437	4798,891341	4169,190123	4780,594394	2985,569353	1342,965391	5229,656976	4761,199378	3676,492173	4995,428177	1,358748487	0,442278429	0,7004283	1	serine/threonine protein kinase, FIKK family
PFIT_1227400	5544,278537	3517,385817	3015,956158	3572,422645	3022,304546	1963,319962	3055,068194	2680,658853	3439,271338	2867,863523	0,833857885	-0,26212657	0,7005951	1	conserved Plasmodium protein, unknown function
PFIT_0718500	30,41432797	18,98526702	29,2524256	50,83735669	36,73519338	34,08541601	12,79962418	35,83768521	33,38498394	24,3186547	0,728430924	-0,457135925	0,7007469	1	conserved Plasmodium protein, unknown function
PFIT_0710600	394,119	364,3710863	272,0467858	244,7724581	362,3425892	783,9645682	289,0020408	229,3611853	403,602748	259,1816131	0,64217009	-0,638972623	0,7007658	1	erythrocyte membrane protein 1, PfEMP1, putative
PFIT_1036800	1304,014312	2444,718231	3951,26017	3998,577771	2875,363773	4437,921165	3769,15249	3668,475776	3168,64257	3718,814133	1,173630048	0,230977714	0,7008697	1	DNA repair helicase rad25, putative
PFIT_0100400	29,14706431	10,22283609	2,193925692	6,903838562	6,679126069	3,408541601	7,410308739	3,257971382	9,759222054	5,33414006	0,54657431	-0,871510443	0,7009424	1	rifin (RIF)
PFIT_1329000	1913,568135	779,1261505	239,869209	508,3735669	587,7630941	207,9210377	416,3246182	501,7275929	706,1035322	459,0261056	0,650083288	-0,621303529	0,7009891	1	conserved Plasmodium protein, unknown function
PFIT_1412700	1916,102662	370,9429095	473,8879494	379,7111209	328,9469589	569,2264474	369,8417725	410,5043942	673,1363414	390,1730833	0,579634554	-0,786784492	0,7012696	1	conserved protein, unknown function
PFIT_0313800	444,8095466	357,7992631	375,1612933	296,2374365	213,7320342	105,6647896	351,6528329	432,0070053	298,9007272	391,8299191	1,310903198	0,390561155	0,7012929	1	conserved Plasmodium protein, unknown function
PFIT_1113300	138,1317395	331,5119703	392,7126988	283,0573811	338,965648	432,8847833	357,7158127	371,4087376	319,5440368	364,5622752	1,140882736	0,190150514	0,7013932	1	conserved Plasmodium protein, unknown function
PFIT_0208600	87,44119292	176,7090238	124,3224559	110,461417	212,0622527	139,7502056	153,5954902	167,4597291	141,7910913	160,5276096	1,132141717	0,179054561	0,7014327	1	2C-methyl-D-erythritol 2,4-cyclodiphosphate synthase
PFIT_0620600	527,1816849	501,649171	1003,35535	814,0253287	549,3581192	1878,106422	842,0805385	925,2638726	878,9460126	883,6722055	1,005377114	0,007736754	0,7014495	1	conserved Plasmodium protein, unknown function
PFIT_0506600	418,1970096	178,169429	163,8131183	224,0609424	327,2771774	354,4883265	181,2157319	192,8719058	277,6676672	187,0438189	0,673624771	-0,569982903	0,7015089	1	histone deacetylase, putative, pseudogene
PFIT_1339900	122,9245756	85,43370161	87,02571911	85,98417119	353,9936816	149,9758304	78,8187384	93,82957581	147,5562799	86,32415711	0,585025301	-0,773429075	0,7016048	1	Rab GTPase 11b
PFIT_1100100	0	24,09668507	28,5230339	16,31816387	26,71650428	20,45124961	22,1003339	27,36695961	19,35060614	24,79894291	1,281558972	0,357899867	0,7016613	1	erythrocyte membrane protein 1, PfEMP1, putative
PFIT_1324600	918,7661575	858,7182315	422,69635	571,1357356	2987,239134	451,3360329	536,9105513	563,6290491	1035,315274	550,2698002	0,531499741	-0,911859105	0,7016708	1	conserved Plasmodium protein, unknown function
PFIT_0913800	1558,734309	732,3931856	555,7945086	656,4922851	988,5106582	1158,904144	606,2979877	684,8255846	941,8048484	645,5617861	0,685451755	-0,544872968	0,7017118	1	conserved Plasmodium protein, unknown function
PFIT_bin03900	0	1,460405156	0,731308564	1,255243375	0	17,04270801	1,347328862	3,257971382	3,414944183	2,302650122	0,674286313	-0,568566782	0,701808	1	rifin (RIF)
PFIT_0936100	4683,806508	11424,74953	11699,47441	14452,2446	7023,101061	3981,17659	12928,28204	12471,54691	8877,425469	12698,91582	1,430585466	0,51660569	0,7018602	1	gametocytogenesis-implicated protein
PFIT_0525500	3538,200154	1068,286371	1663,726983	1959,434908	854,9281368	1274,794559	1248,973855	1558,613509	1726,561852	1403,793682	0,813057279	-0,298571102	0,7018895	1	conserved Plasmodium protein, unknown function, putative
PFIT_1466800	1,267263666	4,381215467	3,65654282	2,51048675	1,669781517	3,408541601	4,041986585	4,561159935	2,815638637	4,30157326	1,52774337	0,61140222	0,7020832	1	plasmepsin VIII
PFIT_0219700	7,603581993	89,81491707	220,1238777	238,4962412	205,3831266	937,3489403	84,88171828	112,0742156	283,1284475	98,47796692	0,347820813	-1,523583829	0,7021728	1	Plasmodium exported protein, unknown function
PFIT_0203200	145,7353215	97,87414543	91,41357049	65,2726555	121,8940508	61,35374882	80,16606726	65,81102192	97,25741678	72,98854459	0,750503667	-0,414068973	0,7022329	1	5'-3' exonuclease, N-terminal resolvase-like domain, putative
PFIT_1324000	468,8875563	508,2209942	313,7313739	269,2497039	459,1899172	783,9645682	427,1032491	493,9084616	467,2073523	460,5058553	0,985656268	-0,020843477	0,7024541	1	dihydrofolate synthase/folylpolyglutamate synthase
PFIT_0421400	144,4680579	178,169429	171,126204	133,6834194	302,2304546	269,2747865	144,8378526	129,0156667	199,8253919	136,9267067	0,685213034	-0,545335498	0,7029251	1	BSD domain, putative
PF_apicoplast_gen	82,37213826	22,63627991	22,67056548	19,45627231	16,69781517	13,6341664	17,5152752	20,85101685	29,57787292	19,18314602	0,648564083	-0,624678965	0,7029919	1	apicoplast ribosomal protein S7
PFIT_1228300	6537,812351	3617,423571	1316,355415	2696,262769	12853,97812	2491,64391	2881,26277	2483,877382	4918,912839	2682,570076	0,545358327	-0,874723633	0,7030006	1	merozoite surface protein 9
PFIT_0227800	225,5729325	449,8047879	364,1966648	528,4576609	394,0684381	207,9210377	317,9669213	337,5258352	361,669387	327,7477233	0,906208087	-0,142085729	0,7031763	1	conserved Plasmodium protein, unknown function
PFIT_1138900	48,15601929	44,54235725	40,22197102	57,11357356	45,08410096	13,6341664	61,30346332	50,82435356	41,45869808	56,06390838	1,3522838	0,43539751	0,7032274	1	conserved Plasmodium membrane protein, unknown function
PFIT_1012100	1970,595	3079,264271	4351,285955	4095,859133	2616,547637	2437,107245	4097,227068	3975,376681	3091,77654	4036,301874	1,30549599	0,384598026	0,7033443	1	GMP synthetase
PFIT_0810200	603,2175048	1743,723756	2340,918713	2122,615547	2250,865485	2167,832458	2004,826443	2422,6752	1871,529077	2213,726433	1,182843729	0,242259485	0,7033819	1	Rab GTPase 18
PFIT_1038300	100,1138296	156,2633517	114,084136	95,3984965	71,80060524	218,1466625	142,8168593	131,6220438	125,9678469	137,2194516	1,089321243	0,123429471	0,7035571	1	rifin, pseudogene
PFIT_0821900	18707,34623	3541,67311	4085,089638	3901,296409	3167,575538	4304,988042	3839,887255	3368,742409	6284,994828	3604,314832	0,573479363	-0,802186523	0,7035707	1	conserved Plasmodium protein, unknown function
PFIT_1306000	106,4501479	94,19613254	105,3085838	86,61179287	171,9874963	143,1587472	86,2294714	76,88812662	117,952125	81,55858098	0,691454694	-0,532292744	0,7036132	1	conserved Plasmodium protein, unknown function
PFIT_1335900	12009,85776	3475,034068	5058,461337	5328,508127	2974,747487	4591,305537	4083,080115	4552,037615	5551,690719	4317,55865	0,77770162	-0,362171351	0,7036308	1	conserved Plasmodium protein, unknown function
PFIT_1411200	2846,274193	4839,782686	5051,552582	5394,408404	3695,226498	5814,971971	5526,069326	5778,989638	4707,036056	5652,529482	1,200868108	0,264077708	0,7036732	1	cytidine triphosphate synthetase
[illegible]	[illegible]	[illegible]	[illegible]	[illegible]	[illegible]	[illegible]	[illegible]	[illegible]	[illegible]	[illegible]	[illegible]	[illegible]	[illegible]	[illegible]	[illegible]
PFIT_1216300	19,00895498	66,44843458	84,83379342	167,5749906	83,48907586	37,49395761	74,10308739	74,93334179	76,4745345	74,51821459	0,974418675	-0,037386313	0,7357506	1	cell traversal protein for ookinetes and sporozoites
PFIT_0914700	139,3990032	696,6132593	634,7758335	679,7142876	564,3861528	293,1345777	744,399196	592,2991973	501,3371857	668,3491067	1,3331331	0,414820827	0,7358476	1	zinc binding protein, putative
Pf_M7661102300	0	4,381215467	0,731308564	3,765730125	21,70715072	10,2256248	6,737651015	7,167537041	6,80183978	5,941594028	0,87352749	-0,19507499	0,7358798	1	unspecified product
[illegible]	[illegible]	[illegible]	[illegible]	[illegible]	[illegible]	[illegible]	[illegible]	[illegible]	[illegible]	[illegible]	[illegible]	[illegible]	[illegible]	[illegible]	[illegible]

PFIT_1145200	48,15601929	300,1132595	337,8645565	245,4000798	218,7413788	228,3722873	308,5383093	270,4116247	229,7745969	289,474967	1,259821455	0,333219286	0,7373984	1 ubiquitin activating enzyme (E1) subunit Aos1, putative
PFIT_1446400	5800,265797	3272,767954	3557,816164	3849,831431	3182,603572	5276,422398	4535,108948	4280,322802	4156,617886	4407,715875	1,060409207	0,084621101	0,7374909	1 RNA helicase, putative
PFIT_0918800	3567,347219	2498,753221	2745,332349	4600,466969	2224,148981	906,6720659	2599,671038	2684,568419	2757,120134	2642,119729	0,958289665	-0,061466286	0,7377027	1 conserved Plasmodium protein, unknown function
PFIT_1239100	16,47442765	2,920810311	5,119159948	4,393351812	0	6,817083202	9,431302031	4,561159935	5,954138821	6,996230983	1,175019796	0,232685063	0,7378113	1 conserved Plasmodium protein, unknown function
PFIT_1020500	148,2698489	194,2338857	161,6191926	199,5836966	322,2678328	163,6099968	142,1431949	168,7629176	198,2640756	155,4530562	0,784070719	-0,350944312	0,737947	1 conserved Plasmodium protein, unknown function
PFIT_1014200	217,9693505	118,2928176	217,1986435	161,9263954	272,1743873	872,5866499	140,1222016	138,1379866	310,0247074	139,1300941	0,448770988	-1,155948684	0,7380578	1 prohibitin, putative
PFIT_1002600	3927,2501	896,6887656	446,098224	665,2789887	1013,557381	388,5737425	622,465934	716,1021098	1222,907867	669,2840219	0,547288999	-0,869625238	0,7380633	1 conserved Plasmodium protein, unknown function
PFIT_0726000	69,69950161	91,27532223	81,1752506	90,377523	308,9095807	146,5672888	77,47140954	87,96522732	131,3340778	82,71831843	0,629831342	-0,666962544	0,738383	1 conserved Plasmodium protein, unknown function
PFIT_1220800	34,21611897	19,7154696	10,96962846	20,71151569	25,04672276	34,08541601	18,18893963	11,72869698	24,12414525	14,9588183	0,620076614	-0,689481615	0,7383941	1 conserved Plasmodium protein, unknown function
PFIT_0916200	101,3810932	229,2836094	255,9579974	209,6256436	225,4205048	299,9516609	221,6355977	271,063219	220,2700849	246,3494084	1,118397028	0,161432432	0,7385182	1 thioredoxin 3
PFIT_0302600	40,5524373	29,20810311	13,16355415	11,29719037	78,47973131	27,26833281	24,92558394	14,33507408	33,32822484	19,63032901	0,589000137	-0,763660124	0,7385447	1 CDGSH iron-sulfur domain-containing protein, putative
PFIT_1358500	3155,486527	5831,397786	6043,533972	5609,682643	4536,796382	6309,210504	6049,506588	6184,932872	5247,684636	6117,21973	1,165698809	0,221195076	0,7385546	1 rRNA associated RNA binding protein, putative
PFIT_1112800	54,49233762	289,1602208	171,126204	162,5540171	171,9874963	37,49395761	245,2138528	165,5049462	147,8023722	205,3593995	1,389418833	0,474481558	0,7385655	1 conserved Plasmodium protein, unknown function
PFIT_1253200	1042,957997	389,9281766	468,0374809	415,4855571	404,0871272	422,6591585	389,378041	426,1426568	523,8592495	407,7603489	0,778377683	-0,361457748	0,7385709	1 gametocyte erythrocyte cytosolic protein
PFIT_0812200	1701,935103	507,4907916	606,9861081	979,7174542	661,2334808	501,0556154	619,7712763	732,3919667	826,4030921	676,0816215	0,818101515	-0,289648223	0,738629	1 unspecified product
PFIT_1362100	354,8338264	394,309392	364,9229734	332,6394944	397,4080011	316,9943689	414,9772894	413,1107713	360,184676	414,0440303	1,149532609	0,201047392	0,7386504	1 conserved Plasmodium protein, unknown function
PFIT_1216600	11367,35508	11308,64732	16533,42401	16718,58651	10257,46786	19711,59608	14908,86752	17396,26399	14316,17948	16152,56576	1,128273488	0,174116812	0,7387025	1 DNA-binding chaperone, putative
PFIT_1117600	47168,8209	34517,40606	45451,55856	47693,59965	37616,83802	58265,61013	49184,24009	49394,75572	45118,97222	49289,49791	1,092433969	0,12754608	0,7389967	1 heat shock protein 101
PFIT_1364400	248,3836785	432,2799261	386,8622303	397,2845282	367,3519338	487,4214489	312,5802959	314,0684413	386,597291	313,3243686	0,810467057	-0,303174549	0,7390522	1 ubiquitin-activating enzyme
PFIT_0421800	5050,045707	2702,479741	3301,858166	3537,903452	1968,672409	3384,68181	2749,224542	2718,451321	3324,273548	2733,837932	0,822386573	-0,282111384	0,7391124	1 conserved Plasmodium protein, unknown function
PFIT_0527000	515,7763119	459,2974214	466,5748638	441,2180463	360,6728077	787,3731098	538,2578802	519,9722326	505,1520935	529,1150564	1,047437125	0,066863645	0,7391278	1 G10 protein, putative
PFIT_1347200	117,8555209	108,8001841	72,39954783	76,56984587	193,694656	64,76229042	86,22904714	71,02377613	105,6803409	78,62641164	0,744002252	-0,426621106	0,7391317	1 conserved Plasmodium protein, unknown function
PFIT_1238300	48,15601929	266,5239409	127,9789987	257,9525136	576,0746234	139,7502056	175,152752	164,2017577	236,0727169	169,6772548	0,718749956	-0,476438133	0,7392908	1 sphingomyelin phosphodiesterase, putative
PFIT_1436200	21,54348231	67,90883974	53,38552517	54,60308681	217,0715972	105,6647896	53,21949003	48,21797646	86,69622015	50,71873325	0,585016661	-0,773450382	0,7393008	1 cytochrome c oxidase assembly protein, putative
PFIT_1227300	286,4015884	506,0303864	218,6612606	278,6640292	1861,806392	446,5189497	314,6012892	269,1084362	599,6804344	291,8548627	0,486683984	-1,038942797	0,7393496	1 cyclin related protein, putative
PFIT_1112500	2003,543855	665,2145484	777,3810035	680,3419092	574,4048419	484,0129073	671,6434375	683,522396	864,1498443	677,5829168	0,784103499	-0,350883997	0,7393707	1 conserved Plasmodium protein, unknown function
PFIT_0109700	0	2,920810311	1,462617128	2,51048675	21,70715972	6,817083202	2,694657723	0,651594276	5,903026186	1,673126	0,2834353	-1,818908646	0,7394169	1 conserved Plasmodium protein, unknown function
PFIT_1214100	30,41432797	49,65377529	103,8458161	99,16422662	26,71650428	143,1587472	46,48284572	71,02377613	75,49223291	58,75331093	0,778269613	-0,361658065	0,7395056	1 conserved Plasmodium protein, unknown function
PFIT_0420800	19,00895498	105,8793738	223,049112	217,1571039	120,2242692	146,5672888	119,9122687	132,2736381	138,6476838	126,0929534	0,909448683	-0,136935861	0,7395245	1 conserved Plasmodium protein, unknown function
PFIT_0530800	462,5512379	476,0920807	691,0865929	593,1024947	1117,083835	1864,472256	784,8190619	727,1792125	867,3980828	755,9991372	0,871571141	-0,198309667	0,7396349	1 conserved Plasmodium protein, unknown function
PFIT_0931600	238,2455691	343,9254142	304,2243626	192,0522364	420,7849423	351,0797849	346,2635174	306,2493099	308,3853849	326,2564137	1,05795031	0,081271869	0,7398696	1 selenoprotein
PFIT_1031000	1748,823858	268,7145486	333,4767052	437,4523162	175,3270593	378,3481177	332,7902288	313,416847	557,0237676	323,1035379	0,580053414	-0,785742339	0,7399711	1 conserved Plasmodium protein, unknown function
PFIT_0604600	22,81074598	51,84438303	19,74533123	35,77443619	76,80994979	10,2256248	41,76719471	44,96000508	36,20174517	43,36359989	1,197831753	0,260425282	0,740127	1 conserved Plasmodium protein, unknown function
PFIT_1401900	34,21611897	84,70349903	36,5654282	23,22200244	28,38628579	10,2256248	59,28246991	40,39884514	36,21982654	49,84065752	1,376060083	0,460543464	0,7402053	1 erythrocyte membrane protein 1, PfEMP1, putative
PFIT_0821100	13550,85038	17428,47513	21150,90629	22840,40845	19431,24752	17724,41633	21211,67193	23110,0942	18687,71735	22160,88307	1,18585286	0,245925012	0,7402509	1 14-3-3 protein (14-3-3I)
PFIT_1455600	7319,714932	8064,357269	8698,915368	8164,730533	7627,561971	13180,83037	9225,160715	9537,385425	8842,685074	9381,27307	1,060907744	0,085299205	0,7404401	1 aminopeptidase P
PFIT_1011400	134,3299486	427,168508	273,5094029	350,8405233	724,6851785	361,3054097	255,3188193	325,7971382	378,6398285	290,5579787	0,767372994	-0,382000102	0,7406303	1 heme oxygenase
PFIT_1236900	2500,311212	2823,693368	3221,414224	3284,971912	2346,043032	3149,492439	2546,451548	2347,042584	2887,654365	2446,747066	0,847312994	-0,239033101	0,7406615	1 conserved Plasmodium protein, unknown function
PFIT_1468600	485,3619839	868,210865	1133,528274	941,4325312	712,9967078	893,0378995	1025,317264	1000,197214	839,0947103	1012,757239	1,206964156	0,271382832	0,740671	1 DNA/RNA-binding protein KIN17, putative
PFIT_0828600	8,870845659	13,87384898	0,731308564	1,882865062	15,02803365	3,408541601	10,10496646	6,515942765	7,299240587	8,310454613	1,138536881	0,187181026	0,7407891	1 conserved Plasmodium protein, unknown function
PFIT_0810100	263,5908424	663,0239407	609,9113423	584,3157911	564,3861528	855,5439419	586,0880548	720,0116755	590,1286685	653,0498651	1,106622843	0,14616361	0,7409375	1 conserved Plasmodium membrane protein, unknown function
PFIT_0620800	2986,94046	4018,304786	6034,026961	6202,157516	3651,812178	4390,201582	5084,145459	6162,127072	4547,24058	5623,136266	1,236604082	0,306383674	0,7411018	1 conserved Plasmodium protein, unknown function
PFIT_1306400	1181,089736	507,4907916	637,7010678	503,9802151	556,0372452	981,6599811	514,6796251	534,958901	727,9931728	524,819263	0,720912342	-0,472104247	0,7411599	1 DnaJ protein, putative
PFIT_0402800	276,2634791	152,6123388	144,0677871	128,0348242	222,0809418	132,9331224	132,0382284	134,228421	175,9987489	133,1333247	0,756444722	-0,402693435	0,7412106	1 transcription factor with AP2 domain(s), putative

[illegible]

PFIT_1110700	9107,823965	5483,091157	6348,489644	5597,130209	5742,378638	10794,85125	7083,58149	7003,335283	7178,96081	7043,458387	0,981125064	-0,027491046	0,7813343	1	apicoplast ribosomal protein L36e precursor, putative
PFIT_0305000	3934,853682	1188,039594	1192,764268	1132,857146	1377,569752	1486,124138	1401,89568	1152,018681	1718,70143	1276,957181	0,742977901	-0,428608795	0,7814221	1	conserved Plasmodium protein, unknown function
PFIT_0607900	28812,5067	15509,50275	14994,01949	15603,30277	11751,92232	13429,65391	13853,90902	14333,77089	16683,48466	14093,83996	0,844777949	-0,243355917	0,7815879	1	conserved Plasmodium protein, unknown function
Pf_M76611100800	0	16,06445671	20,47663979	32,63632775	90,16820193	57,94520722	16,84161077	22,80579968	36,2151189	19,82370522	0,547387248	-0,869366269	0,7816396	1	unspecified product
PFIT_1233000.2	1636,037392	484,8545117	367,1168991	429,2932342	272,1743873	163,6099968	423,0612625	374,0151147	558,8477369	398,5381886	0,713142708	-0,487737289	0,7816993	1	conserved Plasmodium protein, unknown function
PFIT_1314200	381,4463633	784,9677712	883,4207452	781,3890009	716,3362709	504,464157	785,4927263	858,8012564	675,3373848	822,1469913	1,217387057	0,283787933	0,7819698	1	conserved Plasmodium protein, unknown function
PFIT_1114900.2	152,0716399	585,6224674	411,7267215	424,8998824	390,728875	378,3481177	460,7864707	473,709039	390,566284	467,2477548	1,196334077	0,258620319	0,781995	1	rhomboid protease ROM1
PFIT_0106200	2551,001759	189,1224677	215,7360264	188,9141279	185,3457484	122,7074976	183,2367252	195,4782829	575,4712711	189,3575041	0,32904771	-1,603631312	0,7820168	1	conserved Plasmodium membrane protein, unknown function
PFIT_0518400	679,2533247	1136,925414	1024,563298	829,0882492	1314,118054	2024,673711	1054,958499	1180,037235	1168,103675	1117,497867	0,956676955	-0,063896249	0,7821272	1	proteasome subunit beta type 1, putative
PFIT_0705400	6987,691852	4752,158376	6469,155557	7700,290484	2394,466696	6268,308004	5444,55593	4886,957073	5762,011828	5165,756502	0,896519593	-0,157592981	0,7822037	1	conserved Plasmodium protein, unknown function
PFIT_0603900	0	14,60405156	26,3271083	34,51919281	31,72584883	92,03062323	15,49428191	19,54782829	33,20113745	17,5210551	0,527724543	-0,922143013	0,7823284	1	50S ribosomal protein L24, putative
PFIT_0211500	8052,193331	4540,399629	5988,68583	5875,166617	3939,014599	6329,661753	4565,423847	5057,674774	5787,520293	4811,549311	0,831366296	-0,266443833	0,7823897	1	conserved Plasmodium protein, unknown function
PFIT_1444600	125,4591029	227,0930017	156,5000327	167,5749906	567,7257158	371,5310345	191,3206983	164,8533519	269,3139797	178,0870251	0,661261719	-0,59670671	0,7825062	1	conserved Plasmodium protein, unknown function
PFIT_1215800	11,40537299	26,2872928	19,74533123	30,75346269	73,47038676	13,6341664	22,23092622	20,85101685	29,21600214	21,54097153	0,73730045	-0,439675457	0,7826602	1	conserved Plasmodium membrane protein, unknown function
PFIT_0628200	2760,100264	556,4143643	193,0654609	378,4558776	390,728875	248,8235369	416,3246182	340,7838066	754,598063	378,5542124	0,501663377	-0,995208475	0,7826607	1	phospholipase, putative
PFIT_0535800	124817,8675	20861,15745	23061,81556	21212,35779	26728,19275	53964,03063	25517,06131	26765,53809	45107,57028	26141,2997	0,579532428	-0,787038706	0,7826693	1	early transcribed membrane protein 5
PFIT_0832800	1865,412116	640,3876607	823,453443	992,8975096	818,1929434	1295,245808	787,5137196	850,9821251	1072,598247	819,2479223	0,763797559	-0,388737786	0,782679	1	unspecified product
PFIT_1437600	467,6202926	357,0690606	407,3388701	306,2793835	183,6759669	190,8783297	265,4237857	318,6296012	318,8103172	292,0266935	0,915988843	-0,12659807	0,7829794	1	conserved Plasmodium protein, unknown function
PFIT_1306700	112,7864662	458,5672189	83,36917629	216,5294822	198,7040005	30,67687441	167,0687788	181,1432089	183,4388698	174,1059938	0,949122692	-0,0753335	0,78321	1	conserved Plasmodium protein, unknown function
PF_apicoplast_ge	0	2,190607733	0,731308564	1,882865062	0	0	2,020993292	0,651594276	0,800796893	1,336293784	1,668705006	0,738728937	0,7836363	1	apicoplast ribosomal protein S17
PFIT_1224300	363,704672	389,9281766	332,7453966	250,4210533	207,0529081	330,6285353	346,2635174	353,8156921	312,413457	350,0396048	1,120437026	0,164061565	0,7836414	1	cytochrome c heme lyase, putative
PFIT_1213300	343,4284534	1101,145487	1216,89745	1210,054613	896,6726747	1114,593104	1075,168432	1237,377531	980,4652971	1156,272981	1,179310461	0,237943567	0,7836722	1	RNA polymerase subunit 8c, putative
PFIT_0811000	1547,328936	869,6712702	1038,458161	948,9639915	945,0963387	920,3062323	853,5328338	876,3943018	1044,970822	864,9635678	0,827739445	-0,272751386	0,7839439	1	conserved Plasmodium protein, unknown function
PFIT_1201300	110,2519389	31,39871085	21,93925692	29,49821931	43,41431945	10,2256248	30,98856382	27,36695961	41,12134504	29,17776171	0,709552708	-0,495018239	0,7842568	1	NIMA related kinase 3
PFIT_0915700	680,5205884	1001,107734	1369,009632	1466,751884	679,6010775	1830,38684	1006,45466	1582,070903	1171,229626	1294,262781	1,105046144	0,144106614	0,78428	1	conserved Plasmodium protein, unknown function
PFIT_1122900	21,54348231	47,46316756	29,98365112	33,89157112	23,37694124	40,90249921	28,29390609	24,76058251	32,86021876	26,5272443	0,807275341	-0,308867271	0,7843877	1	conserved Plasmodium protein, unknown function
PFIT_1324700	136,8644759	58,41620623	87,75702767	87,23941456	156,9594626	109,0733312	70,73476523	83,40406739	106,051653	77,06941631	0,726715842	-0,460536739	0,7844219	1	conserved Plasmodium protein, unknown function
PFIT_0107200	1757,694704	955,8351744	1491,138162	1523,237836	758,0808088	2021,265169	1009,149317	1279,079565	1417,875309	1144,114441	0,806921761	-0,309499298	0,784459	1	secreted ookinete protein, putative
PFIT_1208800	636,1663601	834,6215464	709,369307	655,8646634	856,5979183	1090,733312	848,8171828	787,7774802	797,2255179	818,2973315	1,026431434	0,037637258	0,7845472	1	U6 snRNA-associated Sm-like protein LSm7, putative
PFIT_1203400	18618,63777	23046,6289	27318,76271	34855,59804	18350,89887	16613,23176	25417,35897	31407,49572	22800,62634	28412,42735	1,246124862	0,317448633	0,7845847	1	nucleosome assembly protein
PFIT_0919200	762,8927267	1939,418047	2166,867275	2055,461027	1946,965249	2161,015375	2015,603977	2148,306329	1838,76995	2081,955153	1,132254284	0,179197998	0,7846185	1	DnaJ protein, putative
PFIT_1414800	2377,386637	2346,871085	2827,238908	2816,138512	1983,700442	2273,497248	2727,66728	2929,567867	2437,472139	2828,617574	1,160471756	0,214711411	0,7848046	1	diphthamide synthesis protein, putative
PFIT_0419700	3,801790997	8,032228356	8,775702767	6,276216875	10,0186891	13,6341664	8,7576376	9,12231987	8,423132417	8,939978735	1,061360346	0,085914553	0,7849367	1	rifin, pseudogene
PFIT_1035300	3379,792196	8395,139037	1676,890537	3245,431746	7343,699113	1390,684973	4174,698478	3052,719185	4238,606267	3613,708831	0,852570068	-0,23010969	0,785008	1	probable protein, unknown function
PFIT_0918900	935,2405852	1621,049723	1830,465336	1862,781168	1270,703735	1475,898513	1826,304272	1748,879038	1499,35651	1787,591655	1,192239233	0,253673754	0,7851384	1	dihydrouridine synthase, putative
PFIT_1112100	276,2634791	255,5709022	306,4182883	242,2619714	405,7569087	654,4399874	340,874202	308,2040928	356,7852562	324,5391474	0,9096204	-0,136663485	0,7851546	1	SNARE protein, putative
PFIT_0102800	29784,49793	13605,86463	16246,75106	24777,24898	9215,524193	9097,397533	15461,94602	16549,84303	17121,21405	16005,89452	0,934857451	-0,097181699	0,7852847	1	ubiquitin carboxyl-terminal hydrolase 1, putative (UBP1)
PFIT_1008300	11461,13259	11578,09207	11635,85056	13032,56434	8206,976157	9271,233155	12669,60695	12980,40958	10864,30815	12825,00827	1,180471696	0,239363451	0,7853677	1	nucleolar protein 5, putative (NOP5)
PFIT_1140900	2492,70763	438,1215467	634,0445249	919,4657722	323,9376143	719,2022778	491,10137	815,7960341	921,246561	653,4487021	0,709309244	-0,495513345	0,7854015	1	conserved Plasmodium protein, unknown function
PFIT_0805100	6679,746781	2522,119704	1997,203688	1677,005149	1621,357853	2178,058083	2568,682475	1630,28888	2779,248543	2099,485677	0,75541487	-0,404658912	0,7854427	1	rRNA processing WD-repeat protein, putative
PFIT_0610300	556,3287492	863,099447	688,1613587	795,1966781	816,5231619	770,3304018	697,9163503	581,8736889	748,2732994	639,8950196	0,855162171	-0,225730059	0,7854681	1	SWI/SNF-related matrix-associated actin-dependent regulator of chromatin
PFIT_1470500	150,8043762	281,858195	215,0047178	178,2445592	178,6666223	439,7018665	186,6050473	175,2788604	240,7133895	180,9419538	0,751690441	-0,411789438	0,7854953	1	nucleoside transporter 3, putative (NT3)
PFIT_1342800	70,96676527	131,436464	97,99534757	90,377523	100,186891	119,298956	86,90271157	80,146096	101,7103245	83,52440379	0,821198872	-0,284196448	0,785541	1	UDP-N-acetylglucosamine pyrophosphorylase, putative
PFIT_0513600	59,56139228	103,6887661	114,084136	91,00514469	70,13082372	40,90249921	108,4599734	97,73914147	79,89546032	103,0995574	1,290430733	0,367852703	0,7857963	1	mitochondrial import inner membrane translocase subunit TIM16, putative (PAM1
PFIT_1015200.2	2,534527331	5,111418045	7,313085639	3,765730125	6,679126069	13,6341664	4,041986585	1,954782829	6,506342269	2,998384707	0,460840298	-1,117661218	0,7858818	1	tubulin binding cofactor c, putative
PFIT_0310300	1200,098691	487,775322	370,7734419	514,6497837	3084,086462	344,2627017	532,1949003	508,2435356	1000,2744	520,219218	0,520076509	-0,94320422	0,7858946	1	parasite-infected erythrocyte surface protein
PFIT_bin04400	172,3478585	320,5589317	744,4721181	715,4887237	185,3457484	841,9097755	456,0708196	683,522396	496,6871926	569,7966078	1,147194082	0,198109486	0,7859106	1	rifin
PFIT_1013800	778,0998907	446,153775	316,6566082	432,4313427	724,6851785	293,1345777	426,4295847	372,0603319	498,5268955	399,2449583	0,800849386	-0,320397151	0,7859615	1	conserved protein, unknown function
PFIT_0923500	2046,63082	804,6832408	524,3482403	461,3019403	592,7724386	1056,647896	688,4850483	606,6342714	914,3974294	647,5596598	0,708181846	-0,497808233	0,7860001	1	conserved Plasmodium protein, unknown function
PFIT_0103900.1	10,13810932	34,31952116	4,387851384	15,0629205	43,41431945	0	16,84161077	9,773914147	17,8871203	13,30776246	0,743985741	-0,426653123	0,7861717	1	conserved Plasmodium protein, unknown function
PFIT_1010400	111,5192026	337,353591	270,5841687	276,7811642	350,6541186	651,0314458	237,8035441	244,3478537	332,9872818	241,0756989	0,723978698	-0,465980845	0,7862417	1	conserved Plasmodium protein, unknown function
PFIT_1421800	0	5,841620623	6,581777075	5,648595187	13,35825214	3,408541601	9,431302031	4,561159935	5,806464437	6,996230983	1,204903786	0,268917949	0,786438	1	dynein-associated protein, putative
PFIT_1412000.2	1605,623064	2871,886739	1320,011958	2980,575394	1967,002627	559,0008226	2104,527682	1686,325987	1884,016767	1895,426835	1,006056245	0,008710963	0,7865893	1	conserved Plasmodium protein, unknown function
PFIT_1317300	12,67263666	24,82688765	25,59579974	18,82865062	138,5918659	47,71958241	25,59924837	17,59304546	44,70590383	21,59614692	0,483071475	-1,049691429	0,7869458	1	ferredoxin, putative
PFIT_0321500	155,8734309	757,2200732	148,4556385	357,7443619	1212,261381	197,6954129	340,874202	320,584384	471,5417165	330,729293	0,701378651	-0,511734577	0,7870021	1	protein kinase, putative
PFIT_0929700	0	21,90607733	0	4,393351812	23,37694124	0	4,715651015	4,561159935	8,279395065	4,638405475	0,560234829	-0,835896419	0,7871358	1	conserved protein, unknown function
PFIT_0209800	460,0167106	534,508287	477,5444923	451,2599933	465,8690433	732,8364442	467,523115	608,5890542	520,3391618	538,0560846	1,034048798	0,048304269	0,7874794	1	conserved Plasmodium protein, unknown function
PFIT_1418400	1587,881373	1124,51197	616,4911194	1075,115951	940,0869942	245,4149953	820,5232767	881,6070561	931,5840671	851,0651664	0,913567757	-0,130416361	0,7874947	1	cyclic nucleotide-binding protein, putative
PFIT_0709400	40,5524373	56,95580107	51,92290804	58,36881694	106,8660171	201,1039545	58,60880548	42,35362797	85,9616582	50,48121672	0,587252726	-0,767946589	0,7874965	1	conserved Plasmodium protein, unknown function
PFIT_1427300	53,22507395	71,55985263	43,14720527	43,30589644	113,5451432	40,90249921	80,83973169	48,21797646	60,94761178	64,52885408	1,058759354	0,082374716	0,7875517	1	dynein-associated protein, putative
PFIT_1146200	40,5524373	54,03499076	123,5911473	114,8547688	168,6479332	184,0612465	81,51339612	84,05566166	114,2904206	82,78452889	0,724334799	-0,465271406	0,7876158	1	transporter, putative
PFIT_1366700	6952,208469	9773,031302	9479,952914	8258,246164	7288,596323	9612,087315	10303,0238	8892,307091	8560,687081	9597,665448	1,12113261	0,164956933	0,7876313	1	alanine--tRNA ligase (AlaRS)
PFIT_0616600	8,870845659	3,651012889	1,462617128	2,51048675	1,669781517	10,2256248	2,694657723	5,864348488	4,73172812	4,279503106	0,904427092	-0,144923887	0,7876966	1	rifin
PFIT_0613900	463,8185016	368,0220992	285,2103399	368,4139306	864,9468259	252,2320785	303,1489939	372,0603319	433,7739626	337,6046629	0,778296283	-0,361608627	0,7879258	1	conserved Plasmodium membrane protein, unknown function
PFIT_0319300	51,95781029	156,9935542	188,6776095	160,0435303	138,5918659	98,84770643	164,3741211	166,1565405	132,5186794	165,2653308	1,247109702	0,318588377	0,7879765	1	ubiquitin conjugating enzyme, putative
PFIT_0402000	83,63940193	198,6151012	226,7056548	221,5504557	153,6198996	207,9210377	140,1222016	183,0979917	182,0085918	161,6100966	0,887925647	-0,171489221	0,7879816	1	conserved Plasmodium protein, unknown function
PFIT_1335800	63,36318328	67,17863716	68,01169645	71,54887237	270,5046058	71,57937362	58,60880548	73,63015324	102,0310614	66,11947936	0,648032848	-0,625861151	0,7879909	1	conserved Plasmodium protein, unknown function
PFIT_0626000	12959,03824	14690,94566	18377,78421	22145,63124	12117,60447	17530,12945	18755,49142	19367,33668	16303,52221	19061,41405	1,169159265	0,22547147	0,7880021	1	ankyrin-repeat protein, putative
PFIT_1477000	35,48338264	21,90607733	18,2827141	11,29719037	38,4049749	34,08541601	20,88359735	12,38029125	26,57662589	16,6319443	0,62581098	-0,676201123	0,7880074	1	conserved Plasmodium protein, unknown function
PFIT_1231200	978,3275498	1754,676795	922,1800991	1555,874163	931,7380866	964,6172731	1092,683707	1124,000127	1184,568994	1108,341917	0,935649947	-0,095959217	0,7880614	1	amino acid transporter, putative
PFIT_1026300	13,93990032	24,82688765	20,47663979	11,92481206	8,348907586	23,85979121	25,59924837	14,33507408	17,22948977	19,96716123	1,158894517	0,212749258	0,7880965	1	biotin--acetyl-CoA-carboxylase, putative
PFIT_1473300	31679,05711	9153,089313	8920,501863	9751,358159	6083,014067	5054,867194	10154,81763	7767,65537	11773,64795	8961,2365	0,761126589	-0,393791676	0,7880968	1	histone deacetylase, putative
PFIT_0810600	1979,465846	868,9410676	547,7501144	503,9802151	510,9531443	954,3916483	875,76376	893,335753	894,2470059	884,5497565	0,989155961	-0,015730085	0,7882952	1	serine protease, putative
PFIT_0208100	479,0256656	208,1077347	274,2407115	197,7008316	243,7881015	327,2199937	181,2157319	257,3797392	288,3471731	219,2977355	0,760533676	-0,394915964	0,7884982	1	conserved Plasmodium protein, unknown function
PFIT_0729900	1812,187042	1242,074585	1161,318	1198,129801	819,8627249	671,4826954	1414,695305	1294,717827	1150,842475	1354,706566	1,17714335	0,235290019	0,78859	1	unspecified product
PFIT_1218300	7270,291649	3625,455799	4105,566278	3991,046311	2644,933923	3838,017843	3289,503416	3858,089711	4245,885301	3573,796563	0,841708221	-0,248607886	0,7886246	1	arginine--tRNA ligase, putative
PFIT_1435300	192,6240772	407,4530384	337,8645565	291,8440847	230,4298494	364,7139513	272,8340945	256,7281449	304,1549263	264,7811197	0,870546872	-0,200006117	0,7886388	1	queuine tRNA-ribosyltransferase, putative
PFIT_1005100	2235,453106	1770,011049	1542,329761	1184,949746	2531,38878	3991,402215	1618,815627	1354,012906	2209,255776	1486,414267	0,672812213	-0,571724202	0,7886479	1	regulator of nonsense transcripts, putative
PFIT_0824300	11637,28224	3490,368322	4360,061658	6057,804528	3685,207808	2784,778488	4068,933162	5068,100282	5335,917174	4568,516722	0,856182091	-0,224010437	0,7887192	1	conserved Plasmodium protein, unknown function
PFIT_1323800	1127,864662	231,4742172	203,3037808	239,7514846	197,034219	115,8904144	229,7195709	216,9808941	352,5531297	223,3502325	0,633522195	-0,658532932	0,7887463	1	conserved Plasmodium protein, unknown function
PFIT_0531700	15863,60657	5899,306626	6641,013069	6588,772475	4915,836787	9271,233155	7107,159745	5700,14673	8196,628113	6403,653238	0,781254578	-0,356135356	0,7887587	1	Hsc70-interacting protein (HIP)
PFIT_1106900	286,4015884	124,8646408	140,4112443	107,3233086	197,034219	163,6099968	135,4065506	117,938564	169,940833	126,6725573	0,745392117	-0,423928533	0,7888048	1	apicoplast ribosomal protein S15 precursor, putative
PFIT_1008000	2268,401961	3110,662982	3322,334806	3298,779589	3391,326261	3234,705979	2489,863736	2825,964377	3104,368597	2657,914057	0,856185074	-0,22400541	0,7889177	1	conserved Plasmodium membrane protein, unknown function
PFIT_1452800	27,87980064	59,1464088	59,23599368	39,54016631	33,39563034	40,90249921	51,87216117	53,43073067	43,35008317	52,65144592	1,214563896	0,28043839	0,7889275	1	LCCL-like protein
PFIT_1435000	6811,542202	527,2062612	676,4604216	758,1669985	489,2459845	132,9331224	529,5002426	634,6528253	1565,925832	582,0765339	0,371713987	-1,42773512	0,7890572	1	conserved Plasmodium protein, unknown function
PFIT_0826700	4311,23099	1683,847144	1213,240908	859,8417119	2190,753351	2041,716419	1466,567466	1446,539294	2050,105087	1456,55338	0,710477423	-0,493139289	0,789062	1	DnaJ protein, putative
PFIT_bin08400	0	1,460405156	12,43224559	6,276216875	0	54,53666562	2,020993292	2,606377106	12,45092221	2,313685199	0,185824404	-2,427988113	0,7893687	1	rifin
PFIT_0513800	6,336318328	7,302025778	9,507011331	12,55243375	23,37694124	13,6341664	12,12595975	13,03188553	12,11814947	12,57892264	1,038023394	0,053838958	0,7894386	1	secreted ookinete protein, putative
PFIT_1148600	253,4527331	200,0755063	195,2593866	198,9560749	382,3799674	184,0612465	207,4886447	164,2017577	235,6974858	185,8452012	0,788490384	-0,342834933	0,7895995	1	serine/threonine protein kinase, putative
PFIT_1473700	173,6151222	530,8572741	178,4392896	425,5275041	1083,688205	231,7808289	305,8436516	337,5258352	437,3180373	321,6847434	0,735585354	-0,443035339	0,7896623	1	protein disulfide isomerase
PFIT_bin10800	140,6662669	45,27255982	36,5654282	57,74119525	26,71650428	44,31104081	54,56681889	37,79246803	58,54549921	46,17964346	0,788782128	-0,34230123	0,7896805	1	erythrocyte membrane protein 1, PfEMP1
PFIT_0609600	257,2545241	559,3351746	474,619258	538,4994079	372,3612783	218,1466625	580,0250749	458,0707764	403,3693842	519,0479256	1,286780668	0,363766167	0,789682	1	conserved Plasmodium protein, unknown function
PFIT_1325900	1003,672823	916,4042352	1446,528339	1646,879308	836,5605401	1639,50851	1268,510123	1530,594955	1248,258959	1399,552539	1,12120368	0,165048385	0,7900968	1	conserved Plasmodium protein, unknown function
PFIT_0810500	11241,89598	8703,284525	10046,71705	9477,715103	6974,677397	14414,72243	10142,69167	10922,02326	10143,16875	10532,35747	1,03836954	0,054319968	0,7901272	1	conserved Plasmodium protein, unknown function
PFIT_1474600	1257,125556	859,4484341	1159,855382	916,9552854	738,0434306	1124,818728	1054,284834	1133,774041	1009,37447	1094,029438	1,083868743	0,116190056	0,790167	1	conserved Plasmodium protein, unknown function
PFIT_0421300	106,4501479	202,9963166	207,6916322	159,4159086	203,7133451	81,80489842	229,7195709	161,5953806	160,3453915	195,6574757	1,220225127	0,287147345	0,7901723	1	clathrin assembly protein, putative
PFIT_1018600	7684,686868	1810,823204	665,4907932	804,6110034	1173,856407	323,8114521	916,1836259	809,9316856	1945,546621	863,0576558	0,443606772	-1,172646706	0,7901784	1	conserved Plasmodium protein, unknown function
Pf_M76611101700	2,534527331	13,87384898	11,70093702	23,84962412	130,2429583	68,17083202	6,736644308	25,41217678	41,72878797	16,07441054	0,385211537	-1,376277183	0,7903105	1	unspecified product
PFIT_1329200	27,87980064	73,75046036	54,11683373	63,38979044	63,45169765	109,0733312	51,87216117	46,91478791	65,27608568	49,39347454	0,756675175	-0,402253982	0,790384	1	conserved Plasmodium protein, unknown function
PFIT_0109400	1987,069428	238,0460404	319,5818424	258,5801352	262,1556982	299,9516609	315,948618	266,5020591	560,8974675	291,2253386	0,519213146	-0,945601184	0,7904274	1	adenylate kinase-like protein 1 (AKLP1)
PFIT_0624500	4083,12353	4841,973294	6120,321372	4922,436895	3631,7748	4840,129073	5490,365111	5437,554237	4739,959827	5463,959674	1,152743878	0,205072004	0,790644	1	poly(A) polymerase PAP, putative
PFIT_1249300	3810,661842	2365,12615	3941,75316	4064,478048	2045,482359	3916,4143	2444,054555	3343,981827	3357,31931	2894,018191	0,862002665	-0,214235766	0,7906474	1	conserved Plasmodium protein, unknown function
PFIT_1301800	780,634418	1969,356352	2257,549537	1959,434908	1893,532244	1404,31914	2343,678555	1766,472083	1710,804433	2055,075319	1,201233338	0,26451642	0,7908467	1	deoxyhypusine hydroxylase
PFIT_1005200	6636,659817	2527,231122	3497,848861	3070,325295	2372,759536	3745,98722	2941,218905	2916,535981	3641,801975	2928,877443	0,804238523	-0,314304651	0,7909144	1	DnaJ protein, putative
PFIT_1019600	12,67263666	35,04972374	4,387851384	11,29719037	18,36759669	0	15,49428191	7,167537041	13,62916647	11,33090947	0,831372153	-0,266433669	0,7910051	1	flagellar outer arm dynein-associated protein, putative
PFIT_0834800	69567,70619	20134,60588	15775,78834	7763,052653	13722,26451	61861,62152	25102,08402	26100,91193	31470,83985	25601,49798	0,813499039	-0,297787453	0,7911764	1	histidine-rich protein II
PFIT_1012200	2193,633405	2008,787292	2184,418681	2831,201432	2122,292308	1775,850174	1805,420675	2111,81705	2186,030549	1958,618862	0,89597049	-0,158476879	0,7911806	1	protein phosphatase, putative
PFIT_1035000	164,7442765	170,1372006	61,42991937	98,53660494	328,9469589	95,43916483	139,4485372	86,66203877	153,2056875	113,055288	0,737931403	-0,438441383	0,7912032	1	merozoite surface protein
PFIT_0626500	1405,395405	361,450276	334,9393223	315,0660871	166,9781517	265,8662449	309,8856382	340,1322123	474,9492479	325,0089252	0,684302432	-0,547294021	0,79124	1	conserved Plasmodium protein, unknown function
PFIT_1236000	73,5012926	94,19613254	71,66823927	55,85833019	126,9033953	71,57937362	58,60880548	69,0689933	82,28446059	63,83889939	0,775831778	-0,366184224	0,7914103	1	conserved Plasmodium protein, unknown function
PFIT_0623900.2	8,870845659	75,21086552	35,10281107	61,50692537	65,12147917	6,817083202	45,80918129	35,83768521	42,10500167	40,82343325	0,969562561	-0,044594104	0,791798	1	sphingomyelin synthase 1, putative (SMS1)
PFIT_0409700	5,069054662	44,54235725	27,78972543	36,40205787	63,45169765	37,49395761	28,96757052	26,06377106	35,79147508	27,51567079	0,768777222	-0,379362503	0,7919292	1	conserved Plasmodium protein, unknown function
PFIT_0715900	860,4720289	207,3775321	261,0771573	267,3668389	247,1276645	276,0918697	273,5077589	252,8185793	353,2521819	263,1631691	0,744972523	-0,42474088	0,7920865	1	conserved Plasmodium protein, unknown function
PFIT_1316800	1074,639588	641,8480659	221,5864949	139,9596363	364,0123708	623,763113	483,0173969	472,4058504	510,9682115	477,7116237	0,934914566	-0,09709356	0,7921201	1	conserved Plasmodium protein, unknown function
PFIT_1321100	759,0909357	2307,440146	1970,145271	2423,247335	1481,096206	937,3489403	2003,478017	2294,915042	1646,394806	2149,106529	1,305395596	0,384487078	0,7922242	1	conserved Plasmodium protein, unknown function
PFIT_1440400	517,0435756	572,478821	764,2174493	701,6810466	509,2833627	637,3972794	683,7693972	732,3919667	617,0169224	708,080682	1,147587135	0,198603699	0,7922673	1	conserved Plasmodium protein, unknown function
PFIT_1014100	30580,33951	14389,372	15794,07106	16847,24896	13713,9156	16902,9578	14469,63831	15219,93911	18037,98415	14844,78871	0,822973819	-0,281081559	0,7923537	1	transcriptional coactivator ADA2
PFIT_1001800	3164,357373	2295,756905	4700,851449	5003,400093	1741,582122	3483,529516	3924,095309	4259,471785	3398,246243	4091,783547	1,20408683	0,267939433	0,7923796	1	tryptophan-rich antigen 3
PFIT_1246400	5203,384611	13621,92909	3800,610607	9565,582139	17968,51891	3486,938058	8181,654512	7095,210076	8941,160568	7638,432294	0,854299868	-0,227185535	0,7925423	1	actin I
PFIT_1118700	562,6650675	542,5405153	590,1660111	550,4242199	809,8440358	893,0378995	468,8704438	534,958901	658,1129582	501,9146724	0,762657331	-0,390893108	0,7927124	1	conserved Plasmodium protein, unknown function
PFIT_1218000	6707,626582	2133,651932	2552,998197	3205,263958	2077,208207	1407,727681	2497,274045	2677,400882	3014,079426	2587,337463	0,858417148	-0,2202492	0,7928534	1	adaptor protein subunit, putative
PFIT_0522600	756,5564083	964,5976053	522,8856232	601,2615766	1788,336005	412,4335337	633,2445649	700,4638472	841,011792	666,8542061	0,792918973	-0,334754649	0,7929139	1	conserved Plasmodium protein, unknown function, putative
PFIT_0715700	584,2085498	580,5110494	315,9252996	403,5607451	2025,44498	432,8847833	459,4391418	419,626714	723,7559013	439,5329279	0,607294431	-0,719531956	0,7929831	1	conserved Plasmodium protein, unknown function
PFIT_1029800	285,1343248	136,5478821	154,306107	141,2148797	43,41431945	64,76229042	175,152752	157,6858149	137,5633006	166,4192835	1,209765125	0,274726976	0,7930384	1	conserved Plasmodium protein, unknown function
PFIT_1450300	423,2660643	693,6924489	200,3785465	306,2793835	1629,706761	289,7260361	394,093692	350,5577207	590,5082067	372,3257064	0,630517412	-0,665391883	0,7930452	1	conserved Plasmodium protein, unknown function
PFIT_0202700	11,40537299	22,63627991	21,20794835	20,71151569	160,2990257	6,817083202	20,88359735	11,0771027	40,51287097	15,98035003	0,394451187	-1,342081317	0,7930635	1	conserved Plasmodium protein, unknown function
PFIT_0802700	193,8913408	18,25506445	21,20794835	15,69054219	11,68847062	23,85979121	17,5152752	20,19942257	47,43219294	18,85734889	0,397564349	-1,330739706	0,7931503	1	Plasmodium exported protein, unknown function, pseudogene
PFIT_1213600	3,801790997	27,74769796	19,01402266	29,49821931	61,78191614	13,6341664	24,92558394	33,2313081	25,91296891	29,07844602	1,122158025	0,166275854	0,7931763	1	conserved Plasmodium protein, unknown function
PFIT_0112100	2400,197383	3751,050642	4343,97287	4286,028504	2952,173722	3006,333692	4268,337833	4043,79408	3456,626136	4156,065957	1,202347547	0,265853978	0,7934083	1	heat shock protein 40, type II
PFIT_1338800	410,5934276	314,717311	205,4977065	178,2445592	243,7881015	572,634989	234,4352219	212,4197341	320,9126825	223,427478	0,696225142	-0,522374181	0,7934147	1	conserved Plasmodium protein, unknown function
PFIT_0625500	119,1227866	173,7882135	286,6729571	165,6921255	190,355093	132,9331224	174,4790876	147,9119008	178,0940493	161,1956942	0,905114431	-0,143827895	0,7934877	1	conserved Plasmodium protein, unknown function
PFIT_1429900	70,96676527	267,2541435	207,6916322	239,1238629	233,7694124	753,2876938	157,6374768	201,3994252	295,3489183	179,8158513	0,608825156	-0,715900124	0,7936319	1	conserved protein, unknown function
PFIT_1103700	717,2712347	709,0267031	1213,240908	1262,147214	647,8752287	562,4093642	1010,496646	1135,07723	851,9951086	1072,786938	1,259146827	0,332446523	0,7937214	1	Plasmodium exported protein (hyp11), unknown function
PFIT_1115600	53,22507395	156,2633517	108,2336675	131,8005544	166,9781517	74,98791522	127,9962418	145,3055237	115,2481191	136,6508827	1,185710308	0,245751575	0,7937244	1	glycerol-3-phosphate dehydrogenase, putative
PFIT_0319400	245,8491511	152,6123388	123,5911473	150,0015833	577,744405	95,43916483	134,7328862	163,5501634	224,2062984	149,1415248	0,665197748	-0,58814481	0,793783	1	kinesin-8, putative
PFIT_1010600	433,4041736	466,5994472	441,7103726	342,0538197	397,4080011	347,6712433	368,4944436	329,0551096	404,8078429	348,7747766	0,861581075	-0,214941533	0,7938491	1	conserved Plasmodium protein, unknown function
PFIT_1230300	247,1164148	414,7550642	370,7734419	374,6901474	367,3519338	351,0797849	380,6204034	424,187874	354,2944465	402,4041387	1,135790881	0,183696218	0,7940324	1	sun-family protein, putative
PFIT_0314000.2	12,67263666	48,19337014	18,2827141	31,38108437	31,72584883	10,2256248	31,66222825	34,53449665	25,41354648	33,09836245	1,302390537	0,381162122	0,7940433	1	vesicle transport v-SNARE protein, putative
PFIT_0323200	6952,208469	1882,462246	1378,516643	979,7174542	2025,44498	4124,335337	1760,958822	1716,299324	2890,447522	1738,629073	0,601508611	-0,733342705	0,7940547	1	conserved Plasmodium protein, unknown function
PFIT_0932500	215,4348231	458,5672189	85,56310198	192,6798581	594,4422201	68,17083202	227,6985776	190,917123	269,143009	209,3078503	0,777682657	-0,36274653	0,7940637	1	conserved Plasmodium protein, unknown function
PFIT_1022700	324,4194984	790,0791892	1023,100681	882,4360926	793,1462207	1179,355394	895,9736929	916,1415527	832,0895156	906,0576228	1,088894415	0,122864069	0,7941199	1	orotidine monophosphate decarboxylase
PFIT_1347300	51,95781029	80,32228356	69,47431357	84,10130612	150,2803365	30,67687441	96,3340136	81,44928456	77,80215408	88,89164908	1,14253455	0,192237792	0,7943295	1	GTPase, putative
PFIT_0909000	443,542283	947,0727434	547,7501144	391,635933	507,6135812	651,0314458	699,9373436	588,3896316	581,4410168	644,1634876	1,107874176	0,147794041	0,7943577	1	transporter, putative
PFIT_1118400.2	10,13810932	18,25506445	13,89486271	19,45627231	16,69781517	17,04270801	11,45229532	12,38029125	15,91413866	11,91629329	0,748786569	-0,417373537	0,7945063	1	conserved Plasmodium protein, unknown function
PFIT_1250300	36347,65646	2436,686002	3827,669024	5704,453518	4647,001962	4707,195951	3393,921402	5551,583235	9611,777152	4472,752319	0,465340826	-1,10364033	0,7948864	1	osmiophilic body protein
PFIT_1351600	1610,692119	1496,915285	1549,642847	1541,438864	1432,672542	2051,942044	1354,73917	1278,42797	1613,88395	1316,58357	0,815785776	-0,293737743	0,794985	1	conserved Plasmodium protein, unknown function
PFIT_1109400	9670,489032	6003,725595	5647,896039	5028,50496	11321,11869	14220,43556	7088,297141	7948,146988	8648,694979	7518,222062	0,869289769	-0,202090929	0,7950438	1	endoplasmic reticulum-resident calcium binding protein
PFIT_1313300	391,5844727	324,9401471	363,4603563	254,1867834	350,6541186	327,2199937	404,1986585	312,1136584	335,3409786	358,1561584	1,068035764	0,094959958	0,7952428	1	conserved Plasmodium protein, unknown function
PFIT_1318000	7047,253244	7614,552482	7392,798273	8968,713914	6670,777161	4867,397406	8335,250002	8576,935461	7093,58208	8456,092732	1,192076533	0,253476862	0,7953265	1	secretory complex protein 63
PFIT_0601700	2518,052904	498,7283607	658,9090161	778,8785142	385,7195305	368,1224929	604,9506588	646,3815222	868,0684696	625,6660905	0,720756614	-0,472415925	0,7954007	1	JmjC domain containing protein
PFIT_0815300	3084,519762	3810,197051	4854,426247	4767,414338	3878,902464	5163,940526	4562,72919	4825,055617	4259,900065	4693,892403	1,101878526	0,139965186	0,7958102	1	karyopherin alpha
PFIT_1427700	4017,22582	3900,742171	5229,587541	5501,731713	5672,247814	6469,411959	3890,412088	4475,149491	5131,824503	4182,780789	0,815066998	-0,295009443	0,7958479	1	phosphoenolpyruvate carboxylase, putative
PFIT_1408500	202,7621865	381,1657456	374,4299847	361,510092	559,3768083	446,5189497	429,1242424	383,7890288	387,6272945	406,4566356	1,048575891	0,068431281	0,7959439	1	tRNA m2G10 methyltransferase subunit, putative
PFIT_0523600	34,21611897	43,81215467	71,66823927	53,34784344	70,13082372	136,341664	57,93514105	69,0689933	68,25280735	63,50206718	0,930394948	-0,104084831	0,7959787	1	conserved Plasmodium protein, unknown function, putative
PFIT_1033700	12,67263666	5,841620623	2,193925692	2,51048675	1,669781517	3,408541601	6,736644308	3,909565659	4,716165473	5,323104983	1,12869343	0,174653682	0,7959848	1	conserved Plasmodium protein, unknown function
PFIT_1450000	255,9872604	98,57734801	111,1589017	85,3565495	135,2523029	88,62208163	95,66034917	105,5582728	129,159074	100,609311	0,778956583	-0,360385176	0,7960935	1	Appr-1-p processing domain protein
PFIT_1129300	219,2366141	309,605893	329,0888538	329,5013859	420,7849423	402,2079089	270,8131012	272,3664076	335,070593	271,5897544	0,81054406	-0,303037485	0,7961534	1	geranylgeranyl pyrophosphate synthase, putative
PFIT_1326200	1027,750833	955,8351744	1039,189469	817,1634371	871,625952	968,0258147	1120,303948	942,2053238	946,5984467	1031,254636	1,089431997	0,123576145	0,796157	1	conserved Plasmodium protein, unknown function
PFIT_0313300	1731,082167	614,8305705	992,3857213	814,6529504	637,8565396	1046,422272	701,9583369	852,9369079	972,8717034	777,4476224	0,799126565	-0,323504081	0,7962649	1	conserved Plasmodium protein, unknown function
PFIT_1474500	495,5000932	877,7034985	237,6752833	641,4293646	619,4889429	184,0612465	472,9124304	492,605273	509,3097382	482,7588517	0,947868881	-0,07724059	0,79631	1	conserved Plasmodium protein, unknown function
PFIT_1219200	0	7,302025778	0,731308564	1,255243375	1,669781517	3,408541601	4,715651015	1,303188553	2,394483473	3,009419784	1,256813763	0,329770885	0,7964584	1	erythrocyte membrane protein 1 (PfEMP1), exon2, pseudogene
PFIT_bin04000	1,267263666	1,460405156	5,850468512	3,138108437	0	23,85979121	3,368322154	5,212754212	5,929339496	4,290538183	0,723611489	-0,46671278	0,7964716	1	rifin (RIF)
PFIT_1122300	50,69054662	48,92357271	40,95327958	55,85833019	116,8847062	47,71958241	44,46185243	45,61159935	60,17166962	45,03672589	0,748470604	-0,417982439	0,7964833	1	conserved Plasmodium protein, unknown function
PFIT_1430000.1	162,2097492	135,0874769	128,7103073	84,10130612	116,8847062	132,9331224	106,4389801	95,78435864	126,6544447	101,1116694	0,798327051	-0,324948197	0,7966392	1	transcription initiation TFIID-like, putative
PFIT_0614900	4185,771887	1093,113259	1182,525948	1655,03839	437,4827575	671,4826954	884,5213976	1508,44075	1537,569156	1196,481074	0,778164071	-0,361853725	0,7966946	1	conserved Plasmodium protein, unknown function
PFIT_1410500	1163,348045	148,2311233	147,7243299	170,713099	113,5451432	74,98791522	129,3435707	170,0661062	303,0916093	149,7048384	0,49392604	-1,017633064	0,7967854	1	conserved Plasmodium protein, unknown function
PFIT_0907000	152,0716399	55,49539591	41,68458814	50,209735	56,77257158	88,62208163	53,21949003	52,12754212	74,14266869	52,67351607	0,710434585	-0,493226279	0,7969021	1	conserved Plasmodium protein, unknown function
PFIT_1115900	173,6151222	81,05248614	232,5561233	258,5801352	53,43300855	398,7993673	180,5420675	227,4064025	199,6727071	203,974235	1,021542893	0,030749782	0,7969215	1	conserved Plasmodium protein, unknown function
PFIT_0928100.1	0	73,02025778	24,13318261	26,36011087	88,49842041	54,53666562	48,50383902	41,05043942	44,42477288	44,77713922	1,007931753	0,011397958	0,7969874	1	cytochrome c oxidase subunit 6B, putative (COX6B)
PFIT_0922300	9228,214013	15318,91988	14359,24365	13787,59323	24273,61392	16599,5976	16316,82618	15968,62093	15594,53038	16142,72356	1,035152913	0,049843899	0,7972266	1	S-adenosylmethionine synthetase
PFIT_1436400	183,7532315	170,1372006	54,8481423	117,9928772	505,9437997	112,4818728	123,2805908	117,938564	190,8595207	120,6095774	0,631928536	-0,662166681	0,797812	1	conserved Plasmodium protein, unknown function
PFIT_0401600	48,15601929	35,77992631	43,14720527	24,47724581	20,03737821	6,817083202	43,788188	31,92811955	29,73580968	37,85815377	1,273150258	0,348402697	0,7978278	1	probable protein, unknown function
PFIT_1405200	0	3,651012889	5,850468512	5,648595187	6,679126069	10,2256248	4,041986585	7,167537041	5,342471243	5,604761813	1,049095364	0,069145826	0,7978779	1	conserved Plasmodium protein, unknown function
PFIT_0316300.1	54,49233762	148,9613259	84,10048485	85,98417119	874,965515	88,62208163	90,94469816	79,49450173	222,8543194	85,21959994	0,382400486	-1,38684374	0,7979499	1	inorganic pyrophosphatase, putative
PFIT_0815100	30,41432797	85,43370161	100,9205818	94,14325312	275,5139503	139,7502056	68,04010751	99,6939243	121,0293368	83,8670159	0,692947827	-0,529181361	0,7979851	1	trypsin-like serine protease, putative
PFIT_0608200	1899,628235	366,5616941	431,4720527	478,2477259	207,0529081	92,03062323	362,4314638	407,8980171	579,1655398	385,1647404	0,665033939	-0,588500126	0,7979903	1	conserved Plasmodium protein, unknown function
PFIT_0105200	14617,88638	5535,665742	6118,858755	6096,717072	4127,699911	7195,43132	6596,522106	5197,115949	7282,043197	5896,819028	0,809775343	-0,30440638	0,798028	1	small ribosomal subunit assembling AARP2 protein
PFIT_1334600	195,1586045	56,95580107	92,14487906	69,66600731	60,11213462	68,17083202	92,29202702	99,6939243	90,3680431	95,99297566	1,062244709	0,087116158	0,7980967	1	reticulocyte binding protein 2 homologue a
PFIT_1031400	756,5564083	205,9171269	393,4440074	384,7320944	364,0123708	586,2691554	428,450578	439,8261366	448,4885272	434,1383573	0,968003262	-0,046916186	0,7981648	1	mitochondrial preribosomal assembly protein rimM precursor, putative
PFIT_0835100	300,3414887	15,33425413	13,89486271	21,33913737	3,339563034	3,408541601	9,431302031	18,24463974	59,60964127	13,83797089	0,232143167	-2,10691328	0,7981924	1	rifin, pseudogene
PFIT_0928700	318,0831801	280,3977899	412,4580301	385,9873378	262,1556982	507,8726986	260,0344703	334,2678638	361,1591224	297,1511071	0,822770764	-0,281437564	0,7983263	1	conserved Plasmodium protein, unknown function
PFIT_1145000	2869,084939	2769,658378	2608,577648	2661,743577	3541,606598	12696,81746	3768,478826	3240,378337	4524,581434	3504,428581	0,774531	-0,368605112	0,7983451	1	conserved Plasmodium protein, unknown function
PFIT_0609900	1,267263666	2,920810311	5,119159948	6,903838562	36,73519338	3,408541601	8,576376	8,470725594	9,392467911	8,614181597	0,917137187	-0,124790544	0,7983552	1	SNARE associated Golgi protein, putative
PFIT_1338600	10,13810932	149,6915285	76,78739921	87,86703625	252,1370091	190,8783297	90,94469816	86,01044449	127,9165687	88,47757132	0,691681869	-0,531819455	0,798383	1	conserved Plasmodium protein, unknown function
PFIT_0825300	240,7800965	248,999079	680,1169645	584,3157911	283,8628579	620,3545714	399,4830075	379,8794632	443,0715601	389,6812353	0,879499545	-0,185245264	0,7984437	1	conserved Plasmodium protein, unknown function
PFIT_0207500	4500,053276	1508,598526	2137,614932	2175,964391	1830,080543	2304,174122	1907,144004	1970,421092	2409,414298	1938,782548	0,804669645	-0,313531485	0,798593	1	RNA binding protein, putative
PFIT_1208600	1307,816103	530,8572741	680,1169645	633,8970494	439,152539	766,9218602	528,8265782	646,3815222	726,4604408	587,6040502	0,808859879	-0,306039898	0,7986412	1	conserved Plasmodium protein, unknown function
PFIT_1002100	438,4732283	530,8572741	440,9790641	377,8282559	415,7755978	477,1958241	594,8456924	380,5310575	446,8515407	487,6883749	1,091387923	0,126163985	0,7986486	1	conserved Plasmodium protein, unknown function
PFIT_1421100	73,5012926	67,90883974	55,57945086	84,72892781	75,14016827	61,35374882	78,14507397	78,84290745	69,70207135	78,49399071	1,126135697	0,17138068	0,7986908	1	conserved Plasmodium protein, unknown function
PFIT_0913300	6831,818421	5429,056166	4738,879494	3898,158301	5142,927073	7212,474028	5628,466319	5414,748437	5542,218914	5521,607378	0,996280996	-0,005375391	0,7987406	1	elongation factor 1-beta
PFIT_0201200	67,16497428	11,68324125	6,581777075	12,55243375	3,339563034	0	20,20993292	17,59304546	16,18669823	18,90148919	1,119292425	0,162587002	0,7988174	1	erythrocyte membrane protein 3
PFIT_0207700	11661,36025	3240,63904	3447,38857	2826,180459	2825,270327	4124,335337	3302,976704	3486,680973	4687,528997	3394,828839	0,724225672	-0,465488777	0,7988222	1	conserved Plasmodium protein, unknown function
PFIT_1434600	12108,70432	5302,000918	7339,412748	6627,057398	5917,705697	7089,76653	5653,391903	6493,136965	7397,441269	6073,264434	0,820995289	-0,284554151	0,7988858	1	conserved Plasmodium membrane protein, unknown function
PFIT_0823200	5167,901228	5552,460402	6311,392907	7029,990522	5633,842839	5958,130719	6661,867556	6730,317282	5942,253103	6696,092419	1,126860856	0,172309384	0,7990454	1	conserved Plasmodium protein, unknown function
PFIT_0322700	81,1048746	63,52762427	66,54907932	56,48595187	41,74453793	27,26833281	77,47140954	59,29507916	56,11340013	68,38324435	1,218661571	0,285297537	0,7990525	1	conserved Plasmodium protein, unknown function
PFIT_1405900	4818,136457	7418,127988	9137,700506	11277,10648	7058,166473	8971,281494	8936,832339	9872,956677	8113,4199	9404,894408	1,159177575	0,213101591	0,799101	1	conserved Plasmodium protein, unknown function
PFIT_0802400	12,67263666	21,17587476	24,13318261	11,92481206	3,339563034	13,6341664	15,49428191	8,470725594	14,48003925	11,98253075	0,827518734	-0,273136122	0,7992285	1	Plasmodium exported protein (PHISTa), unknown function, pseudogene
PFIT_1147800	348,497508	393,5791894	247,9136032	319,4594389	576,0746234	160,2014552	353,0001617	396,1693201	340,954303	374,5847409	1,098636203	0,135713738	0,7993931	1	tubulin-tyrosine ligase, putative
PFIT_1235500	182,4859678	67,90883974	43,14720527	45,1887615	71,80060524	27,26833281	57,93514105	52,12754212	72,96661873	55,03134158	0,754198873	-0,406983101	0,7994009	1	conserved Plasmodium protein, unknown function
PFIT_1134800	98,84656592	228,5534069	473,8879494	439,9628029	644,5356656	640,805821	449,3341753	383,7890288	421,098702	416,5616021	0,989225567	-0,015628568	0,7996214	1	protein disulfide isomerase
PFIT_1026800	1342,032222	913,4834249	1070,635738	751,2631599	786,4670946	760,104777	1133,77237	934,3861924	937,3310693	1034,081715	1,103219288	0,141719585	0,7998372	1	DNA-directed RNA polymerase II, putative
PFIT_1354000	121,6573119	303,0340698	178,4392896	134,3110411	171,9874963	160,2014552	209,509638	202,64582	178,2717773	206,077729	1,155975063	0,209110276	0,7998555	1	metacaspase 1
PFIT_1122100	112,7864662	278,9373847	105,3084332	153,1396917	900,0122378	92,03062323	161,6794434	138,1379866	273,7024728	149,908725	0,547706871	-0,868524116	0,7999778	1	protein kinase, putative
PFIT_1346500	149,5371125	73,02025778	112,6215188	64,01741212	103,5264541	102,256248	67,36644308	87,31363305	100,8298339	77,34003806	0,767035262	-0,382635192	0,8000288	1	conserved Plasmodium membrane protein, unknown function
PFIT_1473000	24223,74467	3155,935541	3614,126923	4094,603889	1978,691098	2143,972667	3205,295362	3744,060713	6535,179181	3474,678037	0,531688258	-0,911347488	0,8000847	1	conserved Plasmodium protein, unknown function
PFIT_0923200	64,63044694	76,67127067	50,46029091	45,1887615	48,423664	95,43916483	41,76719471	53,43073067	63,46893314	47,59896269	0,749956874	-0,415120459	0,800171	1	OTU-like cysteine protease, putative
PFIT_1430000.2	220,5038178	174,5184161	146,9930214	119,2481206	161,9688072	197,6954129	140,795866	125,1061011	170,1546093	132,950936	0,781353994	-0,355951783	0,8001984	1	transcription initiation TFIID-like, putative
PFIT_1457900	2349,506836	2323,504603	2418,437421	2325,338352	2088,896678	2317,808289	2414,41332	2676,097693	2303,915363	2545,255507	1,104752174	0,14372277	0,8002599	1	conserved Plasmodium protein, unknown function
PFIT_1235600	17019,35103	3839,405154	4479,264954	4793,146827	2295,949586	2869,992028	3986,746101	4498,606885	5882,851596	4242,676493	0,721193867	-0,471540966	0,8004357	1	pre-mRNA-splicing factor SYF1, putative
PFIT_1317500	928,9042669	690,7716386	632,5819078	538,4994079	903,3518008	2154,198292	604,9506588	621,6209397	974,7178856	613,2857993	0,629193132	-0,668425172	0,8005357	1	conserved Plasmodium protein, unknown function
PFIT_0813900	129,2608939	146,7707181	65,81777075	116,1100122	547,6883376	156,7929136	105,7653156	119,8933469	193,7401077	112,8293313	0,58237467	-0,779980488	0,8005777	1	cullin-like protein, putative
PFIT_0908300	11,40537299	14,60405156	16,08878841	17,57340725	40,07475641	0	19,53626849	19,54782829	16,6243961	19,54204839	1,175504257	0,233279764	0,8005785	1	conserved Plasmodium membrane protein, unknown function
PFIT_0318800	722,3402894	178,169429	135,2920843	190,1693713	100,186891	61,35374882	152,9218258	165,5049462	231,251969	159,213386	0,688484456	-0,538504013	0,8006263	1	triosephosphate isomerase, putative
PFIT_0407800	1272,33277	3053,70718	3454,701656	3990,418689	2648,273486	3043,82765	3440,404248	3456,707637	2910,543564	3448,555942	1,184849451	0,244703759	0,8007648	1	40S ribosomal processing protein, putative
PFIT_0213900	43,08696463	78,13167583	72,39954783	63,38979044	50,09344552	37,49395761	80,83973169	62,55305054	57,43256364	71,69639112	1,24835784	0,32003154	0,800865	1	conserved Plasmodium protein, unknown function
PFIT_1009400	1146,873617	741,1556165	779,5749292	873,649389	741,3829936	589,677697	954,5824984	871,8331419	812,0523738	913,2078202	1,124567638	0,169370436	0,8008762	1	conserved Plasmodium protein, unknown function
PFIT_1300600	14105,91186	4245,397787	3010,066049	2361,112788	4239,575272	14217,02702	3463,308839	4292,703093	7029,848463	3878,005966	0,551648586	-0,858178568	0,8009029	1	Plasmodium exported protein (hyp12), unknown function
PFIT_1467200	96,31203858	245,3480661	119,2032959	151,2568267	1033,594759	149,9758304	163,0267922	130,9704496	299,2818028	146,9986209	0,491171262	-1,025701941	0,8009641	1	protein phosphatase containing kelch-like domains (PPKL)
PFIT_0313500	15,20716399	13,1436464	10,96962846	16,94578556	26,71650428	27,26833281	14,14695305	9,12231987	18,37517692	11,63463646	0,633171398	-0,659332008	0,8010909	1	conserved Plasmodium protein, unknown function
PFIT_0423000	610,8210868	1604,255063	1631,549406	1740,394939	1238,977886	565,8179058	1564,922473	1638,108011	1231,969381	1601,515242	1,299963511	0,378471128	0,8011529	1	Plasmodium exported protein (PHISTb), unknown function
PFIT_0904200	8358,871138	6148,305705	7880,581085	7884,81126	5749,057764	9216,696489	8698,35513	7453,586928	7539,720574	8075,971029	1,071123386	0,099124678	0,8011711	1	GTPase activator, putative
PFIT_1006300	4401,206711	1799,219152	2265,593931	2382,451926	1223,949852	1867,880797	2063,434151	1958,040801	2323,383728	2010,737476	0,865434948	-0,208502714	0,8012904	1	conserved Plasmodium protein, unknown function
PFIT_1028500.1	204,0294502	95,65653769	108,964976	104,8128218	68,46104221	68,17083202	93,63935588	93,17798153	108,3492767	93,40866871	0,862106989	-0,214061173	0,8013145	1	conserved Plasmodium protein, unknown function
PFIT_0830800	43169,33677	13385,34345	13959,94918	12201,59323	17008,39453	22833,82019	13602,63219	16352,40996	20426,40622	14977,52107	0,733243083	-0,447636538	0,8013536	1	protein disulfide isomerase
PFIT_1008200	870,6101383	1326,778084	1273,20821	1479,931939	8313,842174	1687,228093	1283,330741	1210,662166	2491,933106	1246,996453	0,500413294	-0,998807979	0,8013659	1	tubulin beta chain
PFIT_0720800	3356,98145	2327,885818	3456,164273	3320,118727	2444,560141	2515,503702	3258,514852	3341,37545	2903,535686	3299,945151	1,136526466	0,18463128	0,8014592	1	nucleolar rRNA processing protein, putative
PFIT_0933000	36,7506463	24,09668507	35,10281107	21,96675906	80,14951283	37,49395761	29,64123495	23,45739395	39,26006199	26,54931445	0,676242296	-0,564387842	0,8015082	1	DHHC-type zinc finger protein, putative
PFIT_1442200	202,7621865	520,634438	595,2851711	584,3157911	457,5201357	633,9887378	555,099491	570,1449919	499,08441	562,6222414	1,127308788	0,172882747	0,801516	1	conserved Plasmodium protein, unknown function
PFIT_1113800	736,2801897	718,5193366	419,0398071	483,2686994	1825,071198	381,7566593	582,7197326	511,501507	760,6559817	547,1106198	0,719261575	-0,475411561	0,8017483	1	conserved Plasmodium protein, unknown function
PFIT_0312500	16,47442765	2,920810311	5,850468512	5,0209735	0	3,408541601	6,736644308	6,515942765	5,612536629	6,626293536	1,180623597	0,239549082	0,801811	1	tRNA Valine
PFIT_0313200	30,41432797	20,44567218	40,95327958	27,61535425	38,4049749	40,90249921	38,39887255	30,62493099	33,12268468	34,51190177	1,041941561	0,059274364	0,8019366	1	apicoplast conserved ycf19 protein precursor, unknown function
PFIT_1462600	974,5257588	1521,01197	505,3342177	1306,708353	1592,971567	460,1531161	926,9622568	996,939243	1060,117497	961,9507499	0,907400125	-0,140189236	0,8022047	1	conserved Plasmodium protein, unknown function
PFIT_0824500	5660,866794	2575,424492	3429,105856	3204,008715	2317,656746	3411,950143	3619,598987	3684,765633	3433,168791	3652,18231	1,063793403	0,089217995	0,8022799	1	clp1-related protein, putative
PFIT_0818400	25,34527331	64,98802943	29,98365112	39,54016631	355,6634632	23,85979121	33,68322154	36,48927948	89,89672909	35,08625051	0,390295074	-1,357362841	0,8024334	1	conserved Plasmodium protein, unknown function
PFIT_1406900	3782,782042	2189,147328	3372,795097	4336,865861	1624,697416	2764,327238	2810,528005	2742,56031	3011,769164	2776,544157	0,921898063	-0,117320859	0,802712	1	RNA binding protein, putative
PFIT_0308900	1572,674209	692,9622464	982,8787099	942,0601529	806,5044728	920,3062323	724,189583	910,9287985	986,2310039	817,5590308	0,828973159	-0,270602705	0,8027468	1	dual specificity phosphatase
PFIT_0600700	448,6113376	500,1887658	312,2687568	207,7427786	385,7195305	923,7147739	371,8627658	261,2893049	463,0409905	316,5760353	0,683689008	-0,548587865	0,8027966	1	conserved Plasmodium protein, unknown function
PFIT_1321700	150,8043762	213,9493553	75,32478209	150,0015833	824,8720695	126,1160392	141,4695305	135,5316095	256,8447009	138,50057	0,539238573	-0,891004396	0,8027968	1	DHHC-type zinc finger protein, putative
PFIT_0624700	323,1522347	211,028545	242,0631347	186,4036412	168,6479332	347,6712433	187,9523762	194,8266887	246,4944554	191,3895324	0,776445588	-0,365043268	0,8033345	1	conserved Plasmodium protein, unknown function
PFIT_0316300.2	106,4501479	190,5828728	103,8458161	112,3442821	1137,121213	160,2014552	134,7328862	109,4678384	301,7576312	122,1003623	0,40463057	-1,305322775	0,8033392	1	inorganic pyrophosphatase, putative
PFIT_0814600	316,8159164	98,57734801	76,78739921	76,56984587	233,7694124	37,49395761	101,723329	95,78435864	140,0023133	98,75384384	0,705372944	-0,503541856	0,8033646	1	conserved Plasmodium protein, unknown function
PFIT_0513200	192,6240772	420,5966848	443,9042983	483,8963211	901,6820193	456,7445745	363,7787926	417,6719312	483,2413292	390,7253619	0,808551211	-0,306588943	0,8034608	1	conserved Plasmodium protein, unknown function
PFIT_0309700	1269,798193	972,6298337	2027,918648	2475,339935	724,6851785	1475,898513	1372,254446	2277,321996	1491,04505	1824,788221	1,223831715	0,291405191	0,8035173	1	YTH domain-containing protein, putative
PFIT_0504000	1285,005357	849,9558006	851,2431684	736,8278611	1334,155432	2011,039545	809,0709814	821,6603826	1178,037861	815,365682	0,692138775	-0,530866765	0,8037121	1	cation transporting P-ATPase
PFIT_0800940	13,93990032	11,68324125	10,96962846	5,0209735	1,669781517	20,45124961	7,410308739	5,212754212	10,62246244	6,311531475	0,594168396	-0,751056224	0,8037682	1	surface-associated interspersed protein 1.3 (SURFIN 1.3)
PFIT_0105900	45,62149196	50,38397787	24,13318261	23,22200244	131,9127399	17,04270801	37,0514569	26,71536533	48,71935046	31,88345451	0,654431026	-0,611686948	0,8037765	1	conserved Plasmodium protein, unknown function
PFIT_1433800	1761,496495	642,5782685	777,3810035	564,8595187	716,3362709	732,8364442	649,4125113	720,6632698	865,9146668	685,0378905	0,791114779	-0,338041071	0,8037963	1	conserved Plasmodium protein, unknown function
PFIT_1034500	482,8274566	464,4088395	152,8434899	279,2916509	1634,716105	337,4456185	334,1375577	302,3397443	558,5888601	318,28651	0,569718936	-0,811677737	0,8038236	1	probable protein, unknown function
PFIT_1132700	3213,780656	387,0073662	316,6566082	411,719827	202,0435636	156,7929136	356,3684839	338,1774295	781,3334891	347,2729567	0,444461887	-1,169868384	0,8038272	1	conserved Plasmodium protein, unknown function
PFIT_0927600	2,534527331	1,460405156	1,462617128	0,627621687	6,679126069	0	0,673664431	0	2,127382895	0,336832215	0,158331731	-2,658977686	0,8038842	1	dynein light chain, putative
PFIT_0625100	271,1944244	322,7495394	378,8178361	337,6604679	228,7600679	245,4149953	353,0001617	354,4672864	297,4328885	353,7337241	1,189289207	0,250099587	0,8039137	1	conserved Plasmodium protein, unknown function
PFIT_0324000	16,47442765	6,5718232	2,925234256	4,393351812	6,679126069	3,408541601	11,45229532	3,257971382	6,742084098	7,355133353	1,090928746	0,125556875	0,8040067	1	erythrocyte membrane protein 1 (PfEMP1), exon2, pseudogene
PFIT_1417300	13,93990032	91,27532223	81,90655916	57,74119525	173,6572778	126,1160392	92,29202702	80,79769028	90,77271566	86,54485865	0,953423702	-0,068810603	0,804043	1	conserved protein, unknown function
PFIT_0805900	6489,657231	3190,985265	3616,320849	3515,309072	2733,432344	4693,561785	3666,081832	2859,195685	4039,877758	3262,638759	0,807608288	-0,308272379	0,8042062	1	conserved Plasmodium protein, unknown function
PFIT_1106500	2793,049119	4194,283607	5145,487056	4449,210143	4037,531709	5668,404683	4857,120546	4627,622551	4381,327719	4742,371549	1,082405118	0,114240567	0,8042567	1	conserved protein, unknown function
PFIT_0903700	7557,960502	4368,802023	6004,774619	7125,389018	3416,372984	4676,519077	5579,96248	7176,659361	5524,969704	6378,310921	1,154451746	0,207207872	0,804271	1	conserved Plasmodium protein, unknown function
PFIT_1312200	32,94885531	64,98802943	24,86449117	26,98773256	193,694656	88,62208163	45,13551686	33,88290238	72,01764102	39,50920962	0,548604607	-0,866161358	0,8044768	1	ubiquitin-like protein nedd8 homologue, putative
PFIT_1360500.2	5,069054662	16,06445671	2,193925692	3,138108437	6,679126069	6,817083202	5,389315446	2,606377106	6,660292462	3,997846276	0,60025086	-0,73636253	0,8047302	1	conserved Plasmodium protein, unknown function
PFIT_0400300.1	2293,747235	1928,465008	2351,888342	2025,335186	1384,248878	1874,697881	2291,806394	2187,401986	1976,397088	2239,60419	1,133175212	0,180370949	0,8047875	1	rifin
PFIT_0305500	201,4949228	308,1454878	210,6168664	212,1361304	1056,9717	190,8783297	233,0878931	218,9356769	363,3739063	226,011785	0,621981329	-0,685056823	0,8048314	1	AP endonuclease (DNA-[apurinic or apyrimidinic site] lyase), putative
PFIT_0927500	26,61253698	40,89134436	28,52103399	25,1048675	36,73519338	17,04270801	24,25191951	24,10898823	29,1512807	24,18045387	0,8294817	-0,269717942	0,8048531	1	conserved Plasmodium protein, unknown function
PFIT_1006600	181,2187042	373,8637198	174,7827468	106,0680652	293,881547	439,7018665	184,584054	192,8719058	261,5861083	188,7279799	0,721475545	-0,470977601	0,8049284	1	conserved membrane protein, unknown function
PFIT_0700600	6,336318328	1,460405156	0,731308564	0,627621687	3,339563034	6,817083202	1,347328862	0	3,218716662	0,673664431	0,209295972	-2,256383552	0,8049575	1	rifin (RIF)
PFIT_0503700	1210,236801	2950,748617	928,7618762	2169,060552	1691,488677	616,9460298	1765,674473	1421,778711	1594,540425	1593,726592	0,999489613	-0,000736521	0,8049621	1	myosin B
PFIT_0506400	1,267263666	25,55709022	18,2827141	85,98417119	30,05606731	6,817083202	22,90459065	31,27652527	27,99406495	27,09055796	0,967725052	-0,047330885	0,8049915	1	conserved Plasmodium protein, unknown function
PFIT_1466000	3022,423982	4132,94659	4755,699591	4710,928386	3568,323102	4754,915533	4787,059445	4533,792976	4157,539508	4660,42621	1,120957769	0,164731927	0,8050761	1	ATP-dependent protease la, putative
PFIT_1471300	239,5128328	404,5322281	442,4416812	433,6865861	372,3612783	235,1893705	433,8398934	426,1426568	354,6206628	429,9912751	1,212538693	0,278030786	0,805302	1	conserved Plasmodium protein, unknown function
PFIT_1202100	19,00895498	34,31952116	34,37150251	34,51919281	71,80060524	37,49395761	32,33589268	25,41217678	38,58562238	28,87403473	0,748310716	-0,418290659	0,8055605	1	peptidyl-prolyl cis-trans isomerase
PFIT_1434800	62,09591961	38,70073662	27,78972543	29,49821931	83,48907586	68,17083202	35,0305504	32,57971382	51,62408481	33,80513211	0,65483257	-0,610802015	0,8057127	1	microsomal signal peptidase protein, putative
PFIT_1359700	4321,3691	4346,895946	4665,748638	5545,665231	4543,475508	4151,60367	4901,582398	5481,862648	4595,793015	5191,722523	1,129668483	0,175899456	0,8060086	1	SUMO ligase, putative
PFIT_0529400	2566,208923	1159,561694	828,5726029	946,4535047	1248,996575	620,3545714	1021,948942	1015,835477	1228,357978	1018,892209	0,82947498	-0,26972963	0,8062681	1	conserved Plasmodium protein, unknown function
PFIT_1208300	148,2698489	86,89410676	103,8458161	62,76216875	161,9688072	143,1587472	106,4389801	114,0289984	117,8165825	110,2339892	0,935640696	-0,095973481	0,8062748	1	conserved Plasmodium protein, unknown function
PFIT_0823000	70,96676527	62,06721911	69,47431357	51,46497837	116,8847062	218,1466625	73,42942296	85,35885022	98,16744083	79,39413659	0,808762416	-0,30621214	0,8063567	1	conserved Plasmodium protein, unknown function
PFIT_0521200	3449,491698	1483,041436	2122,257453	1845,207761	1105,395364	3749,395761	1534,607573	1921,551521	2292,464912	1728,079547	0,753808505	-0,407730023	0,806555	1	conserved Plasmodium protein, unknown function
PFIT_1011300	64984,01351	21789,24492	25519,74365	27047,98424	15273,49154	36774,75533	26256,07119	24328,5755	31898,20553	25292,32335	0,792907404	-0,334775697	0,8065755	1	QF122 antigen
PFIT_1476900	81,1048746	57,68600365	29,25234256	47,07162656	60,11213462	40,90249921	39,07253699	43,65681652	52,68824687	41,36467675	0,785083566	-0,349081868	0,8067451	1	conserved Plasmodium protein, unknown function
PFIT_0516400	3925,982836	2552,788212	3002,752964	2391,866251	1658,093047	5572,965518	2698,69971	3393,502992	3184,074804	3046,101351	0,956667647	-0,063910286	0,8067524	1	tRNA pseudouridine synthase, putative
PFIT_1467600	0	5,111418045	4,387851384	5,648595187	5,009344552	51,12812402	3,368322154	1,303188553	11,88088886	2,335755353	0,196597694	-2,346681695	0,8068353	1	conserved Plasmodium protein, unknown function
PFIT_1413900.1	1077,174116	1217,9779	1394,605431	1398,34112	1021,906289	1840,612465	1394,485372	1425,036683	1325,102887	1409,761027	1,063887975	0,089346247	0,8069177	1	non-canonical ubiquitin conjugating enzyme, putative
PFIT_0314900	100,1138296	167,9465929	47,53505666	66,52789887	257,1463536	23,85979121	93,63935588	74,93334179	110,5215871	84,28634884	0,762623402	-0,390957291	0,8069986	1	conserved Plasmodium membrane protein, unknown function
PFIT_0937300	3085,787026	1936,497236	2664,157098	2330,986947	1594,641349	1499,758304	2288,438071	2762,108138	2185,30466	2525,273105	1,155570274	0,208604997	0,8070759	1	lysophospholipase, putative
PFIT_0702900	2921,042749	2430,844382	2632,71083	2181,612986	1973,681753	2000,81392	2281,701427	1872,681951	2356,784437	2077,191689	0,881366856	-0,18218545	0,8070805	1	conserved Plasmodium membrane protein, unknown function
PFIT_0705500	76,03581993	29,93830569	55,57945086	50,209735	46,75388248	34,08541601	39,07253699	41,05043942	48,76710166	40,0614882	0,821485937	-0,283692217	0,8071643	1	conserved Plasmodium protein, unknown function
PFIT_0522500	1554,932518	731,662983	621,6122794	680,9695309	499,2646736	726,019161	627,1815851	696,5542815	802,4102243	661,8679333	0,824849825	-0,277796613	0,8072388	1	S-adenosylmethionine-dependent methyltransferase,, putative
PFIT_1438500	72,23402894	238,0460404	96,53273044	144,3529881	649,5450102	122,7074976	127,9962418	145,9571179	220,569716	136,9766799	0,621013086	-0,687304427	0,8072568	1	conserved Plasmodium protein, unknown function
PFIT_0511000	36,7506463	313,2569059	142,60517	178,2445592	2738,441688	8350,926923	299,7806717	264,5472762	1960,037649	282,163974	0,143958446	-2,796275661	0,8073016	1	small nucleolar RNA snoR04
PFIT_1432500	6911,656032	2914,238488	3225,802076	3177,648604	2619,8872	2989,290984	3382,469107	2704,767842	3639,753897	3043,618474	0,836215459	-0,258053381	0,8073251	1	large ribosomal subunit associated GTPase, putative
PFIT_0809500	3171,960955	1269,822283	1717,112508	1664,452715	1036,934322	1056,647896	1822,262285	1877,24311	1652,82178	1849,752698	1,119148308	0,162401233	0,8073861	1	kinesin-like protein, putative
PFIT_1124700	456,2149196	341,7348064	314,4626825	343,3090631	813,1835989	334,0370769	344,9161886	324,4939497	433,8236912	334,7050691	0,771523261	-0,37421844	0,8074459	1	conserved Plasmodium protein, unknown function
PFIT_1234500	5895,310572	5756,917124	7185,837949	7166,812049	4685,406937	7747,615059	6516,356039	7561,751578	6406,316615	7039,053809	1,098767706	0,135886413	0,8075383	1	Splicing factor 3B subunit 3, putative
PFIT_0903500	774,2980997	802,492633	587,2407768	696,0324514	3468,136211	456,7445745	644,0231958	691,3415273	1130,824124	667,6823616	0,590438731	-0,760140733	0,8075645	1	conserved Plasmodium membrane protein, unknown function
PFIT_0622000	6849,560112	6297,997234	9289,812688	9468,300777	5258,141998	14397,67972	8095,425465	9556,281659	8593,582089	8825,853562	1,027028481	0,038476191	0,8075699	1	coronin binding protein, putative
PFIT_0605300	235,7110418	13,87384898	21,93925692	21,33913737	23,37694124	13,6341664	25,59924837	16,28985691	54,97906545	20,94455264	0,38095505	-1,392307314	0,8075709	1	conserved Plasmodium protein, unknown function
PFIT_0212500	1913,568135	1795,568139	1933,579843	1818,84765	1729,893652	3517,614932	1589,174392	1663,520188	2118,178725	1626,34729	0,767804563	-0,381188961	0,8077454	1	heat shock protein 40, putative
PFIT_0920400	0	2,920810311	1,462617128	5,648595187	8,348907586	6,817083202	2,020993292	1,303188553	4,199668902	1,662090923	0,395767133	-1,337276287	0,8077479	1	conserved Plasmodium protein, unknown function
PFIT_0820600	1010,009141	2730,227438	775,1870778	1677,005149	6368,546707	1687,228093	1577,048432	1548,188001	2374,700601	1562,618217	0,658027465	-0,603780294	0,8078807	1	rhoptry neck protein 5
PFIT_0912700	164,7442765	97,84714543	90,68226193	111,7166604	81,81929434	95,43916483	136,7538794	98,39073575	107,0414672	117,5723076	1,098380942	0,135378498	0,8080813	1	conserved Plasmodium protein, unknown function
PFIT_0612100	95,04477492	192,7734805	166,7383526	157,5330436	212,0622527	71,57937362	219,6146044	137,4863923	149,2885463	178,5504984	1,196009358	0,258228677	0,8081393	1	conserved Plasmodium protein, unknown function
PFIT_1138300	73,5012926	72,2900552	46,80374809	57,11357356	63,45169765	51,12812402	79,49240283	54,73391922	60,71474852	67,11316103	1,105384814	0,144548698	0,808163	1	conserved Plasmodium protein, unknown function
PFIT_0906100	6336,318328	4498,047879	5723,220821	5018,463013	4182,802701	3991,402215	5676,970158	5476,649894	4958,375826	5576,810026	1,124725156	0,1695725	0,8082008	1	beta subunit of coatomer complex, putative
PFIT_0813600	29,14706431	35,77992631	60,69861081	50,209735	35,06541186	126,1160392	59,28246991	46,26319363	56,16946459	52,77283177	0,939528838	-0,089990651	0,8084507	1	conserved Plasmodium protein, unknown function
PFIT_1346700	43740,87268	25351,9033	33057,34102	32900,55648	24128,34292	38615,3678	32147,9403	37167,58912	32965,7307	34657,76471	1,051327059	0,07221155	0,8085929	1	DNA/RNA-binding protein Alba 4 (ALBA4)
PFIT_0618600	202,7621865	194,2338857	114,8354445	107,3233086	340,6354295	122,7074976	148,8798392	127,7124782	180,4129587	138,2961587	0,766553354	-0,383541884	0,809211	1	conserved Plasmodium protein, unknown function
PFIT_0210800	21,54348231	27,01749538	23,40187405	22,59438075	61,78191614	23,85979121	29,64123495	13,68347981	30,03315664	21,66235738	0,721281404	-0,471365866	0,8093389	1	beta-ketoacyl-ACP synthase III
PFIT_0712700	13549,58311	1517,360957	2455,734158	2895,846466	1733,233215	2075,801835	2229,829266	2598,557975	4037,926624	2414,19362	0,597879517	-0,742073309	0,8094414	1	conserved Plasmodium protein, unknown function
PFIT_bin05900	35,48338264	7,302025778	8,775702767	7,53146025	0	3,408541601	6,062979877	7,167537041	10,41685217	6,615258459	0,635053503	-0,655049952	0,8097445	1	rifin (RIF)
PFIT_0308400	979,5948135	952,9143641	751,7852037	665,9066104	632,847195	852,1354003	677,7064174	688,0835559	805,8639312	682,8949867	0,847407311	-0,23887252	0,8097707	1	activator of Hsp90 ATPase, putative
PFIT_1123000	8,870845659	35,77992631	35,10281107	28,87059762	131,9127399	47,71958241	30,98856382	28,01855389	48,04275049	29,50355885	0,614110528	-0,70342976	0,8100017	1	conserved Plasmodium protein, unknown function
PFIT_bin01700	0	2,920810311	3,65654282	6,276216875	3,339563034	3,408541601	2,020993292	6,515942765	3,266945774	4,268468028	1,306562252	0,385775865	0,810015	1	rifin (RIF)
PFIT_1110900	2793,049119	2564,471453	2011,098551	1601,062925	3312,84653	3190,394939	2508,052676	2448,691291	2578,820586	2478,371983	0,961048627	-0,057318665	0,8101916	1	conserved Plasmodium protein, unknown function
PFIT_1107600	63095,79065	3753,971453	4219,650414	5028,50496	1873,494862	2297,357039	3739,511255	4497,303696	13378,12823	4118,407476	0,307846315	-1,699717796	0,8102326	1	protein kinase, putative
PFIT_0302800	8646,53999	5586,04972	7540,522603	7601,753879	6051,288218	6592,119456	6975,795181	8372,986453	7003,045465	7674,390817	1,095864743	0,132069745	0,8102359	1	exportin 1, putative
PFIT_0924900	10,13810932	6,5718232	2,193925692	1,255243375	5,009344552	3,408541601	5,389315446	4,561159935	4,762831291	4,975237691	1,044596667	0,062946005	0,8104177	1	telomeric repeat binding factor 1, putative
PFIT_1447800	201,4949228	600,226519	208,4229407	475,7372391	1607,999601	361,3054097	416,3246182	341,4354009	575,8644387	378,8800095	0,657932638	-0,603988212	0,8105233	1	centrin-2
PFIT_0825000	264,8581061	368,0220992	419,0398071	389,7530679	375,7008414	456,7445745	429,7979068	390,9565659	379,0197494	410,3772364	1,082733121	0,114677683	0,810586	1	RNA polymerase II mediator complex protein MED7, putative
PFIT_0216500	102,6483569	187,6620625	204,0350893	172,5959641	724,6851785	201,1039545	174,4790876	171,3692947	265,455101	172,9241911	0,651425384	-0,618328156	0,8105972	1	ATP synthase F1, alpha subunit
PFIT_1217700	510,7072572	1075,588397	457,0678525	1444,785125	849,9187923	221,5552041	849,4908472	732,3919667	759,9371046	790,941407	1,040798511	0,057690803	0,8106441	1	thrombospondin-related apical membrane protein (TRAMP)
PFIT_0720200	343,4284534	385,5469611	457,0678525	491,4277813	397,4080011	555,592281	363,1051282	377,9246803	438,4118884	370,5149043	0,845129692	-0,242755343	0,8108366	1	conserved Plasmodium protein, unknown function
PFIT_0916700	93,77751125	222,7117862	242,0631347	208,3704002	166,9781517	64,76229042	252,6241615	180,4916146	166,4438791	216,5578881	1,301086524	0,379716906	0,8108855	1	methyltransferase, putative
PFIT_0204000	2121,399376	6422,861875	5527,230126	4262,17888	4122,690566	4311,805125	5751,74691	4692,130385	4461,360991	5221,938647	1,170481084	0,227101619	0,8108968	1	hexose transporter
PFIT_0618700	5522,735055	1929,925413	1317,818032	1329,302734	1272,373516	981,6599811	1454,441506	1643,97236	2058,969122	1549,206933	0,75241873	-0,410392332	0,8111287	1	conserved Plasmodium membrane protein, unknown function
PFIT_0904500	245,8491511	165,0257826	209,8855579	180,1274243	116,8847062	54,53666562	205,4676514	193,5235001	162,0515479	199,4955757	1,231062451	0,29990395	0,8111291	1	conserved protein, unknown function
PFIT_0320200	168,5460675	107,3397789	137,48601	139,9596363	153,6198996	129,5245808	142,8168593	154,4278435	139,4126622	148,6223514	1,066060637	0,0922895	0,8111724	1	CPW-WPC family protein
PFIT_0606400	278,7980064	373,8637198	285,9416485	250,4210533	277,1837219	207,9210377	246,5611817	253,4701735	279,0215329	250,0156776	0,896044384	-0,158357899	0,811224	1	para-hydroxybenzoate--polyprenyltransferase, putative
PFIT_0216800	54,49233762	176,7090238	143,3364785	131,1729327	372,3612783	248,8235369	175,8264164	174,6272661	187,8159313	175,2268413	0,932971128	-0,100095659	0,8113818	1	conserved Plasmodium protein, unknown function
PFIT_0619100	25,34527331	24,82688765	13,16355415	16,31816387	28,38628579	17,04270801	27,62024166	16,94145119	20,84714546	22,28084643	1,068772052	0,095954187	0,8115946	1	conserved Plasmodium protein, unknown function
PFIT_0834950	3,801790997	13,1436464	5,119159948	10,041947	1,669781517	0	7,410308739	10,42550842	5,629387644	8,917908581	1,584170277	0,663727414	0,8117348	1	Plasmodium exported protein, unknown function, pseudogene
PFIT_1117900	140,6662669	101,4981583	76,78739921	85,98417119	497,5948921	115,8904144	76,79774511	115,3321869	169,7368837	96,06496602	0,565960002	-0,821217801	0,8117503	1	deubiquinating/deneddylating enzyme
PFIT_1144900	503,1036752	95,65653769	68,01169645	87,23941456	53,43300855	74,98791522	83,53438942	89,26841588	147,0720413	86,40140265	0,587476735	-0,767396375	0,8117506	1	conserved Plasmodium protein, unknown function
PFIT_0834400	5677,341222	5687,547879	7940,548387	8445,905049	5595,437864	6643,24758	7315,995718	7914,264082	6665,004663	7615,1299	1,142554324	0,192262762	0,8118244	1	Plasmodium exported protein (PHIST), unknown function
PFIT_1334800	484,0947203	349,0368322	288,8668828	298,1203016	290,541984	119,298956	333,4638932	224,8000254	304,9932795	279,1319593	0,915206918	-0,127830137	0,8119239	1	conserved Plasmodium protein, unknown function
PFIT_1238700	542,3888489	1204,834253	974,1030072	1048,128218	1060,311263	981,6599811	880,479411	845,1177766	968,5709287	862,7985938	0,890795468	-0,166833876	0,8119279	1	HD superfamily phosphohydrolase protein
PFIT_0832600	579,1394952	271,6353589	363,4603563	206,4875352	68,46104221	47,71958241	256,6661481	215,0261112	256,1505617	235,8461297	0,920732432	-0,119146129	0,8121397	1	unspecified product
PFIT_1243400	2,534527331	8,032228356	8,044394203	6,276216875	5,009344552	0	7,410308739	7,167537041	4,98278522	7,28892289	1,462821006	0,548753249	0,8122338	1	conserved Plasmodium protein, unknown function
PFIT_bin06300	68,43223794	83,97329645	253,0327631	155,0225568	202,0435636	139,7502056	208,8359735	129,0156667	150,3757706	168,9258201	1,123357968	0,167817729	0,8122687	1	unspecified product
PFIT_1423600	10,13810932	151,1519336	114,084136	110,461417	278,8535134	132,9331224	103,0706579	105,5582728	132,9370386	104,3144653	0,784690756	-0,34980389	0,8123699	1	conserved Plasmodium protein, unknown function
PFIT_0302000	3141,546627	2408,208102	3555,622238	3507,14999	2352,722158	4846,946157	2369,951468	3041,642083	3302,032545	2705,796775	0,819433709	-0,287300853	0,8124235	1	serine/threonine protein kinase
PFIT_0406300	12604,20442	2081,077347	1561,341784	2145,83855	1731,563433	633,9887378	1804,073346	2081,192119	3459,669378	1942,632732	0,561508202	-0,832620998	0,8126023	1	conserved Plasmodium protein, unknown function
PFIT_1357100	367,506463	222,7117862	264,0023916	270,5049473	629,507632	184,0612465	303,1489939	347,2997494	323,0490778	325,2243716	1,006733633	0,009682017	0,8126493	1	patatin-like phospholipase, putative
PFIT_0931000	71109,96607	4339,59392	4959,003372	5702,570653	2803,563167	2201,917874	4221,854988	5438,857426	15186,10251	4830,356207	0,318077413	-1,652550164	0,8128826	1	conserved Plasmodium membrane protein, unknown function
PFIT_1363700	38,01790997	17,52486187	13,89486271	13,18005544	16,69781517	0	16,84161077	22,1542054	16,55258419	19,49790808	1,177937406	0,236262878	0,8129636	1	conserved Plasmodium protein, unknown function, pseudogene
PFIT_0827800	29133,12441	3669,267954	4093,134032	4490,633174	1618,01829	1769,033091	3629,030289	3998,834075	7462,201825	3813,932182	0,511100111	-0,96832219	0,8130295	1	conserved Plasmodium protein, unknown function
PFIT_0826000	2177,158977	1367,669428	1755,871862	1309,21884	1267,364172	2358,710788	1680,11909	1719,557296	1705,999011	1699,838193	0,996388733	-0,005219387	0,8131102	1	RWD domain-containing protein, putative
PFIT_1312900	34,21611897	115,3720073	164,5444269	111,0890387	188,6853114	194,2868713	97,68134246	112,7258098	134,6989624	105,2035761	0,781027368	-0,356554991	0,813501	1	septum formation protein MAF homologue, putative
PFIT_0725000	2570,010714	2124,889501	3255,054418	3124,928382	2294,279805	1602,014552	2164,483816	2539,262895	2495,196229	2351,873356	0,94256048	-0,085342902	0,8134451	1	Plasmodium exported protein, unknown function
PFIT_1003000	553,7942219	927,3572738	346,6402593	542,8927597	833,2209771	606,720405	577,3304172	483,4829531	635,1043161	530,4066852	0,835148922	-0,259894615	0,8135521	1	conserved Plasmodium protein, unknown function
PFIT_1416500	149,5371125	196,4244934	266,9276258	230,3371593	200,3737821	47,71958241	154,2691546	307,5524985	181,8866259	230,9108266	1,26953164	0,344296351	0,8139106	1	conserved Plasmodium membrane protein, unknown function
PFIT_0322300	1453,551424	255,5709022	206,229015	209,6256436	197,034219	170,4270801	214,8989534	258,6829278	415,4063807	236,7909406	0,570022396	-0,810909491	0,813917	1	conserved Plasmodium protein, unknown function
PFIT_1111400	3,801790997	57,68600365	32,17757681	52,72022175	155,2896811	10,2256248	40,41986585	31,27652527	51,98348319	35,84819556	0,689607417	-0,536152804	0,8140651	1	mitochondrial ribosomal protein L11 precursor, putative
PFIT_1323600	784,436209	1619,589318	1825,346176	1968,849234	1254,005919	1107,77602	1675,403439	1829,025134	1426,667146	1752,214287	1,228817172	0,296530439	0,8140704	1	MSF1-like protein, putative
PFIT_0201900.1	136,8644759	58,41620623	60,69861081	44,56113981	53,43300855	126,1160392	64,67178536	46,91478791	80,01491342	55,79328663	0,697286095	-0,52017738	0,8141685	1	conserved Plasmodium protein, unknown function
PFIT_0403600	391,5844727	889,3867398	535,3178688	812,1424636	1516,161618	511,2812402	874,4164312	788,4290745	775,9790671	831,4227528	1,071449976	0,099564495	0,8142574	1	6-cysteine protein
PFIT_1220400	50,69054662	3,651012889	0	0,627621687	1,669781517	3,408541601	3,368322154	0,651594276	10,00791739	2,009958215	0,200836811	-2,315904371	0,8142874	1	conserved Plasmodium protein, unknown function
PFIT_1333000	96,31203858	445,4235725	568,2267542	632,642661	869,9561705	1216,849352	542,9735312	639,2139852	638,2350914	591,0937582	0,92613798	-0,110700947	0,8144152	1	histone H3 variant, putative
PFIT_0924500	4244,066016	5872,289131	4763,743986	7256,561951	3670,179775	3367,639102	4580,918129	4967,103169	4862,413327	4774,010649	0,981819177	-0,026470749	0,8146472	1	conserved Plasmodium protein, unknown function
PFIT_0617400	83,63940193	151,1519336	57,77337655	75,94222419	364,0123708	132,9331224	105,0916512	82,75247311	144,2420716	93,92206216	0,651141939	-0,618956032	0,8148194	1	malate dehydrogenase
PFIT_0830700	14733,20738	5034,746774	4131,893386	3212,167797	4082,61581	7345,40715	4908,992707	4307,038167	6423,339715	4608,015437	0,717386226	-0,479178049	0,8148832	1	SET domain protein, putative
PFIT_1102600	171,0805949	465,1390421	123,5911473	326,3632775	364,0123708	160,2014552	261,3817991	235,8771281	268,3979813	248,6294636	0,926346251	-0,110376549	0,814893	1	Plasmodium repeat_MYXSPDY protein, putative
PFIT_1005500	38,01790997	58,41620623	54,11683373	53,97546512	263,8254797	57,94520722	53,89315446	50,17275929	87,71618366	52,03295688	0,593196771	-0,753417351	0,814905	1	conserved Plasmodium protein, unknown function
PFIT_0537300	0	11,68324125	16,82009697	7,53146025	5,009344552	10,2256248	14,14695305	7,819131318	8,544961303	10,98304218	1,285323806	0,362131857	0,8149482	1	rifin (RIF)
PFIT_0526700	0	16,06445671	5,850468512	15,69054219	60,11213462	20,45124961	8,7576376	12,38029125	19,69480861	10,56896443	0,536637072	-0,897981372	0,8149819	1	50S ribosomal protein L17, apicoplast, putative (RPL17)
PFIT_1244800	1186,158791	1236,232964	999,6988069	918,8381505	1137,121213	749,8791522	983,5500689	847,0725594	1037,98818	915,3113142	0,881812849	-0,181455596	0,8151669	1	ADP-ribosylation factor GTPase-activating protein, putative
PFIT_1470000	1335,695904	1071,937384	1484,556385	1242,690941	1347,513684	2542,772034	1401,89568	1461,525962	1504,194389	1431,710821	0,951812367	-0,071250895	0,8151692	1	conserved Plasmodium protein, unknown function
PFIT_0905500	676,4515338	615,5607731	499,4837492	526,5745958	355,6634632	378,3481177	569,246644	603,3763	508,5137055	586,311372	1,152990304	0,205380381	0,8152153	1	dynein heavy chain, putative
PFIT_0701200	12352,01895	7387,45948	10285,85495	11380,03644	5288,198065	6155,826131	8814,225412	11948,93584	8808,232336	10381,58063	1,178622479	0,237101686	0,8152191	1	sporozoite threonine and asparagine-rich protein (STARP)
PFIT_0914500	211,6330322	396,4999998	297,6425855	392,2635547	1644,734794	398,7993673	296,4123495	376,6214918	556,928889	336,5169207	0,604236784	-0,726814082	0,8153226	1	conserved Plasmodium protein, unknown function
PFIT_0608800	1080,975907	1481,58103	1357,308695	1159,217257	1534,529214	1114,593104	1275,246767	978,6946032	1288,034201	1126,970685	0,874954007	-0,192720913	0,8153408	1	conserved Plasmodium protein, unknown function
PFIT_1228900	11288,78473	1070,476979	1203,002588	1166,121095	305,5700176	409,0249921	1040,137881	1015,835477	2573,830067	1027,986679	0,399399592	-1,324095236	0,8154019	1	unspecified product
PFIT_1206300	7424,897817	5688,278081	6595,671938	6724,966381	5032,721493	6193,320089	6702,287422	7089,997322	6276,642633	6896,142372	1,098699201	0,135796489	0,8157558	1	eukaryotic translation initiation factor 5, putative
PFIT_0728200	4505,122331	3082,915284	3838,638652	4529,545719	2401,145822	3210,846188	2863,747495	3670,430559	3594,702333	3267,089027	0,908862188	-0,137866542	0,8157738	1	60S ribosomal subunit export protein, putative
PFIT_0105700	1,267263666	5,111418045	15,35747984	17,57340725	18,36759669	34,08541601	13,47328862	5,212754212	15,29376358	9,343021414	0,610904004	-0,710982398	0,8159484	1	carbon catabolite repressor protein 4, putative
PFIT_1240900	26,61253698	15,33425413	20,47663979	15,69054219	0	10,2256248	18,86260406	18,24463974	14,72326632	18,5536219	1,260156646	0,333603082	0,8161114	1	rifin, pseudogene
PFIT_1461800	31,68159164	121,2136279	35,10281107	95,3984965	141,931429	20,45124961	75,45041625	60,59826771	74,29653428	68,02434198	0,915578839	-0,127243819	0,8161252	1	inner membrane complex protein, putative
PFIT_1229900	1515,647344	378,9751379	314,4626825	321,9699257	220,4111603	149,9758304	371,1891014	295,8238015	483,5736801	333,5064514	0,689670396	-0,536021054	0,8161308	1	vacuolar transporter chaperone, putative, pseudogene
PFIT_1148800	5147,62501	449,0745854	557,2571257	441,845668	230,4298494	252,2320785	455,3971552	473,0574447	1179,744053	464,2273	0,393498318	-1,345570627	0,8161949	1	unspecified product
PFIT_1311700	58,29412862	291,3508286	146,2617128	360,2548486	207,0529081	17,04270801	202,7729937	187,0075573	180,0428558	194,8902755	1,082466031	0,114321752	0,8162106	1	conserved Plasmodium protein, unknown function
PFIT_0422300	788,238	2175,273479	673,5351874	2301,488728	1442,691231	293,1345777	1303,540674	1382,03146	1279,060201	1342,786067	1,049822414	0,070145304	0,8164804	1	reticulocyte binding protein homologue 5
PFIT_0608500	45,62149196	62,79742169	52,6542166	53,34784344	58,4423531	34,08541601	65,34544979	54,08232495	51,1581238	59,71388737	1,167241543	0,223103136	0,8164849	1	probable protein, unknown function
PFIT_0830100	1145,606354	353,4180477	309,3435225	350,2129016	243,7881015	115,8904144	337,5058798	324,4939497	419,7098902	330,9999148	0,788639776	-0,342561615	0,8168759	1	conserved Plasmodium protein, unknown function
PFIT_1406500	266,1253698	484,8545117	604,7921824	420,5065306	444,1618836	449,9274913	485,0383902	518,6690441	445,0613282	501,8537171	1,12760576	0,173262752	0,817079	1	COBW domain-containing protein 1, putative (CBWD1)
PFIT_0712200	575,3377042	21,17587476	38,02804533	62,76216875	31,72584883	40,90249921	48,50383902	41,70203369	128,3220235	45,10293636	0,351482428	-1,508475531	0,8170907	1	conserved Plasmodium protein, unknown function
PFIT_0302100	368,7737267	401,6114178	151,3808727	296,8650582	1061,981045	126,1160392	251,9504971	306,2493099	401,1213599	279,0999035	0,695799156	-0,523257167	0,8171741	1	cytoadherence linked asexual protein 3.2
PFIT_1453800	3938,655473	2665,969612	2606,383722	2122,616547	2426,192544	2413,247454	2981,638771	2715,844944	2695,510892	2848,741857	1,056846725	0,079766157	0,817239	1	conserved Plasmodium protein, unknown function
PFIT_0527500	44,3542283	48,92357271	51,19159948	50,83735669	148,610555	44,31104081	50,52483231	46,26319363	64,7047255	48,39401297	0,747920845	-0,419042502	0,817256	1	conserved Plasmodium protein, unknown function
PFIT_0911000	472,6893473	519,1740328	628,925365	493,9382681	631,1774135	633,9887378	635,9392227	547,9907865	563,3155274	591,9650046	1,050858668	0,071568651	0,8172686	1	nucleotide binding protein, putative
PFIT_0814500	754,021881	1790,456721	575,5398398	1062,563517	4546,815071	964,6172731	1086,620727	1003,455186	1615,669051	1045,037956	0,646814368	-0,628576368	0,8173618	1	conserved Plasmodium protein, unknown function
PFIT_1242200	147,0025852	332,2421729	248,6449117	200,83894	285,5326394	242,0064537	233,7615575	187,0075573	242,7112838	210,3845574	0,866809957	-0,20621237	0,8174867	1	conserved Plasmodium protein, unknown function
PFIT_0520400	620,9591961	484,1243091	516,3038461	471,3438873	500,9344552	443,1104081	544,9945245	550,5971636	506,1293503	547,7958441	1,082323805	0,114132183	0,8175295	1	conserved Plasmodium protein, unknown function
PFIT_1006800	145,7353215	338,0837935	100,9205818	246,0277015	692,9593296	119,298956	211,5306313	196,1298772	273,837614	203,8302542	0,744347174	-0,425952422	0,8175959	1	rhoGAP GTPase, putative
PFIT_1228600	1452,284161	1308,523019	1708,336805	1643,7412	981,8315321	2096,253085	1178,239089	1425,036683	1531,8283	1301,637886	0,849728319	-0,234926449	0,8179215	1	conserved Plasmodium protein, unknown function
PFIT_0614100	78,57034727	81,05248614	21,20794835	15,69054219	35,06541186	27,26833281	45,80918129	47,56638218	43,14251144	46,68778174	1,082175798	0,113934882	0,8179539	1	enoyl-acyl carrier reductase
PFIT_0713100	617,1574051	1849,60313	2586,638391	2059,226757	1696,498021	2065,57621	2087,012407	2072,721393	1812,449986	2079,8669	1,147544438	0,198550022	0,8179781	1	4-nitrophenylphosphatase
PFIT_0507800	13061,6866	7495,529461	8695,990134	10910,57542	5339,961292	8834,93983	8160,770915	7906,444951	9056,447122	8033,607933	0,887059553	-0,172897132	0,8180241	1	conserved Plasmodium protein, unknown function
PFIT_1448300	852,8684469	936,1197048	868,0632654	800,2176516	721,3456154	766,9218602	941,1092098	893,9873473	824,2560907	917,5482786	1,113183498	0,154691428	0,8180781	1	conserved Plasmodium protein, unknown function
PFIT_1425200	1,267263666	14,60405156	1,462617128	3,138108437	80,14951283	44,31104081	8,083973169	7,167537041	24,3554324	7,625755105	0,315695243	-1,663395573	0,8181178	1	small nucleolar RNA snoR26
PFIT_bin01500	126,7263666	270,1749538	305,6869797	197,7008316	203,7133451	252,2320785	240,4982018	266,5020591	226,0390925	253,5001304	1,121488003	0,165414187	0,8182211	1	Plasmodium exported protein (PHISTa), unknown function
PFIT_0214400	1181,089736	600,9567215	515,5725376	607,5377935	634,5169765	378,3481177	734,967894	593,562943	653,0036472	564,2654185	0,864107606	-0,210717114	0,8182664	1	acyl-CoA synthetase (ACS9)
PFIT_0910700	2757,565736	3153,014731	3472,98437	3634,557192	2933,806126	3694,859096	2747,877213	2973,876278	3274,464542	2860,876745	0,873692999	-0,194801665	0,818524	1	DNA repair protein REV1, putative
PFIT_1330100	0	1,460405156	0,731308564	0	41,74453793	13,6341664	2,020993292	1,303188553	9,595069676	1,662090923	0,173223434	-2,529293978	0,818532	1	small nucleolar RNA snoR17
PFIT_1129700	250,9182058	461,4880292	125,0537644	227,1990509	1409,295601	184,0612465	257,3398126	244,3478537	443,0026495	250,8438331	0,566235514	-0,820525857	0,8186366	1	conserved Plasmodium protein, unknown function
PFIT_1354200	221,7711415	568,8278081	440,9790641	517,7878922	2235,837452	572,634989	452,7024975	473,709039	759,6397244	463,2057682	0,609770334	-0,713662132	0,8186835	1	DNA replication licensing factor MCM6 (MCM6)
PFIT_1135700.3	0	0,730202578	1,462617128	0,627621687	8,348907586	0	3,368322154	0	1,861558163	1,684161077	0,904705053	-0,144480565	0,8187037	1	conserved Plasmodium protein, unknown function
PFIT_0717600	7091,607473	1131,083793	2009,635934	2128,265142	986,8408767	586,2691554	1374,275439	1989,96892	2322,283729	1682,12218	0,724339698	-0,465261748	0,8188025	1	conserved Plasmodium protein, unknown function
PFIT_1145600	1296,41073	870,4014728	905,3600022	892,4780396	1529,51987	1104,367479	889,2370486	845,1177766	1099,756265	867,1774126	0,78851782	-0,342784736	0,8189335	1	Rab GTPase 6
PFIT_1334700	21,54348231	10,22283609	19,74533123	19,45627231	6,679126069	3,408541601	15,49428191	20,19942257	13,50926494	17,84685224	1,321082407	0,401720463	0,8189594	1	conserved Plasmodium protein, unknown function
PFIT_0521400	0	8,762430934	8,775702767	5,0209735	15,02803365	10,2256248	5,389315446	3,909565659	7,968794277	4,649440553	0,583455965	-0,77730432	0,8191254	1	conserved Plasmodium protein, unknown function, putative
PFIT_0717500	7454,044881	2007,326886	1978,920974	1539,555999	1414,304945	2290,539956	2468,980139	1492,150893	2780,782274	1980,565516	0,712233221	-0,489578365	0,8191578	1	actin-related protein, putative (ARP6)
PFIT_1367900	709,6676527	460,027624	633,3132164	586,1986561	525,9811779	637,3972794	601,5823367	650,9426822	592,0976011	626,2625094	1,057701481	0,080932507	0,8191873	1	conserved Plasmodium protein, unknown function
PFIT_1224800	370,0409903	262,1427254	284,4790314	258,5801352	362,3425892	668,0741538	300,4543361	219,5872712	367,6099376	260,0208036	0,707328005	-0,499548713	0,8192522	1	isoleucine--tRNA ligase, putative
PFIT_1213200	24,07800965	54,03499076	44,6098224	47,69924825	238,778757	27,26833281	46,48284572	37,14087376	72,74486014	41,81185974	0,574774076	-0,798933102	0,8194228	1	dynein light chain 1, putative
PFIT_1406000	339,6266624	590,7338855	838,0796143	814,6529504	629,507632	633,9887378	701,2846724	781,2615375	641,098247	741,273105	1,15625508	0,209459704	0,8196085	1	conserved Plasmodium protein, unknown function
PFIT_1134700	4699,013672	3463,350827	3972,468119	3173,882874	3165,905757	6718,235496	3172,285805	3281,428776	4198,809457	3226,85729	0,768517201	-0,379850545	0,8196378	1	heat shock protein 70
PFIT_0308300	54,49233762	193,5036831	170,3948954	129,2900676	490,9157661	1479,307055	133,3855573	130,3188553	419,6506341	131,8522063	0,314195179	-1,670267049	0,8197844	1	U4 Spliceosomal RNA
PFIT_0417100	234,4437781	350,4972374	330,5514709	269,8773256	479,2272954	422,6591585	270,8131012	282,791916	347,8760443	276,8025086	0,795692929	-0,329716317	0,8198537	1	Bet3 transport protein, putative
PFIT_0621500	7,603581993	15,33425413	3,65654282	9,414325312	73,47038676	10,2256248	12,12595975	5,212754212	19,95087597	8,669356983	0,434537115	-1,202448687	0,8198591	1	conserved Plasmodium protein, unknown function
PFIT_1353400	489,1637749	876,2430934	655,2524733	582,432926	1280,722424	732,8364442	683,0957328	568,8418034	769,4418559	625,9687681	0,813536154	-0,297721634	0,8199134	1	inositol-polyphosphate 5-phosphatase, putative
PFIT_0913000	16582,14506	1295,379373	1733,932605	1605,456277	1362,541718	1861,063714	1670,014124	1864,862819	4073,419792	1767,438472	0,433895489	-1,204580508	0,8200235	1	26S proteasome regulatory subunit, putative
PFIT_1463100	6726,635537	4048,973294	4229,157425	3423,676305	2980,560008	4090,249921	4598,433405	4389,790641	4249,875415	4494,112023	1,057469122	0,080615537	0,8200733	1	valine--tRNA ligase, putative
PFIT_1103100	35,48338264	13,87384898	15,35747984	19,45627231	1,669781517	6,817083202	14,14695305	11,72869698	15,44297475	12,93782501	0,837780623	-0,255355578	0,8200817	1	Plasmodium exported protein, unknown function
PFIT_0536800	13240,37078	5408,610494	6072,055006	5440,852409	4089,294936	12908,14704	5946,435931	5616,742663	7859,888444	5781,589297	0,735581597	-0,443042708	0,8201159	1	erythrocyte membrane protein 1 (PfEMP1), pseudogene (VAR1CSA)
PFIT_1410600	2317,825244	2867,505523	3820,355938	4854,653753	2167,376409	3047,236191	2644,806555	3456,707637	3179,158843	3050,757096	0,959611409	-0,059477785	0,8202224	1	conserved Plasmodium protein, unknown function
PFIT_0715000	214,1675595	293,5414363	300,5678198	258,5801352	260,4859167	231,7808289	304,4963227	282,1403217	259,8539494	293,3183222	1,128781467	0,174766206	0,8205367	1	drug metabolite transporter, putative
PFIT_1366800	93,77751125	10,22283609	8,044394203	12,55243375	46,75388248	40,90249921	14,14695305	22,80579968	35,37559283	18,47637636	0,522291639	-0,937072486	0,8206445	1	secreted ookinete protein, putative
PFIT_1362000	3362,050505	1455,293738	1668,114834	1578,468544	1484,435769	1803,118507	1515,744969	1584,67728	1891,913649	1550,211125	0,819387886	-0,287381532	0,8206515	1	conserved Plasmodium protein, unknown function
PFIT_0505300	248,3836785	220,5211785	136,7547015	312,5556004	205,3831266	30,67687441	200,7520004	182,4463974	192,3791933	191,5991989	0,995945537	-0,005861244	0,8206657	1	UDP-N-acetyl glucosamine:UMP antiporter
PFIT_1325200	31,68159164	131,436464	51,19159948	71,54887237	275,5139503	61,35374882	60,62979877	84,05566166	103,7877044	72,34273022	0,697026017	-0,520715589	0,8207015	1	conserved Plasmodium protein, unknown function
PFIT_bin00500	332,0230804	392,1187843	767,8739921	755,0288901	327,2771774	2058,759127	524,7845916	834,0406739	772,1801752	679,4126327	0,879862828	-0,184649472	0,820788	1	rifin (RIF)
PFIT_0406700	1508,043762	1145,687845	1726,619519	1960,690152	1167,177281	1152,087061	1348,002526	1354,012906	1443,38427	1351,007716	0,936000027	-0,095419523	0,8209224	1	conserved Plasmodium protein, unknown function
PFIT_1340900	1,267263666	3,651012889	1,462617128	3,765730125	0	0	3,368322154	1,954782829	1,691103968	2,661552492	1,573855033	0,654302661	0,8209913	1	conserved Plasmodium protein, unknown function
PFIT_0406100	114,0537299	165,7559852	177,707981	142,4701231	106,8660171	139,7502056	184,584054	140,7443637	141,1006737	162,6642089	1,152823758	0,205171973	0,8210161	1	mitochondrial carrier protein, putative
PFIT_0718100	10277,50833	6710,56169	8015,873169	6893,168994	5989,506302	7566,962354	7885,915827	8259,609048	7575,596806	8072,762438	1,065627256	0,091702888	0,8210692	1	conserved Plasmodium membrane protein, unknown function
PFIT_1476500	530,9834759	192,043278	223,7804206	177,6169376	198,7040005	190,8783297	235,1088863	175,2788604	252,334407	205,1938734	0,8131823	-0,298349282	0,8210752	1	cysteine repeat modular protein 4
PFIT_1470100	38,01790997	59,1464088	56,31075942	49,58211331	126,9033953	207,9210377	53,89315446	50,17275929	89,64693741	52,03295688	0,580420909	-0,784828604	0,8212145	1	translation initiation factor IF-1, putative
PFIT_1253500	106,4501479	101,4981583	69,47431357	84,72892781	90,16820193	37,49395761	77,47140954	69,0689933	81,63561786	73,27020142	0,89752737	-0,15597216	0,8213183	1	Plasmodium exported protein (hyp15), unknown function, pseudogene
PFIT_1236600	25,34527331	167,2163903	73,13085639	183,2655327	389,0590935	126,1160392	111,8282955	129,667261	160,6888643	120,7477783	0,7514387	-0,41227331	0,8214568	1	conserved Plasmodium protein, unknown function
PFIT_0534300	390,317209	1247,916205	1085,993217	1312,356949	1110,404709	804,4158178	1212,595975	1154,625058	991,9006845	1183,610517	1,193275229	0,254926839	0,8216036	1	SNARE protein, putative
PFIT_1124100	301,6087524	599,4963164	260,3458488	325,7356558	556,0372452	228,3722873	343,5688597	328,4035153	378,599351	335,9861875	0,887445228	-0,172270013	0,8218601	1	leucine-rich repeat protein
PFIT_1036600	332,0230804	300,1132595	209,8855579	253,5591617	365,6821523	289,7260361	230,3932353	242,3930708	291,8315413	236,3931531	0,810032912	-0,303947569	0,8219068	1	conserved Plasmodium protein, unknown function
PFIT_0807500	6342,654646	1470,627992	1755,140553	2525,54967	930,0683051	913,4891491	1787,231735	1926,764275	2322,921719	1856,998005	0,799423411	-0,322968272	0,8220206	1	conserved Plasmodium protein, unknown function
PFIT_0422000	567,7341222	2535,993553	749,591278	1429,094582	1666,441954	432,8847833	1293,435707	1137,683607	1230,290045	1215,559657	0,988026898	-0,017377777	0,8220276	1	cysteine-rich protective antigen (CyRPA)
PFIT_0516200	624,7609871	514,7928174	330,5514709	352,7233884	422,4547239	282,9089529	522,7635983	396,1693201	421,3653901	459,4664592	1,090422873	0,12488773	0,8221694	1	cation-transporting ATPase 1
PFIT_0835200.1	17,74169132	46,0027624	92,87618762	61,50692537	76,80949479	398,7993673	39,07253699	50,82435356	115,622814	44,94844527	0,38875066	-1,363082969	0,8222162	1	Plasmodium exported protein (PHISTa-like), unknown function
PFIT_1027800	2558,605341	1410,75138	1760,259713	1682,026122	1285,731768	2212,143499	1500,924352	1489,544516	1818,252971	1495,234434	0,822346757	-0,282181235	0,8222628	1	nucleolar preribosomal assembly protein, putative
PFIT_1109900	375,110045	502,3793735	180,6332153	165,0645038	197,034219	115,8904144	325,3799201	274,3211904	256,0186285	299,8505552	1,171206005	0,227994856	0,8223232	1	conserved Plasmodium protein, unknown function
PFIT_1013000	2436,948029	2701,019335	3018,841752	3525,97864	2756,809285	2379,162038	2419,128971	2727,573641	2803,126513	2573,351306	0,918028956	-0,123388436	0,8223675	1	phosphoinositide-specific phospholipase C
PFIT_0201900.2	136,8644759	62,79742169	68,01169645	43,93351812	51,76322703	122,7074976	59,28246991	56,68870205	81,0129728	57,98558598	0,715756798	-0,482458628	0,8223886	1	conserved Plasmodium protein, unknown function
PFIT_1228200	138,1317395	385,5469611	262,5397745	446,8666415	212,0622527	105,6647896	348,2845107	344,041778	258,4686932	346,1631443	1,339284616	0,421462585	0,8225117	1	RNA pseudouridylate synthase, putative
PFIT_0616800	17872,21948	11754,0709	11477,1566	20313,60354	17328,99259	13361,48308	10098,22982	16518,5665	15351,25436	13308,39816	0,86692578	-0,20601961	0,8226529	1	histone H3 variant, putative
PFIT_1214000	29,14706431	216,8701656	192,3341523	121,1309857	101,8566725	40,90249921	177,8474097	138,7895809	117,0402566	158,3184953	1,35268411	0,435824968	0,8226627	1	conserved protein, unknown function
PFIT_0812800	2979,336878	3697,015652	3834,250801	4091,465781	2873,693991	2024,673711	3300,282046	3005,804397	3250,072802	3153,043222	0,970145413	-0,043727089	0,8227571	1	JmjC domain containing protein
PFIT_0706700	1217,840383	487,0451194	529,4674003	466,9505355	487,576203	371,5310345	508,6166452	493,2568673	593,4017792	500,9367563	0,844178049	-0,244380779	0,8227991	1	RAP protein, putative
PFIT_1339000	0	0	0,731308564	1,255243375	1,669781517	6,817083202	0	0	1,745569443	0	0	#DIV/0!	0,8228044	1	mitotic-spindle organizing protein 1, putative
PFIT_0609200	185,0204952	61,33701654	90,68226193	134,9386628	33,39563034	6,817083202	60,62979877	103,60349	85,36519167	82,11664436	0,961945294	-0,055973245	0,822814	1	unspecified product
PFIT_0608700	158,4079582	165,7559852	168,2009697	190,796993	562,7163713	81,80489842	181,8893963	151,1698721	221,280546	166,5296342	0,752572412	-0,410097691	0,8228266	1	zinc finger protein, putative
PFIT_1431800	3569,881746	590,7338855	585,0468512	754,4012684	295,5513285	293,1345777	599,5613434	650,9426822	1014,79161	625,2520128	0,616138335	-0,698673794	0,8228431	1	exodeoxyribonuclease III, putative
PFIT_1200600	558,8632765	2535,26335	2808,956194	2505,465776	2105,594493	1329,331224	2573,398126	2335,965481	1973,912386	2454,681803	1,24356168	0,314478067	0,8229966	1	Plasmodium exported protein (PHISTc), unknown function
PFIT_0422700	17125,80118	9913,230196	13997,24591	13685,2909	8218,664628	9308,727112	13465,20464	13878,30649	12041,49332	13671,75557	1,135387049	0,183184191	0,8229999	1	serine/threonine protein kinase, FIKK family
PFIT_0537600	201,4949228	94,19613254	92,87618762	64,01741212	61,78191614	180,6527049	93,63935588	74,28174752	115,836546	83,9605517	0,724819192	-0,464306939	0,8230091	1	B_ITvar25 - erythrocyte membrane protein 1, PfEMP1, putative
PFIT_0319200	2018,751019	1369,860036	1900,670958	1894,162253	1100,38602	2038,307877	1758,937829	1949,570075	1720,356361	1854,253952	1,077831311	0,108131403	0,8230416	1	endonuclease/exonuclease/phosphatase family protein, putative
PFIT_0204400	254,7199968	109,5303867	156,5000327	179,4998026	75,14016827	54,53666562	122,6069264	140,0927694	138,3211754	131,3498479	0,949600432	-0,074607504	0,8230483	1	conserved Plasmodium protein, unknown function
PFIT_1210900	383,9808907	291,3508286	277,8972543	296,2374365	472,5481694	402,2079089	252,6241615	299,0817729	354,0370814	275,8529672	0,779164053	-0,360000975	0,8230695	1	DNA helicase MCM8, putative (MCM8)
PFIT_0728400	1376,248341	2447,639041	2955,949215	2865,093003	2376,099099	3169,943689	2770,108139	2764,714515	2531,828791	2767,411327	1,093048393	0,128357276	0,8231305	1	mRNA (N6-adenosine)-methyltransferase, putative
PFIT_1128300	72,23402894	127,0552485	66,54907932	59,62406031	71,80060524	95,43916483	97,00767803	79,49450173	82,1170312	88,25108988	1,074698983	0,103932625	0,8231504	1	conserved Plasmodium protein, unknown function
PFIT_1312300	646,3044694	223,4419888	220,8551863	198,3284532	188,6853114	173,8356212	234,4352219	213,0713284	275,2418385	223,7532752	0,812933369	-0,298790986	0,823191	1	conserved Plasmodium protein, unknown function
PFIT_0107400	1055,630633	854,3370161	695,4744443	662,768502	343,9749925	252,2320785	707,3476523	551,2487579	644,0696111	629,2982051	0,977065513	-0,033472795	0,8232037	1	unspecified product
PFIT_1448400	14856,13195	2710,511969	3694,570865	3519,702423	2289,27046	4557,220121	3634,419604	3599,406783	5271,234632	3616,913194	0,686160539	-0,543381937	0,8232509	1	conserved Plasmodium protein, unknown function
PFIT_0318300	3874,025026	3185,873847	3616,320849	3300,034833	2880,373117	3705,08472	3924,095309	3404,580095	3426,952065	3664,337702	1,069270195	0,096626455	0,8232523	1	conserved Plasmodium protein, unknown function
PFIT_0106600	81,1048746	110,9907918	81,90655916	74,05935912	163,6385887	187,4697881	83,53438942	81,44928456	116,5283269	82,49183699	0,707912309	-0,498357434	0,8233817	1	conserved Plasmodium protein, unknown function
PFIT_1467400	8446,312331	9964,344377	11245,33179	10785,6787	10846,90074	14731,7168	10806,25113	11877,91207	11003,38079	11342,0816	1,030781522	0,043738581	0,823417	1	26S proteasome regulatory subunit, putative
PFIT_0418300	292,7379067	182,5506445	85,56310198	150,629205	429,1338499	115,8904144	212,8779601	187,0075573	209,4175204	199,9427587	0,954756595	-0,066795115	0,8235157	1	apical merozoite protein
PFIT_1364300	618,4246688	1234,772559	1072,098355	1211,937479	856,5979183	715,7937362	939,0882165	906,3676386	951,6041193	922,7279275	0,969655247	-0,044456195	0,8235495	1	conserved Plasmodium protein, unknown function
PFIT_0917200	833,859492	256,3011048	268,390243	262,3458654	227,0902863	296,5431193	311,232967	252,166985	357,4216851	281,699976	0,788144614	-0,343467726	0,8238657	1	N-glycosylase/DNA lyase, putative
PFIT_1301200	52163,107	10828,90423	13061,90226	12832,98064	8395,661469	11888,9931	12303,1335	13548,59979	18195,25812	12925,86664	0,710397542	-0,493301505	0,8239145	1	Plasmodium exported protein, unknown function
PFIT_1332300	272,4616881	311,0662982	278,6285629	201,4665617	308,9095807	439,7018665	336,8322154	251,5153907	302,039093	294,1738031	0,973959364	-0,038066514	0,8239369	1	20 kDa chaperonin
PFIT_0419600	0	0	2,193925692	0	6,679126069	6,817083202	0,673664431	2,606377106	2,615022494	1,640020768	0,627153599	-0,673109272	0,8239505	1	unspecified product
PFIT_1457600	111,5192026	175,2486187	90,68226193	160,6714152	148,610555	54,53666562	146,8588459	150,5182779	123,5447426	148,6885619	1,203519945	0,267260051	0,8239709	1	conserved Plasmodium protein, unknown function
PFIT_0530600	22,81074598	74,48066294	88,48833624	99,16422662	91,83798345	40,90249921	88,25004043	83,40406739	69,61407574	85,82705391	1,232897988	0,302053434	0,8239717	1	conserved Plasmodium protein, unknown function
PFIT_1328200	1155,744463	484,1243091	494,3645892	454,3981017	687,9499851	831,6841507	505,2483231	518,0174498	684,7109331	511,6328864	0,747224649	-0,420386049	0,8239755	1	conserved Plasmodium protein, unknown function
PFIT_0921000	892,1536206	1146,418047	1600,103138	1550,85319	1152,149247	947,5745651	1385,05407	1484,331762	1214,875301	1434,692916	1,180938418	0,239933734	0,8241192	1	U4/U6 snRNA-associated-splicing factor, putative
PFIT_0712600	2025,087338	1445,070902	1737,589148	1880,982197	1554,566593	1434,996014	1479,36709	1501,924807	1679,715365	1490,645949	0,88743961	-0,172279147	0,8242126	1	transcription elongation factor s-II, putative
PFIT_1336900	147,0025852	231,4742172	96,53273044	153,7673134	430,8036314	136,341664	159,6584701	152,4730607	199,320357	156,0657654	0,782989594	-0,352934961	0,8243725	1	conserved Plasmodium protein, unknown function
PFIT_1034300	2620,70126	5783,204416	5868,019917	5230,599144	7054,82691	4570,854287	6281,247153	5215,360589	5188,034322	5748,303871	1,107992645	0,147948304	0,8245828	1	methionine--tRNA ligase, putative
PFIT_0413200	110,2519389	172,3278084	84,83179342	158,7882869	405,7569087	143,1587472	124,6279197	146,6087122	179,1859139	135,6183159	0,756858131	-0,401905194	0,8246312	1	conserved protein, unknown function
PFIT_1309600	3260,669412	2351,252301	2671,470184	2942,918093	1998,728476	3909,597216	2357,151843	2367,242006	2855,772614	2362,196925	0,82716562	-0,273751871	0,8246645	1	conserved Plasmodium protein, unknown function
PFIT_1120000	1715,875003	633,8158375	522,1543147	441,845668	400,7475641	593,0862386	555,7731554	562,3258606	717,920771	559,049508	0,778706412	-0,360848588	0,8247764	1	conserved protein, unknown function
PFIT_0601400	79,83761093	242,4272558	356,1472706	271,7601907	287,202421	548,7751978	274,8550878	308,855687	297,6916578	291,8553874	0,980394915	-0,028565094	0,8250002	1	geranylgeranyltransferase, putative
PFIT_1123500	2287,410916	206,6473295	230,3621976	412,3474487	202,0435636	95,43916483	224,3302555	344,041778	572,3751034	284,1860167	0,496503106	-1,010125352	0,825027	1	conserved Plasmodium protein, unknown function
PFIT_1343000	2798,118174	3108,472374	2101,780813	2532,453509	4688,7465	1922,417463	2367,930474	2445,43332	2858,664805	2406,681897	0,841890204	-0,248296	0,8251105	1	conserved Plasmodium protein, unknown function
PFIT_0511900	3771,376669	4241,016572	4133,356003	3413,006737	5545,344419	4202,731794	4756,744546	3954,525664	4217,805366	4355,635105	1,03267807	0,046390575	0,825265	1	myo-inositol 1-phosphate synthase, putative
PFIT_0408600	3415,275579	5542,237566	3303,320783	4245,233094	4379,83692	4502,683455	3687,639094	3685,417228	4231,431233	3686,528161	0,871224878	-0,198802943	0,8252081	1	conserved Plasmodium protein, unknown function
PFIT_1470700	538,5870579	535,9686921	338,5958651	308,7898702	641,1961026	364,7139513	476,2807526	451,5548336	454,6419232	463,9177931	1,020402584	0,029138457	0,8253326	1	biotin carboxylase subunit of acetyl CoA carboxylase, putative
PFIT_0611600	582,9412862	624,323204	525,8108575	542,8927597	572,7350604	477,1958241	494,4696922	481,5281703	554,3164987	487,9989313	0,880361549	-0,18383196	0,8253496	1	PP-loop family protein, putative
PFIT_1433900	19,00895498	32,12891342	33,64019394	28,87059762	252,1370091	34,08541601	29,64123495	27,36695961	66,64518085	28,50409728	0,427699301	-1,225331245	0,8255687	1	protein-L-isoaspartate O-methyltransferase beta-aspartate methyltransferase, put
PFIT_0405100	2321,627035	1411,481583	1955,5191	2128,892764	921,7193975	1216,849352	1983,941749	1912,429201	1659,348205	1948,185475	1,174066702	0,231514375	0,8255755	1	conserved Plasmodium protein, unknown function
PFIT_1241100	435,938701	253,3802945	387,5935389	318,8318172	161,9688072	480,6043657	248,582175	311,4620641	339,7195874	280,0221196	0,824274284	-0,27880361	0,8256868	1	erythrocyte membrane protein 1, PfEMP1
PFIT_1301700	3381,05946	3604,279924	3309,171252	3561,125455	2715,064747	2242,820373	2884,631093	2982,347003	3135,586869	2933,489048	0,935547051	-0,096117883	0,8259806	1	conserved Plasmodium protein, unknown function
PFIT_1306000	610,8210868	606,0681396	599,6730224	589,3367646	606,1306907	777,147485	499,1853432	548,6423808	631,5295315	523,913862	0,82959519	-0,269520565	0,826219	1	RED-like protein, putative
PFIT_1118000	346874,1433	14247,7127	16534,15532	18412,53745	5391,724519	4008,444923	15532,68078	17422,97936	67578,1197	16477,83007	0,243833805	-2,03602994	0,8262395	1	conserved Plasmodium protein, unknown function
PFIT_1342700	35,48338264	59,87661138	76,05609065	99,79184831	395,7382196	105,6647896	71,40842966	76,23653035	128,7684904	73,82248	0,573296152	-0,8026475	0,8263478	1	conserved Plasmodium protein, unknown function
PFIT_1111600	26,61253698	42,35174951	23,40187405	33,26394944	100,186891	13,6341664	27,62024166	33,2313081	39,9085279	30,42577488	0,762387802	-0,391403058	0,8264008	1	conserved Plasmodium protein, unknown function

[illegible]

PFIT_1311800	13,93990032	261,4125229	82,63786773	175,1064508	601,1213462	78,39645682	147,5325103	140,0927694	202,1024241	143,8126399	0,711582954	-0,490896144	0,8725917	1	branched-chain alpha keto-acid dehydrogenase, putative
PFIT_0410900	79,83761093	216,8701656	141,1425528	128,0348242	135,2523029	81,80499842	128,6699063	128,3640725	130,4904092	128,5169894	0,984876898	-0,021984685	0,8727684	1	phosphopantothenoylcysteine synthetase, putative
PFIT_1314400	63,36318328	173,7882135	158,6939584	142,4701231	243,7881015	218,1466625	144,8378526	129,667261	166,7083737	137,2525568	0,823309314	-0,280493547	0,8729226	1	conserved Plasmodium protein, unknown function
PFIT_0835600	102,6483569	75,94106809	86,29441055	73,43173744	106,8660171	109,0733312	90,94469816	87,31363305	92,37582022	89,1291656	0,964853848	-0,051617669	0,873039	1	erythrocyte membrane protein 1, PfEMP1, putative
PFIT_1104700	5,069054662	7,302025778	8,044394203	5,648595187	6,679126069	3,408541601	5,389315446	3,257971382	6,025289584	4,323643414	0,717582675	-0,478783037	0,8731396	1	conserved Plasmodium protein, unknown function
PFIT_1253100	16,47442765	51,84438303	39,49066245	85,3565495	48,423664	10,2256248	43,788188	66,4626162	41,96921857	55,1254021	1,313472206	0,393385672	0,8731835	1	Plasmodium exported protein, unknown function
PFIT_1363900	143,2007942	194,9640883	272,0467858	217,1571039	128,5731768	201,1039545	194,6890205	239,1350995	192,8409839	216,91206	1,124823446	0,169698571	0,8731866	1	ferrochelatase
PFIT_0626700	5398,543215	7210,020253	7400,111359	7459,911378	6216,596589	6711,418412	6987,921141	7630,168977	6732,766868	7309,045059	1,085593071	0,118483417	0,8734329	1	acetyl-CoA synthetase, putative
PFIT_0924400	12,67263666	110,2605893	90,68226193	150,629205	235,4391939	88,62208163	103,7443223	99,04233002	114,7176614	101,3933262	0,883851056	-0,178124823	0,8735438	1	thiamine pyrophosphokinase
PFIT_0503200	119,1227846	114,6418047	111,1589017	74,05935912	195,3644375	265,8662449	99,02867133	104,2550842	146,7022554	101,6418778	0,692844684	-0,529396118	0,8735921	1	4-diphosphocytidyl-2c-methyl-D-erythritol kinase (CMK), putative
PFIT_1415400	325,6867621	861,6390418	369,3108248	472,5991307	2497,99315	780,5560266	631,2235716	509,5467242	884,6308226	570,3851479	0,644771958	-0,633139094	0,8738012	1	serine/threonine protein phosphatase
PFIT_1348300	64,63044694	51,84438303	21,20794835	38,91254462	123,5638323	23,85979121	55,91414776	45,61159935	54,00315774	50,76287355	0,939998246	-0,08927003	0,8738037	1	nucleoside diphosphate hydrolase, putative
PFIT_1202600	18153,55201	8908,471449	9249,590717	10023,11835	12394,7882	23873,42537	12578,66225	10983,92472	13767,15768	11781,29348	0,855753508	-0,224732793	0,8738129	1	high mobility group protein B1 (HMGB1)
PFIT_1358300	252,1854694	164,29558	125,785073	120,503364	248,7974461	156,7929136	128,6699063	156,3826264	178,0599744	142,5262663	0,800439665	-0,321135434	0,8738286	1	high mobility group protein B4, putative (HMGB4)
PFIT_1224900	1198,831428	1376,431859	1711,26204	2164,6672	1137,121213	1513,392471	1364,170472	1566,432641	1516,951035	1465,301556	0,965951783	-0,049976919	0,8739953	1	conserved Plasmodium protein, unknown function
PFIT_0715400	457,4821833	1005,48895	1017,250212	1178,045907	895,0028932	1080,507688	941,7828742	813,8412513	938,9629723	877,8120628	0,934873993	-0,097156171	0,8740691	1	transcription initiation factor IIE, alpha subunit, putative
PFIT_1351100	2042,829029	929,5478816	1779,273736	1549,597946	841,5698847	1407,727681	1461,851815	1570,993801	1425,091026	1516,422808	1,064088384	0,089617987	0,8741042	1	conserved Plasmodium protein, unknown function
PFIT_1421500	46,88875563	52,5745856	62,8925365	55,85833019	40,07475641	54,53666562	58,60880548	55,3855135	52,13760499	56,99715949	1,093206324	0,128565711	0,8741448	1	conserved Plasmodium membrane protein, unknown function
PFIT_0620400	136,8644759	159,184162	163,0818098	111,7166604	187,0155299	197,6954129	156,9638124	152,4730607	159,2596751	154,7184365	0,97148532	-0,041735899	0,8742818	1	conserved Plasmodium protein, unknown function
PFIT_0622500	1448,48237	994,535911	1314,161489	1263,402457	1190,554222	1673,593926	1228,090257	994,9844602	1314,121729	1111,537359	0,845840483	-0,241542483	0,8743126	1	transcription or splicing factor-like protein, putative
PFIT_1343300	961,8531222	1106,987108	873,1824253	957,7506951	801,4951283	1097,550396	904,7313305	820,3571941	966,4698124	862,5442623	0,892468912	-0,164126179	0,8743592	1	krox-like protein. putative
PFIT_0531500	2102,390421	1477,930018	1433,364785	1527,003566	1177,19597	1285,020184	1637,004567	1556,007132	1500,484157	1596,505849	1,063993806	0,089489753	0,8744501	1	methionine aminopeptidase 1a, putative
PFIT_1365800	144,4680579	627,9742169	511,1846862	421,761774	415,7755978	473,7872825	491,7750345	467,1930962	432,4919359	479,4840653	1,108654348	0,148809638	0,8744937	1	phosphatidylserine synthase I, putative
PFIT_1141300	3126,339463	916,4042352	626,7314393	676,5761791	1016,896944	487,4214489	784,1453974	948,0696723	1141,728285	866,1075348	0,758593394	-0,398601286	0,8745094	1	conserved Plasmodium protein, unknown function
PFIT_0107100	164,7442765	185,4714548	175,5140553	252,9315401	156,9594626	98,84770643	192,6680272	153,124655	172,411416	172,8963411	1,002812605	0,004052035	0,8745792	1	conserved Plasmodium protein, unknown function
PFIT_1465700	26528,89758	15122,49539	11276,77806	14255,17139	10294,20305	10845,97937	14276,29662	12356,18226	14720,58747	13316,23944	0,904599729	-0,144648531	0,8747633	1	phosphatase, putative
PFIT_0726500	329,4885531	138,0082872	133,8294672	131,8005544	85,15885738	252,2320785	143,4905238	136,8347981	178,4196329	140,1626609	0,785578687	-0,348172306	0,8749464	1	conserved Plasmodium protein, unknown function
PFIT_0620200	2140,408331	2335,918046	3385,958651	3202,753471	2369,419973	4233,408668	2915,619656	3063,796288	2944,644524	2989,707972	1,015303528	0,021911089	0,8750095	1	mRNA binding Pumilio-homology domain protein, putative
PFIT_1101800	1022,681778	680,5488025	750,3225866	831,5987359	689,6197666	490,8299905	759,2198135	864,6656049	744,2669434	811,9427092	1,09092942	0,125557774	0,875026	1	Plasmodium exported protein (hyp4), unknown function
PFIT_0907300	6811,542202	847,0349903	870,9884997	883,691336	217,0715972	228,3722873	858,9221493	916,1415527	1643,116819	887,531851	0,540151401	-0,888564254	0,875036	1	conserved Plasmodium protein, unknown function
PFIT_1242000	16943,31521	6544,805705	7190,225801	7874,141691	4849,045526	6418,283835	7642,722967	7103,029208	8303,302961	7372,876087	0,887944969	-0,171457828	0,8751042	1	DEAD/DEAH box ATP-dependent RNA helicase, putative
PFIT_0800700	51,95781029	42,35174951	69,47431357	85,3565495	48,423664	40,90249921	44,46185243	63,85623909	56,41109768	54,15904576	0,960077857	-0,05877669	0,8752346	1	rifin (RIF)
PFIT_0519600	19320,70185	6939,8453	7699,94787	6684,170972	7185,069869	11558,36457	8932,790352	9380,351204	9898,016737	9156,570778	0,925091462	-0,112332085	0,8752756	1	40S ribosomal protein S24, putative
PFIT_0300800	17,74169132	3,651012889	2,193925692	0	8,348907586	0	4,041986585	1,303188553	5,322589581	2,672587569	0,50212167	-0,993891106	0,8753433	1	stevor-like
PFIT_0803700	81,1048746	335,8931858	575,5398398	692,2667213	210,3924712	64,76229042	404,8723229	525,1849868	326,6598972	465,0286549	1,423586608	0,509530266	0,8754914	1	Plasmodium exported protein (hyp7), unknown function, pseudogene
PFIT_0419100	0	13,87384898	5,850468512	11,29719037	43,41431945	10,2256248	7,410308739	10,42550842	14,11024202	8,917908581	0,632016699	-0,661965418	0,8755044	1	conserved Plasmodium protein, unknown function
PFIT_1454800	622,2264598	868,9410676	762,7548322	752,5184033	681,270859	1022,56248	719,4736121	882,2586503	785,0456837	800,8661312	1,020152264	0,028784499	0,8755954	1	RAP protein, putative
PFIT_0802100	188,8222862	871,1316753	542,6309544	636,4083911	541,0092116	368,1224929	689,8323771	562,3258606	524,6875019	626,0791189	1,193241914	0,25488656	0,8757129	1	Plasmodium exported protein, unknown function
PFIT_1126300	2781,643746	1376,431859	2202,701395	2082,448759	1272,373516	3469,89535	1597,93203	1981,498195	2197,582437	1789,715112	0,814401809	-0,296187329	0,87582	1	mitochondrial inner membrane translocase subunit TIM44, putative
PFIT_0617000	315,5486527	231,4742172	167,4696611	288,0783546	395,7382196	197,6954129	268,1184435	267,8052476	266,000753	267,9618455	1,007372507	0,010597264	0,8759503	1	conserved Plasmodium protein, unknown function
PFIT_0914900	24,07800965	49,65377529	38,02804533	47,69924825	313,9189252	64,76229042	38,39887255	54,08232495	89,69004903	46,24059875	0,51555967	-0,95578785	0,8760501	1	GINS complex subunit Psf3, putative
PFIT_1011600	82,37213826	95,65653769	88,48833624	121,7586074	305,5700176	177,2441633	107,7863089	106,2098671	145,1816334	106,998088	0,736994656	-0,440273936	0,8763523	1	conserved Plasmodium protein, unknown function
PFIT_bin06900	55,75960129	21,90607733	41,68458814	22,59438075	33,39563034	23,85979121	30,98856382	23,45739395	33,20001151	27,22297888	0,819968959	-0,286358799	0,8764088	1	A_ITvar60 - erythrocyte membrane protein 1, PfEMP1, putative (VAR)
PFIT_0305200	16340,0977	10801,88673	11293,59815	11561,41911	6981,356523	6408,05821	9848,300314	10536,27945	10564,40274	10192,28988	0,964776726	-0,051732991	0,8764292	1	conserved Plasmodium membrane protein, unknown function
PFIT_0313000	466,3530289	622,1325963	758,3669808	643,9398514	891,6633302	1428,178931	650,7598401	585,1316603	801,7724531	617,9457502	0,770724596	-0,375712662	0,8765575	1	ubiquitin-protein ligase, putative
PFIT_0909200	1990,871219	760,8710861	835,15438	711,0953719	322,2678328	388,5737425	735,6415584	775,397189	834,8056053	755,5193737	0,905024318	-0,143971537	0,8765809	1	conserved Plasmodium protein, unknown function
PFIT_0618300	2493,974894	5773,711783	4162,608346	3249,197476	6530,515514	6547,808416	4908,992707	4207,995837	4792,969405	4558,494272	0,95107936	-0,072362368	0,8765998	1	cell division cycle protein 48 homologue, putative
PFIT_1022000	861,7392926	1242,074585	909,7478535	1541,438864	1202,242692	620,3545714	1201,817345	1258,228548	1062,932977	1230,022946	1,157197089	0,210634599	0,8766118	1	citrate synthase, mitochondrial precursor, putative
PFIT_1212200.1	54,49233762	175,2486187	145,5304042	87,86703625	141,931429	115,8904144	131,364564	127,7124782	120,16004	129,5385211	1,078049916	0,10842398	0,8767055	1	conserved protein, unknown function
PFIT_0604700	5806,602116	1834,999078	2699,991218	3652,1306	1270,703735	773,7389434	2099,812031	3102,24035	2673,027615	2601,02619	0,973063718	-0,039393816	0,8767105	1	DNA repair-like protein, putative
PFIT_0834100	4818,136457	4766,762428	5961,627413	6930,198673	4196,160953	3210,846188	4830,847633	5171,703772	4980,622019	5001,275703	1,004146808	0,005970209	0,8767283	1	DnaJ protein, putative
PFIT_1435100	3612,968711	551,3029463	498,021132	554,8175717	298,8908916	163,6099968	580,6987393	572,751369	946,6018748	576,7250542	0,609258305	-0,714874082	0,8767448	1	protein kinase, putative
PFIT_1464400	11,40537299	40,16114178	38,02804533	43,30589644	105,1962356	44,31104081	48,50383902	41,70203369	47,06795549	45,10293636	0,958251445	-0,061523826	0,8767834	1	DNA polymerase alpha subunit, putative
PFIT_0407700	4249,135071	2793,02486	4216,72518	4773,690555	2199,102258	3401,724518	3124,45563	3751,22825	3605,567074	3437,84194	0,953481622	-0,068722964	0,876841	1	U4/U6 small nuclear ribonucleoprotein PRP31, putative
PFIT_0824800	3,801790997	13,87384898	11,70093702	8,786703625	30,05606731	10,2256248	6,062979877	12,38029125	13,07416212	9,221635565	0,705332814	-0,503623935	0,8769356	1	conserved Plasmodium protein, unknown function
PFIT_1030000	3179,564537	1660,480662	2167,598584	2060,482	1354,19281	2488,235369	1742,096218	1986,059355	2151,758994	1864,077786	0,866304169	-0,207054435	0,8770195	1	glycoprotease, putative
PFIT_1429200	115,3209936	75,94106809	73,13085639	82,84606275	101,8566725	68,17083202	75,45041625	72,32696469	86,2110809	73,88869047	0,857067209	-0,222519754	0,8770227	1	conserved Plasmodium membrane protein, unknown function
PFIT_0808100	754,021881	759,4106809	340,7897908	895,6161481	567,7257158	221,5552041	617,750283	574,7061518	589,8532368	596,2282174	1,01080774	0,015508617	0,8770808	1	conserved Plasmodium protein, unknown function
PFIT_0725400	27706,18552	11391,89042	15872,32107	16503,31227	10222,40245	14349,96014	13703,00819	14858,95588	16007,67865	14280,98203	0,892133229	-0,164668921	0,8771777	1	ubiquitin carboxyl-terminal hydrolase, putative
PFIT_1232900	800,9106366	342,465009	351,7594193	348,9576582	327,2771774	296,5431193	388,7043766	448,2968622	411,3188366	418,5006194	1,017460379	0,024972615	0,8772135	1	conserved protein, unknown function
PFIT_bin04300	101,3810932	89,81491707	66,54907932	61,50692537	156,9594626	88,62208163	97,00767803	78,84290745	94,13892654	87,92529274	0,933995064	-0,098513169	0,8774776	1	erythrocyte membrane protein 1, PfEMP1 (VAR)
PFIT_0507700	966,9221768	907,6418042	1390,948889	1301,68738	988,5106582	1237,300601	1026,664593	1382,683055	1132,168585	1204,673824	1,064041027	0,089553779	0,8774853	1	nuclear protein localization protein 4, putative
PFIT_0622200	106,4501479	88,35451192	136,0233929	80,335576	91,83798345	68,17083202	99,02867133	108,8162442	95,19540736	103,9224577	1,09167512	0,126543577	0,877886	1	EGF-like membrane protein, putative
PFIT_1444200	631,0973055	319,8287291	670,6099531	572,390979	699,6384557	262,4577033	648,0651824	491,9536787	526,0038543	570,0094306	1,083660178	0,115912417	0,877889	1	serine/threonine protein kinase
PFIT_1317400	10,13810932	10,95303867	8,044394203	10,66956869	10,0186891	6,817083202	8,7576376	5,864348488	9,440147198	7,310993044	0,774457526	-0,368741976	0,8780434	1	apicoplast glycerol-3-phosphate acyltransferase, putative
PFIT_1476600	13,93990032	14,60405156	10,2383199	15,69054219	33,39563034	20,45124961	16,84161077	16,28985691	18,05328232	16,56573384	0,917602326	-0,124059047	0,8781888	1	LCCL domain-containing protein
PFIT_1327500	411,8606913	528,6666663	311,5374482	385,9873378	1511,152273	504,464157	400,8303363	473,0574447	608,9447623	436,9438905	0,717542736	-0,478863336	0,8782799	1	conserved Plasmodium protein, unknown function
PFIT_0934400	1728,54764	1155,180478	1209,584365	1375,119117	604,4609092	927,1233155	993,6550354	1239,332314	1166,669304	1116,493675	0,956992415	-0,063420605	0,8783146	1	conserved Plasmodium protein, unknown function
PFIT_0400100	16,47442765	4,381215467	8,775702767	6,276216875	0	0	4,715651015	4,561159935	5,984593794	4,638405475	0,775057696	-0,367624386	0,8783359	1	rifin (RIF)
PFIT_0608300	501,8364116	262,872928	167,4696611	164,4368821	265,4952612	504,464157	213,5516246	233,9223452	311,0958835	223,7369849	0,719189796	-0,475555544	0,8785144	1	mitochondrial cardiolipin synthase, putative
[illegible]															

PFIT_0722700	172,3478585	154,0727439	217,1986435	278,6640292	140,2616474	143,1587472	216,2462823	202,64582	184,283945	209,4460511
PFIT_0407200	181,2187042	124,1344382	110,4275932	160,671152	101,8566725	34,08541601	134,7328862	108,1646499	118,7323294	121,448768
PFIT_1464250	2049,165347	946,3425409	1077,948823	881,1808492	268,8348243	245,4149953	951,8878407	877,0458961	911,48123	914,4668684
PFIT_1135400	6547,95136	10652,92541	13038,50039	15220,45354	8487,499452	10893,69896	11132,97838	12939,35914	10806,83818	12036,16876
PFIT_1121200	3780,247514	2345,41068	1785,124205	1902,321335	1377,569752	1796,301424	1942,174554	2030,367765	2164,495818	1986,27116
PFIT_1346900	14919,49513	9513,079184	10447,47414	12287,5774	8090,091451	4546,994496	11341,81436	10959,16414	9967,451968	11150,48925
PFIT_0215500	2766,436582	962,4069976	1025,294607	885,5742011	624,4982874	586,2691554	964,0138005	1077,736933	1141,746638	1020,875367
PFIT_1468200	228,1074598	382,6261508	403,6823273	349,5852799	220,4111603	320,4029105	357,0421483	342,7385894	317,4692148	349,8903689
PFIT_1345100	8,870845659	49,65377529	48,99767378	52,09260006	212,0622527	109,0733312	49,17750345	54,08232495	80,12507979	51,6299142
PFIT_0312900	1094,915807	979,9318594	1205,927822	1118,421847	993,5200027	1192,98956	1114,240969	1117,484184	1097,617816	1115,862576
PFIT_1150200	1,267263666	4,381215467	3,65654282	1,882865062	6,679126069	6,817083202	2,694657723	1,303188553	4,114016048	1,998923138
PFIT_1029600	485,3619839	1272,012891	404,4136359	818,4186805	781,4577501	323,8114521	687,8113838	722,6180526	680,9127322	705,2147182
PFIT_0515200	124,1918392	83,24309387	121,3972216	98,53660494	160,2990257	187,4697881	107,1126445	95,78435864	129,1895956	101,4485016
PFIT_0212800	22,81074598	48,19337014	47,53505666	48,32686994	61,78191614	47,71958241	49,85116788	44,3084108	46,06125688	47,07978934
PFIT_1430600	3520,458463	3088,026702	2069,603236	1651,27266	1918,578963	2757,510155	2181,999091	2193,917929	2500,908363	2187,95851
PFIT_1317900	348,497508	1004,028545	565,3015199	945,1982614	786,4670946	334,0370769	714,7579611	839,9050224	663,9216676	777,3314917
PFIT_1472700	160,9424855	106,6095764	54,8481423	42,67827475	70,13082372	20,45124961	88,25004043	51,47594784	75,94342538	69,86299414
PFIT_1208000	207,8312412	143,8499078	139,6799357	143,0977447	397,4080011	347,6712433	154,2691546	173,9756718	229,9230123	164,1224132
PFIT_1253400	373,8427813	170,1372006	171,8575125	178,8721809	145,270992	132,9331224	185,2577185	170,7177004	195,4856316	177,9877094
PFIT_0922000	3255,600357	2817,121545	3889,830252	4068,8714	1751,600812	4216,36596	3108,287684	3863,302465	3333,231721	3485,795074
PFIT_0108700	73,5012926	116,8324125	112,6215188	97,28136156	128,5731768	136,341664	101,723329	91,2231987	110,8585711	96,47326388
PFIT_0420700	8860,70755	6744,151009	9371,719247	9913,284554	5349,979981	8115,737552	8971,862889	8250,486729	8059,263315	8611,174809
PFIT_1418200	21592,9056	16656,5755	18753,67681	19813,38905	11922,24003	13330,8062	17380,54231	15919,75136	16978,26553	16650,14684
PFIT_0528600	8036,986167	3594,787291	5826,335329	6246,718656	3603,388514	6363,747169	5085,492788	6128,24417	5611,993854	5606,868479
PFIT_0606300	610,8210868	1184,388581	936,0749618	812,7700853	1041,943667	1158,904144	910,7943104	994,9844602	957,4837544	952,8893853
PFIT_1328500	277,5307428	124,1344382	159,4252669	151,8844484	168,6479332	119,298956	141,4695305	190,917123	166,8202976	166,1933267
PFIT_1363600	201,4949228	144,5801104	110,4275932	114,8547688	323,9376143	61,35374882	147,5325103	155,7310321	159,4414597	151,6317712
PFIT_0322200	3028,760161	2568,122466	3685,063854	3761,964395	2302,628712	4414,061373	3034,184596	3595,497218	3293,433494	3314,840907
PFIT_0629800	2097,321367	2045,29742	2691,215515	2989,989719	1968,672409	2437,107245	2194,798715	2352,255338	2371,600613	2273,527027
PFIT_0904700	286,4015884	202,2661141	205,4977065	169,4578556	203,7133451	381,7566593	185,2577185	242,3930708	241,5155448	213,8253947
PFIT_1428400	254,7199968	289,1602208	403,6823273	425,5275041	1242,317449	446,5189497	360,4104705	422,8846854	510,3210746	391,6475779
PFIT_0502100	2066,907039	1666,322283	2327,755159	2096,884058	1384,248878	1871,289339	1958,3425	2065,553856	1902,234459	2011,948178
PFIT_1014700.1	1464,956797	1381,543277	1778,542428	1830,144841	1153,819028	1598,606011	1392,464378	1542,975247	1534,602064	1467,719813
PFIT_1122200	1,267263666	43,81215467	29,25234256	56,48595187	63,45169765	3,408541601	32,33589268	36,48927948	32,94632534	34,41258608
PFIT_1455200	932,7060579	947,802946	1156,930148	1210,682235	858,2676998	1656,551218	1035,42223	1167,005349	1127,156718	1101,21379
PFIT_0420400	26841,9117	7604,329645	7411,812296	7357,609042	4775,575139	6448,960709	7881,87384	8872,759263	10073,36642	8377,316551
PFIT_0618500	139,3990032	170,8674032	187,9463009	157,5330436	136,9220844	177,2441633	150,2271681	149,2150893	161,6519998	149,7211287
PFIT_0909500	823,7213826	1488,883056	1966,488728	2173,453904	1041,943667	2065,57621	1439,620889	1991,272109	1593,344491	1715,446499
PFIT_1016700	31,68159164	50,38397787	26,3271083	29,49821931	76,80994979	13,6341664	45,13551686	31,92811955	38,05583555	38,5318182
PFIT_0936000	702,0640707	329,3213626	205,4977065	181,3826677	505,9437997	354,4883265	303,1489939	287,3530759	379,782989	295,2510349
PFIT_0904900	16,47442765	54,76519334	51,19159948	46,44400487	303,9002361	40,90249921	55,24048332	45,61159935	85,61299345	50,42604134
PFIT_1204300	546,1906399	492,1565375	519,960389	455,0257234	551,0279007	548,7751978	449,3341753	471,1026619	518,8560647	460,2184186
PFIT_0104100	3064,243543	1834,999078	2211,477097	1926,798581	1646,404576	2242,820373	1830,346258	2027,761388	2154,457208	1929,053823
PFIT_1326000	290,2033794	278,2071821	416,8458814	433,6865861	435,812976	821,4585259	314,6012892	393,562943	446,0357552	354,0821161
PFIT_0708700	54,49233762	29,20810311	39,49066245	33,26394944	16,69781517	17,04270801	49,17750345	21,50261112	31,69926263	35,34005729
PFIT_1433500	504,3709389	795,9208098	350,2968021	586,1986561	901,6820193	276,0918697	549,7101755	531,0493353	569,093516	540,3797554
PFIT_0518800	282,5997974	278,2071821	363,4603563	293,0993281	317,2584883	351,0797849	335,4848865	293,8690187	314,2841562	314,6769526
PFIT_1338700	60,82865595	17,52486187	22,67056548	18,20102894	28,38628579	27,26833281	27,62024166	24,76058251	29,14662181	26,19041208
PFIT_0613100	126,7263666	382,6261508	193,7967694	252,3039184	656,2241363	194,2868713	253,9714904	269,7600305	300,9940354	261,8657604
PFIT_1103900	148179,8732	19822,07918	19543,49006	16625,6985	14393,51668	32370,91959	25031,34925	26630,65808	41822,59619	25831,00367
PFIT_1313100	46,88875563	217,6003682	280,09118	222,8056991	250,4672276	327,2199937	225,0039199	220,2388654	224,1788707	222,6213927
PFIT_0628400	3144,081154	2878,458562	3342,080137	3804,64267	2379,438662	1602,014552	3250,430879	3271,654862	2858,452623	3261,04287
PFIT_0930100	0	6,5718232	4,387851384	9,414325312	41,74453793	13,6341664	8,7576376	5,212754212	12,62545071	6,985195906
PFIT_1130000	1182,357	2054,059851	1878,731701	1597,297195	1990,379569	3722,127428	1593,890043	1752,137009	2070,825457	1673,013526
PFIT_1017800	6476,984595	4329,371084	4823,711288	5643,574214	4767,226232	4874,214489	5053,83056	4416,506006	5152,51365	4735,168283
PFIT_1407700	1,267263666	31,39871085	18,2827141	15,0629205	85,15885738	57,94520722	22,90459065	20,19942257	34,85261228	21,55200661
PFIT_1109200	3919,646518	3326,072742	4124,580301	4128,49546	2963,862193	3224,480355	3575,137134	4110,90829	3614,522928	3843,022712
PFIT_0108900	2270,936489	1539,997237	2075,453704	1735,373966	1188,88444	2126,929959	1584,458741	1724,118456	1822,929299	1654,288598
PFIT_1015900	11988,31428	8569,657453	12672,1148	12723,77447	10082,1408	17073,38488	10889,11186	12540,58344	12184,89778	11714,84765
PFIT_1357700	329,4885531	364,3710863	140,4112443	160,671152	395,7382196	119,298956	266,7711146	232,6191567	251,6632019	249,6951356

PFIT_1302700	10206,54156	6479,817676	6374,816752	6270,56828	5865,94247	4942,385322	6760,222563	6113,257502	6690,01201	6436,740032	0,962141775	-0,055678599	0,9711278	1 sodium/hydrogen exchanger, Na , H antiporter
PFIT_1416900	383,9808907	658,6427252	776,6496949	908,7962035	495,9251106	644,2143626	604,2769944	738,9079095	644,7014979	671,592452	1,041710705	0,05895468	0,9714272	1 conserved Plasmodium protein, unknown function
PFIT_1342900	9480,399482	7847,487104	7971,994656	8548,207384	5468,534469	4615,165328	8249,020955	6951,859336	7321,964737	7600,440145	1,03803288	0,053852143	0,9715153	1 kelch protein, putative
PFIT_1134600	3024,95837	1874,430017	1521,853122	1513,82351	1719,874963	1762,216008	1989,331064	1473,906253	1902,859332	1731,618659	0,910008759	-0,136047663	0,9719475	1 conserved Plasmodium protein, unknown function
PFIT_0101200	1449,749633	1664,131675	1227,867079	1977,008316	791,4764392	330,6285353	1364,170472	1507,137561	1240,143613	1435,654017	1,157651422	0,211200912	0,9720107	1 merozoite-associated tryptophan-rich antigen, putative
PFIT_1149500	359,902881	528,6666663	476,8131837	455,6533451	328,9469589	136,341664	473,5860948	405,9432342	381,0541165	439,7646645	1,154074042	0,206735786	0,9720896	1 serine/threonine protein kinase, FIKK family
PFIT_1314700	195,1586045	657,9125226	583,584234	631,3874176	459,1899172	623,763113	462,8074639	591,647603	525,1659682	527,2275335	1,00392555	0,005652285	0,9721197	1 conserved Plasmodium protein, unknown function
PFIT_0406000	12,67263666	35,77992631	56,31075942	46,44400487	38,4049749	78,39645682	45,13551686	33,2313081	44,6681265	39,18341248	0,877211908	-0,189002698	0,9721203	1 conserved Plasmodium protein, unknown function
PFIT_1116600	13742,20719	14431,72375	17185,75125	23624,30794	9133,704899	10450,58855	14041,18773	18316,31511	14761,3806	16178,75142	1,096018852	0,132272614	0,9721526	1 conserved Plasmodium protein, unknown function
PFIT_1123600	20,27621865	32,859116	19,01402266	29,49821931	165,3083702	13,6341664	36,37787926	25,41217678	46,76501887	30,89502802	0,660643976	-0,598055088	0,9722429	1 conserved Plasmodium protein, unknown function
PFIT_0413400	34,21611897	106,6095764	102,383199	294,9821931	123,5638323	57,94520722	158,3111412	135,5316095	119,9500211	146,9213754	1,224854935	0,292610895	0,9723495	1 conserved Plasmodium protein, unknown function
PFIT_0508700	4066,649103	2820,042356	4093,134032	4296,698073	2504,672276	3769,847011	3309,039684	3715,390564	3591,840475	3512,215124	0,977831602	-0,032342064	0,9725425	1 Pre-mRNA-processing ATP-dependent RNA helicase prp5, putative
PFIT_1249600	45,62149196	61,33701654	19,01402266	17,57340725	116,8847062	30,67687441	45,13551686	30,62493099	48,51791984	37,88022393	0,780747074	-0,357072838	0,9726024	1 conserved Plasmodium protein, unknown function
PFIT_1000700.2	5,069054662	5,841620623	1,462617128	6,903838562	5,009344552	0	3,368322154	3,257971382	4,047745921	3,313146768	0,818516486	-0,28891662	0,9727837	1 acyl-CoA binding protein, isoform 1, ACBP1
PFIT_1034100	6,336318328	60,60681396	35,10281107	56,48595187	333,9563034	68,17083202	61,97712763	51,47594784	93,44317178	56,72653774	0,607069908	-0,720065432	0,9728102	1 ADP-ribosylation factor, putative
PFIT_1116500	3089,588817	3346,518414	6130,559692	6306,342716	3666,840212	10978,9125	4584,286451	5049,855643	5586,460391	4817,071047	0,862276058	-0,213778271	0,9729743	1 cysteine proteinase falcipain 2a
PFIT_1407300	255,9872604	301,5736646	383,9369961	585,5710344	515,9624888	316,9943689	374,5574235	412,459177	393,3376355	393,5083003	1,000433889	0,000625833	0,9730143	1 glycerophosphodiester phosphodiesterase, putative (GDPD)
PFIT_0210100	383,9808907	1374,971454	1665,1896	1694,578556	956,7848094	933,9403987	1267,162794	1411,353203	1168,240952	1339,257999	1,146388506	0,197096049	0,9731596	1 conserved Plasmodium protein, unknown function
PFIT_1409900	136,8644759	416,2154694	403,6823273	319,4594389	197,034219	160,2014552	363,1051282	276,9275675	272,2428976	320,0163478	1,175480979	0,233251194	0,9733872	1 tRNA-dihydrouridine synthase, putative
PFIT_1355400	51,95781029	216,8701656	87,75702767	175,1064508	572,7350604	122,7074976	151,5744969	174,6272661	204,5223354	163,1008815	0,797472223	-0,326493826	0,9734591	1 ubiquitin conjugating enzyme, putative
PFIT_1300500	16,47442765	69,36924489	103,1145075	73,43173744	98,51710952	344,2627017	76,79774511	69,0689933	117,5282881	72,93336921	0,620560125	-0,688357097	0,9735056	1 Plasmodium exported protein (PHISTa-like), unknown function, pseudogene
PFIT_1477700	8,870845659	28,47790054	18,2827141	6,276216875	18,36759669	27,26833281	19,53626849	9,773914147	17,92393444	14,65509132	0,81762692	-0,290485397	0,9735096	1 Plasmodium exported protein, unknown function
PFIT_0112400	55,75960129	13,1436464	17,55140553	22,59438075	8,348907586	3,408541601	22,90459065	15,63826264	20,13441386	19,27142664	0,957138697	-0,063200097	0,9736521	1 RESA-like protein with DnaJ domain, putative
PFIT_0613800	271,1944244	199,3453037	142,60517	153,7673134	379,0404044	541,9581146	175,152752	222,1936483	281,3184551	198,6732001	0,706221709	-0,501806925	0,9737057	1 endonuclease III homologue, putative
PFIT_1200500	2099,855894	3064,660219	3121,224951	3424,931549	2300,958931	1479,307055	3108,961348	2517,760284	2581,8231	2813,360816	1,089679931	0,123904437	0,9737113	1 serine/threonine protein kinase, FIKK family
PFIT_1456300	225,5729325	540,3499076	413,1893386	383,4768511	325,6073959	248,8235369	455,3971552	305,5977157	356,1699937	380,4974354	1,068302895	0,095320751	0,9737257	1 protein phosphatase, putative
PFIT_1110000	87,44119292	105,1491712	55,57945086	59,62406031	53,43300855	51,12812402	78,8187384	56,68870205	68,72583464	67,75372023	0,985855182	-0,020552358	0,9737438	1 pre-mRNA splicing factor, putative
PFIT_0600400	74,76855627	107,3397789	90,68226193	102,3023351	237,1089754	245,4149953	99,70233576	115,3321869	142,9361505	107,5172613	0,7522048	-0,410802544	0,9738742	1 Tetratricopeptide repeat protein, putative
PFIT_0701500	201,4949228	112,451197	98,72665613	120,503364	101,8566725	71,57937362	112,5019599	114,6805927	117,7686977	113,5912763	0,964528593	-0,052104088	0,9739205	1 chitinase precursor fragment, truncated
PFIT_0928600	344,695717	482,6639039	653,7898562	590,5920079	424,1245054	1012,336856	431,1452357	605,9826771	584,7004743	518,5639564	0,886888209	-0,173175829	0,974097	1 conserved Plasmodium membrane protein, unknown function
PFIT_1428200	129,2608939	172,3278084	171,126204	223,4333207	143,6012105	54,53666562	132,0382284	198,7362543	149,0476838	165,3872414	1,109626377	0,150073987	0,9741611	1 selenoprotein
PFIT_0102000	149,5371125	151,1519336	141,1425528	199,5836966	170,3177148	139,7502056	144,8378526	164,2017577	158,580536	154,5198051	0,974393258	-0,037423944	0,9742092	1 conserved Plasmodium protein, unknown function
PFIT_1123800	475,2238746	243,887661	293,9860427	261,090622	217,0715972	221,5552041	258,6871414	284,7466988	285,4691669	271,7169201	0,951825807	-0,071230524	0,9742398	1 dynein heavy chain, putative
PFIT_0305400	20726,09725	11674,47881	10857,00694	11903,47292	8245,381132	12233,25581	12402,83584	11230,22735	12606,61548	11816,53159	0,937327835	-0,093374369	0,9742967	1 conserved Plasmodium protein, unknown function
PFIT_0420200	133,0626849	192,7734805	169,6635868	114,8547688	141,931429	303,3602025	143,4905238	153,124655	175,9410254	148,3075894	0,842939212	-0,246499499	0,9745952	1 steroid dehydrogenase, putative
PFIT_0817400	2651,115588	2906,936462	2888,668828	3533,510101	1626,367198	616,9460298	2721,6043	2929,567867	2370,590701	2825,586084	1,191933336	0,25330355	0,9747188	1 conserved Plasmodium protein, unknown function
PFIT_1202300	5268,015058	3316,580108	4539,963565	5372,441645	2496,323368	3793,706802	4367,366505	3974,073492	4131,171758	4170,719998	1,009573129	0,013745417	0,9749239	1 conserved protein, unknown function
PFIT_0917300	5,069054662	12,41344382	10,96962846	25,73248919	65,12147917	20,45124961	14,14695305	20,85101685	23,29289082	17,49898495	0,751258617	-0,412618461	0,9750312	1 conserved Plasmodium membrane protein, unknown function
PFIT_0409000	292,7379067	395,0395946	620,8809708	687,2457478	348,9843371	446,5189497	383,3150611	594,9055744	465,2345845	489,1103178	1,051319773	0,072201551	0,9750929	1 exosome complex component RRP4, putative
PFIT_0204300	114,0537299	53,30478818	58,50468512	63,38979044	83,48907586	37,49395761	63,99812092	61,90145626	68,37267118	62,94978859	0,920686402	-0,119218257	0,9751077	1 conserved Plasmodium protein, unknown function
PFIT_0303800	0	12,41344382	0,731308564	4,393351812	26,71650428	6,817083202	6,736644308	4,561159835	8,511948613	5,648902122	0,663643823	-0,591518938	0,9751828	1 conserved Plasmodium protein, unknown function
PFIT_0814300	6570,762106	5797,078265	5445,323567	4707,162656	4298,017625	7079,540905	5113,11303	5198,419138	5649,647521	5155,766084	0,912581903	-0,131974051	0,9752882	1 conserved protein, unknown function
PFIT_0213500	17949,52256	12051,26334	15247,78356	17663,15715	11468,05946	15246,40658	13351,35535	15548,99422	14937,69878	14450,17479	0,967362845	-0,047870968	0,9756828	1 protein transport protein sec31
PFIT_0929800	44,3542283	65,718232	35,10281107	51,46497837	60,11213462	40,90249921	53,89315446	40,39884514	49,60914726	47,1459998	0,950348926	-0,073470791	0,9757505	1 conserved Plasmodium protein, unknown function
PFIT_0515000	1700,667839	486,3149168	640,626302	556,7004368	566,0559343	426,0677001	725,536592	600,7699229	729,4055216	663,1532574	0,909169506	-0,137378799	0,9760036	1 conserved Plasmodium protein, unknown function
PFIT_0915400	1411,731723	124,8646408	149,186947	135,5662845	125,2336138	180,6527049	216,9199467	228,0579968	354,5393191	222,4889717	0,627543857	-0,672211807	0,9764353	1 conserved Plasmodium protein, unknown function
PFIT_1210400	0	1,460405156	1,462617128	3,138108437	6,679126069	0	2,020993292	0	2,123376132	1,010496646	0,475891497	-1,071295417	0,9770137	1 small nucleolar RNA snoR14
PFIT_1339800	1908,49908	2383,381214	2155,897647	1950,648205	1850,117921	3449,4441	1940,827225	2125,50053	2282,998028	2033,163779	0,890567514	-0,167203109	0,977027	1 RNA lariat debranching enzyme, putative
PFIT_0803900	17678,32813	7543,722831	7253,118337	6085,419882	4705,444315	5688,855932	7277,596846	8037,4154	8159,148239	7657,506123	0,938517833	-0,091543937	0,97717	1 unspecified product
PFIT_1246600	716,0039711	4188,441986	1486,75031	3709,244173	2805,232949	657,848529	2958,73418	2296,21823	2260,586986	2627,476205	1,162298209	0,216980266	0,9774865	1 myosin A tail domain interacting protein
PFIT_1250000	6613,849071	4477,602207	5609,136685	6222,869031	4301,357188	5286,648023	4952,780895	5563,311932	5418,577034	5258,046414	0,970374026	-0,04338716	0,9775008	1 conserved Plasmodium protein, unknown function
PFIT_1425800	7,603581993	21,90607733	3,65654282	8,786703625	58,4423531	88,62208163	18,18893963	11,72869698	31,50289008	14,9588183	0,474839555	-1,074487976	0,9775307	1 unspecified product
PFIT_bin08000	35,48338264	12,41344382	9,507011331	10,66956869	11,68847062	74,98791522	8,083973169	19,54782829	25,79163205	13,81590073	0,535673768	-0,900573445	0,9775527	1 nfin
PFIT_0415300	1,267263666	37,24033147	33,64019394	28,87059762	38,4049749	37,49395761	27,62024166	26,71536533	29,48621987	27,1678035	0,921372886	-0,118142952	0,977572	1 CDGSH iron-sulfur domain-containing protein, putative
PFIT_0317400	177,4169132	289,8904234	509,7220691	431,803721	415,7775578	477,1958241	384,66239	347,9513436	383,6340914	366,3068668	0,954833981	-0,066678184	0,9775755	1 conserved Plasmodium protein, unknown function
PFIT_0812400	16,47442765	152,6123388	119,2032959	101,6747134	108,5357986	119,298956	103,7443223	100,9971129	102,9665884	102,3707176	0,994212969	-0,008373172	0,9776399	1 histone acetyltransferase subunit NuA4, putative
PFIT_1016600	78,57034727	437,3913441	166,7383526	281,8021377	1142,130558	160,2014552	303,1489939	289,3078587	377,8056991	296,2284263	0,784076119	-0,350934375	0,978196	1 conserved Plasmodium protein, unknown function
PFIT_1220300	133,0626849	269,4447512	304,2243626	261,7182437	252,1370091	323,8114521	229,7195709	261,2893049	257,3997506	245,5044379	0,953786619	-0,068261552	0,9782118	1 conserved Plasmodium protein, unknown function
PFIT_0904800	3680,133685	3272,037751	3686,526471	3502,129016	2958,852848	3878,920342	3211,358342	3424,779517	3496,433352	3318,068929	0,948986752	-0,075540148	0,9784231	1 ubiquitin specific protease, putative
PFIT_0709900	1075,906852	1152,98987	1269,551667	1444,157503	1357,532373	1956,502879	1186,996727	1283,640725	1376,106857	1235,318726	0,897690989	-0,155709182	0,9786387	1 AAA family ATPase, CDC48 subfamily

[illegible]

PFIT_1421400	5527,804109	4614,150089	5473,113293	4764,903851	4182,802701	6244,448213	5489,017782	4530,535004	5134,537043	5009,776393	0,975701675	-0,03548799	1	1	glycine--tRNA ligase (GlyRS)
PFIT_1421700	36701,22302	31728,76241	44528,64715	57075,28864	28543,24526	71391,90383	41388,5953	43275,63387	44994,84505	42332,11458	0,940821433	-0,088007169	1	1	surface protein, Pf113
PFIT_1422600	2329,230617	1292,458563	1352,189535	1099,593196	1192,224003	2290,539956	1278,61509	1507,789156	1592,705978	1393,202123	0,874739055	-0,193075387	1	1	conserved Plasmodium protein, unknown function
PFIT_1422900	16,47442765	116,8324125	87,02571911	123,0138507	497,5948921	132,9331224	121,933262	117,2869698	162,3124041	119,6101159	0,736912971	-0,440433848	1	1	Ctr copper transporter domain containing protein, putative
PFIT_1423400	3525,527518	3131,108654	3417,404919	3369,70084	2491,314024	2829,089529	3194,516731	3130,258904	3127,357581	3162,387817	1,011201225	0,016070117	1	1	conserved Plasmodium protein, unknown function
PFIT_1423500	62,09591961	134,3572743	104,5771246	142,4701231	527,6509594	119,298956	144,8378526	170,0661062	181,7417262	157,4519794	0,866350192	-0,206977793	1	1	ERAD-associated E3 ubiquitin-protein ligase HRD1 (HRD1)
PFIT_1423700	5447,966498	2557,89963	3256,517035	2413,205388	2432,871671	4567,445745	3422,215308	2719,102916	3445,984328	3070,659112	0,891083307	-0,166367779	1	1	conserved Plasmodium protein, unknown function
PFIT_1424200	852,8684469	541,8103127	687,4300501	553,5623284	480,897077	1653,142677	635,2655582	624,2273168	794,9518153	629,7464375	0,792181898	-0,336096361	1	1	peptidyl-prolyl cis-trans isomerase
PFIT_1425400	39746,45761	13346,64272	23613,95353	22065,29567	11072,32124	41383,10358	17662,13405	26966,22913	25204,62906	22314,18159	0,885320769	-0,175727828	1	1	60S ribosomal protein L7-3, putative
PFIT_1426700	690,6586977	509,6813993	410,9954129	262,9734871	607,8004723	770,3304018	470,2177727	451,5548336	542,0733119	460,8863031	0,850228729	-0,234077086	1	1	conserved Plasmodium protein, unknown function
PFIT_1427100	11945,22731	3761,273478	5187,902953	4862,185213	4470,005122	10651,6925	4986,464117	6158,217507	6813,047763	5572,340812	0,817892521	-0,290016823	1	1	basic transcription factor 3b, putative
PFIT_1427600	1568,872418	478,2826885	601,8669481	745,6145647	562,7163713	156,7929136	602,9296656	789,7322631	685,690984	696,3309643	1,015517165	0,022214626	1	1	conserved Plasmodium protein, unknown function
PFIT_1427800	5810,403907	4387,057088	4752,043049	5058,003179	3531,587909	3381,273268	4710,2617	4504,471233	4486,728067	4607,366467	1,026887834	0,038278606	1	1	conserved Plasmodium protein, unknown function
PFIT_1431100	752,7546174	720,7099443	718,8763184	667,7894755	973,4826245	668,0741538	747,0938537	678,3096418	750,281189	712,7017478	0,949912857	-0,074132925	1	1	ATP-dependent DNA helicase, putative
PFIT_1432600	794,5743183	560,7955798	380,2804533	449,3771282	1006,878255	630,5801962	635,2655582	451,5548336	637,0809884	543,4101959	0,852968784	-0,229435151	1	1	conserved Plasmodium protein, unknown function
PFIT_1432800	53,22507395	78,8618784	49,72898235	49,58211331	260,4859167	112,4818728	78,14507397	72,32696469	100,7276396	75,23601933	0,746925269	-0,420964188	1	1	ATP-specific succinyl-CoA synthetase beta subunit, putative
PFIT_1433100	100,1138296	79,59208098	47,53505666	51,46497837	158,6292441	57,94520722	75,45041625	68,41739903	82,54673282	71,93390764	0,871432523	-0,198539138	1	1	conserved Plasmodium membrane protein, unknown function
PFIT_1435200	342,1611897	441,042357	436,5912127	325,7356558	320,5980513	296,5431193	366,4734503	376,6214918	360,4452643	371,5474711	1,030801367	0,043766355	1	1	CCR4-associated factor 16, putative
PFIT_1435400	3625,641347	2004,406076	1845,091507	2057,343892	2621,556982	2573,448909	2209,619333	2231,710397	2454,581452	2220,664865	0,904702047	-0,144485359	1	1	calmodulin
PFIT_1435500	6213,393752	3849,62799	4114,341981	3612,590433	3054,030395	5893,368428	3931,505618	4164,990615	4456,225497	4048,248117	0,90844777	-0,138524523	1	1	Hsp70/Hsp90 organizing protein
PFIT_1436600	262,3235788	903,9907913	1219,822685	1055,032057	1188,88444	2191,692249	827,9335854	1122,045344	1136,957634	974,9894648	0,857542477	-0,221719961	1	1	conserved protein, unknown function
PFIT_1437100	7,603581993	24,09668507	19,01402266	21,96675906	25,04672276	23,85979121	22,90459065	17,59304546	20,26459379	20,24881806	0,999221512	-0,001123558	1	1	conserved Plasmodium protein, unknown function
PFIT_1437200	3482,440553	3066,850827	2757,033286	2041,025728	2745,120814	6898,888201	3093,467066	2554,901158	3498,559901	2824,184112	0,807241891	-0,308927051	1	1	glucose-6-phosphate isomerase
PFIT_1438700	160,9424855	589,2734803	495,8272064	458,7914536	671,2521699	310,1772857	492,4486989	438,5229481	447,7106802	465,4858235	1,039702299	0,056170496	1	1	conserved Plasmodium membrane protein, unknown function
PFIT_1444300	675,4515338	468,790055	508,2594519	349,5852799	758,0808088	1305,471433	624,4869273	458,0707764	677,6064271	541,2788518	0,798810091	-0,324075537	1	1	conserved Plasmodium protein, unknown function
PFIT_1445200	2,534527331	1,460405156	2,925234256	1,882865062	3,339563034	10,2256248	3,368322154	1,954782829	3,728036607	2,661552492	0,713928744	-0,486148007	1	1	conserved Plasmodium protein, unknown function
PFIT_1445300	1234,31481	604,6077344	538,9744116	542,265138	709,6571448	398,7993673	654,1281623	628,7884768	671,4364344	641,4583195	0,955352267	-0,065895299	1	1	conserved Plasmodium protein, unknown function
PFIT_1445800	1,267263666	9,492633512	10,96962846	18,82865062	15,02803365	20,45124961	8,7576376	14,33507408	12,67290992	11,54635584	0,911105335	-0,134310238	1	1	conserved Plasmodium protein, unknown function
PFIT_1446100	31,68159164	21,17587476	37,29673676	33,26394944	46,75388248	78,39645682	29,64123495	35,18609093	41,42808198	32,41366294	0,782408004	-0,354006965	1	1	conserved Plasmodium protein, unknown function
PFIT_1446600	4549,476559	5579,477897	5917,748899	6937,102512	4423,251239	10631,24125	5635,202964	6022,034303	6339,716393	5828,618633	0,919381605	-0,121264294	1	1	protein serine/threonine kinase-1
PFIT_1447000	93,77751125	135,8176795	187,9463009	158,7882869	88,49842041	146,5672888	121,933262	160,292192	135,2325813	141,112727	1,043481723	0,061405332	1	1	conserved Plasmodium membrane protein, unknown function
PFIT_1447200	15,20716399	83,24309387	55,57945086	30,125841	81,81929434	98,84770643	54,56681889	47,56638218	60,80375842	51,06660054	0,839859276	-0,251780479	1	1	U5 spliceosomal RNA
PFIT_1450400	188,8222862	595,1151009	190,1402266	382,2216077	2309,307838	167,0185385	541,6262024	417,0203369	638,770933	479,3232696	0,750383658	-0,414299685	1	1	conserved Plasmodium protein, unknown function
PFIT_1453400	158,4079582	71,55985263	87,75702767	96,02611819	65,12147917	54,53666562	85,55538271	95,13276436	88,90151691	90,34407354	1,016226457	0,023221929	1	1	aminomethyltransferase, putative
PFIT_1453700	2,534527331	43,81215467	29,98365112	29,49821931	150,2803365	27,26833281	37,72520812	39,09565659	47,22953697	38,41043236	0,813271415	-0,298191188	1	1	SNARE protein, putative
PFIT_1454900	5524,002318	2935,414363	2413,318261	1867,17452	1524,510525	5743,392598	4089,143095	1600,315543	3334,635431	2844,729319	0,853085555	-0,229237659	1	1	co-chaperone p23
PFIT_1455800	449,8786013	216,8701656	206,9603236	188,2865062	248,7974461	528,3239482	271,4867656	226,7548082	306,5194985	249,1207869	0,812740423	-0,299133444	1	1	mitochondrial ribosomal protein S11 precursor, putative
PFIT_1455900	2926,111804	2703,940146	2052,783139	1486,835778	2269,233082	2457,558494	2441,359897	1959,343989	2316,077074	2200,351943	0,95003399	-0,073948964	1	1	6-phosphogluconate dehydrogenase, decarboxylating, putative
PFIT_1456100	7,603581993	7,302025778	3,65654282	3,765730125	8,348907586	3,408541601	4,715651015	4,561159935	5,680888317	4,638405475	0,816492988	-0,292487599	1	1	conserved Plasmodium protein, unknown function
PFIT_1457100	363,704672	365,1012889	464,3809381	284,3126244	242,11832	374,9395761	355,021155	344,041778	349,0929033	349,5314665	1,001256294	0,001811311	1	1	dfg10 like protein, putative
PFIT_1457800	7,603581993	15,33425413	1,462617128	2,51048675	20,03737821	3,408541601	6,062979877	7,167537041	8,392809969	6,615258459	0,788205438	-0,343356392	1	1	mitochondrial ribosomal protein L28 precursor, putative
PFIT_1460000	523,3798939	866,7504599	909,016545	845,4064131	783,1275316	760,104777	821,1969411	789,7322631	781,2976034	805,4646021	1,030931874	0,043948999	1	1	conserved Plasmodium protein, unknown function
PFIT_1460100	235,7110418	270,9051564	298,3738941	264,8563521	193,694656	235,1893705	212,8779601	304,2945271	249,7884118	258,5862436	1,035221137	0,049938979	1	1	conserved protein, unknown function
PFIT_1461700	530,9834759	317,6381214	375,1612933	407,9540969	741,3829936	552,1837394	447,9868465	421,5814969	487,5506201	434,7841717	0,891772369	-0,165252595	1	1	conserved Plasmodium protein, unknown function
PFIT_1462900	8,870845659	1,460405156	2,193925692	3,138108437	13,35825214	3,408541601	4,041986585	4,561159935	5,405013114	4,30157326	0,795848811	-0,32943371	1	1	conserved Plasmodium protein, unknown function
PFIT_1465000	438,4732283	1256,678636	634,0445249	1135,995254	3628,435237	852,1354003	1154,660834	1095,981573	1324,293714	1125,321204	0,849751979	-0,234886278	1	1	conserved Plasmodium membrane protein, unknown function
PFIT_1466200	98,84656592	160,6445671	133,8294672	128,6624459	365,6821523	112,4818728	144,8378526	162,2469748	166,6911785	153,5424137	0,921119012	-0,118540525	1	1	conserved Plasmodium protein, unknown function
PFIT_1468400	135,5972122	186,9318599	217,1986435	181,3826677	203,7133451	160,2014552	179,868403	185,7043688	180,8375306	182,7863859	1,01077683	0,015464499	1	1	1-deoxy-D-xylulose 5-phosphate reductoisomerase
PFIT_1470400	390,317209	954,3747692	632,5819078	648,9608249	604,4609092	490,8299905	771,3457732	563,6290491	620,2542684	667,4874112	1,076151258	0,105880869	1	1	small subunit rRNA processing KH domain protein, putative
PFIT_1470800	1,267263666	2,190607733	2,193925692	0	1,669781517	0	1,347328862	0	1,220263101	0,673664431	0,552064903	-0,857090209	1	1	mediator of RNA polymerase II transcription subunit 6, putative
PFIT_1472000	1985,802164	1507,868323	1566,462944	1271,561539	2110,603838	2866,583486	1524,502607	1628,334097	1884,813716	1576,418352	0,836378863	-0,257771493	1	1	20S proteasome beta 4 subunit, putative
PFIT_1474000	62,09591961	378,9751379	443,9042983	352,7233884	287,202421	746,4706106	292,370363	393,562943	378,5619626	342,966653	0,905972303	-0,14246115	1	1	dihydroorotase, putative
PFIT_1475100	411,8606913	308,8756904	162,3505012	192,6798581	310,5793622	126,1160392	246,5611817	235,2255338	252,0770237	240,8933577	0,955633933	-0,065470012	1	1	probable protein, unknown function
PFIT_bin01000	77,3030836	18,25506445	19,01402266	17,57340725	20,03737821	10,2256248	37,05154369	12,38029125	27,06809683	24,71591747	0,913101413	-0,131152993	1	1	erythrocyte membrane protein 1, PfEMP1
PFIT_bin01300	1,267263666	0	1,462617128	0	0	10,2256248	0,673664431	0	2,159250933	0,336832215	0,155994938	-2,68042888	1	1	stevor
PFIT_bin02300	354,8338264	503,8397787	759,8295979	674,6933141	297,2211101	1622,465802	586,0880548	654,2006536	702,1472382	620,1443542	0,88321127	-0,179169513	1	1	rifin (RIF)
PFIT_bin02900	1,267263666	1,460405156	1,462617128	0,627621687	1,669781517	0	0,673664431	0,651594276	1,081281526	0,662629354	0,612818529	-0,70648177	1	1	rifin (RIF)
PFIT_bin04100	2,534527331	10,22283609	11,70093702	3,765730125	3,339563034	13,6341664	5,389315446	7,819131318	7,532960001	6,604223382	0,876710268	-0,18982795	1	1	Plasmodium exported protein (PHISTb), unknown function
PFIT_bin04900	3,801790997	13,87384898	17,55140553	19,45627231	1,669781517	17,04270801	6,736644308	20,19942257	12,23263456	13,46803344	1,100992053	0,138804056	1	1	rifin (RIF)
PFIT_bin06000	6,336318328	0	0	0,627621687	0	0	0,673664431	0	1,160656669	0,336832215	0,290208314	-1,784839243	1	1	rifin
PFIT_bin06400	41,81970096	32,12891342	71,66823927	48,95449162	83,48907586	47,71958241	59,28246991	42,35362797	54,29666726	50,81804894	0,935933115	-0,095522661	1	1	unspecified product
PFIT_bin07600	62,09591961	16,79465929	13,89486271	10,66956869	36,73519338	20,45124961	23,57825508	18,89623402	26,77357555	21,23724455	0,793216599	-0,334213226	1	1	B_ITvar17 - erythrocyte membrane protein 1, PfEMP1, putative
PFIT_bin08900	155,8734309	46,0027624	48,26636522	38,28492294	16,69781517	27,26833281	63,99812092	44,3084108	55,39893823	54,15326586	0,977514508	-0,03280998	1	1	erythrocyte membrane protein 1, PfEMP1
PFIT_bin08950	5,069054662	5,841620623	1,462617128	4,393351812	31,72584883	0	4,041986585	5,864348488	8,082082175	4,953167536	0,612857854	-0,706375601	1	1	erythrocyte membrane protein 1, EMP1, exon 2
PFIT_bin09100	93,77751125	46,0027624	83,36917629	58,99643862	20,03737821	149,9758304	68,71377194	57,34029633	75,35984954	63,02703413	0,836347664	-0,257825309	1	1	BC_ITvar16 - erythrocyte membrane protein 1,, putative
PFIT_bin09400	1,267263666	2,920810311	0	1,882865062	1,669781517	3,408541601	0,673664431	2,606377106	1,85821036	1,640020768	0,882580791	-0,180199748	1	1	rifin (RIF)
PFIT_bin09700	21718,3647	6744,881211	8008,560084	5441,480031	6034,590403	14891,91825	10037,60002	7154,505156	10473,29911	8596,052587	0,820758817	-0,284969753	1	1	erythrocyte membrane protein 1, PfEMP1 (VAR)
PFIT_bin09800	8,870845659	2,190607733	5,119159948	0,627621687	6,679126069	6,817083202	3,368322154	4,561159935	5,050740716	3,964741045	0,784982098	-0,349268342	1	1	erythrocyte membrane protein 1, PfEMP1, putative
PFIT_bin10300	1,267263666	0	0,731308564	1,255243375	0	0	0	0	0,542302601	0	0	#DIV/0!	1	1	B_ITvar41 - erythrocyte membrane protein 1, PfEMP1, putative
PFIT_bin10600	3,801790997	8,762430934	8,775702767	3,765730125	0	3,408541601	5,389315446	3,909565659	4,752366071	4,649440553	0,978342258	-0,031588837	1	1	rifin (RIF)
PF_apicoplast_ge	1,267263666	0,730202578	0	0,627621687	0	0	0	0	0,437514655	0	0	#DIV/0!	1	1	unspecified product
PF_apicoplast_ge	181,2187042	104,4189686	100,1892733	58,36881694	96,847328	81,80499842	97,68134246	93,17798153	103,8080149	95,429662	0,919289923	-0,121408169	1	1	apicoplast ribosomal protein S2
Pf_M7661101900	292,7379067	2159,939225	1205,196513	1615,498224	3671,849556	1043,01373	1769,71646	1480,422196	1664,705859	1625,069328	0,976190069	-0,03476602	1	1	apocytochrome b
Pf_M7661102400	8,870845659	9,492633512	3,65654282	5,648595187	13,35825214	3,408541601	9,431302031	3,909565659	7,405901819	6,670433845	0,900691639	-0,150894826	1	1	unspecified product
PFIT_0113500	0	0,730202578	0,731308564	0	0	10,2256248	0,673664431	0	1,947855991	0,336832215	0,172924599	-2,531784987	1	1	rifin
PFIT_0200900	0	0,730202578	0,731308564	0	1,669781517	0	0	0,651594276	0,52188211	0,325797138	0,624273437	-0,679750016	1	1	Plasmodium exported protein (PHISTb), unknown function
PFIT_0218400	0	0,730202578	0	0,627621687	8,348907586	6,817083202	1,347328862	0,651594276	2,753969176	0,999461569	0,36291676	-1,462289413	1	1	small nucleolar RNA snoR01
PFIT_0300100	0	0,730202578	2,193925692	0,627621687	0	0	0	0	0,591958326	0	0	#DIV/0!	1	1	stevor
PFIT_0300500	0	0,730202578	1,462617128	0	0	13,6341664	0,673664431	0	2,637831018	0,336832215	0,127692871	-2,969250115	1	1	rifin (RIF)
PFIT_0408800	0	0,730202578	0,731308564	2,51048675	0	0	2,020993292	0	0,661999649	1,010496646	1,526430789	0,610162177	1	1	tRNA Isoleucine
PFIT_0412800.2	0	2,920810311	0	0,627621687	11,68847062	0	2,694657723	0	2,53948377	1,347328862	0,530552263	-0,914433221	1	1	calmodulin-like protein
PFIT_0424000	0	0,730202578	0	0	0	0	0	0	0,12170043	0	0	#DIV/0!	1	1	rifin (RIF)
PFIT_0424300	0	0,730202578	2,925234256	1,882865062	3,339563034	0	1,347328862	0,651594276	1,479644155	0,999461569	0,675474279	-0,56602726	1	1	rifin (RIF)
PFIT_0500300	0	0,730202578	1,462617128	0	1,669781517	6,817083202	0,673664431	0	1,779947404	0,336832215	0,189237173	-2,401732579	1	1	rifin (RIF)
PFIT_0612000.3	0	8,762430934	13,16355415	6,903838562	41,74453793	3,408541601	10,77863089	7,819131318	12,33048386	9,298881105	0,754137567	-0,407100376	1	1	conserved Plasmodium protein, unknown function
PFIT_0619700	0	8,762430934	3,65654282	11,92481206	38,4049749	149,9758304	8,7576376	24,76058251	35,45409853	16,75911005	0,472698806	-1,081006873	1	1	tRNA Alanine
PFIT_0619800	0	0,730202578	0	0	1,669781517	0	0,673664431	0	0,399997349	0,336832215	0,842086119	-0,247960312	1	1	tRNA Leucine
PFIT_0624000	0	14,60405156	34,37150251	28,24297594	28,38628579	3,408541601	25,59924837	16,28985691	18,1688929	20,94455264	1,152769888	0,205104556	1	1	sphingomyelin synthase 2, putative (SMS2)
PFIT_0628900	0	2,190607733	0,731308564	1,882865062	1,669781517	0	2,020993292	0,651594276	1,079093813	1,336293784	1,238348111	0,308416926	1	1	CPW-WPC family protein
PFIT_0700300	0	4,381215467	10,2383199	11,29719037	6,679126069	3,408541601	5,389315446	7,167537041	6,000732234	6,278426244	1,046276687	0,065264422	1	1	stevor
PFIT_0721400	0	25,55709022	11,70093702	28,87059762	23,37694124	10,2256248	27,62024166	9,12231987	16,62186515	18,37128077	1,105247853	0,144369931	1	1	conserved Plasmodium protein, unknown function
PFIT_0729600	0	0,730202578	0	0	0	0	0	0	0,12170043	0	0	#DIV/0!	1	1	tRNA Threonine
PFIT_0733300	0	0,730202578	0	1,255243375	1,669781517	3,408541601	1,347328862	0	1,177294845	0,673664431	0,572213863	-0,805373646	1	1	rifin (RIF)
PFIT_0733800	0	0,730202578	1,462617128	0	1,669781517	0	0	0	0,64376687	0	0	#DIV/0!	1	1	stevor
PFIT_0734200	0	0,730202578	0,731308564	1,882865062	0	0	0	0,651594276	0,557396034	0,325797138	0,584498486	-0,774728806	1	1	rifin (RIF)
PFIT_0801300	0	0,730202578	0	0,627621687	0	0	0	0	0,226304044	0	0	#DIV/0!	1	1	Plasmodium exported protein (PHISTa), unknown function, pseudogene
PFIT_0832900	0	5,111418045	4,387851384	2,51048675	26,71650428	27,26833281	5,389315446	5,212754212	10,99909888	5,301034829	0,481951739	-1,053039408	1	1	unspecified product
PFIT_0901700	0	0,730202578	0,731308564	1,255243375	0	3,408541601	0,673664431	0,651594276	1,020882686	0,662629354	0,649074926	-0,62354307	1	1	rifin (RIF)
PFIT_0905200	0	13,1436464	7,313085639	11,29719037	31,72584883	10,2256248	11,45229532	10,42550842	12,28423267	10,93890187	0,890483123	-0,167339826	1	1	conserved Plasmodium protein, unknown function
PFIT_0929900	0	5,841620623	8,044394203	10,041947	60,11213462	3,408541601	8,7576376	9,12231987	14,57477301	8,939978735	0,613387168	-0,70513011	1	1	conserved Plasmodium protein, unknown function
PFIT_1033500	0	5,111418045	2,925234256	3,138108437	5,009344552	0	2,694657723	1,954782829	2,697350882	2,324720276	0,861853121	-0,214486072	1	1	conserved Plasmodium protein, unknown function
PFIT_1110300	0	6,5718232	2,193925692	4,393351812	10,0186891	6,817083202	4,715651015	3,909565659	4,999145502	4,312608337	0,862669097	-0,213120819	1	1	small nucleolar RNA SNORD127
PFIT_1128100	0	2,920810311	2,193925692	3,765730125	0	0	2,020993292	1,954782829	1,480077688	1,987888061	1,343097107	0,425563617	1	1	conserved Plasmodium protein, unknown function
PFIT_1147700	0	3,651012889	7,313085639	6,903838562	8,348907586	0	2,694657723	5,864348488	4,369474113	4,279503106	0,979409191	-0,03001636	1	1	conserved Plasmodium protein, unknown function
PFIT_1212900	0	6,5718232	2,925234256	2,51048675	8,348907586	6,817083202	3,368322154	2,606377106	4,528922499	2,98734963	0,659615975	-0,600301756	1	1	conserved Plasmodium protein, unknown function
PFIT_1229400	0	4,381215467	1,462617128	2,51048675	5,009344552	3,408541601	2,020993292	1,954782829	2,795367583	1,987888061	0,711136551	-0,491801486	1	1	conserved Plasmodium protein, unknown function
PFIT_1232100	0	8,762430934	13,16355415	12,55243375	30,05606731	13,6341664	17,5152752	6,515942765	13,02810876	12,01560898	0,922283442	-0,116717899	1	1	cytochrome b5, putative
PFIT_1252100	0	0,730202578	0	0	0	3,408541601	0	0	0,689790696	0	0	#DIV/0!	1	1	tRNA Valine
PFIT_1312500	0	23,36648249	22,67056548	32,00870606	35,06541186	17,04270801	22,23092622	21,50261112	21,69231232	21,86676867	1,008042312	0,011556197	1	1	peptidyl-prolyl cis-trans isomerase, putative
PFIT_1324900	0	1,460405156	1,462617128	0,627621687	3,339563034	0	0	0,651594276	1,148367834	0,325797138	0,283704514	-1,81753899	1	1	conserved Plasmodium protein, unknown function
PFIT_1331100	0	0,730202578	0	0	8,348907586	3,408541601	0,673664431	0,651594276	2,081275294	0,662629354	0,318376601	-1,651193786	1	1	small nucleolar RNA snoR18
PFIT_1333700	0	6,5718232	2,925234256	5,0209735	25,04672276	3,408541601	3,368322154	8,470725594	7,162215886	5,919523874	0,82649336	-0,274924866	1	1	MSP7-like protein
PFIT_1340200	0	0,730202578	0	0	0	0	0	0	0,12170043	0	0	#DIV/0!	1	1	tRNA Arginine
PFIT_1340500	0	0,730202578	0,731308564	0,627621687	11,68847062	17,04270801	2,020993292	1,303188553	5,136718576	1,662090923	0,323570563	-1,62784773	1	1	small nucleolar RNA snoR20
PFIT_1354500	0	1,460405156	0	0,627621687	0	0	0	0	0,348004474	0	0	#DIV/0!	1	1	tRNA Threonine
PFIT_1366900	0	11,68324125	8,044394203	6,276216875	15,02803365	0	7,410308739	5,864348488	6,838647663	6,637328613	0,97056157	-0,043108358	1	1	conserved Plasmodium protein, unknown function
PFIT_1368800	0	0,730202578	0	0	1,669781517	0	0,673664431	0	0,399997349	0,336832215	0,842086119	-0,247960312	1	1	tRNA Arginine
PFIT_1401100	0	0,730202578	0,731308564	1,882865062	0	0	1,347328862	0	0,557396034	0,673664431	1,208592078	0,27332739	1	1	Plasmodium exported protein, unknown function, fragment
PFIT_1425500	0	3,651012889	6,581777075	2,51048675	18,36759669	10,2256248	6,736644308	3,909565659	6,889416368	5,323104983	0,772649626	-0,372113752	1	1	small nucleolar RNA snoR27
PFIT_1432100	0	59,1464088	19,01402266	17,57340725	222,0809418	17,04270801	35,70421483	37,14087376	55,80958142	36,42254429	0,652621707	-0,615681121	1	1	cytochrome c oxidase subunit 2, putative
PFIT_1463400	0	5,111418045	2,193925692	3,765730125	6,679126069	3,408541601	4,041986585	1,954782829	3,526456922	2,998384707	0,850254171	-0,234033916	1	1	conserved Plasmodium protein, unknown function
PFIT_1465900	0	0,730202578	2,193925692	1,255243375	0	0	1,347328862	0	0,696561941	0,673664431	0,967127819	-0,048221521	1	1	conserved Plasmodium protein, unknown function
PFIT_1478300	0	2,190607733	4,387851384	3,138108437	1,669781517	17,04270801	4,715651015	1,954782829	4,738176179	3,335216922	0,703903105	-0,506551246	1	1	Plasmodium exported protein (hyp15), unknown function
PFIT_bin02800	0	2,190607733	2,925234256	3,138108437	1,669781517	17,04270801	3,368322154	1,303188553	4,494406658	2,335755353	0,519702717	-0,944241492	1	1	rifin (RIF)
PFIT_bin03300	0	2,190607733	1,462617128	2,51048675	0	3,408541601	2,020993292	1,303188553	1,595375535	1,662090923	1,041817983	0,059103245	1	1	stevor
PFIT_bin05300	0	0,730202578	0,731308564	0,627621687	0	3,408541601	0,673664431	0,651594276	0,916279072	0,662629354	0,723174166	-0,467584953	1	1	stevor
PFIT_bin05400	0	0,730202578	0	0	1,669781517	0	0	0	0,399997349	0	0	#DIV/0!	1	1	Plasmodium exported protein (PHISTa), unknown, putative
PFIT_bin05820	0	2,190607733	2,193925692	0	0	0	0,673664431	0,651594276	0,730755571	0,662629354	0,906772907	-0,141186808	1	1	rifin (RIF)
PFIT_bin08550	0	0,730202578	3,65654282	3,765730125	1,669781517	3,408541601	3,368322154	1,303188553	2,205133107	2,335755353	1,059235538	0,083023432	1	1	rifin
PFIT_bin09500	0	0,730202578	1,462617128	1,255243375	0	0	0,673664431	0	0,57467718	0,336832215	0,586124223	-0,770721635	1	1	stevor, pseudogene
PF_apicoplast_ge	0	0,730202578	0	0,627621687	5,009344552	3,408541601	1,347328862	0,651594276	1,62928507	0,999461569	0,613435664	-0,70501605	1	1	apicoplast ribosomal protein L36
PF_apicoplast_ge	0	1,460405156	0	0	15,02803365	6,817083202	2,020993292	1,303188553	3,884253669	1,662090923	0,427904834	-1,224638117	1	1	unspecified product
PF_apicoplast_ge	0	0,730202578	0,731308564	0,627621687	0	0	0	0	0,348188805	0	0	#DIV/0!	1	1	unspecified product
PF_apicoplast_ge	0	0,730202578	0	0,627621687	1,669781517	0	0,673664431	0	0,504600964	0,336832215	0,667521942	-0,583112834	1	1	unspecified product
Pf_M7661100200	0	0,730202578	0	0	0	0	0	0	0,12170043	0	0	#DIV/0!	1	1	unspecified product
PFIT_0113400	0	0	0,731308564	3,138108437	3,339563034	0	0	0,651594276	1,201496673	0,325797138	0,271159418	-1,882786817	1	1	stevor, pseudogene
PFIT_0220800	0	0	0,731308564	0,627621687	0	0	0	0	0,226488375	0	0	#DIV/0!	1	1	stevor
PFIT_0408700	0	0	2,193925692	0	0	0	0	0	0,365654282	0	0	#DIV/0!	1	1	tRNA Serine
PFIT_0531900	0	0	0,731308564	0	3,339563034	0	0	0	0,6784786	0	0	#DIV/0!	1	1	tRNA Glutamic acid
PFIT_0705700	0	0	2,925234256	0	1,669781517	0	0	0,651594276	0,765835962	0,325797138	0,425413737	-1,233061476	1	1	tRNA Threonine
PFIT_1000100	0	0	4,387851384	1,882865062	0	3,408541601	0	2,606377106	1,613209675	1,303188553	0,807823418	-0,307888126	1	1	stevor
PFIT_1216500	0	0	4,387851384	1,882865062	1,669781517	3,408541601	2,020993292	0,651594276	1,891506594	1,336293784	0,706470593	-0,501298586	1	1	tRNA Proline
PFIT_1252200	0	0	0,731308564	0	0	0	0	0	0,121884761	0	0	#DIV/0!	1	1	tRNA Glutamic acid
PFIT_1322000	0	0	0,731308564	0	10,0186891	0	1,347328862	0	1,791666278	0,673664431	0,375998834	-1,411199908	1	1	conserved Plasmodium protein, unknown function
PFIT_1368900	0	0	0,731308564	0	0	0	0	0	0,121884761	0	0	#DIV/0!	1	1	tRNA Tryptophan
PFIT_1369200	0	0	0,731308564	0	0	0	0	0	0,121884761	0	0	#DIV/0!	1	1	tRNA Glycine
PFIT_bin05860	0	0	0,731308564	0	0	3,408541601	0	0	0,689975027	0	0	#DIV/0!	1	1	rifin (RIF)
PFIT_bin06700	0	0	2,925234256	3,138108437	1,669781517	10,2256248	1,347328862	1,954782829	2,993124836	1,651055845	0,551616099	-0,858263532	1	1	rifin (RIF)
Pf_M7661100600	0	0	1,462617128	0	0	0	0	0	0,243769521	0	0	#DIV/0!	1	1	unspecified product
Pf_M7661101200	0	0	2,193925692	0	3,339563034	3,408541601	1,347328862	0,651594276	1,490338388	0,999461569	0,670627273	-0,576416939	1	1	unspecified product
PFIT_1038400	0	0	0	0,627621687	0	3,408541601	0	0	0,672693881	0	0	#DIV/0!	1	1	Plasmodium exported protein (PHISTb), unknown function
PFIT_bin10200	0	0	0	1,255243375	0	0	0,673664431	0	0,209207229	0,336832215	1,610040995	0,687097423	1	1	rifin (RIF)
PF_apicoplast_ge	0	0	0	0,627621687	3,339563034	0	0	0	0,661197454	0	0	#DIV/0!	1	1	unspecified product
PF_apicoplast_ge	0	0	0	0,627621687	0	0	0	0	0,104603615	0	0	#DIV/0!	1	1	unspecified product
PFIT_0500800	0	0	0	0	1,669781517	0	0	0	0,27829692	0	0	#DIV/0!	1	1	rifin, pseudogene
PFIT_0701700	0	0	0	0	1,669781517	0	0	0	0,27829692	0	0	#DIV/0!	1	1	tRNA Tyrosine
PFIT_0710500	0	0	0	0	5,009344552	6,817083202	0	0,651594276	1,971071292	0,325797138	0,165289373	-2,596934122	1	1	unspecified product
PFIT_1104100	0	0	0	0	3,339563034	0	0	0	0,556593839	0	0	#DIV/0!	1	1	tRNA Leucine
PFIT_1240300	0	0	0	0	1,669781517	0	0	0	0,27829692	0	0	#DIV/0!	1	1	unspecified product
PF_apicoplast_ge	0	0	0	0	3,339563034	3,408541601	0	0	1,124684106	0	0	#DIV/0!	1	1	unspecified product
PF_apicoplast_ge	0	0	0	0	3,339563034	0	0	0	0,556593839	0	0	#DIV/0!	1	1	unspecified product
PFIT_0514500	0	0	0	0	0	0	0	0	0	0	#NV	#NV	#NV	#NV	tRNA Phenylalanine
PFIT_0729500	0	0	0	0	0	0	0	0	0	0	#NV	#NV	#NV	#NV	tRNA Valine
PF_apicoplast_ge	0	0	0	0	0	0	0	0	0	0	#NV	#NV	#NV	#NV	unspecified product

Table S2. Differentially expressed genes (threshold, ≥ 3-fold; padj ≤ 0.05) in FCR3 knobless and knobby IEs cultivated in the presence of AlbuMAX.

	AlbuMAX_K-_1	AlbuMAX_K-_2	AlbuMAX_K+_1	AlbuMAX_K+_2	baseMeanA	baseMeanB	foldChange	log2FoldCh	pval	padj	name
PF_apicoplast_g	23547,57091	26417,01074	7393,392384	4990,551841	24982,29083	6191,9721	0,247854456	-2,012435	7,02E-28	3,96376E-24	unspecified product
PFIT_1415900	1944,146323	1788,040486	602,7039823	405,2900584	1866,093405	503,99702	0,270081347	-1,888534	7,55E-24	2,13126E-20	ATP-dependent protease, putative
PFIT_1305000	831,5235978	943,7213013	245,6081597	197,2946654	887,6224495	221,45141	0,249488296	-2,002956	5,675E-23	1,06806E-19	conserved Plasmodium protein, unknown function
PFIT_1116800	25066,38923	22540,32819	6901,337811	7654,364222	23803,35871	7277,851	0,305748912	-1,709581	3,857E-22	5,44402E-19	rhoptry neck protein 4
PFIT_0404900	5271,712438	5962,92939	2024,381248	1495,426684	5617,320914	1759,904	0,313299524	-1,674386	7,999E-21	9,03247E-18	sexual stage-specific protein precursor
PFIT_0701300	11754,65305	12807,30406	3930,568808	1798,391035	12280,97856	2864,4799	0,233245251	-2,10008	1,524E-18	1,43393E-15	conserved Plasmodium protein, unknown function
PFIT_0805400	6771,39836	6464,730437	2358,005984	2184,95482	6618,064399	2271,4804	0,343224282	-1,542776	2,76E-18	2,22641E-15	adenylyl cyclase beta (ACbeta)
PFIT_1245200	660,8037087	598,8079323	160,9445961	182,5811649	629,8058205	171,76288	0,272723552	-1,874489	6,526E-18	4,60543E-15	conserved Plasmodium protein, unknown function
PFIT_0629200	2818,349893	2668,288146	1061,228431	876,7908689	2743,31902	969,00965	0,353225288	-1,501339	1,209E-16	7,58243E-14	DNA polymerase epsilon, catalytic subunit a, putative
PFIT_1235400	2200,226157	2262,296368	720,059417	905,5490744	2231,261263	812,80425	0,364280176	-1,45688	4,008E-15	2,26317E-12	ATP synthase subunit beta, mitochondrial
PFIT_0107000	45203,97753	45159,69902	16423,05483	18922,2304	45181,83828	17672,643	0,391144834	-1,354225	7,109E-15	3,64907E-12	conserved Plasmodium protein, unknown function
PFIT_1016400	1395,193576	1482,64844	635,3958534	442,0738096	1438,921008	538,73483	0,37440195	-1,41734	2,227E-14	1,04784E-11	early transcribed membrane protein 10.3
PFIT_0501500	38788,73618	49101,05283	7623,911988	9850,019769	43944,89451	8736,9659	0,198816404	-2,330491	1,436E-13	6,23458E-11	interspersed repeat antigen
PFIT_1467300	996,3565942	1025,15918	386,4346813	357,8055796	1010,757887	372,12013	0,368159512	-1,441597	1,644E-13	6,62848E-11	conserved Plasmodium protein, unknown function
PFIT_1475100	953,6766219	1073,063815	373,0226316	376,5318529	1013,370218	374,77724	0,369832501	-1,435056	1,803E-13	6,78714E-11	probable protein, unknown function
PFIT_0307700	2251,736468	2189,2418	1015,12451	775,8027521	2220,489134	895,46363	0,403273142	-1,310171	4,483E-13	1,58199E-10	conserved Plasmodium protein, unknown function
PFIT_bin05500	347,3266709	297,0087344	116,5171816	68,21713854	322,1677027	92,36716	0,286705214	-1,80236	3,446E-12	1,12464E-09	Plasmodium exported protein (hyp15), unknown function
PFIT_0527900	179,5502282	217,9660874	538,9967463	601,2471328	198,7581578	570,12194	2,868420325	1,5202564	3,585E-12	1,12464E-09	conserved Plasmodium membrane protein, unknown function
PFIT_0819200	660,8037087	768,8693851	295,0650929	204,6514156	714,8365469	249,85825	0,349532009	-1,516504	4,581E-12	1,3614E-09	conserved Plasmodium protein, unknown function
PFIT_1235900	1255,379874	1410,791488	587,6154264	509,6221526	1333,085681	548,61879	0,411540531	-1,280894	6,808E-12	1,92178E-09	conserved Plasmodium protein, unknown function
PFIT_1230400	5201,069725	5688,675357	2193,708375	2523,365331	5444,872541	2358,5369	0,433166587	-1,207006	7,297E-12	1,96178E-09	protein transport protein Sec13, putative
PFIT_0308400	1290,701231	1392,827251	585,1006671	542,393131	1341,764241	563,7469	0,420153468	-1,251012	2,532E-11	6,49742E-09	activator of Hsp90 ATPase, putative
PFIT_0608400	1914,71186	1685,045521	798,8552088	731,6622507	1799,878691	765,25873	0,425172393	-1,23388	3,306E-11	8,11564E-09	conserved Plasmodium protein, unknown function
PFIT_1403700	6054,669171	5865,922505	2955,680447	2406,994918	5960,295838	2681,3377	0,449866543	-1,152431	3,483E-11	8,19473E-09	conserved Plasmodium protein, unknown function
PFIT_0612800	10762,71163	9822,845321	4203,83932	4984,532682	10292,77848	4594,186	0,446350421	-1,163751	4,835E-11	1,09202E-08	transcription factor with AP2 domain(s)
PFIT_0827600	1994,184912	1698,219296	790,4726778	580,5144731	1846,202104	685,49358	0,371299314	-1,429345	7,602E-11	1,65082E-08	conserved Plasmodium membrane protein, unknown function
PFIT_1241600	584,2741032	510,1843583	220,4605665	197,2946654	547,2292308	208,87762	0,381700399	-1,389487	1,05E-10	2,15255E-08	RNA binding protein, putative
PFIT_0501400	42294,3808	46449,53131	19773,55249	21458,30284	44371,95606	20615,928	0,464616156	-1,105889	1,068E-10	2,15255E-08	skeleton-binding protein 1
PFIT_0707800	164,8329964	213,1756239	475,2895104	538,3803581	189,0043101	506,83493	2,681605165	1,4230968	1,683E-10	3,27658E-08	Cg3 protein
PFIT_1141600	900,6945874	865,8762701	343,6837729	385,8949896	883,2854287	364,78938	0,412991508	-1,275816	1,768E-10	3,32669E-08	conserved Plasmodium protein, unknown function
PFIT_1344200	133,9268096	142,5162879	363,8018475	414,6531951	138,2215487	389,22752	2,815968457	1,4936312	1,924E-10	3,50428E-08	conserved Plasmodium protein, unknown function
PFIT_1008900	1520,290047	1513,786453	760,295566	587,8712233	1517,03825	674,08339	0,444341726	-1,170258	2,152E-10	3,79744E-08	zinc finger protein, putative
PFIT_1248800	306,1184218	343,7157531	797,1787026	790,5162525	324,9170875	793,84748	2,443230929	1,2887902	2,769E-10	4,73706E-08	conserved Plasmodium protein, unknown function
PFIT_0834800	16096,23644	18682,80749	8213,20392	8335,198016	17389,52196	8274,201	0,475815321	-1,071526	3,205E-10	5,3228E-08	histidine-rich protein II
PFIT_1421000	1409,910808	1826,364193	419,9648055	410,6404222	1618,137501	415,30261	0,256654712	-1,962099	5,039E-10	8,12899E-08	splicing factor 3B subunit 4, putative
PFIT_1202300	7179,065682	8541,396346	3695,019686	3701,782959	7860,231014	3698,4013	0,47052069	-1,08767	7,983E-10	1,25201E-07	conserved protein, unknown function
PFIT_1340300	1330,437756	2032,354122	3879,435369	4159,239065	1681,395939	4019,3372	2,390476343	1,2572981	1,361E-09	2,07704E-07	U6 spliceosomal RNA
PFIT_1015800	32204,24667	24354,71622	6930,67667	3337,95822	28279,48144	5134,3174	0,18155628	-2,461511	1,435E-09	2,13256E-07	glycophorin binding protein
PFIT_0937400	129,51164	166,4686052	45,26566766	32,77097832	147,9901226	39,018323	0,263654914	-1,923277	1,754E-09	2,47603E-07	Plasmodium exported protein (PHISTc), unknown function
PFIT_bin11000	40607,78604	51187,29967	16037,4584	13888,87565	45897,54285	14963,167	0,326012377	-1,617001	1,753E-09	2,47603E-07	erythrocyte membrane protein 1, PfEMP1, putative
PFIT_1237600	10636,14344	11555,79548	5325,421974	5621,225975	11095,96946	5473,324	0,493271363	-1,019547	3,323E-09	4,57603E-07	conserved Plasmodium protein, unknown function
PFIT_0416900	7813,378373	9458,770099	3321,997054	3846,242782	8636,074236	3584,1199	0,415017266	-1,268757	3,509E-09	4,71714E-07	conserved Plasmodium protein, unknown function
PFIT_bin09300	122,1530241	142,5162879	28,50060556	41,46531951	132,334656	34,982963	0,264352238	-1,919467	5,674E-09	7,42109E-07	BC_ITvar16 - erythrocyte membrane protein 1,, putative
PFIT_1133900	2884,577437	2549,724176	1191,157662	1398,45134	2717,150806	1294,8045	0,476530231	-1,06936	5,783E-09	7,42109E-07	conserved Plasmodium protein, unknown function
PFIT_1023400	14056,42811	17317,5254	5169,506897	6430,468501	15686,97676	5799,9877	0,369732664	-1,435446	8,321E-09	1,04404E-06	chromodomain-helicase-DNA-binding protein 1 homolog, putative
PFIT_0304000	3234,847554	2961,704033	1659,741147	1128,257968	3098,275794	1393,9996	0,449927524	-1,152235	8,822E-09	1,07701E-06	membrane skeletal protein IMC1-related
PFIT_1244600	4644,758362	4614,413926	2455,243344	2160,878183	4629,586144	2308,0608	0,498545808	-1,004202	8,966E-09	1,07701E-06	conserved Plasmodium protein, unknown function
PFIT_1456500	19806,45058	18056,45439	10248,48246	7103,27675	18931,45249	8675,8796	0,458278603	-1,125703	1,015E-08	1,19447E-06	conserved Plasmodium protein, unknown function
PFIT_0401500	674,0492173	491,0225045	1267,438694	1162,366537	582,5358609	1214,9026	2,085541333	1,0604219	1,285E-08	1,48025E-06	erythrocyte membrane protein 1 (PfEMP1), pseudogene
PFIT_0107200	1492,327306	1736,543004	841,6061172	747,7133421	1614,435155	794,65973	0,492221522	-1,02262	1,605E-08	1,8128E-06	secreted ookinete protein, putative
PFIT_0934800	403,2521519	514,9748218	988,3004105	1001,855623	459,1134868	995,07802	2,16739008	1,1159588	1,85E-08	2,04763E-06	conserved Plasmodium protein, unknown function
PFIT_0314600	319,3639305	397,608467	129,9292312	147,1350047	358,4861988	138,53212	0,386436405	-1,371697	2,108E-08	2,24576E-06	zinc finger protein, putative
PFIT_0825600	63,28409682	80,24026293	196,1512265	230,0656437	71,76217988	213,10844	2,969648295	1,5702921	2,099E-08	2,24576E-06	conserved Plasmodium protein, unknown function
PFIT_1024600	6690,453585	7015,633735	2943,944904	3987,358627	6853,04366	3465,6518	0,505709862	-0,983618	2,714E-08	2,83786E-06	glucosamine-fructose-6-phosphate aminotransferase, putative
PFIT_0720400	16973,38346	14590,55408	7295,316771	8585,99632	15781,96877	7940,6565	0,5031474	-0,990947	2,869E-08	2,94519E-06	rhoptry-associated leucine zipper-like protein 1
PFIT_1003900	5833,910693	6532,994541	2802,280129	3513,182635	6183,452617	3157,7314	0,510674469	-0,969524	3,136E-08	3,16155E-06	zinc finger, C3HC4 type, putative
PFIT_1151000	475,3665878	602,4007799	170,1653803	203,3138247	538,8836838	186,7396	0,346530444	-1,528946	3,351E-08	3,31895E-06	erythrocyte membrane protein 1, PfEMP1 (VAR)
PFIT_bin02200	1256,851597	1661,093204	413,2587806	415,990786	1458,972401	414,62478	0,2841896	-1,815074	4,03E-08	3,92304E-06	stB_A4varTres - erythrocyte membrane protein 1,, putative
PFIT_1026700	28850,18954	27070,909	16041,64967	13393,967	27960,54927	14717,808	0,526377654	-0,92583	5,786E-08	5,53656E-06	merozoite capping protein 1
PFIT_0702300	156,0026573	195,2113859	441,7593862	391,9141489	175,6070216	416,83677	2,373690777	1,247132	6,901E-08	6,49399E-06	conserved Plasmodium protein, unknown function
PFIT_1359600	1248,021258	1202,406328	566,6590988	636,0244976	1225,213793	601,3418	0,490805606	-1,026776	8,057E-08	7,45774E-06	guanylyl cyclase beta
PFIT_0823800	6509,431634	6219,219185	2965,739485	3620,189911	6364,325409	3292,9647	0,517409857	-0,950621	8,798E-08	8,01227E-06	conserved Plasmodium protein, unknown function
PFIT_0529600	359,1004564	335,3324421	749,3982756	712,2671819	347,2164492	730,83273	2,104833254	1,0737059	1,06E-07	9,50164E-06	G-protein coupled receptor, putative
PFIT_1219100	126,5681936	168,8638369	42,75090834	40,79652403	147,7160153	41,773716	0,282797475	-1,822159	1,09E-07	9,61843E-06	erythrocyte membrane protein 1 (PfEMP1), pseudogene
PFIT_0618900	116,2661314	125,7496658	32,69187108	38,12134213	121,0078986	35,406607	0,292597483	-1,773011	1,158E-07	9,75998E-06	conserved Plasmodium protein, unknown function
PFIT_0811500	3530,663913	4353,333668	1839,127312	1599,758778	3941,998791	1719,443	0,436185584	-1,196986	1,152E-07	9,75998E-06	erythrocyte membrane protein 1, PfEMP1
PFIT_0403000	572,5003178	634,7364082	290,8738273	278,218918	603,618363	284,54637	0,47140112	-1,084973	1,126E-07	9,75998E-06	conserved Plasmodium protein, unknown function
PFIT_0835600	250,1929409	301,7991979	122,3849533	106,3384807	275,9960694	114,36172	0,414359948	-1,271044	1,343E-07	1,11481E-05	erythrocyte membrane protein 1, PfEMP1, putative
PFIT_1418600	11132,11415	12189,33427	6088,2323	6376,296067	11660,72421	6232,2642	0,534466305	-0,903829	1,371E-07	1,12194E-05	conserved Plasmodium protein, unknown function
PFIT_0104200	1261,266767	1232,346725	530,6142153	703,5728407	1246,806746	617,09353	0,494939196	-1,014677	1,409E-07	1,13636E-05	asparagine-rich antigen Pfa35-2
PFIT_0302200	1524,705216	1620,374265	858,3711793	760,4204561	1572,539741	809,39582	0,514706113	-0,958179	1,476E-07	1,17387E-05	erythrocyte membrane protein 1 (PfEMP1), pseudogene
PFIT_1217800	101,5488996	65,86887255	219,6223134	219,3649161	83,70888605	219,49361	2,622106506	1,3907263	1,528E-07	1,19818E-05	conserved Plasmodium protein, unknown function
PFIT_0710400	1526,17694	1621,571881	853,3416606	769,1147973	1573,87441	811,22823	0,515433902	-0,956141	1,615E-07	1,24882E-05	regulator of chromosome condensation, putative
PFIT_1241800	487,1403732	467,0701872	225,4900852	212,6769613	477,1052802	219,08352	0,459193248	-1,122827	2,026E-07	1,50538E-05	mitochondrial carrier protein, putative
PFIT_0515100	1960,335278	2119,78008	958,1232987	1155,009787	2040,057679	1056,5665	0,517910132	-0,949226	2,01E-07	1,50538E-05	RNA binding protein, putative
PFIT_0935400	251,6646641	241,9184046	537,3202401	527,6796305	246,7915344	532,49994	2,157691254	1,1094884	1,984E-07	1,50538E-05	conserved Plasmodium protein, unknown function
PFIT_0620300	722,6160823	658,6887255	1390,661901	1314,851906	690,6524039	1352,7569	1,958665308	0,9698709	2,054E-07	1,50626E-05	Pf77 protein
PFIT_1432300	320,8356537	354,4942959	139,9882685	152,4853685	337,6649748	146,23682	0,43308258	-1,207286	2,088E-07	1,51125E-05	conserved Plasmodium protein, unknown function
PFIT_0721000	175,1350587	198,8042335	77,11928564	62,86677474	186,9696461	69,99303	0,374355045	-1,417521	2,196E-07	1,56977E-05	conserved Plasmodium protein, unknown function
PFIT_0804100	422,3845532	414,3750891	170,1653803	203,9826202	418,3798212	187,074	0,447139156	-1,161204	2,387E-07	1,68481E-05	von Willebrand factor A-domain related protein
PFIT_0535100	3489,455664	3109,010784	1865,113158	1629,854575	3299,233224	1747,4839	0,529663636	-0,916852	2,632E-07	1,83427E-05	formin 1
PFIT_0411500	1454,062504	1905,406841	558,2765678	575,8329048	1679,734672	567,05474	0,337585897	-1,566673	2,957E-07	1,98743E-05	BC_ITvar27 - erythrocyte membrane protein 1,, putative (VAR)
PFIT_1145100	1162,661314	1239,53242	645,4548906	591,2152007	1201,096867	618,33505	0,514808641	-0,957892	2,911E-07	1,98743E-05	conserved Plasmodium protein, unknown function
PFIT_1326500	8909,812143	8419,239528	4841,749933	4510,35669	8664,525836	4676,0533	0,539677924	-0,889829	2,923E-07	1,98743E-05	conserved Plasmodium protein, unknown function
PFIT_0311400	154,5309341	183,2352273	343,6837729	420,6723544	168,8830807	382,17806	2,262974255	1,1782202	3,447E-07	2,28971E-05	conserved Plasmodium protein, unknown function
PFIT_bin08900	103,0206227	113,7735071	32,69187108	31,43338737	108,3970649	32,062629	0,295788721	-1,757361	3,575E-07	2,34711E-05	erythrocyte membrane protein 1, PfEMP1
PFIT_0926100	2167,848247	1718,578766	3632,988956	3500,475521	1943,213506	3566,7322	1,835481396	0,8761585	4,111E-07	2,66767E-05	protein kinase, putative
PFIT_1221100	98,60545319	110,1806595	29,33885867	31,43338737	104,3930564	30,386123	0,291074177	-1,780541	4,307E-07	2,7399E-05	inner membrane complex protein 1h, putative (IMC1h)
PFIT_0413400	429,7431691	404,7941622	905,3133531	769,1147973	417,2686657	837,21408	2,006414917	1,00462	4,319E-07	2,7399E-05	conserved Plasmodium protein, unknown function
PFIT_1312100	6122,368437	6501,856529	3371,453987	3509,838658	6312,112483	3440,6463	0,545086345	-0,875443	4,537E-07	2,84651E-05	eukaryotic translation initation factor 4 gamma, putative
PFIT_bin02100	164,8329964	165,2709893	59,51597044	60,19159283	165,0519928	59,853782	0,362635922	-1,463406	5,024E-07	3,11712E-05	B_ITvar29 - erythrocyte membrane protein 1, PfEMP1, putative
PFIT_1244700	1084,659985	1237,137188	636,2341065	579,8456776	1160,898587	608,03989	0,523766588	-0,933004	5,08E-07	3,11769E-05	conserved Plasmodium protein, unknown function
PFIT_0421900	5926,629254	5758,137077	3402,469352	2997,541323	5842,383165	3200,0053	0,547722607	-0,868483	5,488E-07	3,33167E-05	early transcribed membrane protein 4
PFIT_0416400	675,5209405	641,9221034	318,5361798	329,0473742	658,721522	323,79178	0,491545769	-1,024602	5,816E-07	3,45673E-05	cdc2-related protein kinase 1
PFIT_0817000	11,77378546	15,56900624	64,54548906	74,9050933	13,67139585	69,725291	5,100085753	2,3505215	5,79E-07	3,45673E-05	conserved Plasmodium protein, unknown function
PFIT_1031900	1393,721853	1475,462745	850,8269013	565,1321771	1434,592299	707,97954	0,493505743	-1,018861	6,504E-07	3,78596E-05	DER1-like protein, putative
PFIT_1350500	36,79307955	52,69509804	134,1204968	143,7910273	44,7440888	138,95576	3,10556692	1,6348567	6,48E-07	3,78596E-05	conserved Plasmodium membrane protein, unknown function
PFIT_0207200	398,8369823	405,9917781	205,3720107	174,5556192	402,4143802	189,96381	0,472060205	-1,082957	8,743E-07	5,03727E-05	serine repeat antigen 2
PFIT_0623500	18290,57571	21225,34597	10129,45052	11955,38793	19757,96084	11042,419	0,558884559	-0,839378	8,956E-07	5,05659E-05	SNF2 helicase, putative
PFIT_1016300	2996,428398	2688,647616	5451,15994	4830,040927	2842,538007	5140,6004	1,808454424	0,8547572	8,889E-07	5,05659E-05	Plasmodium exported protein (PHISTc), unknown function
PFIT_0808500	2546,081105	2791,64258	4698,408652	4982,526296	2668,861843	4840,4675	1,813682296	0,8589218	1,038E-06	5,80309E-05	PAP2-like protein, putative
PFIT_0713300	2129,583444	1885,047371	1026,0218	1118,894831	2007,315408	1072,4583	0,534274938	-0,904346	1,064E-06	5,83387E-05	conserved Plasmodium protein, unknown function
PFIT_0622500	1250,964705	1444,324733	741,8539977	708,254409	1347,644719	725,0542	0,538015839	-0,894279	1,062E-06	5,83387E-05	transcription or splicing factor-like protein, putative
PFIT_bin08300	606,349951	695,8148173	342,8455198	320,353033	651,0823841	331,59928	0,509304636	-0,973399	1,094E-06	5,94125E-05	erythrocyte membrane protein 1, PfEMP1
PFIT_1457000	194,26746	207,1875446	70,4132608	89,61859377	200,7275023	80,015927	0,398629617	-1,326879	1,106E-06	5,94947E-05	LCCL domain-containing protein
PFIT_1207400	314,9487609	268,2659537	134,1204968	122,3895721	291,6073573	128,25503	0,439820983	-1,185012	1,12E-06	5,96632E-05	conserved Plasmodium protein, unknown function
PFIT_1037600	220,7584773	274,254033	66,22199527	107,0072761	247,5062551	86,614636	0,34994928	-1,514782	1,256E-06	6,62712E-05	gametocyte-specific protein
PFIT_1109900	997,8283174	980,8473931	510,4961408	523,6668576	989,3378552	517,0815	0,522654113	-0,936072	1,282E-06	6,70036E-05	conserved Plasmodium protein, unknown function
PFIT_1365400	1947,08977	1912,592536	1031,051319	1061,37842	1929,841153	1046,2149	0,54212486	-0,883303	1,347E-06	6,97578E-05	rhoptry protein
PFIT_0401900	1022,847611	1210,789639	570,0121112	622,6485881	1116,818625	596,33035	0,533954517	-0,905211	1,426E-06	7,32074E-05	conserved Plasmodium protein, unknown function
PFIT_0627300	30,90618682	37,1260918	108,1346505	115,7016173	34,01613931	111,91813	3,290148035	1,7181525	1,49E-06	7,58014E-05	conserved Plasmodium membrane protein, unknown function
PFIT_1302100	364,9873491	323,3562834	1000,035954	776,4715475	344,1718163	888,25375	2,580843953	1,3678429	1,525E-06	7,69001E-05	conserved Plasmodium protein, unknown function
PFIT_1463000	579,8589337	605,9936275	272,432259	323,6970103	592,9262806	298,06463	0,502701001	-0,992228	1,832E-06	9,1517E-05	conserved Plasmodium protein, unknown function
PFIT_1344300	1100,84894	1394,024866	2269,151155	2310,688369	1247,436903	2289,9198	1,835699871	0,8763302	1,979E-06	9,61398E-05	thioredoxin 2
PFIT_1236100	669,6340478	803,6002451	1404,912204	1355,64843	736,6171465	1380,2803	1,873809649	0,9059744	1,984E-06	9,61398E-05	conserved Plasmodium protein, unknown function
PFIT_1114000	236,9474323	238,3255571	487,0250539	483,5391291	237,6364947	485,28209	2,042119381	1,0300672	1,948E-06	9,61398E-05	conserved Plasmodium protein, unknown function
PFIT_1037100	83,88822137	83,83311052	191,1217079	220,0337116	83,86066595	205,57771	2,451419952	1,2936177	1,992E-06	9,61398E-05	conserved Plasmodium protein, unknown function
PFIT_0711000	782,9567328	888,6309715	442,5976393	441,4050141	835,7938522	442,00133	0,528840127	-0,919096	2,045E-06	9,78495E-05	C_ITvar28 - erythrocyte membrane protein 1, PfEMP1, putative
PFIT_0319800	154,5309341	184,4328431	347,8750385	380,5446258	169,4818886	364,20983	2,148960193	1,1036388	2,137E-06	0,000101412	conserved Plasmodium protein, unknown function
PFIT_0724600	613692,3779	426510,5307	47603,55556	122435,719	520101,4543	85019,637	0,163467409	-2,612925	2,315E-06	0,000108907	28S ribosomal RNA
PFIT_0724500	614419,4092	426637,478	47545,7161	122653,7463	520528,4436	85099,731	0,163487187	-2,612751	2,46E-06	0,000114778	28S ribosomal RNA
PFIT_0216100.2	1296,588123	1422,767647	671,4407369	813,9240942	1359,677885	742,68242	0,546219383	-0,872448	3,144E-06	0,000145484	conserved Plasmodium protein, unknown function
PFIT_1223200	7040,723702	7469,530147	4222,280889	4107,741813	7255,126925	4165,0114	0,574078358	-0,80068	3,178E-06	0,000145887	conserved Plasmodium protein, unknown function
PFIT_1455700	379,7045809	504,196279	841,6061172	999,8492365	441,95043	920,72768	2,083327935	1,05889	3,778E-06	0,000172012	nifU protein, putative
PFIT_0900100	223,7019237	210,7803922	77,11928564	107,0072761	217,2411579	92,063281	0,423783788	-1,2386	3,855E-06	0,000173031	B_ITvar41 - erythrocyte membrane protein 1, PfEMP1, putative
PFIT_0205900	86,83166773	101,7973485	231,3578569	215,3521432	94,31450811	223,355	2,368193447	1,2437869	3,861E-06	0,000173031	transcription factor, putative
PFIT_0517600	123,6247473	160,4805259	249,976537	337,0729199	142,0526366	293,52473	2,171904273	1,1189605	4,034E-06	0,000179354	UTP--glucose-1-phosphate uridylyltransferase, putative
PFIT_1203200	2657,932067	2283,853454	1440,957087	1328,896611	2470,89276	1384,9268	0,560496542	-0,835223	4,192E-06	0,000183687	Sua5/YciO/YrdC family protein, putative
PFIT_1225900	76,52960546	62,27602496	176,033152	170,5428464	69,40281521	173,288	2,496843949	1,3201057	4,197E-06	0,000183687	conserved Plasmodium protein, unknown function
PFIT_0411000	1083,188262	1253,90381	598,5127168	686,8529538	1168,546036	642,68284	0,549985037	-0,862536	4,314E-06	0,000187356	erythrocyte membrane protein 1, PfEMP1, putative
PFIT_1345400	133,9268096	153,2948307	316,0214205	304,970737	143,6108201	310,49608	2,162066051	1,1124106	4,621E-06	0,000199175	mog1 homolog, putative
PFIT_1003700	88,30339092	94,6116533	215,4310479	214,6833478	91,45752221	215,0572	2,351443521	1,2335467	4,777E-06	0,000204318	hypothetical protein
PFIT_1241100	915,4118192	967,6736186	596,8362106	444,080196	941,5427189	520,4582	0,552771736	-0,855244	5,137E-06	0,000218088	erythrocyte membrane protein 1, PfEMP1
PFIT_0308500	3036,164924	3494,643093	2081,382459	1724,154737	3265,404009	1902,7686	0,582705415	-0,779161	5,422E-06	0,000227866	pre-mRNA-processing factor 19, putative
PFIT_0930300	975,7524696	833,5406418	1647,167351	1560,968641	904,6465557	1604,068	1,773143318	0,8263092	5,448E-06	0,000227866	conserved Plasmodium protein, unknown function
PFIT_0814900	48,566865	41,91655526	115,6789285	136,4342771	45,24171013	126,0566	2,78629173	1,4783463	5,516E-06	0,000228977	conserved Plasmodium protein, unknown function
PFIT_0109000	1386,363237	1447,91758	791,3109309	793,1914344	1417,140409	792,25118	0,559049179	-0,838953	5,604E-06	0,000230967	bromodomain protein, putative
PFIT_1133500	417,9693837	447,9083334	213,7545417	225,3840754	432,9388585	219,56931	0,507160086	-0,979487	6,579E-06	0,000269186	mitochondrial ribosomal protein L2 precursor
PFIT_1474300	2264,981977	2287,446301	1529,811916	1112,206876	2276,214139	1321,0094	0,580353743	-0,784996	7,337E-06	0,000298003	DnaJ protein, putative
PFIT_1245300	838,8822137	905,3975936	458,5244483	495,5774477	872,1399037	477,05095	0,546989016	-0,870416	7,419E-06	0,000299179	kinesin-13, putative
PFIT_0307600	4013,389117	3385,660049	2180,296325	2061,896452	3699,524583	2121,0964	0,573342964	-0,80253	7,634E-06	0,000305694	conserved Plasmodium protein, unknown function
PFIT_0405100	2329,737797	2606,012121	1453,530884	1405,80809	2467,874959	1429,6695	0,579311963	-0,787588	7,827E-06	0,000309027	conserved Plasmodium protein, unknown function
PFIT_0803700	175,1350587	183,2352273	405,7145027	331,0537606	179,185143	368,38413	2,055885469	1,0397599	7,774E-06	0,000309027	Plasmodium exported protein (hyp7), unknown function, pseudogene
PFIT_1338800	135,3985327	174,8519162	316,0214205	336,4041244	155,1252245	326,21277	2,102899599	1,07238	7,958E-06	0,000312029	conserved Plasmodium protein, unknown function
PFIT_1410100	818,2780892	759,2884581	477,8042697	384,5573987	788,7832737	431,18083	0,546640438	-0,871336	8,467E-06	0,00032967	aldo-keto reductase, putative
PFIT_1228300	39570,22119	34821,87888	17326,69167	22857,42298	37196,05004	20092,057	0,540166424	-0,888524	9,167E-06	0,000354499	merozoite surface protein 9
PFIT_1300600	3329,037838	4328,183735	1652,196869	980,4541677	3828,610786	1316,3255	0,343812832	-1,540305	9,445E-06	0,000359588	Plasmodium exported protein (hyp12), unknown function
PFIT_0315200	534,235515	717,3719029	228,8430976	236,084803	625,803709	232,46395	0,371464641	-1,428703	9,543E-06	0,000359588	circumsporozoite- and TRAP-related protein
PFIT_0400500	10185,79614	8852,776471	5733,651236	5391,829127	9519,286307	5562,7402	0,584365256	-0,775058	9,425E-06	0,000359588	Plasmodium exported protein (PHISTb), unknown function
PFIT_0732700	2309,133672	2747,330793	4311,973971	4425,419664	2528,232233	4368,6968	1,727965003	0,789074	9,553E-06	0,000359588	Plasmodium exported protein (PHIST), unknown function, pseudogene
PFIT_0419300	1145,000636	1651,512277	336,9777481	373,856671	1398,256456	355,41721	0,254185996	-1,976044	9,84E-06	0,000367926	erythrocyte membrane protein 1, PfEMP1
PFIT_0414600	1671,877535	1337,736921	732,6332135	746,3757511	1504,807228	739,50448	0,491428051	-1,024948	1,033E-05	0,000383603	RNA binding protein, putative
PF_apicoplast_g	32848,86142	24481,6635	8954,219665	10659,93109	28665,26246	9807,0754	0,342124039	-1,547409	1,112E-05	0,000410522	unspecified product
PFIT_0302000	2007,43042	2319,78193	1372,220332	1170,392083	2163,606175	1271,3062	0,587586698	-0,767126	1,125E-05	0,000412607	serine/threonine protein kinase
PFIT_0109900	495,9707123	492,2201203	241,4168942	273,5373497	494,0954163	257,47712	0,521108097	-0,940345	1,147E-05	0,000417881	conserved Plasmodium protein, unknown function
PFIT_0418100	183,9653977	235,9303253	404,0379965	430,035491	209,9478615	417,03674	1,986382432	0,9901434	1,187E-05	0,000429441	conserved Plasmodium membrane protein, unknown function
PFIT_bin04200	73,5861591	104,1925802	28,50060556	20,06386428	88,88936966	24,282235	0,273173665	-1,87211	1,225E-05	0,000440364	B_ITvar33 - erythrocyte membrane protein 1, PfEMP1, putative
PFIT_1466600	133,9268096	158,0852941	290,8738273	317,0090556	146,0060518	303,94144	2,081704406	1,0577652	1,244E-05	0,000444483	potassium channel protein
PFIT_1129800	1532,063832	1789,238102	1010,933244	926,2817341	1660,650967	968,60749	0,583269759	-0,777765	1,303E-05	0,000462543	spermidine synthase
PFIT_1442300	3049,410433	3845,544541	1735,183927	1716,797987	3447,477487	1725,991	0,500653293	-0,998116	1,323E-05	0,000467	conserved Plasmodium protein, unknown function
PFIT_1438100	245,7777714	255,0921792	640,425372	497,5838341	250,4349753	569,0046	2,272065243	1,1840043	1,372E-05	0,000480992	histidine triad protein, putative
PFIT_1130200	2808,047831	3184,460584	1916,246597	1653,262416	2996,254208	1784,7545	0,595661911	-0,747434	1,461E-05	0,000506167	RNA methyltransferase, putative
PFIT_0502600	52,98203455	55,09032977	139,1500154	139,109459	54,03618216	139,12974	2,574751428	1,3644332	1,456E-05	0,000506167	conserved Plasmodium protein, unknown function
PFIT_1361800	1147,944082	1165,280236	544,026265	751,0573195	1156,612159	647,54179	0,559860786	-0,83686	1,49E-05	0,000512917	conserved Plasmodium protein, unknown function
PFIT_1431500	2953,748426	3390,450513	1745,242964	1999,698473	3172,099469	1872,4707	0,590293822	-0,760495	1,551E-05	0,000530851	acid phosphatase, putative
PFIT_0317500	1699,840275	1631,152808	2733,543375	2911,935502	1665,496541	2822,7394	1,694833564	0,7611436	1,564E-05	0,000532085	kinesin-5, putative
PFIT_0800100	278,1556814	353,2966801	158,4298368	151,816573	315,7261807	155,1232	0,491321957	-1,025259	1,613E-05	0,000545297	B_ITvar13 - erythrocyte membrane protein 1, PfEMP1, putative
PFIT_1205200	1030,206227	1010,78779	605,2187416	551,7562676	1020,497009	578,4875	0,566868398	-0,818914	1,653E-05	0,00055541	conserved Plasmodium protein, unknown function
PFIT_1430600	1882,33395	2050,31836	1175,230853	1132,270741	1966,326155	1153,7508	0,586754539	-0,769171	1,675E-05	0,000559722	rRNA (adenosine-2'-O-)-methyltransferase, putative
PFIT_0506300	313,4770378	287,4278075	130,7674843	161,1797097	300,4524226	145,9736	0,485845964	-1,041429	1,71E-05	0,000567801	conserved Plasmodium protein, unknown function
PFIT_1021400	4290,073075	3445,540842	2043,661069	1848,550695	3867,806959	1946,1059	0,503154863	-0,990926	1,747E-05	0,000573474	conserved Plasmodium protein (10b antigen), unknown function
PFIT_1314900	353,2135637	386,8299243	698,2648362	663,4451121	370,021744	680,85497	1,840040444	0,8797375	1,745E-05	0,000573474	1-methyladenosine tRNA methyltransferase subunit, putative
PFIT_0203000	434,1583387	574,855615	217,9458072	201,3074383	504,5069768	209,62662	0,415507877	-1,267052	1,922E-05	0,000627157	nucleolar preribosomal assembly protein, putative
PFIT_0536800	5716,172839	6347,364082	3721,843785	3556,654341	6031,76846	3639,2491	0,603346943	-0,72894	1,965E-05	0,000637764	erythrocyte membrane protein 1 (PfEMP1), pseudogene (VAR1CSA)
PFIT_1008400	1872,031887	2055,108824	1189,481156	1128,926763	1963,570356	1159,204	0,590355195	-0,760345	1,992E-05	0,000642647	adenylate kinase
PFIT_0722000	3598,36318	3324,58164	1974,924315	2102,024181	3461,47241	2038,4742	0,588903798	-0,763896	2,055E-05	0,000651728	conserved Plasmodium protein, unknown function
PFIT_0917000	883,0339092	907,7928254	1549,929991	1542,242367	895,4133673	1546,0862	1,726673105	0,787995	2,05E-05	0,000651728	conserved Plasmodium protein, unknown function
PFIT_0219800	247,2494946	211,978008	392,302453	471,5008105	229,6137513	431,90163	1,880992011	0,9114937	2,038E-05	0,000651728	DnaJ protein, putative, pseudogene
PFIT_0214300	5418,884756	5372,504769	3115,78679	3317,225561	5395,694762	3216,5062	0,596124562	-0,746314	2,106E-05	0,000663236	rhoptry neck protein 6 (RON6)
PFIT_0820400	4117,881463	4362,914595	2743,602412	2367,535985	4240,398029	2555,5692	0,602672009	-0,730555	2,114E-05	0,000663236	protein kinase c inhibitor-like protein, putative
PFIT_1030600	1501,157646	1505,403142	386,4346813	802,5545711	1503,280394	594,49463	0,395464897	-1,338378	2,186E-05	0,000681787	MORN repeat-containing protein 1
PFIT_1403800	684,3512796	691,0243539	368,8313661	391,9141489	687,6878167	380,37276	0,55311836	-0,85434	2,2E-05	0,000682633	conserved Plasmodium protein, unknown function
PFIT_0534700	117,7378546	111,3782754	228,0048445	254,1422808	114,558065	241,07356	2,104378794	1,0733944	2,229E-05	0,000687828	conserved Plasmodium membrane protein, unknown function
PFIT_1413200	384,1197505	409,5846257	782,9283998	662,1075212	396,8521881	722,51796	1,820622343	0,8644317	2,253E-05	0,000691395	conserved Plasmodium protein, unknown function
PFIT_0935800	3620,439028	3918,599109	2384,830083	2158,203001	3769,519068	2271,5165	0,602601154	-0,730725	2,305E-05	0,000703435	sec-1 family protein
PFIT_0212200	760,8808851	662,2815731	1259,056163	1192,462334	711,5812291	1225,7592	1,722585136	0,7845753	2,418E-05	0,000727892	multidrug efflux pump, putative
PFIT_1307500	391,4783664	446,7107175	704,9708611	800,5481847	419,0945419	752,75952	1,796156827	0,8449133	2,424E-05	0,000727892	40S ribosomal protein S27, putative
PFIT_0609600	172,1916123	239,5231729	394,8172123	410,6404222	205,8573926	402,72882	1,956348578	0,9681634	2,421E-05	0,000727892	conserved Plasmodium protein, unknown function
PFIT_0206500	317,8922073	300,601582	148,3707995	161,1797097	309,2468947	154,77525	0,500490893	-0,998584	2,574E-05	0,000768823	iron-sulfur assembly protein, putative
PFIT_1021900	2485,740454	3288,653164	904,4751	1251,985131	2887,196809	1078,2301	0,37345224	-1,421004	2,634E-05	0,000782643	pre-mRNA splicing factor, putative
PFIT_1456600	697,5967882	764,0789216	1141,700729	1376,381089	730,8378549	1259,0409	1,72273631	0,7847019	2,707E-05	0,00079843	hemolysin, putative
PFIT_1007400	153,0592109	166,4686052	344,522026	296,9451913	159,763908	320,73361	2,00754734	1,005434	2,715E-05	0,00079843	conserved Plasmodium protein, unknown function
PFIT_0201500	1024,319335	1262,287121	1996,718895	1928,806153	1143,303228	1962,7625	1,71674712	0,7796775	2,798E-05	0,000818569	Plasmodium exported protein, unknown function
PFIT_1365000	107,4357923	108,9830437	219,6223134	238,7599849	108,209418	229,19115	2,118033286	1,0827253	2,844E-05	0,000827698	conserved Plasmodium protein, unknown function
PFIT_1219000	204,5695223	234,7327095	85,50181668	121,0519811	219,6511159	103,2769	0,470186088	-1,088696	2,898E-05	0,000838941	erythrocyte membrane protein 1, PfEMP1
PFIT_1240400	441,5169546	438,3274064	226,3283383	242,1039623	439,9221805	234,21615	0,532403595	-0,909408	2,949E-05	0,000840924	C_ITvar5 - erythrocyte membrane protein 1, PfEMP1, putative
PFIT_0421800	3473,266709	3646,740308	2173,590301	2118,744068	3560,003509	2146,1672	0,602855356	-0,730116	2,941E-05	0,000840924	conserved Plasmodium protein, unknown function
PFIT_0318800	284,0425741	250,3017157	544,8645181	446,7553779	267,1721449	495,80995	1,855769613	0,8920176	2,945E-05	0,000840924	triosephophate isomerase, putative
PFIT_1218700	5443,90405	4583,275914	2907,90002	3056,395325	5013,589982	2982,1477	0,594812835	-0,749492	2,969E-05	0,000842238	formin 2
PFIT_1205900	1689,538213	1489,834136	861,7241917	979,7853722	1589,686174	920,75478	0,579205378	-0,787853	2,986E-05	0,000842994	conserved Plasmodium protein, unknown function
PFIT_0216100.1	1418,741147	1544,924465	805,5612337	931,632098	1481,832806	868,59667	0,586163744	-0,770624	3,054E-05	0,000857818	conserved Plasmodium protein, unknown function
PFIT_0206100	58116,87673	48734,58238	31938,28154	24669,85872	53425,72955	28304,07	0,529783503	-0,916525	3,42E-05	0,000955983	merozoite surface protein 2
PFIT_0412500	1189,152331	1020,368717	578,3946423	682,1713854	1104,760524	630,28301	0,57051551	-0,809662	3,521E-05	0,000979194	chromosome associated protein, putative
PFIT_1301300	11981,29842	14331,86905	8006,993656	6170,975856	13156,58374	7088,9848	0,538816527	-0,892134	3,547E-05	0,000981816	gamete antigen 27/25
PFIT_1468700	245,7777714	341,3205214	90,53133531	108,3448671	293,5491464	99,438101	0,338744304	-1,561731	3,571E-05	0,00098354	conserved Plasmodium protein, unknown function
PFIT_1008200	8325,53804	7992,88828	5001,856276	4923,672294	8159,21316	4962,7643	0,608240548	-0,717286	3,611E-05	0,000989714	tubulin beta chain
PFIT_1242100	3496,81428	3240,74853	2243,165308	1837,181172	3368,781405	2040,1732	0,605611643	-0,723535	3,683E-05	0,001004627	Tetratricopeptide repeat protein, putative
PFIT_1319700	273,7405118	232,3374777	140,8265216	109,0136626	253,0389948	124,92009	0,493679214	-1,018354	3,715E-05	0,001008485	SNARE protein, putative
PFIT_1241000	54,45375773	40,7189394	116,5171816	123,727163	47,58634856	120,12217	2,524299004	1,3358828	3,735E-05	0,001009034	Plasmodium RNA of unkown function RUF6-3
PFIT_0936500	144598,2743	163045,819	103723,7627	88355,23912	153822,0467	96039,501	0,62435459	-0,679562	3,807E-05	0,001023409	ring-exported protein 1 (REX1)
PFIT_1218300	2772,726475	3147,334492	1979,953833	1653,262416	2960,030483	1816,6081	0,613712641	-0,704365	4,028E-05	0,001077753	arginine--tRNA ligase, putative
PFIT_1428400	1713,085784	1622,769497	993,3299291	983,1293496	1667,92764	988,22964	0,592489515	-0,755138	4,117E-05	0,001096384	conserved Plasmodium protein, unknown function
PFIT_0729900	1551,196234	1361,689238	2474,523165	2334,096211	1456,442736	2404,3097	1,650809626	0,7231738	4,391E-05	0,001163792	unspecified product
PFIT_0208300	256,0798337	277,8468806	134,1204968	135,7654816	266,9633571	134,94299	0,505473825	-0,984292	4,516E-05	0,001191443	6-cysteine protein
PFIT_0705400	5286,42967	6774,912946	2970,769003	3055,057734	6030,671308	3012,9134	0,499598339	-1,001159	4,609E-05	0,001198683	conserved Plasmodium protein, unknown function
PFIT_1145900	13370,60511	12148,61533	7028,752283	8444,211679	12759,61022	7736,482	0,606325887	-0,721835	4,593E-05	0,001198683	serine/threonine protein kinase, putative
PFIT_1217200	1253,908151	1461,091355	2242,327055	2303,331619	1357,499753	2272,8293	1,67427606	0,7435374	4,65E-05	0,001198683	conserved Plasmodium protein, unknown function
PFIT_1452200	785,9001792	804,797861	1477,001971	1235,934039	795,3490201	1356,468	1,705500316	0,770195	4,643E-05	0,001198683	conserved Plasmodium protein, unknown function
PFIT_1431800	541,594131	386,8299243	791,3109309	795,8666163	464,2120276	793,58877	1,709539448	0,7736077	4,608E-05	0,001198683	exodeoxyribonuclease III, putative
PFIT_1452000	83,88822137	65,86887255	155,9150775	175,8932102	74,87854696	165,90414	2,215643206	1,1477256	4,759E-05	0,001221366	conserved Plasmodium protein, unknown function
PFIT_0813100	950,7331755	1044,321034	1619,504998	1720,141964	997,5271047	1669,8235	1,673963016	0,7432677	5,509E-05	0,0014075	ABC1 family, putative
PFIT_0602200	572,5003178	604,7960116	379,7286564	294,9388049	588,6481647	337,33373	0,573065119	-0,803229	5,579E-05	0,001412442	dihydroorotate dehydrogenase, mitochondrial precursor (DHODH)
PFIT_1331500	12533,19462	11868,37322	7053,899876	7907,168912	12200,78392	7480,5344	0,613119161	-0,705761	5,573E-05	0,001412442	conserved Plasmodium protein, unknown function
PFIT_1442500	1577,687251	1349,713079	2250,709586	2505,976648	1463,700165	2378,3431	1,624884094	0,7003368	5,789E-05	0,001459075	serine/threonine protein kinase
PFIT_1111200	14617,15464	17451,65838	10515,04695	9526,322759	16034,40651	10020,685	0,624948909	-0,67819	5,837E-05	0,00146461	asparagine-rich antigen
PFIT_1150100	64,75582001	76,64741533	19,27982141	26,08302356	70,70161767	22,681422	0,320804859	-1,640232	5,867E-05	0,001465702	Plasmodium exported protein (PHISTa), unknown, putative
PFIT_1208900	1246,549535	1251,508578	618,6307913	846,6950725	1249,029057	732,66293	0,586585979	-0,769586	6,106E-05	0,001516909	zinc finger transcription factor, putative (KROX1)
PFIT_0529900	216,3433077	185,630459	351,2280509	391,9141489	200,9868834	371,5711	1,84873308	0,8865369	6,126E-05	0,001516909	conserved Plasmodium protein, unknown function
PFIT_1417400	66,22754319	83,83311052	172,6801396	165,1924826	75,03032685	168,93631	2,251573706	1,1709337	6,372E-05	0,001570895	conserved Plasmodium protein, unknown function
PFIT_1477300	1527,648663	2098,222995	759,4573129	594,5591781	1812,935829	677,00825	0,373431996	-1,421083	6,406E-05	0,001572575	Plasmodium exported protein (PHISTb), unknown function
PFIT_1318100	110,3792386	91,01880571	254,8289438	196,6258699	100,6990222	225,72741	2,241604754	1,1645319	6,463E-05	0,001579761	conserved Plasmodium protein, unknown function
PFIT_1351200	1195,039224	1172,465931	635,3958534	759,7516606	1183,752578	697,57376	0,589290169	-0,76295	6,66E-05	0,001609384	conserved Plasmodium protein, unknown function
PFIT_1223500	863,9015078	1059,89004	565,8208457	573,8265183	961,895774	569,82368	0,592396492	-0,755365	6,622E-05	0,001609384	mitochondrial carrier protein, putative
PFIT_1360500.1	3605,721796	3329,372104	2392,374361	1888,678424	3467,54695	2140,5264	0,617302786	-0,69595	6,67E-05	0,001609384	conserved Plasmodium protein, unknown function
PFIT_0707400	2498,985963	2312,596235	1668,123678	1285,424905	2405,791099	1476,7743	0,613841448	-0,704062	6,783E-05	0,001629602	heat shock protein 86 family protein
PFIT_0709100	3221,602045	2932,961252	1622,858011	2085,973089	3077,281649	1854,4156	0,602614828	-0,730692	6,835E-05	0,001635289	conserved Plasmodium protein, unknown function
PFIT_0724700	138,3419791	134,1329768	265,7262342	270,1933723	136,237478	267,9598	1,966858219	0,975893	6,949E-05	0,001655511	unspecified product
PFIT_1021600	286,9860205	326,949131	516,3639125	578,5080867	306,9675758	547,436	1,783367505	0,834604	7,098E-05	0,001683946	conserved Plasmodium protein, unknown function
PFIT_0306600	214,8715846	275,4536489	414,9352868	483,5391291	245,1616167	449,23721	1,832412488	0,8737443	7,206E-05	0,001702217	membrane magnesium transporter, putative
PFIT_1216200	295,8163596	316,1705883	174,3566458	151,1477776	305,9934739	162,75221	0,531881316	-0,910824	7,387E-05	0,001737719	male development gene 1
PFIT_0310600	423,8562764	453,8964127	733,4714466	781,8219113	438,8763445	757,64669	1,726332937	0,7877107	7,52E-05	0,001761857	sybindin-like protein, putative
PFIT_1311900	214,8715846	188,0256907	377,2138971	366,4999208	201,4486377	371,85691	1,84591424	0,8843355	7,582E-05	0,00176884	probable protein, unknown function
PFIT_1335300	222,2302005	263,4754902	119,870194	127,7399359	242,8528453	123,80506	0,509794583	-0,972012	7,635E-05	0,001774012	conserved Plasmodium protein, unknown function
PFIT_0213500	18732,09266	18589,39345	13247,75207	10334,22769	18660,74305	11790,99	0,631860685	-0,662322	7,683E-05	0,001777731	protein transport protein sec31
PFIT_0817100	3655,760384	4301,836186	1445,148353	2273,235823	3978,798285	1859,1921	0,467274779	-1,097657	7,842E-05	0,001807296	DNA/RNA-binding protein Alba 1 (ALBA1)
PFIT_0422900	10278,5147	13549,82589	5838,432874	6444,814733	11914,1703	6141,6238	0,431555339	-1,212383	8,225E-05	0,001886944	serine/threonine protein kinase, FIKK family
PFIT_1223900	713,7857432	573,6579991	1038,595597	1091,474217	643,7218712	1065,0349	1,65449545	0,7263913	8,292E-05	0,001889442	conserved Plasmodium protein, unknown function
PFIT_1128000	94,19028364	102,9949644	188,6069486	220,0337116	98,592624	204,32033	2,072369329	1,0512811	8,299E-05	0,001889442	deoxyuridine 5'-triphosphate nucleotidohydrolase
PFIT_1408300	4154,674543	4734,175513	2703,366263	2853,0815	4444,425028	2778,2239	0,625103104	-0,677834	8,537E-05	0,001935682	pre-mRNA splicing factor, putative
PFIT_1035600	82,41649819	119,7615865	33,53012419	28,75820546	101,0890423	31,144165	0,308086456	-1,698593	8,62E-05	0,001938879	liver stage antigen 1
PFIT_0902200	713,7857432	831,14541	1348,749246	1250,64754	772,4655766	1299,6984	1,682532442	0,7506343	8,615E-05	0,001938879	serine/threonine protein kinase, FIKK family
PFIT_1207300	107,4357923	85,03072639	36,88313661	36,1149557	96,23325933	36,499046	0,379276836	-1,398677	8,735E-05	0,001946063	blood stage antigen 41-3 precursor
PFIT_0526200	213,3998614	240,7207888	137,4735092	96,30654853	227,0603251	116,89003	0,514797241	-0,957924	8,755E-05	0,001946063	conserved Plasmodium protein, unknown function
PFIT_0418800	80,94477501	104,1925802	179,3861644	211,3393704	92,56867761	195,36277	2,110462982	1,0775595	8,707E-05	0,001946063	holo-(acyl-carrier protein) synthase, putative
PFIT_0421600	1127,339957	966,4760027	551,5705429	673,4770442	1046,90798	612,52379	0,585078923	-0,773297	8,921E-05	0,001967542	asparagine-rich protein
PFIT_1126800	236,9474323	256,289795	424,9943241	465,4816512	246,6186137	445,23799	1,805370572	0,852295	8,913E-05	0,001967542	conserved Plasmodium protein, unknown function
PFIT_1318200	242,834325	273,0564171	138,3117623	130,4151178	257,9453711	134,36344	0,520898822	-0,940925	9,125E-05	0,002000011	conserved Plasmodium protein, unknown function
PFIT_0219600	1726,331292	1724,566845	2821,559951	2768,81327	1725,449069	2795,1866	1,619976307	0,6959727	9,139E-05	0,002000011	Plasmodium exported protein, unknown function
PFIT_0113700	89,7751141	99,40211676	182,7391768	210,6705749	94,58861543	196,70488	2,079582992	1,0562943	9,574E-05	0,002086991	Plasmodium exported protein, unknown function, pseudogene
PFIT_0721600	3721,987927	4018,001226	1974,924315	2673,844313	3869,994576	2324,3843	0,600616943	-0,735483	9,744E-05	0,002109051	conserved Plasmodium protein, unknown function
PFIT_1228500	5975,196119	5907,83906	3238,171744	4111,08579	5941,517589	3674,6288	0,618466362	-0,693233	9,75E-05	0,002109051	conserved Plasmodium protein, unknown function
PFIT_1244100	819,7498123	738,9289885	467,7452324	445,417787	779,3394004	456,58151	0,585857085	-0,771379	0,0001011	0,002170989	double C2-like domain-containing protein (DOC2)
PFIT_0710600	1215,643348	1286,239439	801,3699681	723,6367049	1250,941393	762,50334	0,609543613	-0,714199	0,0001008	0,002170989	erythrocyte membrane protein 1, PfEMP1, putative
PFIT_1135900	285,5142973	258,6850268	435,8916145	516,9789029	272,099662	476,43526	1,750958657	0,808145	0,000111	0,002372945	conserved Plasmodium protein, unknown function, pseudogene
PFIT_0111300	78,00132864	67,06648842	145,8560402	167,198869	72,53390853	156,52745	2,157990074	1,1096882	0,0001154	0,002458392	Plasmodium exported protein, unknown function
PFIT_1473900	603,4065046	544,9152184	359,6105819	304,3019415	574,1608615	331,95626	0,578158986	-0,790462	0,0001167	0,002467796	conserved Plasmodium protein, unknown function
PFIT_0508900	3471,794986	3170,089194	1875,172195	2213,713025	3320,94209	2044,4426	0,615621277	-0,699885	0,0001165	0,002467796	conserved Plasmodium protein, unknown function
PFIT_0309900	354,6852868	306,5896613	171,8418865	182,5811649	330,6374741	177,21153	0,53596927	-0,899778	0,0001227	0,002584285	conserved Plasmodium protein, unknown function
PFIT_1034700	42659,36815	37476,99325	26189,54175	18799,84083	40068,1807	22494,691	0,561410348	-0,832872	0,0001245	0,002613757	glutamate-rich protein
PFIT_1461200	14,71723182	13,17377451	54,48645181	50,82845617	13,94550316	52,657454	3,775945075	1,9168378	0,0001301	0,002719873	biotin protein ligase, putative
PFIT_1456200	123,6247473	117,3663547	67,89850148	38,7901376	120,495551	53,34432	0,442707794	-1,175573	0,0001308	0,002724362	protein phosphatase, putative
PFIT_0511400	699,0685114	729,3480615	403,1997434	440,0674232	714,2082865	421,63358	0,59035101	-0,760355	0,000134	0,002781436	MORN repeat protein, putative
PFIT_1417600	596,0478887	531,7414439	356,2575695	298,2827823	563,8946663	327,27018	0,580374661	-0,784944	0,0001368	0,002828955	conserved Plasmodium protein, unknown function
PFIT_1366000	1090,546878	1035,937723	741,8539977	523,6668576	1063,2423	632,76043	0,595123452	-0,748739	0,0001391	0,002867148	suppressor of kinetochore protein 1, putative
PFIT_0217700	267,8536191	261,0802585	452,6566766	476,1823789	264,4669388	464,41953	1,756058923	0,8123413	0,0001417	0,002909721	apicoplast RNA methyltransferase precursor, putative
PFIT_1108100	21314,96684	23661,29664	15019,81913	13934,35374	22488,13174	14477,086	0,643765636	-0,635393	0,000143	0,002924519	polyadenylate-binding protein-interacting protein 1, putative
PFIT_0723800	375576,3974	154529,5726	5412,600297	3260,377945	265052,985	4336,4891	0,016360839	-5,933609	0,0001436	0,002926889	18S ribosomal RNA
PFIT_1103800	8,830339092	23,95231729	51,13343939	66,21075212	16,39132819	58,672096	3,579459521	1,8397418	0,0001477	0,002999509	conserved Plasmodium protein, unknown function
PFIT_0531200	5647,001849	4820,403855	3009,328646	3496,462748	5233,702852	3252,8957	0,621528541	-0,686107	0,0001484	0,003002667	DNA replication licensing factor MCM3, putative (MCM3)
PFIT_0417600	92,71856046	105,3901961	41,91265524	39,45893308	99,05437827	40,685794	0,410742007	-1,283696	0,0001496	0,003006375	conserved Plasmodium protein, unknown function
PFIT_0833200	89,7751141	88,62357398	212,0780355	162,5173006	89,19934404	187,29767	2,099765083	1,0702279	0,0001496	0,003006375	sporozoite invasion-associated protein 2 (SIAP-2)
PFIT_1338000	103,0206227	102,9949644	46,94217387	38,7901376	103,0077935	42,866156	0,416144781	-1,264843	0,0001521	0,003044499	CPW-WPC family protein
PFIT_1104900	519,5182832	594,0174688	890,2247972	965,7406672	556,7676876	927,98273	1,666731814	0,737022	0,0001556	0,0031047	syntaxin, Qa-SNARE family
PFIT_1355200	3262,810294	2552,119407	1563,34204	1391,763385	2907,464851	1477,5527	0,508192803	-0,976552	0,0001586	0,003147893	conserved Plasmodium protein, unknown function
PFIT_0612900	7321,82283	7697,077162	4680,805337	4884,882156	7509,449996	4782,8437	0,636909993	-0,650839	0,0001589	0,003147893	myosin E, putative
PFIT_0403200	272,2687887	408,3870098	732,6332135	630,6741338	340,3278992	681,65367	2,002932099	1,0021135	0,0001599	0,003155865	6-cysteine protein
PFIT_0723900	389950,7177	158672,1259	5472,954521	3375,410767	274311,4218	4424,1826	0,016128321	-5,95426	0,0001662	0,00326262	18S ribosomal RNA
PFIT_1320900	1047,866906	1155,699309	447,6271579	696,2160904	1101,783107	571,92162	0,519087305	-0,945951	0,0001664	0,00326262	splicing factor, putative
PFIT_1235200	588,6892728	629,9459448	341,1690136	376,5318529	609,3176088	358,85043	0,588938229	-0,763812	0,0001703	0,003322641	mRNA methyltransferase, putative
PFIT_1222200	1240,662642	1443,127117	892,7395565	789,1786616	1341,89488	840,95911	0,626695222	-0,674164	0,0001718	0,003343962	ATP-binding protein, putative
PFIT_0704400	51386,68662	56428,06669	33366,66483	36071,48399	53907,37666	34719,074	0,644050528	-0,634754	0,0001775	0,003444028	inositol-phosphate phosphatase, putative
PFIT_1459900	1136,170296	973,6616979	678,1467617	605,9287012	1054,915997	642,03773	0,60861503	-0,716398	0,0001879	0,003633766	exonuclease V, mitochondrial, putative
PFIT_1200600	921,2987119	1112,585138	1580,107102	1710,778827	1016,941925	1645,443	1,618030415	0,6942387	0,0001935	0,003728536	Plasmodium exported protein (PHISTc), unknown function
PFIT_1358000	1217,115071	1390,432019	824,002802	805,8985485	1303,773545	814,95068	0,625070725	-0,677909	0,0001959	0,003762529	GTP binding protein, putative
PFIT_1148900	2415,097742	3225,179523	1375,573345	864,0837549	2820,138632	1119,8285	0,397082802	-1,332488	0,0001971	0,003771636	Plasmodium exported protein (PHISTc), unknown function
PFIT_0934400	1320,135694	1425,162879	921,2401621	604,5911102	1372,649287	762,91564	0,555797933	-0,847368	0,0002033	0,003878475	conserved Plasmodium protein, unknown function
PFIT_0833300	384,1197505	492,2201203	707,4856204	769,7835928	438,1699354	738,63461	1,685726352	0,7533704	0,0002084	0,003961105	conserved Plasmodium protein, unknown function
PFIT_0111000	162303,1042	75894,11495	3540,781114	5683,423954	119098,6096	4612,1025	0,038725075	-4,690588	0,0002121	0,004018398	28S ribosomal RNA
PFIT_1034900	25915,57351	22479,24978	16888,2853	13789,89392	24197,41164	15339,09	0,63391448	-0,65764	0,0002142	0,004027249	merozoite surface protein 6
PFIT_1356300	211,9281382	197,6066177	373,0226316	356,4679887	204,7673779	364,74531	1,781266693	0,8329035	0,0002147	0,004027249	glutamate--tRNA ligase, putative
PFIT_0628800	803,5608573	1114,98037	1596,872165	1826,480445	959,2706136	1711,6763	1,784351861	0,8354001	0,0002142	0,004027249	sec14-like cytosolic factor or phosphatidylinositol/phosphatidylcholine transfer protein, putative
PFIT_0417200	11238,07822	10433,62941	7431,952027	6540,150959	10835,85381	6986,0515	0,644716292	-0,633264	0,0002281	0,004265199	regulator of chromosome condensation, putative
PFIT_1420400	3549,796315	3735,363882	5547,559047	5814,507868	3642,580098	5681,0335	1,559617992	0,6411927	0,0002299	0,004284231	conserved Plasmodium membrane protein, unknown function
PFIT_1203600	9441,104212	9095,892492	6400,900708	4300,35491	9268,498352	5350,6278	0,577291769	-0,792627	0,0002312	0,004293366	ubiquitin conjugating enzyme E2 (UBC)
PFIT_1440800	796,2022414	747,3122995	539,8349995	377,8694439	771,7572705	458,85222	0,594555101	-0,750118	0,000234	0,004332406	conserved Plasmodium protein, unknown function
PFIT_0202400	1147,944082	1132,944608	642,9401313	757,7452742	1140,444345	700,3427	0,614096344	-0,703463	0,0002376	0,00438304	protein kinase, putative
PFIT_0203400	3086,203513	2983,261119	1972,409555	1892,691197	3034,732316	1932,5504	0,636810821	-0,651063	0,0002384	0,004383962	conserved Plasmodium protein, unknown function
PFIT_0729400	3941,274681	3425,181373	2124,971621	2506,645444	3683,228027	2315,8085	0,628744274	-0,669455	0,0002435	0,004463003	conserved Plasmodium protein, unknown function
PFIT_0906300	432,6866155	379,6442291	265,7262342	202,6450292	406,1654223	234,18563	0,576576978	-0,794415	0,0002509	0,004584575	developmental protein, putative
PFIT_1019500	1794,030559	1887,442603	1161,818803	1170,392083	1840,736581	1166,1054	0,633499358	-0,658585	0,0002557	0,004657882	large subunit rRNA processing RRM protein, putative
PFIT_0601100	392,9500896	423,9560161	251,4759314	224,0464844	408,4530528	237,76121	0,582101679	-0,780657	0,0002608	0,004734455	MYND finger protein, putative
PFIT_bin06200	334,0811623	394,0156194	197,8277327	218,6961206	364,0483909	208,26193	0,572072098	-0,805731	0,0002624	0,00473487	B_ITvar54 - erythrocyte membrane protein 1, PfEMP1, putative
PFIT_1413700	925,7138814	996,4163993	638,7488658	559,1130179	961,0651404	598,93094	0,62319495	-0,682245	0,0002625	0,00473487	AAA family ATPase, putative
PFIT_1410600	4263,582058	4023,989305	2982,504547	2160,878183	4143,785682	2571,6914	0,62061399	-0,688232	0,0002738	0,004923325	conserved Plasmodium protein, unknown function
PFIT_0706400	3287,829588	3010,806284	5290,215344	4487,617643	3149,317936	4888,9165	1,552373115	0,6344754	0,0002751	0,004930741	conserved Plasmodium protein, unknown function
PFIT_1410700	185,4371209	137,7258244	265,7262342	310,3211008	161,5814727	288,02367	1,78252904	0,8339256	0,0002782	0,004966819	conserved Plasmodium protein, unknown function
PFIT_0722700	79,47305183	85,03072639	162,6211023	173,8868237	82,25188911	168,25396	2,045593905	1,0325198	0,0002789	0,004966819	conserved Plasmodium protein, unknown function
PFIT_1148800	379,7045809	307,7872772	549,0557836	588,5400188	343,7459291	568,7979	1,6547044	0,7265735	0,0002836	0,005019915	unspecified product
PFIT_0218600	656,3885391	813,1811721	1199,540193	1482,71957	734,7848556	1341,1299	1,825200766	0,8680552	0,000283	0,005019915	40S ribosomal protein S30, putative
PFIT_0828500	14,71723182	27,54516489	65,38374217	65,54195664	21,13119835	65,462849	3,097924136	1,6313018	0,0002869	0,005062303	cytochrome c oxidase assembly protein, putative
PFIT_1139800	2868,388482	3330,569719	5237,405398	4498,318371	3099,479101	4867,8619	1,570541929	0,6512625	0,0002999	0,00527496	RNA binding protein, putative
PFIT_bin04300	425,3279996	536,5319073	272,432259	284,9068727	480,9299535	278,66957	0,579438989	-0,787271	0,0003041	0,005332831	erythrocyte membrane protein 1, PfEMP1 (VAR)
PFIT_0811900	30610,37046	43087,82358	12125,33116	15713,34971	36849,09702	13919,34	0,377738983	-1,404538	0,0003081	0,005385497	erythrocyte membrane protein 1, PfEMP1
PFIT_1440700	24404,1138	21772,65642	14656,01728	8952,496241	23088,38511	11804,257	0,511263854	-0,96786	0,0003159	0,005489992	CCAAT-binding transcription factor, putative
PFIT_1144700	694,6533419	924,5594475	1386,470635	1709,441236	809,6063947	1547,9559	1,911985807	0,9350718	0,000316	0,005489992	40S ribosomal protein S21e, putative
PFIT_0535600	1250,964705	1258,694274	859,2094324	729,6558642	1254,829489	794,43265	0,633100079	-0,659495	0,000321	0,005558662	conserved Plasmodium protein, unknown function
PFIT_1421100	303,1749755	274,254033	154,2385713	163,1860961	288,7145042	158,71233	0,54972068	-0,863229	0,0003226	0,005569961	conserved Plasmodium protein, unknown function
PFIT_1366100	1823,465022	1935,347237	1178,583865	1223,226925	1879,40613	1200,9054	0,638981312	-0,646154	0,0003334	0,005738406	U1 small nuclear ribonucleoprotein, putative
PFIT_1127600	16724,66224	14081,56734	10510,01743	9158,485247	15403,11479	9834,2513	0,638458615	-0,647335	0,0003357	0,005749111	conserved Plasmodium protein, unknown function
PFIT_0926200	7823,680435	8491,09648	12307,23208	12763,96166	8157,388458	12535,597	1,536716921	0,6198514	0,000336	0,005749111	protein kinase, putative
PFIT_1403100	1227,417134	1167,675468	751,0747818	748,3821376	1197,546301	749,72846	0,62605384	-0,675641	0,0003385	0,005768431	conserved Plasmodium protein, unknown function
PFIT_0731900	19478,25631	19463,65303	12107,72784	13250,17597	19470,95467	12678,952	0,651172586	-0,618888	0,0003392	0,005768431	erythrocyte binding like protein 1, pseudogene
PFIT_1405700	1250,964705	1340,132152	2172,752047	1918,105425	1295,548429	2045,4287	1,578813027	0,6588403	0,0003406	0,005774403	conserved Plasmodium protein, unknown function
PFIT_bin05750	32,37791	32,33562834	93,88434773	74,23629783	32,35676917	84,060323	2,597920773	1,3773574	0,0003529	0,005965208	rifin (RIF)
PFIT_0506000	116,2661314	143,7139038	244,7699066	244,7791442	129,9900176	244,77453	1,883025558	0,9130526	0,0003541	0,005968028	conserved Plasmodium protein, unknown function
PFIT_1112900	3596,891457	3223,981907	2049,528841	2308,013187	3410,436682	2178,771	0,63885397	-0,646442	0,0003642	0,006119304	conserved Plasmodium protein, unknown function
PFIT_bin10700	228,1170932	265,8707219	128,252725	142,4534364	246,9939076	135,35308	0,548001698	-0,867748	0,0003665	0,006140573	erythrocyte membrane protein 1, PfEMP1
PFIT_1307800	95,66200683	86,22834225	171,8418865	234,0784166	90,94517454	202,96015	2,23167587	1,1581225	0,0003688	0,006161171	conserved Plasmodium protein, unknown function
PFIT_1119500	1077,301369	1056,297193	496,245838	734,3174326	1066,799281	615,29164	0,576764201	-0,793946	0,0003724	0,006202898	conserved Plasmodium protein, unknown function
PFIT_1464600	757,9374387	810,7859403	1197,025434	1291,444064	784,3616895	1244,2347	1,586302296	0,6656677	0,0003815	0,006334557	conserved Plasmodium protein, unknown function
PFIT_1120800	2372,417769	2401,219808	1479,51673	1587,72046	2386,818789	1533,6186	0,642536669	-0,638149	0,0003827	0,006335822	conserved Plasmodium protein, unknown function
PFIT_1413500	1730,746462	2264,6916	1103,141086	976,4413948	1997,719031	1039,7912	0,52048923	-0,94206	0,0003877	0,006382448	actin II
PFIT_1033300	1317,192248	1397,617714	927,1079338	811,9177078	1357,404981	869,51282	0,640569935	-0,642572	0,0003874	0,006382448	ubiquitin conjugating enzyme, putative
PFIT_1316600	210,456415	217,9660874	128,252725	102,9945033	214,2112512	115,62361	0,539764431	-0,889598	0,0003906	0,006410168	conserved Plasmodium protein, unknown function
PFIT_0602300	593,1044423	714,9766712	327,756964	422,0099453	654,0405567	374,88345	0,573180747	-0,802938	0,0003958	0,006477089	trophozoite exported protein 1 (TEX1)
PFIT_0802000	1952,976662	1731,75254	2581,819563	3034,99387	1842,364601	2808,4067	1,524349043	0,6081933	0,0003993	0,006515138	unspecified product
PFIT_0529200	871,2601237	798,8097817	553,2470491	484,2079246	835,0349527	518,72749	0,62120452	-0,68686	0,0004061	0,006608119	zinc finger protein, putative
PFIT_1124000	4881,705795	5231,186097	2987,534065	3592,100501	5056,445946	3289,8173	0,650618501	-0,620116	0,00041	0,006652152	calcium-dependent protein kinase 7
PFIT_1108600	23831,61349	23088,83625	15151,42487	15616,37436	23460,22487	15383,9	0,655743911	-0,608796	0,0004113	0,00665441	transcription factor with AP2 domain(s)
PFIT_0536100	1355,457051	1888,640218	619,4690444	732,3310461	1622,048635	675,90005	0,416695302	-1,262935	0,0004167	0,006694996	Plasmodium exported protein (PHISTb), unknown function
PFIT_0517300	2312,077119	2398,824577	3554,193164	3691,751027	2355,450848	3622,9721	1,538122563	0,6211705	0,0004157	0,006694996	conserved Plasmodium protein, unknown function
PFIT_0930400	1364,28739	1213,184871	2596,908118	2046,514156	1288,73613	2321,7111	1,801541125	0,8492316	0,0004174	0,006694996	leucine-rich repeat protein
PFIT_0513700	1167,076483	1004,79971	632,042841	714,9423638	1085,938097	673,4926	0,620194286	-0,689208	0,000419	0,006701085	deoxyribodipyrimidine photo-lyase, putative
PFIT_0405200	51,51031137	40,7189394	13,41204968	13,37590952	46,11462538	13,39398	0,290449711	-1,78364	0,000428	0,006827027	conserved Plasmodium protein, unknown function
PFIT_1436900.1	294,3446364	226,3493984	133,2822437	136,4342771	260,3470174	134,85826	0,517994259	-0,948992	0,0004347	0,006913408	ataxin-2 like protein, putative
PFIT_1335900	5657,303911	6244,369118	4437,711936	3495,793953	5950,836515	3966,7529	0,666587451	-0,585134	0,0004388	0,006958716	conserved Plasmodium protein, unknown function
PFIT_1345600	17139,68818	17457,64646	10175,55444	12455,64694	17298,66732	11315,601	0,654131355	-0,612348	0,0004424	0,006978146	conserved Plasmodium protein, unknown function
PFIT_1009600	295,8163596	312,5777407	522,2316843	498,2526296	304,1970501	510,24216	1,67734091	0,7461759	0,0004425	0,006978146	conserved Plasmodium protein, unknown function
PFIT_1223000	719,672636	743,7194519	1172,716094	1146,984241	731,6960439	1159,8502	1,585152984	0,6646221	0,0004449	0,006996743	DNA gyrase subunit A
PFIT_0418900	19394,36809	21560,67841	13149,67645	13985,85099	20477,52325	13567,764	0,662568591	-0,593858	0,0004465	0,007002034	transcription factor with AP2 domain(s)
PFIT_1314300	64,75582001	86,22834225	164,2976085	148,4725957	75,49208113	156,3851	2,071543131	1,0507059	0,000448	0,007006322	serine/threonine protein kinase
PFIT_0725900	547,4810237	547,3104501	302,6093708	355,7991932	547,3957369	329,20428	0,601400888	-0,733601	0,0004562	0,0071151	vacuolar protein sorting-associated protein 53, putative (VPS53)
PFIT_0801900	2434,230143	2578,466956	4140,132084	3600,126047	2506,34855	3870,1291	1,544130431	0,6267946	0,0004593	0,007143061	acyl-CoA synthetase
PFIT_0402700	185,4371209	142,5162879	264,049728	308,3147144	163,9767044	286,18222	1,745261452	0,8034432	0,0004666	0,00723672	conserved Plasmodium protein, unknown function
PFIT_1477000	50,03858819	70,65933601	19,27982141	24,07663713	60,3489621	21,678229	0,359214616	-1,477082	0,0004754	0,007353531	conserved Plasmodium protein, unknown function
PFIT_0823300	150,1157646	156,8876783	269,0792466	282,2316908	153,5017214	275,65547	1,795780961	0,8446114	0,0004782	0,007377446	conserved Plasmodium protein, unknown function
PFIT_1471600	362,0439028	400,0036988	194,4747203	250,129508	381,0238008	222,30211	0,583433669	-0,777359	0,0004826	0,007423998	phosphodiesterase delta, putative
PFIT_0406900	1501,157646	1558,09824	1109,008858	880,1348463	1529,627943	994,57185	0,650205075	-0,621033	0,0004883	0,007471216	zinc finger, RAN binding protein, putative
PFIT_1001500	3558,626654	4231,17685	6165,351585	5828,552573	3894,901752	5996,9521	1,539692773	0,6226425	0,0004886	0,007471216	Plasmodium exported protein (hyp16), unknown function
PFIT_0721400	33,84963318	44,31178699	82,98705737	104,3320942	39,08071009	93,659576	2,396567913	1,2609698	0,0004896	0,007471216	conserved Plasmodium protein, unknown function
PFIT_1354900	33,84963318	34,73086007	77,95753874	113,0264354	34,29024663	95,491987	2,784814823	1,4775814	0,0004995	0,007601725	splicing factor 3a subunit 5, putative
PFIT_1343500	21095,68009	19662,45726	15283,03061	11649,0796	20379,06868	13466,055	0,660778729	-0,597761	0,000504	0,007649266	conserved protein, unknown function
PFIT_1150900	158,9461037	184,4328431	79,63404495	97,64413948	171,6894734	88,639092	0,516275637	-0,953787	0,0005068	0,007650714	A_ITvar8 - erythrocyte membrane protein 1, PfEMP1, putative
PFIT_0704600	445,9321241	534,1366756	805,5612337	1027,938646	490,0343999	916,74994	1,870786909	0,9036452	0,0005061	0,007650714	40S ribosomal protein S29, putative
PFIT_1343000	12472,85397	10760,57854	7682,589705	7533,312241	11616,71626	7607,951	0,654914074	-0,610622	0,0005086	0,007657785	conserved Plasmodium protein, unknown function
PFIT_1357800	1002,243487	953,3022282	1420,000759	1600,427574	977,7728576	1510,2142	1,544544988	0,6271819	0,0005145	0,007720918	HVA22-like protein, putative
PFIT_0219300	740,2767605	857,492959	1285,880263	1243,29079	798,8848598	1264,5855	1,582938406	0,6626051	0,0005169	0,007720918	Plasmodium exported protein, unknown function
PFIT_1351000.2	422,3845532	446,7107175	771,1928564	647,3940207	434,5476354	709,29344	1,632257043	0,7068683	0,000516	0,007720918	conserved Plasmodium protein, unknown function
PFIT_1002300	840,3539369	717,3719029	479,4807759	482,8703336	778,8629199	481,17555	0,617792351	-0,694806	0,0005314	0,007888255	conserved Plasmodium protein, unknown function
PFIT_0628600	10433,04564	12180,95096	6924,808898	8099,782009	11306,9983	7512,2955	0,664393436	-0,58989	0,0005307	0,007888255	SET domain protein, putative
PFIT_1121100	289,9294668	256,289795	421,6413117	481,5327427	273,1096309	451,58703	1,653500924	0,7255239	0,0005323	0,007888255	metal ion channel - Mg2 , Co2 and Ni2
PFIT_1032600	1964,750448	2043,132665	1388,985395	1232,590062	2003,941556	1310,7877	0,654104769	-0,612406	0,0005385	0,007958923	early transcribed membrane protein 10.2
PFIT_bin11100	562,1982555	774,8574644	307,6388894	306,9771234	668,5278599	307,30801	0,459678683	-1,121102	0,0005431	0,008006251	erythrocyte membrane protein 1, PfEMP1 (IT4_var30)
PFIT_1021200	30965,05575	27734,38819	20954,65111	18002,63662	29349,72197	19478,644	0,66367388	-0,591454	0,0005459	0,008027064	conserved Plasmodium membrane protein, unknown function
PFIT_1132700	313,4770378	316,1705883	507,1431284	531,0236079	314,823813	519,08337	1,648805925	0,7214216	0,0005495	0,008046582	conserved Plasmodium protein, unknown function
PFIT_1149600	35,32135637	29,94039661	82,14880426	80,25545711	32,63087649	81,202131	2,488505962	1,3152798	0,0005501	0,008046582	Plasmodium exported protein, unknown function
PFIT_0517200	297,2880828	346,1109849	545,7027712	525,0044486	321,6995338	535,35361	1,664141703	0,7347783	0,0005548	0,008093523	conserved Plasmodium protein, unknown function
PFIT_0916700	58,86892728	75,44979947	122,3849533	155,8293459	67,15936337	139,10715	2,071299408	1,0505361	0,0005593	0,008138881	methyltransferase, putative
PFIT_1362900	575,4437641	441,920254	791,3109309	961,0590989	508,6820091	876,18501	1,722461182	0,7844715	0,0005656	0,008208784	unspecified product
PFIT_1245500	55,92548091	80,24026293	28,50060556	25,41422808	68,08287192	26,957417	0,395950054	-1,33661	0,0005769	0,008334934	ubiquitin conjugating enzyme E2, putative
PFIT_0406700	1080,244816	1069,470967	1545,738725	1758,932102	1074,857891	1652,3354	1,537259415	0,6203606	0,0005772	0,008334934	conserved Plasmodium protein, unknown function
PFIT_0318500	7704,470857	7494,680081	4385,740244	5573,072701	7599,575469	4979,4065	0,655221662	-0,609945	0,0006061	0,008730083	conserved Plasmodium protein, unknown function
PFIT_1351000.1	541,594131	499,4058155	838,2531047	809,9113213	520,4999732	824,08221	1,583251211	0,6628902	0,0006156	0,008843342	conserved Plasmodium protein, unknown function
PFIT_1006600	273,7405118	334,1348262	167,6506209	186,5939378	303,937669	177,12228	0,582758563	-0,77903	0,0006485	0,00922315	conserved membrane protein, unknown function
PFIT_1211400	24673,43915	24503,22059	18724,89785	13894,89481	24588,32987	16309,896	0,663318591	-0,592226	0,0006473	0,00922315	polyubiquitin
PFIT_0822900	6007,574029	6303,052295	4129,234794	4073,633244	6155,313162	4101,434	0,666324184	-0,585704	0,0006471	0,00922315	Snf2-related CBP activator, putative
PFIT_1469700	210,456415	192,8161542	301,7711177	379,8758303	201,6362846	340,82347	1,690288406	0,7572694	0,0006449	0,00922315	aminophospholipid transporter, putative
PFIT_0706200	34734,13882	30457,76667	16562,20484	22335,09371	32595,95274	19448,649	0,596658408	-0,745023	0,0006602	0,009364977	rhoptry-associated membrane antigen
PFIT_1133800	2281,170932	2664,695299	1611,96072	1665,969531	2472,933115	1638,9651	0,662761607	-0,593438	0,0006683	0,009456257	conserved membrane protein, unknown function
PFIT_0926000	7652,960546	8559,360584	12879,75895	11658,44274	8106,160565	12269,101	1,513552655	0,5979389	0,0006759	0,009517136	conserved Plasmodium protein, unknown function
PFIT_1452100	105,9640691	108,9830437	192,7982141	207,995393	107,4735564	200,3968	1,864614983	0,8988778	0,0006758	0,009517136	acetyl-CoA acetyltransferase, putative
PFIT_1000700.1	211,9281382	276,6492647	398,1702247	430,035491	244,2887015	414,10286	1,695137169	0,761402	0,000682	0,009578517	acyl-CoA binding protein, isoform 1, ACBP1
PFIT_0101300	67,69926637	47,90463458	113,1641691	126,4023449	57,80195048	119,78326	2,072304759	1,0512362	0,000685	0,009596404	conserved Plasmodium protein, unknown function
PFIT_0934000	1287,757784	1203,603944	777,0606281	819,274458	1245,680864	798,16754	0,640748017	-0,642171	0,0006938	0,009696608	gamma-tubulin complex component, putative
PFIT_1403600	2475,438392	2718,588013	1912,055332	1561,637436	2597,013202	1736,8464	0,66878612	-0,580383	0,0007069	0,009854515	pre-mRNA splicing factor, putative
PFIT_1006300	1872,031887	2075,468293	1348,749246	1262,685859	1973,75009	1305,7176	0,661541478	-0,596096	0,0007125	0,009868235	conserved Plasmodium protein, unknown function
PFIT_1134700	2862,501589	3179,67012	2097,309268	1936,162903	3021,085855	2016,7361	0,667553384	-0,583045	0,0007114	0,009868235	heat shock protein 70
PFIT_0936600	2026,562822	2467,088681	3261,642831	3586,081342	2246,825751	3423,8621	1,523866319	0,6077363	0,0007131	0,009868235	ring-exported protein 2
PFIT_1469200	23036,88297	24249,32603	14863,0658	16772,05295	23643,1045	15817,559	0,669013639	-0,579892	0,0007249	0,009978315	conserved Plasmodium protein, unknown function
PFIT_0916800	14165,33563	16147,4547	24322,75209	21458,97164	15156,39516	22890,862	1,510310441	0,5948451	0,0007264	0,009978315	RNA-binding protein musashi, putative
PFIT_1349500	573,972041	579,6460785	979,0796263	844,6886861	576,8090597	911,88416	1,580911639	0,6607567	0,0007235	0,009978315	conserved Plasmodium protein, unknown function
PFIT_0310100	12787,80273	14603,72785	9255,990783	9253,454205	13695,76529	9254,7225	0,675736061	-0,565468	0,0007403	0,01014554	phd finger protein, putative
PFIT_0406800	339,968055	348,5062166	577,5563892	542,393131	344,2371358	559,97476	1,626712234	0,7019591	0,0007454	0,010189624	conserved Plasmodium protein, unknown function
PFIT_0401100	33726,00844	34226,66379	53351,4571	48220,15381	33976,33612	50785,805	1,494740495	0,579895	0,0007483	0,010205107	Plasmodium exported protein, unknown function
PFIT_0924400	191,3240137	207,1875446	377,2138971	310,3211008	199,2557791	343,7675	1,725257358	0,7868116	0,000751	0,010216726	thiamine pyrophosphokinase
PFIT_bin06900	72,11443592	80,24026293	28,50060556	34,10856927	76,17734942	31,304587	0,410943511	-1,282988	0,0007531	0,010219954	A_ITvar60 - erythrocyte membrane protein 1, PfEMP1, putative (VAR)
PFIT_0825200	136,8702559	176,0495321	251,4759314	301,6267596	156,459894	276,55135	1,767554218	0,8217545	0,0007548	0,010219954	small nuclear ribonucleoprotein G, putative
PFIT_0820100	744,6919301	756,8932264	1133,318198	1191,793538	750,7925782	1162,5559	1,548438146	0,6308138	0,0007621	0,010294432	conserved Plasmodium protein, unknown function
PFIT_0537600	169,2481659	207,1875446	88,8548291	114,3640264	188,2178552	101,60943	0,539850099	-0,889369	0,0007785	0,010486828	B_ITvar25 - erythrocyte membrane protein 1, PfEMP1, putative
PFIT_0205600	2621,138987	2806,013971	1872,657436	1744,887397	2713,576479	1808,7724	0,666564009	-0,585185	0,000782	0,010486828	conserved Plasmodium protein, unknown function, pseudogene
PFIT_1014100	21556,32945	21287,62199	14934,31731	13906,26433	21421,97572	14420,291	0,673154102	-0,570991	0,0007801	0,010486828	transcriptional coactivator ADA2
PFIT_1424000	2131,055167	2207,206038	1637,946567	1193,799925	2169,130603	1415,8732	0,652737665	-0,615425	0,0007888	0,010553111	GTPase, putative
PFIT_0500900	562,1982555	426,3512478	265,7262342	240,7663713	494,2747516	253,2463	0,512359375	-0,964772	0,0008015	0,010672553	erythrocyte membrane protein 2
PFIT_0321200	2625,554157	2089,839684	1420,000759	1390,425794	2357,69692	1405,2133	0,596010991	-0,746589	0,0007996	0,010672553	conserved Plasmodium protein, unknown function
PFIT_1339800	1542,365895	2029,95889	1023,507041	827,9687992	1786,162393	925,73792	0,518283177	-0,948188	0,0008076	0,010728076	RNA lariat debranching enzyme, putative
PFIT_0215900	890,3925251	797,6121658	1341,204968	1246,634767	844,0023454	1293,9199	1,533076151	0,6164294	0,0008212	0,010858386	conserved Plasmodium protein, unknown function
PFIT_1219400	76,52960546	52,69509804	150,8855589	117,7080038	64,61235175	134,29678	2,078500127	1,0555428	0,0008212	0,010858386	raf kinase inhibitor
PFIT_1444000	97,13373001	108,9830437	207,0485169	181,243574	103,0583868	194,14605	1,883845181	0,9136804	0,0008246	0,010878201	conserved Plasmodium protein, unknown function
PFIT_0304800	320,8356537	400,0036988	559,1148209	610,6102695	360,4196762	584,86255	1,62272646	0,6984198	0,0008395	0,011048507	conserved Plasmodium protein, unknown function
PFIT_1328900	1208,284732	1205,999176	1818,170984	1849,888286	1207,141954	1834,0296	1,519315627	0,6034216	0,0008503	0,01116422	conserved Plasmodium protein, unknown function
PFIT_1001800	2141,35723	2111,396769	3158,537699	3221,587807	2126,376999	3190,0628	1,50023385	0,5851874	0,0008612	0,011281938	tryptophan-rich antigen 3
PFIT_1148600	547,4810237	475,4534982	309,3153956	316,3402601	511,467261	312,82783	0,611628254	-0,709273	0,0008648	0,011302807	serine/threonine protein kinase, putative
PFIT_1317100	1140,585466	1323,36553	2036,955045	1758,932102	1231,975498	1897,9436	1,540569253	0,6234635	0,0008739	0,011368105	small ribosomal subunit nuclear export protein, putative
PFIT_1354300	173,6633355	174,8519162	284,1678025	313,6650782	174,2576258	298,91644	1,715370784	0,7785205	0,0008732	0,011368105	RAP protein, putative
PFIT_1028600	316,4204841	348,5062166	534,8054808	543,7307219	332,4633504	539,2681	1,62203774	0,6978074	0,0008859	0,011498192	conserved Plasmodium protein, unknown function
PFIT_0318600	1409,910808	1574,864862	2320,284594	2225,082548	1492,387835	2272,6836	1,522850507	0,6067743	0,0008967	0,011612467	cleavage and polyadenylation specifity factor, putative
PFIT_0934300	420,91283	385,6323084	633,7193472	642,0436569	403,2725692	637,8815	1,581762685	0,6615332	0,0009057	0,011701008	delta tubulin, putative
PFIT_0922500	161,88955	130,5401292	260,6967156	248,1231216	146,2148396	254,40992	1,739973311	0,7990652	0,0009195	0,011852898	para-aminobenzoic acid synthetase
PFIT_0908300	54,45375773	56,28794564	98,07561325	132,4215042	55,37085168	115,24856	2,081394005	1,0575501	0,0009391	0,012078083	conserved Plasmodium membrane protein, unknown function
PFIT_0107400	855,0711687	663,479189	1103,979339	1187,780765	759,2751788	1145,8801	1,50917623	0,5937613	0,000957	0,012280564	unspecified product
PFIT_0518800	351,7418405	390,4227719	213,7545417	234,747212	371,0823062	224,25088	0,604315736	-0,726626	0,0009642	0,012301383	mRNA-binding protein PUF1
PFIT_1360200	3067,071111	3298,234091	2186,164097	2094,667431	3182,652601	2140,4158	0,67252573	-0,572339	0,0009652	0,012301383	conserved Plasmodium protein, unknown function
PFIT_0414000	727,0312519	712,5814394	1037,757344	1166,37931	719,8063457	1102,0683	1,531062255	0,6145329	0,0009613	0,012301383	nonclathrin coat protein zeta2-cop-related protein, putative
PFIT_1118800	269,3253423	286,2301916	170,1653803	155,8293459	277,777767	162,99736	0,58679053	-0,769083	0,0009812	0,012476551	conserved Plasmodium protein, unknown function
PFIT_1205400	13628,15666	13747,43251	9846,959221	8777,271826	13687,79459	9312,1155	0,680322565	-0,555709	0,0010064	0,012769169	high mobility group protein B3, putative (HMGB3)
PFIT_0505000	1598,291376	1940,137701	1072,963974	1221,220539	1769,214538	1147,0923	0,648362441	-0,625128	0,0010135	0,01280672	conserved Plasmodium membrane protein, unknown function
PFIT_0302500	289,9294668	275,4536489	454,3331828	462,1376739	282,6905578	458,23543	1,620978896	0,6968653	0,0010158	0,01280672	ABC transporter, (TAP family), putative
PFIT_1457100	245,7777714	216,7684715	337,8160012	417,328377	231,2731214	377,57219	1,632581368	0,7071549	0,0010162	0,01280672	dfg10 like protein, putative
PFIT_1009800	2837,482295	2863,499532	4335,445058	4167,26461	2850,490914	4251,3548	1,491446548	0,5767123	0,0010185	0,012806863	succinate dehydrogenase subunit 4, putative
PFIT_1409100	2388,606724	3089,848931	4031,159181	4236,81934	2739,227827	4133,9893	1,509180514	0,5937654	0,001033	0,012960373	histo-aspartic protease
PFIT_0526500	657,8602623	708,9885918	512,172647	374,5254665	683,4244271	443,34906	0,648717019	-0,624339	0,0010369	0,012980661	S-adenosylmethionine-dependent methyltransferase, putative
PFIT_0303100	9792,846053	8008,457286	5945,729272	5184,502529	8900,65167	5565,1159	0,62524814	-0,677499	0,0010472	0,013080289	HAD superfamily protein, putative
PFIT_1323400	691,7098955	965,2783869	327,756964	405,9588539	828,4941412	366,85791	0,442800849	-1,17527	0,0010598	0,013180323	micro-fibrillar-associated protein, putative
PFIT_1411500	26,49101728	16,7666221	51,97169249	64,20436569	21,62881969	58,088029	2,685677255	1,4252859	0,0010577	0,013180323	conserved Plasmodium protein, unknown function
PFIT_0715900	727,0312519	656,2934938	429,1855896	444,7489915	691,6623728	436,96729	0,63176386	-0,662543	0,0010642	0,013205085	conserved Plasmodium protein, unknown function
PFIT_1234100	30,90618682	40,7189394	82,98705737	86,27461639	35,81256311	84,630837	2,363160565	1,2407177	0,0010765	0,013328775	aspartyl protease, putative
PFIT_0207000	5739,72041	4768,906373	3250,74554	3611,49557	5254,313391	3431,1206	0,65301026	-0,614822	0,0010842	0,013394565	serine repeat antigen 4
PFIT_1019200	516,5748369	475,4534982	234,7108693	345,0984656	496,0141676	289,90467	0,584468522	-0,774803	0,0010932	0,013462247	conserved Plasmodium protein, unknown function
PFIT_1359200	498,9141587	463,4773396	352,066304	252,1358944	481,1957491	302,1011	0,627813317	-0,671592	0,0010944	0,013462247	RNA binding protein, putative
PFIT_0718600	844,7691064	995,2187835	648,8079031	565,8009726	919,993945	607,30444	0,660178864	-0,599204	0,0011246	0,013773749	ferredoxin reductase-like protein
PFIT_0725300	4092,862169	4118,600958	2694,983732	2828,336068	4105,731564	2761,6599	0,67263528	-0,572104	0,0011284	0,013773749	conserved Plasmodium membrane protein, unknown function
PFIT_0703500	27481,48698	30536,80932	18233,68153	21261,67697	29009,14815	19747,679	0,680739715	-0,554825	0,0011271	0,013773749	ubiquitin transferase, putative
PFIT_bin06500	133,9268096	126,9472816	202,8572513	253,4734854	130,4370456	228,16537	1,749237475	0,8067262	0,0011295	0,013773749	unspecified product
PFIT_1474800	289,9294668	408,3870098	110,6494098	165,861278	349,1582383	138,25534	0,395967584	-1,336546	0,0011332	0,013789183	conserved Plasmodium protein, unknown function
PFIT_0414800	1496,742476	1480,253209	1068,772709	911,5682337	1488,497842	990,17047	0,665214583	-0,588108	0,0011396	0,01383652	conserved protein, unknown function
PFIT_1325400	33,84963318	40,7189394	72,92802011	98,31293496	37,28428629	85,620478	2,296422597	1,1993882	0,0011484	0,013913358	conserved Plasmodium protein, unknown function
PFIT_0315600	85,35994455	99,40211676	45,26566766	40,79652403	92,38103066	43,031096	0,465800127	-1,102217	0,001152	0,013928137	conserved Plasmodium protein, unknown function
PFIT_0714500	983,1110855	923,3618316	706,6473673	543,0619264	953,2364586	624,85465	0,655508548	-0,609314	0,0011616	0,014013309	conserved Plasmodium protein, unknown function
PFIT_0216600	654,916816	568,8675357	1011,771497	867,4277323	611,8921758	939,59961	1,535564029	0,6187687	0,0011704	0,014089571	conserved Plasmodium protein, unknown function
PFIT_0732100	145,700595	116,1687389	201,1807451	251,4670989	130,9346669	226,32392	1,728525587	0,789542	0,001182	0,014199143	Plasmodium exported protein, unknown function
PFIT_0210100	628,4257987	706,5933601	1021,830535	1037,301783	667,5095794	1029,5662	1,542399077	0,6251761	0,0011914	0,014281843	conserved Plasmodium protein, unknown function
PFIT_0506200	141,2854255	158,0852941	246,4464128	272,8685542	149,6853598	259,65748	1,734688575	0,7946767	0,0011962	0,01430909	transcription initiation factor TFIID, TATA-binding protein
PFIT_1025400	4619,739068	3720,992491	2658,100595	2580,212946	4170,36578	2619,1568	0,628040059	-0,671072	0,001213	0,014453203	conserved Plasmodium protein, unknown function
PFIT_0520800	1230,36058	1497,019831	1994,204136	2128,107204	1363,690205	2061,1557	1,51145448	0,5959375	0,001216	0,014453203	bis(5'-nucleosyl)-tetraphosphatase [asymmetrical]
PFIT_1347000	73,5861591	89,82118984	146,6942933	166,5300735	81,70367447	156,61218	1,91683158	0,9387236	0,0012142	0,014453203	leucine-rich repeat protein
PFIT_1104600	2422,456357	2336,548552	1547,415231	1627,179393	2379,502455	1587,2973	0,667071097	-0,584088	0,0012213	0,014486239	CCR4-NOT transcription complex subunit 1, putative
PFIT_0830800	15651,77604	17602,55798	12779,16858	10233,23958	16627,16701	11506,204	0,6920123	-0,53113	0,001253	0,014818786	protein disulfide isomerase
PFIT_1351400	1599,763099	1800,016644	2647,203305	2465,180124	1699,889872	2556,1917	1,503739599	0,5885548	0,0012546	0,014818786	conserved Plasmodium protein, unknown function
PFIT_1413400	1604,178268	1724,566845	1032,727825	1181,761606	1664,372557	1107,2447	0,665262541	-0,588004	0,0012782	0,015065737	conserved Plasmodium protein, unknown function
PFIT_0707900	2682,951361	3604,823752	4824,146618	5643,296226	3143,887557	5233,7214	1,664729202	0,7352875	0,0012842	0,015105342	chloroquine resistance transporter
PFIT_1231100	759,4091619	686,2338904	490,3780663	438,7298322	722,8215261	464,55395	0,642695233	-0,637793	0,001289	0,015129907	conserved Plasmodium protein, unknown function
PFIT_1408800	9034,908614	10962,97562	15179,92547	14508,84905	9998,942119	14844,387	1,484595779	0,5700702	0,0012921	0,015134795	plasmepsin IV
PFIT_0103500	173,6633355	215,5708556	311,830155	343,7608746	194,6170955	327,79551	1,684309972	0,7521577	0,001295	0,015138127	unspecified product
PFIT_bin00900	133,9268096	162,8757576	65,38374217	90,95618472	148,4012836	78,169963	0,526747219	-0,924817	0,0013079	0,015225563	erythrocyte membrane protein 1, PfEMP1
PFIT_1034100	279,6274046	256,289795	393,1407061	464,1440603	267,9585998	428,64238	1,59965899	0,6777644	0,0013063	0,015225563	ADP-ribosylation factor, putative
PFIT_1460400	482,7252037	592,819853	294,2268398	360,4807615	537,7725283	327,3538	0,608721687	-0,716145	0,0013175	0,015274747	conserved Plasmodium protein, unknown function
PFIT_1473200	725,5595287	777,2526961	1039,43385	1238,609201	751,4061124	1139,0215	1,515853434	0,6001303	0,0013163	0,015274747	Yip1 protein, putative
PFIT_1313800	191,3240137	222,7565508	134,1204968	105,0008897	207,0402822	119,56069	0,577475513	-0,792168	0,0013252	0,015332265	lipoate protein ligase a type 1
PFIT_1332600	702,0119578	788,0312389	1156,789285	1124,91399	745,0215983	1140,8516	1,531300086	0,614757	0,0013405	0,015477906	transmembrane protein Tmp21 homologue, putative
PFIT_1333100	1847,012593	1908,999688	1445,986606	1058,703238	1878,006141	1252,3449	0,666848151	-0,58457	0,0013584	0,015652119	conserved Plasmodium protein, unknown function
PFIT_0316900	1529,120386	1744,926315	2556,671969	2360,84803	1637,02335	2458,76	1,501970023	0,586856	0,0013683	0,015713714	RING zinc finger protein, putative
PFIT_1429900	388,53492	317,3682041	228,0048445	198,6322563	352,9515621	213,31855	0,604384775	-0,726461	0,0013755	0,015773212	conserved protein, unknown function
PFIT_1011800	891,8642483	750,9051471	575,879883	457,4561055	821,3846977	516,66799	0,62902072	-0,668821	0,0013801	0,015773212	ubiquinol-cytochrome c reductase complex subunit, putative
PFIT_0804800	2964,050488	3450,331306	2005,93968	2348,140916	3207,190897	2177,0403	0,678799725	-0,558942	0,0013778	0,015773212	glutamate dehydrogenase, putative (GDH3)
PFIT_1239700	847,7125528	934,1403744	652,9991686	529,6860169	890,9264636	591,34259	0,663738947	-0,591312	0,0013914	0,015851236	3-hydroxyisobutyryl-coenzyme A hydrolase, putative
PFIT_1011300	21357,64682	23037,33877	14680,32662	15788,9236	22197,49279	15234,625	0,686321886	-0,543043	0,0013925	0,015851236	QF122 antigen
PFIT_0830700	4690,381781	5001,243851	2951,489182	3614,839547	4845,812816	3283,1644	0,677526039	-0,561652	0,0014275	0,016216963	SET domain protein, putative

PFIT_1312000	6903,853447	6329,399844	4315,326983	4638,765421	6616,626645	4477,0462	0,67663576	-0,563549	0,0014655	0,016615325	conserved Plasmodium protein, unknown function
PFIT_1212300	8197,498123	10189,31578	5973,391624	6067,981353	9193,40695	6020,6865	0,654891763	-0,610672	0,0014803	0,016715874	eukaryotic translation initiation factor 3 subunit 10, putative
PFIT_0800200	178,078505	215,5708556	342,0072667	319,6842375	196,8246803	330,84575	1,680915989	0,7492476	0,0014781	0,016715874	rifin, pseudogene
PFIT_1477600	120,6813009	132,935361	62,86898286	67,54834307	126,8083309	65,208663	0,514230118	-0,959514	0,0014929	0,016757149	probable protein, unknown function
PFIT_1334200	253,1363873	204,7923128	133,2822437	127,7399359	228,9643501	130,51109	0,570006159	-0,810951	0,0014918	0,016757149	MSP7-like protein
PFIT_1234500	5729,418347	6432,394809	4682,481843	3767,324916	6080,906578	4224,9034	0,694781827	-0,525368	0,0014891	0,016757149	Splicing factor 3B subunit 3, putative
PFIT_1147400	229,5888164	223,9541667	132,4439905	129,0775269	226,7714915	130,76076	0,576619035	-0,79431	0,0015033	0,016838903	conserved Plasmodium protein, unknown function
PFIT_1103300	208,9846918	246,7088681	129,9292312	135,7654816	227,84678	132,84736	0,583055668	-0,778294	0,0015061	0,016838903	Plasmodium exported protein (PHISTb), unknown function
PFIT_0601400	378,2328578	400,0036988	248,9611721	235,4160075	389,1182783	242,18859	0,62240353	-0,684078	0,0015271	0,017039608	geranylgeranyltransferase, putative
PFIT_1230500	1217,115071	1239,53242	751,9130349	871,4405051	1228,323746	811,67677	0,660800357	-0,597714	0,0015364	0,017109712	conserved Plasmodium protein, unknown function
PFIT_1417500	8187,196061	8558,162968	6203,911228	5383,134786	8372,679515	5793,523	0,691955663	-0,531248	0,0015792	0,017551849	NADP-specific glutamate dehydrogenase (GDH1)
PFIT_1315000	298,7598059	322,1586676	493,7310787	494,9086522	310,4592368	494,31987	1,59222148	0,671041	0,0015871	0,017570109	Myb1-related transcription factor
PFIT_0511800	182,4936746	239,5231729	355,4193164	348,442443	211,0084237	351,93088	1,667852275	0,7379915	0,0015859	0,017570109	kinase binding protein CGI-121, putative
PFIT_0204600	282,5708509	308,9848931	476,9660166	469,4944241	295,777872	473,23022	1,5999514	0,6780281	0,0016057	0,017741786	conserved Plasmodium protein, unknown function
PFIT_0516500	825,6367051	681,4434269	502,9518628	369,1751027	753,540066	436,06348	0,578686526	-0,789146	0,0016531	0,018193943	phosducin-like protein, putative (PhLP3)
PFIT_0516900	1228,888857	1129,35176	676,4702555	866,0901413	1179,120309	771,2802	0,65411493	-0,612384	0,0016507	0,018193943	transcription factor with AP2 domain(s)
PFIT_0802300	528,3486223	778,450312	277,4617777	265,5118039	653,3994671	271,48679	0,415498947	-1,267083	0,0016713	0,018286816	Plasmodium exported protein (hyp17), unknown function
PFIT_0730500	1504,101092	1213,184871	647,96965	858,0645956	1358,642981	753,01712	0,554242088	-0,851412	0,0016681	0,018286816	acyl-CoA synthetase (ACS5)
PFIT_0930500	269506,3642	234242,8846	184802,1177	143908,0665	251874,6244	164355,09	0,652527393	-0,61589	0,0016671	0,018286816	merozoite surface protein 1
PFIT_0322300	207,5129687	185,630459	325,2422046	319,6842375	196,5717138	322,46322	1,640435517	0,7140789	0,001689	0,018445306	conserved Plasmodium protein, unknown function
PFIT_1124900	1064,055861	1131,746992	1616,990239	1656,606394	1097,901426	1636,7983	1,490842691	0,576128	0,0017173	0,018681716	endoplasmic reticulum oxidoreductin, putative
PFIT_1143600	289,9294668	211,978008	399,846731	393,9205353	250,9537374	396,88363	1,581501185	0,6612946	0,0017155	0,018681716	conserved Plasmodium protein, unknown function
PFIT_1016200	3864,745076	4143,750891	6804,100451	5533,613768	4004,247984	6168,8571	1,54057819	0,6234719	0,0017233	0,01871065	Plasmodium exported protein (PHISTc), unknown function
PFIT_1411000	693,1816187	758,0908423	1088,890783	1107,525308	725,6362305	1098,208	1,513441583	0,597833	0,0017287	0,018734181	tetratricopeptide repeat family protein, putative
PFIT_1105700	10591,99174	8378,520589	5901,301857	4313,062024	9485,256165	5107,1819	0,538433739	-0,893159	0,0017387	0,018769887	calcium/calmodulin-dependent protein kinase, putative
PFIT_0311000	641,6713073	768,8693851	347,0367854	476,8511743	705,2703462	411,94398	0,58409372	-0,775728	0,0017387	0,018769887	pre-mRNA splicing factor, putative
PFIT_1228000	535,7072382	523,3581328	264,049728	385,8949896	529,5326855	324,97236	0,613696506	-0,704403	0,0017869	0,019253554	NIMA related kinase 1
PFIT_0308600	2588,761077	2220,379813	1646,329098	1113,544467	2404,570445	1379,9368	0,573880788	-0,801177	0,0018204	0,019528361	conserved Plasmodium protein, unknown function
PFIT_1248900	5505,716424	6974,914795	3757,050415	4038,855879	6240,31561	3897,9531	0,624640385	-0,678902	0,0018228	0,019528361	conserved Plasmodium protein, unknown function
PFIT_1245900	1714,557507	1314,982219	2279,210192	2826,329681	1514,769863	2552,7699	1,685252657	0,7529649	0,001818	0,019528361	unspecified product
PFIT_0704100	3485,040495	4038,360695	6280,192261	8519,116772	3761,700595	7399,6545	1,967103529	0,9760729	0,0018411	0,019686948	conserved Plasmodium protein, unknown function
PFIT_1476000	325,2508232	318,56582	182,7391768	207,995393	321,9083216	195,36728	0,606903493	-0,720461	0,0018541	0,019715384	trailer hitch homolog, putative
PFIT_1232800	345,8549478	330,5419786	188,6069486	222,7088935	338,1984632	205,65792	0,608098331	-0,717623	0,0018478	0,019715384	elongation factor G, putative
PFIT_1368700	279,6274046	263,4754902	437,5681207	425,3539227	271,5514474	431,46102	1,588873953	0,6680047	0,0018542	0,019715384	U1 small nuclear ribonucleoprotein a, putative
PFIT_0910500	367,9307955	414,3750891	552,408796	660,1011347	391,1529423	606,25497	1,549917947	0,6321918	0,0018606	0,019746145	conserved Plasmodium protein, unknown function
PFIT_0319500	938,9593901	1198,81348	634,5576003	680,164999	1068,886435	657,3613	0,614996391	-0,70135	0,0018722	0,019832209	RNA binding protein, putative
PFIT_0501200.2	3090,618682	3307,815018	4826,661377	4544,465259	3199,21685	4685,5633	1,464596974	0,5505037	0,0018845	0,019925036	heat shock protein 40, type II
PFIT_0315900	75,05788228	97,00688503	176,8714051	147,1350047	86,03238366	162,0032	1,883049127	0,9130706	0,0018882	0,019926739	conserved Plasmodium protein, unknown function
PFIT_1430400	126,5681936	116,1687389	60,35422354	63,53557021	121,3684663	61,944897	0,510387078	-0,970336	0,0018961	0,019967782	transcription factor with AP2 domain(s), putative
PFIT_0628400	2622,61071	2894,637545	4552,552612	3756,624188	2758,624127	4154,5884	1,506036418	0,5907567	0,0018992	0,019967782	amino acid transporter, putative
PFIT_1230800	2884,577437	2983,261119	2240,650549	1817,786104	2933,919278	2029,2183	0,691640817	-0,531905	0,0019226	0,020177012	Rab GTPase 2
PFIT_1436900.2	303,1749755	225,1517825	144,179534	125,7335495	264,163379	134,95654	0,510882857	-0,968936	0,0019563	0,020441888	ataxin-2 like protein, putative
PFIT_1031000	406,1955982	449,1059492	262,3732218	279,5565089	427,6507737	270,96487	0,633612475	-0,658327	0,0019587	0,020441888	conserved Plasmodium protein, unknown function
PFIT_1104500	908,0532033	1027,554412	1448,501365	1447,27341	967,8038075	1447,8874	1,496054651	0,5811629	0,0019527	0,020441888	casein kinase II beta chain
PFIT_0609300	238035,0357	205270,1616	144975,8745	83759,9454	221652,5986	114367,91	0,515978205	-0,954618	0,0019681	0,02044993	histone H3
PFIT_1325500	1377,532898	1444,324733	755,2660474	1056,028056	1410,928815	905,64705	0,641880045	-0,639624	0,0019662	0,02044993	RNA binding protein, putative
PFIT_1036900	132,4550864	182,0376114	267,4027404	270,1933723	157,2463489	268,79806	1,709407298	0,7734962	0,0019704	0,02044993	enhancer of rudimentary homolog, putative
PFIT_1478100	490,0838196	726,9528298	273,2705121	224,0464844	608,5183247	248,6585	0,408629433	-1,291135	0,0019861	0,020575004	Plasmodium exported protein (hyp17), unknown function
PFIT_0111100	1715353,709	567280,6947	10147,05383	24798,93625	1141317,202	17472,995	0,015309499	-6,029429	0,0020081	0,020706005	28S ribosomal RNA
PFIT_1342700	432,6866155	409,5846257	295,0650929	239,4287804	421,1356206	267,24694	0,634586398	-0,656111	0,0020049	0,020706005	conserved Plasmodium protein, unknown function
PFIT_0522600	4868,460286	4269,500557	3125,845828	3110,567758	4568,980422	3118,2068	0,682473223	-0,551156	0,0020115	0,020706005	conserved Plasmodium protein, unknown function, putative
PFIT_0807300	7851,643176	7276,713993	5034,548147	5340,331875	7564,178584	5187,44	0,685790262	-0,544161	0,0020134	0,020706005	conserved Plasmodium membrane protein, unknown function
PFIT_0914200	5171,635261	4973,698686	3309,423258	3636,909798	5072,666973	3473,1665	0,684682544	-0,546493	0,0020261	0,020798351	conserved Plasmodium protein, unknown function
PFIT_0834300	50,03858819	61,07840909	108,9729036	115,7016173	55,55849864	112,33726	2,021963574	1,015757	0,0020668	0,021178008	Plasmodium exported protein, unknown function
PF_apicoplast_g	47,09514182	56,28794564	94,72260084	116,3704128	51,69154373	105,54651	2,041852481	1,0298786	0,0020812	0,021286599	apicoplast ribosomal protein S2
PFIT_0702200	123,6247473	117,3663547	61,19247665	62,86677474	120,495551	62,029626	0,514787684	-0,957951	0,0020863	0,021301088	conserved Plasmodium protein, unknown function
PFIT_0814100	1365,759113	1384,443939	1909,540573	2114,731295	1375,101526	2012,1359	1,463263545	0,5491896	0,0020997	0,021360333	conserved protein, unknown function
PFIT_0418400	621,0671828	712,5814394	991,6534229	1023,257078	666,8243111	1007,4553	1,510825616	0,5953371	0,0020983	0,021360333	60S ribosomal protein L7ae/L30e, putative
PFIT_1006100	195,7391832	166,4686052	112,325916	92,29377568	181,1038942	102,30985	0,5649235	-0,823873	0,0021217	0,021545321	conserved Plasmodium protein, unknown function
PFIT_0320400	342,9115014	428,7464795	233,8726162	256,1486673	385,8289905	245,01064	0,63502393	-0,655117	0,0021603	0,021897867	oocyst capsule protein
PFIT_0407900	978,695916	1154,501693	1516,399866	1651,25603	1066,598805	1583,8279	1,484933174	0,570398	0,002172	0,021964533	conserved Plasmodium protein, unknown function
PFIT_1111800	236,9474323	289,8230392	430,8620958	415,3219905	263,3852358	423,09204	1,606362035	0,6837971	0,0021747	0,021964533	tRNA m5C-methyltransferase, putative
PFIT_1335800	207,5129687	200,0018494	124,0614595	112,35764	203,757409	118,20955	0,580148473	-0,785506	0,0021879	0,022058459	conserved Plasmodium protein, unknown function
PFIT_1429400	2513,703195	2458,70537	3496,3537	3693,757413	2486,204282	3595,0556	1,446001675	0,5320692	0,0022004	0,022132717	metabolite/drug transporter, putative
PFIT_1352100	1055,225521	1183,244474	1664,770666	1654,600007	1119,234998	1659,6853	1,482874767	0,5683968	0,0022031	0,022132717	conserved Plasmodium protein, unknown function
PFIT_bin10500	1074,357923	1597,619563	481,9955352	589,8776098	1335,988743	535,93657	0,401153509	-1,317774	0,0022253	0,022316612	erythrocyte membrane protein 1, PfEMP1 (VAR)
PFIT_1416600	553,3679164	585,6341578	367,1548599	374,5254665	569,5010371	370,84016	0,651166792	-0,618901	0,0022383	0,022322285	conserved Plasmodium protein, unknown function
PFIT_1252400	631,3692451	685,0362745	472,7747511	401,2772856	658,2027598	437,02602	0,663968681	-0,590813	0,0022454	0,022322285	chitinase
PFIT_0302900	1292,172954	1396,420098	850,8269013	966,4094627	1344,296526	908,61818	0,67590607	-0,565105	0,0022442	0,022322285	N-ethylmaleimide sensitive fusion protein (NSF)
PFIT_1469000	6449,090983	6041,972037	4093,18991	4490,292825	6245,53151	4291,7414	0,687169917	-0,541261	0,0022457	0,022322285	rab GTPase activator, putative
PFIT_0628200	2793,330599	2822,780593	1976,600821	1892,691197	2808,055596	1934,646	0,688962858	-0,537502	0,0022401	0,022322285	phospholipase, putative
PFIT_0311600	32,37791	31,13801248	12,57379657	6,019159283	31,75796124	9,2964779	0,292729053	-1,772362	0,0022763	0,02242941	plasmepsin VI
PFIT_1120700	3364,359194	3582,069051	4666,555034	5354,37658	3473,214122	5010,4658	1,442602048	0,5286734	0,0022648	0,02242941	conserved Plasmodium protein, unknown function
PFIT_1001300	3080,31662	3330,569719	4560,935143	4736,40956	3205,44317	4648,6724	1,45024326	0,5362949	0,0022749	0,02242941	Plasmodium exported protein (PHISTc), unknown function
PFIT_0626800	1028,734504	1050,309113	1744,404711	1411,82725	1039,521809	1578,116	1,518117241	0,6022832	0,002273	0,02242941	conserved Plasmodium protein, unknown function
PFIT_1349600	186,9088441	159,28291	267,4027404	296,9451913	173,095877	282,17397	1,630159948	0,7050135	0,0022609	0,02242941	ubiquitin-activating enzyme e1, putative
PFIT_1339900	363,5156259	293,4158868	176,033152	216,0209387	328,4657564	196,02705	0,596795987	-0,74469	0,0023111	0,022712651	Rab GTPase 11b
PFIT_1238900	3120,053146	2653,916756	1845,833337	2065,24043	2886,984951	1955,5369	0,677363033	-0,561999	0,0023171	0,022712651	transcription factor with AP2 domain(s)
PFIT_1117400	357,6287332	325,7515152	466,9069793	581,852064	341,6901242	524,37952	1,534663968	0,6179228	0,0023142	0,022712651	oligosaccharyl transferase STT3 subunit, putative
PFIT_1250600	139,8137023	165,2708893	72,92802011	94,96895758	152,5423458	83,948489	0,55032908	-0,861634	0,0023415	0,022912268	conserved Plasmodium protein, unknown function
PFIT_0101000	22555,62949	22366,67389	16362,7006	15073,31244	22461,15169	15718,007	0,699786313	-0,515014	0,0023546	0,023000182	erythrocyte binding antigen-181
PFIT_1354800	5126,011843	5126,993516	7462,967391	7294,552256	5126,50268	7378,7598	1,439335993	0,5254034	0,0023647	0,023059296	protein phosphatase, putative
PFIT_0810900	145,700595	125,7496658	213,7545417	241,4351668	135,7251304	227,59485	1,676880719	0,7457801	0,0023794	0,023161851	conserved Plasmodium protein, unknown function
PFIT_0710100	276,6839582	323,3562834	470,2599918	475,5135834	300,0201208	472,88679	1,576183578	0,6564356	0,0023879	0,023204925	conserved Plasmodium protein, unknown function
PFIT_1017900	60,34065046	38,32370767	93,04609463	103,6632988	49,33217906	98,354697	1,993722932	0,9954649	0,0023958	0,02324125	conserved Plasmodium protein, unknown function
PFIT_0213700	345,8549478	414,3750891	543,1880119	630,0053383	380,1150185	586,59668	1,543208362	0,6259329	0,0024288	0,02352104	conserved Plasmodium protein, unknown function
PFIT_1441700	157,4743805	192,8161542	105,6198912	96,97534401	175,1452673	101,29762	0,57836343	-0,789952	0,00246	0,023782848	allantoicase, putative
PFIT_1121300	1290,701231	1419,1748	2072,161675	1917,436629	1354,938015	1994,7992	1,472243844	0,5580166	0,002469	0,02382888	tRNA nucleotidyltransferase, putative
PFIT_0501800	64,75582001	77,8450312	123,2232064	145,7974137	71,3004256	134,51031	1,886528852	0,9157342	0,0025491	0,024554863	anaphase promoting complex subunit, putative
PFIT_1438800	55,92548091	80,24026293	138,3117623	125,7335495	68,08287192	132,02266	1,939146398	0,9554217	0,0025529	0,024554863	conserved Plasmodium protein, unknown function
PFIT_0512800	113,322685	150,8995989	230,5196038	225,3840754	132,111142	227,95184	1,725455069	0,7869769	0,0025714	0,024690622	orotate phosphoribosyltransferase (OPRT)
PFIT_0501200.1	4373,961297	4681,480415	6857,74865	6236,517813	4527,720856	6547,1332	1,446010794	0,5320783	0,0025798	0,024693724	heat shock protein 40, type II
PFIT_1464250	746,1636532	622,7602496	1036,919091	980,4541677	684,4619514	1008,6866	1,47369277	0,5594358	0,0025805	0,024693724	conserved Plasmodium protein, unknown function, pseudogene
PFIT_1009400	466,5362487	590,4246212	826,5175613	787,8410706	528,480435	807,17932	1,527358938	0,6110391	0,0025885	0,024728928	conserved Plasmodium protein, unknown function
PFIT_1018200	3258,395125	3488,655014	5221,478589	4557,172373	3373,525069	4889,3255	1,449322409	0,5353786	0,0026953	0,025705768	conserved Plasmodium protein, unknown function
PFIT_1403200	1309,833632	1186,837322	1642,137832	1947,532426	1248,335477	1794,8351	1,437782681	0,5238456	0,0027581	0,026260288	26S proteasome regulatory subunit
PFIT_0626900	25,01929409	32,33562834	74,60452632	62,19797926	28,67746122	68,401253	2,38519206	1,2541054	0,0027913	0,026531802	6-pyruvoyltetrahydropterin synthase
PFIT_1331700	1320,135694	1366,479701	2193,708375	1788,359103	1343,307698	1991,0337	1,482187396	0,5677279	0,0028141	0,026703163	nucleotidyltransferase, putative
PFIT_0813000	4,415169546	11,97615865	25,98584625	37,45254665	8,195664096	31,719196	3,870241152	1,9524235	0,0028397	0,026901137	apicoplast ribosomal protein L33 precursor, putative
PFIT_1307400	13326,45341	15158,224	20149,92813	20671,13057	14242,33871	20410,529	1,433088327	0,5191275	0,002848	0,026934744	carbamoyl phosphate synthetase
PFIT_0725100	128,0399168	140,1210562	69,57500769	76,24268425	134,0804865	72,908846	0,543769253	-0,878934	0,0028588	0,026991674	serine/threonine protein kinase, FIKK family
PFIT_1340700	1016,960719	1009,590174	782,0901467	604,5911102	1013,275446	693,34063	0,684256814	-0,54739	0,0029352	0,027665875	conserved Plasmodium protein, unknown function
PFIT_0730200	1387,834961	2075,468293	804,7229805	642,7124524	1731,651627	723,71772	0,417934939	-1,25865	0,0029931	0,028024545	Plasmodium exported protein (PHISTb), unknown function
PFIT_0217600	547,4810237	469,4654189	314,3449143	339,7481018	508,4732213	327,04651	0,64319318	-0,636676	0,0029916	0,028024545	conserved Plasmodium protein, unknown function
PFIT_0420500	1145,000636	1352,108311	777,8988812	931,632098	1248,554473	854,76549	0,684604082	-0,546658	0,0029899	0,028024545	alpha tubulin 2
PFIT_1412000.2	4320,979262	4087,462946	6026,20157	5942,916599	4204,221104	5984,5591	1,423464403	0,5094064	0,0029925	0,028024545	conserved Plasmodium protein, unknown function
PFIT_0724400	607,8216741	597,6103164	237,2256286	412,6468086	602,7159953	324,93622	0,539119952	-0,891322	0,003013	0,028118035	unspecified product
PFIT_0209200	468,0079719	479,0463458	803,0464743	1137,621105	473,5271588	970,33379	2,049161851	1,0350339	0,0030127	0,028118035	unspecified product
PFIT_0506800	3471,794986	3680,273552	2788,868079	2018,424746	3576,034269	2403,6464	0,672154189	-0,573136	0,0030255	0,028187763	transcription factor 25, putative (TCF25)
PFIT_0729100	525,405176	634,7364082	383,919922	390,5765579	580,0707921	387,24824	0,667587897	-0,58297	0,003107	0,028783997	adapter-related protein, putative
PFIT_0107600	1598,291376	1652,709893	2462,787622	2250,496776	1625,500634	2356,6422	1,449794697	0,5358486	0,0030978	0,028783997	LCCL domain-containing protein
PFIT_1036500	866,8449542	1079,051894	1452,692631	1425,203159	972,9484241	1438,9479	1,478955985	0,5645791	0,003115	0,028783997	ADP/ATP transporter on adenylate translocase
PFIT_1143700	160,4178268	144,9115196	236,3873755	262,836622	152,6646732	249,612	1,635034442	0,709321	0,00311	0,028783997	alpha/beta hydrolase, putative
PFIT_1231500	64,75582001	40,7189394	171,8418865	115,0328219	52,7373797	143,43735	2,719842263	1,443523	0,0031078	0,028783997	conserved Plasmodium protein, unknown function
PFIT_0613700	2004,486974	1663,488436	1317,733881	1098,162171	1833,987705	1207,948	0,658645651	-0,602426	0,0031719	0,029166881	conserved Plasmodium protein, unknown function
PFIT_1447700	8871,547341	9403,679769	7035,458308	5962,311668	9137,613555	6498,885	0,711223445	-0,491625	0,0031665	0,029166881	conserved Plasmodium protein, unknown function
PFIT_1138500	234,0039859	257,4874109	414,0970337	365,1623299	245,7456984	389,62968	1,585499499	0,6649374	0,0031705	0,029166881	conserved Plasmodium protein, unknown function
PFIT_0314400	550,42447	529,3462121	741,0157446	854,7206182	539,8853411	797,86818	1,477847463	0,5634974	0,0031854	0,029243203	conserved Plasmodium protein, unknown function
PFIT_0323800	1810,219514	1808,399956	1271,62996	1231,921267	1809,309735	1251,7756	0,691852583	-0,531463	0,003198	0,029257574	U4/U6.U5 tri-snRNP-associated protein 1, putative
PFIT_0820200	2980,239443	2961,704033	1952,291481	2168,903728	2970,971738	2060,5976	0,69357698	-0,527872	0,0032025	0,029257574	asparagine-rich antigen
PFIT_0413700	133,9268096	186,8280749	260,6967156	273,5373497	160,3774422	267,11703	1,665552393	0,7360007	0,0031992	0,029257574	conserved Plasmodium protein, unknown function
PFIT_1221000	1923,542199	1580,852941	1243,129354	1060,709625	1752,19757	1151,9195	0,657414158	-0,605126	0,0032394	0,029547372	conserved Plasmodium protein, unknown function
PFIT_1416700	354,6852868	362,877607	234,7108693	223,377689	358,7814469	229,04428	0,638394993	-0,647479	0,0032473	0,029571572	serine C-palmitoyltransferase, putative
PFIT_1437900	128,0399168	120,9592023	191,1217079	258,1550537	124,4995596	224,63838	1,804330727	0,8514638	0,0032638	0,029673366	conserved Plasmodium protein, unknown function
PFIT_0615600	437,101785	392,8180036	243,0934004	284,2380773	414,9598943	263,66574	0,635400535	-0,654262	0,0032821	0,029719826	conserved Plasmodium protein, unknown function
PFIT_0628000	734,3898678	734,138525	1080,508252	1076,760716	734,2641964	1078,6345	1,469000517	0,5548349	0,0032746	0,029719826	nicotinate phosphoribosyltransferase, putative
PFIT_1358600	92,71856046	93,41403744	244,7699066	169,8740509	93,06629895	207,32198	2,227680493	1,1555423	0,0032847	0,029719826	conserved Plasmodium protein, unknown function
PFIT_1477400	3877,990584	3188,053432	2204,605665	2487,91917	3533,022008	2346,2624	0,664095047	-0,590538	0,0033306	0,030087223	Plasmodium exported protein (PHISTb), unknown function
PFIT_1446700	441,5169546	455,0940285	691,5588114	658,7635438	448,3054916	675,16118	1,506029237	0,5907498	0,0033502	0,030192893	conserved Plasmodium protein, unknown function
PFIT_1137700	245,7777714	316,1705883	407,3910089	470,1632196	280,9741798	438,77711	1,561627885	0,6430507	0,003353	0,030192893	U2 spliceosomal RNA
PFIT_1405300	39,73652591	38,32370767	14,25030278	12,70711404	39,03011679	13,478708	0,345341227	-1,533906	0,003374	0,030258952	secreted ookinete adhesive protein
PFIT_0506700	1554,13968	1585,643405	1204,569712	984,4669405	1569,891542	1094,5183	0,697193594	-0,520369	0,003371	0,030258952	GTPase-activating protein, putative
PFIT_0621800	11124,75553	11268,36767	8160,393975	7694,49195	11196,5616	7927,443	0,708024771	-0,498128	0,0033764	0,030258952	transcription factor with AP2 domain(s), putative
PFIT_0317900	388,53492	488,6272728	273,2705121	298,9515777	438,5810964	286,11104	0,65235608	-0,616268	0,0033822	0,03026327	conserved Plasmodium protein, unknown function
PFIT_0526600	4794,874127	4223,991154	3151,831674	3126,61885	4509,432641	3139,2253	0,696146392	-0,522537	0,0034037	0,030407299	conserved Plasmodium protein, unknown function, putative
PFIT_1465500	782,9567328	877,8524287	635,3958534	465,4816512	830,4045808	550,43875	0,662856113	-0,593232	0,0034465	0,030741173	zinc finger protein, putative
PFIT_0219400	22755,78384	28652,95956	10880,5253	16376,79482	25704,3717	13628,66	0,530207866	-0,91537	0,0034661	0,03086655	liver stage antigen 3
PFIT_0512600	284,0425741	283,8349599	419,1265524	454,7809236	283,938767	436,95374	1,538901301	0,6219007	0,0034912	0,031022145	conserved Plasmodium protein, unknown function
PFIT_1207100	219,2867541	174,8519162	292,5503336	325,7033968	197,0693352	309,12687	1,568619821	0,6494957	0,0034945	0,031022145	conserved Plasmodium protein, unknown function
PFIT_1416800	325,2508232	340,1229055	472,7747511	539,7179491	332,6868644	506,24635	1,521690227	0,6056747	0,0035106	0,031116176	small subunit rRNA dimethylase, putative
PFIT_0814300	4167,920051	4829,984782	3233,980478	3161,396215	4498,952417	3197,6883	0,710762873	-0,49256	0,0035369	0,031295055	conserved protein, unknown function
PFIT_1465200	306,1184218	283,8349599	434,2151083	466,8192422	294,9766909	450,51718	1,527297543	0,6109812	0,0035419	0,031295055	conserved Plasmodium protein, unknown function
PFIT_1449700	4369,546127	3559,31435	2606,967156	2738,048678	3964,430238	2672,5079	0,674121565	-0,568919	0,0035476	0,031296306	conserved Plasmodium protein, unknown function
PFIT_1205500	2740,348565	2970,087344	1821,523997	2164,890956	2855,217954	1993,2075	0,698092933	-0,518509	0,0035554	0,031316496	conserved protein, unknown function
PFIT_1435100	516,5748369	450,3035651	704,132608	723,6367049	483,439201	713,88466	1,476679291	0,5623565	0,0035787	0,031472907	protein kinase, putative
PFIT_0305400	18359,74669	18001,36406	12489,97126	13175,27088	18180,55538	12832,621	0,705843183	-0,50258	0,0036171	0,031711582	conserved Plasmodium protein, unknown function
PFIT_1252600	3702,855526	3364,102964	4679,128831	5249,37569	3533,479245	4964,2523	1,404919038	0,490487	0,0036161	0,031711582	reticulocyte binding protein homologue 3, pseudogene
PFIT_1106400	13962,23783	16117,51431	23212,90498	19841,15538	15039,87607	21527,03	1,431330291	0,5173566	0,0036393	0,031856235	translocon component PTEX88
PFIT_0621100	4554,983248	4841,960941	3780,521502	2871,807774	4698,472094	3326,1646	0,70792474	-0,498332	0,0036894	0,03224487	radical SAM protein, putative
PFIT_1354700	66,22754319	58,68317736	32,69187108	22,73904618	62,45536028	27,715459	0,44376429	-1,172135	0,0037369	0,032498471	conserved Plasmodium protein, unknown function
PFIT_0404800	7162,876727	8138,997416	5664,076229	5283,48426	7650,937071	5473,7802	0,715439193	-0,483099	0,0037414	0,032498471	vacuolar ATP synthase subunit b
PFIT_1228700	1212,699902	1270,670432	1702,492056	1869,283355	1241,685167	1785,8877	1,438277393	0,5243419	0,0037328	0,032498471	conserved Plasmodium membrane protein, unknown function
PFIT_1464200	234,0039859	312,5777407	397,3319716	460,1312874	273,2908633	428,73163	1,568774105	0,6496376	0,0037325	0,032498471	conserved Plasmodium protein, unknown function
PFIT_1412100.1	163,3612732	198,8042335	336,139495	491,5646748	181,0827534	413,85208	2,285430706	1,1924661	0,0037604	0,032613199	conserved Plasmodium membrane protein, unknown function
PFIT_0809400	7396,880712	7655,160606	6044,643138	4739,753538	7526,020659	5392,1983	0,716474028	-0,481014	0,0037755	0,032694192	DnaJ protein, putative
PFIT_0911500	687,294726	576,0532309	868,4302165	959,0527125	631,6739784	913,74146	1,446539664	0,5326059	0,0038574	0,033352167	cysteine repeat modular protein 1
PFIT_0208400	1461,42112	1710,195455	1225,526039	1009,212373	1585,808287	1117,3692	0,704605478	-0,505112	0,0038812	0,033506732	phospholipase A2, putative
PFIT_0403300	5686,738375	6779,703409	4552,552612	3369,391608	6233,220892	3960,9721	0,635461534	-0,654123	0,0039511	0,034057808	conserved Plasmodium membrane protein, unknown function
PFIT_1205800	5813,306569	6998,867113	4408,373078	4749,116674	6406,086841	4578,7449	0,714749111	-0,484491	0,0039847	0,034295405	eukaryotic translation initiation factor 3 subunit 8, putative
PFIT_1364800	1172,963376	1251,508578	1810,626706	1694,05894	1212,235977	1752,3428	1,445545963	0,5316145	0,0040023	0,034394252	conserved Plasmodium protein, unknown function
PFIT_1413300	707,8988505	850,3072639	1089,729036	1192,462334	779,1030572	1141,0957	1,4646274	0,5505337	0,0040372	0,03464105	nuclear transport factor 2, putative
PFIT_1141800	1858,786379	1568,876783	2450,213825	2390,275031	1713,831581	2420,2444	1,412183353	0,4979274	0,0040512	0,034708711	conserved Plasmodium protein, unknown function
PFIT_0932700	14269,82797	13042,03677	10757,30209	8179,36867	13655,93237	9468,3354	0,69334961	-0,528345	0,0040735	0,034846708	profilin, putative
PFIT_0614700	953,6766219	936,5356061	717,5446577	586,5336324	945,106114	652,03915	0,689911043	-0,535518	0,0041224	0,035211594	RNA and export factor binding protein, putative
PFIT_0512500	63,28409682	91,01880571	148,3707995	135,7654816	77,15145127	142,06814	1,841418901	0,8808179	0,0041479	0,03532315	conserved Plasmodium protein, unknown function
PFIT_1112800	117,7378546	155,6900624	248,122919	353,7928068	136,7139585	300,95786	2,201368948	1,138401	0,004146	0,03532315	conserved Plasmodium protein, unknown function
PFIT_0507800	9511,746925	9634,819631	6918,102873	6718,719351	9573,283278	6818,4111	0,712233297	-0,489578	0,0041577	0,035353205	conserved Plasmodium protein, unknown function
PFIT_1140100	3259,866848	2741,342714	4287,664631	4117,773745	3000,604781	4202,7192	1,400624039	0,4860698	0,0041934	0,035593204	unspecified product
PFIT_0923100	203,0977991	263,4754902	338,6542543	393,9205353	233,2866647	366,28739	1,570117292	0,6508723	0,0041986	0,035593204	DNA-directed RNA polymerase II, putative
PFIT_0609800	2434,230143	2612,000201	1627,887529	1912,086266	2523,115172	1769,9869	0,701508563	-0,511467	0,0042419	0,035906504	trancription factor, putative
PFIT_0625600	1265,681936	1468,27705	1961,512265	1979,634609	1366,979493	1970,5734	1,441553035	0,5276239	0,0042555	0,035967824	conserved protein, unknown function
PFIT_0218800.2	63,28409682	73,05456774	111,4876629	139,109459	68,16933228	125,29856	1,838048823	0,8781751	0,0043071	0,036349264	ribosome associated membrane protein RAMP4, putative
PFIT_0217000	968,3938537	968,8712345	673,1172431	658,7635438	968,6325441	665,94039	0,687505698	-0,540556	0,0043543	0,036540258	conserved Plasmodium protein, unknown function
PFIT_0929600	125877,9555	109706,4037	91293,30738	76761,66954	117792,1796	84027,488	0,713353711	-0,48731	0,0043528	0,036540258	high molecular weight rhoptry protein 2
PFIT_0933600	1773,426434	1619,176649	2363,035502	2429,065169	1696,301542	2396,0503	1,412514389	0,4982656	0,0043556	0,036540258	conserved Plasmodium protein, unknown function
PFIT_1351100	604,8782278	580,8436943	833,2235861	894,1795513	592,8609611	863,70157	1,456836637	0,5428391	0,0043408	0,036540258	conserved Plasmodium protein, unknown function
PFIT_0729800	6312,220727	8865,950246	3956,554654	4038,187084	7589,085486	3997,3709	0,526726293	-0,924875	0,0044133	0,036966668	Plasmodium exported protein, unknown function
PFIT_0406400	23943,46445	20189,40825	15996,384	11952,04395	22066,43635	13974,214	0,633279146	-0,659087	0,0044243	0,037007027	conserved Plasmodium protein, unknown function
PFIT_1009200	1826,408469	1699,416912	1179,422118	1268,036222	1762,91269	1223,7292	0,694158887	-0,526677	0,0044671	0,037309361	tubulin-tyrosine ligase, putative
PFIT_0628500	260,4950032	238,3255571	403,1997434	365,1623299	249,4102801	384,18104	1,540357665	0,6232654	0,0045024	0,037535616	conserved Plasmodium protein, unknown function
PFIT_0835500	19,13240137	33,53324421	63,70723596	61,52918378	26,33282279	62,61821	2,377952807	1,2497201	0,0045075	0,037535616	rifin (RIF)
PFIT_0722200	1174,435099	1241,927652	1653,035123	1801,066217	1208,181375	1727,0507	1,42946308	0,5154734	0,0045296	0,037664372	Rab GTPase activator and protein kinase, putative
PFIT_1350800	762,3526082	750,9051471	1176,907359	1023,925874	756,6288777	1100,4166	1,454367721	0,5403921	0,0045515	0,037790815	glycerol kinase
PFIT_0518200	881,562186	918,5713681	570,8503643	660,1011347	900,0667771	615,47575	0,683811207	-0,54833	0,0045797	0,037913399	RAP protein, putative
PFIT_1355700	1012,545549	1142,525535	751,9130349	749,7197285	1077,535542	750,81638	0,696790363	-0,521203	0,0045768	0,037913399	regulator of chromosome condensation, putative
PFIT_1356000	266,3818959	279,0444964	396,4937185	436,7234458	272,7131962	416,60858	1,527643649	0,611308	0,0046172	0,038112483	protein kinase 5
PFIT_1018700	67,69926637	47,90463458	100,5903726	113,6952309	57,80195048	107,1428	1,85361914	0,8903448	0,0046164	0,038112483	conserved Plasmodium protein, unknown function
PFIT_0712300	6188,59598	6040,774421	4762,954141	3996,721764	6114,685201	4379,838	0,716281838	-0,481401	0,0046406	0,038249579	conserved Plasmodium protein, unknown function
PFIT_0600300	150,1157646	144,9115196	96,39910704	74,23629783	147,5136421	85,317702	0,578371608	-0,789931	0,0046655	0,038286992	Plasmodium exported protein (PHISTb), unknown function
PFIT_0204700	229,5888164	288,6254234	392,302453	411,9780132	259,1071199	402,14023	1,552023091	0,63415	0,0046644	0,038286992	conserved Plasmodium protein, unknown function
PFIT_1106300	94,19028364	87,42595811	159,2680899	155,8293459	90,80812008	157,54872	1,734962869	0,7949048	0,0046648	0,038286992	centrin-4
PFIT_1027000	78,00132864	87,42595811	139,1500154	153,8229595	82,71364338	146,48649	1,771007556	0,8245704	0,004682	0,038310718	topoisomerase, putative
PFIT_1314000	23,54757091	10,77854278	37,72138971	50,15966069	17,16305685	43,940525	2,560180602	1,3562456	0,0046774	0,038310718	ubiquitin-like protein, putative
PFIT_1220600	4977,367801	4401,238302	3374,807	2150,846251	4689,303052	2762,8266	0,589176386	-0,763228	0,004694	0,03835367	heterochromatin protein 1
PF_apicoplast_g	211,9281382	191,6185383	352,9045571	282,2316908	201,773383	317,56812	1,573885463	0,6543306	0,0047031	0,038372183	probable protein, unknown function
PFIT_bin09700	3171,563457	4597,647304	1689,918259	1997,692087	3884,605381	1843,8052	0,474644138	-1,075082	0,0047226	0,038475984	erythrocyte membrane protein 1, PfEMP1 (VAR)
PFIT_1448000	160,4178268	136,5282086	94,72260084	75,57388878	148,4730177	85,148245	0,573493057	-0,802152	0,0047445	0,038543007	heme detoxification protein
PFIT_0629100	19,13240137	37,1260918	57,00121112	72,89870687	28,12924658	64,949959	2,308983243	1,2072577	0,004738	0,038543007	secreted ookinete protein, putative
PFIT_1038200	2447,475652	3091,046546	1762,008026	1906,735902	2769,261099	1834,372	0,662404843	-0,594215	0,0047972	0,0387233	serine/threonine protein kinase, FIKK family
PFIT_0311200	3217,186876	2829,966288	2202,929159	2055,208497	3023,576582	2129,068	0,704155748	-0,506034	0,0047856	0,0387233	phosphatidylinositol 3- and 4-kinase, putative
PFIT_0505100	2175,206863	2257,505905	1539,0327	1589,726846	2216,356384	1564,3798	0,705830437	-0,502599	0,0048078	0,0387233	conserved Plasmodium protein, unknown function
PFIT_1466500	451,8190169	475,4534982	734,3097197	644,7188388	463,6362575	689,51428	1,487188001	0,572587	0,0047936	0,0387233	conserved Plasmodium protein, unknown function
PFIT_0912000	410,6107678	373,6561498	616,9542851	549,7498812	392,1334588	583,35208	1,487636594	0,5730221	0,0047765	0,0387233	conserved Plasmodium protein, unknown function
PFIT_0402200	270,7970655	305,3920455	434,2151083	446,7553779	288,0945555	440,48524	1,528960665	0,6125513	0,0048012	0,0387233	ubiquitin specific protease, putative
PFIT_1301050	191,3240137	180,8399956	113,1641691	109,0136626	186,0820046	111,08892	0,596989032	-0,744224	0,0048821	0,039265291	surface-associated interspersed protein 13.1 (SURFIN 13.1)
PFIT_0824400	235,4757091	313,7753565	424,156071	426,6915136	274,6255328	425,42379	1,549105023	0,631435	0,0048924	0,039292085	ribosome biogenesis protein nep1 homologue, putative
PFIT_0937100	157,4743805	208,3851604	113,1641691	66,8794759	182,9297705	90,021858	0,49211158	-1,022943	0,0049451	0,03965941	Plasmodium exported protein, unknown function
PFIT_1404900	398,8369823	529,3462121	292,5503336	268,1869858	464,0915972	280,36866	0,604123542	-0,727084	0,0049955	0,040006507	phosphatase, putative
PFIT_1140400	426,7997228	380,8418449	614,4395258	578,5080867	403,8207839	596,47381	1,47707555	0,5627436	0,0050085	0,040053596	adrenodoxin reductase, putative
PFIT_0404100	19747,58166	23405,00684	15401,22429	15885,23014	21576,29425	15643,227	0,725019183	-0,463909	0,0050507	0,040334307	pre-mRNA-processing-splicing factor 8, putative
PFIT_0317700	2825,708509	3295,838859	1898,643282	2345,465734	3060,773684	2122,0545	0,693306571	-0,528435	0,0050846	0,040490166	CPSF (cleavage and polyadenylation specific factor), subunit A, putative
PFIT_0831300	128,0399168	119,7615865	202,8572513	289,5884411	123,9007516	246,22285	1,987258697	0,9907797	0,0050818	0,040490166	mitochondrial inner membrane protein, putative
PFIT_0904300	1117,037895	1250,310963	723,4124294	920,2625749	1183,674429	821,8375	0,69431043	-0,526347	0,0050947	0,040513337	adapter-related protein, putative
PFIT_1123700	4447,547456	4552,137901	3290,143436	3150,026692	4499,842679	3220,0851	0,715599476	-0,482776	0,0051212	0,040667334	calcium-dependent protein kinase 6
PFIT_1412100.2	117,7378546	123,3544341	238,9021348	372,5190801	120,5461443	305,71061	2,536046335	1,3425811	0,0051323	0,040697872	conserved Plasmodium membrane protein, unknown function
PFIT_0407600	279,6274046	267,0683378	386,4346813	438,7298322	273,3478712	412,58226	1,509367002	0,5939436	0,0051827	0,04104025	conserved Plasmodium protein, unknown function
PFIT_1148700	6435,845475	4900,644118	7854,431591	7729,269315	5668,244796	7791,8505	1,374649602	0,4590639	0,0051953	0,04108188	unspecified product
PFIT_1103700	529,8203455	572,4603833	886,8717848	738,3502054	551,1403644	812,611	1,474417494	0,5601451	0,0052579	0,041518797	Plasmodium exported protein (hyp11), unknown function
PFIT_1220000	1421,684594	1144,920767	1790,508632	1801,066217	1283,30268	1795,7874	1,3993483	0,4847551	0,0052886	0,0417035	Cell cycle associated protein, putative
PFIT_0616500	164,8329964	179,6423797	121,5467002	87,61220735	172,2376881	104,57945	0,607181012	-0,719801	0,0053167	0,04180808	erythrocyte membrane protein 1, PfEMP1
PFIT_1304800	33,84963318	43,11417113	66,22199527	92,29377568	38,48190215	79,257885	2,059614547	1,0423744	0,0053099	0,04180808	membrane integral peptidase, M50 family, putative
PFIT_0108500	216,3433077	198,8042335	134,9587499	119,0455947	207,5737706	127,00217	0,611841139	-0,708771	0,0053467	0,041985573	conserved Plasmodium protein, unknown function
PFIT_1329100	185,4371209	198,8042335	317,6979267	288,9196456	192,1206772	303,30879	1,57874098	0,6587745	0,0053543	0,041986484	conserved Plasmodium protein, unknown function
PFIT_1205000	1346,626711	1349,713079	1838,289059	1970,940268	1348,169895	1904,6147	1,412740834	0,4984968	0,005362	0,041988427	conserved Plasmodium protein, unknown function
PFIT_1141500	1224,473687	1313,784603	1893,613764	1733,517874	1269,129145	1813,5658	1,428984454	0,5149902	0,005372	0,042008719	conserved Plasmodium protein, unknown function
PFIT_1140000	3435,001907	4716,211275	2451,052078	2054,539702	4075,606591	2252,7959	0,552751067	-0,855298	0,0053892	0,042084869	transcription factor with AP2 domain(s)
PFIT_1000900	85,35994455	114,971123	52,8099456	55,5100245	100,1655338	54,159985	0,540704801	-0,887087	0,0054386	0,042358992	Plasmodium exported protein (PHISTa), unknown function
PFIT_1451700	912,4683728	810,7859403	373,8608847	601,2471328	861,6271566	487,55401	0,565852649	-0,821502	0,0054393	0,042358992	conserved Plasmodium protein, unknown function
PFIT_1353200	1033,149674	1061,087656	1370,543826	1597,752392	1047,118665	1484,1481	1,417363818	0,5032101	0,0054633	0,042486974	conserved Plasmodium protein, unknown function
PFIT_0623400	86,83166773	69,46172015	129,9292312	184,5875514	78,14669394	157,25839	2,012348615	1,0088803	0,0055399	0,043023412	anaphase promoting complex subunit, putative
PFIT_0316100	915,4118192	710,1862077	1305,998337	1112,206876	812,7990134	1209,1026	1,487578832	0,5729661	0,0055657	0,043164941	mitochondrial ribosomal protein L27 precursor, putative
PFIT_1449500	4784,572065	4701,839884	3373,968747	3407,51295	4743,205974	3390,7408	0,714862662	-0,484262	0,0056133	0,043455237	conserved Plasmodium protein, unknown function
PFIT_1134300	138,3419791	168,8638369	230,5196038	264,174213	153,602908	247,34691	1,610300948	0,6873303	0,0056185	0,043455237	conserved Plasmodium protein, unknown function
PFIT_0413600	838,8822137	1010,78779	1297,615806	1365,011566	924,8350017	1331,3137	1,439514815	0,5255826	0,0056551	0,043677903	arsenical pump-driving ATPase, putative
PFIT_0702600	654,916816	706,5933601	910,3428717	1048,671306	680,755088	979,50709	1,438853864	0,5249201	0,0056779	0,043794217	conserved Plasmodium protein, unknown function
PFIT_1012600	1580,630697	1668,278899	2285,916217	2302,662824	1624,454798	2294,2895	1,412344328	0,4980919	0,0056973	0,043884196	U3 snoRNA-associated small subunit rRNA processing protein, putative
PFIT_1110800	85,35994455	105,3901961	180,2244175	151,1477776	95,37507032	165,6861	1,737205509	0,7967684	0,005721	0,044006452	conserved Plasmodium protein, unknown function
PFIT_0912300	538,6506846	589,2270054	422,4795648	352,4552158	563,938845	387,46739	0,687073419	-0,541464	0,0057529	0,044037603	conserved Plasmodium protein, unknown function
PFIT_1341200	16244,88048	17349,86103	13775,85152	10805,05971	16797,37076	12290,456	0,73168925	-0,450697	0,0057359	0,044037603	40S ribosomal protein S6, putative
PFIT_1036400	41,20824909	47,90463458	84,66356358	92,29377568	44,55644184	88,47867	1,985760053	0,9896957	0,0057453	0,044037603	conserved Plasmodium protein, unknown function
PFIT_0801600	19,13240137	27,54516489	62,03072975	105,6696852	23,33878313	83,850207	3,592741191	1,845085	0,0057562	0,044037603	rifin, pseudogene
PFIT_0214700	3155,374502	3080,268004	5037,062906	4057,582152	3117,821253	4547,3225	1,45849366	0,5444791	0,0057793	0,04415452	acyl-CoA synthetase
PFIT_0302400	150180,5204	125301,7574	100294,4692	75962,45895	137741,1389	88128,464	0,639812222	-0,64428	0,0058269	0,044397946	cytoadherence linked asexual protein 3.1 (CLAG3.1)
PFIT_1316000	501,857605	572,4603833	373,0226316	360,4807615	537,1589942	366,7517	0,682761902	-0,550546	0,0058208	0,044397946	sec20 homolog, putative
PFIT_1011000	253,1363873	222,7565508	384,7581751	341,0856927	237,9464691	362,92193	1,525225129	0,6090222	0,0058616	0,044542748	conserved Plasmodium membrane protein, unknown function
PFIT_0718700	10,30206227	14,37139038	31,01536488	42,13411498	12,33672632	36,57474	2,964703862	1,567888	0,0058617	0,044542748	conserved Plasmodium protein, unknown function
PFIT_bin00500	950,7331755	1118,573218	722,5741763	416,6595815	1034,653197	569,61688	0,550538945	-0,861083	0,0058858	0,044665484	rifin (RIF)
PFIT_1437300	824,1649819	910,1880571	544,8645181	655,4195664	867,1765195	600,14204	0,692064451	-0,531022	0,0059218	0,044818712	conserved Plasmodium membrane protein, unknown function
PFIT_0832200	145,700595	140,1210562	211,2397824	245,4479397	142,9108256	228,34386	1,597806605	0,6760928	0,0059204	0,044818712	U6 snRNA-associated Sm-like protein LSm8, putative
PFIT_0829000	27663,98065	32363,17351	22915,32512	21084,44617	30013,57708	21999,886	0,732997789	-0,448119	0,0059625	0,045066053	E3 ubiquitin-protein ligase, putative
PFIT_0524600	4825,780314	4231,17685	3317,805789	3139,994759	4528,478582	3228,9003	0,713020988	-0,487984	0,0060192	0,045379985	conserved Plasmodium protein, unknown function
PFIT_1406000	335,5528855	368,8656863	543,1880119	510,2909481	352,2092859	526,73948	1,495529792	0,5806567	0,0060201	0,045379985	conserved Plasmodium protein, unknown function
PFIT_0417500	156,0026573	213,1756239	317,6979267	444,7489915	184,5891406	381,22346	2,06525399	1,0463192	0,0060568	0,045595581	conserved Plasmodium protein, unknown function
PFIT_0601800	1124,396511	1052,704345	1560,827281	1511,477776	1088,550428	1536,1525	1,411190964	0,4969132	0,0061424	0,046116834	conserved Plasmodium protein, unknown function
PFIT_1248200	60,34065046	53,89271391	90,53133531	119,0455947	57,11668218	104,78847	1,834638516	0,8754958	0,006139	0,046116834	conserved Plasmodium protein, unknown function
PFIT_0832400	485,66865	485,0344252	642,1018782	761,7580471	485,3515376	701,92996	1,446230017	0,532297	0,0061564	0,04616042	conserved Plasmodium protein, unknown function
PFIT_0211500	4855,214777	4976,093917	3556,707923	3529,902522	4915,654347	3543,3052	0,720820662	-0,472288	0,0062686	0,046862964	conserved Plasmodium protein, unknown function
PFIT_0601000	2135,470337	2489,843382	1679,020969	1656,606394	2312,65686	1667,8137	0,721167809	-0,471593	0,0062833	0,046862964	ATP-dependent RNA helicase, putative
PFIT_1118300	310,5335914	250,3017157	392,302453	437,3922412	280,4176535	414,84735	1,479390979	0,5650034	0,0062681	0,046862964	tyrosine--tRNA ligase
PFIT_1132100	278,1556814	228,7446301	419,9648055	345,767261	253,4501558	382,86603	1,510616682	0,5951376	0,0062824	0,046862964	conserved Plasmodium protein, unknown function
PFIT_1249500	1376,061175	1475,462745	934,6522118	1076,760716	1425,76196	1005,7065	0,705381748	-0,503524	0,006317	0,047052731	protein phosphatase, putative
PFIT_0906800	18966,09665	20382,2244	29467,11139	25392,82663	19674,16052	27429,969	1,394212931	0,4794509	0,0063418	0,04717525	zinc finger protein, putative
PFIT_0927300	765,2960546	840,7263369	570,8503643	551,0874722	803,0111958	560,96892	0,69858169	-0,517499	0,0063641	0,047216739	zinc finger protein, putative
PFIT_0727000	4649,173532	4354,531284	3582,69377	2921,967434	4501,852408	3252,3306	0,722442743	-0,469045	0,0063588	0,047216739	conserved Plasmodium membrane protein, unknown function
PFIT_0212400	52,98203455	55,09032977	86,34006979	115,0328219	54,03618216	100,68645	1,863315316	0,8978718	0,0064356	0,047651397	conserved Plasmodium protein, unknown function
PFIT_0533100	42,67997228	34,73086007	79,63404495	76,24268425	38,70541617	77,938365	2,013629417	1,0097982	0,0064396	0,047651397	conserved Plasmodium protein, unknown function
PFIT_0407100	403,2521519	301,7991979	181,0626706	212,6769613	352,5256749	196,86982	0,558455256	-0,840486	0,0064534	0,047690655	flap endonuclease 1
PFIT_bin00400	132,4550864	102,9949644	56,16295802	71,56111592	117,7250254	63,862037	0,542467812	-0,882391	0,0064941	0,047866548	erythrocyte membrane protein 1, PfEMP1, putative
PFIT_1145700	473,8948646	397,608467	571,6886174	679,4962035	435,7516658	625,59241	1,435662694	0,5217168	0,0064888	0,047866548	conserved Plasmodium protein, unknown function
PFIT_0201400	927,1856046	1000,009247	1377,249851	1365,011566	963,5974258	1371,1307	1,422928987	0,5088637	0,006542	0,048156633	liver stage associated protein 2 (LSAP2)
PFIT_1319000	44,15169546	45,50940285	15,92680899	20,06386428	44,83054916	17,995337	0,401407901	-1,316859	0,0065874	0,048428007	probable protein, unknown function
PFIT_1341100	2863,973312	3176,077273	2383,153577	1734,186669	3020,025292	2058,6701	0,68167314	-0,552848	0,006614	0,048459032	vacuolar ATP synthase subunit d, putative
PFIT_1412000.1	4475,510196	4143,750891	5994,347952	5892,088143	4309,630544	5943,218	1,379055115	0,4636801	0,0066174	0,048459032	conserved Plasmodium protein, unknown function
PFIT_1026800	320,8356537	401,2013146	513,0109001	642,7124524	361,0184842	577,86168	1,600642908	0,6786515	0,0066148	0,048459032	DNA-directed RNA polymerase II, putative
PFIT_0710800	14028,46537	18632,50762	10105,14118	10152,31532	16330,4865	10128,728	0,620234324	-0,689115	0,0066426	0,048580538	erythrocyte membrane protein 1, PfEMP1
PFIT_1415500	8522,748947	8641,996079	4828,337883	6746,808761	8582,372513	5787,5733	0,674355875	-0,568418	0,006666	0,048638182	atypical protein kinase, ABC-1 family, putative
PFIT_0406500	4175,278667	3693,447326	5897,948845	4988,545455	3934,362997	5443,2471	1,383514219	0,4683375	0,0066677	0,048638182	AAA family ATPase, putative
PFIT_0804600	8717,016407	9960,571146	7180,476095	6519,418299	9338,793776	6849,9472	0,733493785	-0,447143	0,0066865	0,048711993	mannose-6-phosphate isomerase, putative
PFIT_1339500	3327,566114	3650,333155	2779,647295	2312,694756	3488,949635	2546,171	0,729781537	-0,454463	0,0067877	0,049258545	nucleolar complex protein 2, putative
PFIT_0313800	339,968055	304,1944296	447,6271579	498,2526296	322,0812423	472,93989	1,468386952	0,5542322	0,0067801	0,049258545	conserved Plasmodium protein, unknown function
PFIT_0915600	52,98203455	41,91655526	79,63404495	99,65052591	47,44929491	89,642285	1,889222708	0,9177928	0,0067824	0,049258545	conserved Plasmodium protein, unknown function
PFIT_0603300	14,71723182	21,55708556	38,55964282	53,50363807	18,13715869	46,03164	2,537974179	1,3436774	0,0068005	0,049288412	conserved Plasmodium protein, unknown function
PFIT_0928600	501,857605	578,4484626	408,229262	282,2316908	540,1530338	345,23048	0,639134569	-0,645808	0,0068217	0,049378431	conserved Plasmodium membrane protein, unknown function
PFIT_0929800	356,15701	302,9968137	239,740388	191,9443016	329,5769119	215,84234	0,654907358	-0,610637	0,0068397	0,049382219	conserved Plasmodium protein, unknown function
PFIT_0420900	2602,006586	2971,28496	2113,236077	1938,169289	2786,645773	2025,7027	0,726932251	-0,460107	0,006835	0,049382219	eukaryotic initiation factor, putative
PFIT_1363200	3348,170239	3543,745343	5148,550569	4482,936075	3445,957791	4815,7433	1,397505023	0,4828535	0,006853	0,049415331	conserved Plasmodium protein, unknown function
PFIT_1448100	922,7704351	1003,602095	1715,065852	1333,578179	963,1862648	1524,322	1,5825828	0,662281	0,0069983	0,050398533	glutaminyl-peptide cyclotransferase, putative
PFIT_1223800	1655,68858	1542,529234	2010,130945	2405,657327	1599,108907	2207,8941	1,380702794	0,4654028	0,007044	0,050663077	conserved Plasmodium protein, unknown function
PFIT_0937200	2154,602738	3113,801248	1487,061008	1052,684079	2634,201993	1269,8725	0,482071059	-1,052682	0,0070795	0,050853507	Plasmodium exported protein (PHISTb), unknown function
PFIT_1446200	167,7764427	173,6543004	79,63404495	123,0583676	170,7153716	101,34621	0,59365601	-0,752301	0,0071181	0,051065867	conserved Plasmodium protein, unknown function
PFIT_0833100	301123,3934	98252,40553	3399,954593	8650,200686	199687,8994	6025,0776	0,030172472	-5,050623	0,007181	0,051451597	unspecified product
PFIT_0926300	156,0026573	161,6781417	246,4464128	254,1422808	158,8403995	250,29435	1,575759993	0,6560478	0,0071968	0,051499607	conserved Plasmodium protein, unknown function
PFIT_1353700	771,1829473	997,6140152	568,335605	590,5464052	884,3984813	579,44101	0,655180914	-0,610035	0,0072287	0,051619242	adenylosuccinate synthetase
PFIT_1113800	3699,912079	3259,910383	2365,550262	2591,582469	3479,911231	2478,5664	0,712249882	-0,489545	0,0072318	0,051619242	conserved Plasmodium protein, unknown function
PFIT_bin07200	33,84963318	37,1260918	12,57379657	13,37590952	35,48786249	12,974853	0,365613822	-1,451607	0,007347	0,052358213	rifin
PFIT_0605500	4145,844204	5255,138414	3246,554275	3199,517557	4700,491309	3223,0359	0,685680646	-0,544391	0,0073655	0,052358213	conserved Plasmodium protein, unknown function
PFIT_0922800	1059,640691	1101,806595	832,385333	707,5856135	1080,723643	769,98547	0,712472127	-0,489095	0,0073717	0,052358213	pre-mRNA splicing factor, putative
PFIT_0620200	1408,439085	1451,510428	1990,012871	2009,740465	1429,974757	1999,8716	1,398536323	0,4839177	0,0073724	0,052358213	mRNA binding Pumilio-homology domain protein, putative
PFIT_0111600	696,1250651	603,5098259	483,6720414	411,9780132	649,8617304	447,82503	0,689108169	-0,537198	0,0074028	0,052507482	Plasmodium exported protein, unknown function
PFIT_0401700	70,64271273	107,7854278	44,42741455	34,77736475	89,21407027	39,60239	0,443902958	-1,171684	0,0074629	0,052801072	tRNA Asparagine
PFIT_0610700	445,9321241	438,3274064	633,7193472	859,4021866	442,1297653	746,56077	1,688555771	0,7557898	0,0074614	0,052801072	60S ribosomal protein L39, putative
PFIT_1402600	83,88822137	69,46172015	134,9587499	129,7463223	76,67497076	132,35254	1,726150461	0,7875582	0,0074933	0,052949957	hypothetical protein
PFIT_1226300	409,1390446	355,6919118	247,2846659	257,4862582	382,4154782	252,38546	0,65997711	-0,599512	0,0075451	0,053249309	proliferating cell nuclear antigen 2
PFIT_1439800	339,968055	532,9390597	734,3097197	707,5856135	436,4535574	720,94767	1,651831345	0,7240664	0,0075871	0,05347898	conserved protein, unknown function
PFIT_0216200	485,66865	444,3154858	307,6388894	318,3466465	464,9920679	312,99277	0,673114209	-0,571077	0,0076919	0,053814765	conserved Plasmodium membrane protein, unknown function
PFIT_0833700	442,9886778	505,3938949	300,0946115	345,767261	474,1912863	322,93094	0,681014067	-0,554241	0,0076882	0,053814765	surface-associated interspersed protein 8.2 (SURFIN 8.2) (SURF8.2)
PFIT_1207800	1415,797701	1234,741956	2030,24902	1688,039781	1325,269829	1859,1444	1,402842169	0,4883527	0,0076859	0,053814765	cysteine repeat modular protein 3
PFIT_1302900	754,9939923	686,2338904	1013,448004	1019,244305	720,6139414	1016,3462	1,410389247	0,4960934	0,0076491	0,053814765	conserved Plasmodium protein, unknown function
PFIT_0918400	910,9966496	1070,668583	1407,426963	1405,139295	990,8326163	1406,2831	1,419294445	0,5051738	0,0076858	0,053814765	eukaryotic translation initiation factor 3 subunit 5, putative
PFIT_0829500	66,22754319	56,28794564	107,2963974	112,35764	61,25774441	109,82702	1,792867493	0,842689	0,0076868	0,053814765	SNARE protein, putative
PFIT_0928100.1	97,13373001	108,9830437	149,2090526	191,2755061	103,0583868	170,24228	1,651901263	0,7241275	0,0077125	0,053892332	cytochrome c oxidase subunit 6B, putative (COX6B)
PFIT_0409900	113,322685	116,1687389	72,08976701	58,85400188	114,7457119	65,471884	0,570582406	-0,809493	0,0077663	0,054200696	conserved Plasmodium protein, unknown function
PFIT_1416200	126,5681936	142,5162879	201,1807451	275,5437361	134,5422408	238,36224	1,771653566	0,8250965	0,0078431	0,054669107	DNA-directed RNA polymerase I/III subunit, putative
PFIT_0201600	22,07584773	27,54516489	56,16295802	56,84761545	24,81050631	56,505287	2,277474149	1,1874347	0,0078686	0,054779355	Plasmodium exported protein (hyp11), unknown function
PFIT_1315700	1046,395182	1077,854278	792,9874371	718,9551366	1062,12473	755,97129	0,711753776	-0,49055	0,0078966	0,054867426	conserved Plasmodium protein, unknown function
PFIT_1434600	11589,82006	9847,995254	7184,667361	8186,725421	10718,90766	7685,6964	0,717022353	-0,47991	0,0079007	0,054867426	conserved Plasmodium membrane protein, unknown function
PFIT_0701400	3716,101034	5324,600134	2620,379205	1953,551585	4520,350584	2286,9654	0,505926554	-0,983	0,0079244	0,0549649	Plasmodium exported protein, unknown function
PFIT_1147500	133,9268096	99,40211676	72,08976701	56,84761545	116,6644632	64,468691	0,552599219	-0,855695	0,0079375	0,054968008	conserved Plasmodium membrane protein, unknown function
PFIT_0911200	54,45375773	69,46172015	120,7084471	187,9315287	61,95773894	154,31999	2,490729819	1,3165685	0,0079444	0,054968008	phosphatidylinositol N-acetylglucosaminyltransferase, putative
PFIT_0535500	275,212235	228,7446301	176,8714051	145,7974137	251,9784326	161,33441	0,640270708	-0,643246	0,0079858	0,055187205	conserved Plasmodium protein, unknown function
PFIT_0320700	238,4191555	215,5708556	310,1536488	367,8375118	226,9950056	338,99558	1,493405458	0,5786059	0,0080017	0,055229106	signal peptidase protein
PFIT_0401600	16,188955	10,77854278	32,69187108	55,5100245	13,48374889	44,100948	3,270674065	1,709588	0,0080431	0,055447509	probable protein, unknown function
PFIT_0829200	178,078505	147,3067513	97,23736015	96,97534401	162,6926282	97,106352	0,596870019	-0,744511	0,0080983	0,055759955	SPRY domain, putative
PFIT_0931600	148,6440414	168,8638369	229,6813507	267,5181904	158,7539391	248,59977	1,56594395	0,6470326	0,0081527	0,056065833	selenoprotein
PFIT_0314000.2	19,13240137	21,55708556	53,6481987	44,80929689	20,34474346	49,228748	2,419728117	1,274845	0,0081994	0,056318775	vesicle transport v-SNARE protein, putative
PFIT_0612100	153,0592109	190,4209225	252,3141845	284,2380773	171,7400667	268,27613	1,562105664	0,643492	0,0082135	0,05634656	conserved Plasmodium protein, unknown function
PFIT_0918300	681,4078332	652,7006462	857,5329261	1016,569123	667,0542397	937,05102	1,404759867	0,4903235	0,008304	0,05689865	50S ribosomal protein L3, apicoplast, putative
PFIT_0815800	275,212235	325,7515152	442,5976393	452,7745372	300,4818751	447,68609	1,489893818	0,5752095	0,0083278	0,056992274	probable protein, unknown function
PFIT_0824500	1412,854255	1520,972148	2261,606877	1861,926605	1466,913201	2061,7667	1,405513795	0,4910976	0,0083417	0,057018523	clp1-related protein, putative
PFIT_0704000	1789,615389	1415,581952	1106,494098	1019,244305	1602,598671	1062,8692	0,663216076	-0,592449	0,0084521	0,05767943	conserved Plasmodium protein, unknown function
PFIT_1427200	1043,451736	1322,167915	1628,725783	1710,110032	1182,809825	1669,4179	1,411400101	0,497127	0,0084588	0,05767943	arginine methyltransferase 1
PFIT_0209000	2872,803651	3307,815018	3968,290198	4557,172373	3090,309335	4262,7313	1,3793866	0,4640269	0,0084862	0,057796534	RING zinc finger protein, putative
PFIT_0111400	3295,188204	5028,789015	1611,122467	2061,896452	4161,98861	1836,5095	0,441257685	-1,180307	0,0085274	0,058006896	glutamic acid-rich protein
PFIT_1204000	3895,651263	4982,081997	6050,51091	6285,339883	4438,86663	6167,9254	1,389527082	0,474594	0,0085521	0,058105126	eukaryotic translation initiation factor 5A
PFIT_1341000	238,4191555	196,4090018	314,3449143	334,397738	217,4140786	324,37133	1,491951801	0,5772009	0,0085929	0,058172027	conserved Plasmodium protein, unknown function
PFIT_0212800	64,75582001	38,32370767	86,34006979	99,65052591	51,53976384	92,995298	1,804340783	0,8514718	0,0085855	0,058172027	protein kinase 7
PF_apicoplast_g	17,66067818	19,16185383	47,78042697	43,47170593	18,41126601	45,626066	2,478160189	1,3092694	0,0085912	0,058172027	ribosomal protein L23, putative
PFIT_0518900	27,96274046	23,95231729	10,05903726	6,019159283	25,95752887	8,0390983	0,309701987	-1,691047	0,0086308	0,058358653	secreted ookinete protein, putative
PFIT_0800300	33,84963318	40,7189394	16,76506209	12,70711404	37,28428629	14,736088	0,39523589	-1,339214	0,0086684	0,058543034	rifin (RIF)
PFIT_1313700	367,9307955	409,5846257	269,0792466	259,4926447	388,7577106	264,28595	0,679821746	-0,556772	0,0087199	0,058820299	cop-coated vesicle membrane protein p24 precursor, putative
PFIT_0220200	1742,520247	1537,73877	2490,449974	2067,246816	1640,129509	2278,8484	1,389431983	0,4744952	0,0087884	0,059193258	cytoadherence linked asexual protein 2
PFIT_0215500	799,1456878	656,2934938	990,8151698	1033,28901	727,7195908	1012,0521	1,390717115	0,475829	0,0087962	0,059193258	conserved Plasmodium protein, unknown function
PFIT_1409700	15941,70551	16636,08197	11764,04407	12163,38332	16288,89374	11963,714	0,734470608	-0,445223	0,0088695	0,059615881	conserved Plasmodium protein, unknown function
PFIT_0804900	6787,587315	6173,709782	4038,703459	5191,190484	6480,648549	4614,947	0,712111903	-0,489824	0,00894	0,060018021	transcription factor with AP2 domain(s)
PFIT_0515000	259,02328	314,9729724	403,1997434	450,0993553	286,9981262	426,64955	1,486593501	0,5720102	0,0089632	0,060102602	conserved Plasmodium protein, unknown function
PFIT_1034800	14924,74479	14600,13501	10239,26167	11303,31234	14762,4399	10771,287	0,729641379	-0,454741	0,0090517	0,06062399	merozoite surface protein 3
PFIT_1466900	522,4617296	559,2866088	335,3012419	408,6340358	540,8741692	371,96764	0,687715665	-0,540116	0,0090647	0,060638718	dynein beta chain, putative
PFIT_1248100	800,617411	844,3191845	1216,305255	1104,850126	822,4682978	1160,5777	1,411091095	0,4968111	0,0092548	0,061837607	nucleolar rRNA processing protein, putative
PFIT_1142500	1234,77575	992,8235517	803,0464743	703,5728407	1113,799651	753,30966	0,676342156	-0,564175	0,0093477	0,06238451	phd finger protein, putative
PFIT_1016700	157,4743805	130,5401292	212,9162886	232,7408256	144,0072549	222,82856	1,547342579	0,6297926	0,0094158	0,062764539	enoyl-CoA hydratase-related protein, putative
PFIT_1422100	2191,395818	2274,272527	3321,158801	2859,100666	2232,834172	3090,1297	1,383949497	0,4687913	0,0094789	0,063111016	conserved Plasmodium protein, unknown function
PFIT_1428100	9351,329098	10008,47578	7356,509247	6959,485722	9679,902439	7157,9975	0,739470003	-0,435436	0,0094933	0,063132099	lipase, putative
PFIT_0809600	2687,38653	2801,223507	2060,426131	1953,551585	2744,295019	2006,9889	0,731331305	-0,451403	0,0095197	0,063233537	conserved Plasmodium membrane protein, unknown function
PFIT_0506500	1838,182254	1752,11201	1227,202545	1348,960475	1795,147132	1288,0815	0,717535341	-0,478878	0,0095722	0,063507186	conserved Plasmodium protein, unknown function
PFIT_1002400	156,0026573	137,7258244	98,07561325	78,91786616	146,8642409	88,49674	0,602575135	-0,730787	0,0095948	0,063533687	DnaJ protein, putative
PFIT_0903600	10387,42222	11589,32872	14075,10788	15861,15351	10988,37547	14968,131	1,362178671	0,4459159	0,0095987	0,063533687	DEAD/DEAH box helicase, putative
PFIT_0825900	575,4437641	564,0770722	390,6259468	399,2708991	569,7604182	394,94842	0,693183328	-0,528691	0,0096407	0,06366276	protein kinase, putative
PFIT_1011600	523,9334528	451,5011809	695,7500769	684,1777719	487,7173169	689,96392	1,414679981	0,5004757	0,0096378	0,06366276	conserved Plasmodium protein, unknown function
PFIT_1409000	2744,763734	3094,639394	4178,691727	3890,383283	2919,701564	4034,5375	1,381832155	0,4665824	0,0096827	0,063865069	plasmepsin II
PFIT_1217700	1930,900815	1803,609492	2736,058134	2400,306963	1867,255153	2568,1825	1,375378477	0,4598287	0,009758	0,06428662	thrombospondin-related apical membrane protein (TRAMP)
PFIT_1409300	2,943446364	3,592847594	18,4415683	16,7198869	3,268146979	17,580728	5,379417668	2,42745	0,0098158	0,064591943	conserved Plasmodium protein, unknown function
PFIT_0710200	229,5888164	231,1398619	173,5183927	99,65052591	230,3643391	136,58446	0,592906262	-0,754124	0,0098488	0,064700304	conserved Plasmodium protein, unknown function
PFIT_0934900	575,4437641	541,3223708	397,3319716	377,2006484	558,3830675	387,26631	0,693549523	-0,527929	0,0098666	0,064700304	transcription factor with AP2 domain(s), putative
PFIT_0625500	58,86892728	97,00688503	140,8265216	132,4215042	77,93790615	136,62401	1,752985417	0,809814	0,0098635	0,064700304	conserved Plasmodium protein, unknown function
PFIT_0732300	167,7764427	281,4397282	83,82531047	83,59943449	224,6080855	83,712372	0,372704181	-1,423897	0,0099351	0,064998048	Plasmodium exported protein (PHISTa), unknown function
PFIT_0417700	39,73652591	33,53324421	58,67771733	84,93702544	36,63488506	71,807371	1,960081798	0,9709139	0,0099316	0,064998048	conserved Plasmodium protein, unknown function
PFIT_1455500	79,47305183	97,00688503	150,0473057	147,8038002	88,23996843	148,92555	1,687733525	0,7550871	0,0100838	0,065895128	serine/threonine protein kinase, putative
PFIT_0323100	1757,237479	1748,519162	1421,677266	1141,633877	1752,878321	1281,6556	0,731172014	-0,451717	0,010164	0,066265706	translation machinery-associated protein 7, putative (TMA7)
PFIT_1139200	9283,629832	10648,00265	6916,426367	7821,563091	9965,816242	7368,9947	0,739427113	-0,43552	0,0101576	0,066265706	protein phosphatase 2c
PFIT_0707500	8,830339092	8,383311052	26,82409935	28,75820546	8,606825072	27,791152	3,2289668	1,6910726	0,0101831	0,066313506	heat shock protein 86 family protein
PFIT_1225200	510,6879441	474,2558824	676,4702555	714,9432638	492,4719133	695,70631	1,412682208	0,498437	0,0102063	0,066387817	small subunit rRNA processing factor, putative
PFIT_1130300	379,7045809	344,913369	498,7605973	539,0491536	362,308975	518,90488	1,432216454	0,5182495	0,0102281	0,066453269	A/G-specific adenine glycosylase, putative
PFIT_bin06100	82,41649819	87,42595811	46,10392076	45,47809236	84,92122815	45,791007	0,539217432	-0,891061	0,0102715	0,066505815	B_ITvar19 - erythrocyte membrane protein 1, PfEMP1, putative
PFIT_1237800	463,5928023	534,1366756	375,5373909	325,7033968	498,864739	350,62039	0,702836594	-0,508739	0,0102704	0,066505815	calcyclin binding protein, putative
PFIT_0219100	1944,146323	2219,182197	3023,578949	2746,074224	2081,66426	2884,8266	1,385827024	0,4707472	0,0102639	0,066505815	Plasmodium exported protein (PHISTc), unknown function
PFIT_0502000	153,0592109	143,7139038	76,28103253	100,3193214	148,3865573	88,300177	0,595068573	-0,748872	0,0102942	0,0665761	conserved protein, unknown function
PFIT_0808100	2465,13633	2109,001538	3358,880191	2872,476569	2287,068934	3115,6784	1,362301911	0,4460465	0,0103411	0,066733521	conserved Plasmodium protein, unknown function
PFIT_0817200	1548,252787	1714,985918	2335,37315	2186,961206	1631,619353	2261,1672	1,385842338	0,4707631	0,0103512	0,066733521	ATPase, putative
PFIT_0514800	173,6633355	177,247148	262,3732218	273,5373497	175,4552417	267,95529	1,527200231	0,6108892	0,010354	0,066733521	conserved Plasmodium protein, unknown function
PFIT_1000800	50,03858819	43,11417113	87,17832289	139,109459	46,57637966	113,14389	2,429211797	1,2804883	0,010438	0,067198213	acyl-CoA binding protein, isoform 2, ACBP2
PFIT_1235800	2576,987292	2875,475691	2042,822816	1975,621836	2726,231491	2009,2223	0,736996228	-0,440271	0,0104659	0,067301332	clustered-asparagine-rich protein
PFIT_0814200	20321,5537	20512,76453	15307,33995	14913,47032	20417,15911	15110,405	0,740083625	-0,43424	0,0104893	0,067374905	CCR4-associated factor 1
PFIT_0917100	1471,723182	1325,760762	979,9178794	1021,919487	1398,741972	1000,9187	0,715584935	-0,482805	0,0105433	0,067644631	conserved Plasmodium protein, unknown function
PFIT_1467700	104,4923459	114,971123	190,2834548	167,198869	109,7317345	178,74116	1,628892159	0,7038911	0,0105908	0,067872709	conserved Plasmodium protein, unknown function
PFIT_bin00800	39,73652591	33,53324421	17,6033152	11,36952309	36,63488506	14,486419	0,395426903	-1,338517	0,0106445	0,068061885	rifin (RIF)
PFIT_1353800	1387,834961	1428,755726	1083,861264	658,0947483	1408,295344	870,97801	0,618462605	-0,693242	0,0106407	0,068061885	60S ribosomal protein L7-2, putative
PFIT_0915800	279,6274046	253,8945633	168,4888741	179,2371875	266,7609839	173,86303	0,651755846	-0,617596	0,0106865	0,068173552	conserved Plasmodium protein, unknown function
PFIT_1410900	1764,596095	1838,340352	1404,912204	1234,596449	1801,468224	1319,7543	0,732599281	-0,448904	0,0106982	0,068173552	cytidine diphosphate-diacylglycerol synthase
PFIT_0520900	478,3100341	525,7533646	764,4868315	674,1458397	502,0316993	719,31634	1,432810591	0,5188479	0,0106935	0,068173552	conserved Plasmodium protein, unknown function
PFIT_1433500	1261,266767	1148,513614	1658,902894	1645,905666	1204,890191	1652,4043	1,371414834	0,455665	0,0107391	0,068357312	conserved Plasmodium protein, unknown function
PFIT_0301600	666,6906014	626,3530972	528,099456	389,9077625	646,5218493	459,00361	0,709958387	-0,494194	0,0107795	0,068537447	Plasmodium exported protein, unknown function
PFIT_0919400	1986,826296	2210,798886	1618,666745	1041,983351	2098,812591	1330,325	0,633846516	-0,657795	0,0109754	0,06914318	thioredoxin, putative
PFIT_1017100	1083,188262	1244,322883	641,2636251	892,1731649	1163,755573	766,71839	0,658831127	-0,602019	0,0109802	0,06914318	conserved Plasmodium protein, unknown function
PFIT_1135000	1171,491653	984,4402407	742,6922508	783,8282978	1077,965947	763,26027	0,708056017	-0,498065	0,0109799	0,06914318	conserved Plasmodium protein, unknown function
PFIT_0717700	1221,530241	1110,189906	844,9591296	824,6248218	1165,860074	834,79198	0,716031018	-0,481906	0,0109927	0,06914318	conserved membrane protein, unknown function
PFIT_1336400	1131,755127	1312,586988	860,0476855	914,9122111	1222,171057	887,47995	0,72615035	-0,46166	0,0109735	0,06914318	1-deoxy-D-xylulose 5-phosphate synthase
PFIT_0502100	1639,499625	1665,883668	1270,791707	1142,302673	1652,691646	1206,5472	0,73004979	-0,453933	0,0109167	0,06914318	conserved Plasmodium protein, unknown function
PFIT_0902700	3386,435042	3970,096591	5524,926213	4681,568331	3678,265816	5103,2473	1,387405785	0,4723898	0,0109705	0,06914318	serine/threonine protein kinase, FIKK family
PFIT_1430700	626,9540755	706,5933601	936,328718	943,6704165	666,7737178	939,99957	1,409772974	0,4954629	0,0109973	0,06914318	diphthamide synthesis protein, putative
PFIT_0621000	409,1390446	409,5846257	653,8374217	527,010835	409,3618351	590,42413	1,442303795	0,5283751	0,0109859	0,06914318	conserved Plasmodium protein, unknown function
PFIT_1224600	94,19028364	97,00688503	136,6352561	174,5556192	95,59858434	155,59544	1,627591441	0,7027386	0,010966	0,06914318	splicing factor 3b subunit, putative
PFIT_1425800	0	2,395231729	10,05903726	14,71350047	1,197615865	12,386269	10,34243887	3,3705045	0,011056	0,069435256	unspecified product
PFIT_0208100	244,3060482	304,1944296	186,9304424	181,9123694	274,2502389	184,42141	0,672456683	-0,572487	0,0111164	0,06973689	conserved Plasmodium protein, unknown function
PFIT_1208600	506,2727746	504,196279	405,7145027	305,6395325	505,2345268	355,67702	0,703983499	-0,506385	0,0111921	0,070133666	conserved Plasmodium protein, unknown function
PFIT_1211600	400,3087055	403,5965464	320,212686	238,0911894	401,9526259	279,15194	0,694489648	-0,525975	0,0112551	0,070450682	glutathione peroxidase
PFIT_0410500	1146,472359	1167,675468	822,3262957	845,3574158	1157,073913	833,84189	0,720647038	-0,472635	0,0112726	0,070481773	DNA polymerase alpha
PFIT_1346900	8405,011092	8519,839261	12230,1128	10766,26957	8462,425176	11498,191	1,358734753	0,4422638	0,0112855	0,070484321	ethanolamine-phosphate cytidylyltransferase, putative
PFIT_0307000	2085,431749	2700,623775	3327,026573	4107,741813	2393,027762	3717,3842	1,553422928	0,6354507	0,0113238	0,070645199	40S ribosomal protein S12, putative
PFIT_0104600	1409,910808	1586,841021	1233,90857	966,4094627	1498,375914	1100,159	0,734234317	-0,445688	0,0113393	0,070663951	vacuolar ATP synthase subunit c, putative
PFIT_1149700	2300,303333	2485,052919	3777,16849	3050,376166	2392,678126	3413,7723	1,426757862	0,5127405	0,0114251	0,071120047	ring-infected erythrocyte surface antigen 2, pseudogene
PFIT_0307300	192,7957368	141,318672	238,0638817	261,4990311	167,0572044	249,78146	1,495185181	0,5803242	0,0114433	0,071154891	ATP-dependent Clp protease proteolytic subunit
PFIT_1425400	18455,4087	20176,23447	14850,492	9195,937794	19315,82159	12023,215	0,622454232	-0,68396	0,011541	0,0716835	60S ribosomal protein L7-3, putative
PFIT_0518400	1439,345272	1571,272014	1158,465791	1051,346488	1505,308643	1104,9061	0,734006374	-0,446136	0,0116602	0,0723443	proteasome subunit beta type 1, putative
PFIT_1125800	3095,033852	3441,947995	2483,743949	1506,796207	3268,490923	1995,2701	0,610456056	-0,712041	0,0116921	0,072463019	60S ribosomal protein L35, putative
PFIT_0707100	269,3253423	268,2659537	372,1843785	411,3092177	268,795648	391,7468	1,457414958	0,5434117	0,0117084	0,072484408	conserved Plasmodium protein, unknown function
PFIT_0904800	3621,910751	3505,421636	2802,280129	2465,180124	3563,666193	2633,7301	0,739050737	-0,436255	0,0118504	0,073283171	ubiquitin specific protease, putative
PFIT_0608100	484,1969269	512,57959	830,7088268	652,7443845	498,3882585	741,72661	1,488250562	0,5736174	0,0119094	0,07348709	citrate synthase-like protein, putative
PFIT_1243500	7,35861591	13,17377451	33,53012419	28,75820546	10,26619521	31,144165	3,033661857	1,6010603	0,0119092	0,07348709	conserved Plasmodium protein, unknown function
PFIT_1136900	4079,61666	3329,372104	2770,426511	2318,713915	3704,494382	2544,5702	0,686887319	-0,541855	0,0119571	0,073628886	conserved Plasmodium protein, unknown function
PFIT_1447500	3161,261395	3012,003899	4600,333039	3800,76469	3086,632647	4200,5489	1,360883961	0,4445441	0,0119585	0,073628886	conserved Plasmodium membrane protein, unknown function
PFIT_1360800	85143,60125	79473,78877	64805,34753	57683,6098	82308,69501	61244,479	0,744082732	-0,426465	0,0119838	0,073704505	conserved Plasmodium protein, unknown function
PFIT_1449600	937,4876669	774,8574644	639,5871189	575,8329048	856,1725656	607,71001	0,709798511	-0,494519	0,012046	0,073947886	ubiquitin-protein ligase, putative
PFIT_0707700	13561,92912	14694,74666	11505,02386	9741,006107	14128,33789	10623,015	0,751894177	-0,411398	0,0120496	0,073947886	heat shock protein 110 (HSP110c)
PFIT_1319900	2546,081105	2106,606306	1526,458904	1758,263306	2326,343705	1642,3611	0,705983858	-0,502293	0,0121094	0,074106972	conserved Plasmodium protein, unknown function
PFIT_1418200	14522,96436	16010,92649	10788,31746	11944,0184	15266,94543	11366,168	0,744495222	-0,425666	0,0121149	0,074106972	NOT family protein, putative
PFIT_1232400	482,7252037	507,7891266	717,5446577	686,1841583	495,2571651	701,86441	1,417171638	0,5030145	0,0120981	0,074106972	conserved Plasmodium protein, unknown function
PFIT_0912700	78,00132864	105,3901961	137,4735092	165,861278	91,69576236	151,66739	1,654028384	0,725984	0,0121425	0,074195438	conserved Plasmodium protein, unknown function
PFIT_0813400	860,9580614	959,2903075	691,5588114	630,0053383	910,1241845	660,78207	0,726035069	-0,461889	0,0121875	0,074390072	protein phosphatase, putative
PFIT_0831500	468,0079719	439,5250223	283,3295494	338,4105108	453,7664971	310,87003	0,685088106	-0,545639	0,0122177	0,07441324	folate transporter 1
PFIT_0402800	200,1543527	183,2352273	254,8289438	335,0665334	191,69479	294,94774	1,538632003	0,6216482	0,0122162	0,07441324	transcription factor with AP2 domain(s), putative
PFIT_1477100	138,3419791	174,8519162	110,6494098	69,5547295	156,5969477	90,10207	0,575375644	-0,797424	0,0122976	0,074738951	conserved Plasmodium protein, unknown function
PFIT_1316100	2756,53752	2803,618739	3556,707923	3953,250058	2780,078129	3754,979	1,350673908	0,4336794	0,0122846	0,074738951	conserved Plasmodium protein, unknown function
PFIT_1318900	148,6440414	153,2948307	76,28103253	107,0072761	150,969436	91,644154	0,6070378	-0,720142	0,0123536	0,074917835	ser/thr protein phosphatase 2A regulatory subunit A, putative
PFIT_0619500	100,0771764	81,43787879	134,9587499	157,8357323	90,75752758	146,39724	1,613058938	0,6897992	0,0123416	0,074917835	conserved Plasmodium protein, unknown function
PFIT_0822000	426,7997228	482,6391934	655,5139279	641,3748614	454,7194581	648,44439	1,426031772	0,5120061	0,0123852	0,075028645	BRIX domain, putative
PFIT_1428600.1	114,7944082	89,82118984	145,8560402	177,2308011	102,307799	161,54342	1,578994194	0,6590059	0,0124028	0,075054791	conserved Plasmodium membrane protein, unknown function
PFIT_1035500	448,8755705	367,6680704	305,9623832	243,4415532	408,2718205	274,70197	0,672840873	-0,571663	0,0125721	0,075997819	merozoite surface protein (MSP3.8)
PFIT_0215000	557,783086	487,4296569	395,6554654	335,7353289	522,6063714	365,6954	0,69975304	-0,515082	0,0126547	0,076415207	conserved Plasmodium protein, unknown function
PFIT_0207400.2	469,479695	403,5965464	264,049728	329,0473742	436,5381207	296,54855	0,679318797	-0,557839	0,0127218	0,076493125	conserved Plasmodium protein, unknown function
PFIT_1142000	841,8256601	713,7790553	604,3804885	504,2717888	777,8023577	554,32614	0,712682513	-0,488669	0,0127217	0,076493125	conserved Plasmodium protein, unknown function
PFIT_0524500	665,2188782	719,7671346	572,5268705	430,7042865	692,4930064	501,61558	0,724361941	-0,465217	0,012704	0,076493125	S-adenosylmethionine-dependent methyltransferase,, putative
PFIT_0504200	475,3665878	518,5676694	766,1633377	648,7316116	496,9671286	707,44747	1,423529715	0,5094726	0,0127032	0,076493125	ATP-dependent helicase, putative
PFIT_1032900	1190,624054	1235,939572	1673,99145	1655,268803	1213,281813	1664,6301	1,372006164	0,456287	0,0127922	0,076834596	WD-repeat protein, putative
PFIT_0510900	72,11443592	85,03072639	155,9150775	115,7016173	78,5725815	135,80835	1,72844421	0,7894743	0,0128265	0,076959198	conserved Plasmodium protein, unknown function
PFIT_1109500	1565,913466	1693,428833	857,5329261	1267,367427	1629,671149	1062,4502	0,651941453	-0,617186	0,0128739	0,077079417	heat shock protein DnaJ homologue Pfj2
PFIT_0935200	2780,085091	2730,564171	2223,047234	1865,270582	2755,324631	2044,1589	0,741894035	-0,430715	0,012864	0,077079417	UBX domain, putative
PFIT_1233300	9092,305818	10234,82518	7018,693246	7437,674488	9663,565498	7228,1839	0,747983119	-0,418922	0,0129222	0,077286667	asparagine and aspartate rich protein 1
PFIT_0808000	2131,055167	2445,531595	3152,669927	3102,542213	2288,293381	3127,6061	1,366785437	0,4507868	0,0129639	0,077453097	conserved Plasmodium protein, unknown function
PFIT_1122500	2114,866212	2503,017157	3162,728964	3155,377055	2308,941685	3159,053	1,368182242	0,4522604	0,0130422	0,077839828	protein GCN20
PFIT_1036700	3680,779678	3188,053432	2534,877389	2499,288694	3434,416555	2517,083	0,732899752	-0,448312	0,0131457	0,078291939	dynamin-like protein
PF_apicoplast_g	26,49101728	17,96423797	46,94217387	50,15966069	22,22762762	48,550917	2,184259972	1,1271446	0,0131364	0,078291939	apicoplast ribosomal protein S8
PFIT_1108700	1267,15366	1123,363681	1776,258329	1488,738729	1195,25867	1632,4985	1,365811911	0,4497588	0,0132226	0,078587579	mechanosensitive ion channel protein
PFIT_1412500	1036,09312	1108,992291	1544,900472	1415,840023	1072,542705	1480,3702	1,38024364	0,464923	0,0132232	0,078587579	conserved Plasmodium protein, unknown function
PFIT_0322700	60,34065046	68,26410428	97,23736015	138,4406635	64,30237737	117,83901	1,832576285	0,8738733	0,0133241	0,079103681	conserved Plasmodium protein, unknown function
PFIT_1472800	42,67997228	63,47364082	23,47108693	28,08940999	53,07680655	25,780248	0,485715892	-1,041815	0,0134067	0,079224123	conserved Plasmodium protein, unknown function
PFIT_1144300	67,69926637	53,89271391	36,0448835	24,74543261	60,79599014	30,395158	0,499953336	-1,000135	0,0134245	0,079224123	conserved Plasmodium protein, unknown function
PFIT_0727300.1	163,3612732	211,978008	99,75211946	130,4151178	187,6696406	115,08362	0,613224485	-0,705513	0,0133993	0,079224123	conserved Plasmodium protein, unknown function
PFIT_0600200	822,6932587	1022,763948	682,3380273	479,5263562	922,7286035	580,93219	0,629580778	-0,667537	0,0133912	0,079224123	Plasmodium repeat_MYXSPDY protein
PFIT_1222100	1177,378546	976,0569296	818,1350302	634,6869066	1076,717738	726,41097	0,674653108	-0,567782	0,0134308	0,079224123	transcription factor with AP2 domain(s)
PFIT_1209800	145,700595	154,4924465	260,6967156	204,6514156	150,0965208	232,67407	1,550162951	0,6324199	0,0134426	0,079224123	zinc finger protein, putative
PFIT_0602800	42,67997228	14,37139038	59,51597044	68,88593402	28,52568133	64,200952	2,250636943	1,1703334	0,0133846	0,079224123	conserved Plasmodium protein, unknown function
PFIT_0722100	1336,324649	1544,924465	759,4573129	1096,155785	1440,624557	927,80655	0,644030774	-0,634798	0,0134797	0,079277576	conserved Plasmodium protein, unknown function
PFIT_1465100	2148,715846	2415,591199	1197,863687	1755,588124	2282,153522	1476,7259	0,64707562	-0,627994	0,0134728	0,079277576	YL1 nuclear protein, putative
PFIT_0616800	59360,48282	51868,7431	39755,83	20189,59783	55614,61296	29972,714	0,538935944	-0,891814	0,0135582	0,079656098	histone H3 variant, putative
PFIT_1319100	154,5309341	166,4686052	113,1641691	90,95618472	160,4997696	102,06018	0,635889866	-0,653151	0,0135797	0,079695368	conserved Plasmodium protein, unknown function
PFIT_0521200	1240,662642	1380,851092	1084,699518	860,7397775	1310,756867	972,71965	0,742105322	-0,430304	0,0135931	0,079695368	conserved Plasmodium protein, unknown function
PFIT_1030800.1	1814,634683	1762,890553	1322,763399	1300,807201	1788,762618	1311,7853	0,733347895	-0,44743	0,0136291	0,07982363	OTU-like cysteine protease, putative
PFIT_1211300	3096,505575	2652,71914	2061,264385	2139,476727	2874,612357	2100,3706	0,730662188	-0,452724	0,0137018	0,07989791	DNA replication licensing factor MCM5, putative (MCM5)
PFIT_1201600	409,1390446	395,2132353	520,555178	609,2726786	402,1761399	564,91393	1,404643071	0,4902036	0,0136907	0,07989791	conserved protein, unknown function
PFIT_1322200	188,3805673	256,289795	355,4193164	314,3338737	222,3351812	334,8766	1,506179064	0,5908933	0,0136864	0,07989791	beta-hydroxyacyl-ACP dehydratase
PFIT_0531600	497,4424355	520,9629011	686,5292928	919,5937794	509,2026683	803,06154	1,577096088	0,6572706	0,0136585	0,07989791	conserved Plasmodium protein, unknown function
PFIT_1221800	113,322685	105,3901961	171,8418865	173,8868237	109,3564405	172,86436	1,580742334	0,6606022	0,0137126	0,07989791	conserved Plasmodium protein, unknown function
PFIT_0618300	6659,547398	7148,569096	4723,556245	5540,970518	6904,058247	5132,2634	0,743369073	-0,427849	0,0137786	0,080193322	cell division cycle protein 48 homologue, putative
PFIT_0909100	718,2009128	615,5745544	932,9757056	900,1987106	666,8877336	916,58721	1,374425052	0,4588282	0,0137917	0,080193322	conserved Plasmodium protein, unknown function
PFIT_0423000	2534,307319	2894,637545	4136,779072	3426,908019	2714,472432	3781,8435	1,39321494	0,4784178	0,013828	0,080321913	Plasmodium exported protein (PHISTb), unknown function
PFIT_1003100	2241,434406	2417,986431	3097,345222	3214,231057	2329,710418	3155,7881	1,354583864	0,4378497	0,0138564	0,080404427	40S ribosomal protein S20e, putative
PFIT_1015600	132,4550864	129,3425134	202,8572513	202,6450292	130,8987999	202,75114	1,548915196	0,6312582	0,0139436	0,080826973	conserved Plasmodium protein, unknown function
PFIT_0810600	1353,985327	1352,108311	1033,566078	951,0271668	1353,046819	992,29662	0,733379369	-0,447368	0,0139641	0,080862969	serine protease, putative
PFIT_1028700	157,4743805	102,9949644	206,2102638	191,9443016	130,2346724	199,07728	1,528604319	0,612215	0,0140543	0,081301985	conserved Plasmodium protein, unknown function
PFIT_0420700	5474,810237	6765,332019	4359,754398	4634,752648	6120,071128	4497,2535	0,734836806	-0,444504	0,0140763	0,081345528	pre-mRNA-splicing helicase BRR2, putative
PFIT_1008600	16,188955	31,13801248	52,8099456	52,8348426	23,66348374	52,822394	2,232232358	1,1584872	0,0141185	0,081506	conserved Plasmodium membrane protein, unknown function
PFIT_1243600	1218,586795	1089,830437	1685,726994	1457,305342	1154,208616	1571,5162	1,361552969	0,4452531	0,0141994	0,081875337	conserved Plasmodium protein, unknown function
PFIT_0931200	39,73652591	28,74278075	57,00121112	74,23629783	34,23965333	65,618754	1,91645499	0,9384401	0,0142114	0,081875337	NifU-like protein, putative
PFIT_0403100	1084,659985	1077,854278	902,7985938	656,0883619	1081,257132	779,44348	0,720867826	-0,472193	0,0142516	0,08202269	6-cysteine protein
PFIT_0614400	5708,814223	6352,154546	4012,717612	4963,131227	6030,484384	4487,9244	0,744206292	-0,426226	0,01431	0,08227509	ribonuclease, putative
PFIT_1252300	43476,17452	36323,68917	30895,49468	28376,32325	39899,93185	29635,909	0,742755879	-0,42904	0,0143286	0,08229829	rhoptry neck protein 3 (RON3)
PFIT_1321400	3359,944024	3000,027741	1947,261962	2542,760399	3179,985883	2245,0112	0,705981493	-0,502298	0,0143673	0,082353145	conserved Plasmodium protein, unknown function
PFIT_1109000	194,26746	219,1637032	273,2705121	353,7928068	206,7155816	313,53166	1,516729687	0,600964	0,0143589	0,082353145	D-tyrosyl-tRNA(Tyr) deacylase
PFIT_0534300	1842,597424	1817,980882	2539,068654	2414,351668	1830,289153	2476,7102	1,353179719	0,4363535	0,0144253	0,082517897	SNARE protein, putative
PFIT_0711900	235,4757091	204,7923128	282,4912963	359,1431706	220,134011	320,81723	1,457372407	0,5433696	0,0144137	0,082517897	conserved Plasmodium protein, unknown function
PFIT_1448600	278,1556814	344,913369	219,6223134	211,3393704	311,5345252	215,48084	0,691675639	-0,531832	0,014528	0,082974004	conserved Plasmodium protein, unknown function
PFIT_0206000	2705,027208	2788,049733	3800,639577	3616,177138	2746,538471	3708,4084	1,350211693	0,4331856	0,0145344	0,082974004	adenylosuccinate lyase
PFIT_1010500	128,0399168	97,00688503	159,2680899	210,6705749	112,5234009	184,96933	1,643829913	0,717061	0,0146783	0,083710733	inner membrane complex protein, putative
PFIT_0825800	18705,60164	16715,12462	13531,08162	12861,6058	17710,36313	13196,344	0,745119883	-0,424456	0,0147165	0,083843995	conserved Plasmodium protein, unknown function
PFIT_0935700	58,86892728	86,22834225	124,8997126	183,2499604	72,54863476	154,07484	2,123745499	1,0866109	0,0147454	0,083923694	conserved protein, unknown function
PFIT_0830200	2618,195541	2881,46377	2155,148732	1367,686748	2749,829655	1761,4177	0,64055522	-0,642605	0,0147612	0,083929478	conserved Plasmodium protein, unknown function
PFIT_0712400	2503,401132	2558,107487	2145,089695	1653,931212	2530,75431	1899,5105	0,750570866	-0,41394	0,0148413	0,084299853	zinc finger protein, putative
PFIT_1469400	60,34065046	89,82118984	136,6352561	120,3831857	75,08092015	128,50922	1,711609562	0,7753536	0,0149373	0,084759858	leucine-rich repeat protein
PFIT_1411100	42,67997228	37,1260918	75,44277943	74,23629783	39,90303204	74,839539	1,875535136	0,9073023	0,0149748	0,084887457	alpha/beta hydrolase, putative
PFIT_0209500	1427,571486	1360,491622	884,3570255	1132,939536	1394,031554	1008,6483	0,723547668	-0,46684	0,0150099	0,085000677	conserved Plasmodium protein, unknown function

PFIT_1213900	958,0917914	972,464082	673,1172431	726,3118868	965,2779367	699,71456	0,724884034	-0,464178	0,01509	0,085368909	geranylgeranyltransferase type2 beta subunit, putative
PFIT_0302700	878,6187396	918,5713681	1482,869742	1160,360151	898,5950539	1321,6149	1,470756979	0,5565589	0,0151101	0,085396965	conserved Plasmodium protein, unknown function
PFIT_0828400	1729,274739	1773,669095	2395,727373	2342,790552	1751,471917	2369,259	1,352724494	0,435868	0,0151889	0,085756592	conserved protein, unknown function
PFIT_1245400	204,5695223	184,4328431	274,1087652	382,5510122	194,5011827	328,32989	1,688061143	0,7553672	0,0152809	0,086189911	conserved Plasmodium membrane protein, unknown function
PFIT_0532200	119,2095777	162,8757576	203,6955045	232,7408256	141,0426677	218,21817	1,547178373	0,6296395	0,0153435	0,08645644	conserved Plasmodium protein, unknown function
PFIT_1102000	30,90618682	29,94039661	62,86898286	59,52279736	30,42329172	61,19589	2,011481554	1,0082585	0,0154331	0,08687474	Pfmc-2TM Maurer's cleft two transmembrane protein
PFIT_0304500	60,34065046	77,8450312	33,53012419	40,12772856	69,09284083	36,828926	0,533035347	-0,907697	0,0156125	0,087683454	circumsporozoite (CS) protein
PFIT_0603000	6906,796893	6227,602496	3985,05526	5277,4651	6567,199694	4631,2602	0,705210805	-0,503874	0,0156557	0,087683454	SPE2-interacting protein
PFIT_0827900	1919,127029	1720,973997	1271,62996	1388,419408	1820,050513	1330,0247	0,730762511	-0,452525	0,0155961	0,087683454	conserved Plasmodium protein, unknown function
PFIT_1410400	4574,115649	4350,938436	3359,718444	3291,142537	4462,527043	3325,4305	0,745189992	-0,42432	0,01567	0,087683454	conserved Plasmodium membrane protein, unknown function
PFIT_0607900	14611,26775	15103,13367	11047,33767	11294,618	14857,20071	11170,978	0,751889811	-0,411407	0,0156426	0,087683454	conserved Plasmodium protein, unknown function
PFIT_1306600	55,92548091	53,89271391	108,9729036	85,60582092	54,90909741	97,289362	1,771825924	0,8252369	0,0156525	0,087683454	conserved Plasmodium protein, unknown function
PFIT_0616200	9725,146786	10228,8371	7533,380652	7492,515717	9976,991943	7512,9482	0,753027388	-0,409226	0,0157358	0,087877871	conserved Plasmodium protein, unknown function
PFIT_1434400	86,83166773	85,03072639	124,0614595	176,5620056	85,93119706	150,31173	1,749210272	0,8067037	0,0157353	0,087877871	conserved Plasmodium protein, unknown function
PFIT_0721100	145,700595	134,1329768	76,28103253	94,3001621	139,9167859	85,290597	0,609580879	-0,71411	0,0159215	0,088826752	conserved Plasmodium protein, unknown function
PFIT_1216000	481,2534805	582,0413102	720,8976701	767,7772064	531,6473953	744,33744	1,400058469	0,4854871	0,0159674	0,088995234	signal recognition particle SRP19
PFIT_0221700	8,830339092	3,592847594	18,4415683	24,07663713	6,211593343	21,259103	3,422487846	1,7750454	0,0160045	0,089113636	RESA-like protein, pseudogene
PFIT_0910200	194,26746	152,0972148	235,5491224	271,5309632	173,1823374	253,54004	1,464006357	0,5499218	0,0160593	0,089330572	SET domain protein, putative
PFIT_0106100	500,3858819	389,225156	595,1597044	627,3301564	444,8055189	611,24493	1,374184682	0,4585759	0,0160805	0,089360648	serine/threonine protein kinase, putative
PFIT_0207400.1	450,3472937	400,0036988	256,50545	328,3785787	425,1754962	292,44201	0,687814836	-0,539908	0,0161785	0,089728544	conserved Plasmodium protein, unknown function
PFIT_1407200	8871,547341	9685,119497	6836,792322	7154,774001	9278,333419	6995,7832	0,753991352	-0,40738	0,01617	0,089728544	conserved Plasmodium protein, unknown function
PFIT_0520500	86,83166773	112,5758913	150,8855589	169,2052554	99,7037795	160,04541	1,605209029	0,6827612	0,0162538	0,090057734	U6 snRNA-associated sm-like protein lsm2, putative
PFIT_0423100	4984,726417	5549,751916	4078,101355	2468,524102	5267,239167	3273,3127	0,621447522	-0,686296	0,0162791	0,090081975	Plasmodium exported protein (PHISTa), unknown function
PFIT_0725000	3454,134308	3500,631172	4901,265903	4432,776414	3477,38274	4667,0212	1,342107415	0,4245001	0,0162901	0,090081975	Plasmodium exported protein, unknown function
PFIT_0501300	1277,455722	1588,038636	1098,111567	878,1284599	1432,747179	988,12001	0,689668092	-0,536026	0,0164168	0,090530747	parasite-infected erythrocyte surface protein (PIESP2)
PFIT_1140600	596,0478887	617,9697861	482,8337883	396,5957172	607,0088374	439,71475	0,724395965	-0,46515	0,0164217	0,090530747	conserved protein, unknown function
PFIT_0413200	1171,491653	1050,309113	917,0488966	716,2799547	1110,900383	816,66443	0,735137406	-0,443914	0,0163985	0,090530747	conserved protein, unknown function
PFIT_1413600	788,8436255	962,8831551	670,6024838	624,6549745	875,8633903	647,62873	0,739417512	-0,435539	0,0164354	0,090530747	deoxyhypusine synthase (DHS)
PFIT_1024500	7857,530068	7931,809871	6100,806096	5792,437617	7894,66997	5946,6219	0,753245149	-0,408809	0,0164563	0,090558007	formin 2, putative
PFIT_0827500	600,4630582	609,5864751	497,0840911	381,2134213	605,0247667	439,14876	0,725835999	-0,462284	0,0165797	0,090982373	anamorsin related protein, putative
PFIT_0628700	1976,524233	2002,413726	2928,018095	2452,47301	1989,468979	2690,2456	1,352243026	0,4353545	0,0165818	0,090982373	cullin-like protein, putative
PFIT_0808400	1015,488996	1034,740107	1408,265216	1379,725067	1025,114551	1393,9951	1,359843287	0,4434404	0,0165802	0,090982373	conserved Plasmodium protein, unknown function
PFIT_0400600	85,35994455	128,1448975	51,11343939	58,85400188	106,752421	54,993721	0,515151976	-0,95693	0,0166057	0,09102483	acyl-CoA synthetase
PFIT_0916000	376,7611346	390,4227719	294,2268398	246,7855306	383,5919532	270,50619	0,705192544	-0,503911	0,0168566	0,092240351	conserved Plasmodium protein, unknown function
PFIT_0418600	6628,641211	7323,421012	5063,887006	5458,039879	6976,031112	5260,9634	0,754148506	-0,407079	0,0168601	0,092240351	zinc finger protein, putative
PFIT_0405900	999,3000405	1007,194942	1497,958298	1244,628381	1003,247491	1371,2933	1,366854491	0,4508597	0,0169813	0,092813509	transcription factor, putative
PFIT_1336000	1189,152331	1395,222482	1031,889572	904,2114834	1292,187407	968,05053	0,749156448	-0,416661	0,0170423	0,092963064	nuclear movement protein, putative
PFIT_1331200	3061,184218	3826,382687	4385,740244	4886,888543	3443,783453	4636,3144	1,346285112	0,428984	0,017058	0,092963064	60S ribosomal protein L23, putative
PFIT_0909400	57,3972041	53,89271391	106,4581443	88,28100282	55,644959	97,369574	1,749836379	0,80722	0,0170535	0,092963064	GDP-fucose protein O-fucosyltransferase 2, putative (POFUT2)
PFIT_0833600	413,5542141	520,9629011	761,1338191	625,9925655	467,2585576	693,56319	1,484324216	0,5698062	0,0171341	0,093287745	Plasmodium exported protein (hyp9), unknown function
PFIT_0907200	317,8922073	306,5896613	497,9223442	395,2581263	312,2409343	446,59024	1,430274465	0,516292	0,0171833	0,093465058	conserved Plasmodium protein, unknown function
PFIT_1226600	2228,188897	1664,686052	1300,968819	989,1485089	1946,437475	1145,0587	0,588284329	-0,765414	0,0172546	0,093691566	conserved Plasmodium protein, unknown function
PFIT_0826200	3988,369823	4439,56201	3095,668716	3257,033968	4213,965917	3176,3513	0,753767687	-0,407808	0,0172581	0,093691566	histone acetyltransferase GCN5
PFIT_1361500	1147,944082	1355,701159	1657,226388	1747,562579	1251,82262	1702,3945	1,359932674	0,4435352	0,0173315	0,093909322	exonuclease, putative
[illegible]	[illegible]	[illegible]	[illegible]	[illegible]	[illegible]	[illegible]	[illegible]	[illegible]	[illegible]	[illegible]	[illegible]

PFIT_0904400	41,20824909	57,4855615	24,30934004	30,76459189	49,3469053	27,536966	0,558028225	-0,84159	0,0495551	0,18688485	conserved Plasmodium protein, unknown function
PFIT_1354000	231,0605396	237,1279412	158,4298368	177,2308011	234,0942404	167,83032	0,716934849	-0,480086	0,0498266	0,187672455	metacaspase 1
PFIT_1140300	200,1543527	174,8519162	127,4144719	133,7590952	187,5031345	130,58678	0,696451203	-0,521906	0,0501586	0,188796857	conserved Plasmodium protein, unknown function
PFIT_0723200	1086,131708	976,0569296	802,2082212	785,8346842	1031,094319	794,02145	0,77007645	-0,376926	0,0502377	0,188968645	exonuclease I, putative
PFIT_1437200	1623,31067	2304,212923	1309,35135	1312,176724	1963,761797	1310,764	0,667476085	-0,583212	0,0503975	0,189355518	glucose-6-phosphate isomerase
PFIT_0109400	119,2095777	142,5162879	179,3861644	195,9570744	130,8629328	187,67162	1,43410831	0,520154	0,0504076	0,189355518	adenylate kinase-like protein 1 (AKLP1)
PFIT_0928100.2	50,03858819	68,26430428	89,69308221	98,98173044	59,15134623	94,337406	1,594848001	0,6734189	0,0505904	0,189789679	cytochrome c oxidase subunit 6B, putative (COX6B)
PFIT_1103400	32,37791	28,74278075	52,8099456	55,5100245	30,56034538	54,159985	1,772230791	0,8255665	0,0505604	0,189789679	Plasmodium exported protein, unknown function
PFIT_0627100	11514,76218	12529,45718	8551,019921	10469,99318	12022,10968	9510,5065	0,79108466	-0,338096	0,0509253	0,190646029	protein kinase PK4
PFIT_1342100	2821,29334	3110,2084	2306,872544	2390,943826	2965,75087	2348,9082	0,792011294	-0,336407	0,0509222	0,190646029	transcription factor with AP2 domain(s)
PFIT_1341300	3540,965976	3585,661899	4686,673109	4363,221685	3563,313937	4524,9474	1,269870541	0,3446814	0,0508623	0,190646029	aconitate hydratase
PFIT_1318000	7280,614581	7825,222059	10404,39754	8855,520897	7552,91832	9629,9592	1,274998459	0,3504955	0,0509537	0,190646029	secretory complex protein 63
PFIT_1408100	1003,71521	1585,643405	836,5765985	549,0810857	1294,679307	692,82884	0,535135487	-0,902024	0,0512715	0,190669565	fibrillarin, putative
PFIT_1324100	43212,73607	54053,19443	39521,11913	27516,25227	48632,96525	33518,686	0,689217397	-0,536969	0,0512701	0,190669565	L-lactate dehydrogenase
PFIT_1147600	863,9015078	725,7552139	582,5859078	623,986179	794,8283609	603,28604	0,759014239	-0,397801	0,0511616	0,190669565	sporozoite asparagine-rich protein
PFIT_1020400	3973,652591	4050,336854	3557,546177	2577,537764	4011,994723	3067,542	0,764592723	-0,387237	0,0512977	0,190669565	ADP-ribosylation factor
PFIT_1446000	29243,13963	30742,79924	26625,43337	19563,60526	29992,96943	23094,519	0,769997761	-0,377074	0,0510323	0,190669565	fructose-bisphosphate aldolase
PFIT_0818700	5027,40639	4299,440954	3310,261511	3929,842216	4663,423672	3620,0519	0,776264847	-0,365379	0,0512304	0,190669565	vacuolar sorting protein VPS9, putative
PFIT_1145800	4300,375138	4482,676181	3466,176588	3493,787566	4391,525659	3479,9821	0,792431229	-0,335642	0,0511664	0,190669565	coatamer gamma subunit, putative
PFIT_0802500	344,3832246	332,9372104	461,0392076	439,3986277	338,6602175	450,21892	1,329411884	0,4107882	0,0511642	0,190669565	Plasmodium exported protein, unknown function
PFIT_0509500	94,19028364	107,7854278	150,8855589	147,1350047	100,9878557	149,01028	1,475526742	0,5612301	0,0512105	0,190669565	conserved Plasmodium protein, unknown function
PF_apicoplast_g	17,66067818	13,17377451	25,14759314	38,7901376	15,41722635	31,968865	2,073580854	1,0521243	0,051051	0,190669565	hypothetical protein
PFIT_1341700	97,13373001	80,24026293	169,3271272	118,3767992	88,68699647	143,85196	1,62201866	0,6977904	0,051457	0,191135691	sporozoite protein essential for cell traversal
PFIT_0215400	370,8742419	380,8418449	285,8443087	276,881327	375,8580434	281,36282	0,748587992	-0,417756	0,0514966	0,19115698	DEAD/DEAH box helicase, putative
PFIT_0422700	7411,597944	8497,084559	10883,04006	9448,073688	7954,341252	10165,557	1,277988529	0,3538749	0,0517809	0,192086195	serine/threonine protein kinase, FIKK family
PFIT_1004400	94,19028364	106,5878119	153,4003182	143,1222318	100,3890478	148,26128	1,476867031	0,5625399	0,0518363	0,192165427	ADP/ATP carrier protein, putative
PFIT_1115100	78,00132864	67,06648842	95,56085394	121,7207766	72,53390853	108,64082	1,49779348	0,5828387	0,0519618	0,192504075	unspecified product
PFIT_0611200	965,4504074	865,8762701	393,9789592	715,6111592	915,6633387	554,79506	0,605894149	-0,722862	0,0521565	0,193098656	leucine-rich repeat protein
PFIT_0726500	92,71856046	83,83311052	122,3849533	137,1030726	88,27583549	129,74401	1,469756839	0,5555775	0,0522515	0,193323679	conserved Plasmodium protein, unknown function
PFIT_0825400	17,66067818	29,94039661	41,91265524	72,2299114	23,8005374	57,071283	2,397898937	1,2617709	0,052328	0,193480032	dynein light chain 1
PFIT_0502200	104,4923459	82,63549466	168,4888741	122,3895721	93,56392029	145,43922	1,554437038	0,6363922	0,0525304	0,194044619	HCNGP-like protein
PFIT_0905900.3	13,24550864	22,75470143	33,53012419	40,79652403	18,00010503	37,163324	2,06461707	1,0458742	0,0525495	0,194044619	autophagy-related protein 3, putative (ATG3)
PFIT_0732200	41,20824909	55,09032977	72,08976701	85,60582092	48,14928943	78,847794	1,637569212	0,7115559	0,0526943	0,19445225	Plasmodium exported protein, unknown function
PFIT_1319500	160,4178268	168,8638369	209,5632762	246,7855306	164,6408319	228,1744	1,385891949	0,4708148	0,0527734	0,194617096	conserved Plasmodium protein, unknown function
PFIT_1226800	588,6892728	713,7790553	500,4371035	510,2909481	651,234164	505,36403	0,776009696	-0,365853	0,0528229	0,194672568	DNA helicase 60
PFIT_1334300	60846,92323	49497,46368	44219,52778	30265,00167	55172,19346	37242,265	0,675018744	-0,567001	0,0530939	0,195543652	merozoite surface protein 7
PFIT_0220000	382,6480273	405,9917781	556,6000615	490,8958793	394,3199027	523,74797	1,328231131	0,4095062	0,0533666	0,19641962	Plasmodium exported protein (hyp9), unknown function
PFIT_1301600	30,90618682	26,34754902	57,83946423	45,47809236	28,62686792	51,658778	1,804555721	0,8516437	0,0534045	0,196431032	conserved Plasmodium membrane protein, unknown function
PFIT_0113300	1,471723182	5,988079323	13,41204968	15,38229595	3,729901252	14,397173	3,85993404	1,9485762	0,0534772	0,196570454	Plasmodium exported protein (hyp10), unknown function
PFIT_1340800	130,9833632	132,935361	164,2976085	206,6578021	131,9593621	185,47771	1,405566853	0,4911521	0,0535935	0,196741833	dolichyl-phosphate-mannose-glygolipidalpha-mannosyltransferase, putative
PFIT_0410800	89,7751141	85,03072639	109,8111567	156,4981414	87,40292024	133,15465	1,523457668	0,6073494	0,0535647	0,196741833	conserved Plasmodium protein, unknown function
PFIT_1308600	1783,728497	1597,619563	1134,994704	1453,292569	1690,67403	1294,1436	0,765460174	-0,385601	0,0538558	0,197576329	HORMA domain protein, putative
PFIT_1457200	941,9028364	735,3361409	545,7027712	642,0436569	838,6194887	593,87321	0,708155751	-0,497861	0,0542529	0,198903763	transcription factor with AP2 domain(s)
PFIT_1368500	2076,60141	2404,812656	1444,310099	1902,723129	2240,707033	1673,5166	0,74686989	-0,421071	0,0546028	0,19967628	conserved Plasmodium protein, unknown function
PFIT_1141100	3620,439028	3150,92734	2759,529221	2568,174628	3385,683184	2663,8519	0,786798935	-0,345933	0,0545667	0,19967628	conserved Plasmodium protein, unknown function
PFIT_1424800	1782,256773	1863,490285	2466,140634	2190,305184	1822,873529	2328,2229	1,277226791	0,3530147	0,0545168	0,19967628	conserved Plasmodium protein, unknown function
PFIT_1016900	286,9860205	410,7822416	478,6425228	485,5455155	348,884131	482,09402	1,381816988	0,4665666	0,0546051	0,19967628	phosphomannomutase, putative
PFIT_1435600	2990,541506	2504,214773	2190,355363	1243,29079	2747,378139	1716,8231	0,624895078	-0,678314	0,0546446	0,199691648	conserved Plasmodium membrane protein, unknown function
PFIT_0323700	320,8356537	371,260918	461,0392076	462,1376739	346,0482858	461,58844	1,333884488	0,4156337	0,054914	0,200546218	conserved Plasmodium protein, unknown function
PFIT_1433100	213,3998614	229,942246	286,6825618	315,0026692	221,6710537	300,84262	1,357157872	0,4405886	0,0549839	0,200671728	conserved Plasmodium membrane protein, unknown function
Pf_M76611014(	38,26480273	41,91655526	20,11807451	22,07025071	40,090679	21,094163	0,526161271	-0,926423	0,0551232	0,200865781	unspecified product
PFIT_1343300	1794,030559	1827,561809	1445,148353	1411,82725	1810,796184	1428,4878	0,78887277	-0,342135	0,0551428	0,200865781	krox-like protein. putative
PFIT_1222900	1028,734504	1032,344875	1448,501365	1204,500652	1030,53969	1326,501	1,287190607	0,3642257	0,0551438	0,200865781	nucleus export protein BRR6, putative
PFIT_0320100	598,9913351	532,9390597	467,7452324	397,2645127	565,9651974	432,50487	0,764189873	-0,387997	0,0551959	0,200870737	SNARE protein
PFIT_0409500	1248,021258	1382,048708	1073,802227	1005,868396	1315,034983	1039,8353	0,790728251	-0,338746	0,0552163	0,200870737	conserved Plasmodium protein, unknown function
PFIT_1229500	14631,87187	12864,78962	10613,96081	11069,23392	13748,33075	10841,597	0,788575542	-0,342679	0,0552833	0,200959749	myosin D
PFIT_1362000	806,5043037	847,9120321	1080,508252	1051,346488	827,2081679	1065,9274	1,288584194	0,3657868	0,055312	0,200959749	conserved Plasmodium protein, unknown function
PFIT_1343800	192,7957368	161,6781417	108,1346505	136,4342771	177,2369393	122,28446	0,689949083	-0,535438	0,0553502	0,200969235	lipoyl synthase (LipA)
PFIT_0416100	423,8562764	323,3562834	279,1182839	252,1358944	373,6062799	265,62709	0,711008094	-0,492062	0,055448	0,201086147	memo-like protein
PFIT_0816900	13330,86858	14803,7297	12611,51796	10177,72955	14067,29914	11394,624	0,810007923	-0,303992	0,0554892	0,201086147	60S ribosomal protein L13-2, putative
PFIT_0511700	139,8137023	159,28291	185,2539361	232,0720301	149,5483061	208,66298	1,395288175	0,4805631	0,0554711	0,201086147	conserved Plasmodium protein, unknown function
PFIT_0810100	528,3486223	780,8455437	444,2741455	384,5573987	654,597083	414,41577	0,633085272	-0,659528	0,0555765	0,201273347	conserved Plasmodium membrane protein, unknown function
PFIT_1471500	116,2661314	118,5639706	153,4003182	180,5747785	117,415051	166,98755	1,422198832	0,5081232	0,0556386	0,2013698	mitochondrial pyruvate carrier protein 2, putative (MPC2)
PFIT_1357300	1823,465022	1966,48525	2565,892754	2276,5798	1894,975136	2421,2363	1,277714008	0,353565	0,055739	0,201603023	conserved Plasmodium protein, unknown function
PFIT_1101100	470,9514182	520,9629011	680,661521	622,6485881	495,9571597	651,65505	1,313934161	0,393893	0,0560415	0,202567492	Plasmodium exported protein (hyp5), unknown function
PFIT_0819700	11,77378546	27,54536489	5,029518628	5,350363807	19,65957517	5,1899412	0,26399185	-1,921415	0,0561797	0,202807449	meiotic recombination protein DMC1, putative (DMC1)
PFIT_1424600	136,8702559	143,7139038	103,1051319	91,6249802	140,2920798	97,365056	0,69401677	-0,526958	0,0561756	0,202807449	serine/threonine protein kinase, putative
PFIT_1211700	139,8137023	155,6900624	201,1807451	213,3457568	147,7518823	207,26325	1,402779089	0,4882878	0,0562748	0,203020884	peripheral plastid protein 1, putative (PPP1)
PFIT_1320800	135,3985327	150,8995989	207,0485169	197,2946654	143,1490658	202,17159	1,412315127	0,498062	0,0565953	0,203916406	phosphodiesterase gamma, putative
PFIT_0515200	69,17098955	81,43787879	95,56085394	132,4215042	75,30443417	113,99118	1,51373794	0,5981155	0,0565638	0,203916406	rhomboid protease ROM9
PFIT_1418400	5246,691144	4856,332331	4081,454367	3925,160648	5051,512737	4003,3075	0,792496766	-0,335523	0,0566461	0,203969265	cyclic nucleotide-binding protein, putative
PFIT_0905400	1524,705216	1550,912545	1981,63034	1925,462175	1537,808881	1953,5463	1,270343982	0,3452192	0,0567861	0,204212869	mitochondrial carrier protein, putative
PFIT_0520600	64,75582001	59,88079323	78,79579185	113,6952309	62,31830662	96,245511	1,54441795	0,6270632	0,0567661	0,204212869	conserved Plasmodium protein, unknown function
PFIT_1143800	17928,5318	16331,88755	14401,18834	12907,08389	17130,20967	13654,136	0,797079334	-0,327205	0,0569441	0,204650769	transcription factor with AP2 domain(s)
PFIT_0414900	573,972041	518,5676694	350,3897978	467,4880377	546,2698552	408,93892	0,748602387	-0,417728	0,0572207	0,205514113	DNA helicase MCM9, putative (MCM9)
PFIT_1215700	295,8163596	299,4039661	370,5078723	417,328377	297,6101629	393,91812	1,323604412	0,404472	0,05734	0,20568078	serine--tRNA ligase, putative
PFIT_1212800	88,30339092	105,3901961	130,7674843	152,4853685	96,8467935	141,62643	1,462375999	0,5483143	0,0573079	0,20568078	conserved Plasmodium protein, unknown function
PFIT_1206600	2091,318642	1821,57373	958,9615518	1561,637436	1956,446186	1260,2995	0,64417795	-0,634469	0,0576043	0,206287045	conserved Plasmodium protein, unknown function
PFIT_0605900	7847,228006	8918,645344	6521,609155	6932,065108	8382,936675	6726,8371	0,802443987	-0,317527	0,0576186	0,206287045	translation initiation factor IF-2, putative
PFIT_0830100	317,8922073	314,9729724	404,8762496	430,7042865	316,4325898	417,79027	1,320313651	0,4008807	0,0576115	0,206287045	conserved Plasmodium protein, unknown function
PFIT_0313500	63,28409682	61,07840909	25,14759314	46,81568331	62,18125296	35,981638	0,578657337	-0,789219	0,0577337	0,206466991	conserved Plasmodium protein, unknown function
PFIT_1239900	176,6067818	173,6543004	225,4900852	253,4734854	175,1305411	239,48179	1,367447298	0,4514852	0,057742	0,206466991	erythrocyte membrane protein 1 (PfEMP1), pseudogene
PFIT_0816300	16102,12333	17002,55243	14620,81065	10293,43117	16552,33788	12457,121	0,75258915	-0,410064	0,0577822	0,206479869	conserved Plasmodium protein, unknown function
PFIT_0306900	8649,31714	10704,2906	7883,77045	7652,357835	9676,803869	7768,0641	0,802751017	-0,316976	0,0579775	0,207046658	40S ribosomal protein S23, putative
PFIT_1366600	500,3858819	562,8794564	470,2599918	358,4743751	531,6326691	414,36718	0,779425853	-0,359751	0,0582173	0,207771742	ribosome biogenesis protein MRT4, putative
PFIT_1253300	853,5994455	936,5356061	1220,49652	1089,46783	895,0675258	1154,9822	1,290385521	0,3678022	0,0582808	0,207866958	Plasmodium exported protein (PHISTa), unknown function
PFIT_1103900	17046,96962	19522,33621	16165,71112	12835,52277	18284,65291	14500,617	0,793048521	-0,334519	0,0583828	0,207967864	60S acidic ribosomal protein P1, putative
PFIT_bin04600	58,86892728	69,46172015	90,53133531	107,0072761	64,16532371	98,769306	1,539294124	0,6222689	0,0583463	0,207967864	rifin (RIF)
PFIT_0934200	192,7957368	124,5520499	99,75211946	68,21713854	158,6738934	83,984629	0,529290781	-0,917868	0,0584483	0,208070014	conserved Plasmodium protein, unknown function
PFIT_0412600	119,2095777	148,5043672	168,4888741	209,332984	133,8569725	188,91093	1,41128942	0,4970139	0,0585619	0,208343019	conserved Plasmodium membrane protein, unknown function
PFIT_1145400	86,83166773	93,41403744	132,4437056	138,4406635	90,12285259	135,44218	1,50285385	0,5534489	0,05936	0,210947024	apicoplast import protein Tic20, putative
PFIT_0203700	29,43446364	32,33562834	46,10392076	78,24907068	30,88504599	62,176496	2,013158593	1,0094608	0,0593685	0,210947024	dynein light chain, putative
PFIT_1454600	2206,11305	2279,06299	1880,201714	1697,402918	2242,58802	1788,8023	0,79760884	-0,326171	0,0594362	0,210956156	conserved Plasmodium protein, unknown function
PFIT_1336200	86,83166773	108,9830437	127,4144719	157,8357323	97,90735571	142,6251	1,45673531	0,5427388	0,0594458	0,210956156	endopeptidase, putative
PFIT_1238600	3246,621339	3246,736609	4164,441424	4002,072128	3246,678974	4083,2568	1,25761858	0,3307556	0,0594934	0,210992135	protein kinase 2
PFIT_1127200	1702,783722	1847,921279	2211,31169	2287,280528	1775,3525	2249,2961	1,26695748	0,3413681	0,0595518	0,211066754	DnaJ protein, putative
PFIT_1425500	0	2,395231729	8,382531047	9,363136663	1,197615865	8,8728339	7,408747761	2,8892297	0,0596164	0,211163078	small nucleolar RNA snoR27
PFIT_0215600	203,0977991	192,8161542	259,0202094	276,2125316	197,9569767	267,61637	1,351891583	0,4349795	0,0596681	0,21121994	conserved Plasmodium protein, unknown function
PFIT_1132200	550,42447	542,5199867	713,3533921	698,2224769	546,4722284	705,78793	1,291534863	0,3690866	0,0601738	0,21287051	conserved Plasmodium protein, unknown function
PFIT_0303400	863,9015078	814,3787879	589,2919326	702,9040452	839,1401479	646,09799	0,769952422	-0,377159	0,0602179	0,212893058	spindle pole body protein, putative
PFIT_1429600	139,8137023	143,7139038	89,69308221	105,6696852	141,763803	97,681384	0,689043195	-0,537334	0,0603026	0,212925907	probable protein, unknown function
PFIT_1359300	660,8037087	823,9597148	912,8576311	1006,537191	742,3817118	959,69741	1,292727711	0,3704184	0,0603013	0,212925907	conserved protein, unknown function
PFIT_0408600	8506,559992	8289,897015	6638,126336	6761,522262	8398,228503	6699,8243	0,797766374	-0,325962	0,0606273	0,213938645	conserved Plasmodium protein, unknown function
PFIT_0929500	129,51164	114,9711123	168,4888741	174,5556162	122,2413815	171,52225	1,403143882	0,4886663	0,0607032	0,21407259	conserved Plasmodium protein, unknown function
PFIT_1244000	1052,282075	1085,039973	1373,058586	1348,291679	1068,661024	1360,6751	1,272252324	0,3485184	0,0607496	0,214102527	microtubule associated katanin, putative
PFIT_0534100	1994,184912	2725,773708	3198,773848	3081,140758	2359,97931	3139,9573	1,330502047	0,4119707	0,0608931	0,214474531	RING zinc finger protein
PFIT_0834400	4329,809601	4613,21631	6954,98601	5336,319102	4471,512956	6145,6526	1,374401152	0,4588032	0,0610597	0,214793276	Plasmodium exported protein (PHIST), unknown function
PFIT_1118400.2	16,188955	8,383311052	24,30934004	28,75820546	12,28613303	26,533773	2,15965208	1,1107989	0,0610227	0,214793276	conserved Plasmodium protein, unknown function
PFIT_0818900	1789,615389	2185,648953	2655,585836	2429,065169	1987,632171	2542,3255	1,279072426	0,355098	0,0611975	0,21514391	nucleolar preribosomal assembly protein, putative
PFIT_1321900	20,60412455	27,54536489	5,867771733	14,71350047	24,07464472	10,290636	0,42744706	-1,226382	0,0612973	0,215360554	conserved Plasmodium protein, unknown function
PFIT_0820500	7925,229335	6464,730437	5893,757579	5320,936806	7194,979886	5607,3472	0,779341608	-0,359672	0,0614146	0,215638557	conserved Plasmodium protein, unknown function
PFIT_0517800	4937,631275	5913,827139	7260,948393	6517,411913	5425,729207	6889,1802	1,269724288	0,3445153	0,0617804	0,21678808	eukaryotic translation initiation factor, putative
PFIT_0522800	666,6906014	591,6222371	419,9648055	535,0363807	629,1564193	477,50059	0,75895701	-0,397916	0,0619447	0,217043745	conserved protein, unknown function, putative
PFIT_0519700	1677,764427	1726,962077	1383,955876	1321,53986	1702,363252	1352,7479	0,794629387	-0,331646	0,0619686	0,217043745	carbon catabolite repressor protein 4, putative
PFIT_1436300	95,66200683	75,44979947	119,0319409	128,4087314	85,55590315	123,72034	1,446075976	0,5321434	0,0619555	0,217043745	ribonuclease H2 subunit B, putative
PFIT_1343100	649,0299232	692,2196697	794,6639433	920,9313703	670,6259465	857,79766	1,279100013	0,3551291	0,0620981	0,217362629	U4/U6 small nuclear ribonucleoprotein PRP4, putative
PFIT_1358500	4710,985905	5265,916957	4053,792015	3986,021036	4988,451431	4019,9065	0,805842571	-0,31143	0,0622613	0,21779889	rRNA associated RNA binding protein, putative
PFIT_1015700	737,3333142	835,8166667	1070,449215	1039,30817	816,5749904	1054,8787	1,291833211	0,3694198	0,0623406	0,217941337	conserved Plasmodium protein, unknown function
[illegible]	[illegible]	[illegible]	[illegible]	[illegible]	[illegible]	[illegible]	[illegible]	[illegible]	[illegible]	[illegible]	[illegible]

PFIT_bin05700	543,0658541	548,508066	664,7347121	688,1905447	545,7869601	676,46263	1,239426146	0,3096723	0,11476	0,324397603 cytoadherence linked asexual protein 2,cytoadherence linked asexual protein 8 (CLAG2)
PFIT_0834950	1,471723182	1,197635865	5,867771733	8,694341187	1,334669523	7,2810565	5,455325332	2,4476652	0,1149908	0,324781484 Plasmodium exported protein, unknown function, pseudogene
PFIT_0611800	685,8230028	805,9954769	1009,256738	857,3958001	745,9092398	933,32627	1,251259831	0,3233814	0,1150748	0,324856131 6-cysteine protein
PFIT_0537100	26,49101728	28,74278075	44,42741455	46,81568331	27,61689901	45,621549	1,651943215	0,7241641	0,1155174	0,325942603 rifin, pseudogene
PFIT_0602500	239,8908787	249,1040998	174,3566458	201,3074383	244,4974892	187,83204	0,7682371	-0,380376	0,1156528	0,32612766 conserved Plasmodium protein, unknown function
PFIT_bin04900	2,943446364	10,77854278	17,6033152	17,38868237	6,860994573	17,495999	2,550067428	1,3505354	0,1156985	0,32612766 rifin (RIF)
PFIT_0802100	235,4757091	256,289795	296,7415991	332,3913515	245,8827521	314,56648	1,279335263	0,3553944	0,1159416	0,326649857 Plasmodium exported protein, unknown function
PFIT_1233100	138,3419791	119,7615865	88,8548291	96,97534401	129,0517828	92,915087	0,719982975	-0,473965	0,1161036	0,326943157 pentatricopeptide repeat protein, putative
PFIT_0314000.1	1162,661314	1171,268316	1515,561613	1346,954088	1166,964815	1431,2579	1,22647901	0,2945225	0,1166249	0,328247462 vesicle transport v-SNARE protein, putative
PFIT_1145300	412,0824909	497,0105838	567,4973519	577,1704957	454,5465374	572,33392	1,259131633	0,3324291	0,1168371	0,328680613 conserved Plasmodium protein, unknown function
PFIT_0213900	47,09514182	38,32370767	73,76627322	56,17881998	42,70942474	64,972547	1,521269532	0,6052758	0,1174025	0,330106837 conserved Plasmodium protein, unknown function
PFIT_1463900	522,4617296	449,1059492	377,2138971	390,5765579	485,7838394	383,89523	0,790259363	-0,339602	0,1175689	0,330410109 cytochrome c1 precursor, putative
PFIT_0213600	169,2481659	192,8161542	233,0343631	240,7663713	181,0321601	236,90037	1,308609294	0,3880344	0,1177912	0,330870188 mitochondrial ribosomal protein L13 precursor, putative
PFIT_1364300	634,3126914	740,1266043	890,2247972	823,2872309	687,2196479	856,75601	1,246698951	0,3181131	0,1179268	0,331078543 conserved Plasmodium protein, unknown function
PFIT_1338700	33,84963318	8,383311052	36,88313661	45,47809236	21,11647212	41,180614	1,950165456	0,9635965	0,1179826	0,331078543 mitochondrial ribosomal protein L49 precursor, putative
PFIT_1422500	48,566865	46,70701872	57,00121112	82,93063901	47,63694186	69,965925	1,468732507	0,5545717	0,1182475	0,331657051 mitochondrial import inner membrane translocase subunit TIM8, putative (TIM8)
PFIT_1011500	3797,045809	3391,648129	3237,33349	2209,031457	3594,346969	2723,1825	0,757629271	-0,400436	0,1185365	0,332302397 ubiquitin-protein ligase e3, putative
PFIT_0721500	6627,169488	5482,685428	5300,274381	4196,022816	6054,927458	4748,1486	0,784179271	-0,350745	0,1190087	0,333239514 conserved Plasmodium protein, unknown function
PFIT_1402200	217,8150309	269,4635695	200,342492	183,2499604	243,6393002	191,79623	0,787213828	-0,345173	0,1189947	0,333239514 aminopeptidase, putative
PFIT_0533500	158,9461037	117,3663547	172,6801396	185,9251423	138,1562292	179,30264	1,297825237	0,3760961	0,1190478	0,333239514 apicoplast TIC22 protein (TIC22)
PFIT_0531700	5317,335856	6264,728588	4601,171292	5076,826458	5791,032222	4838,9989	0,835602133	-0,259112	0,1194283	0,334138866 Hsc70-interacting protein (HIP)
PFIT_0418300	872,7318469	638,3292558	988,3004105	902,205097	755,5305514	945,25275	1,251111225	0,3232101	0,11952	0,334229826 apical merozoite protein
PFIT_1134800	450,3472937	407,189394	483,6720414	571,1513364	428,7683438	527,41169	1,230062099	0,2987312	0,1197319	0,33465666 protein disulfide isomerase
PFIT_0936400	22685,14113	19201,37516	18325,88938	16317,94082	20943,25814	17321,915	0,827087886	-0,273887	0,1200125	0,335274971 cytoadherence linked asexual protein 9
PFIT_0509400	11190,98308	13655,21609	10500,79664	10451,9357	12423,09958	10476,366	0,843297287	-0,245887	0,1206984	0,337024352 RNA polymerase I
PFIT_0217500	92,71856046	99,40211676	112,325916	166,5300735	96,06033861	139,42799	1,451462662	0,5375075	0,1208799	0,337364197 conserved Plasmodium membrane protein, unknown function
PFIT_1148000	39,73652591	35,92847594	48,61868007	66,21075212	37,83250093	57,414716	1,517602979	0,6017944	0,1218115	0,339796224 conserved Plasmodium protein, unknown function
PFIT_1028400	2551,967997	1912,592536	3023,578949	2608,302356	2232,280267	2815,9407	1,261463757	0,3350988	0,1220255	0,340225229 conserved Plasmodium protein, unknown function, pseudogene
PFIT_0823000	70,64271273	49,10225045	90,53133531	79,58666163	59,87248159	85,058998	1,420669333	0,5065708	0,1221329	0,340356587 conserved Plasmodium protein, unknown function
PFIT_1247800	55,92548091	56,28794564	72,92802011	88,9497983	56,10671327	80,938909	1,442588676	0,52866	0,1222165	0,340421531 conserved Plasmodium protein, unknown function
PFIT_1405100	312,0053146	377,2489973	308,4771425	221,3713025	344,627156	264,92422	0,768727066	-0,379457	0,1223462	0,340446716 cytochrome c, putative
PFIT_1364600	523,9334528	481,4415776	558,2765678	670,8018623	502,6875152	614,53922	1,222507416	0,2898432	0,1222962	0,340446716 DNA topoisomerase VI, b subunit, putative
PFIT_0529400	2351,813645	2196,427496	1805,597188	1927,468562	2274,12057	1866,5329	0,82077129	-0,284948	0,1227319	0,341015957 conserved Plasmodium protein, unknown function
PFIT_0721300	197,2109064	174,8519162	232,19611	244,7791442	186,0314113	238,48763	1,281975046	0,3583682	0,1226253	0,341015957 conserved Plasmodium protein, unknown function
PFIT_1352700	72,11443592	98,2045009	109,8111567	176,5620056	85,15946841	143,18658	1,681393553	0,7496574	0,1226734	0,341015957 transcription initiation factor TFIIH, putative
PFIT_1317500	805,0325805	767,6717692	646,2931438	630,6741338	786,3521749	638,48364	0,811956346	-0,300526	0,1228043	0,341049263 conserved Plasmodium protein, unknown function
PFIT_0302800	4709,514182	5134,179211	6382,459139	5585,779815	4921,846697	5984,1195	1,215828092	0,2819393	0,1230373	0,341528316 exportin 1, putative
PFIT_0922400	44,15169546	55,09032977	79,63404495	69,5547295	49,62101261	74,594387	1,503282245	0,5881159	0,123371	0,342286243 conserved Plasmodium protein, unknown function
PFIT_1106600	3533,60736	3438,355147	3192,906076	2629,035016	3485,981254	2910,9705	0,835050545	-0,260065	0,1235581	0,342636984 conserved Plasmodium protein, unknown function
PFIT_0410300	16,188955	22,75470143	31,01536488	38,12134213	19,47182821	34,568354	1,775300867	0,8280635	0,1237223	0,342924097 conserved Plasmodium protein, unknown function
PFIT_1416300	757,9374387	691,0243539	595,1597044	576,5017002	724,4808963	585,8307	0,808621325	-0,306464	0,1238647	0,343122717 RNA-binding protein Nova-1, putative
PFIT_0531300	924,2421583	1119,770833	1177,745612	1329,565406	1022,006496	1253,6555	1,226660999	0,2947366	0,1239156	0,343122717 ubiquitin-conjugating enzyme E2 N, putative
PFIT_1301700	2493,09907	2712,599933	2244,841814	2103,361772	2602,849502	2174,1018	0,835277565	-0,259672	0,1240272	0,343263569 conserved Plasmodium protein, unknown function
PFIT_0110000	1855,842932	1590,433868	1342,043221	1461,98691	1723,1384	1402,0151	0,813640428	-0,297537	0,1241181	0,343343853 UMP-CMP kinase, putative
PFIT_1224400	2063,355901	1944,928164	2349,623453	2465,84892	2004,142033	2407,7362	1,201380015	0,2646926	0,1241779	0,343343853 conserved Plasmodium protein, unknown function
PFIT_1108900	9193,854718	7911,450401	7594,573129	5016,634865	8552,65256	6305,604	0,737268813	-0,439737	0,1242704	0,343431682 conserved Plasmodium protein, unknown function
PFIT_1212200.2	1,471723182	13,17377451	15,92680899	23,40784166	7,322748846	19,667325	2,685784497	1,4253435	0,1244936	0,343880052 conserved protein, unknown function
PFIT_0614000	363,5156259	397,608467	330,2717233	282,9004863	380,5620465	306,5861	0,80561398	-0,311839	0,1250635	0,345285225 conserved Plasmodium protein, unknown function
PFIT_1223600	103,0206227	86,22834225	70,4132608	62,86677474	94,62448249	66,640018	0,704257672	-0,505825	0,1253472	0,345617402 50S ribosomal protein L24, putative,mitochondrial ribosomal protein L24-2 precursor, putative
PFIT_1357000	422,3845532	580,8436943	417,4500462	304,970737	501,6141238	361,21039	0,720096135	-0,473739	0,1253097	0,345617402 pyrroline carboxylate reductase
PFIT_1128900.2	2397,437063	2516,190931	2911,253033	3022,286756	2456,813997	2966,7699	1,207567971	0,2721044	0,1253687	0,345617402 conserved Plasmodium protein, unknown function
PFIT_0929700	13,24550864	22,75470143	29,33885867	63,53557021	18,00010503	46,437214	2,579830193	1,3672761	0,1254286	0,345617402 conserved protein, unknown function
PFIT_1129000	1286,286061	1376,060628	1253,188392	966,4094627	1331,173345	1109,7989	0,833699782	-0,2624	0,1256117	0,345952903 cochaperone prefoldin complex subunit, putative
PFIT_0732400	95,66200683	73,05456774	67,89850148	49,49086522	84,35828728	58,694683	0,695778509	-0,5233	0,1258839	0,346533674 Plasmodium exported protein (PHISTb), unknown function
PFIT_1470900	425,3279996	394,0156194	354,5810633	300,9579642	409,6718095	327,76951	0,800078273	-0,321787	0,1260758	0,346751999 conserved Plasmodium protein, unknown function
PFIT_1230600	3907,425048	4126,984269	4970,840911	4738,415947	4017,204659	4854,6284	1,208459325	0,2731689	0,1260861	0,346751999 atypical protein kinase, RIO family, putative
PFIT_0803200	14,71723182	14,37139038	7,544277943	4,681568331	14,5443111	6,1129231	0,420296506	-1,250521	0,1264088	0,347470371 Plasmodium exported protein (PHISTa), unknown function
PFIT_1025200	82,41649819	57,4855615	84,66356358	123,0583676	69,95102984	103,86097	1,484766783	0,5702363	0,126622	0,347887144 conserved Plasmodium protein, unknown function
PFIT_1236500	272,2687887	205,9899287	281,6530432	411,9780132	239,1293587	346,81553	1,450326008	0,5363772	0,1267329	0,348022418 protein-S-isoprenylcysteine O-methyltransferase, putative
PFIT_1434300	716,7291896	811,9835562	668,9259776	593,8903826	764,3563729	631,40818	0,826065174	-0,275672	0,1270078	0,348607643 conserved Plasmodium membrane protein, unknown function
PFIT_1200800	78,00132864	89,82118984	96,39910704	133,7590952	83,91125924	115,0791	1,371438138	0,4556895	0,127075	0,348622653 RESA-like protein with PHIST and DnaJ domains
PFIT_1431900	67,69926637	77,8450312	50,29518628	50,15966069	72,77214878	50,227423	0,690201187	-0,534911	0,1274135	0,349042525 NADP-specific glutamate dehydrogenase (GDH2)
PFIT_1014600	588,6892728	574,855615	715,8681514	712,9359773	581,7724439	714,40206	1,227975082	0,2962813	0,127386	0,349042525 conserved protein, unknown function
PFIT_0716900	39,73652591	39,52132353	47,78042697	76,91147973	39,62892472	62,345953	1,573243629	0,6537421	0,1273763	0,349042525 conserved Plasmodium membrane protein, unknown function
PFIT_1125500	3957,463636	3827,580303	4473,75682	4830,709723	3892,52197	4652,2333	1,195172001	0,2572183	0,127476	0,349044262 ethanolamine kinase (EK)
PFIT_0308300	186,9088441	274,254033	162,6211023	168,5364599	230,5814385	165,57878	0,718092411	-0,477759	0,1278968	0,349976083 U4 Spliceosomal RNA
PFIT_0615800	257,5515568	243,1160205	181,0626706	205,3202111	250,3337887	193,19144	0,771735377	-0,373822	0,1280056	0,349976083 malate:quinone oxidoreductase, putative
PFIT_0930800	721,1443592	752,102763	805,5612337	985,8045315	736,6235611	895,68288	1,215930266	0,2820605	0,1280642	0,349976083 peptidyl-prolyl cis-trans isomerase
PFIT_1018100	2884,577437	3432,367068	3575,149492	4611,344806	3158,472252	4093,2471	1,295957926	0,3740189	0,1279661	0,349976083 conserved Plasmodium protein, unknown function
PFIT_1408500	211,9281382	228,7446301	274,9470184	288,9196456	220,3363842	281,93333	1,279558676	0,3556463	0,128266	0,350188601 tRNA m2G10 methyltransferase subunit, putative
PFIT_1461000	394,4218128	462,2797237	507,9813815	765,7708199	428,3507682	636,8761	1,486809755	0,5722201	0,1282153	0,350188601 bax inhibitor 1, putative
PFIT_1227000	441,5169546	407,189394	523,9081905	522,9980622	424,3531743	523,45313	1,23353178	0,3027949	0,1294263	0,353185446 conserved Plasmodium protein, unknown function
PFIT_1115200	47,09514182	44,31178699	53,6481987	79,58666163	45,70346441	66,61743	1,457601323	0,5435962	0,1307028	0,35649651 conserved Plasmodium protein, unknown function
PFIT_0301700	2886,04916	2876,673307	3892,009165	3128,625236	2881,361233	3510,3172	1,218284317	0,2848509	0,1308163	0,356633996 Plasmodium exported protein, unknown function
PFIT_1347700	244,3060482	244,3136364	274,9470184	339,0793063	244,3098423	307,01316	1,256654908	0,3295885	0,1308838	0,356645651 TBC domain protein, putative
PFIT_1125400	139,8137023	118,5639706	93,04609463	95,63775306	129,1888364	94,341924	0,730263748	-0,45351	0,130968	0,356703128 pyruvate dehydrogenase E1 alpha subunit
PFIT_0112000	133,9268096	220,3613191	105,6198912	119,0455947	177,1440643	112,33274	0,634132131	-0,657145	0,1313905	0,357681166 surface-associated interspersed protein 1.2 (SURFIN 1.2) (SURF1.2)
PFIT_1408000	238,4191555	190,4209225	276,6235246	264,174213	214,420039	270,39887	1,261070887	0,3346494	0,1316362	0,358086729 LCCL domain-containing protein
PFIT_1309300	100,0771764	65,86887255	108,1346505	114,3640264	82,97302446	111,24934	1,34078924	0,4230825	0,1316663	0,358086729 conserved Plasmodium protein, unknown function
PFIT_0702500	1465,836289	1095,818516	987,4621574	950,3583713	1280,827403	968,91026	0,756472154	-0,402641	0,1318056	0,358168772 conserved Plasmodium protein, unknown function
PFIT_0407700	3193,639305	3373,683891	3101,536488	2366,867189	3283,661598	2734,2018	0,83266858	-0,264186	0,1318234	0,358168772 U4/U6 small nuclear ribonucleoprotein PRP31, putative
PFIT_1324000	431,2148923	495,8129679	388,1111875	367,1687163	463,5139301	377,63995	0,814732692	-0,295601	0,1324721	0,359412397 dihydrofolate synthase/folylpolyglutamate synthase
PFIT_0303000	1707,198891	1535,343538	1198,70194	1445,935819	1621,271215	1322,3189	0,815606215	-0,294055	0,132437	0,359412397 conserved Plasmodium protein, unknown function
PFIT_0532400	3689,610017	4154,529434	4739,483054	4733,734379	3922,069726	4736,6087	1,207680905	0,2722393	0,1323599	0,359412397 eukaryotic translation initiation factor 3, subunit 6, putative
PFIT_0726600	183,9653977	184,4328431	136,6352561	144,4598228	184,1991204	140,54754	0,763019601	-0,390208	0,1326605	0,359750753 conserved Plasmodium protein, unknown function
PFIT_0407300	61,81237364	49,10225045	39,39789592	32,77097832	55,45731204	36,084437	0,650670503	-0,620001	0,1331613	0,359824118 perforin like protein 1,sporozoite micronemal protein essential for cell traversal (SPECT2)
PFIT_0911100	319,3639305	255,0921792	212,9162886	231,4032347	287,2280548	222,15976	0,77346122	-0,370599	0,1332449	0,359824118 DNA primase large subunit, putative
PFIT_0722300	410,6107678	500,6034314	324,4039515	402,6148765	455,6070996	363,50941	0,79785722	-0,325798	0,1329791	0,359824118 conserved Plasmodium protein, unknown function
PFIT_0534800	534,235515	577,2508467	487,0250539	424,0163317	555,7431809	455,52069	0,819660427	-0,286902	0,1330095	0,359824118 XAP-5 DNA binding protein, putative
PFIT_1130500	91,24683728	80,24026293	107,2963974	124,3959585	85,7435501	115,84618	1,351077461	0,4341104	0,1332611	0,359824118 conserved Plasmodium protein, unknown function
PFIT_0112200	91,24683728	116,1687389	138,3117623	143,7910273	103,7077881	141,05139	1,360084883	0,4436967	0,1332125	0,359824118 DBL containing protein, unknown function
PFIT_1113600	86,83166773	85,03072639	115,6789285	119,0455947	85,93119706	117,36226	1,365770123	0,4497147	0,1331026	0,359824118 conserved Plasmodium protein, unknown function
PFIT_0731800	29,43446364	21,55708556	38,55964282	43,47170593	25,4957746	41,015674	1,608724387	0,6859172	0,1332266	0,359824118 probable protein, unknown function
PFIT_bin06300	133,9268096	131,7377451	171,8418865	326,3721922	132,8322773	249,10704	1,875350211	0,90716	0,1327935	0,359824118 unspecified product
PFIT_1453200	18699,71475	17165,42819	14141,32988	15602,99845	17932,57147	14872,164	0,829338067	-0,269968	0,1334824	0,360077098 rhoptry neck protein 2
PFIT_0400800	14478,81266	15711,52253	21905,23013	16888,42336	15095,1676	19396,827	1,28496928	0,3617339	0,133419	0,360077098 Plasmodium exported protein (PHISTb), unknown function
PFIT_1237700	141,2854255	159,28291	120,7084471	108,3448671	150,2841677	114,52666	0,762067348	-0,39201	0,1336134	0,360258403 COPI associated protein, putative
PFIT_1461800	407,6673214	408,3870098	509,6578877	501,5966069	408,0271656	505,62725	1,23919996	0,309409	0,1338182	0,360638476 inner membrane complex protein, putative
PFIT_0101900	3298,131651	3301,826939	2427,580991	3024,961938	3299,979295	2726,2715	0,826148052	-0,275528	0,1341533	0,361369127 zinc-carboxypeptidase, putative
PFIT_1144900	160,4178268	201,1994653	157,5915837	78,9178616	180,808646	118,25472	0,654032468	-0,612566	0,1344319	0,361516549 conserved Plasmodium protein, unknown function
PFIT_0913100	1799,917452	1924,568694	1725,12489	1142,971468	1862,243073	1434,0482	0,770064982	-0,376948	0,1345744	0,361516549 conserved Plasmodium protein, unknown function
PFIT_1229300	809,4477501	748,5099154	577,5563892	682,1713854	778,9788327	629,86389	0,808576383	-0,306544	0,1346563	0,361516549 conserved Plasmodium protein, unknown function
PFIT_1141400	2053,053839	2347,327095	1830,744781	1857,913832	2200,190467	1844,3293	0,83825893	-0,254532	0,1343286	0,361516549 conserved Plasmodium protein, unknown function
PFIT_1417100	2407,739126	2500,621925	2940,591891	2963,432754	2454,180525	2952,0123	1,202850521	0,2664574	0,134602	0,361516549 root hair defective 3 GTP-binding protein (RHD3) homolog, putative
PFIT_1008500	560,7265323	534,1366756	720,8976701	625,9925655	547,431604	673,44512	1,230190425	0,2988817	0,1345725	0,361516549 diphthine synthase
PFIT_0319300	78,00132864	89,82118984	124,8997126	108,3448671	83,91125924	116,62229	1,389828861	0,4749072	0,134555	0,361516549 ubiquitin conjugating enzyme, putative
PFIT_1238500	5779,456935	6134,188458	6691,774535	7537,993809	5956,822697	7114,8842	1,194409257	0,2562973	0,1348242	0,361795229 acyl-CoA synthetase (ACS11)
PFIT_0725600	238,4191555	183,2352273	175,1948989	144,4598228	210,8271914	159,82736	0,758096524	-0,399547	0,1349602	0,361816275 conserved Plasmodium protein, unknown function
PFIT_0900150	57,3972041	68,26430428	107,2963974	75,75388878	62,83065419	91,435143	1,455263267	0,5412802	0,1349064	0,361816275 rifin (RIF)
PFIT_0503100	1511,459708	1413,18672	1290,909781	1133,608332	1462,323214	1212,2591	0,82899529	-0,270564	0,1353674	0,362735809 50S ribosomal protein L28, apicoplast, putative
PFIT_0908400	2688,838253	2192,834648	2083,897218	1937,500494	2440,836451	2010,6989	0,823774512	-0,279679	0,1355421	0,363031701 conserved Plasmodium protein, unknown function
PFIT_1423100	651,9733696	635,9340241	708,3238735	850,7078454	643,9536968	779,51586	1,210515388	0,2756214	0,135753	0,363424133 mitochondrial ribosomal protein L21 precursor, putative
PFIT_0828000	142,7571486	228,7446301	111,4876629	130,4151178	185,7508894	120,95139	0,651148378	-0,618942	0,135838	0,363479365 conserved Plasmodium protein, unknown function
PFIT_1428000	422,3845532	407,189394	451,8184235	562,4569952	414,7869736	507,13771	1,222646181	0,290007	0,1359429	0,363538005 conserved Plasmodium protein, unknown function
Pf_M76611007	17,66067818	22,75470143	30,17711177	39,45893308	20,20768981	34,818022	1,723008556	0,7849299	0,1359887	0,363538005 unspecified product
PFIT_1147800	971,3373001	974,8593138	797,1787026	804,5609575	973,0983069	800,86983	0,823010198	-0,281018	0,1361446	0,363621318 tubulin-tyrosine ligase, putative
PFIT_1144500	7184,952574	7843,186297	9972,697187	8210,133262	7514,069436	9091,4152	1,20991898	0,2749104	0,1361487	0,363621318 conserved Plasmodium protein, unknown function
PFIT_0520100	175,1350587	166,4686052	249,7994252	193,950688	170,8018319	221,87506	1,299020357	0,377424	0,1366818	0,364872543 conserved Plasmodium protein, unknown function
PFIT_1238100	16,188955	7,185695188	16,76506209	28,08940999	11,68732509	22,427236	1,918936614	0,9403071	0,1371087	0,365839241 conserved Plasmodium protein, unknown function
PFIT_1028500.1	79,47305183	101,7973485	114,0024222	133,7590952	90,63520016	123,88076	1,366806257	0,4508088	0,1377531	0,367211575 conserved Plasmodium protein, unknown function
PFIT_0517700	144,2288718	137,7258244	300,9328646	177,8995966	140,9773481	239,41623	1,698260279	0,7640576	0,1377063	0,367211575 F-actin capping protein beta subunit, putative
PFIT_0408000	1156,774421	1135,33984	1014,286257	888,160392	1146,05713	951,22332	0,829996428	-0,268823	0,1381153	0,367976807 heat shock protein 40
PFIT_1367300	210,456415	190,4209225	230,5196038	274,2061451	200,4386687	252,36287	1,259052837	0,3323388	0,1382116	0,367976807 conserved Plasmodium protein, unknown function
PFIT_1471400	366,4590723	340,1229055	523,0699374	393,9205353	353,2909889	458,49524	1,297783557	0,3760498	0,1382357	0,367976807 conserved Plasmodium protein, unknown function
PFIT_1317300	45,62341864	31,13801248	47,78042697	64,87316116	38,38071556	56,326794	1,467580613	0,5534398	0,1384787	0,368449834 ferredoxin, putative
PFIT_0909200	822,6932587	620,3650179	839,0913578	898,1923242	721,5291383	868,64184	1,203890176	0,2677038	0,1388289	0,369207796 conserved Plasmodium protein, unknown function
PFIT_1008800	382,6480273	414,3750891	326,0804577	318,3466465	398,5115582	322,21355	0,808542552	-0,306604	0,1389548	0,369368557 conserved Plasmodium protein, unknown function
PFIT_0507100	27427,03322	33508,09428	27323,6982	23911,44465	30467,56375	25617,571	0,840814567	-0,25014	0,1391505	0,369674454 60S ribosomal protein L4, putative
PFIT_0104100	1724,859569	2067,084982	1570,048065	1619,153847	1895,972276	1594,601	0,841046558	-0,249742	0,1392008	0,369674454 conserved Plasmodium protein, unknown function
PFIT_1030200	699,0685114	578,4484626	557,4383146	479,5263562	638,758487	518,48234	0,811703243	-0,300976	0,1395277	0,370368302 calmodulin, putative
PFIT_1125300	192,7957368	235,9303253	167,6506209	169,8740509	214,3630311	168,76234	0,78727351	-0,345063	0,1397741	0,370848056 U6 snRNA-associated Sm-like protein LSm1, putative
PFIT_1340500	0	0	1,676506209	6,687954759	0	4,1822305	#DIV/0!	#DIV/0!	0,1402175	0,371849652 small nucleolar RNA snoR20
PFIT_1473600	97,13373001	74,2521836	102,2668788	125,7335495	85,69295681	114,00021	1,330333535	0,411788	0,1403615	0,372056772 conserved Plasmodium protein, unknown function
PFIT_1209200	183,9653977	154,4924465	209,5632762	220,7025071	169,2289221	215,13289	1,271253689	0,346252	0,1405482	0,372376766 porphobilinogen deaminase
PFIT_1330900	328,1942696	360,4823752	282,4912963	272,1997587	344,3383224	277,34553	0,805444847	-0,312142	0,1408161	0,372911711 protein tyrosine phosphatase, putative
PFIT_1131800	45,62341864	44,31178699	67,06024838	65,54195664	44,96760281	66,301103	1,474419323	0,5601469	0,1410054	0,373237864 RNA-binding protein s1, putative
PFIT_1027300	92,71856046	129,3425134	182,7391768	137,771868	111,0305369	160,25552	1,44334637	0,5294176	0,1411255	0,373380729 DHHC-type zinc finger protein, putative
PFIT_1216400	827,1084283	925,7570633	817,2967771	594,5591781	876,4327458	705,92798	0,805455959	-0,312122	0,1412429	0,373464709 perforin like protein 2
PFIT_0926500	883,0339092	886,2357398	734,3097197	724,3055004	884,6348245	729,30761	0,824416573	-0,278555	0,1413557	0,373464709 monocarboxylate transporter, putative
PFIT_1100900	495,9707123	520,9629011	671,4407369	585,1960414	508,4668067	628,31839	1,235711714	0,3053422	0,141307	0,373464709 Plasmodium exported protein (hyp5), unknown function
PFIT_0219000	835,9387673	653,8982621	582,5859078	602,5847238	744,9185147	592,58532	0,79550354	-0,33006	0,1415465	0,37361924 replication factor C subunit 1, putative
PFIT_1322100	25,01929409	35,92847594	51,13343939	46,81568331	30,47388502	48,974561	1,607099368	0,6844591	0,1415026	0,37361924 conserved Plasmodium protein, unknown function
PFIT_1137800	752,050546	810,7859403	719,2211639	583,189655	781,4182431	651,20541	0,833363458	-0,262982	0,1417264	0,373918255 mitochondrial ribosomal protein S9 precursor, putative
Pf_M76611023	5,886892728	3,592847594	9,220784152	15,38229595	4,739870161	12,30154	2,595332706	1,3759195	0,1417922	0,373918255 unspecified product
PFIT_1228100	3080,31662	3361,707732	3840,875726	3891,720874	3221,012176	3866,2983	1,200336444	0,2634388	0,1421876	0,374726443 conserved Plasmodium protein, unknown function
PFIT_1146000	22,07584773	16,7666221	30,17711177	35,44616022	19,42123492	32,811636	1,689472175	0,7565726	0,1422314	0,374726443 conserved Plasmodium protein, unknown function
PFIT_0317600	5121,596673	6523,413614	6970,074566	7108,627114	5822,505144	7039,3508	1,208990059	0,2738024	0,1422995	0,374730831 40S ribosomal protein S11, putative
PFIT_1436100	38,26480273	38,32370767	20,11807451	26,08302356	38,2942552	23,100549	0,603237977	-0,729201	0,1424923	0,374970911 conserved Plasmodium protein, unknown function
PFIT_1127300	445,9321241	435,9321747	584,262414	504,9405843	440,9321494	544,6015	1,235114064	0,3046443	0,1425235	0,374970911 large ribosomal subunit processing factor, putative
PFIT_1240500	24800,00734	31922,45087	24803,90937	20399,59961	28361,2291	22601,754	0,796924365	-0,327485	0,1427972	0,375341265 erythrocyte membrane protein 1, PfEMP1
PFIT_0103900.1	105,9640691	89,82118984	108,9729036	149,8101866	97,89262947	129,39155	1,321770043	0,4024712	0,142787	0,375341265 conserved Plasmodium protein, unknown function
PFIT_0709200	264,9101728	341,3205214	259,0202094	233,4096211	303,1153471	246,21492	0,812281257	-0,299949	0,1428932	0,375418749 conserved Plasmodium membrane protein, unknown function
PFIT_1357900	3377,604703	4610,821079	5095,740624	4970,487977	3994,212891	5033,1143	1,260101662	0,3335401	0,1431057	0,375668982 40S ribosomal protein S13, putative
PFIT_0110600	104,4923459	100,5997326	116,5171816	153,8229595	102,5460393	135,17007	1,318140335	0,398504	0,1431215	0,375668982 conserved Plasmodium protein, unknown function
PFIT_0319600	3863,273353	4459,92148	3862,670307	2925,311412	4161,597416	3393,9909	0,815550021	-0,294155	0,1434219	0,376109232 elongation factor 1 (EF-1), putative
PFIT_1451000	329,6659928	409,5846257	322,7274453	282,9004863	369,6253092	302,81397	0,819245756	-0,287632	0,1434225	0,376109232 conserved Plasmodium protein, unknown function
PFIT_1320100	257,5515568	305,3920455	230,5196038	221,3713025	281,4718012	225,94545	0,802728558	-0,317016	0,1435544	0,376280385 RNA-binding protein, putative
PFIT_0410700	173,6633355	201,1994653	160,9445961	133,7590952	187,4314004	147,35185	0,78616414	-0,347098	0,1440188	0,376804047 mitochondrial ribosomal protein S12 precursor, putative
PFIT_1005300	220,7584773	191,6185383	228,0048445	286,2444637	206,1885078	257,12465	1,247036786	0,318504	0,1438854	0,376804047 DnaJ protein, putative
PFIT_1120600	370,8742419	449,1059492	538,1584932	487,5519019	409,9900955	512,8552	1,250896554	0,3229625	0,1440211	0,376804047 alternative splicing factor ASF-1, putative
PFIT_0615500	157,4743805	203,594697	249,7994252	221,3713025	180,5345387	235,58536	1,304932372	0,383975	0,1439961	0,376804047 TRAP-like protein
PFIT_1034600	164,113,3237	157,012,2303	133,692,9877	135,831,0236	160,562,777	134,762,01	0,839310381	-0,252724	0,144185	0,377058143 S-antigen
PFIT_1316200	1252,436428	1326,958378	1538,194447	1571,000573	1289,697403	1554,5975	1,205397101	0,2695085	0,1443372	0,377281283 U4/U6.U5 tri-snRNP-associated protein 2, putative
PFIT_0903400	189,8522905	194,0137701	218,7840603	333,060102	191,9330303	275,9221	1,437595724	0,523658	0,1451148	0,379138326 Rab GTPase 7
PFIT_0706700	495,9707123	453,8964127	509,6578877	656,0883619	474,9335625	582,87312	1,227272972	0,2954562	0,1459083	0,381035159 RAP protein, putative
PFIT_0402300	679,9361101	608,3888592	694,0735707	848,0326635	644,1624846	771,05312	1,196985443	0,2594056	0,146285	0,381842321 conserved Plasmodium protein, unknown function
PFIT_1301000	179,5502282	198,8042335	149,2090526	145,7974137	189,1772309	147,50323	0,779709231	-0,358992	0,1463672	0,381880492 surface-associated interspersed protein 13.1 (SURFIN 13.1)
PFIT_1035000	871,2601237	741,3242202	872,621482	1086,123853	806,2921719	979,37267	1,214662255	0,2805552	0,1466937	0,382202478 merozoite surface protein
PFIT_0404000	372,345965	346,1109849	425,8325772	455,4497191	359,2284749	440,64115	1,226632015	0,2947025	0,1466762	0,382202478 liver specific protein 2, putative (LISP2)
PFIT_1121000	291,40119	322,1586676	363,8018475	400,6084901	306,7799288	382,20517	1,245861065	0,3171432	0,1465666	0,382202478 conserved Plasmodium protein, unknown function
PFIT_0919500	860,9580614	786,833623	621,9838037	720,2922627	823,8958422	671,13827	0,814591155	-0,2958	0,1468549	0,382396483 protein disulfide isomerase
PFIT_0304700	509,216221	433,536943	585,9389202	559,1130179	471,376582	572,52597	1,21458297	0,280461	0,1469036	0,382396483 conserved Plasmodium membrane protein, unknown function
PFIT_0923800	27,96274046	31,13801248	13,41204968	20,06386428	29,55037647	16,737957	0,566421108	-0,820053	0,1473043	0,382909794 conserved protein, unknown function
PFIT_1471000	457,7059096	326,949131	306,8006363	256,1486673	392,3275203	281,47465	0,717448145	-0,479054	0,1472817	0,382909794 conserved Plasmodium protein, unknown function
PFIT_0628300	182,4936746	158,0852941	187,7686955	258,1550537	170,2894843	222,96187	1,30931088	0,3888077	0,1473017	0,382909794 RNA binding protein, putative
PFIT_1126300	1168,548206	1400,012946	1176,069106	870,7717096	1284,280576	1023,4204	0,79688226	-0,327562	0,1477347	0,38371206 mitochondrial inner membrane translocase subunit TIM44, putative
PFIT_0314200	859,4863383	747,3122995	661,3816896	652,075589	803,3993189	656,72864	0,817437392	-0,29082	0,1477489	0,38371206 ATP-dependent DNA helicase UvrD (UvrD)
PFIT_0912500	1168,548206	1227,556261	1437,604075	1448,611001	1198,052234	1443,1075	1,204544758	0,268488	0,1479167	0,383971346 conserved Plasmodium protein, unknown function
PFIT_1035700	5579,302583	5204,838547	4166,956184	4805,96429	5392,070565	4486,4602	0,83204776	-0,265262	0,1483163	0,38416138 probable protein, unknown function
PFIT_0903800.1	6028,178153	5194,060005	4869,412285	4505,006326	5611,119079	4687,2093	0,835343046	-0,259559	0,1480809	0,38416138 conserved Plasmodium protein, unknown function
PFIT_1029900	672,5774941	671,8625	730,9567073	886,8228011	672,2199971	808,88975	1,203311055	0,2670096	0,1483235	0,38416138 26S proteasome regulatory subunit, putative
PFIT_1201100	114,7944082	152,0972148	158,4298368	222,7088935	133,4458115	190,56937	1,428065542	0,5146622	0,1483301	0,38416138 Plasmodium exported protein, unknown function
PFIT_0834900	47,09514182	50,29866631	76,28103253	61,52918392	48,69750407	71,245892	1,46302965	0,548959	0,1482509	0,38416138 conserved Plasmodium protein, unknown function, pseudogene
PFIT_1027200	14291,30782	17480,41016	14395,32057	11867,77572	15886,15249	13131,548	0,82660343	-0,274733	0,1484609	0,384323804 60S ribosomal protein L3, putative
PFIT_0914500	2535,779042	1950,916243	1836,612552	1684,695804	2243,347643	1760,6542	0,784833409	-0,349542	0,148536	0,384341973 conserved Plasmodium protein, unknown function
PFIT_1250200	893,3359714	879,0500446	781,2518936	690,1969311	886,193008	735,72441	0,830207873	-0,268455	0,1486476	0,38445469 rRNA processing protein, putative
PFIT_0323000	16889,49524	18464,8414	16073,50328	8008,825824	17677,16832	12041,165	0,681170442	-0,553912	0,1491279	0,385013815 40S ribosomal protein S3A, putative
PFIT_0308700	288,4577437	240,7207888	218,7840603	197,2946654	264,5892662	208,03936	0,786272874	-0,346898	0,1492048	0,385013815 conserved protein, unknown function
PFIT_1449000	554,8396396	691,0243539	464,39222	548,4122903	622,9319967	506,40226	0,812933447	-0,298791	0,1490621	0,385013815 conserved Plasmodium protein, unknown function
PFIT_0906700	1520,290047	1699,416912	1984,145099	1902,054334	1609,853479	1943,0997	1,207004079	0,2714306	0,1491995	0,385013815 arginase
PFIT_1349400	314,9487609	304,1944296	391,4641999	376,5318529	309,5715953	383,99803	1,240417507	0,3108258	0,1491028	0,385013815 small nuclear ribonucleoprotein E, putative
PFIT_0903500	4391,621975	3491,050245	3082,256666	3285,123378	3941,33611	3183,69	0,807769227	-0,307985	0,1495028	0,385606516 conserved Plasmodium membrane protein, unknown function
PFIT_1007700	19341,38606	18369,03213	17340,10372	14591,77969	18855,20909	15965,942	0,846765561	-0,239966	0,1497036	0,385948098 conserved Plasmodium protein, unknown function
PFIT_0604300	1,471723182	3,592847594	6,706024838	11,36952309	2,532285388	9,037774	3,569018724	1,8355275	0,1500867	0,386759325 calcium-binding protein, putative
PFIT_1435300	178,078505	270,6611854	161,7828492	158,5045278	224,3698452	160,14369	0,713748714	-0,486512	0,1505064	0,386770571 queuine tRNA-ribosyltransferase, putative
PFIT_1410800	169,2481659	161,6781417	113,1641691	137,771868	165,4631538	125,46802	0,758283737	-0,399189	0,1504162	0,386770571 RNA binding protein Bruno, putative
PFIT_0609500	888,9208019	783,2407754	759,4573129	622,6485881	836,0807887	691,05295	0,826538487	-0,274846	0,1505205	0,386770571 calcium-dependent protein kinase 2 (CDPK2)
PFIT_1009300	1327,49431	1409,593873	1684,050487	1613,134688	1368,544091	1648,5926	1,204632425	0,268593	0,1502281	0,386770571 conserved Plasmodium membrane protein, unknown function
PFIT_1353300	69,17098955	57,4855615	82,14880426	92,29377568	63,32827553	87,22129	1,377288253	0,4618305	0,1505706	0,386770571 conserved Plasmodium protein, unknown function
PFIT_0202200	57,3972041	53,89271391	63,70723596	91,6249802	55,644959	77,666108	1,395743828	0,4810342	0,1502572	0,386770571 zinc finger protein, putative
PFIT_0728700	8,830339092	5,988079323	15,08855589	17,38868237	7,409209207	16,238619	2,191680472	1,1320375	0,1505111	0,386770571 dynein light chain, putative
PFIT_0816500	622,538906	586,831736	737,6627322	726,3118868	604,6853198	731,98731	1,210525973	0,275634	0,150664	0,386834396 translation initiation factor SUI1, putative
PFIT_1356500	880,0904628	734,138525	616,9542851	693,5409085	807,1144939	655,2476	0,81183971	-0,300733	0,1508467	0,387127517 conserved Plasmodium protein, unknown function
PFIT_0728800	1018,432442	929,3499109	821,4880426	786,5034797	973,8911764	803,99576	0,825549898	-0,276573	0,1510275	0,387415501 dynein heavy chain, putative
PFIT_1417700	17,66067818	11,97635865	6,706024838	6,019159283	14,81841841	6,3625921	0,429370523	-1,219705	0,1511757	0,387619397 conserved Plasmodium protein, unknown function
PFIT_1118900	410,6107678	449,1059492	386,4346813	321,0218284	429,8583585	353,72825	0,822894909	-0,28122	0,1515871	0,388145466 clathrin assembly protein AP19, putative
PFIT_1210200	3262,810294	3372,486275	3029,446721	2594,926447	3317,648285	2812,1866	0,847644519	-0,238469	0,151556	0,388145466 conserved Plasmodium protein, unknown function
PFIT_1005200	1754,294033	1932,952005	2155,148732	2265,210077	1843,623019	2210,1795	1,19882399	0,2616199	0,1515817	0,388145466 DnaJ protein, putative
PFIT_1214900	32,37791	35,92847594	51,13343939	53,50363807	34,15319297	52,318539	1,531878404	0,6153018	0,1516892	0,388230811 10 kDa chaperonin
PFIT_1365300	7136,385709	7661,148686	6063,92296	6439,162842	7398,767198	6251,5429	0,844943858	-0,243073	0,1520196	0,38890016 conserved Plasmodium protein, unknown function
PFIT_1433700	338,4963318	388,0275401	460,2009545	443,4114005	363,2619036	451,80618	1,243747645	0,3146938	0,1521148	0,388967549 RNA methyltransferase, putative
PFIT_0704300	4176,75039	3742,549577	2859,28134	3624,871479	3959,649984	3242,0764	0,818778534	-0,288455	0,1529882	0,390846778 DNA replication licensing factor MCM7, putative
PFIT_1308200	1296,588123	1505,403142	1764,522785	1629,854575	1400,995633	1697,1887	1,211416111	0,2766945	0,1529343	0,390846778 conserved Plasmodium protein, unknown function
PFIT_1312700	2170,791693	1537,73877	1428,383329	1245,965972	1854,265232	1337,1746	0,7211345	-0,47165	0,1531179	0,39120095 conserved Plasmodium membrane protein, unknown function
PFIT_1210700	85,35994455	93,41403744	75,44277943	56,17881998	89,386991	65,8108	0,736245834	-0,441741	0,1535714	0,391804872 HSP20-like chaperone
PFIT_0506100	518,04656	637,13164	710,0003797	710,2607954	577,5891	710,13059	1,229473665	0,2980408	0,1535413	0,391804872 60S ribosomal subunit protein L24-2, putative
PFIT_0602100	70,64271273	63,47364082	42,75090834	48,82206974	67,05817678	45,786489	0,682787562	-0,550491	0,1538341	0,39212062 mitochondrial chaperone BCS1, putative
PFIT_0418500	15366,26174	16214,52119	13145,48519	13593,93684	15790,39147	13369,711	0,846699149	-0,240079	0,1538246	0,39212062 phosphatidylinositol 4-kinase, putative
PFIT_1221500	523,9334528	452,6987968	390,6259468	397,9333082	488,3161248	394,27963	0,807427008	-0,308596	0,1540831	0,392224166 conserved Plasmodium protein, unknown function
PFIT_1310700	4959,707123	4976,093917	4192,94203	4182,649066	4967,90052	4187,7945	0,842970678	-0,246446	0,1539751	0,392224166 26S proteasome regulatory subunit 7, putative
PFIT_1311100	3252,508232	3731,771034	4076,424848	4276,273873	3492,139633	4176,3516	1,195929144	0,2581319	0,1540783	0,392224166 vacuolar ATP synthase subunit a
PFIT_0301800	41,20824909	35,92847594	46,10392076	79,58666163	38,56836252	62,845291	1,62945189	0,7043868	0,1544447	0,392967503 conserved Plasmodium protein, unknown function
PFIT_0712200	33,84963318	39,52132353	50,29518628	60,19159283	36,68547836	55,24339	1,505865319	0,5905927	0,1545151	0,392969427 conserved Plasmodium protein, unknown function
PFIT_1247900	442,9886778	404,7941622	342,0072667	342,4232837	423,891142	342,21528	0,80731824	-0,308791	0,1548468	0,393635855 conserved Plasmodium protein, unknown function
PFIT_0824800	26,49101728	19,16185383	10,05903726	13,37590952	22,82643555	11,717473	0,51332909	-0,962044	0,1553079	0,394452624 conserved Plasmodium protein, unknown function
PFIT_1036800	1922,070476	2130,558623	2455,243344	2406,994918	2026,314549	2431,1191	1,199773812	0,2627624	0,1552982	0,394452624 DNA repair helicase rad25, putative
PFIT_1115800	41,20824909	28,74278075	41,91265524	66,21075212	34,97551492	54,061757	1,545705437	0,6282617	0,1553798	0,394457917 conserved Plasmodium protein, unknown function
PFIT_1438500	1324,550864	1081,447126	1064,581443	866,0901413	1202,998995	965,33579	0,802441063	-0,317533	0,1555563	0,394728427 conserved Plasmodium protein, unknown function
PFIT_bin07400	30,90618682	44,31178699	30,17711177	18,72627333	37,60898691	24,451693	0,650155576	-0,621143	0,1559154	0,395461865 erythrocyte membrane protein 1 (PfEMP1), pseudogene
PFIT_1233500	89,7751141	116,1687389	119,0319409	155,1605504	102,9719265	137,09625	1,331394394	0,412938	0,1560769	0,395693799 conserved Plasmodium protein, unknown function
PFIT_1248400	781,4850096	938,9308378	1034,404331	1056,696852	860,2079237	1045,5506	1,215462637	0,2815055	0,1561743	0,3956945 RNA binding protein, putative
PFIT_0730600	16,188955	26,34754902	28,50060556	48,15327427	21,26825201	38,32694	1,802072869	0,8496573	0,1562173	0,3956945 Plasmodium exported protein (hyp9), unknown function
PFIT_1305100	259,02328	274,254033	222,9753259	204,6514156	266,6386565	213,81337	0,801884369	-0,318534	0,1563893	0,395853253 conserved Plasmodium protein, unknown function
PFIT_0805200	1674,820981	1828,759425	1662,255907	1258,673086	1751,790203	1460,4645	0,83369829	-0,262403	0,1564202	0,395853253 conserved Plasmodium protein, unknown function
PFIT_0417000	78,00132864	106,5878119	72,92802011	64,87316116	92,2945703	68,900591	0,746529188	-0,421729	0,1578944	0,399362441 conserved Plasmodium protein, unknown function
PFIT_1450600	468,0079719	447,9083334	362,1253412	381,2134213	457,9581526	371,66938	0,811579353	-0,301196	0,1579706	0,399362441 DNA replication related protein, putative
PFIT_1012800	278,1556814	262,2778743	326,9187108	341,0856927	270,2167779	334,0022	1,236052789	0,3057409	0,1580543	0,399362441 conserved Plasmodium protein, unknown function
PFIT_0828200	114,7944082	128,1448975	140,8265216	175,2244147	121,4696529	158,02547	1,300946075	0,3795612	0,1580898	0,399362441 U6 snRNA-associated Sm-like protein LSm3, putative
PFIT_0321500	3217,186876	2655,114372	2602,77589	2271,229436	2936,150624	2437,0027	0,829999198	-0,268818	0,1583303	0,399612337 protein kinase, putative
PFIT_1472100	653,4450928	688,6291221	632,8810941	490,8958793	671,0371075	561,88849	0,83734339	-0,256109	0,1582639	0,399612337 RNA 3'-terminal phosphate cyclase-like protein, putative
PFIT_0728200	2276,755762	2325,770009	2172,752047	1476,031615	2301,262886	1824,3918	0,79277854	-0,33501	0,1585848	0,40005282 60S ribosomal subunit export protein, putative
PFIT_0407200	170,7198891	177,247148	234,7108693	210,6705749	173,9835185	222,69072	1,279952975	0,3560908	0,1586465	0,40005282 sporozoite invasion-associated protein 1 (SIAP-1)
PFIT_1149500	531,2920687	586,8317736	715,8681514	652,7443845	559,0619212	684,30627	1,224025894	0,2916341	0,1587441	0,400120247 serine/threonine protein kinase, FIKK family
PFIT_1010000	30,90618682	29,94039661	16,76506209	18,72627333	30,42329172	17,745668	0,583292166	-0,777709	0,159073	0,400770285 ankyrin-repeat protein, putative
PFIT_0213000	195,7391832	202,3970811	227,1665934	272,1997587	199,0681322	249,68318	1,254259898	0,3268366	0,1592162	0,400952155 conserved Plasmodium protein, unknown function
PFIT_1239800	204,5695223	185,630459	248,122919	242,1039623	195,0999907	245,11344	1,256347783	0,3292359	0,159371	0,400984357 early transcribed membrane protein 12
PFIT_1237900	13,24550864	14,37139038	19,27982141	42,80291046	13,80844951	31,041366	2,247997932	1,1686407	0,1593191	0,400984357 conserved Plasmodium protein, unknown function
PFIT_0622700	30966,52747	33722,46752	29898,81174	25585,43973	32344,49749	27742,126	0,857707737	-0,221442	0,1594928	0,401112046 protein kinase, putative
PFIT_0210700	30,90618682	43,11417113	48,61868007	62,86677474	37,01017897	55,742727	1,506145848	0,5908615	0,1595935	0,4011864 conserved Plasmodium protein, unknown function
PFIT_0813200	15787,17457	13578,56867	12496,67729	12150,00741	14682,87162	12323,342	0,839300558	-0,252741	0,1598431	0,401277891 protein phosphatase, putative
PFIT_1022300	444,4604009	398,8060829	481,1572821	537,0427672	421,6332419	509,10002	1,207447549	0,2719605	0,1598191	0,401277891 4-hydroxy-3-methylbut-2-en-1-yl diphosphate synthase
PFIT_1363700	11,77378546	14,37139038	24,30934004	24,74543261	13,07258792	24,527386	1,876245659	0,9078487	0,1597581	0,401277891 conserved Plasmodium protein, unknown function, pseudogene
PFIT_0413300	703,483681	641,9221034	817,2967771	797,8730028	672,7028922	807,58489	1,200507534	0,2636445	0,1599871	0,401460976 GTP binding protein, putative
PFIT_0526100	66,22754319	62,27602496	42,75090834	45,47809236	64,25178407	44,1145	0,686587944	-0,542484	0,160129	0,401638641 conserved Plasmodium protein, unknown function
PFIT_0605100	919,8269887	952,1046123	1145,053741	1106,856513	935,9658005	1125,9551	1,202987466	0,2666216	0,1604937	0,402374428 ubiquitin conjugating enzyme E2, putative
PFIT_0514700	350,2701173	434,7345588	312,6684081	332,3913515	392,5023381	322,52988	0,821727283	-0,283268	0,1608021	0,402968743 ribose 5-phosphate epimerase, putative
PFIT_1114700	48,566865	43,11417113	67,89850148	64,20436569	45,84051806	66,051434	1,440896316	0,5269665	0,1609738	0,403220001 mitogen-activated protein kinase 2
PFIT_1237300	958,0917914	1152,106462	1382,27937	1187,11197	1055,099127	1284,6957	1,217606609	0,2840481	0,1610533	0,403240223 rRNA processing WD-repeat protein, putative
PFIT_1435700	11110,0383	13316,2908	10246,80595	10661,26868	12213,16455	10454,037	0,855964666	-0,224377	0,1613595	0,403827889 dynein-related AAA-type ATPase, putative
PFIT_1456100	11,77378546	22,75470143	11,73554347	6,019159283	17,26424344	8,8773514	0,514204483	-0,959586	0,1617042	0,404511311 conserved Plasmodium protein, unknown function
PFIT_1114600	301,7032523	234,7327095	298,4181053	399,2708991	268,2179809	348,8445	1,300600732	0,3791781	0,1620087	0,40509352 conserved Plasmodium membrane protein, unknown function
PFIT_1477700	60,34065046	119,7615865	36,88313661	56,17881998	90,05111846	46,530978	0,516717383	-0,952553	0,162763	0,405185154 Plasmodium exported protein, unknown function
PFIT_1476400	51,51031137	51,49748218	44,42741455	26,08302356	51,50389677	35,255219	0,684515566	-0,546845	0,1625833	0,405185154 cytochrome c oxidase assembly protein COX11, putative (COX11)
PFIT_1313900	141,2854255	112,5758913	71,2515139	107,6760716	126,9306584	89,463793	0,704824145	-0,504665	0,162702	0,405185154 conserved Plasmodium protein, unknown function
PFIT_0409300	3114,166253	2540,143249	2137,545417	2459,160965	2827,154751	2298,3532	0,812956274	-0,29875	0,1626988	0,405185154 GTPase, putative
PFIT_0511100	3476,210156	3730,573418	3440,190742	2616,327902	3603,391787	3028,2593	0,840391359	-0,250867	0,1622105	0,405185154 translationally-controlled tumor protein homolog (TCTP)
PFIT_0629500	897,751141	864,6786542	984,109145	1113,544467	881,2148976	1048,8268	1,190205487	0,2512107	0,1624601	0,405185154 conserved Plasmodium protein, unknown function
PFIT_1228200	653,4450928	512,57959	814,7820178	654,7507709	583,0123414	734,76639	1,260293037	0,3337592	0,1621799	0,405185154 RNA pseudouridylate synthase, putative
PFIT_1362500	228,1170932	159,28291	228,0048445	367,1687163	193,7000016	297,58678	1,536328229	0,6194865	0,1627433	0,405185154 DNase I-like protein, putative
PFIT_1246200	26,49101728	43,11417113	47,78042697	92,96257115	34,8025942	70,371499	2,02201878	1,0157964	0,1622801	0,405185154 conserved Plasmodium protein, unknown function
PFIT_1005000	1,471723182	3,592847594	10,05903726	8,025545711	2,532285388	9,0422915	3,570802694	1,8362484	0,1623833	0,405185154 conserved Plasmodium protein, unknown function
PFIT_1316400	429,7431691	401,2013146	286,6825618	379,2070348	415,4722419	332,9448	0,801364724	-0,319469	0,1629929	0,405221409 transcription factor with AP2 domain(s)
PFIT_0607230	993,4131478	863,4810384	1331,984183	1020,581896	928,4470931	1176,283	1,266935993	0,3413436	0,1629631	0,405221409 conserved Plasmodium protein, unknown function
PFIT_1439000	712,3140201	849,109648	1491,252273	943,6704165	780,711834	1217,4613	1,559424735	0,6410139	0,1628645	0,405221409 transport protein particle (TRAPP) component, Bet3
PFIT_0102800	20826,35475	21552,2951	19470,10486	16785,4285	21189,32492	18127,767	0,855514129	-0,225136	0,1631566	0,405449788 ubiquitin carboxyl-terminal hydrolase 1, putative (UBP1)
PFIT_1118600	855,0711687	849,109648	690,7205583	722,9679095	852,0904084	706,84423	0,829541357	-0,269614	0,1633253	0,405512243 DNA mismatch repair protein MLH, putative
PFIT_1136100	1108,207556	1118,573218	963,1528173	903,542688	1113,390387	933,34775	0,838293346	-0,254473	0,1632908	0,405512243 conserved Plasmodium protein, unknown function
PFIT_0315700	80,94477501	104,1925802	123,2232064	126,4023449	92,56867761	124,81278	1,348326225	0,4311696	0,1634411	0,405621401 conserved Plasmodium membrane protein, unknown function
PFIT_0913200	78,00132864	95,80926917	144,179534	102,3257078	86,90529891	123,25262	1,418240573	0,5041023	0,16384	0,406432607 conserved Plasmodium protein, unknown function
PFIT_1429800	256,0798337	221,5589349	186,9304424	188,6003242	238,8193843	187,76538	0,786223379	-0,346989	0,1642575	0,407110649 peptide chain release factor 1, putative
PFIT_1365200	254,6081105	252,6969474	324,4039515	306,9771234	253,652529	315,69054	1,244578711	0,3156575	0,1642117	0,407110649 conserved Plasmodium protein, unknown function
PFIT_1252500	588,6892728	861,0858066	539,8349995	545,7371083	724,8875397	542,78605	0,748786569	-0,417374	0,1643653	0,407199075 conserved Plasmodium protein, unknown function
PFIT_1324600	4373,961297	3392,845744	2890,296705	3225,60058	3883,403521	3057,9486	0,787440354	-0,344757	0,1647177	0,407893101 conserved Plasmodium protein, unknown function
PFIT_0301900	1102,320663	1140,130303	1296,777553	1378,387476	1121,225483	1337,5825	1,192964782	0,2545515	0,1651382	0,408575855 golgi organization and biogenesis factor, putative
PFIT_1034200	111,8509618	113,7735071	129,0909781	163,1860961	112,8122345	146,13854	1,295413904	0,3734131	0,165084	0,408575855 conserved Plasmodium protein, unknown function
PFIT_0103700	376,7611346	289,8230392	282,4912963	234,747212	333,2920869	258,61925	0,775953778	-0,365957	0,1654163	0,408726729 conserved Plasmodium protein, unknown function
PFIT_1107600	3776,441685	3823,987456	4861,868007	4181,978111	3800,21457	4521,9231	1,189912563	0,2508556	0,1653494	0,408726729 protein kinase, putative
PFIT_0220600	55,92548091	69,46172015	76,28103253	97,64413948	62,69360053	86,962586	1,387104669	0,4720767	0,1654007	0,408726729 Plasmodium exported protein (hyp15), unknown function
PFIT_0314900	378,2328578	308,9848931	376,375644	506,9469707	343,6088754	441,66131	1,285360592	0,3621731	0,1655215	0,408807742 conserved Plasmodium membrane protein, unknown function
PFIT_1239300	164,8329964	186,8280749	141,6647747	135,0966861	175,8305356	138,38073	0,787011937	-0,345543	0,1657866	0,409283393 hydroxyethylthiazole kinase, putative
PFIT_1248500	89,7751141	95,80926917	118,1936878	128,4087314	92,79219163	123,30121	1,328788634	0,4101116	0,1658677	0,409304715 conserved Plasmodium membrane protein, unknown function
PF_apicoplast_g	11,77378546	10,77854278	18,4415683	54,84122903	11,27616412	36,641399	3,249455957	1,7001982	0,1660597	0,409599414 probable protein, unknown function
PFIT_1430900	674,0492173	788,0312389	926,2696807	852,7142318	731,0402281	889,49196	1,216748302	0,2830308	0,1661778	0,409672084 mitochondrial ribosomal protein L15 precursor, putative
PFIT_0822200	50,03858819	44,31178699	64,54548906	69,5547295	47,17518759	67,050109	1,421300321	0,5072114	0,1662343	0,409672084 conserved Plasmodium protein, unknown function
PFIT_1026900	82,41649819	77,8450312	63,70723596	52,16604712	80,13076469	57,936642	0,723026191	-0,46788	0,1670096	0,410893117 conserved Plasmodium protein, unknown function
PFIT_0810000	1509,987985	1623,967112	1294,262794	1348,291679	1566,977549	1321,2772	0,843201128	-0,246051	0,1670205	0,410893117 RNA helicase, putative
PFIT_1123900	10452,17804	10409,67709	9380,890495	8403,415155	10430,92757	8892,1528	0,852479587	-0,230263	0,1670208	0,410893117 conserved Plasmodium protein, unknown function
PFIT_0415200	11,77378546	19,16185383	20,95632762	38,12134213	15,46781964	29,538835	1,909696101	0,9333431	0,1668219	0,410893117 prohibitin-like protein, putative
PFIT_0615300	45,62341864	45,50940285	58,67771733	71,56111592	45,56641075	65,119417	1,429110074	0,515117	0,1672018	0,41159118 conserved Plasmodium protein, unknown function
PFIT_1444900	204,5695223	196,4090018	247,2846659	254,1422808	200,489262	250,71347	1,250508236	0,3225146	0,16766	0,412106431 dephospho-CoA kinase, putative (DPCK)
PFIT_0629400	269,3253423	324,5538993	236,3873755	246,7855306	296,9396208	241,58645	0,813587801	-0,29763	0,1677836	0,412188512 microtubule-associated protein ytm1 homologue, putative
PFIT_0409600	9290,988448	12111,48924	9173,841978	8448,893247	10701,23884	8811,3676	0,823396968	-0,28034	0,1678394	0,412188512 conserved Plasmodium protein, unknown function
PFIT_1010300	70,64271273	68,26430428	58,67771733	40,79652403	69,45340851	49,737121	0,716122099	-0,481723	0,1685395	0,412720277 50S ribosomal protein L22, mitochondrial, putative
PFIT_1323700	188,3805673	182,0376114	125,7379657	160,5109142	185,2090894	143,12444	0,772772224	-0,371885	0,1689214	0,412720277 DEAD box helicase, putative
PFIT_0533900	160,4178268	176,0495321	145,8560402	119,7143902	168,2336795	132,78522	0,789290323	-0,341372	0,1682126	0,412720277 conserved Plasmodium protein, unknown function
PFIT_1119400	1127,339957	1439,534269	966,5058298	1122,238809	1283,437113	1044,3723	0,813730808	-0,297376	0,1689336	0,412720277 histone acetyltransferase, putative
PFIT_1225100	387,0631969	383,2370767	352,066304	281,5628954	385,1501368	316,8146	0,822574288	-0,281782	0,1690414	0,412720277 conserved Plasmodium protein, unknown function
PFIT_1318600	2005,958697	1635,943271	1438,442328	1577,019732	1820,950984	1507,731	0,827991002	-0,272313	0,1692231	0,412720277 conserved Plasmodium protein, unknown function
PFIT_1227300	2222,302005	1956,904323	1565,018547	1901,385538	2089,603164	1733,202	0,829440763	-0,269789	0,1683865	0,412720277 cyclin related protein, putative
PFIT_0718900	12967,35296	14226,47886	10519,23821	12555,96626	13596,91591	11537,602	0,848545532	-0,236936	0,1681909	0,412720277 phosphoinositide-binding protein, putative
PFIT_0719500	3128,883485	3470,690776	3146,802155	2479,224829	3299,78713	2813,0135	0,852483321	-0,230256	0,169028	0,412720277 DEAD/DEAH box ATP-dependent RNA helicase, putative
PFIT_1022100	2507,816302	2600,024042	3051,241301	3007,573255	2553,920172	3029,4073	1,186179314	0,2463221	0,1685226	0,412720277 kelch protein, putative
PFIT_1103600	29003,24875	33098,50965	41236,18498	33956,7527	31050,8792	37596,469	1,210802071	0,275963	0,1687954	0,412720277 early transcribed membrane protein 11.2
PFIT_1229800	254,6081105	225,1517825	286,6825618	303,6331461	239,8799465	295,15785	1,230439886	0,2991742	0,1692255	0,412720277 conserved Plasmodium protein, unknown function
PFIT_0618500	111,8509618	110,1806595	133,1822437	154,4917549	111,0158107	143,887	1,296094659	0,3741711	0,1692186	0,412720277 conserved Plasmodium protein, unknown function
PFIT_1134200	97,13373001	95,80926917	163,4593554	110,3512535	96,47149959	136,9053	1,419126945	0,5050036	0,1691708	0,412720277 conserved Plasmodium protein, unknown function
PFIT_0202100	16,188955	17,96423797	23,47108693	34,77736475	17,07659649	29,124226	1,705505302	0,7701992	0,1687801	0,412720277 conserved Plasmodium protein, unknown function
PFIT_1424900	0	0	2,514759314	4,681568331	0	3,5981638	#DIV/0!	#DIV/0!	0,1685446	0,412720277 small nucleolar RNA snoR23
PFIT_0729200	25981,80105	28608,64777	23439,23331	23302,17197	27295,22441	23370,703	0,856219472	-0,223947	0,1695279	0,413279194 transcription factor with AP2 domain(s) (AP2-L)
PF_apicoplast_g	7,35861591	4,790463458	10,05903726	29,42700094	6,07453968	19,743019	3,250125956	1,7004956	0,1700651	0,414409896 apicoplast ribosomal protein L14
PFIT_1112300	142,7571486	149,7019831	168,4888741	203,3138247	146,2295659	185,90135	1,271297964	0,3463022	0,1704402	0,415144669 acylphosphatase, putative
PFIT_1422000	300,2315291	395,2132353	307,6388894	233,4096211	347,7223822	270,52426	0,777989192	-0,362178	0,170566	0,415258411 conserved Plasmodium protein, unknown function
PFIT_1363400	3785,272024	4107,822416	2897,00273	3710,4773	3946,54722	3303,74	0,837121623	-0,256491	0,170634	0,415258411 conserved Plasmodium protein, unknown function
PFIT_1142300	136,8702559	99,40211676	95,56085394	79,58666163	118,1361863	87,573758	0,741294945	-0,43188	0,1712399	0,416553392 dolichol phosphate mannose synthase
PFIT_0910700	4658,003871	4340,159893	4049,600749	3586,081342	4499,081882	3817,841	0,848582254	-0,236874	0,1713194	0,416567365 DNA repair protein REV1, putative
PFIT_1241400	1583,574144	1758,100089	1379,76461	1449,948592	1670,837116	1414,8566	0,846795051	-0,239915	0,1714996	0,416825937 conserved Plasmodium protein, unknown function
PFIT_1420900	791,7870719	800,0073975	687,3675459	644,0500433	795,8972347	665,70879	0,836425565	-0,257691	0,1718617	0,417526224 YTH domain-containing protein, putative
PFIT_0313600.2	58,86892728	58,68317736	36,0448835	44,14050141	58,77605232	40,092692	0,682126323	-0,551889	0,1720653	0,417711269 conserved Plasmodium protein, unknown function
PFIT_0624700	141,2854255	194,0137701	144,179534	111,6888445	167,6495978	127,93419	0,76310466	-0,390047	0,1721123	0,417711269 conserved Plasmodium protein, unknown function
PFIT_1138700	3364,359194	2736,552251	2179,458072	2667,825153	3050,455722	2423,6416	0,79451788	-0,331848	0,1721598	0,417711269 conserved Plasmodium protein, unknown function
PFIT_0403800	3565,98527	3483,86455	2807,309648	3131,969214	3524,92491	2969,6394	0,842468849	-0,247305	0,1723679	0,418036647 Sec24 subunit b
PFIT_1355300	771,1829473	723,3599822	706,6473673	500,9278115	747,2714648	603,78759	0,807989623	-0,307591	0,1724829	0,418135796 mitochondrial import inner membrane translocase subunit TIM23, putative (TIM23)
PFIT_0920800	4162,033159	3797,639907	3669,872093	3091,17269	3979,836533	3380,5224	0,849412372	-0,235463	0,1733461	0,420048221 conserved Plasmodium protein, unknown function
PFIT_0518300	514,6313905	402,3989305	364,6401006	375,194262	458,0151605	369,91718	0,807652701	-0,308193	0,1736671	0,420464951 SWIB/MDM2 domain-containing protein, putative
PFIT_1437100	10,30206227	10,77854278	21,79458072	19,3950688	10,54030253	20,594825	1,953912111	0,9663656	0,1736517	0,420464951 conserved Plasmodium protein, unknown function
PFIT_0814400	585,7458264	617,9697861	726,7654418	724,9742959	601,8578063	725,86987	1,206048773	0,2702883	0,1739001	0,420754584 histone-arginine methyltransferase, putative
PFIT_1301800	975,7524696	1291,029902	1305,998337	1583,038891	1133,391186	1444,5186	1,274510189	0,3499429	0,1739357	0,420754584 deoxyhypusine hydroxylase
PFIT_1445400	58,86892728	64,67125669	73,76627322	95,63775306	61,77009198	84,702013	1,371246349	0,4554878	0,1740619	0,420879395 calmodulin-like protein
PFIT_1133600	1713,085784	1645,524198	1839,965565	2104,699363	1679,304991	1972,3325	1,174493302	0,2320385	0,1742259	0,421095644 aquaglyceroporin (AQP)
PFIT_0106500	1743,991971	1699,416912	1516,399866	1395,107363	1721,704441	1455,7536	0,845530499	-0,242071	0,1745964	0,421810585 beta3 proteasome subunit, putative
PFIT_0404600	7274,727688	5906,641444	5764,666601	5213,260735	6590,684566	5488,9637	0,832836652	-0,263895	0,1750232	0,422660735 apical sushi protein
PFIT_1334400	16,188955	22,75470143	9,220784152	11,36952309	19,47182821	10,295154	0,528720442	-0,919423	0,1752719	0,422899654 reticulocyte binding protein 2 homologue b, putative
PFIT_1438000	157,4743805	132,935361	179,3861644	186,5939378	145,2048707	182,99005	1,260219786	0,3336754	0,1752145	0,422899654 ATP-dependent Clp protease proteolytic subunit, putative (ClpR)
PFIT_0105900	100,0771764	82,63549466	51,13343939	78,91786616	91,35633551	65,025653	0,711780441	-0,490496	0,1755199	0,423317094 conserved Plasmodium protein, unknown function
PFIT_1420800.2	725,5595287	566,4723039	726,7654418	1133,608332	646,0159163	930,18689	1,439882305	0,5259509	0,1755962	0,423320337 glutathione reductase
PFIT_1431700	72,11443592	57,4855615	94,72260084	81,59304806	64,79999871	88,157824	1,360460281	0,4440948	0,176239	0,424688604 conserved Plasmodium protein, unknown function
PFIT_1451200	2004,486974	2122,175312	2770,426511	2215,050616	2063,331143	2492,7386	1,208113672	0,2727562	0,1764785	0,425084255 serine/threonine protein kinase, putative
PFIT_1033800	372,345965	366,4704546	269,9174997	327,7097832	369,4082098	298,81364	0,808898215	-0,30597	0,1769274	0,425983876 flavoprotein subunit of succinate dehydrogenase
PFIT_1033600	66,22754319	40,7189394	64,54548906	82,93063901	53,47324129	73,738064	1,378971281	0,4635924	0,1770836	0,426178265 apicoplast ribosomal protein L27 precursor, putative
PFIT_1432200	157,4743805	138,9234403	107,2963974	119,0455947	148,1989104	113,171	0,763642565	-0,389031	0,1777573	0,427617176 mitochondrial ribosomal protein L17-2 precursor, putative
PFIT_1426700	373,8176882	360,4823752	287,5208149	308,9835099	367,1500317	298,25216	0,812344101	-0,299837	0,1779945	0,427641355 conserved Plasmodium protein, unknown function
PFIT_1409900	201,6260759	222,7565508	269,0792466	262,836622	212,1913134	265,95793	1,253387474	0,3258325	0,1779334	0,427641355 tRNA-dihydrouridine synthase, putative
PFIT_1240900	13,24550864	11,97635865	23,47108693	22,73904618	12,61093364	23,105067	1,832160126	0,8735456	0,1778991	0,427641355 rifin, pseudogene
PFIT_0713800	38,26480273	21,55708556	16,76506209	17,38868237	29,91094415	17,076872	0,570923878	-0,80863	0,1790633	0,428104999 conserved Plasmodium protein, unknown function
PFIT_0717300	117,7378546	125,7496658	95,56085394	90,95618472	121,7437602	93,258519	0,766022991	-0,38454	0,179003	0,428104999 conserved Plasmodium protein, unknown function
PFIT_0606900	253,1363873	250,3017157	197,8277327	205,3202111	251,7190515	201,57397	0,800789494	-0,320505	0,1786417	0,428104999 conserved protein, unknown function
PFIT_0110500	454,7624632	441,920254	342,8455198	389,9077625	448,3413586	366,37664	0,817182341	-0,29127	0,1790623	0,428104999 TatD-like deoxyribonuclease, putative
PFIT_1445700	2201,69788	2428,764973	1985,821605	1965,589904	2315,231427	1975,7058	0,853351303	-0,228788	0,1784191	0,428104999 eukaryotic initiation factor 2alpha kinase 1
PFIT_0920500	454,7624632	353,2966801	460,2009545	500,9278115	404,0295716	480,56438	1,189428737	0,2502688	0,1786673	0,428104999 conserved Plasmodium protein, unknown function
PFIT_0309600	609,2933973	695,8148173	802,2082212	775,8027521	652,5541073	789,00549	1,209103548	0,2739378	0,1784669	0,428104999 SECIS-binding protein 2, putative
PFIT_1030100	260,4950032	239,5231729	324,4039515	292,263623	250,0090881	308,33379	1,233290316	0,3025124	0,1790941	0,428104999 RNA methyltransferase, putative
PFIT_1301400	385,5914737	342,5181373	522,2316843	396,5957172	364,0548055	459,4137	1,261935549	0,3356382	0,1785145	0,428104999 early transcribed membrane protein 13
PFIT_0828100	42,67997228	38,32370767	47,78042697	67,54834307	40,50183997	57,664385	1,423747293	0,5096931	0,1790974	0,428104999 translation initiation factor IF-3, putative
PFIT_0400400	30,90618682	25,14993316	42,75090834	42,80291046	28,02805999	42,776909	1,526217277	0,6099604	0,1784089	0,428104999 Plasmodium exported protein, unknown function, pseudogene
PFIT_0708400	23,54757091	26,34754902	30,17711177	47,48447879	24,94755997	38,830795	1,556496721	0,6383025	0,1787236	0,428104999 conserved Plasmodium protein, unknown function
PFIT_1408200	872,7318469	811,9835562	694,9118238	707,5856135	842,3577015	701,24872	0,832483299	-0,264507	0,1795868	0,428404595 conserved Plasmodium protein, unknown function
PFIT_1426300	1224,473687	977,2545455	1252,350138	1318,195883	1100,864116	1285,273	1,167512858	0,2234384	0,1794945	0,428404595 conserved Plasmodium protein, unknown function
PFIT_0316200	1661,575472	1549,714929	1738,536939	2017,755951	1605,645201	1878,1464	1,169714483	0,2261564	0,1794848	0,428404595 conserved Plasmodium protein, unknown function
PFIT_1466100	129,51164	108,9830437	130,7674843	170,5428464	119,2473418	150,65517	1,263383846	0,337293	0,1796021	0,428404595 conserved Plasmodium protein, unknown function
PFIT_0510300	14,71723182	16,7666221	26,82409935	28,08940999	15,74192696	27,456755	1,744180032	0,802549	0,1793507	0,428404595 conserved Plasmodium protein, unknown function
PFIT_0928400	612,2368437	677,8505794	798,0169557	759,0828652	645,0437115	778,54991	1,206972328	0,2713926	0,1803189	0,429932646 conserved Plasmodium protein, unknown function
PFIT_1462100.2	97,13373001	79,04264706	56,16295802	70,22352497	88,08818854	63,193241	0,717386094	-0,479178	0,1805398	0,430277553 apicoplast ribosomal protein S10 precursor, putative
PFIT_0105800	58,86892728	70,65933601	54,48645181	39,45893308	64,76413164	46,972692	0,725288694	-0,463373	0,1808575	0,430663382 probable protein, unknown function
PFIT_0909600	5118,653227	5736,579991	5057,180981	4322,425161	5427,616609	4689,8031	0,86406307	-0,210791	0,1809305	0,430663382 exoribonuclease, putative
PFIT_1127900	1012,545549	1027,554412	1159,304044	1257,335495	1020,04998	1208,3198	1,18456918	0,2443625	0,1808997	0,430663382 protein phosphatase, putative
PFIT_1149300.2	2320,907458	2416,788815	2197,061388	1861,926605	2368,848136	2029,494	0,856742974	-0,223066	0,1812018	0,430764391 Plasmodium exported protein, unknown function
PFIT_1300700	194,26746	176,0495321	223,813579	236,084803	185,1584961	229,94919	1,241904616	0,3125544	0,1811826	0,430764391 Plasmodium exported protein (PHISTa), unknown function
PFIT_1111400	170,7198891	162,8757576	199,5042389	218,0273251	166,7978233	208,76578	1,25160975	0,3237848	0,1811019	0,430764391 mitochondrial ribosomal protein L11 precursor, putative
PFIT_0107300	301,7032523	354,4942959	304,285877	241,4351668	328,0987741	272,86052	0,831641394	-0,265967	0,1813654	0,43097183 conserved Plasmodium protein, unknown function
PFIT_0722900	5964,894056	6404,849644	5578,574412	5061,444162	6184,87185	5320,0093	0,860164837	-0,217315	0,1820969	0,432164385 conserved Plasmodium protein, unknown function
PFIT_1412900	1464,364566	1615,583801	1906,18756	1765,620056	1539,974184	1835,9038	1,192165315	0,2535843	0,1820605	0,432164385 p1/s1 nuclease, putative
PFIT_1322500	61,81237364	69,46172015	88,016576	91,6249802	65,63704689	89,820778	1,368446363	0,4525389	0,1820513	0,432164385 acetyltransferase, GNAT family, putative
PFIT_0107500	36676,81342	31684,12531	31044,70373	13746,42221	34180,46937	22395,563	0,655215197	-0,609959	0,1824023	0,432525596 photosensitized INA-labeled protein 1, PhIL1, putative
PFIT_0521300	55,92548091	77,8450312	78,79579185	109,6824581	66,88525606	94,239125	1,408967095	0,4946379	0,1823919	0,432525596 conserved Plasmodium protein, unknown function
PFIT_0422300	4937,631275	4256,326783	6695,965801	5024,660411	4596,979029	5860,3131	1,274818325	0,3502917	0,1828291	0,433355681 reticulocyte binding protein homologue 5
PFIT_1142600	80,94477501	76,64741533	63,70723596	51,49725165	78,79609517	57,602244	0,731029167	-0,451999	0,1829978	0,433573467 inner membrane complex protein 1b, putative
PFIT_0606600	3730,818266	2960,506417	2483,743949	2877,826933	3345,662342	2680,7854	0,801271966	-0,319636	0,1835637	0,434003586 conserved Plasmodium protein, unknown function
PFIT_0833400	16815,90908	19234,9084	13818,60243	16861,00274	18025,40874	15339,803	0,851009972	-0,232752	0,1832594	0,434003586 tryptophan/threonine-rich antigen
PFIT_0516300	8936,303161	10916,26861	9074,928112	7981,40521	9926,285883	8528,1667	0,859149813	-0,219018	0,1835046	0,434003586 40S ribosomal protein S14, putative
PFIT_0821200	160,4178268	161,6781417	218,7840603	189,9379152	161,0479843	204,36099	1,26894471	0,3436292	0,1833895	0,434003586 dynactin subunit 6, putative
PFIT_1129600	176,6067818	168,8638369	186,0921893	268,1869858	172,7353094	227,13959	1,314957482	0,3950162	0,1834856	0,434003586 GPI8p transamidase
PFIT_0316400	326,7225464	312,5777407	223,813579	289,5884411	319,6501435	256,70101	0,803068652	-0,316405	0,1845358	0,434756503 conserved Plasmodium protein, unknown function
PFIT_1107100	693,1816187	576,0532309	503,7901159	538,3803581	634,6174248	521,08524	0,82110137	-0,284368	0,1845752	0,434756503 exonuclease, putative
PFIT_0206300	7499,901335	6567,725401	5686,709063	6174,319834	7033,813368	5930,5144	0,843143561	-0,24615	0,1843685	0,434756503 merozoite surface protein 4
PFIT_1003200	13104,22321	12606,10459	14767,50495	15205,73394	12855,1639	14986,619	1,165805396	0,221327	0,1845168	0,434756503 membrane skeletal protein IMC1-related
PFIT_0305200	7005,402346	7053,957442	8353,192189	8137,903351	7029,679894	8245,5478	1,172962054	0,2301563	0,1844891	0,434756503 conserved Plasmodium membrane protein, unknown function
PFIT_1346700	17160,2923	19815,7521	25226,38893	20271,19088	18488,0222	22748,79	1,23046098	0,2991989	0,1839902	0,434756503 DNA/RNA-binding protein Alba 4 (ALBA4)
PFIT_0210300	130,9833632	98,2045009	132,4439905	156,4981414	114,593932	144,47107	1,26072178	0,3342499	0,1842034	0,434756503 unspecified product
PFIT_1126000	64,75582001	50,29986631	72,08976701	83,59943449	57,52784316	77,844601	1,353163902	0,4363366	0,1845148	0,434756503 vacuolar membrane protein-related, putative
PF_apicoplast_g	10,30206227	8,383311052	13,41204968	35,44616022	9,342686663	24,429105	2,614783716	1,3866916	0,1841666	0,434756503 probable protein, unknown function
PFIT_1212400	694,6533419	731,7432933	656,352181	549,7498812	713,1983176	603,05103	0,845558684	-0,242023	0,1851584	0,435403733 iron-sulfur subunit of succinate dehydrogenase
PFIT_0705000	1097,905494	1043,123418	1127,450426	1380,393862	1070,514456	1253,9221	1,171326681	0,2281435	0,1851512	0,435403733 conserved Plasmodium protein, unknown function
PFIT_1406700	2282,642655	2358,105637	2928,856348	2568,843423	2320,374146	2748,8499	1,184658039	0,2444707	0,1850908	0,435403733 conserved protein, unknown function
PFIT_1111000	6437,317198	7231,20459	8787,407297	7444,362442	6834,260894	8115,8849	1,187529273	0,2479631	0,1851503	0,435403733 pre-mRNA-processing factor 6, putative
PFIT_0403600	3473,266709	3204,820054	2976,636775	2703,271314	3339,043382	2839,954	0,850529244	-0,233567	0,1855721	0,436194774 6-cysteine protein
PFIT_1222000	4437,245394	4467,107175	3498,868459	4052,231789	4452,176284	3775,5501	0,848023502	-0,237824	0,1858866	0,436752221 endoplasmin, putative (GRP94)
PFIT_1437700	51,51031137	49,10225045	58,67771733	80,25545711	50,30628091	69,466587	1,380873043	0,4655807	0,1860318	0,436911708 GTP-binding protein, putative
PFIT_1323000	1153,830975	1154,501693	1005,903726	947,0143939	1154,166334	976,45906	0,846029754	-0,24122	0,1863224	0,437412124 vacuolar protein sorting 52 homologue
PFIT_1413100	2529,89215	1985,647103	2635,467761	2594,926447	2257,769627	2615,1971	1,158309986	0,2120214	0,186448	0,437525187 conserved Plasmodium protein, unknown function
PFIT_0533600.1	2732,989949	2391,638882	1944,747203	2342,121757	2562,314415	2143,4345	0,83652282	-0,257523	0,186546	0,437573154 conserved Plasmodium protein, unknown function
PFIT_0611100	2675,592745	2825,175825	3435,899476	3073,784007	2750,384285	3254,8917	1,183431624	0,2429764	0,1868634	0,43813576 eukaryotic translation initiation factor 3 subunit L, putative
PFIT_1013300	4176,75039	3353,324421	3213,862404	3080,471962	3765,037406	3147,1672	0,835892674	-0,25861	0,1875187	0,43828583 conserved Plasmodium protein, unknown function
PFIT_0716100	638,727861	723,3599822	531,4524684	609,941474	681,0439216	570,69697	0,837973812	-0,255023	0,1872631	0,43828583 dynein heavy chain, putative
PFIT_1418700	629,8975219	693,4195856	629,5280817	494,9086522	661,6585537	562,21837	0,849710721	-0,234956	0,1874395	0,43828583 conserved Plasmodium protein, unknown function
PFIT_0612600	3357,000578	3034,758601	2969,93075	2480,56242	3195,879589	2725,2466	0,852737567	-0,229826	0,187354	0,43828583 conserved Plasmodium protein, unknown function
PFIT_1222800	20583,52042	18765,44298	17373,63385	16195,55124	19674,4817	16784,593	0,853114852	-0,229188	0,1873865	0,43828583 cAMP-dependent protein kinase regulatory subunit
PFIT_1473000	3337,868177	3062,303766	3908,774207	3582,737364	3200,085971	3745,7558	1,170517239	0,2271462	0,1875485	0,43828583 conserved Plasmodium protein, unknown function
PFIT_0730100	1638,027902	1841,9332	2450,213825	1902,723129	1739,980551	2176,4685	1,250857934	0,3229179	0,1871372	0,43828583 Plasmodium exported protein, unknown function
PFIT_0103600	26,49101728	15,56900624	33,53012419	32,77097832	21,03001176	33,150551	1,576344875	0,6565832	0,1871613	0,43828583 conserved Plasmodium protein, unknown function
PFIT_1143300	13834,19791	12452,80976	12201,61219	6592,317006	13143,50384	9396,9646	0,714951258	-0,484083	0,1878284	0,438758345 60S ribosomal protein L35ae, putative
PFIT_0420200	125,0964705	166,4686052	129,0909781	100,3193214	145,7825378	114,70515	0,786823659	-0,345888	0,1879297	0,438813433 steroid dehydrogenase, putative
PFIT_0217100	626,9540755	695,8148173	858,3711793	739,0190009	661,3844464	798,69509	1,207610936	0,2721557	0,1881701	0,439193289 E2F-associated phosphoprotein, putative
PFIT_1116600	21284,06066	20765,46148	20010,77812	12907,75269	21024,76107	16459,265	0,782851484	-0,353189	0,1885191	0,439826021 conserved Plasmodium protein, unknown function
PFIT_0913600	234,0039859	216,7684715	237,2256286	312,3274873	225,3862287	274,77656	1,219136411	0,2858596	0,1889692	0,440693894 protease, putative
PFIT_0307900	164,8329964	144,9115196	113,1641691	125,7335495	154,872258	119,44886	0,771273441	-0,374686	0,1890901	0,440755484 DNA polymerase epsilon subunit b, putative
PFIT_0318900	1019,904165	1098,213748	896,9308221	898,8611196	1059,058956	897,89597	0,84782435	-0,238163	0,1893812	0,440755484 conserved protein, unknown function
PFIT_1451600	4418,112992	4323,393271	4008,526347	3488,437202	4370,753132	3748,4818	0,857628345	-0,221576	0,1893859	0,440755484 zinc finger protein, putative
PFIT_0532300	4479,925366	5367,714305	6350,605521	5435,300833	4923,819835	5892,9532	1,196825508	0,2592128	0,1893829	0,440755484 beta adaptin protein, putative
PFIT_0819600	25,01929409	27,54516489	37,72138971	43,47170593	26,28222949	40,596548	1,544638663	0,6272694	0,1892389	0,440755484 conserved Plasmodium protein, unknown function
PFIT_0204100	111,8509618	119,7615865	75,44277943	99,65052591	115,8062741	87,546653	0,755975039	-0,403589	0,1895043	0,44084924 3'-5' exonuclease, putative
PFIT_1474000	250,1929409	340,1229055	253,9906907	216,6897342	295,1579232	235,34021	0,797336591	-0,326739	0,1896176	0,440931306 dihydroorotase, putative
PFIT_0708600	1278,927445	1293,425134	1134,994704	1050,677693	1286,176289	1092,8362	0,84967839	-0,235011	0,1900286	0,441705043 lysophospholipase, putative
PFIT_1433300	591,6327191	645,514951	516,3639125	522,3292667	618,5738351	519,34659	0,839587063	-0,252248	0,1903257	0,442213459 conserved protein, unknown function
PFIT_1206100	98,60545319	108,9830437	76,28103253	80,92425259	103,7942484	78,602643	0,757292853	-0,401077	0,190485	0,442311173 Tat binding protein 1(TBP-1)-interacting protein, putative
PFIT_1249200	170,7198891	170,0614528	131,6057374	136,4342771	170,3906709	134,02001	0,786545452	-0,346398	0,1905244	0,442311173 conserved Plasmodium membrane protein, unknown function
PFIT_1027100.1	238,4191555	209,5827763	161,7828492	191,9443016	224,0009659	176,86358	0,789566128	-0,340868	0,1912337	0,443593115 centrin-3
PFIT_1121400	460,6493559	414,3750891	325,2422046	387,901376	437,5122225	356,57179	0,814998466	-0,295131	0,1912207	0,443593115 conserved Plasmodium protein, unknown function
PFIT_0723300	1720,4444	1930,556774	2044,499322	2261,197504	1825,500587	2152,8484	1,179319486	0,2379546	0,1913255	0,443623658 conserved Plasmodium membrane protein, unknown function
PFIT_0931900	991,9414246	1070,668583	814,7820178	925,6129387	1031,305004	870,19748	0,843782853	-0,245056	0,1914909	0,443824956 conserved Plasmodium protein, unknown function
PFIT_0611500	38,26480273	51,49748218	54,48645181	98,98173044	44,88114245	76,734091	1,709717866	0,7737583	0,1917308	0,444198631 conserved Plasmodium protein, unknown function
PFIT_1422900	731,4464214	616,7721703	497,0840911	608,6038851	674,1092958	552,84399	0,820110315	-0,28611	0,1918571	0,444308983 Ctr copper transporter domain containing protein, putative
PFIT_0535400	45,62341864	33,53324421	20,11807451	29,42700094	39,57833142	24,772538	0,625911625	-0,675969	0,1920508	0,444427166 mitochondrial ribosomal protein S16 precursor, putative
PFIT_0827200	201,6260759	183,2352273	129,0909781	171,2116418	192,4306516	150,15131	0,780287905	-0,357922	0,1920656	0,444427166 GTP binding protein, putative
PFIT_1243100	130,9833632	112,5758913	84,66356358	98,98173044	121,7796272	91,822647	0,754006636	-0,407351	0,1924267	0,444899654 mitochondrial import inner membrane translocase subunit TIM13, putative (TIM13)
PFIT_0408200	1664,518919	1475,462745	1237,261583	1393,769772	1569,990832	1315,5157	0,837912968	-0,255128	0,1924274	0,444899654 replication protein A1, large subunit
PFIT_0418700	1177,378546	1282,646591	1461,913415	1455,298956	1230,012568	1458,6062	1,18584657	0,2459174	0,1927113	0,445373743 RIO-like serine/threonine kinase (RIO2)
PFIT_1467200	1184,737161	1063,482888	867,5919634	1007,874782	1124,110025	937,73337	0,834200703	-0,261534	0,1928604	0,445535988 protein phosphatase containing kelch-like domains (PPKL)
PFIT_1340900	4,415169546	10,77854278	1,676506209	3,34397738	7,596856164	2,5102418	0,33043166	-1,597576	0,1931971	0,446115018 conserved Plasmodium protein, unknown function
PFIT_0322900	23,54757091	21,55708556	9,220784152	15,38229595	22,55232824	12,30154	0,545466522	-0,874437	0,1932691	0,446115018 conserved Plasmodium protein, unknown function
PFIT_0411100	88,30339092	110,1806595	90,53133531	63,53557021	99,24202523	77,033453	0,776218065	-0,365466	0,1933916	0,446215286 Plasmodium RNA of unknown function RUF6-14
PFIT_0603600	123,6247473	122,1568182	88,016576	99,65052591	122,8907827	93,833551	0,763552391	-0,389201	0,193783	0,446575157 glyoxalase I
PFIT_0814000	214,8715846	239,5231729	179,3861644	187,9315287	227,1973787	183,65885	0,808366926	-0,306918	0,1937848	0,446575157 mitochondrial carrier protein, putative
PFIT_1474700	11,77378546	20,3594697	33,53012419	23,40784166	16,06662758	28,468983	1,771932709	0,8253238	0,1937425	0,446575157 conserved Plasmodium protein, unknown function
PFIT_1467800	360,5721796	437,1297906	461,8774607	506,9469707	398,8509851	484,41222	1,214519291	0,2803854	0,1941425	0,44721696 large ribosomal subunit assembling protein, putative
PFIT_1321700	1069,942753	900,6071302	839,9296109	807,9049349	985,2749417	823,91727	0,83623082	-0,258027	0,1946288	0,448154275 DHHC-type zinc finger protein, putative
PFIT_0720300	391,4783664	462,2797237	487,863307	544,3995174	426,8790451	516,13141	1,209081163	0,2739111	0,1947589	0,448270929 PelOta protein homologue, putative
PFIT_0807900	345,8549478	317,3682041	282,4912963	260,1614401	331,6115759	271,32637	0,818205358	-0,289465	0,1949608	0,448448609 conserved Plasmodium protein, unknown function
PFIT_0830600	38,26480273	31,13801248	43,58916145	56,17881998	34,7014076	49,883991	1,43752067	0,5235827	0,195069	0,448448609 conserved Plasmodium membrane protein, unknown function
PFIT_1360500.2	10,30206227	14,37139038	15,92680899	32,10218284	12,33672632	24,014496	1,94658577	0,9609459	0,1951142	0,448448609 conserved Plasmodium protein, unknown function
PFIT_bin08400	1,471723182	0	5,029518628	4,681568331	0,735861591	4,8555435	6,598446691	2,7221264	0,1951538	0,448448609 rifin
PFIT_1400100	126,5681936	162,8757576	83,82531047	127,7399359	144,7219756	105,78262	0,730936838	-0,452181	0,1952631	0,448517325 B_ITvar26 - erythrocyte membrane protein 1, PfEMP1, putative
PFIT_1345900	61,81237364	61,07840909	40,23614903	46,14688784	61,44539137	43,191518	0,702925272	-0,508557	0,1959651	0,449500988 transmission blocking target antigen precursor
PFIT_1105800	26414,48767	26293,65631	27495,54009	35702,97769	26354,07199	31599,259	1,19902757	0,2618648	0,1957984	0,449500988 histone H4
PFIT_1206500	98,60545319	125,7496658	157,5915837	136,4342771	112,1775595	147,01293	1,310537785	0,390159	0,1959356	0,449500988 conserved Plasmodium protein, unknown function
PFIT_1146200	83,88822137	106,5878119	124,0614595	127,0711404	95,23801666	125,5663	1,318447237	0,3988398	0,1960098	0,449500988 transporter, putative
PFIT_0905300	6195,954596	7330,606707	5454,512953	6208,428403	6763,280652	5831,4707	0,862225151	-0,213863	0,196701	0,450743046 nucleoporin NUP100/NSP100, putative
PFIT_1413900.2	10,30206227	8,383311052	11,73554347	24,74543261	9,342686663	18,240488	1,95238144	0,9652349	0,1967111	0,450743046 non-canonical ubiquitin conjugating enzyme, putative
PFIT_0703100	1743,991971	2015,5875	1544,062219	1678,007849	1879,789735	1611,035	0,857029381	-0,222583	0,1969066	0,451008068 tRNA m5C-methyltransferase, putative
PFIT_1242400	653,4450928	645,514951	824,8410551	726,3118868	649,4800219	775,57647	1,19414985	0,2559839	0,1970274	0,451101612 queuine tRNA-ribosyltransferase, putative
PFIT_1208100	173,6633355	158,0852941	197,8277327	213,3457568	165,8743148	205,58674	1,239412775	0,3096567	0,1971128	0,451114152 conserved Plasmodium protein, unknown function
PFIT_1414200	136,8702559	108,9830437	88,8548291	96,97534401	122,9266498	92,915087	0,755857958	-0,403813	0,198278	0,453597172 conserved Plasmodium protein, unknown function
PFIT_1442400	3455,606031	4171,296056	3551,678405	3008,242051	3813,451044	3279,9602	0,860102881	-0,217419	0,1991743	0,454910327 60S ribosomal protein L1, putative
PFIT_1123800	213,3998614	214,3732398	231,3578569	290,926032	213,8865506	261,14194	1,220936724	0,2879884	0,1990625	0,454910327 dynein heavy chain, putative
PFIT_1406400	169,2481659	161,6781417	194,4747203	216,0209387	165,4631538	205,24783	1,240444321	0,310857	0,1991276	0,454910327 DNA mismatch repair protein, putative
PFIT_1310200	11,77378546	10,77854278	15,92680899	45,47809236	11,27616412	30,702451	2,722774372	1,4450774	0,1990611	0,454910327 conserved Plasmodium protein, unknown function
PFIT_1364000	607,8216741	621,5626337	594,3214513	426,0227182	614,6921539	510,17208	0,829963554	-0,26888	0,1993907	0,455220311 conserved Plasmodium protein, unknown function
PFIT_0913300	3842,669228	4652,737634	3921,348024	3479,074066	4247,703431	3700,211	0,871108613	-0,199075	0,1999613	0,456338494 elongation factor 1-beta
PFIT_0708700	19,13240137	14,37139038	20,95632762	47,48447879	16,75189587	34,220403	2,042777932	1,0305324	0,2000677	0,456396819 conserved Plasmodium protein, unknown function
PFIT_0830300	72,11443592	82,63549466	109,8111567	97,64413948	77,37496529	103,72765	1,340584099	0,4228617	0,2002306	0,456583965 conserved Plasmodium protein, unknown function
PFIT_1400400	1,471723182	7,185695188	11,73554347	11,36952309	4,328709185	11,552533	2,668817143	1,4162005	0,2003625	0,456700216 rifin (RIF)
PFIT_1358100	4388,678529	4512,616578	5307,818659	5106,922254	4450,647553	5207,3705	1,170025349	0,2265398	0,2004434	0,456700245 conserved Plasmodium protein, unknown function
PFIT_1236600	1056,697245	930,5475268	865,0772041	807,9049349	993,6223857	836,49107	0,841860129	-0,248348	0,2007103	0,457123985 conserved Plasmodium protein, unknown function
PFIT_0828900	771,1829473	590,4246212	552,408796	546,4059038	680,8037843	549,40735	0,806998085	-0,309363	0,2010217	0,457648534 conserved Plasmodium protein, unknown function
PFIT_1443900	668,1623246	556,891377	461,8774607	545,7371083	612,5268508	503,80728	0,822506448	-0,281901	0,2011141	0,457674345 conserved Plasmodium protein, unknown function
PFIT_1465000	9242,421583	7864,743383	7482,247213	7088,563249	8553,582483	7285,4052	0,851737298	-0,23152	0,2012884	0,457712928 conserved Plasmodium membrane protein, unknown function
PFIT_0213200	88,30339092	99,40211676	124,8997126	121,0519811	93,85275384	122,97585	1,310306217	0,389904	0,2013742	0,457712928 conserved Plasmodium protein, unknown function
PFIT_1242700	66,22754319	61,07840909	71,2515139	97,64413948	63,65297614	84,447827	1,326690939	0,4078323	0,2012962	0,457712928 conserved protein, unknown function
PFIT_0401000	211071,5953	180850,7741	182201,8566	117140,1964	195961,1847	149671,03	0,763778943	-0,388773	0,2015779	0,457991482 reticulocyte binding protein homologue 1
PFIT_1009000	125,0964705	111,3782754	82,98705737	96,30654853	118,2373729	89,646803	0,758193461	-0,399362	0,201672	0,458021006 metallopeptidase, putative
PFIT_1241500	42,67997228	61,07840909	28,50060556	43,47170593	51,87919069	35,986156	0,69365299	-0,527714	0,201841	0,458220507 conserved Plasmodium protein, unknown function
PFIT_1310600	772,6546705	816,7740196	901,9603407	977,7789858	794,7143451	939,86966	1,182650935	0,2420243	0,2020133	0,458243132 clathrin-adaptor medium chain, putative
PFIT_0727100	39,73652591	37,1260918	56,16295802	54,17243355	38,43130886	55,167696	1,435488341	0,5215416	0,2019823	0,458243132 actin-like protein, putative (ALP3)
PFIT_1037200.2	6051,725724	5080,286498	5135,976773	3972,645127	5566,006111	4554,3109	0,818236786	-0,28941	0,2025222	0,458571352 antigen UB05
PFIT_0105200	5429,186818	5956,94131	4177,853474	5396,510695	5693,064064	4787,1821	0,840879714	-0,250029	0,2024095	0,458571352 small ribosomal subunit assembling AARP2 protein
PFIT_0817700	1635,084455	1754,507242	1922,952622	2055,208497	1694,795848	1989,0806	1,173640213	0,2309902	0,2024193	0,458571352 Fe-superoxide dismutase
PFIT_1318500	2646,158281	3044,339528	3622,091666	3121,268486	2845,248904	3371,6801	1,185021131	0,2449128	0,2022589	0,458571352 N2,N2-dimethylguanosine tRNA methyltransferase, putative
PFIT_0807800	148,6440414	111,3782754	151,723812	170,5428464	130,0111584	161,13333	1,239380767	0,3096195	0,2025641	0,458571352 alpha/beta hydrolase, putative
PFIT_1363800	5058,312576	4978,489149	4770,498419	2608,302356	5018,400863	3689,4004	0,735174508	-0,443841	0,2029234	0,458684233 DNA-directed RNA polymerase 2, putative
PFIT_0416700	1961,807002	2038,342202	1621,181505	1789,696694	2000,074602	1705,4391	0,852687744	-0,229911	0,2028398	0,458684233 conserved Plasmodium protein, unknown function
PFIT_0103400	164,8329964	198,8042335	214,5927948	239,4287804	181,8186149	227,01079	1,248556357	0,3202609	0,2028037	0,458684233 RNase MRP

PFIT_0905200	11,77378546	10,77854278	15,92680899	24,74543261	11,27616412	20,336121	1,803460874	0,8507681	0,2029389	0,458684233	conserved Plasmodium protein, unknown function
PFIT_0535900	316,4204841	310,1825089	283,3295494	234,747212	313,3014965	259,03838	0,826802245	-0,274386	0,2033453	0,459418904	Plasmodium exported protein (PHISTc), unknown function
PFIT_0825700	824,1649819	1004,79971	1158,465791	1034,626601	914,4823461	1096,5462	1,199089518	0,2619394	0,2038753	0,460355807	U5 snrnp-specific protein, putative
PFIT_0607300	51,51031137	35,92847594	55,32470491	64,87316116	43,71939365	60,098933	1,374651568	0,459066	0,2039231	0,460355807	conserved Plasmodium protein, unknown function
PFIT_0701200	7078,988505	7553,363258	7096,650785	5309,567283	7316,175882	6203,109	0,847862208	-0,238098	0,2043944	0,46123528	sporozoite threonine and asparagine-rich protein (STARP)
PFIT_1215000	448,8755705	341,3205214	267,4027404	335,0665334	395,098046	301,23464	0,762430085	-0,391323	0,2047856	0,461739803	conserved Plasmodium protein, unknown function
PFIT_1454400	590,160996	468,2678031	399,846731	458,7936965	529,2143995	429,32021	0,811240613	-0,301798	0,2047767	0,461739803	conserved Plasmodium protein, unknown function
PFIT_0511900	3573,343886	3771,292358	3847,581751	4674,880377	3672,318122	4261,2311	1,160365448	0,2145792	0,2048633	0,461739803	myo-inositol 1-phosphate synthase, putative
PFIT_1367100	924,2421583	887,4333557	727,6036949	796,5354118	905,837757	762,06955	0,841287027	-0,24933	0,2057848	0,462277715	proteasome regulatory subunit, putative
PFIT_1226500	650,5016464	659,8863414	574,2033767	533,0299943	655,1939939	553,61669	0,844966057	-0,243035	0,20577	0,462277715	ataxin-3, putative (ATX3)
PFIT_1108800	3682,251401	3759,316199	3298,525967	3111,905349	3720,7838	3205,2157	0,861435609	-0,215185	0,205682	0,462277715	IWS1-like protein, putative
PFIT_1421700	40466,50061	42254,28293	39671,16643	32408,49117	41360,39177	36039,829	0,871360915	-0,198658	0,2053439	0,462277715	surface protein, Pf113
PFIT_0604500	142,7571486	159,28291	185,2539361	194,6194835	151,0200293	189,93671	1,257692179	0,3307789	0,2058025	0,462277715	nucleoside diphosphate kinase, putative
PFIT_1460500	57,3972041	45,50940285	56,16295802	82,93063901	51,45330348	69,546799	1,351648851	0,4347204	0,2057308	0,462277715	OPA3-like protein, putative
PFIT_1213800	33,84963318	28,74278075	39,39789592	51,49725165	31,29620697	45,447574	1,452175142	0,5382155	0,2054873	0,462277715	histone-lysine N-methyltransferase, putative
PFIT_0835200.1	20,60412455	20,3594697	31,85361798	33,4397738	20,48179712	32,646696	1,593937079	0,6725947	0,2058137	0,462277715	Plasmodium exported protein (PHISTa-like), unknown function
PFIT_0907090	16,188955	17,96423797	23,47108693	32,77097832	17,07659649	28,121033	1,64675863	0,7196291	0,2058389	0,462277715	conserved Plasmodium protein, unknown function
PFIT_1018400	512,1596673	475,4534982	782,0901467	540,3867445	493,8065828	661,23845	1,339063651	0,4212245	0,2060562	0,462581734	conserved Plasmodium protein, unknown function
PFIT_0419500	27,96274046	38,32370767	19,27982141	23,40784166	33,14322406	21,343832	0,643987787	-0,634895	0,2062812	0,462902842	erythrocyte membrane protein 1, PfEMP1, putative
PFIT_1359900	500,3858819	567,6699198	395,6554654	497,5838341	534,0279008	446,61965	0,836322688	-0,257868	0,2071502	0,464668177	polyadenylate-binding protein, putative
PFIT_1135700.3	0	2,395231729	8,382531047	4,012772856	1,197615865	6,197652	5,174991527	2,3715565	0,207321	0,464866762	conserved Plasmodium protein, unknown function
PFIT_0703600	1342,211542	1319,772683	1555,797762	1560,299845	1330,992112	1558,0488	1,170592064	0,2272384	0,2076119	0,465314199	phosphopantetheine adenylyltransferase, putative (PPAT)
PFIT_1221700	176,6067818	189,2233066	238,9021348	217,3585297	182,9150442	228,13033	1,247192833	0,3186845	0,2076854	0,465314199	conserved protein, unknown function
PFIT_0305000	1220,058518	1165,280236	1231,393811	1552,943095	1192,669377	1392,1685	1,167271064	0,2231396	0,2078525	0,465503828	conserved Plasmodium protein, unknown function
PFIT_0805300	1833,767085	1718,578766	1458,560402	1556,287072	1776,172925	1507,4237	0,848691992	-0,236687	0,2079391	0,465513117	inositol phosphatase, putative
PFIT_1133000	7973,7962	8837,207465	8106,745776	5865,336324	8405,501832	6986,041	0,831127182	-0,266859	0,2082897	0,46559242	TCP-1/cpn60 chaperonin family, putative
PFIT_1214700	390,0066432	498,2081997	362,1253412	387,901376	444,1074214	375,01336	0,844420382	-0,243967	0,2083044	0,46559242	conserved Plasmodium protein, unknown function
PFIT_1413900.1	1072,8862	1188,034938	1027,698306	917,587393	1130,460569	972,64285	0,860395202	-0,216929	0,2081994	0,46559242	non-canonical ubiquitin conjugating enzyme, putative
PFIT_1022900	104,4923459	69,46172015	108,1346505	113,0264354	86,97703303	110,58054	1,271376352	0,3463912	0,2082896	0,46559242	HORMA domain protein, putative
PFIT_1000600	36,79307955	49,10225045	38,55964282	22,07025071	42,947665	30,314947	0,705857857	-0,50255	0,2087343	0,466040778	Plasmodium exported protein (hyp12), unknown function
PFIT_1105400	154,5309341	119,7615865	119,870194	93,63136663	137,1462603	106,75078	0,778371791	-0,361469	0,2087526	0,466040778	radial spoke head protein, putative
PFIT_0617700	104,4923459	92,21642157	105,6198912	143,1222318	98,35438375	124,37106	1,264519758	0,3385896	0,2086705	0,466040778	conserved Plasmodium protein, unknown function
PFIT_1460900	61,81237364	61,07840909	66,22199527	101,6569123	61,44539137	83,939454	1,366082174	0,4500443	0,2088375	0,466046132	pyridoxal 5'-phosphate synthase, putative
PFIT_1444500	438,5735082	410,7822416	523,9081905	490,2270838	424,6778749	507,06764	1,194005309	0,2558093	0,2089488	0,466110299	U6 snRNA-associated Sm-like protein LSm5, putative
PFIT_0207300	1196,510947	986,8354724	881,0040131	946,3455984	1091,67321	913,67481	0,836949004	-0,256788	0,2092394	0,466574116	serine repeat antigen 1
PF_apicoplast_g	3355,528855	3377,276738	2814,015673	2958,751185	3366,402796	2886,3834	0,857408814	-0,221945	0,2094761	0,466599049	elongation factor Tu, putative
PFIT_0905000	894,8076946	841,9239528	1006,741979	1028,607442	868,3658237	1017,6747	1,17194238	0,2289016	0,2095812	0,466599049	replication protein A1, small fragment
PFIT_1443500	2497,51424	2677,869073	3337,923863	2765,469293	2587,691656	3051,6966	1,179312292	0,2379458	0,2094423	0,466599049	tRNA binding protein, putative
PFIT_1017600	17,66067818	21,55708556	25,98584625	36,78375118	19,60888187	31,384799	1,600539945	0,6785587	0,2095608	0,466599049	conserved Plasmodium protein, unknown function
PFIT_1300500	30,90618682	44,31178699	26,82409935	24,07663713	37,60898691	25,450368	0,676709753	-0,563391	0,2100628	0,467302715	Plasmodium exported protein (PHISTa-like), unknown function, pseudogene
PFIT_0806200	1118,509618	1033,542491	606,0569947	987,8109179	1076,026055	796,93396	0,740627007	-0,433181	0,2100459	0,467302715	DNA repair protein RAD54, putative
PFIT_1332100	1914,71186	2500,621925	2009,292692	1537,560799	2207,666892	1773,4267	0,803303592	-0,315983	0,2101559	0,467325701	translation initiation factor 6, putative
PFIT_1115500	7885,492809	8397,682442	7601,279154	6572,921937	8141,587626	7087,1005	0,870481394	-0,200115	0,2104669	0,467833091	serine/threonine protein kinase, putative
PFIT_0419900	303,1749755	271,8588013	321,8891922	365,8311253	287,5168884	343,86016	1,195965081	0,2581753	0,2107151	0,468016397	conserved Plasmodium protein, unknown function
PFIT_0405500	125,0964705	130,5401292	162,6211023	161,1797097	127,8182999	161,90041	1,266644966	0,3410122	0,2106549	0,468016397	conserved Plasmodium protein, unknown function
PFIT_1134400	8041,495466	10440,81511	5139,329785	8226,853149	9241,155287	6683,0915	0,723187876	-0,467558	0,2112528	0,46808529	conserved Plasmodium protein, unknown function
PFIT_1458500	3757,309284	4028,779768	5208,904793	4137,168814	3893,044526	4673,0368	1,200355345	0,2634616	0,2111962	0,46808529	conserved Plasmodium protein, unknown function
PFIT_1343200	125,0964705	123,3544341	159,2680899	155,1605504	124,2254523	157,21432	1,265556432	0,3397718	0,2112054	0,46808529	aminomethyltransferase, putative
PFIT_1429000	119,2095777	172,4566845	176,8714051	195,288279	145,8331311	186,07984	1,275977829	0,3516033	0,2111988	0,46808529	exosome complex exonuclease rrp41, putative
PFIT_0714600	75,05788228	69,46172015	77,11928564	115,0328219	72,25980121	96,076054	1,329592002	0,4109836	0,2110286	0,46808529	conserved Plasmodium protein, unknown function
PFIT_0218700	58,86892728	62,27602496	73,76627322	88,9497983	60,57247612	81,358036	1,34315189	0,4256225	0,2110244	0,46808529	conserved Plasmodium protein, unknown function
PFIT_1119600	38,26480273	43,11417113	58,67771733	57,51641093	40,68948693	58,097064	1,427815107	0,5138092	0,2113265	0,46808529	conserved Plasmodium protein, unknown function
PFIT_0216300	70,64271273	77,8450312	36,88313661	67,54834307	74,24387197	52,21574	0,703300332	-0,507787	0,2115759	0,468453955	conserved Plasmodium protein, unknown function
PFIT_1317800	94,19028364	77,8450312	64,54548906	62,86677474	86,01765742	63,706132	0,740616913	-0,433201	0,2122148	0,469500275	conserved Plasmodium protein, unknown function
PFIT_0601500	2553,439721	2791,64258	3429,293451	2872,476569	2672,541151	3150,885	1,178984656	0,2375449	0,2121729	0,469500275	conserved Plasmodium protein, unknown function
PFIT_0615900	1089,075155	1216,777718	932,9757056	1037,301783	1152,926437	985,13874	0,854467998	-0,226902	0,2126267	0,46991851	conserved Plasmodium protein, unknown function
PFIT_0620000	3549,796315	2954,518338	3643,047993	3817,484577	3252,157326	3730,2663	1,147012863	0,1978816	0,2126535	0,46991851	conserved Plasmodium protein, unknown function
PFIT_0912400	166,3047196	166,4686052	177,7096582	269,5245768	166,3866624	223,61712	1,343960593	0,4264908	0,2126037	0,46991851	conserved Plasmodium membrane protein, unknown function
PFIT_0423200	33,84963318	38,32370767	19,27982141	27,42061451	36,08667043	23,350218	0,647059363	-0,62803	0,2129843	0,470317108	Plasmodium exported protein, unknown function, pseudogene
PFIT_0703000	12870,21923	9928,235517	9629,013414	8955,171423	11399,22737	9292,0924	0,815151073	-0,294861	0,2130005	0,470317108	conserved Plasmodium membrane protein, unknown function
PFIT_0924500	8187,196061	8080,314238	7693,486995	6428,462115	8133,75515	7060,9746	0,868107587	-0,204054	0,2132291	0,470637814	conserved Plasmodium protein, unknown function
PFIT_0528600	4687,438334	5010,824777	4695,05564	3208,880693	4849,131556	3951,9682	0,814984729	-0,295155	0,2134187	0,470872177	cytosolic preribosomal GTP-binding protein, putative
PFIT_1236700	328,1942696	356,8895276	235,5491224	323,6970103	342,5418986	279,62307	0,816317851	-0,292797	0,213763	0,471079623	ubiquitin-activating enzyme, putative
PFIT_1141200	198,6826296	196,4090018	245,6081597	240,0975759	197,5458157	242,85287	1,229349591	0,2978952	0,2136078	0,471079623	myosin F, putative
PFIT_1405600.1	27,96274046	29,94039661	48,61868007	38,7901376	28,95156854	43,704409	1,509569638	0,5941373	0,2137243	0,471079623	adenylyl cyclase alpha
PFIT_0910100	1961,807002	2222,775045	2411,654182	2495,275921	2092,291023	2453,4651	1,172621315	0,2297372	0,2143608	0,471844374	helicase with Zn-finger motif, putative
PFIT_1404200	229,5888164	208,3851604	232,19611	294,9388049	218,9869884	263,56746	1,203575881	0,2673271	0,2143361	0,471844374	conserved Plasmodium protein, unknown function
PFIT_1339000	0	0	1,676506209	4,681568331	0	3,1790373	#DIV/0!	#DIV/0!	0,2143307	0,471844374	mitotic-spindle organizing protein 1, putative
PFIT_0215200	166,3047196	147,3067513	118,1936878	127,7399359	156,8057355	122,96681	0,784198432	-0,350709	0,2147791	0,472396884	origin recognition complex subunit 5
PFIT_0906500	362,0439028	437,1297906	363,8018475	314,3338737	399,5868467	339,06786	0,848546101	-0,236935	0,2147163	0,472396884	Maf-like protein, putative
PFIT_0504900	98,60545319	112,5758913	77,11928564	85,60582092	105,5906722	81,362553	0,770546787	-0,376046	0,2156032	0,473840335	conserved protein, unknown function
PFIT_1462500	537,1789614	649,1077986	681,4997742	1304,151178	593,14338	992,82548	1,673837237	0,7431592	0,2155955	0,473840335	40S ribosomal protein S28e, putative
PFIT_1443700	201,6260759	191,6185383	149,2090526	165,1924826	196,6223071	157,20077	0,799506271	-0,322819	0,215758	0,473954558	protein prenyltransferase alpha subunit, putative
PFIT_0922700	4675,664549	5877,898663	6669,979954	5930,209485	5276,781606	6300,0947	1,193927509	0,2557152	0,2158231	0,473954558	glutamine synthetase, putative
PFIT_1423400	1936,787707	2057,504055	2295,137001	2368,873576	1997,145881	2332,0053	1,167668977	0,2236313	0,2160325	0,474230011	conserved Plasmodium protein, unknown function
PFIT_1238000	1381,948068	1778,459559	1010,933244	1421,190386	1580,203813	1216,0618	0,769560107	-0,377894	0,2162657	0,474557369	cell cycle control protein, putative
PFIT_0927700	2700,612039	2035,94697	1968,21829	1825,142854	2368,279504	1896,6806	0,80086855	-0,320363	0,2164511	0,474779672	conserved Plasmodium protein, unknown function
PFIT_1344800	984,5828087	1000,009247	1029,374813	1351,635657	992,2960278	1190,5052	1,19974806	0,2627315	0,2166902	0,475119601	inner membrane complex protein
PFIT_0922100	60,34065046	46,70701872	64,54548906	78,24907068	53,52383459	71,39728	1,33393432	0,4156876	0,2168362	0,475255038	dynein intermediate chain 2, ciliary
PFIT_0417300	3024,391139	3099,429858	2624,570471	2651,774062	3061,910498	2638,1723	0,861609857	-0,214893	0,2169988	0,475426895	RNA-binding protein NOB1, putative
PFIT_1235000	4865,51684	5515,021056	3964,098932	4920,997112	5190,268948	4442,548	0,855937923	-0,224422	0,2170999	0,475463936	CCR4-NOT transcription complex subunit 4, putative
PFIT_0814600	420,91283	323,3562834	299,2563584	306,308328	372,1345567	302,78234	0,813636728	-0,297543	0,2173088	0,475736972	conserved Plasmodium protein, unknown function
PFIT_0302100	2649,101728	2535,352785	2273,34242	2179,604456	2592,227256	2226,4734	0,858903645	-0,219432	0,2174596	0,475882553	cytoadherence linked asexual protein 3.2
PFIT_0730300	1027,262781	930,5475268	1557,474269	1065,391193	978,9051539	1311,4327	1,339693356	0,4219028	0,217856	0,47656526	serine/threonine protein kinase, FIKK family, pseudogene
PFIT_0722600	206,0412455	274,254033	211,2397824	185,2563468	240,1476392	198,24806	0,825525769	-0,276615	0,2185937	0,477993792	mitochondrial import inner membrane translocase subunit tim14, putative (PAM18)
PFIT_1015200.1	27,96274046	17,96423797	12,57379657	13,37590952	22,96348921	12,974853	0,565020974	-0,823624	0,2188047	0,478037404	tubulin binding cofactor c, putative
PFIT_0816000	1504,101092	1134,142224	1057,037165	1061,37842	1319,121658	1059,2078	0,802964447	-0,316592	0,2188676	0,478037404	conserved Plasmodium protein, unknown function
PFIT_1462800	2990,541506	3621,590375	3083,933172	2681,201063	3306,06594	2882,5671	0,871902488	-0,197761	0,218804	0,478037404	splicing factor 3B subunit 2, putative
PFIT_0415800	442,9886778	405,9917781	323,5656984	377,2006484	424,4902279	350,38317	0,82542059	-0,276798	0,219048	0,478246369	bifunctional dihydrofolate reductase-thymidylate synthase
PFIT_1472700	94,19028364	94,6116533	102,2668788	137,771868	94,40096847	120,01937	1,271378624	0,3463937	0,219572	0,47920498	conserved Plasmodium protein, unknown function
PFIT_1105000	11158,60517	12412,09082	11172,23738	9494,889372	11785,34799	10333,563	0,876814446	-0,189657	0,2198945	0,479723382	chromatin remodeling protein
PFIT_0605200	117,7378546	88,62357398	122,3849533	135,7654816	103,1807143	129,07522	1,250962628	0,3230387	0,2201211	0,480032285	conserved Plasmodium protein, unknown function
PFIT_1239600	980,1676392	1114,98037	903,6368469	899,5299151	1047,574005	901,58338	0,860639322	-0,216519	0,2202388	0,480103545	vesicle fusion and protein sorting subunit 16, putative
PFIT_1405600.3	26,49101728	26,34754902	38,55964282	40,79652403	26,41928315	39,678083	1,501860713	0,586751	0,2203969	0,480262699	adenylyl cyclase alpha
PFIT_1345300	8759,696379	9861,169029	8592,094324	7709,205451	9310,432704	8150,6499	0,87543191	-0,191933	0,2207106	0,480760763	Sec61 alpha subunit, PfSec61
PFIT_0316600	2740,348565	3378,474354	2814,853926	2503,970262	3059,411459	2659,4121	0,869256107	-0,202147	0,2211877	0,481428482	formate-nitrite transporter, putative
PFIT_0508300	232,5322627	313,7753565	312,6684081	366,4999208	273,1538096	339,58416	1,243197614	0,3140556	0,2211861	0,481428482	triose phosphate transporter
PFIT_0527600	3426,171568	3334,162567	3086,447932	2759,450134	3380,167067	2922,949	0,864735078	-0,20967	0,2214201	0,481748726	DnaJ protein, putative
PFIT_1205100	33209,4336	33177,5523	32296,21562	22412,67399	33193,49295	27354,445	0,824090578	-0,279125	0,2215445	0,481833606	zinc finger protein, putative
PF_apicoplast_g	1081,716539	1103,004211	955,6085394	916,249802	1092,360375	935,92917	0,856795241	-0,222978	0,2218791	0,481956549	molecular chaperone
PFIT_0626000	20936,73399	21945,1131	20051,85252	17412,09022	21440,92354	18731,971	0,873655061	-0,194864	0,2218505	0,481956549	ankyrin-repeat protein, putative
PFIT_1232500	740,2767605	677,8505794	826,5175613	831,981572	709,0636699	829,24957	1,169499442	0,2258912	0,2218788	0,481956549	conserved Plasmodium protein, unknown function
PFIT_1232900	214,8715846	226,3493984	279,1382839	261,4990311	220,6104915	270,31866	1,225320952	0,2931597	0,2219424	0,481956549	conserved protein, unknown function
PFIT_1343600	208,9846918	238,3255571	181,9009237	185,2563468	223,6551244	183,57864	0,820811219	-0,284878	0,2221804	0,482128137	conserved Plasmodium protein, unknown function
PFIT_1421900	428,2714459	341,3205214	331,1099764	303,6331461	384,7959837	317,37156	0,824778778	-0,277921	0,2221922	0,482128137	mannose-1-phosphate guanyltransferase, putative
PFIT_1229100	1695,425106	2088,642068	2109,883065	2330,752234	1892,033587	2220,3176	1,173508581	0,2308284	0,2223264	0,482233812	macrophage migration inhibitory factor
PFIT_1019900	503,3293282	598,8079323	510,4961408	436,0546503	551,0686303	473,2754	0,858832039	-0,21955	0,2225549	0,482544179	S-adenosyl-L-methionine-dependent methyltransferase, putative
PFIT_1150200	11,77378546	8,383311052	2,514759314	5,350363807	10,07854825	3,9325616	0,390191272	-1,357747	0,2226506	0,482566386	rifin
PFIT_0209600	244,3060482	191,6185383	174,3566458	174,5556192	217,9622933	174,45613	0,80039593	-0,321214	0,2228674	0,482613321	monocarboxylate transporter, putative
PFIT_0918000	30884,11097	35669,79091	28895,42277	29284,5475	33276,95094	29089,985	0,874178202	-0,194001	0,2229287	0,482613321	heat shock protein 70
PFIT_0401800	266,3818959	288,6254234	350,3897978	324,3658058	277,5036596	337,3778	1,215759829	0,2818583	0,2228767	0,482613321	regulator of chromosome condensation, putative
PFIT_0320500	2372,417769	1955,706707	1993,365883	1562,975027	2164,062238	1778,1705	0,821681754	-0,283348	0,2232383	0,483098256	conserved Plasmodium protein, unknown function
PFIT_1249000	33,84963318	50,29986631	28,50060556	30,09579642	42,07474975	29,298201	0,696336904	-0,522143	0,2234492	0,483369417	conserved Plasmodium protein, unknown function
PFIT_0902300	1233,304026	1202,406328	1409,103469	1426,54075	1217,855177	1417,8221	1,164195987	0,2193339	0,223885	0,484126697	serine/threonine protein kinase, FIKK family
PFIT_1018900	3002,315291	3245,538993	3047,050036	2277,917391	3123,927142	2662,4837	0,852287391	-0,230588	0,2243035	0,48484599	60S ribosomal protein L30e, putative
PFIT_1427500	66,22754319	67,06648842	82,98705737	92,96257115	66,6470158	87,974814	1,320011304	0,4005503	0,2244198	0,484911666	ATP-binding cassette sub-family G member 2 (ABCG2)
PFIT_0417800	120,6813009	101,7973485	120,7084471	208,6641885	111,2393247	164,68632	1,480468514	0,5660538	0,2247383	0,485414097	CGI-141 protein homolog, putative
PFIT_0203100	72,11443592	62,27602496	51,11343939	46,81568331	67,19523044	48,974561	0,728839845	-0,456326	0,2252777	0,485766964	conserved Plasmodium protein, unknown function
PFIT_1238700	1688,06649	1723,369229	1494,605286	1445,267023	1705,717859	1469,9362	0,861769809	-0,214626	0,2250978	0,485766964	HD superfamily phosphohydrolase protein
PFIT_1337900	679,9361101	640,7244876	691,5588114	844,0198906	660,3302988	767,78935	1,162735304	0,2175227	0,2253319	0,485766964	conserved Plasmodium protein, unknown function
PFIT_1104400	263,4384496	328,1467469	354,5810633	365,1623299	295,7925982	359,8717	1,216635233	0,2828867	0,2252204	0,485766964	actin-like protein, putative (ALP2a)
PFIT_1225600	98,60545319	77,8450312	97,23736015	125,064754	88,22524219	111,15106	1,259855505	0,3332583	0,2251364	0,485766964	conserved Plasmodium protein, unknown function
PFIT_1329500	73,5861591	111,3782754	108,9729036	175,8932102	92,48221725	142,43306	1,540112912	0,6230361	0,2254606	0,485859064	conserved Plasmodium protein, unknown function
PFIT_0819000	465,0645255	526,9509804	542,3497588	633,3493157	496,007753	587,84954	1,185161993	0,2450843	0,2257851	0,48637255	flavoprotein, putative
PFIT_0614100	55,92548091	59,88079323	50,29518628	34,77736475	57,90313707	42,536276	0,734610898	-0,444948	0,2260077	0,486429798	enoyl-acyl carrier reductase
PFIT_1217000	1997,128358	1308,99414	2419,19846	1906,735902	1653,061249	2162,9672	1,308461609	0,3878716	0,2260701	0,486429798	GTP binding protein, putative
PFIT_1137300	1947,08977	2271,877295	2268,312901	3313,881583	2109,483532	2791,0972	1,323118763	0,4039426	0,2260119	0,486429798	conserved Plasmodium protein, unknown function
PFIT_1342600	1767,539542	1806,004724	2008,454439	2138,139137	1786,772133	2073,2968	1,160358811	0,214571	0,2263935	0,486940169	DNA repair protein RAD5, putative (RAD5)
PFIT_1131000	21755,01208	23303,22799	22629,48082	16541,9873	22529,12003	19585,734	0,832404018	-0,264644	0,2267075	0,487429743	60S ribosomal protein P0
PFIT_1125100	696,1250651	585,6341578	496,2462431	585,9389202	640,8796114	541,09258	0,844296763	-0,244178	0,2277967	0,489585147	conserved Plasmodium protein, unknown function
PFIT_0807200	532,7637919	398,8060829	389,7876937	354,4616022	465,7849374	372,12465	0,798919454	-0,323878	0,2280137	0,489865025	methionine aminopeptidase 1c, putative
PFIT_1138900	58,86892728	53,89271391	76,28103253	74,9050933	56,38082059	75,593063	1,340758473	0,4230494	0,2282565	0,490200096	conserved Plasmodium membrane protein, unknown function
PFIT_1001000	7508,731674	9219,246926	7687,619224	6999,613451	8363,9893	7343,6163	0,878004033	-0,187701	0,2290696	0,491759319	alpha/beta hydrolase, putative
PFIT_0806000	2584,345907	2056,306439	1915,408344	1985,653768	2320,326173	1950,5311	0,840627959	-0,250461	0,2294049	0,492188786	filament assembling protein, putative
PFIT_0419700	1,471723182	2,395231729	3,353012419	10,03193214	1,933477456	6,6924723	3,461365562	1,7913413	0,229464	0,492188786	rifin, pseudogene
PFIT_1150600	29,43446364	50,29986631	22,63283383	30,09579642	39,86716498	26,364315	0,661303986	-0,596614	0,2298848	0,492572824	rifin (RIF)
PFIT_1339300	504,8010514	571,2627674	521,3934311	400,6084901	538,0319094	461,00096	0,856828289	-0,222922	0,229853	0,492572824	exosome complex component RRP42, putative
PFIT_0529700	936,0159437	990,42832	1070,449215	1178,417629	963,2221319	1124,4334	1,167366679	0,2232578	0,229724	0,492572824	conserved Plasmodium protein, unknown function
PF_M76611000	50,03858819	37,1260918	27,66235246	30,76459189	43,58233999	29,213472	0,67030527	-0,57711	0,2305227	0,493379393	unspecified product
PFIT_1146700	1345,154988	1560,493472	1292,586288	1234,596449	1452,82423	1263,5914	0,869748275	-0,20133	0,2305944	0,493379393	nucleolar preribosomal assembly protein, putative
PFIT_0521500	827,1084283	857,492959	1141,700729	887,4915965	842,3006936	1014,5962	1,204553398	0,2684983	0,2306767	0,493379393	conserved Plasmodium protein, unknown function, putative
PFIT_1449900	351,7418405	378,4466132	495,4075849	387,2325806	365,0942268	441,32008	1,208784063	0,2735565	0,2306981	0,493379393	conserved Plasmodium protein, unknown function
PFIT_1455800	169,2481659	192,8161542	238,9021348	210,6705749	181,0321601	224,78635	1,241692939	0,3123085	0,2305173	0,493379393	mitochondrial ribosomal protein S11 precursor, putative
PFIT_0826500	130,9833632	178,4447638	236,3873755	178,5683921	154,7140635	207,47788	1,34104088	0,4233532	0,2310003	0,493838517	lipoate-protein ligase (LipB)
[illegible]	[illegible]	[illegible]	[illegible]	[illegible]	[illegible]	[illegible]	[illegible]	[illegible]	[illegible]	[illegible]	[illegible]

PFIT_0218800.1	291,40119	283,8349599	331,9482295	339,0793063	287,618075	335,51377	1,166525323	0,2222176	0,3073076	0,579054067	ribosome associated membrane protein RAMP4, putative
PFIT_bin10900	360,5721796	360,4823752	299,2563584	317,6778511	360,5272774	308,4671	0,855599906	-0,224992	0,3077944	0,579216118	A_ITvar18 - erythrocyte membrane protein 1, PfEMP1, putative
PFIT_1406100	763,8243314	828,7501783	624,498563	761,7580471	796,2872549	693,12831	0,87045008	-0,200167	0,3078689	0,579216118	GTPase-activating protein, putative
PFIT_1200500	1505,572815	1735,345388	1850,862855	1861,926605	1620,459101	1856,3947	1,145598015	0,1961009	0,3077923	0,579216118	serine/threonine protein kinase, FIKK family
PFIT_0501900	643,1430305	536,5319073	426,6708303	552,4250631	589,8374689	489,54795	0,82997092	-0,268867	0,3080907	0,579440323	chromosome assembly factor 1
PFIT_0501000	1014,017272	871,8643494	986,6239043	1132,939536	942,9408109	1059,7817	1,123911181	0,168528	0,3082718	0,579587926	serine/threonine protein kinase, FIKK family
PFIT_0610800	8814,150137	7933,007487	8136,084635	6716,044169	8373,578812	7426,0644	0,886844749	-0,173247	0,3084192	0,579671968	conserved Plasmodium protein, unknown function
PFIT_1131700	488,6120964	487,4296569	580,0711485	547,7434948	488,0208766	563,90732	1,155498358	0,2085152	0,3085685	0,579759728	mitochondrial rpoD precursor, putative
PFIT_0815400	4616,795622	5269,509804	5497,263861	5746,959525	4943,152713	5622,1117	1,13735343	0,1856806	0,3089921	0,580362448	RNA binding protein, putative
PFIT_0812400	50,03858819	70,65933601	65,38374217	98,31293496	60,3489621	81,848339	1,356250973	0,4396242	0,3094623	0,581052201	histone acetyltransferase subunit NuA4, putative
PFIT_1210600	306,1184218	343,7157531	246,4464128	308,3147144	324,9170875	277,38056	0,853696448	-0,228205	0,3097463	0,581198847	kinesin-like protein, putative
PFIT_1329800	718,2009128	617,9697861	741,8539977	772,4587747	668,0853495	757,15639	1,133322841	0,1805589	0,3097428	0,581198847	conserved Plasmodium protein, unknown function
PFIT_0521600	150,1157646	94,6116533	92,20784152	86,94341187	122,3637089	89,575627	0,732044063	-0,449998	0,3100952	0,581473736	conserved Plasmodium protein, unknown function
PFIT_0415400	2217,886835	2511,400468	2119,103849	2092,661044	2364,643652	2105,8824	0,89057074	-0,167198	0,3100987	0,581473736	small GTP-binding protein sar1
PFIT_0509100	606,349951	602,4007799	279,1382839	580,5144731	604,3753654	429,82638	0,711191096	-0,491691	0,3111002	0,582017516	chromosome condensation protein, putative
PFIT_0820800	6010,517475	4802,439617	5125,079482	3702,451755	5406,478546	4413,7656	0,816384562	-0,292679	0,3110354	0,582017516	high mobility group protein B2 (HMGB2)
PFIT_1239200	366,4590723	289,8230392	243,0934004	298,2827823	328,1410558	270,68809	0,824913818	-0,277685	0,3110879	0,582017516	DNA gyrase subunit B
PFIT_0924700	890,3925251	703,0005125	655,5139279	704,9104316	796,6965188	680,21218	0,853790827	-0,228045	0,3111103	0,582017516	conserved Plasmodium protein, unknown function
PFIT_0807700	2493,09907	2347,327095	2026,896007	2215,050616	2420,213082	2120,9733	0,876358089	-0,190408	0,31106	0,582017516	GTPase activator, putative
PFIT_1139400	9466,123506	9398,889305	9449,62725	7436,336897	9432,506406	8442,9821	0,895094232	-0,159889	0,3106656	0,582017516	conserved Plasmodium protein, unknown function
PFIT_1037300	554,8396396	578,4484626	712,515139	601,9159283	566,6440511	657,21553	1,159838407	0,2139238	0,3108143	0,582017516	GDP dissociation inhibitor domain containing protein
PFIT_0318200	10444,81942	12645,62591	9173,841978	11005,02956	11545,22267	10089,436	0,873905689	-0,194451	0,3115456	0,582638815	DNA-directed RNA polymerase II, putative
PFIT_1416100	802,0891342	675,4553476	715,0298983	516,3101074	738,7722409	615,67	0,833369161	-0,262972	0,3116941	0,582723436	conserved Plasmodium protein, unknown function
PFIT_1304500	5254,05176	6051,552964	4337,121564	5488,135675	5652,802362	4912,6286	0,869060743	-0,202471	0,312125	0,583199211	conserved Plasmodium protein, unknown function
PFIT_0832900	1,471723182	4,790463458	5,867771733	10,03193214	3,13109332	7,9498519	2,539001915	1,3442615	0,3121552	0,583199211	unspecified product
PFIT_1310300	186,9088441	191,6185383	171,8418865	147,8038002	189,2636912	159,82284	0,844445347	-0,243924	0,3125478	0,583739651	conserved Plasmodium protein, unknown function
PFIT_1456300	353,2135637	403,5965464	388,9494406	486,8831065	378,405055	437,91627	1,157268561	0,2107237	0,312844	0,58409966	protein phosphatase, putative
PFIT_1204300	298,7598059	292,218271	254,8289438	249,4607125	295,4890384	252,14483	0,853313644	-0,228852	0,3133233	0,584327237	cytidine/deoxycytidylate deaminase, putative
PFIT_0508700	2719,74464	2819,187745	2540,74516	2382,249485	2769,466093	2461,4973	0,888798505	-0,170072	0,3134834	0,584327237	Pre-mRNA-processing ATP-dependent RNA helicase prp5, putative
PFIT_0528000	1807,276067	2206,008423	2296,813507	2305,338005	2006,642245	2301,0758	1,146729449	0,1975251	0,3131383	0,584327237	transporter, putative
PFIT_0506600	204,5695223	171,2590686	230,5196038	212,0081659	187,9142955	221,26388	1,177472338	0,2356932	0,3134655	0,584327237	histone deacetylase, putative, pseudogene
PFIT_0901600	1,471723182	2,395231729	9,220784152	3,34397738	1,933477456	6,2823808	3,249265073	1,7001134	0,3131873	0,584327237	stevor
PFIT_0102300	260,4950032	275,4516489	197,8277327	254,8110763	267,973326	226,3194	0,844559449	-0,243729	0,3141052	0,584328738	actin-related protein
PFIT_0910900	185,4371209	219,1637032	173,5183927	170,5428464	202,3004121	172,03062	0,850372067	-0,233834	0,313742	0,584328738	actin-like protein, putative (ALPSa)
PFIT_1143100	1384,891514	1558,09824	1311,866109	1301,475996	1471,494877	1306,6711	0,887988856	-0,171387	0,3139567	0,584328738	coproporphyrinogen III oxidase
PFIT_1475000	2136,94206	2259,901136	2677,380417	2342,790552	2198,421598	2510,0855	1,141767114	0,1912684	0,314064	0,584328738	conserved Plasmodium protein, unknown function
PFIT_0532900	1145,000636	1322,167915	1620,343251	1290,775269	1233,584275	1455,5593	1,179943105	0,2387173	0,3138696	0,584328738	peptidyl-prolyl cis-trans isomerase
PFIT_1010700.1	36,79307955	53,89271391	49,45693318	76,91147973	45,34289673	63,184206	1,393475296	0,4786874	0,3140694	0,584328738	conserved Plasmodium protein, unknown function
PFIT_1311800	741,7484837	675,4553476	595,1597044	631,3429293	708,6019157	613,25132	0,865438412	-0,208497	0,314235	0,584377765	branched-chain alpha keto-acid dehydrogenase, putative
PFIT_1333500	641,6713073	659,8863414	715,8681514	772,4587747	650,7788244	744,16346	1,143496738	0,1934523	0,3144039	0,584499242	chaperone binding protein, putative
PFIT_1463400	1,471723182	4,790463458	5,029518628	10,70072761	3,13109332	7,8651231	2,511941459	1,3288028	0,3152071	0,585799546	conserved Plasmodium protein, unknown function
PFIT_1454500	17,66067818	15,56900624	20,95632762	28,75820546	16,61484221	24,857267	1,496088029	0,5811951	0,3153499	0,585872085	conserved protein, unknown function
PFIT_0818100	6828,795564	7864,743383	8882,968151	7905,162525	7346,769473	8394,0653	1,142551889	0,1922597	0,3155396	0,586031733	importin beta, putative
PFIT_1234900	1754,294033	1603,607643	1748,595976	2011,736792	1678,950838	1880,1664	1,119846002	0,1633004	0,3168539	0,588279298	V-type K -independent H -translocating inorganic pyrophosphatase
PFIT_1203500	13812,12206	11227,64873	12026,41729	8375,325745	12519,8854	10200,872	0,814773554	-0,295529	0,3170245	0,588402404	unspecified product
PFIT_1253100	11,77378546	15,56900624	21,79458072	22,07025071	13,67139585	21,932416	1,604255773	0,6819042	0,3172405	0,588609788	Plasmodium exported protein, unknown function
PFIT_1317600	818,2780892	708,9885918	502,1136097	728,3182733	763,6333405	615,21594	0,805643113	-0,311787	0,317558	0,589005407	chromosome segregation protein, putative
PFIT_1305500	70,64271273	76,64741533	36,88313661	70,89232045	73,64506403	53,887729	0,731722203	-0,450632	0,3181789	0,589189332	SAM dependent methyltransferase, putative
PFIT_1320300	4989,141587	4229,979234	3807,345602	4243,507295	4609,56041	4025,4264	0,873277729	-0,195488	0,3178728	0,589189332	conserved Plasmodium protein, unknown function
PFIT_1352800	2622,61071	2665,892915	3041,182264	2946,044071	2644,251812	2993,6132	1,132121061	0,1790282	0,3180062	0,589189332	ER lumen protein retaining receptor (ERD2)
PFIT_1475300	832,995321	900,6071302	870,9449758	1217,207766	866,8012256	1044,0764	1,204516491	0,2684541	0,317912	0,589189332	conserved Plasmodium membrane protein, unknown function
PFIT_1462700	16,188955	17,96423797	24,30934004	27,42061451	17,07659649	25,864977	1,514644754	0,5989795	0,3181426	0,589189332	meiotic nuclear division protein 1, putative
PFIT_1475800	103,0206227	126,9472816	119,870194	159,8421187	114,9839522	139,85616	1,216310222	0,2825112	0,3183777	0,589364169	protein kinase, putative
PFIT_0509700	289,9294668	223,9541667	357,9340757	269,5245768	256,9418168	313,72933	1,221013108	0,2880787	0,318721	0,589806205	conserved Plasmodium protein, unknown function
PFIT_1230900	727,0312519	752,102763	910,3428717	791,185048	739,5670074	850,76396	1,150354128	0,2020781	0,3189801	0,589923155	conserved Plasmodium protein, unknown function
PFIT_1332900	1861,729825	2137,744318	2038,631551	2698,589745	1999,737072	2368,6106	1,184461038	0,2442307	0,3189932	0,589923155	U3 snoRNA-associated small subunit rRNA processing associated protein, putative
PFIT_0819100	222,2302005	174,8519162	206,2102638	252,1358944	198,5410584	229,17308	1,154285572	0,2070002	0,319128	0,589979292	vacuolar protein-sorting protein VPS2, putative
PFIT_0525300	222,2302005	295,8111186	221,2988197	227,3904618	259,0206595	224,34464	0,866126436	-0,20735	0,3192523	0,590015958	mitochondrial processing peptidase alpha subunit,, putative
PFIT_1029700	9719,259894	8893,49541	9273,594098	6388,334386	9306,377652	7830,9642	0,841462117	-0,24903	0,3196658	0,59058667	conserved Plasmodium protein, unknown function
PFIT_1453400	67,69926637	75,44979947	92,20784152	88,9497983	71,57453292	90,57882	1,265517443	0,3397274	0,3203382	0,591635406	aminomethyltransferase, putative
PFIT_0725200	984,5828087	1027,554412	811,4290054	950,3583713	1006,06861	880,89369	0,875580134	-0,191689	0,3206453	0,592008971	mismatch repair protein PMS1, putative
PFIT_1107500	757,9374387	764,0789216	626,1750692	700,8976588	761,0081802	663,53636	0,871917519	-0,197736	0,3208756	0,592098014	dna2/nam7 helicase family member, putative
PFIT_0913000	1520,290047	1510,193605	1503,82607	1195,806311	1515,241826	1349,8162	0,890825588	-0,166785	0,3209033	0,592098014	26S proteasome regulatory subunit, putative
PFIT_0914900	345,8549478	285,0325758	254,8289438	277,5501225	315,4437618	266,18953	0,843857338	-0,244929	0,3214457	0,592905033	GINS complex subunit Psf3, putative
PFIT_0408100	423,8562764	316,1705883	391,4641999	464,1440603	370,0134323	427,80413	1,156185405	0,2093728	0,3216243	0,593040809	conserved Plasmodium protein, unknown function
PFIT_1444800	282,5708509	262,2778743	290,8738273	337,7417153	272,4243626	314,30777	1,153743257	0,2063222	0,321866	0,593099011	conserved Plasmodium protein, unknown function
PFIT_1133700	85,35994455	77,8450312	86,34006979	113,0264354	81,60248788	99,683253	1,221571244	0,288738	0,3217759	0,593099011	glycine cleavage H protein
PFIT_1414700	4369,546127	3743,747193	4681,64359	4391,311095	4056,64666	4536,4773	1,118282592	0,1612848	0,3221279	0,593387981	conserved Plasmodium protein, unknown function
PFIT_0724300	4,415169546	8,383311052	7,544277943	19,3950688	6,399240299	13,469673	2,104886321	1,0737423	0,3222446	0,593409244	unspecified product
PFIT_1412600	63,28409682	71,85695188	40,23614903	63,53557021	67,57052435	51,88586	0,76787712	-0,381053	0,3226919	0,593652107	GTP binding protein, putative
PFIT_1218600	153,0592109	185,630459	128,252725	155,8293459	169,344835	142,04104	0,838768041	-0,253656	0,3226104	0,593652107	conserved Plasmodium protein, unknown function
PFIT_1328200	491,5555428	543,7176025	476,1277635	434,0482639	517,6365726	455,08801	0,879165109	-0,185794	0,3226059	0,593652107	conserved Plasmodium protein, unknown function
PFIT_0100700	89176,12276	104670,4289	85044,13049	89087,57016	96923,27586	87065,85	0,898296612	-0,154736	0,3233785	0,594721548	ring-infected erythrocyte surface antigen
PFIT_1461600	397,3652591	434,7345588	544,026265	429,3666955	416,049909	486,69648	1,169803117	0,2262657	0,3235257	0,594798403	ubiquitin carboxyl-terminal hydrolase isozyme L3
PFIT_0500700	13,24550864	3,592847594	11,73554347	16,05109142	8,419178116	13,893317	1,650198779	0,7226398	0,3239028	0,59529801	stevor
PFIT_0701100	2921,370516	3340,150646	4186,236005	3255,027581	3130,760581	3720,6318	1,188411473	0,2490344	0,3242407	0,595725062	lysophospholipase, putative
PFIT_0204800	214,8715846	281,4397282	288,359068	300,2891687	248,1556564	294,32412	1,186046382	0,2461604	0,3245854	0,596164399	DNA-directed RNA polymerase II 16 kDa subunit, putative
PFIT_0623800	20,60412455	22,75470143	10,89729036	16,7198869	21,67941299	13,808589	0,636944766	-0,65076	0,3249598	0,59665781	conserved Plasmodium protein, unknown function
PFIT_0914000	603,4065046	640,7244876	574,2033767	521,6604712	622,0654961	547,93192	0,880826742	-0,18307	0,3252457	0,596886213	arginine--tRNA ligase, putative
PFIT_1003600	5716,172839	6582,096792	7136,048681	6844,452901	6149,134815	6990,2508	1,136786068	0,1849608	0,3252956	0,596886213	60S ribosomal protein L13, putative
PFIT_0923200	26,49101728	59,88079323	31,85361798	22,73904618	43,18590525	27,296332	0,632065761	-0,661853	0,3258934	0,59769934	OTU-like cysteine protease, putative
PFIT_0512400	236,9474323	258,6850268	285,0060556	296,2763958	247,8162295	290,64123	1,17280949	0,2299687	0,3259505	0,59769934	PfMAT1 (MAT1)
PFIT_1026600	603,4065046	694,6172015	849,1503951	676,1522262	649,011853	762,65131	1,17509612	0,2327788	0,3264644	0,598447405	mitochondrial ribosomal protein S22 precursor, putative
PFIT_1473700	3120,053146	2905,416087	2945,62141	2421,039623	3012,734617	2683,3305	0,890662756	-0,167049	0,3267161	0,598520103	protein disulfide isomerase
PFIT_0406100	78,00132864	105,3901961	117,3554347	112,35764	91,69576236	114,85654	1,25258282	0,324906	0,3266693	0,598520103	mitochondrial carrier protein, putative
PFIT_0726200	628,4257987	588,0293895	504,6283691	549,7498812	608,2275941	527,18913	0,866762919	-0,206291	0,3270052	0,59885559	DNA (cytosine-5)-methyltransferase, putative
PFIT_1112400	57,3972041	51,49748218	64,54548906	73,56750235	54,44734314	69,056496	1,268317088	0,3429155	0,327131	0,598891525	endonuclease/exonuclease/phosphatase family protein, putative
PFIT_0826800	351,7418405	280,2421123	329,4334702	387,901376	315,9919764	358,66742	1,135052311	0,1827588	0,3277002	0,599739254	dicarboxylate/tricarboxylate carrier
PFIT_1002500	254,6081105	219,1637032	245,6081597	298,9515777	236,8859068	272,27987	1,14941354	0,2008979	0,3283229	0,600489504	conserved Plasmodium protein, unknown function
PFIT_0716500	161,88955	167,666221	181,0626706	208,6641885	164,7778855	194,86343	1,182582413	0,2419407	0,3283027	0,600489504	cysteine repeat modular protein 2
PFIT_1002900	448,8755705	423,9560161	401,5232372	357,1367841	436,4157933	379,33001	0,86919405	-0,20225	0,3289872	0,601120387	N-acetyltransferase, putative
PFIT_1361100	80,94477501	98,2045009	118,1936878	105,6696852	89,57463795	111,93169	1,24959128	0,3214563	0,3288065	0,601120387	14-3-3 protein (14-3-3I)
PFIT_1312400	44,15169546	50,29986631	47,78042697	76,26268425	47,22578089	62,021556	1,313086929	0,3929624	0,3289487	0,601120387	methionyl-tRNA formyltransferase, putative
PFIT_0714900	3588,061118	4383,274064	3799,801324	3400,1562	3985,667591	3599,9788	0,90323106	-0,146833	0,3296526	0,602141263	eukaryotic translation initiation factor 3 37.28 kDa subunit, putative
PFIT_0624900	1446,703888	1489,834136	1467,781186	1160,360151	1468,269012	1314,0707	0,894979502	-0,160073	0,3298027	0,602220638	conserved Plasmodium protein, unknown function
PFIT_1003300	323,7791	382,0394608	359,6105819	502,2654024	352,9092804	430,93799	1,221101331	0,2881829	0,3300504	0,602477947	conserved Plasmodium protein, unknown function
PFIT_1300100	98,60545319	110,1806595	121,5467002	133,0926997	104,3930564	127,3185	1,219606978	0,2864163	0,3306822	0,603436317	A_ITvar7 - erythrocyte membrane protein 1, PfEMP1, putative
PFIT_0910300	2512,231472	2831,163904	3107,404259	2966,107936	2671,697688	3036,7561	1,136639116	0,1847743	0,3308112	0,603476503	Ran-binding protein, putative
PFIT_0621500	55,92548091	44,31178699	50,29518628	77,58027521	50,11863395	63,937731	1,275727722	0,3513204	0,3309225	0,603484672	conserved Plasmodium protein, unknown function
PFIT_1455100	2014,789036	2235,948819	2496,317746	2338,777779	2125,368928	2417,5478	1,137472055	0,1858311	0,3312303	0,603850871	conserved Plasmodium protein, unknown function
PFIT_1216900	5245,22142	5235,97656	6315,398891	5519,569063	5240,59899	5917,484	1,12916176	0,1752522	0,3318985	0,604678504	snoRNA-associated small subunit rRNA processing protein, putative
PFIT_0701500	67,69926637	71,85695188	82,98705737	91,6249802	69,77810912	87,306019	1,251194965	0,3233066	0,3318876	0,604678504	chitinase precursor fragment, truncated
PFIT_1402500	11541,25319	11764,18064	10074,96407	10678,65769	11652,71692	10376,811	0,89050569	-0,167303	0,3321161	0,604879922	Plasmodium exported protein (PHISTb), unknown function
PFIT_0812700	1560,026573	1402,408177	1487,061008	882,1412327	1481,217375	1184,6011	0,799748329	-0,322382	0,3326073	0,60518875	conserved Plasmodium protein, unknown function
PFIT_1139700	2572,572122	1748,519162	2712,587047	2406,994918	2160,545642	2559,791	1,184789126	0,2446303	0,3325151	0,60518875	unspecified product
PF_apicoplast_g	117,7378546	69,46172015	116,5171816	114,3640264	93,59978735	115,4406	1,233342588	0,3025736	0,332457	0,60518875	apicoplast ribosomal protein S5
PFIT_0817900	48,566865	43,11417113	65,38374217	54,17243355	45,84051806	59,778088	1,304044771	0,3829934	0,332757	0,605266028	selenoprotein
PFIT_1252700	27,96274046	29,94039661	24,30934004	16,7198869	28,95156854	20,514613	0,708583835	-0,49699	0,3335117	0,605575	Plasmodium exported protein, unknown function
PFIT_1313100	144,2288718	172,4566845	133,2822437	134,4278907	158,3427782	133,85507	0,845349998	-0,242379	0,3335704	0,605575	conserved Plasmodium protein, unknown function
PFIT_1011200	1053,753798	1200,011096	1031,889572	979,7853722	1126,882447	1005,8375	0,892584204	-0,16394	0,3334069	0,605575	DNA repair protein RAD23, putative
PFIT_1327900	36,79307955	55,09032977	65,38374217	57,51641093	45,94170466	61,450077	1,337566314	0,4196104	0,3335009	0,605575	conserved Plasmodium protein, unknown function
PFIT_1421800	5,886892728	8,383311052	9,220784152	16,7198869	7,13510189	12,970336	1,817820646	0,8622099	0,3330798	0,605575	dynein-associated protein, putative
PFIT_0611300	2,943446364	1,197615865	3,353012419	8,025545711	2,070531114	5,6892791	2,747738986	1,458245	0,3335079	0,605575	conserved protein, unknown function
PFIT_0524300	314,9487609	253,8945633	241,4168942	240,0975759	284,4216621	240,75724	0,846479952	-0,240452	0,3342591	0,606630347	conserved Plasmodium protein, unknown function, putative
PFIT_1118200	3302,54682	2786,852117	2970,769003	2166,897342	3044,699469	2568,8332	0,843706645	-0,245187	0,3343997	0,606690392	conserved Plasmodium protein, unknown function
PFIT_0928000	4247,393103	4100,63672	4923,060484	4467,553779	4174,014912	4695,3071	1,12488988	0,1697838	0,3347962	0,607019615	phosphatidylserine decarboxylase
PFIT_1035800.1	32,37791	35,92847594	50,29518628	42,80291046	34,15319297	46,549048	1,362948653	0,4467312	0,3347111	0,607019615	conserved Plasmodium protein, unknown function
PFIT_1107400	304,6466987	271,8588013	239,740388	250,7983035	288,25275	245,26935	0,850882934	-0,232967	0,3351012	0,607377601	DEAD/DEAH box helicase, putative
PFIT_1128200	250,1929409	259,8826426	295,0650929	299,6203732	255,0377918	297,34273	1,165877147	0,2214158	0,3353539	0,607640644	methyltransferase, putative
PFIT_0524400	178,078505	174,8519162	161,7828492	137,1030726	176,4652106	149,44296	0,846869252	-0,239789	0,3361956	0,607969332	conserved Plasmodium protein, unknown function, putative
PFIT_0509000	715,2574664	712,5814394	677,3085086	584,527246	713,9194529	630,91788	0,883738179	-0,178309	0,3359766	0,607969332	SNAP protein (soluble N-ethylmaleimide-sensitive factor Attachment Protein), putative
PFIT_1364900	7218,802207	8070,733311	8828,481699	8468,288316	7644,767759	8648,385	1,131281588	0,1779581	0,3361953	0,607969332	60S ribosomal protein L40/UBI, putative
PFIT_0620600	603,4065046	621,5626337	761,1338191	646,7252252	612,4845692	703,92952	1,149301644	0,2007575	0,3356454	0,607969332	conserved Plasmodium protein, unknown function
PFIT_1237500	47,09514182	67,06648842	74,60452632	73,56750235	57,08081512	74,086014	1,297914443	0,3761953	0,3362642	0,607969332	ubiquitin-like protein, putative
PFIT_1407900	97,13373001	104,1925802	99,75211946	173,2180283	100,6631551	136,48507	1,355859288	0,4392075	0,3362891	0,607969332	radical SAM protein, putative
PFIT_0535700	30,90618682	49,10225045	43,58916145	73,56750235	40,00421863	58,578332	1,464303863	0,550215	0,3360521	0,607969332	unspecified product
PFIT_1203300	42,67997228	46,70701872	45,45693318	66,21075212	44,6934955	57,833843	1,294010281	0,3718491	0,3366078	0,6081561	cytochrome c1 heme lyase, putative
PFIT_1204100	26,49101728	26,34754902	34,36837729	38,7901376	26,41928315	36,579257	1,384566615	0,4694345	0,3365829	0,6081561	conserved Plasmodium membrane protein, unknown function
PFIT_0703300	1568,856912	1471,869898	1224,687786	1440,585455	1520,363405	1332,6366	0,876525057	-0,190133	0,3369032	0,608494965	phosphoinositide-binding protein, putative
PFIT_1352900	181,0219514	222,7565508	177,7096582	169,8740509	201,8892511	173,79185	0,860827675	-0,216204	0,3378102	0,609747908	conserved Plasmodium protein, unknown function
PFIT_0717800	7866,360407	9512,662812	10409,42705	9387,2133	8689,51161	9898,3202	1,139111221	0,1879086	0,3378129	0,609747908	60S ribosomal protein L11a, putative
PFIT_1253000	2727,103056	2561,700334	2554,15721	2163,553365	2644,401695	2358,8553	0,89201852	-0,164854	0,3380384	0,609960063	Plasmodium exported protein (PHISTb), unknown function
PFIT_1123600	114,7944082	92,21642157	103,943385	144,4598228	103,5054149	124,2016	1,199952718	0,2629776	0,3382569	0,610159244	conserved Plasmodium protein, unknown function
PFIT_0907000	669,6340478	607,1912433	554,9235553	557,7754269	638,4126456	556,34949	0,871457505	-0,198498	0,3383886	0,610201809	probable protein, unknown function
PFIT_1136800	10,30206227	14,37139038	15,08855589	24,07663713	12,33672632	19,582597	1,587341406	0,6666125	0,3386578	0,610492399	conserved Plasmodium protein, unknown function
PFIT_0629000	684,3512796	555,6937612	595,9979575	441,4050141	620,0225204	518,70149	0,836584912	-0,257416	0,3388735	0,610686122	conserved Plasmodium protein, unknown function
PFIT_0414100	3071,486281	3244,341377	3223,921441	1963,583517	3157,913829	2593,7525	0,821349986	-0,283931	0,3395612	0,61152025	nuclear cap-binding protein, putative
PFIT_0715200	322,3073768	293,4158868	264,8879811	262,836622	307,8616318	263,8623	0,857080826	-0,222497	0,3395111	0,61152025	conserved Plasmodium protein, unknown function
PFIT_0718000	528,3486223	481,4415776	430,0238427	445,417787	504,8950999	437,72081	0,866953977	-0,205973	0,3396613	0,61152025	conserved Plasmodium protein, unknown function
PFIT_0904900	225,1736468	162,8757576	200,342492	265,5118039	194,0247022	232,92715	1,200502541	0,2636385	0,3398018	0,611578179	bacterial histone-like protein
PFIT_1316700	2,943446364	5,988079323	9,220784152	10,03193214	4,465762843	9,6263581	2,155590989	1,1080835	0,3402688	0,612223527	conserved Plasmodium protein, unknown function
PFIT_0732000	1568,856912	1441,929501	1751,110736	1632,529757	1505,393206	1691,8202	1,123839432	0,1684359	0,3406969	0,612781186	serine/threonine protein kinase, FIKK family
PFIT_1032800	4109,051124	4632,378164	5023,650857	4866,824678	4370,714644	4945,2378	1,131448326	0,1781707	0,3407958	0,612781186	haloacid dehalogenase-like hydrolase, putative
PFIT_0930600.1	1084,659985	986,8354724	1022,668788	1374,374703	1035,747729	1198,5217	1,157156045	0,2105834	0,3409435	0,612851716	conserved Plasmodium protein, unknown function
PFIT_0407400	7,35861591	9,580926917	5,867771733	2,675181904	8,469771413	4,2714768	0,504320201	-0,987588	0,3416102	0,613659221	conserved Plasmodium protein, unknown function
PFIT_0210600	125,0964705	92,21642157	99,75211946	74,23629783	108,656446	86,994209	0,800635506	-0,320782	0,341583	0,613659221	Rab GTPase 5a
PFIT_1343900	977,2241928	1167,675468	1292,586288	1167,716901	1072,44983	1230,1516	1,14704815	0,197926	0,3420412	0,614238168	conserved Plasmodium protein, unknown function
PFIT_0608200	590,160996	535,3342915	489,5398132	490,2270838	562,7476437	489,88345	0,870520657	-0,20005	0,3422764	0,614465124	conserved Plasmodium protein, unknown function
PFIT_1208300	66,22754319	59,88079323	62,03072975	96,97534401	63,05416821	79,503037	1,260868855	0,3344182	0,3426564	0,614756326	conserved Plasmodium protein, unknown function
PFIT_0813600	17,66067818	19,16185383	26,82409935	27,42061451	18,41126601	27,122357	1,473139159	0,5588937	0,3426399	0,614756326	conserved Plasmodium protein, unknown function
PFIT_1134100	26908,98666	24624,17979	25842,50497	18270,82361	25766,58323	22056,664	0,856018204	-0,224287	0,3427801	0,61478282	apical membrane antigen 1
PFIT_0108700	36,79307955	55,09032977	48,61868007	86,27461639	45,94170466	67,446648	1,468091982	0,5539424	0,34304	0,615053591	FAD-linked sulfhydryl oxidase ERV1, putative (ERV1)
PFIT_0302600	54,45375773	45,50940285	31,85361798	42,13411498	49,98158029	36,993866	0,740149997	-0,43411	0,3433345	0,615285851	CDGSH iron-sulfur domain-containing protein, putative
PFIT_0907500	172,1916123	220,3613191	181,9009237	158,5045278	196,2764657	170,20273	0,867158093	-0,205633	0,3434965	0,615285851	conserved Plasmodium protein, unknown function
PFIT_1010700.2	1,471723182	9,580926917	10,05903726	12,70711404	5,52632504	11,383076	2,05979119	1,0424981	0,3433989	0,615285851	conserved Plasmodium protein, unknown function
PFIT_0102200	459,1776328	364,0752228	397,3319716	267,5181904	411,6264278	332,42508	0,807589257	-0,308306	0,3446021	0,615694297	L-seryl-tRNA(Sec) kinase, putative (PSTK)
PFIT_1007100	332,6094391	411,9798574	373,0226316	240,7663713	372,2946483	306,8945	0,824332294	-0,278702	0,3448219	0,615694297	conserved Plasmodium protein, unknown function
PFIT_0726900	3835,310612	4784,475379	3570,958226	4066,945289	4309,892996	3818,9518	0,886089692	-0,174475	0,3447223	0,615694297	eukaryotic translation initiation factor 2 alpha subunit, putative
PFIT_1012200	2151,659292	2245,529746	2009,292692	1923,455789	2198,594519	1966,3742	0,894377851	-0,161044	0,3451421	0,615694297	protein phosphatase, putative
PFIT_0214200	8899,510081	9355,775134	8264,33736	8120,514669	9127,642608	8192,426	0,897540183	-0,155952	0,3447995	0,615694297	conserved Plasmodium membrane protein, unknown function
PFIT_1363000	849,184276	724,5575981	833,2235861	924,2753477	786,870937	878,74947	1,116764422	0,1593249	0,3444732	0,615694297	conserved Plasmodium protein, unknown function
PFIT_0513300	4169,391774	4580,880682	4702,599918	5126,317323	4375,136228	4914,4586	1,123269851	0,1677046	0,3447192	0,615694297	conserved Plasmodium protein, unknown function
PFIT_0902900	178,078505	160,4805259	174,3566458	218,6961206	169,2795154	196,52638	1,160957855	0,2153156	0,34432	0,615694297	Plasmodium exported protein (PHISTb), unknown function, pseudogene
PFIT_0809900	158,9461037	215,5708556	220,4605665	226,7216663	187,2584796	223,59112	1,194023987	0,2558318	0,3448881	0,615694297	gas41 homologue, putative
PFIT_1037800	25,01929409	28,74278075	31,85361798	42,13411498	26,88103742	36,993866	1,3762068	0,4606973	0,3445453	0,615694297	Plasmodium exported protein, unknown function
PFIT_0921100.2	63,28409682	68,26410428	64,54548906	129,7463223	65,77410055	97,145906	1,476962891	0,5626336	0,3445249	0,615694297	ubiquitin conjugating enzyme, putative
PFIT_1464900	39,73652591	9,580926917	38,55964282	37,45254665	24,65872641	38,006095	1,541283767	0,6241325	0,3451214	0,615694297	mitochondrial ribosomal protein S6-2 precursor, putative
PFIT_bin05200	4,415169546	3,592847594	10,89729036	6,687954759	4,00400857	8,7926226	2,19595498	1,1348485	0,3451359	0,615694297	rifin (RIF)
PFIT_1358700	1658,632026	1464,684202	1136,67121	1512,146571	1561,658114	1324,4089	0,848078641	-0,23773	0,3453149	0,615808002	conserved Plasmodium protein, unknown function
PFIT_0831800	507,7444978	487,4296569	579,2328954	555,100245	497,5870773	567,16657	1,139833802	0,1888235	0,3454442	0,61584398	conserved Plasmodium protein, unknown function
PFIT_1419800	30324,85616	31948,79842	31667,52579	22336,4313	31136,82729	27001,979	0,867203916	-0,205557	0,3458719	0,616260333	signal recognition particle RNA
PFIT_0915400	219,2867541	222,7565508	236,3873755	276,2125316	221,0216525	256,29995	1,159616483	0,2136455	0,345896	0,616260333	conserved Plasmodium protein, unknown function
PFIT_0221300	60,34065046	75,44979947	93,88434773	78,91786616	67,89522496	86,401107	1,272565294	0,3477397	0,3461976	0,616602972	erythrocyte membrane protein 1 (PfEMP1), pseudogene
PFIT_0323900	2453,362544	2383,255571	2686,601201	2727,347951	2418,309057	2706,9746	1,119366678	0,1626827	0,3464551	0,616867805	conserved Plasmodium protein, unknown function
PFIT_bin00200	35,32135637	37,1260918	26,82409935	26,08302356	36,22372408	26,453561	0,730282767	-0,453473	0,3467173	0,617139124	erythrocyte membrane protein 1 (PfEMP1)-like, putative
PFIT_0523400	5,886892728	1,197615865	4,191265524	12,03831857	3,542254296	8,114792	2,290855305	1,1958863	0,3468406	0,617164271	conserved Plasmodium protein, unknown function, putative
PFIT_0319200	1125,868234	1184,44209	1054,522406	1004,530805	1155,155162	1029,5266	0,891245297	-0,166106	0,3475325	0,617873695	endonuclease/exonuclease/phosphatase family protein, putative
PFIT_0619400	855,0711687	913,7809047	879,3275069	1157,016173	884,4260367	1018,1718	1,151223277	0,2031677	0,3474905	0,617873695	cleavage stimulation factor subunit 1-like protein, putative
PFIT_1436000	163,3612732	136,5282086	167,6506209	182,5811649	149,9447409	175,11589	1,167869523	0,2238791	0,3475676	0,617873695	mitochondrial acidic protein MAM33, putative
PFIT_1019700	323,7791	277,8468806	230,5196038	279,5565089	300,8129903	255,03806	0,847829265	-0,238154	0,3480308	0,618307656	conserved Plasmodium protein, unknown function
PFIT_1424400	337,0246087	332,9372104	398,1702247	373,856671	334,9809095	386,01345	1,152344617	0,2045722	0,3479749	0,618307656	conserved Plasmodium membrane protein, unknown function
PFIT_1118400.1	48,566865	38,32370767	29,33885867	34,10856927	43,44528634	31,723714	0,730199215	-0,453638	0,3484544	0,618861107	conserved Plasmodium protein, unknown function
PFIT_0100900	313,4770378	232,3374777	253,9906907	182,5811649	272,9072577	218,28593	0,799853876	-0,322192	0,3485615	0,618861107	lysophospholipase, putative
PFIT_1020600	1324,550864	1446,719964	1580,107102	1557,624663	1385,635414	1568,8659	1,1322357	0,1791743	0,3486862	0,61888787	conserved Plasmodium protein, unknown function
PFIT_1319300	141,2854255	136,5282086	136,6352561	188,6003242	138,906817	162,61779	1,170696973	0,2273677	0,3491372	0,619493536	ATP-dependent Clp protease adaptor protein ClpS, putative
PFIT_0918100	9238,006413	7655,160606	7789,886102	7187,54498	8446,58351	7488,7155	0,886596993	-0,17365	0,3493686	0,619514729	secreted acid phosphatase
PFIT_1439600	4544,681186	4165,307977	4270,061316	5997,089033	4354,994581	5133,5752	1,178778774	0,237293	0,3492604	0,619514729	metacaspase-like protein
PFIT_0410600	503,3293282	364,0752228	333,6247357	371,8502846	433,7022755	352,73751	0,813317176	-0,29811	0,3499502	0,620014664	conserved Apicomplexan protein, unknown function
PFIT_1128600	17,66067818	20,3594697	25,14759314	30,09579642	19,01007394	27,621695	1,453003016	0,5390377	0,3499799	0,620014664	conserved Plasmodium protein, unknown function
PFIT_0728900	10,30206227	13,17377451	17,6033152	20,06386428	11,73791839	18,83359	1,604508492	0,6821314	0,3499275	0,620014664	conserved Plasmodium protein, unknown function
PFIT_0534600	185,4371209	158,0852941	184,415683	213,3457568	171,7612075	198,88072	1,157890788	0,2114992	0,3503685	0,62050829	conserved Plasmodium protein, unknown function
PFIT_1006200	304,6466987	282,637344	294,2268398	217,3585297	293,6420214	255,79268	0,871103814	-0,199083	0,3507643	0,621014546	phosducin-like protein, putative (PhLP1)
PFIT_1246800	619,5954596	625,1554813	673,9554962	734,3374326	622,3754705	704,14646	1,13138531	0,1780903	0,3509725	0,62118833	pre-mRNA-splicing factor, putative
PFIT_1469500	5633,75634	5097,05312	5258,361726	4351,183366	5365,40473	4804,7725	0,895509805	-0,159219	0,3512138	0,621420665	zinc finger protein, putative
PFIT_1327700	289,9294668	302,9968137	269,9174997	246,1167351	296,4631403	258,01712	0,870317697	-0,200386	0,351728	0,621940603	conserved Plasmodium protein, unknown function
PFIT_0530400.1	310,5335914	267,0683378	336,13495	325,0346013	288,8009646	330,58705	1,144688172	0,1949546	0,3517017	0,621940603	ADP-ribosylation factor GTPase-activating protein, putative
PFIT_1335400	310,5335914	227,5470143	202,0189982	235,4160075	269,0403028	218,7175	0,812954418	-0,298754	0,3523917	0,62252923	conserved Plasmodium protein, unknown function
PFIT_0612300	5108,351165	4152,134203	3797,286564	4267,583932	4630,242684	4032,4352	0,870890691	-0,199436	0,3523359	0,62252923	rhoptry protein (ROP14)
PFIT_0808600	2874,275374	2342,536631	2642,173786	3230,282149	2608,406003	2936,228	1,125679041	0,1707955	0,352318	0,62252923	serine/threonine protein kinase, FIKK family
PFIT_0103200	11,77378546	16,7666221	17,6033152	26,08302356	14,27020378	21,843169	1,530683774	0,6141763	0,352545	0,622605316	transporter, putative
PFIT_1215600	1951,504939	2221,577429	2334,534897	2379,574303	2086,541184	2357,0546	1,129646814	0,1758718	0,3530392	0,623283095	serpentine receptor, putative (SR10)
PFIT_0536400	4,415169546	10,77854278	3,353012419	4,012772856	7,596856164	3,6828926	0,484791677	-1,044563	0,3531779	0,623333071	stevor, pseudogene
PFIT_0110800	207,5129687	190,4209225	150,0473057	183,9187559	198,9669456	166,98303	0,839250109	-0,252827	0,3542293	0,624851545	multidrug resistance-associated protein 1
PFIT_1302200	167,7764427	198,8042335	183,5774299	134,4278907	183,2903381	159,00266	0,867490681	-0,20508	0,3543703	0,624851545	conserved Plasmodium protein, unknown function
PFIT_1348400	6750,794236	7131,802474	6960,853782	5627,245134	6941,298355	6294,0495	0,906753915	-0,141217	0,3543341	0,624851545	glutamate--tRNA ligase, putative
PFIT_0724200	2741,820288	3476,678855	2848,38405	13760,46692	3109,249571	8304,4255	2,670877745	1,4173139	0,3544865	0,624861283	5.8S ribosomal RNA
PFIT_1358400	1602,706545	1826,364193	1367,190814	1685,364599	1714,535369	1526,2777	0,890199021	-0,1678	0,3547896	0,624958393	mitotic control protein dis3 homologue, putative
PFIT_1412800	651,9733696	519,7652852	663,896459	648,0628162	585,8693274	655,97964	1,119668853	0,1630721	0,3548736	0,624958393	conserved Plasmodium protein, unknown function
PFIT_0514100	92,71856046	83,83311052	99,75211946	113,0264354	88,27583549	106,38928	1,205191396	0,2692623	0,3547051	0,624958393	tubulin-tyrosine ligase, putative
PFIT_1241200	4,415169546	10,77854278	5,867771733	2,006386428	7,596856164	3,9370791	0,518251102	-0,948277	0,3554073	0,625702988	unspecified product
PFIT_1200200	172,1916123	201,1994653	155,9150775	163,8548916	186,6955388	159,88498	0,856394243	-0,223653	0,3556553	0,62594443	erythrocyte membrane protein 1, PfEMP1
PFIT_1470800	4,415169546	4,790463458	1,676506209	1,337590952	4,602816502	1,5070486	0,327418784	-1,610791	0,3559746	0,626020523	mediator of RNA polymerase II transcription subunit 6, putative
PFIT_1446900	2582,874184	2790,444964	3441,867248	2730,023133	2686,659574	3085,9452	1,148617867	0,1998989	0,3560312	0,626020523	conserved Plasmodium protein, unknown function
PFIT_1126400	625,4823523	605,9936275	802,2082212	619,3046107	615,7379899	710,75642	1,154316329	0,2070386	0,3558678	0,626020523	small nuclear ribonucleoprotein D1, putative
PFIT_0720000	663,7471551	698,2100491	486,1868007	676,1522262	680,9786021	581,16951	0,853432856	-0,22865	0,3563435	0,626374628	conserved Plasmodium protein, unknown function
PFIT_0607700	1617,423777	1395,222482	940,5199835	1456,636547	1506,32313	1198,5783	0,795697976	-0,329707	0,3567194	0,626645234	ornithine aminotransferase
PFIT_1126500	80,94477501	83,83311052	94,72260084	106,3384807	82,38894276	100,53054	1,220194572	0,2871112	0,3566572	0,626645234	conserved Plasmodium protein, unknown function
PFIT_1327800	1548,252787	1693,428833	1423,353772	1478,038002	1620,84081	1450,6959	0,895026753	-0,159997	0,3574224	0,62676247	alpha/beta-hydrolase, putative
PFIT_0304100	3393,793658	3711,411564	3441,028995	2990,853368	3552,602611	3215,9412	0,905235269	-0,143635	0,3573155	0,62676247	EH (Eps15 homology) protein
PFIT_1359800	7967,909307	8790,500446	9334,786574	9470,812734	8379,204877	9402,7997	1,122158939	0,166277	0,3573209	0,62676247	falcilysin
PFIT_0813800.1	640,1995841	573,6579991	689,0440521	678,1586126	606,9287916	683,60133	1,126328725	0,1716279	0,356899	0,62676247	conserved Plasmodium protein, unknown function
PFIT_1360000	819,7498123	819,1692514	1113,200123	825,2936173	819,4595319	969,24687	1,182787963	0,2421915	0,3574522	0,62676247	arginine methyltransferase 5, putative
PFIT_0108400.2	98,60545319	77,8450312	103,1051319	108,3448671	88,22524219	105,725	1,198353179	0,2610532	0,3573081	0,62676247	phosphatidate cytidylyltransferase, putative
PFIT_1471900	462,1210791	359,2847594	419,1265524	520,9916757	410,7029193	470,05911	1,14452343	0,194747	0,3576006	0,626827931	conserved Plasmodium protein, unknown function
PFIT_1454900	2822,765063	3547,338191	1215,467002	3105,88619	3185,051627	2160,6766	0,6783804	-0,559834	0,3582709	0,627613258	co-chaperone p23
PFIT_1325100	735,861591	659,8863414	684,0145335	943,001621	697,8739662	813,50808	1,165694834	0,2211902	0,358164	0,627613258	conserved Plasmodium protein, unknown function
PFIT_1027800	1000,771764	1076,656662	1005,065473	860,7397775	1038,714213	932,90263	0,898132146	-0,155	0,3587957	0,628337655	nucleolar preribosomal assembly protein, putative
PFIT_0926800	513,6313905	579,6460785	453,4949297	510,9597436	546,6387345	482,22734	0,882168252	-0,180874	0,3590439	0,628577323	NAD synthase, putative
PFIT_1443400	476,838311	397,608467	534,8054808	456,7873101	437,223389	495,7964	1,133965858	0,1813772	0,3593278	0,628879427	GTP binding protein, putative
PFIT_0701900	357,6287332	388,0275401	292,5503336	355,7991932	372,8281367	324,17476	0,869501874	-0,201739	0,3598028	0,629515506	conserved Plasmodium protein, unknown function
PFIT_1029800	130,9833632	128,1448975	159,2680899	149,1413911	129,5641304	154,20474	1,190180802	0,2511808	0,3601843	0,629987771	conserved Plasmodium protein, unknown function
PFIT_0927000	292,8729132	265,8707219	229,6813507	248,791917	279,3718176	239,23663	0,856337751	-0,223748	0,360519	0,630377874	replication termination factor, putative
PFIT_0724100	2457,777714	3221,586676	2566,731007	12234,94444	2839,682195	7400,8377	2,60622042	1,3819591	0,3606846	0,630472137	5.8S ribosomal RNA
PFIT_1212200.1	75,05788228	70,65933601	70,4132608	120,3831857	72,85860914	95,398223	1,309360026	0,3888629	0,360942	0,630726837	conserved protein, unknown function
PFIT_0502800	119,2095777	108,9830437	102,2668788	86,94341187	114,0963107	94,605145	0,829169188	-0,270262	0,361248	0,630921466	conserved Plasmodium protein, unknown function
PFIT_1235100	153,0592109	156,8876783	110,6494098	147,8038002	154,9734446	129,22661	0,833862894	-0,262118	0,361432	0,630921466	tetQ family GTPase, putative
PFIT_1337400	4952,348507	5432,385562	6130,983208	5567,722337	5192,367035	5849,3528	1,12652914	0,1718846	0,3615003	0,630921466	60S ribosomal protein L6-2, putative
PFIT_1478000	16,188955	16,7666221	32,69187108	17,38868237	16,47778855	25,040277	1,519638187	0,6037279	0,3614658	0,630921466	Plasmodium exported protein, unknown function, pseudogene
PFIT_0818300	120,6813009	70,65933601	73,76627322	63,53557021	95,67031847	68,650922	0,717578062	-0,478792	0,3616515	0,63099024	conserved Plasmodium protein, unknown function
PFIT_1326900	419,4411069	435,9321747	500,4371035	478,1887653	427,6866408	489,31293	1,144092164	0,1942033	0,3619233	0,631269301	regulator of nonsense transcripts 3B, putative
PFIT_1217100	2186,980648	2092,234915	2710,910541	2145,495887	2139,607782	2428,2032	1,1348824	0,1825428	0,3621674	0,63149998	conserved Plasmodium protein, unknown function
PFIT_1110500	6353,428976	7631,208289	9373,346217	7279,838755	6992,318633	8326,5925	1,190819945	0,2519553	0,3623435	0,63161205	U3/U14 snoRNA-associated small subunit rRNA processing protein, putative
PFIT_0906000	1664,518919	1582,050557	1653,035123	1116,219649	1623,284738	1384,6274	0,85297875	-0,229418	0,3627308	0,632092006	conserved Plasmodium protein, unknown function
PFIT_0916400	98,60545319	93,41403744	113,1641691	117,7080038	96,00974531	115,43609	1,20233718	0,2658415	0,362933	0,632249159	conserved Plasmodium protein, unknown function
PFIT_0915100	809,4477501	737,7313726	664,7347121	694,8784995	773,5895613	679,80661	0,878769104	-0,186444	0,3638548	0,633073738	type II NADH:ubiquinone oxidoreductase
PFIT_0720600	4447,547456	4947,351137	4736,310042	3788,726371	4697,449296	4262,4282	0,907392063	-0,140202	0,3637303	0,633073738	GTP binding protein, putative
PFIT_1218000	2393,021894	2479,06484	3276,731386	2454,479397	2436,043367	2865,6054	1,176335951	0,2343001	0,3637302	0,633073738	adaptor protein subunit, putative
PFIT_1147000	29,43446364	26,34754902	42,75090834	33,4397738	27,89100633	38,095341	1,365864703	0,4498146	0,3637824	0,633073738	conserved Plasmodium protein, unknown function
PFIT_0602400	213,3998614	198,8042335	157,5915837	190,6067106	206,1020475	174,09915	0,844723036	-0,24345	0,3643594	0,633484609	cation/H antiporter
PFIT_1432600	2481,325285	2311,398619	1685,726994	2363,523212	2396,361952	2024,6251	0,844874499	-0,243191	0,3645398	0,633484609	conserved Plasmodium protein, unknown function
PFIT_1135300	334,0811623	313,7753565	270,7557528	288,9196456	323,9282594	279,8377	0,863887886	-0,211084	0,3644119	0,633484609	zinc finger protein, putative
PFIT_0414400	1683,65132	1489,834136	1569,209812	1265,36104	1586,742728	1417,2854	0,893204299	-0,162938	0,3644629	0,633484609	RING zinc finger protein, putative
PFIT_0108800	80,94477501	74,2521836	75,44277943	115,0328219	77,59847931	95,237801	1,227315297	0,2955059	0,3648029	0,633746898	Plasmodium RNA of unknown function RUF5
PFIT_1020800	163,3612732	138,9234403	107,2963974	141,7846409	151,1423567	124,54052	0,823994821	-0,279293	0,3651084	0,633887474	conserved Plasmodium membrane protein, unknown function
PFIT_1021000	841,8256601	970,0688503	1076,316986	987,8109179	905,9472552	1032,064	1,139209757	0,1880334	0,3651059	0,633887474	RNA helicase, putative
PFIT_1310900	356,15701	462,2797237	387,2729344	337,0729199	409,2183669	362,17293	0,885035855	-0,176192	0,3657668	0,634668067	cytochrome c2 precursor, putative
PFIT_1214300	17,66067818	35,92847594	32,69187108	44,14050141	26,79457706	38,416186	1,433729898	0,5197733	0,3657828	0,634668067	conserved Plasmodium protein, unknown function
PFIT_0704900	4366,602681	4709,02558	5391,64397	4820,008995	4537,81413	5105,8265	1,12517312	0,170147	0,3660932	0,635011419	conserved Plasmodium protein, unknown function
PFIT_0925800	4600,606667	5000,046235	4630,510151	4078,314812	4800,326451	4354,4125	0,907107574	-0,140654	0,3667934	0,635445008	histone deacetylase
PFIT_0422100	551,8961932	562,8794564	704,9708611	569,8137455	557,3878248	637,3923	1,143534672	0,1935001	0,366509	0,635445008	probable protein, unknown function
PFIT_1001700	17916,75802	19842,09964	24305,14877	19225,19475	18879,42883	21765,172	1,152851178	0,2052063	0,3667648	0,635445008	PF70 protein
PFIT_0804400	8,830339092	8,383311052	14,25030278	14,71350047	8,606825072	14,481902	1,682606711	0,750698	0,3667119	0,635445008	conserved Plasmodium protein, unknown function
PFIT_0534500	2755,065797	2451,519675	2901,193995	2870,470183	2603,292736	2885,8321	1,108531534	0,1486498	0,3669372	0,635499161	conserved Plasmodium membrane protein, unknown function
PFIT_0901700	1,471723182	3,592847594	0	0,668795476	2,532285388	0,3343977	0,132053733	-2,920803	0,3670913	0,635571167	rifin (RIF)
PFIT_1124400	9257,138814	9903,085584	8782,377778	8532,492682	9580,112199	8657,4352	0,903688292	-0,146103	0,3673297	0,635594119	conserved Plasmodium protein, unknown function
PFIT_0516200	462,1210791	491,0225045	513,8491532	567,8073591	476,5717918	540,82826	1,134830608	0,182477	0,3672359	0,635594119	cation-transporting ATPase 1
PFIT_0530200	213,3998614	168,8638369	217,9458072	220,7025071	191,1318491	219,32416	1,147501885	0,1984965	0,3680581	0,63665932	RAP protein, putative
PFIT_1359500	78,00132864	102,9949644	114,0024222	109,0136626	90,4981465	111,50804	1,232158301	0,3011876	0,3684967	0,637222727	conserved Plasmodium protein, unknown function
PFIT_0421300	100,0771764	107,7854278	105,6198912	141,7846409	103,9313021	123,70227	1,190231081	0,2512417	0,3686851	0,637353331	clathrin assembly protein, putative
PFIT_0920200	76,52960546	80,24026293	99,75211946	92,96257115	78,38493419	96,357345	1,229283998	0,2978183	0,3688052	0,637365871	chaperone, putative
PFIT_0303600	428,2714459	383,2370767	255,6671969	397,2645127	405,7542613	326,46585	0,804590083	-0,313674	0,3694057	0,638159988	dihydrolipoamide acyltransferase, putative
PFIT_0504000	1058,168968	1002,404479	866,7537103	953,7023487	1030,286723	910,22803	0,8834706	-0,178746	0,3696038	0,638159988	cation transporting P-ATPase
PFIT_1357500	185,4371209	165,2709893	200,342492	205,9890066	175,3540551	203,16575	1,158603085	0,2123864	0,369543	0,638159988	conserved Plasmodium protein, unknown function
PFIT_0708100	128,0399168	158,0852941	110,6494098	131,7527088	143,0626055	121,20106	0,847188955	-0,239244	0,3699872	0,638222715	glutaredoxin-like protein
PFIT_0606300	924,2421583	1010,78779	890,2247972	845,3574816	967,514974	867,79114	0,896927864	-0,156936	0,3699818	0,638222715	G-protein associated signal transduction protein, putative
PFIT_0308100	5367,374445	6055,145811	5622,163573	4791,250789	5711,260128	5206,7072	0,911656458	-0,133438	0,3703184	0,638222715	TCP-1/cpn60 chaperonin family, putative
PFIT_0604700	2357,700537	1964,090018	2560,024982	2235,114481	2160,895278	2397,5697	1,10952611	0,1499436	0,3701202	0,638222715	DNA repair-like protein, putative
PFIT_0917600	347,3266709	289,8230392	316,0214205	411,9780132	318,5748551	363,99972	1,142587718	0,1923049	0,3703133	0,638222715	conserved Plasmodium protein, unknown function
PFIT_1457300	14,71723182	14,37139038	13,41204968	48,15327427	14,5443111	30,782662	2,116474391	1,081663	0,3700102	0,638222715	serine hydroxymethyltransferase, putative
PFIT_1441400	45,62341864	39,52132353	43,58916145	63,53557021	42,57237109	53,562366	1,258148524	0,3313022	0,3708767	0,638989929	delta-aminolevulinic acid dehydratase
PFIT_0212000	4805,176189	4645,551939	4810,734568	3525,889749	4725,364064	4168,3122	0,8821145	-0,180962	0,3710461	0,6390867	secreted protein altered thrombospondin repeat protein
PFIT_1222700	961,0352378	798,8097817	871,7832289	592,5527917	879,9225097	732,16801	0,832082373	-0,265202	0,3715132	0,63962725	conserved Plasmodium protein, unknown function
PFIT_0929300	91,24683728	85,03072639	88,016576	121,7207766	88,13878183	104,86868	1,189813089	0,250735	0,3716292	0,63962725	conserved Plasmodium protein, unknown function
PFIT_1005900	13,24550864	5,988079323	12,57379657	17,38868237	9,61679398	14,981239	1,557820569	0,6395291	0,3716998	0,63962725	conserved Plasmodium protein, unknown function
PFIT_0621300	10,30206227	11,97615865	15,08855589	20,06386428	11,13911046	17,57621	1,577882735	0,65799	0,3719371	0,639721001	conserved Apicomplexan protein, unknown function
PFIT_0521400	1,471723182	5,988079323	5,867771733	10,70072761	3,729901252	8,2842497	2,221037264	1,1512336	0,3719809	0,639721001	conserved Plasmodium protein, unknown function, putative
PFIT_1356700	11,77378546	11,97615865	17,6033152	19,3950688	11,87497205	18,499192	1,557830361	0,6395381	0,3722096	0,639919381	conserved Plasmodium protein, unknown function
PFIT_0536500	10,30206227	4,790463458	0,838253105	5,350363807	7,546262866	3,0943085	0,410045146	-1,286145	0,3728615	0,640845017	rifin
PFIT_0107700	188,3805673	158,0852941	166,8123678	128,4087314	173,2329307	147,61055	0,852092896	-0,230917	0,3737095	0,641716472	mRNA cleavage factor-like protein, putative
PFIT_1323900	676,9926637	746,1146836	834,0618392	782,4907068	711,5536737	808,27627	1,135931558	0,1838759	0,3736603	0,641716472	SNARE protein, putative
PFIT_0301400	5,886892728	5,988079323	6,706024838	14,71350047	5,937486025	10,709763	1,803753745	0,8510024	0,3735687	0,641716472	Plasmodium exported protein, unknown function
PFIT_0504400	426,7997228	377,2489973	313,5066612	380,5446258	402,0243601	347,02564	0,863195562	-0,212241	0,3747717	0,642638309	ATP-dependent helicase, putative
PFIT_1146100	8538,937902	8336,604033	8112,613548	7131,36616	8437,770968	7621,9899	0,903317936	-0,146694	0,3748154	0,642638309	dynamin-like protein
PFIT_0918800	3424,699844	3415,600446	4251,619747	3428,914405	3420,150145	3840,2671	1,122835815	0,167147	0,3747393	0,642638309	conserved Plasmodium protein, unknown function
PFIT_0205100	198,6826296	152,0972148	187,7686955	212,6769613	175,3899222	200,22283	1,141586848	0,1910406	0,3745228	0,642638309	conserved Plasmodium protein, unknown function
PFIT_0928500	67,69926637	56,28794564	72,92802011	78,91786616	61,993606	75,922943	1,2246899	0,2924165	0,3744669	0,642638309	conserved Plasmodium protein, unknown function
PFIT_0727400	16,188955	10,77854278	7,544277943	8,025545711	13,48374889	7,7849118	0,577355147	-0,792469	0,3758184	0,64280707	conserved Plasmodium protein, unknown function
PFIT_1011400	1193,567501	1021,566332	1086,376024	871,4405051	1107,566917	978,90826	0,883836678	-0,178148	0,3755725	0,64280707	heme oxygenase
PFIT_0931400	707,8988505	805,9954769	661,3816996	692,2033176	756,9471637	676,79251	0,894107992	-0,161479	0,3758247	0,64280707	elongation factor Tu, putative
PFIT_1027400	930,129051	1122,166065	950,5790208	905,5490744	1026,147558	928,06405	0,904415783	-0,144942	0,3757863	0,64280707	methyltransferase, putative
PFIT_0715800	3271,640633	3395,240976	3382,351278	2679,194677	3333,440805	3030,773	0,909202579	-0,137326	0,3755822	0,64280707	serine--tRNA ligase, putative
PFIT_0918500	138,3419791	98,2045009	139,1500154	136,4342771	118,27324	137,79215	1,165032312	0,22037	0,3757779	0,64280707	conserved Plasmodium protein, unknown function
PFIT_0700900	2490,155624	2655,114372	3831,654942	2588,907287	2572,634998	3210,2811	1,247857204	0,3194529	0,3756571	0,64280707	Plasmodium exported protein (hyp12), unknown function
PFIT_1315900	107,4357923	79,04264706	150,8855589	94,96895758	93,23921967	122,92726	1,318407197	0,398796	0,375639	0,64280707	conserved Plasmodium protein, unknown function
PFIT_1346200	57,3972041	52,69509804	43,58916145	42,13411498	55,04615107	42,861638	0,77864914	-0,360955	0,3760855	0,642899463	G-beta repeat protein, putative
PFIT_1027700	1293,644677	1250,310963	1804,758934	1270,042609	1271,97782	1537,4008	1,208669481	0,2734198	0,3761064	0,642899463	nucleolar preribosomal assembly protein, putative
PFIT_1222600	64,75582001	62,27602496	54,48645181	46,81568331	63,51592248	50,651068	0,797454647	-0,326526	0,3762499	0,642950095	conserved Plasmodium protein, unknown function
PFIT_0824300	7395,408989	7427,613592	7598,764394	5375,10924	7411,511291	6486,9368	0,87525156	-0,19223	0,3763805	0,642978632	conserved Plasmodium protein, unknown function
PFIT_1135800	5465,979898	6256,345277	6621,361274	6529,450231	5861,162587	6575,4058	1,121860323	0,1658931	0,3765368	0,643051048	protein phosphatase, putative
PFIT_0216500	509,216221	501,8030473	383,919922	497,5838341	505,5086341	440,75188	0,871897824	-0,197769	0,3766782	0,643098019	ATP synthase F1, alpha subunit
PFIT_1105500	504,8010514	423,9560161	319,3744329	447,4241734	464,3785337	383,3993	0,825618058	-0,276454	0,3771569	0,643526077	centrin-1
PFIT_0834000	89,7751141	100,5997326	97,23736015	130,4151178	95,18742336	113,82624	1,195811747	0,2579903	0,3771012	0,643526077	surface-associated interspersed protein 8.1 (SURFIN 8.1) (SURF8.1)
PFIT_1441600	548,9527469	481,4415776	502,9518628	685,5153628	515,1971622	594,23361	1,153410105	0,2059056	0,3776431	0,643966502	conserved Plasmodium protein, unknown function
PFIT_0101500	33,84963318	50,29986631	49,45693318	60,19159283	42,07474975	54,824263	1,303020537	0,3818598	0,3775749	0,643966502	vacuolar protein sorting-associated protein VTA1, putative
PFIT_bin10000	29,43446364	14,37139038	14,25030278	13,37590952	21,90292701	13,813106	0,630651152	-0,665086	0,3784248	0,645104645	rifin
PFIT_1246700	206,0412455	156,8876783	165,9741147	141,1158454	181,4644619	153,54498	0,846143529	-0,241026	0,378789	0,645366471	conserved Plasmodium protein, unknown function
PFIT_1103200	1321,607417	1293,425134	1525,620651	1403,132908	1307,516276	1464,3768	1,119968299	0,1634579	0,378807	0,645366471	flavoprotein, putative
PFIT_1348700	7030,42164	6473,113748	5826,697331	6233,173836	6751,767694	6029,9356	0,893089907	-0,163123	0,3793524	0,64585014	conserved Plasmodium protein, unknown function
PFIT_0703800	292,8729132	291,0206551	317,6979267	347,7736475	291,9467842	332,73579	1,139713829	0,1886716	0,37938	0,64585014	peptide chain release factor, putative
PFIT_0936100	9087,890649	10300,69405	12967,77553	9873,427611	9694,29235	11420,602	1,178074805	0,2364311	0,3794341	0,64585014	gametocytogenesis-implicated protein
PFIT_0921000	747,6353764	834,7382576	943,8729959	852,7142318	791,186817	898,29361	1,13537485	0,1831687	0,3799012	0,646450254	U4/U6 snRNA-associated-splicing factor, putative
PFIT_1001100	36966,74288	39715,3373	39734,03542	24619,69906	38341,04009	32176,867	0,839227813	-0,252866	0,3801058	0,646603651	early transcribed membrane protein 10.1
PFIT_1135700.2	14,71723182	14,37139038	7,544277943	10,03193214	14,5443111	8,788105	0,604229721	-0,726831	0,3806731	0,647081931	conserved Plasmodium protein, unknown function
PFIT_1131300	584,2741032	635,9340241	809,7524992	616,6294288	610,1040637	713,19096	1,168966094	0,2252331	0,3805604	0,647081931	conserved Plasmodium protein, unknown function
PFIT_0927600	7,35861591	3,592847594	9,220784152	10,70072761	5,475731752	9,9607559	1,819073018	0,8632035	0,3807308	0,647081931	dynein light chain, putative
PFIT_1436200	179,5502282	167,666221	167,6506209	245,4479397	173,6082246	206,54928	1,189743635	0,2506507	0,3810378	0,647408744	cytochrome c oxidase assembly protein, putative
PFIT_0922900	3493,870834	3512,607331	2933,047613	3337,95822	3503,239082	3135,5029	0,895029669	-0,159993	0,3813788	0,647598473	conserved Plasmodium protein, unknown function
PFIT_0611600	1187,680608	1180,849242	1085,537771	1035,295397	1184,264925	1060,4166	0,89542176	-0,159361	0,3813609	0,647598473	PP-loop family protein, putative
PFIT_1304400	52,98203455	50,29986631	32,69187108	46,14688784	51,64095043	39,419379	0,763335669	-0,38961	0,3815626	0,64771574	transcription factor with AP2 domain(s)
PFIT_1013800	1011,073826	929,3499109	625,3368161	959,7215079	970,2118685	792,52916	0,816861953	-0,291836	0,3818782	0,648056622	conserved protein, unknown function
PFIT_1354200	2491,627347	2227,565508	1945,585456	2237,789662	2359,596428	2091,6876	0,886459877	-0,173873	0,382272	0,648529964	DNA replication licensing factor MCM6 (MCM6)
PFIT_1455200	722,6160823	773,6598485	729,2802011	617,2982243	748,1379654	673,28921	0,899953276	-0,152078	0,3824318	0,648606143	RNA binding protein, putative
PFIT_0803500	207,5129687	234,7327095	217,9458072	310,3211008	221,1228391	264,13345	1,194510052	0,256419	0,3827617	0,648970738	Plasmodium exported protein, unknown function, pseudogene
PFIT_0407500.2	141,2854255	94,6116533	141,6647747	133,0902997	117,9485394	137,37754	1,164724361	0,2199886	0,3832411	0,649393467	peptidase, M22 family, putative
PFIT_0304400	104,4923459	85,03072639	90,53133531	152,4853685	94,76153615	121,50835	1,282253928	0,358682	0,3832263	0,649393467	1-cys-glutaredoxin-like protein-1
PFIT_0415600	17,66067818	11,97615865	20,95632762	22,07025071	14,81841841	21,513289	1,45179388	0,5378366	0,3836957	0,64977378	conserved Plasmodium protein, unknown function
PFIT_1364200	2,943446364	3,592847594	5,867771733	8,694341187	3,268146979	7,2810565	2,227885253	1,1556749	0,3836814	0,64977378	conserved Plasmodium protein, unknown function
PFIT_1125750	1158,246144	894,6190508	641,2636251	967,7470537	1026,432598	804,50534	0,783787792	-0,351465	0,384135	0,650322727	hypothetical protein
PFIT_1404800	332,6094391	304,1944296	338,6542543	379,8758303	318,4019344	359,26504	1,128338127	0,1741995	0,3851378	0,651820872	nuclear formin-like protein
PFIT_1419100	248,7212177	208,3851604	269,0792466	252,8046899	228,5531891	260,94197	1,141712217	0,191199	0,3852508	0,651820872	liver specific protein 1, putative
PFIT_1418800	1754,294033	1590,433868	1166,848322	1651,25603	1672,363951	1409,0522	0,842551154	-0,247164	0,3863568	0,652909449	DNA replication licensing factor MCM2 (MCM2)
PFIT_0516800	5780,928659	5699,4539	5044,607184	5277,4651	5740,191279	5161,0361	0,899105255	-0,153438	0,3863489	0,652909449	ubiquitin carboxyl-terminal hydrolase 2, putative
PFIT_1427600	816,806366	887,4333557	1045,301622	886,8228011	852,1198608	966,06221	1,133716342	0,1810597	0,3860114	0,652909449	conserved Plasmodium protein, unknown function
PFIT_0106300	179,5502282	178,4447638	172,6801396	254,1422808	178,997496	213,41121	1,192258076	0,2536966	0,3862885	0,652909449	DNA repair exonuclease Mre11, putative
PFIT_1446500	214,8715846	219,1637032	268,2409935	235,4160075	217,0176439	251,8285	1,160405652	0,2146292	0,3866639	0,653232879	mitochondrial ribosomal protein S29 precursor, putative
PFIT_1325600	900,6945874	979,6497772	1069,610962	1047,333715	940,1721823	1058,4723	1,125828182	0,1709867	0,386818	0,653297823	translation initiation factor EIF-2B gamma subunit, putative
PFIT_1306700	746,1636532	740,1266043	932,1374525	749,050933	743,1451288	840,59419	1,131130596	0,1777655	0,3875568	0,654349844	conserved Plasmodium protein, unknown function
PFIT_1447100	7173,178789	8358,161119	9522,55527	8016,182574	7765,669954	8769,3689	1,129248214	0,1753626	0,3878513	0,654651216	DEAD/DEAH box ATP-dependent RNA helicase, putative
PFIT_1345100	132,4550864	123,3544341	126,5762188	169,8740509	127,9047602	148,22513	1,158871137	0,2127202	0,3880299	0,654734325	conserved Plasmodium protein, unknown function
PFIT_0720900	23,54757091	21,55708556	21,79458072	38,7901376	22,55232824	30,292359	1,343203187	0,4256776	0,3881324	0,654734325	conserved Plasmodium protein, unknown function
PFIT_1311300	757,9374387	562,8794564	564,1443395	557,1066314	660,4084475	560,62549	0,848907199	-0,236321	0,388734	0,655357441	conserved Plasmodium protein, unknown function
PFIT_0410900	97,13373001	105,3901961	111,4876629	129,7463223	101,261963	120,61699	1,191138202	0,2523408	0,388723	0,655357441	phosphopantothenoylcysteine synthetase, putative
PFIT_0100300	7,35861591	5,988079323	1,676506209	4,012772856	6,673347616	2,8446395	0,426268748	-1,230165	0,389372	0,655944918	stevor
PFIT_0317200	2379,776385	2214,391734	1254,864898	2285,274141	2297,084059	1770,0695	0,770572375	-0,375998	0,3893856	0,655944918	cdc2-related protein kinase 4
PFIT_0412900	47,09514182	63,47364082	74,60452632	66,21075212	55,28439132	70,407639	1,273553666	0,3488598	0,389431	0,655944918	Rab5-interacting protein, putative
PFIT_1251000	1003,71521	1207,196792	783,7666529	1092,143012	1105,456001	937,95483	0,848477761	-0,237051	0,3900672	0,656037956	DNA repair protein rhp16, putative
PFIT_0312700	10312,36434	11222,85827	11038,11688	8150,610465	10767,6113	9594,3637	0,891039192	-0,166439	0,3899026	0,656037956	60S ribosomal protein L26, putative
PFIT_0206200.2	831,5235978	794,0193182	926,2696807	891,5043694	812,771458	908,88703	1,118256573	0,1612512	0,390048	0,656037956	merozoite surface protein 5
PFIT_0315100	1982,411126	2377,267491	2590,202094	2332,75862	2179,839309	2461,4804	1,129202665	0,1753044	0,3899946	0,656037956	translation initiation factor 4E
PFIT_1464500	828,5801514	913,7809047	1306,83659	875,453278	871,1805281	1091,1449	1,25249004	0,3247991	0,3898058	0,656037956	DNA-directed RNA polymerase III subunit RPC4, putative
PFIT_1442000	241,3626018	194,0137701	217,1075541	276,881327	217,688186	246,99444	1,13462492	0,1822155	0,3902724	0,656187651	major facilitator superfamily, putative
PFIT_bin03500	1,471723182	10,77854278	1,676506209	2,006386428	6,125132982	1,8414463	0,30063777	-1,733902	0,3907135	0,65657867	rifin (RIF)
PFIT_1100600	8,830339092	5,988079323	7,544277943	16,7198869	7,409209207	12,132082	1,637432833	0,7114357	0,3907376	0,65657867	rifin, pseudogene (RIF)
PFIT_1305300	17,66067818	11,97615865	16,76506209	25,41422808	14,81841841	21,089645	1,423204859	0,5091433	0,391059	0,656808733	conserved Plasmodium protein, unknown function
PFIT_1103000	17,66067818	14,37139038	23,47108693	22,73904618	16,01603428	23,105067	1,442620948	0,5286923	0,3911071	0,656808733	DnaJ protein, putative
PFIT_1365800	356,15701	440,7226382	342,0072667	367,8375118	398,4398241	354,92239	0,890780408	-0,166858	0,3912337	0,656825944	phosphatidylserine synthase I, putative
PFIT_1244900	2877,218821	2428,764973	2637,982521	1920,780607	2652,991897	2279,3816	0,859173964	-0,218978	0,3914453	0,656938848	conserved Plasmodium protein, unknown function
PFIT_1204600	1149,415805	1027,554412	1328,631171	1102,174944	1088,485108	1215,4031	1,116600538	0,1591132	0,3915337	0,656938848	conserved Plasmodium protein, unknown function
PFIT_0732500	23123,71463	30288,90283	27337,11025	16083,8624	26706,30873	21710,486	0,812934747	-0,298789	0,3918561	0,657089323	histidine-rich protein III
PFIT_0826700	1424,62804	1419,1748	1285,880263	1267,367427	1421,90142	1276,6238	0,897828659	-0,155488	0,3918442	0,657089323	DnaJ protein, putative
PFIT_1209100.2	615,1802901	590,4246212	588,4536795	488,8894929	602,8024556	538,67159	0,89361213	-0,162279	0,3926521	0,658228482	cGMP-specific phosphodiesterase
PFIT_1206800	445,9321241	325,7515152	386,4346813	521,6604712	385,8418196	454,04758	1,176771291	0,234834	0,3933172	0,659147858	conserved Plasmodium protein, unknown function
PFIT_1112200	329,6659928	275,4516489	290,0355742	238,0911894	302,5588208	264,06338	0,872767091	-0,196331	0,3935187	0,659289827	conserved Plasmodium protein, unknown function
PFIT_0730400	73212,34141	74018,64851	71505,50459	62547,75929	73615,49496	67026,632	0,910496248	-0,135275	0,3942507	0,660320208	erythrocyte binding antigen-175
PFIT_0709500	7137,857432	8430,018071	7718,634588	6585,629051	7783,937752	7152,1318	0,918832093	-0,122127	0,3952722	0,661638485	60S ribosomal protein L34a, putative
PFIT_1336700	4830,195483	4915,015508	5200,522262	5573,741496	4872,605496	5387,1319	1,10559574	0,144824	0,3952645	0,661638485	conserved Plasmodium protein, unknown function
PFIT_0514300	690,2381723	607,1912433	588,4536795	559,7818133	648,7147078	574,11775	0,885008062	-0,176237	0,3956912	0,662143593	conserved Plasmodium protein, unknown function
PFIT_0719400	724,0878055	791,6240865	639,5871189	714,9423638	757,855946	677,26474	0,89365894	-0,162204	0,3959626	0,662401417	conserved Plasmodium protein, unknown function
PFIT_1234700	92,71856046	107,7854278	104,7816381	56,84761545	100,2519941	80,814627	0,806114905	-0,310943	0,3962528	0,662690503	PIH1 domain-containing protein, putative
PFIT_1305200	384,1197505	314,9729724	335,3012419	276,881327	349,5463614	306,09128	0,875681507	-0,191522	0,3966585	0,663030668	conserved Plasmodium protein, unknown function
PFIT_1316800	244,3060482	232,3374777	269,9174997	274,2061451	238,321763	272,06182	1,141573556	0,1910238	0,396691	0,663030668	conserved Plasmodium protein, unknown function
PFIT_1319600	164,8329964	154,4924465	171,0036334	196,6258699	159,6627215	183,81475	1,151269062	0,203225	0,3976108	0,664371233	type I signal peptidase
PFIT_bin02300	450,3472937	491,0225045	411,5822744	426,0227182	470,6848991	418,8025	0,889772536	-0,168492	0,3979065	0,664638936	rifin (RIF)
PFIT_0423700	25,01929409	23,95231729	21,79458072	61,52918378	24,48580569	41,661882	1,701470753	0,7667824	0,3980064	0,664638936	Plasmodium exported protein (hyp10), unknown function
PFIT_0821800	63936,07019	76489,33004	69386,40074	59313,46437	70212,70012	64349,933	0,916499899	-0,125793	0,3982737	0,664888538	heat shock protein 70
PFIT_1147700	1,471723182	3,592847594	7,544277943	5,350363807	2,532285388	6,4473209	2,546048287	1,3482598	0,3991207	0,666105631	conserved Plasmodium protein, unknown function
PFIT_0816200	3464,43637	3967,701359	2405,786411	3725,859596	3716,068865	3065,823	0,825017812	-0,277503	0,3995955	0,666173353	conserved Plasmodium protein, unknown function
PFIT_0921300	2298,83161	2697,030927	2835,810253	2764,800497	2497,931269	2800,3054	1,121049811	0,1648504	0,3996332	0,666173353	conserved Plasmodium membrane protein, unknown function
PFIT_0315800	498,9141587	534,1366756	595,1597044	573,1577229	516,5254171	584,15871	1,130938951	0,1775211	0,3994404	0,666173353	zinc finger protein, putative
PFIT_1458100	356,15701	459,884492	565,8208457	428,0291046	408,020751	496,92498	1,217891428	0,2843855	0,3994269	0,666173353	conserved protein, unknown function
PFIT_0522200	2,943446364	2,395231729	5,867771733	6,687954759	2,669339047	6,2778632	2,351841837	1,233791	0,4005632	0,667526444	conserved Plasmodium protein, unknown function
PFIT_1311400	129,51164	88,62357398	91,36958842	85,60582092	109,067607	88,487705	0,811310591	-0,301674	0,4014947	0,668684148	conserved Plasmodium protein, unknown function
PF_apicoplast_g	0	0	1,676506209	2,675181904	0	2,1758441	#DIV/0!	#DIV/0!	0,4014014	0,668684148	apicoplast ribosomal protein S17
PFIT_1476800	25,01929409	55,09032977	53,6481987	58,1852064	40,05481193	55,916703	1,396004621	0,4813037	0,4020376	0,669390852	tubulin, putative
PFIT_0535800	17949,13593	22219,36714	19487,70818	17180,01819	20084,25153	18333,863	0,912847718	-0,131554	0,4025518	0,670049432	early transcribed membrane protein 5
PFIT_0821600	4984,726417	5456,337879	5954,950056	5674,729613	5220,532148	5814,8398	1,113840442	0,1555426	0,4037866	0,671906651	DNA helicase, putative
PFIT_0215700	295,8163596	311,3801248	290,0355742	448,7617643	303,5982422	369,39867	1,216735205	0,2830152	0,403907	0,671908975	conserved Plasmodium protein, unknown function
PFIT_1361200	1165,60476	1456,300891	1242,291101	1157,684969	1310,952826	1199,988	0,915355619	-0,127596	0,4041144	0,672055968	RuvB-like helicase (RUVB3)
PFIT_1343400	7045,138872	7342,582866	7194,726398	5971,006009	7193,860869	6582,8662	0,915067211	-0,12805	0,404591	0,672650421	heat shock protein 110, putative (HSP110)
PFIT_0205800	4805,176189	5276,695499	3723,520291	5064,119344	5040,935844	4393,8198	0,8716278	-0,198216	0,4051391	0,67336337	conserved Plasmodium protein, unknown function
PFIT_1463800	41,20824909	50,29986631	33,53012419	38,12134213	45,7540577	35,825733	0,783006687	-0,352903	0,4054284	0,673645901	conserved Plasmodium membrane protein, unknown function
PFIT_0717200	183,9653977	194,0137701	173,5183927	155,8293459	188,9895839	164,67387	0,871338335	-0,198695	0,4060642	0,674400273	conserved Plasmodium protein, unknown function
PFIT_1424100	952,2048987	875,457197	872,621482	766,4396154	913,8310479	819,53055	0,896807512	-0,15713	0,4061213	0,674400273	ankyrin, putative
PFIT_0936200	3115,637976	3166,496346	3620,415159	3356,015698	3141,067161	3488,2154	1,110519212	0,1512344	0,406467	0,674775835	Plasmodium exported protein, unknown function
PFIT_0508000	1926,485645	1664,686052	1631,240542	1581,032505	1795,585848	1606,1365	0,894491636	-0,16086	0,4072353	0,675852645	6-cysteine protein
PFIT_0903700	5342,35515	5766,520388	5776,402145	3873,663397	5554,437769	4825,0328	0,868680679	-0,203102	0,407986	0,676899433	conserved Plasmodium protein, unknown function
PFIT_0822300	29,43446364	28,74278075	23,47108693	19,3950688	29,08862219	21,433078	0,736819975	-0,440616	0,408223	0,677065579	perforin like protein 4
PFIT_1344400	590,160996	444,3154858	481,1572821	397,2645127	517,2382409	439,2109	0,849146221	-0,235915	0,4083295	0,677065579	rhomboid protease ROM6, putative
PFIT_1356900	3270,16891	4160,517514	4074,748342	4249,526454	3715,343212	4162,1374	1,120256504	0,1638291	0,4084459	0,677065579	TCP-1/cpn60 chaperonin family, putative
PFIT_0511500	32,37791	29,94039661	29,33885867	50,82845617	31,15915331	40,083657	1,286416772	0,3633581	0,4088045	0,677461147	conserved Plasmodium protein, unknown function
PFIT_1417300	175,1350587	191,6185383	199,5042389	223,377689	183,3767985	211,44096	1,153040983	0,2054438	0,40899	0,677569727	conserved protein, unknown function
PFIT_1251400	17361,91838	16172,60464	16147,26956	14290,82173	16767,26151	15219,046	0,907664358	-0,139769	0,4100427	0,679114455	coronin
Pf_M76611004(	57,3972041	67,06648842	53,6481987	48,15327427	62,23184626	50,900736	0,817921041	-0,289967	0,4111934	0,679823711	unspecified product
PFIT_0831400	192,7957368	156,8876783	129,9292312	164,5236871	174,8417075	147,22646	0,842055716	-0,248012	0,4109811	0,679823711	translation initiation factor EIF-2b alpha subunit, putative
PFIT_0606400	351,7418405	377,2489973	310,1536488	335,0665334	364,4954189	322,61009	0,88508682	-0,176109	0,4108827	0,679823711	para-hydroxybenzoate--polyprenyltransferase, putative
PFIT_1226400	844,7691064	959,2903075	1023,507041	1002,524418	902,029707	1013,0157	1,123040319	0,1674097	0,4111795	0,679823711	conserved protein, unknown function
PFIT_0420300	463,5928023	476,6511141	539,8349995	519,6540848	470,1219582	529,74454	1,126823653	0,1722618	0,4107567	0,679823711	transmembrane emp24 domain-containing protein, putative
PFIT_1140200.2	1,471723182	2,395231729	8,382531047	2,675181904	1,933477456	5,5288565	2,859540182	1,5157832	0,4109256	0,679823711	AAA family ATPase, putative
PFIT_1428200	195,7391832	152,0972148	160,106343	137,1030726	173,918199	148,60471	0,85445174	-0,226929	0,4118407	0,680457164	selenoprotein
PFIT_1143900	401,7804287	297,0087344	374,6991378	401,946081	349,3945815	388,32261	1,11141566	0,1523985	0,4119381	0,680457164	DnaJ protein, putative
PFIT_1361400	3336,396453	3590,452362	3394,086821	4335,132275	3463,424408	3864,6095	1,115834819	0,1581235	0,4118687	0,680457164	calpain
PFIT_1229000	6073,801572	4535,371279	4974,193924	3961,944399	5304,586426	4468,0692	0,842303019	-0,247589	0,4120878	0,680505316	conserved Plasmodium protein, unknown function
PFIT_0503400	3651,345214	3612,009448	4097,381176	3929,842216	3631,677331	4013,6117	1,105167483	0,144265	0,4125789	0,680983836	Ser/Arg-rich splicing factor, putative
PFIT_0709400	67,69926637	46,70701872	72,92802011	65,54195664	57,20314254	69,234988	1,210335399	0,2754069	0,4126818	0,680983836	conserved Plasmodium protein, unknown function
PF_apicoplast_g	63,28409682	98,2045009	85,50181668	123,727163	80,74429886	104,61449	1,295626953	0,3736504	0,4127394	0,680983836	RNA polymerase beta subunit, putative
PFIT_1427300	132,4550864	114,971123	93,88434773	112,35764	123,7131047	103,12099	0,833549478	-0,26266	0,4130565	0,681108889	dynein-associated protein, putative
PFIT_1012900	685,8230028	639,5268717	806,3994868	677,4898171	662,6749372	741,94465	1,119620813

PFIT_0535000	150,1157646	138,9234403	138,3117623	111,6888445	144,5196024	125,0003	0,864936668	-0,209334	0,4326274	0,698288878	CPW-WPC family protein
PFIT_1404300	114,7944082	86,22834225	96,39910704	72,2299114	100,5113752	84,314509	0,838855394	-0,253506	0,4331569	0,698396653	conserved Plasmodium protein, unknown function
PFIT_1446400	2285,586102	2717,390397	2995,078343	2604,289583	2501,488249	2799,684	1,119207322	0,1624773	0,4330321	0,698396653	RNA helicase, putative
PFIT_0621600	231,0605396	279,0444964	346,1985323	259,4926447	255,052518	302,84559	1,187385213	0,2477881	0,433189	0,698396653	conserved Plasmodium membrane protein, unknown function
PFIT_1104700	13,24550864	7,185695188	15,92680899	14,71350047	10,21560191	15,320155	1,499682041	0,5846567	0,4330399	0,698396653	conserved Plasmodium protein, unknown function
PFIT_1218100	2178,150309	2178,463258	2641,335533	2192,31157	2178,306783	2416,8236	1,109496408	0,149905	0,4334499	0,698617757	triose or hexose phosphate/phosphate translocator, putative
PFIT_1143000	1295,1164	1234,741956	922,0784152	1275,392973	1264,929178	1098,7357	0,868614396	-0,203212	0,4337784	0,698947721	conserved Plasmodium membrane protein, unknown function
PFIT_1329600	232,5322627	201,1994653	186,0921893	189,9379152	216,865864	188,01505	0,866964716	-0,205955	0,4339738	0,699063029	DnaJ protein, putative
PFIT_1461300	2232,604067	2410,800735	2972,445509	2308,681983	2321,702401	2640,5637	1,137339456	0,1856629	0,4345001	0,699711209	conserved Plasmodium protein, unknown function
PFIT_1300400	4341,583387	4077,882019	4567,641168	1895,366379	4209,732703	3231,5038	0,767626831	-0,381523	0,4348071	0,700006017	glycophorin binding protein
PFIT_1307300	61,81237364	80,24026293	58,67771733	60,19159283	71,02631828	59,434655	0,836797634	-0,257049	0,4357303	0,700610267	CCAAT-binding transcription factor, putative
PFIT_0312000	163,3612732	183,2352273	130,7674843	169,2052554	173,2982502	149,98637	0,865481156	-0,208426	0,4359026	0,700610267	RING zinc finger protein, putative
PFIT_1309400	635,7844146	565,2746881	531,4524684	538,3803581	600,5295513	534,91641	0,8907412	-0,166922	0,4358265	0,700610267	conserved Plasmodium protein, unknown function
PFIT_1247600	544,5375773	603,5983958	611,0865134	670,1330669	574,0679865	640,60979	1,115912758	0,1582242	0,435927	0,700610267	FK506-binding protein (FKBP)-type peptidyl-prolyl isomerase
PFIT_0835200.2	11,77378546	17,96423797	28,50060556	16,05109142	14,86901171	22,275848	1,498139145	0,5831716	0,4357095	0,700610267	Plasmodium exported protein (PHISTa-like), unknown function
PFIT_0811800	5,886892728	2,395231729	6,706024838	8,694341187	4,141062228	7,700183	1,85947049	0,8948919	0,4358306	0,700610267	unspecified product
PFIT_0602700	1511,459708	1352,108311	999,1977008	1431,222318	1431,784009	1215,21	0,848738358	-0,236608	0,4362414	0,700916101	conserved Plasmodium protein, unknown function
PFIT_0814500	7130,653169	6505,449376	6032,907595	6445,182001	6918,051273	6239,0448	0,901850037	-0,149041	0,4367325	0,701505451	conserved Plasmodium protein, unknown function
PFIT_1454800	715,2574664	657,4911097	608,571754	619,3046107	686,374288	613,93818	0,89446559	-0,160902	0,4370238	0,701769652	RAP protein, putative
PFIT_1253800	0	1,197615865	0,838253105	6,687954759	0,598807932	3,7631039	6,284325456	2,6517579	0,4371456	0,701769652	Plasmodium exported protein, unknown function, pseudogene
PFIT_0819300	398,8369823	318,56582	305,9623832	321,6906239	358,7014011	313,8265	0,87489623	-0,192816	0,4376923	0,702048469	conserved Plasmodium protein, unknown function
PFIT_0311100	709,3705737	808,3907086	714,1916452	669,4642714	758,8806411	691,82796	0,911642649	-0,13346	0,4376522	0,702048469	valine--tRNA ligase, putative
PFIT_0902400	1925,013922	2125,76816	2274,180673	2217,057003	2025,391041	2245,6188	1,108733471	0,1489126	0,4376624	0,702048469	serine/threonine protein kinase, FIKK family
PFIT_0321700	422,3845532	416,7703209	347,0367854	397,2645127	419,577437	372,15065	0,886965352	-0,17305	0,4379163	0,702208271	ATP-dependent RNA helicase, putative
PFIT_0522100	41,20824909	68,26410428	41,07440213	47,48447879	54,73617669	44,27944	0,80896115	-0,305858	0,4381552	0,7022996	conserved Plasmodium protein, unknown function
PFIT_0717500	1230,36058	1501,810294	974,0501077	1368,355544	1366,085437	1171,2028	0,857342296	-0,222057	0,4382531	0,7022996	actin-related protein, putative (ARP6)
PFIT_1123300	497,4424355	559,2866088	456,8479421	494,9086522	528,3645221	475,8783	0,900662851	-0,150941	0,4383464	0,7022996	conserved Plasmodium protein, unknown function
PFIT_0423600	69,17098955	49,10225045	57,00121112	37,45254665	59,13662	47,226879	0,798606327	-0,324444	0,4386985	0,702664255	Plasmodium exported protein (PHISTa), unknown function
PFIT_1124300	5948,705101	6974,914795	6166,189838	5775,71773	6461,809948	5970,9538	0,924037357	-0,113977	0,4394504	0,703469575	translation elongation factor EF-1, subunit alpha, putative
PFIT_1005700	26,49101728	38,32370767	43,58916145	41,46531951	32,40736247	42,52724	1,312270954	0,3920656	0,4394203	0,703469575	CCR4-NOT transcription complex subunit 5, putative
PFIT_1002000.2	2362,115707	2565,293182	2693,307226	2747,411815	2463,704444	2720,3595	1,104174458	0,1429681	0,4398136	0,703851354	RNA binding protein, putative
PFIT_0519300	3985,426377	4778,4873	4989,282479	4757,14222	4381,956838	4873,2123	1,112108706	0,1532978	0,4410725	0,705666055	vacuolar ATP synthetase
PFIT_0809700	23126,65808	23458,89956	23492,04326	19429,84617	23292,77882	21460,945	0,921356137	-0,118169	0,4412357	0,705727165	vacuolar proton translocating ATPase subunit A, putative
PFIT_0612200	35,32135637	23,95231729	37,72138971	37,45254665	29,63683683	37,586968	1,268251683	0,3428411	0,4415286	0,705795749	conserved Plasmodium protein, unknown function
PFIT_0411300	32,37791	46,70701872	54,48645181	47,48447879	39,54246436	50,985465	1,289385124	0,3666832	0,4414129	0,705795749	erythrocyte membrane protein 1, PfEMP1
PFIT_1204800	309,0618682	298,2063503	367,993113	317,6778511	303,6341092	342,83548	1,129107276	0,1751826	0,4419335	0,706043229	conserved Plasmodium protein, unknown function
PFIT_0927100	91,24683728	89,82118984	102,2668788	110,3512535	90,53401356	106,30907	1,174244485	0,2317328	0,4418716	0,706043229	CS domain protein, putative
PFIT_1407700	67,69926637	57,4855615	63,70723596	84,26822997	62,59241393	73,987733	1,182055912	0,2412983	0,4429598	0,707482561	phosphatase, putative
PFIT_1304200	1346,626711	1087,435205	1077,993493	1096,824581	1217,030958	1087,409	0,893493324	-0,162471	0,4440812	0,708071843	conserved Plasmodium protein, unknown function
PFIT_1303300	2079,544856	1917,382999	1713,389346	1888,009629	1998,463928	1800,6995	0,901041776	-0,150334	0,4435538	0,708071843	DNA ligase I
PFIT_0531400	1437,873549	1657,500357	1238,938089	1550,936709	1547,686953	1394,9374	0,901304619	-0,149913	0,4437527	0,708071843	ubiquitin carboxyl-terminal hydrolase, putative
PFIT_1319200	7514,618567	8537,803499	9183,901015	8593,35307	8026,211033	8888,627	1,107449954	0,1472415	0,4439023	0,708071843	rhoptry protein 2, putative
PFIT_0816400	191,3240137	164,0733734	179,3861644	219,3649161	177,6986936	199,37554	1,121986528	0,1660554	0,4439869	0,708071843	conserved Plasmodium protein, unknown function
PFIT_0928800	45,62341864	34,73086007	38,55964282	58,85400188	40,17713936	48,706822	1,212301899	0,277749	0,4437768	0,708071843	conserved Plasmodium protein, unknown function
PFIT_0102600	899,2228642	667,0720366	760,295566	543,7307219	783,1474504	652,01314	0,832554768	-0,264383	0,4445352	0,70859559	conserved Plasmodium membrane protein, unknown function
PFIT_1308400	4415,169546	4201,236453	5161,962619	4321,756365	4308,202999	4741,8595	1,100658324	0,1383667	0,4447279	0,708702723	protein phosphatase 2c-like protein, putative
PFIT_1331000	9338,083589	10535,42676	9474,774843	8873,578374	9936,755175	9174,1766	0,923256782	-0,115196	0,4450556	0,70882475	glutamine--tRNA ligase, putative
PFIT_1236200	23,54757091	14,37139038	25,98584625	24,74543261	18,95948064	25,365639	1,337886829	0,4199561	0,4449312	0,70882475	conserved Plasmodium protein, unknown function
PFIT_1104300	17,66067818	29,94039661	20,11807451	16,05109142	23,8005374	18,084583	0,75983927	-0,396234	0,4458973	0,709965152	CPW-WPC family protein
PFIT_1447200	32,37791	45,50940285	48,61868007	50,82845617	38,94365643	49,723568	1,276807898	0,3525415	0,4461155	0,710112168	U5 spliceosomal RNA
PFIT_1367600	36,79307955	39,52132353	34,36837729	26,08302356	38,15720154	30,2257	0,792136195	-0,33618	0,4471797	0,710403395	mitochondrial import inner membrane translocase subunit Tim9, putative (TIM9)
PFIT_1362800	32,37791	44,31178699	29,33885867	31,43338737	38,3448485	30,386123	0,792443424	-0,33562	0,4478919	0,710403395	conserved Plasmodium membrane protein, unknown function
PFIT_0927500	114,7944082	83,83311052	76,28103253	87,61220735	99,31375936	81,94662	0,825128567	-0,277309	0,4479341	0,710403395	conserved Plasmodium protein, unknown function
PFIT_0306500	97,13373001	81,43787879	68,73675459	78,91786616	89,2858044	73,82731	0,826865042	-0,274276	0,447932	0,710403395	conserved Plasmodium protein, unknown function
PFIT_1110900	3589,532841	3541,350112	3348,821153	3166,077783	3565,441476	3257,4495	0,913617427	-0,130338	0,4476922	0,710403395	conserved Plasmodium protein, unknown function
PFIT_1411300	3395,265381	3497,038325	3452,764538	4069,620471	3446,151853	3761,1925	1,091418099	0,1262039	0,4468358	0,710403395	conserved Plasmodium protein, unknown function
PFIT_1223100	15539,92508	16213,32357	16341,74428	18339,04075	15876,62433	17340,393	1,092196436	0,1272324	0,4468531	0,710403395	phospholipid-transporting ATPase, putative
PFIT_0918600	8873,019064	7009,645655	9132,767576	8216,821217	7941,33236	8674,7944	1,092360073	0,1274485	0,447728	0,710403395	telomerase RNA
PFIT_0103300	2191,395818	2119,78008	2590,202094	2167,566137	2155,587949	2378,8841	1,103589448	0,1422036	0,4475392	0,710403395	novel putative transporter 1
PFIT_0917800	335,5528855	361,6799911	421,6413117	367,1687163	348,6164383	394,40501	1,131343708	0,1780373	0,4475239	0,710403395	XPA binding protein 1, putative
PFIT_0305700	73,5861591	71,05456774	72,08976701	100,3193214	72,32036342	86,204544	1,175724453	0,23355	0,4465517	0,710403395	P-loop containing nucleoside triphosphate hydrolase, putative
PFIT_0919700	10,30206227	25,14993316	19,27982141	36,78375118	17,72599771	28,031786	1,581393992	0,6611968	0,4478767	0,710403395	dolichyl-diphosphooligosaccharide-protein glycosyltransferase, putative
PFIT_1474100	4,415169546	4,790463458	10,05903726	7,356750235	4,602816502	8,7078937	1,891862024	0,9198069	0,4478188	0,710403395	conserved Plasmodium protein, unknown function
PFIT_0819800	47,09514182	44,31178699	60,35422354	52,8348426	45,70346441	56,594533	1,238298536	0,3083592	0,4489682	0,711843426	adenylate kinase 2
PFIT_0903100	4,415169546	4,790463458	2,514759314	1,337590952	4,602816502	1,9261751	0,418477498	-1,256778	0,449623	0,712119806	conserved Plasmodium protein, unknown function
PFIT_1205300	139,8137023	125,7496658	155,0768244	149,8101866	132,781684	152,44351	1,148076307	0,1992185	0,4493297	0,712119806	targeted glyoxalase II
PFIT_1471100	14,71723182	23,95231729	19,27982141	35,44616022	19,33477456	27,362991	1,415221612	0,501028	0,449453	0,712119806	autophagy-related protein 12, putative (ATG12)
PFIT_bin02000	2,943446364	4,790463458	5,867771733	9,363136663	3,866954911	7,6154542	1,969367208	0,9777321	0,449647	0,712119806	rifin
PFIT_0104300	1783,728497	1440,731885	1748,595976	1746,893783	1612,230191	1747,7449	1,084054181	0,1164369	0,4499093	0,712135626	conserved Plasmodium protein, unknown function
PFIT_1426000	2,943446364	2,395231729	5,867771733	6,019159283	2,669339047	5,9434655	2,226568227	1,1548218	0,4497997	0,712135626	unspecified product
PFIT_0805700	275,212235	239,5231729	243,9316535	342,4232837	257,367704	293,17747	1,139138532	0,1879432	0,4504536	0,712797332	conserved Plasmodium protein, unknown function
PFIT_1343700	245,7777214	170,0634528	183,5774299	162,5137006	207,9196121	173,04577	0,832280147	-0,264859	0,4513858	0,713872341	conserved Plasmodium protein, unknown function
PFIT_0914600	1399,608746	1558,09824	1662,255907	1615,141074	1478,853493	1638,6985	1,108087108	0,1480713	0,451336	0,713872341	met-10 like protein, putative
PFIT_1247500	1061,112414	1196,418249	1065,419696	1005,1996	1128,765331	1035,3096	0,917205392	-0,124683	0,4515783	0,713976806	conserved protein, unknown function
PFIT_1456900	607,8216741	468,2678031	475,2895104	473,5071969	538,0447386	474,39835	0,881708006	-0,181627	0,4524558	0,71516394	conserved Plasmodium protein, unknown function
PFIT_0709000	525,405176	467,0701872	576,7181361	522,3292667	496,2376816	549,5237	1,107380035	0,1471504	0,4526502	0,715270887	conserved Plasmodium protein, unknown function
PFIT_0317000	969,8655769	936,5356061	1001,71246	1088,130239	953,2005915	1044,9213	1,096223984	0,1325426	0,4529444	0,715535624	proteasome component C8, putative
PFIT_0204300	85,35994455	79,04264706	79,63404495	111,020049	82,20129581	95,327047	1,159678154	0,2137245	0,4532262	0,715580237	conserved Plasmodium protein, unknown function
PFIT_0525400	4,415169546	0	1,676506209	12,03831857	2,207584773	6,8574124	3,106296289	1,6351954	0,4531659	0,715580237	conserved Plasmodium protein, unknown function
PFIT_1478700	80,94477501	93,41403744	59,51597044	86,27461639	87,17940622	72,895293	0,836152672	-0,258162	0,4540371	0,716591555	surface-associated interspersed protein 14.1 (SURFIN 14.1), pseudogene (SURF14.1)
PF_apicoplast_g	122,1530241	125,7496658	110,6494098	103,6632988	123,9513449	107,15635	0,864503361	-0,210057	0,4541205	0,716591555	SufB protein, putative
PFIT_1032100	601,9347814	528,1485963	523,0699374	489,5582884	565,0416888	506,31411	0,896065057	-0,158325	0,454495	0,716781799	conserved Plasmodium protein, unknown function
PFIT_1316900	375,2894114	388,0275401	468,5834855	391,2453534	381,6584758	429,91442	1,1264375	0,1717673	0,4544935	0,716781799	conserved Plasmodium protein, unknown function
PFIT_0917500	696,1250651	574,855615	663,896459	720,961523	635,49034	692,42899	1,08959798	0,1237959	0,4549123	0,717039384	conserved Plasmodium protein, unknown function
PFIT_0835300	2,943446364	5,988079323	4,191265524	14,04470499	4,465762843	9,1179853	2,041753129	1,0298084	0,4548144	0,717039384	Plasmodium exported protein (PHISTa-like), unknown function
PFIT_1210100	136,8702559	100,5997326	145,0177871	126,4023449	118,7349943	135,71007	1,142966038	0,1927825	0,4558307	0,718286464	conserved Plasmodium protein, unknown function
PFIT_0515400	2041,280053	2064,689751	1793,023391	1942,182062	2052,984902	1867,6027	0,90970115	-0,136535	0,4560821	0,718481963	phosphatidylinositol 3-kinase
PFIT_0103100	216,3433077	203,594697	150,8855589	212,0081659	209,9690024	181,44686	0,864160235	-0,210629	0,4563801	0,718750925	conserved Plasmodium protein, unknown function
PFIT_1476500	179,5502282	210,7803922	183,5774299	294,2700094	195,1653102	238,92372	1,224212025	0,2918534	0,4566658	0,719000361	cysteine repeat modular protein 4
PFIT_1119100	9800,204669	10637,22411	9790,796263	9082,242563	10218,71439	9436,5194	0,923454659	-0,114887	0,4568774	0,719132985	insulinase, putative
PFIT_1037400	32,37791	52,69509804	46,94217387	60,19159283	42,53650402	53,566883	1,259315606	0,3326399	0,4570748	0,719243088	conserved Plasmodium protein, unknown function
PFIT_1302700	7853,114899	7675,520076	7763,062003	9072,879426	7764,317487	8417,9707	1,084186824	0,1166134	0,4575783	0,719834791	sodium/hydrogen exchanger, Na+, H+ antiporter
PFIT_1010800	251,6646641	194,0137701	208,7250231	180,5747785	222,8392171	194,6499	0,873499303	-0,195122	0,4577381	0,719885532	conserved Plasmodium membrane protein, unknown function
PFIT_1339100	266,3818959	205,9899287	176,033152	224,0464844	236,1859123	200,03982	0,846959144	-0,239636	0,4585151	0,720903227	ABC transporter (MDR family), putative
PFIT_1420700	2373,889492	2772,480727	2455,243344	3626,20907	2573,185109	3040,7262	1,181697421	0,2408607	0,4586405	0,720903227	conserved Plasmodium protein, unknown function
PFIT_1453100	2144,300676	2043,132665	2393,212614	2195,655547	2093,716671	2294,4341	1,095866558	0,1320721	0,4596404	0,722273851	small subunit rRNA processing factor, putative
PFIT_1353100	971,3373001	1227,556261	1248,997126	1207,84463	1099,446781	1228,4209	1,117308177	0,1600272	0,460217	0,722978613	proteasome subunit, putative
PFIT_1019400	1879,390503	1703,009759	1733,507421	1530,872844	1791,200131	1632,1901	0,911227117	-0,134117	0,461357	0,723526773	autophagy-related protein 8 (ATG8)
PFIT_0512700	1841,125701	1934,149621	1686,565247	1763,61367	1887,637661	1725,0895	0,913888027	-0,129911	0,4611349	0,723526773	Rab GTPase 1b
PFIT_0708200	2865,445035	2844,337678	2659,777101	2564,83065	2854,891357	2612,3039	0,915027421	-0,128113	0,4607459	0,723526773	Cg2 protein
PFIT_0312400	482,7252037	582,0413102	555,7618084	631,3429293	532,3832569	593,55237	1,114896761	0,1569101	0,461463	0,723526773	transporter, putative
PFIT_1411700	450,3472937	348,5062166	387,2729344	515,6413119	399,4267551	451,45712	1,130262601	0,176658	0,4614008	0,723526773	conserved Plasmodium protein, unknown function
PFIT_0607500	103,0206227	108,9830437	140,8265216	109,0136626	106,0018332	124,92009	1,178471054	0,2369163	0,4612783	0,723526773	conserved Plasmodium protein, unknown function
PFIT_1326300	8,830339092	22,75470143	22,63283383	23,40784166	15,79252026	23,020338	1,457673466	0,5436676	0,4611324	0,723526773	conserved Plasmodium protein, unknown function
PFIT_1035900	128,0399168	147,3067513	116,5171816	123,727163	137,6733341	120,12217	0,872515895	-0,196747	0,4618248	0,723740731	phosducin-like protein, putative (PhLP2)
PFIT_0316700	551,8961932	582,0413102	619,4690444	638,03088	566,9687517	628,74996	1,108967579	0,1492172	0,4618558	0,723740731	HVA22/TB2/DP1 family protein, putative
PFIT_0822500	2334,152967	2170,079947	2175,266807	1939,50688	2252,116457	2057,3868	0,913534839	-0,130468	0,4622634	0,723997388	conserved Plasmodium protein, unknown function
PFIT_0627500	1064,055861	1155,69309	1057,875418	979,1165767	1109,877585	1018,496	0,917665165	-0,12396	0,4622761	0,723997388	conserved Plasmodium protein, unknown function
PFIT_1121600	7,35861591	8,383311052	5,029518628	34,10856927	7,870963481	19,569044	2,48623234	1,3139611	0,462528	0,724191017	conserved Plasmodium protein, unknown function
PFIT_1003000	2191,395818	2077,863525	1645,490845	2160,209387	2134,629671	1902,8501	0,891419313	-0,165824	0,4629892	0,724712156	conserved Plasmodium protein, unknown function
PFIT_0707600	671,105771	627,550713	578,3946423	590,5464052	649,328242	584,47052	0,900115667	-0,151818	0,4637049	0,725631398	Cg8 protein
PFIT_1137400.1	297,2880828	320,9610517	267,4027404	285,5756682	309,1245672	276,4892	0,894426499	-0,160965	0,4638346	0,725633122	conserved Plasmodium protein, unknown function
PFIT_1131600	10,30206227	4,790463458	6,706024838	17,38868237	7,546262866	12,047354	1,596466201	0,674882	0,4642948	0,726151955	armadillo repeat protein PF16, putative
PFIT_1038300	80,94477501	142,5162879	98,07561325	84,93702544	111,7305314	91,506319	0,818991176	-0,28808	0,4650512	0,726932246	rifin, pseudogene
Pf_M7661101?	76,52960546	70,65933601	77,95753874	46,14688784	73,59447074	62,052213	0,843164067	-0,246115	0,4649727	0,726932246	unspecified product
PFIT_1433600	186,9088441	167,6666221	141,6647747	165,1924826	177,2875326	153,42863	0,865422551	-0,208523	0,4660677	0,728319464	leucine-rich repeat protein
[illegible]	[illegible]	[illegible]	[illegible]	[illegible]	[illegible]	[illegible]	[illegible]	[illegible]	[illegible]	[illegible]	[illegible]

PFIT_0608300	239,8908787	216,7684715	241,4168942	255,4798718	228,3296751	248,44838	1,088112541	0,1218278	0,5715648	0,807167365 mitochondrial cardiolipin synthase, putative
PFIT_0422000	5192,239386	4972,50107	5517,381935	5345,013444	5082,370228	5431,1977	1,0686348	0,0957689	0,5717448	0,807219581 cysteine-rich protective antigen (CyRPA)
PFIT_1243000	2919,898793	3455,121769	3386,542543	3505,825885	3187,510281	3446,1842	1,081152345	0,1125698	0,5726452	0,80828872 rab specific GDP dissociation inhibitor
PFIT_bin00100	164,8329964	149,7019831	128,252725	151,1477776	157,2674897	139,70025	0,888297076	-0,170886	0,5735818	0,808670865 stlt_CS2var - erythrocyte membrane protein 1,, putative
PFIT_0422400	4219,430363	3561,709581	4046,247737	3028,97471	3890,569972	3537,6112	0,909278396	-0,137206	0,5736321	0,808670865 reticulocyte binding protein homologue 4
PFIT_1462300	438,5735082	417,9679367	399,0084779	388,5701715	428,2707225	393,78932	0,919486913	-0,121099	0,5736034	0,808670865 conserved Plasmodium protein, unknown function
PFIT_1308300	9123,212005	10346,20345	9619,79263	8810,7116	9734,70773	9215,2521	0,946638807	-0,079114	0,5732716	0,808670865 60S ribosomal protein L24, putative
PFIT_0608800	1324,550864	1499,415062	1300,968819	1845,875514	1411,982963	1573,4222	1,114335093	0,1561831	0,5731061	0,808670865 conserved Plasmodium protein, unknown function
PFIT_0212900	175,1350587	210,7803922	171,0036334	181,9123694	192,9577254	176,458	0,914490472	-0,12896	0,5738788	0,808816658 conserved Plasmodium protein, unknown function
PFIT_1223300	922,7704351	954,4998441	948,9025146	1066,059989	938,6351396	1007,4813	1,073347043	0,1021166	0,5740909	0,808913742 conserved Plasmodium protein, unknown function
PFIT_1115300	60,34065046	58,68317736	55,32470491	47,48447879	59,51191391	51,404592	0,863769764	-0,211281	0,574254	0,808941602 conserved Plasmodium protein, unknown function
PFIT_0815100	136,8702559	140,1210562	148,3707995	159,1733233	138,495656	153,77206	1,110302415	0,1509527	0,5749661	0,809742719 trypsin-like serine protease, putative
PFIT_0821700	1523,233493	1622,769497	1724,286636	1137,621105	1573,001495	1430,9539	0,909696447	-0,136543	0,5755629	0,809840545 U3 small nucleolar ribonucleoprotein protein, putative
PFIT_0926900	557,783086	553,2985294	537,3202401	493,5710612	555,5408077	515,44565	0,927826801	-0,108073	0,576017	0,809840545 conserved Plasmodium protein, unknown function
PFIT_0703700	6874,418983	6637,187122	6536,697711	6110,784263	6755,803052	6323,741	0,936045787	-0,095349	0,5762596	0,809840545 conserved Plasmodium protein, unknown function
PFIT_1448400	2316,492288	2693,438079	2285,077964	2428,396373	2504,965184	2356,7372	0,940826317	-0,088	0,5763265	0,809840545 conserved Plasmodium protein, unknown function
PFIT_1434900	2409,210849	2578,466956	2983,3428	2427,058782	2493,838903	2705,2008	1,084753625	0,1173674	0,575737	0,809840545 conserved Plasmodium protein, unknown function
PFIT_1209100.1	615,1802901	586,8317736	731,7949604	575,1641093	601,0060319	653,47953	1,087309445	0,1207626	0,576099	0,809840545 cGMP-specific phosphodiesterase
PFIT_0503800	4220,902086	4429,981083	5286,862332	4155,895087	4325,441584	4721,3787	1,09153681	0,1263608	0,5753273	0,809840545 60S ribosomal protein L31, putative
PFIT_0423400	110,3792386	136,5282086	167,6506209	119,7143902	123,4537236	143,68251	1,163857204	0,2189141	0,5757033	0,809840545 Plasmodium exported protein (hyp15), unknown function
PFIT_0110400	45,62341864	61,07840909	48,61868007	78,91786616	53,35091387	63,768273	1,195261121	0,2573258	0,5761521	0,809840545 conserved Plasmodium protein, unknown function
PFIT_1124800	1723,387846	1985,647103	1857,56888	1652,593621	1854,517475	1755,0813	0,946381619	-0,079506	0,5770366	0,810636642 splicing factor, putative
PFIT_0413800	328,1942696	258,6850268	271,5940059	369,8438982	293,4396482	320,71895	1,092963933	0,1282458	0,5773136	0,810824074 conserved Plasmodium protein, unknown function
PFIT_1031300	35,32135637	21,55708556	24,30934004	44,80929689	28,43922096	34,559318	1,215199196	0,2811928	0,577544	0,810945957 conserved Plasmodium protein, unknown function
PFIT_1328100	859,4863383	784,4383913	804,7229805	720,2927276	821,9623648	762,50785	0,9276676	-0,10832	0,5778852	0,811223202 transcriptional regulatory protein sir2a
PFIT_0923900.1	309,0618682	241,9184046	207,88677	273,5373497	275,4901364	240,71206	0,873759268	-0,194692	0,5784126	0,811761793 thioredoxin reductase
PFIT_0603200	526,8768991	543,7176025	523,9081905	472,8384015	535,2972508	498,3733	0,931021587	-0,103113	0,5786755	0,811928828 conserved Plasmodium protein, unknown function
PFIT_1315200	29,43446364	39,52132353	24,30934004	32,77097832	34,47789359	28,540159	0,827781405	-0,272678	0,5790084	0,812014661 protein kinase, putative
PFIT_1238200	407,6673214	439,5250223	336,139495	440,0674232	423,5961719	388,10346	0,916210969	-0,126248	0,5790243	0,812014661 conserved Plasmodium protein, unknown function
PFIT_0303900	122,1530241	112,5758913	105,6198912	102,9945033	117,3644577	104,3072	0,888746042	-0,170157	0,5802034	0,812203912 inner membrane complex protein 1a, putative
PFIT_0510500	1202,39784	1177,256395	1043,625115	1160,360151	1189,827117	1101,9926	0,926178784	-0,110637	0,5797032	0,812203912 topoisomerase I
PFIT_1402300	60053,66444	68436,56097	68271,52412	52195,47412	64245,1127	60233,499	0,937557685	-0,093021	0,5796723	0,812203912 early transcribed membrane protein 14.1
PFIT_1320500	3283,414419	3474,283623	3139,257877	3199,517557	3378,849021	3169,3877	0,93800809	-0,092328	0,5801931	0,812203912 conserved Plasmodium membrane protein, unknown function
PFIT_1404700	47,09514182	55,09032977	49,45693318	68,88593402	51,0927358	59,171434	1,158118325	0,2117827	0,5802312	0,812203912 translocation associated membrane protein, putative
PFIT_1362600	25,01929409	14,37139038	24,30934004	24,07663713	19,69534223	24,192989	1,228360914	0,2967345	0,5803101	0,812203912 conserved Plasmodium protein, unknown function
PFIT_0500500	8,830339092	10,77854278	16,76506209	11,36952309	9,804440936	14,067293	1,434787836	0,5208374	0,5801276	0,812203912 rifin (RIF)
PFIT_0800960	1,471723182	1,197615865	5,867771733	1,337590952	1,334669523	3,6026813	2,699305918	1,4325885	0,5795523	0,812203912 rifin (RIF)
PFIT_0207600	1373,117729	1322,167915	1328,631171	1189,787152	1347,642822	1259,2092	0,934379007	-0,09792	0,5806675	0,81221717 conserved Plasmodium protein, unknown function
PFIT_0935900	67,69926637	86,22834225	82,98705737	93,63136663	76,96380431	88,309212	1,147412252	0,1983838	0,5807511	0,81221717 phosphatidylinositol N-acetylglucosaminyltransferase subunit P, putative
PFIT_1216700	47,09514182	46,70701872	62,86898286	48,15327427	46,90108027	55,511129	1,183578891	0,2431559	0,5806478	0,81221717 conserved Plasmodium protein, unknown function
PFIT_0627800	713,7857432	632,3411765	727,6036949	712,9359773	673,0634599	720,26984	1,07013659	0,097795	0,5810575	0,812243322 RAP protein, putative
PFIT_0911400	57,3972041	62,27602496	57,83946423	78,91786616	59,83661453	68,378665	1,142756249	0,1925177	0,5809878	0,812243322 conserved Plasmodium protein, unknown function
PFIT_0920100	73,5861591	92,21642157	86,34006979	63,53557021	82,90129034	74,93782	0,903940333	-0,145701	0,5813752	0,812285231 long chain fatty acid elongation enzyme, putative
PFIT_1200400	1537,950725	1726,962077	1480,354983	1581,701301	1632,456401	1531,0281	0,937867707	-0,092544	0,5812802	0,812285231 acyl-CoA synthetase
PFIT_1439200	4691,853504	4728,187433	4983,414708	3923,154262	4710,020469	4453,2845	0,945491535	-0,080864	0,5827904	0,814060971 eukaryotic translation initiation factor eIF2A, putative
PFIT_1330800	1383,419791	1241,927652	1170,201334	1253,991517	1312,673721	1212,0964	0,923379821	-0,115004	0,5829894	0,814137553 conserved Plasmodium protein, unknown function
PFIT_0915500	21728,52106	25412,21103	22863,35343	21870,28086	23570,36604	22366,817	0,948938048	-0,075614	0,5833919	0,814296876 6-phosphofructokinase
PFIT_0208000.2	235,4757091	289,8230392	310,9919019	272,8685542	262,6493742	291,93023	1,111482671	0,1524855	0,5833041	0,814296876 conserved Plasmodium protein, unknown function
PFIT_0627700	110,3792386	118,5639706	101,4286257	103,6632988	114,4716046	102,54596	0,895820082	-0,158719	0,5842438	0,815082913 glutamyl-tRNA(Gln) amidotransferase subunit B, putative
PFIT_1201400	55,92548091	45,50940285	52,8099454	62,86677474	50,71744188	57,83836	1,140403735	0,1895447	0,5842271	0,815082913 conserved Plasmodium membrane protein, unknown function
PFIT_1017000	7435,145515	7426,415976	7939,933408	5960,974077	7430,780746	6950,4537	0,93535982	-0,096407	0,5843968	0,815095009 conserved Plasmodium protein, unknown function
PFIT_0104800	10892,22327	12052,80606	10901,48163	10762,92559	11472,51467	10832,204	0,944187384	-0,082855	0,5847174	0,815340716 calcium-transporting ATPase
PFIT_1474500	2619,667264	2480,262456	2570,084019	2213,04423	2549,96486	2391,5641	0,937881209	-0,092523	0,5851837	0,815789452 conserved Plasmodium protein, unknown function
PFIT_1038000	2822,765063	3235,958066	3483,779903	3080,471962	3029,361565	3282,1259	1,083438164	0,1156168	0,5857666	0,816400471 RESA-like protein with PHIST and DnaJ domains
PFIT_1219500	5,886892728	2,395231729	6,706024838	6,687954759	4,141062228	6,6969898	1,617215446	0,6935119	0,5859193	0,816411791 conserved Plasmodium protein, unknown function
PFIT_0909500	1286,286061	1270,670432	1370,543826	1009,881169	1278,478247	1190,2125	0,930960304	-0,103208	0,586079	0,816432728 apoptosis-related protein, putative
PFIT_0733100	25,01929409	29,94039661	32,69187108	34,77736475	27,47984535	33,734618	1,227613092	0,2958559	0,5864115	0,816694453 stevor, pseudogene
PFIT_1111700	1589,461037	1550,912545	1840,803818	1540,235981	1570,186791	1690,5199	1,076636175	0,1065308	0,5870544	0,817388195 ES2 protein, putative
PFIT_0533700	344,3832246	429,9440954	466,0687262	391,2453534	387,16366	428,65704	1,107172713	0,1468803	0,5874077	0,817678477 cell cycle regulator protein, putative
PFIT_1124600	33,84963318	19,16185383	25,14759314	36,78375118	26,50574351	30,965672	1,168262726	0,2243648	0,5876964	0,817878692 mitochondrial ribosomal protein L37, putative
PFIT_0915200	566,613425	667,0720366	631,2045879	534,3675853	616,8427308	582,78609	0,944788773	-0,081936	0,5879288	0,818000458 ubiquitin conjugating enzyme, putative
Pf_M76611018I	7682,39501	7506,656239	7033,781802	7175,506661	7594,525624	7104,6442	0,935495459	-0,096197	0,5883248	0,818189455 cytochrome c oxidase subunit 1
PFIT_1407400	192,7957368	185,630459	165,9741147	258,1550537	189,2130979	212,06458	1,120771165	0,1644917	0,5883544	0,818189455 PPR repeat protein
PFIT_0208800	410,6107678	310,1825089	359,6105819	283,5692818	360,3966383	321,58993	0,89232223	-0,164363	0,5886242	0,818362989 conserved Plasmodium protein, unknown function
PFIT_0521900	261,9667264	273,0564171	248,122919	244,1103487	267,5115718	246,11663	0,920022383	-0,120259	0,5895882	0,819350612 DEAD/DEAH box ATP-dependent RNA helicase, putative
PFIT_0713600	8,830339092	11,97615865	15,08855589	14,04470499	10,40324887	14,56663	1,400200132	0,485633	0,5896248	0,819350612 mitochondrial ATP synthase F1, epsilon subunit, putative
PFIT_1334900	535,7072382	485,0344252	501,2753566	585,8648369	510,3708317	543,5701	1,065049299	0,0909202	0,5900277	0,819507057 conserved Plasmodium protein, unknown function
PFIT_1440100	2793,330599	3586,859514	3423,42568	3508,501067	3190,095057	3465,9634	1,086476519	0,119657	0,5899685	0,819507057 thioredoxin peroxidase 1
PFIT_0924600	1371,646006	1288,63467	1576,75409	1284,087314	1330,140338	1430,4207	1,075390815	0,1048611	0,5902869	0,819665448 conserved Plasmodium membrane protein, unknown function
PFIT_1005400	481,2534805	459,884492	557,4383146	463,4752648	470,5689862	510,45679	1,08476505	0,1173826	0,5908361	0,82026376 conserved Plasmodium membrane protein, unknown function
PFIT_1458200	3251,036509	3200,02959	3099,021728	2951,394435	3225,53305	3025,2081	0,937893996	-0,092503	0,5912067	0,82053903 signal peptide peptidase
PFIT_1449200	2079,544856	2404,812656	2292,622241	2524,702922	2242,178756	2408,6626	1,074250916	0,103331	0,5914106	0,820620382 U3 snoRNA-associated small subunit rRNA processing protein, putative
PFIT_0616000	790,3153487	1083,842357	891,9013034	856,0582092	937,0788531	873,97976	0,932664048	-0,100571	0,591759	0,820901991 mitochondrial import receptor subunit tom40, putative
PFIT_0309700	1680,707874	1837,142736	2082,220712	894,8483468	1758,925305	1488,5345	0,84627501	-0,240802	0,5924236	0,821019551 YTH domain-containing protein, putative
PFIT_1353400	1499,685922	1455,103275	1296,777553	1447,27341	1477,394599	1372,0255	0,928679097	-0,106748	0,5922318	0,821019551 inositol-polyphosphate 5-phosphatase, putative
PFIT_0925300	1220,058518	1196,418249	1170,201334	1090,136626	1208,238383	1130,169	0,935385761	-0,096367	0,5921033	0,821019551 rRNA processing and telomere maintaining methyltransferase, putative
PFIT_0610300	560,7265323	589,2270054	615,2777789	625,9925655	574,9767688	620,63517	1,079409127	0,1102418	0,5924254	0,821019551 SWI/SNF-related matrix-associated actin-dependent regulator of chromatin
PFIT_1471700	390,0066432	438,3274064	420,8030586	356,4759887	414,1670248	388,63552	0,938354578	-0,091795	0,5929507	0,821142941 RAP protein, putative
[illegible]	[illegible]	[illegible]	[illegible]	[illegible]	[illegible]	[illegible]	[illegible]	[illegible]	[illegible]	[illegible]

PFIT_0303700	161,88955	114,971123	140,8265216	149,8101866	138,4303365	145,31835	1,049758007	0,0700568	0,7101652	0,891229818	IBR domain protein, putative
PFIT_1010900	1106,735833	1261,089505	1167,686575	1324,215042	1183,912669	1245,9508	1,052400942	0,0736844	0,7101741	0,891229818	proteasome subunit beta type-5
PFIT_1013100	1792,558836	1368,874933	1397,367926	1526,860072	1580,716884	1462,114	0,924968926	-0,112523	0,7107001	0,891295548	conserved Plasmodium protein, unknown function
PFIT_1219800	510,6879441	627,550713	563,3060864	534,3675853	569,1193286	548,83684	0,964361617	-0,052354	0,7103871	0,891295548	pre-mRNA splicing factor, putative
PFIT_0712600	940,4311133	1070,668583	1114,876629	1021,250692	1005,549848	1068,0637	1,062168785	0,087013	0,7106293	0,891295548	transcription elongation factor s-II, putative
PFIT_0419400	0	3,592847594	0,838253105	0,668795476	1,796423797	0,7535243	0,419457976	-1,253402	0,7114092	0,891986739	unspecified product
PFIT_0506900	4453,434349	3829,975535	3751,182644	4068,951675	4141,704942	3910,0672	0,944071877	-0,083031	0,712785	0,893513358	rhomboid protease ROM4
PFIT_0625300	802,0891342	714,9766712	656,352181	767,7772064	758,5329027	712,06469	0,938739363	-0,091203	0,7141632	0,893622156	Sec14 domain containing protein
PFIT_0607400	1370,174282	1410,791488	1329,469424	1330,234202	1390,482885	1329,8518	0,956395671	-0,06432	0,7134566	0,893622156	proteasome subunit alpha type 2, putative
PFIT_1410300	855,0711687	772,4622327	814,7820178	878,1284599	813,7667007	846,45524	1,040169422	0,0568185	0,7133232	0,893622156	DNA damage-inducible protein 1, putative (DDI1)
PFIT_0108400.1	172,1916123	140,1210562	140,8265216	185,9251423	156,1563342	163,37583	1,0462325	0,0652035	0,714218	0,893622156	phosphatidate cytidylyltransferase, putative
PFIT_1328800	41324,51523	46965,70375	47730,97004	45421,91354	44145,10949	46576,442	1,055075915	0,0773468	0,7139121	0,893622156	conserved Plasmodium protein, unknown function
PFIT_1358200	893,3359714	925,7570633	1038,595597	887,4915965	909,5465174	963,0436	1,05881731	0,0824537	0,7134916	0,893622156	riboflavin kinase / FAD synthase family protein, putative
PFIT_0301100	10,30206227	7,185695188	10,05903726	12,03831857	8,743878731	11,048678	1,263590021	0,3375284	0,7141928	0,893622156	serine/threonine protein kinase, FIKK family
PFIT_0300600	2,943446364	2,395231729	3,353012419	5,350363807	2,669339047	4,3516881	1,6302493	0,7050926	0,7142963	0,893622156	rifin (RIF)
PFIT_0537300	2,943446364	2,395231729	3,353012419	5,350363807	2,669339047	4,3516881	1,6302493	0,7050926	0,7142963	0,893622156	rifin (RIF)
PFIT_0402600	1349,570158	1201,208712	1274,144719	1152,334605	1275,389435	1213,2397	0,951269964	-0,072073	0,7150058	0,894311652	SET domain protein, putative
PFIT_bin02800	2,943446364	1,197615865	0	1,337590952	2,070531114	0,6687955	0,323006726	-1,630364	0,7156909	0,894538327	rifin (RIF)
PFIT_1441000	515,1031137	604,7960116	453,4949297	599,2407464	559,9495626	526,36784	0,940027233	-0,089226	0,7153567	0,894538327	vesicle-associated membrane protein, putative
PFIT_1433400	1209,756456	966,4760027	1042,786862	1200,487879	1088,116229	1121,6374	1,030806582	0,0437737	0,7158208	0,894538327	conserved Plasmodium protein, unknown function
PFIT_1119700	8,830339092	7,185695188	10,05903726	10,70072761	8,00801714	10,379882	1,296186341	0,3742731	0,7156498	0,894538327	conserved Plasmodium protein, unknown function
PFIT_1353500	3875,047138	4227,584002	4051,277255	4433,44521	4051,31557	4242,3612	1,047156451	0,066477	0,7159882	0,894549521	large subunit rRNA methyltransferase, putative
PFIT_1423600	447,4038473	361,6799911	371,3461254	383,8886032	404,5419192	377,61736	0,933444339	-0,099364	0,7168365	0,894952098	conserved Plasmodium protein, unknown function
PFIT_0309400	7335,068339	8686,307866	8603,829867	6915,345221	8010,688102	7759,5875	0,968654308	-0,045946	0,7166564	0,894952098	asparagine synthetase, putative
PFIT_1030700	716,7291896	673,0601159	735,1479729	720,2927276	694,8946528	727,72035	1,04723838	0,0665899	0,7165663	0,894952098	conserved Plasmodium protein, unknown function
PFIT_1445800	8,830339092	14,37139038	13,41204968	16,05109142	11,60086473	14,731571	1,269868315	0,3446789	0,7169444	0,894952098	conserved Plasmodium protein, unknown function
PFIT_1449100	731,4464214	706,5933601	767,0015908	741,6941828	719,0198908	754,34789	1,049133545	0,0691983	0,7184772	0,896667131	multidrug resistance protein 2 (heavy metal transport family)
PFIT_0527700	8,830339092	1,197615865	1,676506209	3,34397738	5,013977478	2,5102418	0,500648797	-0,998129	0,7187168	0,896768028	outer arm dynein lc3, putative
PFIT_1220300	189,8522905	222,7565508	186,0921893	203,9826202	206,3044206	195,0374	0,945386454	-0,081024	0,7196675	0,89775578	conserved Plasmodium protein, unknown function
PFIT_1253200	103,0206227	126,9472816	144,179534	109,6824581	114,9839522	126,931	1,103901837	0,1426119	0,7198623	0,897800442	gametocyte erythrocyte cytosolic protein
PFIT_0624000	32,37791	29,94039661	20,95632762	32,10218284	31,15915331	26,529255	0,8514113	-0,232072	0,720055	0,897842473	sphingomyelin synthase 2, putative (SMS2)
PFIT_0807100	342,9115014	476,6511141	340,3307605	428,0291046	409,7813077	384,17993	0,937524297	-0,093072	0,7206841	0,898031897	conserved Plasmodium protein, unknown function
PFIT_0822700	194,26746	173,6543004	201,1807451	189,2691197	183,9608802	195,22493	1,061230693	0,0857383	0,7205437	0,898031897	conserved Plasmodium protein, unknown function
PFIT_0612000.3	30,90618682	22,75470143	22,63283383	36,78375118	26,83044412	29,708293	1,107260557	0,1469948	0,7205946	0,898031897	conserved Plasmodium protein, unknown function
PFIT_0809100	629,8975219	554,4961453	580,0711485	648,7316116	592,1968336	614,40138	1,037495213	0,0531047	0,7210612	0,898263277	conserved Plasmodium membrane protein, unknown function
PFIT_0801000	13,24550864	19,16185383	25,14759314	15,38229595	16,20368124	20,264945	1,250638312	0,3226646	0,721188	0,898263277	rifin (RIF)
PFIT_0316300.2	625,4823523	572,4603833	455,1714359	645,3876343	598,9713678	550,27954	0,918707579	-0,122322	0,7214264	0,898361981	inorganic pyrophosphatase, putative
PFIT_1420000	1387,834961	1223,963414	1224,687786	1249,309949	1305,899187	1236,9989	0,947239174	-0,078199	0,7226921	0,898447974	conserved Plasmodium protein, unknown function
PFIT_1330300	621,0671828	634,7364082	595,1597044	601,9159283	627,9017955	598,53782	0,953234758	-0,069097	0,7226107	0,898447974	protein kinase, putative
PFIT_1217900	3409,982613	3548,535807	3436,837729	3261,046741	3479,25921	3348,9422	0,96254462	-0,055075	0,7222566	0,898447974	conserved Plasmodium protein, unknown function
PFIT_0217800	2191,395818	2556,909871	2474,523165	2144,827091	2374,152844	2309,6751	0,972841801	-0,039723	0,7227685	0,898447974	DEAD/DEAH box ATP-dependent RNA helicase, putative
PFIT_0323600	1482,025244	1565,283935	1586,813127	1609,790711	1523,65459	1598,3019	1,048992291	0,0690041	0,7224323	0,898447974	survival of motor neuron (SMN)-like protein
PFIT_0537500	44,15169546	43,11417113	44,42741455	52,16604712	43,63293329	48,296731	1,106887096	0,1465081	0,7227639	0,898447974	rifin
PFIT_1247200.1	33,84963318	53,89271391	40,23614903	60,19159283	43,87117355	50,213871	1,144575512	0,1948126	0,7227326	0,898447974	DNA replication origin binding protein, putative
PFIT_1464700	1,471723182	11,97615865	11,73554347	8,694341187	6,723940914	10,214942	1,519189781	0,6033021	0,7223226	0,898447974	conserved Plasmodium protein, unknown function
PFIT_0933900	35,32135637	20,3594697	33,53012419	28,75820546	27,84041303	31,144165	1,118667485	0,1617813	0,7231759	0,898756588	conserved Plasmodium protein, unknown function
PFIT_1208000	451,8190169	373,6561498	384,7581751	467,4880377	412,7375833	426,12311	1,032431074	0,0460455	0,7237297	0,899246888	amino acid transporter, putative
PFIT_0213400	3209,82826	3808,418449	4121,690516	3386,78029	3509,123355	3754,2354	1,069849938	0,0974085	0,7240408	0,899435513	T-complex protein 1, putative
PFIT_1333900	114,7944082	88,62357398	101,4286257	113,6952309	101,7089911	107,56193	1,057545917	0,0807203	0,7242842	0,899539957	MSP7-like protein
PFIT_0605600	7663,262608	8403,670522	7840,181289	7676,434473	8033,466565	7758,3079	0,96574845	-0,050281	0,7246999	0,89985831	coatomer alpha subunit, putative
PFIT_0400200	19,13240137	23,95231729	15,92680899	20,73265975	21,54235933	18,329734	0,850869401	-0,23299	0,7254705	0,900227884	stevor, pseudogene
PFIT_1422600	819,7498123	840,7263369	889,3865441	856,0582092	830,2380746	872,72238	1,051171228	0,0719977	0,7257948	0,900227884	conserved Plasmodium protein, unknown function
[illegible]	[illegible]	[illegible]	[illegible]	[illegible]	[illegible]	[illegible]	[illegible]	[illegible]	[illegible]	[illegible]	[illegible]

PFIT_1005600	47,09514182	68,26410428	62,86898286	50,15966069	57,67962305	56,514322	0,979797003	-0,029445	0,8599555	0,971450352	conserved Plasmodium protein, unknown function
PFIT_1227500	4010,445671	4412,016845	4535,78755	4153,219905	4211,231258	4344,5037	1,031646913	0,0449493	0,8608107	0,97221855	histone S-adenosyl methyltransferase, putative
PFIT_1474400	211,9281382	217,9660874	194,4747203	220,7025071	214,9471128	207,58861	0,965766002	-0,050254	0,8609996	0,972240802	conserved Plasmodium protein, unknown function
PFIT_bin08800	22,07584773	31,13801248	32,69187108	18,72627333	26,6069301	25,709072	0,966254735	-0,049525	0,861413	0,972513074	rifin
PFIT_bin03700	0	3,592847594	1,676506209	0,668795476	1,796423797	1,1726508	0,6527696	-0,615354	0,8626162	0,97330717	rifin (RIF)
PFIT_0833900	19,13240137	47,90463458	32,69187108	44,14050141	33,51851797	38,416186	1,146118282	0,1967559	0,8624245	0,97330717	Plasmodium exported protein (PHISTb), unknown function
PFIT_0411700	1,471723182	5,988079323	6,706024838	4,012772856	3,729901252	5,3593988	1,436874192	0,5229337	0,8626336	0,97330717	rifin, pseudogene
PFIT_0927800	186,9088441	202,3970811	185,2539361	216,0209387	194,6529626	200,63744	1,03074433	0,0436865	0,863268	0,973828434	serine/threonine protein phosphatase, putative
PFIT_1231800	119,2095777	126,9472816	103,1051319	132,4215042	123,0784297	117,76332	0,956815247	-0,063688	0,8639463	0,974290164	phenylalanyl-tRNA synthetase alpha chain, putative
PFIT_1221400	259,02328	264,6731061	268,2409935	243,4415532	261,848193	255,84127	0,977059534	-0,033482	0,8640225	0,974290164	FbpA domain protein, putative
PFIT_0709700	415,0259373	437,1297906	456,009689	424,0163317	426,0778639	440,01301	1,032705633	0,0464291	0,86449	0,974622681	conserved Plasmodium protein, unknown function
PFIT_0624500	2937,559471	3206,017669	3052,917807	3003,560482	3071,78857	3028,2391	0,985822779	-0,0206	0,8647612	0,974733833	poly(A) polymerase PAP, putative
PFIT_1117600	36252,95714	43327,34675	45937,94665	37271,97187	39790,15194	41604,959	1,045609459	0,0643441	0,8655979	0,975482134	heat shock protein 101
PFIT_0615000	95,66200683	95,80926917	94,72260084	89,61859377	95,735638	92,170597	0,962761613	-0,054749	0,866467	0,976072017	pyridoxine kinase
PFIT_1225700	129,51164	111,3782754	122,3849533	125,064754	120,4449577	123,72485	1,027231492	0,0387613	0,8663279	0,976072017	conserved Plasmodium protein, unknown function
PFIT_1369100	2,943446364	1,197615865	0	2,006386428	2,070531114	1,0031932	0,484510089	-1,045401	0,8675056	0,976132312	tRNA Cysteine
PFIT_1150800	41,20824909	47,90463458	42,75090834	42,13411498	44,55644184	42,442512	0,952556127	-0,070124	0,8669624	0,976132312	rifin (RIF)
PFIT_1447900	506,2727746	422,7584002	467,7452324	431,373082	464,5155874	449,55916	0,967802092	-0,047216	0,8675829	0,976132312	conserved Plasmodium protein, unknown function
PFIT_1218800	4839,025822	4688,66611	4719,36498	4608,669625	4763,845966	4664,0173	0,979044523	-0,030554	0,8666949	0,976132312	clathrin heavy chain, putative
PFIT_1250500	220,7584773	173,6543004	166,8123678	231,4032347	197,2063888	199,1078	1,009641738	0,0138435	0,8672493	0,976132312	Hbeta58/Vps26 protein homolog, putative
PFIT_0707200	254,6081105	328,1467469	318,5361798	292,263623	291,3774287	305,3999	1,048124773	0,0678105	0,8678175	0,976132312	O-sialoglycoprotein endopeptidase, putative
PFIT_1443100	33,84963318	40,7189394	41,07440213	39,45893308	37,28428629	40,266668	1,079990302	0,1110184	0,8678315	0,976132312	transcription factor TFIIH complex subunit Tfb5, putative
PFIT_0925000	2,943446364	4,790463458	4,191265524	6,019159283	3,866954911	5,1052124	1,320215136	0,400773	0,8679037	0,976132312	conserved protein, unknown function
PFIT_1334500	1777,841604	2529,364706	2722,646084	1541,573572	2153,603155	2132,1098	0,990019829	-0,014471	0,8685969	0,976271221	reticulocyte binding protein homologue 6, pseudogene
PFIT_1422200	6215,086997	7275,516377	7969,272267	5398,517082	6745,301687	6683,8947	0,990896328	-0,013194	0,8690647	0,976271221	40S ribosomal protein S25, putative
PFIT_0830500	746,1636532	621,5626337	603,5422354	771,7899792	683,8631435	687,66611	1,005561001	0,0080006	0,8688866	0,976271221	conserved Plasmodium protein, unknown function
PFIT_1128500	3636,627983	3591,649978	4031,159181	3390,793063	3614,13898	3710,9761	1,026793973	0,0381467	0,8690474	0,976271221	CRAL/TRIO domain-containing protein, putative
PFIT_0804300	1484,968691	1560,493472	1736,860433	1410,489659	1522,731081	1573,675	1,033455654	0,0474765	0,8689596	0,976271221	conserved Plasmodium protein, unknown function
PFIT_1442900	30,90618682	25,14993316	34,36837729	26,08302356	28,02805999	30,2257	1,078408582	0,1089039	0,8685316	0,976271221	metalloprotease, putative
PFIT_1222500	19,13240137	14,37139038	11,73554347	17,38868237	16,75189587	14,562113	0,869281485	-0,202105	0,8695946	0,976667823	conserved Plasmodium protein, unknown function
PFIT_1024700	132,4550864	118,5639706	109,8111567	129,0775269	125,5095285	119,44434	0,951675488	-0,071458	0,8699848	0,976667823	conserved Plasmodium protein, unknown function
PFIT_0925200	29,43446364	39,52132353	39,39789592	27,42061451	34,47789359	33,409255	0,969005114	-0,045424	0,8699529	0,976667823	conserved Plasmodium protein, unknown function
PFIT_1211000	4886,120964	5336,576293	5842,62414	4292,99816	5111,348628	5067,8111	0,991482194	-0,012341	0,8701097	0,976667823	heat shock protein DNAJ homologue Pfj4
PFIT_0828700	91,24683728	102,9949644	93,88434773	94,3001621	97,12090082	94,092255	0,968815714	-0,045706	0,8709497	0,977416456	conserved Plasmodium protein, unknown function
PFIT_1340000	45,62341864	44,31178699	42,75090834	42,13411498	44,96760281	42,442512	0,943846436	-0,083376	0,8719563	0,977650961	mitochondrial pyruvate carrier protein 1, putative (MPC1)
PFIT_0919200	1436,401826	1494,624599	1356,293523	1510,140185	1465,513212	1433,2169	0,977962424	-0,032149	0,8717025	0,977650961	DnaJ protein, putative
PFIT_1106800	2260,566807	2655,114372	2651,39457	2251,834367	2457,84059	2451,6145	0,997466833	-0,003659	0,8722227	0,977650961	RuvB DNA helicase, putative (RUVB2)
PFIT_0615100	92,71856046	81,43787879	72,08976701	105,0008897	87,07821963	88,545328	1,016848171	0,0241043	0,8723708	0,977650961	conserved Plasmodium protein, unknown function
PFIT_0213100	257,5515568	274,254033	267,4027404	280,2253044	265,9027949	273,81402	1,029752329	0,0422974	0,8720636	0,977650961	conserved protein, unknown function
PFIT_0533300	52,98203455	41,91655526	47,78042697	50,82845617	47,44929491	49,304442	1,039097455	0,055331	0,8718584	0,977650961	conserved Plasmodium protein, unknown function
PFIT_1037700	27,96274046	20,3594697	15,08855589	35,44616022	24,16110508	25,267358	1,045786522	0,0645884	0,8723661	0,977650961	Plasmodium exported protein, unknown function
PFIT_0700500	1,471723182	2,395231729	2,514759314	0	1,933477456	1,2573797	0,650320309	-0,620778	0,8727333	0,977863166	stevor
PFIT_1468200	189,8522905	240,7207888	212,0780355	212,6769613	215,2865396	212,3775	0,986487584	-0,019627	0,8737846	0,978846788	DNA-3-methyladenine glycosylase, putative
PFIT_1352600	1532,063832	1422,767647	1563,34204	1447,27341	1477,41574	1505,3077	1,018878901	0,0269826	0,8743686	0,979306731	Ran-binding protein, putative
PFIT_0918700	335,5528855	370,0633022	326,9187108	395,2581263	352,8080938	361,08842	1,02346977	0,0334685	0,8747747	0,979567252	DNA helicase, putative
PFIT_0320600	97,13373001	105,3901961	106,4581443	105,0008897	101,261963	105,72952	1,044118777	0,0622858	0,8751741	0,979820085	conserved Plasmodium protein, unknown function
PFIT_0913700	351,7418405	270,6611854	275,7852715	317,0090556	311,2015129	296,39716	0,952428414	-0,070317	0,8756698	0,980180803	conserved Plasmodium protein, unknown function
PFIT_1427100	3196,582751	3560,511965	3876,920609	2835,024022	3378,547358	3355,9723	0,993318122	-0,009672	0,8761767	0,980258341	basic transcription factor 3b, putative
PFIT_1344100	4272,412397	4891,063191	5007,724048	4458,190642	4581,737794	4732,9573	1,033004846	0,046847	0,87626	0,980258341	conserved Plasmodium protein, unknown function
PFIT_0731700	19,13240137	14,37139038	15,92680899	20,06386428	16,75189587	17,995337	1,074226868	0,1032987	0,8760627	0,980258341	erythrocyte membrane protein 1, PfEMP1, putative
PFIT_0313300	472,4231414	501,8010473	481,1572821	475,5135834	487,1120943	478,33543	0,981982255	-0,026231	0,8773602	0,981108733	conserved Plasmodium protein, unknown function
PFIT_1136600	616,6520132	577,2508467	546,5410243	659,4323393	596,95143	602,98668	1,010110122	0,0145126	0,8773677	0,981108733	3-oxo-5-alpha-steroid 4-dehydrogenase, putative
PFIT_0609200	89,7751141	87,42595811	98,91386636	73,56750235	88,60053611	86,240684	0,973365266	-0,038947	0,878463	0,981361692	unspecified product
PFIT_0922200	7903,153487	8870,740709	8077,406917	8487,683385	8386,947098	8282,5452	0,987551853	-0,018072	0,8783764	0,981361692	ubiquitin-like protein, putative
PFIT_1230100	350,2701173	317,3682041	310,9919019	364,4935344	333,8191607	337,74272	1,011753542	0,0168579	0,8782738	0,981361692	heat shock protein hslv
PFIT_0627400	41,20824909	47,90463458	41,91265524	52,16604712	44,55644184	47,039351	1,055725036	0,0782341	0,8778424	0,981361692	conserved Plasmodium protein, unknown function
PFIT_1300300	1,471723182	1,197615865	4,191265524	0,668795476	1,334669523	2,4300305	1,820698276	0,8644919	0,8779808	0,981361692	stevor
PFIT_0106400	66,22754319	63,47364082	67,06024838	57,51641093	64,85059201	62,28833	0,960489762	-0,058158	0,8791503	0,981716466	conserved Plasmodium protein, unknown function
PFIT_0208500	394,4218128	481,4415776	441,7593862	426,6915136	437,9316952	434,22545	0,991536933	-0,012262	0,8792823	0,981716466	3'exoribonuclease, putative
PFIT_1442200	376,7611346	408,3870098	418,2882993	391,9141489	392,5740722	405,10122	1,031910288	0,0453176	0,8793077	0,981716466	conserved Plasmodium protein, unknown function
PFIT_0804700	2752,12235	2714,995165	3526,530812	2245,815208	2733,558758	2886,173	1,055829878	0,0783774	0,8794761	0,981716466	conserved Plasmodium protein, unknown function
PFIT_bin07100	7,35861591	4,790463458	4,191265524	5,350363807	6,074539684	4,7708147	0,785378796	-0,348539	0,8821929	0,981847881	rifin
PFIT_0512200	20,60412455	13,17377451	15,92680899	14,04470499	16,88894953	14,985757	0,887311373	-0,172488	0,880501	0,981847881	conserved Plasmodium protein, unknown function
PFIT_0828200	54,45375773	43,11417113	42,75090834	48,15327427	48,78396443	45,452091	0,931701469	-0,10206	0,8823065	0,981847881	conserved protein, unknown function
PFIT_0829100	69,17098955	69,46172015	58,67771733	72,89870687	69,31635485	65,788212	0,949100862	-0,075367	0,8804487	0,981847881	alpha/beta hydrolase, putative
PFIT_0903000	883,0339092	681,4434269	670,6024838	816,5992761	782,2386681	743,60088	0,950606139	-0,07308	0,8825501	0,981847881	serine repeat antigen 9
PFIT_0316000	3383,491595	2447,926827	3722,682038	1825,142854	2915,709211	2773,9124	0,951368002	-0,071925	0,8811715	0,981847881	conserved Plasmodium protein, unknown function
PFIT_0323400	97,13373001	97,00688503	88,016576	98,31293496	97,07030752	93,164755	0,959765739	-0,059246	0,8824014	0,981847881	conserved protein, unknown function
PFIT_1367800	699,0685114	558,0889929	584,262414	625,32377	628,5787522	604,79309	0,962159618	-0,055652	0,8801009	0,981847881	DNA repair endonuclease, putative
PFIT_1220100	468,0079719	432,3393271	419,9648055	453,4433327	450,1736495	436,70407	0,970079145	-0,043826	0,8824006	0,981847881	debranching enzyme-associated ribonuclease, putative
PFIT_1477800	111,8509618	111,3782754	124,0614595	94,3001621	111,6146186	109,18081	0,978194543	-0,031807	0,8820739	0,981847881	lysophospholipase, putative
PFIT_0613000	1417,269424	1318,575067	1431,736303	1254,660313	1367,922246	1343,1983	0,981925919	-0,026314	0,8815197	0,981847881	thiamin-phosphate pyrophosphorylase, putative
PFIT_1131200	3471,794986	3803,627986	3484,618156	3685,731868	3637,711486	3585,175	0,985557823	-0,020988	0,8797921	0,981847881	26S proteasome regulatory subunit 6a, putative
PFIT_0708300	328,1942696	249,1040998	233,8726162	347,104852	288,6491847	290,48873	1,006372959	0,0091651	0,8807078	0,981847881	Cg7 protein
PFIT_0725800	231,0605396	214,3732398	201,1807451	250,129508	222,7168897	225,65513	1,013192699	0,0189086	0,8804205	0,981847881	mitochondrial inner membrane translocase subunit tim50, putative (TIM50)
PFIT_0104400	341,4397782	312,5777407	338,6542543	328,3785787	327,0087594	333,51642	1,019900559	0,0284285	0,8823749	0,981847881	DNA binding protein, putative
PFIT_1206200	4571,172203	5470,709269	5210,581299	5139,024437	5020,940736	5174,8029	1,030644084	0,0435462	0,8825374	0,981847881	DNA-directed RNA polymerase III subunit, putative
PFIT_0104000	44,15169546	43,11417113	40,23614903	50,82845617	43,63293329	45,532303	1,043530635	0,061473	0,8813072	0,981847881	conserved Plasmodium protein, unknown function
[illegible]	[illegible]	[illegible]	[illegible]	[illegible]	[illegible]	[illegible]	[illegible]	[illegible]	[illegible]	[illegible]	[illegible]

PFIT_1204700	690,2381723	726,9528298	723,4124294	707,5856135	708,5955011	715,49902	1,009742541	0,0139875	0,9732394	1	O-phosphoseryl-tRNA(Sec) selenium transferase, putative (SEPSECS)
PFIT_1363500	682,8795564	752,102763	733,4714666	715,6111592	717,4911597	724,54131	1,009826119	0,0141069	0,9905004	1	exosome complex exonuclease, putative
PFIT_0901200	2,943446364	1,197615865	0,838253105	3,34397738	2,070531114	2,0911152	1,009941472	0,0142717	1	1	rifin (RIF)
PF_apicoplast_g	2,943446364	1,197615865	0,838253105	3,34397738	2,070531114	2,0911152	1,009941472	0,0142717	1	1	unspecified product
PFIT_0208700	239,8908787	325,7515152	252,3141845	319,015442	282,8211969	285,66481	1,010054467	0,0144331	1	1	conserved protein, unknown function
PFIT_1348100	67,69926637	56,28794564	70,4132608	54,84122903	61,993606	62,627245	1,010221036	0,014671	0,9972659	1	conserved Plasmodium protein, unknown function
PFIT_0726100	918,3552655	1156,896925	1061,228431	1035,964192	1037,626095	1048,5963	1,010572417	0,0151727	0,9691767	1	cysteine desulfurase, putative
PFIT_0921200	19,13240137	13,17377451	19,27982141	13,37590952	16,15308794	16,327865	1,010820069	0,0155262	1	1	conserved Plasmodium protein, unknown function
PFIT_1356100	20945,56433	25649,33897	24298,44275	22813,28248	23297,45165	23555,863	1,011091812	0,015914	0,9670907	1	elongation factor 1-alpha
PFIT_1029600	3539,494253	3548,535807	3896,200431	3272,416264	3544,01503	3584,3083	1,011369398	0,01631	0,9756512	1	conserved Plasmodium protein, unknown function
PFIT_1106500	2849,25608	3443,145611	3242,363009	3122,606077	3146,200845	3182,4845	1,011532543	0,0165427	0,9845089	1	conserved protein, unknown function
PFIT_1349900	157,4743805	174,8519162	160,9445961	175,2244147	166,1631483	168,08451	1,011563076	0,0165863	0,9768796	1	N6-adenine-specific methylase, putative
PFIT_0519400	179,5502282	192,8161542	179,3861644	197,2946654	186,1831912	188,34041	1,011586565	0,0166198	0,9626249	1	cytochrome c oxidase assembly protein (heme A: farnesyltransferase), putative
PFIT_0203500	3507,116343	3373,683891	3711,784748	3249,008422	3440,400117	3480,3966	1,011625528	0,0166753	0,9450517	1	conserved Plasmodium protein, unknown function
PFIT_0826300	54,45375773	83,83311052	46,94217387	92,96257115	69,14343413	69,952373	1,011699424	0,0167807	0,9965893	1	alpha/beta-hydrolase, putative
PFIT_1323600	1293,644677	1349,713079	1461,913415	1212,526198	1321,678878	1337,2198	1,011758475	0,0168649	1	1	MSF1-like protein, putative
PFIT_0809300	23,54757091	23,95231729	29,33885867	18,72627333	23,7499441	24,032566	1,011899897	0,0170666	1	1	glycosyltransferase family 28 protein, putative
PFIT_1114900.2	622,538906	719,7671346	694,9118238	663,4451121	671,1530203	679,17847	1,011957702	0,017149	1	1	rhomboid protease ROM1
PFIT_0205500	6624,226042	6372,514015	7117,607112	6035,210375	6498,370029	6576,4087	1,012008968	0,0172221	0,9497764	1	metabolite/drug transporter, putative
PFIT_1021100	676,9926637	802,4026293	737,6627322	759,7516606	739,6976465	748,7072	1,012180044	0,0174659	0,9909478	1	deoxyribose-phosphate aldolase, putative
PFIT_1321100	3515,946682	3309,012634	4032,835687	2877,826933	3412,479658	3455,3313	1,012557336	0,0180036	1	1	conserved Plasmodium protein, unknown function
PFIT_0702700	107,4357923	128,1448975	128,252725	110,3512535	117,7903449	119,30199	1,012833347	0,0183968	0,9976508	1	conserved Plasmodium protein, unknown function
PFIT_0505200	1236,247473	1207,196792	1301,807072	1173,067265	1221,722132	1237,4372	1,01286302	0,0184391	0,940953	1	actin-like protein, putative
PFIT_1202200	491,5555428	573,6579991	494,5693318	584,527246	532,606771	539,54829	1,013033101	0,0186813	0,9490397	1	trimethylguanosine synthase, putative
PFIT_1119300	4630,04113	5286,276426	5154,418341	4892,907702	4958,158778	5023,663	1,013211405	0,0189352	0,9860856	1	box C/D snoRNP rRNA 2'-O-methylation factor, putative
PFIT_0522400	153,0592109	166,4686052	186,9304424	137,1030726	159,763908	162,01676	1,014101116	0,0202015	0,992328	1	conserved Plasmodium protein, unknown function, putative
PFIT_1474600	644,6147537	752,102763	751,9130349	664,7827031	698,3587583	708,34787	1,014303695	0,0204897	0,998574	1	conserved Plasmodium protein, unknown function
PFIT_1241700	69,17098955	51,49748218	66,22199527	56,17881998	60,33423586	61,200408	1,014356223	0,0205644	0,9504344	1	conserved Plasmodium protein, unknown function
PFIT_1247400	219,2867541	250,3017157	248,9611721	227,3904618	234,7942349	238,17582	1,014402321	0,02063	1	1	conserved Plasmodium protein, unknown function
PFIT_1339600	1,471723182	9,580926917	5,867771733	5,350363807	5,526325049	5,6090678	1,014972467	0,0214406	1	1	conserved Plasmodium protein, unknown function
PFIT_0810500	6583,017793	7119,826315	6995,222159	6922,033176	6851,422054	6958,6277	1,015647206	0,0223994	0,9348971	1	conserved Plasmodium protein, unknown function
PFIT_1456000	69,17098955	85,03072639	109,8111567	46,81568331	77,10085797	78,31342	1,015726959	0,0225126	0,9518183	1	conserved Plasmodium protein, unknown function
PFIT_0112100	2141,35723	2728,16894	2506,376783	2439,765896	2434,763085	2473,0713	1,015733874	0,0225225	1	1	heat shock protein 40, type II
PFIT_1308800	367,9307955	397,608467	382,2434158	395,9269217	382,7696313	389,08517	1,016499578	0,0236096	0,9381246	1	ribosomal RNA methyltransferase, putative
PFIT_0531100	1146,472359	1526,960227	1529,811916	1189,118356	1336,716293	1359,4651	1,017018453	0,0243459	0,9519588	1	transmembrane emp24 domain-containing protein, putative
PFIT_0905500	734,3898678	803,6002451	787,9579185	776,4715475	768,9950565	782,21473	1,017190847	0,0245904	0,9411836	1	dynein heavy chain, putative
PFIT_0825500	14774,62902	16119,90954	16615,01479	14815,15738	15447,26928	15715,086	1,017337485	0,0247983	0,9551842	1	protein transport protein Sec23
PFIT_0729700.2	7823,680435	9306,672884	8851,114533	8579,977161	8565,176659	8715,5458	1,017555877	0,025108	0,9690323	1	Plasmodium exported protein, unknown function
PFIT_0932800	697,5967882	770,0670009	751,0747818	742,3629783	733,8318946	746,71888	1,017561223	0,0251156	0,9420988	1	M18 aspartyl aminopeptidase
PFIT_0834600	49411,63411	57910,71513	60377,69463	48836,11445	53661,17462	54606,905	1,0176241	0,0252047	0,9901757	1	heat shock protein 70 (HSP70-x)
PFIT_1335700	370,8742419	373,6561498	450,9801703	306,9771234	372,2651958	378,97865	1,018034055	0,0257858	1	1	RanBPM and CLTH-like protein, putative
PFIT_bin06700	2,943446364	4,790463458	5,867771733	2,006386428	3,866954911	3,9370791	1,018134209	0,0259277	1	1	rifin (RIF)
PFIT_0609700	8831,810815	10109,07551	10846,15692	8441,536497	9470,443164	9643,8467	1,018309972	0,0261768	0,9943857	1	transketolase
PFIT_1119900	404,723875	414,3750891	455,1714359	379,2070348	409,5494821	417,18924	1,018654042	0,0266642	0,9573375	1	tRNA m(1)G methyltransferase, putative
PFIT_1124500	419,4411069	544,9152184	475,2895104	507,6157662	482,1781626	491,45264	1,019234541	0,0274861	0,9777328	1	RAP protein, putative
PFIT_1144000	1003,71521	1279,053743	1228,879052	1099,499762	1141,384477	1164,1894	1,01998006	0,0285409	1	1	DNA-directed RNA polymerase I, putative
PFIT_1351800	17,66067818	9,580926917	11,73554347	16,05109142	13,62080255	13,893317	1,020007257	0,0285794	0,9525128	1	conserved Plasmodium protein, unknown function
PFIT_0712500	88,30339092	76,64741533	94,72260084	73,56750235	82,47540313	84,145052	1,020244199	0,0289145	0,9620006	1	calmodulin, putative
PFIT_0908100	519,5182832	518,5676694	579,2328954	480,1951517	519,0429763	529,71402	1,020559082	0,0293597	0,9348833	1	peptide deformylase
PFIT_0322100	7502,844782	8698,284024	8929,910325	7605,542152	8100,564403	8267,7262	1,020635826	0,0294682	0,9728927	1	peptidyl-prolyl cis-trans isomerase
PFIT_1010200	1243,606089	1695,824064	1430,89805	1569,662982	1469,715076	1500,2805	1,020796847	0,0296958	0,9823125	1	dolichyl-phosphate-mannose protein mannosyltransferase, putative
PFIT_1415300	10422,74357	12346,22195	12022,22603	11224,39447	11384,48276	11623,31	1,020978335	0,0299523	0,9546386	1	60S ribosomal protein L10, putative
PFIT_1448200	6262,182139	7566,537032	7281,066468	6839,771332	6914,359586	7060,4189	1,021124055	0,0301581	0,9610035	1	40S ribosomal protein S2, putative
PFIT_1337500	7494,014442	8325,825491	9095,884439	7095,919999	7909,919967	8095,9022	1,023512533	0,0335288	0,9662404	1	elongation factor 1-gamma, putative
PFIT_1015900	7663,262608	8556,965352	9324,727537	7279,16996	8110,11398	8301,9487	1,02365377	0,0337278	0,9673299	1	serine/threonine protein kinase, FIKK family
PFIT_0519200	337,0246087	376,0513815	432,538602	297,6139868	356,5379951	365,07629	1,023947796	0,0341422	1	1	mitochondrial ribosomal protein L14 precursor, putative
PFIT_1304000	2,943446364	0	1,676506209	1,337590952	1,471723182	1,5070486	1,024002747	0,0342196	1	1	small nucleolar RNA snoR16
PFIT_1401100	1,471723182	0	0,838253105	0,668795476	0,735861591	0,7535243	1,024002747	0,0342196	1	1	Plasmodium exported protein, unknown function, fragment
PFIT_0730000	27980,40114	32167,96212	34017,14924	27771,06334	30074,18163	30894,106	1,027263407	0,0388062	0,9420474	1	Plasmodium exported protein (PHISTc), unknown function
PFIT_1113900	6378,448271	7009,645655	7977,654798	5782,405685	6694,046963	6880,0302	1,027783384	0,0395362	0,964819	1	protein tyrosine phosphatase
PFIT_1116300	560,7265323	544,9152184	685,6910397	450,7681508	552,8208754	568,2296	1,027872898	0,0396619	0,9613442	1	transcription factor with AP2 domain(s), putative
PFIT_0929200	1908,824967	1729,357308	2261,606877	1482,050775	1819,091138	1871,8288	1,028991229	0,0412307	0,9401537	1	conserved Plasmodium protein, unknown function
PFIT_1478500	5,886892728	5,988079323	7,544277943	4,681568331	5,937486025	6,1129231	1,029547373	0,0420102	1	1	probable protein, unknown function
PFIT_0622600	475,3665878	524,5557487	601,0274761	430,035491	499,9611682	515,53148	1,031143049	0,0442445	0,9607384	1	ATP dependent DEAD-box helicase, putative
PFIT_1367000	17,66067818	19,16185383	9,220784152	28,75820546	18,41126601	18,989495	1,031406249	0,0446127	0,9467527	1	conserved Plasmodium protein, unknown function
PFIT_1036300	236,9474323	258,6850268	288,359068	223,377689	247,8162295	255,86838	1,03249242	0,0461312	0,9344029	1	pyruvate kinase 2, putative
PFIT_0502900	119,2095777	170,0614528	142,5030278	156,4981414	144,6355153	149,50058	1,033636755	0,0477293	0,9520276	1	DnaJ protein, putative
PFIT_0104900	915,4118192	976,0569296	1140,024222	815,9304806	945,7343744	977,97735	1,034093058	0,048366	0,9344638	1	pre-rRNA-processing protein TSR2, putative
PFIT_0413100	201,6260759	253,8945633	256,50545	215,3521432	227,7603196	235,9288	1,035864355	0,0508351	0,9471922	1	RNA binding protein, putative
PFIT_0318000	11,77378546	17,96423797	20,11807451	10,70072761	14,86901171	15,409401	1,036343327	0,051502	1	1	conserved Plasmodium protein, unknown function
PFIT_bin00600	8,830339092	3,592847594	7,544277943	5,350363807	6,211593343	6,4473209	1,037949608	0,0537364	1	1	stevor
PFIT_0815600	806,5043037	810,7859403	1102,302833	579,8456776	808,645122	841,07426	1,040103047	0,0567265	0,9726896	1	RNA-binding protein (U1 snRNP-like), putative
PFIT_1318800	2030,977991	2081,456373	2741,925906	1537,560799	2056,217182	2139,7434	1,040621278	0,0574451	0,9636028	1	conserved Plasmodium protein, unknown function
PFIT_1025100	20,60412455	26,34754902	24,30934004	24,74543261	23,47583678	24,527386	1,044792846	0,0632169	0,9870415	1	cytochrome c oxidase copper chaperone, putative (COX17)
PFIT_0700250	4,415169546	5,988079323	4,191265524	6,687954759	5,201624434	5,4396101	1,04575219	0,064541	1	1	rifin
PFIT_0523800	4,415169546	8,383311052	6,706024838	6,687954759	6,399240299	6,6969898	1,046528882	0,0656121	1	1	conserved Plasmodium protein, unknown function, putative
PFIT_1401900	45,62341864	69,46172015	57,00121112	63,53557021	57,54256939	60,268391	1,047370517	0,0667719	0,9481051	1	erythrocyte membrane protein 1, PfEMP1, putative
PFIT_0711100	20,60412455	28,74278075	24,30934004	27,42061451	24,67345265	25,864977	1,048291767	0,0680403	0,9773806	1	rifin
PFIT_0605300	17,66067818	25,14993316	26,82409935	18,05747785	21,40530567	22,440789	1,048375059	0,0681549	1	1	conserved Plasmodium protein, unknown function
PFIT_1401500	11,77378546	9,580926917	9,220784152	13,37590952	10,67735619	11,298347	1,058159589	0,0815572	0,9495163	1	rifin
PFIT_0312500	1,471723182	3,592847594	3,353012419	2,006386428	2,532285388	2,6796994	1,058213832	0,0816312	1	1	tRNA Valine
PFIT_1149900	10,30206227	9,580926917	11,73554347	9,363136663	9,941494595	10,54934	1,061142262	0,0856181	1	1	rifin (RIF)
PFIT_1214400	8,830339092	9,580926917	10,89729036	8,694341187	9,205633004	9,7958158	1,064111047	0,0896487	1	1	conserved Plasmodium protein, unknown function
PFIT_bin01200	2,943446364	0	2,514759314	0,668795476	1,471723182	1,5917774	1,081573909	0,1131323	1	1	rifin (RIF)
PFIT_1478300	7,35861591	8,383311052	5,029518628	12,03831857	7,870963481	8,5339186	1,08422795	0,1166681	0,9464142	1	Plasmodium exported protein (hyp15), unknown function
PFIT_1006700	13,24550864	20,3594697	18,4415683	18,05747785	16,80248917	18,249523	1,086120211	0,1191838	0,9551407	1	conserved Plasmodium protein, unknown function
PFIT_1102200	8,830339092	10,77854278	10,89729036	10,70072761	9,804440936	10,799009	1,101440567	0,1393917	0,9587856	1	erythrocyte membrane protein 1 (PfEMP1), exon2,, putative
PFIT_0109700	7,35861591	4,790463458	6,706024838	6,687954759	6,074539684	6,6969898	1,102468688	0,1407377	0,9633188	1	conserved Plasmodium protein, unknown function
PFIT_bin01100	7,35861591	9,580926917	10,05903726	8,694341187	8,469771413	9,3766892	1,107077011	0,1467556	0,9796968	1	rifin (RIF)
PFIT_1400600	0	1,197615865	0	1,337590952	0,598807932	0,6687955	1,116878117	0,1594718	1	1	Plasmodium exported protein, unknown function, fragment
PFIT_0733800	0	1,197615865	0	1,337590952	0,598807932	0,6687955	1,116878117	0,1594718	1	1	stevor
PFIT_0734200	0	1,197615865	0	1,337590952	0,598807932	0,6687955	1,116878117	0,1594718	1	1	rifin (RIF)
PFIT_bin03800	1,471723182	1,197615865	1,676506209	1,337590952	1,334669523	1,5070486	1,129154861	0,1752434	1	1	stevor
PFIT_0200200	1,471723182	0	1,676506209	0	0,735861591	0,8382531	1,139145072	0,1879515	1	1	rifin (RIF)
PFIT_1327100	1,471723182	8,383311052	3,353012419	8,025545711	4,927517117	5,6892791	1,154593466	0,207385	1	1	conserved Plasmodium protein, unknown function
PFIT_1220500	4,415169546	8,383311052	7,544277943	7,356750235	6,399240299	7,4505141	1,16428103	0,2194393	0,9614875	1	conserved Plasmodium protein, unknown function
PFIT_0801700	1,471723182	2,395231729	2,514759314	2,006386428	1,933477456	2,2605729	1,169174673	0,2254905	1	1	Plasmodium exported protein, unknown function
PF_apicoplast_g	0	2,395231729	0,838253105	2,006386428	1,197615865	1,4223198	1,187626023	0,2480806	1	1	unspecified product
PFIT_1322000	1,471723182	1,197615865	2,514759314	0,668795476	1,334669523	1,5917774	1,192637853	0,254156	1	1	conserved Plasmodium protein, unknown function
PFIT_0401300	0	3,592847594	1,676506209	2,675181904	1,796423797	2,1758441	1,211208658	0,2764474	1	1	stevor
PFIT_1000300	2,943446364	5,988079323	4,191265524	6,687954759	4,465762843	5,4396101	1,218069641	0,2845966	0,9354175	1	erythrocyte membrane protein 1 (PfEMP1), pseudogene
PFIT_bin09900	1,471723182	7,185695188	5,867771733	4,681568331	4,328709185	5,27467	1,218531855	0,285144	1	1	rifin (RIF)
PFIT_0500400	0	4,790463458	3,353012419	2,675181904	2,395231729	3,0140972	1,258373929	0,3315607	1	1	rifin (RIF)
PFIT_bin05840	0	2,395231729	1,676506209	1,337590952	1,197615865	1,5070486	1,258373929	0,3315607	1	1	rifin (RIF)
PFIT_0731100	0	2,395231729	1,676506209	1,337590952	1,197615865	1,5070486	1,258373929	0,3315607	1	1	stevor
PFIT_bin05400	0	1,197615865	0,838253105	0,668795476	0,598807932	0,7535243	1,258373929	0,3315607	1	1	Plasmodium exported protein (PHISTa), unknown, putative
PFIT_1419400	0	1,197615865	0,838253105	0,668795476	0,598807932	0,7535243	1,258373929	0,3315607	1	1	tRNA Proline
PFIT_bin04000	1,471723182	2,395231729	1,676506209	3,34397738	1,933477456	2,5102418	1,298304145	0,3766284	0,9951493	1	rifin (RIF)
PFIT_1221900	2,943446364	3,592847594	5,867771733	2,675181904	3,268146979	4,2714768	1,307002667	0,3862621	0,9432861	1	small nucleolar RNA snoR15
PFIT_0423500	0	1,197615865	1,676506209	0	0,598807932	0,8382531	1,39986974	0,4852926	1	1	Plasmodium exported protein (PHISTa), unknown function, pseudogene
PFIT_1400500	0	1,197615865	1,676506209	0	0,598807932	0,8382531	1,39986974	0,4852926	1	1	rifin (RIF)
PFIT_bin03900	0	1,197615865	1,676506209	0	0,598807932	0,8382531	1,39986974	0,4852926	1	1	rifin (RIF)
PFIT_0733400	1,471723182	0	1,676506209	0,668795476	0,735861591	1,1726508	1,593575282	0,6722672	1	1	rifin
PFIT_0110900	0	2,395231729	2,514759314	1,337590952	1,197615865	1,9261751	1,608341364	0,6855736	1	1	5.8S ribosomal RNA
PFIT_0733600	0	1,197615865	0	2,006386428	0,598807932	1,0031932	1,675317176	0,7444343	1	1	rifin
PFIT_bin06800	1,471723182	0	2,514759314	0	0,735861591	1,2573797	1,708717607	0,772914	1	1	rifin (RIF)
PFIT_0811400	0	1,197615865	0,838253105	1,337590952	0,598807932	1,087922	1,816812987	0,8614099	1	1	Plasmodium RNA of unkown function RUF6-4
PFIT_1206900	0	1,197615865	0,838253105	1,337590952	0,598807932	1,087922	1,816812987	0,8614099	1	1	conserved Plasmodium protein, unknown function
PFIT_1004800	0	1,197615865	0,838253105	1,337590952	0,598807932	1,087922	1,816812987	0,8614099	1	1	conserved Plasmodium protein, unknown function
PFIT_bin04800	0	1,197615865	1,676506209	0,668795476	0,598807932	1,1726508	1,958308799	0,9696083	1	1	rifin (RIF)
PFIT_0300500	0	1,197615865	1,676506209	0,668795476	0,598807932	1,1726508	1,958308799	0,9696083	1	1	rifin (RIF)
PFIT_0802200	0	1,197615865	1,676506209	0,668795476	0,598807932	1,1726508	1,958308799	0,9696083	1	1	Plasmodium exported protein, unknown function, pseudogene
PFIT_0705700	0	0	0	1,337590952	0	0,6687955	#DIV/0!	#DIV/0!	0,9437054	1	tRNA Threonine
PF_apicoplast_g	0	0	0	1,337590952	0	0,6687955	#DIV/0!	#DIV/0!	0,9437054	1	unspecified product
PFIT_0731600	0	0	0,838253105	0	0	0,4191266	#DIV/0!	#DIV/0!	1	1	rifin (RIF)
PFIT_0801500	0	0	0,838253105	0	0	0,4191266	#DIV/0!	#DIV/0!	1	1	rifin (RIF)
PFIT_0900900	0	0	0	0,668795476	0	0,3343977	#DIV/0!	#DIV/0!	1	1	stevor
PFIT_1425000	0	0	0,838253105	0	0	0,4191266	#DIV/0!	#DIV/0!	1	1	small nucleolar RNA snoR24
PFIT_bin01300	0	0	0	0,668795476	0	0,3343977	#DIV/0!	#DIV/0!	1	1	stevor
PFIT_0218400	0	0	0	0,668795476	0	0,3343977	#DIV/0!	#DIV/0!	1	1	small nucleolar RNA snoR01
PFIT_0733900	0	0	0,838253105	0	0	0,4191266	#DIV/0!	#DIV/0!	1	1	rifin (RIF)
PFIT_1331100	0	0	0,838253105	0	0	0,4191266	#DIV/0!	#DIV/0!	1	1	small nucleolar RNA snoR18
PFIT_1340200	0	0	0,838253105	0	0	0,4191266	#DIV/0!	#DIV/0!	1	1	tRNA Arginine
PFIT_1401000	0	0	0	0,668795476	0	0,3343977	#DIV/0!	#DIV/0!	1	1	Plasmodium exported protein, unknown function, fragment
PFIT_1439400	0	0	0	0,668795476	0	0,3343977	#DIV/0!	#DIV/0!	1	1	tRNA Selenocysteine
Pf_M76611002(	0	0	0	0,668795476	0	0,3343977	#DIV/0!	#DIV/0!	1	1	unspecified product
PFIT_0312600	0	0	0	0,668795476	0	0,3343977	#DIV/0!	#DIV/0!	1	1	tRNA Isoleucine
PFIT_0705900	0	0	0	0,668795476	0	0,3343977	#DIV/0!	#DIV/0!	1	1	tRNA Lysine
PFIT_1110200	0	0	0,838253105	0	0	0,4191266	#DIV/0!	#DIV/0!	1	1	small nucleolar RNA snoR09
PFIT_1369000	0	0	0	0,668795476	0	0,3343977	#DIV/0!	#DIV/0!	1	1	tRNA Arginine
PFIT_bin10300	0	0	0,838253105	0	0	0,4191266	#DIV/0!	#DIV/0!	1	1	B_ITvar19 - erythrocyte membrane protein 1, PfEMP1, putative
PF_apicoplast_g	0	0	0,838253105	0	0	0,4191266	#DIV/0!	#DIV/0!	1	1	unspecified product
PFIT_0113400	0	0	0	0,668795476	0	0,3343977	#DIV/0!	#DIV/0!	1	1	stevor, pseudogene
PFIT_0200400	0	0	0	0,668795476	0	0,3343977	#DIV/0!	#DIV/0!	1	1	Plasmodium exported protein (PHISTa), unknown function
PFIT_0410100	0	0	0,838253105	0	0	0,4191266	#DIV/0!	#DIV/0!	1	1	tRNA Alanine
PF_apicoplast_g	0	0	0,838253105	0	0	0,4191266	#DIV/0!	#DIV/0!	1	1	unspecified product
PFIT_0510600	0	0	0	0,668795476	0	0,3343977	#DIV/0!	#DIV/0!	1	1	tRNA Leucine
PFIT_bin06600	0	0	0	0,668795476	0	0,3343977	#DIV/0!	#DIV/0!	1	1	stevor
PF_apicoplast_g	0	0	0	0,668795476	0	0,3343977	#DIV/0!	#DIV/0!	1	1	unspecified product
PFIT_0300700	0	0	0	0	0	0	#NV	#NV	#NV	#NV	rifin (RIF)
PFIT_0419800	0	0	0	0	0	0	#NV	#NV	#NV	#NV	erythrocyte membrane protein 1 (PfEMP1)-like pseudogene
PFIT_0706000	0	0	0	0	0	0	#NV	#NV	#NV	#NV	tRNA Lysine
PFIT_1400900	0	0	0	0	0	0	#NV	#NV	#NV	#NV	Plasmodium exported protein, unknown function, fragment
PFIT_0619800	0	0	0	0	0	0	#NV	#NV	#NV	#NV	tRNA Leucine
PFIT_0729600	0	0	0	0	0	0	#NV	#NV	#NV	#NV	tRNA Threonine
PFIT_0801300	0	0	0	0	0	0	#NV	#NV	#NV	#NV	Plasmodium exported protein (PHISTa), unknown function, pseudogene
PFIT_1252100	0	0	0	0	0	0	#NV	#NV	#NV	#NV	tRNA Valine
PFIT_1354500	0	0	0	0	0	0	#NV	#NV	#NV	#NV	tRNA Threonine
PFIT_1368800	0	0	0	0	0	0	#NV	#NV	#NV	#NV	tRNA Arginine
PFIT_0423800	0	0	0	0	0	0	#NV	#NV	#NV	#NV	stevor, pseudogene
PFIT_1038600	0	0	0	0	0	0	#NV	#NV	#NV	#NV	Plasmodium exported protein (hyp10), unknown, putative
PFIT_1338300	0	0	0	0	0	0	#NV	#NV	#NV	#NV	tRNA Methionine
Pf_M76611025(	0	0	0	0	0	0	#NV	#NV	#NV	#NV	unspecified product

Table S3. Differentially expressed genes (threshold, ≥3-fold; padj ≤ 0.05) in FCR3 knobless IEs cultivated in the presence of human serum and AlbuMAX.

	HS_K-_1	HS_K-_2	HS_K-_3	HS_K-_4	HS_K-_5	HS_K-_6	AlbuMAX_K-_1	AlbuMAX_K-_2	baseMeanA	baseMeanB	foldChange	log2FoldCh	pval	padj	name
PFIT_bin11(	440,3743766	203,5748323	208,8173055	185,9571737	219,6714278	428,6736289	33132,15548	40529,28908	281,17812	36830,722	130,98715	7,0332815	2,601E-31	1,471E-27	erythrocyte membrane protein 1, PfEMP1, putative
PFIT_12405	1908,666321	1297,551735	1060,293244	753,2372421	915,9884063	2875,321075	20234,48652	25275,68846	1468,5097	22755,087	15,495361	3,9537645	8,196E-14	2,318E-10	erythrocyte membrane protein 1, PfEMP1
PFIT_13050	80,37681425	90,68909976	27,42675057	29,33253037	15,19739437	5,832294271	678,4454858	747,2235043	41,475814	712,8345	17,186751	4,103225	2,01E-11	3,789E-08	conserved Plasmodium protein, unknown function
PFIT_10306	132,4519333	142,6926395	29,29675629	56,45128486	164,4081755	122,4781797	1224,80424	1191,954245	107,96316	1208,3792	11,192514	3,4844623	1,708E-10	2,416E-07	MORN repeat-containing protein 1
PFIT_10117	443,7705801	532,0849979	208,1939703	606,5745902	889,7383615	209,9625937	6203,273238	5085,481794	481,72085	5644,3775	11,717113	3,5505452	3,763E-10	4,256E-07	conserved Plasmodium protein, unknown function
PFIT_11168	974,7103812	1508,736841	539,1849829	1267,940133	6139,747327	2452,479741	20451,82923	17847,07306	2147,1332	19149,451	8,9186134	3,1568194	5,603E-10	5,282E-07	rhoptry neck protein 4
PFIT_07204	1421,877165	1153,590716	461,8914131	1212,042293	2251,97753	941,9155247	13848,69343	11552,56847	1240,5491	12700,631	10,237911	3,3558494	7,445E-10	6,015E-07	rhoptry-associated leucine zipper-like protein 1
PFIT_07273	0	5,07351607	13,71337529	13,28265526	5,526325227	0	138,0906741	153,6170148	6,2659786	145,85384	23,277105	4,5408398	1,991E-09	1,408E-06	conserved Plasmodium protein, unknown function
PFIT_03024	4298,46146	13724,49516	4532,893867	11537,64643	26276,29487	7404,097577	122533,2587	99211,937	11295,648	110872,6	9,8155144	3,2950639	2,241E-09	1,409E-06	cytoadherence linked asexual protein 3.1 (CLAG3.1)
PFIT_08276	41,88650883	89,42072074	201,9606179	231,3395791	140,9212933	23,32917708	1627,068377	1344,623006	121,47632	1485,8457	12,231567	3,6125373	4,808E-09	2,719E-06	conserved Plasmodium membrane protein, unknown function
PFIT_03040	158,4894929	528,2798608	95,37029176	200,3467168	345,3953267	131,2266211	2639,333058	2345,030109	243,18472	2492,1816	10,248101	3,3572847	6,127E-09	3,097E-06	membrane skeletal protein IMC1-related
PFIT_13343	1798,855744	5377,927034	2232,786831	6558,311035	8593,435727	2251,265589	49645,39852	39191,30385	4468,7637	44418,351	9,9397405	3,3132082	6,571E-09	3,097E-06	merozoite surface protein 7
PFIT_14114	3692,805184	7212,003094	2674,108181	6591,517673	18102,85986	2770,339779	65450,17637	56486,49356	6840,6056	60968,335	8,9127101	3,1558642	7,462E-09	3,247E-06	rhoptry-associated protein 1
PFIT_10351	1458,103334	1445,317891	630,1919279	1419,583781	2129,016794	1224,781797	12843,63348	11635,06649	1384,4993	12239,35	8,840272	3,1440908	8,768E-09	3,542E-06	duffy binding-like merozoite surface protein
PFIT_10347	2930,92355	5550,426581	1382,557563	3732,979572	5603,69378	1598,04863	34806,0546	29673,6867	3466,4383	32239,871	9,3005754	3,21732	1,376E-08	5,19E-06	glutamate-rich protein
PFIT_06084	80,37681425	121,1301962	188,8705778	182,0830659	203,0924521	116,6458854	1562,2258	1334,192221	148,69983	1448,209	9,7391436	3,2837949	1,579E-08	5,582E-06	conserved Plasmodium protein, unknown function
PFIT_05364	96,22576353	279,0433839	316,0309668	290,5580838	125,7238989	183,7172695	3,602365411	8,534278602	215,21656	6,068322	0,0281964	-5,148348	1,8E-08	5,987E-06	stevor, pseudogene
PFIT_07273	0	10,14703214	17,45338673	10,51543541	16,57897568	8,748441406	133,2875202	167,8408125	10,573879	150,56417	14,239256	3,8318019	2,182E-08	6,857E-06	conserved Plasmodium protein, unknown function
PFIT_13554	46,41478006	188,3542841	74,80022884	154,4108674	473,8823882	104,9812969	1511,792684	1302,899867	173,80731	1407,3463	8,097164	3,0174167	4,279E-08	1,254E-05	ubiquitin conjugating enzyme, putative
PFIT_12283	5840,337813	3141,774826	1122,003433	2377,595292	10635,4129	2131,703556	32285,59961	27571,4094	4208,138	29928,505	7,112054	2,8302663	4,625E-08	1,254E-05	merozoite surface protein 9
PFIT_07062	2499,605716	4150,136145	923,7828261	2721,837441	7921,987212	2525,383419	28339,80869	24115,97482	3457,1221	26227,892	7,5866258	2,9234584	4,655E-08	1,254E-05	rhoptry-associated membrane antigen
PFIT_12523	1592,819403	3848,261939	1500,991259	3587,977252	12984,10112	2650,777746	35472,49221	28760,51889	4360,8215	32116,506	7,3647834	2,8806431	5,18E-08	1,332E-05	rhoptry neck protein 3 (RON3)
PFIT_10349	2830,169516	3856,506403	976,1429863	2491,051305	2370,793522	1017,73535	21144,68418	17798,71215	2257,0665	19471,698	8,6269935	3,1088579	6,136E-08	1,509E-05	merozoite surface protein 6
PFIT_05268	16,98101709	71,66341449	24,31007437	43,72207357	192,0398016	29,16147135	576,3784658	469,3853231	62,979642	522,88189	8,3023955	3,0535277	6,622E-08	1,518E-05	inner membrane complex protein
PFIT_05325	167,5460353	293,6297426	105,3436556	226,3585834	360,592721	177,8849753	1945,277322	1685,045897	221,89262	1815,1616	8,1803605	3,0321644	6,711E-08	1,518E-05	conserved Plasmodium protein, unknown function
PFIT_05248	24,90549174	71,02922498	23,68673913	42,61518563	167,1713381	20,41302995	533,1500809	458,006285	58,303502	495,57818	8,4999729	3,0874582	7,251E-08	1,565E-05	conserved protein, unknown function, putative
PFIT_10348	1245,274587	2582,41968	572,8450858	1354,830837	2093,09568	898,1733177	12177,19588	11560,15449	1457,7732	11868,675	8,1416473	3,0253207	7,469E-08	1,565E-05	merozoite surface protein 3
PFIT_05228	22,64135613	65,3215194	32,4134325	40,40140975	189,276639	26,24532422	543,9571771	468,4370699	62,716614	506,19712	8,0711807	3,0127797	9,616E-08	1,942E-05	conserved protein, unknown function, putative
PFIT_09296	4161,481256	17327,32576	3848,471774	9207,647316	40411,25322	7258,29022	102704,6387	86863,78412	13702,412	94784,211	6,9173379	2,7902169	1,524E-07	2,973E-05	high molecular weight rhoptry protein 2
PFIT_14532	1123,011264	1801,732394	921,9128204	1809,208335	5624,417499	1288,937034	15257,21831	13591,3128	2094,8699	14424,266	6,8855186	2,7835653	1,637E-07	3,085E-05	rhoptry neck protein 2
PFIT_05017	332,827935	1806,171721	569,1050744	1217,023288	4427,968088	536,5710729	10837,11595	10189,92865	1481,6112	10513,522	7,0960062	2,8270073	1,714E-07	3,128E-05	rhoptry-associated protein 2
PFIT_14504	168,6781031	536,8644497	162,0671625	337,0473772	1910,726947	142,8912096	3882,149125	3387,160352	539,71254	3634,6547	6,734427	2,7515552	1,78E-07	3,147E-05	conserved Plasmodium protein, unknown function
PFIT_10136	1467,159877	2365,526868	796,6224371	2150,683264	2275,464412	621,1393398	14379,44193	11630,32523	1612,766	13004,884	8,0637137	3,0114444	1,854E-07	3,178E-05	conserved Plasmodium protein, unknown function
PFIT_11195	115,4709162	148,4003451	51,1134897	79,69593156	245,9214726	34,99376562	878,9771604	836,359303	112,59932	857,66823	7,616993	2,9292216	2,031E-07	3,379E-05	conserved Plasmodium protein, unknown function
PFIT_14626	870,560143	1321,016747	430,724651	1152,270344	1318,028567	393,6798633	7776,306135	6589,411334	914,38005	7182,8587	7,8554412	2,9736923	2,144E-07	3,464E-05	conserved Plasmodium protein, unknown function
PFIT_11459	1844,138456	1639,37988	555,3916991	1207,061297	2713,425686	915,6702005	10909,16325	9619,080238	1479,1779	10264,122	6,939072	2,7947427	2,298E-07	3,61E-05	serine/threonine protein kinase, putative
PFIT_14104	277,3566125	865,6686795	167,0538444	279,4892044	1226,8442	212,8787409	3732,050566	3445,003796	504,88188	3588,5272	7,1076569	2,829374	2,432E-07	3,686E-05	conserved Plasmodium membrane protein, unknown function
PFIT_07319	2608,284226	3510,873121	876,4093479	2329,445666	1834,739975	781,5274323	15892,43541	15411,01065	1990,2133	15651,723	7,8643445	2,9753265	2,476E-07	3,686E-05	erythrocyte binding like protein 1, pseudogene
PFIT_09305	11727,09041	33147,81724	12709,80555	39111,33186	49962,12479	13641,7363	219891,9871	185469,7873	26716,651	202680,89	7,5863134	2,923399	2,807E-07	4,07E-05	merozoite surface protein 1
PFIT_02063	553,5811573	840,9352886	331,6143478	691,2515175	2164,937908	609,4747513	6119,218045	5200,220428	865,29916	5659,7192	6,5407659	2,7094596	3,452E-07	4,879E-05	merozoite surface protein 4
PFIT_12418	22,64135613	15,22054821	59,21684783	63,09261249	69,07906533	61,23908984	397,4609837	369,8187394	48,41492	383,63986	7,9240007	2,986229	3,606E-07	4,879E-05	mitochondrial carrier protein, putative
PFIT_04217	971,3141778	1569,619034	626,4519165	1148,396236	4524,678779	1014,819203	11157,72647	9785,972797	1642,5466	10471,85	6,3753746	2,6725101	3,683E-07	4,879E-05	glideosome associated protein with multiple membrane spans 2
PFIT_02061	7888,248474	6709,090813	3766,814857	6361,838426	7613,894581	3505,208857	47417,93591	38587,26657	5974,1827	43002,601	7,1980727	2,8476107	3,71E-07	4,879E-05	merozoite surface protein 2
PFIT_bin06	103,0181704	26,63595937	30,54342677	17,15676305	9,671069147	58,32294271	272,5789828	311,9752956	40,891389	292,27714	7,1476452	2,837468	4,059E-07	5,217E-05	B_ITvar54 - erythrocyte membrane protein 1, PfEMP1, putative
PFIT_08206	902,2580416	2371,234573	660,7353547	1478,802286	5269,351104	1443,492832	13884,71708	11988,76493	2020,979	12936,741	6,4012248	2,678348	4,353E-07	5,471E-05	rhoptry neck protein 5
PFIT_05158	553,5811573	2399,138912	748,0022884	1546,875894	6244,747506	1230,614091	14634,00909	12490,39086	2120,4933	13562,2	6,3957759	2,6771194	4,494E-07	5,525E-05	glideosome-associated protein 40, putative
PFIT_06137	442,6385123	192,7936107	108,4603318	171,0141865	284,6057492	78,73597266	1635,473897	1317,123664	213,04139	1476,2988	6,9296335	2,7927791	5,186E-07	6,241E-05	conserved Plasmodium protein, unknown function
PFIT_14774	899,993906	411,5889912	92,8769508	198,132941	1015,46226	244,9563594	3164,07762	2524,24996	477,16857	2844,1638	5,9605011	2,5754336	6,458E-07	7,61E-05	Plasmodium exported protein (PHISTb), unknown function
PFIT_10133	472,0722752	521,9379657	208,8173055	441,6482874	754,3433934	317,8600378	3407,837679	2655,108898	452,77988	3031,4733	6,6952474	2,7431374	7,368E-07	8,505E-05	conserved Plasmodium protein, unknown function
PFIT_13605	253,5831886	773,0770112	181,3905549	366,3799076	607,8957749	128,310474	2941,931753	2636,143835	385,10615	2789,0378	7,2422572	2,8564394	8,603E-07	9,731E-05	conserved Plasmodium protein, unknown function
PFIT_14737	155,0932895	461,0557729	152,0937986	375,2350111	896,646268	198,2980052	2545,671557	2300,46221	373,07036	2423,0669	6,4949328	2,6993146	1,037E-06	0,000115	protein disulfide isomerase
PFIT_14650	391,695461	1091,440145	540,4316533	1001,733584	3002,176179	729,0367838	7540,951594	6227,17862	1126,0856	6884,0651	6,1132696	2,6119442	1,136E-06	0,00012	conserved Plasmodium membrane protein, unknown function
PFIT_14314	204,9042729	474,3737526	189,493913	269,527213	798,5539952	250,7886536	2492,836865	2125,983625	364,60697	2309,4102	6,3339718	2,6631104	1,142E-06	0,00012	plasmepsin IX
PFIT_09056	6538,823649	15356,26477	3220,149851	8234,692818	25618,66217	5738,977562	73435,4197	60686,30689	10784,595	67060,863	6,2182087	2,636499	1,145E-06	0,00012	high molecular weight rhoptry protein 3
PFIT_04046	830,9377698	802,8839181	412,6479291	728,8857074	1902,437459	504,4934544	5935,497409	4676,784674	863,71437	5306,141	6,1433979	2,6190368	1,225E-06	0,0001245	apical sushi protein
PFIT_02123	59,99959373	271,4331098	99,11030321	171,0141865	765,3960439	157,4719453	1643,879416	1406,259463	254,07086	1525,0694	6,0025357	2,5855721	1,249E-06	0,0001245	Leu/Phe-tRNA protein transferase, putative
PFIT_05016	105,282306	463,5925309	152,0937986	229,1258032	1092,830814	90,4005612	2411,183249	2032,10656	355,5543	2221,6449	6,2483983	2,6434864	1,255E-06	0,0001245	rhoptry-associated protein 3
PFIT_10308	175,47051	342,4623347	82,90358696	183,7433978	411,7112294	116,6458854	1480,572184	1395,828678	218,82282	1438,2004	6,5724425	2,7164296	1,289E-06	0,0001257	OTU-like cysteine protease, putative
PFIT_09327	1644,894523	1938,717328	743,0156064	1808,101447	3581,058747	638,6362227	11642,84501	10326,47711	1725,7373	10984,661	6,3651988	2,6702056	1,351E-06	0,0001295	profilin, putative
PFIT_03021	329,4317316	348,8042298	129,0303947	261,7789974	878,685711	107,897444	2161,419247	2007,451978	342,60475	2084,4356	6,0840826	2,6050397	1,433E-06	0,0001351	cytoadherence linked asexual protein 3.2
PFIT_08205	669,0520735	1358,433928	373,3778089	752,6837981	1909,345366	387,847569	6466,245913	5118,670655	908,45676	5792,4583	6,3761519	2,672686	1,568E-06	0,0001454	conserved Plasmodium protein, unknown function
PFIT_11411	172,0743066	366,5615361	190,1172483	356,4179162	1418,884002	449,0866589	2953,939637	2494,854111	492,19028	2724,3969	5,5352513	2,4686488	1,633E-06	0,000149	conserved Plasmodium protein, unknown function
PFIT_02202	119,9991875	230,2107917	115,3170195	161,052195	552,6325227	116,6458854	1421,733549	1217,557081	215,97627	1319,6453	6,1101404	2,6112055	1,763E-06	0,000157	cytoadherence linked asexual protein 2
PFIT_09206	31,69789858	289,8246055	112,2003433	340,3680411	371,6453715	64,15523698	1525,001357	1374,967108	201,64858	1449,9842	7,1906493	2,846122	1,835E-06	0,000157	ADP-ribosylation factor, putative
PFIT_09364	553,5811573	2210,784628	832,1525458	1693,538546	9626,858545	1344,343829	18508,95348	15203,3432	2710,2099	16856,148	6,2194993	2,6367984	1,838E-06	0,000157	cytoadherence linked asexual protein 9
PFIT_10308	236,6021715	334,2178711	77,91690504	170,4607425	395,1322537	107,897444	1488,977703	1313,330652	220,37123	1401,1542	6,3581538	2,6686079	1,855E-06	0,000157	OTU-like cysteine protease, putative
PFIT_13127	124,5274587	225,1372756	97,86363273	241,8550145	602,3694497	174,9688281	1771,162994	1217,557081	244,45361	1494,36	6,1130619	2,6118952	1,859E-06	0,000157	conserved Plasmodium membrane protein, unknown function
PFIT_06123	413,2047493	787,0291804	232,5040446	564,5128486	1359,476006	230,3756237	4167,936781	3287,593768	597,85041	3727,7653	6,235281	2,6404546	2,081E-06	0,0001731	rhoptry protein (ROP14)
PFIT_12224	871,6922108	2284,9848	786,025738	2422,424253	2022,635033	495,745013	11065,26576	9593,477402	1480,5845	10329,372	6,9765498	2,8025137	2,117E-06	0,0001735	glideosome-associated protein 45
PFIT_06193	256,979392	488,3259218	127,7837243	336,4939333	464,211319	104,9812969	2173,427132	1806,422304	296,4626	1989,9247	6,7122286	2,7467918	2,207E-06	0,0001783	merozoite surface protein 10
PFIT_10346	8935,411195	37081,06058	7585,366539	21161,48361	38141,31513	8031,069211	133901,1231	124319,7847	20155,951	129110,45	6,4055749	2,6793281	2,404E-06	0,0001915	S-antigen
PFIT_14599	76,98061083	201,6722638	63,58019451	99,61991446	287,3689118	69,98753125	927,0086992	770,9298337	133,20157	848,96927	6,373568	2,6721012	2,45E-06	0,0001925	exonuclease V, mitochondrial, putative
PFIT_07286	11,32067806	95,76261583	40,51679062	50,91684517	107,7633419	78,73597266	481,5161767	368,8704862	64,169374	425,19333	6,62611	2,7281622	2,511E-06	0,0001946	conserved Plasmodium protein, unknown function
PFIT_11369	403,016139	540,963651	230,6340389	531,3062104	956,0542642	201,2141523	3328,58564	2636,143835	477,19808	2982,3647	6,2497417	2,6437966	2,55E-06	0,0001949	conserved Plasmodium protein, unknown function
PFIT_12398	1107,162315	1617,817437	1961,636001	2926,058265	1673,094962	1041,064527	166,9095974	146,9792426	1721,1389	156,94442	0,0911864	-3,455038	2,607E-06	0,0001966	early transcribed membrane protein 12
PFIT_01010	3770,917863	4196,43198	1254,150503	4017,449772	1877,568996	971,0769961	18403,2841	17709,57635	2681,266	18056,43	6,7342927	2,7515494	2,651E-06	0,0001973	erythrocyte binding antigen-181
PFIT_06270	6732,407244	11117,97628	2447,214153	5403,274371	12743,70597	2787,836661	44461,5947	38623,30019	6872,069	41542,447	6,045115	2,5957698	2,83E-06	0,0002079	HECT-domain (ubiquitin-transferase), putative
PFIT_11276	2546,020496	3799,429347	675,0720652	1386,930587	2315,53027	1603,880924	13645,76018	11149,56087	2054,4773	12397,661	6,0344598	2,5932246	2,93E-06	0,0002124	conserved Plasmodium protein, unknown function
PFIT_02065	4,528271225	30,44109642	29,92009153	32,65319418	92,56594755	29,16147135	259,3703096	238,0115477	36,545012	248,69093	6,8050581	2,7666075	3,17E-06	0,0002263	iron-sulfur assembly protein, putative
PFIT_10367	276,2245447	764,8325476	165,1838387	369,1471275	922,8963128	221,6271823	3003,171965	2524,24996	453,31859	2763,711	6,0966195	2,6080095	3,233E-06	0,0002263	dynamin-like protein
PFIT_10345	431,3178342	403,3445276	130,2770652	246,2825663	1352,568099	288,6985664	2837,463156	2162,965499	475,41478	2500,2143	5,2590169	2,3947931	3,303E-06	0,0002263	probable protein, unknown function
PFIT_10194	58,86752593	275,8724363	134,0170767	254,5842258	482,171876	268,2855365	1533,406877	1348,416019	245,63311	1440,9114	5,8661124	2,5524047	3,316E-06	0,0002263	autophagy-related protein 8 (ATG8)
PFIT_06128	1446,782656	1721,190327	544,795	978,4889375	3523,032332	714,4560482	8781,366084	7777,572566	1488,1242	8279,4693	5,563695	2,4760433	3,32E-06	0,0002263	transcription factor with AP2 domain(s)
PFIT_10249	207,1684085	322,1682705	165,8071739	375,2350111	870,3962232	262,4532422	2249,076805	1944,867268	367,20472	2096,972	5,7106347	2,5136511	3,428E-06	0,0002297	conserved Plasmodium membrane protein, unknown function
PFIT_14159	321,507257	197,8671267	262,4241362	253,4773379	163,0265962	279,950125	1586,241569	1415,741995	246,37543	1500,9918	6,0922949	2,6069858	3,452E-06	0,0002297	ATP-dependent protease, putative
PFIT_04132	98,48989915	149,6687241	72,30688787	140,0213242	335,7242575	122,4781797	955,8276225	831,6180371	153,11488	893,72283	5,8369431	2,545213	3,541E-06	0,0002329	conserved protein, unknown function
PFIT_14408	141,5084758	95,12842632	54,85350115	74,71493584	208,6187773	52,49064844	649,6265625	591,7099831	104,55246	620,66827	5,9364291	2,5695954	3,632E-06	0,0002338	conserved Plasmodium protein, unknown function
PFIT_06093	33731,09236	29593,18505	10595,45421	24406,87904	81748,16591	20687,14778	194214,3264	162529,6465	33460,32	178371,99	5,3308511	2,4143659	3,638E-06	0,0002338	histone H3
PFIT_10144	603,3921407	1430,097342	363,4044451	712,8358323	3377,966295	702,7914596	7491,719267	5422,111672	1198,4146	6456,9155	5,3878812	2,4297181	4,22E-06	0,0002682	conserved Plasmodium protein, unknown function
PFIT_03215	139,2443402	657,6545206	126,5370538	315,4630624	1003,028029	169,1365339	2624,923596	2102,277696	401,84392	2363,6004	5,8818867	2,556279	4,389E-06	0,0002758	protein kinase, putative
PFIT_14356	618,1090222	443,2984666	152,0937986	342,028373	516,7114087	186,6334167	2440,002172	1982,797395	376,47908	2211,3998	5,8738982	2,5543183	4,467E-06	0,0002776	conserved Plasmodium membrane protein, unknown function
PFIT_04216	160,7536285	215,624433	29,29675629	67,52016424	342,632166	96,23285547	919,8039684	765,2403147	152,01	842,52214	5,5425442	2,4705484	4,538E-06	0,0002851	asparagine-rich protein
PFIT_10353	3019,224839	7291,276782	1429,307706	2861,858765	6076,194587	1189,788301	23235,2569	20944,06794	3644,6085	22089,662	6,0609151	2,5995356	4,693E-06	0,0002854	probable protein, unknown function
PFIT_10177	750,5609556	1486,540209	287,980881	505,8477878	2347,30566	542,4033672	5833,430389	4909,106703	986,77331	5371,2685	5,4432649	2,4444723	4,867E-06	0,0002928	serine/threonine protein phosphatase, putative
PFIT_14565	2779,226464	4116,524101	1078,369966	3413,642402	1957,700712	904,0056612	16160,21124	14296,81316	2374,9115	15228,512	6,412244	2,6808293	5,423E-06	0,0003229	conserved Plasmodium protein, unknown function
PFIT_12346	75,84854302	225,1372756	96,61696225	174,8882943	657,632702	113,7297383	1248,820009	1186,264726	223,97559	1217,5424	5,4360495	2,4425586	5,581E-06	0,0003288	conserved Plasmodium protein, unknown function
PFIT_02169	2612,812497	2948,347026	682,5520881	1551,85689	3474,676986	796,108168	11851,7822	10797,75894	2011,0589	11324,771	5,6312475	2,4934546	5,739E-06	0,0003312	calcium-dependent protein kinase 1
PFIT_09353	628,2976325	1917,789075	525,4716076	904,3274457	2602,899182	463,6673945	7043,825168	6409,24323	1173,7421	6726,5342	5,7308453	2,518748	5,739E-06	0,0003312	cAMP-dependent protein kinase catalytic subunit
PFIT_12366	22,64135613	145,2293975	62,33352403	161,605639	321,9084444	107,897444	862,1661218	736,7927193	136,93597	799,47942	5,838345	2,5455595	5,963E-06	0,0003407	conserved Plasmodium protein, unknown function
PFIT_12295	2138,476086	2895,075108	843,9959154	1961,958871	2535,201698	1003,154615	11938,23897	10186,13564	1896,3104	11062,187	5,833532	2,5443697	6,781E-06	0,0003835	myosin D
PFIT_12452	75,84854302	83,07882565	79,16357552	57,5581728	175,4608259	37,90991276	539,1540232	474,126589	84,836643	506,64031	5,9719514	2,5782024	7,056E-06	0,0003952	conserved Plasmodium protein, unknown function
PFIT_12227	11,32067806	194,6961792	53,60683067	125,631781	216,9082651	87,48441406	784,1148712	632,4848069	114,94136	708,29987	6,1622716	2,6234623	7,214E-06	0,0004	conserved Plasmodium protein, unknown function
PFIT_13229	320,5637994	1163,737749	431,3479863	854,5174884	3111,321103	612,3908984	6069,985718	5224,875011	1083,9798	5647,4304	5,2099035	2,3812567	7,387E-06	0,0004056	glideosome associated protein with multiple membrane spans 1 (GAPM1)
PFIT_05326	200,3760017	622,7740976	180,7672197	487,5841369	1005,791191	242,0402122	2812,246598	2363,04692	456,5548	2587,6468	5,6677598	2,5027786	7,749E-06	0,0004214	cell cycle regulator with zn-finger domain, putative
PFIT_13435	2761,11338	4209,749959	1239,190458	3219,383569	4028,69109	2280,42706	17212,10146	15568,42068	2956,4259	16390,261	5,5439445	2,4709128	9,03E-06	0,0004864	conserved protein, unknown function
PFIT_08054	1858,855338	1128,223136	497,4215218	935,3203079	833,0935279	466,5835417	5524,827753	5118,670655	953,24956	5321,7492	5,582745	2,4809747	9,193E-06	0,0004905	adenylyl cyclase beta (ACbeta)
PFIT_08345	714,3347858	1581,668635	821,5558467	1598,899627	4629,678959	1102,303617	9842,863092	8035,49743	1741,4069	8939,1803	5,133093	2,3598892	9,82E-06	0,0005191	cytoadherence linked asexual protein 8
PFIT_10166	70,18820399	379,8795158	142,1204348	248,4963202	945,0016137	137,0589154	1845,611879	1546,600933	320,4575	1696,1064	5,2927654	2,4040217	1,003E-05	0,0005253	conserved Plasmodium protein, unknown function
PFIT_11297	224,1494256	400,8077695	106,5903261	200,3467168	1166,054623	157,4719453	2221,45867	1708,752227	375,90347	1965,1054	5,2276864	2,3861726	1,037E-05	0,0005375	conserved Plasmodium protein, unknown function
PFIT_06293	26,03755954	42,49069709	21,19339817	32,65319418	134,0133867	14,58073568	308,6026369	229,4772691	45,161495	269,03995	5,9572862	2,5746553	1,045E-05	0,0005375	conserved Plasmodium protein, unknown function
PFIT_12162	10,18861026	46,29583414	22,44006865	39,84796578	91,18436624	26,24532422	241,3584826	250,338839	39,367028	245,84866	6,2450399	2,6427108	1,06E-05	0,0005404	male development gene 1
PFIT_08280	0	2,536758035	36,15344394	44,27551754	11,05265045	0	116,4764816	181,116357	15,669728	148,79642	9,4957881	3,2472877	1,079E-05	0,0005449	conserved Plasmodium protein, unknown function
PFIT_09097	13,58481368	72,93179351	16,20671625	28,7790864	96,71069147	40,8260599	246,1616364	278,7864343	44,83986	262,47404	5,8535873	2,549321	1,137E-05	0,0005659	subpellicular microtubule protein 1, putative
PFIT_10118	115,4709162	116,0566801	41,1402586	74,71493584	368,8822089	67,07138411	727,6778131	594,5547426	130,55604	661,11628	5,0638505	2,3402348	1,145E-05	0,0005659	ubiquinol-cytochrome c reductase complex subunit, putative
PFIT_10054	30,56583077	55,17448726	36,15344394	52,57717707	208,6187773	34,99376562	392,6578298	364,1292204	69,68058	378,39353	5,4304015	2,4410589	1,152E-05	0,0005659	conserved Plasmodium membrane protein, unknown function
PFIT_13315	3833,181592	2699,110549	522,9782666	1209,275073	2131,779956	854,4311107	10225,91461	9397,188994	1875,1261	9811,5518	5,2324758	2,3874937	1,161E-05	0,0005659	conserved Plasmodium protein, unknown function
PFIT_09057	11,32067806	468,666047	102,2269794	226,3585834	327,4347697	67,07138411	1299,253125	1197,643764	200,51307	1248,4484	6,2262695	2,638368	1,202E-05	0,0005811	conserved Plasmodium protein, unknown function
PFIT_14385	64,52786496	206,7457799	82,28025172	127,2921129	537,4351283	104,9812969	1080,709623	856,2726197	187,21041	968,49112	5,1732761	2,3710782	1,219E-05	0,0005832	conserved Plasmodium protein, unknown function
PFIT_05075	1521,499132	2826,582641	807,2191362	1779,322361	1552,897389	367,4345391	9180,027857	8492,555462	1475,8259	8836,2917	5,9873538	2,5819185	1,235E-05	0,0005832	subtilisin-like protease 1
PFIT_10092	190,1873915	410,9548017	109,0836671	168,2469666	600,9878684	113,7297383	1490,17849	1345,57126	265,53174	1417,8749	5,3397567	2,416774	1,237E-05	0,0005832	tubulin-tyrosine ligase, putative
PFIT_13559	4776,194075	3378,327513	1055,929897	2747,295863	4023,164765	1111,052059	16382,3571	13945,01124	2848,6607	15163,684	5,3230924	2,4122646	1,301E-05	0,0006084	serine/threonine protein kinase, putative
PFIT_04064	4076,57617	5834,543481	903,8360984	2427,958693	5229,285246	1277,272445	19535,62763	15985,65207	3291,5787	17760,64	5,3957816	2,431832	1,327E-05	0,0006151	conserved Plasmodium protein, unknown function
PFIT_10376	26,03755954	42,49069709	26,18008009	35,42041403	17,96055699	20,41302995	180,1182706	217,1499778	28,083723	198,63412	7,0729271	2,8223074	1,421E-05	0,0006536	gametocyte-specific protein
PFIT_08049	1347,160689	1197,983982	314,7842963	596,0591548	2542,109604	495,745013	5538,036426	4888,245133	1082,3071	5213,1408	4,8166927	2,2680429	1,495E-05	0,0006818	transcription factor with AP2 domain(s)
PFIT_14118	63,39579715	109,714785	37,40011442	52,57717707	250,0662165	37,90991276	522,3429847	448,5237532	91,844	485,43337	5,2854119	2,4020159	1,54E-05	0,0006968	ankyrin-repeat protein, putative
PFIT_12354	73,58440741	91,95747877	401,4278947	379,1091189	862,1067353	396,5960104	1795,178763	1791,250253	367,46361	1793,2145	4,8799785	2,2868748	1,595E-05	0,0007161	ATP synthase subunit beta, mitochondrial
PFIT_10354	2259,607341	2600,811176	545,4183353	1788,730908	1655,134405	597,8101628	9172,823126	8288,681029	1574,5854	8730,7521	5,5447943	2,4711339	1,67E-05	0,0007435	merozoite surface protein
PFIT_05226	675,8444803	837,7634411	445,6846968	530,1993225	1479,673579	352,8538034	3972,20826	3380,52258	720,3367	3676,3654	5,1036764	2,3515369	1,731E-05	0,0007649	conserved protein, unknown function, putative
PFIT_05300	5653,546625	8681,420186	3814,811671	13405,51982	5408,890816	2458,312035	43175,55024	36340,85479	6570,4169	39758,203	6,0510929	2,5971957	1,769E-05	0,0007756	membrane skeletal protein IMC1-related
PFIT_05266	648,674853	851,0823208	400,1812243	548,4629735	1478,291998	382,0152747	3912,168837	3344,488959	718,11811	3628,3289	5,0525517	2,3370122	1,864E-05	0,000811	conserved protein, unknown function, putative
PFIT_13154	139,2443402	564,4286628	164,5605034	491,4582446	513,9482461	131,2266211	2150,612151	1761,854405	334,14444	1956,2333	5,8544541	2,5495347	1,886E-05	0,0008119	ADP-ribosylation factor, putative
PFIT_11057	2898,093584	1797,293068	794,1290961	1411,281229	1051,383374	402,4283047	8642,074622	6633,979233	1392,4349	7638,0269	5,4853744	2,4555901	1,895E-05	0,0008119	calcium/calmodulin-dependent protein kinase, putative
PFIT_09298	39,62237322	57,07705579	29,92009153	45,38240547	49,73692704	34,99376562	290,5908099	239,908054	42,78877	265,24943	6,1990432	2,6320456	1,922E-05	0,0008172	conserved Plasmodium protein, unknown function
PFIT_09193	52,07511909	379,8795158	90,38360984	230,7861352	261,118867	84,56826693	1147,953778	1005,148369	183,13525	1076,5511	5,8784481	2,5554353	2,221E-05	0,0009374	PPPDE peptidase, putative
PFIT_11339	666,7879379	398,2710115	178,2738787	308,2682908	990,5937969	250,7886536	2353,545402	2018,831016	465,49726	2186,1882	4,6964577	2,231573	2,27E-05	0,000951	conserved Plasmodium protein, unknown function
PFIT_07238	8943,33567	6634,890461	6436,559691	18131,37787	3145,860635	4275,0717	306435,2137	122354,0558	7927,8494	214394,63	27,043228	4,7571954	2,346E-05	0,0009757	18S ribosomal RNA
PFIT_09174	1,132067806	101,4703214	71,06021739	105,1543541	357,8295584	64,15523698	630,413947	552,8316028	116,80029	591,62277	5,0652508	2,3406337	2,363E-05	0,0009757	conserved Plasmodium protein, unknown function
PFIT_06170	281,8848838	201,0380743	142,74377	254,0307819	327,4347697	169,1365339	1361,694126	1007,993128	229,37814	1184,8436	5,1654602	2,3688969	2,599E-05	0,0010653	conserved Plasmodium protein, unknown function
PFIT_10357	726,7875316	1282,965376	427,6079748	1125,151589	638,2905637	469,4996888	4552,189092	4121,108312	778,38379	4336,6487	5,5713502	2,478027	2,631E-05	0,0010704	probable protein, unknown function
PFIT_06200	452,8271225	711,5606289	187,6239073	373,5746792	1251,712664	268,2855365	2896,301791	2339,34059	540,93076	2617,8212	4,8394756	2,2748507	2,677E-05	0,0010757	conserved Plasmodium protein, unknown function
PFIT_07239	9230,880892	6808,024377	6500,763221	18378,21389	3264,676628	4562,7076	318163,3147	125634,0636	8124,3882	221898,69	27,312664	4,7714982	2,682E-05	0,0010757	18S ribosomal RNA
PFIT_10350	147,1688148	147,7661555	52,36016018	86,89070316	272,1715174	81,65211979	710,8667745	586,9687172	131,33491	648,91775	4,9409387	2,3047852	2,726E-05	0,0010856	merozoite surface protein
PFIT_05246	627,6368872	894,2072074	473,4779806	580,0092797	1474,147254	382,0152747	3937,385395	3350,178478	740,41565	3643,7819	4,9212654	2,2990293	2,81E-05	0,001116	conserved Plasmodium protein, unknown function
PFIT_14695	593,2035305	1716,116811	316,6543021	822,9711822	939,4752885	309,1115964	4596,618265	4035,765526	782,92212	4316,1919	5,5129262	2,4628183	2,892E-05	0,001136	zinc finger protein, putative
PFIT_09139	1006,40828	1088,903387	432,5946568	1921,004017	400,6585789	102,0651497	5354,31579	4657,81961	825,27234	5006,0677	6,0659584	2,6007356	2,956E-05	0,0011451	conserved Plasmodium protein, unknown function
PFIT_12228	1512,442589	5246,015617	1201,790343	2976,97511	6414,682007	1288,937034	16794,22755	14858,17905	3106,8071	15826,203	5,0940211	2,3488106	2,956E-05	0,0011451	cAMP-dependent protein kinase regulatory subunit
PFIT_06169	873,9563464	2583,688059	281,7475286	615,4296937	1999,148151	390,7637161	6305,340258	5216,340732	1124,1222	5760,8405	5,1247456	2,3574804	3,184E-05	0,0012243	conserved Plasmodium protein, unknown function
PFIT_04151	87,16922108	109,0805955	47,9968135	71,947716	315,0005379	96,23285547	670,0399665	467,4888168	121,23796	568,76439	4,6913063	2,2299897	3,204E-05	0,0012243	repressor of RNA polymerase III transcription MAF1, putative
PFIT_14078	193,5835949	854,2532683	250,5807666	515,8097793	2514,477978	347,0215091	4151,125742	3636,550938	779,28782	3893,8383	4,9966627	2,3209648	3,517E-05	0,0013352	glideosome associated protein with multiple membrane spans 3
PFIT_04115	619,24109	231,4791707	255,5674485	193,7053892	109,1449232	367,4345391	1186,379009	1508,670806	296,09543	1347,5249	4,5509818	2,1861778	3,571E-05	0,0013465	BC_ITvar27 - erythrocyte membrane protein 1,, putative (VAR)
PFIT_08056	441,5064444	757,2222735	177,0272082	361,9523559	1072,107094	215,794888	2687,364597	2204,688639	504,26838	2446,0266	4,8506445	2,2781764	3,635E-05	0,0013555	serine/threonine protein phosphatase, putative
PFIT_08115	1195,463603	549,2081146	484,9548169	494,7789085	426,9086238	1455,157421	2880,691541	3446,900302	767,74525	3163,7959	4,1208929	2,042957	3,643E-05	0,0013555	erythrocyte membrane protein 1, PfEMP1
PFIT_07025	166,4139675	264,4570252	76,42356408	155,5177553	533,2903844	75,81982552	1195,985317	867,6516579	211,82042	1031,8185	4,8711946	2,2842756	3,715E-05	0,0013734	conserved Plasmodium protein, unknown function
PFIT_14378	541,1284114	1123,78381	229,3873684	618,1969136	1424,410327	274,1178307	3573,546488	3316,041364	701,83744	3444,7939	4,9082504	2,2952089	3,841E-05	0,0014105	cGMP-dependent protein kinase
PFIT_13448	55,47132251	306,3135247	47,37478726	89,65792303	411,7112294	61,23908984	803,3274867	791,7914036	161,9611	797,55945	4,924389	2,2999447	3,974E-05	0,00145	inner membrane complex protein
PFIT_07052	67,92406838	173,7679254	66,69687071	115,6697896	74,60539056	40,8260599	591,9887159	477,9196017	89,915017	534,95416	5,949553	2,5727813	4,026E-05	0,0014597	conserved Plasmodium protein, unknown function
PFIT_12091	29,43376296	143,3262978	61,08685355	102,3871343	128,4870615	64,15523698	501,9259807	467,4888168	88,146146	484,7092	5,4989267	2,4591501	4,237E-05	0,0015263	cGMP-specific phosphodiesterase
PFIT_02034	441,5064444	343,7307138	384,5978433	612,1090299	990,5937969	268,2855365	2518,053423	2362,098666	506,80389	2440,076	4,8146355	2,2674266	4,376E-05	0,0015666	conserved Plasmodium protein, unknown function
PFIT_05367	294,3376296	539,0651825	499,2915275	431,686296	251,4477978	781,5274323	55,23626964	51,20567161	466,22529	53,220971	0,1141529	-3,130961	4,443E-05	0,0015808	rifin, pseudogene
PFIT_bin04	90,5654245	78,00530958	56,72350687	54,23750898	129,8686428	75,81982552	347,027868	424,8174238	80,870036	385,92265	4,7721339	2,2546345	4,508E-05	0,0015936	erythrocyte membrane protein 1, PfEMP1 (VAR)
PFIT_12441	157,3574251	141,4242605	73,55335835	123,9714491	193,4213829	32,07761849	668,839178	585,0722108	120,30095	626,95569	5,2115607	2,3817155	4,598E-05	0,0016152	double C2-like domain-containing protein (DOC2)
PFIT_14611	2,264135613	108,446406	37,40011442	74,16149187	186,5134764	34,99376562	416,6735992	365,0774735	73,963232	390,87554	5,2847277	2,4018291	4,727E-05	0,0016503	conserved Plasmodium membrane protein, unknown function
PFIT_13218	24,90549174	44,39326561	32,65319418	32,65319418	154,7371063	23,23291708	264,1734635	237,0632945	50,721385	250,61838	4,9410792	2,3048262	4,89E-05	0,0016967	conserved Plasmodium protein, unknown function
PFIT_01100	266,0359345	275,2382468	133,3937414	201,4536048	812,3698083	262,4532422	1514,194261	1259,28022	325,15743	1386,7372	4,2648179	2,0924842	5,286E-05	0,001823	UMP-CMP kinase, putative
PFIT_09038	1121,879196	1466,246144	364,0277803	1089,177731	893,8831054	329,5246263	4918,429575	4112,574033	877,45643	4515,5018	5,1461265	2,3634869	5,408E-05	0,0018378	conserved Plasmodium protein, unknown function
[illegible]	[illegible]	[illegible]	[illegible]	[illegible]	[illegible]	[illegible]	[illegible]	[illegible]	[illegible]	[illegible]	[illegible]	[illegible]	[illegible]	[illegible]	[illegible]

PFIT_14001	38,49030541	35,51461249	19,94672769	26,01186655	62,1711588	26,24532422	103,2678085	128,9624322	34,729999	116,11512	3,3433666	1,7413016	0,0038733	0,0439911	B_ITvar26 - erythrocyte membrane protein 1, PfEMP1, putative
PFIT_05169	422,2612917	231,4791707	149,6004577	220,8241437	835,8566905	131,2266211	1002,658373	894,2027469	331,87473	948,43056	2,8577969	1,5149034	0,0039045	0,0442558	transcription factor with AP2 domain(s)
PFIT_07042	398,4878678	609,4561179	268,0341533	432,2397399	1228,225782	259,5370951	1875,631591	1326,606196	532,66346	1601,1189	3,0058733	1,5877842	0,0039124	0,0442566	origin recognition complex subunit 2, putative
PFIT_14039	29,43376296	24,09920133	36,15344394	21,5843148	37,30269528	20,41302995	110,4725393	99,56658369	28,164408	105,01956	3,7288041	1,898713	0,0039478	0,0445681	conserved Plasmodium protein, unknown function
PFIT_14621	23,77342393	12,68379018	11,84336957	9,961991446	49,73692704	8,748441406	79,25203905	62,58470975	19,457991	70,918374	3,6446916	1,8657967	0,0039651	0,0446742	apicoplast ribosomal protein S10 precursor, putative
PFIT_08022	0	18,39149575	56,10017163	55,34439692	45,59218312	119,5620326	0	0,948253178	49,165047	0,4741266	0,0096436	-6,696217	0,0039927	0,044896	Plasmodium exported protein, unknown function, pseudogene
PFIT_14569	67,92406838	171,8653569	59,84018307	132,8265526	338,4874201	46,65835417	495,9256383	370,7669926	136,26699	433,34632	3,1801269	1,6690843	0,0040627	0,0455928	conserved Plasmodium protein, unknown function
PFIT_05080	421,1292239	462,9583414	304,8109325	359,73858	1120,46244	387,847569	1571,832108	1318,071917	509,49118	1444,952	2,8360687	1,5038925	0,0041104	0,046037	6-cysteine protein
PFIT_05267	0	13,95216919	4,986681922	13,83609923	49,73692704	17,49688281	64,8425774	56,89519068	16,668127	60,868884	3,6518131	1,8686129	0,0041242	0,0460998	50S ribosomal protein L17, apicoplast, putative (RPL17)
PFIT_03082	1521,499132	666,5331737	177,6505435	463,2326022	431,0533677	116,6458854	1904,450514	1782,715975	562,76912	1843,5832	3,275914	1,7118975	0,0041497	0,0462929	conserved Plasmodium protein, unknown function
PFIT_05155	1116,218857	2089,654431	723,0688787	1183,263206	1260,002152	545,3195143	4079,078434	3454,486328	1152,9212	3766,7824	3,2671639	1,7080388	0,0041738	0,0464709	conserved Plasmodium protein, unknown function
PFIT_14623	73,58440741	92,59166828	62,95685927	77,48215569	303,9478875	148,7235039	357,8349642	330,9403591	126,54775	344,38766	2,7214049	1,4443516	0,0042017	0,0466894	conserved Plasmodium protein, unknown function
PFIT_11473	49,81098348	37,41718102	20,57006293	40,40140975	9,671069147	2,916147135	129,6851548	97,67007734	26,797809	113,67762	4,2420489	2,0847613	0,0042151	0,0467464	kinesin-like protein, putative
PFIT_12266	174,3384422	277,7750048	445,6846968	635,9071206	882,8304549	962,3285547	1817,993744	1318,071917	563,14405	1568,0328	2,7844258	1,4773799	0,0042668	0,047227	conserved Plasmodium protein, unknown function
PFIT_04035	276,2245447	416,0283178	168,9238501	318,2302823	266,6451922	151,639651	948,6228917	806,0152013	266,28197	877,31905	3,2946994	1,7201468	0,0043433	0,0479804	conserved Plasmodium protein, unknown function
PFIT_14738	266,0359345	162,3525142	108,4603318	163,8194149	357,8295584	174,9688281	624,4100046	549,0385901	205,57776	586,7243	2,8540261	1,5129985	0,0043632	0,0480276	DNA-directed RNA polymerase, alpha subunit, putative
PFIT_07157	521,8832587	504,1806595	269,2808238	355,8644722	1675,858125	370,3506862	2034,135669	1633,840226	616,23634	1833,9879	2,9761113	1,5734285	0,0043646	0,0480276	conserved Plasmodium protein, unknown function
PFIT_11137	20,37722051	75,46855154	67,32020595	90,76481095	87,03962232	23,32917708	258,1695211	194,3919015	60,716598	226,28071	3,7268345	1,8979507	0,0045004	0,0494255	conserved Plasmodium protein, unknown function
PFIT_13610	84,90508547	73,56598302	31,16676201	45,93584944	222,4345904	72,90367838	264,1734635	229,4772691	88,485325	246,82537	2,7894497	1,4799806	0,004516	0,0495011	conserved Plasmodium protein, unknown function
PFIT_06163	2,264135613	71,66341449	6,856687643	17,15676305	38,68427659	8,748441406	114,0749047	86,2910392	24,228953	100,18297	4,1348453	2,0478333	0,004583	0,0501255	conserved Plasmodium protein, unknown function
PFIT_03213	19,24515271	31,70947544	22,44006865	22,69120274	60,78957749	46,65835417	115,2756932	101,46309	33,922305	108,36939	3,1946352	1,6756512	0,0045907	0,0501255	N-acetylglucosamine-1-phosphate transferase, putative
PFIT_14479	82,64094986	123,6669542	56,72350687	109,5819059	301,1847248	87,48441406	413,0712338	334,7333718	126,88041	373,9023	2,9468876	1,559192	0,0046787	0,050988	conserved Plasmodium protein, unknown function
PFIT_08238	2037,722051	1698,993694	1750,94869	1709,588421	1633,029104	1157,710413	5311,087405	4924,278753	1664,6654	5117,6831	3,0743014	1,6202586	0,0047001	0,0511228	conserved Plasmodium protein, unknown function
PFIT_11266	1291,689367	1980,573836	546,6650057	1811,422111	1076,251838	297,4470078	4035,850049	3775,944155	1167,3415	3905,8971	3,3459763	1,7424272	0,0047113	0,0511466	kelch protein, putative
PFIT_14760	32,82996638	60,24800333	52,98349542	55,89784089	245,9214726	110,8135911	265,374252	252,2353454	93,115728	258,8048	2,7793887	1,4747676	0,0047388	0,0513465	trailer hitch homolog, putative
PFIT_13339	1,132067806	25,36758035	12,46670481	14,3895432	70,46064664	17,49688281	93,6615007	70,17073517	23,552238	81,916118	3,478061	1,7982832	0,0047727	0,0516141	MSP7-like protein
PFIT_05351	943,0124826	997,5800973	688,7854405	1078,108852	877,3041297	355,7699505	2847,069463	2461,66525	823,42683	2654,3674	3,2235619	1,6886557	0,0047819	0,051615	formin 1
PFIT_07034	1793,195405	960,1629163	295,4609039	385,1970026	2584,938625	466,5835417	3541,125199	2783,123077	1080,9231	3162,1241	2,9253924	1,5486302	0,0047985	0,0516956	serine/threonine protein kinase, putative
PFIT_12206	864,899804	1550,593349	1038,47651	1008,374912	1822,305743	1784,682047	4061,066607	3484,830429	1344,8887	3772,9485	2,8053983	1,4882056	0,0049265	0,0529056	heterochromatin protein 1
PFIT_13330	86,03715328	386,8556004	484,3314817	557,871521	719,8038608	1041,064527	1673,899128	1319,020171	545,99402	1496,4596	2,7407986	1,4545963	0,0049295	0,0529056	histone H3 variant, putative
PFIT_10197	184,5270524	78,63949909	19,32339245	44,27551754	129,8686428	46,65835417	264,1734635	219,9947373	83,882076	242,0841	2,8860051	1,5290738	0,0049717	0,053257	conserved Plasmodium protein, unknown function
PFIT_12290	1484,140894	830,154067	1354,507477	1551,303446	2268,556506	1420,163655	4955,654018	3591,034785	1484,8043	4273,3444	2,8780522	1,5250928	0,0050275	0,0537534	conserved Plasmodium protein, unknown function
PFIT_02024	499,2419026	450,2745512	165,8071739	351,4369204	118,8159924	69,98753125	936,615007	897,0475064	275,92735	916,83126	3,3227271	1,7323678	0,0051072	0,054502	protein kinase, putative
PFIT_06131	113,2067806	332,3153026	165,1838387	222,4844756	542,9614535	166,2203867	881,3787373	664,7254778	257,06204	773,05211	3,0072589	1,5884491	0,0051222	0,0545595	conserved Plasmodium protein, unknown function
PFIT_09035	691,6934296	696,9742701	500,538198	613,7693618	2869,544374	390,7637161	3583,152796	2764,158014	960,54722	3173,6554	3,3040077	1,724217	0,005164	0,0549012	conserved Plasmodium membrane protein, unknown function
PFIT_10167	28,30169516	43,75907611	22,44006865	26,01186655	63,55274011	11,66458854	128,4843663	103,3595964	32,621673	115,92198	3,5535266	1,8292515	0,0051881	0,0549792	enoyl-CoA hydratase-related protein, putative
PFIT_02014	7402,591385	4693,636554	2940,895664	3800,499736	3557,571865	1323,930799	756,4967364	791,7914036	3953,1877	774,14407	0,1958278	-2,352342	0,0051907	0,0549792	liver stage associated protein 2 (LSAP2)
PFIT_14045	4,528271225	83,71301516	17,45338673	34,31352609	129,8686428	17,49688281	192,1261553	150,7722553	47,895621	171,44921	3,5796426	1,8398156	0,0052256	0,0552444	selenoprotein (Sel1)
PFIT_bin09	562,6376997	381,7820843	282,3708639	328,7457177	274,93468	195,3818581	74,44888517	94,8253178	337,64215	84,637101	0,250671	-1,996133	0,005271	0,0556204	alpha/beta hydrolase, putative
PFIT_13588	760,7495658	1105,392314	536,0683067	723,9047117	1322,17331	519,0741901	2707,778001	2204,688639	827,89373	2456,2333	2,9668461	1,5689301	0,0053901	0,0567713	conserved Plasmodium protein, unknown function
PFIT_13114	29,43376296	13,95216919	16,83005149	23,24464671	51,11850835	17,49688281	105,6693854	70,17073517	25,346004	87,92006	3,4687938	1,7944341	0,0054096	0,0568711	conserved Plasmodium protein, unknown function
PFIT_11086	10959,54843	5972,162604	3704,481333	4233,846364	8257,71147	12994,35164	19444,3677	18281,37302	7687,017	18862,87	2,4538609	1,2950535	0,0054981	0,0576779	transcription factor with AP2 domain(s)
PFIT_01095	553,5811573	332,3153026	217,5439989	301,6269632	667,3037711	236,207918	1164,764816	994,7175837	384,76319	1079,7412	2,8062487	1,4886429	0,0055067	0,0576779	kinesin-8, putative
PFIT_07009	1050,558924	699,5110282	650,1386556	930,8927562	321,9084444	125,3943268	2031,734092	2102,277296	629,73402	2067,0057	3,2823472	1,7147278	0,0055171	0,0576801	Plasmodium exported protein (hyp12), unknown function
PFIT_11376	3983,74661	6335,553193	2507,677672	4403,753663	2835,004841	1843,00499	11800,1483	11341,10801	3651,4568	11570,628	3,1687704	1,6639231	0,0056734	0,0592043	subtilisin-like protease 2
PFIT_09063	2,264135613	81,17625712	63,58019451	108,475018	288,7504931	122,4781797	353,0318103	300,5962574	111,12071	326,81403	2,9410721	1,5563422	0,005707	0,0594452	developmental protein, putative
PFIT_11182	1907,534254	906,8909976	483,0848112	644,2087801	775,067113	355,7699505	2694,569328	2206,585145	845,42598	2450,5772	2,8986301	1,5353713	0,0057643	0,0598429	conserved Plasmodium protein, unknown function
PFIT_13307	156,2253573	84,34720467	69,19021167	211,9690402	78,75013448	55,40679557	362,6381181	364,1292204	109,31479	363,38367	3,3241949	1,733005	0,0057663	0,0598429	CPW-WPC family protein
PFIT_05247	3,396203419	10,78122165	6,233352403	13,2826526	56,64483357	5,832294271	76,85046211	53,10217797	16,028427	64,97632	4,0538177	2,0192812	0,0058097	0,0601829	50S ribosomal protein L17, apicoplast, putative (RPL17)
PFIT_06065	78,11267863	245,4313399	61,08685355	101,2802464	563,6851731	87,48441406	704,8628322	573,6931727	189,51345	639,278	3,3732593	1,7541432	0,0059081	0,06109	conserved Plasmodium protein, unknown function
PFIT_12437	87,16922108	130,6430388	56,72350687	89,10447904	251,4477978	72,90367838	359,0357527	308,1822829	114,66529	333,60902	2,909416	1,5407296	0,0059433	0,0613414	endosome sorting protein (SNF7 homologue), putative
PFIT_13308	613,580751	471,202805	195,7272655	337,6008212	332,9610949	128,310474	1128,741162	983,3385456	346,56387	1056,0399	3,0471724	1,6074711	0,0059811	0,0616193	conserved Plasmodium protein, unknown function
PFIT_12056	83,77301766	225,2843078	278,6308524	293,3253037	425,5270424	390,7637161	845,3550832	762,3955551	284,55071	803,87532	2,8250688	1,498286	0,0060522	0,0622391	shewanella-like protein phosphatase 2, putative (SHLP2)
PFIT_03160	618,1090222	864,4003005	404,5445709	1433,4198	530,5272218	349,9376562	2760,612694	1938,229496	700,15644	2349,421	3,3555659	1,7465561	0,006091	0,062485	conserved Plasmodium protein, unknown function
PFIT_05142	271,6962735	230,2107917	120,3037014	161,052195	458,6849938	163,3042396	701,2604667	591,7099831	234,2087	646,48522	2,7602955	1,4648227	0,0060982	0,062485	ATP-dependent DNA helicase UvrD (UvrD)
PFIT_06106	318,1110536	300,6058272	99,1103021	169,3538546	649,3432141	151,639651	888,5834621	649,5534269	281,36065	769,06845	2,7333902	1,4506914	0,0061421	0,0628201	conserved Plasmodium protein, unknown function
PFIT_14672	86,03715328	213,087675	101,6036442	133,3799966	855,1988288	128,310474	966,6347187	842,0488221	252,9363	904,34177	3,5753737	1,838094	0,0062015	0,0633135	protein phosphatase containing kelch-like domains (PPKL)
PFIT_14264	1013,200687	1043,241742	387,0911842	882,1896869	1123,225602	443,2543646	2537,266038	2246,411779	815,36721	2391,839	2,93345	1,5525984	0,0062416	0,0636085	DNA-directed DNA polymerase, putative
PFIT_13607	93,96162792	74,20017253	28,67342105	40,95485372	252,8293791	55,40679557	289,3900214	240,8563072	91,004375	265,12316	2,9133013	1,5426549	0,0064004	0,065093	cytochrome c oxidase subunit 2, putative
PFIT_04223	704,1461755	1889,250547	574,0917563	2029,479035	1193,686249	250,7886536	4028,643518	3370,091795	1106,9071	3699,3686	3,3420769	1,7407449	0,0064148	0,065139	reticulocyte binding protein homologue 5
PFIT_06196	97,35783134	107,178027	49,24348398	60,32539264	183,7503138	37,90991276	295,3939637	236,1150413	89,29416	265,7545	2,9761689	1,5734564	0,006552	0,0664119	DnaJ protein, putative
PFIT_13438	39,62237322	44,39326561	18,70005721	26,56530152	134,0133867	23,32917708	157,3032896	128,014179	47,770595	142,65873	2,9863294	1,5783733	0,0067683	0,0684819	lipoyl synthase (LipA)
PFIT_14543	36,2261698	47,56421316	46,12680778	48,14962532	41,4474392	14,58073568	147,6969819	133,7036981	39,015832	140,70034	3,6062371	1,8504943	0,006801	0,0686903	dynactin subunit 4, putative
PFIT_02064	1618,856963	3669,420498	835,8925572	2056,59779	2485,464771	696,9591654	6175,655104	5438,231976	1893,8653	5806,9435	3,0661862	1,6164453	0,0068504	0,0690654	conserved Plasmodium protein, unknown function
PFIT_08111	2114,702662	4607,386781	1487,277883	3906,207535	2178,753721	708,6237539	8319,062523	7879,983909	2500,4921	8099,5233	3,2391717	1,695625	0,0069073	0,0694123	plasmepsin X
PFIT_09194	1012,068619	1232,230216	153,9638044	381,3228948	388,2243472	151,639651	1621,064435	1750,475367	553,24159	1685,7699	3,0470773	1,6074261	0,0069093	0,0694123	thioredoxin, putative
PFIT_11255	457,3553937	1452,293975	567,2350687	1142,861796	2385,990917	615,3070456	3228,920197	3030,617157	1103,5074	3129,7687	2,836201	1,5039598	0,0069358	0,0695551	ethanolamine kinase (EK)
PFIT_13427	31,69789858	52,00353972	64,82686499	87,9975911	327,4347697	90,4005612	353,0318103	324,3025869	109,0602	338,6672	3,1053233	1,6347435	0,0069832	0,0699062	conserved Plasmodium protein, unknown function
PFIT_08014	1523,763267	1820,75808	2292,627014	2031,139367	1581,910596	1131,465089	452,6972534	549,9868432	1730,2772	501,34205	0,2897467	-1,787136	0,0070238	0,0701642	Plasmodium exported protein (PHISTa), unknown function
PFIT_08001	146,036747	68,49246695	94,74695652	73,05460393	51,11850835	81,65211979	226,9490209	279,7346875	85,850234	253,34185	2,9509745	1,5611915	0,0070338	0,0701642	B_ITvar13 - erythrocyte membrane protein 1, PfEMP1, putative
PFIT_04143	18,1130849	96,39680533	47,37347826	85,76831253	201,7108708	43,74220703	256,9687327	238,0115477	82,18671	247,49014	3,0113158	1,590394	0,0070596	0,0702976	conserved Plasmodium protein, unknown function
PFIT_02161	619,24109	509,2541756	202,5839531	601,0401505	211,3819399	137,0589154	1157,560086	1223,2466	380,09337	1190,4033	3,1318708	1,6470247	0,0070727	0,0703043	conserved Plasmodium protein, unknown function
Pf_M76611	0	9,512842632	11,22003433	6,64132763	4,14474392	0	40,826808	29,39584852	5,2531581	35,111328	6,6838514	2,7406797	0,0070908	0,0703608	unspecified product
PFIT_09119	53,2071869	97,03099484	127,160389	128,9524448	178,2239886	186,6334167	415,4728108	315,7683083	128,53474	365,62056	2,8445272	1,5081889	0,0071186	0,0705129	GTP binding protein, putative
PFIT_13314	153,9612217	209,9167274	179,5205492	318,2302823	200,3292895	113,7297383	664,0360242	587,9169704	195,94797	625,9765	3,1946057	1,6756379	0,0071409	0,0706104	conserved Plasmodium membrane protein, unknown function
PFIT_06041	2,264135613	22,19663281	23,68673913	23,24464671	80,13171579	20,41302995	99,66544305	85,34278602	28,65615	92,504115	3,228072	1,6906727	0,0071807	0,0708797	conserved Plasmodium protein, unknown function
PFIT_03235	2798,471617	4584,555959	1434,294388	4845,955394	2321,056595	775,695138	9674,752706	8365,489536	2793,3382	9020,1211	3,2291547	1,6911566	0,0072585	0,0715231	RhS interacting protein (RIPR)
PFIT_09196	5,660339031	33,61204397	24,31007437	33,20663815	48,35534573	37,90991276	116,4764816	88,18754556	30,509059	102,33201	3,3541517	1,745948	0,0072732	0,0715425	sugar transporter, putative
PFIT_14413	229,8097647	492,1310588	146,4837815	231,3395791	1266,910058	209,9625937	1481,772973	1246,952929	429,43947	1364,363	3,1770786	1,6677008	0,0073117	0,0716755	conserved Plasmodium protein, unknown function
PFIT_04219	1202,25601	1664,113271	1040,346516	1334,35341	4358,889022	1195,620326	4835,575171	4559,20128	1799,2631	4697,3882	2,6107289	1,3844527	0,007312	0,0716755	early transcribed membrane protein 4
PFIT_12377	24,90549174	29,80690691	24,31007437	51,47028914	63,55274011	29,16147135	115,2756932	126,1176727	37,201162	120,69668	3,2444331	1,6979664	0,00734	0,0718254	COPI associated protein, putative
PFIT_09108	14,71688148	244,7169609	114,4305092	259,0117776	116,0528298	104,9812969	516,3390423	500,677678	150,89271	508,50836	3,3699995	1,7527484	0,0075175	0,0734116	SNARE protein, putative
PFIT_12466	639,6183105	3637,711022	1267,240544	3270,853858	2321,056595	562,8163971	6044,76916	6480,362219	1949,8828	6262,5657	3,2117652	1,6833664	0,0075281	0,0734116	myosin A tail domain interacting protein
PFIT_12090	834,3339732	1399,656246	374,6244794	949,1564072	665,9221898	347,0215095	2322,324902	2328,909805	761,7858	2325,6174	3,0528494	1,6101564	0,0076899	0,0747517	conserved Plasmodium protein, unknown function
PFIT_04180	208,3004764	324,070839	215,6739931	296,0925235	1678,621288	367,4345391	1836,005571	1566,51425	515,03228	1701,2599	3,3032103	1,7238688	0,0077003	0,0747517	conserved Plasmodium protein, unknown function
PFIT_11374	87,16922108	162,9867038	28,05008581	45,93584944	143,6844559	29,16147135	242,559271	254,1318517	82,831298	248,34556	2,9982092	1,5841011	0,0077051	0,0747517	conserved Plasmodium protein, unknown function
PFIT_05041	9,05654245	50,7351607	34,90677346	79,1424876	73,22380925	29,16147135	165,7088089	148,8757489	46,037707	157,29228	3,4165967	1,77256	0,0077979	0,0754332	AN1-like zinc finger family protein
PF_apicopla	12838,78099	11216,27565	7579,756522	7201,412927	2847,439073	3254,420203	26801,59866	19384,19146	7489,6809	23092,895	3,0832949	1,6244729	0,0078043	0,0754332	unspecified product
PFIT_05180	302,2621043	293,6297426	231,2573741	254,0307819	536,053547	134,1427682	902,992298	755,7577829	291,89605	829,375	2,841338	1,5065705	0,0078154	0,0754332	zinc finger protein, putative
PFIT_13113	167,5460353	194,6961792	109,7070023	128,9524448	475,2639695	84,56826693	618,4060623	445,6789937	193,45565	532,04253	2,7502041	1,4595387	0,0078601	0,0757351	conserved Plasmodium protein, unknown function
PFIT_08029	2445,266462	10084,24738	9702,213015	8053,163196	8945,738961	7272,870956	2139,805054	2436,062414	7750,5833	2287,9337	0,295195	-1,76026	0,0080236	0,0771794	Plasmodium exported protein (PHISTa), unknown function
PFIT_07215	3256,959079	2046,529545	1049,073209	1870,087172	1170,199367	387,847569	5407,150482	4341,103049	1630,116	4874,1268	2,990049	1,5801692	0,0080508	0,0773099	conserved Plasmodium protein, unknown function
PFIT_14236	9,05654245	131,2772283	97,24029749	97,40613858	230,7240782	113,7297383	365,039695	286,3724598	113,239	325,70608	2,8762711	1,5241997	0,0080846	0,0775027	conserved Plasmodium protein, unknown function
PFIT_13266	382,6389185	182,6465785	79,78691076	128,3990009	538,8167096	122,4781797	768,5046211	523,4357543	239,12772	645,97019	2,7013606	1,4336862	0,0081601	0,0780941	conserved Plasmodium protein, unknown function
PFIT_13479	18,1130849	27,90433839	11,84336957	15,49643114	29,01320744	11,66458854	74,44888517	64,4812161	19,005837	69,465051	3,6549325	1,8698448	0,0082439	0,0787631	conserved Plasmodium protein, unknown function
PFIT_12515	43,01857664	98,29937386	85,39692792	143,895432	323,2900258	160,3880924	410,6696569	346,11241	142,3814	378,39103	2,6575874	1,4101172	0,008321	0,0793653	thymidylate kinase
PFIT_10293	1719,610998	1906,373663	467,5014302	590,5247151	3873,953984	571,5648385	4683,075035	3942,836714	1521,5883	4312,9559	2,8345091	1,5030989	0,008451	0,0804697	conserved Plasmodium protein, unknown function
PFIT_11241	269,4321379	520,6695867	221,9073455	287,23742	460,0665751	195,3818581	1044,685969	857,2208729	325,78249	950,95342	2,9189826	1,5454656	0,0084682	0,0804972	leucine-rich repeat protein
PFIT_12532	931,6918046	338,6571977	398,9345538	366,3799076	334,3426762	361,6022448	84,05519293	100,5148369	455,26806	92,285015	0,2027048	-2,302548	0,0084951	0,0806176	gametocyte erythrocyte cytosolic protein
PFIT_01043	174,3384422	369,0982941	127,7837243	216,396592	1217,173131	134,1427682	1455,355626	1140,748573	373,15549	1298,0521	3,4785823	1,7984995	0,0085577	0,0806556	conserved Plasmodium protein, unknown function
PFIT_14038	321,507257	201,6722638	165,8071739	174,8882943	122,9607363	204,1302995	558,3666388	547,1420837	198,49434	552,75436	2,7847362	1,4775407	0,0085643	0,0806556	conserved Plasmodium protein, unknown function
PFIT_03212	1506,78225	622,1399081	419,5046167	506,9546758	864,869898	282,8662721	2142,206631	1654,701796	700,5196	1898,4542	2,7100658	1,4383279	0,008573	0,0806556	conserved Plasmodium protein, unknown function
PFIT_10114	119,9991875	371,0008626	233,1273799	309,3751788	599,6062871	309,1115964	973,8394495	808,8599608	323,70342	891,34971	2,7535999	1,461319	0,0085904	0,0806556	heme oxygenase
PFIT_03149	89,4333567	145,863587	40,51679062	58,66506073	212,7635212	20,41302995	308,6026369	244,6493199	94,609224	276,62598	2,9238796	1,5478839	0,0085945	0,0806556	conserved Plasmodium membrane protein, unknown function
PFIT_02055	1030,181704	2777,115859	1039,099846	2812,048807	1569,476364	848,5988164	5404,748906	5045,65516	1679,4202	5225,202	3,111313	1,6375235	0,0086046	0,0806556	metabolite/drug transporter, putative
PFIT_08356	91,69749231	65,95570891	73,5535583	64,7529444	88,4212036	93,31670833	204,13404	238,9598009	79,616269	221,54692	2,782684	1,4764771	0,0086124	0,0806556	erythrocyte membrane protein 1, PfEMP1, putative
PFIT_07113	18,1130849	10,14703214	4,986681922	5,534439692	15,19739437	11,66458854	0	0	10,940537	0	0	#DIV/0!	0,0086227	0,0806556	RESA-like protein
PFIT_07288	345,2806809	423,6385919	200,0906121	173,7814063	352,3032332	163,3042396	830,9456216	735,8444661	276,39979	783,39504	2,8342823	1,5029835	0,0086274	0,0806556	dynein heavy chain, putative
PFIT_06114	37,35823761	14,5863587	11,84336957	12,72921129	48,35534573	8,748441406	73,2480967	68,27422882	22,270161	70,761163	3,1773979	1,6678458	0,0087392	0,0815484	conserved Plasmodium protein, unknown function
PFIT_05336	336,2241385	719,8050925	299,2009153	364,1661317	1369,147075	542,4033672	1654,686512	1431,862299	605,15779	1543,2744	2,5502017	1,3506113	0,0087541	0,0815484	conserved Plasmodium protein, unknown function
PFIT_13542	198,1118661	494,0336273	375,8711499	456,5912746	1849,93737	489,9127187	2032,93488	1763,750911	644,07633	1898,3429	2,9473881	1,559437	0,0087662	0,0815484	DNA replication licensing factor MCM6 (MCM6)
PFIT_13118	12,45274587	227,0398441	70,43688215	154,4108674	497,3692704	67,07138411	605,1973891	534,8147924	171,4635	570,00609	3,3243582	1,7330758	0,0088383	0,0818914	branched-chain alpha keto-acid dehydrogenase, putative
PFIT_03194	219,6211544	132,5456073	105,3436556	132,2731086	478,0271321	81,65211979	541,5556002	461,7992977	191,57713	501,67745	2,6186709	1,3888347	0,0088444	0,0818914	kinesin-8, putative
PFIT_08316	463,0157328	187,7200946	81,03358124	135,5937725	269,4083548	69,98753125	653,2289279	452,3167659	201,12651	552,77285	2,7483838	1,4585835	0,0088465	0,0818914	conserved Plasmodium protein, unknown function
PFIT_01023	15,84894929	82,44463614	46,75014302	43,1686296	203,0924521	40,8260599	212,5395593	218,0982309	72,021812	215,3189	2,9896345	1,5799691	0,008951	0,0827238	actin-related protein
PFIT_13509	3174,318129	8749,278463	2047,656264	5835,513211	3926,454073	968,160849	13450,03166	12369,01445	4116,8968	12909,523	3,1357412	1,6488065	0,0089701	0,0827646	alveolin, putative
PFIT_03087	48,67891567	102,1045109	64,20352975	75,82182378	69,07906553	17,49688281	235,3545402	190,5988888	62,897455	212,97671	3,3860943	1,7596222	0,0090415	0,083288	conserved protein, unknown function
PFIT_12263	36,2261698	75,46855154	93,50028604	100,1733584	248,6846352	151,639651	333,8191948	281,6311939	117,61544	307,72519	2,6163673	1,3875651	0,0090759	0,0834687	proliferating cell nuclear antigen 2
PFIT_05035	419,9971561	1125,686378	337,8477002	791,424876	1054,146537	329,5246263	1906,852091	1994,176433	676,43788	1950,5143	2,8835083	1,5278252	0,0091017	0,0835704	actin-depolymerizing factor 1
PFIT_14161	410,9406137	245,4313399	104,7203204	184,2968417	211,3819399	116,6458854	654,4297164	534,8147924	212,23616	594,6225	2,801701	1,486303	0,0091488	0,0838665	conserved Plasmodium protein, unknown function
PFIT_10135	5,660339031	34,24623347	20,57006293	35,42041403	64,93432141	8,748441406	96,06307764	97,67007734	28,263302	96,866577	3,4272916	1,7770689	0,0091737	0,0839589	GDP-fucose synthase, putative
PFIT_07091	1019,993093	675,4118269	720,5755378	715,0496082	1417,502421	1711,778368	2628,525962	2322,272033	1043,3851	2475,399	2,3724691	1,2463893	0,0094316	0,0859782	conserved Plasmodium protein, unknown function
PFIT_08269	49,81098348	31,70947544	52,98349542	118,9904534	70,46064664	34,99376562	198,1300976	189,6506356	59,824803	193,89037	3,2409696	1,6964255	0,0094334	0,0859782	conserved Plasmodium protein, unknown function
PFIT_14661	4,528271225	36,78299151	23,06340389	27,11875449	70,46064664	5,832294271	105,6693854	86,2910392	27,964394	95,980212	3,4322293	1,779146	0,00944	0,0859782	conserved Plasmodium protein, unknown function
PFIT_04144	926,0314655	650,0442465	165,1838387	425,0449683	390,9875098	139,9750625	1373,70201	1179,626953	449,54452	1276,6645	2,8399067	1,5058435	0,0095286	0,0866457	RING zinc finger protein, putative
PFIT_13483	57,73545812	45,02745512	18,07672197	34,31352609	102,2370167	20,41302995	156,1025012	114,7386345	46,300535	135,42057	2,9248165	1,5483461	0,0096469	0,0875808	nucleoside diphosphate hydrolase, putative
PFIT_08292	13,58481368	39,95393905	17,45338673	34,86697006	127,1054802	46,65835417	145,2954049	116,6351409	46,603824	130,96527	2,810183	1,4906641	0,0097104	0,0880159	SPRY domain, putative
PFIT_11059	4134,311629	14278,1426	6664,700389	7194,218156	26719,78247	20165,15744	31549,51627	30000,83405	13192,719	30775,175	2,3327394	1,2220252	0,0097836	0,0885373	histone H2B
PFIT_06089	43,01857664	13,95216919	23,06340389	18,26365098	77,36855317	17,49688281	108,0709623	79,65326695	32,193873	93,862115	2,9155273	1,5437568	0,0099049	0,0894577	mitochondrial ribosomal protein L19 precursor, putative
PFIT_02161	597,7318017	484,5207847	186,3772368	592,738491	203,0924521	104,9812969	1057,894642	1126,524775	361,57368	1092,2097	3,0207113	1,5948883	0,0099169	0,0894577	conserved Plasmodium protein, unknown function
PFIT_13606	1,132067806	27,27014888	23,06340389	21,03087083	116,0528298	11,66458854	134,4883087	110,9456218	33,368985	122,71697	3,6775756	1,878755	0,010074	0,0907302	conserved Plasmodium protein, unknown function
PFIT_13589	24,90549174	87,51815221	23,06340389	18,26365098	81,51329709	11,66458854	147,6969819	115,6868877	41,154764	131,69193	3,1999196	1,6780356	0,0102003	0,091722	ADP-ribosylation factor, putative
PFIT_14263	172,0743066	438,8591401	178,2738787	340,921485	567,829917	177,8849753	999,0560074	773,7745933	312,64062	886,4153	2,8352532	1,5034776	0,0103049	0,09255	conserved Plasmodium protein, unknown function
PFIT_13316	53,2071869	277,7750048	267,4108181	216,396592	232,1056595	163,3042396	613,6029264	586,9687172	201,69992	600,28581	2,9761332	1,5734391	0,0103623	0,0928831	sedlin, putative
PFIT_07010	2061,495475	2031,943186	1714,795246	3555,324058	982,304309	431,589776	5995,536833	5337,717139	1796,242	5666,627	3,1547124	1,6575085	0,0104002	0,0930749	Plasmodium exported protein (PHISTb), unknown function
PFIT_02162	130,1877977	126,8379018	99,73363845	141,6816561	171,316082	160,3880924	396,2601953	351,801929	138,35753	374,03106	2,7033662	1,4347569	0,0105187	0,0939874	conserved Plasmodium membrane protein, unknown function
PFIT_05063	105,282306	64,05314039	67,32020595	91,87169889	116,0528298	69,98753125	255,7679442	227,5807627	85,761285	241,67435	2,8179889	1,4946659	0,0106149	0,0946972	conserved Plasmodium protein, unknown function
PFIT_14724	39,62237322	41,22231807	43,01013158	73,05460393	168,5529194	67,07138411	231,7521748	160,2547871	72,088955	196,00348	2,7189114	1,4430291	0,0106665	0,095007	conserved Plasmodium protein, unknown function
PFIT_12060	41,88650883	43,75907611	37,40011442	45,38240547	51,11850835	23,32917708	150,0985588	111,8837875	40,479298	130,99622	3,2361286	1,694269	0,0106912	0,0950778	rhodanese like protein, putative
PFIT_05296	59,99959373	64,6873299	54,85350115	146,1092079	216,9082651	40,8260599	292,9923868	265,5108898	97,23066	279,25164	2,8720533	1,5220825	0,0107193	0,0951776	G-protein coupled receptor, putative
PFIT_bin02	620,3731578	382,4162738	532,3282952	351,9903464	214,1451025	661,9653997	1025,473354	1315,227168	460,53643	1170,3503	2,5412762	1,3455532	0,010801	0,095677	stB_A4varTres - erythrocyte membrane protein 1,, putative
PFIT_13276	482,2608855	1113,636777	383,3511728	520,790775	1456,186697	813,6050508	2110,986131	1906,937141	794,97189	2008,9616	2,5270851	1,3374743	0,0108093	0,095677	conserved Plasmodium protein, unknown function
PFIT_05052	176,6025778	432,517245	218,7906693	414,5295329	513,9482461	507,4096016	1008,662315	955,8392034	377,29965	982,25076	2,6033705	1,3803806	0,0109399	0,0966129	actin-like protein, putative
PFIT_09342	40,75444103	40,58812856	31,16676201	25,45842258	102,2370167	23,32917708	157,3032896	98,61833051	43,922325	127,96081	2,9133433	1,5426757	0,0109492	0,0966129	conserved Plasmodium protein, unknown function
PFIT_12439	2209,796358	3444,917412	699,3821396	1797,032568	1780,858304	775,695138	5445,575714	4735,576371	1784,6137	5090,576	2,8524807	1,5122171	0,0110536	0,0971443	ubiquitin conjugating enzyme E2, putative
PFIT_14044	15,84894929	59,61381383	40,51679062	79,69593156	134,0133867	34,99376562	190,9253668	170,685572	60,78044	180,80547	2,9747312	1,5727593	0,0110566	0,0971443	conserved Plasmodium protein, unknown function
PFIT_12389	846,7867191	664,6306052	587,8051316	726,6719316	2203,622184	551,1518086	2545,671557	2101,329042	930,1114	2323,5003	2,4980882	1,3210524	0,0110653	0,0971443	transcription factor with AP2 domain(s)
PFIT_11398	13701,41666	5261,236165	8794,01357	7670,733413	5098,035022	12034,93923	2340,336729	2637,092088	8760,0623	2488,7144	0,2840978	-1,81554	0,0110782	0,0971443	RNA binding protein, putative
PFIT_03033	12,45274587	50,7351607	33,03676774	37,63418991	113,2896671	23,32917708	139,2914626	127,0659259	45,079618	133,17869	2,9542995	1,5628161	0,0111336	0,0974792	palmitoyl transferase
PFIT_08149	126,7915943	117,9592486	189,493913	139,4678802	81,51329709	139,9750625	39,6260953	33,18886123	132,5335	36,40744	0,2747037	-1,864052	0,0112789	0,0984992	conserved Plasmodium protein, unknown function
PFIT_03226	35,09410199	107,178027	56,10017163	70,84082806	120,1975737	84,56826693	226,9490209	217,1499778	78,996495	222,0495	2,8108779	1,4910208	0,0112849	0,0984992	iron-sulfur cluster assembly accessory protein, putative
PFIT_10061	20,37722051	39,95393905	61,08685355	57,5581728	41,4474392	64,15523698	159,7048666	131,8071917	47,42981	145,75603	3,073089	1,6196896	0,0113056	0,0985278	conserved Plasmodium protein, unknown function
PFIT_05160	33,96203419	109,0805955	110,3303375	132,8265526	352,3032332	440,3382174	536,7524463	423,8691706	196,4735	480,31081	2,4446596	1,2896336	0,0114435	0,0995762	protein phosphatase, putative
PFIT_13042	358,8654946	359,5854515	239,3607323	333,1732695	723,9486047	209,9625937	1098,72456	861,0138856	370,81602	979,86767	2,6424631	1,4018833	0,0114758	0,0997041	conserved Plasmodium protein, unknown function
PFIT_12320	5,660339031	82,44463614	46,75014302	41,50829769	216,9082651	113,7297383	237,7561172	211,4604587	84,500237	224,60829	2,6580788	1,4103839	0,0115157	0,0998974	lipoamide dehydrogenase
PFIT_12187	2188,28707	1359,068117	1180,596945	1344,315401	1816,779408	1644,706984	4441,716552	3628,964912	1588,959	4035,3407	2,5396129	1,3446086	0,0115659	0,100179	formin 2
PFIT_09149	21,50928832	43,1248866	32,4134325	42,06174166	259,7372857	55,40679557	282,1852906	225,6842564	75,708905	253,93477	3,3540939	1,7459231	0,0116168	0,1004662	GINS complex subunit Psf3, putative
PFIT_08016	260,3755954	114,1541116	246,8407552	188,1709495	49,73692704	29,16147135	15,61025012	21,80982309	148,07333	18,710037	0,1263566	-2,984427	0,0116722	0,1007908	rifin, pseudogene
PFIT_10224	0	22,19663281	11,84336957	30,43941831	31,77637005	2,916147135	72,04730823	59,73995021	16,528656	65,893629	3,9866295	1,9951695	0,0117276	0,101115	PfMNL-3 mitoNEET-like iron-sulfur protein, putative
PFIT_01038	53,2071869	169,3285988	48,62014874	104,0474662	587,1720553	84,56826693	622,0084277	553,779856	174,49062	587,89414	3,369202	1,7524069	0,0117925	0,101457	cyclase-associated protein, putative
PFIT_10081	5,660339031	22,83082232	8,103358124	8,855103507	17,96059599	5,832294271	40,826808	51,20567161	11,540412	46,01624	3,9874	1,9954483	0,0118032	0,101457	zinc finger C-x8-C-x5-C-x3-H type, putative
PFIT_02011	0	1,902568526	6,233352403	4,427551754	4,14474392	0	27,61813482	16,12030403	2,7847028	21,869219	7,8533406	2,9733065	0,0119037	0,1021318	knob associated heat shock protein 40 (KAHsp40)
PFIT_12146	3,396203419	19,02568526	6,233352403	8,30165958	45,59218312	2,916147135	58,83863505	42,67139301	14,244205	50,755014	3,5632044	1,8331752	0,0119178	0,1021318	thioredoxin peroxidase 2
PFIT_12365	16,98101709	64,05314039	31,79009725	38,18763387	179,6055699	23,32917708	222,145867	163,0995466	58,991106	192,62271	3,2652839	1,7072084	0,0120825	0,1033863	protein-S-isoprenylcysteine O-methyltransferase, putative
PFIT_02016	12,45274587	76,10274105	100,9803089	137,2541004	64,93432141	107,897444	18,01182706	21,80982309	83,270278	19,910825	0,2391108	-2,064249	0,0122294	0,1044856	Plasmodium exported protein (hyp11), unknown function
PFIT_14176	235,4701037	282,2143314	92,25361556	107,921574	193,4212829	49,5745013	486,3193305	421,024411	160,14258	453,67187	2,8329246	1,5022922	0,012253	0,1045297	conserved Plasmodium protein, unknown function
PFIT_bin00	32,82996638	42,49069709	16,20671625	16,60331908	63,55274011	17,49688281	108,0709623	81,54977331	31,530054	94,810368	3,006984	1,5883172	0,0123851	0,1054974	erythrocyte membrane protein 1, PfEMP1, putative
PFIT_13178	5,660339031	15,85473772	17,45338673	16,60331908	63,55274011	17,49688281	76,85046211	61,63645657	22,770234	69,243459	3,0409639	1,6045287	0,0125877	0,1070618	conserved Plasmodium protein, unknown function
PFIT_03148	62,26372935	79,90787811	20,57006293	23,24464671	167,1713381	37,90991276	194,5277322	153,6170148	65,177928	174,07237	2,6707258	1,4172319	0,0126278	0,1072412	conserved Plasmodium protein, unknown function
PFIT_04052	6,792406838	5,707705579	8,103358124	8,30165958	23,48688221	2,916147135	42,02759647	32,24060805	9,2180266	37,134102	4,0284221	2,0102149	0,0127915	0,1084686	conserved Plasmodium protein, unknown function
PFIT_03123	157,3574251	324,070839	213,8039874	267,8668811	946,3831951	452,002806	1005,05995	862,910392	393,58086	933,98517	2,3730452	1,2467396	0,0128608	0,1087659	glycogen synthase kinase 3
PFIT_04134	30,56583077	92,59166828	87,26693364	260,1186655	102,2370167	49,5745013	350,6302334	320,5095742	103,72577	335,5699	3,2351643	1,693839	0,012865	0,1087659	conserved Plasmodium protein, unknown function
PFIT_10082	777,7305829	1152,322337	1085,226653	1305,020879	6878,893326	1443,492832	6792,860377	6328,64171	2107,1144	6560,751	3,1136188	1,6385923	0,0129805	0,1094905	tubulin beta chain
PF_apicopla	381,5068507	1315,309041	719,9522025	1012,802464	2189,806371	559,90025	2737,797713	2674,073962	1029,8795	2705,9358	2,6274295	1,393652	0,0129894	0,1094905	elongation factor Tu, putative
PFIT_12411	389,4313254	220,0637595	330,3676774	281,1495364	134,0133867	411,1767461	746,8904286	766,1885678	294,36707	756,5395	2,5700548	1,3617991	0,0130768	0,1099217	erythrocyte membrane protein 1, PfEMP1
PFIT_bin07	56,60339032	71,02922498	72,30688787	111,7956818	150,5923624	87,48441406	241,3584826	274,9934216	95,017671	258,17595	2,7171362	1,4420869	0,0130794	0,1099217	stB_A4varICAM - erythrocyte membrane protein 1,, putative
PFIT_11478	311,3186467	341,8281452	211,3106465	281,7029803	476,6455508	137,0589154	792,5203905	771,8780869	293,31081	782,19924	2,666793	1,4151059	0,0134724	0,1130566	tubulin-tyrosine ligase, putative
PFIT_10168	134,7160689	156,0106192	92,25361556	124,5248931	214,1451025	154,5557982	397,4609829	353,6984354	146,03435	375,57971	2,5718587	1,3628114	0,0136044	0,1139951	golgi re-assembly stacking protein 2
PFIT_01085	35,09410199	41,85650758	48,62014874	81,90970744	91,18436624	43,74220703	176,5159052	157,4100276	57,06784	166,96297	2,9256928	1,5487783	0,0136745	0,1144125	conserved Plasmodium protein, unknown function
PFIT_05223	87,16922108	124,9353332	36,15344394	63,64605646	142,3028776	52,49064844	259,3703096	203,8744333	84,449596	231,62237	2,7427292	1,4556122	0,0138828	0,1159843	conserved Plasmodium protein, unknown function
PFIT_03079	15,84894929	27,90433839	35,5301087	38,74107784	103,618598	69,98753125	134,4883087	114,7386345	48,605101	124,61347	2,5637941	1,3582804	0,0139348	0,1162467	DNA polymerase epsilon subunit b, putative
PFIT_12386	666,7879379	1612,109731	566,6117334	1378,628927	754,343934	349,9376562	2648,939366	2570,714366	888,0699	2609,8269	2,9387629	1,555209	0,0140411	0,1169608	protein kinase 2
PFIT_12404	238,8663071	107,8122165	84,77359268	74,16149187	190,6582203	250,7886536	360,2365411	347,0606632	157,84341	353,6486	2,2405027	1,1638225	0,0141288	0,1175181	C_ITvar5 - erythrocyte membrane protein 1, PfEMP1, putative
PFIT_11058	1888,289101	11344,38193	4032,979005	5231,705841	19787,00747	5208,238784	21551,75147	20818,89852	7915,4337	21185,325	2,6764579	1,420325	0,0142987	0,1186604	histone H4
PFIT_13163	650,9389886	611,992876	417,0112758	539,60787	2644,346621	524,9064844	2988,762503	2208,481652	898,13402	2598,6221	2,8933567	1,5327442	0,0143177	0,1186604	DNA replication licensing factor MCM4 (MCM4)
PFIT_11350	422,2612917	390,6607374	190,1172483	267,3134371	592,6983806	151,639651	955,8276226	779,4641123	335,78179	867,64587	2,5839575	1,3695823	0,0143227	0,1186604	conserved Plasmodium protein, unknown function
PFIT_08138	1,132067806	5,707705579	3,116676201	2,767219846	24,86846352	8,748441406	31,2205023	24,65458263	7,7234291	27,937541	3,6172458	1,8548916	0,01435	0,1186604	conserved Plasmodium protein, unknown function
PFIT_03135	13,58481368	11,41541116	9,350028604	14,94298717	22,10530091	23,32917708	51,63390423	48,36091208	15,787953	49,997408	3,1668075	1,6630292	0,0144054	0,1189448	conserved Plasmodium protein, unknown function
PFIT_13189	26,03755954	31,70947544	42,38679634	30,43941831	113,2896671	20,41302995	121,2796355	121,3764068	44,045991	121,32802	2,7545758	1,4618302	0,0146725	0,1209729	ser/thr protein phosphatase 2A regulatory subunit A, putative
PFIT_03161	196,9797983	266,9937832	145,237111	354,7575843	302,5663062	107,897444	746,8904286	562,3141346	229,072	654,60228	2,8576267	1,5148175	0,0147038	0,1210547	mitochondrial ribosomal protein L27 precursor, putative
PFIT_06046	20,37722051	45,02745512	16,83005149	31,54630624	63,55274011	8,748441406	90,05913528	101,46309	31,013702	95,761113	3,0877033	1,6265341	0,0147564	0,1213115	conserved Plasmodium protein, unknown function
PFIT_10168	138,1122724	125,5695227	61,71018879	115,1163456	223,8161717	122,4781797	363,8389065	297,7514979	131,13378	330,7952	2,5225781	1,3348989	0,01484	0,1216644	golgi re-assembly stacking protein 2
PFIT_13461	99,62196695	66,58989842	20,57006293	21,03087083	270,7899361	40,8260599	288,1892329	233,2702818	86,571466	260,72976	3,0117286	1,5905918	0,0148424	0,1216644	conserved Plasmodium protein, unknown function
PFIT_02062	166,4139675	500,3755224	107,2136613	307,7148469	294,2768183	93,31670833	749,2920056	666,6219841	244,88525	707,95699	2,8909744	1,5315558	0,0149898	0,1226948	merozoite surface protein 5
PFIT_13410	4,528271225	101,4703214	34,28343822	47,04273738	140,9212933	20,41302995	194,5277322	155,5135212	58,109849	175,02063	3,0118297	1,5906706	0,0150526	0,1230311	conserved Plasmodium protein, unknown function
PFIT_bin00	43,01857664	35,51461249	40,51679062	42,06174166	52,50008965	37,90991276	109,2717508	128,9624322	41,920287	119,11709	2,8415142	1,5066599	0,015129	0,1234767	erythrocyte membrane protein 1, PfEMP1
PFIT_13052	114,3388484	108,446406	66,69687071	70,84082806	243,15831	58,32294271	313,4057908	249,3905858	110,3007	281,39819	2,5511913	1,3511711	0,0152303	0,1241247	conserved Plasmodium protein, unknown function
PFIT_14219	45,28271225	128,7404703	74,80022884	113,4560137	236,2504034	119,5620326	349,4294499	270,2521557	119,68198	309,8408	2,5888677	1,3723212	0,0153081	0,1245015	mannose-1-phosphate guanylyltransferase, putative
PFIT_13071	350,9410199	195,9645582	89,7602746	149,9833157	379,9348593	204,1302995	594,3902929	487,4021335	228,45239	540,89621	2,367654	1,2434583	0,0153206	0,1245015	tripartite motif protein, putative
PFIT_08193	16,98101709	58,34543481	26,80341533	45,93584944	301,1847248	52,49064844	325,4136755	252,2353454	83,623515	288,82451	3,4538671	1,7882126	0,0154597	0,1254524	conserved Plasmodium protein, unknown function
PFIT_02062	170,9422387	464,2267204	103,4736499	288,344308	251,4477978	78,73597266	678,4454858	628,691857	226,19511	653,56867	2,8894022	1,5307711	0,0157156	0,1272676	merozoite surface protein 5
PFIT_02140	2069,41995	2467,631379	621,4652346	1592,258299	1027,896492	390,7637161	3913,369625	3644,136963	1361,5725	3778,7533	2,7752861	1,4726365	0,0157284	0,1272676	serine/threonine protein kinase, putative
PFIT_07210	150,5650182	32,34366495	33,66010298	26,01186655	15,19739437	17,49688281	142,893828	157,4100276	45,879155	150,15193	3,2727701	1,7105123	0,0159845	0,1291544	conserved Plasmodium protein, unknown function
PFIT_14595	294,3376296	471,8369945	252,4507723	370,2540154	1172,962529	189,5495638	1283,642875	1093,335914	458,56525	1188,4894	2,5917563	1,3739301	0,016037	0,1293943	conserved Plasmodium protein, unknown function
PFIT_10009	9,05654245	39,95393905	22,44006865	23,24464671	44,21060181	14,58073568	69,64573129	91,03230509	25,581089	80,339018	3,1405629	1,6510231	0,0163333	0,1313062	Plasmodium exported protein (PHISTa), unknown function
PFIT_10198	89,4333567	154,1080506	112,2003433	126,185225	243,15831	128,310474	411,8704454	325,2508401	142,21263	368,56064	2,5912525	1,3736496	0,0163446	0,1313062	cytoplasmic dynein intermediate chain, putative
PFIT_10366	296,6017652	260,6518881	178,897214	223,5913636	302,5663062	247,8725065	701,2604667	568,9519068	251,69684	635,10619	2,5232982	1,3353107	0,0163628	0,1313062	conserved Plasmodium protein, unknown function
PFIT_13663	232,0739003	56,44286628	127,160389	113,4560137	59,40799619	154,5557982	27,61813482	27,49934216	123,84949	27,558738	0,222518	-2,168006	0,0163668	0,1313062	conserved Plasmodium protein, unknown function
PFIT_11374	66,79200057	101,4703214	19,32339245	26,56530152	122,9607363	26,24532422	152,5001357	165,9443062	60,559514	159,22222	2,6291859	1,3946162	0,0164397	0,1317043	conserved Plasmodium protein, unknown function
PFIT_07153	432,449902	1029,289573	287,980881	551,7836373	793,02767	282,8662721	1571,832108	1441,344831	562,89966	1506,5885	2,6764779	1,4203357	0,0165387	0,1323095	conserved Plasmodium protein, unknown function
PFIT_05243	83,77301766	136,9849339	47,9968135	62,53916852	116,0528298	49,5745013	256,9687327	201,0296737	82,820211	228,9992	2,7650159	1,4672878	0,0166879	0,1331632	conserved Plasmodium protein, unknown function, putative
PFIT_04233	258,1114598	350,7067984	294,2142334	268,973769	270,7899361	157,4719453	86,45676987	85,34278602	266,71136	85,899778	0,3220702	-1,634553	0,0166925	0,1331632	Plasmodium exported protein (hyp6), unknown function
PFIT_14572	259,2435276	233,3817392	105,3436556	148,3229837	674,2116776	107,897444	768,5046211	582,2274513	254,7335	675,36604	2,651265	1,4066809	0,0168436	0,1341791	transcription factor with AP2 domain(s)
PFIT_14710	294,3376296	71,02922498	81,65691648	96,85269461	120,1975737	96,23285547	373,4452143	258,8731176	126,71782	316,15917	2,4949859	1,3190317	0,0169121	0,1345359	conserved Plasmodium protein, unknown function
PFIT_14331	89,4333567	69,12665646	40,51679062	45,38240547	131,2502241	49,5745013	174,1143282	182,0646102	70,880656	178,08947	2,5125257	1,3291384	0,017206	0,1366814	conserved Plasmodium membrane protein, unknown function
PFIT_03038	0	10,78122165	0,62333524	3,874107784	22,10530091	5,832294271	27,61813482	27,49934216	7,20271	27,558738	3,8261625	1,9358981	0,0172403	0,1367621	conserved Plasmodium protein, unknown function
PFIT_03187	61,13166154	449,6403617	190,7405835	523,5579949	357,8295584	145,8073568	826,1424677	829,7215308	288,11792	827,932	2,8735873	1,5228529	0,0173738	0,1376279	trophozoite stage antigen
PFIT_10267	11111,24552	8361,788673	8661,243164	9924,357256	8002,118928	5963,520892	23539,05639	21434,31484	8670,7124	22486,686	2,5934069	1,3748486	0,0175904	0,1391487	merozoite capping protein 1
PFIT_09238	4,528271225	4,439326561	6,856687643	2,767219846	9,671069147	0	22,81498094	24,65458263	4,7104291	23,734782	5,0387728	2,3330724	0,0176705	0,139587	conserved protein, unknown function
PFIT_04082	312,4507145	453,4454988	384,5978433	428,3656322	875,9225484	947,747819	1358,09176	1168,247915	567,08834	1263,1698	2,2274657	1,1554032	0,0178711	0,1409751	replication protein A1, large subunit
PFIT_11192	512,8267162	348,8042298	450,6713787	565,0662926	823,4224588	586,1455742	1404,92251	1223,2466	547,82278	1314,0846	2,3987403	1,262277	0,0179648	0,1415166	haloacid dehalogenase-like hydrolase, putative
PFIT_12343	142,6405436	70,39503547	115,3170195	114,0094577	455,9218312	119,5620326	479,1145997	380,2495244	169,64099	429,68206	2,5328906	1,3407848	0,0180655	0,1421119	conserved Plasmodium protein, unknown function
PFIT_08314	33,96203419	40,58812856	18,07672197	19,37053892	146,4476185	14,58073568	157,3032896	124,2211663	45,504296	140,76223	3,0933832	1,6291856	0,0181427	0,1425212	translation initiation factor EIF-2b alpha subunit, putative
PFIT_12074	98,48989915	90,05491025	98,48696797	109,0284619	23,48688221	52,49064844	256,9687327	212,4087119	78,672962	234,68872	2,9830925	1,5768087	0,0182588	0,143234	conserved Plasmodium protein, unknown function
PFIT_03156	49,81098348	29,1727174	14,96004577	13,83609923	35,92111397	11,66458854	69,64573129	78,70501378	25,894258	74,175373	2,8645491	1,518308	0,0184298	0,1443756	conserved Plasmodium protein, unknown function
PFIT_10131	322,6393248	418,5650758	271,1508295	301,0735192	1302,831172	259,5370951	1462,560357	1083,853382	479,2995	1273,2069	2,656391	1,4094675	0,0186493	0,1458925	conserved Plasmodium protein, unknown function
PFIT_04193	637,3541749	383,6846528	445,0613616	341,474929	292,895237	1058,56141	934,21343	1307,641132	526,50529	1120,9273	2,1289953	1,0901727	0,0187546	0,1465142	erythrocyte membrane protein 1, PfEMP1
PFIT_13418	5141,851976	14768,37109	5082,675549	15000,54534	7758,960618	2055,88373	24093,82066	23581,16003	8301,3814	23837,49	2,8715089	1,521809	0,0187924	0,146607	myosin A
PFIT_03136	27,16962735	25,36758035	33,03676774	30,99286228	118,8159924	32,07761849	128,4843663	99,56658369	44,576741	114,02548	2,557959	1,3549931	0,0189715	0,1477998	conserved Plasmodium protein, unknown function
PFIT_13459	4,528271225	22,83082232	15,58338101	12,17576732	16,57897568	8,748441406	50,43311576	48,36091208	13,40761	49,397014	3,684252	1,8813718	0,0190758	0,1484085	transmission blocking target antigen precursor
PFIT_07061	1082,256823	654,4835731	509,2648913	649,7432198	1080,396582	393,6798633	1878,033168	1677,459872	728,30416	1777,7465	2,4409397	1,2874366	0,0191066	0,1484435	conserved Plasmodium protein, unknown function
PFIT_12152	43,01857664	17,75730625	7,480022884	8,855103507	56,64483357	2,916147135	61,24021199	63,53296293	22,778665	62,386587	2,7388167	1,4535527	0,0192782	0,1495717	conserved Plasmodium protein, unknown function
PFIT_13275	367,922037	459,1532044	265,5408124	340,3680411	1250,331083	431,589776	1270,434202	1072,474344	519,15083	1171,4543	2,2564816	1,174075	0,0193539	0,1499527	conserved Plasmodium protein, unknown function
PFIT_14497	1590,555268	1096,513661	939,9895424	1213,14918	2293,424969	772,7789909	3565,140969	2818,208445	1317,7353	3191,6747	2,422091	1,2762531	0,0195353	0,1511511	conserved Plasmodium protein, unknown function
PFIT_09001	104,1502382	49,46678168	34,90677346	30,43941831	124,3423176	166,2203867	182,5198475	166,8925593	84,920986	174,7062	2,0572795	1,0407378	0,019586	0,1513368	B_ITvar41 - erythrocyte membrane protein 1, PfEMP1, putative
PFIT_09275	23,77342393	35,51461249	24,31007437	22,13775877	30,39478875	14,58073568	93,6615007	66,37772246	25,118566	80,019612	3,185676	1,6715995	0,0196778	0,1518383	conserved Plasmodium protein, unknown function
PFIT_06124	161,8856963	267,6279727	104,7203204	173,7814063	660,3958646	169,1365339	700,0596783	572,7449195	256,25797	636,4023	2,483444	1,3123422	0,0198304	0,1526888	apicoplast ribosomal protein L18 precursor, putative
PFIT_14160	55,47132251	52,00353972	43,01013158	76,92871172	225,197753	43,74220703	222,145867	203,8744333	82,725611	213,01015	2,5748997	1,3645162	0,0198423	0,1526888	uracil-DNA glycosylase, putative
PFIT_07111	28,30169516	71,66341449	127,7837243	89,65792301	31,77637005	96,23285547	16,81103859	22,75807627	74,235997	19,784557	0,2665089	-1,907744	0,019869	0,1526888	rifin
PFIT_03064	21,50928832	64,05314039	41,14012586	44,27551754	230,7240782	26,24532422	230,5513863	209,5639523	71,324579	220,05767	3,0852992	1,6254104	0,0200422	0,153811	conserved Plasmodium protein, unknown function
PFIT_05091	301,1300365	134,4481759	135,2637471	135,0403285	435,1981116	145,8073568	494,7248498	476,9713485	214,48129	485,8481	2,2652237	1,1796535	0,0204232	0,1565226	chromosome condensation protein, putative
PFIT_02007	0	0,634189509	13,09004005	4,427551754	0	0	24,01576941	19,91331674	3,0252969	21,964543	7,2602934	2,8600279	0,0204744	0,1567028	Plasmodium exported protein (hyp10), unknown function
PFIT_13032	130,1877977	434,4198135	91,00694508	123,4180051	607,8957749	87,48441406	791,319602	602,140768	245,73546	696,73019	2,8352855	1,503494	0,0205068	0,1567385	chromosome condensation protein, putative
PFIT_05132	172,0743066	365,2931571	378,3644909	426,7053003	746,0539056	390,7637161	1059,095431	950,1496844	413,20915	1004,6226	2,4312689	1,2817095	0,0205887	0,1571519	conserved Plasmodium protein, unknown function
PFIT_04065	1009,804483	1797,293068	716,8355263	1753,310494	1019,607004	793,1920208	3406,636891	2924,412801	1181,6738	3165,5248	2,6788484	1,4216129	0,0206192	0,1571724	AAA family ATPase, putative
PFIT_11323	4,528271225	15,22054821	7,480022884	7,1947716	34,53953267	5,832294271	38,42523105	42,67139301	12,465907	40,548312	3,2527367	1,701654	0,0207659	0,1580782	conserved Plasmodium protein, unknown function
PFIT_10027	0	6,341895088	5,610017163	6,087883661	16,57897568	5,832294271	28,81892329	25,60283581	6,7418443	27,21088	4,0361181	2,0129684	0,0208616	0,1584119	conserved Plasmodium protein, unknown function
PFIT_04054	1,132067806	20,92825379	23,06340389	28,7790864	64,93432141	17,49688281	97,26386611	58,79169704	26,055669	78,027782	2,9946566	1,5823906	0,0208658	0,1584119	conserved Plasmodium protein, unknown function
PFIT_02069	1343,764486	3820,357601	1433,047717	2664,279268	25848,00467	2615,78398	26063,11375	20677,6088	6287,5396	23370,361	3,7169326	1,8941125	0,0209595	0,1589103	serine repeat antigen 5
PFIT_11443	19,24515271	10,14703214	9,973363845	16,04987511	22,10530091	17,49688281	55,23626964	42,67139301	15,836268	48,953831	3,091248	1,6281894	0,0210294	0,1592265	conserved Plasmodium protein, unknown function
PFIT_03090	0	16,48892723	13,71337529	21,5843148	19,34213829	14,58073568	46,83075035	52,15392479	14,284915	49,492338	3,4646574	1,7927127	0,0213558	0,1614815	conserved Plasmodium protein, unknown function
PFIT_10360	124,5274587	83,07882565	72,93022311	81,35626347	287,3689118	64,15523698	325,4136755	238,9598009	118,90282	282,18674	2,3732552	1,2468673	0,0214189	0,1617429	acetyl-CoA transporter, putative
PFIT_05046	69,05613618	138,8875024	92,8769508	80,8028195	379,9348593	285,7824193	407,0672915	343,2676504	174,55678	375,16747	2,1492575	1,1038383	0,0214914	0,1620736	3-methyl-2-oxobutanoate dehydrogenase (lipoamide), putative
PFIT_08253	215,0928832	216,2586225	579,7017735	515,2563353	234,8688221	530,7387786	123,6812125	130,8589386	381,9862	127,27008	0,3331798	-1,585627	0,0216066	0,1627256	conserved Plasmodium protein, unknown function
PFIT_03063	35,09410199	215,624433	107,8369966	120,6507853	688,0274907	128,310474	698,8588898	600,2442617	215,92405	649,55158	3,008241	1,5889202	0,021929	0,1649344	FAD-dependent glycerol-3-phosphate dehydrogenase, putative
PFIT_09207	189,0553236	189,6226631	54,72350687	85,21037126	234,8688221	49,5745013	389,0554644	281,6311939	134,1792	335,34333	2,49922	1,3214779	0,0223842	0,168134	conserved Plasmodium protein, unknown function
PFIT_14576	99,62196695	152,2054821	77,2935698	141,6816561	122,9607363	46,65835417	327,8152524	264,5626367	106,73696	296,18894	2,7749426	1,472458	0,0224321	0,168205	conserved Plasmodium protein, unknown function
PFIT_04231	1828,289507	1516,347115	1881,84909	2079,842436	1320,791729	1673,868456	4067,070549	4394,205227	1716,8314	4230,6379	2,4642128	1,3011268	0,0226086	0,16937	Plasmodium exported protein (PHISTa), unknown function
PFIT_04009	965,6538388	805,4206762	607,7518593	1577,868756	421,3822985	504,4934544	2119,39165	2232,187981	813,76181	2175,7898	2,6737428	1,4188607	0,0226925	0,1697738	surface-associated interspersed protein 4.1 (SURFIN 4.1) (SURF4.1)
PFIT_11114	3,396203419	50,10097119	27,42675057	46,48929341	128,4870615	8,748441406	139,2914626	128,9624322	44,10812	134,12695	3,0408675	1,6044829	0,0227997	0,1703504	mitochondrial ribosomal protein L11 precursor, putative
PFIT_04128	6,792406838	3,170947544	4,986681922	9,408547476	60,78957749	8,748441406	68,44494282	43,61964619	15,649434	56,032295	3,5804678	1,8401481	0,022916	0,170993	calmodulin-like protein
PFIT_14621	7,924474644	9,512842632	7,480022884	6,087883661	41,4474392	11,66458854	33,62207717	44,56789937	14,019542	39,094988	2,7886067	1,4795445	0,0234155	0,17449	apicoplast ribosomal protein S10 precursor, putative
PFIT_07108	11319,546	4717,101566	5714,114148	3726,891689	2021,253452	8815,51279	11445,9157	14752,92196	6052,4033	13099,419	2,1643335	1,1139228	0,0234739	0,1746954	erythrocyte membrane protein 1, PfEMP1
PFIT_05165	349,8089521	232,1133602	190,1172483	230,2326912	306,7110501	192,4657109	673,6423319	539,5560583	250,2415	606,5992	2,4240551	1,2774225	0,0236379	0,1756847	phosducin-like protein, putative (PhLP3)
PFIT_13651	125,6595265	387,4897899	263,6708066	448,289615	263,8820296	262,4532422	833,3471985	720,6724153	291,9075	777,00981	2,6618357	1,4124215	0,0237216	0,1760752	conserved Plasmodium protein, unknown function
PFIT_14232	71,3202718	145,863587	107,8369966	117,3301215	210,0003586	116,6458854	357,8349642	281,6311939	128,1662	319,73308	2,4946754	1,3188521	0,0238267	0,1766235	conserved Plasmodium protein, unknown function
PFIT_07119	110,942645	75,46855154	47,37347826	53,68406501	87,03962232	40,8260599	192,1261553	162,1512934	69,222404	177,13872	2,5589797	1,3555687	0,0239952	0,1776396	conserved Plasmodium protein, unknown function
PFIT_11011	1939,232152	1238,572111	1034,113164	1001,18014	860,725154	845,6826693	384,2523105	412,4901324	1153,2509	398,37122	0,3454333	-1,533521	0,0242131	0,1790189	Plasmodium exported protein (hyp5), unknown function
PFIT_13616	13,58481368	5,707705579	13,09004005	18,81709495	20,7237196	8,748441406	37,22444258	50,25741843	13,445303	43,74093	3,25325	1,7018817	0,0244789	0,1807472	conserved Plasmodium protein, unknown function
PFIT_13556	0	5,07351607	4,363346682	1,660331908	5,526325227	0	21,61419247	14,22379767	2,7705866	17,918995	6,4675815	2,6932263	0,024792	0,1828211	conserved Plasmodium protein, unknown function
PFIT_12309	204,9042729	505,4490385	98,48696797	216,9500359	237,6319847	90,4005612	593,1895044	595,5029958	225,63714	594,34625	2,6340798	1,3972991	0,0248724	0,1831752	conserved Plasmodium protein, unknown function
PFIT_13176	218,4890866	253,041614	157,0804806	160,4987511	613,4221002	312,0277435	667,6383896	561,3658814	285,75996	614,50214	2,1504137	1,1046142	0,0249753	0,1836936	chromosome segregation protein, putative
PFIT_04210	12,45274587	31,07528593	21,81673341	35,42041403	120,1975737	29,16147135	115,2756932	105,2561028	41,687371	110,2659	2,6450672	1,4033044	0,0251872	0,1850117	serpentine receptor, putative
PFIT_11285	2634,321785	1437,707616	437,5813387	1059,291757	1352,568099	603,642457	2967,148311	2843,811288	1254,1855	2905,4798	2,3166268	1,2120257	0,0252517	0,1852449	CRAL/TRIO domain-containing protein, putative
PFIT_04049	1486,40503	2206,979491	1699,8352	1617,716722	1728,358215	3820,152747	4301,224301	4721,352573	2093,2412	4511,2884	2,1551689	1,1078009	0,0254894	0,1866988	sexual stage-specific protein precursor
PFIT_10078	45,28271225	25,36758035	16,20671625	13,83609923	93,94752888	20,41302995	78,05125058	78,70501378	35,842278	78,378132	2,1867167	1,1288021	0,0255159	0,1866988	endonuclease, putative
PFIT_13423	26,03755954	243,5287714	134,6404119	172,1210744	1297,304847	256,6209479	1221,201874	1062,043559	355,04227	1141,6227	3,2154558	1,6850233	0,0256141	0,1868367	conserved Plasmodium protein, unknown function
PFIT_14428	132,4519333	218,7953805	228,1406979	229,6792472	353,6848145	253,7048008	616,0044853	512,0567161	236,07615	564,0306	2,3891893	1,2565212	0,0256186	0,1868367	acid cluster protein 33 homologue, putative
PFIT_14728	26,03755954	17,12311674	3,116676201	13,83609923	13,81581307	5,832294271	34,82286564	50,25741843	13,293593	42,540142	3,2000484	1,6780937	0,0256139	0,1868367	conserved Plasmodium protein, unknown function
PFIT_09304	140,376408	449,0061722	326,0043307	793,0852079	335,7242575	169,1365339	1113,130912	960,5804693	368,88882	1036,8557	2,8107539	1,4909571	0,025677	0,1869098	leucine-rich repeat protein
PFIT_14454	10,18861026	16,48892723	8,726693364	12,17576732	46,97376443	11,66458854	48,03153882	51,20567161	17,703059	49,618605	2,8028267	1,4868825	0,0257575	0,1872551	calmodulin-like protein
PF_apicopla	6,792406838	9,512842632	21,81673341	14,94298717	8,28948784	2,916147135	45,62996188	39,82663348	10,711768	42,728298	3,988912	1,9959953	0,0258127	0,1874155	probable protein, unknown function
PFIT_08146	283,0169516	85,61558369	65,45020023	67,52016424	193,4212829	32,07761849	343,4255026	256,0283581	121,18365	299,72693	2,4733281	1,3064536	0,0258656	0,1875588	conserved Plasmodium protein, unknown function
PFIT_10037	109,8105772	200,4038848	497,4215218	449,949947	134,0133867	469,4996888	72,04730823	74,91200106	310,18317	73,479655	0,2368912	-2,077704	0,0261198	0,1891595	hypothetical protein
PFIT_14147	1588,291132	1791,585362	643,9053032	1894,438707	960,1990081	749,4498138	3565,140969	2964,239434	1271,3116	3264,6902	2,5679702	1,3606285	0,0262096	0,1893616	conserved Plasmodium protein, unknown function
PFIT_06282	2465,643682	483,2524057	164,5605034	333,7267134	323,2900258	212,8787409	2279,096517	2235,032741	663,89201	2257,0646	3,3997466	1,7654272	0,0262424	0,1893616	phospholipase, putative
PFIT_14709	35,09410199	149,0345346	90,38360984	123,4180051	258,3557043	236,207918	347,027868	311,9752956	148,74898	329,50126	2,2151519	1,1474056	0,0262481	0,1893616	conserved Plasmodium protein, unknown function
PFIT_14683	1081,124755	513,0593126	635,1786099	566,1731805	458,6849938	361,6022448	178,9174821	238,0115477	602,63718	208,46451	0,3459204	-1,531488	0,0264364	0,1904769	conserved Plasmodium protein, unknown function
PFIT_13080	225,2814934	199,1355058	155,8338101	163,2659709	502,8955956	247,8725065	584,7839851	500,677678	249,04748	542,73083	2,1792263	1,1238161	0,0265756	0,1912362	tyrosine recombinase (INT)
PFIT_14617	474,3364108	275,8724363	319,7709783	359,73858	613,4221002	472,4158359	967,8355072	880,9272024	419,25939	924,38135	2,2047958	1,1406451	0,0265739	0,1922746	conserved Plasmodium protein, unknown function
PFIT_09114	5,660339031	13,95216919	6,233352403	10,51543541	52,50008965	2,916147135	46,83075035	49,30916526	15,296255	48,069958	3,1425964	1,651957	0,0268892	0,1929041	conserved Plasmodium protein, unknown function
PFIT_11127	0	15,22054821	9,973363845	11,62232335	22,10530091	11,66458854	36,02365411	41,72313983	11,764354	38,873397	3,3043375	1,7243611	0,0269097	0,1929041	conserved Plasmodium protein, unknown function
PFIT_03045	9,05654245	27,90433839	18,07672197	12,72921129	9,671069147	49,5745013	49,23232729	61,63645657	21,168731	55,434392	2,6186923	1,3888465	0,0270536	0,1934163	circumsporozoite (CS) protein
PFIT_05136	53,2071869	90,05491025	97,24029749	80,24937566	58,02641488	34,99376562	34,82286564	11,37903814	68,961992	23,100952	0,3349809	-1,577849	0,0270551	0,1934163	mitochondrial import inner membrane translocase subunit TIM16, putative (PAM16)
PFIT_14630	177,7346456	202,9406428	192,6105893	267,8668811	134,0133867	104,9812969	473,1106574	479,8161081	180,02457	476,46338	2,6466575	1,4041715	0,0271086	0,1934163	conserved Plasmodium protein, unknown function
PFIT_12265	89,4333567	271,4310398	160,820492	206,9880445	334,3426762	335,3569206	530,7485039	522,4875011	233,06243	526,618	2,2595576	1,1760404	0,0271319	0,1934163	ataxin-3, putative (ATX3)
PFIT_03190	216,224951	901,183292	395,1945423	473,1945937	2326,58292	475,3319831	2454,411634	1949,608534	797,95205	2202,0101	2,759577	1,4644471	0,0272502	0,1938424	P-type ATPase, putative
PFIT_02163	36,2261698	26,00176986	11,84336957	11,62232335	24,86846352	23,32917708	57,63784658	61,63645657	22,315212	59,637152	2,6724887	1,4181838	0,0272702	0,1938424	conserved Plasmodium protein, unknown function
PFIT_10179	67,92406838	74,20017253	150,8471282	149,9833157	96,71069147	218,7110352	49,23232729	30,3441017	126,39607	39,788214	0,31479	-1,667539	0,0273039	0,1938424	conserved Plasmodium protein, unknown function
PFIT_05031	613,580751	777,5163378	239,9840675	420,0639726	559,5404292	332,4407734	1233,209759	1118,93875	490,52106	1176,0743	2,397602	1,2615922	0,0273148	0,1938424	50S ribosomal protein L28, apicoplast, putative
PFIT_04066	379,2427151	624,0424766	398,3112185	827,3987339	418,6191359	227,4594766	1293,249183	1250,745942	479,17896	1271,9976	2,6545355	1,4084594	0,027395	0,1941683	conserved Plasmodium protein, unknown function
PFIT_04125	529,8077333	272,0672993	241,8540732	268,973769	751,5802308	492,8288659	970,2370841	807,9117077	426,18533	889,0744	2,0861215	1,0608232	0,0275426	0,19497	chromosome associated protein, putative
PFIT_11009	1983,382797	1123,78381	1064,033255	1032,726447	869,0146419	819,437345	404,6657145	412,4901324	1148,7297	408,57792	0,355678	-1,491356	0,0277538	0,1962197	Plasmodium exported protein (hyp5), unknown function
PFIT_11025	202,6401373	230,8449812	60,46351831	120,0973413	163,0265942	93,31670833	312,2050023	382,1460307	145,06488	347,17552	2,3932431	1,2589669	0,0279695	0,1973511	Plasmodium repeat_MYXSPDY protein, putative
PFIT_13159	31,69789858	36,148802	18,07672197	24,90497861	16,57897568	29,16147135	87,65755834	62,58470975	26,094808	75,121134	2,878777	1,5254561	0,0279837	0,1973511	conserved Plasmodium protein, unknown function
PFIT_01042	506,0343094	389,3923584	349,6910698	363,6126878	602,3694497	521,9903372	1029,075719	975,7525202	455,51504	1002,4141	2,200617	1,1379081	0,0286449	0,2017627	asparagine-rich antigen Pfa35-2
PFIT_08293	15,84894929	60,24800333	34,28343822	47,04273738	243,15831	46,65835417	226,9490209	204,8226865	74,539965	215,88585	2,896243	1,5341827	0,0287503	0,2022532	dehydrodolichyl diphosphate synthetase, putative
PFIT_07329	82,64094986	29,1727174	17,45338673	14,3895432	82,8948784	23,32917708	98,46465458	92,92881145	41,646775	95,696733	2,2978185	1,2002649	0,0290516	0,2041189	acyl-CoA synthetase
PFIT_03214	234,3380359	149,6687241	109,0836671	112,3491257	161,6450129	55,40679557	409,4688684	274,0451684	137,08189	341,75702	2,4930865	1,3179329	0,0291052	0,2042421	conserved Plasmodium protein, unknown function
Pf_M76611	22,64135613	345,6332823	122,7970423	159,3918631	415,8559733	64,15523698	475,5122343	513,9532225	188,41246	494,73273	2,6257963	1,392755	0,0292176	0,2047768	cytochrome c oxidase subunit 3
PFIT_10247	40,75444103	34,88042298	21,19339817	29,33253037	91,18436624	37,90991276	108,0709623	93,87706462	42,542512	100,97401	2,373485	1,2470069	0,0296638	0,2076467	conserved Plasmodium protein, unknown function
PFIT_10170	3014,696568	3489,944867	1460,474468	3377,668544	1981,187594	790,2758737	6066,383353	5880,117957	2352,3747	5973,2507	2,5392429	1,3443984	0,0298758	0,2088721	conserved Plasmodium protein, unknown function
PFIT_03206	28,30169516	24,09920133	16,83005149	22,69120274	91,18436624	20,41302995	79,25203905	83,44627967	33,919924	81,349159	2,3982707	1,2619945	0,0300495	0,209725	conserved Plasmodium protein, unknown function
PFIT_14407	17571,95649	8301,54067	3829,148381	8253,509913	4231,783542	1589,300189	19911,47442	17239,24278	7296,2065	18575,359	2,5458927	1,3481716	0,030072	0,209725	CCAAT-binding transcription factor, putative
PFIT_08318	249,0549174	185,8175261	72,93022311	156,6246433	288,7504931	81,65211979	414,2720223	385,9390435	172,47165	400,10553	2,3198336	1,2140213	0,0301308	0,2098766	conserved Plasmodium protein, unknown function
PFIT_12506	123,3953909	31,07528593	26,80341533	34,86697006	78,75013448	20,41302995	114,0749047	130,8589386	52,550704	122,46692	2,3304525	1,2206101	0,030276	0,2105221	conserved Plasmodium protein, unknown function
PFIT_07285	11,32067806	20,29406428	36,77677918	53,13062104	120,1975737	52,49064844	124,8820009	107,1526091	49,035061	116,01731	2,3660072	1,2424544	0,0303122	0,2105221	conserved Plasmodium protein, unknown function
PFIT_05049	22,64135613	34,88042298	14,96004577	23,79809068	92,56594755	20,41302995	80,45282752	89,13579873	34,876482	84,794313	2,4312748	1,281713	0,0303351	0,2105221	conserved protein, unknown function
PFIT_07305	399,6199356	255,578372	476,2281236	393,4986621	810,988227	831,1019336	1227,205817	960,5804693	527,83588	1093,8931	2,0724115	1,0513105	0,0303857	0,2106144	acyl-CoA synthetase (ACS5)
PFIT_10268	1198,859807	793,3710755	912,5627918	662,4724311	650,7247954	650,3008112	261,7718866	317,6648146	811,38195	289,71835	0,3570678	-1,48573	0,030496	0,2111201	DNA-directed RNA polymerase II, putative
PFIT_12496	40,75444103	53,27191874	16,20671625	15,49643114	96,71069147	26,24532422	109,2717508	89,13579873	41,447587	99,203775	2,3934753	1,2591069	0,0308769	0,2134962	conserved Plasmodium protein, unknown function
PFIT_12388	31,69789858	61,51638235	90,38360984	112,3491257	136,7765494	67,07138411	210,1379823	207,667446	83,299158	208,90271	2,507861	1,3264574	0,0311935	0,21511	mitochondrial ribosomal protein L23 precursor, putative
PFIT_13473	46,41478006	69,76084597	59,21684783	74,16149187	124,3423176	26,24532422	187,3230014	157,4100276	66,690268	172,36651	2,5845827	1,3699314	0,0312191	0,21511	GTPase, putative
PFIT_14321	0	51,36935021	16,20671625	15,49643114	183,7503138	14,58073568	162,1064435	162,1512934	46,900591	162,12887	3,4568619	1,789463	0,0312244	0,21511	cytochrome c oxidase subunit 2, putative
PFIT_11218	250,1869852	76,10274105	72,93022311	114,0094577	233,4872408	72,90367838	363,8389065	254,1318517	136,60339	308,98538	2,2619159	1,1775453	0,0312762	0,2152047	zinc finger, DHHC-type, putative
PFIT_02114	11,32067806	50,7351607	67,94354119	77,48215569	168,5529194	81,65211979	205,3348284	154,565268	76,281096	179,95005	2,3590386	1,238199	0,0313546	0,2154483	GDP-fructose:GMP antiporter, putative
PFIT_05019	237,7342393	188,3542841	134,6404119	117,3301215	461,4481564	207,0464466	524,7445616	424,8174238	224,42561	474,78099	2,1155384	1,0810249	0,0313878	0,2154483	chromosome assembly factor 1
PFIT_13437	9,05654245	71,66341449	22,44006865	28,22564243	171,316082	26,24532422	200,5316746	134,6519513	54,824512	167,59181	3,0568774	1,6120587	0,0314716	0,2157615	conserved Plasmodium protein, unknown function
PFIT_12030	28,30169516	67,22408793	38,6467849	53,13062104	186,5134764	49,5745013	166,9095974	166,8925593	70,565194	166,90108	2,365204	1,2419646	0,0315398	0,2159674	conserved Plasmodium protein, unknown function
PFIT_07033	376,9785795	575,844074	370,2611327	404,5675415	1077,633419	644,4685169	1280,04051	1165,403156	574,95888	1222,7218	2,1266248	1,0885656	0,0316566	0,2165052	phosphoinositide-binding protein, putative
PFIT_13399	109,8105772	74,20017253	74,17689359	75,82182378	292,895237	128,310474	296,5947522	232,3220286	125,8692	264,45839	2,1010573	1,0711155	0,0317511	0,2168893	Rab GTPase 11b
PFIT_02110	9,05654245	19,02568526	21,19339817	27,11875449	44,21060181	29,16147135	66,04336588	66,37772246	24,961076	66,210544	2,6525517	1,4073809	0,0318592	0,2173652	dolichol-linked oligosaccharide biosynthesis enzyme, putative
PFIT_03037	15,84894929	46,29583414	45,50347254	38,18763387	60,78957749	52,49064844	132,0867318	91,03230509	43,186019	111,55952	2,5832323	1,3691774	0,0319415	0,2176637	IBR domain protein, putative
PFIT_14772	84,90508547	27,27014888	92,25361556	74,16149187	23,48688221	125,3943268	15,61025012	18,96506356	71,245258	17,287657	0,2426499	-2,043052	0,0320246	0,2179678	early transcribed membrane protein 14.2
PFIT_11420	191,3194593	199,1355058	86,6435984	135,5937725	712,8959542	128,310474	686,8510051	565,1588941	242,31646	626,00495	2,583419	1,3692817	0,0320841	0,2181104	conserved Plasmodium protein, unknown function
PFIT_14265	5,660339031	13,95216919	6,856687643	14,3895432	15,19739437	2,916147135	36,02365411	36,03362076	9,8287134	36,028637	3,6656514	1,8740696	0,0323052	0,2193493	conserved Plasmodium protein, unknown function
PFIT_07166	24,90549174	134,4481759	62,95685927	85,78381523	207,237196	137,0589154	237,7561172	245,5975731	108,73174	241,67685	2,222689	1,1523061	0,0326096	0,2213134	mitochondrial ribosomal protein S8 precursor, putative
PFIT_14334	929,427669	329,1443551	168,3005149	219,7172558	489,0797826	236,207918	987,0481227	765,2403147	395,31292	876,14422	2,2163309	1,1481733	0,0327021	0,2215126	conserved Plasmodium protein, unknown function
PFIT_12031	654,335192	355,7803144	294,2142334	367,4867955	509,8035022	227,4594766	990,6504881	847,7383411	401,51325	919,19441	2,2893252	1,1949224	0,0327978	0,2218951	transporter, putative
PFIT_01070	33861,28015	11588,54489	15811,52171	16365,89161	5147,771949	12043,68767	36882,21787	35756,73084	15803,116	36319,474	2,2982476	1,2005342	0,0330961	0,2233416	conserved Plasmodium protein, unknown function
PFIT_06130	237,7342393	758,4906525	226,8940275	606,5745902	606,5141936	186,6334167	1156,359297	1044,026749	437,14019	1100,193	2,5167968	1,3315887	0,0331767	0,2233416	thiamin-phosphate pyrophosphorylase, putative
PFIT_05125	139,2443402	116,6908696	208,8173055	219,7172558	125,7238989	341,1892148	51,63390423	72,06724153	191,89715	61,850573	0,3223111	-1,633474	0,0331805	0,2233416	conserved Plasmodium protein, unknown function
PFIT_05263	86,03715328	140,1558814	34,28343822	76,92871172	122,9607363	69,98753125	231,7521748	195,3401547	88,392242	213,54616	2,4158926	1,2725563	0,0331827	0,2233416	conserved Plasmodium protein, unknown function, putative
PFIT_12359	406,4123425	622,7740976	362,1577746	318,7837263	700,4617225	577,3971328	1024,272565	1117,042244	497,9978	1070,6574	2,149924	1,1042856	0,0332104	0,2233416	conserved Plasmodium protein, unknown function
PFIT_09070	135,8481368	48,19840267	35,5301087	44,27551754	46,97376443	75,81982552	13,2086718	14,22379767	64,440959	13,716235	0,2128496	-2,232093	0,0333485	0,2233416	conserved Plasmodium protein, unknown function
PFIT_12151	0	2,536758035	0,62333524	3,320663815	0	0	3,602365411	18,96506356	1,0801262	11,283714	10,446663	3,3849703	0,0334468	0,2244068	conserved Plasmodium protein, unknown function
PFIT_13571	328,2996638	193,4278002	225,0240217	238,5343507	520,8561526	157,4719453	643,6226202	590,7617299	277,26899	617,19218	2,225969	1,1544335	0,0335075	0,224548	patatin-like phospholipase, putative
PFIT_10304	0	73,56598302	45,50347254	68,62705218	44,21060181	32,07761849	122,480424	128,9624322	43,997455	125,72143	2,8574705	1,5147386	0,0335885	0,2248915	25 kDa ookinete surface antigen precursor
PFIT_13566	278,4886803	488,3259218	491,8115046	494,2254645	985,0674716	714,4560482	1290,847606	1179,626953	575,39585	1235,2373	2,1467608	1,1021615	0,0336437	0,2249274	DNA helicase, putative
PFIT_11093	272,8283413	191,5252317	114,6936842	131,1662207	563,6851731	291,6147135	577,5792543	462,7475509	260,91889	520,1634	1,9935827	0,9953635	0,0337233	0,225151	succinyl-CoA synthetase alpha subunit, putative
PFIT_14067	698,4858365	855,5216473	753,6123055	1047,669434	694,9353972	565,7325443	1862,422918	1867,110508	769,32619	1864,7667	2,423896	1,2773278	0,0337567	0,225151	conserved protein, unknown function
PFIT_12392	89,4333567	80,54206762	69,19021167	74,16149187	283,2241679	201,2141523	298,9963291	229,4772691	132,96091	264,2368	1,987327	0,9908293	0,0338261	0,2253482	DNA gyrase subunit B
PFIT_09349	176,6025778	194,0619897	97,24029749	201,4536048	366,1190463	183,7172695	469,5082919	428,6104365	203,19913	449,05936	2,2099473	1,1440119	0,0338808	0,2254465	transcription factor with AP2 domain(s), putative
PFIT_14150	54,3392547	166,7918408	170,1705206	206,9880445	408,9480668	204,1302995	489,9216959	418,1796515	201,84967	454,05067	2,2489483	1,1692505	0,0340262	0,226148	26S proteasome regulatory subunit RPN13, putative (RPN13)
PFIT_05085	2317,342799	1513,176168	1222,983741	1520,310583	1483,818323	772,7789909	3567,542546	3276,21473	1471,7351	3421,8786	2,3250642	1,2172706	0,0345848	0,2295505	guanine nucleotide exchange factor
PFIT_08007	46,41478006	36,78299151	59,21684783	75,26837891	40,06585789	34,99376562	129,6851548	132,7554449	48,790437	131,2203	2,6894676	1,4273206	0,0346482	0,2295505	rifin (RIF)
PFIT_10149	45,28271225	99,56775288	76,04689932	79,1424876	288,7504931	90,4005612	278,5829251	236,1150413	113,19848	257,34898	2,2734314	1,1848714	0,0346599	0,2295505	conserved Plasmodium protein, unknown function
PFIT_13513	341,8844775	608,1877389	611,4918707	814,6695227	670,0669337	825,2696393	1706,320417	1287,727816	645,2617	1497,0241	2,3200263	1,2141412	0,0347507	0,2298832	ABC transporter, (heavy metal transporter family), putative
PFIT_13425	379,2427151	402,7103381	564,1183925	655,2776595	1210,265225	1624,293954	1784,371667	1344,623006	805,98471	1564,4973	1,9411005	0,9568748	0,0348767	0,2304472	conserved Plasmodium protein, unknown function
PFIT_12097	62,26372935	67,85827744	29,29675629	41,50829769	121,579155	84,56826693	162,1064435	133,7036981	67,845747	147,90507	2,1800198	1,1243412	0,035067	0,2314342	syntaxin, Qa-SNARE family
PFIT_05026	184,5270524	87,51815221	157,0804806	114,0094577	51,11850835	221,6271823	43,22838494	43,61964619	135,98014	43,424016	0,3193409	-1,646831	0,0352322	0,2321901	conserved Plasmodium protein, unknown function
PFIT_13646	405,2802746	194,6961792	81,03358124	109,5819059	225,197753	78,73597266	427,4806955	381,1977776	182,42094	404,33924	2,2165176	1,1482948	0,0352873	0,2321901	DNA topoisomerase VI, b subunit, putative
PFIT_08170	6,792406838	55,80867677	105,9669908	57,00472883	29,01320744	55,40679557	9,606307764	12,32729131	51,665468	10,9668	0,2122656	-2,236058	0,0353047	0,2321901	conserved Plasmodium protein, unknown function
PFIT_09167	83,77301766	193,4278002	206,3239645	183,7433978	138,1581307	55,40679557	48,03153882	59,73995021	143,47218	53,885745	0,3755832	-1,412795	0,0354715	0,2328802	methyltransferase, putative
PFIT_12049	0	21,5624433	14,96004577	13,83609923	63,55274011	2,916147135	70,84651976	53,10217797	19,471246	61,974349	3,1828651	1,670326	0,035492	0,2328802	conserved Plasmodium protein, unknown function
PFIT_10234	8380,69797	5052,587816	4365,216688	7677,928185	2750,728382	4435,459793	11468,73068	13711,74095	5443,7698	12590,236	2,3127789	1,2096273	0,0355901	0,2332531	chromodomain-helicase-DNA-binding protein 1 homolog, putative
PFIT_09083	10,18861026	12,68379018	13,71337529	15,49643114	33,15795136	0	44,42917341	44,56789937	14,206693	44,498536	3,1322234	1,6471871	0,0356526	0,2333924	conserved Plasmodium membrane protein, unknown function
PFIT_14384	391,695461	1347,018517	1100,186699	1135,113581	3579,677166	1743,855987	3361,006929	3119,752956	1549,5912	3240,3799	2,0911192	1,0642753	0,0357351	0,2334656	ribonucleoside-diphosphate reductase, large subunit, putative
PFIT_14027	544,5246148	903,72005	503,6548742	554,5508571	1536,318413	1166,458854	1868,42686	1663,236074	868,20461	1765,8315	2,0338886	1,0242407	0,0357463	0,2334656	choline kinase
PFIT_02066	33,96203419	73,56598302	59,84018307	120,0973413	37,30269528	14,58073568	174,1143282	158,3582807	56,558162	166,2363	2,9392098	1,5554283	0,0361462	0,2357325	serine repeat antigen 8
PFIT_03120	52,07511909	44,39326561	46,12680778	48,70306929	145,0660372	23,32917708	133,2875202	145,0827362	59,948913	139,18513	2,321729	1,2151996	0,0361768	0,2357325	RING zinc finger protein, putative
PFIT_14396	1187,539129	2078,87321	738,6522597	917,6101009	3647,37465	1848,837284	3708,034797	3298,024553	1736,4811	3503,0297	2,0173152	1,0124365	0,0366334	0,238433	metacaspase-like protein
PFIT_14313	54,3392547	36,78299151	41,7634611	78,03559966	99,47385408	55,40679557	157,3032896	132,7554449	60,966993	145,02937	2,3788178	1,2502448	0,0373141	0,2425848	phosphotyrosyl phosphatase activator, putative
Pf_M76611	0	0	0,62333524	1,106887938	0	5,832294271	7,204730823	8,534278602	1,2604196	7,8695047	6,2435596	2,6423688	0,0373778	0,2427198	unspecified product
PFIT_11479	258,1114598	235,2843078	194,480595	251,817006	797,1724139	320,7761849	768,5046211	630,5883634	342,94033	699,54649	2,039849	1,0284624	0,037695	0,2444989	conserved Plasmodium protein, unknown function
PFIT_12021	16,98101709	29,80690691	29,29675629	30,43941831	59,40799619	32,07761849	75,64967364	87,23929238	33,001619	81,444483	2,4678936	1,3032802	0,0379793	0,2460605	peptidyl-prolyl cis-trans isomerase
PFIT_14496	228,6776969	291,7207174	221,2840103	216,9500359	589,9352179	521,9903372	764,9022557	613,5198062	345,09408	689,21103	1,9971685	0,9979561	0,0383312	0,2479717	ubiquitin-protein ligase, putative
PFIT_13480	234,3380359	147,7661555	357,7944279	253,4773379	227,9609156	160,3880924	551,1619079	545,2455774	230,28749	548,20374	2,3805189	1,2512761	0,0383979	0,2479717	E1-E2 ATPase, putative
PFIT_08036	59,99959373	99,56775288	92,25361556	124,5248931	118,8159924	230,3756237	274,9805597	242,7528136	120,92291	258,86669	2,140758	1,0981217	0,0384219	0,2479717	Plasmodium exported protein (hyp7), unknown function
PFIT_14540	41,88650883	26,63595937	26,18008009	17,15676305	38,68427659	55,40679557	72,04730823	80,60152013	34,325066	76,324414	2,2235768	1,1528822	0,0384987	0,2479717	conserved Plasmodium protein, unknown function
PFIT_05088	86,03715328	104,0070794	76,67023456	103,4940222	375,7901154	195,3818581	377,0475797	280,6829407	156,89674	328,86526	2,0960617	1,0676812	0,0385309	0,2479717	single-stranded DNA-binding protein
PFIT_02068	459,6195294	539,0610825	238,1140618	344,2421488	3397,308433	358,6860977	3122,050023	2474,940795	889,50523	2798,4954	3,1461259	1,6535764	0,0385373	0,2479717	serine repeat antigen 6
PFIT_10127	1518,102928	1036,265657	561,6250515	676,8619743	1286,252196	1134,381236	2294,706767	1968,573598	1035,5815	2131,6402	2,0583992	1,0415228	0,0387236	0,2488874	conserved Plasmodium protein, unknown function
PFIT_13511	1824,893304	807,3232447	1516,57464	1366,45316	696,3169786	1204,368767	493,5240614	459,9027913	1235,9883	476,71343	0,3856941	-1,374471	0,0387854	0,2490017	conserved Plasmodium protein, unknown function
PFIT_03058	3,396203419	4,439326561	2,493340961	2,767219846	15,19739437	2,916147135	22,81498094	16,12030403	5,2016054	19,467642	3,742622	1,9040493	0,0389098	0,2495168	conserved Plasmodium protein, unknown function
PFIT_05042	146,036747	186,4517156	96,61696225	200,9001608	309,4742127	134,1427682	387,854676	410,5936261	178,93709	399,22415	2,2310866	1,1577465	0,0390373	0,2500511	ATP-dependent helicase, putative
PFIT_08034	39,62237322	38,05137053	38,02344966	33,20663815	29,01320744	107,897444	103,2678085	100,5148369	47,635747	101,89132	2,1389677	1,0969147	0,0392309	0,2507654	surface-associated interspersed protein 8.3 (SURFIN 8.3) (SURF8.3)
PFIT_10311	119,9991875	169,9627884	97,24029749	157,7315312	204,4740334	78,73597266	343,4255026	312,9235487	138,02397	328,17453	2,3776633	1,2495444	0,0392375	0,2507654	protein phosphatase inhibitor 3 (I3)
Pf_M76611	0	13,95216919	17,45338673	28,7790864	74,60539056	49,5745013	68,44494282	70,17073517	30,727422	69,307839	2,2555696	1,1734918	0,0393849	0,2508772	unspecified product
PFIT_04094	2389,795139	1944,425034	704,3688215	875,5483593	2028,161358	1703,029927	3597,562257	2990,790523	1607,5548	3294,1764	2,0491845	1,0350499	0,0393873	0,2508772	conserved Plasmodium protein, unknown function
PFIT_05137	542,2604792	251,1390455	319,7709783	314,9096185	721,1854421	384,9314219	952,2252571	795,5844164	422,36616	873,90484	2,0690692	1,0489819	0,0393881	0,2508772	deoxyribodipyrimidine photo-lyase, putative
PFIT_09098	144,9046792	205,4774008	132,7704062	169,3538546	420,0007172	84,56826693	455,0988303	410,5936261	192,84589	432,84623	2,2445188	1,1664062	0,0394744	0,2511441	conserved Plasmodium protein, unknown function
PFIT_05344	14,71688148	64,05314039	55,47683639	57,5581728	269,4083548	40,8260599	234,1537517	210,4255223	83,673241	232,28964	2,776152	1,4730866	0,0398227	0,2530757	phosphoenolpyruvate/phosphate translocator (PPT)
PFIT_13577	294,3376296	316,4605649	119,6803661	141,6816561	327,4347697	102,0651497	511,5358884	462,7475509	216,94336	487,14172	2,2454789	1,1670231	0,04013	0,2547423	zinc finger protein, putative
PFIT_05365	22,64135613	12,04960067	38,02344966	26,56531052	13,81581307	58,32294271	8,405519293	3,793012712	28,569745	6,099266	0,2134869	-2,227781	0,0405861	0,2573485	rifin
PFIT_09254	86,03715328	88,15234172	87,26693364	116,2232335	410,3296481	69,98753125	408,26808	311,9752956	142,99947	360,12169	2,5183428	1,3324747	0,0409214	0,2589963	proline--tRNA ligase, putative
PFIT_13190	3,396203419	13,31797968	8,726693364	7,1947716	35,92111397	8,748441406	36,02365411	36,03362076	12,884201	36,028637	2,7963425	1,4835411	0,0409375	0,2589963	probable protein, unknown function
PFIT_14102	4,528271225	22,19663281	8,726693364	14,94298717	71,84222795	11,66458854	78,05125058	52,15392479	22,3169	65,102588	2,9171877	1,5445782	0,041017	0,2591755	conserved Plasmodium protein, unknown function
PFIT_04176	59,99959373	31,70947544	23,68673913	20,47746286	34,53953267	32,07761849	75,64967364	83,44627967	33,748398	79,547977	2,357089	1,2370062	0,0413556	0,2610573	conserved Plasmodium protein, unknown function
PFIT_13565	401,8840712	337,3888187	192,6105893	208,0949324	377,1716967	323,692332	718,0715053	581,2791981	306,80707	649,67535	2,1175371	1,0823872	0,0415506	0,2619959	conserved Plasmodium protein, unknown function
PFIT_09280	1112,822654	1607,670405	1479,79786	1202,080301	2370,793522	1828,424254	3465,475526	3246,818882	1600,2648	3356,1472	2,0972449	1,0684953	0,0416737	0,2624792	phosphatidylserine decarboxylase
PFIT_14528	24,90549174	51,36935021	50,49015446	34,86697006	27,63162613	34,99376562	120,078847	82,49802649	37,376226	101,28844	2,7099696	1,4382767	0,041825	0,26313	LCCL-like protein
PFIT_11509	26,03755954	50,7351607	72,93022311	61,43228058	67,69748403	67,07138411	129,6851548	146,0309894	57,650682	137,85807	2,3912652	1,2577742	0,041902	0,2632988	A_ITvar8 - erythrocyte membrane protein 1, PfEMP1, putative
PFIT_13462	19,24515271	18,39149575	4,986681922	11,06887938	34,53953267	14,58073568	46,83075035	41,72313983	17,135413	44,276945	2,5839439	1,3695747	0,0419435	0,2632988	G-beta repeat protein, putative
PFIT_05128	202,6401373	296,1665006	340,9643764	248,4963422	268,0267735	419,9251875	92,46071223	119,4799004	296,03655	105,97031	0,3579636	-1,482115	0,0422685	0,2650452	orotate phosphoribosyltransferase (OPRT)
PFIT_05141	22,64135613	34,88042298	10,59669908	26,01186655	62,1711588	8,748441406	75,64967364	66,37772246	27,508324	71,013698	2,5815349	1,3682291	0,0423512	0,2652695	tubulin-tyrosine ligase, putative
PFIT_08103	22,64135613	35,51461249	21,19339817	35,42041403	85,65804101	14,58073568	104,4685969	74,91200106	35,83476	89,690299	2,5028855	1,3235923	0,0424615	0,2656663	conserved Plasmodium protein, unknown function
PFIT_06230	35,09410199	31,07528593	29,29675629	54,79095295	147,8291998	40,8260599	153,7009242	109,0491155	56,485393	131,37502	2,3258229	1,2177412	0,042505	0,265679	conserved Plasmodium protein, unknown function
PFIT_14103	88,30128889	263,1886461	60,46351831	109,0284619	766,7776252	107,897444	697,6581013	611,6232998	232,6095	654,6407	2,8143335	1,4927933	0,0426287	0,2661235	DNA damage-inducible protein 1, putative (DDI1)
PFIT_01133	3,396203419	15,22054821	13,71337529	28,7790864	13,81581307	34,99376562	1,20078847	4,74126589	18,319799	2,9710272	0,1621758	-2,62437	0,0429147	0,2676136	Plasmodium exported protein (hyp10), unknown function
PFIT_06253	322,6393248	304,4109642	177,0272082	206,9880445	472,5008069	259,5370951	654,4297164	566,1071473	290,51724	610,26843	2,1006272	1,0708202	0,0429653	0,2676344	Sec14 domain containing protein
PFIT_12118	318,1110536	171,2311674	142,74377	156,0711993	102,2370167	93,31670833	373,4452143	378,353018	163,95182	375,89912	2,2927414	1,1970736	0,0437363	0,2721372	conserved Plasmodium protein, unknown function
PFIT_13478	216,224951	507,351607	293,5908982	290,0046399	646,5800515	656,1331055	946,2213147	813,6012267	434,98088	879,91127	2,0228735	1,0164061	0,043884	0,2727561	conserved Plasmodium protein, unknown function
PFIT_03030	735,8440741	738,8307777	385,2211785	380,2160068	918,7515689	664,8815469	1392,914626	1215,660574	637,29086	1304,2876	2,0466128	1,0332382	0,0440919	0,2737472	conserved Plasmodium protein, unknown function
PFIT_08336	453,9591903	763,5641686	1184,336957	1176,621879	723,9486047	1627,210102	337,4215602	412,4901324	988,27348	374,95585	0,3794049	-1,39819	0,044463	0,2757486	Plasmodium exported protein (hyp9), unknown function
PF_apicopla	398,4878678	455,9822568	284,8642048	383,5366707	647,9616328	230,3756237	882,5795258	873,341177	400,20138	877,96035	2,1937964	1,1334297	0,0446126	0,2761769	molecular chaperone
PFIT_05001	106,4143738	83,71301516	67,32020595	79,69593156	120,1975737	69,98753125	165,7088089	220,9429905	87,888105	193,3259	2,1996822	1,1372951	0,0446297	0,2761769	B_ITvar11_C1 - erythrocyte membrane protein 1,, putative
PFIT_12073	6,792406838	15,22054821	31,16676201	55,89784089	30,39478875	23,32917708	87,65755834	67,32597564	27,133587	77,491767	2,8559352	1,5139632	0,0446874	0,2762314	blood stage antigen 41-3 precursor
PFIT_09257	33,96203419	45,66164463	28,67342105	37,08074594	66,31590272	14,58073568	97,26386611	92,92881145	37,712414	95,096339	2,521619	1,3343503	0,0448162	0,2762756	zinc binding protein (Yippee), putative
PFIT_10292	91,69749231	83,07882565	41,14012586	50,3634012	179,6055699	102,0651497	176,5159052	188,7023824	91,325094	182,60914	1,9995506	0,9996758	0,0449595	0,2773076	RAP protein, putative
PFIT_05354	1,132067806	7,610274105	11,22003433	6,64132763	31,77637005	5,832294271	37,22444258	26,55108898	10,702061	31,88766	2,979591	1,5751143	0,0454805	0,2802154	mitochondrial ribosomal protein S16 precursor, putative
PFIT_14502	14,71688148	43,1248866	31,79009725	45,38240547	67,69748403	23,32917708	100,8662315	91,03230509	37,673489	95,949268	2,5468644	1,3487222	0,0455929	0,2806024	gamete egress and sporozoite traversal protein, putative (GEST)
PFIT_05279	578,486649	254,9441825	549,7816819	531,8596544	164,4081755	294,5308607	146,4961934	172,5820784	395,66853	159,53914	0,4032141	-1,310382	0,0461232	0,2835573	conserved Plasmodium membrane protein, unknown function
PFIT_14369	67,92406838	67,22408793	74,80022884	80,8028195	131,2502241	221,6271823	240,1576941	179,2198506	107,27144	209,68877	1,9547494	0,9669837	0,0466979	0,2867788	ataxin-2 like protein, putative
PFIT_12036	3304,505927	3021,913009	3673,314571	4217,243045	2155,266838	3820,152747	7703,058038	7201,982887	3365,3994	7452,5205	2,2144535	1,1469507	0,0469087	0,287761	ubiquitin conjugating enzyme E2 (UBC)
PFIT_05304	98,48989915	99,56775288	45,50347254	90,21136698	244,5398913	93,31670833	253,3663673	211,4604587	111,93818	232,41341	2,0762657	1,0539911	0,0471758	0,289086	ADP-ribosylation factor GTPase-activating protein, putative
PFIT_12442	182,2629168	128,1062808	138,3804233	222,4844756	276,3162613	294,5308607	455,0988303	390,6803093	207,01354	422,88957	2,0428112	1,0305559	0,0473937	0,2898653	glucose inhibited division protein a homologue, putative
PFIT_07030	3168,65779	2397,870533	4368,956699	5356,784178	5207,179945	5354,046141	10500,89517	7861,018866	4308,9159	9180,957	2,1306884	1,0913196	0,0474055	0,2898653	conserved Plasmodium membrane protein, unknown function
PFIT_10153	99,62196695	127,4720913	109,7070023	104,0474662	596,8431245	145,8073568	504,3311576	455,1615254	197,24983	479,74634	2,4321761	1,2822477	0,0475113	0,2901988	ribonucleotide reductase small subunit, putative
PFIT_06148	119,9991875	87,51815221	89,7602746	92,42514286	285,9873305	125,3943268	277,3821367	258,8731176	133,51407	268,12763	2,008235	1,0059281	0,0477526	0,2912543	conserved Plasmodium protein, unknown function
PFIT_14416	223,0173578	199,7696953	61,71018879	87,44414713	453,1586686	46,65835417	447,8940995	381,1977776	178,6264	414,54594	2,3207428	1,2145867	0,0477871	0,2912543	conserved Plasmodium protein, unknown function
PFIT_06042	11,32067806	34,88042298	26,80341533	24,90497861	118,8159924	20,41302995	106,8701739	94,8253178	39,523086	100,84775	2,5516162	1,3514113	0,0479686	0,291738	serine/threonine protein kinase
PFIT_bin05	9,05654245	74,83436204	127,7837243	104,6009102	67,69748403	151,639651	26,41734635	25,60283581	89,268779	26,010091	0,2913683	-1,779084	0,0479697	0,291738	rifin (RIF)
PFIT_02185	126,7915943	124,9353332	168,9238501	145,5557639	82,8948784	262,4532422	54,03548117	53,10217797	151,92578	53,56883	0,3525987	-1,503901	0,0482163	0,2929228	conserved protein, unknown function
PFIT_13171	2766,773719	1976,134509	2889,782174	2880,122416	1776,71356	2295,007796	930,6110646	1047,819762	2430,7557	989,21541	0,406958	-1,297048	0,0483089	0,2931705	small ribosomal subunit nuclear export protein, putative
PFIT_04191	0	12,04960067	4,986681922	9,961991446	35,92111397	8,748441406	38,42523105	28,44759534	11,944638	33,436413	2,7992822	1,4850569	0,0486043	0,294647	conserved Plasmodium protein, unknown function
PFIT_06138	242,2625105	173,1337359	121,5503719	135,5937725	313,6189566	463,6673945	475,5122343	403,0076007	241,63779	439,25992	1,8178445	0,8622288	0,0491865	0,2978572	endonuclease III homologue, putative
PFIT_09320	49,81098348	140,1558814	82,9035896	87,9975911	222,4345904	110,8135911	276,1813482	223,78775	115,68604	249,98455	2,1608878	1,1116242	0,0492767	0,2980843	conserved Plasmodium protein, unknown function
PFIT_09086	3,396203419	17,12311674	8,103358124	7,748215569	60,78957749	2,916147135	49,23232729	52,15392479	16,679436	50,693126	3,0392589	1,6037196	0,0495197	0,2984026	conserved Plasmodium protein, unknown function
PFIT_14298	44,15064444	63,41895088	57,34684211	90,21136698	196,1845455	99,1490026	208,9371939	175,4268379	91,743559	192,18202	2,0947739	1,0667946	0,0495205	0,2984026	peptide chain release factor 1, putative
PFIT_10045	1,132067806	24,73339084	24,31007437	38,18763387	99,47385408	40,8260599	91,25992375	83,44627967	38,110513	87,353102	2,2920998	1,1966599	0,0495365	0,2984026	conserved Plasmodium protein, unknown function
PFIT_13319	355,4692912	244,7971504	226,8940275	255,6911138	816,5145522	338,2730677	833,3471985	628,691857	372,93987	731,01953	1,9601539	0,9709669	0,0495725	0,2984026	apurinic/apyrimidinic endonuclease Apn1, putative
PFIT_10145	22,64135613	59,61381383	43,01013158	53,68406501	107,7634163	55,40679557	153,7009242	107,1526091	56,812139	130,42677	2,2957553	1,1989688	0,0495931	0,2984026	FAD synthetase, putative
PFIT_13488	75,84854302	91,32328926	63,58019451	73,6080479	212,7635212	317,8600378	267,7758289	248,6423326	139,16394	258,20908	1,8547124	0,8911955	0,0497496	0,2990264	conserved Plasmodium protein, unknown function
PFIT_12312	873,9563464	1523,95739	786,025738	1371,9876	770,9223691	825,2696393	2603,309404	2151,586461	1025,3532	2377,4479	2,3186625	1,2132928	0,049504	0,2999146	amino acid transporter, putative
PFIT_08245	5056,946891	2236,786397	2922,818942	2825,331463	1917,634854	2919,063283	1152,756932	1204,281536	2979,7636	1178,5192	0,3955076	-1,338223	0,0501341	0,300698	clp1-related protein, putative
PFIT_13604	41,88650883	41,85650758	8,726693364	18,26365098	29,01320744	5,832294271	69,64573129	58,79169704	24,263144	64,218714	2,6467598	1,4042273	0,0503193	0,3014892	actin-depolymerizing factor 2
PFIT_14389	52,07511909	67,22408793	26,18008009	21,03087083	161,6450129	11,66458854	164,5080205	124,2211663	56,636627	144,36459	2,5489617	1,3499097	0,050674	0,3023498	succinyl CoA ligase, putative
PFIT_10196	11,32067806	30,44109642	3,740011442	9,961991446	15,19739437	0	31,22050023	43,61964619	11,776862	37,420073	3,1774231	1,6678572	0,0507464	0,3023498	flagellar outer arm dynein-associated protein, putative
PFIT_13458	2,264135613	23,46501182	12,46670481	18,81709495	67,69748403	8,748441406	63,64178893	55,94693375	22,243145	59,794363	2,6882153	1,4266487	0,0507675	0,3023498	conserved Plasmodium protein, unknown function
PFIT_14438	130,1877977	162,9867038	60,46351831	121,7576732	40,06585789	52,49064844	213,7403477	245,5975731	94,6587	229,66896	2,4262848	1,2787489	0,0507912	0,3023498	TRAP-like protein,sporozoite-specific transmembrane protein S6 (TREP)
PFIT_07246	457760,674	152643,0729	126820,048	75051,42977	33373,47804	130474,2551	500715,5835	337704,249	162687,16	419209,92	2,5767855	1,3655725	0,0508153	0,3023498	28S ribosomal RNA
PFIT_08079	118,8671197	196,5987477	62,95685927	100,1733584	147,8291998	78,73597266	282,1852906	251,2870922	117,52688	266,73619	2,2695761	1,1824229	0,050856	0,3023498	conserved Plasmodium protein, unknown function
PFIT_bin10	93,96162792	76,10274105	117,8103604	73,6080479	75,98697187	102,0651497	186,1222129	210,5122055	89,922483	198,31721	2,2054241	1,1410561	0,050885	0,3023498	erythrocyte membrane protein 1, PfEMP1
PFIT_14412	101,8861026	223,2347071	113,4470137	156,0711993	672,8300963	96,23285547	612,4021199	531,0217797	227,28366	571,71195	2,5154116	1,3307945	0,050901	0,3023498	conserved Plasmodium protein, unknown function
PFIT_03179	129,0557299	173,1337359	187,0005721	137,8075483	186,5134764	166,2203867	317,0081562	386,8872966	163,28857	351,94773	2,1553726	1,1079373	0,050944	0,3023498	conserved Plasmodium protein, unknown function
PFIT_07245	458788,5916	153006,4635	127293,7828	75037,59368	33390,05702	130803,7798	501308,773	337804,7639	163053,38	419556,77	2,5731253	1,3635217	0,0513483	0,3044297	28S ribosomal RNA
PFIT_12301	45,28271225	116,0566801	87,26693364	103,4940222	288,7504931	163,3042396	285,787656	251,2870922	134,02585	268,53737	2,0036238	1,0026116	0,0515028	0,3050261	heat shock protein hslv
PFIT_14544	189,0553236	209,2825379	94,12362128	123,9714491	484,9350386	104,9812969	481,5161767	370,7669926	201,05821	426,14158	2,1194936	1,0837196	0,0517949	0,3064352	conserved Plasmodium protein, unknown function
PFIT_12259	132,4519333	131,9114178	296,7075744	261,2255535	46,97376443	119,5620326	62,44100046	49,30916526	164,80538	55,875083	0,3390368	-1,560486	0,052003	0,3072939	conserved Plasmodium protein, unknown function
PFIT_11221	100,7540348	242,2603924	89,7602746	135,0403285	744,6723243	78,73597266	712,067563	554,7281091	231,87055	633,39784	2,7316872	1,4497923	0,0520487	0,3072939	protein kinase, putative
PFIT_12293	576,2225134	280,9459524	181,3905549	209,7552643	389,6059285	207,0464466	660,4336588	592,6582363	307,49444	626,54595	2,0375846	1,02686	0,0521849	0,3077558	conserved Plasmodium protein, unknown function
PFIT_14786	2335,455884	1207,496825	1500,991259	1424,011333	1428,555071	7264,122514	118,8780586	186,8058761	2526,7721	152,84197	0,060489	-4,047183	0,0522358	0,3077558	Plasmodium exported protein (PHISTb), unknown function
PFIT_13599	46,41478006	145,863587	94,74695652	106,8146861	536,053547	172,052681	408,26808	449,4720064	183,65771	428,87004	2,3351595	1,2235211	0,0525775	0,3093191	polyadenylate-binding protein, putative
PFIT_12267	2,264135613	7,610274105	3,740011442	3,320663815	30,39478875	5,832294271	22,81498094	25,60283581	8,8603613	24,208908	2,732271	1,4501006	0,0526105	0,3093191	conserved Plasmodium protein, unknown function
PFIT_10322	20,37722051	31,07528593	21,19339817	28,7790864	98,09227277	32,07761849	96,06307764	73,96374789	38,599147	85,013413	2,2024687	1,1391215	0,05267	0,3093473	leucine-rich repeat protein
PFIT_09195	90,5654245	180,1098205	154,5871396	164,3728589	809,6066457	113,7297383	702,4612552	623,002338	252,16194	662,7318	2,6281992	1,3940746	0,0530937	0,3115122	protein disulfide isomerase
PFIT_11251	191,3194593	218,7953805	241,8540732	250,1566741	247,3030539	291,6147135	567,9729465	463,695804	240,17389	515,83438	2,1477537	1,1028286	0,0532554	0,3121374	conserved Plasmodium protein, unknown function
PFIT_02146	157,3574251	50,10097119	150,8471282	239,6412387	60,78957749	218,7110352	46,83075035	49,30916526	146,24123	48,069958	0,3287032	-1,605143	0,053959	0,3159338	conserved Plasmodium protein, unknown function
PFIT_12176	146,036747	301,8742062	177,6505435	215,289704	471,1192256	501,5773073	608,7997545	540,5043115	302,25796	574,65203	1,9011974	0,9269083	0,0543962	0,3181646	conserved protein, unknown function
PFIT_13073	6,792406838	29,80690691	22,44006865	22,13775877	40,06585789	2,916147135	50,43311576	63,53296293	20,693191	56,983039	2,7537096	1,4613764	0,0545724	0,3188649	CCAAT-binding transcription factor, putative
PFIT_05157	41,88650883	133,1797968	79,78691076	92,42514286	283,2241679	61,23908984	236,5553287	263,6143835	115,29027	250,08486	2,1691757	1,1171469	0,0547715	0,319698	conserved Plasmodium protein, unknown function
PFIT_14643	55,47132251	70,39503547	105,3436556	116,2232335	256,974123	67,07138411	267,7758289	216,2017246	111,91313	241,98878	2,1622913	1,1125609	0,05492	0,3202343	replication factor C3
PFIT_08118	22,64135613	5,07351607	16,20671625	14,3895432	44,21060181	14,58073568	4,803153882	1,896506356	19,517078	3,3498301	0,1716358	-2,542577	0,0551741	0,321385	unspecified product
PFIT_06272	9036,16523	12929,85571	8369,522271	14491,37689	9448,634556	2977,386225	21543,34595	22706,8706	9542,1568	22125,108	2,3186695	1,2132972	0,0553932	0,322329	choline/ethanolaminephosphotransferase, putative
PFIT_05113	280,752816	277,1408153	232,5040446	277,2754286	1199,212574	417,0090404	985,8473342	903,6852786	447,31579	944,76631	2,1120791	1,0786638	0,055628	0,3233625	stearoyl-CoA desaturase (SCD)
PFIT_03039	49,81098348	67,85827744	23,68673913	36,52730197	33,15795136	5,832294271	99,66544305	89,13579873	36,145591	94,400621	2,6116773	1,3849767	0,0562819	0,3259041	inner membrane complex protein 1a, putative
PFIT_07078	234,3380359	314,5579964	493,0581751	380,2160068	338,4874201	591,9778685	134,4883087	168,7890657	392,10592	151,63869	0,3867289	-1,370606	0,0562954	0,3259041	Cg3 protein
PFIT_14162	599,9959373	290,458795	248,0874256	219,7172558	255,5925417	189,5495638	103,2678085	112,8421282	300,56692	108,05497	0,3595039	-1,475921	0,0562989	0,3259041	DNA-directed RNA polymerase I/III subunit, putative
PFIT_05371	41,88650883	63,41895088	89,13693936	73,05460393	16,57897568	119,5620326	21,61419247	22,75807627	67,273002	22,186134	0,3297925	-1,600369	0,0563148	0,3259041	rifin, pseudogene
PFIT_12095	70,18820399	69,12665646	99,73363845	102,3871343	244,5398913	183,7172695	294,1931753	207,667446	128,28213	250,93031	1,9560815	0,9679665	0,0563607	0,3259041	ABC transporter, (TAP family), putative
PFIT_08021	168,6781031	756,588084	462,5147483	561,1921848	447,6323434	314,9438906	192,1261553	202,9261801	451,92489	197,52617	0,4370774	-1,194039	0,0564109	0,3259041	Plasmodium exported protein, unknown function
PFIT_08252	228,6776969	313,9238068	351,5610755	277,2754286	421,3822985	358,6860977	111,6733278	139,3932172	325,25107	125,53327	0,3859581	-1,373484	0,0566601	0,3270099	small nuclear ribonucleoprotein G, putative
PFIT_14312	328,2996638	286,0194685	340,3410412	326,5319418	295,6583996	247,8725065	692,8549475	634,3813761	304,1205	663,61816	2,1820895	1,1257103	0,0570655	0,3288454	conserved Plasmodium protein, unknown function
PFIT_13068	409,8085459	500,3755224	610,2452002	1099,693167	780,5934383	1358,924565	1658,288878	1513,412072	793,27341	1585,8505	1,9991222	0,9993667	0,0570944	0,3288454	DNA-directed RNA polymerase alpha chain, putative
PFIT_07328	59,99959373	39,31974954	14,33671053	17,71020701	78,75013448	20,41302995	96,06307764	71,11898835	38,421571	83,591033	2,1756277	1,1214317	0,0572861	0,3296137	acyl-CoA synthetase
PFIT_13642	11,32067806	21,5624433	18,70005721	15,49643114	9,671069147	8,748441406	2,401576941	2,844759534	14,249853	2,6231682	0,1840839	-2,441565	0,0575402	0,3307394	conserved Plasmodium protein, unknown function
PFIT_02186	1421,877165	1576,595119	1129,483455	1006,161136	2091,714098	2222,104117	535,5516578	643,8639079	1574,6558	589,70778	0,3744995	-1,416964	0,0576459	0,3310103	40S ribosomal protein S30, putative
PFIT_13182	178,8667134	87,51815221	64,20352975	84,12348332	117,4344111	55,40679557	198,1300976	216,2017246	97,925514	207,16591	2,1155458	1,0810299	0,0577817	0,3312595	conserved Plasmodium protein, unknown function
PFIT_06156	206,0363407	164,2550828	132,1470709	157,1780873	163,0265942	67,07138411	356,6341757	311,0270424	148,28576	333,83061	2,2512655	1,1707362	0,0578064	0,3312595	conserved Plasmodium protein, unknown function
PFIT_08063	1578,102522	2550,076015	1340,794102	2026,158371	1423,028746	1073,142146	3760,869489	3676,377571	1665,217	3718,6235	2,2331165	1,1590585	0,0582399	0,3334056	AAA family ATPase, putative
PFIT_09303	289,8093584	246,6997189	223,7773513	465,4463781	541,5798722	341,1892148	796,1227559	659,9842119	351,41698	728,05348	2,0717652	1,0508605	0,0585796	0,3348012	conserved Plasmodium protein, unknown function
PFIT_04106	65,65993276	129,3746598	82,28025172	100,7268024	418,6191359	201,2141523	410,6696569	288,2689661	166,31249	349,46931	2,1012812	1,0712693	0,058602	0,3348012	conserved Apicomplexan protein, unknown function
PFIT_10341	5,660339031	52,63772923	29,92009153	49,80995723	276,3162613	58,32294271	228,1498094	202,9261801	78,777887	215,53799	2,7360215	1,4520796	0,0587904	0,3355385	ADP-ribosylation factor, putative
PFIT_11497	525,2794621	722,3418505	1210,517037	1421,797557	493,2245265	542,4033672	1876,832379	1967,625344	819,26063	1922,2289	2,3462971	1,2303857	0,0592917	0,337724	ring-infected erythrocyte surface antigen 2, pseudogene
PFIT_11046	1072,068213	953,8210212	526,0949428	1029,959227	1295,923266	696,9591654	1976,497822	1850,04195	929,13764	1913,2699	2,0591889	1,0420762	0,0592928	0,337724	CCR4-NOT transcription complex subunit 1, putative
PFIT_07227	153,9612217	133,8139864	185,1305664	245,7291223	116,0528298	122,4781797	64,8425774	67,32597564	159,52765	66,084277	0,4142497	-1,271428	0,0597912	0,3399811	conserved Plasmodium protein, unknown function
PFIT_05072	850,1829225	763,5641686	1123,873438	1019,443791	1161,909879	2120,038967	2219,057093	2184,775322	1173,1689	2201,9162	1,8768962	0,9083489	0,0598751	0,3399811	subtilisin-like protease 3 (SUB3)
PFIT_10271	52,07511909	50,7351607	33,03676774	48,70306929	232,1056595	67,07138411	194,5277322	165,9443062	80,621193	180,23602	2,2355911	1,1606563	0,0599205	0,3399811	centrin-3
PFIT_14688	13,58481368	21,5624433	15,58338101	16,04987511	13,81581307	17,49688281	0	5,689519068	16,348868	2,8447595	0,1740035	-2,522812	0,0599295	0,3399811	conserved Plasmodium protein, unknown function

PFIT_14442	563,7697675	277,7750048	571,5984153	504,7408999	578,8825675	224,5433294	1029,075719	931,1846208	453,55166	980,13017	2,1610111	1,1117065	0,0599918	0,3399897	serine/threonine protein kinase
PFIT_13615	116,602984	840,3010991	500,538198	530,1993225	125,7238989	43,74220703	936,615007	1073,422598	359,51795	1005,0188	2,7954621	1,4830868	0,0601039	0,3399897	exonuclease, putative
PFIT_10208	31,69789858	34,24623347	19,32339245	28,7790864	154,7371063	26,24532422	133,2875202	109,9973686	49,171507	121,64244	2,4738401	1,3067522	0,0601929	0,3399897	conserved Plasmodium membrane protein, unknown function
PFIT_09278	64,52786496	89,42072074	33,66010298	28,7790864	185,1318951	32,07761849	152,5001357	160,2547871	72,266215	156,37746	2,1639083	1,1136394	0,0602485	0,3399897	serine/threonine protein phosphatase, putative
PFIT_07138	2,264135613	3,170947544	7,480022884	5,534439692	22,10530091	11,66458854	31,22050023	17,0685572	8,7032392	24,144529	2,7742003	1,472072	0,0602985	0,3399897	conserved Plasmodium protein, unknown function
PFIT_09030	155,0932895	236,5526868	228,7640332	291,1115278	710,1327916	268,2855365	720,4730823	539,5560583	314,98998	630,01457	2,0001099	1,0000793	0,0603308	0,3399897	serine repeat antigen 9
PFIT_10387	157,3574251	36,78299151	79,16357552	68,07360821	33,15795136	78,73597266	16,81103859	23,70632945	75,545254	20,258684	0,2681662	-1,898801	0,0603518	0,3399897	Plasmodium exported protein (hyp5), unknown function
PFIT_13306	19,24515271	40,58812856	38,6467849	70,28738409	305,3294688	64,15523698	249,7640019	224,7360032	89,708693	237,25	2,6446713	1,4030884	0,0604221	0,3400374	signal peptidase 21 kDa subunit
PFIT_05363	14,71688148	38,05137053	122,1737071	116,2232335	100,8554354	399,5121575	6,003842352	4,74126589	131,92213	5,3726041	0,0407256	-4,617921	0,060631	0,3408838	Plasmodium exported protein, unknown function
PFIT_13620	3003,37589	1263,939691	1421,827683	1391,911583	1228,225782	1542,641835	658,0320818	671,36325	1641,9871	664,69767	0,404813	-1,304673	0,061136	0,343315	conserved Plasmodium protein, unknown function
PFIT_04232	18,1130849	10,14703214	5,610017163	12,17576732	4,14474392	5,832294271	27,61813482	30,3441017	9,3371566	28,981118	3,1038484	1,6340581	0,0613801	0,344106	Plasmodium exported protein, unknown function, pseudogene
PFIT_14787	36,2261698	31,70947544	25,55674485	23,24464671	8,28948784	81,65211979	66,04336588	73,96374789	34,446441	70,003557	2,0322435	1,0230733	0,0618784	0,3465902	surface-associated interspersed protein 14.1 (SURFIN 14.1), pseudogene (SURF14.1)
PFIT_10212	26671,51752	11895,49262	6157,928839	8364,75215	6847,116956	14609,89715	25264,58942	21959,6471	12424,451	23612,118	1,9004557	0,9263454	0,0618911	0,3465902	conserved Plasmodium membrane protein, unknown function
PFIT_09281	0	63,41895088	20,57006293	23,24464671	73,22380925	46,65835417	79,25203905	86,2910392	37,867537	82,771539	2,1866783	1,128741	0,0620355	0,347055	cytochrome c oxidase subunit 6B, putative (COX6B)
PFIT_13149	683,768955	812,3967607	806,5958009	580,5627237	623,0931693	807,7727565	288,1892329	306,2857765	719,03169	297,2375	0,4133858	-1,274439	0,0622738	0,3480443	1-methyladenosine tRNA methyltransferase subunit, putative
PFIT_10338	5,660339031	104,6412689	88,51360412	84,67692729	364,737465	163,3042396	303,799483	290,1654725	135,25564	296,98248	2,1957123	1,1346891	0,062774	0,3504936	flavoprotein subunit of succinate dehydrogenase
PFIT_10041	6,792406838	51,36935021	34,90677346	35,973858	200,3292895	64,15523698	175,3151167	142,2379767	65,587819	158,77655	2,4208237	1,275498	0,0631834	0,352431	conserved Plasmodium protein, unknown function
PFIT_09288	140,376408	61,51638235	91,63028032	73,6080479	71,84222795	93,31670833	37,22444258	27,49934216	88,715009	32,361892	0,3647849	-1,454882	0,0643196	0,3584153	conserved Plasmodium protein, unknown function
PFIT_05060	142,6405436	234,6501182	329,7443421	266,2065492	255,5925417	317,8600378	94,86228917	131,7903816	257,78236	104,32634	0,404707	-1,30505	0,0644482	0,3587787	conserved Plasmodium protein, unknown function
PFIT_13370	1809,044354	3225,487842	2371,167254	5677,228236	3181,781749	804,8566094	6840,891916	6434,846066	2844,9277	6637,869	2,3332294	1,2223282	0,0648181	0,360115	calcium-dependent protein kinase 5
PFIT_13013	3107,526128	4869,941238	3973,762157	5313,062104	10131,13572	5905,197949	9775,618938	11347,74578	5550,1042	10561,682	1,9029701	0,9282529	0,0648261	0,360115	gamete antigen 27/25
PFIT_03034	533,2039368	312,0212383	218,1673341	262,3324414	554,014106	128,310474	704,8628322	644,812161	334,67492	674,8375	2,016397	1,0117797	0,0649279	0,360115	spindle pole body protein, putative
PFIT_02022	1,132067806	30,44109642	9,350028604	13,28265526	35,92111397	14,58073568	46,83075035	42,67139301	17,451283	44,751072	2,5643428	1,3585891	0,0649429	0,360115	zinc finger protein, putative
PFIT_12279	53,2071869	114,7883011	69,19021167	100,1733584	114,6712485	69,98753125	210,1379823	180,1681038	87,002973	195,15304	2,2430618	1,1654694	0,0650635	0,3602031	conserved Plasmodium protein, unknown function
PFIT_12431	14,71688148	43,75907611	40,51679062	38,74107794	53,88167096	96,22285547	106,8701739	89,13579873	47,974725	98,002986	2,0428045	1,0305532	0,0651403	0,3602031	mitochondrial import inner membrane translocase subunit TIM13, putative (TIM13)
PFIT_01029	230,9418325	247,3339084	216,2973284	265,6531052	323,2900258	239,1240651	555,9650618	494,9881589	253,77338	525,47661	2,070653	1,0500858	0,0651499	0,3602031	4-hydroxy-3-methylbut-2-enyl diphosphate reductase
PFIT_11367	485,6570889	223,2347071	142,74377	158,2849752	482,171876	157,4719453	560,7682157	489,2986399	274,92739	525,03343	1,9097167	0,9333586	0,065292	0,3606364	conserved Plasmodium protein, unknown function
PFIT_05205	388,2992576	169,3285988	183,2605606	130,0593328	211,3819399	195,3818581	70,84651976	89,13579873	212,95192	79,991159	0,375636	-1,412615	0,0654848	0,3613484	U6 snRNA-associated sm-like protein lsm2, putative
PFIT_06003	95,09369573	42,49069709	33,03676774	33,76008212	67,69748403	116,6458854	122,480424	114,7386345	64,787435	118,60953	1,830749	0,872434	0,0656446	0,3618769	Plasmodium exported protein (PHISTb), unknown function
PFIT_11486	226,4135613	173,7679254	166,4305092	175,4417382	316,3821192	157,4719453	446,693311	376,4565117	202,6513	411,57491	2,0309513	1,0221556	0,0657792	0,3622658	serine/threonine protein kinase, putative
PFIT_14231	254,7152564	249,236477	195,7272655	198,6863849	449,0139247	244,9563594	531,9492924	503,5224375	265,38928	517,73586	1,9508545	0,9641062	0,0661347	0,3638695	mitochondrial ribosomal protein L21 precursor, putative
PFIT_07270	3829,785389	1071,14608	1582,02484	2052,723682	1192,304668	571,5648385	3793,290778	3447,848555	1716,5916	3620,5697	2,109162	1,0766699	0,0664332	0,364761	conserved Plasmodium membrane protein, unknown function
PFIT_14639	103,0181704	144,595208	71,06021739	73,05460393	504,2771769	102,0651497	426,279907	355,5949418	166,34509	390,93742	2,3501591	1,2327584	0,0664801	0,364761	cytochrome c1 precursor, putative
PFIT_09021	16,98101709	120,4960067	145,8604462	119,5438973	110,5265045	84,56826693	37,22444258	43,61964619	99,66269	40,422044	0,4055885	-1,301911	0,0664902	0,364761	probable protein, unknown function
PFIT_10053	87,16922108	97,66518435	43,01013158	59,77194867	134,0133867	49,5745013	180,1182706	151,7205085	78,534062	165,91939	2,1127061	1,0790921	0,066661	0,3653435	DnaJ protein, putative
PFIT_11177	84,90508547	94,49423681	18,70005721	29,33253037	117,4344111	29,16147135	142,893828	118,5316473	62,337965	130,71274	2,09684	1,0682168	0,0668676	0,3657695	conserved Plasmodium protein, unknown function
PFIT_14337	1133,199874	633,5553193	624,5819108	527,9855466	621,711588	857,3472578	276,1813482	307,2340297	733,06358	291,70769	0,3979296	-1,329415	0,0669968	0,3657695	RNA methyltransferase, putative
PFIT_10286	352,0730878	679,8511534	820,3091762	666,3465389	580,2641488	521,9903372	258,1695211	275,9416748	603,47241	267,0556	0,4425316	-1,176148	0,0670235	0,3657695	conserved Plasmodium protein, unknown function
PFIT_14275	43,01857664	26,00176986	14,96004577	14,94298717	22,10530091	11,66458854	54,03548117	53,10217797	22,115545	53,56883	2,4222252	1,276333	0,0670895	0,3657695	ATP-binding cassette sub-family G member 2 (ABCG2)
PFIT_13156	46,41478006	52,00353972	91,63028032	87,9975911	53,88167096	64,15523698	20,413404	28,44759534	66,01385	24,4305	0,3700814	-1,434085	0,0670954	0,3657695	conserved Plasmodium protein, unknown function
PFIT_06255	106,4143738	150,9371031	244,3474142	146,1092079	157,500269	113,7297383	48,03153882	76,80850742	153,17302	62,420023	0,4075132	-1,295081	0,0671267	0,3657695	conserved Plasmodium protein, unknown function
PFIT_14369	65,65993276	70,39503547	76,67023456	95,1923627	138,1581307	256,6209479	247,3624249	178,2715975	117,11611	212,81701	1,8171455	0,861674	0,0673263	0,3664124	ataxin-2 like protein, putative
PFIT_14770	31,69789858	19,02568526	15,58338101	9,961991446	31,77637005	29,16147135	40,826808	55,9469375	22,8678	48,386873	2,1159392	1,0812981	0,0673743	0,3664124	conserved Plasmodium protein, unknown function
PFIT_09164	1,132067806	29,1727174	31,16676201	31,54630624	85,65804101	32,07761849	80,45282752	73,96374789	35,125585	77,208288	2,1980641	1,1362334	0,0674758	0,3666122	conserved Plasmodium protein, unknown function
PFIT_bin01[illegible]	113,2067806	234,6501182	260,5541304	174,3348503	168,5529194	215,794888	79,25203905	82,49802649	194,51561	80,875033	0,4157766	-1,26612	0,067993	0,3688693	Plasmodium exported protein (PHISTa), unknown function
PFIT_14111	87,16922108	43,75907611	122,1737071	114,0094577	30,39478875	90,4005612	34,82286564	29,39584852	81,317802	32,109357	0,3948626	-1,340577	0,0680549	0,3688693	alpha/beta hydrolase, putative
PFIT_09231	500,3739704	523,8405343	488,0714931	485,923805	291,5136557	244,9563594	165,7088089	208,6156992	422,44664	187,16225	0,4430435	-1,17448	0,068147	0,3688693	DNA-directed RNA polymerase II, putative
PFIT_10074	285,2810872	169,9627884	410,7779234	433,9000719	99,47385408	449,0866589	124,8820009	131,8071917	308,0804	128,3446	0,4165945	-1,263284	0,0681521	0,3688693	conserved Plasmodium protein, unknown function
PFIT_07313	5,660339031	15,85473772	34,90677346	29,88597434	22,10530091	64,15523698	3,602365411	6,637772246	28,761394	5,1200688	0,1780188	-2,489898	0,068281	0,3691258	rifin
PFIT_04023	110,942645	145,2293975	110,3303375	141,6816561	672,8300963	134,1427682	554,7642734	481,7126144	219,19282	518,23844	2,364304	1,2414155	0,0683363	0,3691258	conserved Plasmodium protein, unknown function
PFIT_09357	89,4333567	147,7661555	177,6505435	139,4678802	225,197753	93,31670833	48,03153882	68,27422882	145,47207	58,152884	0,3997529	-1,32282	0,0684568	0,3691258	conserved protein, unknown function
PFIT_13363	26,03755954	80,54206762	107,8369966	89,65792301	56,64483357	172,052681	22,81498094	32,24060805	88,795344	27,527794	0,3100139	-1,689595	0,0685069	0,3691258	protein kinase 6
PFIT_14508	107,5464416	221,3321386	339,0943707	397,3727699	356,4479771	268,2855365	745,6896402	492,1433994	281,67987	618,91652	2,1972337	1,1356884	0,0685258	0,3691258	conserved Plasmodium protein, unknown function
PFIT_10055	33,96203419	50,7351607	46,12680778	47,59618135	218,2898465	49,5745013	183,720636	156,4617744	74,380755	170,09121	2,2867636	1,1933072	0,0686357	0,3693658	conserved Plasmodium protein, unknown function
PFIT_13477	32,82996638	84,34720467	57,97017735	86,33725919	239,0135661	72,90367838	199,3308861	193,4436483	95,566975	196,38727	2,05497	1,0391173	0,0689064	0,3704703	TBC domain protein, putative
PFIT_11134	70,18820399	86,8839627	39,27012014	41,50829769	140,9212933	23,32917708	156,1025012	128,9624322	67,016842	142,53247	2,1268156	1,088695	0,0694053	0,3727981	DNA helicase, putative
PFIT_11030	80,37681425	36,148802	21,81673341	30,43941831	41,4474392	43,74220703	14,40946165	11,37903814	42,328569	12,89425	0,3046229	-1,714904	0,0696254	0,3732857	DnaJ protein, putative
PFIT_03234	23,77342393	39,95393905	26,80341533	28,22564243	60,78957749	32,07761849	79,25203905	76,80850742	35,270603	78,030273	2,2123317	1,1455677	0,0696695	0,3732857	conserved protein, unknown function
PFIT_10216	204,9042729	530,5225546	751,7422998	670,7740907	541,5798722	775,695138	234,1537517	258,8731176	575,8697	246,51343	0,4280715	-1,224076	0,0697895	0,3732857	conserved Plasmodium protein, unknown function
PFIT_08272	70,18820399	60,88219284	26,80341533	32,65319418	192,0398016	17,49688281	164,5080205	145,0827362	66,677282	154,79538	2,321561	1,2150952	0,0697953	0,3732857	GTP binding protein, putative
PFIT_02106	0	25,36758035	22,44006865	30,43941831	105,0001793	26,24532422	102,06702	73,01549471	34,915428	87,541257	2,5072371	1,3260984	0,0699221	0,3732857	Rab GTPase 5a
PFIT_11217	10,18861026	5,07351607	8,103358124	8,855103507	22,10530091	8,748441406	31,22050023	24,65458263	10,512388	27,937541	2,6575827	1,4101146	0,0699766	0,3732857	heat shock factor binding protein 1, putative
PFIT_12019	39,62237322	113,5199221	29,29675629	33,20663815	171,316082	37,90991276	159,7048666	147,9274958	70,811947	153,81618	2,1721784	1,1191426	0,0699952	0,3732857	mitochondrial phosphate carrier protein
PFIT_11190	2807,52816	1185,300192	1843,202306	1598,899627	1096,975557	3703,506862	626,8115816	781,3606187	2039,2355	704,0861	0,3452696	-1,534205	0,0700241	0,3732857	heat shock protein 90, putative
PFIT_14462	112,0747128	58,34543481	38,6467849	31,54630624	146,4476185	37,90991276	136,8898856	137,4967108	70,828462	137,1933	1,9369798	0,9538089	0,070353	0,3746862	conserved Plasmodium protein, unknown function
PFIT_14400	71,3202718	63,41895088	41,7634611	46,48929341	42,82902051	43,74220703	109,2717508	117,5833941	51,593867	113,42757	2,1984701	1,1364999	0,0708711	0,3770904	conserved Plasmodium protein, unknown function
PFIT_14125	2902,621855	1671,089356	2128,06651	2146,255713	1201,975737	1638,87469	845,3550832	878,0824428	1948,1473	861,71876	0,4423273	-1,176814	0,0714357	0,3797374	conserved Plasmodium protein, unknown function
PFIT_01053	124,5274587	93,8600473	97,86363273	88,55103507	241,7767287	131,2266211	265,374252	229,4772691	129,63425	247,42576	1,9086449	0,9325487	0,0715142	0,3797977	Rab GTPase 5c
PFIT_09118	110,942645	39,95393905	52,98349542	51,47028914	171,316082	17,49688281	159,7048666	144,1344831	74,027222	151,91962	2,0522136	1,0371809	0,0716637	0,3802343	conserved Plasmodium protein, unknown function
PFIT_11288	1161,501569	874,5473326	317,2776373	647,529444	473,8823882	335,3569206	1329,272837	1297,210348	635,01588	1313,2416	2,0680453	1,0482678	0,0718311	0,3807655	conserved Plasmodium protein, unknown function
PFIT_11474	165,2818997	85,61558369	115,9403547	68,62705218	23,48688221	5,832294271	187,3230014	177,3233443	77,464011	182,32317	2,35365	1,2348998	0,072381	0,383321	conserved Plasmodium protein, unknown function
PFIT_10126	4297,329393	2797,409923	3027,539262	3011,288636	2077,898285	2534,131861	1289,646817	1320,916677	2957,5996	1305,2817	0,4413315	-1,180065	0,0725663	0,3839432	U3 snoRNA-associated small subunit rRNA processing protein, putative
PFIT_04027	29,43376296	114,1541116	24,93340961	72,50115996	58,02641488	20,41302995	151,2993473	112,8421282	53,243648	132,07074	2,4804975	1,3106295	0,0727315	0,3841634	conserved Plasmodium protein, unknown function
PFIT_01083	1613,196624	2981,324881	3979,995509	3605,687459	2531,056954	3738,500628	1318,465741	1426,17278	3074,9603	1372,3193	0,4462884	-1,163952	0,0727438	0,3841634	phenylalanine--tRNA ligase, putative
PFIT_05304	121,1312553	95,76261583	41,14012586	75,82182378	171,316082	67,07138411	222,145867	161,2030403	95,373881	191,67445	2,0097164	1,0069919	0,0729738	0,3850183	ADP-ribosylation factor GTPase-activating protein, putative
PFIT_13457	2907,150127	901,8174815	1346,404139	1411,835565	909,0804998	1323,930799	525,9453501	588,8652235	1466,7031	557,40529	0,3800396	-1,395778	0,0730571	0,3850988	conserved Plasmodium protein, unknown function
PFIT_13379	109,8105772	206,7457799	153,3404691	192,0450573	670,0669337	169,1365339	554,7642734	507,3154502	250,19089	531,03986	2,125387	1,0857909	0,0731873	0,3854259	conserved Plasmodium protein, unknown function
PFIT_12468	2034,325848	837,1301516	1141,95016	898,793306	793,02767	1703,029927	505,5319461	494,9881589	1234,7095	500,26005	0,4051642	-1,303421	0,073518	0,3864865	pre-mRNA-splicing factor, putative
PFIT_05191	26,03755954	36,148802	87,26693364	95,1923627	96,71069147	125,3943268	162,1064435	152,6687617	77,791779	157,3876	2,0231907	1,0166323	0,0735754	0,3864865	conserved Plasmodium protein, unknown function
PFIT_14399	41,88650883	75,46855154	75,42356408	66,4132763	222,4345904	55,40679557	187,3230014	174,4785848	89,505548	180,90079	2,0211126	1,0151497	0,0735937	0,3864865	small subunit DNA primase
PFIT_03207	14,71688148	88,15234172	69,19021167	101,2802464	157,500269	96,22285547	194,5277322	170,685572	87,845468	182,60665	2,0787259	1,0556996	0,0739376	0,3879321	signal peptidase protein
PFIT_05321	5770,149609	3773,427577	5431,743284	5233,366173	3507,834938	5441,530555	1995,710438	2237,8775	4859,6754	2116,794	0,4355834	-1,198979	0,0742272	0,388787	RNA helicase 1
PFIT_12423	43,01857664	20,92825379	17,45338673	19,37053892	35,92111397	20,41302995	8,405519293	5,689519068	26,18415	7,0475192	0,2691521	-1,893506	0,0742881	0,388787	conserved Plasmodium protein, unknown function
PFIT_10253	0	18,39149575	3,116676201	4,980995723	46,97376443	2,916147135	57,63784658	27,49934216	12,729847	42,568594	3,343999	1,7415744	0,0743067	0,388787	conserved Plasmodium protein, unknown function
PFIT_06263	58,86752593	65,3215194	44,8801373	39,29452181	69,07906533	110,8135911	14,40946165	26,55108898	64,709393	20,480275	0,3164962	-1,65974	0,0748884	0,3914684	mitochondrial import inner membrane translocase subunit TIM22, putative (TIM22)
PFIT_12375	226,4135613	88,78653123	93,50028604	85,23037126	145,0660372	107,897444	38,42523105	53,10217797	124,48237	45,763705	0,367632	-1,443666	0,0750168	0,3917774	ubiquitin-like protein, putative
PFIT_03068	32,82996638	5,707705579	7,480022884	7,748215569	20,7237196	49,5745013	2,401576941	0,948253178	20,677355	1,6749151	0,0810024	-3,625892	0,0752996	0,3925813	small nucleolar RNA snoR03
PFIT_06174	74,71647521	131,2772283	49,24348398	66,96672027	301,1847248	113,7297383	242,559271	231,3737754	122,85306	236,96652	1,9288614	0,9477494	0,075309	0,3925813	malate dehydrogenase
PFIT_07096	2433,945784	1165,006128	1928,599233	2082,056212	1168,817785	1443,492832	739,6856978	784,2053782	1703,653	761,94554	0,4472422	-1,160872	0,0756609	0,3940146	conserved Plasmodium protein, unknown function
PFIT_11430	580,7507846	544,1345985	388,9611899	461,5722303	723,9484047	396,8566204	1056,693854	977,6490265	515,98391	1017,1714	1,9712857	0,9791369	0,0757238	0,3940146	conserved Plasmodium membrane protein, unknown function
PFIT_12177	456,2233259	934,1611464	389,5845252	1274,028017	703,2248851	189,5495438	1575,434473	1428,069286	657,79524	1501,7519	2,2830081	1,190936	0,0758089	0,3940948	thrombospondin-related apical membrane protein (TRAMP)
PFIT_10276	29,43376296	40,58812856	38,02344966	39,84796578	85,65804101	26,24532422	92,46071223	94,8253178	43,299445	93,643015	2,1626839	1,1128228	0,0764259	0,3969376	zinc finger, C3HC4 type, putative
PFIT_04032	604,5242086	479,4472686	761,0923284	633,1399008	389,6059285	1303,51777	222,145867	323,3543337	695,22123	272,7501	0,3923213	-1,349892	0,0765211	0,3970671	6-cysteine protein
PFIT_08020	2516,586733	2016,088448	3347,33024	2947,089136	6479,616328	4345,059232	1593,4463	1371,174095	3608,625	1482,3102	0,4107687	-1,283602	0,0767886	0,3980901	unspecified product
PFIT_13687	113,2067806	436,0283178	698,7588044	649,7432198	399,2769976	594,8940106	208,2148094	208,6156992	478,65136	218,38275	0,456246	-1,132116	0,0769702	0,3986493	U1 small nuclear ribonucleoprotein a, putative
PFIT_02190	182,2629168	278,4091944	177,6505435	278,3823165	712,8959549	233,2917708	682,0478512	537,7462352	302,2157	609,89704	1,98497	0,9891172	0,0770374	0,3986493	replication factor C subunit 1, putative
PFIT_13284	1134,331942	2060,821839	1623,788301	1983,543186	3132,044822	3995,121575	4510,161495	3824,305067	2318,2753	4167,2333	1,7975576	0,8460738	0,0772601	0,3994363	myosin C
PFIT_02217	3,396203419	20,92825379	18,70005721	23,79809068	27,63162613	20,41302995	7,204730823	2,844759534	19,144544	5,0247452	0,2624636	-1,929811	0,077632	0,4007105	RESA-like protein, pseudogene
PFIT_09301	0	5,707705579	3,740011442	8,301659538	34,53953267	11,66458854	33,62207717	20,86156992	10,658916	27,241824	2,555778	1,3537625	0,0776483	0,4007105	conserved Plasmodium protein, unknown function
PFIT_14594	84,90508547	37,77112007	79,78691076	75,26837981	75,98697187	8,748441406	145,2954049	161,2030403	67,011152	153,24922	2,2869184	1,1934067	0,0780793	0,4025677	conserved Plasmodium protein, unknown function
PFIT_14272	2399,983749	1957,108824	2202,866739	1691,32477	1852,700532	4003,870017	851,3590256	1046,871509	2351,3091	949,11527	0,403654	-1,308809	0,0789935	0,4067688	arginine methyltransferase 1
PFIT_14225	71,3202718	108,446406	83,5269222	77,48215569	153,355525	104,9812969	39,62603953	36,98187394	99,865298	38,303947	0,3836068	-1,3823	0,0790899	0,4067688	mitochondrial import inner membrane translocase subunit TIM8, putative (TIM8)
PFIT_09006	5,660339031	41,22231807	42,38656811	54,79095295	85,65804101	43,74220703	98,46465699	98,61833015	46,927335	102,74425	1,8943295	1,7305572	0,0793223	0,4067688	signal peptidase, putative
PFIT_12531	14,71688148	45,02745512	33,66010298	75,26837981	40,06585789	8,748441406	9,606307764	12,32729131	34,247853	10,9668	0,3025503	-1,724753	0,0791818	0,4067688	Plasmodium exported protein, unknown function
PFIT_12006	499,2419026	2201,905974	2394,230658	2209,348325	1742,174028	1137,297383	751,6935825	880,9272024	1697,3664	816,31039	0,4809276	-1,056108	0,0793778	0,4072053	Plasmodium exported protein (PHISTc), unknown function
PFIT_13057	704,1461755	260,6518881	380,2344966	440,5413995	210,0003586	262,4532422	894,5874105	665,673731	376,33793	780,13057	2,0729523	1,0516869	0,0795016	0,4072053	MORN repeat protein, putative
PFIT_14461	28,30169516	18,39149575	31,79009725	29,33253037	38,68427659	67,07138411	6,003842352	13,27554449	35,595247	9,6397434	0,2708155	-1,884618	0,0795339	0,4072053	conserved Plasmodium protein, unknown function
PFIT_11038	5,660339031	56,44286628	52,36016018	42,61518564	35,92111397	37,90991276	7,204730823	18,96506356	38,48493	13,084897	0,3400005	-1,556391	0,0796171	0,4072053	conserved Plasmodium protein, unknown function
PFIT_08208	1419,613029	1453,562354	2061,36964	3066,633203	3115,465846	1896,032013	4904,020113	3802,495394	2119,1156	3598,2577	2,0542806	1,0386332	0,0796804	0,4072053	high mobility group protein B2 (HMGB2)
PFIT_05301	4,528271225	16,48892723	3,116676201	13,28265526	4,14474392	8,748441406	31,22050023	22,75807627	8,3849525	26,989288	3,2187765	1,6865124	0,0796988	0,4072053	NIMA related kinase 2
PFIT_05288	59,99959373	31,70947544	32,4134325	28,22564243	140,9212933	43,74220703	122,480424	94,8253178	56,168607	108,65287	1,9344056	0,9518904	0,0798753	0,4077387	50S ribosomal protein L12, apicoplast, putative
PFIT_03077	1648,290726	750,8803784	821,5558467	645,3156681	855,1988288	1128,548941	1837,20636	1733,406809	974,96506	1785,3066	1,8311493	0,8727494	0,0802913	0,4092437	conserved Plasmodium protein, unknown function
PFIT_13059	61,13166154	32,34366495	32,4134325	37,08074594	34,53953267	11,66458854	92,46071223	71,11898835	34,862271	81,78985	2,346085	1,2302553	0,0803564	0,4092437	conserved Plasmodium protein, unknown function
PFIT_04234	179,9987812	237,8210658	264,9174771	281,7029803	210,0003586	154,5557982	90,05913528	108,1008623	221,49941	99,079999	0,447315	-1,160637	0,0804585	0,4092437	Plasmodium exported protein (hyp15), unknown function
PFIT_09367	1222,633231	1223,985752	2608,034645	1816,956551	874,5409671	1437,660538	641,2210432	789,8948973	1530,6353	715,55797	0,4674908	-1,09699	0,0804663	0,4092437	early transcribed membrane protein
PFIT_14615	78,11267863	26,63595879	49,86683192	36,52729926	38,68427659	81,65211979	21,61412347	28,44759534	51,580015	25,030859	0,4852837	-1,043059	0,0805119	0,4092437	conserved Plasmodium protein, unknown function
PFIT_13148	149,4329504	383,0504633	398,9345538	408,4416493	595,4615432	533,6549258	927,0086992	680,8457818	411,49601	803,92724	1,9536696	0,9661865	0,0806632	0,4095429	phosphatidylinositol synthase
PFIT_14145	18,1130849	19,65987477	33,03676774	33,20663815	163,0265942	84,56826693	129,6851548	112,8421282	58,601871	121,26364	2,0692793	1,0491284	0,0808541	0,4101443	FeS assembly ATPase SufC
PFIT_12393	32,82996638	106,5438375	34,90677346	51,47028914	128,4870615	72,90367838	134,4883087	147,9274958	71,190268	141,2079	1,9835282	0,9880689	0,0810212	0,4106238	hydroxyethylthiazole kinase, putative
PFIT_06212	354,3372234	149,0345346	294,2142334	273,4013208	109,1449232	253,7048008	118,8785843	114,7386345	238,97284	101,79849	0,4259835	-1,23113	0,0813575	0,411783	vacuolar transporter chaperone, putative
PFIT_03055	179,9987812	267,6279727	179,5205492	187,0640616	874,5409671	163,3042396	722,8746592	625,8470975	308,6761	674,36098	2,184877	1,1274271	0,0814612	0,411783	AP endonuclease (DNA-[apurinic or apyrimidinic site] lyase), putative
PFIT_09051	442,6385123	411,5889912	323,2131278	1153,620334	1171,57447	294,5407768	1026,67442	888,5132278	557,96589	957,59369	1,7225484	0,7845464	0,0816484	0,411783	Cu2+-transporting ATPase, putative
PFIT_12315	58,86752593	80,54206762	215,0506579	141,6816561	41,4474392	174,9688281	52,8346927	32,24060805	118,7597	42,53765	0,3581825	-1,481233	0,0816566	0,4118003	conserved Plasmodium protein, unknown function
PFIT_07260	62,26372935	79,2736886	69,19021167	79,69593156	255,5925417	125,3943268	222,145867	186,8058761	111,90174	204,47587	1,8272806	0,8696982	0,0816683	0,4118003	conserved Plasmodium protein, unknown function
PFIT_14173	12,45274587	79,2736886	69,81354691	50,91684517	143,6844559	107,897444	142,893828	151,7205085	77,339788	147,30717	1,9046751	0,9295449	0,0816902	0,4118003	conserved protein, unknown function
PFIT_14248	3648,65454	2272,30101	3877,76853	3806,58762	2399,80673	3437,736911	1454,154838	1475,481945	3225,4759	1464,8184	0,454102	-1,13879	0,0820008	0,4128735	conserved Plasmodium protein, unknown function
PFIT_07231	1862,251541	1744,021149	441,3213501	1297,826108	603,751031	279,950125	2083,367996	2300,46221	1038,1869	2191,9151	2,1112915	1,0781258	0,0820563	0,4128735	kinesin-like protein, putative
PFIT_12167	136,9802046	56,44286628	107,8369966	90,76481095	53,88167096	148,7230639	38,42523105	36,98187394	99,105009	37,703552	0,3804404	-1,394258	0,0821221	0,4128735	conserved Plasmodium protein, unknown function
PFIT_13652	127,9236621	110,9831164	95,9940502	95,1923627	30,39478875	75,81982552	207,0144066	200,0814205	101,2521	203,90891	2,0138754	1,0099023	0,0822273	0,4135373	conserved Plasmodium protein, unknown function
PFIT_12415	26,03755954	16,48892723	16,20671625	6,64132763	33,15795136	20,41302995	34,82286564	48,36091208	19,824252	41,591889	2,0980307	1,0690358	0,0828112	0,4152432	conserved Plasmodium protein, unknown function
PFIT_10029	59,99959373	303,1425852	119,6803661	115,1163456	225,197753	275,9510135	366,2404835	335,681625	183,84779	350,96105	1,9089761	0,9327991	0,0828137	0,4152432	N-acetyltransferase, putative
PFIT_07230	177,7346456	665,2647947	246,8407552	535,1803182	406,1849042	265,3693893	874,1740065	780,4123655	382,76247	827,29319	2,1613749	1,1119493	0,083035	0,4155219	kelch protein, putative
PFIT_05044	81,50888205	89,42072074	91,63028032	116,7766775	426,9086238	110,8135911	348,2286564	298,6995111	152,84313	323,4642	2,116315	1,0815544	0,083096	0,4159212	ATP-dependent helicase, putative
PFIT_13639	127,9236621	169,3285988	231,8807094	191,4916113	106,3817606	172,052681	61,24021199	83,44627967	166,50984	72,343246	0,4344683	-1,202677	0,0832754	0,4162217	ferrochelatase
PFIT_14506	387,1671897	138,8875024	150,8471282	196,4702016	322,9607763	84,56826693	381,8503736	354,6466686	180,15057	368,24871	2,0441162	1,0314772	0,0834324	0,4162217	DNA replication related protein, putative
PFIT_09239	27,16962735	125,5695227	70,43688215	149,4298717	258,3557043	52,49064844	270,1774059	217,1499778	113,90871	243,66369	2,1391138	1,0970132	0,0834956	0,4162217	thioredoxin reductase
PFIT_07303	395,0916644	432,517245	258,6841247	646,976	167,1713381	250,7886536	838,1503524	736,7927193	258,53817	787,47154	2,1963395	1,1351011	0,0835221	0,4162217	serine/threonine protein kinase, FIKK family, pseudogene
PFIT_05278	44,15064444	48,83259218	27,42675057	30,43941831	174,0792446	104,9812969	150,0985588	116,6351409	71,651658	133,36685	1,8613226	0,8962381	0,083524	0,4162217	conserved Plasmodium protein, unknown function
PFIT_14307	1406,028215	1057,828101	1388,16758	1277,348681	895,2646867	991,490026	511,5358884	559,469375	1169,3545	535,50263	0,4579472	-1,126747	0,0837602	0,4162852	diphthamide synthesis protein, putative
PFIT_05208	1447,914724	2404,846617	2955,855709	2664,279268	2238,161717	2723,681424	1003,859161	1185,316473	2405,7899	1094,5878	0,4549806	-1,136123	0,0837807	0,4162852	bis(5'-nucleosyl)-tetraphosphatase [asymmetrical]
PFIT_05048	1621,121099	1047,046879	845,2250843	969,633834	2048,885078	603,642457	2497,64019	2045,382105	1189,262	2271,5111	1,9100174	0,9335858	0,0837833	0,4162852	conserved Plasmodium protein, unknown function
PFIT_13486	715,4668536	464,2267204	609,0118478	490,9048007	426,9086238	518,2408958	265,7442324	245,5979741	535,79346	224,6729	0,4585977	-1,124699	0,0839233	0,4162852	cytidine and deoxycytidylate deaminase, putative
PFIT_07046	2621,869039	1120,612862	706,238873	611,002142	1080,396582	1767,185164	363,8389065	422,9209174	1317,8841	393,37991	0,2984936	-1,744228	0,0839364	0,4162852	40S ribosomal protein S29, putative
PFIT_14533	127,9236621	148,4003451	141,4970995	125,0783337	98,09227277	58,32294271	50,43311576	52,15392479	116,55244	51,29352	0,4400896	-1,184131	0,0839783	0,4162852	leucine-rich repeat protein
PFIT_13631	107,5464416	369,7324836	259,30746	364,7195757	2449,543657	233,2917708	1935,671014	1625,305947	630,69023	1780,4885	2,8230792	1,4972696	0,0842179	0,4171074	cysteine-rich surface protein
PFIT_01087	65,65993276	101,4703214	95,99362701	85,78381523	106,3817606	116,6458854	30,01971176	43,61964619	95,322557	36,819679	0,3862641	-1,372341	0,0844028	0,4171568	FAD-linked sulfhydryl oxidase ERV1, putative (ERV1)
PFIT_13004	4532,799496	1104,758124	602,1418421	422,2777485	472,5008089	1761,35287	3542,35988	3228,802071	1482,6385	3385,564	2,2834724	1,1912293	0,0844673	0,4171568	glycophorin binding protein
PFIT_13174	9,05654245	9,512842632	6,856687643	9,408567476	8,28948784	2,916147135	20,413404	27,49934216	7,6570016	23,95637	3,1286514	1,6455747	0,0845168	0,4171568	glycerol-3-phosphate acyltransferase, putative
PFIT_04137	337,3562063	333,5836816	304,8109325	241,8550145	305,3294688	294,5308607	109,2717508	147,9274958	302,91103	128,59962	0,4245459	-1,236008	0,0845229	0,4171568	conserved Plasmodium protein, unknown function
[illegible]	[illegible]	[illegible]	[illegible]	[illegible]	[illegible]	[illegible]	[illegible]	[illegible]	[illegible]	[illegible]	[illegible]	[illegible]	[illegible]	[illegible]	[illegible]

PFIT_12321	0	7,610274105	11,22003433	11,06887938	24,86846352	11,66458854	25,21655788	23,70632945	11,07204	24,461444	2,2092987	1,1435885	0,1521196	0,5743583	cytochrome b5, putative
PFIT_07134	27,16962735	46,29583414	13,09004005	23,79809068	214,1451025	29,16147135	146,4961934	120,4281536	58,943361	133,46217	2,2642444	1,1790297	0,1527082	0,576196	calcium/calmodulin-dependent protein kinase, putative
PFIT_13433	859,239465	961,4312953	744,2622769	844,555497	663,1590272	938,9993776	1463,761145	1447,03435	835,27449	1455,3977	1,7424185	0,8010912	0,1529112	0,5765772	krox-like protein. putative
PFIT_13206	193,5835949	536,5243244	312,2909554	395,712438	222,4345904	180,8011224	560,7682157	631,5366166	306,89117	596,15242	1,9425532	0,9579541	0,1534416	0,5776333	DHHC-type zinc finger protein, putative
PFIT_09001	52,07511909	81,81044663	172,6638636	121,7576732	40,06585789	148,7235039	46,83075035	54,05043115	102,84941	50,440591	0,4904315	-1,027876	0,1535531	0,5776333	rifin (RIF)
PFIT_11205	208,3004764	430,6146765	289,8508867	420,6174166	397,8954163	122,4781797	654,4297164	550,9350964	311,62618	602,68241	1,9339916	0,9515815	0,1535886	0,5776333	conserved Plasmodium protein, unknown function
PFIT_09360	627,1655647	286,0194685	175,1572025	159,9453071	418,6191359	303,2793021	559,5674272	483,6091208	328,36433	521,58827	1,5884438	0,667614	0,1536771	0,5776333	Plasmodium exported protein, unknown function
PFIT_07187	33,96203419	17,12311674	26,18008009	16,60331908	41,4474392	43,74220703	8,405519293	11,37903814	29,843033	9,8922787	0,331477	-1,593019	0,1537019	0,5776333	conserved Plasmodium protein, unknown function
PFIT_05256	26,03755954	38,68556004	61,71018879	55,34439692	88,42120363	107,897444	118,8780586	94,8253178	63,016059	106,85169	1,6956263	0,7618183	0,1541776	0,5780564	conserved Plasmodium protein, unknown function, putative
PFIT_09211	37,35823761	110,3489745	152,0937986	109,5819059	106,3817606	131,2266211	51,63390423	54,05043115	107,83188	52,842168	0,4900421	-1,029022	0,1542348	0,5780564	ubiquitin conjugating enzyme, putative
PFIT_09316	212,8287476	298,7032586	259,30746	169,3538546	348,1584893	300,3631549	121,2796355	133,7036981	264,78583	127,49167	0,4814898	-1,054423	0,1543002	0,5780564	selenoprotein
PFIT_05135	20,37722051	54,54029776	23,06340389	28,22564243	31,77637005	23,32917708	57,63784658	65,42946928	30,218685	61,533658	2,0362785	1,0259349	0,1543428	0,5780564	GTP binding protein, putative
PFIT_04079	2289,041104	1480,832503	1798,945503	1814,189331	1178,488855	1035,232233	798,5243329	914,1160636	1599,4549	856,3202	0,5353825	-0,901358	0,1543731	0,5780564	conserved Plasmodium protein, unknown function
PFIT_bin0	136,9802046	27,27014888	28,67342105	32,65319418	34,53953267	40,8260599	106,8701739	82,49802649	50,157094	94,6841	1,8877509	0,9166684	0,1544277	0,5780564	stB_ITGvarT11 - erythrocyte membrane protein 1,, putative
PFIT_11257	613,580751	374,1718102	342,2110469	388,5176664	846,909341	463,6673945	945,0205263	708,345124	504,843	826,68283	1,6375048	0,7114991	0,1546401	0,5784686	hypothetical protein
PFIT_13348	432,449902	303,1425852	246,2174199	262,8858854	240,3951474	102,0651497	110,4725393	161,2030403	264,52601	135,83779	0,5135139	-0,961525	0,154881	0,5789868	conserved Plasmodium protein, unknown function
PFIT_12425	2,264135613	25,36758035	18,70005721	16,60331908	52,50008965	17,49688281	52,8346927	36,03362076	22,155344	44,434157	2,0055729	1,0040144	0,15519	0,5796754	conserved Plasmodium membrane protein, unknown function
PFIT_06269	6,792406838	54,54029776	69,81354691	78,58904363	31,77637005	46,65835417	20,413404	25,60283581	48,028337	23,00812	0,479053	-1,061743	0,1552702	0,5796754	6-pyruvoyltetrahydropterin synthase
PFIT_11335	173,2063744	212,4534854	182,6372254	151,6436476	229,3424969	347,0215091	341,0239256	354,6466886	216,05079	347,83531	1,6099701	0,6870339	0,1556049	0,5805419	mitochondrial ribosomal protein L2 precursor
PFIT_14492	4224,877053	3114,504678	3714,454697	3174,554607	2792,175821	4336,31079	1696,714109	1904,092381	3559,4796	1800,4032	0,5058052	-0,983346	0,1561544	0,582208	U3 snoRNA-associated small subunit rRNA processing protein, putative
PFIT_13064	1055,087195	440,7617086	543,5483295	444,4155073	460,0665751	839,850375	300,1971176	301,5445106	630,62162	300,87081	0,477102	-1,06763	0,1563862	0,5826879	DnaJ protein, putative
PFIT_11253	361,1296302	308,2161013	390,8311957	333,1732695	244,5398913	419,9251875	157,3032896	186,8058761	342,96921	172,05458	0,5016619	-0,995213	0,1568986	0,5842121	U6 snRNA-associated Sm-like protein LSm1, putative
PFIT_11247	407,5444103	296,8006901	268,0341533	302,7338512	672,8300963	285,7824193	671,240755	564,2106409	372,2876	617,7257	1,6592701	0,7305488	0,1571414	0,5847315	conserved Plasmodium protein, unknown function
PFIT_08152	88,30128889	59,61381383	172,0405263	118,9904534	87,03962232	29,16147135	184,9214245	184,9093697	92,524529	184,9154	1,9985554	0,9989576	0,1573102	0,5849745	conserved Plasmodium protein, unknown function
PFIT_04016	43,01857664	31,07528593	36,77677918	21,5843148	16,57897568	5,832294271	13,20867318	8,534278602	25,811038	10,871476	0,4211948	-1,24744	0,1580748	0,5874317	probable protein, unknown function
PFIT_10277	1390,179266	1364,141633	2420,410738	3017,929964	1054,146537	1525,144952	1055,493066	989,9763178	1795,3255	1022,7347	0,5696653	-0,811814	0,1583296	0,5877182	nucleolar preribosomal assembly protein, putative
PFIT_05374	13,58481368	15,22054821	31,79009725	21,03087083	20,7237196	52,49064844	45,62996188	47,4126589	25,806783	46,52131	1,8026776	0,8501414	0,1583597	0,5877182	rifin
PFIT_05290	215,0928832	474,3737526	452,5413845	478,7290334	453,1586686	577,3971328	784,1148712	750,0682638	441,88214	767,09157	1,7359642	0,7957372	0,1585294	0,5879622	ubiquitin fusion degradation protein UFD1, putative
PFIT_12128	100,7540348	136,9849339	187,6239073	140,5747682	167,1713381	262,4532422	72,04730823	83,44627967	165,92704	77,746794	0,4685601	-1,093694	0,1587299	0,58832	conserved Plasmodium protein, unknown function
PFIT_14226	2080,740628	1122,515431	1152,546859	969,633834	986,4490529	1959,650875	668,839178	665,673731	1378,5894	667,25645	0,4840139	-1,04688	0,1590508	0,5891232	conserved Plasmodium protein, unknown function
PFIT_12432	24,90549174	64,05314039	34,90677346	30,99286228	92,56594755	46,65835417	98,46465458	77,7567606	49,013762	88,110708	1,7976728	0,8461305	0,1592087	0,5892391	syntaxin, Qa-SNARE family
PFIT_14616	444,9026479	404,6129066	1165,013564	971,8476099	471,1192256	1705,946074	324,212887	344,2159036	860,57367	334,2144	0,3883623	-1,364525	0,1592904	0,5892391	ubiquitin carboxyl-terminal hydrolase isozyme L3
PFIT_14093	26,03755954	8,244463614	9,350028604	7,748215569	4,14474392	14,58073568	2,401576941	2,844759534	11,684291	2,6231682	0,2245038	-2,155188	0,1594476	0,5892672	conserved Plasmodium protein, unknown function
PFIT_13536	365,6579014	1978,037078	2636,084731	2662,618936	1515,594693	3373,982236	3568,743334	3690,601369	2088,6626	3629,6724	1,7377974	0,7972599	0,1597347	0,5892672	V-type ATPase, putative
PFIT_08346	107866,8168	96741,17024	74097,73002	50969,42234	55813,12163	168783,68	40315,27211	45852,78242	92378,657	43084,027	0,4663851	-1,100406	0,1598011	0,5892672	heat shock protein 70 (HSP70-x)
PFIT_05171	8437,30136	4554,749052	3432,707168	2414,122594	3951,322537	6062,669894	1966,891515	2578,300391	4808,8121	2272,596	0,4725899	-1,081339	0,1598192	0,5892672	60S ribosomal protein L12, putative
PFIT_11044	238,8663071	553,0132517	555,3916991	481,4962532	360,592721	498,6611602	214,9411362	259,8213708	448,00357	237,38125	0,5298647	-0,916304	0,1599369	0,5892672	actin-like protein, putative (ALP2a)
PFIT_12329	715,4668536	297,4348796	299,8242506	307,7148469	270,7899361	253,7048008	175,3151167	179,2198506	357,48926	177,26748	0,495868	-1,011972	0,1601697	0,5892672	conserved protein, unknown function
PFIT_11490	758,4854302	1464,977765	2341,247163	2399,179606	1445,134047	1146,045824	916,201603	905,581785	1592,5116	910,89169	0,5719843	-0,805952	0,1601745	0,5892672	Plasmodium exported protein (hyp11), unknown function
PFIT_03118	65,65993276	78,63949909	104,7203204	84,12348332	93,94752885	90,4005612	159,7048666	152,6687617	86,248554	156,18681	1,810892	0,8567005	0,1602006	0,5892672	conserved Plasmodium protein, unknown function
PFIT_02165	91,69749231	162,9867038	173,910532	152,1970915	599,6062871	172,052681	415,4728108	397,3180816	225,40846	406,39545	1,8029289	0,8503425	0,1605136	0,5892672	ATP synthase F1, alpha subunit
PFIT_12517	6629,389074	2780,286806	3713,831362	3689,810943	2238,161717	4412,130616	1813,19059	2137,362663	3910,6018	1975,2766	0,5051081	-0,985336	0,1605293	0,5892672	DEAD/DEAH box ATP-dependent RNA helicase, putative
PFIT_08144	604,5242086	884,0601752	1204,907019	1107,441382	752,9618121	644,4685169	477,9138112	489,2986399	866,39385	483,60623	0,5581829	-0,84119	0,1605429	0,5892672	histone-arginine methyltransferase, putative
PF_apicopla	181,130849	346,2674718	426,9846396	330,4060496	215,5266838	274,1178307	172,9135397	151,7205085	295,73892	162,31702	0,5488524	-0,86551	0,1605908	0,5892672	probable protein, unknown function
PFIT_05216	30,56583077	39,95393905	65,45020023	44,27551754	69,07906533	81,65211979	122,480424	74,91200106	55,162779	98,696213	1,7891813	0,8392996	0,1606524	0,5892672	conserved Plasmodium protein, unknown function
PFIT_14080	76,98061083	97,66518435	102,2269794	122,8645612	66,31590272	52,49064844	194,5277322	150,7722553	86,423981	172,64999	1,9977093	0,9983467	0,1611125	0,5905718	LCCL domain-containing protein
PFIT_11107	8136,171324	4762,129021	5411,173221	4935,613317	4751,258114	9235,437978	2879,490752	3169,062121	6205,2972	3024,2764	0,4873701	-1,03691	0,1615419	0,5915336	apicoplast ribosomal protein L36e precursor, putative
PFIT_12023	4706,005871	2880,488749	3869,665172	4737,480376	2065,464053	3245,671762	5857,446159	6762,941666	3584,1293	6310,1939	1,7605933	0,8160616	0,161584	0,5915336	conserved protein, unknown function
PFIT_05257	44,15064444	33,61204397	34,28343822	22,69120274	29,01320744	49,5745013	13,20867318	14,22379767	35,554173	13,716235	0,3857841	-1,374134	0,1618195	0,5920125	ribosomal large subunit pseudouridylate synthase, putative
PFIT_11207	4462,611292	4545,870399	6321,866007	6178,648472	4150,270245	5249,064844	2745,002443	2836,225255	5151,3885	2790,6138	0,5417207	-0,884379	0,1620165	0,5920732	conserved Plasmodium protein, unknown function
PFIT_12281	9704,085235	4356,881925	4830,848112	4889,124024	2297,569713	4222,581052	2513,250269	2661,746671	5050,1817	2587,4985	0,5123575	-0,964777	0,1620455	0,5920732	conserved Plasmodium protein, unknown function
PFIT_06209	23,77342393	71,02922498	81,03358124	54,79095295	60,78957749	102,0651497	27,61813482	31,29235487	65,580318	29,455245	0,4491476	-1,154738	0,1622106	0,5922937	conserved Plasmodium protein, unknown function
PFIT_08167	82,64094986	195,9645582	125,9137185	131,1662207	70,46064664	52,49064844	210,1379823	226,6325095	109,77279	218,38525	1,9894297	0,9923549	0,1625472	0,5929363	GDP-mannose 4,6-dehydratase, putative
PFIT_09308	2050,174797	1029,289573	1024,1398	1024,424787	779,211857	918,5863476	588,3863505	595,5029958	1137,6379	591,94467	0,5203279	-0,942507	0,1625962	0,5929363	peptidyl-prolyl cis-trans isomerase
PFIT_05149	713,202718	525,1089133	407,6612472	346,4559247	431,0533677	571,5648385	210,1379823	276,889928	499,1745	243,51396	0,4878333	-1,03554	0,1627631	0,5931624	kinase, putative
PF_apicopla	161,8856963	90,68909976	85,39692792	51,47028914	80,13171579	69,98753125	38,42523105	44,56789937	89,926877	41,496565	0,4614479	-1,11576	0,1629461	0,5934471	apicoplast ribosomal protein S2
PFIT_11482	62,26372935	53,90610825	41,7634611	41,50829769	145,0660372	52,49064844	103,2678085	113,7903814	66,16638	108,52909	1,6402453	0,7139116	0,1631894	0,5936783	conserved Plasmodium protein, unknown function
PFIT_13388	366,7899692	273,3356783	175,1572025	157,1780873	201,7108708	489,9127187	110,4725393	138,444964	277,34742	124,45875	0,4487467	-1,156027	0,1632195	0,5936783	conserved Plasmodium protein, unknown function
PFIT_13508	168,6781031	265,7254042	219,4140046	731,6529273	216,9082651	160,3880924	622,0084277	594,5547426	293,79447	608,28159	2,0704324	1,0499321	0,1639742	0,5942948	glycerol kinase
PFIT_02045	1,132067806	7,610274105	1,246670481	4,427551754	26,25004483	0	21,61419247	14,22379767	6,7777682	17,918995	2,6437899	1,4026075	0,1640025	0,5942948	conserved Plasmodium protein, unknown function
PFIT_01049	1866,779813	1014,069025	1553,974754	1583,403196	1106,646627	1662,203867	746,8904286	772,8263401	1464,5129	759,85838	0,5188472	-0,946618	0,1640753	0,5942948	pre-rRNA-processing protein TSR2, putative
PFIT_02174	46,41478006	60,24800333	79,16357552	105,1543541	283,2241679	104,9812969	206,5356169	185,8576229	113,1977	196,19662	1,733221	0,7934556	0,1641978	0,5942948	replication factor C, subunit 2, putative
PFIT_05284	15,84894929	15,85473772	2,493340961	6,64132763	15,19739437	5,832294271	25,21655788	20,86156992	10,311341	23,039064	2,2343422	1,1598502	0,1642554	0,5942948	conserved Plasmodium membrane protein, unknown function
PFIT_08154	12781,04553	5361,034222	7731,226985	8285,056219	4284,283632	7036,663038	3766,873432	4172,313983	7546,5516	3969,5937	0,5260143	-0,926826	0,1643182	0,5942948	RNA binding protein, putative
PFIT_14678	319,2431214	639,2630249	716,8355263	613,7693618	632,7642384	804,8566094	294,1931753	346,11241	621,12198	320,15279	0,5154427	-0,956116	0,1643758	0,5942948	large ribosomal subunit assembling protein, putative
PFIT_11329	66,79200057	121,1301962	75,42356408	126,185225	51,11850835	46,65835417	156,1025012	167,8408125	81,217975	161,97166	1,9942834	0,9958704	0,1643964	0,5942948	conserved Plasmodium protein, unknown function
PFIT_13421	8834,65716	3768,354061	4389,526762	3719,143473	2821,189028	4301,317025	2301,911498	2462,613503	4639,0313	2382,2625	0,5135259	-0,961491	0,1644214	0,5942948	transcription factor with AP2 domain(s)
PFIT_05059	851,3149903	322,80246	393,9478719	327,0853858	353,6848145	399,5121575	210,1379823	226,6325095	441,39128	218,38525	0,4947657	-1,015183	0,1644398	0,5942948	conserved Plasmodium protein, unknown function
PFIT_13350	75,84854302	60,24800333	57,34684211	87,44414713	274,93468	172,052681	248,5632134	149,8240021	121,31248	199,19361	1,6419877	0,7156433	0,1645958	0,5944787	conserved Plasmodium protein, unknown function
PFIT_14673	672,4482769	370,3666731	471,2414417	494,7789085	291,5136557	618,2231927	812,9337945	811,7047204	486,42869	812,31926	1,6699658	0,7398185	0,1652453	0,5959959	conserved Plasmodium protein, unknown function
PFIT_14269	1098,105772	2151,170814	2230,29349	1867,319952	2098,622005	2825,746574	951,0244686	1145,489839	2045,2098	1048,2572	0,5125426	-0,964256	0,1652557	0,5959959	conserved Plasmodium protein, unknown function
PFIT_13211	678,108616	2004,038848	1679,265137	2136,847165	1225,462619	801,9404622	2868,683656	2620,023531	1420,9438	2744,3536	1,9313597	0,9496169	0,165332	0,5959959	conserved Plasmodium protein, unknown function
PFIT_08284	7426,364809	2273,569389	3145,972958	3258,124647	1924,54276	2306,672384	1410,926453	1404,362957	3389,2078	1407,6447	0,4153315	-1,267665	0,165472	0,5961208	conserved protein, unknown function
PFIT_13633	4826,005058	2256,446272	2611,151322	3200,566474	1703,489751	1918,824815	1366,497279	1585,479314	2752,7473	1475,9883	0,5361874	-0,899191	0,1656817	0,5964963	pre-mRNA-splicing factor ATP-dependent RNA helicase PRP16 (PRP16)
PFIT_04146	629,4297003	344,9990928	563,4950572	461,0188263	768,1592065	1936,321698	1364,095702	1059,1988	783,90393	1211,6473	1,5456578	0,628221	0,1660412	0,5972811	RNA binding protein, putative
PFIT_10176	31,69789858	42,49069709	52,98349542	36,52730197	20,7237196	26,24532422	14,40946165	17,0685572	35,111406	15,739009	0,4482592	-1,157595	0,1661928	0,5972811	conserved Plasmodium protein, unknown function
PFIT_11118	263,7717989	511,7909336	409,5312529	465,4463781	413,0928107	253,7048008	193,3269437	229,4772691	386,223	211,40211	0,5473576	-0,869444	0,1662165	0,5972811	tRNA m5C-methyltransferase, putative
PFIT_08242	4367,517597	2304,644675	2584,347906	2673,134371	1849,93737	2531,215714	1397,71778	1457,465135	2718,4663	1427,5915	0,5251459	-0,92921	0,1663904	0,5975265	ATP-dependent RNA helicase prh1, putative
PFIT_12339	276,2245447	331,0469236	266,7874828	292,7718597	429,6717864	332,4407734	566,7721581	515,8497288	321,49056	541,31094	1,6837538	0,7516812	0,1669344	0,5990997	conserved Plasmodium protein, unknown function, pseudogene
PFIT_08093	13,58481368	8,878653123	3,740011442	4,980995723	13,81581307	8,748441406	19,21261553	18,96506356	8,9581214	19,08884	2,1308976	1,0914613	0,1670881	0,599271	glycosyltransferase family 28 protein, putative
PFIT_12353	38,49030541	131,9114178	97,86363273	120,0973413	910,4620811	110,8135911	593,1895044	504,4706907	234,93973	548,8301	2,3360464	1,2240689	0,1673452	0,5998126	serine hydroxymethyltransferase
PFIT_09299	0	5,07351607	6,856687643	8,855103507	49,73692704	2,916147135	31,2205023	30,3441017	12,23973	30,782301	2,5149493	1,3305293	0,167562	0,6000776	conserved Plasmodium protein, unknown function
PFIT_11097	1444,518521	643,7023514	700,0054748	589,4178272	512,5666648	1318,098505	386,6538875	441,885981	868,05156	414,26993	0,4772412	-1,06721	0,1676313	0,6000776	conserved Plasmodium protein, unknown function
PFIT_14638	16,98101709	29,1727174	19,94672769	12,17576732	11,05265045	11,66458854	33,62207717	39,82663348	16,832245	36,724255	2,181786	1,1255096	0,1680222	0,6008029	conserved Plasmodium membrane protein, unknown function
PFIT_06046	2726,019278	[illegible]	1555,84476	1317,55647	1152,24881	2009,23476	772,30775	887,5649744	1463,3451	830,43637	0,4992548	-1,002146	0,1683243	0,6008029	DNA excision-repair helicase, putative
PFIT_14781	2509,794327	1578,497687	1237,320452	1359,258388	1706,252914	6631,318586	399,8625607	575,5896791	2503,7404	487,72612	0,194799	-2,359942	0,1682227	0,6010534	Plasmodium exported protein (hyp17), unknown function
PFIT_05126	588,6752593	348,8042298	483,0848112	472,6411497	301,1847248	373,2668333	231,7521748	224,7360032	427,94283	228,24409	0,5333518	-0,906841	0,1683336	0,60107	conserved Plasmodium protein, unknown function
PFIT_14024	911,3145841	878,9866592	1474,187843	1513,115812	390,9875098	1119,8005	534,3508694	635,3296293	1048,0655	584,84025	0,5580188	-0,841614	0,1686302	0,601749	lysophospholipase, putative
PFIT_05196	17259,50577	6027,337091	6563,096745	5894,178372	5944,944363	9888,654936	3758,467913	3923,871651	8596,2862	3841,1698	0,4468406	-1,162168	0,1688367	0,6021062	40S ribosomal protein S24, putative
PFIT_12197	10,18861026	8,244463614	13,71337529	18,26365098	23,48688221	2,916147135	24,01576941	33,18886123	12,802188	28,602315	2,2341739	1,1597415	0,1693036	0,6032328	conserved Plasmodium protein, unknown function
PFIT_13467	39074,4524	22018,42556	28176,62287	29012,08631	19963,84988	33037,0309	14001,19357	15689,79708	28547,078	14845,495	0,5200355	-0,943318	0,1693659	0,6032328	DNA/RNA-binding protein Alba 4 (ALBA4)
PFIT_11432	1832,817778	1736,410875	1633,761665	1401,32013	1438,22614	2035,470701	800,9259098	926,4433549	1679,6679	863,68463	0,5141996	-0,959599	0,1700093	0,6050018	60S ribosomal protein L28, putative
PFIT_bin0	83,77301766	39,95393905	71,06021739	52,0237331	16,57897568	128,310474	99,66544305	112,8421282	65,283393	106,25379	1,6275776	0,7027263	0,1700765	0,6050018	BC_ITvar16 - erythrocyte membrane protein 1,, putative
PFIT_12248	330,5637994	227,6740337	242,4774085	228,0189153	299,8031435	571,5648385	140,492251	150,7722553	316,68369	145,63225	0,4598666	-1,120713	0,1703988	0,6054323	isoleucine--tRNA ligase, putative
PFIT_07327	1124,143332	4851,549742	4165,749411	4356,710926	3191,815218	4458,78897	1884,03711	2175,29279	3691,3992	2029,665	0,5498362	-0,862926	0,1704667	0,6054323	Plasmodium exported protein (PHIST), unknown function, pseudogene
PFIT_08095	2833,565719	1302,855556	1463,591144	1467,733406	857,9619914	904,005612	697,6581013	762,3955551	1438,2856	730,02683	0,5075674	-0,978329	0,1705499	0,6054323	kinesin-like protein, putative
PFIT_bin0	7,924474644	1,902568526	4,363346682	0,553443969	22,10530091	2,916147135	0	0	6,627547	0	0	#DIV/0!	0,1707177	0,6054323	Plasmodium RNA of unkown function RUF6-10
PFIT_13603	4,528271225	3,805137053	3,116676201	1,106887938	1,381581307	0	8,405519293	9,48253178	2,3230923	8,9440255	3,8500518	1,9448778	0,1707705	0,6054323	conserved Plasmodium protein, unknown function
PFIT_04111	81,50888205	65,3215194	279,8775229	289,4511959	505,6587582	676,5461354	72,04730823	87,23929238	316,394	79,6433	0,2517219	-1,990097	0,1708398	0,6054323	Plasmodium RNA of unkown function RUF6-14
PFIT_06134	1557,725301	1269,013207	1160,650217	1028,852339	895,2646867	2417,485975	626,8115816	720,6724153	1388,1653	673,742	0,4853471	-1,042911	0,171001	0,6055932	conserved protein, unknown function
PFIT_12410	153,9612217	84,34720467	99,11030321	64,7529444	42,82902051	29,16147135	44,42917341	32,24060805	79,027028	38,334891	0,4850858	-1,043688	0,1711258	0,6055932	Plasmodium RNA of unkown function RUF6-3
PFIT_09222	21449,28873	9207,163288	12696,09217	13051,86913	7754,815874	12580,25874	6448,234086	7023,71129	12789,915	6735,9727	0,5266628	-0,925048	0,1712591	0,6055932	ubiquitin-like protein, putative
PFIT_02036	3455,070945	2154,975951	1833,852277	1344,315401	1794,674117	3420,64059	1091,51672	1175,833941	2333,9215	1133,6753	0,4857384	-1,041749	0,171372	0,6055932	conserved Plasmodium protein, unknown function
PFIT_13129	30,56583077	100,2019424	140,2504291	97,95958255	156,1186877	166,2203867	55,23626964	54,99868432	115,21948	55,117477	0,4783694	-1,063803	0,1714228	0,6055932	septum formation protein MAF homologue, putative
PFIT_11187	502,638106	471,202805	503,0315389	485,370391	670,0669337	764,0305495	1023,071777	813,6012267	566,05672	918,3365	1,6223401	0,6980763	0,1715276	0,6055932	conserved Plasmodium protein, unknown function
PFIT_11380	2564,133581	2507,585318	2963,335732	3037,853947	1942,503317	2012,141523	1336,477568	1454,620375	2504,5922	1395,549	0,5571961	-0,843743	0,1722554	0,6077834	conserved Plasmodium membrane protein, unknown function
PFIT_13312	4960,721127	4881,990839	5301,466219	4097,145704	5642,378056	8926,326381	2497,640019	3029,668904	5635,0047	2763,6545	0,490444	-1,02784	0,1724047	0,6079306	60S ribosomal protein L23, putative
PFIT_11274	11411,24349	4354,345167	5654,8973	6691,691032	3500,927031	3957,211663	3071,616907	3159,579589	5928,3859	3115,5982	0,525539	-0,92813	0,1725188	0,607954	conserved Plasmodium protein, unknown function
PFIT_13147	174,3384422	571,4047474	497,4215218	556,764633	379,9348593	533,6549258	870,5716411	752,9130233	452,25319	811,74233	1,7948847	0,8438912	0,1728182	0,6084726	conserved Plasmodium protein, unknown function
PFIT_11446	295,4696974	105,2754585	182,6372254	179,86929	90,09227277	242,0402122	79,25203905	98,61833051	183,89736	88,935185	0,4836132	-1,048075	0,1729389	0,6084726	carbohydrate kinase, putative
PFIT_09137	98,48989915	93,8600471	88,51360412	106,8416861	359,2111397	49,5745013	286,9884444	214,3052182	132,74398	250,64683	1,8881974	0,9170096	0,1729887	0,6084726	conserved Plasmodium protein, unknown function
PFIT_02092	1132,067806	702,0477862	658,2420137	574,47484	623,0931693	746,5336667	381,8507336	379,3012712	739,40988	380,576	0,5147023	-0,95819	0,1731141	0,6085352	unspecified product
PFIT_14664	472,0722752	834,5933936	1170,000246	1156,697896	719,8038608	1049,812969	439,4885802	542,4008178	900,49677	490,9447	0,545193	-0,875161	0,1732858	0,6087604	tRNA 3'-trailer sequence RNase, putative
PFIT_08335	12706,32906	12986,93276	10774,97296	10718,54935	9501,134646	6004,346952	5716,953908	5990,115326	10448,711	5853,5346	0,560216	-0,835945	0,1734038	0,6087969	Plasmodium exported protein (PHISTc), unknown function
PFIT_14100	913,5787197	790,2001279	971,7796396	830,7193978	886,9751989	1061,477557	426,279907	511,1084629	909,12177	468,6948	0,5155461	-0,955827	0,1737724	0,6097128	conserved Plasmodium protein, unknown function
PFIT_12384	119,9991875	55,17448726	65,47683639	68,07360821	91,18436624	139,9750625	146,4961934	129,9106854	88,313925	138,20344	1,5649111	0,6460807	0,1740449	0,6102902	conserved Plasmodium protein, unknown function
PFIT_12318	5,660339031	20,29406428	41,14012586	42,61518563	140,9212933	102,0651497	97,26386611	100,5148369	58,782693	98,889351	1,6822869	0,750423	0,17433	0,6104728	phenylalanyl-tRNA synthetase alpha chain, putative
PFIT_05235	3082,620637	939,2346625	1376,947546	1738,920951	757,106556	1108,135911	700,0596783	687,4835541	1500,4944	693,77162	0,462362	-1,112905	0,1744736	0,6104728	conserved Plasmodium protein, unknown function, putative
PFIT_13104	1565,649776	550,4764936	652,0086613	499,2064602	610,6589375	796,108168	361,4373296	366,0257267	779,01808	363,73153	0,4669103	-1,098783	0,1744792	0,6104728	alternative splicing regulator, putative
PFIT_11304	573,9583778	183,9149575	84,77359268	151,6436476	107,7633419	81,65211979	399,8625607	392,5768157	197,28434	396,21969	2,0083687	1,0060241	0,1745287	0,6104728	phosphatidylinositol-4-phosphate-5-kinase, putative
PFIT_10148	1592,819403	1397,753077	1728,508621	1660,885352	1157,765135	1668,036161	780,5125058	868,5999111	1534,2947	824,55621	0,5374171	-0,895886	0,1748333	0,6110742	methionine aminopeptidase 1b, putative
PFIT_02195	14224,43199	6321,601024	8204,338433	8674,127329	4839,679317	6794,622825	4229,176993	4579,114597	8176,4668	4404,1458	0,5386368	-0,892615	0,1749255	0,6110742	DnaJ protein, putative
PFIT_bin0	19401,37806	5858,008493	6826,144236	4798,359213	4993,034842	12740,64683	2587,699154	3640,34395	9102,9286	3114,0216	0,3420901	-1,547552	0,1750248	0,6110742	erythrocyte membrane protein 1, PfEMP1 (VAR)
PFIT_12537	183,3949846	156,6448087	143,9904405	136,1472164	62,17115398	64,15523698	61,24021199	72,06724153	124,41731	66,653727	0,5357271	-0,900343	0,1752592	0,6114836	Plasmodium exported protein (PHISTa), unknown function
PFIT_07222	2695,453447	2306,143359	1585,145158	1174,961547	1000,264866	3199,013408	958,2291994	983,3385456	1960,163	970,78387	0,4952567	-1,013752	0,1754744	0,6118884	Rab GTPase activator and protein kinase, putative
PFIT_04015	1462,631606	982,9937386	898,8494165	1069,807192	305,3294688	367,4345391	549,9611195	388,783803	847,84099	469,37246	0,5536091	-0,853061	0,1757573	0,6122532	erythrocyte membrane protein 1 (PfEMP1), pseudogene
PFIT_13367	7861,078847	5539,01117	8654,386476	7825,697724	4669,744816	9302,509362	3940,98776	3891,631043	7308,7381	3916,3094	0,5358393	-0,900128	0,1757955	0,6122532	conserved Plasmodium protein, unknown function
PFIT_10355	176,6025778	219,42957	97,86363273	282,2564243	192,0398016	78,73597266	366,2404835	291,1137257	174,488	328,6771	1,883666	0,9135432	0,1759688	0,6124798	merozoite surface protein (MSP3.8)
PFIT_14557	263,7717989	855,5216473	814,6991591	814,6695227	587,1720553	332,4407734	309,8034254	399,2145879	611,37916	354,50901	0,5798513	-0,786245	0,1762126	0,6127806	nifU protein, putative
PFIT_05139	116,602984	81,17625712	80,410246	83,57003935	302,5663062	93,31670833	213,7403477	193,4436483	126,27376	203,592	1,6123065	0,689126	0,1762719	0,6127806	Rab GTPase 1a
PFIT_06268	816,2208883	1121,881241	2041,422912	2145,148825	1037,567561	1761,35287	839,3511409	831,6180371	1487,2657	835,48459	0,5617588	-0,831977	0,1764403	0,6129891	conserved Plasmodium protein, unknown function
PFIT_03102	2971,677991	2174,001636	2474,017569	2215,436209	1874,805833	2720,765277	1174,371124	1351,260779	2405,1174	1262,816	0,5250538	-0,929463	0,1767266	0,6134689	phosphoglycerate mutase, putative
PFIT_10199	2037,722051	708,3896813	845,2425858	773,7146689	625,8563319	839,850375	410,6686569	474,126589	971,79595	442,39812	0,4552377	-1,135308	0,1767953	0,6134689	S-adenosyl-L-methionine-dependent methyltransferase, putative
PFIT_10031	2438,474055	3595,223025	3599,137677	3329,518919	2924,807626	5908,114096	1828,800841	1914,523166	3632,5454	1871,662	0,5152481	-0,956661	0,1770058	0,6138225	40S ribosomal protein S20e, putative
PFIT_09117	0	1,268379018	4,986681922	7,1947716	2,763162613	2,916147135	10,80709623	11,37903814	3,1881904	11,093067	3,4794243	1,7988486	0,1772307	0,6142261	conserved Plasmodium protein, unknown function
PFIT_07143	21053,06499	14089,78361	12236,013	16944,794	9060,410209	9681,60849	7598,589441	8673,671819	14510,947	8136,1306	0,5606892	-0,834727	0,1774022	0,614444	conserved Plasmodium protein, unknown function
PFIT_09124	174,3384422	220,0637595	319,7709783	267,3134371	245,9214726	291,6147135	135,6890972	131,8071917	253,17047	133,74814	0,5282928	-0,92059	0,1779474	0,6155006	conserved Plasmodium membrane protein, unknown function
PFIT_13460	5,660339031	11,41541116	16,20671625	12,72921129	17,96059699	8,748441406	18,01182706	34,13711441	12,120113	26,074471	2,151339	1,1052349	0,1780033	0,6155006	6-cysteine protein
PFIT_14077	1,132067806	27,27014888	15,58338101	13,28265526	70,46064664	49,5745013	55,23626964	45,51615254	29,550567	50,376211	1,704746	0,7695568	0,1780338	0,6155006	phosphatase, putative

[illegible]

PFIT_08184	22,64135613	56,44286628	25,55674485	34,86697006	294,2768183	20,41302995	187,3230014	136,5484576	75,699631	161,93573	2,1391878	1,0970632	0,2406435	0,6804898	conserved Plasmodium protein, unknown function
PFIT_02136	81,50888205	210,5509169	364,6511156	313,2492866	192,0398016	329,5246263	138,0906741	152,6687617	248,58744	145,37972	0,5848233	-0,773927	0,2407887	0,6804898	mitochondrial ribosomal protein L13 precursor, putative
PFIT_04139	2878,848431	1595,620804	2040,176241	2379,809068	859,3435727	1933,405551	1006,260738	1281,090043	1947,8673	1143,6754	0,5871424	-0,768218	0,2407924	0,6804898	CDK-related kinase
PFIT_09334	59,99959373	30,44109642	61,08685355	43,1686296	34,53953267	40,8260599	24,01576941	21,80982309	45,010294	22,912796	0,5090568	-0,974101	0,2407994	0,6804898	conserved Plasmodium protein, unknown function
PFIT_03193	46,41478006	136,3507444	160,820492	141,1282121	114,6712485	84,56826693	63,64178893	71,11898835	113,99229	67,380389	0,591096	-0,758536	0,2409354	0,6804898	ubiquitin conjugating enzyme, putative
PFIT_05259	193,5835949	384,3188423	448,1780378	384,0901146	323,2900258	268,2855365	182,5198475	219,0464841	333,62436	200,78317	0,6018241	-0,732586	0,2411336	0,6804898	DEAD/DEAH box ATP-dependent RNA helicase, putative
PFIT_13357	96,22576353	175,6704939	201,9606179	270,6341009	142,3028746	148,7235039	302,5986946	295,8549915	172,58623	299,22684	1,7337817	0,7939223	0,2412198	0,6804898	RanBPM and CLTH-like protein, putative
PFIT_06281	974,7103812	1174,51897	1404,374296	1157,25134	1345,660193	1831,340401	731,2801785	743,4304916	1314,6426	737,35534	0,5608789	-0,834239	0,2412274	0,6804898	DnaJ protein, putative
PFIT_13318	467,544004	818,1044663	978,6363273	796,4058717	726,7117673	799,0243151	441,8901571	467,4888168	764,40446	454,68949	0,5948284	-0,749455	0,2414846	0,6807054	SAM-dependent RNA methyltransferase, putative
PFIT_05349	1108,294382	1369,849339	1357,624153	1209,275073	1034,804399	1624,293954	694,0557359	780,4123655	1284,0236	737,23405	0,5741593	-0,800477	0,2416616	0,6807054	conserved Plasmodium protein, unknown function
PFIT_12331	69,05613618	55,80867677	52,98349542	44,8289615	93,94752885	102,0651497	112,8741162	94,8253178	69,781658	103,84972	1,4882094	0,5735775	0,2418528	0,6807054	pentatricopeptide repeat protein, putative
PFIT_03065	58,86752593	11,41541116	13,71337529	14,3895432	127,1054802	20,41302995	79,25203905	64,4812161	40,984061	71,866628	1,7535263	0,810259	0,2418687	0,6807054	conserved Plasmodium protein, unknown function
PFIT_13573	2981,866602	2260,251409	2780,698507	3196,692366	1812,634674	1985,896199	1487,776915	1557,031718	2503,0066	1522,4043	0,6082302	-0,717311	0,2419056	0,6807054	conserved Plasmodium protein, unknown function
PFIT_06037	124,5274587	93,8600471	74,17689359	68,07360821	117,4344111	183,7172695	46,83075035	58,79169704	110,29828	52,811224	0,4788037	-1,062494	0,242131	0,6809257	RAP protein, putative
PFIT_04104	122,2633231	157,2789982	88,51360412	102,9405783	335,7242575	104,9812969	241,3584826	216,2017246	151,95034	228,7801	1,5056241	0,5903617	0,2422717	0,6809257	conserved Plasmodium protein, unknown function
PFIT_01130	611,3166154	409,0522332	405,1679062	284,4702002	234,8688221	895,2571706	236,5553287	231,3737754	473,35549	233,96455	0,4942682	-1,016634	0,2424239	0,6809257	Plasmodium exported protein (hyp4), unknown function
PFIT_13242	204,9042729	724,244419	835,8925572	748,2562464	621,711588	1003,154615	411,8704454	394,4733221	689,69395	403,17188	0,5845664	-0,774561	0,2425528	0,6809257	U6 snRNA-associated Sm-like protein LSm6, putative
PFIT_11164	1030,181704	1478,929934	1444,267752	1194,332086	3793,822268	3406,059854	2923,919926	2635,195582	2057,9323	2779,5578	1,3506556	0,4336599	0,2425858	0,6809257	peptidyl-prolyl cis-trans isomerase
PFIT_11280	97,35783134	126,2037122	162,6904977	173,2279624	156,1186877	102,0651497	76,85046211	81,54977331	136,27731	79,200118	0,5811688	-0,782971	0,2427691	0,6810969	deoxyuridine 5'-triphosphate nucleotidohydrolase
PFIT_12083	132,4519333	75,46855154	88,51360412	55,34439692	134,0133867	122,4781797	54,03548117	47,4126589	101,37834	50,72407	0,5003442	-0,999007	0,2428876	0,6810969	conserved Plasmodium protein, unknown function
PFIT_06217	24927,00103	12080,04176	12700,45552	10682,02205	9466,595113	15096,89372	7470,105075	8372,127309	14158,835	7921,1162	0,5594469	-0,837927	0,2431204	0,6810974	leucine--tRNA ligase, putative
PFIT_11233	999,6158729	820,6412244	680,6820824	713,9427203	519,4745713	577,3971328	405,866503	442,8342341	718,6256	424,35037	0,5905027	-0,759984	0,2431739	0,6810974	conserved Plasmodium protein, unknown function
PFIT_14585	2525,643276	4460,889005	6644,130326	7143,30131	4473,560271	5249,064844	3065,612965	3189,923691	5082,7648	3127,7683	0,6153675	-0,70048	0,2432491	0,6810974	conserved Plasmodium protein, unknown function
PFIT_08112	1061,879602	2088,386052	1424,321024	1868,980284	1537,699994	1081,890587	2485,632134	2542,26677	1510,5263	2513,9495	1,6642872	0,7349044	0,2435704	0,6815893	ubiquitin regulatory protein, putative
PFIT_06245	3647,522472	4205,310633	5216,692626	4340,66105	3004,939342	4140,928932	2396,773787	2538,473758	4092,6758	2467,6238	0,6029365	-0,729922	0,2437001	0,6815893	poly(A) polymerase PAP, putative
PFIT_14247	21538,72208	11405,26413	15690,59467	15166,02509	8453,896015	20873,7812	8657,684872	8954,35476	15521,381	8806,0198	0,5673477	-0,817695	0,2438754	0,6815893	conserved Plasmodium protein, unknown function
PFIT_05187	284,1490194	188,3542841	239,3607323	222,4844756	290,1320744	192,4657109	392,6578298	351,801929	236,15772	372,22988	1,5761919	0,6564432	0,2439694	0,6815893	WD-repeat protein, putative
PFIT_bin05	767,5419727	97,03099484	79,78691076	107,921574	251,4477978	69,98753125	443,0909456	434,2999555	228,9528	438,69545	1,9160956	0,9381696	0,24431	0,6815893	cytoadherence linked asexual protein 2,cytoadherence linked asexual protein 8 (CLAG2
PFIT_14530	418,8650883	328,5101655	459,3980721	459,9119384	2043,358753	1460,989715	1337,678356	1181,52346	861,83896	1259,6009	1,461527	0,5474765	0,2445797	0,6815893	sortilin, putative
PFIT_09212	3,396203419	27,90433839	38,6467849	29,33253037	31,77637005	78,73597266	15,61025012	10,43078496	34,965367	13,020518	0,3723833	-1,42514	0,244931	0,6815893	conserved Plasmodium protein, unknown function
PFIT_05239	194,7156627	391,2949269	426,3613044	396,2658819	295,6583996	262,4532422	170,5119628	225,6842564	327,79157	198,09811	0,6043417	-0,726564	0,2449943	0,6815893	DEAD/DEAH box ATP-dependent RNA helicase, putative
PFIT_12316	21827,39937	17053,35589	19753,49376	14962,91115	12098,5075	14741,12377	9522,252571	10376,73453	16739,465	9949,4935	0,5943734	-0,750558	0,2450404	0,6815893	asparagine-rich protein, putative
PFIT_10003	5,660339031	10,78122165	10,59669908	12,17576732	5,526325227	29,16147135	2,401576941	4,74126589	12,316971	3,5714214	0,2899594	-1,786077	0,2451285	0,6815893	erythrocyte membrane protein 1 (PfEMP1), pseudogene
PFIT_09306	2007,156221	1126,954757	1290,303947	1312,769095	877,3041297	1425,995949	762,5006787	782,3088719	1340,0807	772,40478	0,5763868	-0,794891	0,2451796	0,6815893	conserved Plasmodium protein, unknown function
PFIT_08129	100,7540348	227,6740337	167,0538444	106,8146861	486,3166199	244,9563594	84,05519293	97,67007734	222,2616	90,862635	0,4088094	-1,2905	0,2451882	0,6815893	acyl-CoA binding protein, putative
PFIT_14181	14,71688148	33,61204397	25,55674485	16,04987511	23,48688221	17,49688281	54,03548117	30,3441017	21,819885	42,189791	1,9335478	0,9512505	0,2452768	0,6815893	conserved Plasmodium protein, unknown function
PFIT_bin04	135,8481368	11,41541116	13,09004005	9,961991446	5,526325227	20,41302995	60,03942352	82,49802649	32,709156	71,268725	2,1788616	1,1235746	0,2453097	0,6815893	B_ITvar33 - erythrocyte membrane protein 1, PfEMP1, putative
PFIT_14464	5181,474349	2842,437378	3032,525944	3394,825307	2633,29397	4514,195766	1864,824495	2151,586461	3599,7921	2008,2055	0,5578671	-0,842007	0,2455897	0,6815893	RNA helicase, putative
PFIT_14646	656,5993276	1164,371938	1354,507477	1236,947271	965,7253333	624,055487	618,4060623	641,9674015	1000,3678	630,18673	0,629955	-0,666679	0,245638	0,6815893	conserved Plasmodium protein, unknown function
PFIT_13289	3904,501864	1458,001681	1389,414251	1552,963778	1239,278432	1673,868456	985,8473342	954,8909503	1869,6714	970,36914	0,5190052	-0,946179	0,2456403	0,6815893	conserved Plasmodium protein, unknown function
PFIT_09244	11,32067806	95,76261583	77,2935698	132,8265526	194,8029642	75,81982552	156,1025012	164,0477998	97,971034	160,07515	1,6339028	0,7083222	0,2456877	0,6815893	thiamine pyrophosphokinase
PFIT_10380	6152,788527	2661,693368	4339,659943	4795,591993	2847,439073	4359,639967	2303,112286	2562,180087	4192,8021	2432,6462	0,5801958	-0,785388	0,2457144	0,6815893	RESA-like protein with PHIST and DnaJ domains
PFIT_11359	388,2992576	372,2692417	417,0112758	370,8074594	303,9478875	376,1829805	232,9529633	204,8226865	371,41968	218,88782	0,5893275	-0,762859	0,2459996	0,6819068	conserved Plasmodium protein, unknown function, pseudogene
Pf_M76611	2,264135613	12,04960067	9,973363845	21,03087083	107,7633419	58,32294271	62,44100046	55,9469375	35,234043	59,193969	1,6800221	0,7484802	0,24607	0,6819068	unspecified product
PFIT_12122	48,67891567	152,2054821	124,0437128	77,48215569	117,4344111	99,1490026	61,24021199	55,9469375	103,16561	58,593575	0,5679564	-0,816148	0,2462641	0,6821107	conserved protein, unknown function
PFIT_10139	753,957159	916,4038402	1186,206962	1131,239473	842,7645971	976,9092904	528,346927	626,7953507	967,91355	577,57114	0,5967177	-0,74488	0,2466663	0,6823254	MO15-related protein kinase
PFIT_03052	14596,88229	9381,565403	9626,166116	10194,99136	5776,391443	5482,356615	5715,753119	5585,211219	9176,3922	5650,4822	0,6157629	-0,699553	0,2467539	0,6823254	conserved Plasmodium membrane protein, unknown function
PFIT_11117	3946,388373	1761,778455	2205,983415	2598,972879	1141,186159	1029,399939	1296,851548	1227,987866	2113,9515	1262,4197	0,5971848	-0,743751	0,246782	0,6823254	ES2 protein, putative
PFIT_10318	19,24515271	42,49069709	49,24348398	40,95485372	147,8291998	14,58073568	109,2717508	84,39453284	52,390687	96,833142	1,8482892	0,8861905	0,2468832	0,6823254	N-acetylglucosaminyl-phosphatidylinositol biosynthetic protein, putative
PFIT_14390	528,6756655	672,2408793	1451,747775	1510,902036	697,6985599	1956,734728	581,1816197	672,3115032	1136,3333	626,74656	0,5515517	-0,858432	0,2469448	0,6823254	transport protein particle (TRAPP) component, Bet3
PFIT_14434	346,4127487	162,3525142	153,9638044	287,790864	227,9609156	107,897444	389,0554644	314,8200551	214,39638	351,93776	1,6415284	0,7150398	0,2470708	0,68234	GTP binding protein, putative
PFIT_07224	1031,313772	485,1549742	777,9223799	606,0211463	509,8035022	1023,567645	408,26808	407,7488665	738,9639	408,00847	0,5521359	-0,856905	0,2473541	0,6827891	immunoglobulin-binding protein 1-related, putative
Pf_M76611	33,96203419	4,439326561	6,233352403	6,087883661	48,35534573	43,74220703	31,22050023	33,18886123	23,803358	32,204681	1,3529469	0,4361053	0,2478516	0,6838285	unspecified product
PFIT_05082	13,58481368	45,02745512	54,23016591	41,50829769	161,6450129	116,6458854	124,8820009	92,92881145	72,106938	108,90541	1,5103319	0,5948656	0,2481229	0,6841623	longevity-assurance (LAG1) domain protein, putative
PFIT_08076	1289,425231	1191,007897	1506,601276	1265,172914	1131,51509	1446,408979	701,2604667	815,4977331	1305,0219	758,3791	0,5811237	-0,783083	0,2482468	0,6841623	peptidyl-prolyl cis-trans isomerase
PFIT_06153	56,60339031	78,63949909	74,17689359	67,52016424	70,46064664	49,5745013	37,22444258	36,03362076	66,162516	36,629032	0,5536221	-0,853027	0,2483354	0,6841623	conserved Plasmodium protein, unknown function
PFIT_13560	55,47132251	256,2125615	109,0836671	92,42514286	175,4608259	113,7297383	217,3427132	220,9429905	133,73054	219,14285	1,6386896	0,7125426	0,2485182	0,6842184	protein kinase 5
PFIT_11302	8626,356684	3332,665869	3778,658227	3333,393026	2818,425866	4345,059232	2291,104402	2521,4052	4372,4265	2406,2548	0,5503248	-0,861645	0,2485977	0,6842184	RNA methyltransferase, putative
PFIT_09156	100,7540348	58,97962432	69,81354691	69,18049615	55,26325227	72,90367838	43,22838494	33,18886123	71,149105	38,208623	0,5370218	-0,896947	0,2487992	0,6842322	conserved Plasmodium protein, unknown function
PFIT_11159	155,0932895	70,39503547	198,2206064	228,0189153	44,21060181	341,1892148	67,24415435	85,34278602	172,85461	76,29347	0,4413736	-1,179928	0,2488447	0,6842322	conserved Plasmodium protein, unknown function
PFIT_06120	0	7,610274105	11,22003433	6,087883661	34,53953267	2,916147135	25,21655788	18,01681038	10,395645	21,616684	2,079398	1,0561659	0,2490036	0,6843363	conserved Plasmodium protein, unknown function
PFIT_03084	875,0884143	827,617309	640,78627	587,2040513	523,6193152	729,0367838	1053,091489	1102,818446	697,22575	1077,955	1,546063	0,6285992	0,2495868	0,6852876	activator of Hsp90 ATPase, putative
PFIT_05015	20079,48668	13822,16034	20294,54875	33886,26735	15582,85556	39201,76594	31647,98093	38877,43205	23811,181	35262,706	1,4809306	0,566504	0,249592	0,6852876	interspersed repeat antigen
PFIT_09033	295,4696974	553,0132517	426,9846396	368,0402395	270,7899361	224,5433294	178,9174821	257,9248644	356,47352	218,42117	0,6127276	-0,706682	0,2499066	0,6855105	retrieval receptor for endoplasmic reticulum membrane proteins, putative
PFIT_11126	23,77342393	72,93179351	73,55355835	71,947716	146,4476185	40,8260599	130,8859433	108,1008623	71,580028	119,4934	1,6693679	0,7393019	0,2499888	0,6855105	peptidyl-prolyl cis-trans isomerase, putative
PFIT_01036	5,660339031	5,07351607	3,740011442	8,301659538	24,86846352	2,916147135	21,61419247	12,32729131	8,4266895	16,970742	2,0139275	1,0100118	0,2501472	0,6855105	conserved Plasmodium protein, unknown function
PFIT_10370	29,43376296	56,44286628	39,27012014	42,61518563	34,53953267	29,16147135	79,25203905	61,63645657	38,577157	70,444248	1,8260612	0,8687351	0,2502442	0,6855105	dynein intermediate chain, putative
PFIT_07212	71,3202718	247,9680979	273,6441705	294,4321916	233,4872408	207,0464466	142,893828	128,014179	221,3164	135,454	0,6120378	-0,708307	0,2502792	0,6855105	tRNAHis guanylyltransferase, putative
PFIT_09348	133,5840011	1300,722683	879,5260241	675,7550864	896,646268	279,950125	329,0160409	407,7488665	694,36403	368,38245	0,5305322	-0,914488	0,2505454	0,6857466	conserved Plasmodium protein, unknown function
PFIT_04131	283,0169516	389,3923584	352,807746	320,9975021	211,3819399	277,0339779	164,5080205	201,0296737	305,77175	182,76885	0,5977297	-0,742435	0,2506079	0,6857466	RNA binding protein, putative
PF_apicopla	3,396203419	24,09920133	14,96004577	9,408547476	87,03962232	14,58073568	52,8346927	45,51615254	25,580726	49,175423	1,9223623	0,9428802	0,2509885	0,6857615	apicoplast ribosomal protein L16
PFIT_04096	16562,15201	13419,45001	15908,13867	12445,29454	10844,03168	24026,13625	7580,577614	9589,684389	15534,201	8585,131	0,55266	-0,855536	0,2509927	0,6857615	conserved Plasmodium protein, unknown function
PFIT_13584	4715,062413	2354,111457	1965,999348	1734,493399	1682,766031	2038,386848	1307,658644	1446,086096	2415,1366	1376,8724	0,5701012	-0,81071	0,2511404	0,6857615	mitotic control protein dis3 homologue, putative
PFIT_05061	585,2790558	828,2514985	949,339571	964,6528383	620,3300067	501,5773073	422,6775416	504,4706907	741,57171	463,57412	0,6251238	-0,677786	0,251138	0,6857615	60S ribosomal subunit protein L24-2, putative
PFIT_14446	112,0747128	197,2329372	133,3937414	147,7695398	469,7376443	317,8600378	345,8270795	293,9584852	229,6781	319,89278	1,3927875	0,4779751	0,2513393	0,6857615	conserved Plasmodium protein, unknown function
PFIT_06061	40,75444103	32,97785446	23,68673913	26,01186655	51,11850835	14,58073568	57,63784658	50,25741843	31,521691	53,947633	1,7114448	0,7752147	0,2513408	0,6857615	RING zinc finger protein, putative
PFIT_08320	2693,189311	1228,425079	1800,192174	1894,992151	1262,765314	1720,52681	955,8276225	1098,07718	1766,6818	1026,9524	0,5812888	-0,782673	0,2518038	0,6862303	conserved protein, unknown function
PFIT_14536	2568,661852	2206,979491	1783,362122	1617,716722	1428,555071	1880,914902	1147,953778	1110,404471	1914,365	1129,1791	0,5898453	-0,761592	0,2518948	0,6862303	conserved Plasmodium protein, unknown function
PFIT_05219	196,9797983	428,0779184	441,9446854	399,0331018	345,3953267	285,7824193	213,7403477	216,2017246	349,53554	214,97104	0,6150191	-0,701297	0,2519661	0,6862303	DEAD/DEAH box ATP-dependent RNA helicase, putative
PFIT_08094	3393,939283	3645,955486	3940,102054	5259,931483	2623,622901	3207,761849	6035,162853	6061,234314	3678,5522	6048,1986	1,6441791	0,7173675	0,2519979	0,6862303	DnaJ protein, putative
PFIT_12534	333,9600029	147,7661555	146,4837835	157,7315312	120,1975737	113,7297383	84,05519293	106,2043559	169,97813	95,129774	0,5596589	-0,83738	0,2525965	0,6872671	Plasmodium exported protein, unknown function
PFIT_14398	156,2253573	654,4835731	848,359262	821,3108503	538,8167096	312,0277435	277,3821367	421,9726642	555,20392	349,6774	0,629818	-0,666993	0,2526217	0,6872671	conserved protein, unknown function
PFIT_09306	2112,438527	1215,741288	1389,414251	1365,346272	945,0016137	1466,822009	884,9811027	781,3606187	1415,794	833,17086	0,5884831	-0,764927	0,2528597	0,6874969	conserved Plasmodium protein, unknown function
PFIT_11415	1587,159064	1580,400256	1801,438844	2096,999199	1425,791908	1571,803306	999,0560074	1040,233736	1677,2654	1019,6449	0,607921	-0,718064	0,2529493	0,6874969	conserved Plasmodium protein, unknown function
PFIT_03163	48,67891567	129,3746598	71,68355263	75,82182378	723,9486047	75,81982552	403,4649261	355,5949418	187,55456	379,52993	2,0235708	1,0169033	0,253086	0,687538	inorganic pyrophosphatase, putative
PFIT_10035	208,3004764	179,475631	291,0975572	185,4037297	41,4474392	58,32294271	103,2678085	85,34278602	160,67463	94,305297	0,5869333	-0,768731	0,2533297	0,6878699	conserved Plasmodium protein, unknown function
PFIT_14169	343,0165453	572,0389369	661,9820252	801,3868674	410,3296481	551,1518086	321,8113101	366,0257267	556,65097	343,91852	0,6178351	-0,694706	0,2534878	0,6879687	conserved Plasmodium protein, unknown function
PFIT_13446	1409,424419	412,2231807	283,6175343	342,5818169	592,6983806	341,1892148	883,7803143	885,6684683	563,62242	884,72439	1,5697111	0,6504991	0,2539564	0,6889099	conserved Plasmodium protein, unknown function
PFIT_09157	607,920412	869,4738165	1166,88357	1293,398556	562,3035918	1565,971012	666,4376011	548,0903369	1010,9918	607,26397	0,6006616	-0,735376	0,2544448	0,689041	conserved Plasmodium protein, unknown function
PFIT_11357	1,132067806	4,439326561	5,610017163	3,320663815	12,43423176	5,832294271	12,0078847	11,37903814	5,4614336	11,693461	2,1410974	1,0983505	0,2545782	0,689935	conserved Plasmodium protein, unknown function
PFIT_03104	3295,449384	3118,309815	3058,706024	2361,545417	2591,846531	3554,783358	1723,131455	1745,734101	2996,7734	1734,4328	0,5787667	-0,788946	0,2548021	0,6900016	DEAD box helicase, putative
PFIT_07240	31,69789858	147,7661555	152,0937986	80,24937553	23,48688221	72,90367838	44,42917341	44,56789937	84,699631	44,498536	0,5253687	-0,928598	0,254871	0,6900016	unspecified product
PFIT_04187	1897,345643	1626,69609	1686,121825	1727,852072	1365,002331	1685,533044	960,6307764	1015,579154	1664,7585	988,10497	0,5935425	-0,752577	0,2549688	0,6900016	RIO-like serine/threonine kinase (RIO2)
PFIT_14499	633,9579715	365,9273466	480,5914703	579,4558357	479,4087134	484,0804245	286,9884444	299,6480043	503,90363	293,31822	0,5820919	-0,780681	0,2553084	0,6901005	conserved Plasmodium protein, unknown function
PFIT_13018	697,3537687	1710,409105	1924,235887	1727,852072	1566,713202	1201,45262	796,1227559	1022,216926	1471,1361	909,16984	0,6179212	-0,694505	0,2555449	0,6901005	deoxyhypusine hydroxylase
PFIT_10368	1164,897773	2123,266475	3367,880303	3525,991528	2379,08301	3796,82357	1568,229742	1686,942404	2726,3238	1627,5861	0,5969893	-0,744223	0,2555833	0,6901005	DNA repair helicase rad25, putative
PFIT_14403	1814,704693	2501,877612	2960,842391	2703,020346	2098,622005	2755,759043	1481,772973	1510,567313	2472,471	1496,1701	0,6051315	-0,724679	0,2556488	0,6901005	DEAD/DEAH box helicase, putative
PFIT_10120	923,7673299	1304,52782	1350,14413	1255,210922	989,2122156	1504,731922	682,0478512	762,3955551	1221,2657	722,2217	0,5913715	-0,757863	0,2558255	0,6901005	phosphoglucomutase, putative
PFIT_05329	863,7677362	1422,487068	2010,25615	2204,367329	1273,817965	2000,476935	934,21343	1046,871509	1629,1955	990,54247	0,6079948	-0,717869	0,2558974	0,6901005	peptidyl-prolyl cis-trans isomerase
PFIT_14649	0	10,78122165	5,610017163	12,72921129	15,19739437	8,748441406	32,4212887	7,586025424	8,844381	20,003657	2,2617362	1,1774307	0,2560228	0,6901005	mitochondrial ribosomal protein S6-2 precursor, putative
PFIT_12143	6,792406838	65,95570891	57,97017735	24,90497861	60,78957749	40,8260599	14,40946165	28,44759534	42,873152	21,428528	0,4998123	-1,000542	0,2563027	0,6901005	conserved Plasmodium protein, unknown function
PFIT_04177	71,3202718	51,36935021	46,75014302	44,27551754	56,64483357	87,48441406	32,4212887	26,55108898	59,640755	29,486189	0,4943966	-1,016259	0,2565302	0,6901005	conserved Plasmodium protein, unknown function
PFIT_14311	672,4482769	625,9450452	612,7385412	588,8643832	805,4619018	571,5648385	983,4457573	993,7693306	646,1705	988,60754	1,529484	0,613483	0,2565756	0,6901005	ATP-dependent DNA helicase, putative
PFIT_13609	18,1130849	153,4738611	167,6771796	160,4987511	788,8829261	139,9750625	475,5122343	422,9209174	238,10348	449,21658	1,8866443	0,9158225	0,2567392	0,6901005	proliferating cell nuclear antigen
PFIT_14732	1464,895741	1103,489745	1009,803089	938,0875278	826,1856214	635,7200755	591,9887159	615,4163125	996,36363	603,70251	0,6059058	-0,722835	0,2567989	0,6901005	Yip1 protein, putative
PFIT_07205	4356,196919	2033,211565	2262,706922	2547,50259	1358,094424	1283,10474	1355,690183	1456,516881	2306,8029	1406,1035	0,6095465	-0,714192	0,2568101	0,6901005	ubiquitin carboxyl-terminal hydrolase, putative
PFIT_06004	66,79200057	93,22585779	77,2935698	90,21136698	196,1845455	209,9625937	193,3269437	149,8240021	122,27832	171,57547	1,4031553	0,4886747	0,2568219	0,6901005	Tetratricopeptide repeat protein, putative
PFIT_14448	249,0549174	98,29937386	64,82686499	95,74580667	292,895237	46,65835417	230,5513863	207,667446	141,24676	219,10942	1,5512527	0,6334337	0,2568355	0,6901005	conserved Plasmodium protein, unknown function
PFIT_14653	1095,841636	777,5163378	1009,179754	1512,562368	424,1454611	621,1393398	610,000543	562,3141346	906,73082	586,15734	0,6464513	-0,629386	0,2580415	0,6930117	zinc finger protein, putative
PFIT_07049	5149,776451	6235,35125	7121,60512	7119,50322	4608,955239	4490,865589	3562,739392	3728,531496	5787,6763	3645,6354	0,6298962	-0,666814	0,2583486	0,6933319	conserved Plasmodium protein, unknown function
PFIT_04227	15298,76433	8609,756771	11930,6365	12067,84575	6800,143191	7963,997827	6047,170737	6727,856298	10445,191	6387,5135	0,6115267	-0,709512	0,2584694	0,6933319	serine/threonine protein kinase, FIKK family
PFIT_13089	2167,909849	2163,220414	2910,352237	2424,638029	2192,569534	4327,562349	1445,749318	1592,117086	2697,7087	1518,9332	0,5630457	-0,828676	0,2585755	0,6933319	conserved Plasmodium protein, unknown function
PFIT_11393	588,6752593	771,1744427	773,5590332	811,3488588	599,6062871	478,2481302	408,268608	428,6104365	670,43694	418,43926	0,6241307	-0,68008	0,2586511	0,6933319	beta-catenin-like protein 1, putative
PFIT_09050	190,1873915	377,3427577	389,5845252	361,9523559	1008,554354	679,4622825	730,07939	666,6219841	501,18061	698,35069	1,3934112	0,4786211	0,2590602	0,6940997	replication protein A1, small fragment
PFIT_09028	1410,556487	1251,89009	932,5095195	766,5198973	686,6459094	1568,887159	558,3666388	673,2597564	1102,8348	615,8132	0,5583911	-0,840652	0,2592118	0,694177	serine/threonine protein kinase, FIKK family
PFIT_11154	26,03755954	21,5624433	19,32339245	16,60331908	8,28948784	5,832294271	27,61813482	36,03362076	16,274749	31,825878	1,9555372	0,967565	0,2594246	0,6944181	conserved Plasmodium protein, unknown function
PFIT_10010	10129,74273	11516,88148	12495,37823	10359,91766	9369,884422	15814,26592	6126,422776	7299,652964	11614,345	6713,0379	0,5779954	-0,79087	0,2595656	0,6944668	alpha/beta hydrolase, putative
PFIT_04213	95,09369573	176,3046834	177,0272082	140,5747682	168,5529194	69,98753125	81,65361599	85,34278602	137,92347	83,498201	0,6053952	-0,724051	0,2597755	0,6946998	clathrin assembly protein, putative
PFIT_03158	550,1849539	639,8972144	875,1626774	821,8642943	573,3562423	530,7387786	407,0672915	422,9209174	665,20069	414,9941	0,623863	-0,680699	0,2603991	0,6960385	zinc finger protein, putative
PFIT_13546	1394,707537	1760,510076	1793,958822	1676,381783	1465,857766	1122,716647	880,1779488	1018,423913	1535,6888	949,30093	0,6181597	-0,693948	0,2606599	0,6964064	serine/threonine protein phosphatase
PFIT_10038	1629,045573	1115,539346	863,942643	797,5127596	2549,017511	1907,160227	2204,647632	1794,095013	1477,0363	1999,3713	1,3536372	0,4368411	0,2608227	0,6965124	conserved Plasmodium membrane protein, unknown function
PFIT_04129	127,9236621	93,22585779	61,08685355	75,82182378	87,03962232	37,90991276	38,42523105	50,25741843	80,501289	44,341325	0,5508151	-0,86036	0,261402	0,69773	Rab5-interacting protein, putative
PFIT_10019	123,3953909	91,95747877	76,04689932	63,09261249	56,64483357	87,48441406	49,23232729	41,72313983	83,103605	45,477734	0,5472414	-0,869751	0,2620943	0,6989301	conserved Plasmodium protein, unknown function
PFIT_13212	44,15064444	94,49423681	79,78691076	161,052195	483,5534573	58,32294271	308,6026369	268,3556494	153,56006	288,47914	1,8786079	0,9096639	0,2620988	0,6989301	adenosine-diphosphatase, putative
PFIT_13051	130,1877977	145,2293975	112,8236785	100,7268024	203,0924521	201,2141523	211,3387708	217,1499778	148,87905	214,24437	1,4390499	0,5251166	0,2623638	0,6992062	conserved Plasmodium protein, unknown function
PFIT_07148	28,30169516	86,8839627	109,0836671	71,947716	118,8159924	107,897444	148,8977703	127,0659259	87,15508	137,98185	1,5831762	0,6628218	0,2625535	0,6992062	conserved Plasmodium protein, unknown function
PFIT_02021	0	8,244463614	3,116676201	2,767219846	23,48688221	0	13,20867318	14,22379767	6,269207	13,716235	2,1878741	1,1295297	0,2625732	0,6992062	conserved Plasmodium protein, unknown function
PFIT_13394	4,528271225	6,976084597	8,103358124	6,087883661	12,43423176	2,916147135	1,20078847	1,896506356	6,8409961	1,5486474	0,2263775	-2,143198	0,2630272	0,7000855	conserved Plasmodium protein, unknown function
PFIT_11471	6,792406838	9,512842632	8,726693364	9,408547476	15,19739437	8,748441406	24,01576941	15,17205085	9,7310543	19,59391	2,0135444	1,0097373	0,2635733	0,701209	leucine-rich repeat protein
PFIT_07280	1015,464822	255,578372	175,7805378	193,1519452	420,0007172	358,6860977	614,8036969	601,1925149	403,11042	607,99811	1,5082669	0,5928918	0,2640071	0,7020329	conserved Plasmodium protein, unknown function
PFIT_13409	1,132067806	3,170947544	1,246670481	3,320663815	0	0	3,602365411	8,534278602	1,4783916	6,068322	4,1046783	2,0372692	0,2646019	0,7030275	conserved Plasmodium protein, unknown function
PFIT_bin07	5,660339031	13,31797968	12,46670481	23,79809068	6,907906533	37,90991276	27,61813482	29,39584852	16,676822	28,506992	1,7093779	0,7734714	0,2646297	0,7030275	rifin
PFIT_11213	1456,971267	1856,906882	1954,155978	2125,224842	1569,476364	1557,22257	1053,091489	1123,680016	1753,3263	1088,3858	0,6207548	-0,687905	0,2650547	0,7038261	tRNA nucleotidyltransferase, putative
PFIT_09112	5,660339031	26,00176986	21,81673341	48,14962532	56,64483357	2,916147135	44,42917341	54,99868432	26,864908	49,713929	1,8505155	0,8879272	0,265261	0,7039574	phosphatidylinositol N-acetylglucosaminyltransferase, putative
PFIT_03163	95,09369573	165,5234618	88,51360412	99,06647049	940,8568698	137,0589154	510,3350999	453,2650191	254,35217	481,80006	1,8942243	0,9216072	0,2654634	0,7039574	inorganic pyrophosphatase, putative
PFIT_14560	39,62237322	49,46678168	39,89345538	44,8289615	8,28948784	14,58073568	56,43705811	67,32597564	32,780299	61,881517	1,8877655	0,9166795	0,2655743	0,7039574	conserved Plasmodium protein, unknown function
PFIT_13440	1315,462791	696,3400806	769,1956865	786,4438802	577,5009862	743,6175195	463,5043496	496,8846653	814,76016	480,19451	0,5893692	-0,762757	0,2656888	0,7039574	aspartate carbamoyltransferase
PFIT_13337	0	5,707705579	2,493340961	4,427551754	20,7237196	2,916147135	8,405519293	15,17205085	6,0447442	11,788785	1,9502538	0,9636619	0,2657951	0,7039574	MSP7-like protein
PFIT_14391	5579,962217	5250,454943	5429,249943	4720,323613	4607,573658	7287,451691	2943,132541	3375,781314	5479,1693	3159,4569	0,5766306	-0,794281	0,2660622	0,7039574	HSP40, subfamily A, putative
PFIT_11199	1781,874727	438,8591401	683,1754234	603,2539264	317,7637005	813,6050508	330,2168294	328,0955996	773,08866	329,15621	0,4257677	-1,231861	0,2661415	0,7039574	tRNA m(1)G methyltransferase, putative
PFIT_12445	31,69789858	83,71301516	44,8801373	37,63418991	531,9088031	402,4283047	20,413404	20,86156992	188,71039	20,637487	0,1093606	-3,192835	0,2661587	0,7039574	ACEA small nucleolar RNA U3
PFIT_04197	3,396203419	6,976084597	7,480022884	5,534439692	8,28948784	11,66458854	1,20078847	1,896506356	7,2234712	1,5486474	0,214391	-2,221684	0,2662244	0,7039574	rifin, pseudogene
PFIT_03182	18839,87243	11341,84518	15689,97133	15934,75876	13558,83894	22206,46044	8521,995775	10012,60531	16261,958	9267,3005	0,5698761	-0,81128	0,2667904	0,705056	DNA-directed RNA polymerase II, putative
PFIT_13667	6210,523985	8487,992386	8080,29472	7282,215747	6030,602404	8223,534922	4201,558858	4740,317637	7385,8607	4470,9382	0,6053375	-0,724188	0,2669214	0,705056	alanine--tRNA ligase (AlaRS)
PFIT_14780	54,3392547	27,27014888	29,29675629	26,56531052	12,43423176	8,748441406	13,20867318	13,27554449	26,442357	13,242109	0,5007915	-0,997718	0,2671674	0,705056	Plasmodium exported protein, unknown function, pseudogene
PFIT_14526	1156,973298	1084,46406	1050,943215	942,5150795	1029,278073	1213,117208	620,8076392	643,8639079	1079,5485	632,33577	0,585741	-0,771665	0,2672677	0,705056	transcriptional regulatory protein sir2b
PFIT_08217	2327,53141	1771,925488	2354,337203	2811,495364	1152,23881	1408,499066	1242,816067	1284,883056	1971,0046	1263,8496	0,641221	-0,641106	0,2678796	0,705056	U3 small nucleolar ribonucleoprotein protein, putative
PFIT_09072	207,1684085	407,1496646	522,9782666	524,6648828	339,8690014	387,847569	259,3703096	242,7528136	398,27963	251,06156	0,6303651	-0,665741	0,2681593	0,705056	conserved Plasmodium protein, unknown function
PFIT_08134	2049,042729	1139,004358	1115,146745	956,3511788	1012,699098	1414,331361	702,4612552	759,5507956	1281,0959	731,00603	0,5706099	-0,809423	0,2682144	0,705056	protein phosphatase, putative
PFIT_14457	5388,642758	3239,440011	2529,494405	3154,630624	2088,950936	1749,688281	1796,379552	1923,057445	3025,142	1859,7185	0,6147543	-0,701918	0,2682627	0,705056	eukaryotic initiation factor 2alpha kinase 1
PFIT_10330	6409,767919	3604,098978	4792,201327	5741,98118	3152,768542	5036,186103	2849,47104	2935,791839	4789,5007	2892,6314	0,6039526	-0,727493	0,2682951	0,705056	myb2 transcription factor, putative
PFIT_08137	549,052886	515,5960706	468,1247655	355,3110282	548,4877787	1592,216336	247,3624249	249,3905858	671,46481	248,37651	0,3699025	-1,434783	0,268421	0,705056	dihydropteroate synthetase
PFIT_11334	3184,506739	1221,448994	1907,405835	1738,920951	1269,673221	2776,172073	1194,784528	1094,284167	2016,3546	1144,5343	0,5676255	-0,816989	0,2684506	0,705056	pre-mRNA splicing factor, putative
PFIT_13426	1040,370314	2130,87675	3020,059239	2970,333783	2229,872229	2245,433294	1442,146953	1429,965792	2272,8243	1436,0564	0,6318378	-0,662374	0,2685113	0,705056	DNA repair protein RAD5, putative (RAD5)
PFIT_08216	9518,426115	5841,519565	7636,480029	7118,949776	4433,494413	7287,451691	4067,070549	4320,241479	6972,7203	4193,656	0,6014376	-0,733513	0,268623	0,705056	DNA helicase, putative
PFIT_05282	32818,6457	28855,62265	30047,25192	29387,87476	22577,80171	29464,75066	16149,40414	18632,22669	28858,658	17390,815	0,6026204	-0,730679	0,2687297	0,705056	karyopherin beta
PFIT_14198	10364,08077	21071,58062	10643,44923	11206,13349	37094,0765	14026,66772	24742,24643	25296,55003	17400,998	25019,398	1,437814	0,523877	0,268833	0,705056	signal recognition particle RNA
PFIT_12305	2575,454259	1314,040662	1359,494159	1673,061119	899,4094306	2644,945452	993,0520651	981,4420392	1744,4008	987,24705	0,5659519	-0,821249	0,2690808	0,705056	conserved Plasmodium protein, unknown function
PFIT_12391	14,71688148	2,536758035	4,363346682	3,874107784	0	5,832294271	7,204730823	14,22379767	5,2205647	10,714264	2,052319	1,037255	0,2691734	0,705056	conserved Plasmodium protein, unknown function
PFIT_08119	126686,312	49570,78876	55997,94465	35953,38057	39271,44864	154826,9999	24975,1994	34116,25284	77051,146	29545,726	0,383456	-1,382867	0,2691829	0,705056	erythrocyte membrane protein 1, PfEMP1
PFIT_14065	237,7342393	421,1018338	515,4982437	370,8074594	367,5006276	384,9314219	236,5553287	232,3220286	382,92897	234,43868	0,612225	-0,707866	0,2694196	0,705056	CDBW domain-containing protein 1, putative (CBWD1)
PFIT_12217	324,9034604	157,2789982	281,7475286	230,7861352	187,8950577	431,589776	144,0946165	149,8240021	269,03349	146,95931	0,5462491	-0,872369	0,2696182	0,705056	conserved protein, unknown function
PFIT_10314	675,8444803	178,8414415	335,3543593	339,2611531	301,1847248	501,5773073	252,1655788	183,0128634	388,67724	217,58922	0,5598198	-0,836966	0,2696346	0,705056	mitochondrial preribosomal assembly protein rimM precursor, putative
PFIT_13024	1,132067806	8,244463614	5,610017163	4,980995723	13,81581307	5,832294271	18,01182706	10,43078496	6,6026086	14,221306	2,153892	1,1069459	0,269636	0,705056	SNARE protein, putative
PFIT_10241	30,56583077	49,46678168	29,29675629	13,28265526	37,30209528	23,32917708	50,43311576	52,15392479	30,540649	51,29352	1,6795164	0,7480458	0,2697247	0,705056	RAP protein, putative
PFIT_01051	76,98061083	26,63595937	14,33671053	24,90497861	12,43423176	8,748441406	56,43705811	44,56789937	27,340155	50,502479	1,8471906	0,8853328	0,2697412	0,705056	conserved Plasmodium protein, unknown function
PFIT_11388	31,69789858	151,5712926	124,6670481	116,7766775	95,32911016	26,24532422	34,82286564	71,11898835	91,04076	52,970927	0,5817919	-0,781425	0,2697562	0,705056	ubiquitin related modifier homologue
PFIT_13277	209,4325442	126,8379018	158,9504863	131,1662207	107,7633419	250,7886536	236,5553287	239,908054	164,15652	238,23169	1,4512472	0,5372933	0,2703699	0,7060253	conserved Plasmodium protein, unknown function
PFIT_07076	182,2629168	257,4809406	402,6745652	317,1233943	404,8033228	615,3070456	547,5595425	496,8846653	363,27536	522,2221	1,4375379	0,5236	0,2706743	0,7060253	Cg8 protein
PFIT_bin08	139,2443402	39,95393905	41,14012586	33,76008212	13,81581307	23,32917708	84,05519293	90,08405191	48,54058	87,069622	1,7937491	0,8429781	0,2707911	0,7060253	erythrocyte membrane protein 1, PfEMP1
PFIT_14180	56,60339031	25,36758035	29,92009153	32,65319418	16,57897568	8,748441406	49,23232729	52,15392479	28,311946	50,693126	1,7905208	0,8403792	0,2708741	0,7060253	conserved Plasmodium protein, unknown function
PFIT_14624	392,8275288	594,2355697	663,2286957	706,1945047	585,790476	769,8628437	353,0318103	385,9390435	618,68994	369,48543	0,5972061	-0,743699	0,2708746	0,7060253	U3 small nucleolar ribonucleoprotein IMP3, putative (IMP3)
PFIT_13185	2619,604904	3285,101655	4481,157042	4832,672739	2709,280942	4146,761227	2159,01767	2410,459579	3679,0964	2284,7386	0,6210054	-0,687322	0,2708761	0,7060253	N2,N2-dimethylguanosine tRNA methyltransferase, putative
PFIT_08344	5071,663772	4939,702084	6768,174039	7447,695493	4629,678959	5683,570767	3532,71968	3652,671242	5756,745	3592,6955	0,6240843	-0,680187	0,2711955	0,7062506	Plasmodium exported protein (PHIST), unknown function
PFIT_14474	16778,37696	9473,522882	10599,19243	7653,023206	8026,987392	25953,70951	5667,721581	6726,908045	13080,802	6197,3148	0,4737718	-1,077736	0,2712122	0,7062506	M17 leucyl aminopeptidase
PFIT_05140	30,56583077	16,48892723	11,22003433	11,06887938	53,88167096	17,49688281	40,826808	31,29235487	23,453704	36,059581	1,5374792	0,6205669	0,2716622	0,7070968	conserved Plasmodium protein, unknown function
PFIT_14132	424,5254274	433,785624	570,3517449	721,1374919	371,6453715	594,8940156	313,4057908	324,3025869	519,38995	318,85419	0,6139013	-0,703921	0,2718022	0,7071359	conserved Plasmodium protein, unknown function
PFIT_13202	101,8861026	29,80690691	32,4134325	31,54630624	29,01320744	46,65835417	18,01182706	21,80982309	45,220718	19,910825	0,4403032	-1,183431	0,2722771	0,7077117	conserved Plasmodium protein, unknown function
PFIT_14663	892,0694313	810,4941922	1063,40992	1207,614741	759,8697187	1175,207296	563,1692606	623,9505911	984,77755	593,56019	0,6027353	-0,730404	0,2722856	0,7077117	conserved Plasmodium protein, unknown function
PFIT_05104	298,8659009	409,6864227	467,5014302	470,4273738	254,2109604	306,1954492	208,9371939	253,1835985	367,81459	231,0604	0,628198	-0,670709	0,272449	0,7077117	conserved Plasmodium protein, unknown function
PFIT_11262	972,4462456	896,1097759	820,3091762	630,3726809	802,6987392	1994,644641	524,7445616	553,779856	1019,432	539,26221	0,5289839	-0,918704	0,2726147	0,7077117	DNA-directed RNA polymerase (mtRNAP)
PFIT_10094	1024,521365	643,7023514	664,4753662	770,3940051	613,4221002	504,4934544	380,6499451	467,4888168	703,50144	424,06938	0,6027982	-0,730253	0,2726492	0,7077117	conserved Plasmodium protein, unknown function
PFIT_12414	2296,965579	2250,104377	2219,073455	2220,970648	1863,753183	2665,358482	1292,048394	1392,035665	2252,7043	1342,042	0,5957471	-0,747228	0,2727856	0,7077409	conserved Plasmodium protein, unknown function
PFIT_03129	978,1065846	851,0823208	1027,879811	986,2371531	822,0408775	1020,651497	567,9729465	573,6931727	947,66637	570,83306	0,6023566	-0,73131	0,273158	0,708387	conserved Plasmodium protein, unknown function
PFIT_07316	0	10,14703214	19,32339245	16,04987511	0	14,58073568	3,602365411	3,793012712	10,016839	3,6976891	0,3691473	-1,437732	0,2735492	0,7090717	rifin (RIF)
PFIT_12463	14,71688148	98,29937386	78,54024028	91,31825492	96,71069147	46,65835417	111,6733278	129,9106854	71,040633	120,79201	1,7003228	0,7658087	0,2738774	0,709597	delta-aminolevulinic acid synthetase
PFIT_12241	577,3545812	1619,720005	1482,291201	1623,251162	1284,870615	1714,694516	827,3432561	875,2376833	1383,697	851,29047	0,615229	-0,700805	0,2742292	0,7100212	conserved Plasmodium protein, unknown function
PFIT_01014	3153,940908	3246,416095	3430,837163	2623,324414	3080,926314	5517,35038	1904,450514	2064,347169	3508,792	1984,3988	0,5655493	-0,822275	0,2742921	0,7100212	aspartate--tRNA ligase
PFIT_02191	1884,892897	3148,116722	3144,726287	3870,233677	1977,147285	679,4622825	1586,241569	1757,113139	2450,7458	1671,6774	0,6821097	-0,551924	0,274431	0,7100557	Plasmodium exported protein (PHISTc), unknown function
PFIT_08204	1332,443808	2567,833321	2385,503965	1830,79265	1787,766211	4625,009357	3359,80614	3454,486328	2421,5582	3407,1462	1,4070057	0,4926282	0,2746527	0,7101101	protein kinase c inhibitor-like protein, putative
PFIT_02093	698,4858365	500,3755224	632,0619337	378,5556749	540,1982909	927,3347891	324,212887	352,7501822	612,83534	338,48153	0,5523205	-0,856422	0,2748021	0,7101101	secretory complex protein 61 gamma subunit
PFIT_10086	44,15064444	47,56421316	45,5034724	39,29452181	17,96055699	17,49688281	13,20867318	24,65458263	35,328382	18,931628	0,5358759	-0,900029	0,2749945	0,7101101	conserved Plasmodium membrane protein, unknown function
PFIT_14762	2308,286257	2537,392225	1880,60242	1324,944862	2039,214009	4164,258109	1332,875202	1278,245284	2375,783	1305,5602	0,5495284	-0,863734	0,2750511	0,7101101	conserved Plasmodium protein, unknown function
PFIT_09228	2409,040292	1123,78381	1213,010378	1302,25366	1094,212395	2085,045202	864,5676987	872,3929238	1537,891	868,48031	0,5647216	-0,824388	0,2750798	0,7101101	pre-mRNA splicing factor, putative
PFIT_12235	765,277837	1394,58273	1457,357792	1097,479391	1260,002152	2070,464466	704,8628322	839,2040625	1340,8607	772,03345	0,5757745	-0,796424	0,2753183	0,7104016	mitochondrial carrier protein, putative
PFIT_13043	59,99959373	65,95570891	41,7634611	53,13062104	273,5530987	49,5745013	146,4961934	155,5135212	90,662831	151,00486	1,6655652	0,7360118	0,275613	0,710606	conserved Plasmodium protein, unknown function
PFIT_09074	6022,600729	3247,050285	5415,536568	5395,525256	2960,7274	4846,636539	2938,329387	2780,278318	4648,013	2859,3039	0,6151669	-0,70095	0,275648	0,710606	GTPase activator, putative
PFIT_14113	6653,162498	3911,68089	5040,912088	4424,784534	3191,452818	3860,978807	2770,219001	2768,89928	4513,828	2769,559	0,6135721	-0,704695	0,2759969	0,7111794	conserved Plasmodium protein, unknown function
PFIT_13320	935,088008	1917,789075	2531,987746	2741,761423	1543,22632	2073,380613	1161,162451	1328,502702	1957,2055	1244,8326	0,6360255	-0,652864	0,2763422	0,7112774	DEAD/DEAH box ATP-dependent RNA helicase, putative
PFIT_14670	12123,31414	12712,3287	13472,76788	10840,30702	14387,78773	26933,53494	7791,916385	8752,376833	15078,34	8272,1466	0,5486112	-0,866144	0,2765397	0,7112774	40S ribosomal protein S3, putative
PFIT_14628	8103,341357	3493,750004	4134,582649	4233,846364	2902,702325	3974,708546	2440,002172	2867,51761	4473,8219	2653,7599	0,5931751	-0,75347	0,276801	0,7112774	splicing factor 3B subunit 2, putative
PFIT_13419	96,22576353	383,6846528	453,1647197	437,2207357	480,7902947	443,2543646	220,9450786	220,8563072	382,39009	230,90069	0,6038355	-0,727773	0,2768264	0,7112774	transcription activator, putative
PFIT_07275	676,9765482	461,6899624	295,4609039	317,1233943	549,86936	309,1115964	682,0478512	607,8302871	435,03863	644,93907	1,4824869	0,5680194	0,2768851	0,7112774	zinc finger, C3HC4 type, putative
PFIT_10096	174,3384422	482,6182162	410,1545881	584,4368315	338,4874201	236,207918	241,3584826	247,4940795	371,04057	244,42628	0,6587589	-0,602178	0,2771787	0,7112774	conserved Plasmodium protein, unknown function
PFIT_10073	717,7309892	920,2089772	1042,216522	1071,467524	2329,346083	3333,156176	2249,076805	1939,177749	1569,021	2094,1273	1,3346712	0,4164844	0,2771842	0,7112774	transcription factor with AP2 domain(s)
PFIT_13414	153,9612217	72,93179351	72,93022311	69,73394012	89,80278493	157,4719453	56,43705811	49,30916526	102,80532	52,873112	0,5143033	-0,959309	0,2772506	0,7112774	conserved Plasmodium membrane protein, unknown function
PFIT_11316	28,30169516	16,48892723	11,22003433	9,408547476	11,05265045	14,58073568	8,405519293	3,793012712	15,175432	6,099266	0,4019171	-1,31503	0,2773906	0,7112774	armadillo repeat protein PF16, putative
PFIT_02027	10,18861026	19,65987477	18,07672197	18,26365098	132,6318054	5,832294271	80,45282752	59,73995021	34,108826	70,096389	2,0550806	1,039195	0,2774845	0,7112774	conserved Plasmodium protein, unknown function
PFIT_11149	135,8481368	508,619986	350,9377403	374,6815671	323,2900258	323,692332	507,933523	569,90016	336,1783	538,91684	1,6030685	0,6808361	0,2775556	0,7112774	rhomboid protease ROM1
PFIT_08057	45,28271225	35,51461249	187,6239073	126,7386689	284,6057492	110,8135911	224,547444	189,6506356	131,76321	207,09904	1,5717517	0,6523733	0,277666	0,7112774	conserved Plasmodium protein, unknown function
PFIT_13415	23,77342393	11,41541116	11,22003433	17,15676305	45,59218312	32,07761849	32,4212887	36,03362076	23,539239	34,227455	1,4540595	0,5400863	0,277741	0,7112774	tetratricopeptide repeat family protein, putative
PFIT_03239	4430,913394	2744,138004	3595,397666	2824,778019	2235,398554	3586,860977	2001,71438	1887,023824	3236,2478	1944,3691	0,6008097	-0,73502	0,2778216	0,7112774	conserved Plasmodium protein, unknown function
PFIT_12111	215,0928832	204,2090218	221,9073455	224,6982515	171,316082	314,9438906	128,4843663	130,8589386	225,36125	129,67165	0,5753946	-0,797376	0,2780852	0,7112774	mitochondrial ribosomal protein S18 precursor, putative
PFIT_11480	0	9,512842632	2,493340961	6,087883661	62,1711588	2,916147135	32,4212887	28,44759534	13,863562	30,434442	2,195283	1,1344069	0,2780864	0,7112774	conserved Plasmodium protein, unknown function
PFIT_09177	2174,702256	3515,312447	4729,867803	5014,202361	3100,268452	5473,608173	2245,47444	2636,143835	4001,3269	2440,8091	0,6099999	-0,713119	0,2782644	0,7112774	pre-mRNA-splicing factor ATP-dependent RNA helicase PRP43, putative
PFIT_06157	146,036747	67,85827744	56,10017163	49,80995723	70,46064664	142,8912096	38,42523105	48,36091208	88,859502	43,393072	0,4883335	-1,034061	0,278377	0,7112774	ras GTPase, putative
PFIT_13305	2653,566938	1239,2063	1264,123867	1109,101714	917,3699876	1329,763094	803,3274867	861,9621388	1418,853	832,64481	0,5868426	-0,768954	0,2784486	0,7112774	conserved Plasmodium protein, unknown function
PFIT_09016	58,86752593	8,878653123	4,363346682	7,748215569	4,14474392	17,49688281	1,20078847	1,896506356	16,916561	1,5486474	0,0915462	-3,449356	0,2787092	0,7112774	stevor
PFIT_05096	981,502788	417,9308863	526,0949428	520,237331	547,1061974	1116,884353	384,2523105	360,3362076	684,95942	372,29426	0,543575	-0,879575	0,278834	0,7112774	asparagine-tRNA ligase, putative
PFIT_06159	2981,866602	1402,193004	1385,050904	1334,906854	990,5937969	1166,458854	888,5834681	963,4252289	1543,5117	926,00435	0,5999335	-0,737126	0,2789756	0,7112774	conserved Plasmodium protein, unknown function
PFIT_14249	3,396203419	3,805137053	0	2,213775877	4,14474392	20,41302995	0	0	5,6621484	0	0	#DIV/0!	0,279049	0,7112774	small nucleolar RNA snoR23
PFIT_12341	139,2443402	71,66341449	43,01013158	42,06174166	24,86846352	55,40679557	25,21655788	32,24060805	62,709148	28,728583	0,4581243	-1,126189	0,2790531	0,7112774	aspartyl protease, putative
PFIT_11068	2007,156221	2903,319571	3970,022145	3765,079322	2522,767466	4152,593521	1844,411091	2102,277296	3220,1564	1973,3442	0,6128101	-0,706488	0,279798	0,7126568	RuvB DNA helicase, putative (RUVB2)
PFIT_13576	367,922037	537,1585139	646,3986442	509,1684517	507,0403395	1032,316086	312,2050023	366,0257267	600,00068	339,11536	0,5651916	-0,823188	0,2798463	0,7126568	zinc finger protein, putative
PFIT_12348	0	5,07351607	1,246670481	1,660331908	13,81581307	0	8,405519293	8,534278602	3,6327219	8,4698989	2,3315572	1,2212938	0,2800946	0,7127438	conserved Plasmodium protein, unknown function
PFIT_06078	152,8291538	117,9592486	65,45020023	59,2185047	240,3951474	49,5745013	196,9293092	144,1344831	114,23779	170,5319	1,49278	0,5780016	0,2801325	0,7127438	conserved Plasmodium protein, unknown function
PFIT_09126	4666,383497	1742,118581	1970,362695	2131,866169	1410,594514	1589,300189	1206,792413	1319,020171	2251,7709	1262,9063	0,5608502	-0,834312	0,2807667	0,7129798	conserved Plasmodium protein, unknown function
PFIT_12108	2159,985374	2879,22037	3165,919685	3583,549701	2292,043388	2507,886536	1724,332244	1803,577545	2764,7675	1763,9549	0,638012	-0,648344	0,2808657	0,7129798	conserved Plasmodium protein, unknown function
PFIT_05373	0	10,14703214	14,33671053	6,64132763	4,14474392	8,748441406	2,401576941	1,896506356	7,3363759	2,1490416	0,2929296	-1,771374	0,2809324	0,7129798	rifin (RIF)
PFIT_13475	2376,210325	2117,55877	2138,663209	1999,593061	1996,384988	2128,787409	1244,016855	1325,657943	2126,1996	1284,8374	0,6042882	-0,726691	0,2809367	0,7129798	elongation factor Tu, putative
PFIT_12040	2473,568157	7250,688654	6304,41262	4900,192903	5951,852269	10066,53991	3178,487081	3944,733221	6157,8758	3561,6102	0,5783829	-0,789903	0,2809726	0,7129798	eukaryotic translation initiation factor 5A
PFIT_12397	658,8634633	1107,294882	1480,421196	1582,849752	849,6725036	871,9279935	691,654159	739,6374789	1091,8383	715,64582	0,6554504	-0,609442	0,2811777	0,7129798	3-hydroxyisobutyryl-coenzyme A hydrolase, putative
PFIT_07070	79,24474644	178,8414415	163,313833	154,9643114	138,1581307	78,73597266	84,05519293	82,49802649	132,20974	83,27661	0,6298826	-0,666845	0,2812971	0,7129798	DNA-directed RNA polymerase 2 8.2 kDa polypeptide, putative
PFIT_11130	0	26,00176986	29,29675629	32,65319418	105,0001793	29,16147135	62,44100046	63,53296293	37,018895	62,986982	1,7014819	0,7667918	0,2813025	0,7129798	ubiquinone biosynthesis protein COQ4, putative (COQ4)
PFIT_13116	32,82996638	32,34366495	43,63346682	33,76008212	82,8948784	55,40679557	69,64573129	69,222482	46,811476	69,434107	1,483271	0,5687823	0,2813598	0,7129798	translation initiation factor IF-2, putative
PFIT_12474	476,6005464	383,6846528	274,890841	273,9547648	273,5530987	172,052681	178,9174821	198,1849142	309,12276	188,5512	0,6099557	-0,713224	0,281678	0,7131967	conserved Plasmodium protein, unknown function
PFIT_11289	5578,830149	3687,811994	5034,055401	5558,791227	3277,110859	3327,323881	2723,388251	2868,465863	4410,6539	2795,9271	0,6339031	-0,657666	0,2817804	0,7131967	conserved Plasmodium protein, unknown function
PFIT_03202	150,5650182	93,22585779	117,1870252	123,4180051	127,1054802	110,8135911	60,03942352	75,86025424	120,38583	67,949839	0,5644339	-0,825124	0,2820767	0,7131967	CPW-WPC family protein
PFIT_07003	0	3,805137053	8,726693364	9,961991446	5,526325227	2,916147135	1,20078847	0,948253178	5,156049	1,0745208	0,2084	-2,262573	0,2820891	0,7131967	stevor
PFIT_14139	9,05654245	19,65987477	15,58338101	21,5843148	17,96055699	17,49688281	8,405519293	6,637772246	16,890259	7,5216458	0,4453245	-1,167071	0,2821373	0,7131967	non-canonical ubiquitin conjugating enzyme, putative
PFIT_11502	1,132067806	3,805137053	3,116676201	1,660331908	5,526325227	5,832294271	9,606307764	6,637772246	3,5121387	8,12204	2,3125624	1,2094923	0,2823256	0,7131967	rifin
PFIT_10044	174,3384422	117,9592486	122,7970423	86,33725919	161,6450129	294,5308607	76,8504621	84,39453284	159,60131	80,622497	0,5051493	-0,985218	0,282328	0,7139082	ADP/ATP carrier protein, putative
PFIT_13256	528,6756655	1213,20453	1560,831442	1319,963867	1167,436204	1600,964777	734,8825439	775,6710996	1231,8461	755,27682	0,613126	-0,705745	0,282801	0,7139082	translation initiation factor EIF-2B gamma subunit, putative
PFIT_09236	90,5654245	73,56598302	67,94354119	64,19950043	165,7897568	37,90991276	144,0946165	112,8421282	83,32902	128,46837	1,5417003	0,6245223	0,2832016	0,7139082	cyclin-dependent kinase, putative
PFIT_02181	1917,722864	1752,265613	2060,746304	2356,010977	1490,72623	2297,923943	1229,607394	1209,971055	1979,2327	1219,7892	0,616294	-0,698309	0,2832297	0,7139082	pre-mRNA-processing protein 45, putative
PFIT_01080	370,1861727	470,5686155	313,5376259	226,9120274	388,2243472	740,7013724	207,7364054	242,7528136	418,35503	225,24461	0,5384054	-0,893235	0,2834031	0,7139082	N-acetyltransferase, putative
PFIT_07137	30,56583077	70,39503547	57,34684211	71,39427203	332,9610949	69,98753125	193,3269437	171,6338252	105,44177	182,48038	1,7306271	0,7912949	0,2834193	0,7139082	GTP-binding translation elongation factor tu family protein, putative
PFIT_05178	5599,20737	7238,004864	8275,39865	8369,733146	4928,100521	6879,191092	4028,645318	4682,474193	6881,6059	4355,5598	0,6329278	-0,659887	0,2834253	0,7139082	eukaryotic translation initiation factor, putative
PFIT_08059	5797,319236	2771,408153	3082,392763	3099,839671	2261,648599	4015,534605	1948,879688	2152,534714	3504,6905	2050,7072	0,5851322	-0,773166	0,283505	0,7139082	conserved Plasmodium protein, unknown function
PFIT_bin09	38,49030541	16,48892723	12,46670481	14,94298717	22,10530091	26,24532422	38,42523105	30,3441017	21,789925	34,384666	1,5780076	0,6581041	0,2836195	0,7139082	erythrocyte membrane protein 1, PfEMP1 (VAR)
PFIT_06140	730,1837351	479,4472686	489,9414989	523,0045509	381,3164406	387,847569	296,5947522	314,8200551	498,62351	305,7074	0,6131027	-0,705799	0,2838643	0,7142066	conserved Plasmodium protein, unknown function
PFIT_09134	2834,697787	1272,184155	1458,604462	1159,465115	998,8832847	1679,70075	852,559814	967,2182416	1567,2559	909,88903	0,5805619	-0,784478	0,2841902	0,7144219	conserved Plasmodium protein, unknown function
PFIT_13184	82,64094986	161,0841352	111,577008	137,8075483	77,36855317	81,65211979	180,1182706	184,9093697	108,68839	182,51382	1,6792394	0,7478079	0,2842025	0,7144219	conserved Plasmodium protein, unknown function
PFIT_12212	104,1502382	237,8210658	397,6878833	359,185136	464,211319	338,2730677	492,3232729	476,0230954	316,88812	484,17318	1,5278995	0,6115496	0,2843694	0,7144242	conserved Plasmodium protein, unknown function
PFIT_10184	414,3368171	395,7342535	978,012992	886,0637947	292,895237	1195,620326	417,8743877	376,4565117	693,77724	397,16545	0,5724683	-0,804732	0,2844758	0,7144242	conserved Plasmodium protein, unknown function
PFIT_07056	157,3574251	397,636822	339,717706	218,6103678	198,9477082	230,3756237	146,4961934	175,4268379	257,10761	160,96152	0,6260473	-0,675657	0,2849659	0,7144242	DNA mismatch repair protein, putative
PFIT_13077	53,2071869	84,34720467	95,37029176	90,21136698	82,8948784	29,16147135	111,6733278	134,6519513	72,532067	123,16264	1,698044	0,7638738	0,285045	0,7144242	conserved Plasmodium protein, unknown function
PFIT_10323	2044,514458	1274,720913	1395,647603	1466,073074	1007,172773	1236,446385	941,4181608	806,0152013	1404,0959	873,71668	0,6222628	-0,684404	0,2851219	0,7144242	RNA polymerase II-associated protein 1, putative
PFIT_10140	356,601359	484,5207847	450,6713787	595,5057109	426,9086238	172,052681	253,3663673	288,2689661	414,37676	270,81767	0,6535542	-0,613621	0,2851537	0,7144242	conserved Plasmodium protein, unknown function
PFIT_07171	13,58481368	81,81044663	74,17689359	45,93584944	31,77637005	34,99376562	15,61025012	37,93012712	47,046357	26,770189	0,5690173	-0,813456	0,2851902	0,7144242	conserved Plasmodium protein, unknown function
PFIT_08257	1967,533847	1191,007897	1262,253862	1251,890258	661,7774459	615,3070456	672,4415435	795,5844164	1158,2951	734,01298	0,6337012	-0,658125	0,2852139	0,7144242	U5 snrnp-specific protein, putative
PFIT_10175	1031,313772	296,1665006	365,2744508	322,657834	330,1979323	367,4345391	214,9411362	214,3052182	452,17417	214,6238	0,4746471	-1,075073	0,2855344	0,7148709	tRNA pseudouridine synthase, putative
PFIT_05112	15,84894929	22,19663281	29,29675629	18,81709495	22,10530091	49,5745013	13,20867318	10,43078496	26,306539	11,819729	0,4493076	-1,154225	0,285645	0,7148709	conserved Plasmodium protein, unknown function
PFIT_08254	1,132067806	35,51461249	72,30688787	74,71493584	16,57897568	40,8260599	14,40946165	23,70632945	40,178923	19,057896	0,4743257	-1,07605	0,2859607	0,7153444	dynein light chain 1
PFIT_08270	57,73545812	77,37112007	94,74695652	164,9263028	185,1318951	110,8135911	184,9214245	171,6338252	115,12089	178,27762	1,5486123	0,630976	0,2861457	0,7154833	exonuclease, putative
PFIT_09373	2756,585108	1681,870577	2270,83028	2055,490902	1319,410148	1283,10474	1115,532489	1272,555765	1894,5453	1194,0441	0,6302537	-0,665995	0,2863019	0,7154833	lysophospholipase, putative
PFIT_11201	4168,273663	2421,335545	3506,260727	3441,3146	2341,780315	3636,435478	1945,277322	2002,710712	3252,5667	1973,994	0,6069035	-0,720461	0,2863957	0,7154833	U2 snRNP auxiliary factor, small subunit, putative
PFIT_13124	103,0181704	58,34543481	47,9968135	40,95485372	87,03962232	137,0589154	36,02365411	39,82663348	79,068968	37,925144	0,4796464	-1,059957	0,2866149	0,7157146	methionyl-tRNA formyltransferase, putative
PFIT_03157	176,6025778	109,714785	107,2136613	125,631781	132,6318054	154,5557982	66,04336588	82,49802649	134,39173	74,270696	0,5526433	-0,855579	0,2867573	0,7157544	conserved Plasmodium membrane protein, unknown function
PFIT_08349	3,396203419	11,41541116	4,363346682	8,855103507	1,381581307	0	1,20078847	0,948253178	4,901941	1,0745208	0,2192031	-2,18966	0,2870513	0,7161722	Plasmodium exported protein, unknown function, pseudogene
PFIT_12376	6896,557076	5087,468239	5035,925406	10067,1458	1906,582203	3067,786786	8678,098276	9149,694915	5343,5776	8913,8966	1,6681514	0,7382503	0,2872213	0,7162802	conserved Plasmodium protein, unknown function
PFIT_05204	554,7132251	420,4676443	440,0746796	415,6364209	414,474392	379,0991276	252,1655788	275,9416748	437,41091	264,05363	0,6036741	-0,728158	0,287459	0,7165572	conserved Plasmodium protein, unknown function
PFIT_13510	340,7524097	579,649211	752,9889703	785,3369923	826,1856214	819,437345	441,8901571	395,4215752	684,05842	418,65587	0,6120177	-0,708355	0,2883606	0,7179716	conserved Plasmodium protein, unknown function
PFIT_07050	730,1837351	622,7740976	403,2979005	407,8882053	922,8963128	545,3195143	895,788199	825,9285181	605,39329	860,85836	1,421982	0,5079032	0,2883861	0,7179716	conserved Plasmodium protein, unknown function
PFIT_11191	9318,050114	14434,15322	15183,82312	16721,20264	9806,464115	9010,894648	7996,050425	8422,384727	12412,431	8209,2176	0,6613706	-0,596469	0,2884073	0,7179716	insulinase, putative
PFIT_14571	324,9034604	317,0947544	395,8178776	250,710118	200,3292895	320,7761849	200,5316746	171,6338252	301,60528	186,08275	0,6169744	-0,696717	0,2889542	0,7188845	dfg10 like protein, putative
PFIT_05138	5,660339031	6,341895088	8,103358124	11,06887938	13,81581307	11,66458854	21,61419247	16,12030403	10,363533	18,867248	1,8205421	0,8643681	0,2890282	0,7188845	secreted ookinete protein, putative
PFIT_05062	59,99959373	296,8006901	270,5274943	314,9296185	169,9345007	55,40679557	115,2756932	125,1694195	194,59646	120,22256	0,6178045	-0,694778	0,2892769	0,7191868	transcription initiation factor TFIID, TATA-binding protein
PFIT_05156	101,8861026	39,95393905	21,19339817	23,69120274	42,82902051	11,66458854	75,64967364	56,89519068	40,036375	66,272432	1,6553055	0,7270975	0,2895986	0,7192911	amino acid transporter, putative
PFIT_14392	8004,851458	4474,841174	6769,42071	7036,486624	3833,888126	6453,433611	3828,113644	3743,703547	6095,487	3785,9086	0,6211003	-0,687102	0,2897204	0,7192911	eukaryotic translation initiation factor eIF2A, putative
PFIT_11268	208,3004764	334,8520606	320,3943135	369,1471275	342,632164	399,5121575	193,3269437	202,9261801	329,13972	198,12656	0,6019528	-0,732278	0,2898157	0,7192911	conserved Plasmodium protein, unknown function
PFIT_09315	16525,92584	5151,52138	7369,069211	8980,181844	3550,663958	3473,131238	3867,739663	3777,840661	7508,4156	3822,7902	0,5091341	-0,973882	0,2899742	0,7192911	nucleolar protein Nop52, putative
PFIT_07207	3704,125862	1399,656246	2921,572271	3346,122238	1439,607722	3193,181113	1625,867589	1536,684985	2667,3776	1631,2763	0,6115656	-0,709421	0,2902422	0,7192911	cell cycle control protein cwf15, putative
PFIT_09109	106,4143738	127,4720913	94,12362128	94,08547476	88,42120363	145,8073568	151,2993473	173,5303316	109,38735	162,41484	1,484768	0,5702375	0,290303	0,7192911	actin-like protein, putative (ALP5a)
PFIT_12340	33,96203419	63,22408793	74,17689359	75,26837981	189,276639	102,0651497	135,6890972	124,2211663	90,128864	129,95513	1,4386889	0,5247546	0,2903411	0,7192911	DNA polymerase epsilon subunit b, putative
PFIT_04037	2452,058868	1433,26829	2599,931287	2867,946648	1674,476544	2496,221948	1372,501222	1422,379767	2253,9839	1397,4405	0,6199869	-0,68969	0,2905721	0,7192911	ATP-dependent RNA helicase, putative
PFIT_12106	378,1106473	162,3525142	120,3037014	153,8574234	139,539712	84,56826693	249,7640019	272,1486621	173,12204	260,95633	1,5073547	0,592019	0,2906143	0,7192911	kinesin-like protein, putative
PFIT_06054	3492,429182	2738,430299	2755,141762	2885,103411	1758,753003	2248,349441	1561,025012	1763,750911	2646,3678	1662,388	0,6281772	-0,670757	0,290716	0,7192911	polypyrimidine tract binding protein, putative
PFIT_06044	30,56583077	42,49069709	28,05008581	23,24464671	129,8686428	26,24532422	63,64178893	83,44627967	46,744205	73,544034	1,5733295	0,6538208	0,2907177	0,7192911	cyclin dependent kinase binding protein, putative
PFIT_10327	198,1118661	338,0230082	460,0214073	436,1138477	451,7770873	653,2169583	204,13404	281,6311939	422,87736	242,88262	0,5743571	-0,79998	0,2911461	0,720036	conserved Plasmodium protein, unknown function
PFIT_07022	13,58481368	67,22408793	57,34684211	40,40140975	88,42120363	154,5557982	100,8662315	92,92881145	70,255693	96,897521	1,3792124	0,4638446	0,291354	0,720084	conserved Plasmodium protein, unknown function
PFIT_13451	7,924474644	43,1248866	41,7634611	45,93584944	175,4608259	93,31670833	108,0709623	97,67007734	67,921034	102,87052	1,5145606	0,5988993	0,2914259	0,720084	conserved Plasmodium protein, unknown function
PFIT_14602	8025,228679	6350,773741	8235,505195	7886,576561	5164,350924	7649,053936	4283,212474	4687,215459	7218,581	4485,214	0,6213428	-0,686539	0,2917351	0,7205476	ATP-dependent RNA helicase DBP5
PFIT_14372	3110,922332	2663,595937	2349,973856	1799,799788	2271,319668	5902,281802	1324,469683	1824,439114	3016,3156	1574,4544	0,5219793	-0,937935	0,2922274	0,7210735	glucose-6-phosphate isomerase
PFIT_08281	7,924474644	17,12311674	5,610017163	11,62232335	59,40799619	8,748441406	34,8286564	30,3441017	18,406062	32,583484	1,7702583	0,8239599	0,292304	0,7210735	translation initiation factor IF-3, putative
PFIT_08299	17549,31513	4577,579874	8390,71567	9144,001259	3833,888126	6368,865344	4360,062936	4481,444519	8310,726	4420,7537	0,5319334	-0,910682	0,2924008	0,7210735	ATP-dependent RNA helicase DBP10, putative
PFIT_14589	2964,885585	3436,038759	3836,005069	3962,658819	2406,714636	2959,889342	2061,753804	2140,207423	3261,032	2100,9806	0,6442686	-0,634266	0,2926083	0,7210735	large ribosomal subunit nuclear export factor, putative
PFIT_14126	59,99959373	27,90433839	16,83005149	19,92398289	63,55274011	49,5745013	51,63390423	56,89519068	39,630808	54,264547	1,3692495	0,4533854	0,2927505	0,7210735	GTP binding protein, putative
PFIT_12148	0	15,85473772	8,726693364	13,28265526	59,40799619	8,748441406	36,02365411	30,3441017	17,67008	33,183878	1,8779691	0,9091733	0,2928937	0,7210735	peptidyl-prolyl cis-trans isomerase
PFIT_08352	6,792406838	23,46501182	42,38679634	37,08074594	33,15795136	113,7297383	9,606307764	14,22379767	42,768775	11,915053	0,2785923	-1,843773	0,2929899	0,7210735	Plasmodium exported protein (PHISTa-like), unknown function
PFIT_08009	12,45274587	10,14703214	9,350028604	4,427551754	1,381581307	17,49688281	2,401576941	2,844759534	9,2093037	2,6231682	0,2848389	-1,811782	0,2931396	0,7210735	surface-associated interspersed protein 1.3 (SURFIN 1.3)
PFIT_09160	876,2204821	357,0486934	486,2014874	508,6150077	310,855796	580,3132799	307,4018484	309,1305036	519,87579	308,26619	0,5929612	-0,75399	0,2932204	0,7210735	conserved Plasmodium protein, unknown function
PFIT_07195	4976,570076	3748,059997	4546,607243	4879,715476	3217,702863	3992,205428	2552,876288	2748,03771	4226,8102	2650,457	0,6270594	-0,673328	0,2935141	0,7210735	DEAD/DEAH box ATP-dependent RNA helicase, putative
PFIT_09116	2441,870258	1203,691688	819,0625057	814,6695227	711,5143729	959,4124075	620,8076392	630,5883634	1158,3701	625,698	0,5401538	-0,888558	0,293617	0,7210735	conserved Plasmodium protein, unknown function
PFIT_06036	37,35823761	43,75907611	21,19339817	18,81709495	194,8029642	55,40679557	100,8662315	96,72182416	61,889594	98,794028	1,5962946	0,674727	0,2936939	0,7210735	glyoxalase I
PFIT_12450	4233,933595	2788,53127	3355,413598	3067,186477	2124,87205	4251,742523	1963,289149	2004,607218	3303,6133	1983,9482	0,6005389	-0,73567	0,2937779	0,7210735	cytoplasmic translation machinery associated protein, putative
PFIT_09354	332,827935	254,9441825	436,3346682	362,5057998	200,3292895	288,6986664	205,3348284	191,547142	312,60674	198,44099	0,6347943	-0,655639	0,2941109	0,7210735	conserved Plasmodium protein, unknown function
PFIT_03092	3712,050337	3174,752681	3402,787077	3086,003572	2724,478337	4108,851314	1927,265495	2126,931878	3368,1539	2027,0987	0,6018427	-0,732542	0,2941492	0,7210735	N2227-like protein, putative
PFIT_13082	752,8250912	2011,649122	2397,970669	2001,806837	1353,949681	2017,973818	1057,894642	1191,954245	1756,0292	1124,9244	0,6406069	-0,642489	0,2941539	0,7210735	conserved Plasmodium protein, unknown function
PFIT_14578	6,792406838	13,31797968	1,246670481	2,213775877	16,57897568	2,916147135	12,0078847	15,17205085	7,1776593	13,589968	1,8933704	0,9209567	0,2942336	0,7210735	mitochondrial ribosomal protein L28 precursor, putative
PFIT_01089	2028,665509	1337,505674	1769,025412	1530,272575	983,6858903	1819,675812	911,3984491	1009,889635	1578,185	960,64404	0,6087197	-0,71615	0,2943154	0,7210735	DNA-directed RNA polymerase 2, putative
PFIT_05177	119,9991875	123,6669542	264,2941419	366,9333516	59,40799619	411,1767461	117,6772701	109,0491155	224,2464	113,36319	0,5055296	-0,984133	0,2943896	0,7210735	f-actin capping protein beta subunit, putative
PFIT_14091	1246,406655	4374,005042	4149,542695	3164,592616	3592,111397	5441,530555	1948,879688	2446,493199	3661,3648	2197,6864	0,6002369	-0,736396	0,2944978	0,7210735	histo-aspartic protease
PFIT_07267	4716,194481	2130,87675	2359,323885	1722,317632	2102,766749	3263,168645	1518,997415	1597,806605	2715,7747	1558,402	0,5738333	-0,801296	0,2949445	0,7218547	cation transporting ATPase, putative
PFIT_07277	72,4523396	54,54029776	76,67023456	73,6080479	225,197753	157,4719453	154,9017127	136,5484576	109,9901	145,72509	1,3248927	0,4058755	0,2953278	0,7224802	conserved Plasmodium protein, unknown function
PFIT_14137	2256,211138	931,6243884	1203,037014	1154,48412	595,4615432	1793,430488	755,2959479	788,9466441	1322,3748	772,1213	0,5838899	-0,776232	0,2958145	0,7228863	AAA family ATPase, putative
PFIT_04208	16,98101709	91,95747877	190,1172483	191,4916133	99,47385408	125,3943268	67,24415435	78,70501378	119,23592	72,974584	0,6120184	-0,708353	0,2958151	0,7228863	conserved Plasmodium protein, unknown function
PFIT_06256	1118,482993	2369,332005	2093,783072	1627,678713	1476,910417	1580,551747	1032,678085	1162,558396	1711,1232	1097,6182	0,6414607	-0,640567	0,2959056	0,7228863	conserved protein, unknown function
PFIT_03088	3769,785795	5385,537309	7475,036202	7658,557646	4383,757486	6712,970706	3493,093661	4031,02426	5897,6075	3762,059	0,6378958	-0,648607	0,2961356	0,7228863	splicing factor 3B subunit 1, putative
PFIT_14729	4,528271225	11,41541116	9,350028604	7,748215569	12,43423176	2,916147135	18,01182706	16,12030403	8,0653842	17,066066	2,1159644	1,0813153	0,2961925	0,7228863	conserved Plasmodium protein, unknown function
PFIT_11125	1789,799202	577,7466425	662,6053604	599,9332626	475,2639695	414,0928932	368,6420604	338,5263846	753,24022	353,58422	0,4694176	-1,091056	0,2963211	0,7228863	conserved Plasmodium protein, unknown function
PFIT_14718	474,3364108	126,2037122	152,0937986	128,9524448	201,7108708	393,6798633	108,0709623	109,0491155	246,16285	108,56004	0,441009	-1,18112	0,2963956	0,7228863	conserved Plasmodium protein, unknown function
PFIT_01128	45,28271225	60,24800333	360,9111041	317,1233943	328,816351	1808,011224	39,62601953	35,08536759	486,71323	37,355694	0,076748	-3,703728	0,2965163	0,7228863	Pfmc-2TM Maurer's cleft two transmembrane protein
PFIT_11400	11251,62193	5035,4647	5309,569577	3880,195668	4155,79657	14283,28867	2802,64029	3734,221015	7319,3229	3268,4307	0,4465482	-1,163112	0,2972109	0,7239379	transcription factor with AP2 domain(s)
PFIT_08174	2368,285851	2524,708434	2462,174199	3115,889547	1345,660193	527,8226315	1430,139068	1429,017539	2057,4235	1429,5783	0,6948391	-0,525249	0,2972638	0,7239379	conserved Plasmodium protein, unknown function
PFIT_14682	203,7722051	332,3153026	344,0810526	308,2682908	182,3687325	274,1178307	154,9017127	190,5988888	274,1539	172,7503	0,6301216	-0,666298	0,2973762	0,7239379	DNA-3-methyladenine glycosylase, putative
PFIT_04061	101,8861026	143,9610185	151,4704634	125,631781	88,42120363	119,5620326	63,64178893	83,44627967	121,8221	73,544034	0,6037003	-0,728096	0,2974597	0,7239379	mitochondrial carrier protein, putative
PFIT_11468	3,396203419	15,22054821	5,610017163	4,980995723	13,81581307	8,748441406	18,01182706	15,17205085	8,6286698	16,591939	1,9228849	0,9432724	0,2978427	0,7240283	conserved Plasmodium protein, unknown function
PFIT_11366	96,22576353	423,6385919	280,5008581	234,106799	565,0667544	399,5121575	503,1303691	457,0580318	333,17515	480,0942	1,4409664	0,5270367	0,2978556	0,7240283	3-oxo-5-alpha-steroid 4-dehydrogenase, putative
PFIT_09068	16369,70048	24893,2066	32905,244	32702,4507	17135,75295	16108,79678	15474,56102	16138,32084	23352,525	15806,441	0,6768622	-0,563066	0,297951	0,7240283	zinc finger protein, putative
PFIT_08207	58,86752593	22,83082232	5,610017163	4,980995723	48,35534573	2,916147135	37,22444258	41,72313983	23,92609	39,473791	1,6497725	0,7222671	0,2980088	0,7240283	conserved Plasmodium protein, unknown function
PFIT_01057	1,132067806	4,439326561	13,09004005	15,49643114	15,19739437	29,16147135	22,81498094	19,91331674	13,086122	21,364149	1,6325806	0,7071542	0,2981853	0,7241461	carbon catabolite repressor protein 4, putative
PFIT_10142	194,7156627	102,7387004	185,1305664	142,7885441	225,197753	746,5336667	58,83863505	72,06724153	266,18415	65,452938	0,2458934	-2,023895	0,2984932	0,7242474	prohibitin, putative
PFIT_12269	8338,811461	3692,25132	4403,863473	4500,052914	2707,899361	3645,183919	2764,215059	2877,948395	4548,0104	2821,0817	0,6202892	-0,688987	0,2986654	0,7242474	potassium channel protein
PFIT_bin07	5,660339031	4,439326561	16,20671625	18,26365098	5,526325227	32,07761849	6,003942352	3,793012712	13,695663	4,8984775	0,3576663	-1,483314	0,2987221	0,7242474	rifin
PFIT_12490	14,71688148	27,27014888	12,46670481	18,81709495	27,63162613	26,24532422	27,61813482	39,82663348	21,191297	33,722384	1,5913318	0,6702347	0,2987954	0,7242474	conserved Plasmodium protein, unknown function
PFIT_11171	1099,23784	832,690825	809,0891419	767,6267853	643,8168889	1043,980674	481,5161767	551,8833496	866,07369	516,69976	0,5966002	-0,745164	0,2988673	0,7242474	peptidyl-prolyl cis-trans isomerase
PFIT_06010	3378,090334	3110,065351	3022,55258	3085,450128	1979,806012	3446,885914	1742,344071	1971,418357	3003,8084	1856,8812	0,6181757	-0,693911	0,2990659	0,7244184	ATP-dependent RNA helicase, putative
PFIT_07039	27492,26668	4866,136101	8072,191362	7175,954505	4684,942211	9360,832305	3320,180121	3672,584558	10275,387	3496,3823	0,3402677	-1,555258	0,2992651	0,7244816	methyltransferase, putative
PFIT_07241	644,1465818	10183,18094	846,4892563	2329,99911	218672,5245	539568,8722	2005,316746	2550,801049	128707,54	2278,0589	0,0176995	-5,820148	0,2993482	0,7244816	5.8S ribosomal RNA
PFIT_14541	3,396203419	17,5730625	17,45338673	15,49643114	35,92111397	14,58073568	25,21655788	33,18886123	17,434196	29,20271	1,6750247	0,7441824	0,2996104	0,7246706	conserved Plasmodium protein, unknown function
PFIT_14058	13763,68039	24057,97902	29751,79102	29008,2122	21965,76119	20547,17272	14213,73312	15985,65207	23182,433	15099,693	0,651342	-0,618513	0,2996826	0,7246706	conserved Plasmodium protein, unknown function
PFIT_11465	1636,970048	653,8493836	1298,407306	1477,695398	871,7778045	1370,589154	750,492794	744,3787447	1218,2148	747,43577	0,61355	-0,704767	0,2998413	0,7247446	conserved Plasmodium protein, unknown function
PFIT_10229	7,924474644	26,63595937	34,28343822	28,7790864	116,0528298	61,23908984	85,2559814	54,99868432	45,819146	70,127333	1,5305246	0,6140263	0,30046	0,7257776	HORMA domain protein, putative
PFIT_10332	10,18861026	8,244463614	5,610017163	4,980995723	9,671069147	0	15,61025012	12,32729131	6,4491927	13,968771	2,165972	1,1150146	0,3005253	0,7257776	plasmepsin VII
PFIT_13490	2443,002326	637,3604563	690,6554462	663,0258751	585,790474	632,8039284	356,6341757	374,5600053	942,10642	365,59709	0,3880635	-1,365635	0,3007833	0,7260216	nucleolar preribosomal associated cytoplasmic ATPase, putative
PFIT_06100	29,43376296	7,610274105	7,480022884	12,17576732	93,94752885	5,832294271	54,03548117	40,77488665	26,079942	47,405184	1,8176875	0,8621042	0,3009474	0,7260216	succinate dehydrogenase subunit 3, putative
PFIT_14239	199,2439339	339,9255767	95,37029176	146,1092079	164,4081755	58,32294271	114,0749047	85,34278602	167,23002	99,708845	0,5962377	-0,74604	0,3010718	0,7260216	14-3-3 protein, putative
PFIT_13268	179,9987812	238,4552553	158,327151	205,3277126	149,2107811	198,2980052	267,7758289	305,3375233	188,26961	286,55668	1,5220548	0,6060203	0,3011398	0,7260216	nicotinic acid mononucleotide adenylyltransferase
PFIT_07020	27,16962735	88,15234172	114,6936842	107,921574	91,18436624	87,48441406	51,63390423	53,10217797	86,101001	52,368041	0,6082164	-0,717343	0,3013216	0,7260641	conserved Plasmodium protein, unknown function
PFIT_04184	643,014514	943,0397996	912,5627918	930,3393122	715,6591168	1017,73535	506,7327345	564,2106409	860,39181	535,47169	0,622358	-0,684184	0,3014837	0,7260641	60S ribosomal protein L7ae/L30e, putative
PFIT_08304	43,01857664	49,46678168	43,63346682	53,13062104	290,1320744	40,8260599	192,1261553	119,4799004	86,701263	155,80303	1,7970099	0,8455983	0,3015425	0,7260641	apicoplast ribosomal protein L21 precursor, putative
PFIT_03089	1404,896148	601,8458438	837,762563	830,7193978	667,3037711	787,3597266	512,7366769	526,2805138	854,98124	519,5086	0,6076257	-0,718745	0,3018485	0,7264915	dual specificity phosphatase
PFIT_11243	13482,92757	7307,13152	8087,774743	7483,115908	5744,615073	9789,505934	4853,586998	5522,626509	8649,1785	5188,1068	0,5998381	-0,737355	0,3024066	0,7275252	translation elongation factor EF-1, subunit alpha, putative
PFIT_09260	2445,266462	10169,22877	14482,57087	14770,86609	7213,236002	7902,758737	6244,100046	6777,165463	9497,3212	6510,6328	0,6855231	-0,544723	0,3027648	0,727984	conserved Plasmodium protein, unknown function
PFIT_10112	1382,254791	1362,873254	1489,147889	1357,598056	1123,225602	2615,78398	859,7645448	950,1496844	1555,1473	904,95711	0,5819109	-0,78113	0,3028547	0,727984	DNA repair protein RAD23, putative
PFIT_13635	460,7515972	1045,14431	1158,780212	1048,222878	721,1854421	854,4311107	557,1658503	595,5029958	881,41926	576,33442	0,6538709	-0,612922	0,3030883	0,7280491	exosome complex exonuclease, putative
PFIT_14177	7,924474644	2,536758035	4,363346682	2,213775877	16,57897568	8,748441406	14,40946165	9,48253178	7,0609621	11,945997	1,691837	0,7585906	0,3033549	0,7280491	conserved Plasmodium protein, unknown function
PFIT_13544	333,9600029	1034,997278	1261,630526	1215,9164	780,5934383	594,8940156	521,1421962	651,4499333	870,33194	586,29606	0,6736465	-0,569936	0,3033961	0,7280491	histone-lysine N-methyltransferase, putative
PFIT_09190	6156,184731	5781,905752	8532,836104	9358,184075	4461,126039	6260,9679	4446,519706	4452,996924	6758,5341	4449,7583	0,658391	-0,602983	0,3034505	0,7280491	gamma-glutamylcysteine synthetase
PFIT_07242	680,3727516	11538,44392	1021,646459	2503,780517	225985,2543	553315,5898	2237,06892	2752,778976	132507,51	2494,9239	0,0188285	-5,730935	0,3035254	0,7280491	5.8S ribosomal RNA
PFIT_14522	1738,85615	906,8909976	1074,006639	812,4557468	396,513835	1577,6356	641,2210432	637,2261356	1084,392	639,22359	0,5894759	-0,762495	0,304108	0,729039	conserved Plasmodium protein, unknown function
PFIT_02196	5322,982825	1834,710249	2221,566796	2452,310228	1374,6734	1749,688281	1408,524876	1365,484576	2492,6553	1387,0047	0,5564366	-0,845711	0,3041959	0,729039	Plasmodium exported protein, unknown function
PFIT_06249	1630,177641	1631,135417	2051,396276	1831,346094	1616,450129	3178,600378	1180,375066	1179,626953	1989,851	1180,001	0,5930097	-0,753872	0,3046406	0,729575	conserved Plasmodium protein, unknown function
PFIT_05122	55,47132251	8,244463614	21,19339817	20,47742686	27,63162613	52,49064844	16,81103859	10,43078496	30,918148	13,620912	0,4405475	-1,182631	0,3047741	0,729575	conserved Plasmodium protein, unknown function
PFIT_13494	149,4329504	350,7067984	560,378381	561,1921848	283,2241679	294,5308607	256,9687327	240,8563072	366,57756	248,91252	0,6790173	-0,55848	0,305001	0,729575	small nuclear ribonucleoprotein E, putative
PFIT_01118	175,47051	84,34720467	62,33352403	48,14962532	30,39478875	160,3880924	42,0275647	40,77488665	93,513958	41,401242	0,4427279	-1,175508	0,305002	0,729575	Plasmodium exported protein, unknown function
PFIT_12233	1048,294789	1420,5845	1221,737071	1092,498395	1405,068189	979,8254375	752,894371	755,7577829	1194,6681	754,32608	0,6314106	-0,66335	0,3051351	0,729575	conserved Plasmodium protein, unknown function
PFIT_05013	4202,235697	1493,516293	1896,185801	1765,486262	1744,93719	6319,290842	1042,284392	1257,383714	2903,6087	1149,8341	0,3960017	-1,336421	0,3053883	0,729575	parasite-infected erythrocyte surface protein (PIESP2)
PFIT_11051	219,6211544	72,297604	104,0969851	90,76481095	163,0265942	236,207918	204,13404	181,116357	147,66918	192,6252	1,3044374	0,3834277	0,3054145	0,729575	conserved Plasmodium protein, unknown function
PFIT_03192	1803,384015	1189,739518	1620,04829	1670,293899	910,4620811	1743,855987	918,6031799	937,8223931	1489,6306	928,21279	0,6231161	-0,682427	0,3054515	0,729575	endonuclease/exonuclease/phosphatase family protein, putative
PFIT_07021	177,7346456	137,6191234	163,313833	191,4916133	273,5530987	204,1302995	309,8034254	247,4940795	191,3071	278,64875	1,4565853	0,5425573	0,3056943	0,7298467	conserved Plasmodium protein, unknown function
PFIT_13366	1550,932895	2159,415277	2343,117168	2297,89936	1599,871153	1953,818581	1228,406605	1332,295715	1984,1757	1280,3512	0,6452811	-0,632	0,3060076	0,7301106	conserved Plasmodium protein, unknown function
PFIT_14636	11698,78871	8228,608876	7976,82107	9416,295692	5115,995579	4939,953247	4964,059537	5349,096177	7896,0772	5156,5779	0,6530557	-0,614722	0,306063	0,7301106	conserved Plasmodium protein, unknown function
PFIT_05095	46,41478006	74,83436204	233,1273799	240,1946826	82,8948784	154,5557982	76,85046211	85,34278602	138,67031	81,096624	0,584816	-0,773945	0,3062903	0,7303449	conserved Plasmodium protein, unknown function
PFIT_12513	594,3355983	1053,388774	1459,227798	1353,723949	1229,607363	2099,625937	737,2841209	817,3942394	1298,3182	777,33918	0,5987278	-0,740028	0,3066781	0,7309614	conserved Plasmodium protein, unknown function
PFIT_13624	892,0694313	1272,184155	1202,413679	921,4842087	1149,475647	1131,465089	692,8549475	676,1045159	1094,8487	684,47973	0,625182	-0,677652	0,3069465	0,7312929	polyubiquitin binding protein, putative (DOA1)
PFIT_01131	84,90508547	17,12311674	15,58338101	108,475018	129,8686428	536,5710729	31,22050023	18,96506356	164,64943	25,092782	0,1524013	-2,714053	0,3074892	0,7314106	Plasmodium exported protein, unknown function, pseudogene
PFIT_04155	4812,420245	1972,329372	2253,980229	2141,828161	1522,5026	2580,790215	1504,587953	1566,51425	2547,3085	1535,5511	0,6028132	-0,730217	0,3074927	0,7314106	conserved Plasmodium protein, unknown function
PFIT_13441	7859,946779	5533,303464	5745,28091	5817,803004	4440,40232	6415,523698	3485,88893	3872,665979	5968,71	3679,2775	0,6164276	-0,697997	0,3078731	0,7314106	conserved Plasmodium protein, unknown function
PFIT_01121	2144,136425	3257,831507	3702,611327	3779,468866	2442,63575	2572,041773	1747,147225	2160,12074	2983,1209	1953,634	0,654896	-0,610662	0,3079004	0,7314106	heat shock protein 40, type II
PFIT_13299	54,3392547	75,46855154	90,38360984	96,29925064	323,2900258	113,7297383	216,1419247	164,0477998	125,58507	190,09486	1,513674	0,5980546	0,3079062	0,7314106	elongation factor Tu, putative
PFIT_09066	67,92406838	72,93179351	150,2237929	169,9453071	33,15795136	78,73597266	54,03548117	67,32597564	93,819814	60,680728	0,6467795	-0,628654	0,3079578	0,7314106	dynein light intermediate chain 2, cytosolic
PFIT_02206	65,65993276	71,66341449	92,8768508	86,89070316	63,55274011	180,8011224	45,62996188	54,99868432	93,574144	50,314323	0,5376947	-0,895141	0,308085	0,7314106	Plasmodium exported protein (hyp15), unknown function
PFIT_12007	753,957159	150,9371031	509,2648913	516,9166672	335,7242575	2583,706362	127,2835779	134,6519513	808,41774	130,96776	0,1620051	-2,625889	0,3081192	0,7314106	Plasmodium exported protein (PHISTb), unknown function
PFIT_11085	6055,430696	2194,2957	3686,404611	4155,810765	1982,569175	3406,059854	2222,659459	2259,687323	3580,0951	2241,1734	0,6260095	-0,675764	0,3081621	0,7314106	pescadillo-like protein
PFIT_13623	268,3000701	147,7661555	207,570635	163,2659709	158,8818503	221,6271823	118,8780586	110,9456218	194,56864	114,91184	0,5905979	-0,759752	0,308289	0,7314106	mitochondrial ribosomal protein L9 precursor, putative
PFIT_12518	7787,494439	1884,177031	2482,744262	2725,711548	1396,778017	2481,641212	1203,190047	1416,690248	3126,425	1309,9401	0,4189899	-1,255013	0,3087562	0,7315348	conserved Plasmodium protein, unknown function
PFIT_06090	6281,844257	2198,100837	3700,741322	4102,680144	2384,609335	4686,248447	2393,171422	2326,065046	3892,3707	2359,6182	0,6062162	-0,722096	0,3087649	0,7315348	Pre-mRNA-splicing factor SLU7, putative
PFIT_07322	30,56583077	106,5438375	94,12362128	66,96672027	109,1449232	300,3631549	33,62207717	43,61964619	117,95135	38,620862	0,3274304	-1,61074	0,3087957	0,7315348	Plasmodium exported protein, unknown function
PFIT_09020	14,71688148	17,12311674	25,55674485	22,13775877	49,73692704	37,90991276	39,62601953	42,67139301	27,863557	41,148706	1,476793	0,5624676	0,3089497	0,7315348	conserved Plasmodium protein, unknown function
PFIT_12216	9332,766995	7317,278552	10515,6655	11344,49448	6360,800336	11346,7285	5786,599639	6031,838465	9369,6224	5909,2191	0,6306785	-0,665023	0,3090213	0,7315348	conserved Plasmodium membrane protein, unknown function
PFIT_12276	2191,683273	586,6252956	766,7023456	774,8215569	413,0928107	752,3659609	393,8586183	432,4034492	914,2521	413,13103	0,4518711	-1,145934	0,3092508	0,7315348	RNA pseudouridylate synthase, putative
PFIT_14397	1038,106178	1414,876794	1500,367923	1609,968506	1062,436025	1256,859415	829,7448331	878,0824428	1313,7691	853,91364	0,6499724	-0,62155	0,3094873	0,7315348	cleavage and polyadenylation specifity factor, putative
PFIT_03032	517,3549875	788,9317489	911,9394565	772,054337	625,8563319	799,0243151	427,4806955	503,5224375	735,8602	465,50157	0,6325951	-0,660646	0,3095088	0,7315348	DNA-directed RNA polymerase I, putative
PFIT_14747	4,528271225	20,92825379	34,28343822	34,31352609	22,10530091	40,8260599	9,606307764	16,12030403	26,164142	12,863306	0,4916387	-1,024329	0,3095449	0,7315348	conserved Plasmodium protein, unknown function
PFIT_12473	190,1873915	286,653658	376,4944851	271,7409889	283,2241679	189,549638	165,7088089	177,3233443	266,30838	171,51608	0,6440506	-0,634754	0,3096348	0,7315348	conserved Plasmodium protein, unknown function
PFIT_10169	267,1680023	592,9671907	453,7880549	358,6361692	476,6455508	723,2044896	234,1537517	325,2508401	478,73416	279,7023	0,5842539	-0,775333	0,3100646	0,7321268	phosphomannomutase, putative
PFIT_11467	1274,70835	1887,982168	2062,61631	2064,346005	1755,989841	2000,476935	1097,520662	1235,573891	1841,0199	1166,5473	0,6336418	-0,65826	0,3102306	0,7321268	nucleolar preribosomal assembly protein, putative
PFIT_14440	62,26372935	120,4960067	166,4305092	157,1780873	122,9607363	207,0464466	79,25203905	86,2910392	139,39592	82,771539	0,5937874	-0,751982	0,3102737	0,7321268	conserved Plasmodium protein, unknown function
PFIT_13416	2319,606935	1295,649166	980,506333	778,6956647	784,7381822	1361,840712	665,2368126	808,8599608	1253,5062	737,04839	0,5879894	-0,766138	0,3105207	0,7324041	casein kinase II beta chain
PFIT_03053	207,1684085	68,49246695	104,0969851	125,078337	66,31590272	64,15523698	56,43705811	70,17073517	105,88456	63,303897	0,5978577	-0,742126	0,3112616	0,7338457	conserved Plasmodium protein, unknown function
PFIT_12271	2406,776156	1281,696997	1374,454205	1494,852161	1067,96235	1341,427682	995,453642	882,8237087	1494,5283	939,13868	0,6283847	-0,67028	0,3117086	0,7345932	conserved Plasmodium protein, unknown function
PFIT_04156	130,1877977	14,5863587	31,16676201	29,33253037	17,96055699	43,74220703	14,40946165	9,48253178	44,496035	11,945997	0,2684733	-1,89715	0,3123074	0,7355537	conserved Plasmodium protein, unknown function
PFIT_06241	91,69749231	48,83259218	36,77677918	42,61518563	88,42120363	20,41302995	91,25992375	75,86025424	54,792714	83,560089	1,5250219	0,60883	0,3123763	0,7355537	conserved Plasmodium protein, unknown function
PFIT_07120	14481,41138	6464,927853	7040,571539	6382,869297	4672,507979	7867,764971	4611,027727	4894,882905	7818,3422	4752,9553	0,6079237	-0,718038	0,3126034	0,7356127	conserved Plasmodium protein, unknown function
PFIT_10162	4646,006277	5134,398263	5463,533381	6888,717085	3553,427121	2496,221948	3153,270523	3280,955996	4697,0507	3217,1133	0,684922	-0,545988	0,3126614	0,7356127	Plasmodium exported protein (PHISTc), unknown function
PF_apicopla	46,41478006	36,78299151	44,25680206	22,69120274	15,19739437	5,832294271	57,63784658	45,51615254	28,529244	51,577	1,8078642	0,8542863	0,3128351	0,7357153	apicoplast ribosomal protein L6
PFIT_10110	474,3364108	236,5526868	276,1375114	384,6435586	198,9477082	221,6271823	206,5356169	176,3750911	298,70751	191,45535	0,6409459	-0,641726	0,313314	0,7365353	conserved Plasmodium membrane protein, unknown function
PFIT_10206	1667,535879	2199,369216	1814,528884	1927,645345	1482,436742	834,0180807	1080,709623	1145,489839	1654,2557	1113,0997	0,6728704	-0,571599	0,3137171	0,7371765	conserved Plasmodium protein, unknown function
PFIT_07041	5192,795027	8767,035769	6924,007849	5606,387408	1242,041595	1463,905862	2843,467098	3197,509716	4866,0289	3020,4884	0,6207296	-0,687963	0,3138974	0,7372939	conserved Plasmodium protein, unknown function
PF_apicopla	65,65993276	106,5438375	106,5903261	95,1923627	53,88167096	163,3042396	50,43311576	62,58470975	98,528728	56,508913	0,5735273	-0,802066	0,3142897	0,7377188	probable protein, unknown function
PFIT_05322	123,3953909	253,041614	220,0373398	179,86929	161,6450129	119,5620326	97,26386611	128,9624322	176,25845	113,11315	0,641746	-0,639926	0,3143418	0,7377188	conserved Plasmodium protein, unknown function
PFIT_14510	937,3521436	426,1753499	351,5610755	389,6245543	418,6191359	548,2356615	268,9766174	324,3025869	511,92799	296,6396	0,5794557	-0,78723	0,3144696	0,7377188	conserved Plasmodium protein, unknown function
PFIT_03020	2806,396092	2091,557	3030,655938	3092,6449	1946,648061	4146,761227	1637,875474	1836,766406	2852,4439	1737,3209	0,609064	-0,715334	0,3146377	0,7378071	serine/threonine protein kinase
PFIT_10237	0	7,610274105	6,856687643	9,408547476	35,92111397	40,8260599	28,81892329	19,91331674	16,770447	24,36612	1,4529201	0,5389554	0,314917	0,7379596	RNA binding protein, putative
PFIT_01019	4261,103223	1834,076059	762,9623341	1297,272664	961,5805894	904,005612	2690,966962	2614,334012	1670,1667	2652,6505	1,5882549	0,6674425	0,3150614	0,7379596	zinc-carboxypeptidase, putative
PFIT_11434	1293,953503	1342,57919	1493,511236	1228,645612	1363,62075	2064,632172	814,134583	935,9258867	1464,4904	875,03023	0,5974981	-0,742994	0,3150941	0,7379596	conserved protein, unknown function
PFIT_05152	110,942645	72,297604	103,4736499	86,89070316	132,6318054	160,3880924	56,43705811	64,4812161	111,10408	60,459137	0,5441667	-0,87788	0,3152618	0,7379959	rhomboid protease ROM9
PFIT_04190	105,282306	83,71301516	69,19021167	82,46315141	102,2370167	90,4005612	115,2756932	142,2379767	88,881044	128,75683	1,4486422	0,5347014	0,3153706	0,7379959	ribosome recycling factor, putative
PFIT_13570	1319,991062	598,6748963	637,0486156	483,7100291	486,3166199	1714,694516	344,626291	459,9027913	873,40596	402,26454	0,46057	-1,118508	0,3155994	0,738226	pyrroline carboxylate reductase
PFIT_05285	1531,687742	710,2922498	941,2362128	860,6053721	558,1588479	1073,142146	559,5674272	591,7099831	945,85376	575,63871	0,6085917	-0,716454	0,315805	0,7384014	conserved Plasmodium protein, unknown function
PFIT_12458	224,1494256	146,4977765	172,6638616	186,5106176	42,82902051	78,73597266	93,6615007	92,92881145	141,89778	93,295156	0,6574814	-0,604978	0,3160815	0,7386638	kinesin, putative
PFIT_10128	302,2621043	313,2896173	398,3112185	320,9975021	323,2900258	492,8288659	226,9490209	207,667446	358,49656	217,30823	0,6061655	-0,722216	0,3161784	0,7386638	conserved Plasmodium protein, unknown function
PFIT_13514	1171,69018	2312,254949	2609,904651	2603,953875	1905,200622	1627,210102	1305,257067	1425,224527	2038,3691	1365,2408	0,6697712	-0,57826	0,316365	0,738666	conserved Plasmodium protein, unknown function
PFIT_10181	7333,535249	3264,173402	4043,575704	3815,996168	2242,306461	3890,140279	2353,545402	2717,693608	4098,2879	2535,6195	0,6187021	-0,692683	0,3164434	0,738666	conserved Plasmodium protein, unknown function
PFIT_14702	221,88529	202,3064533	153,9638044	133,3799966	158,8818503	104,9812969	85,2559814	112,8421282	162,56645	99,049055	0,6092835	-0,714814	0,3165712	0,738666	conserved Plasmodium protein, unknown function
PFIT_06191	22,64135613	21,5624433	11,22003433	14,3895432	23,48688221	14,58073568	36,02365411	24,65458263	17,980166	30,339118	1,6873659	0,7547728	0,317505	0,7403773	conserved Plasmodium protein, unknown function
PFIT_14133	627,1655647	1187,20276	1023,516465	1004,500804	1178,488855	851,5149635	577,5792543	673,2597564	978,73157	625,41951	0,6390103	-0,646089	0,3176053	0,7403773	nuclear transport factor 2, putative
PFIT_14635	4283,744579	5745,75695	7835,947306	8731,132058	5594,022711	6202,644957	4322,838494	4176,106996	6398,8748	4249,4727	0,6640969	-0,590534	0,3176973	0,7403773	conserved Plasmodium protein, unknown function
PFIT_13024	13,58481368	23,46501182	15,58338101	11,62232335	46,97376443	14,58073568	33,62207717	32,24060805	20,968338	32,931343	1,5705271	0,6512488	0,3178965	0,7405197	SNARE protein, putative
PFIT_13168	959,9934997	557,4525782	188,8705778	123,4180051	301,1847248	533,6549258	199,3308861	183,9611165	444,09572	191,646	0,4315421	-1,212427	0,3181358	0,7405197	conserved Plasmodium protein, unknown function
PFIT_14072	28865,46492	9620,020659	11307,30126	12681,06167	7068,169965	11416,71604	7238,3529	7668,523451	13493,122	7453,438	0,5523879	-0,856246	0,3183434	0,7405197	conserved Plasmodium protein, unknown function
PFIT_13484	10632,38084	6818,171409	10247,00802	11229,37814	5501,456763	5412,369083	5508,016714	5646,847675	8306,794	5577,4322	0,6714302	-0,574691	0,3184019	0,7405197	glutamate--tRNA ligase, putative
PFIT_10036	9082,58001	8139,188156	7928,200921	7309,887945	5773,62828	9445,400572	4663,862419	5211,599466	7946,481	4937,7309	0,6213733	-0,686468	0,318413	0,7405197	60S ribosomal protein L13, putative
PFIT_11352	4186,386748	2194,2957	1794,582157	1723,977964	2220,20116	3061,954492	1480,572184	1484,964477	2530,233	1482,7683	0,5860205	-0,770977	0,3185481	0,7405295	alpha/beta hydrolase, putative
PFIT_07218	149,4329504	389,3923584	386,467849	330,9594936	377,1716967	504,4934544	171,7127513	254,1318517	356,31963	212,9223	0,5975598	-0,742845	0,318786	0,740778	conserved Plasmodium protein, unknown function
PFIT_10152	11,32067806	7,610274105	6,233352403	8,301659538	19,34213829	14,58073568	22,81498094	14,22379767	11,231473	18,519389	1,6488834	0,7214894	0,3193991	0,7418978	tubulin binding cofactor c, putative
PFIT_11259	117,7350519	70,39503547	43,01013158	59,2185047	222,4345904	11,66458854	159,7048666	120,4281536	87,40965	140,06651	1,6024147	0,6802476	0,3198244	0,7422581	conserved Plasmodium protein, unknown function
PFIT_12132	21,50928832	46,93002365	38,02344966	42,06174166	197,5661269	23,32917708	121,2796355	93,87706462	61,569968	107,57835	1,7472536	0,805089	0,3198969	0,7422581	dynein light chain 1, putative
PFIT_05067	1031,313772	734,3914512	668,2153776	783,6766604	911,8436624	1638,87469	1268,032625	1255,487208	961,38594	1261,7599	1,3124385	0,3922498	0,3200122	0,7422581	GTPase-activating protein, putative
PFIT_14704	348,6768843	828,885688	539,1849829	572,2610641	500,132433	419,9251875	350,6302334	359,3879545	534,84437	355,00909	0,6637615	-0,591263	0,3203894	0,7422581	small subunit rRNA processing KH domain protein, putative
PFIT_12255	3083,752704	4057,544477	4072,87246	3676,528287	3447,04536	3575,196388	2286,301248	2417,097351	3652,1566	2351,6993	0,6439207	-0,635045	0,320527	0,7422581	ubiquitin-activating enzyme E1 (UBA1)
PFIT_13643	552,4490895	1072,414459	913,8094623	1068,700305	708,7512103	612,3908984	517,5398308	586,020464	821,41924	551,78015	0,67174	-0,574025	0,3206258	0,7422581	conserved Plasmodium protein, unknown function
PFIT_02102	11033,13284	4448,205215	7024,364823	9431,792123	5497,312019	7509,078874	4587,011957	4844,625486	7490,6476	4715,8187	0,6295609	-0,667582	0,3208175	0,7422581	conserved Plasmodium protein, unknown function
PFIT_13524	4498,837462	12833,4589	10872,21326	6608,674436	8444,224946	15618,88406	5241,441674	6643,461765	9812,7155	5942,4517	0,6055869	-0,723594	0,3208384	0,7422581	membrane associated histidine-rich protein
PFIT_14208	47,54684786	84,34720467	87,89026888	156,0711993	198,9477082	157,4719453	175,3151167	173,5303316	122,04586	174,42272	1,4291572	0,5151646	0,3209316	0,7422581	glutathione reductase
PFIT_13374	8602,58326	6268,963294	6493,906533	6106,147312	4729,152813	8497,652753	4040,653203	4301,276415	6783,0677	4170,9648	0,6149083	-0,701557	0,3209524	0,7422581	60S ribosomal protein L6-2, putative
PFIT_03026	36,2261698	25,36758035	11,22003433	9,961991446	64,93432141	23,32917708	44,42917341	36,03362076	28,506546	40,231397	1,4113038	0,4970286	0,3209977	0,7422581	CDGSH iron-sulfur domain-containing protein, putative
PFIT_12140	26,03755954	188,3542841	163,9371682	106,8146861	84,27645971	34,99376562	51,63390423	66,37772246	100,73565	59,005813	0,585749	-0,771645	0,3212612	0,7423845	conserved protein, unknown function
PFIT_08181	6923,726703	8844,406889	10790,55634	10528,71807	7982,77679	9734,099138	5571,658503	6227,17862	9134,0473	5899,4186	0,6458712	-0,630681	0,3213149	0,7423845	importin beta, putative
PFIT_06294	167,5460353	414,1257492	461,2680778	389,6245543	371,6453715	481,1642773	219,7442901	256,9766112	380,89568	238,36045	0,6257893	-0,676251	0,3215688	0,7426678	microtubule-associated protein ytm1 homologue, putative
PFIT_04241	1,132067806	1,268379018	0	0,553443969	0	2,916147135	1,20078847	4,74126589	0,9783397	2,9710272	3,0368054	1,6025545	0,3219182	0,7431557	rifin (RIF)
PFIT_04163	43,01857664	19,65987477	9,350028604	12,72921129	52,50008965	0	36,02365411	36,98187394	22,876297	36,502764	1,5956588	0,6741522	0,3220429	0,7431557	conserved Plasmodium protein, unknown function
PFIT_08037	72,4523396	291,727174	490,5648341	610,448698	174,0792446	55,40679557	142,893828	145,0827362	282,44651	143,98828	0,5097896	-0,972026	0,3222657	0,7433454	Plasmodium exported protein (hyp7), unknown function, pseudogene
PFIT_09285	22,64135613	68,49246695	102,2269794	110,1353499	85,65804101	93,31670833	55,23626964	44,56789937	80,411817	49,902085	0,6205815	-0,688307	0,3224755	0,7433454	conserved Plasmodium protein, unknown function
PFIT_13107	3772,049931	2317,962655	2396,100664	2366,526412	3176,255424	3476,047385	4046,657145	3939,991955	2917,4904	3993,3246	1,3687533	0,4528624	0,3225194	0,7433454	26S proteasome regulatory subunit 7, putative
PFIT_08321	121,1312553	88,78653123	267,4108181	214,1828161	190,6582203	492,8288659	326,614464	291,1137257	229,16642	308,86409	1,3477721	0,4305765	0,3230672	0,7443047	prohibitin, putative
PFIT_04169	6166,373341	3560,974092	4397,006785	6924,690943	1948,029642	4922,456365	6374,98599	7489,3036	4653,2552	6932,1448	1,4897409	0,5750615	0,3232621	0,7444504	conserved Plasmodium protein, unknown function
PFIT_13417	150,5650182	147,131966	64,82686499	147,2160958	93,94752885	61,23908984	79,25203905	63,53296293	110,82109	71,392501	0,644214	-0,634388	0,3240249	0,7447574	sporozoite protein essential for cell traversal
PFIT_09292	3175,450197	1478,929934	2316,937088	2735,67354	1652,371243	2411,653681	1557,422646	1369,277589	2295,1693	1463,3501	0,6375783	-0,649326	0,3240559	0,7447574	conserved Plasmodium protein, unknown function
PFIT_14552	833,2019054	823,1779824	986,1163501	1067,593417	710,1327916	1417,247508	589,587139	612,571553	972,91166	601,07935	0,6178149	-0,694753	0,3240988	0,7447574	RNA binding protein, putative
PFIT_09044	57,73545812	19,65987477	16,83005149	22,69120274	9,671069147	34,99376562	33,62207717	45,51615254	26,930237	39,569115	1,4693192	0,5551479	0,3241583	0,7447574	conserved Plasmodium protein, unknown function
PFIT_13352	440,3743766	993,1407707	1069,019937	1168,320219	958,8174268	644,4685169	511,5358884	659,0359587	879,02354	585,28592	0,6658365	-0,58676	0,3241965	0,7447574	conserved Plasmodium protein, unknown function
PFIT_04062	32,82996638	46,29583414	19,32339245	20,47742686	38,68427659	8,748441406	44,42917341	47,4126589	27,726556	45,920916	1,656207	0,727883	0,3243931	0,7447574	conserved Plasmodium protein, unknown function
PFIT_06111	3509,410199	3353,594122	3860,315143	3380,435764	2916,518138	3858,06266	2183,033439	2236,929247	3479,7227	2209,9813	0,6351027	-0,654938	0,3247661	0,7447574	eukaryotic translation initiation factor 3 subunit L, putative
PFIT_09054	1678,856557	2151,170814	2138,039874	2136,847165	1403,686558	1621,377807	1244,016855	1227,987866	1854,9965	1236,0024	0,6663098	-0,585735	0,3248488	0,7447574	mitochondrial carrier protein, putative
PFIT_08231	641,8824462	1148,5172	910,6927861	723,3512677	925,6594755	740,7013724	521,1421962	570,8484132	848,46742	545,9953	0,6435077	-0,635971	0,3248692	0,7447574	phosphatidylglycerophosphate synthase
PFIT_12168	73,58440741	92,59166828	81,65691648	63,64605646	31,77637005	49,5745013	38,42523105	42,67139301	65,471653	40,548312	0,6193262	-0,691229	0,3249868	0,7447574	type IIB DNA topoisomerase, putative
PFIT_12100	1749,044761	1454,196544	1498,497918	1814,742775	1016,843842	982,7415846	877,7763719	1005,148369	1419,3446	941,46237	0,6633078	-0,59225	0,3250496	0,7447574	general transcription factor 3C polypeptide 5, putative
PFIT_10014	829,805702	2215,223954	3311,780132	4316,309156	2024,016614	1160,62656	1525,001357	1549,445693	2309,6271	1537,2235	0,6655722	-0,587333	0,3250987	0,7447574	Plasmodium exported protein (PHISTc), unknown function
PFIT_10378	44,15064444	60,88219284	39,27012014	29,33253037	29,01320744	23,32917708	20,413404	22,75807627	37,662979	21,58574	0,5731289	-0,803069	0,3251897	0,7447574	Plasmodium exported protein, unknown function
PFIT_07274	3,396203419	19,65987477	6,233352403	6,087883661	4,14474392	2,916147135	13,20867318	8,534278602	4,5362762	10,871476	2,3965639	1,2609674	0,325277	0,7447574	conserved Plasmodium protein, unknown function
PFIT_10227	289,8093584	686,1930485	872,0460012	778,1422207	656,2511207	1008,986909	431,0830609	469,3853231	715,23811	450,23419	0,6294885	-0,667748	0,3255671	0,7447574	orotidine monophosphate decarboxylase
PFIT_08197	30,56583077	8,878653123	1,246670481	2,213775877	5,526325227	2,916147135	9,606307764	21,80982309	8,5579004	15,708065	1,8355046	0,8761767	0,3257745	0,7447574	meiotic recombination protein DMC1, putative (DMC1)
PFIT_14322	80,37681425	39,9539905	49,24348398	50,3634012	214,1451025	52,49064844	128,4843663	109,9973686	81,095565	119,24087	1,4703747	0,5561839	0,3259415	0,7447574	mitochondrial ribosomal protein L17-2 precursor, putative
PFIT_12413	460,7515972	1007,727129	895,7327403	918,1635449	627,2379132	384,9314219	477,9138112	513,9532225	715,75739	495,93352	0,6928794	-0,529324	0,3260605	0,7447574	conserved Plasmodium protein, unknown function
PFIT_12453	510,5625806	501,6439014	404,5445709	448,843059	747,4354869	271,2016836	684,4494282	716,8794026	480,70521	700,66442	1,4575761	0,5435712	0,3263301	0,7447574	kinesin-13, putative
PFIT_12130	33,96203419	157,2789982	26,80341533	24,90497861	51,11850835	26,24532422	105,6693854	94,8253178	53,385543	100,24735	1,8777996	0,9090431	0,326392	0,7447574	conserved Plasmodium protein, unknown function
PFIT_01109	0	1,902568526	0	0,553443969	178,2239886	90,4005612	0	1,896506356	45,180094	0,9482532	0,0209883	-5,574271	0,3264486	0,7447574	5.8S ribosomal RNA
PFIT_13436	116,602984	139,5216919	163,9371682	68,07360821	183,7503138	309,1115964	170,5119628	188,7023824	146,46173	179,60717	1,2263079	0,2943212	0,3264698	0,7447574	conserved Plasmodium protein, unknown function
PFIT_12401	1,132067806	3,805137053	17,45338673	10,51543541	9,671069147	20,41302995	3,602365411	3,793012712	10,498354	3,6976891	0,3522161	-1,505467	0,3267306	0,7447574	rifin
PFIT_07144	1052,82306	603,1142228	740,5222655	663,5793191	2395,661986	1364,756859	1628,269166	1299,106854	1136,743	1463,688	1,2876156	0,364702	0,3271773	0,7447574	conserved Plasmodium protein, unknown function
PFIT_10104	99,62196695	292,9955531	230,6340389	244,0687904	290,1320744	556,9841029	154,9017127	161,2030403	285,73942	158,05238	0,5531347	-0,854297	0,3272641	0,7447574	conserved Plasmodium protein, unknown function
PFIT_09214	106,4143738	222,6005176	266,1641476	266,7599932	232,1056595	344,105362	134,4883087	158,3582807	239,69168	146,42329	0,6108819	-0,710085	0,3273112	0,7447574	conserved Plasmodium protein, unknown function
PFIT_12438	9150,504078	11195,3474	8832,03702	14368,51233	7768,631687	5572,757176	15281,23408	14690,33823	9481,2983	14985,786	1,5805627	0,6604382	0,3274084	0,7447574	translation initiation factor SUI1, putative
PFIT_04021	10456,91033	5951,23435	5446,079994	6458,691121	4365,796929	3481,87968	3836,519163	4038,610285	6026,7654	3937,5647	0,6533463	-0,61408	0,3274226	0,7447574	conserved Plasmodium protein, unknown function
PFIT_13447	2,264135613	32,34366495	22,44006865	16,60331908	172,7003289	84,56826693	75,64967364	49,30916526	43,409412	62,479419	1,4393058	0,5253732	0,3274336	0,7447574	ubiquitin conjugating enzyme
PFIT_12270	422,2612917	213,087675	176,403873	176,5486262	339,8690014	87,48441406	360,2365411	322,4060805	235,94248	341,32131	1,4466293	0,5326953	0,327466	0,7447574	conserved Plasmodium protein, unknown function
PFIT_14155	10264,4588	3997,930663	3848,471774	3999,186121	3003,557761	4590,015591	6953,766032	6842,594933	4950,6035	6898,1805	1,3934019	0,4786115	0,3275066	0,7447574	atypical protein kinase, ABC-1 family, putative
PFIT_11399	104,1502382	155,3764297	156,4571453	116,7766775	102,2370167	37,90991276	69,64573129	78,70501378	112,15124	74,175373	0,661387	-0,596433	0,327609	0,7447574	conserved Plasmodium protein, unknown function
PFIT_14107	150,5650182	246,6997189	226,2706922	204,7742686	182,3687325	174,9688281	151,2993473	109,0491155	197,60788	130,17423	0,6587502	-0,602197	0,3281618	0,7457144	conserved Plasmodium protein, unknown function
PFIT_13510	264,9038667	526,3772923	616,4785526	664,132763	580,2641488	580,3132799	344,626291	353,6984354	538,74498	349,16236	0,6481032	-0,625704	0,328937	0,7466126	conserved Plasmodium protein, unknown function
PFIT_03132	27,16962735	17,7573625	34,90677346	24,35153464	31,77637005	34,99376562	14,40946165	14,22379767	28,492563	14,31663	0,502469	-0,992893	0,3289521	0,7466126	apicoplast conserved ycf19 protein precursor, unknown function
PFIT_14088	6062,223103	13265,97614	15623,8978	15992,31693	9845,148391	9757,428315	7371,64042	8680,309592	11757,832	8025,975	0,6826067	-0,550873	0,3290451	0,7466126	plasmepsin IV
PFIT_10034	7531,647115	4941,604652	5057,74214	4189,017403	2875,07069	6304,710107	2925,120714	3417,504454	5149,9654	3171,3126	0,615793	-0,699483	0,329181	0,7466126	U5 small nuclear ribonuclear protein, putative
PFIT_06109	57,73545812	93,22585779	139,6270938	105,1543541	91,18436624	195,3818581	163,307232	161,2030403	113,71816	162,25514	1,4268181	0,5128014	0,3292171	0,7466126	lsm12, putative
PFIT_06275	1144,520552	1201,15493	1511,587958	1572,334316	1178,488855	1921,740962	868,1700641	915,0643168	1421,6379	891,61719	0,627176	-0,673058	0,329583	0,7467615	conserved Plasmodium protein, unknown function
PFIT_04006	779,9947185	172,4995464	215,6739931	327,0853858	49,73692704	93,31670833	69,64573129	101,46309	273,05121	85,554411	0,3133273	-1,674257	0,3296608	0,7467615	acyl-CoA synthetase
PFIT_05029	130,1877977	289,190416	212,5573169	149,4298717	156,1186877	154,5557982	97,26386611	134,6519513	182,00665	115,95791	0,6371081	-0,65039	0,329679	0,7467615	DnaJ protein, putative

PFIT_07031	2858,471211	2256,446272	2327,533787	2083,716544	2149,740513	3216,51029	1422,934337	1595,910099	2482,0698	1509,4222	0,6081305	-0,717547	0,3298109	0,7467615 tRNA m5C-methyltransferase, putative
PFIT_13519	3749,408574	3318,713699	3378,477002	3737,960568	2104,14833	3572,280241	1982,501765	2280,548893	3310,1647	2131,5253	0,6439333	-0,635017	0,3303349	0,7476487 P-loop containing nucleoside triphosphate hydrolase, putative
PFIT_05324	6276,183918	4700,612639	5272,169462	4833,226183	4248,362518	3858,06266	3010,376695	3289,490275	4864,7696	3149,9335	0,647499	-0,62705	0,331006	0,748344 eukaryotic translation initiation factor 3, subunit 6, putative
PFIT_10091	136,9802046	161,0841352	237,4907266	246,2825663	262,5004483	629,8877812	121,2796355	131,8071917	279,03764	126,54341	0,4534994	-1,140827	0,3310152	0,748344 protein phosphatase, putative
PFIT_03044	11,32067806	22,83082232	44,25680206	47,59618135	107,7633419	102,0651497	85,2559814	67,32597564	55,972163	76,290979	1,3630165	0,446803	0,3311166	0,748344 1-cys-glutaredoxin-like protein-1
PFIT_13361	1267,915943	899,2807234	1090,836671	814,6695227	772,3039504	1189,788031	582,3824082	657,1394524	1005,7991	619,76093	0,6161876	-0,698559	0,3311713	0,748344 tryptophan--tRNA ligase, putative
PFIT_14675	11636,52498	13376,32512	16762,10795	17582,9149	9589,555849	11326,31547	8732,133757	9384,861703	13378,957	9058,4977	0,6770705	-0,562622	0,3316058	0,7487246 transcription factor with AP2 domain(s)
PF_apicopla	2,264135613	13,31797968	14,33671053	12,17576732	2,763162613	2,916147135	1,20078847	4,74126589	7,9623171	2,9710272	0,373136	-1,422227	0,3318725	0,7487246 hypothetical protein
PFIT_14255	0	3,170947544	5,610017163	2,213775877	15,19739437	8,748441406	0	1,896506356	5,8234294	0,9482532	0,1628342	-2,618525	0,3318804	0,7487246 small nucleolar RNA snoR27
PFIT_14471	3346,392435	8368,130568	11414,51492	12280,92168	7724,421085	16100,04833	5852,643005	6617,858929	9872,4048	6235,251	0,6315838	-0,662954	0,3320012	0,7487246 DEAD/DEAH box ATP-dependent RNA helicase, putative
PFIT_11242	1986,779	1369,849339	1279,707248	1255,764366	1146,712485	906,9217591	830,9456216	879,9789492	1324,289	855,46229	0,6459785	-0,630442	0,3320016	0,7487246 RING zinc finger protein, putative
PFIT_06228	109,8105772	105,909648	119,0570309	116,7766775	129,8686428	113,7297383	80,45282752	62,58470975	115,85872	71,518769	0,6172929	-0,695973	0,3322367	0,748956 ribonuclease H2 subunit A, putative
PFIT_12240	165913,5936	53623,02601	107635,6593	131172,3086	46679,48761	96442,81806	64818,56164	64935,42938	99911,149	64876,996	0,6493469	-0,622939	0,3324681	0,7491032 polyadenylate-binding protein, putative
PFIT_06052	3,396203419	58,97962432	86,02026316	68,07360821	31,77637005	5,832294271	96,06307764	70,17073517	42,346394	83,116906	1,9627859	0,9729028	0,3326653	0,7491032 conserved Plasmodium protein, unknown function
PFIT_14355	5550,528454	3343,44709	3506,884062	3185,623487	2526,91221	5042,018397	2382,364325	2346,926616	3859,2356	2364,6455	0,6127238	-0,706691	0,3326993	0,7491032 Hsp70/Hsp90 organizing protein
PFIT_08106	1768,289913	754,6855154	466,878095	444,4155073	422,7638798	816,5211979	1104,725393	1070,577838	778,92568	1087,6516	1,3963484	0,4816589	0,3328653	0,7491788 serine protease, putative
PF_apicopla	19,24515271	19,65987477	19,94672769	14,94298717	142,3028746	23,32917708	60,03942352	72,06724153	39,904466	66,053333	1,6552867	0,7270811	0,3333048	0,7498695 apicoplast ribosomal protein L2
PFIT_11364	515,0908519	840,9352886	926,8995023	877,2086912	384,0796032	519,0741901	469,5082919	471,2818295	677,21469	470,39506	0,6946026	-0,52574	0,3335288	0,7500751 conserved Plasmodium protein, unknown function
PFIT_10159	10709,36145	7442,848075	10801,15304	11219,96959	8341,98793	14606,981	6252,505566	6775,268957	10520,384	6513,8873	0,6191682	-0,691597	0,333705	0,7501731 serine/threonine protein kinase, FIKK family
PFIT_07254	24750,39845	9893,990526	13528,86806	14552,80917	8458,040759	12276,97944	8607,251756	9114,609547	13910,181	8860,9307	0,6370104	-0,650611	0,3342027	0,7507073 ubiquitin carboxyl-terminal hydrolase, putative
PFIT_14469	2775,830261	2921,076877	3821,045023	4052,870186	2518,622722	3706,423009	2107,383766	2209,429905	3299,3113	2158,4068	0,6541992	-0,612198	0,3342924	0,7507073 conserved Plasmodium protein, unknown function
PFIT_13360	6552,408463	1367,94677	1905,53583	1773,234477	1389,870794	2414,569828	970,2370841	1104,714952	2567,261	1037,476	0,4041179	-1,307152	0,3344296	0,7507073 nuclear movement protein, putative
PFIT_13614	6247,882223	4004,272558	4370,826705	4279,782214	3488,492799	3394,395266	2722,187463	2842,863028	4297,6086	2782,5252	0,647459	-0,627139	0,3345423	0,7507073 calpain
PFIT_14588	804,9002103	1005,190371	1074,629954	902,6671138	895,2646867	851,5149635	572,7761004	624,8988443	922,36122	598,83747	0,649244	-0,623167	0,3346063	0,7507073 conserved Plasmodium protein, unknown function
PFIT_14558	401,8840712	188,3542841	176,403873	166,0331908	205,8556147	452,002806	138,0906741	152,6687617	265,08897	145,37972	0,5484186	-0,866651	0,3353181	0,752006 mitochondrial ribosomal protein S11 precursor, putative
PFIT_05291	2143,004357	1395,851109	1756,558707	1703,500537	1113,554533	2426,234417	1092,717508	1087,646395	1756,4506	1090,182	0,6206733	-0,688094	0,3360159	0,7531911 tRNA-YW synthesizing protein, putative
PFIT_08234	2836,961923	2739,064488	3228,876545	3320,110371	2352,832965	2286,259354	1866,025283	1869,955267	2794,0176	1867,9903	0,6685678	-0,580854	0,3361128	0,7531911 C-13 antigen
PFIT_08279	1461,499538	790,8343174	890,1227231	1016,123127	1094,212395	933,1670833	1565,828165	1362,639817	1030,9932	1464,234	1,4202169	0,5061113	0,3367176	0,7541824 conserved Plasmodium protein, unknown function
PFIT_11466	21,50928832	49,46678168	29,92009153	34,31352609	46,97376443	43,74220703	72,04730823	46,46440572	37,654277	59,255857	1,573682	0,654144	0,3368219	0,7541824 conserved Plasmodium protein, unknown function
PFIT_04206	10313,13772	14705,58633	20768,28354	21490,22932	16359,30425	36758,03464	11905,81768	12504,61466	20065,763	12205,216	0,6082608	-0,717238	0,3381964	0,7569603 40S ribosomal protein S19, putative
PFIT_10279	3663,371421	3139,872258	3605,994365	4190,677735	2934,478695	2933,644018	2304,313075	2233,136234	3411,3397	2268,7247	0,6650539	-0,588457	0,3384728	0,7572794 conserved Plasmodium protein, unknown function
PFIT_13583	225,2814934	142,6926395	107,2136613	106,2612421	205,8556147	134,1427682	213,7403477	210,5122055	153,57457	212,12628	1,3812591	0,465984	0,3386613	0,7574014 high mobility group protein B4, putative (HMGB4)
PFIT_13251	796,9757356	294,8981216	238,1140618	234,6602429	682,5011655	218,7110352	600,3942352	522,4875011	410,97673	561,44087	1,3661135	0,4500774	0,3389016	0,7576392 conserved Plasmodium protein, unknown function
PFIT_12372	4185,25468	3233,098116	4809,031379	5304,207001	3235,66342	4721,242212	2751,006386	2799,243381	4248,0828	2775,1249	0,6532653	-0,614259	0,3391143	0,7578153 conserved Plasmodium protein, unknown function
PFIT_12507	78,11267863	204,8432113	103,4736499	101,2802464	204,4740334	119,5620326	205,3348284	191,547142	135,29098	198,44099	1,4667718	0,5526444	0,3392776	0,7578808 adaptor-related protein complex 3, sigma 2 subunit, putative
PFIT_05287	168,6781031	52,63772923	41,7634611	37,63418991	38,68427659	14,58073568	14,40946165	23,70632945	58,996416	19,057896	0,3230348	-1,630238	0,3394531	0,7579735 conserved Plasmodium protein, unknown function
PFIT_09351	88,30128889	201,6722638	69,19021167	79,1424876	105,0001793	43,74220703	139,2914626	166,8925593	97,84144	153,09201	1,564695	0,6458815	0,3398476	0,7585549 conserved Plasmodium protein, unknown function
PFIT_12115	8353,528343	13612,87781	10984,4136	10922,21673	11797,32278	15723,86535	16555,27064	16822,95963	11899,037	16689,115	1,4025601	0,4880626	0,3401413	0,7585874 non-SERCA-type Ca2 -transporting P-ATPase
PFIT_14518	1196,595671	1449,123028	2042,046247	1872,854392	1341,515449	2195,858793	1030,276508	1132,214295	1682,9989	1081,2454	0,6424516	-0,63834	0,340405	0,7585874 conserved Plasmodium protein, unknown function
PFIT_14202	504,9022416	140,7900709	250,5807666	254,5842258	285,9873305	271,2016836	175,3151167	166,8925593	284,67439	171,10384	0,601051	-0,734441	0,340563	0,7585874 conserved Plasmodium protein, unknown function
PFIT_12017	2089,79717	873,9131431	920,6661499	624,2847973	634,1458198	1767,185164	619,6068508	626,7953507	1151,6654	623,2011	0,5411304	-0,885952	0,3406141	0,7585874 ATP-dependent RNA helicase, putative
PFIT_03175	1831,685711	1396,485298	2513,911024	2682,542919	1463,094604	2178,36191	1386,910683	1291,520828	2011,0136	1339,2158	0,6659407	-0,586534	0,3406584	0,7585874 kinesin-5, putative
PFIT_13474	375,8465117	287,2878475	304,1875973	427,2587442	219,6714278	288,6985664	222,145867	197,236661	317,15845	209,69126	0,6611562	-0,596937	0,3407968	0,7585874 step II splicing factor, putative
PFIT_08222	59,99959373	71,02922498	61,08685355	50,91684517	70,46064664	81,65211979	40,826808	35,08536759	65,857547	37,956088	0,5763362	-0,795017	0,340801	0,7585874 conserved Plasmodium protein, unknown function
PFIT_04195	53,2071869	6,341895088	4,363346682	8,301659538	17,96055699	8,748441406	22,81498094	30,3441017	16,487181	26,579541	1,6121338	0,6889715	0,3410738	0,7588959 erythrocyte membrane protein 1, PfEMP1, putative
PFIT_06022	1436,594046	496,5703854	735,5355835	658,0448794	373,0269528	1251,027121	467,106715	478,8678549	825,13316	472,98728	0,5732254	-0,802826	0,3415799	0,7597232 dihydroorotate dehydrogenase, mitochondrial precursor (DHODH)
PFIT_09135	41,88650883	34,24623347	65,45020023	63,64605646	38,68427659	125,3943268	28,81892329	36,98187394	61,551267	32,900399	0,5345202	-0,903683	0,3421069	0,7600501 conserved Plasmodium protein, unknown function
PFIT_05283	3358,845181	2206,979491	3626,564428	4079,988941	1967,371781	2131,703556	1980,100188	1984,693902	2895,2422	1982,397	0,6847085	-0,546438	0,3422382	0,7600501 conserved Plasmodium protein, unknown function
PFIT_09297	0	19,02568526	0	3,874107784	19,34213829	0	10,80709623	18,01681038	7,0403219	14,411953	2,0470589	1,0335526	0,3422639	0,7600501 conserved protein, unknown function
PFIT_03176	3288,656977	7348,353838	8183,76837	6602,033109	8717,778045	11731,65993	4178,743877	5165,135061	7645,375	4671,9395	0,6110805	-0,710566	0,3424402	0,7600501 40S ribosomal protein S11, putative
PFIT_13569	2344,512427	4824,913783	5289,622849	4441,387853	4244,217774	7841,519647	2668,151981	3294,23154	4831,0291	2981,1918	0,6170925	-0,696441	0,3428279	0,7600501 TCP-1/cpn60 chaperonin family, putative
PFIT_12157	352,0730878	329,1443551	412,0245938	358,0782481	370,2637902	484,0804245	241,3584826	237,0632945	384,27742	239,21089	0,6224953	-0,683865	0,3428352	0,7600501 serine--tRNA ligase, putative
PFIT_11105	5782,602354	7512,608921	10709,52276	11086,03615	6313,826571	8894,248763	5183,803827	6042,26925	8383,1409	5613,0365	0,6695625	-0,578709	0,3429133	0,7600501 U3/U14 snoRNA-associated small subunit rRNA processing protein, putative
PFIT_02178	2922,999076	2449,874072	2879,185475	3002,986977	2247,832786	5645,660854	1787,974033	2024,520535	3191,4232	1906,2473	0,5973032	-0,743465	0,3429212	0,7600501 DEAD/DEAH box ATP-dependent RNA helicase, putative
PFIT_13085	18452,70524	3150,01929	4153,906041	4374,421133	3500,927031	4557,937973	2042,541188	2065,295422	6364,9861	2053,9183	0,3226901	-1,631779	0,3429363	0,7600501 U4/U6 small nuclear ribonucleoprotein PRP3, putative
PFIT_14253	28048,11197	18680,68617	27669,22798	27176,31266	18008,91233	37274,19268	15503,37994	17113,1251	26142,907	16308,253	0,6238117	-0,680817	0,3431755	0,7602822 60S ribosomal protein L5, putative
PFIT_11144	1009,804483	779,4189063	1035,983169	1059,845201	616,1852628	976,9092904	599,1934468	596,451249	913,02439	597,82235	0,6547715	-0,610937	0,3433248	0,7603151 Rpr2, RNAse P, putative
PFIT_14527	83,77301766	225,7714651	375,2478147	382,9832267	205,8556147	160,3880924	169,3111743	167,8408125	239,00321	168,57599	0,7053294	-0,503631	0,3436196	0,7606574 pre-mRNA-splicing factor cwf18, putative
PFIT_02050	121,1312553	277,7750048	383,3511728	405,1209855	223,8161717	291,6147135	187,3230014	196,2884078	283,80155	191,8057	0,6758445	-0,565237	0,3439027	0,7606574 conserved Plasmodium protein, unknown function
PFIT_09209	7880,324	6219,496513	6739,500618	5433,71289	5292,837986	9174,198888	3876,145183	4476,703253	6790,0118	4176,4242	0,6150835	-0,701146	0,3439266	0,7606574 inosine-5'-monophosphate dehydrogenase
PFIT_08100	3671,295896	2214,589765	1514,081299	1473,82129	1388,489213	1720,52681	1232,008971	1285,831309	1997,134	1258,9201	0,6303634	-0,665744	0,3440617	0,7606574 RNA helicase, putative
PFIT_12134	2201,871883	4212,920907	5238,509359	4803,340209	3111,321103	5368,626876	2708,978789	2788,812597	4156,0984	2748,8957	0,6614126	-0,596378	0,3441959	0,7606574 proline--tRNA ligase, putative
PFIT_03096	904,5221772	702,0477862	864,5659783	901,5602258	653,487958	863,1795521	497,1264268	550,9350964	814,89395	524,03076	0,6430662	-0,636961	0,3442862	0,7606574 SECIS-binding protein 2, putative
PFIT_05170	18989,30538	11270,18176	11538,55863	9205,43354	8967,844261	20786,29678	7275,577343	8629,10392	13459,603	7952,3406	0,5908302	-0,759184	0,344678	0,7612257 60S ribosomal protein L8, putative
PFIT_07281	0	10,14703214	13,71337529	11,62232335	38,68427659	17,49688281	24,01576941	22,75807627	15,277315	23,386923	1,5308268	0,614311	0,3449017	0,7612336 1-cys peroxiredoxin
PFIT_10107	65,65993276	70,39503547	45,50347254	44,27551754	81,51329709	104,9812969	30,01971176	42,67139301	68,721425	36,345552	0,5288824	-0,918981	0,3450526	0,7612336 conserved Plasmodium protein, unknown function
PFIT_06288	609,0524798	1357,799738	1451,747775	1399,659798	1203,357318	752,3659609	655,6305049	882,8237087	1128,9972	769,22711	0,6813366	-0,55356	0,3450854	0,7612336 sec14-like cytosolic factor or phosphatidylinositol/phosphatidylcholine transfer protein, putative
PFIT_14656	4141,104035	1986,281541	1203,037014	1405,194238	2778,360008	1968,399316	3255,337543	2723,383127	2247,0627	2989,3603	1,3303413	0,4117964	0,3452379	0,7612731 conserved Plasmodium membrane protein, unknown function
Pf_M76611	7,924474644	8,244463614	3,116676201	4,980995723	11,05265045	2,916147135	16,81103859	8,534278602	6,372568	12,672659	1,9886267	0,9917725	0,3456958	0,7617126 unspecified product
PFIT_14523	31545,06942	32667,73579	31841,21074	26339,50538	23930,36981	47489,4561	18137,90985	21679,91241	32302,225	19908,911	0,6163325	-0,698219	0,3457065	0,7617126 elongation factor 2
PFIT_12332	1186,407061	1451,659786	1848,812323	1909,935138	1029,278073	979,8254375	1030,276508	939,7188994	1400,9863	984,9977	0,7030745	-0,508251	0,3461914	0,761877 conserved Plasmodium membrane protein, unknown function
PFIT_11342	20,37722051	65,3215194	51,1134897	65,85983233	31,77637005	49,5745013	79,25203905	75,86025424	47,337156	77,556147	1,6383778	0,7122681	0,3462634	0,761877 conserved Plasmodium protein, unknown function
PFIT_bin06	37,35823761	27,90433839	61,08685355	43,1686296	69,07906533	40,8260599	27,61813482	25,60283581	46,570531	26,610485	0,5714018	-0,807423	0,3464155	0,761877 unspecified product
PFIT_11049	380,3747829	705,8529233	781,0390561	783,1232164	799,9355765	609,4747513	423,8783301	470,3335763	676,63338	447,10595	0,6607802	-0,597758	0,34651	0,761877 syntaxin, Qa-SNARE family
PFIT_14257	14,71688148	10,78122165	10,59669908	28,7790864	114,6712485	177,8849753	9,606307764	6,637772246	59,571685	8,12204	0,1363406	-2,874713	0,3467654	0,761877 small nucleolar RNA snoR29
PFIT_14327	166,4139675	241,6262028	225,647357	226,9120274	201,7108708	297,4470078	314,6065793	331,8886123	226,62624	323,2476	1,4263467	0,5123247	0,346768	0,761877 mitogen-activated protein kinase 1
PFIT_14227	3,396203419	4,439326561	3,116676201	1,106887938	2,763162613	17,49688281	0	0,948253178	5,3865233	0,4741266	0,0880209	-3,50601	0,3469448	0,761877 unspecified product
PFIT_04113	193,5835949	46,29583414	43,63346682	37,63418991	27,63162613	113,7297383	88,85834681	126,1176727	77,084742	107,48801	1,3944136	0,4796585	0,3470304	0,761877 erythrocyte membrane protein 1, PfEMP1
PFIT_11282	166,4139675	322,1682705	450,6713787	337,0473772	219,6714278	349,9376562	204,13404	205,7709396	307,65168	204,95249	0,6661836	-0,586008	0,3472485	0,761877 methyltransferase, putative
PFIT_14216	103,0181704	194,0619897	200,7139474	145,5557639	305,3294688	431,589776	128,4843663	124,2211663	230,04485	126,35277	0,5492527	-0,864458	0,3475017	0,761877 pantothenate kinase, putative (PANK)
PFIT_14370	2,264135613	24,73339084	24,93340961	15,49643114	22,10530091	34,99376562	30,01971176	34,13711441	20,754406	32,078413	1,5456195	0,6281852	0,3478624	0,761877 GTPase, putative
PFIT_13578	491,3174279	1338,139864	1123,873438	1018,890347	1577,765852	2315,420826	817,7369484	754,8095297	1310,9013	786,27324	0,5997959	-0,737456	0,3479497	0,761877 HVA22-like protein, putative
PFIT_13020	1469,424013	3118,309815	3106,079502	2860,751877	3213,558119	4330,478496	1814,391379	1981,849142	3016,4336	1898,1203	0,6292597	-0,668272	0,3480148	0,761877 40S ribosomal protein S7, putative
PFIT_03028	7724,098642	4851,549742	6427,209663	6703,313355	5006,850655	5639,82856	3842,523105	4065,161374	6058,8084	3953,8422	0,6525775	-0,615779	0,3480921	0,761877 exportin 1, putative
PFIT_08114	6,792406838	0,634189509	0	0,553443969	23,48688221	17,49688281	0	0,948253178	8,1606342	0,4741266	0,0580992	-4,105337	0,3481112	0,761877 Plasmodium RNA of unknown function RUF6-4
PFIT_09248	838,8622445	1177,689918	987,9863559	1103,567275	1095,593976	1277,272445	694,0557359	693,1730731	1080,162	693,6144	0,6421392	-0,639042	0,3481637	0,761877 splicing factor 3A subunit 3, putative
PFIT_06183	2227,909443	5014,536446	3548,024188	2865,179429	5403,36449	5601,918647	5433,567829	5660,12322	4110,1554	5546,8455	1,3495464	0,4324746	0,3482832	0,761877 cell division cycle protein 48 homologue, putative
PFIT_10259	112,0747128	60,88219284	51,1134897	70,84082806	69,07906533	14,58073568	109,2717508	89,13579873	63,095171	99,203775	1,5722879	0,6528654	0,348532	0,761877 cell division cycle protein 20 homolog, putative (CDC20)
PFIT_10239	59,99959373	32,34366495	19,94672769	29,88597434	63,55274011	11,66458854	67,24415435	43,61964619	36,232215	55,4319	1,5299065	0,6134435	0,3486118	0,761877 conserved Plasmodium protein, unknown function
PFIT_11142	2613,944565	5603,6985	6131,748759	7856,690587	5968,431245	8611,382491	8806,582642	8681,257845	6130,9827	8743,9202	1,4261858	0,5121619	0,3487483	0,761877 ubiquitin domain containing protein
PF_apicopla	207,1684085	172,4995464	167,0538444	166,0331908	305,3294688	116,6458854	315,8073677	234,218535	189,12172	275,01295	1,4541584	0,5401845	0,3488631	0,761877 RNA polymerase beta subunit, putative
PFIT_13395	7590,514641	2440,995419	4170,112758	4506,140797	2193,951115	6747,964471	2714,982732	2890,275687	4608,2799	2802,6292	0,6081725	-0,717447	0,3490343	0,761877 nucleolar complex protein 2, putative
PFIT_09229	4900,721533	4112,718964	4542,867231	4620,150255	3806,2565	3242,755615	2850,671829	2781,226571	4204,245	2815,9492	0,6697871	-0,578225	0,3491265	0,761877 conserved Plasmodium protein, unknown function
PFIT_04145	13514,62547	13709,27461	15620,78112	12925,6839	13985,74757	31019,05708	9312,114588	10500,95569	16795,862	9906,5351	0,58982	-0,761653	0,3491408	0,761877 60S ribosomal protein L15, putative
PFIT_01032	61,13166154	28,5385279	11,84336957	19,37053892	16,57897568	14,58073568	9,606307764	13,27554449	25,340635	11,440926	0,4514854	-1,147249	0,349105	0,761877 transporter, putative
PFIT_bin10	938,4842114	383,6846528	359,6644336	214,73626	449,0139247	2615,78398	876,5755834	1264,969739	826,89458	1070,7727	1,2949325	0,3728769	0,3493647	0,761877 erythrocyte membrane protein 1, PfEMP1 (VAR)
PFIT_14648	419,9971561	548,5739251	491,1881694	442,7551754	332,9610949	373,2668333	302,5986946	282,579447	434,79039	292,58907	0,6729428	-0,571444	0,3496354	0,761877 cyclin homologue
PFIT_08105	10042,57351	7558,904755	8563,379531	8357,557379	5770,865118	12332,38624	5371,126828	5637,365143	8770,9444	5504,246	0,6275545	-0,672187	0,3496957	0,761877 conserved Plasmodium protein, unknown function
PFIT_06160	1312,066587	1198,618172	1242,307134	1005,054248	958,8174268	1563,054865	644,8234086	858,1691261	1213,3197	751,49627	0,619372	-0,691122	0,3498409	0,761877 mitochondrial import receptor subunit tom40, putative
PFIT_11136	147,1688148	105,909648	130,9004005	94,63891873	88,42120363	93,31670833	70,84651976	67,32597564	110,05928	69,086248	0,6277185	-0,67181	0,3499279	0,761877 conserved Plasmodium protein, unknown function
PFIT_02145	168,6781031	344,3649033	324,7576602	259,0117776	392,3690911	443,2543646	425,0791185	459,9027913	322,07265	442,49095	1,3738855	0,4582618	0,3499569	0,761877 RING zinc finger protein, putative
PFIT_12085	1109,42645	1790,951173	1459,227798	1513,115812	2543,491186	2172,529616	2484,431345	2326,065046	1764,7903	2405,2482	1,3629088	0,446689	0,3505312	0,7625627 conserved Plasmodium protein, unknown function
PFIT_11156	47,54684786	135,7165549	92,25361556	116,2232335	138,1581307	64,15523698	151,2993473	154,565268	99,008937	152,93231	1,5446314	0,6272626	0,3505664	0,7625627 glycerol-3-phosphate dehydrogenase, putative
PFIT_04147	427,9216308	535,8901349	360,9111041	361,3989119	899,4094306	1589,300189	951,0244686	786,1018846	695,80523	868,56318	1,2482849	0,3199473	0,3510665	0,7625627 glutamyl-tRNA(Gln) amidotransferase subunit A, putative
PFIT_06178	80,37681425	71,66341449	30,54342677	45,93584944	172,6976633	46,65835417	116,4764816	91,98055827	74,64592	104,2285	1,3963056	0,4816148	0,3511341	0,7625627 N-acetylglucosamine transferase
PFIT_07174	12,45274587	25,36758035	64,20352975	62,53916852	30,39478875	81,65211979	28,81892329	24,65458263	46,101656	26,736753	0,5799521	-0,785994	0,351179	0,7625627 NIMA related kinase 4
PFIT_13045	10553,13609	6461,756905	6891,594417	6110,02142	4615,863146	8876,75188	4286,81484	4791,523309	7251,5206	4539,1691	0,625961	-0,675855	0,3512303	0,7625627 conserved Plasmodium protein, unknown function
PFIT_09273	275,0924769	628,4818032	193,2339245	765,4130094	371,6453715	104,9812969	624,4100046	665,673731	389,80798	645,04187	1,6547682	0,7266292	0,351376	0,7625627 zinc finger protein, putative
PFIT_07233	2046,778594	2075,702262	2513,911024	2569,086905	1961,845455	2047,135289	1403,721722	1528,584123	2202,4099	1466,1529	0,6657039	-0,587047	0,3515702	0,7625627 conserved Plasmodium membrane protein, unknown function
PFIT_13061	2384,1348	1052,754585	1464,837815	1549,643114	1232,370526	1434,744391	939,0165839	997,5623433	1519,7475	968,28946	0,6371384	-0,650321	0,3516494	0,7625627 U1 small nuclear ribonucleoprotein a, putative
PFIT_13181	44,15064444	98,93356337	224,4006865	286,1305321	60,78957749	104,9812969	90,05913528	72,06724153	136,56438	81,063188	0,5935895	-0,752462	0,3517608	0,7625627 conserved Plasmodium protein, unknown function
PFIT_11403	86,03715328	86,24977319	137,1337529	102,3871343	110,5265045	58,32294271	163,307232	138,444964	96,77621	150,8761	1,5590205	0,6406399	0,3519045	0,7625627 conserved Plasmodium protein, unknown function
PFIT_10188	532,071869	374,1718102	431,3479863	382,9832267	483,5534573	431,589776	685,6502166	556,6246155	439,28635	621,13742	1,4139693	0,4997508	0,3520578	0,7625627 zinc finger protein, putative
PFIT_12183	6494,673005	3148,750911	3499,404039	3519,3502	2188,42479	3283,581674	2262,285478	2492,009352	3689,0308	2377,1474	0,6443826	-0,63401	0,3520978	0,7625627 arginine--tRNA ligase, putative
PFIT_09245	3791,295083	5100,15203	4060,405755	6398,919172	3036,715712	2881,15337	6679,986261	6397,864192	4211,4402	6538,9252	1,5526577	0,6347398	0,3521594	0,7625627 conserved Plasmodium protein, unknown function
PFIT_02096	33,96203419	80,54206762	119,0570309	117,3301215	207,237196	242,0402122	199,3308861	151,7205085	133,36144	175,5257	1,3161652	0,3963406	0,3524692	0,7627273 monocarboxylate transporter, putative
PFIT_bin08	7,924474644	1,902568526	4,363346682	0,553443969	5,526325227	5,832294271	0	0,948253178	4,3504089	0,4741266	0,1089844	-3,197807	0,3525051	0,7627273 erythrocyte membrane protein 1, PfEMP1, putative
PFIT_11060	20,37722051	46,29583414	18,07672197	18,26365098	17,96055699	32,07761849	42,02759647	38,8783803	25,508601	40,452988	1,5858568	0,6652625	0,3526569	0,7627639 conserved Plasmodium protein, unknown function
PFIT_14245	392,8275288	273,3356783	185,1305664	210,3087083	490,4613639	148,7235039	421,4767531	366,9739799	283,46456	394,22537	1,3907395	0,4758522	0,3528809	0,7629475 conserved Plasmodium protein, unknown function
PFIT_14124	294,3376296	218,7953805	228,1406979	261,7789974	628,6194945	163,3042396	480,3153882	356,5431949	299,16274	418,42929	1,3986678	0,4840533	0,3535948	0,764208 plastid replication-repair enzyme
PFIT_14371	6,792406838	20,92825379	16,20671625	19,37053892	20,7237196	20,41302995	8,405519293	8,534278602	17,405778	8,4698989	0,4866142	-1,03915	0,3537902	0,7642489 conserved Plasmodium protein, unknown function
PFIT_14608	1824,893304	2054,774008	2614,891333	3158,504732	1859,608439	1609,713219	1517,796627	1529,532376	2187,0642	1523,6645	0,6966711	-0,52145	0,353884	0,7642489 conserved Plasmodium protein, unknown function
PFIT_13507	13469,34276	13522,18871	17584,91046	16047,66133	16650,81791	35562,41432	9942,528535	12175,57081	18806,223	11059,05	0,5880527	-0,765983	0,3540871	0,7643378 60S ribosomal protein L17, putative
PFIT_09085	10,18861026	12,68379018	22,44006865	21,03087083	45,59218312	8,748441406	31,2205023	33,18886123	20,113994	32,204681	1,6011082	0,6790708	0,3541954	0,7643378 conserved Plasmodium membrane protein, unknown function
PFIT_04047	1535,083945	626,5792347	475,6047883	514,7028914	776,4486943	898,1733177	1116,733278	978,5972797	804,43215	1047,6653	1,3023663	0,3811352	0,3549865	0,7656878 conserved Plasmodium protein, unknown function
PFIT_05117	112,0747128	82,44463614	64,20352975	69,73394012	118,8159924	61,23908984	114,0749047	126,1176727	84,751984	120,09629	1,4170322	0,5028725	0,3551029	0,7656878 conserved Plasmodium protein, unknown function
PFIT_03199	376,9785795	618,3347711	700,6288101	692,3584055	520,8561526	810,6889036	397,4609837	410,5936261	619,97427	404,0273	0,651684	-0,617756	0,3553811	0,7656878 conserved Plasmodium protein, unknown function
PFIT_06185	124,5274587	148,4003451	160,1971568	138,9144363	113,2896671	151,639651	91,25992375	87,23929238	139,49479	89,249608	0,6398061	-0,644293	0,3554261	0,7656878 conserved Plasmodium protein, unknown function
PFIT_08330	1746,780625	1062,901617	779,1690504	626,4985731	819,2777148	831,1019336	662,8352357	579,3826918	977,62159	621,10896	0,6353266	-0,65443	0,3554979	0,7656878 unspecified product
PFIT_13548	3018,092772	4790,667549	7877,710767	8754,930149	4684,942211	6867,526504	4182,346243	4059,471855	5998,9783	4120,909	0,6869351	-0,541754	0,3557472	0,7659134 protein phosphatase, putative
PFIT_03198	132,4519333	201,0380743	269,2808238	318,2302823	142,3028746	81,65211979	126,0827894	146,0309894	190,82602	136,05689	0,7129892	-0,488048	0,3559953	0,7659134 conserved Plasmodium protein, unknown function
PFIT_14116	5243,738079	7043,942874	7898,28083	7877,721458	5606,456942	7649,053936	4253,192762	4896,779411	6886,5324	4574,9861	0,6643381	-0,59001	0,3565854	0,7659134 eukaryotic translation initiation factor 2 gamma subunit, putative
PFIT_05123	234,3380359	323,4366495	326,0043307	254,5842258	349,5400706	545,3195143	202,9332515	204,8226865	338,87047	203,87797	0,6016398	-0,733028	0,3565988	0,7659134 glutathione synthetase
PFIT_14584	189,0553236	439,4933296	269,904159	192,0450573	653,487958	600,7263099	492,3232729	497,8329185	390,78536	495,0781	1,2668799	0,3412797	0,356612	0,7659134 thioredoxin 1
PFIT_14032	1295,08557	1281,696997	1606,334914	1399,659798	2137,306281	1775,933605	1068,701739	939,7188994	1582,6695	1004,2103	0,6345041	-0,656299	0,3568183	0,7659134 26S proteasome regulatory subunit
PFIT_07298	9640,689438	10816,10207	7818,493939	8214,768835	7459,157475	22603,05645	5150,18175	7019,918277	11092,045	6085,05	0,5485959	-0,866184	0,3570107	0,7659134 Plasmodium exported protein, unknown function
PFIT_11034	11,32067806	54,54029776	70,43688215	44,27551754	22,10530091	20,41302995	26,41734635	22,75807627	37,181951	24,587711	0,6612808	-0,596665	0,3570575	0,7659134 Plasmodium exported protein, unknown function
PFIT_10294	479,9967499	1510,63941	1468,577826	1467,733406	1528,028925	1522,228805	840,5519293	916,01257	1329,5342	878,28225	0,660594	-0,598164	0,3570583	0,7659134 transcription factor, putative
PFIT_12144	15,84894929	17,75730625	19,32339245	9,408547476	12,43423176	23,32917708	7,204730823	7,586025424	16,350267	7,3953781	0,4523093	-1,144618	0,3571627	0,7659134 conserved Plasmodium protein, unknown function
PFIT_06298	1873,572219	1776,364814	2293,874168	2636,607069	1628,884361	2085,045202	1305,257067	1436,603565	2049,0579	1370,9303	0,669054	-0,579806	0,357176	0,7659134 DEAD/DEAH box ATP-dependent RNA helicase
PFIT_14782	6,792406838	6,976084597	8,726693364	7,1947716	12,43423176	8,748441406	13,20867318	16,12030403	8,4787716	14,664489	1,7295534	0,7903996	0,3572276	0,7659134 Plasmodium exported protein (hyp6), unknown function
PFIT_10079	2902,621855	2353,477267	2048,902935	2154,003928	2696,846711	3260,252497	3487,089718	3401,38415	2569,3509	3444,2369	1,3405086	0,4227805	0,3578452	0,7668626 26S proteasome regulatory subunit 4, putative
PFIT_02187	6,792406838	69,12665646	109,7070023	75,82182378	113,2896671	110,8135911	48,03153882	49,30916526	80,925191	48,670352	0,601424	-0,733546	0,3579951	0,7668626 conserved Plasmodium protein, unknown function
PFIT_14339	16,98101709	27,90433839	28,67342105	25,45842258	208,6187773	29,16147135	100,8662315	89,13579873	56,132908	95,001015	1,69243	0,7590961	0,3581345	0,7668626 protein-L-isoaspartate O-methyltransferase beta-aspartate methyltransferase, putative
PFIT_05105	2384,1348	1199,886551	1299,653976	1160,018559	1283,489034	2105,458232	981,0441804	932,132874	1572,1069	956,58853	0,6084755	-0,716729	0,3582127	0,7668626 topoisomerase I
PFIT_14746	1123,011264	746,4410518	988,6096911	808,581639	610,6589375	962,3285547	525,9453501	595,5029958	873,27186	560,72417	0,6420958	-0,63914	0,3586327	0,7670333 conserved Plasmodium protein, unknown function
PFIT_05307	4324,49902	1394,58273	1628,774983	1707,928089	1149,475647	1700,11378	1020,6702	1203,333283	1984,229	1112,0017	0,5604201	-0,83542	0,3586814	0,7670333 conserved Plasmodium protein, unknown function
PFIT_05315	1878,100491	1283,599666	1221,737071	1346,529177	974,0148212	1099,38747	823,7408907	887,5649746	1300,5614	855,65293	0,6579104	-0,604037	0,3586993	0,7670333 methionine aminopeptidase 1a, putative
PFIT_02051	39,62237322	67,85827744	81,65691648	100,1733584	203,0924521	125,3943268	162,1064435	120,4281536	102,96628	141,2673	1,3719763	0,4562555	0,3591686	0,7674989 conserved Plasmodium protein, unknown function
PFIT_12297	1968,665915	846,0088047	826,5425286	929,7858683	773,6855317	1140,21353	684,4494282	666,6219841	1080,817	675,53571	0,6250232	-0,678018	0,3595503	0,7674989 conserved Plasmodium protein, unknown function
PFIT_bin01	241,1304427	216,2586225	266,1641476	261,2255535	51,11850835	78,73597266	311,0042138	294,9067384	185,77221	302,95548	1,6307901	0,7055711	0,3595645	0,7674989 Plasmodium exported protein (hyp10), unknown function, pseudogene
PFIT_14022	373,5823761	333,5836816	339,0943707	290,0046399	230,7240782	979,8254375	177,7166936	213,3569651	424,4691	195,53683	0,4606621	-1,118219	0,3595833	0,7674989 aminopeptidase, putative
PFIT_07053	23076,07016	5771,12453	6054,455189	6191,377683	5191,98255	10142,35974	3997,424818	4294,638643	9404,5616	4146,0317	0,4408533	-1,18163	0,3595955	0,7674989 ribosomal protein, L37e, putative
PFIT_10374	19,24515271	55,17448726	62,95685927	83,01659538	67,69748403	40,8260599	26,41734635	41,72313983	54,81944	34,070243	0,6214993	-0,686175	0,3597437	0,7675255 conserved Plasmodium protein, unknown function
PFIT_14203	176,6025778	576,4782635	522,9782666	387,4107784	415,8559733	274,1178307	238,9569056	298,6997511	392,24062	268,82833	0,6853659	-0,545054	0,3600008	0,7675353 glutathione S-transferase (GST)
PFIT_13243	5051,286552	4926,384104	5714,114148	5153,116797	4183,428197	6363,033049	3089,628734	3660,257267	5231,8938	3374,943	0,645071	-0,63247	0,3600197	0,7675353 phosphoribosylpyrophosphate synthetase
PFIT_05124	243,3945784	301,8742062	340,3410412	308,2682908	283,2241679	399,5121575	193,3269437	204,8226865	312,76907	199,07482	0,6364914	-0,651787	0,3603547	0,767757 PfMAT1 (MAT1)
PFIT_02131	299,9979687	411,5889912	274,2675057	206,4346005	350,9216519	761,1144023	210,1379823	217,1499778	384,05419	213,64398	0,556286	-0,846101	0,3605093	0,767757 conserved protein, unknown function
PFIT_09153	45,28271225	78,63949909	69,19021167	58,11161677	70,46064664	61,23908984	45,62996188	34,13711441	63,820629	39,883538	0,6249318	-0,678229	0,3605309	0,767757 ribonuclease H2 subunit C, putative
PFIT_05027	434,7140376	276,5066258	178,2738787	187,0640616	233,4872408	306,1954492	157,3032896	167,8408125	269,37355	162,57205	0,603519	-0,728529	0,3606974	0,7678225 conserved Plasmodium protein, unknown function
PFIT_10289	75,84854302	14,5863587	24,31007437	22,69120274	27,63162613	5,832294271	10,80709623	11,37903814	28,48335	11,093067	0,389458	-1,360461	0,3609665	0,7679608 conserved Plasmodium protein, unknown function
PFIT_08185	1836,213982	2720,672993	3542,414171	3403,680411	2369,411941	5109,089781	1852,81661	2163,913752	3163,5805	2008,3652	0,6348393	-0,655537	0,3610339	0,7679608 eukaryotic translation initiation factor, putative
PFIT_12286	1297,349706	1136,4676	1456,111121	1449,469755	812,3698083	1793,430488	863,3669103	861,0138856	1324,1997	862,1904	0,651103	-0,619042	0,3616704	0,7689437 conserved Plasmodium protein, unknown function
PFIT_02133	4244,122206	1619,085816	1595,11488	1929,859121	1182,633598	1423,079802	1180,375066	1218,505334	1998,9826	1199,4402	0,6000253	-0,736905	0,3618544	0,7689437 conserved Plasmodium protein, unknown function
PFIT_12081	792,4474644	294,8981216	202,5839531	166,0331908	183,7503138	198,2980052	141,6930395	125,1694195	306,33517	133,43123	0,4355727	-1,199015	0,3619038	0,7689437 conserved Plasmodium protein, unknown function
PFIT_14373	1471,688148	1235,401163	982,9996739	923,1445406	797,1724139	988,5738789	672,4415435	720,6724153	1066,4966	696,55698	0,6531263	-0,614566	0,3622191	0,7693244 conserved Plasmodium membrane protein, unknown function
PFIT_03196	11088,60416	3845,090992	4683,11766	4212,26205	3304,742486	6955,010918	3152,069735	3531,294835	5681,4714	3341,6823	0,588172	-0,76569	0,3627259	0,7698081 elongation factor 1 (EF-1), putative
PFIT_08168	3739,219964	5795,857921	6281,972552	4176,841636	5122,903485	11673,33698	3400,632948	3939,991955	6131,6888	3670,3125	0,598581	-0,740382	0,362854	0,7698081 40S ribosomal protein S16, putative
PFIT_11204	995,0876017	865,03449	1482,914537	1240,267935	739,1459991	1277,272445	694,0557359	767,136821	1099,9538	730,59628	0,6642063	-0,590297	0,3628551	0,7698081 peptidase, putative
PFIT_11383	65,65993276	62,78476137	39,89345538	50,3634012	52,50008965	43,74220703	26,41734635	35,08536759	52,490641	30,75137	0,5858446	-0,77141	0,3631071	0,7699418 conserved Plasmodium protein, unknown function
PFIT_08074	769,8061083	1139,004358	1403,127626	1481,016062	898,0278493	1154,794266	841,7527178	739,6374789	1140,9627	790,6951	0,693007	-0,529058	0,3632178	0,7699418 U2 snRNA/tRNA pseudouridine synthase, putative
PFIT_14165	133,5840011	170,5969779	227,5173627	203,1139367	165,7897568	40,8260599	282,1852906	226,6325095	156,90468	254,4089	1,6214232	0,6972607	0,3633656	0,7699418 conserved Plasmodium membrane protein, unknown function
PFIT_13582	704,1461755	980,4569806	1338,924096	1368,113492	932,567382	1119,8005	728,8786016	732,9997066	1074,0014	730,93915	0,6805756	-0,555173	0,3634863	0,7699418 riboflavin kinase / FAD synthase family protein, putative
PFIT_03164	451,6950547	80,54206762	102,2269794	121,7576732	210,0003586	192,4657109	266,5750404	247,4940795	193,11464	257,03456	1,3309947	0,4125048	0,3637706	0,7699418 conserved Plasmodium protein, unknown function
PFIT_14531	2882,244635	2252,641135	2796,281888	2717,963333	1820,924162	2659,526187	1749,548801	1617,719922	2521,5969	1683,6344	0,6676858	-0,582759	0,3638555	0,7699418 small subunit rRNA processing factor, putative
PFIT_02053	5686,376591	3444,917412	2927,805624	4030,732428	2471,648958	2735,346013	5093,744692	5089,274806	3549,4712	5091,5097	1,4344418	0,5204894	0,3639035	0,7699418 DNA repair endonuclease, putative
PFIT_10119	1357,3493	3038,401937	1375,07754	867,2466997	1244,804757	1708,862221	978,6426034	1126,524775	1598,6237	1052,5837	0,6584312	-0,602895	0,3640071	0,7699418 hypoxanthine-guanine phosphoribosyltransferase
PFIT_04067	1347,160689	995,0433393	1471,694502	1728,95896	965,7253333	985,6577318	881,3787373	846,790088	1249,0401	864,08441	0,6917988	-0,531576	0,3644407	0,7705707 conserved Plasmodium protein, unknown function
PFIT_11483	19,24515271	13,31797968	9,350028604	12,72921129	71,84222795	8,748441406	50,43311576	24,65458263	22,53884	37,543849	1,6657401	0,7361633	0,3646434	0,7706651 mitochondrial ATP synthase delta subunit, putative
PFIT_13465	133,5840011	63,41895088	95,99362701	56,45128486	85,65804101	87,48441406	42,02759647	58,79169704	87,098386	50,409647	0,5787667	-0,788946	0,3647936	0,7706651 conserved Plasmodium membrane protein, unknown function
PFIT_13106	673,5803447	922,7457353	1140,080154	1041,028106	1001,646447	953,5801133	630,413947	646,7086674	955,44348	638,56131	0,6683402	-0,581345	0,3648941	0,7706651 clathrin-adaptor medium chain, putative
PFIT_03178	607,920412	243,5287714	268,0341533	201,4536048	196,1845455	475,3319831	201,732463	185,8576229	332,07558	193,79504	0,5835872	-0,77698	0,3650523	0,7706856 proteasome regulatory protein, putative
PFIT_04128	0	2,536758035	0	0,553443969	9,671069147	0	6,003942352	3,793012712	2,1268785	4,8984775	2,3031299	1,2035958	0,3651764	0,7706856 calmodulin-like protein
PFIT_11344	23691,91505	10641,06577	10862,2399	9493,224404	5363,298632	12297,39247	6561,108203	8266,871206	12058,189	7413,9897	0,614851	-0,701691	0,3657433	0,7715943 conserved Plasmodium protein, unknown function
PFIT_12440	528,6756655	942,40561	423,2446282	596,6125988	534,6719657	481,1642773	858,5637564	859,1173793	584,46246	858,84057	1,4694538	0,55528	0,3659163	0,7716714 microtubule associated katanin, putative
PFIT_12013	98,48989915	27,27014888	18,70005721	26,01186655	35,92111397	8,748441406	61,24021199	52,15392479	35,856921	56,697068	1,5812029	0,6610225	0,3665163	0,7726485 NIMA related kinase 3
PFIT_07269	8719,186244	3980,807547	5347,593026	4944,468421	3929,217236	5835,210418	3129,254754	3788,271446	5459,4138	3458,7631	0,6335411	-0,65849	0,3666549	0,7726528 eukaryotic translation initiation factor 2 alpha subunit, putative
PFIT_04164	879,6166855	232,7475497	174,5338673	153,8574234	370,2637902	749,4498138	551,1619079	508,2637034	426,74485	529,71281	1,2412869	0,3118366	0,3667933	0,7726528 cdc2-related protein kinase 1
PFIT_13088	296,6017652	464,8609099	569,1050744	455,4843866	509,8035022	519,0741901	300,1971176	314,8200551	469,15497	307,50859	0,655452	-0,609438	0,3676237	0,7738097 ribosomal RNA methyltransferase, putative
PFIT_08161	235,4701037	223,8688966	292,9675629	374,6815671	519,4745713	344,105362	505,5319461	416,2831451	331,76134	460,90755	1,3892744	0,4743316	0,3677018	0,7738097 CS domain protein, putative
PFIT_08084	1198,859807	1373,654476	1461,721138	1300,593328	933,9489633	810,6889036	828,5440446	819,2907458	1179,9111	823,9174	0,6982877	-0,518107	0,3677796	0,7738097 conserved Plasmodium protein, unknown function
PFIT_bin02	15,84894929	34,24623347	90,38360984	68,07360821	15,19739437	75,81982552	28,81892329	29,39584852	49,92827	29,107386	0,5829841	-0,778472	0,3678879	0,7738097 erythrocyte membrane protein 1 (PfEMP1)-like, putative
PFIT_08158	328,2996638	449,0061722	375,2478147	290,0046399	339,8690014	533,6549258	224,547444	257,9248644	386,0137	241,23615	0,6249419	-0,678206	0,368039	0,7738396 probable protein, unknown function
PFIT_13109	834,3339732	573,3073159	443,8146911	415,0829769	348,1584893	463,6673945	290,5908099	366,0257267	513,06081	328,30827	0,6399013	-0,644079	0,3684178	0,7740828 cytochrome c2 precursor, putative
PFIT_14708	1,132067806	1,902568526	1,870005721	0	1,381581307	0	3,602365411	3,793012712	1,0477039	3,6976891	3,5293264	1,8193929	0,3684284	0,7740828 mediator of RNA polymerase II transcription subunit 6, putative
PFIT_13452	139,2443402	79,2736886	157,0804806	184,2968417	256,974123	332,4407734	126,0827894	96,72182416	191,55171	111,40231	0,5815782	-0,781955	0,3690806	0,7748958 dynactin subunit 2, putative
PFIT_05009	252,4511208	161,0841352	196,3506007	236,8740188	370,2637902	720,2883424	458,7011957	337,5781314	322,88533	398,13966	1,2330683	0,3022527	0,3690893	0,7748958 erythrocyte membrane protein 2
PFIT_02090	4489,78092	3739,815533	3897,715258	3567,499825	2854,34698	4310,065466	2343,939094	2619,075278	3809,8707	2481,5072	0,6513363	-0,618525	0,3693324	0,7751184 RING zinc finger protein, putative
PFIT_13600	967,9179744	806,6890552	1117,016751	1240,821379	563,6851731	1175,207296	668,839178	648,6051738	978,55627	658,72218	0,6731572	-0,570985	0,3696383	0,7754134 arginine methyltransferase 5, putative
PFIT_11314	1019,993093	1179,592486	1164,390229	935,3203079	1022,370167	1735,107546	707,2644091	767,136821	1176,129	737,20062	0,6268025	-0,673917	0,3698302	0,7754134 conserved Plasmodium protein, unknown function
PFIT_13044	24,90549174	36,148802	27,42675057	20,47742686	35,92111397	5,832294271	43,22838494	39,82663348	25,118647	41,527509	1,6532543	0,7253086	0,3698842	0,7754134 transcription factor with AP2 domain(s)
PFIT_01046	809,4284815	816,2018978	848,9825973	784,2301044	946,3831951	1198,536473	1150,355355	1256,435461	900,62712	1203,3954	1,336175	0,4181089	0,3701835	0,7757533 vacuolar ATP synthase subunit c, putative
PFIT_03186	2052,438933	1697,091125	2034,566224	2180,015795	1041,712305	1361,840712	1150,355355	1246,952929	1727,9442	1198,6541	0,693688	-0,527641	0,3704348	0,7757813 cleavage and polyadenylation specifity factor, putative
PFIT_11119	81,50888205	36,148802	43,63346682	51,47028914	187,8950577	61,23908984	100,8662315	104,3078496	76,982598	102,58704	1,3326004	0,4142442	0,3704712	0,7757813 replication factor C subunit 5, putative
PFIT_09125	1443,386453	920,8431667	1594,491545	1561,265437	1555,660551	2076,29676	953,4260455	971,9595075	1525,324	962,69278	0,6311399	-0,663968	0,3706425	0,7758526 conserved Plasmodium protein, unknown function
PFIT_03134	2599,227683	563,1602838	579,7017735	442,2017314	487,6982012	799,0243151	283,386079	338,5263846	911,83566	310,95623	0,3410222	-1,552062	0,3709765	0,7762646 GTP-binding protein EngA, putative
PFIT_06091	2881,112567	752,1487574	900,096087	931,4462002	563,6851731	1495,98348	563,1697926	649,5534269	1254,0787	606,36161	0,4835116	-1,048378	0,3714212	0,7769076 conserved Plasmodium protein, unknown function
PFIT_11096	3,396203419	11,41541116	52,36016018	85,23037126	31,77637005	20,41302995	16,81103859	15,17205085	34,098591	15,991545	0,4689796	-1,092403	0,3719984	0,7776863 conserved Plasmodium protein, unknown function
PFIT_14189	5,660339031	10,78122165	4,363346682	8,301659538	51,11850835	8,748441406	24,01576941	23,70632945	14,828919	23,861049	1,6090889	0,686244	0,3721395	0,7776863 conserved Plasmodium protein, unknown function
PFIT_10067	19,24515271	7,610274105	3,116676201	5,534439692	8,28948784	5,832294271	10,80709623	16,12030403	8,2713875	13,4637	1,6277439	0,7028737	0,3722059	0,7776863 conserved Plasmodium protein, unknown function
PFIT_02220	2,264135613	28,5385279	74,80022884	63,64605646	96,71069147	545,3195143	10,80709623	7,586025424	135,21319	9,1965608	0,0680153	-3,877998	0,3725036	0,7778484 Pfmc-2TM Maurer's cleft two transmembrane protein
PFIT_06119	5973,921814	4327,075018	5517,140212	5940,667565	3306,124067	4875,79801	3275,750947	3477,244404	4990,1211	3376,4977	0,6766364	-0,563547	0,3725586	0,7778484 nucleolar GTP-binding protein 1, putative
PFIT_01084	58,86752593	78,00530958	65,45020023	107,921574	132,6318054	67,07138411	140,492251	110,9456218	84,9913	125,71894	1,4791977	0,5648149	0,3729332	0,7783433 phosphatidate cytidylyltransferase, putative
PFIT_09371	143,7726114	242,2603924	288,6042163	243,5153464	157,500269	256,6209479	128,4843663	164,996053	222,04563	146,74021	0,6608561	-0,597592	0,3732458	0,7784951 Plasmodium exported protein, unknown function
PFIT_06285	289,8093584	291,0929845	329,7443421	337,0473772	250,0662165	291,6147135	212,5395593	188,7023824	298,22917	200,62097	0,6727074	-0,571949	0,3733252	0,7784951 conserved Plasmodium protein, unknown function
PFIT_01025	2,264135613	7,610274105	5,610017163	2,213775877	5,526325227	5,832294271	8,405519293	10,43078496	4,8428037	9,4181521	1,9447726	0,9596015	0,3734189	0,7784951 sporozoite stage TSP1 domain protein, S21
PFIT_12226	9,05654245	19,02568526	33,66010298	37,08074594	71,84222795	49,5745013	52,8346927	49,30916526	36,706634	51,071929	1,3913542	0,4764897	0,373603	0,7785926 conserved Plasmodium protein, unknown function
PFIT_14363	14,71688148	38,68556004	41,14012586	38,74107784	113,2896671	14,58073568	78,05125058	59,73995021	43,525675	68,8956	1,5828727	0,6625452	0,3738061	0,7787282 ribonuclease H2 subunit B, putative
PFIT_07208	2998,847619	2021,796154	2945,882346	2927,718597	2022,635033	2152,116586	1765,159052	1667,97734	2511,4994	1716,5682	0,6834834	-0,549022	0,3742281	0,7793204 nucleolar rRNA processing protein, putative
PFIT_10305	9,05654245	78,63949909	61,08685355	59,2185047	73,22380925	72,90367838	85,2559814	89,13579873	59,021481	87,19589	1,4773586	0,56302	0,3744498	0,7794951 conserved Plasmodium protein, unknown function
PFIT_05218	5,660339031	12,68379018	5,610017163	13,28265526	2,763162613	0	2,401576941	2,844759534	6,6666607	2,6231682	0,3934756	-1,345654	0,3750844	0,7805288 conserved Plasmodium protein, unknown function, putative
PFIT_08135	5020,720721	7773,260809	9435,425532	10209,93434	7445,341662	9471,645896	5294,276366	5840,291323	8226,0548	5567,2838	0,6767866	-0,563227	0,3754814	0,7806225 RNA helicase, putative
PFIT_06073	2,264135613	18,39149575	18,70005721	16,60331908	62,1711588	14,58073568	42,02759647	28,44759534	22,118484	35,237596	1,5931289	0,671863	0,375537	0,7806225 conserved Plasmodium protein, unknown function
PFIT_14705	134,7160689	244,7971504	183,2605606	157,1780873	147,8291998	376,1829805	96,06307764	149,8240021	207,32734	122,94354	0,5929924	-0,753914	0,375764	0,7806225 nucleoside transporter 3, putative (NT3)
PFIT_14404	461,883665	497,2045749	651,3853261	618,7503576	421,3822985	545,3195143	325,4136755	389,7320562	532,65429	357,57287	0,6713038	-0,574962	0,3758043	0,7806225 conserved Plasmodium protein, unknown function
PFIT_04057	169,8101709	176,9388729	201,9606179	195,3657211	118,8159924	78,73597266	111,6733278	108,1008623	156,93789	109,8871	0,7001948	-0,514172	0,3758195	0,7806225 methyltransferase, putative
PFIT_03203	1612,064556	3380,230082	3586,047637	2653,763832	2992,50511	3595,609418	1809,588225	2117,449347	2970,0368	1963,5188	0,6611093	-0,597039	0,3764408	0,7812782 T-complex protein 1 epsilon subunit, putative
PFIT_14625	861,5036006	969,6757589	590,2984726	411,7623131	989,2122156	2134,619703	438,2877917	513,9532225	992,84534	476,12051	0,4795515	-1,060242	0,3767469	0,7812782 40S ribosomal protein S28e, putative
PFIT_01002	67,92406838	199,7696953	330,3676774	297,1994115	89,80278493	341,1892148	129,6851548	156,4617744	221,04214	143,07346	0,6472678	-0,627565	0,3770353	0,7812782 rifin (RIF)
PFIT_14513	6002,223509	6249,30342	9653,592866	9140,680595	5835,799439	9579,54334	5193,410135	5251,4261	7743,5239	5222,4181	0,674424	-0,568272	0,3771504	0,7812782 signal recognition particle SRP54
PFIT_09143	1540,744284	3012,400167	3497,534033	4664,425772	2352,832965	1565,971012	4143,921012	4495,668317	2772,318	4319,7947	1,5581887	0,63987	0,3771756	0,7812782 phospholipid or glycerol acyltransferase, putative
PFIT_13047	82,64094986	130,6430388	119,0570309	90,21136698	107,7633419	169,1365339	64,8425774	76,80850742	116,57538	70,825542	0,6075515	-0,718921	0,3771816	0,7812782 protein phosphatase, putative
PFIT_04060	11,32067806	31,07528593	47,9968135	40,95485372	31,77637005	67,07138411	24,01576941	19,91331674	38,365898	21,964543	0,5725017	-0,804648	0,3772155	0,7812782 conserved Plasmodium protein, unknown function
PFIT_14099	122,2633231	361,48802	344,0810526	281,7029803	163,0265942	137,0589154	164,5080205	176,3750911	234,93681	170,44156	0,7254783	-0,462996	0,3772402	0,7812782 tRNA-dihydrouridine synthase, putative
PFIT_11202	3892,049118	3316,811131	3713,831362	3667,673188	3183,163331	3674,345391	2470,021884	2314,686008	3574,6456	2392,3539	0,6692563	-0,579369	0,3776141	0,7817238 ubiquitin-protein ligase, putative
PFIT_02038	443,7705801	1048,315258	850,2292678	740,5080308	787,5013448	1090,639029	489,9216959	587,9169704	826,82725	538,91933	0,6517919	-0,617517	0,3777628	0,7817238 aspartate aminotransferase
PFIT_14767	1384,518927	2321,133602	3011,332546	3339,48091	1677,239706	2190,026499	1707,521205	1612,030403	2320,622	1659,7758	0,7152288	-0,483523	0,37787	0,7817238 bromodomain protein, putative
PFIT_12178	53,2071869	32,97785446	30,54342677	81,35626347	26,25004483	8,748441406	82,85440446	52,15392479	38,847203	67,504165	1,737684	0,7971658	0,3781664	0,7820508 conserved Plasmodium protein, unknown function
PFIT_03140	316,9789858	1050,217827	549,1583467	811,3488588	567,829917	312,0277435	948,6228917	927,3916081	601,26028	938,00725	1,5600685	0,6416094	0,3786205	0,7827038 vesicle transport v-SNARE protein, putative
PFIT_02198	339,6203419	298,7032586	226,2706922	334,8336014	259,7372857	134,1427682	201,732463	167,8408125	265,55132	184,78664	0,6958604	-0,52313	0,3789504	0,7830069 DnaJ protein, putative, pseudogene
PFIT_10201	1469,424013	677,9485849	538,5616476	485,923805	578,8825675	609,4747513	427,4806955	473,1783358	726,70256	450,32952	0,6196889	-0,690384	0,3791313	0,7830069 conserved Plasmodium membrane protein, unknown function
PFIT_13031	7,924474644	6,976084597	4,986681922	5,534439692	5,526325227	2,916147135	7,204730823	14,22379767	5,6440255	10,714264	1,8983373	0,9247364	0,3791825	0,7830069 conserved Plasmodium protein, unknown function
PFIT_bin08	198,1118661	13,95216919	16,83005149	13,28265526	13,81581307	17,49688281	1,20078847	3,793012712	45,581573	2,4969006	0,0547787	-4,19024	0,3796156	0,7831187 erythrocyte membrane protein 1 (PfEMP1), exon1, pseudogene
PFIT_14493	135,8481368	128,1062808	105,3436556	114,5629016	114,6712485	87,48441406	74,44888517	73,96374789	114,33611	74,206317	0,6490191	-0,623667	0,379758	0,7831187 conserved Plasmodium protein, unknown function
PFIT_13442	112,0747128	213,7218645	225,647357	179,315846	110,5265045	102,0651497	109,2717508	112,8421282	157,22524	111,05694	0,7063557	-0,501533	0,3798996	0,7831187 conserved Plasmodium protein, unknown function
PFIT_08189	1530,555674	2461,923673	2944,635675	3040,067723	1681,38445	1808,011224	1460,15878	1730,56205	2244,4297	1595,3604	0,7108088	-0,492467	0,3799496	0,7831187 nucleolar preribosomal assembly protein, putative
PFIT_12344	118,8671197	102,1045109	216,2973284	226,9120274	63,55274011	373,2668333	84,05519293	114,7386345	183,50009	99,396914	0,5416723	-0,884508	0,3799917	0,7831187 CPW-WPC family protein
PFIT_02003	0	0	0	0,553443969	0	0	0	2,844759534	0,0922407	1,4223798	15,420312	3,94676	0,3800834	0,7831187 rifin (RIF)
PFIT_07102	141,5084758	162,9867038	120,3037014	132,2731086	111,9080858	64,15523698	187,3230014	183,0128634	122,18922	185,16793	1,5154196	0,5997173	0,3802058	0,7831187 conserved Plasmodium protein, unknown function
PFIT_01040	14,71688148	13,95216919	20,57006293	18,81709495	48,35534573	40,8260599	36,02365411	34,13711441	26,206269	35,080384	1,3386257	0,4207526	0,3804099	0,7832539 conserved Plasmodium protein, unknown function
PFIT_12086	1168,293976	461,0557729	579,7017735	558,9784089	363,3558836	656,1331055	413,0712338	399,2145879	631,25315	406,14291	0,6433915	-0,636231	0,380764	0,7835432 conserved Plasmodium protein, unknown function
PFIT_06024	52,07511909	10,14703214	126,5370538	120,0973413	185,1318951	87,48441406	174,1143282	157,4100276	113,61247	165,76218	1,459014	0,5449937	0,3808823	0,7835432 cation/H antiporter
PFIT_14717	558,1094285	386,8556004	536,0683067	442,2017314	402,0401602	982,7415846	318,2089447	347,0606632	551,33614	332,6348	0,6033249	-0,728993	0,380966	0,7835432 RAP protein, putative
PFIT_13371	1953,949034	493,3994378	509,8882266	736,633923	410,3296481	212,8787409	296,5947522	310,0787892	719,51317	303,33677	0,4215861	-1,246101	0,3816267	0,7842218 conserved Plasmodium protein, unknown function
PFIT_03060	783,3909219	1113,636777	1280,330584	1462,752411	1015,46226	848,5988164	760,0991018	770,9298337	1084,0286	765,51447	0,7061755	-0,501901	0,3817069	0,7842218 conserved Plasmodium protein, unknown function
PFIT_06291	62,26372935	38,05137053	49,86681922	40,95485372	17,96055699	11,66458854	15,61025012	29,39584852	36,793653	22,503049	0,6116014	-0,709336	0,3817315	0,7842218 secreted ookinete protein, putative
PFIT_02049	298,8659009	294,8981216	426,9846396	432,2397399	349,5400706	312,0277435	253,3663673	229,4772691	352,42604	241,42182	0,6850283	-0,545764	0,3818506	0,7842218 conserved Plasmodium protein, unknown function
PFIT_13662	35,09410199	71,66341449	86,6435984	87,9975911	17,96055699	8,748441406	80,45282752	98,61833051	51,351284	89,535579	1,7435899	0,8020607	0,3822331	0,7847225 conserved Plasmodium protein, unknown function
PFIT_07150	191,3194593	254,9441825	256,1907838	228,0189153	215,5266838	198,2980052	162,1064435	142,2379767	224,04967	152,17221	0,6791896	-0,558114	0,3826023	0,7851954 drug metabolite transporter, putative
PFIT_06076	2445,266462	4178,674673	4296,026476	3582,442813	3354,479413	4336,31079	2349,943037	2618,127024	3698,8668	2484,035	0,6715665	-0,574398	0,3830868	0,7858081 chaperone, putative
PFIT_14501	0	2,536758035	3,740011442	2,767219846	12,43423176	5,832294271	8,405519293	7,586025424	4,5517526	7,9957724	1,756636	0,8128153	0,3833021	0,7858081 conserved Plasmodium protein, unknown function
PFIT_07002	10,18861026	4,439326561	9,973363845	18,26365098	2,763162613	37,90991276	3,602365411	4,74126589	13,923005	4,1718157	0,2996347	-1,738723	0,3835209	0,7858081 rifin
PFIT_05012	2750,924769	4016,956349	4066,639108	5008,667921	3196,979144	2449,563594	2521,655788	2619,075278	3581,6218	2570,3655	0,7176541	-0,478639	0,3835415	0,7858081 heat shock protein 40, type II
PFIT_08248	3,396203419	12,04960067	9,973363845	7,748215569	24,86846352	8,748441406	21,61419247	15,17205085	11,130715	18,393122	1,6524655	0,7246201	0,3838353	0,7858081 conserved Plasmodium protein, unknown function
PFIT_13081	9696,160761	5461,64005	6002,095029	4816,622864	3904,348773	8416,000633	3729,648989	4319,293226	6382,814	4024,4711	0,630517	-0,665393	0,3840077	0,7858081 mRNA-decapping enzyme 2, putative
PFIT_10205	132,4519333	168,6944093	137,7570881	175,9951822	266,6451922	139,9750625	253,3663673	225,6842564	170,25314	239,52531	1,4068775	0,4924967	0,384101	0,7858081 conserved Plasmodium protein, unknown function
PFIT_14612	7,924474644	18,39149575	28,05008581	31,54630624	16,57897568	11,66458854	12,0078847	10,43078496	19,025988	11,219335	0,5896847	-0,761984	0,3842741	0,7858081 biotin protein ligase, putative
PFIT_06252	706,4103111	731,2205036	692,525452	448,843059	507,0403395	766,9466966	354,2325988	468,4370699	642,16439	411,33483	0,6405444	-0,642629	0,3842792	0,7858081 3-oxoacyl-acyl-carrier protein synthase I/II
PFIT_14640	8206,359528	25417,68132	18827,84093	11072,75349	13813,0499	20765,88375	8878,629951	12738,83319	16350,595	10808,732	0,6610605	-0,597146	0,3842901	0,7858081 glyceraldehyde-3-phosphate dehydrogenase
PFIT_08131	1507,914318	1695,188557	1297,78397	1260,745362	518,09299	279,950125	775,7093519	826,8767712	1093,2792	801,29306	0,7329263	-0,44826	0,3851549	0,7867393 ABC1 family, putative
PFIT_13404	5333,171435	9384,736351	12399,3846	10501,04587	9726,332399	19057,02153	6535,891645	7513,009929	11066,949	7024,4508	0,6347234	-0,6558	0,385208	0,7867393 60S ribosomal protein L18, putative
PFIT_11109	2495,077445	2227,273555	1714,171911	1411,835565	2741,057312	2729,513719	2928,723079	2803,984647	2219,8216	2866,3539	1,2912542	0,368773	0,3852945	0,7867393 conserved Plasmodium protein, unknown function
PFIT_03228	5559,584997	4635,29112	5474,753415	5407,147579	3375,203132	4289,652436	3275,750947	3341,644199	4790,2721	3308,6976	0,6907118	-0,533844	0,3853887	0,7867393 conserved Plasmodium protein, unknown function
PFIT_02056	8470,131327	2594,46928	3283,730046	3642,214761	1819,542581	3050,289904	2138,604266	2221,757196	3810,063	2180,1807	0,5722165	-0,805367	0,3854411	0,7867393 conserved Plasmodium protein, unknown function, pseudogene
PFIT_14057	302,2621043	932,2585779	693,1487872	1100,800055	663,1590272	279,950125	1020,6702	1061,095306	661,92978	1040,8828	1,5724972	0,6530575	0,3858636	0,7870549 conserved Plasmodium protein, unknown function
PFIT_14270	2765,641651	5001,852656	6647,247002	5421,537122	4946,061078	7395,349135	3276,951736	3811,977776	5362,9481	3544,4648	0,6609172	-0,597459	0,385874	0,7870549 60S ribosomal protein L21e, putative
PFIT_13622	58,86752593	348,8042298	426,9846396	369,1471275	299,8031435	297,4470078	182,5198475	233,2702818	300,17561	207,89506	0,6925781	-0,529951	0,3863933	0,78783 bifunctional polynucleotide phosphatase/kinase
PFIT_09047	255,8473242	175,6704939	175,1572025	149,4298717	168,5529194	326,6084792	138,0906741	115,6868877	208,54438	126,88878	0,6084498	-0,71679	0,3871364	0,788805 prefoldin subunit, putative
PFIT_09219	545,6566826	971,5783275	1161,896888	1176,068435	864,869898	1055,645263	628,0123701	694,1213263	962,61925	661,06685	0,6867376	-0,542169	0,3871504	0,788805 nucleolar preribosomal assembly protein, putative
PFIT_08347	670,1841413	214,356054	370,2611327	414,5295329	40,06585789	285,7824193	192,1261553	201,9779269	332,52986	197,05204	0,5925845	-0,754907	0,3875209	0,7892756 Plasmodium exported protein (PHISTa), unknown function, pseudogene
PFIT_02020	37,35823761	31,70947544	26,18008009	29,88597434	55,26325227	37,90991276	56,43705811	46,46440572	36,384489	51,450732	1,4140842	0,4998681	0,3877466	0,789451 octaprenyl pyrophosphate synthase
PFIT_04044	387,1671897	497,2045749	385,8445137	432,2397399	560,9220105	860,2634049	679,6462743	654,2946928	520,60691	666,97048	1,2811403	0,3574285	0,3881034	0,7898931 lysine decarboxylase, putative
PFIT_14620	70,18820399	121,7643857	61,71018879	63,64605646	160,2634316	61,23908984	136,8898856	116,6351409	89,801893	126,76251	1,4115795	0,4973104	0,3885339	0,7900852 snRNA-activating protein complex subunit 3, putative
PFIT_12385	8231,265019	7715,549564	6943,954575	7776,994655	6349,747685	3642,267772	4715,496323	4856,952778	6776,6299	4786,2246	0,7062839	-0,50168	0,3885488	0,7900852 acyl-CoA synthetase (ACS11)
PFIT_13273	358,8654946	235,2843078	230,6340389	270,080657	476,6455508	119,5620326	431,0830609	359,3879545	281,84535	395,23551	1,4023134	0,4878088	0,3889098	0,7900852 conserved Plasmodium protein, unknown function
PFIT_12032	1821,4971	1555,666865	1373,207534	1282,329677	1275,199546	1073,142146	2168,623978	1808,31881	1396,8405	1988,4714	1,4235494	0,5094925	0,3889187	0,7900852 Sua5/YciO/YrdC family protein, putative
PFIT_12358	3326,015215	4083,546247	2681,588204	1990,184513	3394,54527	6240,55487	2102,58024	2276,75588	3619,4057	2189,6682	0,60498	-0,725041	0,3889768	0,7900852 clustered-asparagine-rich protein
PFIT_12322	1360,745503	605,6509809	704,9921568	941,4081916	657,632702	790,2758737	547,5595425	563,2623877	843,4509	555,41097	0,6584983	-0,602748	0,3890359	0,7900852 conserved protein, unknown function
PFIT_12070	29,43376296	43,75907611	33,03676774	28,22564243	6,907906533	17,49688281	49,23232729	40,77488665	26,476673	45,003607	1,6997455	0,7653188	0,3892502	0,790158 conserved Plasmodium protein, unknown function
PFIT_08246	1413,95269	2766,334637	2373,03726	2041,101358	2131,779956	2496,221948	1392,914626	1575,996782	2203,738	1484,4557	0,6736081	-0,570019	0,3893512	0,790158 60S ribosomal protein L22, putative
PFIT_13650	32,82996638	152,2054821	189,493913	150,5367596	82,8948784	303,2793021	87,65755834	86,2910392	151,87338	86,974299	0,5726764	-0,804208	0,3895584	0,7902378 conserved Plasmodium protein, unknown function
PFIT_08352	15,84894929	39,95393905	79,16357552	54,23750898	63,55274011	341,1892148	16,81103859	16,12030403	98,990988	16,465671	0,1663351	-2,587836	0,38967	0,7902378 Plasmodium exported protein (PHISTa-like), unknown function
PFIT_11121	246,7907818	221,9663281	261,1774657	213,6293721	325,7242575	559,90025	182,5198475	178,2715975	306,53141	180,39572	0,5885065	-0,76487	0,3898715	0,7903632 SNARE protein, putative
PFIT_02157	1693,573438	541,5978405	435,711333	280,0426484	327,4347697	396,5960104	241,3584826	246,5458263	612,49267	243,95215	0,398294	-1,328094	0,3902978	0,7908298 conserved Plasmodium protein, unknown function
PFIT_12190	185,6591202	95,76261583	94,74695652	69,73394012	105,0001793	338,2730677	166,9095974	185,8576229	148,19598	176,38361	1,1902051	0,2512102	0,3903813	0,7908298 erythrocyte membrane protein 1, PfEMP1
PFIT_11395	3931,671491	3384,669408	4363,346682	4661,105109	3046,386781	4400,466027	2544,470769	2822,949711	3964,6076	2683,7102	0,676917	-0,562949	0,3909662	0,7915455 conserved Plasmodium protein, unknown function
PFIT_06035	221,88529	209,2825379	178,897214	218,0569239	284,6057492	61,23908984	296,5947522	277,8381812	195,66113	287,21647	1,467928	0,5537813	0,3910631	0,7915455 DNA helicase, putative
PFIT_08023	1333,575876	714,7315764	956,1962586	1095,819059	1054,146537	4307,149319	431,0830609	616,3645657	1576,9364	523,72381	0,3321147	-1,590246	0,3911905	0,7915455 Plasmodium exported protein (hyp17), unknown function
PFIT_14047	9,05654245	30,44109642	30,54342677	24,3515344	52,50008965	11,66458854	38,42523105	43,61964619	26,426213	41,022439	1,5523389	0,6344436	0,3913225	0,7915455 translocation associated membrane protein, putative
PFIT_12198	544,5246148	717,9025239	701,8754806	783,6766604	505,6587582	834,0180807	416,6735992	496,8846653	681,27602	456,77913	0,6704759	-0,576743	0,3915702	0,7915455 pre-mRNA splicing factor, putative
PFIT_07082	5410,152046	3366,508217	3393,437048	3261,44531	1749,081934	3449,802061	2337,935152	2252,101298	3405,0701	2295,0182	0,6740001	-0,569179	0,3915874	0,7915455 Cg2 protein
PFIT_13110	15440,27281	21037,96857	23383,17487	22645,26689	18357,07082	30777,01687	14068,43772	14987,14148	21940,128	14527,79	0,6621561	-0,594757	0,3918729	0,7915455 M1-family alanyl aminopeptidase
PFIT_04203	447,1667835	643,7023514	637,6719508	471,5342618	526,3824778	694,0430182	378,2483682	377,4047648	570,08347	377,82657	0,6627566	-0,593449	0,3920834	0,7915455 transmembrane emp24 domain-containing protein, putative
PFIT_01112	741,5044131	192,1594212	261,8008009	426,7053003	158,8818503	61,23908984	146,4961934	160,2547871	307,04848	153,37549	0,4995156	-1,001399	0,3921317	0,7915455 Plasmodium exported protein (hyp11), unknown function
PFIT_04003	1589,4232	1509,371031	1790,21881	1540,78801	1121,844021	1493,067333	939,0165839	1110,404471	1507,4521	1024,7105	0,6797633	-0,556896	0,3922337	0,7915455 rifin
PFIT_12166	10154,64822	9821,692922	14092,36311	14742,64045	8487,053967	16864,07888	7908,392866	8664,189288	12360,413	8286,2911	0,6703895	-0,576929	0,3923133	0,7915455 DNA-binding chaperone, putative
PFIT_09178	400,7520034	462,9583414	442,5680206	449,949947	367,5006276	294,5308607	273,7797713	286,3724598	403,0433	280,07612	0,6949033	-0,525116	0,3924839	0,7915455 XPA binding protein 1, putative
PFIT_08130	2,264135613	21,5624433	14,96004577	14,3895432	19,34213829	8,748441406	3,602365411	9,48253178	13,544458	6,5424486	0,4830351	-1,0498	0,3926403	0,7915455 apicoplast ribosomal protein L33 precursor, putative
PFIT_14662	88,30128889	139,5216919	114,070349	113,4560137	302,5663062	96,23285547	202,9332515	184,9093697	142,35808	193,92131	1,3622079	0,4459469	0,3927719	0,7915455 conserved Plasmodium protein, unknown function
PFIT_14581	170,9422387	494,6678168	586,5584611	540,1613139	443,4875994	769,8628437	290,5908099	364,1292204	500,94671	327,36002	0,6534827	-0,613779	0,3929696	0,7915455 conserved protein, unknown function
PFIT_07068	416,6009527	901,8174815	975,5196511	955,7977348	614,8036815	626,9716341	519,9414077	554,7281091	748,58519	537,33476	0,7178004	-0,478345	0,3929738	0,7915455 large ribosomal subunit processing protein, putative
PFIT_06026	4,528271225	8,878653123	18,07672197	14,94298717	33,15795136	23,32917708	32,4212887	18,96506356	17,152294	25,693176	1,497944	0,5829837	0,3931856	0,7916903 phenylalanyl-tRNA synthetase subunit, putative
PFIT_03027	363,3937658	950,0158842	1495,381241	1666,973235	600,9878684	778,6112852	716,8707169	727,3101875	975,89388	722,09045	0,7399272	-0,434545	0,3934739	0,7919886 conserved Plasmodium protein, unknown function
PFIT_13557	519,6191231	774,9795797	647,6453147	492,5651326	617,5668441	799,0243151	826,1424677	904,6335318	641,90005	865,388	1,3481663	0,4309984	0,3941862	0,7926795 regulator of chromosome condensation, putative
PFIT_05146	2,264135613	11,41541116	24,93340961	21,5843148	15,19739437	64,15523698	4,803153882	9,48253178	23,258317	7,1428428	0,3071092	-1,703176	0,3941967	0,7926795 conserved Plasmodium membrane protein, unknown function
PFIT_13634	10137,66721	4121,597618	4228,70627	4259,304787	2836,386423	4202,168022	3088,427946	3252,508401	4964,3051	3170,4682	0,638653	-0,646896	0,3943491	0,7926795 conserved Plasmodium protein, unknown function
PFIT_13183	13282,55157	4484,354017	6861,674325	9117,435949	3908,493517	3566,447947	4608,62615	4545,925735	6870,1596	4577,2759	0,6662547	-0,585854	0,3944407	0,7926795 conserved protein, unknown function
PFIT_13523	75270,05637	13988,95218	14138,48992	10663,7584	14010,61603	19532,35351	6591,127914	8293,422295	24600,704	7442,2751	0,3025228	-1,724884	0,3945178	0,7926795 Plasmodium exported protein, unknown function
PFIT_12024	4035,821729	1166,274507	1846,318982	2270,780606	1095,593976	1816,759665	1329,272837	1299,106854	2038,5916	1314,1898	0,6446558	-0,633399	0,3947048	0,7927736 conserved Plasmodium protein, unknown function
PFIT_14666	253,5831886	178,8414415	187,6239073	189,8312814	96,71069147	134,1427682	109,2717508	125,1694195	173,45555	117,22059	0,6757961	-0,56534	0,3949491	0,7929826 potassium channel protein
PFIT_08045	3662,239353	899,914913	1231,710435	1062,612421	987,8306343	1729,275251	829,7448331	819,2907458	1595,5972	824,51779	0,5167456	-0,952474	0,3955025	0,7932996 sentrin-specific protease 2, putative
PFIT_13629	1557,725301	774,3453902	915,679468	682,396414	154,7371063	180,8011224	469,5082919	349,9054227	710,94747	409,70686	0,5762829	-0,795151	0,3955728	0,7932996 unspecified product
PFIT_12472	10,18861026	27,27014888	18,07672197	23,24464671	63,55274011	17,49688281	42,02759647	36,03362076	26,638292	39,030609	1,4652069	0,5511044	0,3955943	0,7932996 DNA replication origin binding protein, putative
PFIT_06154	881,8808211	990,6040127	1051,56655	1053,203873	748,8170682	682,3784297	637,6186778	638,1743888	901,40846	637,89653	0,7076665	-0,498859	0,395668	0,7932996 conserved Plasmodium protein, unknown function
PFIT_08071	610,1845476	531,7909336	508,6415561	615,9831377	237,6319847	323,692332	279,7837136	377,4047648	467,98742	328,59424	0,7021433	-0,510163	0,3962278	0,7935885 conserved Plasmodium protein, unknown function
PFIT_11264	549,052886	715,9999554	1012,919765	877,7621351	453,1586686	396,5960104	510,3350999	479,8161081	667,58157	495,0756	0,7415957	-0,431295	0,396256	0,7935885 small nuclear ribonucleoprotein D1, putative
PFIT_11065	2495,077445	3642,784538	4385,786751	3923,364298	3340,663599	4849,552686	2324,726479	2726,227887	3772,8716	2525,4772	0,669378	-0,579107	0,3963145	0,7935885 conserved protein, unknown function
PFIT_10050	10,18861026	3,805137053	4,363346682	8,301659538	5,526325227	8,748441406	1,20078847	2,844759534	6,8222534	2,022774	0,2964965	-1,753913	0,3963733	0,7935885 conserved Plasmodium protein, unknown function
PFIT_08229	13198,77855	6497,905707	6488,919851	6372,353861	4632,442121	8252,696393	4901,618536	4990,656476	7573,8494	4946,1375	0,6530654	-0,614724	0,3975773	0,7952924 Snf2-related CBP activator, putative
PFIT_14069	3379,222402	1901,300147	2874,822128	3824,297827	1344,278611	2364,995327	1798,781129	1887,972077	2614,8194	1843,3766	0,7049728	-0,50436	0,397699	0,7952924 RNA binding protein, putative
PFIT_14414	29,43376296	35,51461249	57,97017735	45,38240547	89,80278493	122,4781797	37,22444258	31,29235487	63,43032	34,258399	0,540095	-0,888715	0,3977572	0,7952924 delta-aminolevulinic acid dehydratase
PFIT_07154	408,6764781	873,2789536	867,0593192	1038,81433	740,5275804	924,4186419	1175,571913	1136,007307	808,79588	1155,7893	1,4290251	0,5150312	0,3979664	0,7952924 transcription initiation factor IIE, alpha subunit, putative
PFIT_09144	1006,40828	1548,056591	2375,530601	2227,611976	1228,225782	1525,144952	1141,949835	1233,677385	1651,8297	1187,8136	0,7190896	-0,475756	0,3980628	0,7952924 met-10 like protein, putative
PFIT_13602	8814,27994	2545,002499	3567,970915	3958,784712	2343,161896	4082,60599	2502,443172	2611,489252	4218,6343	2556,9662	0,6061123	-0,722343	0,398068	0,7952924 conserved Plasmodium protein, unknown function
PFIT_08128	2661,491413	3210,901483	3268,146665	3607,901235	2377,701429	1732,191398	1941,674957	2116,501093	2809,7223	2029,088	0,7221668	-0,469596	0,3986091	0,7960921 JmjC domain containing protein
PFIT_07004	49,81098348	13,95216919	18,07672197	18,26365098	6,907906533	11,66458854	9,606307764	9,48253178	19,779337	9,5444198	0,482545	-1,051265	0,3988999	0,7963918 rifin (RIF)
PFIT_09276	2,264135613	1,268379018	1,246670481	0,553443969	5,526325227	0	6,003942352	2,844759534	1,8098257	4,4243509	2,4446282	1,2896151	0,3997808	0,7975315 dynein light chain, putative
PFIT_11163	671,3162091	379,2453263	756,7289817	937,5340838	378,553278	702,7914596	457,5004072	431,455196	637,69489	444,4778	0,697007	-0,520755	0,3997905	0,7975315 transcription factor with AP2 domain(s), putative
PFIT_10123	82,64094986	32,34366495	39,89345538	45,38240547	48,35534573	29,16147135	61,24021199	70,17073517	46,296215	65,705474	1,4192407	0,5051193	0,4001189	0,7975315 conserved Plasmodium protein, unknown function
PFIT_07284	1229,425638	2125,803233	2519,521041	2526,471719	1965,990199	2712,016836	1412,127241	1562,721237	2179,8714	1487,4242	0,6823449	-0,551427	0,4002161	0,7975315 mRNA (N6-adenosine)-methyltransferase, putative
PFIT_09132	48,67891567	62,15057186	129,65373	133,3799966	120,1975737	244,9563594	63,64178893	75,86025424	123,16952	69,751022	0,566301	-0,820359	0,4003091	0,7975315 conserved Plasmodium protein, unknown function
PFIT_01060	82377,17806	28040,05494	32107,37489	34785,06035	20057,79741	32532,53744	22340,66949	23911,15214	38316,667	23125,911	0,603547	-0,728462	0,4003168	0,7975315 lipid/sterol:H symporter
PFIT_14401	2814,320566	4700,612639	3979,995509	2945,428804	3307,505648	6126,825131	2279,096517	2840,018268	3979,1147	2559,5574	0,643248	-0,636553	0,4011124	0,7988351 thioredoxin peroxidase 1
PFIT_01082	4630,157328	793,3710755	991,1030321	1034,940222	570,5930796	971,0769961	497,1264268	469,3853231	1498,5403	483,25587	0,3224844	-1,632699	0,4015168	0,7990206 conserved protein, unknown function
PFIT_07029	2609,416293	2311,216875	2244,006865	1923,771237	1633,029104	1711,778368	1293,249183	1495,395262	2038,8698	1394,3222	0,6838702	-0,548206	0,4018456	0,7990206 conserved Plasmodium membrane protein, unknown function
PFIT_09268	1745,648557	722,97604	541,6783238	373,0212352	587,1720553	857,3472578	419,0751762	458,9545382	804,64058	439,01486	0,5456037	-0,874075	0,4021426	0,7990206 NAD synthase, putative
PFIT_10375	52,07511909	55,17448726	39,27012014	44,27551754	16,57897568	14,58073568	22,81498094	25,60283581	36,992493	24,208908	0,6544276	-0,611694	0,4021772	0,7990206 conserved Plasmodium protein, unknown function
PFIT_14609	13,58481368	38,68556004	29,29675629	29,88597434	63,55274011	29,16147135	50,43311576	48,36091208	34,027886	49,397014	1,4516627	0,5377063	0,4021882	0,7990206 pyridoxal 5'-phosphate synthase, putative
PFIT_08196	48,67891567	43,1248866	29,92009153	20,47742686	45,59218312	37,90991276	20,413404	21,80982309	37,617236	21,111614	0,5612218	-0,833357	0,4022185	0,7990206 conserved Plasmodium protein, unknown function
PFIT_04175	1518,102928	292,9955531	297,3309096	272,2944328	360,592721	172,052681	127,2835779	168,7890657	485,56154	148,03632	0,3048765	-1,713703	0,4023342	0,7990206 conserved Plasmodium protein, unknown function
PFIT_12112	2722,623074	1780,804141	1980,336058	1860,678624	1592,963247	2134,619703	1343,682298	1342,7265	2012,0041	1343,2044	0,6675952	-0,582954	0,4024304	0,7990206 lysine-specific histone demethylase 1, putative
PFIT_11145	571,6942422	856,1558369	877,6560183	806,3678631	642,4353076	1216,033355	530,7485039	565,1588941	828,39044	547,9537	0,6614679	-0,596257	0,4024769	0,7990206 glyoxalase I
PFIT_14358	3020,356907	3318,713699	3993,708885	4194,551843	2294,80655	2962,80549	2268,289421	2428,476389	3297,4906	2348,3829	0,7121727	-0,489701	0,402825	0,7994222 methionine aminopeptidase 2
PFIT_09215	11,32067806	7,610274105	9,973363845	3,320663815	24,86846352	0	16,81103859	14,22379767	9,5155739	15,517418	1,6307391	0,705526	0,4029619	0,7994222 Fe-S-cluster redox enzyme, putative
PFIT_13026	5318,454554	3380,230082	3217,65651	3485,590118	2497,899002	2881,15337	2273,092575	2453,130972	3463,4973	2363,1118	0,6822906	-0,551542	0,4037946	0,8006548 conserved Plasmodium protein, unknown function
PF_apicopla	0	0	0	0	0	0	0	1,896506356	0	0,9482532	#DIV/0!	#DIV/0!	0,4039674	0,8006548 unspecified product
PFIT_05120	132,4519333	77,37112007	142,74377	122,3111172	67,69748403	134,1427682	76,85046211	71,11898835	112,78637	73,984725	0,6559722	-0,608293	0,4040079	0,8006548 conserved Plasmodium protein, unknown function
PFIT_14023	94724,64162	67260,87092	75207,26675	78413,04844	59326,48289	88904,57772	48998,17354	54186,9796	77306,148	51592,577	0,66738	-0,58342	0,4041814	0,800718 early transcribed membrane protein 14.1
PFIT_14545	6,792406838	22,19663281	21,81673341	33,20663815	40,06585789	11,66458854	14,40946165	12,32729131	22,62381	13,368376	0,5908986	-0,759018	0,4044311	0,8009322 conserved protein, unknown function
PFIT_02113	65,65993276	53,90610825	73,55355835	62,53916852	192,0398016	81,65211979	115,2756932	111,893875	88,225115	113,58478	1,2874427	0,3645083	0,4046794	0,8011326 conserved Plasmodium protein, unknown function
PFIT_11325	24,90549174	6,341895088	3,116676201	3,874107784	5,526325227	0	15,61025012	10,43078496	7,2940827	13,020518	1,7850795	0,8359883	0,4048156	0,8011326 conserved Plasmodium protein, unknown function
PFIT_14138	2796,207482	3251,489612	3553,01087	2947,64258	2782,504752	3029,876874	1951,281264	2242,618766	3060,122	2096,95	0,6852505	-0,545297	0,4051946	0,8012287 N-myristoyltransferase (NMT)
PFIT_14519	8810,883736	2128,974181	2454,694176	2756,704411	1627,502779	2504,970389	1546,61555	1595,910099	3380,6216	1571,2628	0,4647852	-1,105364	0,4051948	0,8012287 conserved Plasmodium protein, unknown function
PFIT_02125	1709,422387	1559,472002	1648,098375	1603,880623	1431,318234	3009,463844	1049,489123	1252,642448	1826,9426	1151,0658	0,6300503	-0,666461	0,4052891	0,8012287 heat shock protein 40, putative
PFIT_09252	52,07511909	36,148802	51,73682494	52,57717707	40,06585789	34,99376562	24,01576941	31,29235487	44,599591	27,654062	0,6200519	-0,689539	0,4056453	0,8014503 conserved Plasmodium protein, unknown function
PFIT_12142	140,376408	169,3285988	200,0906121	164,3728589	77,36855317	172,052681	91,25992375	116,6351409	153,93162	103,94753	0,6752838	-0,566434	0,4057942	0,8014503 adrenodoxin-type ferredoxin, putative
PFIT_06132	2261,871477	826,3489299	723,0688787	803,6006433	612,0405188	481,1642773	485,1185421	553,779856	951,34912	519,4492	0,5460132	-0,872992	0,4058263	0,8014503 conserved Plasmodium protein, unknown function
PFIT_04186	13737,64283	6249,30342	7704,42357	9635,459504	4531,586686	7115,39901	5408,351271	5798,568184	8162,3025	5603,4597	0,6865048	-0,542658	0,4060681	0,8016478 zinc finger protein, putative
PFIT_07028	21501,36384	8593,267844	10546,20893	10634,42587	3828,361801	6433,020581	6406,20649	6587,514828	10256,108	6496,8607	0,6334626	-0,658669	0,4064154	0,8020536 conserved Plasmodium membrane protein, unknown function
PFIT_04003	2049,042729	1674,894493	2004,646133	1785,963689	1145,330903	1603,880924	1105,926181	1261,176727	1710,6265	1183,5515	0,6918819	-0,531402	0,4068361	0,8026038 rifin
PFIT_12275	3808,2761	5318,94741	5251,599399	6065,192458	3597,637723	4342,143085	3272,148582	3493,364708	4730,6327	3382,7566	0,7150749	-0,483834	0,4074967	0,8034944 histone S-adenosyl methyltransferase, putative
PFIT_03220	18271,57439	4708,222913	6705,840515	7040,360732	4296,717864	4552,105678	3992,621664	4172,313983	7595,8037	4082,4678	0,5374636	-0,895761	0,4077093	0,8034944 cyclic amine resistance locus protein (CARL)
PFIT_13619	195,8477305	95,12842632	44,8801373	70,28738409	174,0792446	96,23285547	146,4961934	138,444964	112,74263	142,47058	1,2636798	0,3376309	0,407718	0,8034944 conserved Plasmodium protein, unknown function
PFIT_14596	72,4523396	150,3029136	110,9536728	89,65792301	110,5265045	137,0589154	75,64967364	70,17073517	111,82538	72,910204	0,6520005	-0,617055	0,4078558	0,8034944 aminotransferase, putative
PFIT_14783	0	1,902568526	3,740011442	2,767219846	1,381581307	14,58073568	6,003942352	6,637772246	4,0620195	6,3208573	1,5560874	0,6379231	0,4081937	0,803567 Plasmodium exported protein (hyp15), unknown function
PFIT_05002	21,50928832	54,54029776	71,68355263	37,08074594	30,39478875	212,8787409	20,413404	20,86156992	71,347902	20,637487	0,2892515	-1,789604	0,4082723	0,803567 rifin (RIF)
PFIT_11491	773,2023117	3163,971459	3221,396522	2217,649985	1623,358035	1574,719453	1262,028682	1805,474051	2095,7163	1533,7514	0,7318507	-0,450379	0,4083189	0,803567 probable protein, unknown function
PFIT_13194	115,4709162	79,90787811	80,410246	95,74580667	157,500269	64,15523698	142,893828	129,9106854	98,865059	136,40226	1,3796811	0,4643349	0,4092517	0,8051227 TBC domain protein, putative
PFIT_13144	56,60339031	150,9371031	135,2637471	125,631781	201,7108708	186,6334167	208,9371939	177,3233443	142,79672	193,1302	1,3524839	0,4356115	0,4096506	0,8056272 conserved Plasmodium protein, unknown function
PFIT_11311	15,84894929	8,878653123	11,22003433	4,980995723	128,4870615	104,9812969	7,204730823	6,637772246	45,732832	6,9212515	0,151341	-2,724125	0,4098423	0,805724 small nucleolar RNA snoR14a
PFIT_05200	6367,88141	3971,294704	4133,335978	4044,568527	2417,767287	6045,173012	2723,388251	3152,941817	4496,6702	2938,165	0,6534091	-0,613942	0,4103735	0,8061067 conserved protein, unknown function
PFIT_10107	21,50928832	7,610274105	6,856687643	4,980995723	17,96055699	14,58073568	1,20078847	7,586025424	12,249756	4,3934069	0,3586526	-1,479341	0,4105035	0,8061067 conserved Plasmodium protein, unknown function
PFIT_13226	5891,280864	8033,912697	9067,034405	7823,483949	5969,812826	8555,975695	4811,559401	5627,882612	7556,9167	5219,721	0,690721	-0,533825	0,4107252	0,8061067 60S ribosomal protein L23a, putative
PFIT_08219	16711,58496	3077,721686	3481,950652	3440,207713	2620,859739	3683,093832	1687,107801	2017,882763	5502,5698	1852,4953	0,33666	-1,570636	0,4108053	0,8061067 conserved Plasmodium protein, unknown function
PFIT_14481	310,1865789	983,6279281	1544,624725	1448,362867	736,3828364	2061,716025	752,894371	794,6361632	1180,8168	773,76527	0,6552797	-0,609817	0,4108226	0,8061067 glutaminyl-peptide cyclotransferase, putative
PFIT_07183	1366,405842	794,6394545	1216,750389	1608,861618	635,5274011	901,0894648	841,7527178	731,1032002	1087,2124	786,42796	0,7233435	-0,467247	0,4109231	0,8061067 small subunit rRNA processing protein, putative
PFIT_12052	1079,992687	379,2453263	416,3879405	418,4036407	685,2643281	1326,846947	840,5519293	800,3256822	717,69014	820,43881	1,1431658	0,1930346	0,4112487	0,8061067 conserved Plasmodium protein, unknown function
PFIT_12172	1540,744284	1708,506537	1744,092002	1651,476804	1619,213291	976,9092904	1023,071777	1156,868877	1540,157	1089,9703	0,7077008	-0,498789	0,4115242	0,8061067 conserved Plasmodium protein, unknown function
PFIT_14346	10816,90789	4604,850023	6255,792472	5843,814871	4896,324151	6065,586042	9456,209205	7797,485883	6413,8792	8626,8475	1,3450281	0,4276363	0,4117239	0,8061067 conserved Plasmodium membrane protein, unknown function
PFIT_14223	169,8101709	86,24977319	75,42356408	65,30638837	81,51329709	32,07761849	120,078847	120,4281536	85,063469	120,2535	1,4136915	0,4994673	0,4117693	0,8061067 conserved Plasmodium protein, unknown function
PFIT_08203	93,96162792	79,90787811	105,3436556	104,0474662	176,8424073	122,4781797	206,5356169	107,1526091	113,76354	156,84411	1,3786853	0,4632932	0,41193	0,8061067 conserved Plasmodium protein, unknown function
PFIT_12084	3350,920707	3002,253135	4036,719016	4718,663281	2446,780494	2519,551125	2406,380095	2462,613503	3345,8146	2434,4968	0,7276245	-0,458734	0,4119363	0,8061067 zinc finger protein, putative
PFIT_10331	10065,21487	2980,690691	4124,609285	4943,361533	2817,044284	3458,550503	2809,845021	3072,340297	4731,5785	2941,0927	0,621588	-0,685969	0,4124363	0,8061067 bromodomain protein, putative
PFIT_14742	324,9034604	358,3170725	572,8450858	538,500982	317,6453715	504,4934544	321,8113101	298,6997511	445,11757	310,25553	0,6970193	-0,52073	0,4124368	0,8061067 GTPase activator, putative
PFIT_13541	2205,268087	617,7005816	605,2585183	578,3489478	531,9088031	874,8441406	462,3035611	437,1447151	902,22151	449,72414	0,4984631	-1,004441	0,4124447	0,8061067 conserved Plasmodium protein, unknown function
PFIT_06122	1,132067806	10,14703214	10,59669908	6,087883661	51,11850835	14,58073568	28,81892329	18,96506356	15,610488	23,891993	1,5305091	0,6140116	0,412459	0,8061067 conserved Plasmodium protein, unknown function
PFIT_12430	2944,508364	4554,749052	4345,26996	3666,012852	3100,268452	2959,889342	2382,364325	2735,710419	3595,1163	2559,0374	0,7118093	-0,490437	0,4124598	0,8061067 rab specific GDP dissociation inhibitor
PFIT_07098	196,9797983	314,5579964	261,1774657	215,843148	247,3030539	137,0589154	139,2914626	179,2198506	228,82006	159,25566	0,6959864	-0,522869	0,4127386	0,8061862 mitochondrial ribosomal protein L1 precursor, putative
PFIT_03217	235,4701037	232,1133602	176,403873	143,895432	468,356063	469,4996888	344,626291	329,9921059	287,62309	337,3092	1,1727473	0,2298922	0,4127856	0,8061862 ATP-dependent RNA helicase, putative
PFIT_09227	2403,379953	6881,59036	8100,864783	7371,320226	5051,061257	5050,766839	3814,904971	4654,026598	5809,8306	4234,4658	0,728845	-0,456316	0,4132675	0,8068489 glutamine synthetase, putative
PFIT_04122	147,1688148	100,8361319	165,1838387	152,7505355	493,2245265	209,9625937	303,799483	242,7528136	211,52107	273,27615	1,2919571	0,3695581	0,4136993	0,8073663 lysine decarboxylase-like protein, putative
PFIT_09201	9,05654245	40,58812856	34,90677746	78,03559966	75,98697187	23,32917708	60,03942352	73,01549471	43,650532	66,527459	1,5240927	0,6079507	0,4138239	0,8073663 long chain fatty acid elongation enzyme, putative
PFIT_08150	4264,499426	3947,829692	4865,13155	4549,309427	3900,204029	4202,168022	2844,667887	3083,719335	4288,1904	2964,1936	0,6912458	-0,532729	0,4139608	0,8073663 conserved Plasmodium protein, unknown function
PFIT_05053	221,88529	191,5252317	116,5636899	275,6150967	169,9345007	26,24532422	256,9687327	251,2870922	166,96152	254,12791	1,5220747	0,6060392	0,4141907	0,8075311 UDP-N-acetyl glucosamine:UMP antiporter
PFIT_10076	12388,218	7932,442376	9461,605612	10309,55426	7138,630611	9016,726943	6606,738164	6364,675331	9374,5296	6485,7067	0,6918434	-0,531483	0,4143309	0,8075311 inositol polyphosphate kinase, putative (IPK1)
PFIT_12158	10,18861026	22,83082232	16,83005149	27,11875449	60,78957749	11,66458854	33,62207717	38,8783803	24,903734	36,250229	1,4556142	0,541628	0,4151637	0,8088757 conserved Plasmodium membrane protein, unknown function
PFIT_12292	6991,650772	3299,053855	3569,840921	2771,093954	2536,583279	4633,757798	2361,950921	2749,934216	3966,9968	2555,9426	0,6443017	-0,634192	0,415468	0,8091898 T-complex protein 1, gamma subunit, putative
PFIT_10233	506,0343094	409,0522332	180,7672197	307,7148469	190,6582203	67,07138411	446,693311	374,5600053	276,88304	410,62666	1,4830329	0,5685556	0,4159577	0,8098647 conserved Plasmodium protein, unknown function
PFIT_01086	835,466041	823,1779824	752,365635	601,0401505	536,053547	784,4435794	494,7248498	489,2986399	722,09116	492,01174	0,6813707	-0,553488	0,4162003	0,810058 selenocysteine-specific elongation factor selB homologue, putative
PFIT_06242	703,0141077	241,6262028	163,313833	198,6863849	497,3692704	347,0215091	511,5358884	372,663499	358,50522	442,09989	1,2331751	0,3023776	0,416914	0,8103609 DNA polymerase 1, putative
PFIT_05264	75,84854302	173,7679254	206,3239645	209,7552643	187,8950577	341,1892148	135,6890972	122,32466	199,12999	129,00688	0,6478526	-0,626263	0,4169682	0,8105976 conserved Plasmodium protein, unknown function, putative
PFIT_12075	39,62237322	39,95393905	61,08685355	99,06647049	48,35534573	69,98753125	32,4212887	44,56789937	59,678752	38,494594	0,6450301	-0,632562	0,4170373	0,8105976 conserved Plasmodium protein, unknown function
PFIT_12493	3404,127893	2054,139819	3359,776945	3584,103145	1692,437101	3350,653059	1993,308861	2041,589092	2907,5397	2017,449	0,6938681	-0,527267	0,4170507	0,8105976 conserved Plasmodium protein, unknown function
PFIT_10182	8883,336076	3334,568437	3546,154182	3717,483141	3127,900078	2712,016836	2658,545674	2762,261508	4220,2431	2710,4036	0,6422387	-0,638818	0,4172359	0,8106789 conserved Plasmodium protein, unknown function
PFIT_02130	583,0149202	194,0619897	212,5573169	205,8811565	146,4476185	285,7824193	159,7048666	160,2547871	271,2909	159,97983	0,5896985	-0,761951	0,4174924	0,8106982 conserved Plasmodium protein, unknown function
PFIT_14441	28929,99279	14810,2276	17994,44172	19368,87859	10806,72898	11851,22196	11559,99061	12769,1773	17293,582	12164,584	0,7034161	-0,50755	0,4178948	0,8106982 guanine nucleotide exchange factor, putative
PFIT_14567	1570,178047	2075,068073	2913,468913	2768,326734	2145,595769	2919,063283	1642,678628	1685,045897	2398,6168	1663,8623	0,6936757	-0,527667	0,4178997	0,8106982 gamma-adaptin, putative
PFIT_10315	8872,015398	5270,114818	7167,731928	8228,05149	3846,322358	5718,564533	4615,83088	4641,699306	6517,1334	4628,7651	0,7102456	-0,49361	0,417925	0,8106982 mRNA-decapping enzyme subunit 1, putative
PFIT_12455	37,35823761	46,29583414	33,66010298	28,7790864	37,30269528	67,07138411	45,62996188	63,53296293	41,744557	54,581462	1,3075109	0,386823	0,4179625	0,8106982 ubiquitin conjugating enzyme E2, putative
PFIT_06080	1788,667134	3264,173402	4555,33936	4859,23805	3187,308074	4406,298322	2516,852634	2681,659987	3676,8365	2599,2563	0,7069274	-0,500366	0,4183023	0,8109765 Zn2 or Fe2 permease
PFIT_09022	662,2596667	885,9627438	1086,473324	1172,194327	705,9880477	536,5710729	582,3824082	658,0877055	841,57486	620,23506	0,7369933	-0,440277	0,4183981	0,8109765 serine/threonine protein kinase, FIKK family
PFIT_14233	387,1671897	383,0504633	326,0043307	371,3609033	392,3690911	347,0215091	258,1695211	242,7528136	367,82891	250,46117	0,6809176	-0,554448	0,4185361	0,8109765 DnaJ protein, putative
PFIT_08305	452,8271225	288,5562265	310,4209497	288,8977519	867,6330606	475,3319831	608,7997545	492,1433994	447,27785	550,47158	1,230715	0,2994968	0,4190442	0,8112628 conserved Plasmodium protein, unknown function
PFIT_13230	2225,645307	1137,101789	1260,383856	1220,343952	1050,001793	1446,408979	941,4181608	914,1160636	1389,9809	927,76711	0,6674675	-0,58323	0,4191881	0,8112628 vacuolar protein sorting 52 homologue
PFIT_13420	1827,157439	498,4729539	597,1551602	552,3370813	422,7638798	1323,930799	439,4885802	451,3685127	870,30289	445,42855	0,5118087	-0,966324	0,4192924	0,8112628 phosphoenolpyruvate carboxykinase
PFIT_14425	835,466041	734,3914512	2391,111982	2553,03703	1425,791908	1743,855987	1287,24524	1068,681332	1613,9427	1177,9633	0,7298668	-0,454295	0,4193142	0,8112628 serine/threonine protein kinase, putative
PFIT_14376	417,7330205	310,1186698	347,1977288	270,080657	151,9739437	163,3042396	230,5513863	172,5820784	276,73471	201,56673	0,7283753	-0,457246	0,4195674	0,8112628 conserved Plasmodium protein, unknown function
PFIT_10084	1104,898179	1224,619941	1217,99706	1021,657567	1088,68607	1539,725687	1527,402934	1627,202453	1199,5974	1577,3027	1,31486	0,3949092	0,4195677	0,8112628 adenylate kinase
PFIT_12497	75,84854302	22,83082232	19,94672769	18,26365098	120,1975737	20,41302995	85,2559814	46,46440572	46,250058	65,860194	1,4240024	0,5099516	0,4198774	0,8112628 cutA, putative
PFIT_13300	78,11267863	50,10097119	105,9669908	89,65792301	230,7240782	110,8135911	153,7009242	130,8589386	110,89604	142,27993	1,2830028	0,3595243	0,4199433	0,8112628 GPI transamidase subunit PIG-U, putative
PFIT_03236	726,7875316	1055,925532	810,3358124	915,949769	830,3303653	1122,716647	1209,19399	1239,366904	910,34094	1224,2804	1,3448593	0,4274552	0,4200088	0,8112628 survival of motor neuron (SMN)-like protein
PFIT_12230	604,5242086	527,0114818	992,9730378	1135,667025	823,4224588	1032,316086	587,1855621	588,8652235	852,65238	588,02539	0,6896426	-0,536079	0,4201182	0,8112628 DNA gyrase subunit A
PFIT_13131	41,88650883	188,9884736	238,737397	196,4726091	207,237196	279,950125	117,6772701	136,5484576	192,21205	127,11286	0,6613158	-0,596589	0,4203305	0,8113958 conserved Plasmodium protein, unknown function
PFIT_11291	626,0334969	705,2187338	1027,256476	1118,510262	747,4354869	1548,474129	644,8234086	639,122642	962,15476	641,97303	0,6672243	-0,583756	0,4211854	0,8126711 multiprotein bridging factor type 1, putative
PFIT_12244	1873,572219	2746,674763	2071,343003	3065,526145	2084,806192	985,6577318	1683,505436	1539,963161	2137,93	1611,7343	0,7538761	-0,407601	0,421403	0,8126711 conserved Plasmodium protein, unknown function
PFIT_13527	39,62237322	121,1301962	120,3037014	111,2422378	111,9080858	110,8135911	58,83863505	77,7567606	102,50336	68,297698	0,6662971	-0,585762	0,4215029	0,8126711 transcription initiation factor TFIIH, putative
PFIT_14706	86,03715328	195,3303687	167,6771796	141,1282121	120,1975737	137,0589154	92,46071223	103,3595964	141,23823	97,910154	0,693227	-0,5286	0,421566	0,8126711 conserved Plasmodium protein, unknown function
PFIT_08325	57,73545812	31,07528593	32,4134325	37,08074594	56,64483357	26,24532422	45,62996188	64,4812161	40,19918	55,055589	1,3695699	0,453723	0,4218063	0,8128573 early transcribed membrane protein 8
PFIT_13005	14,71688148	60,24800333	87,89026888	64,7529444	81,51329709	294,5308607	25,21655788	35,08536759	100,60871	30,150963	0,2996854	-1,738479	0,4221451	0,8132331 Plasmodium exported protein (PHISTa-like), unknown function, pseudogene
PFIT_11295	69,05613618	49,46678168	16,83005149	17,15676305	31,77637005	11,66458854	50,43311576	44,56789937	32,658448	47,500508	1,4544631	0,5404867	0,4224056	0,8133708 CCR4-NOT transcription complex subunit 2, putative
PFIT_09059	29,43376296	17,75730625	26,18008009	24,90497861	37,30269528	23,32917708	10,80709623	18,01681038	26,484667	14,411953	0,5441622	-0,877891	0,4228837	0,8133708 autophagy-related protein 3, putative (ATG3)
PFIT_11110	8090,888611	6610,15725	8479,229274	10574,10048	5460,009324	6485,511229	5252,24877	5725,552689	7616,6494	5488,9007	0,7206451	-0,472639	0,4229956	0,8133708 pre-mRNA-processing factor 6, putative
PFIT_14665	215,0928832	539,695272	733,0422426	644,2087801	370,2637902	600,7263099	368,6420604	376,4565117	517,17155	372,54929	0,7203592	-0,473212	0,4230465	0,8133708 conserved Plasmodium protein, unknown function
PFIT_04030	1219,237027	354,5119354	703,122151	963,5459504	283,2241679	705,7076068	467,106715	502,5741843	704,89147	484,84045	0,6878228	-0,539891	0,4231006	0,8133708 conserved Plasmodium protein, unknown function
PFIT_05012	3415,448572	5622,724185	5880,544657	6868,239658	4019,020021	3429,389031	3568,743334	3706,721673	4872,561	3637,7325	0,7465751	-0,421641	0,4233265	0,8133708 heat shock protein 40, type II
PFIT_13041	135,8481368	633,5553193	475,6047883	491,4582446	473,8823882	618,2231927	292,9923868	352,7501822	471,42868	322,87128	0,6848783	-0,54608	0,4233908	0,8133708 DNA-directed RNA polymerase 2, putative
PFIT_14619	3072,432026	5830,738344	5711,620807	5452,529985	3611,453536	3995,121575	3129,254754	3594,827798	4612,316	3362,0413	0,7289269	-0,456154	0,4234382	0,8133708 60S ribosomal protein L27, putative
PFIT_05094	16539,51065	13773,32775	15363,34367	16833,55177	8825,541387	13361,78617	9130,795529	10811,98274	14116,177	9971,3891	0,7063803	-0,501483	0,423571	0,8133708 RNA polymerase I
PFIT_04207	7915,418102	5857,374303	7988,041104	8741,647493	4426,586507	6943,346329	4466,93311	5356,682203	6978,7356	4911,8077	0,7038249	-0,506712	0,423666	0,8133708 pre-mRNA-splicing helicase BRR2, putative
PFIT_05108	275,0924769	100,8361319	112,8236785	127,8455569	81,51329709	104,9812969	96,06307764	81,54977331	133,84874	88,806425	0,6634835	-0,591868	0,4238387	0,8133708 conserved Plasmodium protein, unknown function
PFIT_09340	1259,991468	671,6066898	518,6149199	505,2943439	987,8306343	915,6702005	1050,689912	952,9944439	809,83471	1001,8422	1,2370946	0,3069559	0,4239422	0,8133708 gamma-tubulin complex component, putative
PFIT_11069	255,8473242	108,446406	119,6803661	94,63891873	163,0265942	139,9750625	94,86228917	89,13579873	146,93578	91,999044	0,6261174	-0,675495	0,4241297	0,8134546 apicoplast ribosomal protein S15 precursor, putative
PFIT_11234	2219,984968	3910,412511	3284,353381	2507,10118	1950,792805	2507,886536	2032,93488	1930,64347	2730,0886	1981,7892	0,7259066	-0,462144	0,4243736	0,8136464 conserved Plasmodium protein, unknown function
PFIT_07278	1468,291945	1225,88832	1624,411636	1769,36037	1095,593976	1528,061099	959,4299879	1070,577838	1451,9346	1015,0039	0,69907	-0,516491	0,4250248	0,8137663 RNA binding protein, putative
PFIT_11414	3735,823761	2132,779318	2664,134817	3398,145971	1152,23881	1309,350064	1675,099916	1858,576229	2398,7455	1766,8381	0,7365676	-0,44111	0,4250661	0,8137663 conserved Plasmodium protein, unknown function
PFIT_06247	288,6772906	183,280768	206,3239645	164,3728589	139,539712	297,4470078	115,2756932	153,6170148	213,2736	134,44635	0,6303938	-0,665675	0,4251998	0,8137663 conserved Plasmodium protein, unknown function
PFIT_09165	1403,76408	1595,620804	1877,485744	2011,768828	1760,134585	1274,356298	2509,647903	2188,568335	1653,8551	2349,1081	1,4203833	0,5062803	0,4252988	0,8137663 conserved Plasmodium protein, unknown function
PFIT_09322	9,05654245	33,61204397	12,46670481	20,47742686	17,96055699	14,58073568	21,61419247	35,08536759	18,025668	28,34978	1,572745	0,6532848	0,425354	0,8137663 conserved Plasmodium protein, unknown function
PFIT_05337	207,1684085	427,4437289	585,9351259	452,1637228	562,3035918	1274,356298	280,9845021	340,4228909	584,89515	310,7037	0,5312126	-0,912639	0,4253895	0,8137663 cell cycle regulator protein, putative
PFIT_08294	6430,14514	2453,679209	3323,623501	3041,174611	2409,477799	3802,655865	2331,93121	2405,718313	3576,7927	2368,8248	0,6622762	-0,594495	0,4254835	0,8137663 ubiquitin conjugation factor E4 B, putative (UBE4B)
PFIT_12117	250,1869852	143,9610185	191,3639188	142,2351001	161,6450129	221,6271823	114,0749047	123,2729131	185,16987	118,67391	0,6408921	-0,641847	0,4255871	0,8137663 peripheral plastid protein 1, putative (PPP1)
PFIT_10020	2247,154595	2680,719054	3246,329931	3362,172113	2428,819937	2691,603806	1927,265495	2031,158307	2776,1332	1979,2119	0,7129384	-0,488151	0,4259112	0,8138555 RNA binding protein, putative
PFIT_02035	1278,104553	2468,899758	1951,662637	2218,756873	1819,542581	2242,517147	2861,478925	2671,229202	1996,5806	2766,3541	1,3855459	0,4704545	0,4259216	0,8138555 conserved Plasmodium protein, unknown function
PFIT_14127	1711,686523	322,1682705	403,9212357	334,8336014	272,1715174	486,9965716	252,1655788	200,0814206	588,62962	226,1235	0,3841524	-1,380249	0,4261224	0,8139642 conserved protein, unknown function
PFIT_03054	18514,96897	10139,42187	9254,034977	10496,61832	6822,248492	10466,05207	14979,83617	14253,19352	10948,891	14616,515	1,3349768	0,4168146	0,4268328	0,814856 conserved Plasmodium protein, unknown function
PFIT_13472	105,282306	94,49423681	61,71018879	67,52016424	160,2634316	55,40679557	150,0985588	98,61833051	90,77952	124,35844	1,3698954	0,4540657	0,4269858	0,814856 conserved Plasmodium protein, unknown function
PFIT_04007	6,792406838	21,5624433	172,6638636	152,7505355	52,50008965	271,2016836	45,62996188	45,51615254	112,91184	45,573057	0,4036163	-1,308944	0,4270214	0,814856 Plasmodium exported protein (PHISTa), unknown function
PFIT_09090	396,2237322	822,5437929	466,878095	345,3490368	420,0007172	556,9841029	337,4215602	354,6466886	501,32999	346,03412	0,6902324	-0,534846	0,4272934	0,8151 transporter, putative
PFIT_05224	90,5654245	169,9627884	209,4406407	206,4346005	158,8818503	361,6022448	124,8820009	131,8071917	199,48126	128,3446	0,6433918	-0,636231	0,4277564	0,8155279 conserved Plasmodium protein, unknown function, putative
PFIT_02139	38,49030541	67,85827744	61,71018879	55,89784089	41,4474392	32,07761849	38,42523105	30,3441017	49,580278	34,384666	0,693515	-0,528001	0,4278359	0,8155279 conserved Plasmodium protein, unknown function
PFIT_13391	58,86752593	65,95570891	102,2269794	110,6887938	245,9214726	355,7699505	217,3427132	163,0995466	156,57174	190,22113	1,2149136	0,2808537	0,4279503	0,8155279 ABC transporter (MDR family), putative
PFIT_12373	8236,925359	1393,94854	1672,40845	1896,099038	914,606825	1557,22257	781,7132943	912,2195572	2611,8685	846,96643	0,3242761	-1,624706	0,4281762	0,8156836 rRNA processing WD-repeat protein, putative
PFIT_05199	11936,52295	7250,688654	10569,27233	12267,63902	7760,342199	15831,7628	7322,408093	7317,669775	10936,038	7320,0389	0,6693502	-0,579167	0,4286249	0,8161428 conserved Plasmodium protein, unknown function
PFIT_10384	0	0	0	0,553443969	0	2,916147135	2,401576941	0,948253178	0,5782652	1,6749151	2,896448	1,5342848	0,4287059	0,8161428 Plasmodium exported protein (PHISTb), unknown function
PFIT_06055	7055,046569	4181,845621	6153,565492	8298,892318	1791,910955	2723,681424	3382,621121	4160,934945	5034,1571	3771,778	0,7492373	-0,416505	0,4290981	0,8166044 conserved Plasmodium protein, unknown function
PFIT_05109	35,09410199	98,93356337	128,4070595	121,2042293	37,30269528	204,1302995	58,83863505	67,32597564	104,17866	63,082305	0,6055204	-0,723752	0,4294545	0,8166044 conserved Plasmodium protein, unknown function
PFIT_14490	1676,592421	622,7740976	761,7156636	559,5318529	392,3690911	1883,831049	452,6972534	547,1420837	982,80236	499,91967	0,5086675	-0,975205	0,4296538	0,8166044 conserved Plasmodium protein, unknown function
PFIT_05310	2515,454666	3167,776596	3688,274617	2936,573701	2553,162255	6485,511229	2243,072863	2361,150413	3557,7922	2302,1116	0,6470619	-0,628024	0,429689	0,8166044 conserved Plasmodium protein, unknown function
PFIT_08236	239,9983749	395,7342535	334,1076888	275,0616527	757,106556	352,8538034	516,3390423	494,0399057	392,47705	505,18947	1,2871822	0,3642163	0,430036	0,8166044 2-oxoglutarate dehydrogenase E1 component
PFIT_11215	91,69749231	75,46855154	64,82686499	89,10447904	99,47385408	61,23908984	128,4843663	98,61833051	80,301722	113,55135	1,4140587	0,499842	0,4301186	0,8166044 conserved Plasmodium protein, unknown function
PFIT_03106	163,0177641	594,2355697	618,3485584	641,9950043	415,8559733	414,0928932	345,8270795	359,3879545	474,59096	352,60752	0,7429714	-0,428621	0,430185	0,8166044 sybindin-like protein, putative
PFIT_09269	940,748347	539,4012077	439,4513444	978,4889375	509,8035022	268,2855365	455,0988031	438,0929682	609,36315	446,5959	0,7328896	-0,448332	0,4303007	0,8166044 conserved Plasmodium protein, unknown function
PFIT_09055	603,3921407	534,6217559	425,7379691	464,3394902	294,2768183	323,692332	599,1934468	636,2778824	441,01008	617,73566	1,4007291	0,486178	0,4304575	0,8166044 dynein heavy chain, putative
PFIT_07216	7421,836538	3184,265524	4587,124033	4420,910426	2688,557223	5491,105056	3036,794042	3181,389412	4632,2998	3109,0917	0,6711767	-0,575235	0,4308553	0,8166044 conserved Plasmodium protein, unknown function
PFIT_11322	2139,608154	575,2098845	676,942071	802,4937553	418,6191359	449,0866589	449,094888	429,5586896	843,65994	439,32679	0,5207392	-0,941367	0,4308693	0,8166044 conserved Plasmodium protein, unknown function
PFIT_06222	95,09369573	76,73693056	115,9403547	70,84082806	75,98697187	58,32294271	54,03548117	56,89519068	82,153621	55,465336	0,6751417	-0,566738	0,430908	0,8166044 EGF-like membrane protein, putative
PFIT_05357	29,43376296	61,51638235	53,60683067	46,48929341	52,50008965	72,90367838	25,21655788	38,8783803	52,741673	32,047469	0,6076309	-0,718733	0,4311134	0,8166044 unspecified product
PFIT_10313	84,90508547	29,1727174	24,31007437	29,33253037	27,63162613	34,99376562	28,81892329	17,0685572	38,390967	22,94374	0,5976338	-0,742666	0,4312274	0,8166044 conserved Plasmodium protein, unknown function
PFIT_05058	4467,139564	858,0584054	1123,873438	862,265704	1557,042133	2892,817958	889,7842566	880,9272024	1960,1995	885,35573	0,4516661	-1,146671	0,4313734	0,8166044 small ubiquitin-related modifier, putative
PFIT_14135	3391,675148	2065,55523	2280,160309	1830,79265	1300,06801	4511,279618	1412,127241	1793,14676	2563,2552	1602,637	0,6252351	-0,677529	0,4314692	0,8166044 actin II
PFIT_13381	230,5637994	97,66518435	118,4336957	124,5248931	348,1584893	61,23908984	261,7718866	219,0464841	180,09753	240,40919	1,3348833	0,4167137	0,4316687	0,8166044 serine/threonine protein kinase, putative
PFIT_13529	247,9228496	223,8688966	247,4640904	212,5224842	214,1451025	670,7138411	147,6969819	176,3750911	302,77288	162,03604	0,5351736	-0,901921	0,4318405	0,8166044 conserved Plasmodium protein, unknown function
PFIT_14487	92,82956012	19,65987477	24,93340961	18,26365098	22,10530091	113,7297383	18,01182706	15,17205085	48,586922	16,591939	0,3414898	-1,550086	0,4318683	0,8166044 conserved Plasmodium protein, unknown function
PFIT_12008	93,96162792	225,7714651	225,0240217	236,8740188	272,1715174	1437,660538	63,64178893	71,11898835	402,77147	67,380389	0,1672919	-2,579561	0,4322609	0,8166044 RESA-like protein with PHIST and DnaJ domains
PFIT_05229	1,132067806	8,878653123	2,493340961	2,213775877	6,907906533	8,748441406	7,204730823	9,48253178	5,0623643	8,3436313	1,6481689	0,7208641	0,4323572	0,8166044 iron-sulfur assembly protein, putative, putative
PFIT_13405	0	0,634189509	0,62333524	0,553443969	9,671069147	14,58073568	0	0	4,3437956	0	0	#DIV/0!	0,4324095	0,8166044 small nucleolar RNA snoR20
PFIT_01094	1775,08232	206,7457799	272,3975	228,0189153	216,9082651	256,6209479	97,26386611	112,8421282	492,62895	105,053	0,2132497	-2,229384	0,4324485	0,8166044 adenylate kinase-like protein 1 (AKLP1)
PFIT_13625	82,64094986	86,8839627	59,84018307	65,30638837	291,5136557	163,3042396	186,1222129	126,1176727	124,9149	156,11994	1,2498104	0,3217093	0,4326006	0,8166044 DNase I-like protein, putative
PFIT_12363	1,132067806	12,04960067	4,986681922	7,748215569	9,671069147	8,748441406	13,20867318	12,32729131	7,3893461	12,767982	1,7278907	0,7890119	0,4326315	0,8166044 p25-alpha family protein, putative
PFIT_04170	40,75444103	116,6908696	102,8503146	106,2612421	139,539712	242,0402122	63,64178893	84,39453284	124,68947	74,018161	0,59362	-0,752388	0,4327021	0,8166044 conserved Plasmodium protein, unknown function

PFIT_07191	117,7350519	117,9592486	98,48696797	87,9975911	165,7897568	183,7172695	162,1064435	159,3065339	128,61431	160,70649	1,2495226	0,321377	0,4333417	0,8175387	conserved Plasmodium protein, unknown function
PFIT_13002	16,98101709	7,610274105	5,610017163	1,660331908	5,526325227	8,748441406	4,803153882	0,948253178	7,6894012	2,8757035	0,3739828	-1,418956	0,4335421	0,8176439	rifin (RIF)
PFIT_05056	268,3000701	243,5287714	274,890841	262,3324414	239,0135661	478,2481302	213,7403477	170,685572	294,38564	192,21296	0,6529291	-0,615002	0,4339337	0,8181085	conserved Plasmodium protein, unknown function
PFIT_14036	3088,280976	2394,699585	3536,804153	2861,305321	1977,04285	5042,018397	2019,726207	2152,534714	3150,0252	2086,1305	0,6622583	-0,594534	0,4340777	0,8181085	pre-mRNA splicing factor, putative
PFIT_05147	173,2063744	582,1859691	527,3416133	401,2468777	533,2903844	533,6549258	285,787656	344,2159036	458,48769	315,00178	0,6870452	-0,541523	0,4344335	0,8184584	ribose 5-phosphate epimerase, putative
PFIT_14353	172,0743066	353,8777459	287,980881	257,3514457	190,6582203	312,0277435	145,2954049	214,3052182	262,32839	179,80031	0,6854016	-0,544978	0,4345528	0,8184584	queuine tRNA-ribosyltransferase, putative
PFIT_13039	5,660339031	31,70947544	34,90677346	37,08074594	19,34213829	17,49688281	39,62601953	40,77488665	24,366059	40,200453	1,6498545	0,7223388	0,4348176	0,8186846	cyclin
PFIT_08061	147,1688148	225,1372756	200,0906121	116,7766775	258,3557043	110,8135911	250,9647903	236,1150413	176,39045	243,53992	1,3806865	0,4653858	0,4353232	0,8193637	mitogen-activated protein kinase organizer 1, putative
PFIT_13223	7652,77837	7563,344082	8021,701207	6055,783911	6201,918486	8520,98193	4386,480283	5558,66013	7336,0847	4972,5702	0,6778234	-0,561019	0,4354773	0,819381	60S ribosomal protein L6, putative
PFIT_03115	90,5654245	199,1355058	370,884468	443,8620633	245,9214726	239,1240651	416,6735992	367,9222331	264,9155	392,29792	1,4808417	0,5664174	0,4359479	0,8199938	dolichyl-diphosphooligosaccharide--protein glycosyltransferase subunit 1, putative
PFIT_08043	982,6348559	1683,138956	1949,169296	2374,828072	1273,817965	1746,772134	1211,595567	1235,573891	1668,3935	1223,5847	0,733391	-0,447346	0,4363389	0,8204563	conserved Plasmodium protein, unknown function
PFIT_02118	5866,375372	6682,454854	5663,000658	5610,81496	4602,047332	4595,847885	3878,54676	4026,282994	5503,4235	3952,4149	0,7181739	-0,477595	0,4367332	0,8207189	conserved Plasmodium membrane protein, unknown function
PFIT_08096	3093,941315	3162,068891	3401,540406	3331,732695	2666,451922	2924,895577	2192,639747	2217,964183	3096,7718	2205,302	0,7121293	-0,489789	0,4367687	0,8207189	conserved Plasmodium membrane protein, unknown function
PFIT_12314	4279,216308	2557,686289	2630,474714	2061,025341	1518,357856	2849,075751	1767,560629	1839,611165	2649,306	1803,5859	0,6807767	-0,554746	0,4369166	0,8207241	pre-mRNA-splicing factor ATP-dependent RNA helicase PRP2, putative
PFIT_01073	264,9038667	316,4605649	400,1812243	353,0972523	436,5796929	921,5024948	246,1616364	280,6829407	448,78752	263,42229	0,5869644	-0,768655	0,4370901	0,8207775	conserved Plasmodium protein, unknown function
PFIT_05280	2008,288288	2567,833321	2872,328787	2704,127233	1583,292177	1026,483792	1474,568242	1746,682354	2127,0589	1610,6253	0,7572077	-0,401239	0,4372565	0,8208173	transporter, putative
PFIT_10333	1710,554455	584,7227271	688,1621053	622,0710214	859,3435727	746,5336667	1074,705681	1106,611459	868,56459	1090,6586	1,2557023	0,3284945	0,4382276	0,8223518	ubiquitin conjugating enzyme, putative
PFIT_14086	452,8271225	433,785624	407,0379119	335,9404893	277,6978426	323,692332	255,7679442	267,4073962	371,83022	261,58767	0,7035137	-0,507349	0,4383834	0,8223518	conserved Plasmodium protein, unknown function
PFIT_14192	61,13166154	38,05137053	39,89345538	37,08074594	73,22380925	145,8073568	46,83075035	19,91331674	65,864733	33,372034	0,5066753	-0,980867	0,4385101	0,8223518	conserved Plasmodium protein, unknown function
PFIT_09198	100,7540348	150,3029136	162,0671625	108,475018	193,4213829	457,8351003	103,2678085	88,18754556	195,47594	95,727677	0,4897159	-1,029983	0,4387571	0,8225424	pyridoxal 5'-phosphate dependent enzyme class III, putative
PFIT_04089	1357,3493	1015,971593	1102,68004	1092,498395	1011,317516	1198,536473	736,0833324	807,9117077	1129,7256	771,99752	0,6833496	-0,549304	0,4391588	0,8229435	protein phosphatase, putative
PFIT_10266	804,9002103	542,23203	947,4695652	899,8998939	440,7244368	764,0305495	492,3232729	549,9868432	733,20945	521,15506	0,7107861	-0,492513	0,4392621	0,8229435	mitochondrial ribosomal protein S22 precursor, putative
PFIT_12526	2879,980499	7281,76394	1919,249205	7132,785875	4106,059643	1116,884353	3021,183792	2663,643177	4072,7873	2842,4135	0,6979037	-0,5189	0,4394823	0,8230834	reticulocyte binding protein homologue 3, pseudogene
PFIT_14333	657,7313954	603,7484124	757,352317	700,660065	589,9352179	1242,27868	482,7169651	511,1084629	758,61768	496,91271	0,6550239	-0,610381	0,4397638	0,8233379	conserved protein, unknown function
PFIT_11426	49,81098348	43,1248866	47,37347826	54,79095295	26,25004483	29,16147135	66,04336588	60,68820339	41,75197	63,365785	1,5176717	0,6018598	0,4400302	0,8235641	inner membrane complex protein 1b, putative
PFIT_12160	3705,25793	656,3861416	830,9058753	776,4818888	656,2511207	589,0617213	392,6578298	460,8510445	1202,3908	426,75444	0,3549216	-1,494428	0,4404987	0,8239689	signal recognition particle SRP19
PFIT_13090	113,2067806	31,07528593	39,27012014	21,03087083	45,59218312	110,8135911	26,41734635	29,39584852	60,164805	27,906597	0,4638359	-1,108314	0,4405378	0,8239689	conserved Plasmodium protein, unknown function
PFIT_05066	373,5823761	154,7422401	139,6270938	197,579497	270,7899361	303,2793021	166,9095974	135,6002045	239,93341	151,2549	0,6304037	-0,665652	0,4410801	0,8245601	histone deacetylase, putative, pseudogene
PFIT_13141	811,6926171	1171,348023	1138,833484	1047,11599	736,3828364	1055,645263	641,2210432	769,9815805	993,50304	705,60131	0,7102156	-0,493671	0,4411455	0,8245601	TFIIH basal transcription factor subunit
PFIT_02208	0	0	0,62333524	0,553443969	0	0	1,20078847	1,896506356	0,1961299	1,5486474	7,8960305	2,9811276	0,4413318	0,8246358	stevor
PFIT_14357	18867,04206	13446,72015	14248,19692	15484,80881	8777,186041	11577,10413	9064,752164	10543,62709	13733,51	9804,1896	0,7138881	-0,48623	0,4415059	0,824681	dynein-related AAA-type ATPase, putative
PFIT_08275	373,5823761	651,3126255	838,3858982	877,2086912	545,7246161	714,4560482	489,9216959	482,6608676	666,77838	486,29128	0,7293147	-0,455387	0,4416476	0,824681	anamorsin related protein, putative
PFIT_06266	6341,843851	8326,274061	9153,678004	9209,307647	6783,564216	6756,712913	5452,780444	5869,687172	7761,8968	5661,2338	0,7293622	-0,455293	0,4419811	0,8247436	transportin
PFIT_07283	8825,600618	2000,233711	1853,799005	1845,182193	1485,199905	1947,986286	1053,091489	1265,917993	2993,0003	1159,5047	0,3874055	-1,368084	0,4420927	0,8247436	BRIX protein, putative
PFIT_14733	28299,43102	7949,565493	7603,443261	8598,858949	5033,1007	4324,646202	4445,318918	5075,999262	10301,508	4760,6591	0,4621323	-1,113622	0,4421185	0,8247436	histone deacetylase, putative
PFIT_05045	0	11,41541116	4,363346682	6,087883661	37,30269528	5,832294271	15,61025012	18,01681038	10,833605	16,81353	1,5519792	0,6341093	0,442881	0,8253249	conserved Plasmodium protein, unknown function
PFIT_06186	181,130849	168,6944093	97,86363273	94,63891873	281,8425866	104,9812969	228,1498094	174,4785848	154,85862	201,3142	1,2999871	0,3784973	0,4429832	0,8253249	conserved Plasmodium protein, unknown function
PFIT_14274	0	6,341895088	1,870005721	1,106887938	19,34213829	2,916147135	10,80709623	6,637772246	5,2628457	8,7224342	1,6573608	0,7288877	0,4430821	0,8253249	MORN repeat protein, putative
PFIT_07141	6604,483582	1595,620804	2615,514668	2634,393293	1018,225423	2461,228182	1415,729607	1717,286505	2821,5777	1566,5081	0,5551887	-0,84895	0,4431201	0,8253249	RNA binding protein, putative
PFIT_09331	84,90508547	45,02745512	38,6467849	30,43941831	127,1054802	67,07138411	94,86228917	66,37772246	65,532601	80,620006	1,2302275	0,2989251	0,4431689	0,8253249	apicoplast ribosomal protein S6, putative (RPS6)
PFIT_08264	124,5274587	60,88219284	25,55674485	35,973858	22,10530091	34,99376562	60,03942352	80,60152013	50,67322	70,320472	1,3877246	0,4727212	0,443524	0,8253249	membrane skeletal protein IMC1-related
PF_apicopla	11,32067806	36,78299151	33,66010298	32,09975021	49,73692704	26,24532422	10,80709623	26,55108898	31,640962	18,679093	0,5903453	-0,760369	0,4436714	0,8253249	apicoplast ribosomal protein S4
PFIT_06180	252,4511208	188,9884736	163,9371682	134,4868845	180,9871512	163,3042396	120,078847	121,3764068	180,69251	120,72763	0,6681385	-0,581781	0,4437472	0,8253249	conserved Plasmodium protein, unknown function
PFIT_03014	0	1,268379018	3,740011442	2,767219846	2,763162613	0	4,803153882	4,74126589	1,7564622	4,7722099	2,7169443	1,441985	0,443842	0,8253249	Plasmodium exported protein, unknown function
PFIT_11113	398,4878678	320,2657019	251,2041018	201,4536048	533,2903844	431,589776	456,2996188	411,5418793	356,04857	433,92075	1,2187122	0,2853575	0,4438894	0,8253249	vacuolar sorting protein 35, putative
PFIT_11212	3376,958266	2037,016702	1521,561322	1677,488671	1139,804578	1536,80954	1195,985317	1410,052476	1881,6065	1303,0189	0,6925034	-0,530107	0,4444649	0,8258736	alpha/beta hydrolase fold domain containing protein, putative
PFIT_12427	16,98101709	27,27014888	25,55674485	35,42041403	93,94752885	23,32917708	54,03548117	48,36091208	37,084172	51,198197	1,3805943	0,4652894	0,4444765	0,8258736	conserved protein, unknown function
PFIT_01018	2226,777375	1163,103559	1179,350275	991,2181488	978,1595651	1705,946074	858,5637564	943,5119121	1374,0925	901,03783	0,655733	-0,60882	0,4447632	0,8260736	conserved Plasmodium protein, unknown function
PFIT_04109	71,3202718	188,3542841	120,3037014	112,9025697	111,9080858	69,98753125	79,25203905	83,44627967	112,46274	81,349159	0,7233432	-0,467248	0,4448763	0,8260736	phosphopantothenoylcysteine synthetase, putative
PFIT_10020	12065,57868	5834,543481	7302,995675	7746,001793	4202,770335	5625,247824	4780,338901	5328,234607	7129,523	5054,2868	0,7089236	-0,496298	0,4453996	0,8264104	RNA binding protein, putative
PFIT_02134	2025,269305	3481,066214	4998,525292	5189,644099	3375,203132	4187,587286	2618,919654	3015,445106	3876,2159	2817,1824	0,7267868	-0,460396	0,4454607	0,8264104	T-complex protein 1, putative
PFIT_09235	1828,289507	698,8768387	446,9313673	406,7813174	490,4613639	904,005612	359,0357527	471,2818295	795,891	415,15879	0,5216277	-0,938908	0,445496	0,8264104	conserved Plasmodium protein, unknown function
PFIT_14689	14,71688148	30,44109642	24,93340961	23,24464671	29,01320744	29,16147135	43,22838494	31,29235487	25,251786	37,26037	1,4755539	0,5612566	0,4460543	0,8269884	conserved Plasmodium protein, unknown function
PFIT_12251	652,0710564	384,3188423	402,05123	532,9665423	299,8031435	312,0277435	315,8073677	303,441017	430,53976	309,62419	0,7191535	-0,475628	0,4462402	0,8269884	conserved Plasmodium protein, unknown function
PFIT_14344	289,8093584	103,3728899	99,73363845	110,1353499	58,02641488	61,23908984	70,84651976	67,32597564	120,38612	69,086248	0,5738722	-0,801199	0,4462462	0,8269884	conserved Plasmodium protein, unknown function
PFIT_04124	1441,122317	1497,32143	2383,633959	1867,319952	1785,003048	2534,131861	1335,276779	1314,278905	1918,0888	1324,7778	0,690676	-0,533919	0,4463975	0,8269977	ubiquitin carboxyl-terminal hydrolase 13, putative (USP13)
PFIT_09045	219,6211544	143,326829	178,897214	158,8384192	96,71069147	46,65835417	214,9411362	198,1849142	140,67544	206,56303	1,4683659	0,5542115	0,4468544	0,8273207	conserved protein, unknown function
PFIT_11307	3131,299552	2487,925443	3221,396522	3046,70905	2703,754617	6931,681741	2065,356169	2449,337959	3587,1278	2257,3471	0,6292909	-0,668201	0,4468644	0,8273207	transporter, putative
PFIT_05011	1509,046386	2633,78903	3478,210641	3088,217348	2774,215264	8083,559859	1711,12357	2152,534714	3594,5064	1931,8291	0,5374393	-0,895826	0,4472071	0,8273534	Plasmodium exported protein, unknown function
PFIT_14026	61,13166154	116,6908696	87,89026888	71,947716	59,40799619	183,7172695	68,44494282	54,99868432	96,79763	61,721814	0,6376377	-0,649191	0,4473315	0,8273534	hypothetical protein
PFIT_05087	3632,80559	2449,239883	3488,80734	3788,877413	2072,37196	3225,258732	2219,057093	2232,187981	3109,5602	2225,6225	0,7157355	-0,482502	0,4474493	0,8273534	Pre-mRNA-processing ATP-dependent RNA helicase prp5, putative
PFIT_10103	11,32067806	43,75907611	33,03676774	40,95485372	77,36855317	32,07761849	57,63784658	54,05043115	39,752925	55,844139	1,4047806	0,4903449	0,4475484	0,8273534	50S ribosomal protein L22, mitochondrial, putative
PFIT_10087	1423,009232	1180,226676	1584,518181	1844,075305	870,3962232	2029,638406	989,4496997	1087,646395	1488,644	1038,548	0,697647	-0,519431	0,4477975	0,8273534	small subunit rRNA synthesis-associated protein, putative
PFIT_10321	1187,539129	565,6970418	580,948444	539,60787	458,6849938	661,9653997	491,1224844	418,1796515	665,74048	454,65107	0,6829254	-0,5502	0,4478077	0,8273534	conserved Plasmodium protein, unknown function
PFIT_10337	11,32067806	5,07351607	1,870005721	2,213775877	1,381581307	2,916147135	6,003942352	8,534278602	4,129284	7,2691105	1,7603804	0,8158872	0,447906	0,8273534	conserved Plasmodium protein, unknown function
PFIT_13605	4,528271225	13,95216919	1,870005721	2,767219846	5,526325227	5,832294271	8,405519293	11,37903814	5,7460476	9,8922787	1,7215797	0,7837329	0,4482395	0,8275828	conserved Plasmodium protein, unknown function
PFIT_11238	424,5254274	211,8192959	250,5807666	230,2326912	179,6055699	189,5495638	174,1143282	169,7373189	247,71889	171,92582	0,694036	-0,526918	0,4487237	0,8275828	dynein heavy chain, putative
PFIT_04070	18,1130849	24,09920133	20,57006293	23,24464671	95,32910016	58,32294271	55,23626964	43,61964619	39,946508	49,427958	1,2373537	0,3072579	0,4487686	0,8275828	conserved Plasmodium protein, unknown function
PFIT_11419	0	9,512842632	4,986681922	2,213775877	17,96055699	5,832294271	9,606307764	11,37903814	6,7510253	10,492673	1,554234	0,6362037	0,4488076	0,8275828	conserved Plasmodium protein, unknown function
PFIT_01064	168,6781031	53,90610825	60,46351831	50,91684517	77,36855317	177,8849753	54,03548117	50,25741843	98,203017	52,14645	0,5310066	-0,913198	0,4491406	0,8275828	conserved Plasmodium protein, unknown function
PFIT_13668	83,77301766	8,878653123	6,856687643	11,06887938	38,68427659	34,99376562	40,826808	36,98187394	30,709213	38,904341	1,2668622	0,3412596	0,449171	0,8275828	secreted ookinete protein, putative
PFIT_13403	789,051261	2276,106147	201,3372826	1701,840205	33133,0829	30960,73414	1085,512777	1609,185643	11510,359	1347,3492	0,1170554	-3,094737	0,4491808	0,8275828	U6 spliceosomal RNA
PFIT_14267	616,9769544	442,6642771	350,314405	231,8930231	502,8955956	659,0492526	305,0002715	285,4242066	467,29892	295,21224	0,6317418	-0,662593	0,4492007	0,8275828	conserved Plasmodium protein, unknown function
PFIT_11249	639,6183105	1361,604875	1487,277883	1426,225109	1377,436563	796,108168	868,1700641	896,0992532	1181,3785	882,13466	0,7466994	-0,4214	0,4499202	0,8276658	endoplasmic reticulum oxidoreductin, putative
PF_apicopla	18,1130849	41,22231807	38,02344966	35,42041403	12,43423176	2,916147135	21,61419247	14,22379767	24,688274	17,918995	0,7258099	-0,462336	0,4499649	0,8276658	apicoplast ribosomal protein S8
PFIT_11108	297,7338331	118,5934381	107,2136613	101,8336903	89,80278493	58,32294271	69,64573129	83,44627967	128,91673	76,546005	0,5937632	-0,75204	0,4500225	0,8276658	conserved Plasmodium protein, unknown function
PFIT_13161	3807,144033	3115,138867	2940,895664	2948,749468	3020,136736	3677,261538	2249,076805	2219,86069	3251,5544	2234,4687	0,6872002	-0,541198	0,4500228	0,8276658	conserved Plasmodium protein, unknown function
PFIT_12369	2233,569782	2452,41083	2745,791733	2896,725735	1941,121736	2694,519953	1744,745648	1831,076887	2494,0233	1787,9113	0,7168783	-0,4802	0,4500625	0,8276658	conserved Plasmodium protein, unknown function
PFIT_06290	592,0714627	405,8812856	261,1774657	472,0877057	174,0792446	148,7235039	558,3666388	439,9894746	342,33678	499,17806	1,4581491	0,5441382	0,4501238	0,8276658	conserved Plasmodium protein, unknown function
PFIT_09103	1713,950659	2810,727903	3669,57456	3280,815849	3188,689656	3578,112535	2049,745919	2241,670513	3040,3119	2145,7082	0,7057527	-0,502765	0,4505837	0,8278203	Ran-binding protein, putative
PFIT_01037	75,84854302	136,3507444	133,3937414	159,9453071	261,118867	556,9841029	307,4018484	229,4772691	220,60688	268,43956	1,2168231	0,2831195	0,4509069	0,8278203	conserved Plasmodium protein, unknown function
PFIT_14242	761,8816336	470,5686155	585,9351259	488,1375808	397,8954163	1414,331361	411,8704454	395,4215752	686,45829	403,64601	0,5880124	-0,766081	0,4510125	0,8278203	peptidyl-prolyl cis-trans isomerase
PFIT_05308	413,2047493	413,4915597	589,0518021	523,0045509	924,2778941	1595,132483	856,1621794	851,5313539	743,02717	853,84677	1,1491461	0,2005622	0,4511604	0,8278203	conserved Plasmodium protein, unknown function
PFIT_05182	787,9191932	387,4897899	457,5280664	395,712438	536,053547	1288,937034	719,2722938	727,3101875	642,27334	723,29124	1,1261424	0,1713893	0,4512331	0,8278203	RAP protein, putative
PFIT_11184	13,58481368	26,00176986	21,81673341	25,45842258	34,53953267	17,49688281	39,62601953	30,3441017	23,149693	34,985061	1,5112538	0,595746	0,451261	0,8278203	conserved Plasmodium protein, unknown function
PFIT_08110	1382,254791	755,319705	885,1360412	836,8072814	781,9750196	787,3597266	670,0399665	596,451249	904,80876	633,24561	0,6998668	-0,514848	0,4513286	0,8278203	conserved Plasmodium protein, unknown function
PFIT_14035	3532,051556	3514,678258	5164,332466	4591,924612	4563,363056	7401,18143	3158,076677	3269,576958	4794,5886	3213,8253	0,6703026	-0,577116	0,4513787	0,8278203	40S ribosomal protein S31/UBI, putative
PFIT_06136	183,3949846	86,8839627	53,60683067	57,5581728	100,8554354	37,90991276	132,0867318	100,5148369	86,70155	116,30078	1,3413922	0,4237311	0,4516069	0,8279704	conserved Plasmodium protein, unknown function
PFIT_14259	0	10,14703214	12,46670481	19,92398289	96,71069147	40,8260599	9,606307764	4,74126589	30,012412	7,1737868	0,2390273	-2,064752	0,4518955	0,8280363	small nucleolar RNA snoR30
PFIT_14070	124,5274587	293,6297426	395,8178776	382,9832267	256,974123	443,2543646	247,3624249	204,8226865	316,1978	226,09256	0,7150352	-0,483914	0,4519357	0,8280363	RNA binding protein, putative
PFIT_11271	11916,14573	12194,83006	13186,03367	12232,21861	12728,50858	31535,21512	8912,252028	10397,5961	15632,159	9654,9241	0,6176322	-0,69518	0,4522475	0,8283375	40S ribosomal protein S18, putative
PFIT_07135	0	4,439326561	10,59669908	11,06887938	60,78957749	2,916147135	26,41734635	23,70632945	14,968438	25,061838	1,6743121	0,7435685	0,452393	0,8283375	secreted ookinete protein, putative
PFIT_13172	433,5819698	508,619986	701,2521453	771,5008931	558,1588479	402,4283047	870,5716411	740,585752	562,59036	805,57869	1,4319099	0,5179407	0,453149	0,8294275	conserved Plasmodium membrane protein, unknown function
PFIT_10381	67,92406838	20,29406428	16,20671625	22,13775877	15,19739437	20,41302995	44,42917341	31,29235487	27,028839	37,860764	1,4007544	0,486204	0,4536217	0,83005	phospholipase, putative
PFIT_08290	41374,81418	30014,92107	38095,75655	41738,53038	18587,7949	26315,31175	22571,22088	25624,64563	32687,855	24097,933	0,7372137	-0,439845	0,4538568	0,8302116	E3 ubiquitin-protein ligase, putative
PFIT_08070	7613,155997	4202,139685	4690,597683	4792,824773	3909,875098	7972,746268	3553,133084	3752,237825	5530,2233	3652,6855	0,6604951	-0,59838	0,454043	0,8302835	cactin homolog, putative
PFIT_07074	5464,491301	2213,955575	2330,027128	2591,778108	1416,120839	2744,094454	2038,938823	1831,076887	2793,412	1935,0079	0,6927041	-0,529689	0,4543344	0,8305355	heat shock protein 86 family protein
PFIT_13083	12119,91793	11771,82566	10988,15362	9439,540339	9997,122335	14536,99347	7443,687728	8191,959205	11475,592	7817,8235	0,6812566	-0,55373	0,4544745	0,8305355	60S ribosomal protein L24, putative
PFIT_05249	1,132067806	6,976084597	3,740011442	3,874107784	12,43423176	2,916147135	12,0078847	6,637772246	5,1787751	9,3228285	1,8001995	0,8481568	0,45605	0,8331457	iron-sulfur assembly protein, putative, putative
PFIT_09152	556,9773607	679,2169639	736,782254	637,5674525	783,3566009	1067,309852	462,3035611	528,1770202	743,53508	495,24029	0,6660618	-0,586272	0,4564311	0,8335726	ubiquitin conjugating enzyme, putative
PFIT_06059	31978,65139	8536,190788	9243,478278	9427,364571	5723,891353	12801,88592	6402,604124	7061,641417	12951,904	6732,1228	0,5197786	-0,944081	0,4570337	0,8342345	translation initiation factor IF-2, putative
PFIT_08286	7,924474644	12,04960067	0,62333524	1,660331908	12,43423176	2,916147135	10,80709623	9,48253178	6,2680202	10,144814	1,6185037	0,6946607	0,4570885	0,8342345	conserved Plasmodium protein, unknown function
PFIT_08117	22,64135613	98,29937386	101,6036442	75,26837981	29,01320744	55,40679557	50,43311576	44,56789937	63,705459	47,500508	0,745627	-0,423474	0,4571818	0,8345994	rifin, pseudogene
PFIT_14029	195,8477305	242,8945819	188,2472426	127,8455569	160,2634316	212,8787409	117,6772701	136,5484576	187,99621	127,11286	0,6761459	-0,564594	0,4576409	0,8346309	conserved Plasmodium protein, unknown function
PFIT_13056	1537,348081	1825,831596	1623,164966	1353,723949	2703,754617	3586,860977	2532,462884	2415,200844	2105,114	2473,8319	1,1751534	0,2328491	0,4577484	0,8346309	26S proteasome regulatory subunit, putative
PFIT_02193	272,8283413	1105,392314	1215,503719	1182,156318	656,2511207	414,0928932	603,9966006	678,9492755	807,7041	641,47294	0,794193	-0,332438	0,4579988	0,8348183	Plasmodium exported protein, unknown function
PFIT_06165	421,1292239	178,207252	203,2072883	158,2849752	67,69748403	425,7574818	134,4883087	142,2379767	242,38062	138,36314	0,5708507	-0,808815	0,458615	0,8356722	erythrocyte membrane protein 1, PfEMP1
PFIT_05184	606,7883442	987,4330652	873,2926716	731,0994833	1087,304488	1732,191398	1174,371124	1244,10817	1003,0182	1209,2396	1,2056009	0,2697523	0,4593028	0,8366559	proteasome subunit beta type 1, putative
PFIT_05317	14171,2248	5123,617041	5660,507317	5810,054789	4067,375367	7931,920208	4338,448744	4960,312478	7127,4499	4649,3806	0,6523203	-0,616347	0,4594643	0,8366806	Hsc70-interacting protein (HIP)
PFIT_02109	165,2818997	71,66341449	52,98349542	58,66506073	64,93432141	32,07761849	108,0709623	93,87706462	74,267635	100,97401	1,3595965	0,4431785	0,459647	0,8367439	GAF domain-related protein, putative
PFIT_02117	3659,975218	4961,264527	7102,281728	6789,650614	4444,547063	7873,597266	3930,180664	4319,293226	5805,2194	4124,7369	0,7105221	-0,493048	0,4599426	0,8368193	peptide chain release factor subunit 1, putative
PFIT_14482	5427,133063	7838,582329	8510,396036	7164,885625	6152,181559	15333,10164	5109,354942	5991,063579	8404,38	5550,2093	0,6603948	-0,598599	0,4600317	0,8368193	40S ribosomal protein S2, putative
PFIT_09115	816,2208883	783,2240433	993,596373	815,2229666	237,6319847	183,7172695	560,7682157	456,1097786	638,26892	508,439	0,7965906	-0,32809	0,4601779	0,8368193	cysteine repeat modular protein 1
PFIT_03221	8933,147059	8399,840044	9033,374302	8290,037215	9034,160164	15388,50843	6121,619622	6887,162832	9846,5112	6504,3912	0,6605783	-0,598199	0,4602802	0,8368193	peptidyl-prolyl cis-trans isomerase
PFIT_07175	6658,822837	1743,38696	1686,74516	1357,598056	1170,199367	1959,650875	1003,859161	1189,109485	2429,4005	1096,4843	0,4513395	-1,147715	0,4606216	0,8370437	actin-related protein, putative (ARP6)
PFIT_04168	2163,381578	3738,547154	3615,967729	4214,475825	2696,846711	2924,895577	2280,297305	2529,939479	3225,6858	2405,1184	0,7456146	-0,423498	0,4606996	0,8370437	golgi organization and biogenesis factor, putative
PFIT_12350	10113,89378	4287,121079	5525,866905	6652,949954	3317,176717	5581,505617	3969,806683	4366,705885	5913,0857	4168,2563	0,7049207	-0,504467	0,461044	0,8370489	CCR4-NOT transcription complex subunit 4, putative
PFIT_09186	9810,499609	4760,226453	3902,701939	4258,751343	2680,267735	1166,458854	7239,553688	5550,125851	4429,8177	6394,8398	1,4435898	0,5296609	0,4611244	0,8370489	telomerase RNA
PFIT_09179	1240,746316	720,439282	1055,929897	1293,952	780,5934383	976,9092904	713,2683515	737,7409725	1011,4284	725,50466	0,717307	-0,479337	0,4611465	0,8370489	conserved Plasmodium protein, unknown function
PFIT_11273	3604,503895	569,5021789	721,8222083	664,686207	479,4087134	548,2356615	363,8389065	345,1641568	1098,0265	354,50153	0,3228534	-1,631049	0,4614704	0,8373377	large ribosomal subunit processing factor, putative
PFIT_10072	321,507257	671,6066898	827,1658639	1056,524537	580,2641488	597,8101628	482,7169651	539,5560583	675,81311	511,13651	0,7563282	-0,402916	0,4616016	0,8373377	conserved Plasmodium protein, unknown function
PFIT_02085	219,6211544	568,2337999	640,1652918	522,4511069	447,6323434	495,745013	321,8113101	381,1977776	482,30812	351,50454	0,7287967	-0,456412	0,4619015	0,8373987	3'exoribonuclease, putative
PFIT_13579	1854,327067	5693,11922	5687,310732	4757,404359	4097,770156	3779,326687	2755,80954	3650,774735	4311,543	3203,2921	0,7429572	-0,428649	0,4619314	0,8373987	40S ribosomal protein S13, putative
PFIT_12093	115,4709162	86,8839627	95,37029176	84,12348332	163,0265942	169,1365339	152,5001357	137,4967108	119,00196	144,99842	1,218454	0,2850518	0,4625745	0,8378546	conserved Plasmodium protein, unknown function
PFIT_12234	1156,973298	1801,098205	2058,252963	1666,419791	1920,398016	1831,340401	1168,367182	1303,84812	1739,0804	1236,1077	0,7107823	-0,49252	0,4626468	0,8378546	integral membrane protein, putative
PFIT_14520	43,01857664	88,78653123	120,9270366	89,10447904	77,36855317	84,56826693	68,44494282	52,15392479	83,962241	60,299434	0,7181732	-0,477596	0,4627848	0,8378546	conserved Plasmodium protein, unknown function
PFIT_11146	99,62196695	157,2789982	135,8870824	110,1353499	349,5400706	192,4657109	246,1616364	185,8576229	174,15486	216,00963	1,2403307	0,3107249	0,4630926	0,8378546	conserved Plasmodium membrane protein, unknown function
PFIT_11092	3501,485725	2888,733212	3515,610755	3640,554429	2452,306819	2758,67519	2209,450786	2351,667881	3126,2277	2280,5593	0,7294924	-0,455035	0,4631036	0,8378546	casein kinase 2, alpha subunit
PFIT_07292	18008,93466	21722,25905	15769,13491	15598,81827	12634,56105	13484,26435	21198,71966	22651,87192	16202,995	21925,296	1,3531631	0,4363358	0,4631756	0,8378546	transcription factor with AP2 domain(s) (AP2-L)
PFIT_10210	847,9187869	932,2585779	1088,34333	1034,940222	1055,528118	1420,163655	686,8510051	768,0850742	1063,1921	727,46804	0,6842301	-0,547447	0,4632198	0,8378546	RNA helicase, putative
PFIT_06287	3635,069726	1746,557907	2151,129914	2135,740277	1796,055699	2426,234417	1612,658916	1585,479314	2315,1313	1599,0691	0,6907034	-0,533862	0,4634331	0,8379724	cullin-like protein, putative
PFIT_08046	39380,11071	9758,908161	11304,80792	12506,72682	7239,486047	9162,534299	7112,270111	7886,621682	14892,096	7499,4459	0,5035857	-0,989691	0,4638011	0,8383697	mannose-6-phosphate isomerase, putative
PFIT_12237	139,2443402	105,2754585	117,8103604	89,10447904	385,4611846	778,6112852	79,25203905	71,11898835	269,25118	75,185514	0,2792393	-1,840426	0,4640674	0,8384646	GTP cyclohydrolase I
PFIT_08050	115,4709162	168,6944093	195,7272655	161,605639	542,9614535	239,1240651	361,4373296	244,6493199	237,26396	303,04332	1,2772413	0,3530311	0,4641501	0,8384646	1-cys peroxiredoxin
PFIT_14647	26,03755954	7,610274105	13,09004005	8,301659538	12,43423176	2,916147135	1,20078847	9,48253178	11,731652	5,3416601	0,4553204	-1,135046	0,4644652	0,8385929	conserved Plasmodium protein, unknown function
PFIT_14754	374,7144439	289,8246055	357,1710927	312,6958426	197,5661269	224,5433294	219,7442901	209,5639523	292,75257	214,65412	0,7332271	-0,447668	0,4649337	0,8385929	DNA repair metallo-beta-lactamase protein, putative
PFIT_10115	4950,532517	1823,294838	1487,901219	1903,847254	1579,147433	1589,300189	3098,034254	2685,453	2222,3372	2891,7436	1,3012173	0,3798619	0,4650428	0,8385929	ubiquitin-protein ligase e3, putative
PFIT_06273	13,58481368	39,95393905	61,71018879	86,89070316	23,48688221	14,58073568	25,21655788	29,39584852	40,034544	27,306203	0,6820661	-0,552017	0,4650743	0,8385929	conserved Plasmodium membrane protein, unknown function
PFIT_09003	13,58481368	15,22054821	14,33671053	4,980995723	6,907906533	17,49688281	4,803153882	6,637772246	12,087976	5,7204631	0,4732358	-1,079369	0,4653192	0,8385929	erythrocyte membrane protein 1 (PfEMP1), exon2, pseudogene
PFIT_11066	2541,492225	1777,633193	2103,133101	2178,908907	1779,476723	2758,67519	2883,093118	2722,434874	2189,8866	2802,764	1,2798672	0,3559941	0,465451	0,8385929	conserved Plasmodium protein, unknown function
PFIT_04157	14,71688148	65,95570891	7,480022884	26,01186655	20,7237196	2,916147135	31,22050023	47,4126589	22,967391	39,31658	1,7118435	0,7755508	0,4654696	0,8385929	mRNA-binding protein PUF2 (PUF2)
PFIT_13170	7179,574027	6675,478769	8194,988404	7287,750186	7336,196738	17762,2522	5384,335502	6176,921202	9072,7067	5780,6284	0,6371448	-0,650307	0,4655245	0,8385929	40S ribosomal protein S15/S19, putative
PFIT_14094	678,108616	1177,689918	1104,550046	1122,38437	1253,094245	1224,781797	1529,804511	1368,329336	1093,4348	1449,0669	1,3252431	0,406257	0,4655555	0,8385929	DNA-repair helicase, putative
PFIT_01013	132,4519333	71,02922498	43,63346682	51,47028914	89,80278493	29,16147135	55,23626964	37,93012712	69,591528	46,583198	0,6693803	-0,579102	0,4657999	0,8387661	conserved Plasmodium protein, unknown function
PFIT_11104	31,69789858	40,58812856	31,79009725	34,31352609	35,92111397	20,41302995	19,21261553	22,75807627	32,453966	20,985346	0,6466188	-0,629013	0,4663211	0,8394373	small nucleolar RNA snoR11
PFIT_13271	3,396203419	1,902568526	1,246670481	0,553443969	4,14474392	2,916147135	1,20078847	6,637772246	2,3599629	3,9192804	1,6607381	0,7318246	0,4666051	0,8396813	conserved Plasmodium protein, unknown function
PFIT_06103	496,977767	749,6119994	586,5584611	701,213509	675,593259	659,0492526	457,5004072	466,5405636	644,83404	462,02049	0,7164952	-0,480971	0,4668741	0,8398982	SWI/SNF-related matrix-associated actin-dependent regulator of chromatin
PFIT_08065	74,71647521	130,6430388	162,0671625	143,895432	442,1060181	376,1829805	290,5908099	236,1150413	221,60185	263,35293	1,1884058	0,2490276	0,467146	0,8401201	tubulin gamma chain
PFIT_08009	20,37722051	10,78122165	6,856687643	8,301659538	15,19739437	2,916147135	16,81103859	16,12030403	10,738388	16,465671	1,5333466	0,6166838	0,467686	0,8408239	Plasmodium exported protein (PHISTa), unknown function
PFIT_11158	0	19,65987477	18,07672197	16,60331908	48,35534573	8,748441406	33,62207717	22,75807627	18,57395	28,190077	1,5177211	0,6019067	0,4682008	0,8411105	conserved Plasmodium protein, unknown function
PFIT_07228	716,5989214	360,8538305	412,6479291	345,9024807	737,7644178	341,1892148	666,4376011	560,4176282	485,82613	613,42761	1,2626485	0,336453	0,4682551	0,8411105	protein kinase, putative
PFIT_14472	13,58481368	72,297604	47,37347826	26,56531052	67,69748403	84,56826693	26,41734635	36,03362076	52,014944	31,225484	0,6003228	-0,73619	0,4682915	0,8411105	U5 spliceosomal RNA
PFIT_07088	969,0500422	273,9698678	216,9206636	287,23742	317,7637005	128,310474	560,7682157	473,1783358	365,54203	516,97328	1,4142649	0,5000524	0,4684906	0,8412008	conserved Plasmodium membrane protein, unknown function
PFIT_09337	141,5084758	58,34543481	54,85350115	40,95485372	153,355525	236,207918	128,4843663	118,5316473	114,20428	123,50801	1,0814656	0,1129878	0,4686436	0,8412086	calcyclin binding protein, putative
PFIT_05068	8532,395056	2907,124708	3160,933004	3383,202984	2949,67609	6263,884047	2832,660002	2913,982016	4532,8693	2873,321	0,6338857	-0,657705	0,4689192	0,8414362	transcription factor 25, putative (TCF25)
PFIT_06033	38,49030541	26,63595937	18,70005721	24,90497861	22,10530091	14,58073568	12,0078847	17,0685572	24,236223	14,538221	0,5998551	-0,737314	0,4694194	0,8415157	conserved Plasmodium protein, unknown function
PFIT_07251	124,5274587	88,15234172	77,91690504	65,85983233	37,30269528	96,23285547	104,4685969	110,9456218	81,665348	107,70711	1,3188839	0,3993175	0,4694963	0,8415157	serine/threonine protein kinase, FIKK family
PFIT_10060	95,09369573	81,81044663	89,7602746	76,37526775	142,3028746	122,4781797	134,4883087	120,4281536	101,30346	127,45823	1,2581825	0,3313411	0,4695537	0,8415157	conserved Plasmodium protein, unknown function
PFIT_07179	4733,175498	3997,930663	5644,923936	4511,675237	4893,560988	12174,91429	3652,798527	3784,478433	5992,6968	3718,6385	0,6205284	-0,688431	0,4695586	0,8415157	40S ribosomal protein S10, putative
PFIT_09333	767,5419727	1713,580053	1631,891659	1533,039795	1537,699994	1259,775562	1035,079662	1051,612774	1407,2548	1043,3462	0,7414053	-0,431666	0,4697913	0,841666	conserved Plasmodium protein, unknown function
PFIT_09002	122,2633231	127,4720913	290,474222	209,2018204	103,618598	530,7387786	84,05519293	151,7205085	230,62814	117,88785	0,5111599	-0,968154	0,4704648	0,8426058	rifin (RIF)
PFIT_06179	1674,328285	1533,470232	1558,961436	1450,023199	1445,134047	2038,386848	1113,130912	1126,524775	1616,7173	1119,8278	0,6926553	-0,529791	0,4706971	0,8426137	conserved Plasmodium protein, unknown function
PFIT_10154	49932,11473	36068,89412	24596,80858	18115,328	27896,88974	56211,65218	20651,16011	24519,93068	35470,281	22585,545	0,6367456	-0,651211	0,4707672	0,8426137	enolase
PFIT_09079	3871,671897	3917,388596	4347,763301	3983,68969	4278,757307	6759,62906	2970,750676	3150,097057	4526,4833	3060,4239	0,6761151	-0,564659	0,4711301	0,8427567	subunit of proteaseome activator complex, putative
PFIT_04051	2073,948221	1225,88832	1666,798433	1877,281944	762,6328813	1041,064527	1900,848149	2063,398915	1441,2691	1982,1235	1,3752627	0,4597072	0,4712303	0,8427567	conserved Plasmodium protein, unknown function
PFIT_11064	9100,693095	16428,04504	18702,55055	19729,72406	14932,13076	22366,84853	11391,88022	12761,59127	16876,665	12076,736	0,7155878	-0,482799	0,4712941	0,8427567	translocon component PTEX88
PFIT_bin08	31,69789858	10,78122165	8,103358124	9,408547476	9,671069147	64,15523698	7,204730823	5,689519068	22,302889	6,4471249	0,2890713	-1,790503	0,4721099	0,838919	rifin
PFIT_12149	5,660339031	54,54029776	48,62014874	43,1686296	105,0001793	209,9625937	26,41734635	28,44759534	77,825365	27,432471	0,3524875	-1,504356	0,4722274	0,838919	10 kDa chaperonin
PFIT_11371	5009,400043	3748,694186	5209,212603	6405,560499	3348,953087	4520,02806	3710,436374	3319,834376	4706,9747	3515,1354	0,7467929	-0,42122	0,4730746	0,8447469	conserved Plasmodium protein, unknown function
PFIT_13019	5518,830556	4037,884602	4342,153284	4244,3618	2705,136198	3548,951064	2781,026098	3128,287234	4066,2196	2954,6567	0,7266348	-0,460698	0,4731413	0,8447469	DEAD/DEAH box ATP-dependent RNA helicase, putative
PFIT_07100	473,204343	870,7421956	1035,359834	1224,21806	714,2775355	367,4345391	623,2092162	619,2093252	780,87275	621,20927	0,795532	-0,330008	0,4734328	0,8447469	conserved Plasmodium membrane protein, unknown function
PFIT_06238	13,58481368	13,31797968	8,726693364	9,961991446	15,19739437	8,748441406	16,81103859	18,01681038	11,589552	17,413924	1,5025537	0,5874165	0,4735135	0,8447469	conserved Plasmodium protein, unknown function
PFIT_13466	159,6215607	145,2293975	157,7038158	182,0830659	129,8686428	116,6458854	105,6693854	109,0491155	148,52539	107,35925	0,7228343	-0,468263	0,4736897	0,8447469	conserved Plasmodium protein, unknown function
PFIT_01124	49,81098348	11,41541116	14,96004577	19,92398289	6,907906533	2,916147135	24,01576941	30,3441017	17,655746	27,179936	1,5394385	0,6224042	0,4737066	0,8447469	RESA-like protein with DnaJ domain, putative
PFIT_14667	49,81098348	132,5456073	74,80022884	47,59618135	78,75013448	37,90991276	86,45676987	111,893875	70,235508	99,175322	1,4120397	0,4977806	0,4739848	0,8447469	conserved Plasmodium protein, unknown function
PFIT_12027	724,523396	301,8742062	374,0011442	395,712438	563,6851731	530,7387786	630,413947	550,9350964	481,75586	590,67452	1,2260869	0,2940612	0,474098	0,8447469	origin recognition complex subunit 1
PFIT_04099	46,41478006	53,27191874	44,25680206	44,27551754	138,1581307	169,1365339	92,46071223	91,98055827	82,585614	92,220635	1,1166671	0,1591991	0,474103	0,8447469	conserved Plasmodium protein, unknown function
PFIT_13326	388,2992576	958,8945373	982,3763487	641,4415603	1248,949501	2405,821387	572,7761004	623,9505911	1104,2971	598,36335	0,54185	-0,884035	0,4745561	0,8447469	transmembrane protein Tmp21 homologue, putative
PFIT_01011	269,4321379	104,0070794	305,4342677	379,6625629	150,5923624	1230,614091	115,2756932	143,1862299	406,62375	129,23096	0,3178146	-1,653743	0,4746462	0,8447469	serine/threonine protein kinase, FIKK family
PFIT_12502	7539,57159	878,3524697	1064,65659	1272,367685	1050,001793	2015,057671	728,8786016	696,0178327	2303,3346	712,44822	0,3093116	-1,692867	0,4747226	0,8447469	rRNA processing protein, putative
PFIT_09223	8243,717765	13304,6617	12239,18744	12158,05712	20084,04745	14201,63655	16865,07407	17022,0928	13371,885	16943,583	1,2671051	0,3415362	0,4748332	0,8447469	S-adenosylmethionine synthetase
PFIT_01106	56,60339031	157,9131877	88,51360412	201,4536048	361,9743023	32,07761849	85,2559814	79,65326695	149,75595	82,454624	0,5505933	-0,860941	0,4748538	0,8447469	conserved Plasmodium protein, unknown function
PFIT_12236	21,50928832	35,51461249	39,89345538	37,63418991	142,3028746	104,9812969	84,05519293	68,27422882	63,639286	76,164711	1,1968191	0,2592051	0,4750339	0,8447469	50S ribosomal protein L24, putative,mitochondrial ribosomal protein L24-2 precursor, putative
PFIT_01030	249,0549174	430,6146765	414,5179348	316,0165064	389,6059285	548,2356615	259,3703096	277,8381812	391,34094	268,60425	0,6863689	-0,542944	0,4750955	0,8447469	conserved protein, unknown function
PFIT_11256	2007,156221	348,8042298	370,2611327	358,631692	368,8822089	947,747819	228,1498094	348,0089163	733,58055	288,07936	0,3927031	-1,348489	0,4753546	0,844942	GrpE protein homolog, mitochondrial, putative (MGE1)
PFIT_10171	3352,052774	993,1407707	1146,313507	1476,58851	690,7906533	1437,660538	883,7803143	985,235052	1516,0911	934,50768	0,6163928	-0,698078	0,4758358	0,845491	conserved Plasmodium protein, unknown function
PFIT_05110	32,82996638	272,0672993	121,5503719	157,1780873	2265,793343	7144,560482	93,6615007	93,87706462	1665,6633	93,769283	0,0562955	-4,150838	0,4760282	0,845491	small nucleolar RNA snoR04
PFIT_13270	927,1635333	2224,102607	2101,263095	2265,79961	1533,55525	1851,753431	1191,182163	1511,515566	1817,2729	1351,3489	0,7436136	-0,427375	0,4761144	0,845491	ribose-phosphate pyrophosphokinase, putative
PFIT_09095	735,8440741	1293,112408	1676,148461	1916,576465	862,1067353	1767,185164	1049,489123	1006,096622	1375,1622	1027,7929	0,7473975	-0,420052	0,4766847	0,845491	apoptosis-related protein, putative
PFIT_13304	1927,911474	2613,494966	2444,720812	2477,76865	2573,885974	3776,41054	3379,018756	3255,35316	2635,6987	3317,186	1,2585604	0,3317744	0,4767087	0,845491	DEAD box helicase, putative
PFIT_03154	40,75444103	36,148802	47,9968135	34,86697006	145,0660372	87,48441406	76,85046211	75,86025424	65,386246	76,355358	1,1677587	0,2237422	0,4767518	0,845491	conserved Plasmodium protein, unknown function
PFIT_14025	11742,93935	6846,709937	6508,866579	4602,440048	7696,789459	9089,630621	9416,583185	9314,690968	7747,896	9365,6371	1,2087975	0,2735725	0,4768973	0,845491	Plasmodium exported protein (PHISTb), unknown function
PFIT_12221	1102,634043	410,3206122	567,2350687	732,7598152	617,5668441	577,3971328	960,6307764	772,8263401	667,98559	866,72856	1,2975258	0,3757633	0,4769806	0,845491	transcription factor with AP2 domain(s)
PFIT_08265	209,4325442	217,5270015	153,9638044	152,1970915	107,7633419	285,7824193	106,8701739	141,2897235	187,7777	124,07995	0,6607811	-0,597756	0,4770088	0,845491	lipoate-protein ligase (LipB)
PFIT_14744	275,0924769	372,9034312	256,1907838	123,4180051	218,2898465	215,794888	172,9135397	172,5820784	243,61491	172,74781	0,709102	-0,495935	0,4774476	0,8458052	conserved Plasmodium protein, unknown function
PFIT_03113	434,7140376	306,9477222	236,2440561	346,4559247	254,2109604	169,1365339	375,8467913	411,5418793	291,28487	393,69434	1,3515784	0,4346452	0,4774852	0,8458052	protein kinase, putative
PFIT_13231	1623,385234	1091,440145	1558,338101	1617,163278	848,2909223	1495,98348	1038,682027	967,2182416	1372,4335	1002,9501	0,7307823	-0,452486	0,4779705	0,8463999	conserved Plasmodium protein, unknown function
PFIT_14443	603,3921407	407,1496646	433,217992	308,2682908	627,2379132	1116,884353	648,425774	645,7604142	582,69173	647,09309	1,1105239	0,1512405	0,4784094	0,8467979	conserved Plasmodium protein, unknown function
PFIT_14112	2542,624293	4203,408064	4817,134737	4756,850915	3057,439432	4974,947013	2787,03004	3150,097057	4058,741	2968,5635	0,7314013	-0,451265	0,4786936	0,8467979	cytidine triphosphate synthetase
PFIT_14158	973,5783134	1551,861728	1909,899176	2310,625171	1476,910417	1907,160227	1192,382951	1301,951613	1688,3397	1247,1673	0,7386945	-0,43695	0,4787012	0,8467979	small nuclear ribonucleoprotein-associated protein B, putative
PFIT_02108	19,24515271	23,46501182	19,94672769	19,92398289	51,11850835	20,41302995	39,62601953	30,3441017	25,685402	34,985061	1,3620601	0,4457903	0,4787941	0,8467979	beta-ketoacyl-ACP synthase III
PFIT_10295	6114,298222	3398,621578	4641,977534	5072,313978	3172,11068	5161,58043	3198,900485	3332,161668	4593,4837	3265,5311	0,7109051	-0,492271	0,4790395	0,8469671	pre-mRNA-splicing factor ATP-dependent RNA helicase PRP22, putative
PFIT_06250	168,6781031	114,1541116	390,2078604	314,9096185	284,6057492	1239,362533	150,0985588	135,6002045	418,653	142,84938	0,3412119	-1,55126	0,4793855	0,8470051	oxidoreductase, short-chain dehydrogenase family, putative
PFIT_13012	46598,17504	9405,030415	11133,39073	11316,26884	6946,59081	10171,52121	6551,501895	7143,19119	15928,496	6847,3465	0,4298803	-1,217993	0,4793973	0,8470051	Plasmodium exported protein, unknown function
PFIT_06051	664,5238023	563,1602838	499,2915275	535,1803182	788,8829261	463,6673945	750,492794	753,8612765	585,78438	752,17704	1,284051	0,3607025	0,4795103	0,8470051	ubiquitin conjugating enzyme E2, putative
PFIT_13659	7585,98637	5035,4647	7178,951962	6392,277844	3033,952549	7937,752503	4439,314975	4433,083607	6194,0643	4436,1993	0,7162017	-0,481562	0,4798336	0,8471431	conserved Plasmodium protein, unknown function
PFIT_03073	70,18820399	52,00353972	64,82686499	75,82182378	262,5004483	137,0589154	157,3032896	111,893875	110,39997	134,59858	1,2191904	0,2859235	0,479888	0,8471431	ATP-dependent Clp protease proteolytic subunit
PFIT_04024	8110,133764	2237,420587	1846,318982	2106,961159	1258,62057	2642,029305	1236,812125	1669,873846	3033,5807	1453,343	0,479085	-1,061646	0,4801652	0,8473679	CGI-201 protein, short form
PFIT_02077	10417,28795	2814,53304	2938,402323	2492,158193	2337,635571	3528,538034	2261,08469	2049,175118	4088,0925	2155,1299	0,5271725	-0,923653	0,4805429	0,84777	conserved Plasmodium protein, unknown function
PFIT_10143	173,2063744	42,49069709	36,77677918	30,99286228	66,31590272	29,16147135	94,86228917	77,7567606	63,157348	86,309525	1,3665793	0,4505692	0,480791	0,8479433	conserved Plasmodium protein, unknown function
PFIT_13613	305,6583077	452,1771198	733,0422426	620,9641334	515,3298274	1192,704178	421,4767531	435,2482087	636,64597	428,36248	0,6728425	-0,571659	0,4811915	0,848286	conserved protein, unknown function
PFIT_12171	2135,079883	992,5065812	1319,600704	1430,099216	799,9355765	1181,0359	1784,371667	1656,598302	1309,7103	1720,485	1,3136379	0,3935676	0,4814157	0,848286	conserved Plasmodium protein, unknown function
PFIT_05375	23,77342393	56,44286628	71,06021739	51,47028914	17,96055699	96,23285547	36,02365411	34,13711441	52,823368	35,080384	0,6641073	-0,590512	0,4814697	0,848286	rifin
PFIT_10157	799,2398712	945,5765576	1004,193072	1036,04711	830,3303653	670,7138411	601,5950237	709,2933772	881,0168	655,4442	0,7439633	-0,426697	0,481717	0,848286	conserved Plasmodium protein, unknown function
PFIT_11324	448,2988513	204,8432113	182,6372254	178,7624021	99,47385408	90,4005612	122,480424	136,5484576	200,73602	129,51444	0,6451978	-0,632187	0,4817352	0,848286	conserved Plasmodium protein, unknown function
PFIT_08212	113,2067806	107,8122165	69,19021167	111,2422378	82,8948784	96,23285547	130,8859433	128,014179	96,763197	129,45006	1,3378027	0,4198653	0,4825392	0,849016	dynactin subunit 6, putative
PFIT_14437	269,4321379	182,012389	223,7773513	154,9643114	198,9477082	524,9064844	164,5080205	151,7205085	259,00673	158,11426	0,6104639	-0,712022	0,4827202	0,849016	protein prenyltransferase alpha subunit, putative
PFIT_14359	500,3739704	268,2621622	225,647357	219,1638118	285,9873305	889,4248763	177,7166936	222,8394968	398,14325	200,2781	0,5030302	-0,991283	0,4830557	0,849016	mitochondrial import inner membrane translocase subunit TIM17, putative (TIM17)
PFIT_01105	156,2253573	268,2621622	333,4843536	287,790864	320,5268531	221,6271823	371,0436374	349,9054227	264,6528	360,47453	1,3620658	0,4457964	0,4831107	0,849016	TatD-like deoxyribonuclease, putative
PFIT_01007	59481,10668	73167,07782	57795,64348	80631,25187	32420,18694	19584,84416	72759,37579	82876,37951	53846,685	77817,878	1,4451749	0,5312441	0,4836	0,849016	ring-infected erythrocyte surface antigen
PFIT_13186	1460,36747	665,8989842	1001,699731	1301,146772	1545,989482	758,1982552	1636,674685	1295,313841	1122,2168	1465,9943	1,3063379	0,3855281	0,4838105	0,849016	conserved Plasmodium protein, unknown function
PFIT_10146	407,5444103	589,7962432	706,8621625	806,9213071	595,4615432	688,210724	480,3153882	455,1615254	632,46607	467,73846	0,7395471	-0,435286	0,4840827	0,849016	conserved protein, unknown function
PFIT_11347	4197,707426	3007,96084	3385,957025	2798,766152	2619,478157	5747,726004	2335,533575	2517,612188	3626,2659	2426,5729	0,6691657	-0,579565	0,4841678	0,849016	heat shock protein 70
PFIT_06125	418,8650883	249,8706665	213,1806522	280,5960924	367,5006276	536,5710729	444,2917341	369,8187394	344,4307	407,05524	1,1818204	0,2410108	0,4843539	0,849016	adapter-related protein, putative
PFIT_10028	57,73545812	31,07528593	24,93340961	21,03087083	38,68427659	64,15523698	52,8346927	42,67139301	39,602423	47,753043	1,2058111	0,270004	0,4847344	0,849016	conserved Plasmodium protein, unknown function
PFIT_11170	354,3372234	499,7413329	477,4747941	371,3609033	321,9084444	355,7699505	254,5671557	325,2508401	396,76544	289,909	0,7306811	-0,452686	0,4850207	0,849016	SNO glutamine amidotransferase family protein (PDX2)
PF_apicopla	74,71647521	19,02568526	19,32339245	15,49643114	13,81581307	17,49688281	14,40946165	10,43078496	26,64578	12,420123	0,4661197	-1,101228	0,4850521	0,849016	hypothetical protein
PFIT_06068	835,466041	687,4614275	237,4907266	642,5484482	397,8954163	148,7235039	739,6856978	636,2778824	491,59759	687,98179	1,3994816	0,4848925	0,4851377	0,849016	conserved Plasmodium protein, unknown function
PFIT_bin04	3,396203419	12,04960067	14,96004577	17,15676305	1,381581307	14,58073568	2,401576941	8,534278602	10,587488	5,4679278	0,5164518	-0,953294	0,4853027	0,849016	rifin (RIF)
PFIT_14033	1222,633231	637,3604563	399,557889	349,7765885	693,5538159	661,9653997	417,8743877	451,3685127	660,8079	434,62145	0,6577123	-0,604472	0,4853727	0,849016	U1 small nuclear ribonucleoprotein C, putative
PFIT_03183	3460,731284	2766,968827	3082,392763	2910,00839	2383,227754	3169,851936	2018,525419	2219,86069	2962,1968	2119,1931	0,7154126	-0,483152	0,4854115	0,849016	conserved Plasmodium protein, unknown function
PFIT_12174	6847,87816	2875,415233	4118,999268	4847,062282	1273,817965	1113,968206	3057,207446	2025,468788	3512,8569	2541,3381	0,7234391	-0,467056	0,4854842	0,849016	conserved Plasmodium protein, unknown function
PFIT_13450	2242,626324	1149,785579	1168,13024	1031,619559	1062,436025	1420,163655	893,386622	956,7874566	1345,7936	925,08704	0,6873915	-0,540796	0,4855844	0,849016	conserved Plasmodium protein, unknown function
PFIT_08322	2358,09724	302,5083957	239,9840675	238,5343507	259,7372857	209,9625937	118,8780586	110,9456218	601,47066	114,91184	0,1910514	-2,387967	0,4856475	0,849016	U6 snRNA-associated Sm-like protein LSm8, putative
PFIT_02155	2471,304021	835,8617726	873,9160069	780,9094405	516,7114087	501,5773073	652,0281395	519,6427416	996,71333	585,83544	0,5877672	-0,766683	0,485908	0,849016	conserved Plasmodium protein, unknown function
PFIT_12164	356,601359	910,6961346	1519,067981	1563,479213	273,5530987	997,3223203	674,8431204	732,9997066	936,78668	703,92141	0,7514212	-0,412306	0,4860766	0,849016	perforin like protein 2
PFIT_05038	3280,732503	4535,723367	5159,969139	5209,568082	4133,691269	7051,243773	3443,861333	3507,588505	4895,1547	3475,7249	0,7100337	-0,494041	0,4861039	0,849016	60S ribosomal protein L31, putative
PFIT_10269	24,90549174	76,73693056	114,070349	106,2612421	77,36855317	174,9688281	67,24415435	61,63645657	95,718566	64,440305	0,6732268	-0,570835	0,4865016	0,849016	conserved Plasmodium protein, unknown function
PFIT_14185	1385,650995	2510,756265	2553,804479	2465,592883	1809,819041	2685,771512	1504,587953	1777,974709	2250,8992	1641,2813	0,729167	-0,455679	0,4866276	0,849016	pseudouridine synthase, putative
PFIT_10007	89,4333567	615,798013	287,3575458	341,474929	331,5795136	55,40679557	172,9135397	219,0464841	286,84169	195,98001	0,6832341	-0,549548	0,4866513	0,849016	acyl-CoA binding protein, isoform 1, ACBP1
PFIT_13649	23662,48129	7600,761263	6029,521779	5846,028647	8130,60599	14898,59571	5889,867448	6390,278167	11027,999	6140,0728	0,5567712	-0,844843	0,4867287	0,849016	60S ribosomal protein L40/UBI, putative
PFIT_07259	408,6764781	280,3117629	325,3809954	297,1994115	310,855794	562,8163971	446,693311	433,3517024	364,20681	440,02251	1,2081666	0,2728194	0,4868243	0,849016	vacuolar protein sorting-associated protein 53, putative (VPS53)
PFIT_02151	7957,30461	8364,959621	10003,90727	10770,01964	6179,811185	10687,67925	6096,403065	7033,193821	8993,9473	6564,7984	0,7299129	-0,454204	0,487174	0,849016	DNA-directed RNA polymerase II second largest subunit, putative
PFIT_09368	271,6962735	178,207252	504,2782094	444,9689512	453,1586686	2755,759043	139,2914626	159,3065339	768,0114	149,299	0,1943969	-2,362923	0,4871834	0,849016	Plasmodium exported protein (hyp11), unknown function
PFIT_11210	233,2059681	195,3303687	147,1071167	172,6745184	294,2768183	96,23285547	237,7561172	255,0801049	189,80461	246,41811	1,2982725	0,3765933	0,4874545	0,849016	conserved Plasmodium protein, unknown function
PFIT_05346	100,7540348	49,49423681	109,0836671	125,078337	63,55274011	107,897444	151,2993473	125,1694195	100,14341	138,23438	1,3803643	0,465049	0,4876754	0,849016	conserved Plasmodium protein, unknown function
PFIT_11423	7,924474644	32,34366495	54,85350115	36,52730197	160,2634316	192,4657109	111,6733278	78,70501378	80,729681	95,189171	1,17911	0,2376983	0,4876838	0,849016	dolichol phosphate mannose synthase
PFIT_13347	19,24515271	8,878653123	16,83005149	17,15676305	5,526325227	2,916147135	8,405519293	5,689519068	11,758849	7,0475192	0,5993375	-0,738559	0,487736	0,849016	conserved Plasmodium protein, unknown function
PFIT_13205	5062,60723	4349,905841	3624,694422	3709,181482	2531,056954	2344,582297	2678,959078	2750,882469	3603,6714	2714,9208	0,753763	-0,408557	0,4878536	0,849016	conserved Plasmodium membrane protein, unknown function
PFIT_14680	2018,476899	2465,094621	3087,379445	3164,592616	2459,214726	3718,087598	2185,435016	1905,988888	2818,8077	2045,712	0,7257366	-0,462482	0,4878742	0,849016	rhoptry protein, putative
PFIT_bin00	296,6017652	340,5597662	654,5020023	665,7930949	270,7899361	1761,35287	775,7093519	885,6684683	664,93324	830,68891	1,2492817	0,3210988	0,4878995	0,849016	rifin (RIF)
PFIT_14409	27,16962735	86,24977319	129,65373	113,4560137	287,3689118	142,8912096	180,1182706	152,6687617	131,13154	166,39352	1,2689053	0,3435844	0,4880247	0,849016	leucine carboxyl methyltransferase, putative
PFIT_13258	3889,784982	1875,298577	2656,654794	2155,66426	2805,991634	1157,710413	1777,166936	1758,061392	2423,5174	1767,6142	0,729359	-0,455299	0,4881264	0,849016	conserved Plasmodium protein, unknown function
PFIT_11508	18,1130849	36,78299151	91,63028032	84,12348332	31,77637005	183,7172695	33,62207717	37,93012712	74,357247	35,776102	0,4811381	-1,055477	0,4881658	0,849016	rifin (RIF)
PFIT_14550	1656,215201	2867,804959	3200,203124	3394,271863	2427,438356	1825,508107	1796,379552	2143,052182	2561,9069	1969,7159	0,7688475	-0,379231	0,4883615	0,849016	glucose-6-phosphate dehydrogenase-6-phosphogluconolactonase
PFIT_14734	901,1259738	1571,521603	1916,755864	1984,650074	1550,134226	1773,017458	2286,301248	2057,709396	1616,2009	2172,0053	1,3438957	0,4264211	0,4885593	0,849016	conserved Plasmodium membrane protein, unknown function
PFIT_14262	329,4317316	97,03099484	87,89026888	64,19950043	122,9607363	131,2266211	64,8425774	83,44627967	138,78998	74,144429	0,5342203	-0,904493	0,4886658	0,849016	enoyl-CoA hydratase, putative
PFIT_06236	72,4523396	47,5642316	65,45020203	90,7648109	120,197573	43,74220703	97,26386611	98,61833051	73,361891	97,941098	1,3350405	0,4168836	0,4887203	0,849016	N-acetylglucosaminylphosphatidylinositol deacetylase, putative
PFIT_14711	81,50888205	15,85473772	18,70005721	14,3895432	34,53953267	34,99376562	12,0078847	18,96506356	33,331086	15,486474	0,4646255	-1,10586	0,4887546	0,849016	autophagy-related protein 12, putative (ATG12)
PFIT_13152	14,71688148	24,09920133	23,06340389	24,90497861	11,05265045	2,916147135	24,01576941	31,29235487	16,79221	27,654062	1,6468387	0,7196993	0,4889637	0,84904	protein kinase, putative
PFIT_14316	261,5076633	161,6208933	168,9238501	125,078337	120,197573	209,9625937	120,078847	117,5833941	174,88182	118,83113	0,6794939	-0,557468	0,4891085	0,84904	conserved Plasmodium protein, unknown function
PFIT_13103	1158,105366	221,9663281	263,0474714	400,1399897	93,94752885	116,6458854	152,5001357	151,7205085	375,64209	152,11032	0,4049342	-1,304261	0,4892188	0,84904	conserved Plasmodium protein, unknown function
PFIT_12530	2696,585515	1536,64118	1355,754148	1709,588421	1038,949451	1469,738156	2225,061036	2028,313548	1634,5428	2126,6873	1,30109	0,3797208	0,4896439	0,8495172	Plasmodium exported protein (PHISTb), unknown function
PFIT_11401	8209,755731	3358,667638	3629,681104	3452,936929	1112,172952	2283,341207	2659,746462	2170,551524	3674,4263	2415,149	0,657286	-0,605407	0,4904116	0,8505882	unspecified product
PFIT_05116	124700,6651	41753,76888	49294,59747	58052,9517	27436,82317	34031,43707	34450,62122	38143,48409	55878,374	36297,053	0,6495725	-0,622438	0,4908068	0,8509483	RNA pseudouridylate synthase, putative
PFIT_12051	40569,91397	16996,91302	17364,24979	22714,44738	11544,4934	11994,11317	27095,79184	26269,45779	20197,355	26682,625	1,321095	0,4017342	0,4910996	0,8509483	zinc finger protein, putative
PFIT_14422	181,130849	452,1771198	507,3948856	515,2563353	378,553278	542,4033672	307,4018484	323,3543337	429,48597	315,37809	0,7343152	-0,445529	0,4913291	0,8509483	conserved Plasmodium protein, unknown function
PFIT_07287	1,132067806	3,170947544	1,870005721	2,213775877	6,907906533	5,832294271	7,204730823	4,74126589	3,6250555	5,9729984	1,6476985	0,7204522	0,491354	0,8509483	dynein light chain, putative
PFIT_05039	107,5464416	104,6412689	66,07353547	49,80995723	127,1054802	169,1365339	124,8820009	116,6351409	104,0522	120,75857	1,1605576	0,2148181	0,4915027	0,8509483	conserved Plasmodium protein, unknown function
PFIT_12511	384,9030541	227,6740337	244,3474142	210,8621523	183,7503138	233,2917708	162,1064435	183,9611165	247,47146	173,03378	0,699207	-0,516208	0,4915219	0,8509483	conserved Plasmodium protein, unknown function
PFIT_bin01	69,05613618	15,85473772	16,20671625	15,49643114	16,57897568	8,748441406	25,21655788	40,77488665	23,656906	32,995722	1,3947607	0,4800176	0,4918259	0,8512313	erythrocyte membrane protein 1, PfEMP1
PFIT_08201	806,0322781	858,0584054	786,025738	990,1112609	906,3173372	393,6798633	607,5989661	599,2960085	790,03748	603,44749	0,7638213	-0,388693	0,4923088	0,8513813	conserved Plasmodium protein, unknown function
PFIT_07036	3001,111754	1249,987522	1338,300761	1303,360547	1102,501883	930,2509362	1095,119085	1044,975002	1487,5856	1070,047	0,719318	-0,475298	0,4924793	0,8513813	phosphopantetheine adenylyltransferase, putative (PPAT)
PFIT_14484	13271,23089	2354,111457	3149,089634	3103,717379	1894,147971	3898,88872	1890,041053	2132,621397	4611,8637	2011,3312	0,4361211	-1,197199	0,4925403	0,8513813	conserved Plasmodium protein, unknown function
PFIT_10113	58051,30504	18924,21494	21751,90655	23851,2213	12637,32421	31462,31144	17425,84228	18240,59813	27779,714	17833,22	0,6419512	-0,639465	0,4929117	0,8513813	QF122 antigen
PFIT_12016	761,8816336	400,8077695	380,2344966	380,2160068	357,8295584	533,6549258	333,8191948	312,9235487	469,10407	323,37137	0,6893382	-0,536716	0,4929839	0,8513813	conserved protein, unknown function
PFIT_14329	4146,764374	6628,548746	8934,263999	8248,529187	6011,260265	17170,27433	5228,233	5837,446564	8523,2734	5532,8398	0,6491449	-0,623387	0,4930312	0,8513813	60S ribosomal protein L14, putative
PFIT_06050	6432,409275	4105,74288	5712,867477	7213,588695	3578,295584	5211,154931	3897,759375	4107,832767	5375,6765	4002,7961	0,7446125	-0,425438	0,4931799	0,8513813	RNA binding protein, putative
PFIT_12205	30,56583077	13,95216919	6,233352403	7,1947716	6,907906533	2,916147135	3,602365411	6,637772246	11,29503	5,1200688	0,4533028	-1,141453	0,4933757	0,8513813	conserved Plasmodium protein, unknown function
PFIT_12218	31,69789858	119,8618172	156,4571453	135,5937725	116,0528298	186,6334167	92,46071223	83,44627967	124,38281	87,953496	0,7071194	-0,499874	0,4933996	0,8513813	conserved Plasmodium protein, unknown function
PFIT_12317	403,016139	311,3870488	260,5541304	245,1756784	250,0662165	300,3631549	232,9529633	187,7541292	295,09373	210,35355	0,7128364	-0,488357	0,4935095	0,8513813	conserved Plasmodium protein, unknown function
PFIT_14505	1666,403811	2863,365632	2932,16897	3451,830036	1866,516345	2096,70979	1906,852091	1955,298053	2479,4991	1931,0751	0,7788166	-0,360644	0,4935784	0,8513813	transcription factor IIIb subunit, putative
PFIT_07161	708,6744467	392,5633059	423,2446282	427,2587442	344,0137454	134,1427682	521,1421962	572,7449195	404,98294	546,94356	1,3505348	0,4335308	0,4941211	0,8516702	dynein heavy chain, putative
PFIT_10011	183888,5662	40186,6866	34042,83081	35997,10264	41211,1888	71416,44335	30161,4048	31445,97189	67790,47	30803,688	0,4543956	-1,137979	0,4941394	0,8516702	early transcribed membrane protein 10.1
PFIT_06049	13271,23089	8156,311272	8633,193078	9963,651777	5545,667365	5943,107862	6270,517393	6619,755436	8585,527	6445,1364	0,7506978	-0,413696	0,4941976	0,8516702	conserved Plasmodium protein, unknown function
PFIT_13677	237,7342393	63,41895088	84,15025744	96,29925064	149,2107811	186,6334167	91,25992375	78,70501378	136,24115	84,982469	0,6237651	-0,680925	0,4945335	0,8517179	mitochondrial carrier protein, putative
PFIT_09059	45,28271225	62,78476137	100,9803089	99,61991446	109,1449232	160,3880924	45,62996188	76,80850742	96,366785	61,219235	0,6352732	-0,654551	0,4946822	0,8517179	autophagy-related protein 3, putative (ATG3)
PFIT_11290	1218,10496	1119,344483	1600,724897	1553,517222	1056,9097	2924,895577	1049,489123	1089,542902	1578,9161	1069,516	0,6773735	-0,561976	0,4949311	0,8517179	cochaperone prefoldin complex subunit, putative
PFIT_08019	2561,869446	2094,093758	3220,149851	4395,452003	1858,226857	659,0492526	1986,10413	2041,589092	2464,8069	2013,8466	0,8170403	-0,291521	0,4953097	0,8517179	acyl-CoA synthetase
PFIT_06075	695,0896331	51,36935021	165,1838387	144,448876	51,11850835	303,2793021	84,05519293	86,2910392	235,08158	85,173116	0,362313	-1,464691	0,4954913	0,8517179	conserved Plasmodium protein, unknown function
PFIT_05114	478,8646821	466,7634785	371,5078032	360,8454679	525,0008965	653,2169583	570,3745235	577,4861854	476,03322	573,93035	1,2056519	0,2698134	0,4956138	0,8517179	MORN repeat protein, putative
PFIT_12472	10,18861026	25,36758035	21,81673341	24,90497861	62,1711588	14,58073568	27,61813482	42,67139301	26,504966	35,144764	1,325969	0,4070471	0,4958999	0,8517179	DNA replication origin binding protein, putative
PFIT_03001	0	0,634189509	1,870005721	0,553443969	0	0	3,602365411	0,948253178	0,5096065	2,2753093	4,4648354	2,158607	0,4962585	0,8517179	stevor
PFIT_bin04	6,792406838	27,27014888	47,37347826	37,63418991	84,27645971	29,16147135	48,03153882	54,99868432	38,751359	51,515112	1,3293756	0,4107488	0,4966562	0,8517179	rifin (RIF)
PFIT_11048	2096,589577	1957,743014	2419,164068	2101,426751	1924,54276	3175,68423	1555,021069	1643,322757	2279,1917	1599,1719	0,7016399	-0,511197	0,4968227	0,8517179	phenylalanyl-tRNA synthetase beta chain, putative
PFIT_03181	243,3945784	167,4260303	196,9739359	173,7814063	169,9345007	574,4809857	145,2954049	137,4967108	254,33191	141,39606	0,5559509	-0,846971	0,4972175	0,8517179	stomatin-like protein
PFIT_09295	423,3933595	114,7883011	130,2770652	148,8764277	113,2896671	84,56826693	105,6693854	91,03230509	169,19885	98,350845	0,5812737	-0,78271	0,497237	0,8517179	conserved Plasmodium protein, unknown function
PFIT_11206	312,4507145	421,1018338	553,5216934	575,028284	425,5270424	294,5308607	302,5986946	355,5949418	430,36007	329,09682	0,7647011	-0,387032	0,4972452	0,8517179	alternative splicing factor ASF-1, putative
PFIT_13429	8468,999259	6815,634651	6794,977454	7537,90686	4524,678779	3948,463221	4613,429304	5083,585287	6348,4434	4848,5073	0,7637317	-0,388862	0,497394	0,8517179	kelch protein, putative
PFIT_09168	8326,358715	15212,93794	19381,36263	21669,54517	14318,70866	18170,5128	11557,58903	12785,2976	16179,906	12171,443	0,7522568	-0,410703	0,4974145	0,8517179	RNA-binding protein musashi, putative
PFIT_12272	1966,40178	2980,056502	3170,283032	2258,051394	8351,658999	3137,774318	2312,718594	2046,330358	3644,0377	2179,5245	0,598107	-0,741524	0,4974572	0,8517179	cyclin
PFIT_14209	676,9765482	949,3816946	930,0161785	1050,436654	735,0012551	650,3008112	646,0241971	633,4331229	832,01886	639,72866	0,768872	-0,379156	0,4975225	0,8517179	YTH domain-containing protein, putative
PFIT_08162	8089,756544	4551,578105	3180,879731	2845,80889	2311,385526	5132,418958	2826,656059	3141,562779	4351,9713	2984,1094	0,6856914	-0,544369	0,4976746	0,8517179	conserved Plasmodium protein, unknown function
PFIT_13069	3766,389591	3706,837679	4146,426018	5164,739121	3125,136956	3840,565777	5536,835637	5064,620224	3958,3492	5300,7279	1,3391259	0,4212916	0,4977296	0,8517179	conserved Plasmodium protein, unknown function
PFIT_06146	4532,799496	4160,283178	4436,90024	4046,228859	3325,466205	4079,689842	2838,663944	3153,89007	4096,8946	2996,277	0,7313532	-0,45136	0,4979416	0,8517179	conserved Plasmodium protein, unknown function
PFIT_14693	1334,707944	1023,581867	1108,290057	708,9617245	951,9095203	2277,510913	848,9574486	762,3955551	1234,1603	805,6765	0,6528135	-0,615257	0,4980351	0,8517179	conserved protein, unknown function
PFIT_11196	9,05654245	38,68556004	18,07672197	23,24464671	30,39478875	11,66458854	31,22050023	34,13711441	21,853808	32,678807	1,495337	0,5804706	0,4981176	0,8517179	conserved Plasmodium protein, unknown function
PFIT_05348	1111,690586	525,7431028	613,9852117	578,9023918	446,250762	393,6798633	435,8862148	457,0580318	611,70805	446,47212	0,7298771	-0,454275	0,498148	0,8517179	XAP-5 DNA binding protein, putative
PFIT_08188	43,01857664	58,34543481	59,21684783	52,0237321	30,39478875	43,74220703	70,84651976	65,42946889	47,790265	68,137995	1,4257714	0,5117427	0,4981929	0,8517179	lipoamide dehydrogenase
PF_apicopla	0	0,634189509	0,62333524	0,553443969	0	0	1,20078847	1,896506356	0,3018281	1,5486474	5,1308918	2,3592096	0,4984041	0,8517179	unspecified product
PFIT_11193	7307,49769	4804,619719	5592,563776	5837,726987	3804,874919	5444,446702	3777,680528	4185,589528	5465,2883	3981,635	0,7285316	-0,456937	0,4984327	0,8517179	box C/D snoRNP rRNA 2'-O-methylation factor, putative
PFIT_07258	254,7152564	272,7014888	213,8039874	168,8004106	357,8295584	775,695138	188,5237899	169,7373189	340,59097	179,13055	0,5259404	-0,927029	0,4986004	0,8517179	mitochondrial inner membrane translocase subunit tim50, putative (TIM50)
PFIT_06297	380,3747829	273,3356783	243,1007437	248,4963422	107,7633419	84,56826693	180,1182706	165,9443062	222,93986	173,03129	0,7761344	-0,365622	0,4991532	0,8517179	conserved Plasmodium protein, unknown function
PFIT_07262	624,9014291	751,5145679	756,7289817	661,9189872	501,5140143	583,2294271	512,7366769	465,5923104	646,63457	489,16449	0,7564775	-0,402631	0,4992092	0,8517179	DNA (cytosine-5)-methyltransferase, putative
PFIT_05173	5497,321267	1941,888276	2674,108181	2753,937791	903,5541745	2668,274629	1886,438687	1899,351116	2739,8473	1892,8949	0,6908761	-0,533501	0,4995166	0,8517179	conserved Plasmodium protein, unknown function
PFIT_14296	133,5840011	61,51638235	77,91690504	78,03559966	109,1449232	87,48441406	114,0749047	113,7903814	91,280371	113,93264	1,2481615	0,3198046	0,4996992	0,8517179	probable protein, unknown function
PFIT_13558	132,4519333	222,6005176	133,3937414	104,6009102	207,237196	335,3569206	240,9450786	219,9947373	189,27504	230,46991	1,1648216	0,220109	0,4998956	0,8517179	DnaJ protein, putative
PFIT_09267	91,69749231	144,595208	167,6771796	287,23742	167,1713381	87,48441406	232,9529633	221,8912437	157,64384	227,4221	1,4426323	0,5287037	0,4999418	0,8517179	conserved Plasmodium membrane protein, unknown function
PFIT_02029	71,3202718	41,85650758	59,21684783	58,66506073	156,1186877	29,16147135	86,45676987	89,13579873	69,389808	87,796284	1,265262	0,3394361	0,5002545	0,8517179	conserved Plasmodium protein, unknown function
PFIT_09123	645,2786496	636,0920773	588,4284668	743,2752506	449,0139247	594,8940156	439,4885802	466,5405636	609,49706	453,01457	0,7432596	-0,428062	0,5003829	0,8517179	conserved Plasmodium protein, unknown function
PFIT_13224	1621,121099	1814,416185	3727,544737	3582,996257	2753,491544	8593,885608	2014,923053	1997,021193	3682,2426	2005,9721	0,5447691	-0,876283	0,5003954	0,8517179	vacuolar ATP synthase subunit g, putative
PFIT_06014	71,3202718	210,5509169	303,564262	237,4381662	237,6319847	469,4996888	308,6026369	316,7165615	255,36806	312,6596	1,2243489	0,2920147	0,500642	0,8517179	geranylgeranyltransferase, putative
PFIT_08173	408,6764781	322,80246	420,7512872	509,7218956	225,197753	169,1365339	309,8034254	239,908054	342,7144	274,85574	0,8019965	-0,318332	0,5008473	0,8517179	phospholipase DDHD1, putative
PFIT_03141	219,6211544	199,1355058	202,5839531	185,9571737	319,1452818	139,9750625	295,3939637	253,1835985	211,06969	274,28878	1,2995176	0,3779762	0,50102	0,8517179	conserved Plasmodium protein, unknown function
PFIT_09192	681,5048194	1684,407335	1846,942317	1812,528999	1610,923804	1848,837284	1171,969547	1183,819966	1580,8574	1177,6948	0,7449722	-0,424742	0,5012632	0,8517179	DnaJ protein, putative
PFIT_11170	329,4317316	283,4827104	386,467849	289,4511959	254,2109604	288,6985664	189,7245783	250,338839	305,2905	220,03171	0,720729	-0,472471	0,5013714	0,8517179	SNO glutamine amidotransferase family protein (PDX2)
PFIT_11317	1356,217232	452,8113093	574,7150915	563,9594046	294,2768183	355,7699505	398,6617722	385,9390435	599,62497	392,30041	0,654243	-0,612102	0,5014648	0,8517179	mitochondrial rpoD precursor, putative
PFIT_07012	11034,26491	6416,09526	8767,210155	10035,04605	4375,467998	5266,561727	5775,792543	5980,632794	7649,1077	5878,2127	0,7684835	-0,379914	0,5014847	0,8517179	sporozoite threonine and asparagine-rich protein (STARP)
PFIT_14468	2130,551611	3340,276143	6488,919851	7125,591103	3253,623977	4948,701689	3575,948065	3518,967544	4547,9441	3547,4578	0,7800135	-0,358429	0,5016045	0,8517179	RNA binding protein, putative
PFIT_01020	133,5840011	111,6190503	131,2772283	120,3037014	175,4611822	140,9212933	192,1261553	175,4268379	136,94057	183,7765	1,3420164	0,4244023	0,5016994	0,8517179	conserved Plasmodium protein, unknown function
PFIT_bin08	0	1,268379018	10,59669908	5,534439692	0	46,65835417	1,20078847	0	10,676312	0,6003942	0,0562361	-4,152359	0,5017236	0,8517179	rifin
PFIT_03232	6210,523985	1634,940554	1174,986928	863,9260359	1675,858125	3528,538034	1149,154566	1179,626953	2514,7956	1164,3908	0,4630161	-1,110866	0,501775	0,8517179	conserved Plasmodium protein, unknown function
PFIT_07237	28,30169516	29,80690691	31,79009725	32,65319418	4,14474392	2,916147135	33,62207717	36,98187394	21,602131	35,301976	1,6341895	0,7085753	0,5020832	0,8517179	conserved Plasmodium membrane protein, unknown function
PFIT_09017	0	0,634189509	0,62333524	1,106887938	0	2,916147135	1,20078847	2,844759534	0,8800933	2,022774	2,2983631	1,2006068	0,5023453	0,8517179	rifin (RIF)
PFIT_13340	842,2584479	1898,763389	1881,225755	2112,49563	1428,555071	1113,968206	1127,540374	1296,262094	1546,2111	1211,9012	0,7837877	-0,351465	0,5023467	0,8517179	probable protein, unknown function
PFIT_13313	267,1680023	777,5163378	670,7087186	689,5911856	689,409072	822,3534922	755,2959479	914,1160636	652,79133	834,70601	1,2786724	0,3546467	0,502498	0,8517179	syntaxin, Qa-SNARE family
PFIT_11402	13,58481368	3,170947544	7,480022884	1,106887938	1,381581307	2,916147135	1,20078847	1,896506356	4,9400667	1,5486474	0,3134871	-1,673522	0,5027236	0,8517179	AAA family ATPase, putative
PFIT_13464	5397,6993	9041,639827	9217,258198	7360,80479	6315,208151	8940,907117	5329,099232	6058,389554	7712,2529	5693,7444	0,7382725	-0,437775	0,5027836	0,8517179	nucleoside transporter 1
PFIT_08142	24363,23126	15592,18326	24470,27153	25796,0234	19615,69139	25364,64778	16580,4872	16241,68043	22533,675	16411,084	0,7282915	-0,457412	0,5028474	0,8517179	CCR4-associated factor 1
PFIT_06248	53,2071869	12,04960067	9,973363845	21,03087083	44,21060181	20,41302995	16,81103859	13,27554449	26,814109	15,043292	0,5610215	-0,833872	0,5028678	0,8517179	conserved Plasmodium protein, unknown function
PFIT_10022	41,88650883	47,56421316	59,21684783	58,11161677	78,75013448	75,81982552	88,85834681	67,32597564	60,224858	78,092161	1,2966766	0,3748187	0,5030747	0,8517179	conserved Plasmodium protein, unknown function
PFIT_14605	67,92406838	51,36935021	50,49015446	79,69593156	66,31590272	23,32917708	46,83075035	36,03362076	56,520764	41,432186	0,7330436	-0,448029	0,5031934	0,8517179	OPA3-like protein, putative
PFIT_13434	7765,985151	6776,949091	8277,891991	7353,610019	7108,235823	12860,20887	5748,174408	5813,740284	8357,1468	5780,9573	0,6917382	-0,531702	0,5032984	0,8517179	heat shock protein 110, putative (HSP110)
PFIT_11333	473,204343	187,0859051	324,134325	295,5390796	183,7503138	148,7235039	395,0594068	331,8886123	268,73958	363,47401	1,3525139	0,4356434	0,5035106	0,8517179	amino acid transporter, putative
PFIT_05050	2755,45304	1721,190327	2098,769754	2089,804428	1003,028029	1945,070139	1304,056279	1536,170148	1935,5526	1420,1132	0,7336991	-0,44674	0,5035107	0,8517179	conserved Plasmodium membrane protein, unknown function
PFIT_14699	2106,778187	1026,752815	1040,969851	1105,78105	685,2643281	2335,833855	841,7527178	981,4420392	1383,5631	911,59738	0,6588765	-0,60192	0,5036309	0,8517179	U2 snRNP auxiliary factor, putative
PFIT_13691	0	0	0	0,553443969	2,763162613	0	2,401576941	0,948253178	0,5527678	1,6749151	3,030052	1,5993425	0,5036659	0,8517179	tRNA Cysteine
PFIT_07045	288,6772906	216,892812	120,3037014	126,6507853	110,5265045	256,6209479	212,5395593	234,218535	185,61201	223,37905	1,203473	0,2672038	0,5037123	0,8517179	RNA helicase, putative
PFIT_12434	2,264135613	6,976084597	6,856687643	5,534439692	4,14474392	0	2,401576941	0,948253178	4,2960152	1,6749151	0,3898764	-1,358911	0,5042295	0,8523377	conserved Plasmodium protein, unknown function
PFIT_08277	4267,89563	2577,980353	3811,694994	4740,247596	2369,411941	2446,647447	2690,966962	2519,508694	3368,9797	2605,2378	0,7733017	-0,370897	0,5044034	0,852377	conserved Plasmodium membrane protein, unknown function
PFIT_06023	1065,275806	623,4082871	653,8786671	717,816828	374,4085341	1166,458854	483,9177536	566,1071473	766,8745	525,01245	0,6846133	-0,546639	0,5052069	0,8534129	trophozoite exported protein 1 (TEX1)
PFIT_06264	3982,614542	7997,129706	9292,058427	9856,283647	5798,496744	12376,12844	10826,30885	10351,13169	8217,1186	10588,72	1,2886172	0,3658237	0,5053646	0,8534129	4-methyl-5(B-hydroxyethyl)-thiazol monophosphate biosynthesis enzyme
PFIT_07264	53,2071869	27,27014888	24,31007437	25,45842258	122,9607363	75,81982552	79,25203905	47,41262894	54,837732	63,332349	1,1549046	0,2077737	0,5054351	0,8534129	conserved Plasmodium protein, unknown function
PFIT_13053	0	3,170947544	3,740011442	3,320663815	33,15795136	0	14,40946165	9,48253178	7,2315957	11,945997	1,6519171	0,7241413	0,5056486	0,8534129	conserved Plasmodium protein, unknown function
PFIT_12277	36,2261698	27,90433839	43,01013158	35,973858	40,06585789	17,49688281	25,21655788	20,86156092	33,446206	23,039064	0,6888394	-0,53776	0,5058435	0,8534129	conserved Plasmodium protein, unknown function
PFIT_05358	111501,8865	18118,16008	19656,8768	18705,29927	22114,97198	46168,44145	14644,81619	17592,94121	39377,606	16118,879	0,4093413	-1,288624	0,5059217	0,8534129	early transcribed membrane protein 5
PFIT_06179	1609,800421	1518,249684	1547,118066	1407,408014	1352,568099	1802,17893	1021,870988	1175,833941	1539,5539	1098,8525	0,7137473	-0,486515	0,5061504	0,853544	conserved Plasmodium protein, unknown function
PFIT_14352	305,6583077	383,0504633	372,1311385	287,23742	265,2636109	253,7048008	216,1419247	247,4940795	311,17429	231,818	0,744978	-0,42473	0,5065479	0,8539597	CCR4-associated factor 16, putative
PFIT_06172	3678,088303	6310,819802	7053,038244	6251,149632	6551,458556	16636,61941	4291,617993	5069,36149	7746,8623	4680,4897	0,6041788	-0,726953	0,5068501	0,8542145	60S ribosomal protein L27a, putative
PFIT_02166	1163,765705	502,278091	587,1817964	754,34413	408,9480668	594,8940156	534,3508694	450,4202596	668,56863	492,38556	0,7364772	-0,441287	0,5071964	0,8542908	conserved Plasmodium protein, unknown function
PFIT_07206	5266,379435	4354,345167	5459,79337	5994,905074	3763,427479	6010,179246	3628,782758	3917,233878	5141,505	3773,0083	0,7338334	-0,446475	0,5071974	0,8542908	GTP binding protein, putative
PFIT_03240	14,71688148	5,707705579	2,493340961	3,874107784	5,526325227	2,916147135	1,20078847	2,844759534	5,872418	2,022774	0,3444533	-1,53762	0,5077992	0,8549324	erythrocyte membrane protein 1 (PfEMP1), exon2, pseudogene
PFIT_02042	4,528271225	41,85650758	38,6467849	28,22564243	110,5265045	81,65211979	73,2480967	51,20567161	50,905972	62,226884	1,2223887	0,2897031	0,5078806	0,8549324	ubiE/COQ5 methyltransferase, putative
PFIT_12044	3,396203419	29,1727174	31,16676201	28,7790864	106,3817606	49,5745013	64,8425774	42,67139301	41,411839	53,756985	1,2981067	0,376409	0,5081144	0,8554079	conserved Plasmodium protein, unknown function
PFIT_05133	6115,43029	5124,251231	5369,430464	4226,651593	4252,507262	4989,527749	3401,833737	3627,068406	4869,6998	3514,4511	0,7216977	-0,470533	0,5086573	0,8557305	conserved Plasmodium protein, unknown function
PFIT_14697	235,4701037	232,1133602	257,4374542	222,4844756	190,6582203	139,9750625	171,7127513	152,6687617	213,02311	162,19076	0,7613763	-0,393318	0,5088686	0,8558313	aminophospholipid transporter, putative
PFIT_13412	41287,64496	12894,34109	18814,12756	18066,62493	9345,015958	18675,00626	13254,30314	13737,34379	19847,127	13495,823	0,6799888	-0,556417	0,5100103	0,8573997	40S ribosomal protein S6, putative
PFIT_04042	105,282306	241,6262028	298,5775801	263,9927733	193,4213829	227,4594766	169,3111743	167,8408125	221,72662	168,57599	0,7602876	-0,395383	0,5101806	0,8573997	conserved Plasmodium protein, unknown function
PFIT_04107	121,1312553	97,03099484	119,0570309	119,5438573	117,4344111	145,8073568	141,6930395	159,3065339	120,00082	150,49979	1,2541563	0,3267171	0,5104136	0,8573997	mitochondrial ribosomal protein S12 precursor, putative
PFIT_14019	30,56583077	73,56598302	31,16676201	20,47742686	23,48688221	8,748441406	37,22444258	54,99868432	31,335221	46,111563	1,471557	0,5573434	0,5106898	0,8573997	erythrocyte membrane protein 1, PfEMP1, putative
PFIT_02212	44,15064444	69,76084597	208,1939703	163,2659709	67,69748403	347,0215091	80,45282752	81,54977331	150,01507	81,0013	0,5399544	-0,88909	0,5106996	0,8573997	hypothetical protein
PFIT_07147	27,16962735	27,27014888	39,89345538	38,18763387	116,0528298	137,0589154	75,6496764	67,32597564	64,272182	71,487825	1,1122684	0,1535049	0,5107107	0,8573997	cysteine desulfurase, putative
PFIT_04174	0	10,14703214	3,740011442	6,64132763	5,526325227	2,916147135	9,606307764	8,534278602	4,8284739	9,0702932	1,878501	0,9095819	0,5110301	0,8574065	conserved Plasmodium protein, unknown function
PFIT_09289	301,1300365	161,0841352	161,4438272	216,396592	211,3819399	99,1490026	150,0985588	132,7554449	191,76426	141,427	0,7375045	-0,439276	0,5112373	0,8574065	conserved Plasmodium protein, unknown function
PFIT_02159	1301,877977	857,4242159	919,4194794	1021,657567	569,2114983	644,4685169	726,4770246	631,5366166	885,67654	679,00682	0,7666533	-0,383354	0,5112577	0,8574065	conserved Plasmodium protein, unknown function
PFIT_13287	386,0351219	807,3232447	779,1690504	561,1921848	518,09299	574,4809857	441,8901571	476,9713485	604,38226	459,43075	0,7601658	-0,395614	0,5113211	0,8574065	AMP deaminase, putative
PFIT_13665	80,37681425	145,05845	142,1204348	173,7814063	250,0662165	55,40679557	199,3308861	183,9611165	140,6502	191,646	1,3627189	0,446488	0,5118205	0,8574065	NADH-cytochrome b5 reductase, putative
PFIT_11296	181,130849	97,03099484	82,28025172	84,67692729	165,7897568	40,8260599	144,0946165	133,7036981	108,62247	138,89916	1,2787331	0,3547152	0,5119826	0,8579454	GPI8p transamidase
PFIT_03171	584,146988	239,7236343	243,1007437	223,0379196	121,579155	329,5246263	192,1261553	199,1331674	290,18551	195,62966	0,6741538	-0,56885	0,5122081	0,8579454	conserved Plasmodium protein, unknown function
PFIT_03225	8679,563871	1397,119488	1234,827111	1061,505533	927,0410568	1697,197633	683,2486397	810,7564672	2499,5424	747,00255	0,2988557	-1,742479	0,5124076	0,8579454	regulator of initiation factor 2 (eIF2)
PFIT_08155	295,4696974	435,054003	923,7828261	916,5023175	607,8957749	799,0243151	469,5082919	525,3322606	662,95497	497,42028	0,7503078	-0,414466	0,5125021	0,8579454	ubiquitin conjugating enzyme, putative
PFIT_10007	4,528271225	5,07351607	1,246670481	6,087883661	4,14474392	0	8,405519293	5,689519068	3,5135142	7,0475192	2,005832	1,0042008	0,5130791	0,8579454	acyl-CoA binding protein, isoform 1, ACBP1
PFIT_10156	82,64094986	60,88219284	89,7602746	109,0284619	46,97376443	64,15523698	108,0709623	102,4113432	75,57348	105,24115	1,3925672	0,477747	0,5132207	0,8579454	conserved Plasmodium protein, unknown function
PFIT_03151	1335,840011	2376,942279	2632,968055	2627,751966	2286,517063	3146,522759	1617,46207	1882,282558	2401,0904	1749,8723	0,7287824	-0,45644	0,5134108	0,8579454	translation initiation factor 4E
PFIT_12214	533,2039368	227,0398441	251,2041018	259,0117776	190,6582203	370,3506862	211,3387708	209,5639523	305,24476	210,45136	0,6894512	-0,53648	0,5134179	0,8579454	FbpA domain protein, putative
PFIT_08302	5467,887504	2028,138049	2847,395378	3365,492777	1724,213471	2694,519953	2136,202689	2281,497146	3021,2745	2208,8499	0,7310987	-0,451862	0,5138729	0,8579454	conserved Plasmodium protein, unknown function
PFIT_13587	1219,237027	503,54647	825,2958581	861,1588161	1514,213112	1825,508107	1353,288606	1159,713637	1124,8266	1256,5011	1,1170621	0,1597094	0,5139672	0,8579454	conserved Plasmodium protein, unknown function
PFIT_12382	310,1865789	237,8210658	170,1705206	185,9571737	424,1454611	554,0679557	332,6184063	348,0089163	313,72479	340,31366	1,0847522	0,1173655	0,5139904	0,8579454	conserved Plasmodium protein, unknown function
PFIT_07127	12104,06898	1317,845799	2093,159737	2553,590474	1434,081396	1775,933605	1170,768759	1168,247915	3546,4467	1169,5083	0,329769	-1,600472	0,5140192	0,8579454	conserved Plasmodium protein, unknown function
PFIT_13693	277326,0467	65435,03933	36833,50268	25989,17535	34761,96726	46550,45672	21813,52335	28719,744	81149,365	25266,634	0,3113596	-1,683346	0,5141669	0,8579454	membrane associated histidine-rich protein
PFIT_14500	228,6776969	85,61558369	94,74695652	75,26837981	111,9080858	75,81982552	73,2480967	78,70501378	112,00609	75,976555	0,6783252	-0,559951	0,5141866	0,8579454	Appr-1-p processing domain protein
PFIT_08194	15,84894929	8,244463614	8,103358124	10,51543541	11,05265045	5,832294271	8,405519293	19,91331674	9,9328585	14,159418	1,4255129	0,5114811	0,514219	0,8579454	small heat shock protein HSP20, putative (HSP20)
PFIT_13048	2,264135613	65,95570891	56,10017163	53,68406501	49,73692704	20,41302995	27,61813482	34,13711441	41,359006	30,877625	0,7465756	-0,42164	0,5144541	0,8579454	membrane integral peptidase, M50 family, putative
PFIT_09242	504,9022416	444,5668457	527,3416133	545,1423097	541,5798722	874,8441406	414,2720223	382,1460307	573,06284	398,20903	0,6948785	-0,525167	0,5145356	0,8579454	conserved Plasmodium protein, unknown function
PFIT_12435	1,132067806	8,244463614	16,20671625	33,76008212	26,25004483	2,916147135	6,003942352	10,43078496	14,751587	8,2173637	0,5570495	-0,844123	0,5149579	0,8579454	conserved Plasmodium protein, unknown function
PFIT_12173	87,16922108	117,9592486	69,19021167	66,4132763	45,59218312	52,49064844	63,64178893	46,46440572	73,135798	55,053097	0,7527517	-0,409754	0,5149649	0,8579454	anaphase promoting complex subunit 10, putative
PFIT_05148	1149,048823	223,2347071	252,4507723	256,2445577	129,8686428	137,0589154	141,6930395	140,3414703	357,9844	141,01725	0,3939201	-1,344025	0,5154534	0,8579454	conserved Plasmodium protein, unknown function
PFIT_08251	5800,715439	901,8174815	934,3795252	810,2419709	639,672145	982,7415846	548,760331	514,9014757	1678,2614	531,8309	0,316894	-1,657928	0,5154602	0,8579454	conserved Plasmodium membrane protein, unknown function
PFIT_07024	26368,12334	24130,91081	37193,79045	37489,18759	22337,40657	45486,06302	22846,20144	24113,13006	32167,58	23479,666	0,7299171	-0,454196	0,5156759	0,8579454	erythrocyte membrane-associated antigen
PFIT_bin04	2,264135613	8,878653123	9,973363845	3,320663815	2,763162613	11,66458854	1,20078847	3,793012712	6,4774279	2,4969006	0,3854772	-1,375283	0,5157951	0,8579454	Plasmodium exported protein (PHISTb), unknown function
PFIT_04029	1,132067806	7,610274105	6,856687643	4,980995723	24,86846352	26,24532422	16,81103859	11,37903814	11,948969	14,095038	1,1796029	0,2383013	0,5158064	0,8579454	conserved Plasmodium protein, unknown function
PFIT_10300	2840,358126	1442,146943	1847,565652	1816,956551	1120,46244	2128,787409	1341,280722	1351,260779	1866,0462	1346,2708	0,7214563	-0,471016	0,5159023	0,8579454	glycoprotease, putative
PFIT_02209	5,660339031	3,805137053	6,233352403	5,534439692	2,763162613	11,66458854	1,20078847	2,844759534	5,9435032	2,022774	0,3403336	-1,554978	0,5160105	0,8579454	Pfmc-2TM Maurer's cleft two transmembrane protein, pseudogene
PFIT_11071	439,2423088	211,8192959	191,3639188	238,5343507	522,2377339	1236,446385	565,5713696	456,1097786	473,274	510,84057	1,079376	0,1101975	0,5160302	0,8579454	exonuclease, putative
Pf_M76611	0	10,78122165	6,856687643	5,534439692	37,30269528	17,49688281	14,40946165	18,01681038	12,995321	16,213136	1,2476133	0,3191709	0,5161504	0,8579454	unspecified product
PFIT_13017	3020,356907	3130,359415	2820,591962	3140,241081	2246,451205	1918,824815	2034,135669	2147,793448	2712,8042	2090,9646	0,7707761	-0,375616	0,5162861	0,8579454	conserved Plasmodium protein, unknown function
PFIT_03094	7572,401556	8175,971147	8983,507483	8345,935055	7453,631149	15152,30052	5984,729737	6877,6803	9280,6245	6431,205	0,6929711	-0,529133	0,5163577	0,8579454	asparagine synthetase, putative
PFIT_11305	112,0747128	125,5695821	82,28025172	73,05460393	110,5265045	75,81982552	74,44888517	63,53296293	96,554237	68,990924	0,7145303	-0,484933	0,5165574	0,8579454	conserved Plasmodium protein, unknown function
PFIT_06237	12,45274587	15,22054821	21,19339817	20,47742686	24,86846352	8,748441406	27,61813482	23,70632945	17,160171	25,662232	1,4954532	0,5805828	0,5166481	0,8579454	conserved Plasmodium protein, unknown function
PFIT_11311	0	1,902568526	1,246670481	0	33,15795136	29,16147135	1,20078847	0,948253178	10,911444	1,0745208	0,0984765	-3,344077	0,5171848	0,8580309	small nucleolar RNA snoR13
PFIT_06017	2249,418731	433,1514345	561,6250535	686,8239658	319,1452818	314,9438906	347,027868	359,3879545	760,85139	353,20791	0,4642272	-1,107097	0,5172336	0,8580309	JmjC domain containing protein
PFIT_13095	382,6389185	472,471184	498,044857	636,4605646	460,0665751	565,7325443	640,0202548	659,9842119	502,56911	650,00223	1,2933589	0,3711227	0,5172361	0,8580309	zinc finger protein, putative
PFIT_11429	10,18861026	29,1727174	49,86681922	29,33253037	55,26325227	119,5620326	31,22050023	17,0685572	48,89766	24,144529	0,4937768	-1,018069	0,517416	0,8580309	conserved Plasmodium protein, unknown function
PFIT_02107	10,18861026	24,73339084	17,45338673	19,37053892	49,73692704	11,66458854	25,21655788	34,13711441	22,19124	29,676836	1,3373221	0,419347	0,5174581	0,8580309	conserved Plasmodium protein, unknown function
PFIT_14134	4467,139564	1857,541071	1648,098375	1601,666847	960,1990081	1571,803306	1308,859433	1365,484576	2017,7414	1337,172	0,6627073	-0,593556	0,5176127	0,8580356	conserved Plasmodium protein, unknown function
PFIT_12064	202,6401373	254,9441825	359,6644336	279,4892044	247,3030591	580,3132799	206,5356169	230,4255223	320,72572	218,48057	0,6812069	-0,553835	0,5179682	0,8581888	conserved Plasmodium protein, unknown function
PFIT_13561	13838,39686	26313,7911	27608,14113	22205,83238	24593,52884	49332,46109	17089,62151	20308,73831	27315,359	18699,18	0,6845665	-0,546737	0,5181314	0,8581888	elongation factor 1-alpha
PFIT_14679	1764,89371	2397,236343	3031,279274	3301,84672	1767,042491	3172,768083	1741,143282	2119,345853	2572,5111	1930,2446	0,7503348	-0,414394	0,5186412	0,8581888	conserved Plasmodium protein, unknown function
PFIT_14427	2917,338737	903,0858605	879,5260241	992,8784807	703,2248851	898,1733177	782,9140827	713,0863899	1215,7046	748,00024	0,6152813	-0,700682	0,5187674	0,8581888	conserved Plasmodium protein, unknown function
PFIT_12088	568,2980388	724,8786085	604,6351831	578,3489478	708,7512103	933,1670833	488,7209075	480,7643613	686,34651	484,74263	0,7062652	-0,501718	0,5188077	0,8581888	U6 snRNA-associated Sm-like protein LSm7, putative
PFIT_08271	107,5464416	57,07705579	73,55355835	66,4132763	139,539712	64,15523698	60,03942352	54,05043115	84,714213	57,044927	0,673808	-0,570505	0,5188107	0,8581888	conserved Plasmodium protein, unknown function
PFIT_06244	105,282306	147,131966	153,3404691	149,4298717	72,22380925	198,2980052	100,8662315	98,61833051	137,7844	99,742281	0,7239011	-0,466136	0,5188817	0,8581888	conserved Plasmodium membrane protein, unknown function
PFIT_07142	8063,718984	1693,920178	1913,639188	2163,412476	928,4226381	1659,28772	1191,182163	1322,813183	2737,0669	1256,9977	0,4592499	-1,122649	0,5189189	0,8581888	large ribosomal subunit assembling factor, putative
PFIT_13657	67,92406838	157,2789982	120,9270366	118,9904534	121,579155	107,897444	91,25992375	81,54977331	115,76619	86,404849	0,7463738	-0,42203	0,519163	0,858273	conserved Plasmodium protein, unknown function
PFIT_02017	481,1288177	184,5491471	177,6505435	171,5676305	149,2107811	303,2793021	156,1025012	168,7890657	244,56437	162,44578	0,6642251	-0,590256	0,5193072	0,858329	conserved Plasmodium protein, unknown function
PFIT_14696	78,11267863	43,12436204	42,38679634	30,43941831	96,71069147	58,32294271	72,04730823	81,54977331	63,467815	76,798541	1,2100391	0,2750537	0,5196022	0,8584768	derlin-1 (DER1-1)
PFIT_09338	416,6009527	195,3303687	140,8737643	144,448876	379,9348593	244,9563594	311,0042138	274,9934216	253,69086	292,99882	1,1549443	0,2078233	0,5197002	0,8584768	conserved Plasmodium protein, unknown function
PFIT_14310	4591,667022	6501,076655	7600,326585	8021,063446	6157,707884	6351,368461	4958,055595	5058,930705	6537,2017	5008,4931	0,7661525	-0,384297	0,5200609	0,858543	coatamer beta subunit, putative
PFIT_06097	11564,07264	9547,088865	11059,83717	12296,41811	7148,301681	7579,066405	7205,931611	8004,205076	9865,7975	7605,0683	0,7708519	-0,375474	0,5202959	0,858543	transketolase
PFIT_12356	15203,67064	3334,568437	3817,928347	4226,651593	1899,674297	2455,395888	2391,970633	2447,441452	5156,3149	2419,706	0,4692704	-1,091509	0,5203659	0,858543	pre-mRNA-splicing factor SYF1, putative
PFIT_10336	21,50928832	23,46501182	18,70005721	21,5841148	87,03962232	37,90991276	54,03548117	32,24060805	35,034701	43,138045	1,2312948	0,3001762	0,5204558	0,858543	apicoplast ribosomal protein L27 precursor, putative
PFIT_14280	436,9781732	203,5748323	258,6841247	192,0450573	330,1979323	204,1302995	344,626291	322,4060805	270,93507	333,51619	1,230982	0,2998096	0,5204994	0,858543	conserved Plasmodium protein, unknown function
PFIT_10334	566,0339031	1076,219596	1031,619823	888,2775706	1018,225423	1160,62656	716,8707169	692,22482	956,83381	704,54777	0,7363324	-0,441571	0,5210773	0,858543	Sec1 family protein, putative
PFIT_08233	133,5840011	153,4738611	221,2840103	205,3277126	103,618598	145,8073568	128,4843663	124,2211663	160,51592	126,3508	0,7684645	-0,379949	0,5211683	0,8585913	conserved Plasmodium protein, unknown function
PFIT_11303	2515,454666	448,3719827	512,3815675	477,6221454	262,5004483	396,5960104	309,8034254	273,0969153	768,82114	291,45017	0,3790871	-1,399399	0,5212106	0,8585913	A/G-specific adenine glycosylase, putative
PFIT_12484	590,9393949	1145,346253	978,012992	1002,287028	863,4883167	889,4248763	637,6186778	743,4304916	911,58314	690,52458	0,7575004	-0,400681	0,5216021	0,8585913	RNA binding protein, putative
PFIT_03008	15,84894929	3,170947544	1,870005721	0	6,907906533	0	3,602365411	9,48253178	4,6329682	6,5424486	1,4121506	0,4978939	0,5216714	0,8585913	stevor-like
PFIT_bin01	29,43376296	11,41541116	8,726693364	6,087883661	5,526325227	5,832294271	6,003942352	5,689519068	11,170395	5,8467307	0,5234131	-0,933978	0,5218403	0,8585913	rifin
PFIT_14580	29140,5574	29454,29755	35789,41636	33597,36959	21797,20828	32235,09043	20597,12463	24895,43894	30335,657	22746,282	0,74982	-0,415384	0,521899	0,8585913	V-type H( )-translocating pyrophosphatase, putative
PFIT_06208	2668,283819	3489,944867	5143,139068	5469,133304	3021,518318	3755,99751	3099,235042	3062,857765	3924,6695	3081,0464	0,7850461	-0,349151	0,521906	0,8585913	conserved Plasmodium protein, unknown function
PFIT_08122	1520,367064	440,7617086	517,3682494	863,9260359	547,1061974	428,6736289	1045,886758	816,4459863	719,70048	931,16637	1,2938249	0,3716423	0,522124	0,8585913	unspecified product
PFIT_09287	284,1490194	243,5287714	351,5610755	340,3680411	216,9082651	434,5059232	225,7482324	224,7360032	311,83685	225,24212	0,7223076	-0,469315	0,5225026	0,8585913	conserved Plasmodium protein, unknown function
PFIT_07139	59,99959373	54,54029776	51,73682494	58,66506073	261,118867	154,5557982	135,6890972	109,9973686	106,76941	122,84323	1,1505471	0,2023201	0,5225036	0,8585913	drug/metabolite exporter, drug/metabolite transporter
PFIT_12105	448,2988513	407,1496646	307,9276087	551,7836373	70,46064664	32,07761849	416,6735992	499,7294248	302,94967	458,20151	1,5124674	0,5969041	0,5225474	0,8585913	GPI mannosyltransferase I
PFIT_05202	13866,69856	16061,4835	22467,4954	20105,51251	15509,63175	37105,05615	13708,20118	15341,78817	20852,646	14524,995	0,696554	-0,521693	0,5225767	0,8585913	40S ribosomal protein S9, putative
PFIT_13165	1538,480149	566,3312313	222,5306808	190,9381694	501,5140143	775,695138	309,8034254	319,561321	632,58156	314,68237	0,4974574	-1,007355	0,5229058	0,8585913	conserved Plasmodium protein, unknown function
PFIT_07095	8204,095392	7952,8773	8499,176001	7752,643121	6912,051277	16132,12595	5823,824082	6674,75412	9168,9948	6249,2891	0,6815675	-0,553072	0,522988	0,8585913	60S ribosomal protein L34a, putative
PFIT_bin01	0	3,805137053	2,493340961	2,767219846	1,381581307	8,748441406	0	0,948253178	3,1992868	0,4741266	0,1481976	-2,754406	0,5230353	0,8585913	stevor
PFIT_13244	264,9038667	419,1992653	496,7981865	414,5295329	208,6187773	979,8254375	272,5789828	317,6648146	463,97918	295,1219	0,6360671	-0,652749	0,5231887	0,8585913	oxidoreductase, putative
PFIT_10065	261,5076633	310,7528593	355,301087	290,0046399	308,0926314	288,6985664	222,145867	226,6325095	302,39291	224,38909	0,7420451	-0,430421	0,5232085	0,8585913	PPPDE peptidase, putative
PFIT_08169	20534,57794	11202,32348	15694,95802	14650,21531	9702,845517	26262,8211	10876,74197	11721,35753	16341,29	11299,05	0,6914417	-0,53232	0,5234128	0,8585913	60S ribosomal protein L13-2, putative
PFIT_12110	3592,051149	4605,484213	6210,912334	7614,282128	4133,691269	5803,132799	3986,617722	4225,416551	5326,5923	4106,0169	0,7708525	-0,375473	0,5238765	0,8589691	heat shock protein DNAJ homologue Pfj4
PFIT_11248	1356,217232	1810,611048	2283,90032	2110,835299	1722,831889	3201,929555	1406,123299	1572,203769	2081,0542	1489,1635	0,7155813	-0,482812	0,5241954	0,8589691	splicing factor, putative
PFIT_12462	27,16962735	50,10097119	47,9968135	40,40140975	22,10530091	67,07138411	21,61419247	34,13711441	42,474251	27,875653	0,6562953	-0,607583	0,5243683	0,8589691	conserved Plasmodium protein, unknown function
PFIT_04119	389,4313254	86,24977319	81,65691648	73,6080479	16,57897568	17,49688281	30,01971176	39,82663348	110,83699	34,923173	0,3150859	-1,666183	0,5244686	0,8589691	erythrocyte membrane protein 1 (PfEMP1), exon1, pseudogene
PFIT_13469	13327,83428	8262,22092	8904,967243	10835,32603	6693,761431	3890,140279	6857,702955	6745,873108	8652,375	6801,788	0,786118	-0,347182	0,5246902	0,8589691	ethanolamine-phosphate cytidylyltransferase, putative
PFIT_12467	309,0545111	201,0380743	182,6372254	185,9571737	124,3423176	172,052681	168,1103859	124,2211663	195,847	146,16578	0,7463264	-0,422121	0,5247043	0,8589691	conserved Plasmodium protein, unknown function
PFIT_11368	61,13166154	17,75730625	16,83005149	12,17576732	13,81581307	5,832294271	8,405519293	11,37903814	21,257149	9,8922787	0,4653624	-1,103573	0,5249073	0,8589691	conserved Plasmodium protein, unknown function
PFIT_14435	1385,650995	2551,344394	3207,683147	3672,100736	2449,543657	2895,734105	2037,738034	2120,294106	2693,6762	2079,0161	0,7718137	-0,373676	0,5249884	0,8589691	tRNA binding protein, putative
PFIT_05293	8174,661629	12454,21357	9867,396854	11898,49189	8071,197993	5187,825754	12360,91651	13013,82662	9275,6313	12687,372	1,3678176	0,4518758	0,52501	0,8589691	acyl-CoA synthetase
PFIT_08147	280,752816	126,2037122	124,6670481	110,1353499	164,4081755	148,7235039	115,2756932	103,3595964	159,14843	109,31764	0,6868911	-0,541867	0,5253007	0,8591516	conserved Plasmodium protein, unknown function
PFIT_14324	15244,42508	2987,666776	3344,816899	4071,687281	2003,292895	2155,032733	2252,679171	2141,155676	4967,8203	2196,9174	0,4422297	-1,177132	0,5254253	0,8591516	conserved Plasmodium protein, unknown function
PFIT_04020	74,71647521	172,4995464	193,2339245	195,3657211	127,1054802	177,8849753	104,4685969	129,9106854	156,80102	117,18964	0,7473781	-0,42009	0,5258929	0,8591516	conserved Plasmodium protein, unknown function
PFIT_07193	55,47132251	98,93356337	167,6771796	137,8075483	686,6459094	373,2668333	315,8073677	285,4242066	253,30039	300,61579	1,1867956	0,2470715	0,5259283	0,8594761	conserved Plasmodium protein, unknown function
PFIT_02080	90,5654245	337,3888187	340,9643764	250,710118	310,855794	696,9591654	192,1261553	229,4772691	337,90728	210,80171	0,6238448	-0,680741	0,526278	0,8594761	conserved Plasmodium protein, unknown function
PFIT_06171	1164,897773	1998,331142	2042,669582	1777,108585	1378,818144	2397,072945	1332,875202	1337,036981	1793,1497	1334,9561	0,7444755	-0,425704	0,5264459	0,8594761	conserved Plasmodium protein, unknown function
PFIT_06201	2219,984968	2273,569389	2450,33083	2177,802019	2243,688042	7756,95138	1462,560357	1862,369242	3187,0544	1662,4648	0,5216305	-0,9389	0,5266328	0,8594761	pyridoxine biosynthesis protein PDX1 (PDX1)
PFIT_11330	12865,95062	7683,205899	8381,365641	9326,084325	6107,970957	12262,3987	6505,871933	6997,160201	9437,8294	6751,5161	0,7153675	-0,483264	0,5270472	0,8594761	TCP-1/cpn60 chaperonin family, putative
PFIT_14089	2960,357313	12215,12413	12552,72507	12473,52018	6649,550829	3522,70574	6316,147355	7515,854689	8395,6639	6916,001	0,8237587	-0,279706	0,5270644	0,8594761	plasmepsin I
PFIT_06226	424,5254274	313,2896173	271,1508295	345,9024807	229,3424969	314,9438906	387,854676	415,334892	316,52579	401,59478	1,2687585	0,3434175	0,5272509	0,8594761	ATP dependent DEAD-box helicase, putative
PFIT_07203	367,922037	504,814849	535,4449714	405,6744294	411,7112294	629,8877812	319,4097331	366,0257267	475,90922	342,71773	0,7201326	-0,473666	0,527281	0,8594761	PelOta protein homologue, putative
PFIT_09329	43,01857664	86,8839627	80,410246	76,37526775	121,579155	67,07138411	117,6772701	91,98055827	79,223099	104,82891	1,3232115	0,4040437	0,5274067	0,8594761	peptide release factor, putative
PFIT_06067	238,8663071	239,7236343	82,90358696	188,7243935	149,2107811	87,48441406	238,9569056	202,9261801	164,48552	220,94154	1,3432279	0,4257041	0,5275023	0,8594761	GTP-binding protein, putative
PFIT_11435	69,05613618	98,29937386	36,15344394	22,69120274	74,60539056	37,90991276	79,25203905	67,32597564	56,452577	73,289007	1,2982403	0,3765574	0,5275594	0,8594761	conserved Plasmodium protein, unknown function
PFIT_11232	561,5056319	539,0610825	596,531825	638,6743405	429,6717864	253,7048008	402,2641376	397,3180816	503,19158	399,79111	0,7945107	-0,331861	0,5280146	0,8594761	conserved Plasmodium protein, unknown function
PFIT_05151	5165,6254	1572,155792	1984,699405	1637,087261	1652,371243	3476,047385	1599,450243	1678,408125	2581,3311	1638,9292	0,6349163	-0,655362	0,5284348	0,8594761	RNA binding protein, putative
PFIT_13550	697,3537687	1268,379018	1326,457391	1025,531675	936,7121259	912,7540534	715,6699284	861,9621388	1027,8647	788,81603	0,7674118	-0,38189	0,528618	0,8594761	conserved protein, unknown function
PFIT_05277	0	3,805137053	3,740011442	0,553443969	0	0	7,204730823	0,948253178	1,3497654	4,076492	3,0201485	1,5946195	0,5286393	0,8594761	outer arm dynein lc3, putative
PFIT_04166	1313,198655	671,6066898	469,3714359	510,8287836	788,8829261	562,8163971	927,0086992	815,4977331	719,45081	871,25322	1,2109976	0,276196	0,5287402	0,8594761	conserved Plasmodium protein, unknown function
PFIT_08338	33,96203419	184,5491471	175,1572025	232,999911	237,6319847	1041,064527	417,8743877	334,7333718	317,5608	376,30388	1,1849821	0,2448653	0,5289398	0,8594761	Plasmodium exported protein, unknown function
PFIT_12378	280,752816	549,2081146	578,455103	461,5722703	600,9878684	1259,775562	378,2483682	422,9209174	621,79196	400,58464	0,6442422	-0,634325	0,5290082	0,8594761	calcyclin binding protein, putative
PFIT_12067	11530,11061	5436,272469	5702,894113	6748,69576	4104,678062	5981,017775	5054,118672	4712,818295	6583,9448	4883,4685	0,7417238	-0,431046	0,5290539	0,8594761	small subunit rRNA processing factor, putative
PFIT_14773	5812,036117	1438,341806	1767,155406	1826,918542	2149,740513	10471,88436	1246,418432	1661,339568	3911,0128	1453,879	0,3717398	-1,427635	0,5291227	0,8594761	Plasmodium exported protein (PHISTb), unknown function
PFIT_10024	167,5460353	62,15057186	59,21684783	73,05460393	153,355525	81,65211979	127,2835779	109,0491155	99,495951	118,16635	1,1876498	0,2481095	0,5291385	0,8594761	DnaJ protein, putative
PFIT_08052	1633,573844	1886,079599	2090,043061	2184,443346	1342,89703	1889,663344	1366,497279	1447,982603	1837,7834	1407,2399	0,7657268	-0,385098	0,5292158	0,8594761	conserved Plasmodium protein, unknown function
PFIT_12029	375,8465117	511,7909336	574,0917563	649,7432198	349,5400706	215,794888	348,2286564	377,4047648	446,13456	362,81671	0,813245	-0,298238	0,5295076	0,8594761	signal recognition particle SRP14
PFIT_10250	1085,653026	541,5978405	420,127952	521,897663	436,5796929	169,1365339	804,5282752	598,3477553	529,16545	701,43802	1,3255552	0,4065967	0,5295107	0,8594761	conserved Plasmodium protein, unknown function
PFIT_09232	57,73545812	66,58989842	43,01013158	39,84796578	40,06585789	81,65211979	21,61419247	47,4126589	54,816905	34,513426	0,6296128	-0,667463	0,529658	0,8594761	OTU-like cysteine protease, putative
PFIT_14579	2098,853713	2017,991017	2061,36964	2050,509906	1728,358215	1982,980052	1467,363511	1516,256832	1990,0104	1491,8102	0,7496494	-0,415712	0,5299386	0,8594761	conserved Plasmodium protein, unknown function
PFIT_14573	11,32067806	22,19663281	22,44006865	16,60331908	23,48688221	14,58073568	12,0078847	11,37903814	18,438053	11,693461	0,6342026	-0,656984	0,5300489	0,8594761	serine hydroxymethyltransferase, putative
PFIT_07104	3268,279757	1345,115948	1524,054663	1185,476982	1148,094066	2808,249691	1245,217644	1283,934803	1879,8785	1264,5762	0,6726904	-0,571985	0,5301609	0,8594761	regulator of chromosome condensation, putative
PFIT_06155	316,9789858	76,10274105	62,33352403	80,8028195	41,4474392	67,07138411	128,4843663	161,2030403	107,45615	144,8437	1,3479331	0,4307489	0,5301825	0,8594761	TRAP-like protein
PFIT_13373	2445,266462	2019,893585	1496,004577	1176,068435	2329,346083	3274,833233	2390,769845	2388,649755	2123,5687	2389,7098	1,1253271	0,1703446	0,5305502	0,8598258	proteasome regulatory subunit, putative
PFIT_09355	1299,613842	823,1779824	1170,623581	1367,560048	791,6460887	618,2231927	766,1030442	817,3942394	1011,8075	791,74864	0,7825092	-0,35382	0,530781	0,8598785	small nuclear ribonucleoprotein (snRNP), putative
PFIT_05175	3200,355688	1876,566756	1671,785114	1355,384281	1443,752465	2394,156798	1395,316203	1406,259463	1990,3335	1400,7878	0,7037955	-0,506772	0,5308882	0,8598785	FACT complex subunit SPT16, putative
PFIT_11132	1,132067806	21,5624433	29,92009153	21,03087083	92,56594755	43,74220703	50,43311576	37,93012712	34,992271	44,181621	1,2626108	0,3364101	0,5312318	0,8598785	conserved Plasmodium protein, unknown function
PFIT_08285	2,264135613	57,7112453	34,90677346	35,973858	26,25004483	0	12,0078847	21,80982309	26,184343	16,908854	0,645762	-0,630926	0,5312644	0,8598785	cytochrome c oxidase assembly protein, putative
PFIT_08235	33,96203419	72,297604	104,7203204	107,921574	95,32911016	26,24532422	108,0709623	103,3595964	73,412661	105,71528	1,4400143	0,5260831	0,5316491	0,8598785	conserved Plasmodium membrane protein, unknown function
PFIT_13030	3410,9203	1192,276277	1380,687557	1407,961458	2543,491186	953,5801133	2418,38798	1997,969446	1814,8195	2208,1787	1,2167484	0,2830309	0,5317033	0,8598785	conserved Plasmodium protein, unknown function
PFIT_09091	12872,74303	722,3418505	624,5819108	658,5983233	1163,29146	3003,631549	585,9847736	487,4021335	3174,198	536,69345	0,16908	-2,564222	0,5317569	0,8598785	conserved Plasmodium protein, unknown function

PFIT_05356	1969,797983	913,2328926	1299,030641	1284,543453	758,4881374	2773,255926	1020,6702	996,6140901	1499,7248	1008,6421	0,6725515	-0,572283	0,5318646	0,8598785	conserved Plasmodium protein, unknown function
PFIT_10251	2,264135613	6,976084597	17,45338673	11,62232335	37,30269528	0	16,81103859	20,86156992	12,603104	18,836304	1,4945766	0,5797368	0,531951	0,8598785	cytochrome c oxidase copper chaperone, putative (COX17)
PFIT_03189	5204,115705	1247,450764	992,9730378	987,344041	1201,975737	1198,536473	832,14641	869,5481642	1805,3993	850,84729	0,4712793	-1,085346	0,5321699	0,8599865	conserved protein, unknown function
PFIT_07290	90,5654245	72,93179351	50,49015446	64,7529444	69,07906533	99,1490026	50,43311576	49,30916526	74,494731	49,871141	0,6694586	-0,578933	0,5325679	0,8603839	tRNA pseudouridine synthase D, putative
PFIT_09107	2463,379546	2738,430299	2960,219056	3204,994026	2427,438356	3161,103495	3800,495509	3436,469517	2825,9275	3618,4825	1,2804584	0,3566604	0,5328724	0,8606299	DNA repair protein REV1, putative
PFIT_08262	12187,842	3801,966105	3825,40837	3899,012763	2998,031435	5940,191715	3254,136755	3515,174531	5442,0754	3384,6556	0,6219421	-0,685148	0,5330245	0,8606299	histone acetyltransferase GCN5
PFIT_14286	28,30169516	113,5199221	121,5503719	95,1923627	172,6976633	183,7172695	93,6615007	71,11898835	119,16321	82,390245	0,6914067	-0,532394	0,5338526	0,861721	conserved Plasmodium membrane protein, unknown function
PFIT_10325	18347,42294	16661,42677	26898,78562	27791,74236	16203,18556	23262,1057	15888,83304	17113,1251	21527,445	16500,979	0,7665089	-0,383625	0,5340867	0,8618529	S-adenosylmethionine decarboxylase/ornithine decarboxylase
PFIT_13621	316,9789858	342,4623347	311,0442849	293,3253037	328,816351	271,2016836	237,7561172	226,6325095	310,63816	232,19431	0,7474752	-0,419902	0,5346165	0,8623716	conserved Plasmodium protein, unknown function
PFIT_14564	4,528271225	3,170947544	9,350028604	12,17576732	27,63162613	8,748441406	19,21261553	11,37903814	10,93418	15,295827	1,3989002	0,484293	0,5347131	0,8623716	methyltransferase, putative
PFIT_07219	1673,196218	443,9326561	460,6447426	446,6292831	236,2504034	801,9404622	356,6341757	383,0942839	677,09896	369,86423	0,5462484	-0,872371	0,5348755	0,8623876	metallo-hydrolase/oxidoreductase, putative
PFIT_05211	13484,05964	8466,429942	8910,57726	7074,674258	10538,70221	10355,23848	6869,71084	7067,330936	9804,947	6968,5209	0,7107148	-0,492657	0,5355984	0,8632666	S-adenosyl-L-homocysteine hydrolase
PFIT_05197	4104,877866	1124,417999	1771,518753	1487,103945	1376,054981	4878,714158	1368,898856	1367,381083	2457,1146	1368,14	0,5568075	-0,844749	0,5357567	0,8632666	carbon catabolite repressor protein 4, putative
PFIT_08200	329,4317316	88,15234172	94,7469565	84,67692729	40,06585789	81,65211979	73,2480967	54,99868432	119,78766	64,123391	0,5353088	-0,901557	0,5358786	0,8632666	GTPase, putative
PFIT_11422	1,132067806	1,902568526	1,246670481	2,213775877	4,14474392	0	2,401576941	4,74126589	1,7733044	3,5714214	2,0139923	1,0100581	0,5367122	0,8641439	conserved Plasmodium protein, unknown function
PFIT_12028	7451,270301	989,9698232	1341,417437	1349,849841	1229,607363	1417,247508	896,9889874	868,5999111	2296,5604	882,79445	0,3843985	-1,379325	0,5367287	0,8641439	conserved Plasmodium protein, unknown function
PFIT_13016	10,18861026	27,90433839	46,12680778	50,91684517	23,48688221	26,24532422	25,21655788	20,86156992	30,811468	23,039064	0,7477431	-0,419385	0,5372335	0,8643607	conserved Plasmodium membrane protein, unknown function
PFIT_12337	2033,19378	247,3339084	194,480595	208,6483764	205,8556147	104,9812969	91,25992375	106,2043559	499,08226	98,73214	0,1978274	-2,337686	0,5372955	0,8643607	conserved Plasmodium protein, unknown function
PFIT_13204	14,71688148	84,98139418	57,34684211	58,11161677	59,40799619	37,90991276	37,22444258	40,77488665	52,079107	38,999665	0,7488543	-0,417243	0,5375386	0,8643607	conserved protein, unknown function
PFIT_05111	24788,88875	4038,518792	3543,660841	2975,868222	3988,625232	7581,982552	2836,262367	2953,80865	7819,5907	2895,0355	0,3702285	-1,433512	0,5376963	0,8643607	translationally-controlled tumor protein homolog (TCTP)
PFIT_12501	5107,889942	882,1576067	1343,910778	1333,246522	802,6987392	1076,058293	868,1700641	841,1005689	1757,6603	854,63532	0,4862346	-1,040275	0,5378305	0,8643607	apicoplast dimethyladenosine synthase, putative
PFIT_11101	38,49030541	55,80867677	34,28343822	209,7552643	439,3428555	172,052681	74,44888517	61,63645657	158,28887	68,042671	0,4298639	-1,218048	0,5378728	0,8643607	small nucleolar RNA snR61/Z1/Z11
PFIT_13187	3,396203419	5,07351607	8,103358124	7,748215569	11,05265045	17,49688281	18,01182706	6,637772246	8,8118044	12,3248	1,3986692	0,4840548	0,5379331	0,8643607	conserved Plasmodium protein, unknown function
PFIT_07334	12,45274587	1,268379018	1,246670481	2,213775877	2,763162613	0	1,20078847	0	3,3241223	0,6003942	0,1806174	-2,468991	0,5381697	0,8644739	rifin
PFIT_06175	3,396203419	20,29406428	25,55674485	19,92398289	45,59218312	11,66458854	26,41734635	31,29235487	21,071295	28,854851	1,3693915	0,4535349	0,5383093	0,8644739	rhomboid protease ROM10
PFIT_06204	122,2633231	138,2533129	139,0037586	98,51302652	154,7371063	169,1365339	111,6733278	83,44627967	136,98451	97,559804	0,7121959	-0,489654	0,5388962	0,8645499	conserved Plasmodium protein, unknown function
PFIT_13428	63,39579715	114,1541116	83,5269222	79,69593156	82,8948784	102,0651497	60,03942352	65,42946928	87,622132	62,734446	0,7159658	-0,482037	0,5388967	0,8645499	UDP-N-acetylglucosamine pyrophosphorylase, putative
PFIT_12137	245,658714	269,5305412	345,9510584	308,8217348	469,7376443	215,794888	455,0988303	346,11241	309,2491	400,60562	1,295414	0,3734133	0,5389358	0,8645499	phosphatidylinositol-glycan biosynthesis class O protein, putative
PFIT_09237	15,84894929	24,73339084	24,31007437	29,33253037	59,40799619	17,49688281	39,62601953	36,03362076	28,521637	37,82982	1,3263551	0,4074671	0,5390242	0,8645499	lipoate protein ligase a type 2
PFIT_09272	3,396203419	8,878653123	5,610017163	7,748215569	34,53953267	0	13,20867318	16,12030403	10,02877	14,664489	1,4622419	0,548182	0,5391208	0,8645499	conserved Plasmodium protein, unknown function
PFIT_12102	5762,225134	3419,549831	3248,823272	4094,931928	2192,569534	1504,731922	2662,148039	2670,280949	3370,4719	2666,2145	0,7910508	-0,338158	0,5396446	0,8649352	conserved Plasmodium protein, unknown function
PFIT_06240	0	12,68379018	29,29675629	24,90497861	23,48688221	2,916147135	26,41734635	23,70632945	15,548092	25,061838	1,6118915	0,6887546	0,5397674	0,8649352	sphingomyelin synthase 2, putative (SMS2)
PFIT_12364	498,1098348	268,8963517	142,74377	126,185225	363,3558836	393,6798633	323,0120986	329,9921059	298,82849	326,5021	1,092607	0,1277746	0,5398199	0,8649352	s-adenosyl-methyltransferase, putative
PFIT_02121	1588,291132	176,9388729	120,3037014	125,631781	180,9871512	256,6209479	76,85046211	101,46309	408,12893	89,156776	0,2184525	-2,194609	0,5401533	0,8652244	conserved Plasmodium protein, unknown function
PFIT_14752	3,396203419	3,805137053	11,22003433	7,748215569	38,68427659	11,66458854	21,61419247	12,32729131	12,753076	16,970742	1,3307175	0,4122044	0,5404352	0,8654308	mitochondrial ribosomal protein L20 precursor, putative
PFIT_09335	3692,805184	3559,071523	5045,275435	7271,700311	2520,004303	5657,325443	6591,127914	5596,590257	4624,3637	6093,8591	1,3177725	0,3981013	0,5418316	0,866319	mRNA processing protein, putative
PFIT_14658	867,1639396	1298,820114	990,4796968	1195,438973	1253,094245	1434,744391	1450,552472	1443,241337	1173,2902	1446,8969	1,2331961	0,3024022	0,5419347	0,866319	ATP synthase (C/AC39) subunit, putative
PFIT_07295	0	0	0	0	0	0	0	0,948253178	0	0,4741266	#DIV/0!	#DIV/0!	0,5420768	0,866319	tRNA Valine
PF_apicopla	0	0	0	0	0	0	0	0,948253178	0	0,4741266	#DIV/0!	#DIV/0!	0,5420768	0,866319	unspecified product
PFIT_bin10	0	0	0	0	0	0	1,20078847	0	0	0,6003942	#DIV/0!	#DIV/0!	0,5421226	0,866319	rifin (RIF)
PFIT_05145	0	0	0	0	0	0	1,20078847	0	0	0,6003942	#DIV/0!	#DIV/0!	0,5421226	0,866319	tRNA Phenylalanine
PFIT_11088	9303,333232	3411,939557	3747,491465	4251,003127	1909,345366	3035,709168	3004,372753	2976,566726	4276,4703	2990,4697	0,6992846	-0,516068	0,5421755	0,866319	IWS1-like protein, putative
PFIT_02124	0	21,5624433	26,18008009	33,20663815	85,65804101	43,74220703	43,22838494	43,61964619	35,058235	43,424016	1,2386253	0,3087398	0,5422152	0,866319	conserved Plasmodium protein, unknown function
PFIT_06007	400,7520034	434,4198135	266,1641476	183,1899538	319,1452818	790,2758737	193,3269437	297,7514979	398,99118	245,53922	0,6154001	-0,700403	0,5428298	0,867056	conserved Plasmodium protein, unknown function

[illegible]

PFIT_10287	168,6781031	100,8361319	104,0969851	120,0973413	189,276639	163,3042396	128,4843663	81,54977331	141,04824	105,01707	0,7445472	-0,425565	0,6350268	0,898377 conserved Plasmodium protein, unknown function
PFIT_02001	1,132067806	0,634189509	0,62333524	0	0	0	1,20078847	0,948253178	0,3982654	1,0745208	2,6980018	1,4318913	0,6352724	0,8984998 rifin (RIF)
PFIT_13684	717,7309892	1023,581867	936,2495309	845,6623849	960,1990081	676,5461354	642,4218317	750,0682638	859,99499	696,24505	0,809592	-0,304733	0,6357536	0,8987742 conserved Plasmodium protein, unknown function
PFIT_09147	124,5274587	605,0167914	541,0549886	599,3798186	466,9744816	250,7886536	582,3824082	557,5728687	431,29037	569,97764	1,3215636	0,4022458	0,6358169	0,8987742 zinc binding protein, putative
PFIT_05311	449,4309191	1383,801508	1772,765423	1752,203606	1083,159744	1186,871884	935,4142185	1209,022802	1271,3722	1072,2185	0,8433553	-0,245787	0,6359587	0,8987742 transmembrane emp24 domain-containing protein, putative
PFIT_02144	1055,087195	521,9379657	439,4513444	535,7337622	525,0008965	323,692332	623,2092162	708,345124	566,81725	665,77717	1,1745888	0,2321557	0,6361417	0,8987742 acyl-CoA synthetase (ACS9)
PFIT_12508	1641,498319	1907,642042	1728,508621	1524,184691	1371,910238	1708,862221	1283,642875	1353,157285	1647,101	1318,4001	0,8004367	-0,321141	0,6362609	0,8987742 eukaryotic translation initiation factor 2b, subunit 2, putative
PFIT_05334	46,41478006	36,148802	36,15344394	38,74107784	30,39478875	75,81982552	30,01971176	30,3441017	43,945453	30,181907	0,6868039	-0,54203	0,636815	0,8993322 sugar transporter, putative
PFIT_08283	27,16962735	44,39326561	52,98349542	53,13062104	80,13171579	46,65835417	58,83863505	63,53296293	50,744513	61,185799	1,2057619	0,269945	0,637096	0,8995044 conserved Plasmodium protein, unknown function
PFIT_14128	2695,453447	658,9228996	531,0816247	700,660065	309,4742127	320,7761849	531,9492924	411,5418793	869,39474	471,74559	0,5426138	-0,882002	0,6374943	0,8998422 conserved Plasmodium protein, unknown function
PFIT_09240	1833,949846	2586,224817	3972,515486	3815,442724	2390,135661	4522,944207	2447,206903	2637,092088	3186,8688	2542,1495	0,7976951	-0,326091	0,6382916	0,9002908 RNA binding protein, putative
PFIT_03145	3082,620637	666,5331737	1085,849989	1284,543453	471,1192256	863,1795521	934,21343	765,2403147	1242,3077	849,72687	0,6839907	-0,547951	0,6387792	0,9002908 conserved Plasmodium protein, unknown function
PFIT_11185	12782,1776	4534,454988	4186,942809	3745,708784	4864,547781	4073,857548	3814,904971	4247,225984	5697,9483	4031,0655	0,7074591	-0,499281	0,6389645	0,9002908 GTP-binding nuclear protein ran/tc4
PFIT_13236	700,7499721	1406,63233	1555,84476	1736,153731	1037,567561	947,747819	1055,493066	1068,681332	1230,7827	1062,0872	0,8629364	-0,212674	0,6389849	0,9002908 MSF1-like protein, putative
PFIT_10256	1041,502382	216,892812	275,5141762	323,211278	49,73692704	148,7235039	207,7364054	173,5303316	342,59685	190,63337	0,5564364	-0,845711	0,6390611	0,9002908 conserved Plasmodium protein, unknown function
PFIT_03010	3,396203419	5,707705579	4,363346682	3,320663815	4,14474392	0	6,003942352	5,689519068	3,4887772	5,8467307	1,6758682	0,7449087	0,6393457	0,9002908 Plasmodium exported protein (hyp13), unknown function
PFIT_12126	581,8828524	273,9698678	234,3740503	159,3918631	81,51329709	99,1490026	176,5159052	157,4100276	238,38016	166,96297	0,7004063	-0,513736	0,6394433	0,9002908 unspecified product
PFIT_04098	140,376408	87,51815221	74,80022884	76,92871172	73,22380925	239,1240651	72,04730823	78,70501378	115,32856	75,376161	0,6535776	-0,61357	0,6394477	0,9002908 conserved Plasmodium protein, unknown function
PFIT_12047	1035,842043	677,3143954	680,6820824	594,3988229	573,3562423	938,9993776	563,1697926	575,5896791	750,09883	569,37974	0,759073	-0,39769	0,6397119	0,9002908 O-phosphoseryl-tRNA(Sec) selenium transferase, putative (SEPSECS)
PFIT_05323	1939,232152	4498,940375	6270,129182	6956,790693	3824,217057	4312,981613	3655,200104	4250,070744	4633,7152	3952,6354	0,8530165	-0,229354	0,6399059	0,9002908 beta adaptin protein, putative
PFIT_11405	2281,11663	1522,054821	1480,421196	1576,208424	1447,897209	1105,219764	1269,233413	1264,969739	1568,8197	1267,1016	0,8076783	-0,308147	0,6399516	0,9002908 conserved Plasmodium protein, unknown function
PFIT_09080	161,8856963	119,2276277	86,02026316	77,48215569	169,9345007	116,6458854	134,4883087	137,4967108	121,86602	135,99251	1,1159182	0,1582313	0,6402538	0,9002908 apicoplast ribosomal protein L35 precursor, putative
PFIT_10200	452,8271225	280,9459524	199,4672769	146,6626518	154,7371063	288,6985664	187,3230014	192,4953951	253,88978	189,9092	0,7479986	-0,418893	0,6404801	0,9002908 conserved Plasmodium protein, unknown function
PFIT_12336	1061,879602	1278,52605	1605,088244	1722,871076	1446,515628	1469,738156	1107,12697	1208,074549	1430,7698	1157,6008	0,8090755	-0,305654	0,6405387	0,9002908 sentrin-specific protease 1
PFIT_13310	8000,323187	8858,993248	11378,98481	9087,549974	7790,736988	19272,81642	7619,002845	8341,783207	10731,567	7980,393	0,7436372	-0,427329	0,6407525	0,9002908 glutamine--tRNA ligase, putative
PFIT_09081	555,8452929	653,8493836	415,14127	310,4820667	491,8429452	1105,219764	423,8783301	410,5936261	588,73012	417,23598	0,708705	-0,496743	0,6407742	0,9002908 peptide deformylase
PFIT_14756	1919,986999	3429,696863	4598,344068	4510,014905	3114,084265	5030,353809	2913,112829	3156,73483	3767,0802	3034,9238	0,8056436	-0,311786	0,6408525	0,9002908 splicing factor 3A subunit 1, putative
PFIT_03006	14,71688148	3,170947544	6,856687643	1,106887938	2,763162613	0	2,401576941	1,896506356	4,7690945	2,1490416	0,4506184	-1,150022	0,6409327	0,9002908 rifin (RIF)
PFIT_10262	19088,92735	21453,99689	23408,10828	23344,26662	17283,58215	36142,7276	16510,84147	19399,36352	23453,601	17955,102	0,7655584	-0,385416	0,6412555	0,9002908 40S ribosomal protein S28, putative
PFIT_09359	71,3202718	117,3250591	93,50028604	68,07360821	71,84222795	26,24532422	55,23626964	68,27422882	74,717796	61,755249	0,8265133	-0,27489	0,6414283	0,9002908 phosphatidylinositol N-acetylglucosaminyltransferase subunit P, putative
PFIT_04159	1670,932082	1317,21161	2297,613696	2558,018026	1449,278791	4062,19296	1708,721993	1660,391315	2225,8745	1684,5567	0,7568067	-0,402003	0,6415052	0,9002908 LETM1-like protein, putative
PFIT_09089	93,96162792	183,9149575	150,8471282	115,1163456	128,4870615	177,8849753	181,319059	158,3582807	141,70202	169,83867	1,1985621	0,2613047	0,6415436	0,9002908 tRNA pseudouridine synthase, putative
PFIT_09226	9153,900282	11594,2526	6771,290715	4176,841636	10367,38613	14300,78555	5970,320275	7491,200106	9394,0762	6730,7602	0,7164898	-0,480982	0,6416126	0,9002908 phosphoglycerate kinase
PFIT_12201	3390,54308	537,7927034	508,0182208	447,1827271	350,9216519	338,2730677	381,8507336	342,3193973	928,78858	362,08507	0,3898466	-1,359022	0,641707	0,9002908 debranching enzyme-associated ribonuclease, putative
PFIT_05233	101,8861026	234,6501182	243,1007437	158,2849752	193,4213829	309,1115964	223,3466555	251,2870922	206,74249	237,31687	1,1478863	0,1989798	0,6418874	0,9002908 mitochondrial processing peptidase alpha subunit,, putative
PFIT_03013	0	0	2,493340961	1,106887938	2,763162613	0	2,401576941	1,896506356	1,0605653	2,1490416	2,0263172	1,0188601	0,6420261	0,9002908 Plasmodium exported protein, unknown function
PF_apicopla	113,2067806	45,02745512	46,75014302	44,8289615	95,32911016	40,8260599	96,06307764	54,99868432	64,328085	75,530881	1,1741509	0,2316179	0,6423411	0,9002908 apicoplast ribosomal protein S5
PFIT_13257	330,5637994	306,3135327	258,6841247	187,0640616	309,4742127	201,2141523	329,0160409	299,6480043	265,55231	314,33202	1,1836915	0,2432932	0,6424066	0,9002908 conserved Plasmodium protein, unknown function
PFIT_11318	114,3388484	39,95393905	29,92009153	34,31352609	58,02641488	34,99376562	37,22444258	35,08536759	51,924431	36,154905	0,6962985	-0,522022	0,6424281	0,9002908 RNA-binding protein s1, putative
PFIT_14610	379,2427151	598,6748963	420,7512872	309,3751788	505,6587582	411,1767461	321,8113101	366,0257267	437,47993	343,91852	0,786135	-0,34715	0,6430195	0,9007842 bax inhibitor 1, putative
PFIT_01117	1975,458322	4233,849161	4535,387208	5001,47315	3373,821551	2452,479741	2849,47104	3343,540706	3595,4115	3096,5059	0,8612382	-0,215516	0,6430988	0,9007842 Plasmodium exported protein (hyp1), unknown function
PFIT_10344	739,2402775	535,2559454	686,2920996	659,7052113	484,9350386	565,7325443	518,7406192	476,9713485	611,86019	497,85598	0,8136761	-0,297474	0,6432624	0,9007903 U2 snRNA/tRNA pseudouridine synthase, putative
PFIT_14292	103,0181704	65,95570891	62,33352403	73,05460393	84,27645971	58,32294271	86,45676987	90,08405191	74,493568	88,270411	1,18494	0,244814	0,6438237	0,9013532 conserved Plasmodium membrane protein, unknown function
PFIT_10100	20,37722051	28,5385279	7,480022884	22,13775877	16,57897568	20,41302995	25,21655788	23,70632945	19,254256	24,461444	1,2704435	0,3453322	0,6446541	0,9021786 ankyrin-repeat protein, putative
PFIT_13209	4411,668241	777,5163378	995,4663787	1190,457978	449,0139247	1673,868456	854,961391	915,0643168	1582,9986	885,01285	0,5590737	-0,83889	0,6447323	0,9021786 splicing factor, putative
PFIT_03015	3369,033791	4315,025418	4687,481007	5165,292565	3090,597383	3414,808296	3113,644504	3615,689368	4007,0397	3364,6669	0,8396889	-0,252073	0,6455162	0,9028779 Plasmodium exported protein (hyp1), unknown function
PFIT_14075	174,3384422	85,61558369	79,78691076	114,5629016	52,50008965	32,07761849	109,2717508	117,5833941	89,813591	113,42757	1,2629221	0,3367657	0,6455513	0,9028779 conserved Plasmodium protein, unknown function
PFIT_14379	48,67891567	128,1062808	142,74377	138,9144363	158,8818503	134,1427682	104,4685969	95,77357098	125,24467	100,12108	0,7994039	-0,323003	0,6461914	0,9034099 conserved Plasmodium protein, unknown function
PFIT_02026	79,24474644	107,178027	62,33352403	39,84796578	74,60539056	87,48441406	37,22444258	70,17073517	75,115678	53,697589	0,7148653	-0,484257	0,6462511	0,9034099 ERCC1 nucleotide excision repair protein, putative
PFIT_09220	2908,282194	2446,703125	3315,520143	3587,977252	1449,278791	3607,274006	2382,364325	2333,651071	2885,8393	2358,0077	0,817096	-0,291423	0,6464457	0,9034586 conserved Plasmodium protein, unknown function
PFIT_14179	33,96203419	104,6412689	118,4336957	110,6887938	227,9609156	347,0215091	105,6693854	83,44627967	157,11804	94,557833	0,6018267	-0,73258	0,6466272	0,903489 prefoldin subunit 2, putative
PFIT_08166	1670,932082	533,3533769	388,3378547	450,5033939	408,9480668	828,1857865	791,319602	783,257125	713,37676	787,28836	1,1036081	0,1422279	0,6469263	0,9035276 ABC transporter, putative
PFIT_14043	18,1130849	45,66164463	34,90677346	45,93584944	214,1451025	58,32294271	93,6615007	68,27422882	69,514233	80,967865	1,1647667	0,220041	0,6475112	0,9035276 conserved Plasmodium protein, unknown function
PFIT_11407	2121,495069	198,5013162	164,5605034	278,9357605	95,32911016	55,40679557	117,6772701	110,9456218	485,70476	114,31145	0,2353517	-2,08711	0,647637	0,9035276 carbonic anhydrase, putative
PFIT_13499	61,13166154	74,83436204	102,2269794	111,2422378	227,9609156	163,3042396	128,4843663	138,444964	123,45007	133,46467	1,0811227	0,1125302	0,6476664	0,9035276 N6-adenine-specific methylase, putative
PFIT_05234	0	8,244463614	6,856687643	2,767219846	11,05265045	2,916147135	4,803153882	0,948253178	5,3061948	2,8757035	0,5419521	-0,883763	0,6477295	0,9035276 conserved Plasmodium protein, unknown function, putative
PFIT_10058	2822,245041	6331,748056	10243,268	10669,29284	6192,247416	18336,73319	5945,103717	7134,656911	9099,2558	6539,8803	0,7187269	-0,476484	0,6477512	0,9035276 DNA/RNA-binding protein Alba 3
PFIT_12180	5992,034899	1853,101745	2176,063324	2826,438351	1718,687145	1204,368767	1952,482053	1962,884078	2628,449	1957,6831	0,7448054	-0,425065	0,6478511	0,9035276 adaptor protein subunit, putative
PFIT_06260	11576,52539	12759,25873	15664,41459	19528,27045	10026,13554	14997,74472	17082,41678	17375,79123	14092,058	17229,104	1,2226109	0,2899653	0,6480266	0,9035276 ankyrin-repeat protein, putative
PFIT_08088	113,2067806	69,12665646	70,43688215	79,69593156	78,75013448	23,32917708	64,8425774	53,10217797	72,42426	58,972378	0,8142628	-0,296434	0,6480925	0,9035276 conserved protein, unknown function
PFIT_14087	61,13166154	200,4038848	146,4837815	161,052195	42,82902051	110,8135911	99,66544305	109,0491155	120,45236	104,35728	0,8663781	-0,206931	0,649237	0,9045195 conserved Plasmodium protein, unknown function
PFIT_13364	1802,251948	936,063715	1285,940601	1008,374912	961,5805894	1971,315464	923,4063338	1039,285483	1327,5879	981,34591	0,7391947	-0,435974	0,6492546	0,9045195 1-deoxy-D-xylulose 5-phosphate synthase
PFIT_05086	2210,928426	1031,192141	1444,891087	1519,757139	1123,225602	1539,725687	1224,80424	1115,145737	1478,2867	1169,975	0,7914398	-0,337448	0,6494009	0,9045195 conserved Plasmodium protein, unknown function
PFIT_10218	0	10,14703214	4,363346682	6,64132763	8,28948784	5,832294271	7,204730823	9,48253178	5,8789148	8,3436313	1,4192469	0,5051255	0,6494438	0,9045195 zinc transporter, putative
PFIT_08038	41804,99995	31759,57641	28786,24473	33046,1394	19938,98142	23133,79523	23218,44586	25779,2109	29744,956	24498,828	0,8236297	-0,279932	0,6498497	0,9048208 Plasmodium exported protein (PHISTc), unknown function
PFIT_08182	309,0545111	130,0088493	109,7070023	125,631781	197,5661269	113,7297383	189,7245783	181,116357	164,283	185,42047	1,128665	0,1746173	0,64998	0,9048208 FAD-dependent monooxygenase, putative
PFIT_14559	2613,944565	2348,403751	1749,70202	1311,108763	1877,568996	2102,542085	1484,174549	1610,133896	2000,545	1547,1542	0,7733664	-0,370776	0,6505553	0,9050234 6-phosphogluconate dehydrogenase, decarboxylating, putative
PFIT_14182	19289,30335	14292,72896	15984,8089	17471,67266	9864,490529	11405,05145	11849,38063	12677,19674	14718,009	12263,289	0,8332165	-0,263237	0,6505755	0,9050234 NOT family protein, putative
PFIT_11321	247,9228496	227,6740337	364,0277803	423,3846364	55,26325227	61,23908984	226,9490209	181,116357	229,91861	204,03269	0,8874127	-0,172323	0,6506526	0,9050234 conserved Plasmodium protein, unknown function
PFIT_06161	4590,534954	4878,819891	6634,780298	7623,690676	4077,046436	4931,204806	4611,027727	4650,233585	5456,0128	4630,6307	0,8487206	-0,236638	0,6509795	0,9050234 alpha adaptin-like protein, putative
PFIT_13449	597,7318017	513,6935021	730,5489036	707,8548366	758,4881374	1396,834478	830,9456216	849,6348475	784,19194	840,29023	1,0715364	0,0996809	0,6510015	0,9050234 isocitrate dehydrogenase (NADP), mitochondrial precursor
PFIT_13117	52,07511909	253,041614	124,6670481	317,6768383	171,316082	14,58073568	228,1498094	219,0464841	155,55957	223,59815	1,4373795	0,5234411	0,6510856	0,9050234 conserved Plasmodium protein, unknown function
PFIT_14308	78,11267863	142,6926395	172,6638636	162,7125269	221,0530091	209,9625937	190,9253668	187,7541292	164,53288	189,33975	1,1507715	0,2026013	0,6521659	0,9058946 conserved Plasmodium protein, unknown function
PFIT_13661	1124,143332	1114,905156	1515,327969	1260,191918	649,3432141	2487,473506	1487,776915	1532,377136	1358,5642	1510,077	1,1115242	0,1525394	0,6522753	0,9058946 U1 small nuclear ribonucleoprotein, putative
PFIT_13392	862,6356684	173,1337359	153,9638044	170,4607425	134,0133867	271,2016836	152,5001357	162,1512934	294,23484	157,32571	0,5346944	-0,903214	0,6523217	0,9058946 secreted ookinete protein, putative
PFIT_13539	106,4143738	29,80690691	26,18008009	22,69120274	9,671069147	11,66458854	15,61025012	20,86156992	34,404704	18,23591	0,5300412	-0,915824	0,652353	0,9058946 conserved Plasmodium protein, unknown function
PFIT_12063	6632,785277	4940,336273	5621,860532	5930,1523	4164,086058	5298,639345	4332,444801	4485,237532	5431,3099	4408,8412	0,8117455	-0,300901	0,6529898	0,9059385 eukaryotic translation initiation factor 5, putative
PFIT_06283	67,92406838	138,2533129	169,5471854	152,1970915	399,2769976	621,1393398	148,8977703	125,1694195	258,05633	137,03359	0,531022	-0,913156	0,653395	0,9059385 RNA binding protein, putative
PFIT_14556	6538,823649	7003,988935	7414,572683	7199,752595	6311,063409	11276,74097	5586,067965	6158,904391	7624,157	5872,4862	0,7702473	-0,376606	0,653498	0,9059385 aminopeptidase P
PFIT_13538	3662,239353	1201,789119	1478,55119	1665,312903	696,3169786	533,6549258	1132,343528	1131,266041	1539,6441	1131,8048	0,7351081	-0,443972	0,6535061	0,9059385 60S ribosomal protein L7-2, putative
PFIT_11021	13,58481368	8,244463614	4,986681922	3,874107784	5,526325227	5,832294271	7,204730823	0,948253178	7,0081144	4,076492	0,5816817	-0,781698	0,6539772	0,9059385 RESA-like protein
PFIT_10106	387,1671897	405,2470961	376,4944851	301,6269632	328,816351	297,4470078	249,7640019	309,130536	349,46652	279,44727	0,7996396	-0,322578	0,6540216	0,9059385 conserved Plasmodium protein, unknown function
PFIT_05319	0	0	0,62333524	0	2,763162613	0	1,20078847	0,948253178	0,5644163	1,0745208	1,9037735	0,9288619	0,6540799	0,9059385 tRNA Glutamic acid
PFIT_05181	1006,40828	634,8236983	759,2223227	703,9807288	678,3564216	743,6175195	573,9768889	614,4680594	754,4015	594,22247	0,787674	-0,344329	0,6542865	0,9059385 conserved protein, unknown function
PFIT_06195	559,2414963	98,29937386	93,50028604	109,0284619	75,98697187	26,24532422	81,65361599	64,4812161	160,38365	73,067416	0,455579	-1,134227	0,6545078	0,9059385 conserved Plasmodium protein, unknown function
PFIT_06072	2412,436495	893,5730179	1013,543101	1084,75018	265,2636109	384,9314219	810,5322176	683,6905413	1009,083	747,11138	0,7403865	-0,43365	0,6548208	0,9059385 conserved Plasmodium protein, unknown function
PFIT_14021	29,43376296	87,51815221	165,1838387	173,7814063	163,0265942	358,6860977	202,9332515	158,3582807	162,93831	180,64577	1,1086758	0,1488376	0,6549232	0,9059385 Plasmodium exported protein, unknown function
PFIT_11031	31,69789858	12,04960067	13,09004005	17,15676305	1,381581307	5,832294271	7,204730823	11,37903814	13,534696	9,2918845	0,6865233	-0,542619	0,6551796	0,9059385 Plasmodium exported protein, unknown function
PFIT_04201	0	0	0,62333524	2,213775877	1,381581307	0	0	2,844759534	0,7031154	1,4223798	2,0229677	1,0164733	0,6554912	0,9059385 conserved Plasmodium protein, unknown function
PFIT_13279	0	31,70947544	62,95685927	128,9524448	20,7237196	69,98753125	30,01971176	43,61964619	52,388338	36,819679	0,702822	-0,508769	0,6555034	0,9059385 conserved Plasmodium protein, unknown function
PFIT_13025	320,3751892	214,9902435	258,6841247	236,3205748	164,4081755	256,6209479	201,732463	184,9093697	241,89988	193,32092	0,7991774	-0,323412	0,6556969	0,9059385 conserved Plasmodium protein, unknown function
PFIT_05368	11827,84444	4697,441692	5175,5525	4797,805769	3383,49262	11043,4492	4663,862419	5025,741843	6820,931	4844,8021	0,7102864	-0,493531	0,6557799	0,9059385 erythrocyte membrane protein 1 (PfEMP1), pseudogene (VAR1CSA)
PFIT_06064	249,0549174	324,7050285	243,724079	220,8241437	229,3424969	177,8849753	286,9884444	298,6997511	240,92261	292,8441	1,2155111	0,281563	0,6561099	0,9059385 para-hydroxybenzoate--polyprenyltransferase, putative
PFIT_11402	21,50928832	10,14703214	8,726693364	6,087883661	4,14474392	0	6,003942352	4,74126589	8,4359402	5,3726041	0,6368708	-0,650927	0,6563023	0,9059385 AAA family ATPase, putative
PFIT_05174	10769,36104	19312,97311	23111,4007	23297,77733	15686,47416	24113,62066	15440,93894	16450,29613	19381,935	15945,618	0,8227052	-0,281553	0,6567471	0,9059385 pre-mRNA-splicing factor
PFIT_13349	417,7330205	313,2896173	291,7208925	246,8360103	581,6457301	411,1767461	437,0870032	384,0425371	377,067	410,56477	1,0888377	0,1227889	0,6567646	0,9059385 conserved Plasmodium protein, unknown function
PFIT_02119	39950,67288	7624,226275	9570,689279	11320,69639	5187,837806	6418,439845	7646,62098	7627,748564	13345,427	7637,1848	0,5722698	-0,805233	0,6567694	0,9059385 conserved Plasmodium protein, unknown function
PFIT_01088	173,2063744	83,07882565	67,32020595	66,96672027	67,69748403	23,32917708	66,04336588	58,79169704	80,266465	62,417531	0,777629	-0,362846	0,6569379	0,9059385 Plasmodium RNA of unknown function RUF5
PFIT_08348	62145,99429	17487,14152	13446,5878	6845,548455	11353,83518	52925,15436	13133,0235	14792,74958	27367,377	13962,887	0,5102019	-0,97086	0,6569601	0,9059385 histidine-rich protein II
PFIT_12399	243,3945784	223,2347071	201,9606179	164,9263028	81,51329709	58,32294271	144,0946165	137,4967108	162,22541	140,79566	0,8679014	-0,204397	0,6569958	0,9059385 erythrocyte membrane protein 1 (PfEMP1), pseudogene
PFIT_03169	1853,194999	1601,32851	1722,275269	1989,631069	1105,265045	988,5738789	1247,619221	1381,60488	1543,3781	1314,6121	0,8517757	-0,231454	0,6571707	0,9059385 RING zinc finger protein, putative
PFIT_10379	294,3376296	256,2125615	628,3219222	889,9379025	120,1975737	262,4532422	404,6657145	394,9067384	408,57681	349,78623	0,8561089	-0,224134	0,6572052	0,9059385 Plasmodium exported protein, unknown function
PFIT_11261	907,9183806	96,39680533	90,38360984	70,84082806	93,94752885	102,0651497	60,03942352	75,86025424	226,92538	67,949839	0,2994369	-1,739676	0,6575034	0,9059385 Plasmodium exported protein, unknown function
PFIT_04154	2825,641244	1933,643812	2211,593433	1882,262939	2818,425866	3928,050191	1809,588225	1988,486914	2599,9362	1899,0376	0,730417	-0,453208	0,6575314	0,9059385 small GTP-binding protein sar1
PFIT_12168	48,67891567	87,51815221	60,46351831	69,18049615	27,63162613	37,90991276	42,02759647	50,25741843	55,230437	46,142507	0,8354543	-0,259367	0,6577306	0,9059385 type IIB DNA topoisomerase, putative
PFIT_12123	10015,40388	7417,480495	10692,69271	13005,37983	3383,49262	6931,681741	6688,39178	8067,738039	8574,3552	7378,0649	0,8604804	-0,216786	0,6578792	0,9059385 eukaryotic translation initiation factor 3 subunit 10, putative
PFIT_11028	70,18820399	1,268379018	1,870005721	2,767219846	0	0	0	0,948253178	12,68231	0,4741266	0,0373849	-4,7414	0,6579045	0,9059385 Plasmodium exported protein, unknown function, pseudogene
[illegible]														

PFIT_11463	441,5064444	120,4960067	119,6803661	101,2802464	138,1581307	78,73597266	122,480424	110,9456218	166,64286	116,71302	0,7003782	-0,513794	0,7382194	0,9277825	conserved protein, unknown function
PFIT_02112	7335,799385	3978,904978	3633,421116	2990,257766	3494,019125	7622,808612	3513,507065	3821,460307	4842,5352	3667,4837	0,7573479	-0,400972	0,7382283	0,9277825	asparagine--tRNA ligase, putative
PFIT_12104	0	1,268379018	1,246670481	2,767219846	5,526325227	0	2,401576941	2,844759534	1,8014324	2,6231682	1,4561569	0,5421658	0,7385922	0,9277825	small nucleolar RNA snoR14
PFIT_03173	14908,20094	12261,41996	12405,61795	15152,74243	9241,39736	12037,85537	11108,49414	10850,86112	12667,872	10979,678	0,8667342	-0,206339	0,7386597	0,9277825	conserved Plasmodium protein, unknown function
PFIT_05032	106,4143738	95,56775288	94,74695652	65,30638837	161,6450129	227,4594766	94,86228917	86,2910392	125,85666	90,576664	0,7196811	-0,47457	0,7386684	0,9277825	4-diphosphocytidyl-2c-methyl-D-erythritol kinase (CMK), putative
PFIT_08354	1,132067806	6,976084597	13,09004005	18,81709495	6,907906533	20,41302995	10,8070623	5,689519068	11,222704	8,2483077	0,7349662	-0,44425	0,7388371	0,9277825	stevor
PFIT_14003	1,132067806	3,805137053	3,116676201	1,106887938	1,381581307	8,748441406	1,20078847	0,948253178	3,215132	1,0745208	0,3342074	-1,581185	0,7393002	0,9277825	rifin
PFIT_14295	2083,004764	2219,029091	2469,654222	2083,716544	1539,081576	2399,989092	1682,304647	1931,591724	2132,4125	1806,9482	0,8473727	-0,238931	0,739415	0,9277825	proliferation-associated protein 2g4, putative
PFIT_02097	31,69789858	22,19663281	33,03676774	36,52730197	63,55274011	58,32294271	49,23232729	14,22379767	40,889047	31,728062	0,775955	-0,365955	0,7395102	0,9277825	50S ribosomal protein L33, putative
PFIT_14156	1179,616654	1929,204486	2258,343576	2275,208157	1733,88454	2729,513719	1696,714109	1717,286505	2017,6282	1707,0003	0,8460431	-0,241197	0,7397587	0,9277825	RNA guanylyltransferase
PFIT_07063	638,4862427	714,7315764	578,455103	584,4368315	542,9614535	1146,045824	543,9571771	564,2106409	700,85284	554,08391	0,7905852	-0,339007	0,7398637	0,9277825	AAA family ATPase, putative
PFIT_08312	3510,542267	472,471184	404,5445709	495,3323524	594,0799619	504,4934544	493,5240614	489,2986399	996,91063	491,41135	0,4929342	-1,020533	0,7401057	0,9277825	conserved Plasmodium protein, unknown function
PFIT_14205	45,28271225	27,90433839	13,09004005	17,71020701	44,21060181	8,748441406	25,21655788	32,24060805	26,157723	28,728583	1,098283	0,1352498	0,7401206	0,9277825	conserved Plasmodium protein, unknown function
PFIT_13130	8484,848208	9880,038357	9280,838393	10137,98663	7291,986136	8730,944523	7936,011001	7700,764059	8967,7737	7818,3875	0,8718315	-0,197879	0,7401429	0,9277825	conserved Plasmodium membrane protein, unknown function
PFIT_10062	237,7342393	289,8246055	170,7938558	167,1400787	183,7503138	207,0464466	248,5632134	223,78775	209,38159	236,17548	1,1279668	0,1737246	0,7402397	0,9277825	phosducin-like protein, putative (PhLP1)
PFIT_08288	213,9608154	52,63772923	84,15025744	75,26837981	38,68427659	29,16147135	58,83863505	62,58470975	82,310488	60,711672	0,7375934	-0,439102	0,7403379	0,9277825	conserved Plasmodium protein, unknown function
PFIT_03046	269,4321379	43,1248866	44,25680206	35,42041403	19,34213829	43,74220703	50,43311576	27,49934216	75,886431	38,966229	0,5134808	-0,961618	0,7405727	0,9277825	conserved Plasmodium protein, unknown function
PFIT_11484	179,9987812	171,2311674	135,8870824	67,52016424	98,09227277	180,8011224	109,2717508	110,9456218	138,92177	110,10869	0,7925949	-0,335344	0,7406185	0,9277825	merozoite adhesive erythrocytic binding protein
PFIT_14583	743,7685487	703,9503547	458,7747369	415,0829769	623,0931693	1606,797072	756,4967364	703,6038581	758,57781	730,0503	0,9623934	-0,055301	0,7411044	0,9281857	conserved Plasmodium protein, unknown function
PFIT_03085	2264,135613	2475,875842	2685,328215	2398,626163	1650,989661	2347,498444	2477,226615	2767,002773	2303,7423	2622,1147	1,1381979	0,1867514	0,7415343	0,9281947	pre-mRNA-processing factor 19, putative
PFIT_12489	8845,977838	4953,654253	6043,235155	7310,441389	2910,991813	4423,795204	4492,149668	5522,626509	5748,0159	5007,3881	0,8711507	-0,199006	0,7415821	0,9281947	conserved Plasmodium protein, unknown function
PFIT_12485	93,96162792	55,17448726	76,04689932	58,11161677	58,02641488	52,49064844	73,2480967	75,86025424	65,635282	74,554175	1,1358857	0,1838176	0,7416039	0,9281947	conserved Plasmodium membrane protein, unknown function
PFIT_03170	1259,991468	825,0805509	872,6693364	654,1707716	1090,067651	1020,651497	791,319602	741,5339852	953,77188	766,42679	0,8035745	-0,315496	0,7419205	0,9282471	proteasome component C8, putative
PFIT_12078	5395,435165	1211,301962	1226,723753	1763,272486	327,4347697	221,6271823	1155,158509	977,6490265	1690,9659	1066,4038	0,6306477	-0,665094	0,7421201	0,9282471	cysteine repeat modular protein 3
PFIT_04196	0	0	1,870005721	0	5,526325227	5,832294271	0	3,793012712	2,2047709	1,8965064	0,860183	-0,217285	0,7421939	0,9282471	unspecified product
PFIT_07027	107,5464416	82,44463614	122,1737071	188,1709495	64,93432141	58,32294271	87,65755834	101,46309	103,93217	94,560324	0,9098273	-0,136335	0,7423022	0,9282471	conserved Plasmodium protein, unknown function
PFIT_14614	81,50888205	66,58989842	49,86681922	93,53203079	74,60539056	43,74220703	81,65361599	78,70501378	68,307538	80,179315	1,1737989	0,2311853	0,7431422	0,9286076	conserved Plasmodium protein, unknown function
PFIT_09197	35,09410199	11,41541116	17,45338673	13,28265526	41,4474392	5,832294271	8,405519293	19,91331674	20,754215	14,159418	0,682243	-0,551642	0,7435466	0,9286076	dolichyl-diphosphooligosaccharide-protein glycosyltransferase, putative
PFIT_14426	744,9006165	914,5012717	587,8051316	425,5984123	772,3039504	1274,356298	585,9847736	622,0540848	786,57761	604,01943	0,7679082	-0,380994	0,7436438	0,9286076	FACT complex subunit SSRP1, putative
PFIT_bin05	0	0,634189509	0,6233524	0,553443969	0	2,916147135	2,401576941	0	0,7878526	1,2007885	1,5241283	0,6079843	0,7438814	0,9286076	stevor
PFIT_11361	838,8622445	893,5730179	1194,31032	990,6647049	1091,449232	1612,629366	904,1937183	885,6684683	1103,5815	894,93109	0,8109334	-0,302345	0,743961	0,9286076	conserved Plasmodium protein, unknown function
PFIT_11237	3033,941721	3556,534765	3205,813141	3698,112602	2455,069982	2694,519953	3628,782758	3604,31033	3107,332	3616,5465	1,1638752	0,2189363	0,744065	0,9286076	calcium-dependent protein kinase 6
PFIT_14489	224,1494256	114,1541116	56,10017163	47,04273738	134,0133867	218,7110352	85,2559814	94,8253178	132,36181	90,04065	0,6802615	-0,555839	0,7440922	0,9286076	conserved Plasmodium protein, unknown function
PFIT_11260	23,77342393	40,58812856	29,92009153	31,54630624	92,56594755	37,90991276	52,8346927	39,82663348	42,717302	46,330663	1,0845878	0,1171468	0,7441486	0,9286076	vacuolar membrane protein-related, putative
PFIT_05318	559,2414963	528,2798608	759,8456579	730,5460393	607,8957749	825,2696393	787,7172366	704,5521113	668,51308	746,13467	1,1161108	0,1584803	0,7442508	0,9286076	conserved Plasmodium protein, unknown function
PFIT_09092	1778,478524	660,8254681	711,8488444	627,0520171	266,6451922	332,4407734	671,240755	491,1951462	729,54847	581,21795	0,7966817	-0,327925	0,7443157	0,9286076	conserved Plasmodium protein, unknown function
PFIT_04138	283,0169516	176,3046834	209,4406407	184,8502857	245,9214726	160,3880924	267,7758289	204,8226865	209,98702	236,29926	1,1253041	0,1703149	0,7444	0,9286076	conserved Plasmodium protein, unknown function
PFIT_02019	122,2633231	54,54029776	57,97017735	38,74107784	42,82902051	104,9812969	63,64178893	77,7567606	70,220866	70,699275	1,0068129	0,0097956	0,7445607	0,9286076	conserved Plasmodium protein, unknown function
PFIT_12335	43,01857664	65,3215194	58,59351259	61,98572455	185,1318951	52,49064844	73,2480967	91,98055827	77,756979	82,614327	1,0624683	0,0874198	0,7448007	0,9287021	conserved Plasmodium protein, unknown function
PFIT_08353	1,132067806	5,707705579	2,49334096	2,213775877	2,763162613	0	2,401576941	4,74126589	2,3850088	3,5714214	1,4974458	0,5825038	0,7450396	0,9287953	Plasmodium exported protein (PHISTa-like), unknown function
PFIT_14535	22,64135613	32,34366495	51,1134897	42,06174166	80,13171579	32,07761849	62,44100046	38,8783803	43,394931	50,65969	1,1674103	0,2233117	0,7452511	0,9288542	DER1-like protein
PFIT_05242	1,132067806	2,536758035	4,363346682	2,767219846	2,763162613	2,916147135	1,20078847	0,948253178	2,7464504	1,0745208	0,3912399	-1,353875	0,7454885	0,9289454	conserved Plasmodium protein, unknown function, putative
PFIT_07299	1618,856963	1078,756354	989,8563636	1056,524537	678,3564216	574,4809857	1265,631048	1078,163863	999,47194	1171,8975	1,1725166	0,2296084	0,7461361	0,9295475	unspecified product
PFIT_14574	11,32067806	28,5385279	29,29675629	26,56531052	38,68427659	40,8260599	33,62207717	31,29235487	29,205268	32,457216	1,111348	0,1523106	0,7466795	0,9300197	SNARE protein, putative
PFIT_09027	3556,957047	4167,893452	3553,634205	4215,582713	2470,267376	1148,961971	2763,014271	3143,459285	3185,5495	2953,2368	0,927073	-0,109245	0,7468938	0,9300817	serine/threonine protein kinase, FIKK family
PFIT_12524	242,2625105	1083,195681	362,7811099	1017,230015	581,6457301	192,4657109	515,1382538	542,4008178	579,93013	528,76954	0,9117815	-0,13324	0,7471665	0,9302166	chitinase
PFIT_10071	338,4882741	247,3339084	385,2211785	512,4891155	216,9082651	364,5183919	271,3781943	326,1990932	344,15986	298,78864	0,8681682	-0,203954	0,7482814	0,9313995	conserved Plasmodium protein, unknown function
PFIT_06187	4933,5515	1676,162872	1123,250103	1172,194327	1052,764956	839,850375	1271,63499	1248,849435	1799,629	1260,2422	0,7002789	-0,513998	0,7485222	0,9314943	conserved Plasmodium membrane protein, unknown function
PFIT_12213	163,0177641	35,51461249	25,55674485	28,7790864	55,26325227	20,41302995	34,82286564	32,24060805	54,757415	33,531737	0,6123689	-0,707527	0,7490912	0,9319973	conserved Plasmodium protein, unknown function
PFIT_07116	700,7499721	790,2001279	808,4658067	954,6908469	410,3296481	571,5648385	691,654159	603,0890212	706,00021	647,37159	0,9169567	-0,125075	0,7496067	0,9323915	conserved Plasmodium protein, unknown function
PFIT_13028	65,65993276	166,7918408	140,2504291	109,5819059	143,6844559	67,07138411	124,8820009	147,9274958	115,50666	136,40475	1,1809254	0,2399179	0,7497377	0,9323915	conserved Plasmodium protein, unknown function
PFIT_03146	1334,707944	98,29937386	50,49015446	61,98572455	102,2370167	72,90367838	260,5710981	314,8200551	286,77065	287,69558	1,0032253	0,0046457	0,7503804	0,9327702	zinc finger protein, putative
PFIT_06011	224,1494256	452,8113093	407,0379119	396,8193259	287,3689118	577,3971328	320,6105216	335,681625	390,93067	328,14607	0,8393971	-0,252575	0,7505848	0,9327702	MYND finger protein, putative
PFIT_13444	251,319053	303,1425852	429,4779806	392,9452181	384,0796032	457,8351003	481,5161767	351,801929	369,79992	416,65905	1,1267148	0,1721224	0,7507463	0,9327702	rhomboid protease ROM6, putative
PFIT_06043	0	5,07351607	6,856687643	4,980995723	2,763162613	2,916147135	1,20078847	2,844759534	3,7650849	2,022774	0,5372453	-0,896347	0,7510543	0,9327702	calcium-binding protein, putative
PFIT_05372	2,264135613	4,439326561	8,103358124	4,980995723	4,14474392	2,916147135	2,401576941	2,844759534	4,4747845	2,6231682	0,5862111	-0,770508	0,7510567	0,9327702	rifin (RIF)
PFIT_14622	99,62196695	55,80867677	57,34684211	55,34439692	58,02641488	11,66458854	49,23232729	47,4126589	56,302148	48,322493	0,8582709	-0,220495	0,7513865	0,9327702	conserved Plasmodium protein, unknown function
PFIT_04202	118,8671197	167,4260303	144,6137757	101,2802464	117,4344111	259,5370951	102,06702	131,8071917	151,52645	116,93711	0,7717274	-0,373837	0,7518091	0,9327702	steroid dehydrogenase, putative
PFIT_14727	143,7726114	92,59166828	46,75014302	37,63418991	58,02641488	17,49688281	76,85046211	74,91200106	66,045318	75,881232	1,1489267	0,2002868	0,7518347	0,9327702	conserved Plasmodium protein, unknown function
PFIT_12054	9527,482658	7841,119087	10549,94894	13497,39152	6398,103031	8868,003439	11119,30124	10884,99823	9447,0081	11002,15	1,1646174	0,219856	0,7520696	0,9327702	high mobility group protein B3, putative (HMGB3)
PFIT_09291	286,413155	334,8520606	388,9611899	340,3680411	258,3557043	393,6798633	270,1774059	296,8032447	333,77167	283,49033	0,8493541	-0,235562	0,7521983	0,9327702	guanylate kinase
PFIT_03200	26,03755954	59,61381383	101,6036442	103,4940222	265,2636109	209,9625937	139,2914626	115,6868877	127,66254	127,48918	0,998642	-0,001961	0,7521985	0,9327702	conserved Plasmodium protein, unknown function
PFIT_bin00	0	1,268379018	4,986681922	5,534439692	2,763162613	8,748441406	7,204730823	2,844759534	3,8835174	5,0247452	1,2938645	0,3716865	0,7522262	0,9327702	stevor
PFIT_14148	2123,759205	2038,285081	2409,814039	2483,30309	1641,318592	1945,070139	1849,214245	1861,420988	2106,925	1855,3176	0,8805807	-0,183473	0,7523047	0,9327702	diphthamide synthesis protein, putative
PFIT_02122	499,2419026	530,1824293	624,5819108	675,7550864	1065,199187	796,108168	620,8076392	524,3840074	698,51145	572,59582	0,8197372	-0,286767	0,7523511	0,9327702	multidrug efflux pump, putative
PFIT_13155	6,792406838	14,5863587	11,84336957	7,748215569	6,907906533	2,916147135	4,803153882	7,586025424	8,4657341	6,1945897	0,731725	-0,450626	0,7527572	0,9330692	conserved Plasmodium protein, unknown function
PFIT_03051	2675,076226	2336,98834	2161,103278	1981,882854	2300,332876	2286,259354	2677,758289	2363,995173	2290,2738	2520,8767	1,1006879	0,1384055	0,7536498	0,9330944	conserved Plasmodium protein, unknown function
PFIT_01114	13475,0031	2916,003361	2555,674485	3105,927555	2037,832427	26805,22447	2688,565385	3981,715094	8482,6109	3335,1402	0,3931738	-1,346761	0,7538413	0,9330944	glutamic acid-rich protein
PFIT_14341	53,2071869	202,3064533	424,4912986	386,3038905	449,0139247	661,9653997	378,2483682	378,353018	362,88136	378,30069	1,0424914	0,0600355	0,753843	0,9330944	SF-assemblin, putative
PFIT_11477	0	3,170947544	6,233352403	6,087883661	6,907906533	0	1,20078847	2,844759534	3,7333484	2,022774	0,5418123	-0,884135	0,753885	0,9330944	conserved Plasmodium protein, unknown function
PFIT_04040	346,4127487	180,1098205	263,0474714	296,0925235	179,6055699	300,3631549	303,799483	274,0451684	260,93855	288,92233	1,1072428	0,1469716	0,7538903	0,9330944	liver specific protein 2, putative (LISP2)
PFIT_10088	163,0177641	319,6315124	415,7646053	503,6334012	344,0137454	486,9965716	312,2050023	328,0955996	372,17637	320,1503	0,8602113	-0,217237	0,7539176	0,9330944	conserved Plasmodium protein, unknown function
PFIT_05188	252,4511208	241,6262028	309,7976144	258,4583386	262,5004483	300,3631549	286,9884444	309,130536	270,86615	298,05949	1,100394	0,1380202	0,7539323	0,9330944	mRNA-binding protein PUF1
PFIT_13197	1284,89696	158,5473772	129,0089497	137,8075483	219,6714278	469,4996888	223,3466555	183,9611165	329,8389	203,65389	0,6174507	-0,695552	0,7544227	0,9334321	SNARE protein, putative
PFIT_12409	23,77342393	13,31797968	17,45338673	13,83609923	0	8,748441406	10,8070623	9,48253178	12,854888	10,144814	0,7891795	-0,341575	0,7545353	0,9334321	rifin, pseudogene
PFIT_bin05	0	0,634189509	0	0	1,381581307	0	0	0,948253178	0,3359618	0,4741266	1,4112515	0,4969751	0,7552847	0,9337385	Plasmodium exported protein (PHISTa), unknown, putative
PFIT_08044	2,264135613	8,878653123	6,233352403	5,534439692	5,526325227	0	7,204730823	6,637772246	4,7394843	6,9212515	1,4603385	0,5463028	0,7555568	0,9337385	conserved Plasmodium protein, unknown function
PFIT_14221	1308,670384	2591,932522	1942,935944	3149,649629	1504,542043	594,8940156	1787,974033	1800,732785	1848,7708	1794,3534	0,9705657	-0,043102	0,7555632	0,9337385	conserved Plasmodium protein, unknown function
PFIT_14351	3227,525316	478,8310791	424,4912986	489,2444688	247,3030539	139,9750625	421,4767531	356,5431949	834,55871	389,00997	0,4661266	-1,101206	0,7559024	0,9337385	protein kinase, putative
PFIT_08278	26025,1068	3186,802282	3488,80734	3959,8916	1338,752286	1513,480363	2962,345157	2844,759534	6585,4734	2903,5523	0,4409026	-1,181468	0,755978	0,9337385	conserved Plasmodium protein, unknown function
PFIT_10033	235,2810872	332,9484921	413,2712643	287,23742	216,1450325	556,3833231	264,1734635	302,4922538	348,31141	283,33261	0,8134559	-0,297879	0,7560521	0,9337385	conserved Plasmodium protein, unknown function
PFIT_09122	6181,090222	1381,26475	2321,92377	3208,861833	721,1854421	807,7727565	2109,785343	1705,907467	2437,0175	1907,8464	0,7828612	-0,353172	0,756143	0,9337385	conserved Plasmodium protein, unknown function
PFIT_07152	357,7334268	192,1594212	183,8838959	187,0640616	268,0267735	189,5495638	262,972675	232,3220286	229,73619	247,64735	1,077964	0,1083091	0,7562522	0,9337385	conserved Plasmodium protein, unknown function
PFIT_03025	143,7726114	256,2125615	386,467849	317,1233943	250,0662165	78,73597266	236,5553287	218,0982309	238,72977	227,32678	0,9522347	-0,070611	0,7563769	0,9337385	ABC transporter, (TAP family), putative
PFIT_05129	1923,383203	3871,726951	6391,679554	13775,77384	3623,887767	2519,551125	5086,539961	4478,59976	5351,0004	4782,5699	0,8937712	-0,162023	0,7565464	0,9337385	conserved Plasmodium protein, unknown function
PFIT_02079	950,9369573	245,4313399	280,5008581	347,0093687	497,3692704	373,2668333	369,8428489	323,3543337	449,08577	346,59859	0,7717871	-0,373725	0,7566257	0,9337385	ribosome-recycling factor, putative
PFIT_14063	362,2616598	487,0575427	349,0674736	342,028373	375,7901154	335,3569206	434,6854261	413,4383856	375,2604	424,06191	1,1300471	0,1763828	0,756764	0,9337385	conserved Plasmodium protein, unknown function
PFIT_12400	143,7726114	62,78476137	48,62041874	51,47028934	40,06595189	84,56826693	58,79160704	71,880323	55,212801	61,782312	1,1190066	0,1622389	0,7575459	0,9340696	C_iTvarS - erythrocyte membrane protein 1, PfEMP1, putative
PFIT_11181	607,920412	394,4658745	417,0112758	468,7670419	388,2243472	104,9812969	350,6302334	365,0774735	396,89504	357,85385	0,9016335	-0,149387	0,757642	0,9340696	conserved Plasmodium protein, unknown function
PFIT_13655	11,32067806	64,6873299	153,9638044	147,2160958	345,3953267	104,9812969	157,3032896	148,8757489	137,92742	153,08952	1,1099281	0,1504662	0,7576441	0,9340696	nucleoside diphosphate kinase
PFIT_08157	14,71688148	6,976084597	3,116676201	3,320663815	6,907906533	2,916147135	8,405519293	6,637772246	6,3257266	7,5216458	1,1890564	0,2498172	0,7577343	0,9340696	conserved Plasmodium protein, unknown function
PFIT_08296	5,660339031	74,83436204	42,38679634	22,13775877	63,55274011	37,90991276	20,413404	43,61964619	41,080318	32,016525	0,7793641	-0,359631	0,7579297	0,9340696	small nucleolar RNA snoR05
PFIT_14291	1481,876758	1372,386097	1214,880383	991,7715928	2231,25381	2545,796449	1350,887029	1163,506649	1639,6608	1257,1968	0,766742	-0,383187	0,758074	0,9340696	conserved protein, unknown function
PFIT_07083	498,1098348	112,8857326	116,6936842	99,61991446	429,6717864	90,4005612	267,7758289	197,236661	224,23025	232,50624	1,0369085	0,0522885	0,7581884	0,9340696	Cg7 protein
PFIT_14354	3238,845994	1740,850202	2122,456493	1814,183531	2382,808251	2201,691087	2293,505979	2180,982309	2122,889	2237,2441	1,0538677	0,0756938	0,7585319	0,9340945	calmodulin
PFIT_13669	0	10,14703214	6,856687643	5,534439692	12,43423176	0	10,8070623	5,689519068	5,8287319	8,2483077	1,4151119	0,5009161	0,7585389	0,9340945	conserved Plasmodium protein, unknown function
PFIT_11076	56364,52401	3260,368265	3596,644336	4434,193081	1550,134226	1965,483169	3081,223215	3027,772397	11861,891	3054,4978	0,2575051	-1,957327	0,759169	0,934203	protein kinase, putative
PFIT_09265	489,0532923	667,1673632	705,615492	616,5365817	768,1592065	536,5710729	720,4730823	701,7073517	630,51717	711,09022	1,1277888	0,173497	0,7592462	0,934203	monocarboxylate transporter, putative
PFIT_01108	170,9422387	166,7918408	148,9771224	162,159083	200,3292895	440,3382174	169,3111743	150,7722553	214,92297	160,04171	0,7446469	-0,425372	0,7594653	0,934203	multidrug resistance-associated protein 1
PFIT_10080	2026,401373	2701,647307	2831,811997	2908,901502	2805,991634	2767,423631	3094,431888	2911,137257	2673,6962	3002,7846	1,1230837	0,1674654	0,7595126	0,934203	conserved Plasmodium membrane protein, unknown function
PFIT_01136	6,792406838	4,439326561	3,116676201	4,427551754	2,763162613	2,916147135	0	3,793012712	4,0758785	1,8965064	0,4653	-1,103767	0,7596151	0,934203	Plasmodium exported protein (hyp7), unknown function
PFIT_06110	0	10,14703214	8,726693373	8,855103507	12,43423176	8,748441406	14,40946165	6,637772246	8,151917	10,523617	1,2909377	0,3684194	0,7597629	0,934203	conserved Plasmodium protein, unknown function
PFIT_12303	220,7532222	360,219641	316,0309668	330,4060496	303,9478875	300,3631549	248,5632134	279,7346875	305,28682	264,14895	0,8652485	-0,208814	0,7599906	0,934203	sun-family protein, putative
PFIT_05143	4469,403699	661,4596577	478,7214645	416,1898648	440,7244368	489,9127187	563,1697926	480,7643613	1159,402	521,96708	0,4502037	-1,15135	0,7603151	0,934203	conserved Plasmodium protein, unknown function
PFIT_03009	5,660339031	5,07351607	6,233352403	3,320663815	5,526325227	0	3,602365411	7,586025424	4,3023661	5,5941954	1,3002602	0,3788004	0,7605385	0,934203	acyl-CoA synthetase, pseudogene
PFIT_14101	367,9220237	340,5597662	843,3725801	1892,778384	490,4613639	408,260599	667,6383896	601,1925149	723,89245	634,41545	0,8763946	-0,190347	0,7607424	0,934203	aldo-keto reductase, putative
PFIT_14268	1077,728552	247,3339084	241,8540732	306,054515	328,816351	224,5433294	492,3232729	392,5708157	404,38845	442,45004	1,0941214	0,1297728	0,7607806	0,934203	conserved Plasmodium protein, unknown function

[illegible]	0,8506272	0,9621468	erythrocyte membrane protein 1, PfEMP1, putative
[illegible]	0,8506753	0,9621468	conserved Plasmodium protein, unknown function
[illegible]	0,8506882	0,9621468	parasite-infected erythrocyte surface protein
[illegible]	0,8507243	0,9621468	unspecified product
[illegible]	0,8511083	0,9623887	stevor
[illegible]	0,851808	0,9629874	conserved Plasmodium protein, unknown function
[illegible]	0,8521946	0,9632319	GTP-binding protein, putative
[illegible]	0,8528148	0,9636674	3-demethylubiquinone-9 3-methyltransferase, putative
[illegible]	0,8529206	0,9636674	Plasmodium exported protein, unknown function
[illegible]	0,8543014	0,9647737	serine/threonine protein kinase, putative
[illegible]	0,854361	0,9647737	unspecified product
[illegible]	0,8544115	0,9647737	conserved Plasmodium protein, unknown function
[illegible]	0,8547279	0,9649383	rifin (RIF)
[illegible]	0,8549555	0,9650027	conserved Plasmodium protein, unknown function
[illegible]	0,8551842	0,9650682	mitochondrial inner membrane protein, putative
[illegible]	0,8567951	0,9666932	protein phosphatase, putative
[illegible]	0,8574297	0,9668966	unspecified product
[illegible]	0,8574377	0,9668966	BolA-like protein, putative
[illegible]	0,8574882	0,9668966	conserved Plasmodium membrane protein, unknown function
[illegible]	0,8576695	0,9669082	mRNA methyltransferase, putative
[illegible]	0,8580113	0,9671009	conserved protein, unknown function
[illegible]	0,8584306	0,9671353	conserved Plasmodium protein, unknown function
[illegible]	0,8585411	0,9671353	rifin, pseudogene
[illegible]	0,8585549	0,9671353	conserved Plasmodium protein, unknown function
[illegible]	0,8588312	0,9671365	probable protein, unknown function
[illegible]	0,8588979	0,9671365	conserved Plasmodium protein, unknown function
[illegible]	0,859573	0,967704	conserved Plasmodium protein, unknown function
[illegible]	0,8598386	0,9678103	40S ribosomal protein S3A, putative
[illegible]	0,8601969	0,9678193	conserved Plasmodium protein, unknown function
[illegible]	0,8602695	0,9678193	conserved Plasmodium protein, unknown function
[illegible]	0,8604834	0,9678193	erythrocyte membrane protein 3
[illegible]	0,8605773	0,9678193	conserved Plasmodium protein, unknown function
[illegible]	0,8610088	0,9678193	conserved Plasmodium protein, unknown function
[illegible]	0,8610437	0,9678193	RNA methyltransferase, putative
[illegible]	0,861193	0,9678193	Plasmodium exported protein, unknown function
[illegible]	0,8612155	0,9678193	conserved Plasmodium protein, unknown function
[illegible]	0,861507	0,9679546	conserved Plasmodium protein, unknown function
[illegible]	0,8616879	0,9679656	reticulocyte binding protein 2 homologue a
[illegible]	0,8625809	0,9682789	selenoprotein
[illegible]	0,8626164	0,9682789	60S ribosomal protein L18-2, putative
[illegible]	0,8626539	0,9682789	coproporphyrinogen III oxidase
[illegible]	0,862775	0,9682789	conserved Plasmodium membrane protein, unknown function
[illegible]	0,8628228	0,9682789	mitochondrial ribosomal protein S29 precursor, putative
[illegible]	0,8638795	0,9690261	SNARE protein, putative
[illegible]	0,8641784	0,9690261	conserved Plasmodium protein, unknown function
[illegible]	0,8642079	0,9690261	conserved Plasmodium protein, unknown function
[illegible]	0,8643451	0,9690261	erythrocyte membrane protein 1 (PfEMP1), pseudogene
[illegible]	0,8644515	0,9690261	conserved Plasmodium protein, unknown function
[illegible]	0,8645166	0,9690261	conserved Plasmodium protein, unknown function
[illegible]	0,8649755	0,9693463	stevor
[illegible]	0,8652979	0,9693463	conserved Plasmodium protein, unknown function
[illegible]	0,8653164	0,9693463	conserved Plasmodium protein, unknown function
[illegible]	0,865722	0,9696086	conserved Plasmodium protein, unknown function
[illegible]	0,8660084	0,9697374	polyadenylate-binding protein-interacting protein 1, putative
[illegible]	0,8665682	0,9699835	pantothenate kinase, putative
[illegible]	0,8665712	0,9699835	cation-transporting ATPase 1
[illegible]	0,8669619	0,9702288	exosome complex component RRP4, putative
[illegible]	0,8673781	0,9703414	early transcribed membrane protein 11.1
[illegible]	0,8674056	0,9703414	cysteine repeat modular protein 2
[illegible]	0,8681123	0,9709399	conserved Plasmodium protein, unknown function
[illegible]	0,8685493	0,9712366	transcription factor with AP2 domain(s), putative
[illegible]	0,8689299	0,9713588	cytosolic glyoxalase II
[illegible]	0,869002	0,9713588	Plasmodium exported protein, unknown function
[illegible]	0,8701786	0,9724818	conserved Plasmodium protein, unknown function
[illegible]	0,8706343	0,9727633	conserved Plasmodium protein, unknown function
[illegible]	0,8707745	0,9727633	DnaJ protein, putative
[illegible]	0,8712178	0,9730486	DNA repair exonuclease Mre11, putative
[illegible]	0,871374	0,9730486	anaphase promoting complex subunit, putative
[illegible]	0,8720673	0,973128	erythrocyte membrane protein 1, PfEMP1
[illegible]	0,8721295	0,973128	conserved Plasmodium membrane protein, unknown function
[illegible]	0,8726963	0,973128	tryptophan-rich antigen, pseudogene
[illegible]	0,8727047	0,973128	conserved Plasmodium protein, unknown function
[illegible]	0,8727856	0,973128	conserved Plasmodium protein, unknown function
[illegible]	0,8729275	0,973128	conserved Plasmodium protein, unknown function
[illegible]	0,8729609	0,973128	bromodomain protein, putative
[illegible]	0,873018	0,973128	rifin
[illegible]	[illegible]	[illegible]	[illegible]

PFIT_05153	18,1130849	6,341895088	6,233352403	3,874107784	12,43423176	2,916147135	7,204730823	7,586025424	8,3188032	7,3953781	0,8889954	-0,169752	1	1	conserved Plasmodium protein, unknown function
PFIT_05333	50,94305128	29,80690691	23,06340389	20,47742686	99,47385408	55,40679557	43,22838494	33,18886123	46,528573	38,208623	0,8211862	-0,284219	1	1	conserved Plasmodium protein, unknown function
PFIT_06082	1696,969642	318,3631334	367,7677918	421,7243045	171,316082	78,73597266	481,5161767	423,8691706	509,14615	452,69267	0,8891213	-0,169548	1	1	conserved Plasmodium protein, unknown function
PFIT_06143	405,2802746	333,5836816	222,5306808	240,1946826	189,276639	128,310474	273,7797713	248,4423326	253,19607	261,11105	1,0312603	0,0444085	1	1	GPI-anchored wall transfer protein 1, putative (GWT1)
PFIT_06188	122,2633231	341,8281452	259,9307952	294,9856356	248,6846352	265,3693893	283,386079	251,2870922	255,51032	267,33659	1,0462849	0,0652757	1	1	splicing factor 3A subunit 2, putative
PFIT_07060	1,132067806	0	0	0	0	0	0	0	0,188678	0	0	#DIV/0!	1	1	tRNA Lysine
PFIT_07064	1676,592421	2850,681842	2492,094291	3908,974754	1965,990199	772,7789909	2682,561443	2383,90849	2277,8521	2533,235	1,1121157	0,1533068	1	1	conserved Plasmodium protein, unknown function
PFIT_07084	43,01857664	14,5863587	16,20671625	11,06887938	42,82902051	14,58073568	19,21261553	20,86156992	23,715048	20,037093	0,8449105	-0,24313	1	1	conserved Plasmodium protein, unknown function
PFIT_07109	187,9232558	27,90433839	25,55674485	23,24464671	9,671069147	17,49688281	37,22444258	42,67139301	48,632823	39,947918	0,8214189	-0,28381	1	1	A_ITvar64 - erythrocyte membrane protein 1, PfEMP1, putative
PFIT_07170	1,132067806	7,610274105	8,103358124	2,767219846	1,381581307	0	2,401576941	4,74126589	3,4990835	3,5714214	1,0206734	0,0295213	1	1	conserved Plasmodium protein, unknown function
PFIT_07176	6335,051444	982,3595491	1712,92524	1876,7285	816,5145522	501,5773073	2201,045266	1577,893288	2037,5261	1889,4693	0,927335	-0,108837	1	1	conserved Plasmodium protein, unknown function
PFIT_07336	6,792406838	0	0	0	0	0	0	0,948253178	1,1320678	0,4741266	0,4188147	-1,255616	1	1	rifin
PFIT_07341	1,132067806	1,902568526	1,246670481	0,553443969	0	0	0	0,948253178	0,8057918	0,4741266	0,5883984	-0,765135	1	1	stevor
PFIT_08010	37,35823761	12,04960067	13,71337529	10,51543541	0	2,916147135	10,80709623	15,17205085	12,758799	12,989574	1,0180875	0,0258615	1	1	rifin (RIF)
PFIT_08113	332,827935	671,6066898	703,7454863	845,108941	685,2643281	472,4158359	750,492794	567,0554005	618,49487	658,7741	1,0651246	0,0910222	1	1	coatomer epsilon subunit, putative
PFIT_08175	168,6781031	421,7360233	220,0373398	234,106799	208,6187773	104,9812969	230,5513863	248,4423326	226,35972	239,49686	1,0580365	0,0813895	1	1	conserved Plasmodium protein, unknown function
PFIT_08213	42185,37473	12960,93099	28503,87387	38479,29885	1341,515449	1793,430488	26303,27145	20664,33326	20877,404	23483,802	1,124843	0,1697237	1	1	nucleolar preribosomal assembly protein, putative
PFIT_08214	2714,698599	3073,28236	2527,001064	2823,117687	2250,595949	2006,309229	2662,148039	2613,385759	2565,8341	2637,7669	1,0280348	0,0398892	1	1	zinc finger protein, putative
PFIT_08289	1952,816966	249,8706665	369,0144623	380,2160068	223,8161717	621,1393398	629,2131585	467,4888168	632,81227	548,35099	0,8665303	-0,206678	1	1	conserved Plasmodium protein, unknown function
PFIT_08303	174,3384422	59,61381383	40,51679062	47,04273738	41,4474392	29,16147135	58,83863505	65,42946928	65,353449	62,134052	0,9507387	-0,072879	1	1	conserved Plasmodium protein, unknown function
PFIT_08342	198,1118661	83,07882565	92,25361556	65,85983233	78,75013448	285,7824193	87,65755834	119,4799004	133,97278	103,56873	0,773058	-0,371351	1	1	Plasmodium exported protein, unknown function
PFIT_09009	2,264135613	0	0	0,553443969	0	0	0	0	0,4695966	0	0	#DIV/0!	1	1	stevor
PFIT_09014	1,132067806	1,268379018	0	0,553443969	0	0	0	0,948253178	0,4923151	0,4741266	0,9630551	-0,05431	1	1	Plasmodium exported protein (PHISTa), unknown function, pseudogene
PFIT_09058	130,1877977	55,17448726	54,23016591	64,7529444	40,06585789	17,49688281	67,24415435	56,89519068	60,318023	62,069673	1,0290402	0,0412994	1	1	conserved Plasmodium protein, unknown function
PFIT_09159	1,132067806	12,04960067	4,363346682	8,301659538	9,671069147	11,66458854	13,20867318	2,844759534	7,8637221	8,0267164	1,0207274	0,0295976	1	1	glycolipid transfer protein, putative
PFIT_09183	125,6595265	780,0530958	656,9953433	406,2278734	610,6589375	449,0866589	555,9650618	516,797982	504,78024	536,38152	1,062604	0,0876041	1	1	50S ribosomal protein L3, apicoplast, putative
PFIT_09241	12637,27292	6301,941149	6513,229926	7998,372243	5419,943466	4890,378746	7404,061709	7262,67109	7293,5231	7333,3664	1,0054628	0,0078598	1	1	patatin-like phospholipase, putative
PFIT_09251	33,96203419	20,92825379	9,350028604	10,51543541	15,19739437	17,49688281	22,81498094	11,37903814	17,908338	17,09701	0,9546955	-0,066887	1	1	conserved Plasmodium protein, unknown function
PFIT_09271	87,16922108	81,17625712	74,17689359	49,80995723	92,56594755	81,65211979	74,44888517	71,11898835	77,758399	72,783937	0,9360267	-0,095378	1	1	CS domain protein, putative
PFIT_09294	2495,077445	1750,363044	3162,179674	3744,048452	1641,318592	2207,523381	2698,171693	2583,98991	2500,0851	2641,0808	1,0563964	0,0791512	1	1	RNA binding protein, putative
PFIT_09374	74,71647521	134,4481759	140,8737643	117,8835654	74,60539056	195,3818581	105,6693854	131,8071917	122,98487	118,73829	0,9654707	-0,050696	1	1	Plasmodium exported protein (PHISTc), unknown function
PFIT_10051	1996,96761	1537,275369	1314,614022	1044,902214	2094,477261	3414,808296	1550,217915	1627,202453	1900,5075	1588,7102	0,83594	-0,258529	1	1	regulator of nonsense transcripts, putative
PFIT_10098	817,3529561	2971,812038	2343,117168	2833,079678	2370,793522	787,3597266	2315,120171	2267,273349	2020,5858	2291,1968	1,133927	0,1813277	1	1	succinate dehydrogenase subunit 4, putative
PFIT_10130	2176,966391	2345,866993	2573,127872	3109,248219	2280,990737	2035,470701	2641,734635	2313,737754	2420,2785	2477,7362	1,0237401	0,0338495	1	1	phosphoinositide-specific phospholipase C
PFIT_10180	230,9418325	727,4153666	666,9687071	804,7075312	468,356063	533,6549258	612,4021199	641,9674015	572,0074	627,18476	1,0964627	0,1328567	1	1	PHFS-like protein, putative
PFIT_10185	16725,16977	11138,27034	16276,52979	18040,05962	9002,383794	16823,25282	15067,49373	14522,49742	14667,611	14794,996	1,0086848	0,0124754	1	1	eukaryotic translation initiation factor subunit eIF2A, putative
PFIT_10193	5284,49252	694,4375121	731,1722369	885,5103507	712,8959542	411,1767461	1263,229471	1141,696826	1453,2809	1202,4631	0,8274128	-0,273321	1	1	tRNA methyltransferase, putative
PFIT_10248	110,942645	215,624433	157,0804806	134,4868845	360,592721	387,847569	218,5435016	175,4268379	227,76246	196,98517	0,8648711	-0,209443	1	1	conserved Plasmodium protein, unknown function
PFIT_10263	12,45274587	21,5624433	17,45338673	10,51543541	6,907906533	20,41302995	14,40946165	15,17205085	14,884158	14,790756	0,9937248	-0,009082	1	1	biotin--acetyl-CoA-carboxylase, putative
PFIT_10288	510,5625806	675,4118269	824,6725229	703,9807288	770,9223691	956,4962604	739,6856978	699,8108454	740,34105	719,74827	0,9721847	-0,040698	1	1	conserved Plasmodium protein, unknown function
PFIT_10320	13,58481368	43,1248866	29,92009153	27,67219846	31,77637005	23,32917708	26,41734635	31,29235487	28,23459	28,854851	1,0219681	0,0313502	1	1	conserved protein, unknown function
PFIT_11003	3,396203419	5,07351607	5,610017163	4,427551754	1,381581307	20,41302995	1,20078847	7,586025424	6,7169833	4,3934069	0,6540744	-0,612473	1	1	stevor
PFIT_11032	543,392547	1282,965376	989,8563616	1019,997235	1197,830993	1589,300189	1078,308046	1024,113432	1103,8905	1051,2107	0,9522781	-0,070545	1	1	flavoprotein, putative
PFIT_11055	144,9046792	156,6448087	65,45020023	40,40140975	118,8159924	256,6209479	98,46465458	112,8421282	130,47301	105,65339	0,809772	-0,304412	1	1	RNA polymerase subunit, putative
PFIT_11091	103,0181704	109,714785	99,73363845	91,31825492	105,0001793	84,56826693	104,4685969	93,87706462	98,892216	99,172831	1,0028376	0,004088	1	1	pterin-4a-carbinolamine dehydratase
PFIT_11094	8638,80943	5214,306141	4814,018061	4434,193081	9367,121259	12166,16585	6341,363912	6176,921202	7439,1023	6259,1426	0,8413841	-0,249164	1	1	endoplasmic reticulum-resident calcium binding protein
PFIT_11229	19,24515271	41,22231807	25,55674485	29,88597434	19,34213829	34,99376562	32,4212887	24,65458263	28,374349	28,537936	1,0057653	0,0082937	1	1	conserved Plasmodium protein, unknown function
PFIT_11338	1946,024559	1789,682794	2257,096905	2572,961013	1072,107094	1904,244079	1861,222129	2109,863321	1923,6861	1985,5427	1,0321553	0,04566	1	1	conserved membrane protein, unknown function
PFIT_11404	26,03755954	276,5066258	253,0741076	701,7669529	292,895237	90,4005612	348,2286564	301,5445106	273,44684	324,88658	1,1881161	0,2486758	1	1	adrenodoxin reductase, putative
PFIT_11458	2479,228496	3680,835909	3978,748839	3976,494919	3230,137095	4012,618458	3508,703911	3549,311645	3559,6773	3529,0078	0,9913842	-0,012484	1	1	coatomer gamma subunit, putative
PFIT_11493	2184,890866	2012,283311	2116,846476	1821,384103	1190,923086	3166,935789	1953,682841	1997,021193	2082,2106	1975,352	0,9486802	-0,076006	1	1	Plasmodium exported protein, unknown function
PFIT_11494	82806,23176	38898,01352	31872,37751	43774,0973	18656,87396	11889,13187	38930,763	41329,61476	37982,788	40130,189	1,0565362	0,0793422	1	1	ring-infected erythrocyte surface antigen, pseudogene
PFIT_11498	6817,312329	3854,603834	4687,481007	5753,603504	2916,518138	4085,522137	4626,637977	4906,261943	4685,8402	4766,45	1,0172028	0,0246074	1	1	DnaJ protein, putative
PFIT_12035	26322,84063	7355,329923	12980,95638	9293,431131	1413,357677	2181,278057	11269,3998	8889,873544	9924,5323	10079,637	1,0156284	0,0223726	1	1	unspecified product
PFIT_12153	91,69749231	22,83082232	7,480022884	13,83609923	11,05265045	5,832294271	26,41734635	18,01681038	25,454897	22,217078	0,8728017	-0,196274	1	1	conserved Plasmodium protein, unknown function
PFIT_12170	1811,30849	1201,15493	1375,07754	1654,244024	696,3169786	737,7852253	1629,469954	1036,440724	1245,9812	1332,9553	1,0698037	0,0973461	1	1	GTP binding protein, putative
PFIT_12193	3862,615355	5215,57452	4034,225675	3435,226717	3710,92739	4782,481302	4051,460299	4175,158743	4173,5085	4113,3095	0,9855759	-0,020961	1	1	aminophospholipid-transporting P-ATPase
PFIT_12222	1985,646932	1246,816574	837,1392277	939,1944157	737,7644178	918,5863476	1012,264681	1142,64508	1110,858	1077,4549	0,9699304	-0,044047	1	1	ATP-binding protein, putative
PFIT_12278	82,64094986	107,8122165	152,7171139	128,3990009	146,4476185	157,4719453	141,6930395	111,893875	129,24814	126,79346	0,981008	-0,027663	1	1	leucine-rich repeat protein
PFIT_12296	123,3953909	190,8910421	124,6670481	149,4298717	172,6976633	128,310474	166,9095974	136,5484576	148,23192	151,72903	1,0235922	0,033641	1	1	conserved Plasmodium protein, unknown function
PFIT_12408	9,05654245	2,536758035	1,870005721	0,553443969	9,671069147	0	3,602365411	2,844759534	3,9479699	3,2235625	0,8165114	-0,292455	1	1	rifin, pseudogene
PFIT_12487	8656,922515	8319,297976	7457,582835	8728,918282	7073,69629	7474,085108	7869,967635	8200,493483	7951,7505	8035,2306	1,0104983	0,0150669	1	1	bax inhibitor 1, putative
PFIT_12494	29,43376296	5,07351607	0,62333524	3,320663815	4,14474392	11,66458854	12,0078847	2,844759534	9,0434351	7,4263221	0,8211838	-0,284223	1	1	conserved Plasmodium protein, unknown function
PFIT_12500	5908,261881	3888,850068	4780,981293	5487,396955	3558,953446	4522,944207	4697,484496	4581,011103	4691,2313	4639,2478	0,988919	-0,016076	1	1	conserved Plasmodium protein, unknown function
PFIT_12536	6,792406838	8,878653123	8,103358124	13,28265526	2,763162613	2,916147135	7,204730823	8,534278602	7,1227305	7,8695047	1,1048438	0,1438424	1	1	acyl-CoA binding protein, putative
PFIT_13015	92,82956012	643,7023514	549,1583467	667,4534269	279,0794239	221,6271823	456,2996188	497,8329185	408,97505	477,06627	1,1664924	0,2221769	1	1	Plasmodium exported protein, unknown function
PFIT_13022	112,0747128	193,4278002	104,7203204	103,4940222	198,9477082	344,105362	136,8898856	157,4100276	176,12832	147,14996	0,8354702	-0,25934	1	1	conserved Plasmodium protein, unknown function
PFIT_13135	16,98101709	58,97962432	44,8801373	30,99286228	75,98697187	52,49064844	40,826808	46,46440572	46,718544	43,645607	0,9342245	-0,098159	1	1	conserved Plasmodium protein, unknown function
PFIT_13177	9,05654245	8,244463614	6,233352403	4,980995723	15,19739437	2,916147135	9,606307764	4,74126589	7,7714826	7,1737868	0,9230912	-0,115455	1	1	conserved Plasmodium protein, unknown function
PFIT_13331	7000,707314	884,6943647	1009,803089	904,8808896	717,0406982	1353,092271	1506,98953	1511,515566	1978,3698	1509,2525	0,7628769	-0,390478	1	1	conserved Plasmodium protein, unknown function
PFIT_13432	173,2063744	95,76261583	84,77359268	96,85269461	85,65804101	75,81982552	102,06702	97,67007734	102,01219	99,868549	0,9789864	-0,030639	1	1	aminomethyltransferase, putative
PFIT_13463	1468,291945	320,8998914	304,1875973	359,73858	330,1979323	128,310474	485,1185421	437,1447151	485,27107	461,13163	0,9502558	-0,073612	1	1	DNA topoisomerase III, putative
PFIT_13547	217,3570188	44,39326561	21,19339817	27,11875449	26,25004483	49,5745013	54,03548117	46,46440572	64,314497	50,249943	0,781316	-0,356022	1	1	conserved Plasmodium protein, unknown function
PFIT_13656	2119,230933	2034,479944	2585,594577	3085,450128	1775,331979	2469,976624	2375,159595	2455,027478	2345,0107	2415,0935	1,0298859	0,0424846	1	1	signal recognition particle receptor alpha subunit
PFIT_14006	1,132067806	0	1,246670481	1,106887938	0	0	0	0,948253178	0,5809377	0,4741266	0,8161402	-0,293111	1	1	Plasmodium exported protein, unknown function, fragment
PFIT_14008	1,132067806	1,268379018	2,493340961	0,553443969	0	0	1,20078847	0	0,907872	0,6003942	0,6613204	-0,596579	1	1	Plasmodium exported protein, unknown function, fragment
PFIT_14009	1,132067806	0,634189509	1,246670481	0,553443969	0	0	0	0	0,5943953	0	0	#DIV/0!	1	1	Plasmodium exported protein, unknown function, fragment
PFIT_14105	1039,238246	128,7404703	125,9137185	150,5367596	93,94752885	64,15523698	234,1537517	210,5122055	267,08866	222,33298	0,8324314	-0,264597	1	1	conserved Plasmodium protein, unknown function
PFIT_14293	1989,043136	530,8166188	504,9015446	704,5341728	433,8165303	443,2543646	762,5006787	678,9492755	767,72773	720,72498	0,9387768	-0,091146	1	1	WW domain-binding protein 11, putative
PFIT_14330	348,6768843	186,4517156	87,89026888	96,85269461	161,6450129	122,4781797	151,2993473	154,565268	167,33246	152,93231	0,9139429	-0,129824	1	1	apyrase, putative
PFIT_14380	33,96203419	118,5934381	140,2504291	141,6816561	103,618598	93,31670833	128,4843663	105,2561028	105,23714	116,87023	1,1105417	0,1512635	1	1	ATP-dependent Clp protease proteolytic subunit, putative (ClpR)
PFIT_14439	2339,984156	276,5066258	221,2840103	279,4892044	519,4745713	209,9625937	545,1579656	440,9377278	641,11686	493,04785	0,7690452	-0,37886	1	1	conserved Plasmodium protein, unknown function
PFIT_14538	3518,466742	2315,425897	2221,566796	1871,747504	2007,437639	2064,632172	2305,513863	2246,411779	2333,2128	2275,9628	0,975463	-0,035841	1	1	conserved Plasmodium protein, unknown function
PFIT_14553	4,528271225	8,244463614	8,726693364	5,534439692	29,01320744	5,832294271	3,602365411	12,32729131	10,313228	7,9648284	0,7722925	-0,372781	1	1	tRNA intron endonuclease, putative
PFIT_14627	3,396203419	24,73339084	18,70005721	13,28265526	13,81581307	5,832294271	13,20867318	14,22379767	13,293402	13,716235	1,0318077	0,0451742	1	1	meiotic nuclear division protein 1, putative
PFIT_bin03	13,58481368	8,878653123	2,493340961	4,980995723	2,763162613	5,832294271	1,20078847	8,534278602	6,4222101	4,8675335	0,7579219	-0,399879	1	1	rifin (RIF)
PFIT_bin04	1,132067806	1,268379018	0,62333524	2,213775877	2,763162613	0	0	0,948253178	1,3334534	0,4741266	0,3555629	-1,491823	1	1	rifin (RIF)
PFIT_bin07	156,2253573	15,22054821	22,44006865	20,47742686	13,81581307	20,41302995	25,21655788	35,08536759	41,432041	30,150963	0,7277209	-0,458543	1	1	erythrocyte membrane protein 1 (PfEMP1), pseudogene
PFIT_bin09	1,132067806	2,536758035	0	1,660331908	1,381581307	2,916147135	1,20078847	0,948253178	1,604481	1,0745208	0,6696999	-0,578413	1	1	rifin (RIF)
PFIT_bin10	1,132067806	0	0,62333524	1,106887938	0	0	1,20078847	0	0,4770485	0,6003942	1,2585602	0,3317742	1	1	B_ITvar41 - erythrocyte membrane protein 1, PfEMP1, putative
PF_apicopla	32,82996638	0,634189509	0	0	0	0	2,401576941	3,793012712	5,5773593	3,0972948	0,5553336	-0,848574	1	1	unspecified product
PF_apicopla	1,132067806	0,634189509	0	0,553443969	0	0	0	0	0,3866169	0	0	#DIV/0!	1	1	unspecified product
PF_apicopla	49,81098348	36,78299151	34,28343822	39,29452181	31,77637005	34,99376562	36,02365411	37,93012712	37,823678	36,976891	0,9776122	-0,032666	1	1	ribosomal protein S3, putative
Pf_M76611	53,2071869	30,44109642	21,19339817	45,38240547	987,8306343	612,3908984	171,7127513	141,2897235	291,74094	156,50124	0,5364391	-0,898514	1	1	unspecified product
PFIT_01135	0	0,634189509	0,62333524	0	0	8,748441406	1,20078847	0	1,667661	0,6003942	0,3600217	-1,473844	1	1	rifin
PFIT_02009	0	0,634189509	0,62333524	0	1,381581307	0	0	0	0,439851	0	0	#DIV/0!	1	1	Plasmodium exported protein (PHISTb), unknown function
PFIT_02010	0	1,268379018	0,62333524	1,660331908	1,381581307	0	1,20078847	0	0,8222712	0,6003942	0,7301657	-0,453704	1	1	DnaJ protein, putative
PFIT_03005	0	0,634189509	1,246670481	0	0	11,66458854	0	0,948253178	2,2575748	0,4741266	0,2100159	-2,25143	1	1	rifin (RIF)
PFIT_04088	0	0,634189509	0,62333524	2,213775877	0	0	1,20078847	0	0,5785501	0,6003942	1,0377567	0,0534682	1	1	tRNA Isoleucine
PFIT_04240	0	0,634189509	0	0	0	0	0	0	0,1056983	0	0	#DIV/0!	1	1	rifin (RIF)
PFIT_05190	0	13,95216919	16,83005149	26,01186655	8,28948784	8,748441406	13,20867318	15,17205085	12,305136	14,190362	1,1531877	0,2056273	1	1	conserved Plasmodium protein, unknown function
PFIT_06001	0	1,902568526	1,870005721	0	0	0	0	0	0,6287624	0	0	#DIV/0!	1	1	erythrocyte membrane protein 1 (PfEMP1), truncated, pseudogene
PFIT_06031	0	1,902568526	3,116676201	2,767219846	2,763162613	0	3,602365411	0,948253178	1,7582712	2,2753093	1,2940605	0,3719051	1	1	mitochondrial ribosomal protein L41 precursor, putative
PFIT_06198	0	0,634189509	0	0	1,381581307	0	0	0	0,3359618	0	0	#DIV/0!	1	1	tRNA Leucine
PFIT_06213	0	9,512842632	9,350028604	8,855103507	27,63162613	5,832294271	8,405519293	9,48253178	10,196983	8,9440255	0,8771247	-0,189146	1	1	conserved Apicomplexan protein, unknown function
PFIT_07296	0	0,634189509	0	0	0	0	0	0	0,1056983	0	0	#DIV/0!	1	1	tRNA Threonine
PFIT_07311	0	1,268379018	3,116676201	2,213775877	1,381581307	0	0	1,896506356	1,3300687	0,9482532	0,7129355	-0,488157	1	1	stevor
PFIT_07335	0	6,341895088	1,246670481	5,534439692	5,526325227	5,832294271	3,602365411	2,844759534	4,0802708	3,2235625	0,7900364	-0,340009	1	1	rifin
PFIT_07338	0	0,634189509	1,246670481	0	1,381581307	0	0	0,948253178	0,5437402	0,4741266	0,8719726	-0,197645	1	1	stevor
PFIT_07342	0	0,634189509	0,62333524	1,660331908	0	0	0	0,948253178	0,4863094	0,4741266	0,9749484	-0,036602	1	1	rifin (RIF)
PFIT_08013	0	0,634189509	0	0,553443969	0	0	0	0	0,1979389	0	0	#DIV/0!	1	1	Plasmodium exported protein (PHISTa), unknown function, pseudogene
PFIT_08017	0	2,536758035	3,740011442	1,106887938	0	2,916147135	1,20078847	1,896506356	1,7166341	1,5486474	0,9021418	-0,148574	1	1	Plasmodium exported protein, unknown function
PFIT_08069	0	0,634189509	0,62333524	0,553443969	0	0	0	0	0,3018281	0	0	#DIV/0!	1	1	Plasmodium RNA of unkown function RUF3
PFIT_09052	0	11,41541116	6,233352403	9,961991446	26,25004483	8,748441406	9,606307764	8,534278602	10,434874	9,0702932	0,8692289	-0,202192	1	1	conserved Plasmodium protein, unknown function
PFIT_11357	0	0,634189509	1,246670481	0,553443969	6,907906533	0	0	1,896506356	1,5570351	0,9482532	0,6090121	-0,715457	1	1	conserved Plasmodium protein, unknown function
PFIT_11390	0	29,80690691	18,70005721	19,92398289	8,28948784	5,832294271	16,81103859	17,0685572	13,758788	16,939798	1,2311984	0,3000633	1	1	conserved Plasmodium membrane protein, unknown function
PFIT_12192	0	6,341895088	0,62333524	1,106887938	1,381581307	2,916147135	1,20078847	1,896506356	2,0616411	1,5486474	0,7511722	-0,412784	1	1	erythrocyte membrane protein 1 (PfEMP1), exon2, pseudogene
PFIT_12412	0	2,536758035	0	1,106887938	51,11850835	29,16147135	3,602365411	8,534278602	13,987271	6,068322	0,433846	-1,204745	1	1	unspecified product
PFIT_12521	0	0,634189509	0	0	0	2,916147135	0	0	0,5917228	0	0	#DIV/0!	1	1	tRNA Valine
PFIT_12538	0	1,902568526	1,246670481	0	1,381581307	0	0	0,948253178	0,7551367	0,4741266	0,6278685	-0,671466	1	1	Plasmodium exported protein, unknown function, pseudogene
PFIT_13001	0	1,268379018	2,493340961	2,213775877	6,907906533	5,832294271	3,602365411	0,948253178	3,1192828	2,2753093	0,7294335	-0,455152	1	1	rifin (RIF)
PFIT_13402	0	0,634189509	0	0	0	0	0	0	0,1056983	0	0	#DIV/0!	1	1	tRNA Arginine
PFIT_13545	0	1,268379018	0	0,553443969	0	0	0	0	0,3036372	0	0	#DIV/0!	1	1	tRNA Threonine
PFIT_13688	0	0,634189509	0	0	1,381581307	0	0	0	0,3359618	0	0	#DIV/0!	1	1	tRNA Arginine
PFIT_14007	0	1,268379018	0,62333524	0,553443969	0	0	0	0	0,4075264	0	0	#DIV/0!	1	1	Plasmodium exported protein, unknown function, fragment
PFIT_14010	0	1,902568526	0	0	0	0	0	0	0,3170948	0	0	#DIV/0!	1	1	Plasmodium exported protein, unknown function, fragment
PFIT_14011	0	0,634189509	0,62333524	1,660331908	0	0	1,20078847	0	0,4863094	0,6003942	1,234593	0,3040355	1	1	Plasmodium exported protein, unknown function, fragment
PFIT_14014	0	1,268379018	1,246670481	2,767219846	1,381581307	2,916147135	1,20078847	0,948253178	1,5966663	1,0745208	0,6729777	-0,571369	1	1	rifin
PFIT_14052	0	3,170947544	4,986681922	4,980995723	5,526325227	8,748441406	1,20078847	5,689519068	4,5688986	3,4451538	0,7540447	-0,407278	1	1	conserved Plasmodium protein, unknown function
PFIT_14394	0	0,634189509	0	0	1,381581307	0	0	0	0,3359618	0	0	#DIV/0!	1	1	tRNA Selenocysteine
PFIT_14634	0	4,439326561	1,870005721	3,320663815	5,526325227	2,916147135	1,20078847	3,793012712	3,0120781	2,4969006	0,8289628	-0,270621	1	1	conserved Plasmodium protein, unknown function
PFIT_14659	0	0,634189509	1,870005721	1,106887938	0	0	0	0	0,6018472	0	0	#DIV/0!	1	1	conserved Plasmodium protein, unknown function
PFIT_bin05	0	1,902568526	3,116676201	2,767219846	4,14474392	2,916147135	2,401576941	0,948253178	2,4745593	1,6749151	0,6768539	-0,563084	1	1	rifin (RIF)
PFIT_bin05	0	1,902568526	1,870005721	0	0	0	1,20078847	0	0,6287624	0,6003942	0,9548826	-0,066605	1	1	rifin (RIF)
PFIT_bin06	0	0,634189509	1,246670481	1,106887938	0	0	1,20078847	0	0,497958	0,6003942	1,2057126	0,2698861	1	1	rifin (RIF)
PFIT_bin09	0	0,634189509	1,246670481	1,106887938	0	0	0	0,948253178	0,497958	0,4741266	0,9521417	-0,070752	1	1	stevor, pseudogene
PF_apicopla	0	1,902568526	0,62333524	1,660331908	0	0	0	0	0,6977059	0	0	#DIV/0!	1	1	apicoplast ribosomal protein S17
PF_apicopla	0	0,634189509	0	0,553443969	4,14474392	2,916147135	0	0,948253178	1,3747541	0,4741266	0,344881	-1,535829	1	1	apicoplast ribosomal protein L36
Pf_M76611	0	0,634189509	0	0	0	0	0	0	0,1056983	0	0	#DIV/0!	1	1	unspecified product
Pf_M76611	0	3,805137053	0,62333524	3,320663815	17,96055699	8,748441406	4,803153882	2,844759534	5,7430224	3,8239567	0,6658439	-0,586744	1	1	unspecified product
PFIT_03126	0	0	0,62333524	0	0	0	0	0	0,1038892	0	0	#DIV/0!	1	1	tRNA Isoleucine
PFIT_04087	0	0	1,870005721	0	0	0	0	0	0,3116676	0	0	#DIV/0!	1	1	tRNA Serine
PFIT_04238	0	0	0,62333524	0	0	0	0	0	0,1038892	0	0	#DIV/0!	1	1	stevor, pseudogene
PFIT_06205	0	0	0,62333524	0,553443969	6,907906533	5,832294271	0	1,896506356	2,3194967	0,9482532	0,4088185	-1,290468	1	1	tRNA Serine
PFIT_07057	0	0	2,493340961	0	1,381581307	0	0	0	0,6458204	0	0	#DIV/0!	1	1	tRNA Threonine
PFIT_10386	0	0	1,246670481	0,553443969	0	0	0	0	0,3000191	0	0	#DIV/0!	1	1	Plasmodium exported protein (hyp10), unknown, putative
PFIT_12522	0	0	0,62333524	0	0	0	0	0	0,1038892	0	0	#DIV/0!	1	1	tRNA Glutamic acid
PFIT_13689	0	0	0,62333524	0	0	0	0	0	0,1038892	0	0	#DIV/0!	1	1	tRNA Tryptophan
PFIT_13690	0	0	1,246670481	0,553443969	1,381581307	0	0	0	0,5302826	0	0	#DIV/0!	1	1	tRNA Arginine
PFIT_13692	0	0	0,62333524	0	0	0	0	0	0,1038892	0	0	#DIV/0!	1	1	tRNA Glycine
PFIT_bin10	0	0	0,62333524	0	0	0	0	0	0,1038892	0	0	#DIV/0!	1	1	B_ITvar19 - erythrocyte membrane protein 1, PfEMP1, putative
PFIT_07059	0	0	0	1,660331908	1,381581307	0	0	0	0,5069855	0	0	#DIV/0!	1	1	tRNA Lysine
PF_apicopla	0	0	0	0,553443969	2,763162613	0	0	0	0,5527678	0	0	#DIV/0!	1	1	unspecified product
PF_apicopla	0	0	0	0,553443969	0	0	0	0	0,0922407	0	0	#DIV/0!	1	1	unspecified product
PFIT_02004	0	0	0	0	1,381581307	0	0	0	0,2302636	0	0	#DIV/0!	1	1	Plasmodium exported protein (PHISTa), unknown function
PFIT_05008	0	0	0	0	1,381581307	0	0	0	0,2302636	0	0	#DIV/0!	1	1	rifin, pseudogene
PFIT_07017	0	0	0	0	1,381581307	0	0	0	0,2302636	0	0	#DIV/0!	1	1	tRNA Tyrosine
PFIT_11041	0	0	0	0	2,763162613	0	0	0	0,4605271	0	0	#DIV/0!	1	1	tRNA Leucine
PFIT_12403	0	0	0	0	1,381581307	0	0	0	0,2302636	0	0	#DIV/0!	1	1	unspecified product
PFIT_07128	0	0	0	0	0	0	0	0	0	0	#NV	#NV	#NV	#NV	tRNA Asparagine
PFIT_11102	0	0	0	0	0	0	0	0	0	0	#NV	#NV	#NV	#NV	small nucleolar RNA snoR09
PFIT_13383	0	0	0	0	0	0	0	0	0	0	#NV	#NV	#NV	#NV	tRNA Methionine
PF_apicopla	0	0	0	0	0	0	0	0	0	0	#NV	#NV	#NV	#NV	unspecified product
Pf_M76611	0	0	0	0	0	0	0	0	0	0	#NV	#NV	#NV	#NV	unspecified product

Table S4. Differentially expressed genes (threshold, ≥3-fold; padj ≤ 0.05) in FCR3 knobby IEs cultivated in the presence of human serum and AlbuMAX.

	HS_K+_1	HS_K+_2	AlbuMAX_K+_1	AlbuMAX_K+_2	baseMeanA	baseMeanB	foldChange	log2FoldChang	pval	padj	name
PFIT_1239800	4047,3531	4134,9926	337,1170952	322,5826806	4091,172873	329,8498879	0,080624774	-3,63263298	5,786E-57	3,269E-53	early transcribed membrane protein 12
PFIT_1035100	1945,1849	1609,8725	18368,32605	17708,18516	1777,52868	18038,25561	10,14794068	3,343115087	1,22E-53	3,447E-50	duffy binding-like merozoite surface protein
PFIT_1478600	1803,2961	1975,0081	170,8363658	129,2112947	1889,152098	150,0238303	0,079413315	-3,654475273	7,012E-53	1,321E-49	Plasmodium exported protein (PHISTb), unknown function
PFIT_bin11000	1541,6571	1477,8841	21789,60901	18505,73074	1509,770631	20147,66987	13,34485481	3,738211704	3,766E-52	5,319E-49	erythrocyte membrane protein 1, PfEMP1, putative
PFIT_0936400	2807,5871	3064,6345	24898,83087	21742,25089	2936,110817	23320,54088	7,942663725	2,989622924	1,829E-44	2,067E-41	cytoadherence linked asexual protein 9
PFIT_0207000	605,79478	542,40467	4416,689511	4812,006837	574,0997225	4614,348174	8,037537719	3,006753603	4,544E-43	4,279E-40	serine repeat antigen 4
PFIT_1001200	796,99247	712,92976	5689,989891	6145,110953	754,9611116	5917,550422	7,838218859	2,970525856	8,008E-43	6,464E-40	alpha/beta hydrolase, putative
PFIT_1252300	5194,5393	5599,3889	41976,7729	37809,00705	5396,964065	39892,88998	7,391727923	2,885911655	3,292E-42	2,325E-39	rhoptry neck protein 3 (RON3)
PFIT_0501700	2671,7361	2217,7896	17777,23223	17765,21635	2444,762852	17771,22429	7,269099446	2,861776643	1,546E-41	9,706E-39	rhoptry-associated protein 2
PFIT_0820600	2355,7568	2289,0826	18131,43296	15638,13111	2322,419672	16884,78203	7,270340603	2,862022953	3,983E-41	2,25E-38	rhoptry neck protein 5
PFIT_0731900	2870,9842	1931,6543	16450,40312	17654,71842	2401,319249	17052,56077	7,101330145	2,82808928	6,363E-41	2,996E-38	erythrocyte binding like protein 1, pseudogene
PFIT_0720400	1603,0417	1370,9447	9911,925946	11440,1007	1486,99316	10676,01332	7,17959814	2,843903095	6,238E-41	2,996E-38	rhoptry-associated leucine zipper-like protein 1
PFIT_0834500	2097,1367	2166,7284	15969,78348	14674,83863	2131,932576	15322,31105	7,18705236	2,845400197	9,206E-41	4,001E-38	cytoadherence linked asexual protein 8
PFIT_0536600	346,16844	393,07479	13,66690927	17,82224754	369,621615	15,74457841	0,042596476	-4,553122094	1,14E-40	4,603E-38	rifin (RIF)
PFIT_1453200	3153,7555	2570,4008	19213,39661	20789,65176	2862,07817	20001,52419	6,988461878	2,804974961	2,035E-40	7,664E-38	rhoptry neck protein 2
PFIT_1477400	451,83032	377,66009	2995,330948	3314,938043	414,7452061	3155,134495	7,607404374	2,927404294	4,206E-40	1,485E-37	Plasmodium exported protein (PHISTb), unknown function
PFIT_1234900	339,12432	321,78181	2375,764394	2680,466031	330,4530644	2528,115212	7,650451712	2,935544933	2,169E-39	7,21E-37	V-type K -independent H -translocating inorganic pyrophosphatase
PFIT_1407800	1176,3689	952,82099	7152,349183	7657,328657	1064,594958	7404,83892	6,955545735	2,798163714	3,227E-39	1,013E-36	glideosome associated protein with multiple membrane spans 3
PFIT_0616900	1133,0979	1067,4678	7697,886644	7640,397522	1100,282837	7669,142083	6,970155153	2,80119077	3,965E-39	1,179E-36	conserved Plasmodium membrane protein, unknown function
PFIT_1034800	2517,7717	1769,8	13911,77472	15060,69029	2143,785802	14486,23251	6,75731339	2,756449766	4,403E-39	1,244E-36	merozoite surface protein 3
PFIT_1035300	6236,0635	4513,6161	32666,19097	37881,18716	5374,83981	35273,68906	6,562742389	2,714298803	1,097E-38	2,951E-36	probable protein, unknown function
PFIT_1034600	31249,753	24053,672	181644,6132	180983,1416	27651,71226	181313,8774	6,557057867	2,713048627	1,402E-38	3,602E-36	S-antigen
PFIT_0620000	765,79705	672,46618	4949,698973	5086,469449	719,131615	5018,084211	6,977977474	2,80280894	1,77E-38	4,168E-36	conserved Plasmodium protein, unknown function
PFIT_1006800	315,97934	289,989	2396,264758	2221,543156	302,984166	2308,903957	7,620543302	2,929893858	1,766E-38	4,168E-36	rhoGAP GTPase, putative
PFIT_1013300	581,64349	613,69764	4366,577511	4104,463609	597,670567	4235,52056	7,08671431	2,82511689	2,535E-38	5,728E-36	conserved Plasmodium protein, unknown function
PFIT_0302100	376,35755	452,80674	3088,721494	2904,135237	414,5821456	2996,428366	7,227586614	2,853513993	7,623E-38	1,657E-35	cytoadherence linked asexual protein 3.2
PFIT_0206300	1375,617	1079,0288	7726,359372	8226,749466	1227,322934	7976,554419	6,499148837	2,700250787	4,597E-37	9,619E-35	merozoite surface protein 4
PFIT_1014400	1133,0979	1179,2244	7464,410278	7659,110882	1156,161115	7561,76058	6,540403826	2,709379715	5,072E-37	1,023E-34	conserved Plasmodium protein, unknown function
PFIT_1450400	809,06811	616,5879	4931,476427	4614,179889	712,8280037	4772,828158	6,695623816	2,743218474	6,952E-37	1,337E-34	conserved Plasmodium protein, unknown function
PFIT_1034500	499,12659	447,02623	3374,58768	3171,46895	473,0764105	3273,028315	6,918603935	2,790480954	7,099E-37	1,337E-34	probable protein, unknown function
PFIT_1127600	2018,6451	2080,9842	14279,64237	12202,89289	2049,814608	13241,26763	6,45973913	2,691475904	7,588E-37	1,383E-34	conserved Plasmodium protein, unknown function
PFIT_0216900	2712,9946	2207,192	15417,41256	15686,25118	2460,093273	15551,83187	6,321643182	2,660299606	1,083E-36	1,912E-34	calcium-dependent protein kinase 1
PFIT_0532600	724,5386	690,77113	4977,032791	4423,48184	707,6548675	4700,257316	6,64201934	2,731621924	2,075E-36	3,552E-34	cell cycle regulator with zn-finger domain, putative
PFIT_0627000	9124,1549	8034,9111	54102,73815	52128,29184	8579,532978	53115,515	6,190956446	2,63016231	2,468E-36	4,102E-34	HECT-domain (ubiquitin-transferase), putative
PFIT_0421700	2126,3195	2066,5329	13638,43654	12724,19363	2096,426212	13181,31509	6,287516829	2,652490356	3,83E-36	6,183E-34	glideosome associated protein with multiple membrane spans 2
PFIT_0403400	2262,1705	1823,7514	12875,36744	12662,70688	2042,960963	12769,03716	6,250259985	2,643916201	4,27E-36	6,701E-34	dipeptidyl peptidase 3
PFIT_0612300	884,54088	818,9058	5159,258248	5686,188079	851,7233415	5422,723164	6,366765943	2,670560728	8,684E-36	1,326E-33	rhoptry protein (ROP14)
PFIT_1322900	1455,115	1410,4448	8696,70993	9163,308575	1432,779925	8930,009252	6,232645431	2,639844642	9,973E-36	1,483E-33	glideosome associated protein with multiple membrane spans 1 (GAPM1)
PFIT_1116800	1685,5586	1479,811	9376,638666	10198,78116	1582,684758	9787,709912	6,184244753	2,628597418	1,133E-35	1,6E-33	rhoptry neck protein 4
PFIT_1410400	773,84748	609,84397	4564,747695	4385,164008	691,8457253	4474,955852	6,468141217	2,693351177	1,117E-35	1,6E-33	conserved Plasmodium membrane protein, unknown function
PFIT_0321500	509,18963	474,00195	3536,312773	3026,217633	491,5957888	3281,265203	6,674721951	2,73870774	1,446E-35	1,993E-33	protein kinase, putative
PFIT_0515800	2969,602	2780,4261	18338,71442	16893,70845	2875,014016	17616,21143	6,127348018	2,615262795	1,801E-35	2,423E-33	glideosome-associated protein 40, putative
PFIT_1413100	510,19593	570,34381	3580,730228	3457,516024	540,2698703	3519,123126	6,513639423	2,703463858	3,445E-35	4,527E-33	conserved Plasmodium protein, unknown function
PFIT_1141100	502,14551	603,10004	3749,288775	3421,871528	552,6227719	3585,580152	6,488296058	2,69783965	5,538E-35	7,111E-33	conserved Plasmodium protein, unknown function
PFIT_1016600	452,83663	427,75785	2839,3004	2923,73971	440,2972414	2881,520055	6,544488096	2,71028035	6,482E-35	8,138E-33	conserved Plasmodium protein, unknown function
PFIT_1200400	12936,033	12138,111	2011,31348	2107,480772	12537,07192	2059,397126	0,164264602	-2,605906475	8,399E-35	1,01E-32	acyl-CoA synthetase
PFIT_1314500	94,59254	112,71998	851,9040109	896,4590515	103,6562577	874,1815312	8,433466064	3,076125685	8,271E-35	1,01E-32	conserved Plasmodium protein, unknown function
PFIT_0805600	614,85151	615,62448	3829,012413	3936,934483	615,2379953	3882,973448	6,311335577	2,657945334	1,257E-34	1,48E-32	serine/threonine protein phosphatase, putative
PFIT_1331500	1985,437	1388,2862	9583,920123	10535,62164	1686,861614	10059,77088	5,963601753	2,576183918	1,302E-34	1,501E-32	conserved Plasmodium protein, unknown function
PFIT_0820500	1302,1569	1170,5536	8007,669921	7089,690073	1236,355235	7548,679997	6,105591486	2,610131065	1,589E-34	1,796E-32	conserved Plasmodium protein, unknown function
PFIT_0501600	588,68762	499,05083	3265,252406	3568,013958	543,8692234	3416,633182	6,282085904	2,651243671	2,508E-34	2,778E-32	rhoptry-associated protein 3
PFIT_1129700	384,40798	361,28197	2585,32367	2274,118787	372,8449769	2429,721228	6,516706348	2,704142987	6,926E-34	7,525E-32	conserved Plasmodium protein, unknown function
PFIT_1035700	1154,2302	883,45485	5661,517164	6403,533543	1018,842549	6032,525353	5,920959386	2,565830958	9,074E-34	9,673E-32	probable protein, unknown function
PFIT_1036700	600,76326	491,34348	3444,061135	3330,086954	546,0533708	3387,074044	6,202825998	2,632925655	1,094E-33	1,144E-31	dynamin-like protein
PFIT_1465000	1724,8044	1620,4701	10165,90268	9444,900086	1672,637236	9805,401381	5,862240281	2,551452102	1,37E-33	1,408E-31	conserved Plasmodium membrane protein, unknown function
PFIT_1140000	17382,889	17378,145	3330,170225	2737,497223	17380,51663	3033,833724	0,174553714	-2,518257043	1,496E-33	1,488E-31	transcription factor with AP2 domain(s)
PFIT_0506900	916,7426	848,77178	5096,618247	5421,527703	882,7571875	5259,072975	5,957553277	2,574719948	1,501E-33	1,488E-31	rhomboid protease ROM4
PFIT_1342300	387,42689	347,79411	2183,288755	2484,421308	367,6105022	2333,855031	6,348716964	2,666465062	1,788E-33	1,741E-31	conserved Plasmodium protein, unknown function
PFIT_1030800.1	297,86587	231,22046	1797,198569	1733,213574	264,5431664	1765,206071	6,672657982	2,738261558	1,911E-33	1,83E-31	OTU-like cysteine protease, putative
PFIT_1355900	3531,1194	3351,7333	20246,38717	19235,55177	3441,426338	19740,96947	5,736275467	2,520114307	2,05E-33	1,931E-31	serine/threonine protein kinase, putative
PFIT_1222800	4174,1474	3762,1496	23605,03012	21579,17733	3968,148503	22592,10372	5,693361452	2,509280694	3,593E-33	3,251E-31	cAMP-dependent protein kinase regulatory subunit
PFIT_0606600	584,6624	612,73422	3374,58768	3834,456559	598,6983131	3604,522119	6,020598422	2,589906892	3,617E-33	3,251E-31	conserved Plasmodium protein, unknown function
PFIT_0104300	391,45211	350,68437	2375,764394	2327,585529	371,0682373	2351,674962	6,337580868	2,663932251	3,625E-33	3,251E-31	conserved Plasmodium protein, unknown function
PFIT_1437800	970,07669	742,79573	4849,474971	5139,936192	856,4362108	4994,705582	5,831964504	2,543981939	7,538E-33	6,654E-31	cGMP-dependent protein kinase
PFIT_0404600	1258,8858	1315,0664	7832,277919	6946,22098	1286,976102	7389,24945	5,741559179	2,521442569	1,733E-32	1,506E-30	apical sushi protein
PFIT_1447800	621,89564	504,83134	3282,336042	3324,740279	563,3634889	3303,538161	5,863955024	2,551874038	5,162E-32	4,419E-30	centrin-2
PFIT_1220700	424,66013	402,70897	2356,402939	2609,17704	413,684549	2482,78999	6,001650281	2,585359255	5,598E-32	4,721E-30	histone-lysine N-methyltransferase, H3 lysine-4 specific (SET10)
PFIT_1303300	434,72316	380,55034	2327,930212	2515,610241	407,6367533	2421,770226	5,941000675	2,570705952	1,403E-31	1,165E-29	DNA ligase I
PFIT_0532500	396,48363	355,50146	2390,570213	2143,125267	375,9925429	2266,84774	6,028969943	2,591911537	2,038E-31	1,669E-29	conserved Plasmodium protein, unknown function
PFIT_1229500	2861,9275	2565,5837	14420,86709	14748,80096	2713,755599	14584,83402	5,374409556	2,426106266	2,553E-31	2,061E-29	myosin D
PFIT_1411800	124,78165	88,63451	848,4872836	759,2277454	106,7080795	803,8575145	7,533239453	2,913270388	3,023E-31	2,406E-29	ankyrin-repeat protein, putative
PFIT_1223800	560,51111	477,85562	2731,104035	3205,331221	519,1833683	2968,217628	5,717089201	2,5152808	3,088E-31	2,423E-29	conserved Plasmodium protein, unknown function
PFIT_0905700	377,36386	298,65976	2227,70621	1890,940464	338,01181	2059,323337	6,09245972	2,607024808	3,505E-31	2,712E-29	conserved Plasmodium protein, unknown function
PFIT_0814500	1623,1677	1483,6646	8196,728833	8587,649979	1553,416183	8392,189406	5,402408895	2,433602838	4,34E-31	3,313E-29	conserved Plasmodium protein, unknown function
PFIT_0932500	340,13062	282,28165	1756,197841	1983,616152	311,2061351	1869,906996	6,008580119	2,58702411	4,956E-31	3,734E-29	conserved Plasmodium protein, unknown function
PFIT_1431400	502,14551	497,12399	3023,803675	2750,863908	499,6347493	2887,333792	5,77888907	2,530792177	5,976E-31	4,442E-29	plasmepsin IX
PFIT_1441300	391,45211	318,89155	1988,535298	2195,700897	355,1718306	2092,118098	5,890439269	2,558375224	7,043E-31	5,168E-29	conserved Plasmodium protein, unknown function
PFIT_1450300	588,68762	518,3192	2883,717855	3308,700257	553,5034094	3096,209056	5,593839177	2,483838777	1,181E-30	8,556E-29	conserved Plasmodium protein, unknown function
PFIT_0920000	1822,4159	2071,35	10717,13468	10040,16315	1946,882912	10378,64892	5,330905549	2,414380621	1,42E-30	1,016E-28	regulator of chromosome condensation, putative
PFIT_1232300	1662,4136	1632,0311	9517,863395	8179,52051	1647,222335	8848,691953	5,37188682	2,425428909	1,481E-30	1,046E-28	CG2-related protein, putative
PFIT_1145900	2057,8909	1757,2755	9549,75285	11251,18487	1907,583205	10400,46886	5,452170493	2,446830677	1,84E-30	1,283E-28	serine/threonine protein kinase, putative
PFIT_0616700	25077,086	23806,073	129171,654	122233,8848	24441,57978	125702,7694	5,142988733	2,362606993	2,892E-30	1,993E-28	histone H2A
PFIT_0823900	470,95009	472,07511	2767,549126	2580,661444	471,5126015	2674,105285	5,671333654	2,503688035	3,161E-30	2,152E-28	conserved Plasmodium protein, unknown function
PFIT_0813200	3449,6088	2942,2804	16978,85695	16188,83856	3195,94459	16583,84775	5,189028559	2,375464476	3,671E-30	2,469E-28	protein phosphatase, putative
PFIT_1030800.2	308,93521	280,35481	1813,143296	1692,222404	294,6450104	1752,68285	5,948455899	2,572515222	4,159E-30	2,732E-28	OTU-like cysteine protease, putative
PFIT_0501000	206,29224	260,12302	1340,496017	1509,544367	233,2076307	1425,020192	6,110521289	2,611295462	4,127E-30	2,732E-28	serine/threonine protein kinase, FIKK family
PFIT_1436800	1776,1259	1733,19	9589,614669	8876,370389	1754,657966	9232,992529	5,26198992	2,395608485	4,241E-30	2,754E-28	conserved Plasmodium protein, unknown function
PFIT_1147200	2522,8032	2501,0347	14099,69473	12131,6039	2511,918917	13115,64932	5,221366513	2,384427432	5,648E-30	3,626E-28	CCAAT-box DNA binding protein subunit B
PFIT_1470300	322,01716	228,33021	1617,25093	1648,557898	275,1736819	1632,904414	5,934086437	2,569025942	5,765E-30	3,66E-28	shewanella-like protein phosphatase 1, putative (SHLP1)
PFIT_1024900	554,47329	476,8922	2801,7164	2885,421877	515,6827481	2843,569139	5,514183185	2,463147195	8,084E-30	5,019E-28	conserved Plasmodium membrane protein, unknown function
PFIT_0808600	605,79478	543,36809	3589,841501	4304,072782	574,5814318	3946,957141	6,869273741	2,780157577	8,077E-30	5,019E-28	serine/threonine protein kinase, FIKK family
PFIT_0813900	157,98967	177,26902	1104,741832	1063,988178	167,6293444	1084,365005	6,468825664	2,693503832	8,642E-30	5,307E-28	cullin-like protein, putative
PFIT_1312700	300,88478	311,18421	1940,701116	1660,142359	306,0344934	1800,421737	5,883068007	2,556568714	1,018E-29	6,183E-28	conserved Plasmodium membrane protein, unknown function
PFIT_0813800.1	140,88251	132,95177	936,1832848	903,5879505	136,9171361	919,8856176	6,718557253	2,748154161	1,167E-29	7,013E-28	conserved Plasmodium protein, unknown function
PFIT_0703700	1711,7225	1565,5552	8881,213205	8142,093791	1638,638833	8511,653498	5,194343823	2,376941512	1,251E-29	7,441E-28	conserved Plasmodium protein, unknown function
PFIT_0802900	22213,146	23129,754	4669,527333	4267,537174	22671,4498	4468,532254	0,197099537	-2,343003709	1,278E-29	7,52E-28	Plasmodium exported protein (PHISTa), unknown function
PFIT_1410300	202,26703	161,85432	1107,019651	1170,030551	182,0606753	1138,525101	6,253547611	2,644674857	1,711E-29	9,966E-28	DNA damage-inducible protein 1, putative (DDI1)
PFIT_0536400	160,00228	194,61056	4,55636422	5,346674263	177,3064154	4,951155343	0,027924288	-5,162335713	1,858E-29	1,071E-27	stevor, pseudogene
PFIT_1320300	1068,6944	1014,4798	5172,925157	5654,108033	1041,58711	5413,516595	5,19737288	2,377782567	1,944E-29	1,11E-27	conserved Plasmodium protein, unknown function
PFIT_1019400	382,39537	395,00162	2355,26403	2039,756231	388,6984987	2197,510131	5,653508152	2,499146376	2,236E-29	1,263E-27	autophagy-related protein 8 (ATG8)
PFIT_0629300	60,378217	66,475883	509,0923702	542,6874377	63,42704995	525,889904	8,291255929	3,051590652	2,281E-29	1,276E-27	conserved Plasmodium protein, unknown function
PFIT_0816000	242,51917	236,03756	1436,164382	1414,195343	239,2783634	1425,179862	5,956158518	2,57438215	2,835E-29	1,57E-27	conserved Plasmodium protein, unknown function
PFIT_1236600	167,0464	191,7203	1175,354197	1076,463752	179,3833502	1125,908974	6,276552274	2,649972301	3,587E-29	1,967E-27	conserved Plasmodium protein, unknown function
PFIT_0927700	437,74207	492,3069	2674,15858	2431,845677	465,0244871	2553,002129	5,490038051	2,456816148	4,44E-29	2,412E-27	conserved Plasmodium protein, unknown function
PFIT_0918100	1870,7184	2096,3989	10583,88232	9576,784718	1983,558641	10080,33352	5,081943791	2,345380419	5,208E-29	2,802E-27	secreted acid phosphatase
PFIT_1029300	1116,997	1053,9799	5102,312793	6028,375232	1085,488478	5565,344012	5,127041077	2,358126456	7,83E-29	4,174E-27	conserved Plasmodium protein, unknown function
PFIT_1006900	277,7398	356,46488	1891,728024	1706,480202	317,1023387	1799,104113	5,673575668	2,504258254	9,305E-29	4,913E-27	RING zinc finger protein, putative
PFIT_0935300	1671,4703	1700,4338	9332,221211	7909,51346	1685,952062	8620,867335	5,113352586	2,354269509	9,51E-29	4,975E-27	cAMP-dependent protein kinase catalytic subunit
PFIT_0903800.1	1345,4279	1135,8705	6615,922994	6002,532973	1240,649228	6309,227984	5,085424502	2,346368207	1,131E-28	5,862E-27	conserved Plasmodium protein, unknown function
PFIT_0206800	667,1793	613,69764	3063,665494	3549,300598	640,4384707	3306,483046	5,162842642	2,368165627	1,782E-28	9,154E-27	serine repeat antigen 6
PFIT_0702500	205,28594	237,00097	1341,634926	1266,270688	221,1434558	1303,952807	5,896441461	2,559837201	1,911E-28	9,729E-27	conserved Plasmodium protein, unknown function
PFIT_0619200	305,9163	264,94011	1740,253113	1503,30658	285,4282063	1621,779847	5,681918645	2,506378175	2,047E-28	1,032E-26	conserved Plasmodium protein, unknown function
PFIT_1336900	238,49396	225,43995	1348,468381	1342,906352	231,9669539	1345,687367	5,801202904	2,53635208	2,472E-28	1,236E-26	conserved Plasmodium protein, unknown function
PFIT_0524800	92,579933	73,219813	553,5098253	652,2942601	82,8998729	602,9020427	7,272653403	2,862481823	2,551E-28	1,264E-26	conserved protein, unknown function, putative
PFIT_0419200	150,94554	120,42732	798,375283	929,4302094	135,6864333	863,9027462	6,366905852	2,670592431	2,696E-28	1,324E-26	conserved Plasmodium protein, unknown function
PFIT_0529600	163,02119	152,22014	1018,18474	949,0346817	157,6206618	983,609711	6,240360242	2,641629315	2,786E-28	1,357E-26	G-protein coupled receptor, putative
PFIT_1467200	243,52548	193,64714	1178,770924	1342,906352	218,5863062	1260,838638	5,768150166	2,528108724	3,866E-28	1,867E-26	protein phosphatase containing kelch-like domains (PPKL)
PFIT_1472500	244,53178	203,28132	1209,52147	1364,29305	223,906551	1286,90726	5,747519464	2,522939447	4,018E-28	1,924E-26	diacylglycerol kinase, putative
PFIT_1133600	544,41026	475,92878	2499,905487	2804,330651	510,1695207	2652,118069	5,198503558	2,378096389	4,247E-28	2,016E-26	aquaglyceroporin (AQP)
PFIT_1003200	4557,5491	3828,6255	20064,16171	20260,33101	4193,087284	20162,24636	4,808449001	2,265571617	5,337E-28	2,492E-26	membrane skeletal protein IMC1-related
PFIT_1324600	802,02398	833,35708	3926,958596	4297,834995	817,6905326	4112,396796	5,029282634	2,330352632	5,322E-28	2,492E-26	conserved Plasmodium protein, unknown function
PFIT_0929600	14394,167	15601,601	124037,4518	102278,3142	14997,88381	113157,883	7,544923298	2,915506234	6,365E-28	2,948E-26	high molecular weight rhoptry protein 2
PFIT_1461100	155,97706	120,42732	814,3200105	918,7368609	138,2021924	866,5284357	6,27000499	2,648466591	6,457E-28	2,966E-26	conserved Plasmodium membrane protein, unknown function
PFIT_1326700	157,98967	146,43963	997,6843765	896,4590515	152,214647	947,071714	6,221948629	2,637366483	6,589E-28	3,002E-26	conserved Plasmodium protein, unknown function
PFIT_0110000	394,47102	308,29395	1823,393478	1947,971657	351,3824837	1885,682567	5,366467182	2,423972655	7,158E-28	3,236E-26	UMP-CMP kinase, putative
PFIT_1134000	94,59254	92,488185	609,3163715	679,9187438	93,54036244	644,6175576	6,891330553	2,78478256	1,182E-27	5,302E-26	LEM3/CDC50 family protein, putative
PFIT_1234600	287,80283	258,19618	1470,331655	1536,277738	272,9995086	1503,304697	5,506620522	2,461167192	1,376E-27	6,123E-26	conserved Plasmodium protein, unknown function
PFIT_0930300	399,50254	420,05051	2237,956392	2079,856288	409,7765211	2158,90634	5,268496922	2,397391426	1,739E-27	7,676E-26	conserved Plasmodium protein, unknown function
PFIT_1461800	112,70601	89,597929	692,4567362	668,3342829	101,1519671	680,3955095	6,726468392	2,749849243	2,067E-27	9,052E-26	inner membrane complex protein, putative
PFIT_0101000	4956,0453	3969,2846	22231,50574	20083,89076	4462,664957	21157,69825	4,741045642	2,245205282	2,156E-27	9,372E-26	erythrocyte binding antigen-181
PFIT_0303100	1473,2285	1620,4701	8078,282286	6907,903148	1546,849284	7493,092717	4,844100065	2,276228667	3,282E-27	1,416E-25	HAD superfamily protein, putative
PFIT_0908700	833,2194	788,07641	4228,769509	3791,683165	810,6479018	4010,226337	4,946939761	2,306536332	3,814E-27	1,633E-25	conserved Plasmodium protein, unknown function
PFIT_1111500	205,28594	209,06183	1228,882925	1123,692708	207,1738862	1176,287816	5,677780331	2,505327033	6,074E-27	2,58E-25	actin-like protein, putative (ALP1)
PFIT_0612800	1360,5225	1252,4442	5711,629164	6641,460547	1306,48333	6176,544856	4,727610918	2,241111307	6,507E-27	2,744E-25	transcription factor with AP2 domain(s)
PFIT_0908200	335,0991	313,11104	1679,890931	1726,084675	324,1050735	1702,987803	5,254431176	2,393534592	8,147E-27	3,41E-25	P1 nuclease, putative
PFIT_1360800	18969,829	16672,922	88049,06295	76858,44253	17821,37582	82453,75274	4,626677175	2,209976439	8,244E-27	3,425E-25	conserved Plasmodium protein, unknown function
PFIT_1355200	386,42059	380,55034	2124,065482	1854,404857	383,4854665	1989,235169	5,187250478	2,374970035	1,031E-26	4,25E-25	conserved Plasmodium protein, unknown function
PFIT_1034100	92,579933	76,110069	534,1483705	618,4319898	84,34500079	576,2901801	6,832535121	2,772420971	1,353E-26	5,539E-25	ADP-ribosylation factor, putative
PFIT_1243600	411,57818	414,26999	2290,346211	1941,73387	412,924087	2116,040041	5,124525565	2,357418444	1,604E-26	6,52E-25	conserved Plasmodium protein, unknown function
PFIT_1232500	216,35528	183,04953	1122,964378	1108,543797	199,7024051	1115,754088	5,587083876	2,482095479	1,894E-26	7,642E-25	conserved Plasmodium protein, unknown function
PFIT_1227300	469,94379	397,89188	2126,3433	2533,432488	433,9178339	2329,887894	5,36942184	2,424766752	2,106E-26	8,439E-25	cyclin related protein, putative
PFIT_1011600	161,00858	157,03723	945,2945576	911,6079619	159,0229046	928,4512597	5,838475041	2,545591599	2,207E-26	8,783E-25	conserved Plasmodium protein, unknown function
PFIT_0220200	324,02976	384,40402	3383,698953	2754,428358	354,2168915	3069,063655	8,664362792	3,115093652	2,489E-26	9,833E-25	cytoadherence linked asexual protein 2
PFIT_1138700	599,75696	476,8922	2961,163674	3554,647273	538,3245795	3257,905474	6,051935203	2,597396541	2,557E-26	1,003E-24	conserved Plasmodium protein, unknown function
PFIT_1459900	137,8636	151,25672	921,3774664	807,3478138	144,5601573	864,3626401	5,979259129	2,579966736	2,667E-26	1,039E-24	exonuclease V, mitochondrial, putative
PFIT_1246400	12221,557	10490,665	52009,42321	50575,97408	11356,11124	51292,69865	4,516748521	2,175284591	3,067E-26	1,187E-24	actin I
PFIT_0316500	116,73122	93,451603	648,0392811	688,8298676	105,0914115	668,4345743	6,360506198	2,669141586	3,213E-26	1,235E-24	conserved Plasmodium protein, unknown function
PFIT_1363100	566,54894	620,44157	2613,796397	3067,208802	593,4952549	2840,5026	4,786057809	2,258837823	5,756E-26	2,197E-24	cysteine-rich surface protein
PFIT_0818700	1102,9088	1005,809	4497,552058	5236,176328	1054,358887	4866,864193	4,615946478	2,206626496	6,209E-26	2,354E-24	vacuolar sorting protein VPS9, putative
PFIT_0536700	527,3031	629,11234	91,11272844	81,9823387	578,2077181	86,54753357	0,149682425	-2,740023255	6,595E-26	2,484E-24	rifin, pseudogene
PFIT_0809200	277,7398	285,1719	1400,8582	1513,999929	281,4558508	1457,429064	5,178180024	2,372445123	6,884E-26	2,576E-24	ferlin like protein, putative
PFIT_0104500	360,2567	359,35513	1792,642932	1829,45371	359,8059149	1811,048321	5,033403416	2,331534231	7,193E-26	2,674E-24	conserved Plasmodium protein, unknown function
PFIT_1437400	1412,8503	1526,055	6614,784085	6858,000855	1469,452664	6736,39247	4,584286812	2,196697308	7,534E-26	2,782E-24	conserved Plasmodium protein, unknown function
PFIT_1232400	168,0527	165,708	974,9061943	914,281299	166,880351	944,5937467	5,66030537	2,500879888	1,033E-25	3,792E-24	conserved Plasmodium protein, unknown function
PFIT_1251400	4852,396	4268,9078	21938,8061	19041,28928	4560,651912	20490,04769	4,492789207	2,167611375	1,153E-25	4,202E-24	coronin
PFIT_0421600	152,95815	96,341859	749,4021914	897,3501639	124,6500045	823,3761777	6,605504595	2,723668773	1,543E-25	5,589E-24	asparagine-rich protein
PFIT_1116900	2430,2232	2369,0463	10852,66487	10624,73287	2399,634777	10738,69887	4,475138873	2,161932453	1,928E-25	6,937E-24	serine esterase, putative
PFIT_0825800	4344,2127	3652,3199	18384,27078	17136,98213	3998,266299	17760,62645	4,442081924	2,151236	2,005E-25	7,153E-24	conserved Plasmodium protein, unknown function
PFIT_1321700	211,32376	200,39107	1141,186924	1076,463752	205,8574134	1108,825338	5,386375547	2,42931482	2,013E-25	7,153E-24	DHHC-type zinc finger protein, putative
PFIT_1316300	681,26755	675,35643	3144,52804	3204,440108	678,311991	3174,484074	4,679976348	2,226501239	2,305E-25	8,139E-24	DNA replication licensing factor MCM4 (MCM4)
PFIT_0308000	582,64979	551,07543	2702,631307	2674,228244	566,8626146	2688,429776	4,742647877	2,245692758	2,435E-25	8,546E-24	conserved Plasmodium protein, unknown function
PFIT_1326600	183,14726	186,90321	994,2676491	1010,521436	185,0252326	1002,394542	5,417609957	2,43765653	3,887E-25	1,356E-23	conserved Plasmodium protein, unknown function
PFIT_0801400	3911,5022	4131,1389	905,4327389	850,1212079	4021,320542	877,7769734	0,218280777	-2,195743009	4,043E-25	1,401E-23	Plasmodium exported protein (PHISTa), unknown function
PFIT_0531200	1003,2847	937,40629	4088,683689	4658,735508	970,3454983	4373,709598	4,507373514	2,172287007	4,576E-25	1,576E-23	DNA replication licensing factor MCM3, putative (MCM3)
PFIT_1243900	2022,6703	1891,1907	9096,467026	8323,880716	1956,930484	8710,173871	4,450936783	2,15410901	4,906E-25	1,68E-23	ubiquitin conjugating enzyme E2, putative
PFIT_0201500	11901,553	11293,193	2712,881489	2569,968096	11597,37281	2641,424793	0,227760618	-2,134409785	6,849E-25	2,331E-23	Plasmodium exported protein, unknown function
PFIT_0417200	2251,1012	1993,3131	10097,56813	8714,187937	2122,20713	9405,878033	4,432120646	2,147997153	7,024E-25	2,377E-23	regulator of chromosome condensation, putative
PFIT_0914500	442,77359	556,85595	2495,34985	2244,712078	499,8147689	2370,030964	4,741818593	2,245440471	8,742E-25	2,94E-23	conserved Plasmodium protein, unknown function
PFIT_1035000	208,30485	128,13467	1185,604379	1447,166501	168,2197608	1316,38544	7,825391224	2,96816288	9,152E-25	3,06E-23	merozoite surface protein
PFIT_1469000	1419,8944	1197,5293	5561,293162	5982,928501	1308,711857	5772,110831	4,410528414	2,140951512	9,978E-25	3,316E-23	rab GTPase activator, putative
PFIT_1247700	435,72947	435,4652	2026,119299	2101,242985	435,597335	2063,681142	4,737588999	2,244153046	1,054E-24	3,483E-23	serine/threonine protein kinase, putative
PFIT_1125500	1491,342	1357,4568	6078,357896	6436,504701	1424,399379	6257,431298	4,393031472	2,135216835	1,202E-24	3,95E-23	ethanolamine kinase (EK)
PFIT_1029200	64,403432	61,65879	424,8130964	462,4873238	63,0311107	443,6502101	7,038591025	2,815286661	1,277E-24	4,172E-23	RAP protein, putative
PFIT_0628200	621,89564	503,86792	2685,547671	2521,848028	562,8817796	2603,697849	4,625656654	2,209658184	1,514E-24	4,916E-23	phospholipase, putative
PFIT_0715700	686,29907	620,44157	2824,494582	3103,74441	653,3703202	2964,119496	4,536660764	2,181630784	1,557E-24	5,028E-23	conserved Plasmodium protein, unknown function
PFIT_0510100	877,49675	820,83264	3446,338953	4085,75025	849,1646974	3766,044601	4,434999021	2,148933786	1,851E-24	5,944E-23	conserved Plasmodium protein, unknown function
PFIT_0103800	161,00858	118,50049	1013,629104	1248,44844	139,7545328	1131,038772	8,093038197	3,016681405	2,004E-24	6,397E-23	cyclase-associated protein, putative
PFIT_1312000	1478,26	1282,3101	5863,104075	6180,755448	1380,28508	6021,929762	4,362815949	2,125259613	2,026E-24	6,432E-23	conserved Plasmodium protein, unknown function
PFIT_1133900	378,37016	356,46488	1618,389839	1863,315981	367,4175196	1740,85291	4,738078118	2,244301985	2,189E-24	6,91E-23	conserved Plasmodium protein, unknown function
PFIT_1436400	184,15356	174,37877	1035,268377	883,9834782	179,2661636	959,6259276	5,353078955	2,420368932	2,307E-24	7,242E-23	conserved Plasmodium protein, unknown function
PFIT_1224200	144,90772	145,47621	791,5418284	808,2389261	145,1919642	799,8903772	5,509191792	2,461840689	3,135E-24	9,787E-23	histone chaperone ASF1, putative (ASF1)
PFIT_0508800	129,81317	119,46391	658,289463	753,8810711	124,638536	706,0852671	5,665063868	2,502092221	3,155E-24	9,794E-23	single-stranded DNA-binding protein
PFIT_1309400	145,91402	107,90288	722,0683729	717,3454637	126,9084534	719,7069183	5,671071539	2,503621356	3,241E-24	1,001E-22	conserved Plasmodium protein, unknown function
PFIT_0615200	181,13465	153,18356	827,9869197	938,3413332	167,1591036	883,1641265	5,283374386	2,401459644	4,433E-24	1,361E-22	Ndc80 homologue, putative
PFIT_1436500	1964,3047	1593,4944	7138,682274	8000,406923	1778,899506	7569,544598	4,255183933	2,089221495	4,788E-24	1,462E-22	NAD(P)H-dependent glutamate synthase, putative
PFIT_0914200	1126,0537	1024,114	4496,413149	4845,869107	1075,083856	4671,141128	4,344908635	2,11932584	4,848E-24	1,473E-22	conserved Plasmodium protein, unknown function
Pf_M76611101900	2643,5596	2188,887	9391,444484	10956,22668	2416,223322	10173,83558	4,210635453	2,074037976	5,158E-24	1,559E-22	apocytochrome b
PFIT_1474500	706,42514	728,34446	3491,895318	2948,690856	717,3847975	3220,293087	4,488934109	2,16637292	6,514E-24	1,958E-22	conserved Plasmodium protein, unknown function
PFIT_1320700.1	446,79881	384,40402	1912,228388	1934,604971	415,6014122	1923,41668	4,628032108	2,210398873	6,786E-24	2,029E-22	3',5'-cyclic nucleotide phosphodiesterase, putative
PFIT_0808100	922,78042	849,7352	4563,608786	3827,32766	886,2578077	4195,468223	4,733913977	2,24303349	6,957E-24	2,069E-22	conserved Plasmodium protein, unknown function
PFIT_1424100	208,30485	225,43995	1185,604379	1021,214784	216,8723997	1103,409582	5,087828526	2,347050049	7,471E-24	2,21E-22	ankyrin, putative
PFIT_1117300	446,79881	463,40434	2272,123666	1941,73387	455,1015745	2106,928768	4,629579167	2,210881057	8,09E-24	2,381E-22	folate transporter 2
PFIT_1238300	261,63894	242,78149	1263,050198	1211,912833	252,2102129	1237,481516	4,906548	2,294708374	1,048E-23	3,067E-22	sphingomyelin phosphodiesterase, putative
PFIT_0903500	962,02626	1022,1871	4187,768781	4377,143997	992,1066922	4282,456389	4,316528074	2,109871371	1,2E-23	3,495E-22	conserved Plasmodium membrane protein, unknown function
PFIT_0409300	740,63946	674,39301	2904,218219	3276,620211	707,5162384	3090,419215	4,367983443	2,126967388	1,243E-23	3,602E-22	GTPase, putative
PFIT_0311200	660,13517	633,92943	2993,053129	2738,388335	647,0323033	2865,720732	4,429022659	2,146988378	1,721E-23	4,96E-22	phosphatidylinositol 3- and 4-kinase, putative
PFIT_1418400	1225,6778	1303,5054	5545,348435	5229,938542	1264,591581	5387,643488	4,260382223	2,090982868	2,195E-23	6,264E-22	cyclic nucleotide-binding protein, putative
PFIT_0617000	400,50884	395,96504	1980,562935	1696,677966	398,2369405	1838,62045	4,616900803	2,206924735	2,186E-23	6,264E-22	conserved Plasmodium protein, unknown function
PFIT_0713300	303,90369	312,14762	1394,024745	1490,831007	308,0256581	1442,427876	4,682817285	2,227376748	2,619E-23	7,436E-22	conserved Plasmodium protein, unknown function
PFIT_0732000	639,0028	383,4406	2379,181121	2175,205313	511,2216983	2277,193217	4,454414248	2,155235731	2,819E-23	7,963E-22	serine/threonine protein kinase, FIKK family
PFIT_0413400	236,48135	200,39107	1230,021834	1024,779234	218,4362086	1127,400534	5,161234674	2,367716229	5,127E-23	1,441E-21	conserved Plasmodium protein, unknown function
PFIT_0403900	612,8389	635,85627	2733,381853	2695,614941	624,3475869	2714,498397	4,347735867	2,120264297	5,366E-23	1,496E-21	ag-1 blood stage membrane protein homologue
PFIT_0212300	313,96673	350,68437	2289,207302	1875,791554	332,3255481	2082,499428	6,266443974	2,647646987	5,376E-23	1,496E-21	Leu/Phe-tRNA protein transferase, putative
PFIT_0522600	945,9254	1035,675	4246,992055	4144,563666	990,8001934	4195,77786	4,234736618	2,082272243	5,681E-23	1,573E-21	conserved Plasmodium protein, unknown function, putative
PFIT_1433400	334,0928	329,48916	1416,802927	1599,546717	331,7909797	1508,174822	4,545557035	2,1844571	7,932E-23	2,186E-21	conserved Plasmodium protein, unknown function
PFIT_0915100	183,14726	183,04953	903,1549207	925,8657599	183,0983954	914,5103403	4,994638747	2,320380334	8,402E-23	2,304E-21	type II NADH:ubiquinone oxidoreductase
PFIT_0201400	7407,4009	7750,7026	1871,22766	1818,760362	7579,05175	1844,994011	0,243433357	-2,038401224	9,871E-23	2,694E-21	liver stage associated protein 2 (LSAP2)
PFIT_1421200	939,88758	922,95501	4231,047327	3658,016308	931,4212949	3944,531818	4,234959883	2,082348303	1,024E-22	2,78E-21	tetratricopeptide repeat family protein, putative
PFIT_bin05700	203,27333	162,81774	903,1549207	916,9546361	183,0455364	910,0547784	4,9717398	2,313750794	1,028E-22	2,78E-21	cytoadherence linked asexual protein 2,cytoadherence linked asexual protein 8 (CLAG2)
PFIT_1303400.2	187,17247	203,28132	977,1840126	949,9257941	195,2268979	963,5549033	4,935564278	2,303215036	1,084E-22	2,917E-21	CorA-like Mg2 transporter protein, putative
PFIT_1142500	201,26072	210,98867	1091,074923	937,4502208	206,1246976	1014,262572	4,920626125	2,298841903	1,101E-22	2,948E-21	phd finger protein, putative
PFIT_1204800	65,409735	80,927162	499,9810973	423,2783792	73,16844843	461,6297383	6,309136631	2,657442594	1,311E-22	3,494E-21	conserved Plasmodium protein, unknown function
PFIT_1402800	239,50026	260,12302	1255,077834	1115,672696	249,8116404	1185,375265	4,745076184	2,24643125	1,448E-22	3,84E-21	conserved Plasmodium protein, unknown function
PFIT_0729300	549,44178	388,25769	2023,841481	2059,360704	468,8497339	2041,601092	4,354489178	2,122503487	1,478E-22	3,901E-21	conserved Plasmodium protein, unknown function
PFIT_0611700	598,75065	564,56329	2431,57094	2545,016949	581,6569736	2488,293945	4,277940535	2,096916429	1,601E-22	4,207E-21	6-cysteine protein
PFIT_1473700	456,86184	499,05083	4002,126597	3225,826806	477,9563365	3613,976701	7,561311411	2,918636473	1,614E-22	4,222E-21	protein disulfide isomerase
PFIT_1413000	383,40168	464,36776	1957,784752	1797,373665	423,8847198	1877,579209	4,429457164	2,147129905	1,876E-22	4,886E-21	p1/s1 nuclease, putative
PFIT_0107600	744,66468	760,13727	3346,114952	2998,593149	752,400973	3172,354051	4,216307746	2,075980172	2,569E-22	6,659E-21	LCCL domain-containing protein
PFIT_0422000	1932,1029	1682,1289	7496,299733	7121,770119	1807,115904	7309,034926	4,04458558	2,015991888	2,668E-22	6,884E-21	cysteine-rich protective antigen (CyRPA)
PFIT_0612400	239,50026	205,20816	963,5171033	1125,474932	222,3542105	1044,496018	4,697442047	2,231875363	2,809E-22	7,214E-21	apicoplast ribosomal protein L18 precursor, putative
PFIT_0526600	1015,3603	1037,6018	4282,298237	4165,950363	1026,481087	4224,1243	4,115150639	2,040945243	3,18E-22	8,13E-21	conserved Plasmodium protein, unknown function, putative
PFIT_0207100	357,23778	341,05018	1411,108382	1662,815696	349,1439829	1536,962039	4,402086572	2,138187518	3,541E-22	9,012E-21	serine repeat antigen 3
PFIT_0533600.2	601,76956	544,3315	2216,317119	2590,463681	573,0505339	2403,3904	4,194028725	2,068336743	3,957E-22	1,003E-20	conserved Plasmodium protein, unknown function
PFIT_0206700	272,70828	216,76918	1050,074195	1203,001709	244,7387318	1126,537952	4,603022759	2,202581576	4,032E-22	1,017E-20	serine repeat antigen 7
PFIT_0524600	1033,4738	1086,7362	4507,80224	4183,772611	1060,104993	4345,787425	4,099393411	2,035410449	4,543E-22	1,139E-20	conserved Plasmodium protein, unknown function
PFIT_0927400	119,75013	116,57365	608,1774624	650,5120354	118,1618901	629,3447489	5,326122903	2,41308572	4,555E-22	1,139E-20	fumarate hydratase, putative
PFIT_0831500	69,43495	64,549046	384,9512777	450,9028629	66,99199763	417,9270703	6,238462578	2,641190532	4,742E-22	1,18E-20	folate transporter 1
PFIT_1107900	78,491682	70,329557	443,0356421	458,0317619	74,41061969	450,533702	6,054696277	2,598054591	5,282E-22	1,309E-20	nucleic acid binding protein, putative
PFIT_1113800	870,45263	756,28359	3214,001496	3453,060462	813,3681119	3333,530979	4,098428411	2,035070798	5,361E-22	1,323E-20	conserved Plasmodium protein, unknown function
PFIT_1019000.2	123,77534	126,20784	625,2610989	683,4831933	124,9915902	654,3721461	5,235329392	2,38828031	5,446E-22	1,338E-20	conserved Plasmodium membrane protein, unknown function
PFIT_1028100	4018,1703	3475,0509	15646,33329	13948,58204	3746,610603	14797,45767	3,949558477	1,981691383	6,475E-22	1,584E-20	merozoite TRAP-like protein
PFIT_0608700	271,70198	223,51311	1068,296741	1188,743911	247,607545	1128,520326	4,55769765	2,188305222	7,614E-22	1,854E-20	zinc finger protein, putative
PFIT_1426400	975,10821	1086,7362	4519,191331	3888,814414	1030,922189	4204,002872	4,077905121	2,027828209	9,576E-22	2,322E-20	DNA-directed DNA polymerase, putative
PFIT_1469500	1037,499	975,94303	7144,376819	5797,577126	1006,721032	6470,976973	6,427775689	2,684319584	9,763E-22	2,357E-20	zinc finger protein, putative
PFIT_1005400	96,605147	115,61023	757,3745552	617,5408774	106,1076892	687,4577163	6,478868042	2,695741774	1,028E-21	2,472E-20	conserved Plasmodium membrane protein, unknown function
PFIT_1354200	676,23603	700,40532	2643,408034	2981,662014	688,3206737	2812,535024	4,086082449	2,030718315	1,15E-21	2,753E-20	DNA replication licensing factor MCM6 (MCM6)
PFIT_0319000	970,07669	814,08871	3290,308406	3851,387694	892,0826987	3570,84805	4,002821773	2,001017381	1,252E-21	2,984E-20	P-type ATPase, putative
PFIT_1416300	157,98967	162,81774	808,6254649	768,1388692	160,403705	788,382167	4,914987263	2,297187678	1,499E-21	3,558E-20	RNA-binding protein Nova-1, putative
PFIT_0613700	254,59482	291,91583	1790,365114	1463,206523	273,2553243	1626,785819	5,953354515	2,573702808	1,544E-21	3,65E-20	conserved Plasmodium protein, unknown function
PFIT_1459500	667,1793	571,30723	2412,209486	2657,297109	619,2432617	2534,753297	4,093307839	2,033267169	1,559E-21	3,671E-20	conserved Plasmodium protein, unknown function
PFIT_0920800	939,88758	895,97929	4986,144064	4118,721407	917,9334346	4552,432736	4,959436669	2,310176258	2,27E-21	5,323E-20	conserved Plasmodium protein, unknown function
PFIT_1209000	1196,495	1034,7116	4598,914968	4269,319399	1115,603284	4434,117184	3,974636186	1,990822811	2,498E-21	5,832E-20	conserved Plasmodium protein, unknown function
PFIT_1130100	832,21309	790,00325	3147,944768	3348,800314	811,1081685	3248,372541	4,004857387	2,001750869	2,592E-21	6,027E-20	conserved Plasmodium protein, unknown function
PFIT_0704200	526,29679	643,56362	2397,403667	2413,132317	584,9302057	2405,267992	4,112059813	2,03986125	2,661E-21	6,161E-20	origin recognition complex subunit 2, putative
PFIT_1320700.2	388,4332	322,74523	1440,720019	1587,962256	355,5892123	1514,341137	4,258681323	2,090406777	2,926E-21	6,747E-20	3',5'-cyclic nucleotide phosphodiesterase, putative
PFIT_0822500	630,95237	723,52736	2955,469129	2584,225894	677,2398654	2769,847511	4,089906181	2,032067749	3,209E-21	7,37E-20	conserved Plasmodium protein, unknown function
PFIT_1346100	67,422342	56,841697	389,5069141	385,8516593	62,13201965	387,6792867	6,239605423	2,641454799	3,35E-21	7,663E-20	conserved Plasmodium protein, unknown function
PFIT_1307100	264,65785	263,01328	1095,63056	1233,29953	263,8355635	1164,465045	4,413601523	2,141956383	3,41E-21	7,768E-20	tripartite motif protein, putative
PFIT_1009200	407,55297	372,843	1602,445112	1689,549067	390,1979801	1645,997089	4,218363942	2,07668357	3,526E-21	8,001E-20	tubulin-tyrosine ligase, putative
PFIT_0729400	817,11854	752,42992	2887,134583	3339,88819	784,7742289	3113,511886	3,96739823	1,988193216	3,786E-21	8,555E-20	conserved Plasmodium protein, unknown function
PFIT_1034000	82,516897	104,04921	528,453825	499,9140436	93,28305228	514,1839343	5,512083082	2,462597633	4,052E-21	9,121E-20	conserved Plasmodium protein, unknown function
PFIT_1143800	4950,0075	4650,4215	19566,45843	17197,57777	4800,214519	18382,0181	3,829415962	1,937124378	4,351E-21	9,756E-20	transcription factor with AP2 domain(s)
PFIT_1325200	90,567326	124,281	585,3992803	554,2718986	107,424162	569,8355894	5,304538374	2,407227206	4,385E-21	9,793E-20	conserved Plasmodium protein, unknown function
PFIT_1303200	240,50656	192,68372	955,5447396	1152,208304	216,5951415	1053,876522	4,865651715	2,282633055	4,51E-21	1,003E-19	chromosome condensation protein, putative
PFIT_1019000.1	151,95185	119,46391	625,2610989	713,7810141	135,7078758	669,5210565	4,933546064	2,302624979	4,85E-21	1,075E-19	conserved Plasmodium membrane protein, unknown function
PFIT_0903800.2	109,68709	72,256394	519,3425521	482,0917961	90,97174435	500,7171741	5,504095559	2,460505518	5,22E-21	1,152E-19	conserved Plasmodium protein, unknown function
PFIT_0812300	295,85326	303,47686	1390,608018	1227,061743	299,66506	1308,834881	4,367659281	2,126860317	5,456E-21	1,199E-19	conserved Plasmodium protein, unknown function
PFIT_1321800	71,447557	70,329557	402,0349143	433,0806153	70,88855703	417,5577648	5,890340872	2,558351125	6,206E-21	1,359E-19	conserved Plasmodium protein, unknown function
PFIT_0722000	688,31167	684,99062	2683,269853	2800,766202	686,6511466	2742,018027	3,993320394	1,997588827	6,424E-21	1,401E-19	conserved Plasmodium protein, unknown function
PFIT_1013100	501,1392	462,44092	1898,561479	2034,409557	481,7900628	1966,485518	4,081623242	2,029143019	7,461E-21	1,621E-19	conserved Plasmodium protein, unknown function
PFIT_0207300	300,88478	269,75721	1196,99347	1260,924014	285,3209937	1228,958742	4,307284669	2,106778675	9,399E-21	2,035E-19	serine repeat antigen 1
PFIT_1440600	244,53178	265,90353	1137,770196	1108,543797	255,2176552	1123,156997	4,400780957	2,137759565	9,519E-21	2,053E-19	ubiquinol-cytochrome c reductase iron-sulfur subunit, putative
PFIT_1319900	531,32831	569,38039	2073,953481	2342,73444	550,3543489	2208,34396	4,012585645	2,004532186	9,904E-21	2,128E-19	conserved Plasmodium protein, unknown function
PFIT_0206400	2923,312	2501,9981	10229,68159	10220,16785	2712,655046	10224,92472	3,769342046	1,914312717	1,108E-20	2,371E-19	conserved Plasmodium protein, unknown function
PFIT_1456800	233,46244	239,89123	987,4341945	1089,830437	236,6768343	1038,632316	4,388398716	2,13369461	1,406E-20	2,997E-19	ferlin, putative
PFIT_1303400.1	238,49396	200,39107	1005,65674	951,7080189	219,4425122	978,6823795	4,459857707	2,156997681	1,631E-20	3,465E-19	CorA-like Mg2 transporter protein, putative
PFIT_0418300	317,99194	276,50114	1342,773835	1202,110597	297,2465395	1272,442216	4,280763767	2,097868223	1,722E-20	3,643E-19	apical merozoite protein
PFIT_0908400	720,51339	648,38071	2831,328036	2581,552557	684,4470513	2706,440297	3,954199659	1,983385719	1,855E-20	3,911E-19	conserved Plasmodium protein, unknown function
PFIT_0210000	291,82805	259,1596	1265,328016	1113,890472	275,4938252	1189,609244	4,318097668	2,110395875	2,002E-20	4,189E-19	syntaxin, Qa-SNARE family
PFIT_1028000	178,11574	163,78116	807,4865558	779,7233301	170,9484505	793,6049429	4,642364062	2,214859666	2,001E-20	4,189E-19	conserved Plasmodium protein, unknown function
PFIT_1300800	11454,754	9070,586	40685,24998	35296,96126	10262,67006	37991,10562	3,70187343	1,888255569	2,443E-20	5,093E-19	erythrocyte binding antigen-140
PFIT_0610600	326,04237	299,62318	1293,800744	1331,321892	312,8327771	1312,561318	4,195728242	2,068921237	2,511E-20	5,215E-19	conserved Plasmodium protein, unknown function
PFIT_0905600	11228,336	12951,236	92924,73283	74451,548	12089,78595	83688,14042	6,922218539	2,791234489	2,561E-20	5,3E-19	high molecular weight rhoptry protein 3
PFIT_1025400	856,36438	969,1991	3611,480774	3437,911551	912,781741	3524,696162	3,861488463	1,949157061	2,92E-20	6,021E-19	conserved Plasmodium protein, unknown function
PFIT_0526800	93,586236	65,512464	588,8160076	742,2966102	79,54935034	665,5563089	8,366583838	3,064638676	2,941E-20	6,043E-19	inner membrane complex protein
PFIT_0907700	125,78795	105,01263	571,732371	582,7874947	115,4002894	577,2599328	5,002239908	2,322574251	3,028E-20	6,198E-19	conserved Plasmodium protein, unknown function
PFIT_1136900	593,71913	721,60053	3764,094594	3089,486612	657,6598299	3426,790603	5,2105822	2,38144458	3,75E-20	7,649E-19	conserved Plasmodium protein, unknown function
PFIT_0533600.1	787,93573	719,67369	2642,269125	3120,675545	753,8047103	2881,472335	3,822571411	1,934543453	4,089E-20	8,311E-19	conserved Plasmodium protein, unknown function
PFIT_0806000	659,12887	690,77113	2602,407306	2645,712648	674,9499999	2624,059977	3,887784247	1,958948159	4,122E-20	8,348E-19	filament assembling protein, putative
PFIT_1415400	942,90649	753,39334	3524,923682	4254,170489	848,1499142	3889,547085	4,585919329	2,197210977	4,15E-20	8,375E-19	serine/threonine protein phosphatase
PFIT_0412000	83,5232	90,561348	421,3963691	515,9540664	87,04227395	468,6752177	5,384455121	2,428800359	4,376E-20	8,798E-19	phosphoglucomutase-2
PFIT_1447900	132,83208	112,71998	635,5112809	574,7674833	122,7760264	605,1393821	4,928807357	2,301238594	4,435E-20	8,886E-19	conserved Plasmodium protein, unknown function
PFIT_1350900	6252,1644	5157,1797	21223,57118	20432,3157	5704,672049	20827,94344	3,651032568	1,868304538	5,072E-20	1,013E-18	alveolin, putative
PFIT_0402300	267,67676	210,98867	943,0167394	1129,930494	239,3327169	1036,473617	4,330680862	2,114593861	5,694E-20	1,133E-18	conserved Plasmodium protein, unknown function
PFIT_0903900	685,29276	597,31953	2745,909853	3339,88819	641,3061453	3042,899522	4,744846972	2,246361559	6,586E-20	1,306E-18	alpha tubulin 1
PFIT_1417200	1039,5116	1232,2124	4282,298237	4261,299388	1135,862008	4271,798812	3,760843115	1,911056125	7,133E-20	1,409E-18	metacaspase-like protein
PFIT_0515500	1513,4806	1757,2755	6333,473536	5854,608318	1635,378076	6094,040927	3,726380472	1,897774984	7,59E-20	1,494E-18	conserved Plasmodium protein, unknown function
PFIT_0414300	123,77534	109,82972	596,7883713	555,163011	116,8025322	575,9756912	4,931191818	2,301936373	8,077E-20	1,584E-18	conserved Plasmodium protein, unknown function
PFIT_1029600	1027,436	1068,4312	5293,649523	4360,212862	1047,933606	4826,931192	4,606142187	2,203558947	1,214E-19	2,373E-18	conserved Plasmodium protein, unknown function
PFIT_0202300	700,38732	552,03885	2293,762939	2473,727959	626,2130855	2383,745449	3,80660434	1,928504623	1,373E-19	2,675E-18	conserved Plasmodium protein, unknown function
PFIT_1231100	131,82577	130,06151	666,2618267	584,5697195	130,9436419	625,4157731	4,776221006	2,255869595	1,389E-19	2,697E-18	conserved Plasmodium protein, unknown function
PFIT_0316100	408,55927	402,70897	1774,420386	1481,919883	405,6341201	1628,170135	4,013888513	2,005000546	1,401E-19	2,711E-18	mitochondrial ribosomal protein L27 precursor, putative
PFIT_1369300	104036,7	106242,91	33267,53497	26412,57086	105139,8058	29840,05292	0,283813088	-1,816986976	1,531E-19	2,953E-18	membrane associated histidine-rich protein
PFIT_1031200	137,8636	137,76886	702,7069181	598,8275175	137,8162271	650,7672178	4,721992696	2,23939581	1,543E-19	2,965E-18	conserved Plasmodium protein, unknown function
PFIT_0413300	262,64524	256,26935	1110,436378	1063,097066	259,4572948	1086,766722	4,188615019	2,06647329	1,624E-19	3,111E-18	GTP binding protein, putative
PFIT_0402600	424,66013	397,89188	1731,14184	1535,386626	411,2760025	1633,264233	3,971212089	1,989579412	1,928E-19	3,68E-18	SET domain protein, putative
PFIT_1457200	190,19138	179,19586	741,4298277	855,4678821	184,6936209	798,4488549	4,323099255	2,11206596	3,272E-19	6,224E-18	transcription factor with AP2 domain(s)
PFIT_1008200	1917,0084	1790,0317	6795,870633	6560,369321	1853,520068	6678,119977	3,602939128	1,849174278	3,581E-19	6,789E-18	tubulin beta chain
PFIT_1404500	50,315181	43,353837	315,4778222	283,373736	46,83450876	299,4257791	6,393272547	2,676554598	3,66E-19	6,917E-18	selenoprotein (Sel1)
PFIT_1441200	202,26703	228,33021	879,2378295	1068,44374	215,2986167	973,8407849	4,523209669	2,177346872	3,977E-19	7,49E-18	conserved Plasmodium protein, unknown function
PFIT_1338600	135,85099	127,17125	649,1781902	581,0052699	131,5111213	615,0917301	4,677108097	2,225616773	4,024E-19	7,529E-18	conserved Plasmodium protein, unknown function
PFIT_1360700	74,466468	72,256394	413,4240053	386,7427717	73,36143105	400,0833885	5,453593023	2,447207042	4,015E-19	7,529E-18	cytochrome c oxidase subunit 2, putative
PFIT_0504200	224,40571	224,47653	1040,962922	864,3790059	224,4411193	952,6709642	4,244636487	2,085641006	4,298E-19	8,015E-18	ATP-dependent helicase, putative
PFIT_0715600	2543,9355	2221,6433	8749,099749	8278,433984	2382,789409	8513,766867	3,57302531	1,837146133	4,345E-19	8,075E-18	calcium-dependent protein kinase 4
PFIT_1034900	3447,5962	3172,5374	22945,60175	18373,84611	3310,066808	20659,72393	6,24148246	2,641888735	4,686E-19	8,681E-18	merozoite surface protein 6
PFIT_0321600	227,42462	240,85465	982,8785581	968,639154	234,1396328	975,7588561	4,167422851	2,059155492	5,029E-19	9,286E-18	peptidase, putative
PFIT_0821000	1378,636	1313,1395	5633,044436	4697,944453	1345,887748	5165,494444	3,837983109	1,940348362	5,246E-19	9,654E-18	zinc finger protein, putative
PFIT_1143900	104,65558	111,75656	509,0923702	535,5585387	108,2060664	522,3254544	4,827136515	2,271167628	6,041E-19	1,108E-17	DnaJ protein, putative
PFIT_1364600	235,47505	158,00065	758,5134643	893,7857143	196,7378478	826,1495893	4,199240759	2,070128506	7,249E-19	1,325E-17	DNA topoisomerase VI, b subunit, putative
PFIT_0206200.1	358,24409	291,91583	1292,661835	1274,290699	325,0799606	1283,476267	3,948186362	1,98119009	7,823E-19	1,426E-17	merozoite surface protein 5
PFIT_1411400	11188,084	11224,79	87891,89349	69593,20332	11206,43682	78742,54841	7,026546412	2,812815771	8,258E-19	1,5E-17	rhoptry-associated protein 1
PFIT_1458700	196,22921	176,3056	798,375283	793,0900157	186,2674039	795,7326494	4,271990873	2,094908565	9,409E-19	1,704E-17	vacuolar protein sorting-associated protein 4 (VPS4)
PFIT_1139100	953,97583	919,10134	3258,418951	3482,46717	936,5385831	3370,443061	3,598829906	1,847527917	1,159E-18	2,093E-17	guanylyl cyclase
PFIT_1414700	1750,9683	1691,763	6360,807354	5851,043869	1721,365671	6105,925612	3,54714034	1,826656411	1,256E-18	2,259E-17	conserved Plasmodium protein, unknown function
PFIT_0522800	114,71861	81,89058	570,5934619	712,8899018	98,30459635	641,7416818	6,528094368	2,706661913	1,279E-18	2,295E-17	conserved protein, unknown function, putative
Pf_M76611101800	2755,2593	2721,6575	9556,586305	9560,744695	2738,458413	9558,6655	3,490527902	1,803445244	1,342E-18	2,4E-17	cytochrome c oxidase subunit 1
PFIT_0408200	494,09508	455,69699	1681,02984	1857,078194	474,8960351	1769,054017	3,725139581	1,897294484	1,811E-18	3,229E-17	replication protein A1, large subunit
PFIT_1330700	140,88251	115,61023	617,2887352	554,2718986	128,2463687	585,7803169	4,567617178	2,19144174	1,969E-18	3,498E-17	CPW-WPC family protein
PFIT_1478100	1241,7787	1332,4079	371,2843684	298,5226464	1287,093288	334,9035074	0,260201425	-1,942299233	1,981E-18	3,509E-17	Plasmodium exported protein (hyp17), unknown function
PFIT_1438500	191,19769	215,80576	1446,414564	1153,990528	203,501726	1300,202546	6,389147513	2,675623449	2,131E-18	3,763E-17	conserved Plasmodium protein, unknown function
PFIT_0518300	95,598844	112,71998	495,4254609	499,9140436	104,1594095	497,6697523	4,777962498	2,25639553	2,18E-18	3,837E-17	SWIB/MDM2 domain-containing protein, putative
PFIT_1452900	121,76274	125,24442	904,2938298	720,0188008	123,5035773	812,1563153	6,575974015	2,717204598	2,188E-18	3,84E-17	protein phosphatase 2b regulatory subunit, putative
PFIT_1444900	79,497986	41,426999	335,9781861	338,6227033	60,46249262	337,3004447	5,578672498	2,479921859	2,296E-18	4,016E-17	dephospho-CoA kinase, putative (DPCK)
PFIT_0307600	764,79075	814,08871	2962,302584	2747,299459	789,4397298	2854,801021	3,616236824	1,854489162	2,572E-18	4,486E-17	conserved Plasmodium protein, unknown function
PFIT_0834800	37496,885	38591,659	11159,03142	11105,93356	38044,27197	11132,48249	0,292619149	-1,772903912	2,609E-18	4,535E-17	histidine-rich protein II
PFIT_1422900	182,14095	173,41535	675,3730996	810,9122633	177,7781507	743,1426814	4,180168815	2,063561206	2,978E-18	5,161E-17	Ctr copper transporter domain containing protein, putative
PFIT_1327600	1037,499	913,32083	3419,005135	3487,813844	975,4099274	3453,40949	3,540469902	1,823940852	3,044E-18	5,26E-17	conserved Plasmodium protein, unknown function
PFIT_0902300	503,15181	529,88023	1914,506206	1900,742701	516,5160172	1907,624454	3,693253239	1,884892189	3,102E-18	5,344E-17	serine/threonine protein kinase, FIKK family
PFIT_0320700	115,72492	75,14665	421,3963691	490,1118075	95,43578309	455,7540883	4,775505303	2,255653395	4,007E-18	6,881E-17	signal peptidase protein
PFIT_0206200.2	341,13693	293,84267	1258,494562	1187,852799	317,4897984	1223,17368	3,852639317	1,945847128	4,562E-18	7,811E-17	merozoite surface protein 5
PFIT_1221000	383,40168	439,31888	1689,002204	1413,30423	411,3602781	1551,153217	3,770789985	1,914866801	5,584E-18	9,531E-17	conserved Plasmodium protein, unknown function
PFIT_1214500	110,6934	106,93946	493,1476427	512,3896169	108,8164308	502,7686298	4,620337444	2,207998222	6,296E-18	1,071E-16	conserved Plasmodium membrane protein, unknown function
PFIT_1358800	1176,3689	1154,1755	4117,156417	3994,856787	1165,272201	4056,006602	3,480737461	1,799393001	6,795E-18	1,153E-16	conserved Plasmodium protein, unknown function
PFIT_0612600	814,09963	860,3328	4035,154961	3305,135807	837,2162146	3670,145384	4,383748571	2,132165055	7,23E-18	1,223E-16	conserved Plasmodium protein, unknown function
PFIT_1238600	1703,672	1491,372	5658,100436	5332,416465	1597,522002	5495,258451	3,439864016	1,782351534	7,551E-18	1,273E-16	protein kinase 2
PFIT_0508900	793,97355	782,2959	2547,739669	2949,581969	788,1347254	2748,660819	3,487551976	1,802214718	9,526E-18	1,602E-16	conserved Plasmodium protein, unknown function
PFIT_0704300	902,65434	743,75915	3884,818959	4829,829084	823,2067489	4357,324022	5,293110178	2,404115685	1,007E-17	1,689E-16	DNA replication licensing factor MCM7, putative
PFIT_0914900	57,359306	79,963743	346,2283681	369,8116365	68,66152467	358,0200023	5,214273992	2,382466393	1,125E-17	1,88E-16	GINS complex subunit Psf3, putative
PFIT_1142000	169,05901	168,59825	821,1534651	671,8987324	168,8286307	746,5260988	4,421797984	2,144633115	1,376E-17	2,294E-16	conserved Plasmodium protein, unknown function
PFIT_0909700	61,384521	65,512464	310,9221858	360,9005128	63,44849246	335,9113493	5,294236888	2,404422749	1,397E-17	2,322E-16	subpellicular microtubule protein 1, putative
PFIT_1238900	773,84748	737,01522	2507,87785	2751,755021	755,4313524	2629,816436	3,481211665	1,799589535	1,43E-17	2,369E-16	transcription factor with AP2 domain(s)
PFIT_1205700	52,327788	50,097767	263,0880034	319,018231	51,21277745	291,0531172	5,683212896	2,50670676	1,454E-17	2,402E-16	haloacid dehalogenase hydrolase, putative
PFIT_1416000	55,346699	60,695371	315,4778222	320,8004558	58,02103513	318,139139	5,483168963	2,455009931	1,554E-17	2,559E-16	uracil-DNA glycosylase, putative
PFIT_0514200	193,21029	252,41567	898,5992843	868,8345678	222,8129828	883,716926	3,966182378	1,987751019	1,566E-17	2,573E-16	ATP-dependent DNA helicase UvrD (UvrD)
PFIT_1022300	187,17247	145,47621	653,7138266	715,5632389	166,3243401	684,6485327	4,116346004	2,041364254	1,69E-17	2,767E-16	4-hydroxy-3-methylbut-2-en-1-yl diphosphate synthase
PFIT_1229800	84,529504	77,073487	389,5069141	404,5650193	80,80149561	397,0359667	4,913720515	2,296815802	1,7E-17	2,776E-16	conserved Plasmodium protein, unknown function
PFIT_1105900	17373,832	15082,318	50427,47847	55663,33464	16228,07501	53045,40655	3,268742998	1,708735951	1,79E-17	2,914E-16	histone H2B
PFIT_1337900	269,68937	275,53772	939,6000121	1124,58382	272,6135434	1032,091916	3,785915781	1,920642318	1,869E-17	3,034E-16	conserved Plasmodium protein, unknown function
PFIT_1426300	603,78217	367,06248	1701,530204	1756,382495	485,422327	1728,95635	3,561756956	1,832589074	2,158E-17	3,494E-16	conserved Plasmodium protein, unknown function
PFIT_0305900	43,271056	52,024604	252,8378214	300,3048711	47,64782976	276,5713463	5,804489893	2,537169287	2,222E-17	3,586E-16	conserved Plasmodium protein, unknown function
PFIT_0932600	1469,2033	1712,9583	5413,234979	5313,703105	1591,080769	5363,469042	3,370959631	1,75315935	2,555E-17	4,113E-16	Protein MAM3, putative
PFIT_1012700	1079,7638	1044,3458	3683,232047	3552,865048	1062,054768	3618,048548	3,406649693	1,768353602	2,977E-17	4,779E-16	conserved Plasmodium protein, unknown function
PFIT_1473800	253,58851	153,18356	898,5992843	1087,1571	203,3860339	992,8781922	4,881742239	2,287396121	3,109E-17	4,977E-16	DNA-directed RNA polymerase, alpha subunit, putative
PFIT_0402700	103,64927	55,878278	358,7563682	410,8028059	79,76377547	384,7795871	4,823989146	2,270226661	3,384E-17	5,401E-16	conserved Plasmodium protein, unknown function
PFIT_0703200	1752,9809	1878,6663	5469,041525	6512,249253	1815,823578	5990,645389	3,299134047	1,722087397	3,512E-17	5,59E-16	conserved Plasmodium membrane protein, unknown function
PFIT_1404400	62,390824	65,512464	339,3949135	329,7115796	63,95164427	334,5532465	5,231347064	2,387182486	3,607E-17	5,725E-16	conserved Plasmodium protein, unknown function
PFIT_1456900	147,92663	158,96407	645,7614628	630,9075631	153,4453497	638,334513	4,160012109	2,056587728	3,701E-17	5,858E-16	conserved Plasmodium protein, unknown function
PFIT_1246600	4419,6855	3395,0871	13683,9929	12089,72162	3907,386303	12886,85726	3,29807607	1,721624675	3,725E-17	5,879E-16	myosin A tail domain interacting protein
PFIT_1137900	309,94151	268,79379	1149,159287	1030,125908	289,3676507	1089,642598	3,765599213	1,912879457	3,773E-17	5,938E-16	conserved Plasmodium protein, unknown function
PFIT_1137400.1	88,554718	62,622208	363,3120047	380,5049851	75,58846341	371,9084949	4,920175356	2,298709734	3,951E-17	6,201E-16	conserved Plasmodium protein, unknown function
PFIT_0416100	64,403432	76,110069	379,2567321	335,9493662	70,25675014	357,6030492	5,089945784	2,347650289	4,217E-17	6,6E-16	memo-like protein
PFIT_1407600	286,79653	288,06216	971,489467	1154,881641	287,429345	1063,185554	3,69894575	1,887114141	4,714E-17	7,357E-16	ATP-dependent Clp protease, putative
PFIT_0503500	1373,6044	961,49175	3885,957868	4580,317619	1167,548096	4233,137743	3,62566455	1,858245452	5,304E-17	8,256E-16	actin-depolymerizing factor 1
PFIT_0831600	182,14095	217,7326	839,3760108	744,9699473	199,9367783	792,1729791	3,962117355	1,986271612	5,405E-17	8,367E-16	conserved Plasmodium protein, unknown function
PFIT_0804900	1187,4383	898,86955	5487,264071	6916,814272	1043,153907	6202,039171	5,945468954	2,571790608	5,397E-17	8,367E-16	transcription factor with AP2 domain(s)
PFIT_1357500	51,321484	44,317255	272,1992762	274,4626122	47,81936986	273,3309442	5,71590435	2,514981775	5,999E-17	9,261E-16	conserved Plasmodium protein, unknown function
PFIT_0819300	88,554718	93,451603	415,7018235	428,6250534	91,00316088	422,1634385	4,638997529	2,213813078	6,517E-17	1,003E-15	conserved Plasmodium protein, unknown function
PFIT_1311800	220,38049	207,135	808,6254649	841,2100841	213,7577448	824,9177745	3,859124615	1,948273631	6,845E-17	1,051E-15	branched-chain alpha keto-acid dehydrogenase, putative
PFIT_1341000	94,59254	96,341859	427,0909146	445,5561886	95,46719962	436,3235516	4,570402749	2,192321303	6,909E-17	1,058E-15	conserved Plasmodium protein, unknown function
PFIT_0306400	81,510593	69,366139	365,5898229	368,9205242	75,43836582	367,2551735	4,86828114	2,283412485	8,401E-17	1,283E-15	conserved Plasmodium protein, unknown function
PFIT_0103300	824,16266	785,18615	3519,229136	2888,095215	804,6744076	3203,662175	3,981314859	1,99324497	8,617E-17	1,312E-15	novel putative transporter 1
PFIT_0422500	1231,7156	907,54031	3659,314956	3475,338271	1069,627971	3567,326614	3,335109694	1,737734213	8,832E-17	1,341E-15	erythrocyte binding antigen-165, pseudogene
PFIT_0505200	470,95009	473,03853	1768,725841	1563,01111	471,9943108	1665,868475	3,529424904	1,819433125	9,164E-17	1,388E-15	actin-like protein, putative
PFIT_1350800	418,6223	410,41632	1599,028384	1364,29305	414,5193125	1481,660717	3,574406963	1,837703901	9,576E-17	1,447E-15	glycerol kinase
PFIT_0820200	866,42741	789,03983	2652,519307	2889,877439	827,7336207	2771,198373	3,347935016	1,743271525	9,967E-17	1,502E-15	asparagine-rich antigen
PFIT_1431300	57,359306	63,585627	292,6996401	334,1671415	60,47246663	313,4333908	5,183076005	2,37380855	9,998E-17	1,502E-15	phosphotyrosyl phosphatase activator, putative
PFIT_0406500	2022,6703	2125,3014	8013,364467	6646,807222	2073,985843	7330,085844	3,534298882	1,821424048	1,191E-16	1,785E-15	AAA family ATPase, putative
PFIT_0109500	426,67273	469,18485	1486,276383	1634,3001	447,9287941	1560,288241	3,483339901	1,800471257	1,267E-16	1,893E-15	kinesin-8, putative
PFIT_0623900.2	68,428646	52,988023	338,2560043	293,1759721	60,70833427	315,7159882	5,200537817	2,378660828	1,399E-16	2,085E-15	sphingomyelin synthase 1, putative (SMS1)
PFIT_0937400	302,89739	263,01328	61,5010917	43,66450648	282,9553322	52,58279909	0,185834275	-2,427911478	1,545E-16	2,288E-15	Plasmodium exported protein (PHISTc), unknown function
PFIT_0831800	220,38049	174,37877	786,9861919	739,6232731	197,3796287	763,3047325	3,867191045	1,951286037	1,547E-16	2,288E-15	conserved Plasmodium protein, unknown function
PFIT_0535500	30,189109	35,646488	240,3098213	194,2624982	32,91779821	217,2861598	6,600871612	2,722656538	1,544E-16	2,288E-15	conserved Plasmodium protein, unknown function
PFIT_1215000	98,617755	74,183232	363,3120047	446,447301	86,40049305	404,8796528	4,686080351	2,228381692	1,572E-16	2,319E-15	conserved Plasmodium protein, unknown function
PFIT_1016700	67,422342	47,207511	289,2829128	310,1071073	57,31492669	299,69501	5,228917271	2,386512245	1,582E-16	2,328E-15	enoyl-CoA hydratase-related protein, putative
PFIT_1016800.1	166,0401	142,58595	604,760735	630,9075631	154,3130242	617,8341491	4,003771892	2,001359781	2,015E-16	2,957E-15	golgi re-assembly stacking protein 2
PFIT_0504600	156,98336	132,95177	548,9541889	616,649765	144,967565	582,801977	4,020223261	2,007275623	2,529E-16	3,702E-15	3-methyl-2-oxobutanoate dehydrogenase (lipoamide), putative
PFIT_1209700	55,346699	43,353837	268,7825489	271,789275	49,3502678	270,285912	5,476888455	2,453356498	2,663E-16	3,888E-15	syntaxin, Qa-SNARE family
PF_apicoplast_genom	1280,0182	1114,6753	3823,317867	3942,281157	1197,346756	3882,799512	3,24283629	1,697256193	2,797E-16	4,074E-15	elongation factor Tu, putative
PFIT_0314800	46,289966	52,988023	275,6160035	266,4426008	49,63899448	271,0293022	5,460007903	2,448903039	3,559E-16	5,17E-15	conserved Plasmodium protein, unknown function
PFIT_1209100.2	179,12204	165,708	799,5141921	651,4031477	172,4150209	725,4586699	4,207630323	2,073007957	3,607E-16	5,226E-15	cGMP-specific phosphodiesterase
PFIT_1230900	357,23778	283,24507	1236,855289	1054,185942	320,2414251	1145,520615	3,577053203	1,838771576	3,642E-16	5,263E-15	conserved Plasmodium protein, unknown function
PFIT_1111400	60,378217	46,244092	271,0603671	290,502635	53,31115473	280,781501	5,266843355	2,396938552	3,977E-16	5,732E-15	mitochondrial ribosomal protein L11 precursor, putative
PFIT_0917400	269,68937	234,11072	883,7934659	945,4702322	251,9000437	914,6318491	3,630931681	1,860339785	4,375E-16	6,29E-15	conserved Plasmodium membrane protein, unknown function
PFIT_1128500	1438,0079	1317,9566	5477,013889	4517,939752	1377,982252	4997,476821	3,626662691	1,858642569	4,676E-16	6,706E-15	CRAL/TRIO domain-containing protein, putative
PFIT_1108600	7456,7098	5860,4753	20585,78208	20807,47401	6658,59255	20696,62805	3,108258673	1,636106572	5,223E-16	7,47E-15	transcription factor with AP2 domain(s)
PFIT_1330800	572,58676	397,89188	1589,917111	1670,835707	485,2393184	1630,376409	3,359942914	1,748436721	5,696E-16	8,127E-15	conserved Plasmodium protein, unknown function
PFIT_1243700	108,68079	131,98835	517,0647339	487,4384703	120,3345689	502,2516021	4,173793173	2,061359111	5,713E-16	8,13E-15	endosome sorting protein (SNF7 homologue), putative
PFIT_1019100	199,24812	171,48851	759,6523734	943,6880075	185,3683128	851,6701904	4,594475601	2,199900206	5,735E-16	8,141E-15	conserved Plasmodium protein, unknown function
PFIT_0711900	90,567326	90,561348	383,8123686	478,5273466	90,56433661	431,1698576	4,76092327	2,251241378	5,795E-16	8,207E-15	conserved Plasmodium protein, unknown function
PFIT_1248000	95,598844	106,93946	448,7301876	430,4072782	101,2691537	439,5687329	4,340598464	2,117893969	5,922E-16	8,365E-15	dipeptidyl peptidase 2, putative
PFIT_0503700	2637,5218	2102,1794	7542,995006	7357,914899	2369,850575	7450,454952	3,143850094	1,652532428	6,379E-16	8,987E-15	myosin B
PFIT_0700800	1879,7752	1977,8984	11705,70779	9270,24206	1928,836763	10487,97492	5,437461128	2,442933182	6,856E-16	9,636E-15	Plasmodium exported protein, unknown function
PFIT_1462300	136,85729	124,281	542,1207342	517,7362912	130,5691452	529,9285127	4,058604443	2,02098374	8,006E-16	1,122E-14	conserved Plasmodium protein, unknown function
PFIT_0809100	230,44353	223,51311	788,125101	864,3790059	226,9783209	826,2520535	3,640224539	1,864027443	8,369E-16	1,17E-14	conserved Plasmodium membrane protein, unknown function
PFIT_1330600	83,5232	84,780836	335,9781861	414,3672554	84,15201817	375,1727208	4,458273597	2,156485155	9,161E-16	1,278E-14	signal peptidase 21 kDa subunit
PFIT_1209200	55,346699	58,768534	284,7272764	294,0670845	57,05761654	289,3971804	5,072016639	2,342559478	9,721E-16	1,353E-14	porphobilinogen deaminase
PFIT_1228300	4303,9606	3672,5517	23541,25121	30455,54772	3988,256122	26998,39946	6,769474837	2,759043917	1,106E-15	1,536E-14	merozoite surface protein 9
PFIT_1247900	112,70601	107,90288	464,6749151	456,2495371	110,3044437	460,4622261	4,17446669	2,061591897	1,292E-15	1,789E-14	conserved Plasmodium protein, unknown function
PFIT_1137400.2	59,371913	37,573325	248,282185	266,4426008	48,47261926	257,3623929	5,309438541	2,408559308	1,311E-15	1,811E-14	conserved Plasmodium protein, unknown function
PFIT_1211300	858,37699	906,5769	2800,577491	2850,668495	882,4769404	2825,622993	3,201922751	1,678938502	1,355E-15	1,868E-14	DNA replication licensing factor MCM5, putative (MCM5)
PFIT_0410600	115,72492	115,61023	453,285824	495,4584817	115,6675735	474,3721529	4,101168015	2,036034849	1,362E-15	1,872E-14	conserved Apicomplexan protein, unknown function
PFIT_0516000	183,14726	202,3179	763,0690007	681,7009686	192,7325813	722,3850346	3,748120995	1,906167526	1,379E-15	1,891E-14	protein phosphatase, putative
PFIT_1223900	467,93118	387,29427	1411,108382	1454,2954	427,612728	1432,701891	3,350465963	1,744361751	1,408E-15	1,926E-14	conserved Plasmodium protein, unknown function
PFIT_0203400	720,51339	888,27194	2679,853125	2521,848028	804,392666	2600,850576	3,233309659	1,693011683	1,448E-15	1,977E-14	conserved Plasmodium protein, unknown function
PFIT_0529900	115,72492	131,98835	477,2029152	522,191853	123,8566316	499,6973841	4,034482271	2,01238355	1,494E-15	2,034E-14	conserved Plasmodium protein, unknown function
PFIT_1207100	105,66188	89,597929	397,4792778	433,9717277	97,62990444	415,7255028	4,258177913	2,090236229	1,567E-15	2,128E-14	conserved Plasmodium protein, unknown function
PFIT_0612900	2087,0737	2075,2036	6359,668445	6508,684803	2081,138675	6434,176624	3,091661647	1,628382439	1,813E-15	2,456E-14	myosin E, putative
PFIT_1323300	172,07792	150,2933	561,482189	688,8298676	161,1856095	625,1560283	3,878485371	1,95549336	2,385E-15	3,216E-14	conserved Plasmodium membrane protein, unknown function
PFIT_0530400.1	113,71231	100,19553	456,7025513	433,0806153	106,9539212	444,8915833	4,159656593	2,056464429	2,385E-15	3,216E-14	ADP-ribosylation factor GTPase-activating protein, putative
PFIT_0917600	84,529504	80,927162	429,3687328	548,9252244	82,7283328	489,1469786	5,91268991	2,563814617	2,61E-15	3,511E-14	conserved Plasmodium protein, unknown function
PFIT_1359900	145,91402	155,11039	537,5650978	662,9876086	150,5122089	600,2763532	3,988223663	1,99574632	2,754E-15	3,696E-14	polyadenylate-binding protein, putative
PFIT_0613100	379,37646	398,8553	1269,883653	1331,321892	389,1158804	1300,602772	3,342456162	1,740908639	2,779E-15	3,721E-14	conserved Plasmodium protein, unknown function
PFIT_0318700	591,70653	480,74588	1891,728024	1615,58674	536,2262022	1753,657382	3,270368689	1,709453289	3,382E-15	4,518E-14	trophozoite stage antigen
PFIT_0814800	185,15987	163,78116	652,5949175	639,8186868	174,4705131	646,2068022	3,703816711	1,889012707	4,25E-15	5,664E-14	RNA binding protein, putative
PFIT_1344800	291,82805	203,28132	1398,580382	1800,938114	247,554686	1599,759248	6,462245873	2,692035643	4,286E-15	5,698E-14	inner membrane complex protein
PFIT_0603000	1046,5558	782,2959	5414,373888	7031,767769	914,4258294	6223,070828	6,805440778	2,766688606	4,65E-15	6,167E-14	SPE2-interacting protein
PFIT_1361000	105,66188	72,256394	366,728732	390,3072212	88,95913712	378,5179766	4,25496457	2,089147119	5,351E-15	7,08E-14	conserved Plasmodium protein, unknown function
PFIT_0403600	1306,1821	1165,7365	4044,266234	3601,876229	1235,959296	3823,071231	3,093201567	1,629100849	5,797E-15	7,653E-14	6-cysteine protein
PFIT_0613000	578,62458	545,29492	1945,256752	1671,72682	561,9597516	1808,491786	3,21818739	1,686248334	7,049E-15	9,284E-14	thiamin-phosphate pyrophosphorylase, putative
PFIT_1406600	268,68307	244,70832	810,9032831	944,5791198	256,6956941	877,7412015	3,419384203	1,773736534	7,159E-15	9,407E-14	ribonucleotide reductase small subunit
PFIT_0715300	712,46296	590,5756	2099,009482	2013,913972	651,519279	2056,461727	3,156409631	1,658284447	7,675E-15	1,006E-13	conserved Plasmodium protein, unknown function
PFIT_0613900	452,83663	550,11202	1772,142568	1489,939895	501,4743219	1631,041231	3,252492022	1,701545518	7,752E-15	1,014E-13	conserved Plasmodium membrane protein, unknown function
PFIT_0214000	1827,4474	1379,6154	4965,6437	4758,540094	1603,531397	4862,091897	3,032115185	1,60032456	7,838E-15	1,023E-13	serine/threonine protein kinase, putative
PFIT_1015300	154,97076	157,03723	569,4545528	593,4808432	156,0039938	581,467698	3,727261616	1,898116085	7,888E-15	1,027E-13	ribonucleotide reductase small subunit, putative
PFIT_0306300	260,63264	208,09842	835,9592835	796,6544652	234,3655264	816,3068743	3,483050118	1,800351232	8,984E-15	1,167E-13	FAD-dependent glycerol-3-phosphate dehydrogenase, putative
PFIT_0205500	2967,5894	2768,865	9670,477215	8041,398092	2868,227201	8855,937654	3,087599773	1,626485757	9,22E-15	1,195E-13	metabolite/drug transporter, putative
PFIT_1347700	107,67449	97,305278	373,5621866	451,7939752	102,4898824	412,6780809	4,026525069	2,009535316	1,057E-14	1,367E-13	TBC domain protein, putative
PFIT_0504100	68,428646	61,65879	317,7556404	286,0470731	65,04371794	301,9013568	4,641514451	2,21459561	1,128E-14	1,455E-13	AN1-like zinc finger family protein
PFIT_1018700	16,100858	21,195209	136,6690927	151,4891041	18,64803345	144,0790984	7,726235518	2,949765656	1,149E-14	1,479E-13	conserved Plasmodium protein, unknown function
PFIT_0937000	1690,5901	1807,3733	627,5389172	491,8940322	1748,981678	559,7164747	0,320024207	-1,643747058	1,176E-14	1,51E-13	ring-exported protein 4
PFIT_0619600	139,8762	108,8663	521,6203703	449,1206381	124,3712519	485,3705042	3,902594024	1,964433391	1,224E-14	1,568E-13	DnaJ protein, putative
PFIT_1309800	357,23778	331,416	1189,021106	1088,048213	344,3268899	1138,534659	3,306551689	1,725327454	1,34E-14	1,713E-13	Rab GTPase 5b
PFIT_0312300	375,35125	381,51376	1169,659651	1283,201823	378,4325059	1226,430737	3,240817631	1,696357839	1,39E-14	1,772E-13	glycogen synthase kinase 3
PFIT_1304200	463,90597	451,84332	1464,63711	1461,424299	457,8746436	1463,030704	3,19526474	1,675935469	1,52E-14	1,935E-13	conserved Plasmodium protein, unknown function
PFIT_1106100	894,60392	967,27227	3215,140405	2664,426008	930,9380911	2939,783206	3,157871865	1,658952633	1,586E-14	2,01E-13	conserved Plasmodium protein, unknown function
PFIT_1016800.2	169,05901	141,62253	604,760735	545,3607749	155,3407704	575,0607549	3,701930624	1,888277858	1,583E-14	2,01E-13	golgi re-assembly stacking protein 2
PFIT_0805400	972,08929	1026,0408	3203,751314	2911,264136	999,0650475	3057,507725	3,060369025	1,613705626	1,641E-14	2,074E-13	adenylyl cyclase beta (ACbeta)
PFIT_1358900	42,264752	35,646488	210,6981845	216,5403077	38,95561992	213,6192461	5,483656698	2,455138255	1,649E-14	2,08E-13	ADP-ribosylation factor, putative
PFIT_0917500	243,52548	311,18421	902,0160116	960,6191426	277,3548403	931,3175771	3,357855864	1,747540304	1,659E-14	2,087E-13	conserved Plasmodium protein, unknown function
PFIT_1321200	163,02119	138,73228	511,3701884	595,263068	150,8767316	553,3166282	3,667342354	1,874734951	1,667E-14	2,093E-13	adenosine-diphosphatase, putative
PFIT_0307500	81,510593	84,780836	334,839277	369,8116365	83,14571455	352,3254568	4,237445774	2,083194906	1,703E-14	2,133E-13	conserved Plasmodium protein, unknown function
PFIT_1205600	556,4859	546,25834	1667,362931	1769,749181	551,3721211	1718,556056	3,116871511	1,640098683	2,156E-14	2,695E-13	shewanella-like protein phosphatase 2, putative (SHLP2)
PFIT_0320800	296,85957	291,91583	1020,462559	941,9057827	294,3877003	981,1841706	3,332965915	1,736806563	2,228E-14	2,779E-13	ATP-dependent RNA helicase, putative
PFIT_0616300	50,315181	46,244092	220,9483665	280,7003988	48,27963664	250,8243826	5,195241723	2,377190876	2,257E-14	2,809E-13	conserved Plasmodium protein, unknown function
PFIT_0534400	77,485379	73,219813	358,7563682	458,9228743	75,35259577	408,8396213	5,425687292	2,439805901	2,281E-14	2,833E-13	phosphoenolpyruvate/phosphate translocator (PPT)
PFIT_1239400	517,24006	444,13597	1384,913472	1617,368965	480,6880151	1501,141218	3,122901282	1,642886965	2,353E-14	2,916E-13	cell division protein FtsH, putative
PFIT_0409400	1482,2852	1404,6643	4188,90769	4382,490671	1443,474768	4285,699181	2,969015653	1,569984699	2,425E-14	2,998E-13	conserved Plasmodium protein, unknown function
PFIT_1253200	581,64349	630,07576	195,8923662	146,1424299	605,859625	171,017398	0,282272313	-1,824840465	2,531E-14	3,123E-13	gametocyte erythrocyte cytosolic protein
PFIT_1011800	123,77534	118,50049	782,4305555	609,520866	121,1379159	695,9757108	5,745316862	2,522386462	2,559E-14	3,149E-13	ubiquinol-cytochrome c reductase complex subunit, putative
PFIT_1147900	285,79023	255,30593	861,0152838	937,4502208	270,5480771	899,2327523	3,323744755	1,732809595	2,578E-14	3,167E-13	conserved Plasmodium protein, unknown function
PFIT_0916100	1777,1322	1944,1787	5954,216804	5148,847316	1860,655454	5551,53206	2,983643237	1,577075038	2,597E-14	3,183E-13	sugar transporter, putative
PFIT_1423500	216,35528	251,45225	793,8196466	802,0011395	233,9037651	797,910393	3,411276396	1,770311653	2,654E-14	3,246E-13	ERAD-associated E3 ubiquitin-protein ligase HRD1 (HRD1)
PFIT_0508000	645,04062	761,10069	2216,317119	2106,58966	703,0706532	2161,45339	3,074304666	1,620260144	3,002E-14	3,663E-13	6-cysteine protein
PFIT_1444800	105,66188	111,75656	395,2014596	450,0117505	108,7092183	422,6066051	3,887495576	1,958841034	3,582E-14	4,362E-13	conserved Plasmodium protein, unknown function
PFIT_0925400	151,95185	171,48851	568,3156437	593,4808432	161,7201778	580,8982434	3,591996072	1,844785772	3,683E-14	4,475E-13	proline--tRNA ligase, putative
PFIT_1333000	811,08072	945,11364	2866,634219	2473,727959	878,0971772	2670,181089	3,040871965	1,604485073	3,869E-14	4,691E-13	histone H3 variant, putative
PFIT_1205900	427,67904	358,39172	1170,798561	1305,479633	393,0353768	1238,139097	3,150197589	1,655442321	4,263E-14	5,157E-13	conserved Plasmodium protein, unknown function
PFIT_0305500	348,18105	323,70865	1040,962922	1116,563809	335,9448493	1078,763366	3,211132327	1,683082118	4,292E-14	5,182E-13	AP endonuclease (DNA-[apurinic or apyrimidinic site] lyase), putative
PFIT_1411700	92,579933	96,341859	526,1760068	687,0476428	94,460896	606,6118248	6,421830095	2,682984496	5,106E-14	6,151E-13	conserved Plasmodium protein, unknown function
PFIT_1432100	53,334092	54,91486	225,5040029	285,1559607	54,12447573	255,3299818	4,717458753	2,238009904	5,471E-14	6,577E-13	cytochrome c oxidase subunit 2, putative
PFIT_0831400	37,233234	35,646488	176,5309114	219,2136448	36,43986087	197,8722781	5,430105201	2,440980149	5,641E-14	6,766E-13	translation initiation factor EIF-2b alpha subunit, putative
PFIT_0518000	416,6097	449,91648	1365,552018	1342,01524	433,26309	1353,783629	3,124622567	1,643681933	6,776E-14	8,111E-13	zinc finger protein, putative
PFIT_1132600	1401,7809	1343,9689	4315,326601	3764,058681	1372,874937	4039,692641	2,942505927	1,557045321	7,523E-14	8,986E-13	oxysterol-binding protein-related protein 2
PFIT_1330900	78,491682	106,93946	383,8123686	362,6827375	92,71557294	373,247553	4,025726652	2,009249216	7,852E-14	9,36E-13	protein tyrosine phosphatase, putative
PFIT_0834600	197658,16	212740,17	82033,34505	65069,9169	205199,1616	73551,63098	0,358440212	-1,480195598	7,998E-14	9,514E-13	heat shock protein 70 (HSP70-x)
PFIT_1406700	1274,9867	1235,1026	3979,348415	3422,762641	1255,044659	3701,055528	2,948943291	1,560198079	8,22E-14	9,736E-13	conserved protein, unknown function
PFIT_1247000	156,98336	138,73228	485,175279	568,5296967	147,8578208	526,8524878	3,563237203	1,833188526	8,22E-14	9,736E-13	signal recognition particle receptor, beta subunit
PFIT_1343000	3537,1572	3615,71	10438,30195	10037,48982	3576,433596	10237,79588	2,862571221	1,517311588	8,592E-14	1,016E-12	conserved Plasmodium protein, unknown function
PFIT_0920300	180,12835	184,01295	597,9272804	655,8587096	182,0706493	626,892995	3,443130441	1,783720838	8,714E-14	1,028E-12	CS-domain containing protein, conserved in Apicomplexa
PFIT_1403100	321,01085	309,25737	1020,462559	997,1547501	315,134111	1008,808654	3,201204246	1,678614728	9,565E-14	1,126E-12	conserved Plasmodium protein, unknown function
PFIT_1412000.2	3143,6925	2493,3273	8187,61756	7918,424584	2818,509909	8053,021072	2,857190974	1,514597469	9,629E-14	1,131E-12	conserved Plasmodium protein, unknown function
PFIT_1217700	1268,9489	1082,8825	3717,399321	3198,202322	1175,91568	3457,800821	2,940517659	1,556070155	1E-13	1,173E-12	thrombospondin-related apical membrane protein (TRAMP)
PFIT_1112900	1008,3162	1002,9188	2784,632763	3075,228814	1005,617489	2929,930788	2,913563874	1,542784939	1,106E-13	1,294E-12	conserved Plasmodium protein, unknown function
PFIT_1036600	344,15584	358,39172	1089,936014	1120,128258	351,2737767	1105,032136	3,14578602	1,653420541	1,141E-13	1,332E-12	conserved Plasmodium protein, unknown function
PFIT_1312800	524,28418	539,51441	1527,277111	1685,093505	531,8992981	1606,185308	3,019716916	1,59441331	1,145E-13	1,334E-12	conserved Plasmodium protein, unknown function
PFIT_0623900.1	79,497986	55,878278	307,5054585	280,7003988	67,68813206	294,1029287	4,344970377	2,119346341	1,167E-13	1,357E-12	sphingomyelin synthase 1, putative (SMS1)
PFIT_0730400	35653,337	28337,031	97152,36343	83339,50285	31995,18411	90245,93314	2,820609903	1,496007152	1,288E-13	1,491E-12	erythrocyte binding antigen-175
PFIT_1228400	222,3931	189,79346	617,2887352	752,0988464	206,093281	684,6937908	3,322251882	1,732161458	1,287E-13	1,491E-12	conserved Plasmodium protein, unknown function
PFIT_0706200	4253,6454	3342,0991	22502,56611	29759,58895	3797,872243	26131,07753	6,880451962	2,782503336	1,296E-13	1,497E-12	rhoptry-associated membrane antigen
PFIT_1019700	99,624058	72,256394	313,200004	372,4849737	85,94022626	342,8424888	3,989313314	1,996140435	1,347E-13	1,553E-12	conserved Plasmodium protein, unknown function
PFIT_1124100	513,21484	485,56297	1608,139657	1439,146489	499,3889076	1523,643073	3,05101505	1,609289297	1,545E-13	1,778E-12	leucine-rich repeat protein
PFIT_1433100	112,70601	101,15895	389,5069141	419,7139297	106,9324787	404,6104219	3,783793539	1,919833371	1,652E-13	1,893E-12	conserved Plasmodium membrane protein, unknown function
PFIT_1232000	70,441253	76,110069	287,0050946	362,6827375	73,275661	324,8439161	4,433176196	2,148340703	1,65E-13	1,893E-12	lipoamide dehydrogenase
PFIT_1453700	56,353003	57,805116	279,0327309	241,4914542	57,07905905	260,2620925	4,559677347	2,18893174	1,944E-13	2,224E-12	SNARE protein, putative
PFIT_1360500.1	543,40395	520,24604	3250,446587	2516,501353	531,8249966	2883,47397	5,421847391	2,438784506	2,119E-13	2,419E-12	conserved Plasmodium protein, unknown function
PFIT_0403500	491,07617	428,72127	1518,165838	1293,895172	459,8987194	1406,030505	3,057261187	1,612239809	2,152E-13	2,451E-12	conserved Plasmodium protein, unknown function
PFIT_1222400	2524,8158	2803,5481	18725,94351	14308,59144	2664,181939	16517,26748	6,199752064	2,632210521	2,185E-13	2,484E-12	glideosome-associated protein 45

PFIT_0701400	7974,9562	8964,61	3560,229864	2602,939254	8469,783083	3081,584559	0,36383276	-1,458652642	2,741E-13	3,11E-12	Plasmodium exported protein, unknown function
PFIT_1341800	16674,451	13026,383	43474,43838	38870,32189	14850,41686	41172,38014	2,772473023	1,471173423	2,97E-13	3,362E-12	myosin A
PFIT_0211400	62,390824	69,366139	294,9774583	271,789275	65,87848145	283,3833667	4,301607451	2,104875876	3,023E-13	3,416E-12	GDP-fructose:GMP antiporter, putative
PFIT_0932400	129,81317	122,35416	635,5112809	501,6962684	126,0836639	568,6037746	4,50973391	2,173042312	3,045E-13	3,434E-12	adenylate kinase-like protein 2 (AKLP2)
PFIT_1324800	32,201716	17,341535	150,3360019	155,944666	24,77162521	153,140334	6,182086669	2,62809388	3,214E-13	3,617E-12	conserved Plasmodium protein, unknown function
PFIT_1240500	8767,9234	8032,0208	33700,32043	27180,70973	8399,97211	30440,51508	3,623882875	1,857536328	3,255E-13	3,657E-12	erythrocyte membrane protein 1, PfEMP1
PFIT_0606500	167,0464	157,03723	895,182557	1181,615012	162,0418155	1038,398785	6,40821495	2,679922541	3,393E-13	3,804E-12	conserved Plasmodium protein, unknown function
PFIT_0409500	3680,0523	4209,1758	1458,942564	1340,233015	3944,614079	1399,58779	0,354809815	-1,494882178	3,644E-13	4,077E-12	conserved Plasmodium protein, unknown function
PFIT_0717700	374,34495	358,39172	1148,020378	1098,741561	366,368331	1123,38097	3,066261123	1,616480562	3,863E-13	4,313E-12	conserved membrane protein, unknown function
PFIT_1402700	1203,5391	1168,6268	3269,808042	3440,584888	1186,082939	3355,196465	2,828804255	1,50019235	4,104E-13	4,573E-12	choline kinase
PFIT_1230000	81,510593	52,988023	287,0050946	279,8092864	67,24930779	283,4071905	4,214276694	2,075285039	4,119E-13	4,581E-12	subpellicular microtubule protein 2, putative
PFIT_1126600	1657,3821	1570,3723	6913,178271	5528,461188	1613,877181	6220,819729	3,854580634	1,946573908	4,138E-13	4,593E-12	kelch protein, putative
PFIT_1107100	213,33637	217,7326	684,4843724	717,3454637	215,5344843	700,914918	3,251985037	1,701320619	4,226E-13	4,682E-12	exonuclease, putative
PFIT_0704000	416,6097	534,69732	1503,360019	1358,055263	475,6535081	1430,707641	3,007877829	1,58874597	4,314E-13	4,769E-12	conserved Plasmodium protein, unknown function
PFIT_1360400	30,189109	28,902558	144,6414564	187,1335992	29,54583314	165,8875278	5,614582842	2,489178836	4,931E-13	5,441E-12	actin-depolymerizing factor 2
PFIT_1128400	102,64297	77,073487	332,5614588	416,1494802	89,85822817	374,3554695	4,166067784	2,058686313	5,213E-13	5,741E-12	protein disulfide isomerase, putative
PFIT_0703300	669,19191	574,19748	1663,946203	1919,456061	621,6946932	1791,701132	2,881963046	1,527051837	5,548E-13	6,098E-12	phosphoinositide-binding protein, putative
PFIT_1421000	1613,1047	1660,9337	570,5934619	547,1429996	1637,019176	558,8682307	0,341393821	-1,55049115	5,764E-13	6,323E-12	splicing factor 3B subunit 4, putative
PFIT_1022200	555,4796	549,1486	1772,142568	1496,177681	552,3140971	1634,160125	2,95875143	1,564988498	5,987E-13	6,555E-12	phospholipid scramblase 1, putative
PFIT_1306700	249,5633	267,83037	1266,466925	998,0458625	258,6968329	1132,256394	4,376769446	2,12986639	7,143E-13	7,806E-12	conserved Plasmodium protein, unknown function
PFIT_1343700	44,277359	56,841697	249,4210941	216,5403077	50,55952805	232,9807009	4,608047383	2,204155551	7,602E-13	8,291E-12	conserved Plasmodium protein, unknown function
PFIT_0829300	101,63667	91,524766	342,8116408	380,5049851	96,5807158	361,6583129	3,744622412	1,904820252	7,621E-13	8,296E-12	dehydrodolichyl diphosphate synthetase, putative
PFIT_1326500	2400,0341	2151,3137	6578,338994	6009,661872	2275,673922	6294,000433	2,765774293	1,467683427	7,854E-13	8,534E-12	conserved Plasmodium protein, unknown function
PFIT_0530400.2	105,66188	116,57365	387,2290959	418,8228173	111,1177647	403,0259566	3,627016414	1,858783274	8,147E-13	8,835E-12	ADP-ribosylation factor GTPase-activating protein, putative
PFIT_1015500	340,13062	323,70865	1576,250202	1243,992879	331,9196348	1410,12154	4,248382417	2,086913636	8,84E-13	9,568E-12	conserved Plasmodium protein, unknown function
PFIT_1014900	113,71231	130,06151	437,3410965	429,5161658	121,8869093	433,4286312	3,555990003	1,830251268	1,009E-12	1,09E-11	conserved Plasmodium protein, unknown function
PFIT_1412000.1	3297,657	2572,3276	8144,339014	7850,700043	2934,992298	7997,519528	2,724886036	1,446195893	1,053E-12	1,135E-11	conserved Plasmodium protein, unknown function
PFIT_0302400	14023,847	16957,131	136267,0578	101213,4349	15490,48893	118740,2463	7,665364657	2,938354426	1,094E-12	1,177E-11	cytoadherence linked asexual protein 3.1 (CLAG3.1)
PFIT_0407100	69,43495	57,805116	246,0043668	283,373736	63,62003256	264,6890514	4,160467084	2,056745505	1,117E-12	1,2E-11	flap endonuclease 1
PFIT_0307900	26,163894	30,829395	153,7527292	167,5291269	28,4966445	160,6409281	5,637187497	2,494975553	1,133E-12	1,215E-11	DNA polymerase epsilon subunit b, putative
PFIT_0206900	5209,6338	4340,2008	29612,77565	39894,21002	4774,917292	34753,49283	7,278344463	2,863610331	1,169E-12	1,251E-11	serine repeat antigen 5
PFIT_0930500	37964,817	40073,396	251085,0403	191745,1058	39019,10646	221415,073	5,674529561	2,504500793	1,221E-12	1,304E-11	merozoite surface protein 1
PFIT_0422900	22351,01	27606,76	7932,50192	5922,332859	24978,8847	6927,41739	0,277330933	-1,850319556	1,296E-12	1,382E-11	serine/threonine protein kinase, FIKK family
PFIT_0208900	654,09735	737,01522	2133,176755	1846,384846	695,5562871	1989,7808	2,860704212	1,516370335	1,519E-12	1,616E-11	transporter, putative
PFIT_0801600	413,59079	414,26999	84,27927381	140,7957556	413,9303907	112,5375147	0,271875458	-1,878982167	1,522E-12	1,617E-11	rifin, pseudogene
PFIT_0732800	28,176501	26,012302	144,6414564	163,9646774	27,09440164	154,3030669	5,695016593	2,509700045	1,571E-12	1,665E-11	acyl-CoA synthetase
PFIT_0209900	1654,3631	1581,9333	8548,651746	6648,589446	1618,148238	7598,620596	4,695874222	2,231393767	1,624E-12	1,718E-11	conserved Plasmodium protein, unknown function
PFIT_0103900.1	25,15759	14,451279	148,0581837	199,6091725	19,80443466	173,8336781	8,77751277	3,13381219	1,628E-12	1,72E-11	conserved Plasmodium protein, unknown function
PFIT_0832300	135,85099	124,281	397,4792778	491,0029198	130,0659934	444,2410988	3,41550537	1,772099061	1,738E-12	1,832E-11	prolyl 4-hydroxylase alpha subunit 1, putative
PFIT_0833800	217,36158	213,87893	704,9847363	662,9876086	215,6202544	683,9861725	3,172179601	1,665474455	1,785E-12	1,879E-11	Plasmodium exported protein, unknown function
PFIT_1410200.2	22,13868	17,341535	124,1410925	135,4490813	19,74010712	129,7950869	6,575196686	2,717034051	1,876E-12	1,97E-11	conserved Plasmodium protein, unknown function
PFIT_1347800	428,68534	615,62448	1564,861111	1457,859849	522,1549107	1511,36048	2,894467617	1,533298016	1,923E-12	2,015E-11	conserved Plasmodium protein, unknown function
PFIT_0413200	186,16617	216,76918	1245,966561	954,381356	201,4676762	1100,173959	5,460796389	2,449111365	2,642E-12	2,764E-11	conserved protein, unknown function
PFIT_0616500	470,95009	465,33118	165,1418203	116,7357214	468,1406365	140,9387709	0,301060749	-1,731873465	2,667E-12	2,785E-11	erythrocyte membrane protein 1, PfEMP1
PFIT_0403300	14168,755	16813,581	6185,415352	4489,424156	15491,1681	5337,419754	0,344545984	-1,537231554	2,721E-12	2,837E-11	conserved Plasmodium membrane protein, unknown function
PFIT_0713400	47,29627	46,244092	198,1701844	222,7780943	46,77018122	210,4741393	4,500177973	2,169982058	3,302E-12	3,436E-11	calcium/calmodulin-dependent protein kinase, putative
PFIT_1209600	108,68079	92,488185	365,5898229	360,0094004	100,5844878	362,7996116	3,606914145	1,850765083	3,319E-12	3,447E-11	mitochondrial ribosomal protein L1-2 precursor, putative
PFIT_1433500	821,14375	785,18615	2253,90112	2193,02756	803,1649522	2223,46434	2,768378194	1,469041046	3,374E-12	3,498E-11	conserved Plasmodium protein, unknown function
PFIT_1311300	240,50656	251,45225	766,485828	742,2966102	245,9794085	754,3912191	3,066887686	1,616775334	3,454E-12	3,574E-11	conserved Plasmodium protein, unknown function
PFIT_0423300	650,07214	666,68567	230,0596393	194,2624982	658,3789013	212,1610688	0,322247673	-1,633758155	3,483E-12	3,597E-11	Plasmodium exported protein (hyp6), unknown function
PFIT_0504400	151,95185	130,06151	425,9520055	507,0429426	141,0066781	466,4974741	3,308336034	1,726105779	3,709E-12	3,824E-11	ATP-dependent helicase, putative
PFIT_1200200	727,55752	618,51474	211,8370936	218,3225324	673,0361257	215,079813	0,319566521	-1,645811822	3,846E-12	3,958E-11	erythrocyte membrane protein 1, PfEMP1
PFIT_1423600	153,96445	156,07381	504,5367338	511,4985045	155,0191327	508,0176191	3,277128509	1,712432248	4,036E-12	4,146E-11	conserved Plasmodium protein, unknown function
PFIT_1472600	105,66188	110,79314	413,4240053	357,3360633	108,227509	385,3800343	3,560832528	1,832214585	4,048E-12	4,151E-11	conserved Plasmodium membrane protein, unknown function
PFIT_1234300	178,11574	206,17158	571,732371	633,5809002	192,1436595	602,6566356	3,136489839	1,649150889	4,71E-12	4,82E-11	conserved Plasmodium protein, unknown function
PFIT_0818400	50,315181	53,951441	231,1985484	301,1959835	52,13331101	266,197266	5,10608785	2,35221836	4,719E-12	4,821E-11	conserved Plasmodium protein, unknown function
PFIT_1472400	80,504289	80,927162	277,8938218	327,9293548	80,71572556	302,9115883	3,752819989	1,90797509	4,751E-12	4,845E-11	conserved Plasmodium protein, unknown function
PFIT_0320500	460,88706	498,08741	2708,325853	2082,529626	479,4872344	2395,427739	4,99581129	2,320718982	4,956E-12	5,046E-11	conserved Plasmodium protein, unknown function
PFIT_1002600	929,82454	1058,797	4470,218239	3525,240564	994,3107876	3997,729402	4,020603469	2,007412058	5,18E-12	5,264E-11	conserved Plasmodium protein, unknown function
PFIT_1005300	110,6934	74,183232	309,7832767	381,3960974	92,43831475	345,5896871	3,738597875	1,902497303	5,354E-12	5,431E-11	DnaJ protein, putative
PFIT_0321400	190,19138	184,97637	610,4552806	575,6585957	187,5838767	593,0569381	3,161556039	1,660634792	5,477E-12	5,546E-11	conserved Plasmodium protein, unknown function
PFIT_0507300	934,85606	974,0162	6588,589176	4957,258154	954,4361286	5772,923665	6,048517541	2,596581589	5,757E-12	5,818E-11	conserved Plasmodium protein, unknown function
PFIT_0730200	2449,343	2759,2308	1093,352741	856,3589945	2604,286926	974,8558679	0,374327367	-1,417627568	6,089E-12	6,144E-11	Plasmodium exported protein (PHISTb), unknown function
PFIT_0706100	1072,7197	1067,4678	2816,522218	2937,106395	1070,093728	2876,814307	2,68837601	1,426734935	6,314E-12	6,359E-11	conserved Plasmodium protein, unknown function
PFIT_0522300	91,573629	99,232115	364,4509138	325,2560177	95,40287208	344,8534657	3,614707379	1,853878862	7,21E-12	7,247E-11	conserved Plasmodium protein, unknown function
PFIT_0414400	513,21484	488,45323	2132,037846	1685,984618	500,8340355	1909,011232	3,811664336	1,930421078	7,221E-12	7,247E-11	RING zinc finger protein, putative
PFIT_1019800	284,78392	242,78149	814,3200105	761,9010825	263,7827045	788,1105465	2,987726387	1,579048033	7,592E-12	7,605E-11	cytoplasmic dynein intermediate chain, putative
PFIT_0418000	529,3157	511,57527	2069,397845	2686,703817	520,4454876	2378,050831	4,569260159	2,191960587	7,775E-12	7,775E-11	conserved Plasmodium protein, unknown function
PFIT_0716800	249,5633	219,65944	690,178918	723,5832503	234,6113681	706,8810841	3,01298735	1,591194616	7,837E-12	7,823E-11	conserved Plasmodium protein, unknown function
PFIT_1335000	70,441253	78,036906	274,4770944	291,3937473	74,23907959	282,9354209	3,811138587	1,930222071	8,533E-12	8,503E-11	conserved Plasmodium protein, unknown function
PFIT_0320600	27,170198	23,122046	144,6414564	139,9046432	25,14612194	142,2730498	5,657852537	2,500254576	9,024E-12	8,976E-11	conserved Plasmodium protein, unknown function
PFIT_0625300	339,12432	329,48916	891,7658296	1022,997009	334,3067388	957,3814193	2,863781397	1,51792137	9,444E-12	9,378E-11	Sec14 domain containing protein
PFIT_1036000	126,79426	123,31758	394,0625505	439,318402	125,0559178	416,6904762	3,332033251	1,736402797	9,684E-12	9,599E-11	acetyl-CoA transporter, putative
PFIT_1028200	21,132376	11,561023	112,7520014	113,1712719	16,34669954	112,9616367	6,910363553	2,788761613	9,948E-12	9,844E-11	conserved Plasmodium protein, unknown function
PFIT_1010500	57,359306	40,463581	216,3927301	280,7003988	48,91144353	248,5465644	5,081562647	2,345272213	1,036E-11	1,023E-10	inner membrane complex protein, putative
PFIT_0903000	359,25039	345,86727	911,1272844	1088,048213	352,558833	999,5877485	2,835236718	1,503469193	1,045E-11	1,03E-10	serine repeat antigen 9
PFIT_1034200	56,353003	32,756232	175,3920023	217,43142	44,55461735	196,4117111	4,40833572	2,140234097	1,05E-11	1,034E-10	conserved Plasmodium protein, unknown function
PFIT_1105800	11772,746	8361,51	37357,35757	47571,14315	10067,12799	42464,25036	4,218109713	2,07659662	1,077E-11	1,058E-10	histone H4
PFIT_1244100	185,15987	212,91551	635,5112809	593,4808432	199,0376872	614,4960621	3,087335221	1,626362138	1,151E-11	1,129E-10	double C2-like domain-containing protein (DOC2)
PFIT_0409600	29937,533	30683,919	12464,22125	11257,42266	30310,72568	11860,82196	0,391307753	-1,3536244	1,175E-11	1,151E-10	conserved Plasmodium protein, unknown function
PFIT_0937200	4318,0488	5030,0085	2020,424753	1402,610882	4674,028646	1711,517817	0,366176151	-1,449390265	1,27E-11	1,241E-10	Plasmodium exported protein (PHISTb), unknown function
PFIT_1311400	21,132376	15,414697	124,1410925	114,0623843	18,27353672	119,1017384	6,517716861	2,704366681	1,308E-11	1,277E-10	conserved Plasmodium protein, unknown function
PFIT_0303700	44,277359	43,353837	191,3367297	199,6091725	43,8155979	195,4729511	4,461264035	2,157452535	1,347E-11	1,312E-10	IBR domain protein, putative
PFIT_1445400	21,132376	13,48786	100,2240013	127,4290699	17,31011813	113,8265356	6,575722637	2,717149448	1,434E-11	1,395E-10	calmodulin-like protein
PFIT_0400900	1639,2686	1238,9563	3654,75932	3846,04102	1439,112451	3750,40017	2,606050811	1,381865213	1,475E-11	1,432E-10	surface-associated interspersed protein 4.1 (SURFIN 4.1) (SURF4.1)
PFIT_1100900	2258,1453	2234,1677	912,2661935	779,7233301	2246,156516	845,9947618	0,376641056	-1,408737825	1,498E-11	1,451E-10	Plasmodium exported protein (hyp5), unknown function
PFIT_1111600	41,258448	49,134348	225,5040029	178,2224754	45,19639825	201,8632392	4,466356767	2,159098496	1,529E-11	1,479E-10	conserved Plasmodium protein, unknown function
PFIT_0503100	633,97128	556,85595	1753,920023	1510,435479	595,4136126	1632,177751	2,741250311	1,454834071	1,629E-11	1,573E-10	50S ribosomal protein L28, apicoplast, putative
PFIT_1239700	1951,2227	2257,2898	887,2101932	705,7610028	2104,256238	796,485598	0,378511696	-1,401590214	1,646E-11	1,587E-10	3-hydroxyisobutyryl-coenzyme A hydrolase, putative
PFIT_0536000	34158,976	36859,432	16981,13476	11574,65867	35509,2041	14277,89672	0,402090024	-1,314409554	1,798E-11	1,728E-10	Plasmodium exported protein (PHISTb), unknown function
PFIT_0611400	26,163894	19,268372	120,7243652	140,7957556	22,71613295	130,7600604	5,756264092	2,525132783	1,797E-11	1,728E-10	conserved Plasmodium protein, unknown function
PFIT_1327500	598,75065	699,4419	1596,750566	1880,247116	649,0962751	1738,498841	2,678337417	1,421337723	1,813E-11	1,739E-10	conserved Plasmodium protein, unknown function
PFIT_1418300	610,8263	632,96601	1635,473476	1721,629113	621,8961554	1678,551294	2,699086141	1,432471021	1,833E-11	1,755E-10	cysteine protease ATG4, putative (ATG4)
PFIT_0302200	2706,9567	3292,9647	1166,242924	1013,194773	2999,960739	1089,718848	0,36324437	-1,460987658	1,89E-11	1,807E-10	erythrocyte membrane protein 1 (PfEMP1), pseudogene
PFIT_1007800	38,239537	28,902558	152,6138201	173,7669136	33,57104761	163,1903668	4,861044813	2,281266434	1,918E-11	1,83E-10	endonuclease, putative
PFIT_1239600	3175,8942	3187,9521	1227,744016	1198,546147	3181,923169	1213,145082	0,381261588	-1,391146906	1,928E-11	1,837E-10	vesicle fusion and protein sorting subunit 16, putative
PFIT_0423400	601,76956	557,81936	227,7818211	159,5091155	579,794464	193,6454683	0,333989854	-1,582123818	2,156E-11	2,051E-10	Plasmodium exported protein (hyp15), unknown function
PFIT_1307200	534,34722	395,00162	2251,623302	2998,593149	464,6744218	2625,108225	5,649349528	2,498084764	2,379E-11	2,259E-10	conserved Plasmodium membrane protein, unknown function
PFIT_0505500	455,85554	436,42862	1127,520014	1306,370745	446,1420804	1216,94538	2,72770813	1,447689281	2,496E-11	2,366E-10	DNA repair protein, putative
PFIT_0919300	348,18105	337,19651	1759,614568	1354,490813	342,6887794	1557,052691	4,543634879	2,183846906	2,551E-11	2,414E-10	PPPDE peptidase, putative
PFIT_1203100	505,16442	516,39237	1465,776019	1330,430779	510,7783906	1398,103399	2,737201543	1,452701666	2,627E-11	2,482E-10	transporter, putative
PFIT_1035200	7794,8278	9349,9774	3482,784045	2602,939254	8572,402628	3042,861649	0,354960188	-1,494270872	2,661E-11	2,51E-10	probable protein, unknown function
PFIT_1139800	16862,63	16016,834	7115,904091	5993,621849	16439,7319	6554,76297	0,398714712	-1,326571256	2,814E-11	2,65E-10	RNA binding protein, putative
PFIT_0321300	47,29627	34,683069	170,8363658	194,2624982	40,98966967	182,549432	4,453547284	2,154954912	2,837E-11	2,667E-10	N-acetylglucosamine-1-phosphate transferase, putative
PFIT_1321400	842,27613	689,80771	2645,685852	3388,009258	766,0419198	3016,847555	3,938227761	1,97754655	3,276E-11	3,075E-10	conserved Plasmodium protein, unknown function
PFIT_1454400	223,3994	163,78116	543,2596433	611,3030908	193,5902819	577,2813671	2,981974929	1,576268128	3,336E-11	3,125E-10	conserved Plasmodium protein, unknown function
PFIT_1329100	122,76904	126,20784	431,646551	384,960547	124,4884384	408,303549	3,279851158	1,713630346	3,397E-11	3,178E-10	conserved Plasmodium protein, unknown function
PFIT_1026100	9502,5251	6991,5287	19586,9588	21482,93719	8247,026891	20534,94799	2,489981937	1,316135277	3,524E-11	3,291E-10	conserved Plasmodium protein, unknown function
PFIT_0930400	660,13517	764,95436	3528,340409	2726,803874	712,5447676	3127,572142	4,389299149	2,133990599	3,536E-11	3,296E-10	leucine-rich repeat protein
PFIT_0501900	191,19769	177,26902	579,7047347	736,0588236	184,2333541	657,8817791	3,570915713	1,836294081	3,777E-11	3,515E-10	chromosome assembly factor 1
PFIT_0806300	2617,3957	2509,7054	7293,573912	6013,226321	2563,550571	6653,400117	2,595384773	1,375948438	3,84E-11	3,569E-10	AAA family ATPase, putative
PFIT_1101100	2290,347	2267,8874	924,7941937	829,6256232	2279,117199	877,2099084	0,384890215	-1,377481101	4,313E-11	4,001E-10	Plasmodium exported protein (hyp5), unknown function
PFIT_0920700	182,14095	155,11039	512,5090975	516,8451788	168,625674	514,6771381	3,052187285	1,60984349	4,45E-11	4,122E-10	conserved Plasmodium protein, unknown function
PFIT_1438400	2144,433	2151,3137	5104,590611	5712,030338	2147,873362	5408,310474	2,517983867	1,332269039	4,59E-11	4,245E-10	ribonucleoside-diphosphate reductase, large subunit, putative
PFIT_0501100	5463,2223	6141,7935	2679,853125	1998,765062	5802,507932	2339,309094	0,403154829	-1,310594092	5,36E-11	4,948E-10	Plasmodium exported protein, unknown function
PFIT_0511300	606,80108	652,23439	1517,026929	1779,551417	629,5177341	1648,289173	2,618336361	1,388650443	5,43E-11	4,997E-10	stearoyl-CoA desaturase (SCD)
PFIT_0908300	29,182805	28,902558	133,2523653	176,4402507	29,04268133	154,846308	5,331680854	2,414590426	5,429E-11	4,997E-10	conserved Plasmodium membrane protein, unknown function
PFIT_0929800	80,504289	59,731953	325,7280042	255,7492523	70,11812105	290,7386282	4,146412138	2,051863522	5,559E-11	5,107E-10	conserved Plasmodium protein, unknown function
PFIT_0217400	99,624058	96,341859	306,3665494	352,8805014	97,98295866	329,6235254	3,364090347	1,750216451	5,878E-11	5,392E-10	replication factor C, subunit 2, putative
PFIT_0527800	58,36561	59,731953	222,0872756	236,14478	59,04878126	229,1160278	3,880114422	1,956099197	6,011E-11	5,504E-10	conserved Plasmodium protein, unknown function
PFIT_1355400	226,41831	258,19618	2004,480026	1461,424299	242,3072483	1732,952162	7,151879172	2,838322363	6,288E-11	5,749E-10	ubiquitin conjugating enzyme, putative
PFIT_0310300	794,97986	751,4665	2506,738941	3212,46012	773,2231798	2859,599531	3,698284797	1,886856328	6,494E-11	5,928E-10	parasite-infected erythrocyte surface protein
PFIT_0513200	543,40395	617,55132	1611,556384	1476,573209	580,4776354	1544,064797	2,659990157	1,411420907	6,645E-11	6,056E-10	conserved Plasmodium protein, unknown function
PFIT_0716600	118,74383	119,46391	355,3396409	409,0205811	119,1038661	382,180111	3,208796855	1,682032457	6,854E-11	6,236E-10	mitochondrial ribosomal protein S8 precursor, putative
PFIT_0321800	370,31973	360,31855	904,2938298	1075,572639	365,3191423	989,9332345	2,709776521	1,438173875	6,99E-11	6,35E-10	microtubule and actin binding protein, putative
PFIT_0732900	29,182805	29,865976	126,4189107	163,9646774	29,52439063	145,1917941	4,91768978	2,297980729	7,198E-11	6,528E-10	acyl-CoA synthetase
PFIT_1251500	145,91402	138,73228	474,925097	417,9317049	142,3231509	446,428401	3,136723704	1,649258456	7,281E-11	6,593E-10	thymidylate kinase
PFIT_1333900	29,182805	28,902558	137,8080018	151,4891041	29,04268133	144,6485529	4,98055091	2,316305331	7,671E-11	6,935E-10	MSP7-like protein
PFIT_0728500	31,195412	42,390418	177,6698205	159,5091155	36,79291509	168,589468	4,582117714	2,196014522	7,985E-11	7,207E-10	conserved Plasmodium protein, unknown function
PFIT_1113700	66,416039	94,415022	404,3127325	314,5626692	80,41553038	359,4377008	4,469754774	2,160195682	8,102E-11	7,301E-10	conserved Plasmodium protein, unknown function
PFIT_1339100	80,504289	71,292976	239,1709122	298,5226464	75,8986326	268,8467793	3,542182119	1,824638389	8,314E-11	7,48E-10	ABC transporter (MDR family), putative
PFIT_1215200	10,063036	19,268372	87,69600113	112,2801595	14,66570401	99,98808033	6,817816607	2,769309793	8,741E-11	7,852E-10	conserved Plasmodium protein, unknown function
PFIT_1022500	939,88758	751,4665	2240,234211	2105,698547	845,6770402	2172,966379	2,569499083	1,361487137	9,919E-11	8,896E-10	conserved Plasmodium membrane protein, unknown function
PFIT_0610000	10,063036	13,48786	80,86254649	98,91347387	11,77544823	89,88801018	7,633510709	2,932346716	1,023E-10	9,159E-10	succinate dehydrogenase subunit 3, putative
PFIT_0725600	49,308877	58,768534	238,0320031	192,4802735	54,03870568	215,2561383	3,983369615	1,993989353	1,147E-10	1,026E-09	conserved Plasmodium protein, unknown function
PFIT_0728600	193,21029	131,98835	527,3149159	455,3584248	162,5993208	491,3366703	3,021763362	1,595390686	1,171E-10	1,045E-09	conserved Plasmodium protein, unknown function
PFIT_1223500	2170,5969	1781,361	768,7636462	764,5744196	1975,97894	766,6690329	0,387994537	-1,365891757	1,197E-10	1,066E-09	mitochondrial carrier protein, putative
PFIT_1328400	2242,0445	2338,2169	5584,071344	5752,130395	2290,130691	5668,10087	2,475011968	1,307435502	1,235E-10	1,099E-09	myosin C
PFIT_0924400	154,97076	146,43963	512,5090975	413,476143	150,7051915	462,9926203	3,072174326	1,619260082	1,36E-10	1,208E-09	thiamine pyrophosphokinase
PFIT_1342700	106,66818	112,71998	400,8960052	319,018231	109,6940794	359,9571181	3,281463505	1,714339388	1,369E-10	1,215E-09	conserved Plasmodium protein, unknown function
PFIT_1343500	3397,281	3476,0143	20764,59081	15521,39539	3436,647646	18142,9931	5,279270663	2,400338634	1,536E-10	1,36E-09	conserved protein, unknown function
PFIT_0932700	2356,7631	2374,8268	14615,62055	10898,30437	2365,794951	12756,96246	5,392251961	2,43088791	1,543E-10	1,364E-09	profilin, putative
PFIT_1413500	3431,4953	3503,9534	1498,804383	1301,024071	3467,724377	1399,914227	0,40369824	-1,308650799	1,563E-10	1,38E-09	actin II
PFIT_0314900	139,8762	110,79314	511,3701884	675,4631819	125,3346705	593,4166852	4,734657083	2,243259939	1,663E-10	1,465E-09	conserved Plasmodium membrane protein, unknown function
PFIT_0406400	2791,4862	3168,6837	21733,80246	15925,06929	2980,084992	18829,43588	6,318422437	2,659564396	1,676E-10	1,475E-09	conserved Plasmodium protein, unknown function
PFIT_1001000	24220,722	23534,389	10444,93541	9326,38214	23877,55557	9885,658773	0,41401469	-1,272246136	1,7E-10	1,494E-09	alpha/beta hydrolase, putative
PFIT_1317900	1067,6881	1241,8466	5342,622614	4124,068082	1154,767352	4733,345348	4,098960142	2,035257962	1,747E-10	1,532E-09	conserved Plasmodium protein, unknown function
PFIT_0724000	286,79653	260,12302	64,91781902	88,22012534	273,4597754	76,56897218	0,28000086	-1,836496839	1,808E-10	1,582E-09	unspecified product
PFIT_0109800	167,0464	136,80544	428,2298237	479,4184589	151,9259203	453,8241413	2,987140973	1,578765325	1,809E-10	1,582E-09	replication factor c protein, putative
PFIT_0904900	82,516897	67,439301	272,1992762	353,7716138	74,97809904	312,985445	4,174358233	2,061554414	1,869E-10	1,632E-09	bacterial histone-like protein
PFIT_1476900	58,36561	64,549046	218,6705483	237,0358923	61,45732774	227,8532203	3,707502892	1,890447818	2,083E-10	1,816E-09	conserved Plasmodium protein, unknown function
PFIT_1221500	182,14095	183,04953	530,7316432	530,2118644	182,5952436	530,4717538	2,905178379	1,538626748	2,234E-10	1,945E-09	conserved Plasmodium protein, unknown function
PFIT_0710600	431,70425	339,12334	1088,797105	964,1835921	385,4137981	1026,490349	2,663346132	1,413239935	2,317E-10	2,014E-09	erythrocyte membrane protein 1, PfEMP1, putative
PFIT_1122100	241,51287	204,24474	881,5156477	1166,466102	222,8788048	1023,990875	4,594384268	2,199871527	2,341E-10	2,031E-09	protein kinase, putative
PFIT_0608400	335,0991	435,4652	1085,380378	974,8769407	385,2821541	1030,128659	2,673699387	1,418837267	2,354E-10	2,039E-09	conserved Plasmodium protein, unknown function
PFIT_1013600	2640,5407	2926,8657	20832,92536	15181,88157	2783,703187	18007,40346	6,468866202	2,693512873	2,356E-10	2,039E-09	conserved Plasmodium protein, unknown function
PFIT_1305200	144,90772	121,39074	455,5636422	368,9205242	133,1492318	412,2420832	3,096090588	1,630447684	2,41E-10	2,082E-09	conserved Plasmodium protein, unknown function
PFIT_1330300	360,2567	235,07414	808,6254649	802,0011395	297,6654157	805,3133022	2,705431198	1,435858552	2,484E-10	2,143E-09	protein kinase, putative
PFIT_1304300	99,624058	70,329557	266,5047307	302,9782083	84,97680767	284,7414695	3,350813914	1,744511569	2,528E-10	2,177E-09	conserved Plasmodium protein, unknown function
PFIT_0516900	360,2567	312,14762	919,0996482	1153,990528	336,2021594	1036,545088	3,083100626	1,624381977	2,697E-10	2,319E-09	transcription factor with AP2 domain(s)
PFIT_1126500	34,214323	21,195209	128,6967289	141,686868	27,70476601	135,1917985	4,879730744	2,286801544	2,716E-10	2,333E-09	conserved Plasmodium protein, unknown function
PFIT_0405300	108,68079	105,01263	313,200004	363,5738499	106,8467086	338,386927	3,167031829	1,663131367	2,886E-10	2,474E-09	conserved Plasmodium protein, unknown function
PFIT_0307300	95,598844	114,64681	323,450186	348,4249395	105,1228281	335,9375627	3,195667097	1,676117126	2,952E-10	2,527E-09	ATP-dependent Clp protease proteolytic subunit
PFIT_0203000	805,04289	726,41762	296,1163674	268,2248255	765,7302562	282,1705965	0,36849869	-1,440268603	2,998E-10	2,563E-09	nucleolar preribosomal assembly protein, putative
PFIT_1244200	210,31746	216,76918	715,2349183	572,0941462	213,5433196	643,6645322	3,014210575	1,591780208	3,113E-10	2,657E-09	glucose inhibited division protein a homologue, putative
PFIT_0102300	100,63036	86,707673	268,7825489	339,5138157	93,66901751	304,1481823	3,247052124	1,699130544	3,155E-10	2,688E-09	actin-related protein
PFIT_1327800	889,5724	672,46618	1933,867661	1969,358354	781,0192875	1951,613007	2,498802576	1,321236922	3,207E-10	2,727E-09	alpha/beta-hydrolase, putative
PFIT_1441600	173,08422	155,11039	683,3454633	913,3901866	164,0973078	798,367825	4,86520977	2,28250201	3,209E-10	2,727E-09	conserved Plasmodium protein, unknown function
PFIT_1331900	542,39765	479,78246	1364,413108	1268,944025	511,0900543	1316,678567	2,576216375	1,365253769	3,433E-10	2,912E-09	apurinic/apyrimidinic endonuclease Apn1, putative
PFIT_1339800	2899,1607	3142,6714	1390,608018	1103,197123	3020,916084	1246,90257	0,412756441	-1,276637368	3,764E-10	3,189E-09	RNA lariat debranching enzyme, putative
PFIT_0714000	88,554718	84,780836	282,4494582	365,3560747	86,66777722	323,9027664	3,737291723	1,901993181	3,801E-10	3,215E-09	zinc transporter, putative
PFIT_1476000	72,45386	81,89058	248,282185	277,1359493	77,17222039	262,7090672	3,404192154	1,767312474	4,322E-10	3,65E-09	trailer hitch homolog, putative
PFIT_0513700	312,96043	376,69667	858,7374656	952,5991312	344,8285473	905,6682984	2,626430745	1,393103544	4,485E-10	3,782E-09	deoxyribodipyrimidine photo-lyase, putative
PFIT_1470900	155,97706	139,6957	481,7585516	401,0005697	147,8363783	441,3795607	2,98559506	1,578018504	4,553E-10	3,833E-09	conserved Plasmodium protein, unknown function
PFIT_0403000	1058,6314	940,29655	395,2014596	370,7027489	999,4639757	382,9521043	0,383157486	-1,383990602	4,57E-10	3,842E-09	conserved Plasmodium protein, unknown function
PFIT_0927900	222,3931	218,69602	618,4276443	606,8475289	220,5445599	612,6375866	2,777840391	1,473963707	4,669E-10	3,92E-09	cytochrome c oxidase subunit 5B, putative (COX5B)
PFIT_0415800	166,0401	160,8909	439,6189147	502,5873807	163,4655009	471,1031477	2,881972925	1,527056782	4,685E-10	3,927E-09	bifunctional dihydrofolate reductase-thymidylate synthase
PFIT_1320000	92,579933	60,695371	236,893094	278,9181741	76,63765205	257,905634	3,365260118	1,750718023	5,408E-10	4,527E-09	dihydrolipamide succinyltransferase component of 2-oxoglutarate dehydrogenase complex
PFIT_0214300	1795,2457	1810,2635	4233,325145	4419,917391	1802,754594	4326,621268	2,400005682	1,263037821	5,8E-10	4,848E-09	rhoptry neck protein 6 (RON6)
PFIT_0408300	90,567326	82,853999	259,6712761	305,6515454	86,71066224	282,6614107	3,259822995	1,70479363	6,002E-10	5,009E-09	peptide chain release factor 2, putative
PFIT_0105400	121,76274	141,62253	355,3396409	426,8428287	131,6926354	391,0912348	2,969727454	1,570330534	6,469E-10	5,391E-09	2-C-methyl-D-erythritol 4-phosphate cytidylyltransferase, putative
PFIT_1442000	101,63667	113,68339	294,9774583	368,9205242	107,6600296	331,9489913	3,083307635	1,62447884	6,868E-10	5,707E-09	major facilitator superfamily, putative
PFIT_1015000	42,264752	46,244092	160,5861839	190,6980487	44,25442217	175,6421163	3,968916725	1,988745292	6,859E-10	5,707E-09	conserved Plasmodium protein, unknown function
PFIT_1252500	1772,1007	1841,0929	733,457464	727,1476998	1806,5968	730,3025819	0,404242154	-1,306708322	7,163E-10	5,942E-09	conserved Plasmodium protein, unknown function
PFIT_1016500	399,50254	348,75753	1005,65674	936,5591084	374,1300332	971,1079243	2,595642793	1,376091856	7,172E-10	5,942E-09	DNA polymerase delta catalytic subunit
PFIT_1447400	25010,67	25931,375	12477,88816	9550,051347	25471,02247	11013,96975	0,432411764	-1,209522322	7,463E-10	6,173E-09	M17 leucyl aminopeptidase
PFIT_0624900	3949,7417	4398,0059	1994,229844	1546,079974	4173,873786	1770,154909	0,424103603	-1,237511357	7,562E-10	6,246E-09	conserved Plasmodium protein, unknown function
PFIT_0526700	13,081947	18,304953	88,83493023	104,2601481	15,69345014	96,54752918	6,152090734	2,621076781	7,668E-10	6,324E-09	50S ribosomal protein L17, apicoplast, putative (RPL17)
PFIT_1148000	13,081947	6,7439301	66,05672812	88,22012534	9,912938585	77,13842673	7,781590299	2,960065024	7,793E-10	6,418E-09	conserved Plasmodium protein, unknown function
PFIT_0318800	228,43092	244,70832	740,2909186	595,263068	236,5696218	667,7769933	2,822750395	1,497101562	7,953E-10	6,541E-09	triosephosphate isomerase, putative
PFIT_0216100.1	535,35352	396,92846	1094,49165	1241,319541	466,1409922	1167,905596	2,505477131	1,32508537	8,463E-10	6,94E-09	conserved Plasmodium protein, unknown function
PFIT_0319400	201,26072	241,81807	564,8989164	639,8186868	221,539395	602,3588016	2,718969245	1,443059833	8,457E-10	6,94E-09	kinesin-8, putative
PFIT_1312600	168,0527	196,53739	517,0647339	507,0429426	182,2950484	512,0538383	2,808928946	1,490020131	9,788E-10	8,015E-09	DEAD box helicase, putative
PFIT_0928100.1	72,45386	60,695371	202,7258208	254,8581399	66,57461588	228,7919803	3,436624866	1,780992379	9,853E-10	8,056E-09	cytochrome c oxidase subunit 6B, putative (COX6B)
PFIT_1471000	142,89511	108,8663	416,8407326	341,2960405	125,8807073	379,0683866	3,011330288	1,590400954	1,044E-09	8,52E-09	conserved Plasmodium protein, unknown function
PFIT_1027100.1	75,472771	64,549046	219,8094574	255,7492523	70,01090849	237,7793548	3,396318659	1,763971826	1,053E-09	8,582E-09	centrin-3
PFIT_0316400	115,72492	84,780836	304,0887312	385,8516593	100,2528761	344,9701953	3,441000486	1,78282809б	1,13E-09	9,199E-09	conserved Plasmodium protein, unknown function
PFIT_1203000	100,63036	78,036906	267,6436398	301,1959835	89,33363385	284,4198117	3,183793152	1,670746608	1,142E-09	9,28E-09	conserved Plasmodium protein, unknown function
PFIT_1463900	143,90142	227,36679	512,5090975	520,4096283	185,6341025	516,4593629	2,782136234	1,476193067	1,323E-09	1,074E-08	cytochrome c1 precursor, putative
PFIT_0810300	63,397128	53,951441	193,6145479	222,7780943	58,67428453	208,1963211	3,548340177	1,827144326	1,392E-09	1,128E-08	conserved Plasmodium protein, unknown function
PFIT_1212300	19328,074	18253,892	8115,866286	8085,062598	18790,98282	8100,464442	0,431082531	-1,213963993	1,465E-09	1,186E-08	eukaryotic translation initiation factor 3 subunit 10, putative
PFIT_1025300	10,063036	11,561023	68,33454633	89,11123772	10,81202964	78,72289203	7,281046635	2,86414585	1,469E-09	1,187E-08	conserved Plasmodium protein, unknown function
PFIT_0303300	63,397128	65,512464	195,8923662	246,8381285	64,45479608	221,3652473	3,434426308	1,780069129	1,484E-09	1,198E-08	palmitoyl transferase
PFIT_0919500	409,56557	304,44028	845,0705563	959,7280303	357,0029237	902,3992933	2,527708412	1,337830049	1,493E-09	1,203E-08	protein disulfide isomerase
PFIT_1137600	6021,7208	5673,5721	17942,37405	14391,46489	5847,646468	16166,91947	2,764688249	1,467116809	1,498E-09	1,206E-08	subtilisin-like protease 2
PFIT_1108400	352,20627	319,85497	789,2640101	917,8457485	336,0306193	853,5548793	2,540110425	1,344891216	1,589E-09	1,277E-08	conserved Plasmodium protein, unknown function
PFIT_0827000	158,99597	141,62253	415,7018235	439,318402	150,3092523	427,5101127	2,844203575	1,50802473	1,605E-09	1,288E-08	exonuclease, putative
PFIT_0610500	45,283663	49,134348	197,0312753	166,6380145	47,20900549	181,8346449	3,851694036	1,945493106	1,632E-09	1,308E-08	conserved Plasmodium protein, unknown function
PFIT_1443600	996,24058	1148,395	2423,598577	2667,099345	1072,317771	2545,348961	2,373689058	1,247130961	1,653E-09	1,323E-08	conserved Plasmodium protein, unknown function
PFIT_1309900	6064,9919	5814,2312	23936,45267	18478,99737	5939,611553	21207,72502	3,570557575	1,836149382	1,661E-09	1,327E-08	conserved Plasmodium protein, unknown function
PFIT_0617800.2	35,220627	47,207511	167,4196385	163,073565	41,21406881	165,2466018	4,009470711	2,0034118	1,666E-09	1,329E-08	N-acetylglucosamine transferase
PFIT_0925500	100,63036	85,744255	273,3381853	308,3248825	93,18730822	290,8315339	3,120935023	1,641978321	1,682E-09	1,34E-08	protein phosphatase-beta
PFIT_1235800	6313,5489	6271,855	2775,52149	2632,345962	6292,701965	2703,933726	0,429693594	-1,218619824	1,692E-09	1,346E-08	clustered-asparagine-rich protein
PFIT_1226700	8,0504289	6,7439301	59,22327349	73,96232731	7,397179542	66,5928004	9,002458305	3,170319013	1,845E-09	1,466E-08	conserved Plasmodium protein, unknown function
PFIT_0526300	106,66818	109,82972	325,7280042	330,6026919	108,2489515	328,1653481	3,031579924	1,600069858	1,856E-09	1,473E-08	conserved Plasmodium protein, unknown function, putative
PFIT_0936500	270257,93	315213,22	140926,3349	117725,7473	292735,5764	129326,0411	0,441784503	-1,178585283	1,955E-09	1,549E-08	ring-exported protein 1 (REX1)
PFIT_0408100	254,59482	179,19586	531,8705523	618,4319898	216,8953367	575,151271	2,651745676	1,406942416	1,984E-09	1,57E-08	conserved Plasmodium protein, unknown function
PFIT_0202700	31,195412	16,378116	107,0574559	124,7557328	23,7867641	115,9065944	4,872734848	2,28473172	2,04E-09	1,612E-08	conserved Plasmodium protein, unknown function
PFIT_1437200	4620,9462	3777,5643	1778,976023	1748,362484	4199,255255	1763,669253	0,419995725	-1,251553453	2,13E-09	1,681E-08	glucose-6-phosphate isomerase
Pf_M76611100800	25,15759	33,719651	123,0021834	140,7957556	29,43862058	131,8989695	4,480473844	2,163651316	2,144E-09	1,689E-08	unspecified product
PFIT_0905000	564,53633	557,81936	1367,829836	1370,530836	561,1778471	1369,180336	2,439833188	1,286782514	2,303E-09	1,812E-08	replication protein A1, small fragment
PFIT_0915800	74,466468	65,512464	228,9207302	238,8181171	69,98946597	233,8694237	3,341494615	1,740493549	2,333E-09	1,833E-08	conserved Plasmodium protein, unknown function
PFIT_1415700	5451,1467	5978,9758	2643,408034	2328,476642	5715,061239	2485,942338	0,434980875	-1,200976124	2,384E-09	1,868E-08	ubiquitin carboxyl-terminal hydrolase, putative
PFIT_0732100	91,573629	108,8663	273,3381853	335,0582538	100,219965	304,1982196	3,035305585	1,60184177	2,383E-09	1,868E-08	Plasmodium exported protein, unknown function
PFIT_0705000	727,55752	689,80771	1531,832747	1839,255947	708,6826136	1685,544347	2,378419217	1,250003025	2,397E-09	1,876E-08	conserved Plasmodium protein, unknown function
PFIT_0911100	102,64297	92,488185	289,2829128	308,3248825	97,56557691	298,8038977	3,062595509	1,614754836	2,587E-09	2,021E-08	DNA primase large subunit, putative
PFIT_0206100	9031,575	9869,2601	43393,57583	32870,46226	9450,417512	38132,01904	4,034956022	2,012552949	2,636E-09	2,057E-08	merozoite surface protein 2
PFIT_1334200	50,315181	42,390418	181,0865478	170,202464	46,35279946	175,6445059	3,789296611	1,921930073	2,703E-09	2,106E-08	MSP7-like protein
PFIT_0308200	568,56154	537,58757	3532,896045	2569,076983	553,0745591	3050,986514	5,516410879	2,463729917	2,864E-09	2,229E-08	conserved Plasmodium protein, unknown function
PFIT_0219000	335,0991	292,87925	791,5418284	802,8922519	313,9891783	797,2170401	2,538995275	1,34425771	2,892E-09	2,247E-08	replication factor C subunit 1, putative
PFIT_1216200	77,485379	50,097767	236,893094	201,3913972	63,79157266	219,1422456	3,435285202	1,780429879	2,942E-09	2,283E-08	male development gene 1
PFIT_1218700	1690,5901	1776,5439	3950,875687	4072,383564	1733,56698	4011,629625	2,314089776	1,210444835	3,075E-09	2,383E-08	formin 2
PFIT_0321200	735,60794	849,7352	1929,312025	1852,622632	792,6715713	1890,967329	2,385562189	1,254329296	3,187E-09	2,466E-08	conserved Plasmodium protein, unknown function
PFIT_bin04300	144,90772	116,57365	370,1454593	379,6138727	130,7406853	374,879666	2,867352769	1,51971941	3,201E-09	2,474E-08	erythrocyte membrane protein 1, PfEMP1 (VAR)
PFIT_1130200	5058,6883	5882,6339	2603,546215	2202,829796	5470,661104	2403,188006	0,43928658	-1,186765669	3,258E-09	2,514E-08	RNA methyltransferase, putative
PFIT_1471900	250,5696	239,89123	569,4545528	694,1765418	245,2304151	631,8155473	2,576415927	1,365365516	3,332E-09	2,568E-08	conserved Plasmodium protein, unknown function
PFIT_0514100	38,239537	30,829395	135,5301836	150,5979917	34,5344662	143,0640877	4,142646561	2,050552739	3,34E-09	2,571E-08	tubulin-tyrosine ligase, putative
PFIT_0607800	103,64927	95,378441	284,7272764	367,1382994	99,5138566	325,9327879	3,275250292	1,71160516	3,452E-09	2,654E-08	conserved Plasmodium protein, unknown function
PFIT_0315400	56,353003	40,463581	154,8916384	199,6091725	48,40829172	177,2504054	3,661571171	1,872462838	3,738E-09	2,87E-08	conserved Plasmodium protein, unknown function
PFIT_0524700	16,100858	22,158628	105,9185468	98,91347387	19,12974275	102,4160103	5,353757847	2,420551887	3,76E-09	2,883E-08	50S ribosomal protein L17, apicoplast, putative (RPL17)
PFIT_0721100	24,151287	24,085465	103,6407286	125,6468452	24,11837581	114,6437869	4,753379241	2,248953509	3,86E-09	2,955E-08	conserved Plasmodium protein, unknown function
PFIT_0414700	635,98389	579,01457	1481,720746	1431,126478	607,49923	1456,423612	2,397408161	1,26147555	4,111E-09	3,143E-08	glutamyl-tRNA(Gln) amidotransferase subunit A, putative
PFIT_0811100	4296,9164	4047,3215	16112,14712	12435,47322	4172,118976	14273,81017	3,421237566	1,774518286	4,169E-09	3,183E-08	plasmepsin X
PFIT_0820700	15,094554	16,378116	96,80727397	137,2313061	15,73633516	117,01929	7,436247946	2,894574875	4,229E-09	3,225E-08	conserved Plasmodium protein, unknown function
PFIT_0412300	21,132376	22,158628	111,6130923	106,0423729	21,64550179	108,8277326	5,027729718	2,329907095	4,245E-09	3,232E-08	apicoplast ribosomal protein L10 precursor, putative
PFIT_0206500	55,346699	66,475883	201,5869117	214,7580829	60,9112909	208,1724973	3,417633976	1,772997894	4,371E-09	3,324E-08	iron-sulfur assembly protein, putative
PFIT_0827200	60,378217	55,878278	175,3920023	228,1247686	58,12824769	201,7583854	3,470918072	1,795317312	4,47E-09	3,394E-08	GTP binding protein, putative
PFIT_1109300	251,5759	232,18388	597,9272804	648,7298106	241,8798925	623,3285455	2,577016795	1,36570194	4,512E-09	3,421E-08	succinyl-CoA synthetase alpha subunit, putative
PFIT_1408000	138,8699	114,64681	375,8400048	351,989389	126,7583558	363,9146969	2,870932607	1,521519465	4,636E-09	3,511E-08	LCCL domain-containing protein
PFIT_1300600	5173,4069	6347,0017	2244,789847	1306,370745	5760,204291	1775,580296	0,308249535	-1,697829375	4,742E-09	3,587E-08	Plasmodium exported protein (hyp12), unknown function
PFIT_0624200	292,83435	283,24507	674,2341905	774,3766558	288,0397094	724,3054231	2,514602673	1,330330461	4,767E-09	3,6E-08	DNA polymerase 1, putative
PFIT_1003000	862,4022	714,8566	2235,678574	2878,292978	788,6293977	2556,985776	3,242316079	1,697024739	4,835E-09	3,648E-08	conserved Plasmodium protein, unknown function
PFIT_1324200	1502,4113	1599,2749	670,8174632	638,9275745	1550,843082	654,8725188	0,422268717	-1,243766722	5,085E-09	3,83E-08	U6 snRNA-associated Sm-like protein LSm6, putative
PFIT_0507400	415,60339	384,40402	1692,418931	1289,43961	400,0037061	1490,92927	3,727288641	1,898126546	5,298E-09	3,986E-08	conserved Plasmodium protein, unknown function
PFIT_0814600	151,95185	141,62253	406,5905507	408,1294688	146,7871896	407,3600097	2,775174119	1,472578292	5,406E-09	4,061E-08	conserved Plasmodium protein, unknown function
PFIT_0719300	170,06531	121,39074	432,7854601	553,3807862	145,728027	493,0831232	3,383584705	1,758552506	5,701E-09	4,277E-08	conserved Plasmodium protein, unknown function
PF_apicoplast_genon	539,37874	508,68502	1298,35638	1220,823957	524,0318778	1259,590169	2,403651804	1,265227921	5,832E-09	4,37E-08	molecular chaperone
Pf_M76611101100	361,263	219,65944	818,8756469	1001,610312	290,4612188	910,2429794	3,133784893	1,647906155	6,076E-09	4,547E-08	cytochrome c oxidase subunit 3
PFIT_1421900	163,02119	147,40304	449,8690967	404,5650193	155,2121153	427,217058	2,752472364	1,460728079	6,402E-09	4,785E-08	mannose-1-phosphate guanyltransferase, putative
PFIT_0727700	67,422342	51,061185	209,5592754	195,1536106	59,24176387	202,356443	3,415773431	1,772212284	6,548E-09	4,887E-08	conserved Plasmodium protein, unknown function
PFIT_1331400	320,00455	359,35513	1789,226205	1324,192993	339,6798426	1556,709599	4,582873057	2,196252325	6,701E-09	4,995E-08	conserved Plasmodium membrane protein, unknown function
PFIT_1337800	1106,934	1343,0055	7722,942645	5593,512392	1224,969748	6658,227518	5,435421999	2,442392049	6,772E-09	5,041E-08	conserved Plasmodium protein, unknown function
PFIT_0913700	160,00228	131,02493	374,7010957	422,3872668	145,5136018	398,5441813	2,738879226	1,45358565	7,008E-09	5,21E-08	conserved Plasmodium protein, unknown function
PFIT_0105500	110,6934	96,341859	433,9243692	596,1541804	103,5176286	515,0392748	4,97537745	2,314805978	7,117E-09	5,284E-08	centrin-1
PFIT_1322800	104,65558	107,90288	329,1447315	297,631534	106,2792293	313,3881327	2,948724176	1,560090879	7,422E-09	5,503E-08	conserved Plasmodium protein, unknown function
PFIT_1017700	1132,0916	825,64973	4931,476427	6950,676542	978,8706515	5941,076485	6,069317203	2,601534223	7,433E-09	5,504E-08	serine/threonine protein phosphatase, putative
PFIT_1348800	119,75013	133,91518	357,6174591	360,0094004	126,8326574	358,8134298	2,829030292	1,500307625	7,548E-09	5,582E-08	conserved Plasmodium protein, unknown function
PFIT_1033800	172,07792	124,281	366,728732	436,6450648	148,1794585	401,6868984	2,710813648	1,43872594	7,809E-09	5,767E-08	flavoprotein subunit of succinate dehydrogenase
PFIT_1004100	64,403432	60,695371	281,3105491	390,3072212	62,54940141	335,8088851	5,368698622	2,42457242	7,839E-09	5,782E-08	conserved Plasmodium protein, unknown function
PFIT_0524300	120,75643	103,08579	328,0058224	319,9093434	111,9211117	323,9575829	2,8945172	1,533322729	7,92E-09	5,834E-08	conserved Plasmodium protein, unknown function, putative
PFIT_1449700	1381,6549	1785,2147	3542,007318	3648,214072	1583,434759	3595,110695	2,270450788	1,182978766	8,183E-09	6,02E-08	conserved Plasmodium protein, unknown function
PFIT_1307900	52,327788	41,426999	149,1970928	195,1536106	46,87739378	172,1753517	3,672886605	1,876914356	8,289E-09	6,09E-08	conserved Plasmodium protein, unknown function
PFIT_1024700	51,321484	35,646488	149,1970928	171,9846888	43,4839862	160,5908908	3,693104171	1,884833957	8,491E-09	6,231E-08	conserved Plasmodium protein, unknown function
PFIT_0501800	57,359306	46,244092	167,4196385	194,2624982	51,80169931	180,8410684	3,491025793	1,803651016	8,502E-09	6,231E-08	anaphase promoting complex subunit, putative
PFIT_1120500	480,00683	476,8922	1220,910561	1077,354864	478,4495143	1149,132713	2,401784678	1,264106818	9,186E-09	6,723E-08	conserved Plasmodium protein, unknown function
PFIT_0917300	21,132376	30,829395	116,1687288	118,5179462	25,98088546	117,3433375	4,516525723	2,175213425	9,549E-09	6,98E-08	conserved Plasmodium membrane protein, unknown function
PFIT_1209100.1	250,5696	273,61088	994,2676491	766,3566444	262,0902404	880,3121468	3,358813153	1,747951542	9,591E-09	7,001E-08	cGMP-specific phosphodiesterase
PFIT_0713900	56,353003	87,671092	214,1149118	243,273679	72,01204723	228,6942954	3,175778279	1,667110192	1,039E-08	7,573E-08	drug/metabolite exporter, drug/metabolite transporter
PFIT_0721400	41,258448	13,48786	112,7520014	139,0135308	27,3731543	125,8827661	4,598767273	2,20124719	1,098E-08	7,994E-08	conserved Plasmodium protein, unknown function
PFIT_0936600	11202,172	9367,319	4431,49533	4778,144567	10284,74542	4604,819948	0,447733003	-1,159289431	1,111E-08	8,077E-08	ring-exported protein 2
PFIT_0729800	12490,24	11341,364	5375,650978	5380,536534	11915,80208	5378,093756	0,451341313	-1,147709255	1,177E-08	8,545E-08	Plasmodium exported protein, unknown function
PFIT_1231200	1632,2245	1661,8971	4658,138242	3734,651973	1647,060769	4196,395107	2,547808305	1,349256735	1,199E-08	8,696E-08	amino acid transporter, putative
PFIT_0932000	205,28594	210,98867	547,8152798	526,6474149	208,1373048	537,2313473	2,581139156	1,368007923	1,215E-08	8,801E-08	conserved Plasmodium protein, unknown function
PFIT_0800200	987,18385	1120,4558	464,6749151	425,9517163	1053,819836	445,3133157	0,422570634	-1,242735584	1,267E-08	9,158E-08	rifin, pseudogene
PFIT_0529500	471,9564	461,47751	2278,95712	1691,331292	466,7169511	1985,144206	4,253422126	2,088624041	1,268E-08	9,158E-08	conserved Plasmodium protein, unknown function
PFIT_0923300	3775,6512	3894,1379	1785,809477	1650,340123	3834,894561	1718,0748	0,448010962	-1,158394063	1,272E-08	9,168E-08	flavodoxin-like protein
PFIT_0507500	2693,8748	2781,3895	16194,14857	11726,14777	2737,63213	13960,14817	5,099351378	2,350313752	1,272E-08	9,168E-08	subtilisin-like protease 1
PFIT_1360600	40,252145	38,536744	146,9192746	152,3802165	39,39444419	149,6497456	3,798752556	1,925525741	1,299E-08	9,347E-08	conserved Plasmodium protein, unknown function
PFIT_1226300	123,77534	118,50049	335,9781861	343,0782652	121,1379159	339,5282257	2,802823734	1,486881018	1,34E-08	9,631E-08	proliferating cell nuclear antigen 2
PFIT_1209500	102,64297	133,91518	353,0618227	319,018231	118,2790766	336,0400269	2,84107753	1,506438202	1,376E-08	9,877E-08	ABC transporter, (TAP family), putative
PFIT_1101800	1134,1042	1278,4565	601,3440077	445,5561886	1206,280324	523,4500982	0,433937359	-1,204441297	1,418E-08	1,017E-07	Plasmodium exported protein (hyp4), unknown function
PFIT_1014500	88,554718	90,561348	239,1709122	290,502635	89,55803299	264,8367736	2,957152639	1,564208713	1,455E-08	1,042E-07	FAD synthetase, putative
PFIT_1239200	124,78165	142,58595	330,2836406	397,4361202	133,6838001	363,8598804	2,721794864	1,444558338	1,501E-08	1,073E-07	DNA gyrase subunit B
PFIT_1239500	1815,3717	1597,348	3754,983321	4668,537744	1706,359876	4211,760533	2,468272134	1,303501465	1,519E-08	1,085E-07	conserved Plasmodium protein, unknown function
PFIT_1413700	1736,88	1915,2762	867,8487384	744,9699473	1826,078102	806,4093429	0,441607258	-1,179164213	1,564E-08	1,116E-07	AAA family ATPase, putative
PFIT_0202400	432,71056	366,09906	873,543284	1009,630323	399,4048102	941,5868037	2,357474871	1,237242393	1,649E-08	1,174E-07	protein kinase, putative
PFIT_0313900	182,14095	165,708	448,7301876	460,705099	173,9244763	454,7176433	2,614454578	1,386510006	1,649E-08	1,174E-07	co-chaperone p23
PFIT_0711100	139,8762	136,80544	33,02836406	36,53560747	138,3408214	34,78198576	0,251422432	-1,991814722	1,68E-08	1,193E-07	rifin
PFIT_1011400	381,38907	481,7093	1476,026201	1161,119428	431,5491835	1318,572814	3,05544041	1,611380344	1,681E-08	1,193E-07	heme oxygenase
PFIT_1466100	69,43495	56,841697	177,6698205	227,2336562	63,13832327	202,4517383	3,206479486	1,680990177	1,696E-08	1,203E-07	conserved Plasmodium protein, unknown function
PFIT_1403600	5345,4848	6617,7223	2597,85167	2080,747401	5981,603562	2339,299535	0,391082343	-1,354455694	1,709E-08	1,21E-07	pre-mRNA splicing factor, putative
PFIT_1230100	178,11574	172,45193	422,5352782	485,6562456	175,2838341	454,0957619	2,590631156	1,373303625	1,785E-08	1,263E-07	heat shock protein hslv
PFIT_bin09300	464,91227	424,8676	190,1978206	152,3802165	444,8899352	171,2890186	0,385014371	-1,377015798	1,802E-08	1,273E-07	alpha/beta hydrolase, putative
PFIT_0928100.2	35,220627	26,975721	121,8632743	131,8846318	31,09817359	126,8739531	4,079787923	2,02849416	1,851E-08	1,304E-07	cytochrome c oxidase subunit 6B, putative (COX6B)
PFIT_0619300	361,263	440,2823	2780,077127	1972,031691	400,7726476	2376,054409	5,928684063	2,567711918	1,85E-08	1,304E-07	merozoite surface protein 10
PFIT_1356500	397,48993	345,86727	838,2371017	924,0835352	371,6786017	881,1603184	2,370758807	1,245348895	1,887E-08	1,328E-07	conserved Plasmodium protein, unknown function
PFIT_0829000	61142,001	66674,347	31134,35822	28093,2088	63908,17429	29613,78351	0,463380215	-1,10973165	1,92E-08	1,349E-07	E3 ubiquitin-protein ligase, putative
PFIT_1448600	659,12887	751,4665	298,3941857	281,5915112	705,2976856	289,9928484	0,41116376	-1,282214984	2,079E-08	1,459E-07	conserved Plasmodium protein, unknown function
PFIT_1306600	31,195412	32,756232	148,0581837	114,0623843	31,97582213	131,060284	4,098730705	2,035177205	2,082E-08	1,459E-07	conserved Plasmodium protein, unknown function
PFIT_1032200	24,151287	34,683069	129,835638	117,6268338	29,41717807	123,7312359	4,206087873	2,072478991	2,101E-08	1,471E-07	leucine-rich repeat protein
PFIT_1218300	4913,7806	5704,4015	2690,103307	2202,829796	5309,091024	2446,466552	0,460807046	-1,117765318	2,144E-08	1,499E-07	arginine--tRNA ligase, putative
PFIT_0412800.1	11,06934	13,48786	72,89018275	79,30900157	12,27860004	76,09959216	6,197741755	2,631742643	2,159E-08	1,508E-07	calmodulin-like protein
PFIT_1030700	339,12432	486,52639	998,8232856	959,7280303	412,825354	979,2756579	2,372130608	1,246183446	2,186E-08	1,524E-07	conserved Plasmodium protein, unknown function
PFIT_1358700	578,62458	553,00227	1544,360747	2014,805085	565,8134259	1779,582916	3,145176191	1,653140839	2,19E-08	1,526E-07	conserved Plasmodium protein, unknown function
PFIT_1424800	6353,801	7242,981	3350,670589	2918,393035	6798,391008	3134,531812	0,461069657	-1,116943369	2,231E-08	1,551E-07	conserved Plasmodium protein, unknown function
PFIT_0911400	17,107161	19,268372	78,58472828	105,1512605	18,18776667	91,8679944	5,051087144	2,336593932	2,23E-08	1,551E-07	conserved Plasmodium protein, unknown function
PFIT_1230500	2618,402	2312,2046	1021,601468	1161,119428	2465,303317	1091,360448	0,442688103	-1,175637494	2,288E-08	1,588E-07	conserved Plasmodium protein, unknown function
PFIT_0617400	156,98336	122,35416	338,2560043	404,5650193	139,6687628	371,4105118	2,659223899	1,411005253	2,311E-08	1,602E-07	malate dehydrogenase
PFIT_0614500	475,98161	576,12432	1186,743288	1244,883991	526,0529645	1215,813639	2,311200053	1,208642142	2,341E-08	1,621E-07	cdc2-related protein kinase 5
PFIT_1231800	43,271056	46,244092	140,08582	176,4402507	44,75757398	158,2630353	3,536005669	1,822120588	2,348E-08	1,624E-07	phenylalanyl-tRNA synthetase alpha chain, putative
PFIT_1101600	1138,1294	1243,7734	620,7054625	430,4072782	1190,951397	525,5563704	0,441291199	-1,180197119	2,36E-08	1,63E-07	Plasmodium exported protein (hyp4), unknown function
PFIT_1340700	259,62633	268,79379	1062,602195	805,565589	264,2100602	934,0838922	3,535383518	1,821866727	2,461E-08	1,697E-07	conserved Plasmodium protein, unknown function
PFIT_0105300	117,73752	133,91518	351,9229136	339,5138157	125,8263538	345,7183647	2,747583112	1,458163122	2,468E-08	1,7E-07	Rab GTPase 5c
PFIT_0415100	107,67449	135,84202	693,5956453	504,3696055	121,7582543	598,9826254	4,919441634	2,298494576	2,521E-08	1,735E-07	repressor of RNA polymerase III transcription MAF1, putative
PFIT_1249800	111,6997	156,07381	380,3956413	348,4249395	133,8867567	364,4102904	2,721779953	1,444550434	2,716E-08	1,867E-07	leucine-rich repeat protein
PFIT_0703400	945,9254	728,34446	3874,568777	5484,796682	837,134928	4679,682729	5,590117642	2,482878644	2,755E-08	1,891E-07	serine/threonine protein kinase, putative
PFIT_0323500	4044,3342	4082,0046	17974,2635	13413,0235	4063,169406	15693,6435	3,862414271	1,949502912	2,894E-08	1,984E-07	Rh5 interacting protein (RIPR)
PFIT_0513900	115,72492	102,12237	292,6996401	314,5626692	108,9236434	303,6311546	2,787559663	1,479002684	3,01E-08	2,061E-07	Rab GTPase 1a
PFIT_1109700	1397,7557	1561,7015	757,3745552	569,420809	1479,728631	663,3976821	0,448323881	-1,157386746	3,024E-08	2,068E-07	conserved Plasmodium protein, unknown function
PFIT_1034700	4738,6837	5442,3516	35582,93718	25049,16892	5090,51768	30316,05305	5,955396869	2,574197653	3,033E-08	2,072E-07	glutamate-rich protein
PFIT_0728800	439,75468	490,38006	1116,130923	1047,948156	465,0673721	1082,03954	2,326629655	1,218241586	3,12E-08	2,129E-07	dynein heavy chain, putative
PFIT_0921300	7984,0129	8290,217	3852,929504	3683,858567	8137,114943	3768,394036	0,463111811	-1,110567544	3,204E-08	2,183E-07	conserved Plasmodium membrane protein, unknown function
PFIT_0310800	377,36386	394,0382	994,2676491	835,8634098	385,7010303	915,0655295	2,372473645	1,246392061	3,212E-08	2,187E-07	conserved Plasmodium protein, unknown function
PFIT_0211000	32,201716	25,048883	128,6967289	110,4979348	28,62529958	119,5973319	4,178029003	2,062822507	3,299E-08	2,243E-07	dolichol-linked oligosaccharide biosynthesis enzyme, putative
PFIT_1428400	538,37244	625,25867	1349,60729	1309,935194	581,8155508	1329,771242	2,285554658	1,192544321	3,336E-08	2,266E-07	conserved Plasmodium protein, unknown function
PFIT_1027400	2862,9338	2634,9498	1291,522926	1206,566159	2748,94182	1249,044542	0,454372855	-1,138051448	3,36E-08	2,279E-07	methyltransferase, putative
PFIT_0925300	3024,9487	3607,0392	1589,917111	1452,513175	3315,993941	1521,215143	0,458750881	-1,124217165	3,375E-08	2,287E-07	rRNA processing and telomere maintaining methyltransferase, putative
PFIT_0902500	7198,0898	7752,6294	3973,653869	3053,842117	7475,359593	3513,747993	0,470044009	-1,089132255	3,465E-08	2,344E-07	serine/threonine protein kinase, FIKK family
PFIT_0819100	128,80686	96,341859	280,17164	335,9493662	112,5743611	308,0605031	2,736506786	1,452335434	3,482E-08	2,353E-07	vacuolar protein-sorting protein VPS2, putative
PFIT_0802000	8574,7131	7876,9104	3507,840045	4043,867968	8225,811766	3775,854006	0,459025092	-1,123355075	3,717E-08	2,509E-07	unspecified product
PFIT_1238400	79,497986	69,366139	218,6705483	230,7981057	74,4320622	224,734327	3,019321517	1,594224393	4,016E-08	2,708E-07	conserved Plasmodium protein, unknown function
PFIT_1115700	720,51339	808,3082	1562,583293	1824,107036	764,4107944	1693,345164	2,215229268	1,14745602	4,155E-08	2,798E-07	conserved Plasmodium protein, unknown function
PFIT_1208900	290,82175	216,76918	840,5149199	1128,14827	253,7954643	984,3315947	3,87844439	1,955478116	4,257E-08	2,863E-07	zinc finger transcription factor, putative (KROX1)
PFIT_0813700	1053,5999	1021,2237	492,0087336	417,0405925	1037,411797	454,5246631	0,438133308	-1,190558197	4,302E-08	2,89E-07	dihydropteroate synthetase
PFIT_1365200	153,96445	174,37877	440,7578238	409,0205811	164,1716093	424,8892025	2,588079658	1,371882022	4,335E-08	2,909E-07	conserved Plasmodium protein, unknown function
PFIT_1423100	465,91857	455,69699	962,3781942	1133,494944	460,8077844	1047,936569	2,274129484	1,1853144	4,363E-08	2,924E-07	mitochondrial ribosomal protein L21 precursor, putative
PFIT_1352400	25386,021	24635,577	12057,6307	11471,28963	25010,79909	11764,46017	0,470375222	-1,088116031	4,578E-08	3,064E-07	membrane associated histidine-rich protein
PFIT_0723000	718,50078	521,20946	1350,746199	1417,759792	619,8551206	1384,252996	2,233187966	1,159104687	4,91E-08	3,283E-07	kelch protein, putative
PF_apicoplast_genon	25,15759	23,122046	96,80727397	113,1712719	24,13981832	104,9892729	4,349215538	2,120755208	4,989E-08	3,332E-07	apicoplast ribosomal protein L16
PFIT_0723600	1829,46	1543,3966	7236,628457	5412,616579	1686,42828	6324,622518	3,750306249	1,90700841	5,083E-08	3,391E-07	conserved Plasmodium protein, unknown function
PFIT_0614800	124,78165	113,68339	299,5330948	341,2960405	119,2325212	320,4145676	2,687308499	1,42616195	5,117E-08	3,409E-07	conserved Plasmodium protein, unknown function
PFIT_0604600	62,390824	66,475883	218,6705483	187,1335992	64,43335357	202,9020737	3,149022401	1,65490402	5,33E-08	3,547E-07	conserved Plasmodium protein, unknown function
PFIT_1443800	162,01488	134,8786	381,5345504	387,6338841	148,4467426	384,5842172	2,590721833	1,373354121	5,592E-08	3,717E-07	TRAP-like protein,sporozoite-specific transmembrane protein S6 (TREP)
PFIT_0312000	71,447557	60,695371	177,6698205	225,4514314	66,07146407	201,5606259	3,050645673	1,609114623	5,698E-08	3,783E-07	RING zinc finger protein, putative
PFIT_0702300	1167,3122	1337,225	600,2050986	522,191853	1252,268601	561,1984758	0,44814545	-1,157961045	5,884E-08	3,902E-07	conserved Plasmodium protein, unknown function
PFIT_0602700	368,30712	289,989	1357,579654	1906,980487	329,1480601	1632,280071	4,959105851	2,31008002	6,161E-08	4,081E-07	conserved Plasmodium protein, unknown function
PFIT_0318500	2258,1453	2919,1583	5958,77244	7425,639439	2588,651826	6692,20594	2,585208978	1,370280907	6,256E-08	4,139E-07	conserved Plasmodium protein, unknown function
PFIT_0316300.1	135,85099	117,53707	507,9534611	711,107677	126,6940283	609,530569	4,811044193	2,266350052	6,309E-08	4,169E-07	inorganic pyrophosphatase, putative
PFIT_1347900	38,239537	28,902558	128,6967289	127,4290699	33,57104761	128,0628994	3,814682846	1,931563116	6,349E-08	4,191E-07	conserved Plasmodium protein, unknown function
PFIT_1013500	47,29627	58,768534	168,5585476	178,2224754	53,03240207	173,3905115	3,269520232	1,709078951	6,844E-08	4,512E-07	GDP-fucose synthase, putative
PFIT_1121800	141,88881	171,48851	435,0632783	375,1583108	156,6886597	405,1107946	2,585450634	1,370415758	6,873E-08	4,526E-07	zinc finger, DHHC-type, putative
PFIT_0103700	133,83838	127,17125	383,8123686	312,7804444	130,5048176	348,2964065	2,668839456	1,416212523	6,965E-08	4,581E-07	conserved Plasmodium protein, unknown function
PFIT_0410500	453,84293	539,51441	1117,269833	1126,366045	496,6786715	1121,817939	2,258639243	1,175453857	7,071E-08	4,645E-07	DNA polymerase alpha
PFIT_1457700	416,6097	362,24539	957,8225578	1234,190642	389,4275441	1096,0066	2,814404417	1,492829652	7,276E-08	4,775E-07	conserved Plasmodium protein, unknown function
PFIT_1112000	771,83487	798,67401	340,5338226	336,8404786	785,2544437	338,6871506	0,431308798	-1,213206948	7,946E-08	5,202E-07	conserved Plasmodium protein, unknown function
PFIT_1143200	4424,717	3879,6867	1913,367297	1961,338342	4152,201838	1937,35282	0,466584452	-1,099789861	7,943E-08	5,202E-07	60S ribosomal protein L28, putative
PFIT_0420700	13401,952	12198,806	5923,466258	6175,408774	12800,37889	6049,437516	0,472598316	-1,081313605	7,988E-08	5,219E-07	pre-mRNA-splicing helicase BRR2, putative
PFIT_1337000	6123,3575	5925,9878	16412,81912	13076,18302	6024,672636	14744,50107	2,44735307	1,291222248	7,99E-08	5,219E-07	calcium-dependent protein kinase 5
PFIT_1013200	28,176501	28,902558	113,8909106	115,844609	28,53952952	114,8677598	4,024865221	2,008940473	8,054E-08	5,254E-07	conserved Plasmodium protein, unknown function
PFIT_1249700	24,151287	37,573325	115,0298197	125,6468452	30,86230595	120,3383324	3,899200941	1,963178504	8,318E-08	5,421E-07	cutA, putative
PFIT_0422300	1947,1975	2043,4108	9097,605935	6694,92729	1995,304167	7896,266613	3,95742501	1,984562013	8,749E-08	5,695E-07	reticulocyte binding protein homologue 5
PFIT_1434500	373,33864	421,97734	1539,805111	1162,901652	397,6579927	1351,353381	3,398280448	1,764804918	9,004E-08	5,854E-07	chromatin assembly factor 1 P55 subunit, putative
PFIT_1021500	3928,6093	3845,0036	1970,312753	1708,262427	3886,806462	1839,28759	0,473213063	-1,079438196	9,222E-08	5,989E-07	RNA binding protein, putative
PFIT_0217500	35,220627	19,268372	152,6138201	221,8869819	27,24449923	187,250401	6,872961749	2,78093193	9,332E-08	6,054E-07	conserved Plasmodium membrane protein, unknown function
PFIT_0907110	1833,4852	2229,3506	950,9891031	658,5320468	2031,417906	804,7605749	0,396157075	-1,335855529	9,619E-08	6,233E-07	tubulin-specific chaperone, putative
PFIT_1308300	27347,307	24226,124	13070,1209	11739,51446	25786,71551	12404,81768	0,481054583	-1,055727496	9,972E-08	6,454E-07	60S ribosomal protein L24, putative
PFIT_0403200	1858,6428	2074,2402	995,4065582	840,3189717	1966,441505	917,862765	0,46676332	-1,099236903	1,041E-07	6,731E-07	6-cysteine protein
PFIT_1125100	299,87848	321,78181	796,0974648	661,2053839	310,8301439	728,6514243	2,344210942	1,229102395	1,061E-07	6,854E-07	conserved Plasmodium protein, unknown function
PFIT_1219100	697,36841	451,84332	58,08436438	54,35785501	574,6058633	56,2211097	0,09784291	-3,353388879	1,068E-07	6,891E-07	erythrocyte membrane protein 1 (PfEMP1), pseudogene
PFIT_1345700	2194,7482	2441,3027	1143,464742	1029,234796	2318,025451	1086,349769	0,468653081	-1,093407728	1,116E-07	7,193E-07	conserved Plasmodium protein, unknown function
PFIT_0617800.1	75,472771	79,963743	207,2814572	272,6803874	77,71825722	239,9809223	3,087832009	1,626594266	1,121E-07	7,215E-07	N-acetylglucosamine transferase
PFIT_0908600	20,126072	23,122046	129,835638	192,4802735	21,62405928	161,1579558	7,452715222	2,897766134	1,132E-07	7,276E-07	conserved Plasmodium protein, unknown function
PFIT_1227900	150,94554	130,06151	402,0349143	327,0382424	140,5035263	364,5365783	2,594501277	1,375457246	1,134E-07	7,281E-07	conserved Plasmodium protein, unknown function
PFIT_1148900	4843,3393	6428,8923	1868,949842	1151,317191	5636,115788	1510,133517	0,267938696	-1,900025141	1,167E-07	7,485E-07	Plasmodium exported protein (PHISTc), unknown function
PFIT_1427000	12852,51	11944,464	6178,581898	5741,437046	12398,48675	5960,009472	0,480704588	-1,05677752	1,173E-07	7,517E-07	60S ribosomal protein L21e, putative
PFIT_1424300	3731,3738	3759,2593	22512,81629	15901,90037	3745,31658	19207,35833	5,128367101	2,358499537	1,175E-07	7,517E-07	serine/threonine protein phosphatase
PFIT_1208000	230,44353	257,23276	522,7592794	622,8875517	243,8381462	572,8234156	2,349195253	1,232166628	1,186E-07	7,582E-07	amino acid transporter, putative
PFIT_1136700	281,76501	263,97669	564,8989164	695,9587666	272,8708535	630,4288415	2,310356102	1,208115235	1,205E-07	7,691E-07	conserved Plasmodium protein, unknown function
PFIT_1243200	79,497986	55,878278	183,364366	217,43142	67,68813206	200,397893	2,960606046	1,565892531	1,251E-07	7,971E-07	syntaxin, Qa-SNARE family
PFIT_1360900	199,24812	162,81774	626,4000081	862,5967811	181,0329292	744,4983946	4,112502615	2,040016596	1,251E-07	7,971E-07	proliferating cell nuclear antigen
PFIT_1336000	2701,9252	3359,4406	1401,997109	1204,783934	3030,682922	1303,390521	0,430064957	-1,217373513	1,274E-07	8,095E-07	nuclear movement protein, putative
PFIT_1201900	117,73752	96,341859	259,6712761	310,1071073	107,0396912	284,8891917	2,661528527	1,412255029	1,273E-07	8,095E-07	mitochondrial phosphate carrier protein
PFIT_1225900	514,22115	652,23439	239,1709122	227,2336562	583,2277677	233,2022842	0,399847705	-1,322477489	1,284E-07	8,152E-07	conserved Plasmodium protein, unknown function
PFIT_bin09700	14993,924	10578,336	2296,040757	2661,752671	12786,13002	2478,896714	0,193873886	-2,366809603	1,297E-07	8,224E-07	erythrocyte membrane protein 1, PfEMP1 (VAR)
PFIT_1466400	2178,6473	2330,5096	1124,103287	998,9369749	2254,578453	1061,520131	0,470828651	-1,086725981	1,351E-07	8,56E-07	tRNA 3'-trailer sequence RNase, putative
PFIT_1402100	105,66188	106,93946	318,8945496	259,3137018	106,3006718	289,1041257	2,71968296	1,443438483	1,402E-07	8,868E-07	Plasmodium exported protein, unknown function
PFIT_1439600	1894,8697	1468,2499	5801,602984	7990,604686	1681,559823	6896,103835	4,101016057	2,035981392	1,406E-07	8,884E-07	metacaspase-like protein
PFIT_0927800	124,78165	63,585627	251,6989123	287,8292978	94,18363782	269,7641051	2,864235353	1,518150043	1,447E-07	9,133E-07	serine/threonine protein phosphatase, putative
PFIT_0309900	73,460164	95,378441	233,4763666	243,273679	84,41930234	238,3750228	2,823702829	1,497588265	1,451E-07	9,147E-07	conserved Plasmodium protein, unknown function
PFIT_1035400	2023,6766	2159,9845	12633,91871	8898,648199	2091,830529	10766,28345	5,146823944	2,363682434	1,475E-07	9,289E-07	merozoite surface protein
PFIT_0519800	4155,0276	3475,0509	1815,421114	1803,611451	3815,039249	1809,516283	0,474311315	-1,07609381	1,49E-07	9,363E-07	zinc finger protein, putative
PFIT_0514400	165,03379	169,56167	408,8683689	418,8228173	167,2977327	413,8455931	2,473707123	1,306674701	1,489E-07	9,363E-07	aspartate--tRNA ligase, putative
PFIT_1352600	4025,2145	4427,8718	2124,065482	1928,367184	4226,543159	2026,216333	0,479402731	-1,060689967	1,496E-07	9,393E-07	Ran-binding protein, putative
PFIT_1030200	295,85326	304,44028	757,3745552	638,9275745	300,1467693	698,1510648	2,326032249	1,217871099	1,511E-07	9,473E-07	calmodulin, putative
PFIT_0627200	19725,564	18554,479	43131,62674	36475,90294	19140,02109	39803,76484	2,079609247	1,056312475	1,587E-07	9,943E-07	choline/ethanolaminephosphotransferase, putative
PFIT_1015400	75843,091	74060,877	39118,11105	34252,57756	74951,98423	36685,3443	0,489451276	-1,030762846	1,625E-07	1,017E-06	enolase
PFIT_0322600	127,80056	144,51279	367,8676411	330,6026919	136,1566741	349,2351665	2,564950773	1,358931138	1,791E-07	1,119E-06	iron-sulfur cluster assembly accessory protein, putative
PFIT_1003400	9347,5543	7707,3487	3653,620411	4306,746119	8527,451519	3980,183265	0,466749445	-1,099279788	1,796E-07	1,121E-06	U5 small nuclear ribonuclear protein, putative
PFIT_1119500	165,03379	123,31758	674,2341905	978,4413902	144,1756865	826,3377903	5,731464231	2,518903755	1,798E-07	1,121E-06	conserved Plasmodium protein, unknown function
PFIT_0833600	1874,7436	2056,8987	1034,129468	834,0811851	1965,821167	934,1053265	0,475173094	-1,073474946	1,804E-07	1,122E-06	Plasmodium exported protein (hyp9), unknown function
PFIT_1009700	12835,403	13759,544	7061,236454	5968,670703	13297,47349	6514,953578	0,489939204	-1,029325356	1,802E-07	1,122E-06	DNA2/NAM7 helicase, putative
PFIT_1430600	3259,4174	3243,8304	1596,750566	1508,653255	3251,623908	1552,70191	0,477515836	-1,066379515	1,812E-07	1,126E-06	rRNA (adenosine-2'-O-)-methyltransferase, putative
PFIT_1334300	6574,1815	7419,2866	60079,73314	40325,50841	6996,734055	50202,62077	7,175150631	2,843009119	1,868E-07	1,16E-06	merozoite surface protein 7
PFIT_0711000	298,87217	213,87893	601,3440077	588,134169	256,3755509	594,7390883	2,319796432	1,213998211	1,93E-07	1,197E-06	C_ITvar28 - erythrocyte membrane protein 1, PfEMP1, putative
PFIT_0102600	157,98967	176,3056	1032,990559	724,4743627	167,1476351	878,7324607	5,257223412	2,394301045	1,936E-07	1,199E-06	conserved Plasmodium membrane protein, unknown function
PFIT_1114100	198,24181	128,13467	437,3410965	556,9452358	163,1882427	497,1431661	3,046439854	1,607124257	1,955E-07	1,21E-06	UDP-galactose transporter, putative
PFIT_0623100	56,353003	52,024604	166,2807294	172,8758012	54,18880327	169,5782653	3,129396758	1,645884581	1,98E-07	1,224E-06	conserved Plasmodium protein, unknown function
PFIT_0423000	2337,6433	2422,0343	5620,516436	4566,059821	2379,838822	5093,288128	2,140181966	1,097733465	2,012E-07	1,242E-06	Plasmodium exported protein (PHISTb), unknown function
PFIT_1148200	62,390824	55,878278	164,0029112	218,3225324	59,13455131	191,1627218	3,23267392	1,69272799	2,069E-07	1,276E-06	conserved Plasmodium protein, unknown function
PFIT_0911300	3787,7268	4164,8586	19487,87371	14076,90222	3976,292695	16782,38796	4,220611824	2,077452149	2,085E-07	1,285E-06	conserved Plasmodium protein, unknown function
PFIT_0906300	68,428646	98,268696	361,0341865	270,0070503	83,34867119	315,5206184	3,785550674	1,92050318	2,186E-07	1,345E-06	developmental protein, putative
PFIT_0505000	3400,2999	3097,3908	1457,803655	1627,171201	3248,845348	1542,487428	0,474780195	-1,074668339	2,213E-07	1,357E-06	conserved Plasmodium membrane protein, unknown function
PFIT_1239300	59,371913	63,585627	192,4756388	180,0047002	61,47877025	186,2401695	3,029341165	1,599004064	2,211E-07	1,357E-06	hydroxyethylthiazole kinase, putative
PFIT_1433900	44,277359	40,463581	129,835638	153,2713289	42,37047002	141,5534835	3,34085233	1,740216215	2,212E-07	1,357E-06	protein-L-isoaspartate O-methyltransferase beta-aspartate methyltransferase, putative
PFIT_bin01500	359,25039	394,0382	168,5585476	138,1224185	376,6442978	153,340483	0,407122805	-1,296464058	2,226E-07	1,364E-06	Plasmodium exported protein (PHISTa), unknown function
PFIT_0204500	4,0252145	4,817093	46,69527333	43,66450648	4,421153715	45,17988991	10,21902716	3,353185955	2,249E-07	1,376E-06	conserved Plasmodium protein, unknown function
PFIT_1361500	1287,0623	900,79638	2251,623302	2328,476642	1093,929355	2290,049972	2,093416692	1,065859507	2,287E-07	1,399E-06	exonuclease, putative
PFIT_1236500	92,579933	90,561348	382,6734595	548,9252244	91,57064023	465,7993419	5,086776075	2,346751587	2,299E-07	1,404E-06	protein-S-isoprenylcysteine O-methyltransferase, putative
PFIT_0924700	348,18105	478,81904	890,6269205	939,2324456	413,5000459	914,9296831	2,212647113	1,14577338	2,315E-07	1,413E-06	conserved Plasmodium protein, unknown function
PFIT_0715100	17,107161	18,304953	77,44581918	110,4979348	17,70605737	93,97187698	5,307329294	2,407986064	2,319E-07	1,413E-06	conserved Plasmodium membrane protein, unknown function
PFIT_1357700	398,49623	343,94044	813,1811014	836,7545222	371,2183349	824,9678118	2,222325069	1,152069861	2,391E-07	1,456E-06	zinc finger protein, putative
PFIT_0605500	8776,9802	8992,5491	4410,994966	4263,081613	8884,764645	4337,038289	0,488143295	-1,03462338	2,431E-07	1,479E-06	conserved Plasmodium protein, unknown function
PFIT_1217800	124,78165	102,12237	298,3941857	292,2848597	113,4520097	295,3395227	2,603211028	1,380292268	2,539E-07	1,542E-06	conserved Plasmodium protein, unknown function
PFIT_1410200.1	30,189109	18,304953	103,6407286	146,1424299	24,24703089	124,8915792	5,150798868	2,364796206	2,542E-07	1,543E-06	conserved Plasmodium protein, unknown function
PFIT_1201500	28,176501	38,536744	104,7796377	135,4490813	33,35662248	120,1143595	3,600914918	1,848363512	2,577E-07	1,562E-06	cytochrome c oxidase assembly protein COX19, putative (COX19)
PFIT_1329900	83,5232	106,93946	252,8378214	261,0959265	95,23133198	256,966874	2,698343797	1,432074175	2,612E-07	1,581E-06	elongation factor Tu, putative
PFIT_0935100	168,0527	132,95177	342,8116408	393,8716707	150,5022349	368,3416557	2,447416518	1,29125965	2,738E-07	1,656E-06	conserved Plasmodium protein, unknown function
PFIT_1247800	29,182805	27,939139	99,08509218	118,5179462	28,56097204	108,8015192	3,809447348	1,929581715	2,752E-07	1,663E-06	conserved Plasmodium protein, unknown function
PFIT_1366700	15390,408	13147,774	6645,534631	7248,308076	14269,09053	6946,921354	0,486851025	-1,038447714	2,764E-07	1,669E-06	alanine--tRNA ligase (AlaRS)
PFIT_1352300	28472,355	28736,85	14480,09037	13740,06174	28604,60216	14110,07606	0,493279927	-1,019521515	2,774E-07	1,673E-06	Plasmodium exported protein, unknown function
PFIT_0306200	2203,8049	2128,1917	1015,906922	1041,710369	2165,998296	1028,808646	0,474981281	-1,074057436	2,787E-07	1,679E-06	glutaredoxin 1
PFIT_1414400	168,0527	156,07381	380,3956413	407,2383564	162,063258	393,8169988	2,43002025	1,280968337	2,826E-07	1,7E-06	30S ribosomal protein S9, putative
PFIT_0710800	7454,6972	5953,9269	13729,54927	13527,08589	6704,312048	13628,31758	2,032768982	1,023446267	2,851E-07	1,714E-06	erythrocyte membrane protein 1, PfEMP1
PFIT_1231900	669,19191	556,85595	2010,174571	2777,59728	613,0239259	2393,885926	3,905044851	1,965339119	2,878E-07	1,728E-06	60 kDa chaperonin
PFIT_1305000	123,77534	98,268696	333,7003679	262,8781513	111,0220207	298,2892596	2,686757616	1,425866176	2,929E-07	1,757E-06	conserved Plasmodium protein, unknown function
PFIT_1450900	3977,9182	4256,3833	2165,06621	1865,098206	4117,15077	2015,082208	0,489436098	-1,030807585	2,938E-07	1,76E-06	exosome complex exonuclease RRP6, putative
PFIT_1410500	193,21029	251,45225	485,175279	548,034112	222,3312735	516,6046955	2,323580877	1,216349862	2,969E-07	1,777E-06	conserved Plasmodium protein, unknown function
PFIT_0822600	83,5232	48,17093	200,4480026	261,0959265	65,84706492	230,7719645	3,504665923	1,809276928	2,996E-07	1,791E-06	conserved Plasmodium protein, unknown function
PFIT_1307400	56979,93	54413,882	27377,09708	27542,50135	55696,9059	27459,79922	0,493021987	-1,020276108	3,03E-07	1,81E-06	carbamoyl phosphate synthetase
PFIT_0306500	23,144983	26,012302	93,39054665	105,1512605	24,57864259	99,27090358	4,038909114	2,013965682	3,093E-07	1,845E-06	conserved Plasmodium protein, unknown function
PFIT_0821500	630,95237	579,01457	1660,529476	1310,826307	604,983471	1485,677891	2,455733029	1,296153729	3,11E-07	1,853E-06	conserved Plasmodium protein, unknown function
PFIT_0307400	69,43495	47,207511	175,3920023	233,4714428	58,32123031	204,4317225	3,50527109	1,809526024	3,14E-07	1,869E-06	conserved Plasmodium protein, unknown function
PFIT_0501300	2589,2192	2852,6825	1491,970928	1170,030551	2720,950829	1331,00074	0,489167509	-1,031599513	3,251E-07	1,934E-06	parasite-infected erythrocyte surface protein (PIESP2)
PFIT_1449600	321,01085	411,37974	868,9876475	767,2477568	366,1952964	818,1177022	2,234102159	1,159695157	3,27E-07	1,942E-06	ubiquitin-protein ligase, putative
PFIT_0829900	11542,302	12185,318	6334,612445	5453,607749	11863,81042	5894,110097	0,496814252	-1,009221535	3,279E-07	1,946E-06	ATP-dependent RNA helicase DBP10, putative
PFIT_0316300.2	201,26072	161,85432	618,4276443	859,923444	181,5575235	739,1755442	4,071302197	2,025490311	3,306E-07	1,96E-06	inorganic pyrophosphatase, putative
PFIT_0625000	562,52372	611,77081	272,1992762	244,1647914	587,147264	258,1820338	0,439722791	-1,185333784	3,32E-07	1,966E-06	oxidoreductase, short-chain dehydrogenase family, putative
PFIT_1310900	1003,2847	1088,663	526,1760068	449,1206381	1045,973858	487,6483224	0,466214637	-1,100933796	3,398E-07	2,01E-06	cytochrome c2 precursor, putative
PFIT_0802100	1030,4549	831,43024	403,1738234	442,8828515	930,9425746	423,0283374	0,454408627	-1,137937869	3,636E-07	2,149E-06	Plasmodium exported protein, unknown function
PFIT_0627700	46,289966	36,609906	137,8080018	138,1224185	41,44993645	137,9652101	3,328478206	1,734862722	3,64E-07	2,149E-06	glutamyl-tRNA(Gln) amidotransferase subunit B, putative
PFIT_1231600	37930,602	40966,485	22253,14501	17475,60483	39448,54381	19864,37492	0,503551538	-0,98978865	3,681E-07	2,17E-06	asparagine-rich protein, putative
PFIT_1427500	34,214323	31,792814	112,7520014	123,8646204	33,00356826	118,3083109	3,584712719	1,841857505	3,683E-07	2,17E-06	ATP-binding cassette sub-family G member 2 (ABCG2)
PFIT_0102700	5572,9094	6081,0982	24327,09849	17880,16985	5827,003793	21103,63417	3,62169563	1,856665306	3,695E-07	2,175E-06	conserved Plasmodium protein, unknown function
PFIT_1462600	1384,6738	1474,034	9831,063399	6727,007336	1429,352112	8279,035367	5,792159468	2,534101323	3,712E-07	2,194E-06	conserved Plasmodium protein, unknown function
PFIT_0714400	904,66695	800,60085	2061,425481	2699,179391	852,633901	2380,302436	2,79170513	1,481146567	3,76E-07	2,208E-06	conserved Plasmodium protein, unknown function
PFIT_1368700	1180,3941	1290,9809	594,5105531	566,7474719	1235,687528	580,6290125	0,469883364	-1,089625405	3,852E-07	2,26E-06	U1 small nuclear ribonucleoprotein a, putative
PFIT_1206800	219,37419	155,11039	525,0370977	695,0676542	187,242291	610,0523759	3,258090749	1,704026788	3,963E-07	2,323E-06	conserved Plasmodium protein, unknown function
PFIT_1357000	985,17124	1072,2849	567,1767346	406,347244	1028,728067	486,7619893	0,473168765	-1,079573253	4,022E-07	2,355E-06	pyrroline carboxylate reductase
PFIT_1105400	47,29627	39,500162	162,8640021	124,7557328	43,39821615	143,8098675	3,313727619	1,728455021	4,065E-07	2,378E-06	radial spoke head protein, putative
PFIT_1342100	6636,5724	6221,7573	3134,277858	3185,726749	6429,164812	3160,002303	0,491510545	-1,024705725	4,113E-07	2,403E-06	transcription factor with AP2 domain(s)
PFIT_0902000	16,100858	9,6341859	61,5010917	74,85343969	12,8675219	68,17726569	5,298399041	2,405556502	4,137E-07	2,414E-06	Plasmodium exported protein, unknown function
PFIT_0501400	60755,581	51245,198	26865,72689	28591,34062	56000,38962	27728,53376	0,495148944	-1,014065534	4,255E-07	2,481E-06	skeleton-binding protein 1
PFIT_0813800.2	8,0504289	5,7805116	42,13963691	59,70452927	6,915470246	50,92208309	7,363502593	2,880392176	4,276E-07	2,49E-06	conserved Plasmodium protein, unknown function
PFIT_1204900	17,107161	27,939139	108,196365	81,9823387	22,52315033	95,08935187	4,22184954	2,077875164	4,295E-07	2,499E-06	conserved Plasmodium protein, unknown function
PFIT_1104600	1101,9025	964,38201	2102,426209	2168,076414	1033,142236	2135,251311	2,066754448	1,047366992	4,304E-07	2,502E-06	CCR4-NOT transcription complex subunit 1, putative
PFIT_0922400	28,176501	22,158628	108,196365	92,67568723	25,16756445	100,4360261	3,990693113	1,996639339	4,335E-07	2,517E-06	conserved Plasmodium protein, unknown function
PFIT_1244000	911,71108	842,02785	1865,533115	1796,482552	876,8694634	1831,007834	2,088119053	1,062203969	4,355E-07	2,526E-06	microtubule associated katanin, putative
PFIT_1238200	218,36788	171,48851	456,7025513	586,3519442	194,9281972	521,5272478	2,675483872	1,419799832	4,375E-07	2,535E-06	conserved Plasmodium protein, unknown function
PFIT_1343800	55,346699	53,951441	146,9192746	181,786925	54,64907006	164,3530998	3,007427201	1,588529815	4,483E-07	2,595E-06	lipoyl synthase (LipA)
PFIT_0210600	32,201716	26,012302	135,5301836	98,91347387	29,10700887	117,2218287	4,027271549	2,009802753	4,589E-07	2,654E-06	Rab GTPase 5a
PFIT_1356100	63602,414	62740,709	33013,55824	30396,7343	63171,5614	31705,14627	0,501889546	-0,994558199	4,726E-07	2,73E-06	elongation factor 1-alpha
PFIT_0612500	328,05498	356,46488	790,4029192	724,4743627	342,2599292	757,438641	2,21305089	1,146036627	4,845E-07	2,796E-06	adapter-related protein, putative
PFIT_0601000	4579,6878	4516,5064	2281,234938	2207,285358	4548,097061	2244,260148	0,493450364	-1,019023124	4,966E-07	2,863E-06	ATP-dependent RNA helicase, putative
PFIT_1452300	76007,119	71045,377	38529,29504	35391,41917	73526,24788	36960,35711	0,502682486	-0,992280669	5,062E-07	2,915E-06	elongation factor 2
PFIT_0313600.1	42,264752	26,975721	105,9185468	132,7757442	34,62023625	119,3471455	3,447323254	1,785476586	5,126E-07	2,949E-06	conserved Plasmodium protein, unknown function
PFIT_1111200	25302,498	28219,494	14286,47582	12693,047	26760,99607	13489,74026	0,504082144	-0,988269245	5,174E-07	2,974E-06	asparagine-rich antigen
PFIT_1204500	340,13062	334,30625	1580,805838	1137,059393	337,2184371	1358,932616	4,029828937	2,010718599	5,234E-07	3,005E-06	probable protein, unknown function
PFIT_1119100	24243,867	26230,035	13302,45835	12101,30608	25236,95067	12701,88222	0,503304951	-0,990495305	5,252E-07	3,013E-06	insulinase, putative
PFIT_0212400	33,208019	50,097767	117,3076379	153,2713289	41,65289308	135,2894834	3,248021287	1,699561088	5,356E-07	3,069E-06	conserved Plasmodium protein, unknown function
PFIT_1103900	37391,224	39374,918	21963,8621	17102,22874	38383,07068	19533,04542	0,508897414	-0,974553234	5,41E-07	3,097E-06	60S acidic ribosomal protein P1, putative
PFIT_0306700	12865,592	12775,894	6701,341177	6170,953212	12820,74285	6436,147195	0,502010474	-0,99421063	5,645E-07	3,226E-06	T-complex protein beta subunit, putative
PFIT_1226600	378,37016	509,64844	1767,586932	1317,955206	444,0092977	1542,771069	3,474636853	1,796862204	5,647E-07	3,226E-06	conserved Plasmodium protein, unknown function
PFIT_0731800	9,0567326	7,7073487	52,38981886	57,92230452	8,382040647	55,15606169	6,58026655	2,718146025	5,714E-07	3,261E-06	probable protein, unknown function
PFIT_1356800	10169,704	10455,982	5449,68097	4917,158097	10312,84317	5183,419084	0,502617852	-0,99246618	5,852E-07	3,336E-06	U3 snoRNA-associated small subunit rRNA processing protein, putative
PFIT_0719500	7086,3901	7874,0202	4275,464782	3303,353582	7480,205113	3789,409182	0,506591614	-0,981104899	5,901E-07	3,361E-06	DEAD/DEAH box ATP-dependent RNA helicase, putative
PFIT_1119000	2656,6416	2594,4863	1334,801472	1241,992879	2625,563909	1289,397175	0,491093426	-1,025930585	5,912E-07	3,364E-06	heat shock protein 90, putative
PFIT_0535900	684,28646	819,86922	384,9512777	312,7804444	752,0778408	348,865861	0,463869352	-1,108209563	6,016E-07	3,42E-06	Plasmodium exported protein (PHISTc), unknown function
PFIT_0802800	1729,8359	2084,8378	979,4618308	884,8745906	1907,336876	932,1682107	0,488727619	-1,032897457	6,206E-07	3,524E-06	Plasmodium exported protein (hyp13), unknown function
PFIT_0319600	8456,9756	9493,5268	5248,093158	3897,725538	8975,251203	4572,909348	0,509502101	-0,972839998	6,246E-07	3,543E-06	elongation factor 1 (EF-1), putative
PFIT_0216500	260,63264	253,37909	521,6203703	662,9876086	257,0058633	592,3039895	2,304632205	1,20453653	6,312E-07	3,577E-06	ATP synthase F1, alpha subunit

PF3D7_1336700	13893,028	15067,867	7065,792091	7426,530552	14480,44726	7246,161321	0,500410049	-0,998817334	6,361E-07	3,601E-06	conserved Plasmodium protein, unknown function
PF3D7_1362700	92,579933	86,707673	215,2538209	255,7492523	89,64380304	235,5015366	2,627081054	1,393460713	6,38E-07	3,608E-06	conserved Plasmodium protein, unknown function
PF3D7_1126700	2615,3831	2865,2069	13622,49181	9691,738215	2740,294997	11657,11501	4,253963542	2,088807669	6,392E-07	3,611E-06	kelch protein, putative
PF3D7_0702400	56272,498	66492,261	34623,97572	28103,90215	61382,37963	31363,93894	0,510959971	-0,96871782	6,412E-07	3,619E-06	erythrocyte membrane-associated antigen
PF3D7_0809600	5153,2808	5635,9988	2799,438581	2602,939254	5394,639794	2701,188918	0,500717197	-0,997932089	6,737E-07	3,799E-06	conserved Plasmodium membrane protein, unknown function
PF3D7_1351300	1000,2658	1062,6507	2542,045124	2032,627332	1031,458251	2287,336228	2,21757519	1,148983022	6,806E-07	3,834E-06	ABC transporter, (heavy metal transporter family), putative
PF3D7_0212000	1387,6927	1456,6889	6536,199357	4697,944453	1422,1908	5617,071905	3,949590945	1,981703243	6,956E-07	3,914E-06	secreted protein altered thrombospondin repeat protein
PF3D7_1436900.1	45,283663	79,963743	181,0865478	181,786925	62,62370296	181,4367364	2,897253401	1,534685872	6,968E-07	3,918E-06	ataxin-2 like protein, putative
PF3D7_0624500	8201,3745	8039,7282	4147,906962	4001,985686	8120,551316	4074,946324	0,50180661	-0,994796621	7,002E-07	3,932E-06	poly(A) polymerase PAP, putative
PF3D7_0709600	2953,5011	3462,5264	1654,83493	1538,059963	3208,013768	1596,447447	0,497643577	-1,006815272	7,304E-07	4,098E-06	conserved Plasmodium protein, unknown function
PF3D7_1113400	86,542111	82,853999	212,9760027	237,0358923	84,69805501	225,0059475	2,656565697	1,409562391	7,431E-07	4,165E-06	DNA helicase, putative
Pf_M76611101600	11,06934	12,524442	58,08436438	69,50676542	11,79689074	63,7955649	5,407828748	2,435049466	7,544E-07	4,224E-06	unspecified product
PF3D7_0604100	29,182805	31,792814	103,6407286	114,9534967	30,48780922	109,2971126	3,584944784	1,841950898	7,79E-07	4,358E-06	conserved Plasmodium protein, unknown function
PF3D7_1134100	5949,267	6174,5498	35111,42881	24344,29903	6061,908371	29727,86392	4,90404376	2,293971853	7,982E-07	4,461E-06	apical membrane antigen 1
PF3D7_0103900.2	22,13868	10,597605	115,0298197	179,1135878	16,36814205	147,0717037	8,985241166	3,167557227	8,057E-07	4,498E-06	conserved Plasmodium protein, unknown function
PF3D7_1213300	1606,0606	1829,5319	922,5163755	769,9210939	1717,79624	846,2187347	0,492618807	-1,021456387	8,101E-07	4,518E-06	RNA polymerase subunit 8c, putative
PF3D7_1148100	669,19191	656,08806	300,6720039	302,9782083	662,6399834	301,8251061	0,455488823	-1,134512442	8,199E-07	4,568E-06	farnesyltransferase beta subunit, putative
PF3D7_0933800	281,76501	237,96439	539,842916	605,9564165	259,8647026	572,8996663	2,204607477	1,140521812	8,318E-07	4,63E-06	conserved Plasmodium protein, unknown function
PF3D7_1477300	1727,8233	1943,2153	1031,85165	792,1989033	1835,519306	912,0252765	0,496875883	-1,009042575	8,361E-07	4,65E-06	Plasmodium exported protein (PHISTb), unknown function
PF3D7_1442300	4278,803	4976,057	2357,541848	2287,485472	4627,430005	2322,51366	0,501901414	-0,994524086	8,505E-07	4,725E-06	conserved Plasmodium protein, unknown function
PF3D7_0303000	941,90019	644,52704	1628,640021	1926,58496	793,213612	1777,61249	2,241026204	1,164159518	8,729E-07	4,845E-06	conserved Plasmodium protein, unknown function
PF3D7_1306800	1042,5305	1241,8466	3089,860403	2443,430138	1142,188556	2766,645271	2,422231649	1,276336843	8,912E-07	4,941E-06	DNA-directed RNA polymerase alpha chain, putative
PF3D7_1470000	2094,1178	2160,9479	1117,269833	991,8080758	2127,532865	1054,538954	0,495662827	-1,012569029	8,987E-07	4,978E-06	conserved Plasmodium protein, unknown function
PF3D7_1338900	542,39765	502,90451	1558,027656	2156,491953	522,6510774	1857,259805	3,553536738	1,829255614	9,035E-07	5E-06	conserved Plasmodium protein, unknown function
PF3D7_0819500	247,55069	226,40337	499,980973	554,2718986	236,9770295	527,126498	2,22437803	1,153401993	9,106E-07	5,034E-06	ClpB protein, putative
PF3D7_1446300	8325,1498	8334,5342	4642,193514	3870,101054	8329,842033	4256,147284	0,51095174	-0,96874106	9,548E-07	5,273E-06	histidine--tRNA ligase, putative
PF3D7_1356200	110731,64	98781,235	55373,76071	51737,98462	104756,4363	53555,87267	0,511241835	-0,967922199	9,838E-07	5,428E-06	elongation factor 1-alpha
PF3D7_1431700	32,201716	36,609906	128,6967289	108,71571	34,40581113	118,7062195	3,450179362	1,786671364	9,883E-07	5,448E-06	conserved Plasmodium protein, unknown function
PF3D7_1467900	5058,6883	5766,0603	3128,583313	2417,587879	5412,374279	2773,085596	0,512360279	-0,96476946	1,001E-06	5,509E-06	conserved Plasmodium protein, unknown function
PF3D7_1123600	54,340395	37,573325	141,2247291	192,4802735	45,95686022	166,8525013	3,630633174	1,860221173	1,001E-06	5,509E-06	conserved Plasmodium protein, unknown function
PF3D7_1135000	472,9627	507,7216	1009,073468	1044,383706	490,3421491	1026,728587	2,093902367	1,066194175	1,004E-06	5,516E-06	conserved Plasmodium protein, unknown function
PF3D7_0308700	123,77534	99,232115	297,2552765	262,8781513	111,50373	280,0667139	2,51172507	1,328678557	1,006E-06	5,526E-06	conserved protein, unknown function
PF3D7_0909800	316,98564	321,78181	677,6509178	703,0876656	319,3837246	690,3692917	2,161566913	1,112077497	1,01E-06	5,538E-06	conserved Plasmodium protein, unknown function
PF3D7_0726000	115,72492	130,06151	293,8385492	387,6338841	122,893213	340,7362167	2,772620297	1,471250057	1,013E-06	5,549E-06	conserved Plasmodium protein, unknown function
PF3D7_1462100.2	17,107161	24,085465	76,30691007	93,56679961	20,59631315	84,93685484	4,123886359	2,044004577	1,016E-06	5,563E-06	apicoplast ribosomal protein S10 precursor, putative
PF3D7_0522700	24,151287	18,304953	83,1403647	90,0023501	21,22812003	86,5713574	4,078145275	2,027913169	1,023E-06	5,593E-06	50S ribosomal protein L17, apicoplast, putative (RPL17)
PF3D7_0412200	170,06531	199,42765	446,4523694	589,0252813	184,7464799	517,7388254	2,802428634	1,486677634	1,026E-06	5,607E-06	lysine decarboxylase-like protein, putative
PF3D7_0605400	4001,0632	4433,6524	2290,346211	1987,180601	4217,357772	2138,763406	0,5071335	-0,979562517	1,034E-06	5,643E-06	polypyrimidine tract binding protein, putative
PF3D7_1105700	1312,2199	1946,1056	8017,920103	5746,783721	1629,162737	6882,351912	4,224471723	2,078770941	1,04E-06	5,67E-06	calcium/calmodulin-dependent protein kinase, putative
PF3D7_1021200	12439,925	14004,253	28470,44982	23986,96297	13222,08899	26228,7064	1,983703666	0,988196526	1,041E-06	5,671E-06	conserved Plasmodium membrane protein, unknown function
PF3D7_1322600	19275,746	18438,868	10162,48595	9149,941889	18857,30711	9656,213919	0,51206749	-0,965594127	1,047E-06	5,699E-06	60S ribosomal protein L23a, putative
PF3D7_1222700	143,90142	168,59825	1184,46547	789,5255662	156,2498354	986,995518	6,316777968	2,659188863	1,073E-06	5,834E-06	conserved Plasmodium protein, unknown function
PF3D7_1353500	11828,093	10749,825	5504,347707	5907,183949	11288,95868	5705,765828	0,505428887	-0,984419972	1,078E-06	5,854E-06	large subunit rRNA methyltransferase, putative
PF3D7_1477500	3483,8231	3504,9168	1772,142568	1734,995798	3494,369981	1753,569183	0,501826994	-0,994738017	1,088E-06	5,906E-06	serine/threonine protein kinase, FIKK family, pseudogene (FIKK14)
PF3D7_0824500	5406,8693	5448,1321	3072,776767	2480,856858	5427,500737	2776,816812	0,511619795	-0,966856012	1,095E-06	5,94E-06	clp1-related protein, putative
PF3D7_1442400	8402,6352	8789,2678	4825,55788	4008,223473	8595,95151	4416,890676	0,51383829	-0,96062622	1,127E-06	6,105E-06	60S ribosomal protein L1, putative
PF3D7_1418800	558,49851	506,75818	1585,361475	2200,156459	532,6283435	1892,758967	3,553620437	1,829289595	1,132E-06	6,124E-06	DNA replication licensing factor MCM2 (MCM2)
PF3D7_0405400	40,252145	32,756232	110,4741832	130,1024071	36,50418841	120,2882952	3,295191604	1,720362355	1,154E-06	6,241E-06	conserved Plasmodium protein, unknown function
PF3D7_0917700	8373,4524	9474,2584	5072,701156	4129,414756	8923,855417	4601,057956	0,515590823	-0,95570151	1,168E-06	6,312E-06	pre-mRNA-splicing factor ATP-dependent RNA helicase PRP43, putative
[illegible]	[illegible]	[illegible]	[illegible]	[illegible]	[illegible]	[illegible]	[illegible]	[illegible]	[illegible]	[illegible]	[illegible]

PFIT_0515100	2822,6816	2317,0217	1301,773108	1538,951075	2569,85168	1420,362092	0,552701972	-0,855426335	4,634E-05	0,0001748	RNA binding protein, putative
PFIT_1323200	4372,3892	4342,1276	2379,181121	2523,630252	4357,258406	2451,405687	0,562602779	-0,829811417	4,643E-05	0,000175	conserved Plasmodium protein, unknown function
PFIT_1122500	7771,6828	7191,9198	4297,104055	4204,268196	7481,801313	4250,686125	0,568136729	-0,815689921	4,669E-05	0,0001759	protein GCN20
PFIT_0532900	3149,7303	3700,4908	2201,511301	1719,846888	3425,110567	1960,679095	0,57244257	-0,804797133	4,678E-05	0,000176	peptidyl-prolyl cis-trans isomerase
PFIT_0612200	9,0567326	13,48786	51,25090975	49,90229312	11,27229642	50,57660144	4,486805487	2,165688641	4,678E-05	0,000176	conserved Plasmodium protein, unknown function
PFIT_0814100	4624,9714	4982,801	2594,434942	2817,697337	4803,886192	2706,06614	0,563307712	-0,828004872	4,726E-05	0,0001777	conserved protein, unknown function
PFIT_0527600	2238,0192	2172,5089	4193,463327	3676,729668	2205,264085	3935,096497	1,784410549	0,835447582	4,746E-05	0,0001783	DnaJ protein, putative
PFIT_0420600	35156,223	43044,579	23633,50285	19186,54059	39100,40123	21410,02172	0,547565269	-0,868897152	4,751E-05	0,0001784	40S ribosomal protein S19, putative
PFIT_0709800	582,64979	520,24604	282,4494582	287,8292978	551,4479171	285,139378	0,517073996	-0,951557343	4,786E-05	0,0001796	mitochondrial ribosomal protein L1 precursor, putative
PFIT_0213700	1356,4973	1713,9217	738,0131004	839,4278593	1535,209476	788,7204799	0,513754307	-0,960849512	4,794E-05	0,0001797	conserved Plasmodium protein, unknown function
PFIT_1023300	387,42689	411,37974	796,0974648	714,6721265	399,4033158	755,3847957	1,891283236	0,919365436	4,8E-05	0,0001799	conserved Plasmodium protein, unknown function
PFIT_1475000	5671,5272	6118,6715	3637,675683	3121,566657	5895,099333	3379,62117	0,573293337	-0,802654582	4,863E-05	0,0001821	conserved Plasmodium protein, unknown function
PFIT_0922700	14752,411	14697,914	9062,299753	7901,493449	14725,16254	8481,896601	0,576013785	-0,795824758	4,988E-05	0,0001866	glutamine synthetase, putative
PFIT_1401500	53,334092	66,475883	12,52800016	17,82224754	59,90498729	15,17512385	0,253319874	-1,980967826	5,032E-05	0,0001882	rifin
PFIT_0323100	3110,4845	2936,4999	1931,589843	1521,128828	3023,492175	1726,359335	0,57098191	-0,808483058	5,04E-05	0,0001883	translation machinery-associated protein 7, putative (TMA7)
PFIT_1027800	2242,0445	2202,3749	1365,552018	1146,861629	2222,209681	1256,206824	0,565296261	-0,82292094	5,062E-05	0,000189	nucleolar preribosomal assembly protein, putative
PFIT_1021300	1115,9907	759,17385	3564,7855	5614,007976	937,5822811	4589,396738	4,89492691	2,291287318	5,071E-05	0,0001892	conserved Plasmodium protein, unknown function
PFIT_1017000	3892,3824	3512,6242	10787,74705	7942,484618	3702,503289	9365,115833	2,529401084	1,338795821	5,08E-05	0,0001895	conserved Plasmodium protein, unknown function
PFIT_0908500.2	21,132376	23,122046	67,19563723	79,30900157	22,12721109	73,2523194	3,310508455	1,727052815	5,23E-05	0,0001949	conserved Plasmodium membrane protein, unknown function
PFIT_0813500	15532,296	15498,515	9302,609574	8553,787709	15515,40561	8928,198641	0,575440879	-0,797260382	5,31E-05	0,0001978	RNA helicase, putative
PFIT_0514700	825,16897	795,78376	424,8130964	442,8828515	810,4763617	433,8479739	0,535299972	-0,901580517	5,335E-05	0,0001986	ribose 5-phosphate epimerase, putative
PFIT_1417600	235,47505	208,09842	484,0363699	397,4361202	221,7867312	440,736245	1,987207452	0,990742489	5,354E-05	0,0001992	conserved Plasmodium protein, unknown function
PFIT_1108900	1641,2812	2024,1425	10318,5165	6684,233941	1832,711831	8501,375219	4,638686276	2,213716277	5,372E-05	0,0001997	conserved Plasmodium protein, unknown function
PFIT_1228200	520,25897	508,68502	1107,019651	872,3990173	514,4719934	989,7093339	1,92373802	0,943912342	5,438E-05	0,000202	RNA pseudouridylate synthase, putative
PFIT_1412400	345,16214	334,30625	601,3440077	674,5720695	339,7341961	637,9580386	1,877815204	0,909055094	5,458E-05	0,0002026	plastid replication-repair enzyme
PFIT_1446400	6774,436	6328,6967	4069,322234	3469,991597	6551,566342	3769,656915	0,575382545	-0,797406639	5,465E-05	0,0002027	RNA helicase, putative
PFIT_0925900	2015,6261	2256,3263	1339,357108	1088,048213	2135,976244	1213,70266	0,568219176	-0,815480576	5,471E-05	0,0002028	conserved Plasmodium protein, unknown function
PFIT_1451700	157,98967	108,8663	507,9534611	801,1100271	133,4279844	654,5317441	4,905505745	2,294401882	5,484E-05	0,0002032	conserved Plasmodium protein, unknown function
PFIT_1466200	216,35528	239,89123	417,9796417	470,5073352	228,1232536	444,2434885	1,947383625	0,961537116	5,519E-05	0,0002044	conserved Plasmodium protein, unknown function
PFIT_1410000	1388,699	1394,0667	814,3200105	734,2765988	1391,382847	774,2983046	0,556495508	-0,845558052	5,556E-05	0,0002056	conserved Plasmodium protein, unknown function
PFIT_1471600	160,00228	109,82972	264,2269125	333,2760291	134,9159973	298,7514708	2,21435172	1,146884393	5,582E-05	0,0002064	phosphodiesterase delta, putative
PFIT_0319900	1010,3288	1091,5533	600,2050986	554,2718986	1050,941048	577,2384986	0,549258685	-0,864442317	5,59E-05	0,0002066	conserved Plasmodium protein, unknown function
PFIT_0307000	11107,579	8825,8777	4520,33024	5473,212221	9966,728525	4996,77123	0,501345172	-0,996123865	5,615E-05	0,0002072	40S ribosomal protein S12, putative
PFIT_0709700	996,24058	1154,1755	619,5665534	564,9652472	1075,208027	592,2659003	0,550838429	-0,860298884	5,613E-05	0,0002072	conserved Plasmodium protein, unknown function
PFIT_0832000	3094,3836	3037,6588	1734,558568	1731,431349	3066,021222	1732,994958	0,565226015	-0,823100226	5,63E-05	0,0002076	conserved protein, unknown function
PFIT_1427900	174,09053	189,79346	527,3149159	388,5249965	181,9419942	457,9199562	2,516845867	1,331616868	5,637E-05	0,0002078	ubiquinol-cytochrome c reductase hinge protein, putative
PFIT_0714100	3947,7291	3309,3429	1580,805838	2161,838627	3628,535977	1871,322233	0,515723764	-0,955329569	5,678E-05	0,0002091	RNA binding protein, putative
PFIT_1464900	10,063036	13,48786	52,38981886	49,90229312	11,77544823	51,14605599	4,343448758	2,118841017	5,725E-05	0,0002107	mitochondrial ribosomal protein S6-2 precursor, putative
PFIT_1450600	231,44983	286,13532	492,0087336	507,934055	258,7925769	499,9713943	1,931938699	0,950049318	5,767E-05	0,0002121	DNA replication related protein, putative
PFIT_1402300	136813,01	164766,74	92758,4521	69545,97437	150789,8762	81152,21323	0,53818078	-0,893837225	5,79E-05	0,0002128	early transcribed membrane protein 14.1
PFIT_0500200	86,542111	98,268696	29,61163674	32,97115796	92,40540374	31,29139735	0,338631683	-1,562211136	5,848E-05	0,0002148	rifin (RIF)
PFIT_0931400	1498,3861	1766,9097	898,5992843	922,3013104	1632,647892	910,4502973	0,557652573	-0,842561517	5,959E-05	0,0002188	elongation factor Tu, putative
PFIT_1249600	67,422342	45,280674	128,6967289	173,7669136	56,3515081	151,2318212	2,683722696	1,424235608	6,004E-05	0,0002203	conserved Plasmodium protein, unknown function
PFIT_0627800	459,88075	592,50243	988,5731036	949,9257941	526,1915936	969,2494489	1,842008615	0,881279809	6,025E-05	0,0002209	RAP protein, putative
PFIT_1214600	19,119769	25,048883	62,64000081	81,9823387	22,08432606	72,31116975	3,274320871	1,711195708	6,046E-05	0,0002215	thioredoxin peroxidase 2
PFIT_1127100	27410,704	27249,331	16991,38495	14763,05875	27330,01785	15877,22185	0,580944438	-0,783527906	6,093E-05	0,0002231	40S ribosomal protein S18, putative
PFIT_1149300.1	4422,7044	5010,7401	2961,163674	2487,094645	4716,722248	2724,12916	0,57754708	-0,79198954	6,162E-05	0,0002254	Plasmodium exported protein, unknown function
PFIT_0821800	146579,19	150368,45	94273,20121	79030,0834	148473,8191	86651,6423	0,583615636	-0,77690956	6,164E-05	0,0002254	heat shock protein 70
PFIT_0921000	2068,9602	2194,6676	1282,411653	1136,168281	2131,813895	1209,289967	0,567258694	-0,817921278	6,193E-05	0,0002263	U4/U6 snRNA-associated-splicing factor, putative
PFIT_0926200	29772,499	28810,07	16721,46349	17006,87972	29291,2842	16864,1716	0,575740261	-0,796509993	6,243E-05	0,000228	protein kinase, putative
PFIT_1331000	22840,073	19830,045	12873,08962	11823,27902	21335,05904	12348,18432	0,578774321	-0,78892718	6,416E-05	0,0002342	glutamine--tRNA ligase, putative
PFIT_1246800	1729,8359	1668,641	915,6829209	978,4413902	1699,23846	947,0621555	0,557345057	-0,843357307	6,457E-05	0,0002355	pre-mRNA-splicing factor, putative
PFIT_0321700	288,80914	237,00097	471,5083697	529,3207521	262,9050559	500,4145609	1,903404098	0,928581882	6,495E-05	0,0002367	ATP-dependent RNA helicase, putative
PFIT_0401100	107293,1	126964,12	72487,00893	64249,2024	117128,6086	68368,10566	0,583701168	-0,776698139	6,574E-05	0,0002395	Plasmodium exported protein, unknown function
PFIT_0511000	447,80511	391,14795	190,1978206	231,6892181	419,4765291	210,9435194	0,502873235	-0,991733324	6,586E-05	0,0002397	small nucleolar RNA snoR04
PFIT_0722200	3835,0231	4324,7861	2245,928756	2399,765632	4079,904573	2322,847194	0,569338609	-0,812641157	6,601E-05	0,0002401	Rab GTPase activator and protein kinase, putative
PFIT_0618100	6568,1437	5927,9146	3471,394954	3665,145207	6248,029154	3568,270081	0,571103302	-0,808176369	6,621E-05	0,0002407	conserved Plasmodium protein, unknown function
PFIT_1361900	142,89511	110,79314	242,5876395	283,373736	126,8441259	262,9806877	2,073258702	1,051900147	6,646E-05	0,0002415	conserved Plasmodium protein, unknown function
PFIT_0727200	14,088251	18,304953	56,94545528	63,26897878	16,19660195	60,10721703	3,711100466	1,891847058	6,675E-05	0,0002424	conserved Plasmodium protein, unknown function
PFIT_1308500	7315,8273	6552,2098	3741,316412	4164,168139	6934,018572	3952,742275	0,570050719	-0,810837808	6,735E-05	0,0002442	U4/U6 small nuclear ribonucleoprotein PRP3, putative
PFIT_0614600	7089,409	7405,7987	4417,82842	3970,796753	7247,603851	4194,312587	0,578717142	-0,789069717	6,734E-05	0,0002442	conserved Plasmodium protein, unknown function
PFIT_0421900	2444,3115	2442,2661	4622,832059	3993,965675	2443,288809	4308,398867	1,763360456	0,818327412	6,756E-05	0,0002449	early transcribed membrane protein 4
PFIT_1361600	23,144983	21,195209	62,64000081	81,09122633	22,17009611	71,86561357	3,241556248	1,696686607	6,8E-05	0,0002463	conserved Plasmodium protein, unknown function
PFIT_1304600	1241,7787	1185,9683	653,7338266	686,1565305	1213,873475	669,9451785	0,551906926	-0,857503104	6,813E-05	0,0002465	AAR2 protein, putative
PFIT_0611900	8088,6685	9163,0742	5659,239345	4434,175189	8625,871353	5046,707267	0,585066373	-0,773327794	6,814E-05	0,0002465	nucleolar GTP-binding protein 1, putative
[illegible]	[illegible]	[illegible]	[illegible]	[illegible]	[illegible]	[illegible]	[illegible]	[illegible]	[illegible]	[illegible]	[illegible]

PFIT_1420100	3338,9154	3068,4882	1982,840753	1947,971657	3203,701809	1965,406205	0,613479756	-0,704912357	0,0005295	0,0014965	ATP-dependent RNA helicase, putative
PFIT_0934700	9,0567326	15,414697	41,0007278	48,12006837	12,23571501	44,56039808	3,641830333	1,864663711	0,0005293	0,0014965	conserved Plasmodium protein, unknown function
PFIT_1418200	25962,633	23538,243	14657,76019	15914,37594	24750,43819	15286,06807	0,617607977	-0,695236708	0,0005343	0,0015093	NOT family protein, putative
PFIT_0530800	1172,3437	1075,1751	1734,558568	1936,387196	1123,759432	1835,472882	1,633332571	0,707818575	0,0005345	0,0015093	conserved Plasmodium protein, unknown function
PFIT_1413800	5891,9077	5238,1069	3359,781861	3488,704957	5565,007282	3424,243409	0,615316968	-0,700598318	0,0005381	0,0015171	N-myristoyltransferase (NMT)
PFIT_1132300	35,220627	27,939139	83,1403647	80,20011395	31,57988289	81,67023933	2,586147631	1,370804634	0,0005379	0,0015171	conserved Plasmodium protein, unknown function
PFIT_0315500	22,13868	26,012302	66,05672812	69,50676542	24,07549078	67,78174677	2,815383802	1,493331608	0,0005379	0,0015171	mitochondrial ribosomal protein L29/L47 precursor, putative
PFIT_1208400	6649,6543	6964,553	4538,552786	3957,430067	6807,103653	4247,991426	0,624052702	-0,680260224	0,0005393	0,0015195	zinc finger protein, putative
PFIT_0108400.1	128,80686	101,15895	191,3367297	247,7292409	114,9829076	219,5329853	1,909266254	0,933018306	0,0005395	0,0015195	phosphatidate cytidylyltransferase, putative
PFIT_1332600	2564,0616	2446,1198	1571,694566	1498,851018	2505,090711	1535,272792	0,612861157	-0,706367824	0,0005399	0,0015198	transmembrane protein Tmp21 homologue, putative
PFIT_0322000	9766,1766	10689,129	6681,979722	6090,753098	10227,65294	6386,36641	0,624421502	-0,679407878	0,0005473	0,0015401	cyclic amine resistance locus protein (CARL)
PFIT_0922900	6595,3139	7109,0658	3985,04296	4447,541875	6852,18985	4216,292417	0,615320432	-0,700590197	0,0005485	0,0015425	conserved Plasmodium protein, unknown function
PFIT_0525900	602,77587	565,52671	334,839277	338,6227033	584,1512902	336,7309902	0,576444828	-0,794745561	0,0005514	0,0015493	DEAD/DEAH box ATP-dependent RNA helicase, putative
PFIT_0518100	1382,6612	1446,0913	891,7658296	824,2789489	1414,376238	858,0223893	0,606643668	-0,721078744	0,0005513	0,0015493	conserved protein, unknown function
PFIT_1439200	9449,191	11777,792	6770,814632	5227,265205	10613,49163	5999,039919	0,565227743	-0,823095816	0,0005537	0,001555	eukaryotic translation initiation factor eIF2A, putative
PFIT_0523900	599,75696	544,3315	350,7840045	312,7804444	572,0442302	331,7822245	0,579994006	-0,785890105	0,0005544	0,0015559	DEAD/DEAH box ATP-dependent RNA helicase, putative
PFIT_1030000	2602,3012	2936,4999	1956,645843	1499,742131	2769,400512	1728,193987	0,6240318	-0,680308545	0,0005546	0,0015559	glycoprotease, putative
PFIT_1121500	121,76274	121,39074	202,7258208	258,4225894	121,5767402	230,5742051	1,896532222	0,923363883	0,0005576	0,0015634	conserved Plasmodium protein, unknown function
PFIT_0108000	647,05323	613,69764	349,6450954	378,7227603	630,3754346	364,1839279	0,577725444	-0,79154406	0,0005628	0,0015774	N-acetyltransferase, putative
PFIT_1411000	2542,9292	2290,046	1479,442928	1475,682097	2416,487617	1477,562512	0,61145048	-0,709692433	0,0005647	0,0015818	tetratricopeptide repeat family protein, putative
PFIT_0408500	253,58851	302,51344	511,3701884	470,5073352	278,0509747	490,9387618	1,765643016	0,820193684	0,0005676	0,0015891	actin-like protein, putative (ALP5b)
PFIT_1141000	46,289966	54,91486	119,5854561	111,3890472	50,60241307	115,4872516	2,282247913	1,190455515	0,0005687	0,0015914	P-loop containing nucleoside triphosphate hydrolase, putative
PFIT_1461600	1185,4257	1604,092	739,1520095	572,0941462	1394,758809	655,6230778	0,470061973	-1,08907712	0,0005696	0,0015931	ubiquitin carboxyl-terminal hydrolase isozyme L3
PFIT_1468000	4237,5445	4371,9936	2625,185488	2684,921593	4304,769052	2655,05354	0,616770263	-0,697194886	0,0005705	0,0015949	rhoptry protein, putative
PFIT_0918400	3313,7578	2844,0117	1912,228388	1872,227105	3078,884748	1892,227746	0,614582195	-0,702322124	0,0005709	0,0015954	eukaryotic translation initiation factor 3 subunit 5, putative
PFIT_0624100	62,390824	64,549046	124,1410925	147,0335422	63,46993497	135,5873174	2,136244782	1,095076968	0,0005731	0,0015998	conserved Plasmodium protein, unknown function
PFIT_1362500	135,85099	80,927162	309,7832767	489,2206951	108,389075	399,5019859	3,685814145	1,881983326	0,0005731	0,0015998	DNase I-like protein, putative
PFIT_1149000	3302,6885	3243,8304	1974,868389	2045,102906	3273,259436	2009,985647	0,614062431	-0,703542754	0,0005743	0,0016024	Plasmodium exported protein (hyp11), unknown function
PFIT_0821900	5735,9306	4980,8741	3026,081493	3534,151688	5358,40237	3280,116591	0,61214451	-0,708055823	0,0005752	0,0016041	conserved Plasmodium protein, unknown function
PFIT_1361800	450,82402	282,28165	739,1520095	1000,7192	366,552834	869,9356046	2,373288442	1,246887452	0,0005773	0,0016092	conserved Plasmodium protein, unknown function
PFIT_0400600	332,08019	210,02525	69,47345544	78,41788919	271,0527234	73,94567232	0,272809184	-1,874035882	0,0005785	0,001611	acyl-CoA synthetase
PFIT_1212100	7541,2393	8085,0088	5373,37316	4446,650762	7813,124067	4910,011961	0,628431332	-0,670172984	0,0005784	0,001611	glycerol-3-phosphate acyltransferase
PFIT_0218600	3210,1085	2733,2185	1629,77893	1975,59614	2971,663543	1802,687535	0,60662572	-0,721121428	0,0005795	0,0016129	40S ribosomal protein S30, putative
PFIT_0715800	6219,9627	6734,296	4595,498241	3569,796183	6477,129309	4082,647212	0,630317386	-0,665849639	0,0005819	0,0016188	serine--tRNA ligase, putative
PFIT_1232900	580,63719	662,83199	379,2567321	348,4249395	621,7345893	363,8408358	0,585202821	-0,77299137	0,0005823	0,001619	conserved protein, unknown function
PFIT_1038700	93,586236	99,232115	34,16727317	44,55561886	96,4091757	39,36144601	0,408274894	-1,292387239	0,0005845	0,0016244	Plasmodium exported protein (hyp6), unknown function
PFIT_1428700	126,79426	110,79314	195,8923662	260,2048141	118,7936969	228,0485901	1,919702779	0,940882961	0,0005861	0,001628	DNA mismatch repair protein Msh2p, putative
PFIT_1315100	8429,8054	9159,2206	6308,417536	4814,680174	8794,512979	5561,548855	0,632388498	-0,661116967	0,0005865	0,0016284	exportin 1-like protein, putative
PFIT_0818300	35,220627	39,500162	100,2240013	84,65567584	37,36039444	92,43983856	2,474273624	1,307005054	0,0005915	0,0016416	conserved Plasmodium protein, unknown function
PFIT_1407900	43,271056	33,719651	135,5301836	230,7981057	38,49535314	183,1641446	4,75808454	2,250380905	0,0005919	0,0016417	radical SAM protein, putative
PFIT_0603400	34632,945	40208,275	25439,81269	21796,60875	37420,61012	23618,21072	0,631155148	-0,663933409	0,0005929	0,0016436	conserved Plasmodium protein, unknown function
PFIT_0910000	1180,3941	935,47945	556,9265526	650,5120354	1057,936798	603,719294	0,570657241	-0,809303629	0,0006001	0,0016628	small nuclear ribonucleoprotein Sm D3, putative
PFIT_1030300	107,67449	94,415022	189,0589115	206,7380715	101,0447545	197,8984915	1,958523156	0,969766185	0,0006024	0,0016684	28 kDa ookinete surface protein
PFIT_1345800	39,245841	30,829395	91,11272844	83,76456346	35,03761801	87,43864595	2,495564793	1,319366362	0,0006109	0,0016911	conserved Plasmodium protein, unknown function
PFIT_0703000	6267,2589	8882,7194	13082,6489	11931,99473	7574,989174	12507,32181	1,651133952	0,723457167	0,0006136	0,0016977	conserved Plasmodium membrane protein, unknown function
PFIT_1307800	144,90772	142,58595	233,4763666	311,889332	143,7468363	272,6828493	1,896965919	0,923693759	0,000614	0,001698	conserved Plasmodium protein, unknown function
PFIT_1422500	172,07792	209,06183	77,44581918	110,4979348	190,5698765	93,97187698	0,493109817	-1,02001912	0,0006157	0,0017013	mitochondrial import inner membrane translocase subunit TIM8, putative (TIM8)
PFIT_0727800	2446,3241	2942,2804	1807,448751	1546,971087	2694,302237	1677,209919	0,622502515	-0,683848429	0,0006158	0,0017013	RNA binding protein, putative
PFIT_0726900	9037,6128	7596,5556	4851,75279	5418,854366	8317,084193	5135,303578	0,617440374	-0,695628271	0,0006258	0,001728	eukaryotic translation initiation factor 2 alpha subunit, putative
PFIT_1248100	2425,1917	2613,7546	1652,557112	1472,117647	2519,473179	1562,33738	0,620104787	-0,689416068	0,0006311	0,0017418	nucleolar rRNA processing protein, putative
PFIT_1451500	3555,2707	3491,429	2551,156396	1887,376015	3523,349829	2219,266206	0,629873931	-0,666864993	0,0006336	0,0017479	DNA polymerase delta interacting protein, putative
PFIT_1015600	123,77534	167,63484	275,6160035	270,0070503	145,70509	272,8115269	1,87235413	0,904853326	0,0006352	0,0017515	conserved Plasmodium protein, unknown function
PFIT_0513600	162,01488	144,51279	69,47345544	76,63566444	153,2638356	73,05455994	0,476658826	-1,068971085	0,000639	0,0017602	mitochondrial import inner membrane translocase subunit TIM16, putative (PAM16)
PFIT_0213100	644,03432	619,47815	363,3120047	373,3760861	631,7562349	368,3440454	0,583047741	-0,778314077	0,0006387	0,0017602	conserved protein, unknown function
PFIT_1432700	293,84066	280,35481	431,646551	552,4896739	287,0977333	492,0681124	1,713939385	0,777316088	0,000647	0,0017814	mitogen-activated protein kinase 1
PFIT_0221300	193,21029	248,562	127,5578198	105,1512605	220,8861456	116,3545402	0,526762508	-0,924775427	0,0006492	0,0017857	erythrocyte membrane protein 1 (PfEMP1), pseudogene
PFIT_1315700	1595,9975	1712,9583	1077,408014	957,9458055	1654,477897	1017,67691	0,615104567	-0,701096408	0,000649	0,0017857	conserved Plasmodium protein, unknown function
PFIT_1426700	702,39992	667,64908	390,6458232	411,6939183	685,0245046	401,1698707	0,585628497	-0,771942338	0,0006508	0,0017893	conserved Plasmodium protein, unknown function
PFIT_1245000	5249,886	5594,5718	3428,116408	3332,760291	5422,228868	3380,438349	0,623440735	-0,681675673	0,0006517	0,0017909	cytoplasmic translation machinery associated protein, putative
PFIT_1444200	968,06408	727,38104	1288,106198	1530,931064	847,7225585	1409,518631	1,662712189	0,733538464	0,0006547	0,0017983	serine/threonine protein kinase
PFIT_0320000	118,74383	98,268696	179,9476387	237,9270047	108,5062616	208,9373217	1,925578474	0,945291919	0,0006592	0,0018097	conserved Plasmodium protein, unknown function
PFIT_0505900	648,05953	681,13694	420,2574599	367,1382994	664,5982371	393,6978797	0,592384779	-0,755393523	0,0006599	0,0018108	conserved Plasmodium protein, unknown function
PFIT_1358200	1897,8886	2270,7776	1411,108382	1182,506125	2084,333122	1296,807253	0,622168904	-0,684621805	0,0006659	0,0018263	riboflavin kinase / FAD synthase family protein, putative
PFIT_0502600	362,2693	315,03788	189,0589115	185,3513745	338,6535909	187,205143	0,552792435	-0,855190224	0,0006662	0,0018263	conserved Plasmodium protein, unknown function
PFIT_1301400	325,03607	245,67174	709,5403728	528,4296397	285,3539047	618,9850062	2,169183586	1,117152159	0,0006691	0,0018334	early transcribed membrane protein 13
PFIT_1429500	4862,4591	3941,3455	2342,73603	2841,757371	4401,90227	2592,246701	0,588892379	-0,763924091	0,0006698	0,0018335	proliferation-associated protein 2g4, putative
PFIT_1343600	142,89511	119,46391	247,1432759	246,8381285	131,1795095	246,9907022	1,882845141	0,912914347	0,0006697	0,0018335	conserved Plasmodium protein, unknown function
PFIT_1001700	44014,714	47924,294	33022,66952	25615,9164	45969,50418	29319,29296	0,637798764	-0,648826793	0,0006743	0,001845	PF70 protein
PFIT_1305300	6,0378217	4,817093	22,77818211	33,86227033	5,427457332	28,32022622	5,21795465	2,383484405	0,0006795	0,0018582	conserved Plasmodium protein, unknown function
PFIT_0400300.2	3206,0833	2727,438	1879,200024	1795,59144	2966,76068	1837,395732	0,61932725	-0,691226171	0,0006804	0,0018599	rifin
PFIT_0609700	19623,927	21113,318	14736,34492	11247,62043	20368,62264	12991,98267	0,637842966	-0,648726813	0,0006824	0,0018635	transketolase
PFIT_0830500	554,47329	568,41697	820,014556	1028,343683	561,4451312	924,1791196	1,646072017	0,719027456	0,0006823	0,0018635	conserved Plasmodium protein, unknown function
PFIT_0602400	125,78795	123,31758	214,1149118	253,9670275	124,552766	234,0409697	1,879050761	0,91000404	0,0006832	0,0018649	cation/H antiporter
PFIT_0600400	148,93294	170,52509	260,8101852	319,9093434	159,7290131	290,3597643	1,817827323	0,86215163	0,0006875	0,0018757	Tetratricopeptide repeat protein, putative
PFIT_1201100	442,77359	470,14827	215,2538209	296,7404216	456,4609323	255,9971213	0,560830299	-0,834363801	0,0006906	0,0018831	Plasmodium exported protein, unknown function
PFIT_1324900	0	0,9634186	14,80581837	13,36668566	0,481709296	14,08625202	29,24222582	4,869981224	0,0006933	0,0018895	conserved Plasmodium protein, unknown function
PFIT_1244600	4862,4591	5016,5206	3335,86477	2879,184091	4939,489844	3107,52443	0,629118498	-0,668596313	0,000703	0,0019151	conserved Plasmodium protein, unknown function
PFIT_1234200	996,24058	1005,809	608,1774624	597,9364051	1001,024796	603,0569337	0,602439556	-0,731111594	0,0007048	0,0019192	conserved Plasmodium protein, unknown function
[illegible]	[illegible]	[illegible]	[illegible]	[illegible]	[illegible]	[illegible]	[illegible]	[illegible]	[illegible]	[illegible]	[illegible]

PFIT_0528500	1308,1947	1389,2496	931,6276483	842,1011965	1348,722156	886,8644224	0,657559022	-0,604807699	0,0035509	0,0080315	conserved Plasmodium protein, unknown function
PFIT_1212000	34,214323	46,244092	71,75127365	98,02236149	40,2292077	84,88681757	2,110079279	1,077297204	0,0035672	0,0080652	tetratricopeptide repeat family protein, putative
PFIT_0304400	60,378217	47,207511	123,0021834	203,173622	53,79286403	163,0879027	3,031775788	1,600163064	0,0035851	0,0081023	1-cys-glutaredoxin-like protein-1
PFIT_1469600	82,516897	93,451603	134,3912745	182,6780373	87,98425003	158,5346559	1,801852671	0,849481053	0,0035891	0,008108	derlin-1 (DER1-1)
PFIT_0811200	3082,308	2771,7553	4841,502608	4032,283507	2927,031635	4436,893057	1,515833654	0,600111442	0,0036018	0,0081337	ubiquitin regulatory protein, putative
PFIT_0202500	1145,1735	1216,7977	810,9032831	736,949936	1180,985599	773,9266096	0,655322648	-0,609722704	0,0036087	0,0081458	conserved Plasmodium protein, unknown function
PFIT_1433000	180,12835	150,2933	300,6720039	260,2048141	165,2108239	280,438409	1,697457844	0,763375746	0,0036128	0,008152	apyrase, putative
PFIT_1466500	1383,6675	1426,8229	997,6843765	859,0323316	1405,245204	928,358354	0,660637981	-0,598068181	0,0036211	0,0081674	conserved Plasmodium protein, unknown function
PFIT_0708300	203,27333	164,74458	317,7556404	462,4873238	184,008955	390,1214821	2,120122263	1,084147465	0,0036271	0,0081776	Cg7 protein
PFIT_0811800	35,220627	34,683069	9,111272844	11,5844609	34,95184796	10,34786687	0,296060651	-1,756035336	0,0036391	0,008198	unspecified product
PFIT_0204000	8591,8203	6937,5773	5030,561519	5298,554195	7764,698783	5164,557857	0,665133059	-0,588285115	0,0036383	0,008198	hexose transporter
PFIT_1233300	14983,861	14112,156	9536,085941	9910,060747	14548,0082	9723,073344	0,668343955	-0,581337335	0,003643	0,0082037	asparagine and aspartate rich protein 1
PFIT_0928700	388,4332	494,23374	299,5330948	229,9069933	441,333467	264,720044	0,599818649	-0,737401717	0,0036475	0,0082105	conserved Plasmodium protein, unknown function
PFIT_0722800	609,81999	658,97832	928,210921	1021,214784	634,3991546	974,7128527	1,536434665	0,61958642	0,0036519	0,0082172	protein kinase, putative
PFIT_1457500	298,87217	265,90353	154,8916384	177,3313631	282,3878529	166,1115007	0,588238832	-0,76552607	0,0036648	0,0082429	conserved Plasmodium protein, unknown function
PFIT_0407300	19,119769	16,378116	53,52872796	43,66450648	17,7489424	48,59661722	2,738000729	1,453122831	0,0036897	0,0082956	perforin like protein 1,sporozoite micronemal protein essential for cell traversal (SPECT2)
PFIT_1027000	342,14323	314,07446	189,0589115	204,9558468	328,1088455	197,0073791	0,600433002	-0,735924819	0,0036986	0,0083123	topoisomerase, putative
PFIT_1448100	2948,4696	3661,9541	2330,20803	1776,87808	3305,211834	2053,543055	0,621304521	-0,686627543	0,0037033	0,0083196	glutaminyl-peptide cyclotransferase, putative
PFIT_1362600	11,06934	7,7073487	33,02836406	32,08004558	9,388344264	32,55420482	3,467512897	1,793901249	0,0037186	0,0083507	conserved Plasmodium protein, unknown function
PFIT_0106900.1	611,8326	773,62513	474,925097	360,0094004	692,7288644	417,4672487	0,602641625	-0,73062777	0,003738	0,0083909	mitochondrial carrier protein, putative
PFIT_1306400	768,81596	790,96666	492,0087336	507,934055	779,8913139	499,9713943	0,641078296	-0,641427529	0,0037512	0,0084171	DnaJ protein, putative
PFIT_0624700	280,75871	288,06216	195,8923662	148,815767	284,4104341	172,3540666	0,606004724	-0,722599055	0,003763	0,0084402	conserved Plasmodium protein, unknown function
PFIT_1007600	14461,589	16529,373	10890,24887	10050,8565	15495,48103	10470,55268	0,675716531	-0,565509946	0,0037666	0,008445	inositol polyphosphate kinase, putative (IPK1)
PFIT_0520400	814,09963	814,08871	515,9258248	530,2118644	814,0941684	523,0688446	0,642516388	-0,638194843	0,0037794	0,0084703	conserved Plasmodium protein, unknown function
PFIT_1109900	486,04465	405,59923	693,5956453	697,7409914	445,8219372	695,6683183	1,560417423	0,641932012	0,0037913	0,0084937	conserved Plasmodium protein, unknown function
PFIT_0722900	10466,564	10722,849	7579,440097	6743,938471	10594,70643	7161,689284	0,675968639	-0,564971779	0,0038047	0,0085203	conserved Plasmodium protein, unknown function
PFIT_1301700	4308,9921	4409,5669	3049,998585	2802,548426	4359,279493	2926,273505	0,671274579	-0,575025086	0,0038097	0,008528	conserved Plasmodium protein, unknown function
PFIT_0711500	3924,5841	4048,2849	3006,720039	2389,963396	3986,434516	2698,341717	0,676880984	-0,563025908	0,0038207	0,0085492	serpentine receptor, putative
PFIT_0530500	1626,1866	1616,6164	1200,410197	963,2924798	1621,401522	1081,851339	0,667232221	-0,583739136	0,0038257	0,0085537	nucleolar Jumonji domain interacting protein, putative
Pf_M7661101400	6,0378217	8,6707673	27,33381853	29,40670845	7,354294516	28,37026349	3,857645819	1,947720692	0,0038245	0,0085537	unspecified product
PFIT_0723100	1494,3609	950,89415	2168,482937	3009,286498	1222,627511	2588,884717	2,11747625	1,082345788	0,0038295	0,0085587	kinesin-like protein, putative
PFIT_1423400	4771,8918	4628,2629	3118,333131	3156,32004	4700,077335	3137,326586	0,667505311	-0,583148778	0,0038325	0,0085622	conserved Plasmodium protein, unknown function
PFIT_0612000.3	16,100858	11,561023	30,75054585	49,01118075	13,83094049	39,8808633	2,883452743	1,527797378	0,0038492	0,0085961	conserved Plasmodium protein, unknown function
PFIT_1433300	967,05778	1173,4438	701,568009	695,9587666	1070,250811	698,7633878	0,652896854	-0,615073004	0,0038764	0,0086534	conserved protein, unknown function
PFIT_1455900	3646,8443	2896,9997	1995,368753	2236,692067	3271,922008	2116,03041	0,646723976	-0,628777999	0,0038838	0,0086665	6-phosphogluconate dehydrogenase, decarboxylating, putative
PFIT_0714800	141,88881	138,73228	218,6705483	256,6403646	140,3105437	237,6554565	1,69378188	0,760248101	0,0038943	0,0086864	conserved Plasmodium protein, unknown function
PFIT_1111000	15932,805	16192,176	11939,18415	9918,971871	16062,49073	10929,07801	0,680409919	-0,555523922	0,0039134	0,0087257	pre-mRNA-processing factor 6, putative
PFIT_1415600	3497,9114	3450,9654	2474,849486	2193,918673	3474,438385	2334,38408	0,671873788	-0,573737848	0,0039185	0,0087335	RNA guanylyltransferase
PFIT_1362300	230,44353	230,25704	150,3360019	121,1912833	230,3502859	135,7636426	0,589379093	-0,76273221	0,0039237	0,0087417	mitochondrial ribosomal protein L9 precursor, putative
PFIT_1432900	14294,543	18859,882	10608,93832	7665,348669	16577,21262	9137,143493	0,551186964	-0,859386328	0,0039369	0,0087675	60S ribosomal protein L14, putative
PFIT_0817000	181,13465	159,92749	87,69600113	99,80458625	170,5310687	93,75029369	0,549754918	-0,86313949	0,0039618	0,0088196	conserved Plasmodium protein, unknown function
PFIT_0718400	725,54491	706,18583	447,5912785	467,833998	715,8653681	457,7126383	0,639383687	-0,645246159	0,0039672	0,0088282	conserved Plasmodium protein, unknown function
PFIT_1477100	258,62003	203,28132	150,3360019	92,67568723	230,9506763	121,5058446	0,526111664	-0,92655906	0,0039772	0,0088469	conserved Plasmodium protein, unknown function
PFIT_0301500	7821,998	9029,159	6244,638626	4229,219342	8425,578531	5236,928984	0,621551264	-0,686054709	0,0039926	0,0088776	Plasmodium exported protein (hyp1), unknown function
PFIT_0623000	94,59254	100,19553	189,0589115	163,073565	97,3940368	176,0662383	1,807772263	0,854212943	0,0039953	0,0088801	conserved Plasmodium protein, unknown function
PFIT_0211900	13859,82	15499,478	11114,61396	8936,966031	14679,64902	10025,79	0,682972051	-0,550101554	0,0039981	0,008883	conserved Plasmodium protein, unknown function
PFIT_1472100	1073,726	1317,9566	859,8763747	654,0764849	1195,841297	756,9764298	0,633007433	-0,659705654	0,0040226	0,0089338	RNA 3'-terminal phosphate cyclase-like protein, putative
PFIT_0811000	1274,9867	1295,798	859,8763747	830,5167356	1285,392345	845,1965551	0,657539745	-0,604849993	0,0040253	0,0089364	conserved Plasmodium protein, unknown function
PFIT_0308900	1081,7764	1346,8592	867,8487384	728,0388122	1214,31779	797,9437753	0,65711281	-0,605787028	0,004034	0,0089521	dual specificity phosphatase
PFIT_0609800	3904,458	3292,0013	2211,761483	2547,690286	3598,229682	2379,725885	0,661360195	-0,596491877	0,0040594	0,009005	transcription factor, putative
PFIT_1002000.1	11671,109	11644,841	8492,8452	7342,765988	11657,97494	7917,805594	0,67917504	-0,558144655	0,0040689	0,0090224	RNA binding protein, putative
PFIT_1308800	855,35807	768,80804	519,3425521	527,5385273	812,0830556	523,4405397	0,644565277	-0,633601623	0,0040802	0,0090441	ribosomal RNA methyltransferase, putative
PFIT_0820300	122,76904	167,63484	223,2261847	266,4426008	145,2019382	244,8343927	1,68616477	0,753745522	0,0041044	0,0090941	conserved Plasmodium protein, unknown function
PFIT_1016300	4618,9336	4610,9214	7406,325913	6435,613588	4614,927493	6920,969751	1,49969198	0,584666218	0,0041467	0,0091843	Plasmodium exported protein (PHISTc), unknown function
PFIT_0701200	13166,477	17667,17	9642,004488	7074,541163	15416,82334	8358,272825	0,542152728	-0,88322877	0,0041812	0,009257	sporozoite threonine and asparagine-rich protein (STARP)
PFIT_0705600	645,04062	567,45355	358,7563682	407,2383564	606,2470847	382,9973623	0,631751264	-0,662571448	0,0042061	0,0093084	DNA mismatch repair protein, putative
PFIT_1130700	4599,8138	4744,8366	3298,28077	3010,17761	4672,3252	3154,22919	0,675087682	-0,5668532	0,0042241	0,0093446	transporter, putative
PFIT_0806500	258,62003	234,11072	341,6727317	446,447301	246,3653738	394,0600163	1,599494321	0,67761587	0,0042357	0,0093665	tubulin gamma chain
PFIT_1347400	498,12029	503,86792	350,7840045	288,7204102	500,9941071	319,7522074	0,638235466	-0,647839315	0,0042479	0,00939	step II splicing factor, putative
PFIT_0527900	1197,5013	1153,2121	732,3185549	801,1100271	1175,35668	766,714291	0,652324783	-0,616337655	0,0042774	0,0094515	conserved Plasmodium membrane protein, unknown function
PFIT_1367600	81,510593	87,671092	46,69527333	34,75338271	84,59084244	40,72432802	0,481427148	-1,054610596	0,0042816	0,009457	mitochondrial import inner membrane translocase subunit Tim9, putative (TIM9)
PFIT_1412700	552,46069	606,95371	395,2014596	349,3160519	579,7071995	372,2587557	0,642149616	-0,639018621	0,0042833	0,009457	conserved protein, unknown function
PFIT_1405800	48675,912	50693,159	36202,50374	31605,08268	49684,53588	33903,79321	0,6823812	-0,551350195	0,0042983	0,0094865	conserved Plasmodium protein, unknown function
PFIT_0207400.2	250,5696	250,48883	358,7563682	438,4272896	250,5292173	398,5918289	1,590999378	0,669933272	0,0043003	0,0094871	conserved Plasmodium protein, unknown function
PFIT_0910500	1363,5414	1143,5779	750,5411006	879,5279163	1253,559635	815,0345084	0,650176095	-0,621097581	0,0043245	0,0095351	conserved Plasmodium protein, unknown function
PFIT_1442200	829,19418	842,99127	568,3156437	522,191853	836,0927244	545,2537484	0,65214507	-0,616735166	0,0043254	0,0095351	conserved Plasmodium protein, unknown function
PFIT_1128700	2867,9653	3013,5734	1859,838569	2052,231805	2940,769333	1956,035187	0,665144037	-0,588261304	0,0043363	0,0095555	conserved Plasmodium protein, unknown function
PFIT_1208100	438,74838	447,98965	268,7825489	284,2648483	443,3690112	276,5236986	0,623687474	-0,681104811	0,0043393	0,0095583	conserved Plasmodium protein, unknown function
PFIT_1245200	144,90772	128,13467	218,6705483	243,273679	136,5211968	230,9721136	1,691840674	0,758593712	0,0043747	0,0096326	conserved Plasmodium protein, unknown function
PFIT_1242700	64,403432	54,91486	96,80727397	130,1024071	59,65914563	113,4548405	1,901717487	0,92730294	0,004407	0,0096998	conserved protein, unknown function
PFIT_1145200	460,88706	399,81872	241,4487304	289,6115226	430,3528862	265,5301265	0,617005567	-0,696644589	0,0044277	0,0097416	ubiquitin activating enzyme (E1) subunit Aos1, putative
PFIT_1245600	99,624058	95,378441	177,6698205	170,202464	97,50124937	173,9361423	1,783937574	0,835065131	0,0044296	0,009742	mitochondrial ribosomal protein L3 precursor, putative
PFIT_1226900	6348,7695	7925,0813	5096,618247	4165,059251	7136,92543	4630,838749	0,648856261	-0,624029177	0,0044391	0,0097554	potassium channel protein
PFIT_0825100	1764,0502	1714,8851	1127,520014	1175,377226	1739,467668	1151,44862	0,661954598	-0,595195826	0,0044386	0,0097554	conserved Plasmodium membrane protein, unknown function
PFIT_1418900	5,0315181	13,48786	28,47272764	34,75338271	9,259689188	31,61305517	3,414051437	1,771484794	0,0044973	0,0098794	conserved Plasmodium protein, unknown function
PFIT_1225500	7012,9299	6420,2215	4192,324418	4788,837915	6716,575704	4490,581166	0,668581933	-0,580823725	0,0045102	0,0099038	ubiquitin-activating enzyme E1 (UBA1)
PFIT_0407200	201,26072	159,92749	318,8945496	280,7003988	180,5941049	299,7974742	1,66006235	0,731237429	0,0045147	0,0099099	sporozoite invasion-associated protein 1 (SIAP-1)
PFIT_0909400	77,485379	60,695371	144,6414564	117,6268338	69,09037492	131,1341451	1,89800888	0,924486742	0,0045286	0,0099366	GDP-fucose protein O-fucosyltransferase 2, putative (POFUT2)
PFIT_1229000	2301,4164	3771,7838	6758,286632	5278,949723	3036,60008	6018,618177	1,982025297	0,986975376	0,0045482	0,0099757	conserved Plasmodium protein, unknown function
PFIT_1129000	2097,1367	2545,3519	1702,669113	1287,657385	2321,244329	1495,163249	0,644121444	-0,634595373	0,0045655	0,0100096	cochaperone prefoldin complex subunit, putative
PFIT_0617900.2	2613,3705	2317,9851	1395,163654	1843,711508	2465,677813	1619,437581	0,656792048	-0,606491434	0,0045728	0,0100218	conserved Plasmodium protein, unknown function
PFIT_1133000	13995,671	17411,864	11014,38996	7815,055548	15703,76746	9414,722754	0,599520005	-0,738120199	0,0045894	0,0100518	TCP-1/cpn60 chaperonin family, putative
PFIT_1323400	824,16266	717,74685	445,3134603	540,905213	770,9547569	493,1093366	0,639608657	-0,644738629	0,00459	0,0100518	micro-fibrillar-associated protein, putative
PFIT_1235400	794,97986	662,83199	978,3229217	1206,566159	728,9059245	1092,44454	1,49874559	0,583755509	0,0046007	0,0100713	ATP synthase subunit beta, mitochondrial
PFIT_0609000	4418,6792	5717,8893	3409,893862	2678,683806	5068,284264	3044,288834	0,600654714	-0,735392199	0,0046119	0,0100919	Pre-mRNA-splicing factor SLU7, putative
PFIT_1347100	89,561022	103,08579	169,6974567	254,8581399	96,32340565	212,2777983	2,203802875	1,139995184	0,0046269	0,0101207	conserved Plasmodium protein, unknown function
PFIT_0204700	923,78672	744,72257	533,0094614	548,9252244	834,2546462	540,9673429	0,648443908	-0,624946312	0,0046915	0,0102541	conserved Plasmodium protein, unknown function
PFIT_1335300	85,535807	101,15895	162,8640021	170,202464	93,34737982	166,5332331	1,784016149	0,835128675	0,0046909	0,0102541	conserved Plasmodium protein, unknown function
PFIT_0825000	642,02171	578,05116	436,2021874	355,5538385	610,0364316	395,878013	0,648941592	-0,623839461	0,004701	0,0102709	RNA polymerase II mediator complex protein MED7, putative
PFIT_0301600	267,67676	352,6112	717,5127365	519,5185159	310,1439835	618,5156262	1,994285426	0,995871906	0,0047114	0,0102897	Plasmodium exported protein, unknown function
PFIT_1413300	2474,5006	2138,7893	1480,581837	1588,853369	2306,644935	1534,717603	0,66534627	-0,587822729	0,0047458	0,0103607	nuclear transport factor 2, putative
PFIT_1346300	430,69795	588,64876	799,5141921	776,1588806	509,673354	787,8365363	1,545767559	0,628323394	0,0047479	0,0103613	DNA topoisomerase III, putative
PFIT_1343300	1351,4658	1212,944	1963,479298	1881,138228	1282,204883	1922,308763	1,499221216	0,584213274	0,004814	0,0105016	krox-like protein. putative
PFIT_1014200	209,31115	204,24474	127,5578198	114,0623843	206,777947	120,8101021	0,584250418	-0,775341232	0,0048242	0,0105198	prohibitin, putative
PFIT_1125700	4398,5531	5350,8269	3529,479318	2530,759151	4874,689986	3030,119235	0,621602449	-0,685935907	0,0048267	0,0105213	nuclear preribosomal assembly protein, putative
PFIT_0409200	966,05147	869,96699	1316,578926	1433,799815	918,0092307	1375,18937	1,498012574	0,583049733	0,0048365	0,0105343	conserved Plasmodium protein, unknown function
PFIT_1338000	30,189109	17,341535	63,77890991	51,68451788	23,76532159	57,73171389	2,429241855	1,280506131	0,0048347	0,0105343	CPW-WPC family protein
PFIT_1247100	208,30485	243,7449	387,2290959	347,5338271	226,0248763	367,3814615	1,625402777	0,700797264	0,0048737	0,0106113	RAC-beta serine/threonine protein kinase
PFIT_0829400	5327,3714	5449,0956	3815,345504	3510,091654	5388,233454	3662,718579	0,679762414	-0,556897502	0,0048771	0,0106134	ubiquitin conjugation factor E4 B, putative (UBE4B)
PFIT_1464400	72,45386	61,65879	123,0021834	128,3201823	67,05632517	125,6611829	1,873964649	0,906093738	0,0048784	0,0106134	DNA polymerase alpha subunit, putative
PFIT_0610700	2032,7333	1559,7747	861,0152838	1145,079405	1796,254004	1003,047344	0,558410638	-0,840601669	0,0048926	0,0106402	60S ribosomal protein L39, putative
PFIT_1128900.2	6029,7713	5785,3286	3955,431324	4026,936833	5907,54996	3991,184078	0,675607334	-0,565743106	0,004897	0,0106417	conserved Plasmodium protein, unknown function
PFIT_1143600	293,84066	384,40402	543,2596433	524,8651902	339,1223373	534,0624168	1,574837037	0,655202547	0,0048965	0,0106417	conserved Plasmodium protein, unknown function
PFIT_0813600	88,554718	68,40272	36,44509138	36,53560747	78,47871918	36,49034942	0,464971266	-1,10478653	0,0049055	0,010656	conserved Plasmodium protein, unknown function
PFIT_1103400	123,77534	146,43963	71,75127365	73,96232731	135,1074855	72,85680048	0,539250658	-0,890972062	0,0049192	0,0106817	Plasmodium exported protein, unknown function
PFIT_1404000	268,68307	207,135	591,0938258	1040,819257	237,9090316	815,9565412	3,429699729	1,778082274	0,0049326	0,0107066	conserved Plasmodium protein, unknown function
PFIT_0402900	5,0315181	4,817093	22,77818211	22,27780943	4,924305524	22,52799577	4,574857442	2,193726788	0,0049516	0,0107437	conserved Plasmodium protein, unknown function
PFIT_1020200	8005,1453	8369,2173	6376,752082	4914,48476	8187,181293	5645,618421	0,689568023	-0,536235219	0,0049801	0,0108014	ribosomal processing ATPase, putative
PFIT_1443100	104,65558	106,93946	55,80654617	52,57563026	105,79752	54,19108821	0,512215109	-0,965178286	0,005003	0,0108428	transcription factor TFIIH complex subunit Tfb5, putative
PFIT_1023500	500,1329	636,81969	1136,631287	867,052343	568,4762936	1001,841815	1,762328221	0,817482641	0,0050023	0,0108428	conserved Plasmodium protein, unknown function
PFIT_0528100	2339,6559	2184,0699	1605,861839	1446,275388	2261,862929	1526,068614	0,674695444	-0,567691675	0,0050272	0,0108909	conserved Plasmodium membrane protein, unknown function
PFIT_1414900	1876,7562	1767,8731	1292,661835	1158,44609	1822,314681	1225,553963	0,672525978	-0,572338099	0,0050427	0,0109205	DnaJ protein, putative
PFIT_0208400	2312,4857	2117,5941	1665,085112	1344,688577	2215,039889	1504,886845	0,679394918	-0,557677667	0,0051117	0,0110656	phospholipase A2, putative
PFIT_1234400	264,65785	332,37941	167,4196385	194,2624982	298,5186328	180,8410684	0,60579491	-0,723098638	0,0051373	0,0111166	CPW-WPC family protein
PFIT_1353800	1940,1534	2435,5222	1472,609473	876,8545792	2187,837788	1174,732026	0,536937443	-0,897174082	0,005168	0,011179	60S ribosomal protein L7-2, putative
PFIT_0512500	278,7461	340,08676	201,5869117	180,8958126	309,4164325	191,2413621	0,618071124	-0,694155231	0,0051942	0,0112311	conserved Plasmodium protein, unknown function
PFIT_1018500	28385,812	30635,748	22023,08537	18667,02208	29510,78012	20345,05373	0,689410908	-0,536563969	0,005216	0,0112741	eukaryotic translation initiation factor subunit eIF2A, putative
PFIT_1007200	1303,1632	1354,5665	944,1556485	836,7545222	1328,864862	890,4550853	0,670087012	-0,57757965	0,0052283	0,0112963	conserved Plasmodium protein, unknown function
PFIT_0509300	625,92085	703,29557	495,4254609	381,3960974	664,6082111	438,4107792	0,659652968	-0,600220847	0,0052617	0,0113642	conserved Plasmodium protein, unknown function
PFIT_1127800	1792,2267	1918,1664	1456,664746	1079,137089	1855,19658	1267,900917	0,683432112	-0,549130058	0,0053341	0,011516	small nuclear ribonucleoprotein F, putative
PFIT_0420300	1133,0979	1017,37	733,457464	692,3943171	1075,233953	712,9258905	0,663042576	-0,592826581	0,0053453	0,0115343	transmembrane emp24 domain-containing protein, putative
PFIT_0110800	139,8762	129,09809	203,8647299	245,0559037	134,4871471	224,4603168	1,669009431	0,738992107	0,0053466	0,0115343	multidrug resistance-associated protein 1
PFIT_0706900	169,05901	160,8909	246,0043668	343,0782652	164,9749563	294,541316	1,785369868	0,836222983	0,0054081	0,0116624	cytoskeleton associated protein, putative
PFIT_1435200	547,42917	556,85595	362,1730956	349,3160519	552,142557	355,7445737	0,644298414	-0,634199052	0,005436	0,0117183	CCR4-associated factor 16, putative
PFIT_0810400	3553,2581	3678,3322	2503,322214	2409,567868	3615,795129	2456,445041	0,679365106	-0,557740975	0,0054799	0,0118061	proteasome subunit alpha, putative
PFIT_1445100	1159,2618	1306,3956	1855,282933	1824,998149	1232,828689	1840,140541	1,492616579	0,577843616	0,005481	0,0118061	heat shock protein 90, putative
PFIT_0609500	562,52372	646,45388	1031,85165	829,6256232	604,4887987	930,7386364	1,539711966	0,622660491	0,0054851	0,0118106	calcium-dependent protein kinase 2 (CDPK2)
PFIT_1008100	27,170198	23,122046	56,94545528	60,59564165	25,14612194	58,77054846	2,337161516	1,224757439	0,0054894	0,0118152	zinc finger C-x8-C-x5-C-x3-H type, putative
PFIT_1402400	1958,2668	2587,7423	1446,414564	848,3389831	2273,004589	1147,376774	0,504784187	-0,986261378	0,0055061	0,0118421	lysophospholipase, putative
PFIT_0808400	2858,9086	2684,0842	1913,367297	1838,364834	2771,496387	1875,866066	0,676842328	-0,563108302	0,0055049	0,0118421	conserved Plasmodium protein, unknown function
PFIT_1120700	10389,079	9529,1733	6340,306991	7134,245692	9959,12592	6737,276341	0,676492736	-0,563853652	0,0055205	0,0118687	conserved Plasmodium protein, unknown function
PFIT_1363400	8211,4375	6446,2338	3936,069869	4943,891469	7328,835659	4439,980669	0,605823473	-0,723030619	0,0055232	0,01187	conserved Plasmodium protein, unknown function
PFIT_1446200	62,390824	62,622208	108,196365	163,9646774	62,50651638	136,0805212	2,177061354	1,122382066	0,0055329	0,0118862	conserved Plasmodium protein, unknown function
PFIT_1475200	8,0504289	7,7073487	21,63927301	33,86227033	7,878888838	27,75077167	3,522168194	1,816463804	0,0055376	0,0118917	mitochondrial ribosomal protein L20 precursor, putative
PFIT_0502500	10778,518	8872,1218	6891,538998	6530,0715	9825,31993	6710,805249	0,683011372	-0,550018495	0,0055622	0,0119401	merozoite surface protein 8
PFIT_0723400	446,79881	436,42862	264,2269125	292,2848597	441,6137142	278,2558861	0,63008887	-0,666372769	0,0055785	0,0119614	mago nashi protein homolog, putative
PFIT_1131800	50,315181	37,573325	91,11272844	87,32901297	43,94425298	89,22087071	2,030319431	1,021706725	0,0055772	0,0119614	RNA-binding protein s1, putative
PFIT_0701000	3228,222	3751,552	11795,68161	7347,22155	3489,887001	9571,451578	2,742625069	1,455557412	0,0055751	0,0119614	Plasmodium exported protein (PHISTb), unknown function
PFIT_0930600.2	2755,2593	2253,4361	1466,914928	1808,067013	2504,347696	1637,490971	0,653859276	-0,612947924	0,0055825	0,0119655	conserved Plasmodium protein, unknown function
PFIT_1415000	323,02346	441,24572	593,371644	592,5897308	382,1345882	592,9806874	1,551758741	0,633904273	0,0055871	0,0119708	26S proteasome regulatory subunit RPN13, putative (RPN13)
PFIT_1236100	2870,9842	2610,8644	1908,811661	1806,284789	2740,924302	1857,548225	0,677708692	-0,561262819	0,0056047	0,0120039	conserved Plasmodium protein, unknown function
PFIT_0913400	2097,1367	2399,8757	1591,05602	1461,424299	2248,506226	1526,24016	0,678779601	-0,558984885	0,0056078	0,0120062	conserved Plasmodium protein, unknown function
PFIT_0417000	166,0401	159,92749	99,08509218	86,43790059	162,9837916	92,76149639	0,56914553	-0,8131305	0,0056115	0,0120095	conserved Plasmodium protein, unknown function
PFIT_0306600	1003,2847	845,88152	563,7600072	644,2742487	924,5831152	604,017128	0,653285917	-0,614213556	0,0056532	0,0120942	membrane magnesium transporter, putative
PFIT_1306500	525,29049	500,97767	305,2276403	347,5338271	513,1340781	326,3807337	0,636053514	-0,652779943	0,005706	0,0122024	DEAD box helicase, putative
PFIT_1002800	43,271056	26,012302	74,02909186	73,96232731	34,64167877	73,99570958	2,136031284	1,094932777	0,0057196	0,0122269	conserved Plasmodium protein, unknown function
PFIT_0312900	1664,4262	1652,2629	1191,298924	1052,403717	1658,344534	1121,851321	0,67648869	-0,563862281	0,0057601	0,0123089	conserved Plasmodium protein, unknown function
PFIT_0918000	58925,115	55303,117	39259,33578	39019,13766	57114,11603	39139,23672	0,685281318	-0,545231737	0,0057897	0,0123674	heat shock protein 70
PFIT_1469100	267,67676	245,67174	357,6174591	443,7739639	256,6742516	400,6957115	1,561105989	0,642568491	0,0057934	0,0123706	conserved Plasmodium protein, unknown function
PFIT_1462000	160,00228	150,2933	246,0043668	264,660376	155,1477878	255,3323714	1,645736463	0,718733331	0,0058119	0,0124055	snRNA-activating protein complex subunit 3, putative
PFIT_0826500	449,81772	415,23341	321,1723678	237,9270047	432,5255651	279,5496862	0,646319452	-0,629680682	0,0058521	0,0124866	lipoate-protein ligase (LipB)
PFIT_0930600.1	2641,547	2257,2898	1389,469109	1831,235935	2449,418378	1610,352522	0,657442818	-0,605062675	0,0058815	0,0125353	conserved Plasmodium protein, unknown function
PFIT_0929100	664,16039	814,08871	520,4814612	458,0317619	739,1245489	489,2566116	0,661940687	-0,595226145	0,0058807	0,0125353	guanylate kinase
PFIT_0522500	936,86867	1029,8945	750,5411006	575,6585957	983,3815714	663,0998481	0,67430575	-0,568525195	0,0058816	0,0125353	S-adenosylmethionine-dependent methyltransferase,, putative
PFIT_1034300	9382,7749	7711,2024	5315,288796	6233,331079	8546,98867	5774,309937	0,675595834	-0,565767663	0,0058915	0,0125468	methionine--tRNA ligase, putative
PFIT_0934100	1675,4955	1749,5682	1199,271288	1115,672696	1712,531843	1157,471992	0,675883486	-0,56515353	0,0058912	0,0125468	mitochondrial-processing peptidase subunit beta, putative (MAS1)
PFIT_1331800	1565,8084	1488,4817	932,7665574	1096,068224	1527,145077	1014,417391	0,664257382	-0,590185739	0,0059408	0,0126471	SAM-dependent RNA methyltransferase, putative
PFIT_1117800	150,94554	175,34218	234,6152757	315,4537815	163,1438632	275,0345286	1,68584048	0,75346803	0,0059612	0,0126859	conserved Plasmodium membrane protein, unknown function
PFIT_0906600	232,45614	319,85497	152,6138201	160,4002279	276,1555541	156,507024	0,566735022	-0,819253737	0,0059705	0,0127007	dynein light intermediate chain 2, cytosolic
PFIT_1206100	51,321484	58,768534	103,6407286	107,8245976	55,0450093	105,7326631	1,920840135	0,941737454	0,0061616	0,0131024	Tat binding protein 1(TBP-1)-interacting protein, putative
PFIT_1036300	216,35528	206,17158	391,7847323	297,631534	211,2634282	344,7081331	1,63165076	0,706332294	0,0061822	0,0131413	pyruvate kinase 2, putative
PFIT_0322500	2273,2399	1724,5193	1171,93747	1280,528486	1998,879576	1226,232978	0,613460157	-0,704958449	0,0062169	0,0132101	regulator of initiation factor 2 (eIF2)
PFIT_1204400	42,264752	31,792814	63,77890991	88,22012534	37,02878273	75,99951763	2,052444396	1,037343138	0,0063302	0,0134458	conserved Plasmodium protein, unknown function
PFIT_1411300	7515,0754	7372,0791	4691,166606	5422,418815	7443,577241	5056,792711	0,6793498	-0,557773479	0,0063396	0,0134606	conserved Plasmodium protein, unknown function
PFIT_1141400	3736,4053	3540,5633	2487,377487	2475,510184	3638,484329	2481,443835	0,681999319	-0,552157796	0,006349	0,0134755	conserved Plasmodium protein, unknown function
Pf_M7661102400	14,088251	5,7805116	25,05600032	40,99116935	9,934381098	33,02358484	3,324171331	1,732994742	0,0063582	0,01349	unspecified product
PFIT_0817100	6195,8114	4449,0671	1963,479298	3028,89097	5322,439215	2496,185134	0,468992699	-1,09236263	0,0064146	0,0135994	DNA/RNA-binding protein Alba 1 (ALBA1)
PFIT_1453100	4417,6729	4555,0431	3251,585496	2925,521934	4486,357992	3088,553715	0,688432292	-0,538613324	0,006414	0,0135994	small subunit rRNA processing factor, putative
PFIT_0208800	285,79023	259,1596	488,5920063	377,8316479	272,4749143	433,2118271	1,589914537	0,668949218	0,0064195	0,0136047	conserved Plasmodium protein, unknown function
PFIT_1132000	79,497986	73,219813	138,9469109	218,3225324	76,35889939	178,6347216	2,33940933	1,226144314	0,0064434	0,0136503	conserved Plasmodium protein, unknown function
PFIT_0825700	2193,7419	2125,3014	1573,972384	1378,550848	2159,52165	1476,261616	0,683605842	-0,548763369	0,0064497	0,0136584	U5 snrnp-specific protein, putative
PFIT_0320200	213,33637	228,33021	142,3636382	123,8646204	220,8332866	133,1141293	0,60278109	-0,730293935	0,0064542	0,0136628	CPW-WPC family protein
PFIT_1463300	244,53178	248,562	367,8676411	405,4561316	246,5468879	386,6618864	1,568309743	0,649210521	0,0065297	0,0138175	conserved Plasmodium protein, unknown function
PFIT_0906700	3976,9119	3653,2833	2695,797853	2534,323601	3815,097599	2615,060727	0,685450545	-0,544875516	0,0065495	0,0138543	arginase
PFIT_1360500.2	8,0504289	3,8536744	21,63927301	42,77339411	5,952051654	32,20633356	5,410963384	2,435885479	0,0065767	0,0139066	conserved Plasmodium protein, unknown function
PFIT_1005200	4393,5216	4312,2616	2928,13531	3018,197622	4352,891606	2973,166466	0,683032507	-0,549973854	0,0065862	0,0139215	DnaJ protein, putative
PFIT_0930700	3811,8781	4268,9078	6232,110626	5631,830224	4040,392942	5931,970425	1,468166713	0,554015798	0,0065932	0,013931	diacylglycerol kinase, putative
PFIT_1248800	1525,5563	1631,0677	1083,102559	1053,29483	1578,31198	1068,198695	0,676798192	-0,56320238	0,0066051	0,013951	conserved Plasmodium protein, unknown function
PFIT_0907200	867,43372	1135,8705	676,5120087	526,6474149	1001,652119	601,5797118	0,60058747	-0,735553719	0,0066271	0,0139922	conserved Plasmodium protein, unknown function
PFIT_0913000	2494,6267	2757,304	2043,202935	1593,30893	2625,965339	1818,255933	0,692414293	-0,53029259	0,0066445	0,0140236	26S proteasome regulatory subunit, putative
PFIT_1471300	648,05953	630,07576	419,1185508	417,9317049	639,0676444	418,5251279	0,654899574	-0,610654403	0,0066674	0,0140667	conserved Plasmodium protein, unknown function
PFIT_0411500	1323,2893	1022,1871	758,5134643	767,2477568	1172,738192	762,8806105	0,650512293	-0,620351774	0,006722	0,0141767	BC_ITvar27 - erythrocyte membrane protein 1,, putative (VAR)
PFIT_0533500	135,85099	157,03723	234,6152757	247,7292409	146,4441094	241,1722583	1,646855304	0,719713802	0,0067621	0,0142559	apicoplast TIC22 protein (TIC22)
PFIT_1238000	3562,3148	2635,9133	1373,524381	1893,613802	3099,114037	1633,569091	0,527108416	-0,923828368	0,0067665	0,0142598	cell cycle control protein, putative
PFIT_1327000	3187,9699	3186,9887	2376,903303	2024,607321	3187,479281	2200,755312	0,690437527	-0,534417214	0,0068395	0,0144083	ribose-phosphate pyrophosphokinase, putative
PFIT_1134300	492,08247	540,47783	313,200004	351,989389	516,2801495	332,5946965	0,644213605	-0,634388965	0,0068502	0,0144255	conserved Plasmodium protein, unknown function
PFIT_1237800	710,45035	989,43089	510,2312793	433,9717277	849,940624	472,1015035	0,555452334	-0,848264982	0,0068648	0,0144508	calcyclin binding protein, putative
PFIT_1129600	177,10944	159,92749	252,8378214	357,3360633	168,5184615	305,0869423	1,810406644	0,856313785	0,0068689	0,0144542	GPI8p transamidase
PFIT_1310000	4386,4775	3830,5523	2854,106219	2779,379505	4108,514895	2816,742862	0,685586625	-0,54458913	0,0068745	0,0144601	conserved Plasmodium protein, unknown function
PFIT_0618600	222,3931	188,83004	312,0610949	440,2095143	205,6115717	376,1353046	1,82934891	0,871330265	0,0068769	0,0144601	conserved Plasmodium protein, unknown function
PFIT_1303600	1093,852	1163,8097	707,2625545	797,5455776	1128,830846	752,4040661	0,666533936	-0,585249765	0,0069191	0,0145433	conserved Plasmodium protein, unknown function
PFIT_0917800	820,13745	767,84462	572,8712801	489,2206951	793,991033	531,0459876	0,668831215	-0,580285913	0,0069225	0,0145433	XPA binding protein 1, putative
PFIT_1323600	2502,6771	2704,316	1986,25748	1615,58674	2603,496542	1800,92211	0,691732092	-0,531714705	0,0069242	0,0145433	MSF1-like protein, putative
PFIT_0932900	176,10313	115,61023	220,9483665	253,9670275	145,856682	237,457697	1,628020696	0,70311904	0,00693	0,0145502	peptide release factor, putative
PFIT_1126900	8907,7996	7228,5297	5203,675703	5810,052699	8068,164659	5506,864201	0,682542367	-0,551009495	0,006958	0,0145982	threonine--tRNA ligase, putative (ThrRS)
PFIT_1118700	700,38732	790,96666	1154,853833	1079,137089	745,6769909	1116,995461	1,49796155	0,583000593	0,0069575	0,0145982	conserved Plasmodium protein, unknown function
PFIT_1345000	2261,1642	2137,8259	1435,025473	1545,188862	2199,495042	1490,107168	0,677476939	-0,561756257	0,0069607	0,0145984	conserved Plasmodium protein, unknown function
PFIT_0927300	505,16442	491,34348	775,5971009	734,2765988	498,2539489	754,9368498	1,51516481	0,599474729	0,0069851	0,0146441	zinc finger protein, putative
PFIT_1103700	1509,4554	1678,2752	1204,965834	983,7880644	1593,865307	1094,376949	0,686618213	-0,542419968	0,0070239	0,01472	Plasmodium exported protein (hyp11), unknown function
PFIT_1471400	859,38329	956,67466	710,6792819	524,8651902	908,0289756	617,772236	0,680344188	-0,555663301	0,0070627	0,0147958	conserved Plasmodium protein, unknown function
PFIT_0605000	8695,4696	10329,774	7211,572456	5193,402934	9512,621851	6202,487695	0,652027148	-0,616996061	0,0070993	0,0148669	RNA binding protein, putative
PFIT_0805900	5476,3043	4227,4808	2595,573852	3240,975716	4851,892534	2918,274784	0,601471439	-0,733431863	0,0071845	0,0150398	conserved Plasmodium protein, unknown function
PFIT_0512400	597,74435	596,35611	387,2290959	394,7627831	597,0502286	390,9959395	0,654879474	-0,610698682	0,0072617	0,0151958	PfMAT1 (MAT1)
PFIT_0820400	4619,9399	5263,1558	3727,649502	3154,537815	4941,547838	3441,093659	0,696359475	-0,522095848	0,0072769	0,015222	protein kinase c inhibitor-like protein, putative
PFIT_0614200	21,132376	24,085465	53,52872796	53,46674263	22,60892038	53,4977353	2,366222464	1,242585717	0,0073464	0,0153617	conserved Plasmodium protein, unknown function
PFIT_1412900	3396,2747	3749,6252	2589,879306	2352,536676	3572,949934	2471,207991	0,69164361	-0,531899257	0,0073587	0,0153817	p1/s1 nuclease, putative
PFIT_1118900	739,63316	683,06378	525,0370977	427,7339411	711,3484703	476,3855194	0,669693602	-0,57842691	0,0073653	0,0153899	clathrin assembly protein AP19, putative
PFIT_0305800	14,088251	12,524442	34,16727317	62,3778664	13,30634617	48,27256979	3,627785507	1,859089159	0,0074387	0,0155375	conserved Plasmodium protein, unknown function
PFIT_1224300	517,24006	523,1363	362,1730956	320,8004558	520,1881774	341,4867757	0,656467776	-0,607203901	0,0074422	0,015539	cytochrome c heme lyase, putative
PFIT_1004700	2758,2782	2607,0107	1969,173843	1734,104686	2682,644463	1851,639265	0,690229097	-0,534852802	0,0074962	0,0156458	conserved protein, unknown function
PFIT_1445700	3988,9875	3731,3202	2698,075671	2618,979277	3860,153873	2658,527474	0,688710233	-0,538030982	0,0075045	0,0156575	eukaryotic initiation factor 2alpha kinase 1
PFIT_1319200	16911,939	17434,986	12477,88816	11449,90293	17173,46243	11963,89555	0,696650172	-0,521493718	0,0075234	0,0156912	rhoptry protein 2, putative
PFIT_1313300	603,78217	461,47751	269,921458	349,3160519	532,629838	309,6187549	0,581301934	-0,782640386	0,0076415	0,0159316	conserved Plasmodium protein, unknown function
PFIT_1221700	468,93749	667,64908	324,5890951	289,6115226	568,293285	307,1003088	0,540390529	-0,887925705	0,0076559	0,0159557	conserved protein, unknown function
PFIT_0514900	657,11626	582,86825	391,7847323	420,605042	619,9922552	406,1948872	0,655161228	-0,610078113	0,0076723	0,015984	kinase, putative
PFIT_1125600	663,15408	689,80771	513,6480066	396,5450079	676,4808979	455,0965072	0,672741106	-0,571876683	0,0076755	0,0159848	GrpE protein homolog, mitochondrial, putative (MGE1)
PFIT_1135500	3086,3332	3851,7475	2537,489487	1963,120567	3469,040363	2250,305027	0,648682284	-0,624416056	0,0076839	0,0159963	coatomer delta subunit, putative
PFIT_0804700	6441,3495	8843,2193	4791,390607	2992,355363	7642,284356	3891,872985	0,509255192	-0,973539312	0,0077125	0,01605	conserved Plasmodium protein, unknown function
PFIT_1431100	1115,9907	1002,9188	1513,610201	1587,962256	1059,454733	1550,786229	1,463758838	0,549677881	0,0077163	0,016052	ATP-dependent DNA helicase, putative
PFIT_bin09300	102,64297	84,780836	38,72290959	55,24896739	93,71190254	46,98593849	0,501387094	-0,996003234	0,0077508	0,0161177	BC_ITvar16 - erythrocyte membrane protein 1,, putative
PFIT_0318900	1719,7729	1817,0075	1218,632743	1197,655035	1768,390173	1208,143889	0,683188533	-0,549644334	0,0077647	0,0161406	conserved protein, unknown function
PFIT_0518800	501,1392	434,50179	290,4218219	312,7804444	467,8204932	301,6011332	0,644694146	-0,633313212	0,0077763	0,016159	mRNA-binding protein PUF1
PFIT_0814900	285,79023	265,90353	157,1694566	181,786925	275,8468794	169,4781908	0,614392271	-0,702768027	0,0077911	0,0161837	conserved Plasmodium protein, unknown function
PFIT_0934800	1997,5127	1920,0933	1342,773835	1334,886341	1958,802967	1338,830088	0,683490407	-0,548999408	0,007823	0,0162441	conserved Plasmodium protein, unknown function
PFIT_1038300	213,33637	194,61056	133,2523653	113,1712719	203,9734612	123,2118186	0,604058086	-0,727240809	0,0078379	0,016269	rifin, pseudogene
PFIT_1360000	1868,7058	2356,5219	1512,471292	1099,632673	2112,613847	1306,051983	0,618216142	-0,69381677	0,0079008	0,0163934	arginine methyltransferase 5, putative
PFIT_1252400	939,88758	800,60085	642,3447355	534,6674263	870,2442143	588,5060809	0,676253943	-0,564362995	0,0079125	0,0164117	chitinase
PFIT_0605600	15320,973	14694,06	10652,21686	10228,18787	15007,51647	10440,20236	0,695664895	-0,523535575	0,007928	0,0164379	coatomer alpha subunit, putative
PFIT_0318300	5861,7186	5033,8621	3544,285136	3930,696696	5447,790357	3737,490916	0,686056304	-0,543601114	0,0079356	0,0164476	conserved Plasmodium protein, unknown function
PFIT_0925800	8447,9189	8316,2293	6291,333899	5434,003276	8382,074077	5862,668588	0,699429346	-0,515749767	0,0079967	0,0165681	histone deacetylase
PFIT_1402900	357,23778	346,83069	219,8094574	227,2336562	352,0342387	223,5215568	0,634942662	-0,655301777	0,0080851	0,0167452	conserved Plasmodium protein, unknown function
PFIT_1100200	146,92033	211,95209	103,6407286	77,52677682	179,4362092	90,58375271	0,504824267	-0,986146833	0,0081789	0,0169332	rifin (RIF)
PFIT_0807100	986,17755	749,53966	462,3970969	570,3119214	867,8586048	516,3545091	0,594975387	-0,749098107	0,0081995	0,0169697	conserved Plasmodium protein, unknown function
PFIT_1251300	2462,425	2308,3509	1692,418931	1595,982268	2385,387949	1644,200599	0,689280165	-0,536837595	0,0082241	0,017002	conserved Plasmodium protein, unknown function
PFIT_0212200	988,19015	1250,5173	1710,641477	1588,853369	1119,353742	1649,747423	1,473839199	0,55957913	0,0082216	0,017002	multidrug efflux pump, putative
PFIT_0629000	410,57188	384,40402	809,764374	588,134169	397,4879471	698,9492715	1,758416266	0,814276637	0,0082241	0,017002	conserved Plasmodium protein, unknown function
PFIT_0722600	410,57188	409,4529	287,0050946	246,8381285	410,0123888	266,9216115	0,65100865	-0,619251383	0,0082457	0,0170402	mitochondrial import inner membrane translocase subunit tim14, putative (PAM18)
PFIT_1126100	179,12204	185,93979	116,1687288	101,586811	182,5309161	108,8777699	0,596489473	-0,745431419	0,0082519	0,0170469	conserved Plasmodium protein, unknown function
PFIT_1408300	5801,3404	5024,228	3672,981865	3801,485401	5412,784156	3737,233633	0,690445716	-0,534400103	0,0082993	0,017136	pre-mRNA splicing factor, putative
PFIT_0815100	101,63667	147,40304	201,5869117	212,0847458	124,519855	206,8358287	1,661067055	0,732110314	0,0083011	0,017136	trypsin-like serine protease, putative
PFIT_1365100	689,31798	837,21076	1753,920023	1230,626193	763,2643672	1492,273108	1,955119578	0,967256848	0,0083044	0,0171366	conserved Plasmodium protein, unknown function
PFIT_0531700	10616,503	8427,9858	6251,47208	6764,434055	9522,244503	6507,953068	0,683447381	-0,549097827	0,0083118	0,0171456	Hsc70-interacting protein (HIP)
PFIT_1464600	2633,4966	2257,2898	1626,362203	1720,738	2445,393164	1673,550102	0,68436852	-0,547154695	0,0083158	0,0171476	conserved Plasmodium protein, unknown function
PFIT_1114000	933,84976	999,06508	661,7061903	644,2742487	966,4574185	652,9902195	0,675653378	-0,565644786	0,0083205	0,017151	conserved Plasmodium protein, unknown function
PFIT_1003300	986,17755	829,50341	488,5920063	669,2253953	907,8404764	578,9087008	0,63767668	-0,649102973	0,0083368	0,0171783	conserved Plasmodium protein, unknown function
PFIT_0830800	20319,283	24177,953	17362,66931	13634,91048	22248,61781	15498,7899	0,696618101	-0,521560134	0,0083456	0,0171902	protein disulfide isomerase
PFIT_1133400	2534,8788	3537,6731	1874,644388	1456,968737	3036,275941	1665,806562	0,548634773	-0,86608203	0,0083615	0,0172167	pre-mRNA splicing factor, putative
PFIT_0212800	74,466468	65,512464	117,3076379	132,7757442	69,98946597	125,041691	1,786578727	0,837199489	0,0083692	0,0172262	protein kinase 7
PFIT_1213900	1393,7305	1379,6154	914,5440118	967,7480417	1386,672967	941,1460267	0,678707993	-0,559137091	0,0083797	0,0172416	geranylgeranyltransferase type2 beta subunit, putative
PFIT_1457000	52,327788	64,549046	95,66836487	119,4090585	58,43841689	107,5387117	1,840205766	0,879867093	0,0084087	0,017295	LCCL domain-containing protein
PFIT_0907090	79,497986	77,073487	31,88945496	43,66450648	78,28573657	37,77698072	0,482552536	-1,051242076	0,0084384	0,0173496	conserved Plasmodium protein, unknown function
PFIT_0834300	79,497986	94,415022	148,0581837	154,1624413	86,9565039	151,1103125	1,73776895	0,797236277	0,0084714	0,0174112	Plasmodium exported protein, unknown function
PFIT_0407600	402,52145	335,26967	525,0370977	584,5697195	368,8955585	554,8034086	1,503957952	0,588764232	0,0085091	0,0174823	conserved Plasmodium protein, unknown function
PFIT_1126200	1278,0056	1418,1522	908,8494662	927,6479847	1348,078881	918,2487255	0,681153558	-0,553948023	0,0085138	0,0174855	DNA-directed RNA polymerase (mtRNAP)
PFIT_0418900	14419,325	11164,095	17866,06714	18634,94203	12791,70959	18250,50459	1,426744757	0,512727261	0,0085205	0,0174931	transcription factor with AP2 domain(s)
PFIT_0719100	143,90142	148,36646	215,2538209	255,7492523	146,1339402	235,5015366	1,611545793	0,688445184	0,0085767	0,017602	conserved Plasmodium protein, unknown function
PFIT_1345200	315,97934	351,64779	165,1418203	249,5114656	333,813561	207,326643	0,621085142	-0,68713704	0,0086317	0,0177085	dynactin subunit 2, putative
PFIT_0923600	160,00228	127,17125	203,8647299	270,8981627	143,5867647	237,3814463	1,653226513	0,725284406	0,0087331	0,0179099	cyclin-dependent kinase, putative
PFIT_1306200	693,34319	566,49013	429,3687328	408,1294688	629,9166623	418,7491008	0,664769049	-0,589074881	0,0087532	0,0179446	PNAS-3 related protein, putative
PFIT_1211000	9969,4499	12970,505	7938,196466	5720,050349	11469,97722	6829,123407	0,595391192	-0,748090216	0,0088074	0,0180394	heat shock protein DNAJ homologue Pfj4
PFIT_0815500	1201,5265	1256,2978	993,12874	679,0276314	1228,912182	836,0781857	0,680340059	-0,555672057	0,008809	0,0180394	ubiquitin conjugating enzyme, putative
PFIT_1125750	550,44808	569,38039	871,2654657	1289,43961	559,9142333	1080,352538	1,929496472	0,948224406	0,0088074	0,0180394	hypothetical protein
PFIT_1318200	97,611451	115,61023	187,9200024	173,7669136	106,610841	180,843458	1,696295202	0,76238726	0,0088277	0,0180712	conserved Plasmodium protein, unknown function
PFIT_0411100	298,87217	583,83167	123,0021834	84,65567584	441,3519206	103,8289296	0,235252017	-2,087721001	0,0088584	0,0181275	Plasmodium RNA of unknown function RUF6-14
PFIT_0617700	111,6997	88,63451	143,5025473	190,6980487	100,167106	167,100298	1,668215292	0,738305488	0,0088755	0,0181559	conserved Plasmodium protein, unknown function
PFIT_0713800	4,0252145	7,7073487	22,77818211	23,16892181	5,866281603	22,97355196	3,9162034	1,969455698	0,0088959	0,0181911	conserved Plasmodium protein, unknown function
PFIT_1457900	3606,5922	3956,7602	2827,911309	2467,490172	3781,676161	2647,700741	0,700139469	-0,514285756	0,0089372	0,0182689	conserved Plasmodium protein, unknown function
PFIT_1451000	588,68762	627,1855	438,4800056	376,9405356	607,9365598	407,7102706	0,67064608	-0,576376481	0,0089742	0,018338	conserved Plasmodium protein, unknown function
PFIT_1114400	1679,5207	1931,6543	1346,190563	1162,01054	1805,587507	1254,100551	0,694566476	-0,525815317	0,0089872	0,0183578	Rpr2, RNAse P, putative
PFIT_0926000	22281,575	24580,662	17499,33841	15533,87096	23431,11833	16516,60468	0,7049004	-0,504508671	0,0090186	0,0184152	conserved Plasmodium protein, unknown function
PFIT_1021400	1249,8291	1951,8861	2776,660399	2463,034611	1600,85758	2619,847505	1,636527532	0,710637874	0,0090614	0,018496	conserved Plasmodium protein (10b antigen), unknown function
PFIT_1148400.1	394,47102	273,61088	115,0298197	184,4602621	334,0409491	149,7450409	0,448283485	-1,157516743	0,009185	0,0187414	merozoite adhesive erythrocytic binding protein
PFIT_0723300	4258,6769	4115,7242	2777,799308	3012,850947	4187,200567	2895,325128	0,691470371	-0,532260661	0,009201	0,0187675	conserved Plasmodium membrane protein, unknown function
PFIT_0113700	401,51514	419,08709	248,282185	280,7003988	410,3011154	264,4912919	0,64462728	-0,633462852	0,0092242	0,0188012	Plasmodium exported protein, unknown function, pseudogene
PFIT_0715900	408,55927	373,80641	583,121462	592,5897308	391,1828412	587,8555964	1,502764269	0,587618719	0,0092237	0,0188012	conserved Plasmodium protein, unknown function
PFIT_1144000	2133,3637	2364,2292	1669,640749	1464,988748	2248,796447	1567,314748	0,696957144	-0,520858148	0,0092287	0,0188036	DNA-directed RNA polymerase I, putative
PFIT_0625600	4044,3342	3592,5879	2665,047307	2637,692637	3818,461084	2651,369972	0,694355635	-0,526253323	0,00925	0,0188333	conserved protein, unknown function
PFIT_1009300	3183,9446	3188,9155	2288,068393	2149,363054	3186,430093	2218,715723	0,696301397	-0,522216178	0,0092474	0,0188333	conserved Plasmodium membrane protein, unknown function
PFIT_0302800	10420,274	12379,929	8671,65393	7442,570574	11400,10143	8057,112252	0,706757944	-0,5007119	0,009261	0,0188356	exportin 1, putative
PFIT_0105100	37,233234	26,975721	55,80654617	76,63566444	32,10447721	66,22110531	2,062675086	1,044516585	0,0092611	0,0188356	conserved Plasmodium protein, unknown function
PFIT_0415000	436,73577	301,55002	863,293102	1565,684447	369,1428946	1214,488774	3,290023435	1,718097861	0,0092593	0,0188356	histone acetyltransferase, putative
PFIT_1354800	13455,286	14839,537	10139,70777	9719,362698	14147,41112	9929,535232	0,701862351	-0,510739977	0,0092739	0,0188549	protein phosphatase, putative
PFIT_1242500	29,182805	34,683069	55,80654617	76,63566444	31,93293711	66,22110531	2,073755542	1,052245837	0,0092913	0,0188835	conserved Plasmodium membrane protein, unknown function
PFIT_1407300	559,50481	609,84397	977,1840126	778,8322177	584,67439	878,0081151	1,501704419	0,586600875	0,0093067	0,0189079	glycerophosphodiester phosphodiesterase, putative (GDPD)
PFIT_1125400	218,36788	199,42765	126,4189107	127,4290699	208,8977668	126,9239903	0,607589024	-0,718832286	0,009512	0,0193111	pyruvate dehydrogenase E1 alpha subunit
PFIT_1404900	580,63719	550,11202	397,4792778	357,3360633	565,3746017	377,4076705	0,667535594	-0,583083329	0,009512	0,0193111	phosphatase, putative
PFIT_1131900	179,12204	123,31758	347,3672772	625,5608888	151,2198118	486,464083	3,216933529	1,685686126	0,0095401	0,0193613	serpentine receptor, putative
PFIT_1326800	263,65155	288,06216	593,371644	420,605042	275,8568534	506,988343	1,837867491	0,878032753	0,0096189	0,0195141	nicotinic acid mononucleotide adenylyltransferase
PFIT_0607250	520,25897	520,24604	337,1170952	349,3160519	520,252505	343,2165736	0,659711525	-0,600092786	0,0096895	0,0196502	sorting assembly machinery 50 kDa subunit, putative (SAM50)
PFIT_0527000	804,03659	768,80804	610,4552806	467,833998	786,4223134	539,1446393	0,685566305	-0,544631892	0,0097712	0,0198088	G10 protein, putative
PFIT_1118500	9939,2608	8205,4361	6346,001536	6334,026777	9072,348489	6340,014157	0,698828331	-0,516989997	0,0097932	0,0198464	GTP-binding nuclear protein ran/tc4
PFIT_1113100	98,617755	79,963743	135,5301836	166,6380145	89,29074882	151,084099	1,6920465	0,758769217	0,0098085	0,0198702	conserved Plasmodium protein, unknown function
PFIT_0803000	81,510593	100,19553	43,27854601	50,7934055	90,85306329	47,03597576	0,517714803	-0,949770525	0,0098737	0,0199943	acyl-CoA synthetase, pseudogene
PFIT_1115300	25,15759	40,463581	75,16800097	63,26897878	32,81058565	69,21848987	2,109638963	1,076996121	0,0098768	0,0199943	conserved Plasmodium protein, unknown function
PFIT_0413600	2598,2759	2571,3642	1763,031295	1818,760362	2584,820081	1790,895829	0,692851252	-0,529382442	0,0098869	0,0200075	arsenical pump-driving ATPase, putative
PFIT_1210600	273,71458	216,76918	334,839277	410,8028059	245,2418836	372,8210415	1,520217656	0,604277895	0,0098974	0,0200217	kinesin-like protein, putative
PFIT_1115500	12922,951	13990,765	10327,62777	8757,852443	13456,85792	9542,740106	0,709135829	-0,495866104	0,0099014	0,0200225	serine/threonine protein kinase, putative
PFIT_1365800	734,60164	690,77113	464,6749151	490,1118075	712,6863856	477,3933613	0,669850541	-0,578088861	0,0099134	0,020037	phosphatidylserine synthase I, putative
PFIT_0504500	13,081947	3,8536744	25,05600032	29,40670845	8,467810697	27,23135438	3,215867165	1,685207816	0,0099157	0,020037	conserved Plasmodium protein, unknown function
PFIT_1351400	5003,3416	4797,8246	3596,674955	3284,640222	4900,583087	3440,657589	0,702091471	-0,510269092	0,0099266	0,020052	conserved Plasmodium protein, unknown function
PFIT_1459400	166,0401	146,43963	243,7265486	254,8581399	156,2398614	249,2923442	1,595574535	0,674076004	0,0099635	0,0201174	conserved Plasmodium protein, unknown function
PFIT_1309200	5,0315181	6,7439301	29,61163674	16,93113517	5,887724116	23,27138596	3,952526562	1,982775158	0,0099661	0,0201174	mitochondrial ATP synthase delta subunit, putative
PFIT_0933300	2723,0576	2528,0104	1696,974567	1921,238285	2625,533987	1809,106426	0,689043233	-0,53733359	0,0100192	0,020214	conserved Plasmodium protein, unknown function
PFIT_1013700	43,271056	43,353837	78,58472828	89,11123772	43,3124461	83,847983	1,935886577	0,952994428	0,0100211	0,020214	male gamete fusion factor HAP2, putative
PFIT_1350500	280,75871	309,25737	182,2254569	191,5891611	295,0080387	186,907309	0,633566834	-0,65843128	0,0100346	0,020234	conserved Plasmodium membrane protein, unknown function
PFIT_1471700	780,89161	752,42992	571,732371	474,9628971	766,6607637	523,347634	0,682632605	-0,550818771	0,0100685	0,0202951	RAP protein, putative
PFIT_0323200	2630,4777	2537,6446	1747,086568	1833,01816	2584,061114	1790,052364	0,692728339	-0,5296384	0,0101171	0,0203857	conserved Plasmodium protein, unknown function
PFIT_1143700	216,35528	217,7326	321,1723678	350,2071642	217,0439398	335,689766	1,546644271	0,629141414	0,0101207	0,0203857	alpha/beta hydrolase, putative
PFIT_0606900	418,6223	412,34316	268,7825489	273,5714998	415,4827311	271,1770244	0,652679411	-0,615553564	0,0101549	0,0204473	conserved protein, unknown function
PF_apicoplast_genon	26,163894	30,829395	55,80654617	65,94231591	28,4966445	60,87443104	2,136196458	1,095044332	0,0101595	0,0204493	apicoplast ribosomal protein S7
PFIT_1442800	268,68307	366,09906	911,1272844	589,0252813	317,3910654	750,0762829	2,363255821	1,240775809	0,0102003	0,0205242	acid cluster protein 33 homologue, putative
PFIT_0710000	2077,0107	1991,3862	1563,722202	1288,548497	2034,198448	1426,13535	0,701079755	-0,51234952	0,0102328	0,0205749	conserved Plasmodium membrane protein, unknown function
PFIT_1148600	309,94151	242,78149	420,2574599	421,4961544	276,3614997	420,8768072	1,522921274	0,606841365	0,01023	0,0205749	serine/threonine protein kinase, putative
PFIT_1105200	5129,1295	5413,4491	3827,873504	3585,836206	5271,289303	3706,854855	0,703215976	-0,507960247	0,0102696	0,0206348	conserved protein, unknown function
PFIT_1123900	16433,944	17335,754	12745,5318	11196,82702	16884,84926	11971,17941	0,708989416	-0,496164005	0,0102699	0,0206348	conserved Plasmodium protein, unknown function
PFIT_1120200	5818,4475	5713,0723	3983,904051	4079,512463	5765,759883	4031,708257	0,699250114	-0,516119511	0,0102814	0,0206505	ubiquitin-protein ligase, putative
PFIT_0828000	97,611451	96,341859	151,474911	173,7669136	96,97665505	162,6209123	1,676907831	0,745803395	0,0102967	0,0206739	conserved Plasmodium protein, unknown function
PFIT_1214800	27,170198	11,561023	38,72290959	54,35785501	19,36561039	46,5403823	2,403248923	1,264986087	0,0103136	0,0207004	peptidyl-prolyl cis-trans isomerase
PFIT_1006500	645,04062	605,02688	413,4240053	422,3872668	625,0337473	417,9056361	0,668612915	-0,580756871	0,0103195	0,020705	PPPDE peptidase, putative
PFIT_0502200	105,66188	111,75656	228,9207302	163,073565	108,7092183	195,9971476	1,802948736	0,850358376	0,0103639	0,0207866	HCNGP-like protein
PFIT_0402500	6206,8807	6810,406	14713,56673	10190,76115	6508,64337	12452,16394	1,913173488	0,935967705	0,0104452	0,0209424	alpha/beta hydrolase, putative
PF_apicoplast_genon	2,0126072	0	11,38909106	10,69334853	1,006303617	11,04121979	10,97205615	3,455762005	0,01046	0,0209645	unspecified product
PFIT_1003900	7543,2519	5879,7437	3807,37314	4681,013317	6711,497792	4244,193229	0,632376462	-0,661144425	0,0105839	0,0212054	zinc finger, C3HC4 type, putative
PFIT_0412400	2878,0283	3277,5501	2191,261119	2120,847458	3077,789198	2156,054288	0,700520455	-0,513500918	0,0106289	0,0212879	ubiquitin carboxyl-terminal hydrolase 13, putative (USP13)
PFIT_0209700	20,126072	24,085465	46,69527333	54,35785501	22,10576857	50,52656417	2,285673262	1,192619184	0,0106359	0,0212944	50S ribosomal protein L33, putative
PFIT_1474000	436,73577	581,90483	345,089459	288,7204102	509,3202998	316,9049346	0,622211474	-0,684523095	0,010652	0,0213191	dihydroorotase, putative
PFIT_1408800	29274,379	27214,648	20624,50499	19331,79191	28244,51346	19978,14845	0,707328469	-0,499547767	0,0107584	0,0215184	plasmepsin IV
PFIT_1206700	8575,7194	8988,6955	6621,61754	5834,112734	8782,207446	6227,865137	0,709145756	-0,49584591	0,0107592	0,0215184	small subunit rRNA processing factor, putative
PFIT_0721900	1040,5179	870,93041	653,7338266	649,620923	955,7241738	651,6773748	0,681867627	-0,552436402	0,0107656	0,0215235	metallo-hydrolase/oxidoreductase, putative
PFIT_0411400	339,12432	442,20913	576,2880074	591,6986185	390,6667264	583,9933129	1,4948632	0,580013465	0,0108089	0,0216025	B_ITvar13 - erythrocyte membrane protein 1, PfEMP1, putative (IT4VAR13)
PFIT_0621200	409,56557	581,90483	304,0887312	230,7981057	495,7352009	267,4434184	0,539488457	-0,890336004	0,0108283	0,0216337	vacuolar transporter chaperone, putative
PFIT_1443700	310,94782	347,79411	202,7258208	220,1047572	329,3709648	211,415289	0,641875914	-0,63963367	0,0108361	0,0216415	protein prenyltransferase alpha subunit, putative
PFIT_1419900	5365,6109	5364,3147	3969,098233	3607,222903	5364,962804	3788,160568	0,706092606	-0,502070685	0,0108568	0,0216752	DEAD/DEAH box ATP-dependent RNA helicase, putative
PFIT_0210100	1892,8571	2086,7647	1388,3302	1382,115297	1989,810887	1385,222748	0,69615799	-0,522513338	0,0109478	0,0218492	conserved Plasmodium protein, unknown function
PFIT_1410800	110,6934	93,451603	153,7527292	183,5691497	102,0725007	168,6609395	1,652364137	0,724531653	0,0109579	0,0218616	RNA binding protein Bruno, putative
PFIT_1108200	705,41884	920,06476	536,4261887	546,2518872	812,7417956	541,339038	0,666065214	-0,586264657	0,01099	0,0219179	Rad51 homolog
PFIT_1216000	1588,9534	1320,8469	979,4618308	1022,997009	1454,900151	1001,22942	0,688177412	-0,539147556	0,0110158	0,0219616	signal recognition particle SRP19
PFIT_0419500	9,0567326	9,6341859	26,19490943	31,1889332	9,345459239	28,69192131	3,070145681	1,618307114	0,0110544	0,0220308	erythrocyte membrane protein 1, PfEMP1, putative
PFIT_0524500	958,00104	979,79671	777,8749191	573,8763709	968,8988759	675,875645	0,697570884	-0,519588271	0,0111247	0,0221632	S-adenosylmethionine-dependent methyltransferase,, putative
PFIT_bin00200	80,504289	144,51279	36,44509138	34,75338271	112,5085391	35,59923704	0,316413646	-1,660116275	0,0111464	0,0221985	erythrocyte membrane protein 1 (PfEMP1)-like, putative
PFIT_1451300	16102,87	16110,286	12038,26925	10843,05541	16106,57809	11440,66233	0,710309928	-0,493479445	0,0111741	0,0222459	signal recognition particle SRP54
PFIT_1365500	197,23551	154,14697	248,282185	332,3849167	175,6912419	290,3335509	1,652521479	0,724669024	0,0111857	0,0222611	nucleoside diphosphate kinase
PFIT_1242400	1428,9511	1556,8844	1120,68656	967,7480417	1492,917791	1044,217301	0,699447289	-0,515712757	0,0112366	0,0223466	queuine tRNA-ribosyltransferase, putative
PFIT_0727300.2	60,378217	36,609906	107,0574559	188,915824	48,49406177	147,9866399	3,051644563	1,609586936	0,0112336	0,0223466	conserved Plasmodium protein, unknown function
PFIT_1312500	33,208019	31,792814	70,61236454	64,16009116	32,50041646	67,38622785	2,073395827	1,051995564	0,0112518	0,022369	peptidyl-prolyl cis-trans isomerase, putative
PFIT_0525500	1865,6869	2304,4973	1579,666929	1222,606182	2085,092089	1401,136555	0,671978261	-0,573513534	0,0112848	0,0224267	conserved Plasmodium protein, unknown function, putative
PFIT_0909000	1045,5495	869,96699	610,4552806	688,8298676	957,7582236	649,6425741	0,678294958	-0,560015326	0,0112901	0,0224293	transporter, putative
PFIT_1211200	2967,5894	2925,9023	1901,978206	2187,680886	2946,745816	2044,829546	0,693928039	-0,527142033	0,0113141	0,022469	lysine-specific histone demethylase 1, putative
PFIT_0415400	4002,0695	4053,102	2879,162219	2788,290628	4027,585752	2833,726424	0,703579414	-0,507214823	0,0113468	0,0225262	small GTP-binding protein sar1
PFIT_1407500	156,98336	133,91518	214,1149118	246,8381285	145,4492743	230,4765202	1,5845835	0,664103685	0,0113988	0,0226176	conserved Plasmodium protein, unknown function
PFIT_1332400	103,64927	112,71998	154,8916384	219,2136448	108,1846239	187,0526416	1,729013189	0,789948874	0,0114009	0,0226176	conserved Plasmodium protein, unknown function
PFIT_1124000	6844,8772	5860,4753	4059,072052	4786,164578	6352,676251	4422,618315	0,696181915	-0,522463759	0,0114232	0,0226539	calcium-dependent protein kinase 7
PFIT_1325000	348,18105	280,35481	449,8690967	620,2142145	314,267931	535,0416556	1,702501601	0,767656155	0,0114781	0,0227547	conserved Plasmodium protein, unknown function
PFIT_0507700	1533,6067	2044,3743	1220,910561	1011,412548	1788,990483	1116,161555	0,623905809	-0,680599853	0,0114861	0,0227627	nuclear protein localization protein 4, putative
PFIT_0923500	1028,4423	896,94271	599,0661895	705,7610028	962,6925031	652,4135961	0,677696766	-0,561288208	0,0115148	0,0228115	conserved Plasmodium protein, unknown function
PFIT_0535300	1682,5396	1671,5313	2468,016032	2343,625552	1677,035453	2405,820792	1,43456764	0,520615993	0,0115363	0,0228462	conserved Plasmodium protein, unknown function
PFIT_1361300	1082,7827	1383,4691	891,7658296	654,0764849	1233,125895	772,9211573	0,626798253	-0,673926935	0,0115785	0,0229218	conserved protein, unknown function
PFIT_0609100	1690,5901	1195,6025	554,6487344	849,2300955	1443,096275	701,9394149	0,486412048	-1,039749131	0,0115848	0,0229262	conserved Plasmodium protein, unknown function
PFIT_1334400	48,302574	28,902558	12,52800016	15,14891041	38,6025657	13,83845529	0,358485376	-1,480013827	0,0115903	0,02929	reticulocyte binding protein 2 homologue b, putative
PFIT_1455000	6140,4647	5682,2429	4502,107694	3896,834426	5911,353765	4199,47106	0,710407671	-0,493280935	0,011635	0,0230094	glucose-6-phosphate dehydrogenase-6-phosphogluconolactonase
PFIT_1002000.2	5195,5456	5198,6067	3659,314956	3660,689646	5197,07615	3660,002301	0,704242577	-0,505855643	0,0116461	0,0230232	RNA binding protein, putative
PFIT_0718300	1574,8652	2008,7278	1310,88438	1059,532617	1791,796463	1185,208498	0,661463801	-0,596265889	0,0117664	0,0232529	small subunit rRNA processing protein, putative
PFIT_1001800	5861,7186	6297,8673	4291,40951	4292,488321	6079,792954	4291,948915	0,70593669	-0,502389289	0,0118203	0,0233513	tryptophan-rich antigen 3
PFIT_1146500	1715,7477	2296,7899	1358,718563	911,6079619	2006,268796	1135,163262	0,565808163	-0,821615103	0,0118402	0,0233753	conserved Plasmodium protein, unknown function
PFIT_0505700	2287,3281	1999,0936	1323,412381	1629,844538	2143,21085	1476,628459	0,688979556	-0,537466921	0,0118407	0,0233753	conserved Plasmodium membrane protein, unknown function
PFIT_0317300	19513,233	20029,473	13942,52527	14060,8622	19771,35299	14001,69374	0,708180859	-0,497810245	0,011953	0,0235886	conserved Plasmodium protein, unknown function
PFIT_bin08950	6,0378217	8,6707673	25,05600032	24,95114656	7,354294516	25,00357344	3,399860229	1,765475437	0,0120127	0,0236982	erythrocyte membrane protein 1, EMP1, exon 2
PFIT_1443500	5648,3822	5846,024	4535,136058	3684,74968	5747,203111	4109,942869	0,715120519	-0,483741696	0,0120346	0,0237332	tRNA binding protein, putative
PFIT_0416400	253,58851	322,74523	432,7854601	438,4272896	288,16687	435,6063748	1,511646273	0,596120586	0,0120699	0,0237946	cdc2-related protein kinase 1
PFIT_0934300	501,1392	670,53934	861,0152838	855,4678821	585,8392708	858,241583	1,464977897	0,550878898	0,0120908	0,0238273	delta tubulin, putative
PFIT_1451400	891,58501	1121,4192	719,7905547	436,6450648	1006,502123	578,2178098	0,574482454	-0,799665264	0,0120994	0,0238359	conserved Plasmodium protein, unknown function
PFIT_1225000	2087,0737	2156,1308	1558,027656	1427,562028	2121,602256	1492,794842	0,70361673	-0,507138308	0,012153	0,0239332	conserved Plasmodium protein, unknown function
PFIT_1112100	509,18963	455,69699	318,8945496	323,4737929	482,4433122	321,1841712	0,665744893	-0,586958639	0,0122326	0,0240816	SNARE protein, putative
PFIT_1414200	192,20399	213,87893	120,7243652	129,2112947	203,0414592	124,9678299	0,615479373	-0,700217588	0,0122649	0,0241344	conserved Plasmodium protein, unknown function
PFIT_0532300	10461,532	11677,597	8628,375384	7242,07029	11069,56458	7935,222837	0,716850494	-0,480255833	0,012268	0,0241344	beta adaptin protein, putative
PFIT_1437300	1166,3059	1188,8585	740,2909186	873,2901297	1177,582218	806,7905241	0,685124582	-0,545561746	0,0122911	0,0241715	conserved Plasmodium membrane protein, unknown function
PFIT_0911700.2	11,06934	3,8536744	20,5003639	28,51559607	7,46150708	24,50797999	3,284588451	1,715712617	0,012315	0,0242101	conserved Plasmodium protein, unknown function
PFIT_1235600	5955,3048	6651,442	4825,55788	4178,425937	6303,373384	4501,991908	0,714219456	-0,48556066	0,0123634	0,0242967	pre-mRNA-splicing factor SYF1, putative
PFIT_1353200	1456,1213	1362,2739	1862,116388	2128,867469	1409,197612	1995,491928	1,416048333	0,501870508	0,0123855	0,0243317	conserved Plasmodium protein, unknown function
PFIT_0414800	1982,4181	1794,8488	1452,10911	1214,58617	1888,633482	1333,34764	0,705985387	-0,502289773	0,0123928	0,0243376	conserved protein, unknown function
PFIT_1357400	104,65558	108,8663	193,6145479	162,1824527	106,7609386	177,8985003	1,666325743	0,736670455	0,0124464	0,0244344	rhomboid protease ROM7
PFIT_1454800	1074,7323	1304,4688	826,8480106	825,1700613	1189,600519	826,009036	0,694358336	-0,526247712	0,0125252	0,0245805	RAP protein, putative
PFIT_0701800	519,25267	690,77113	416,8407326	299,4137587	605,0118985	358,1272457	0,591934219	-0,756491235	0,0125345	0,0245902	centrin, putative
PFIT_0528900	439,75468	474,96537	331,4225497	284,2648483	457,3600233	307,843699	0,67308834	-0,571132229	0,0125832	0,0246773	mitochondrial import receptor subunit tom22, putative
PFIT_1332500	1142,1546	944,15022	1329,106926	1642,320111	1043,152413	1485,713519	1,424253542	0,510205994	0,0126317	0,0247637	ubiquitin-activating enzyme
PFIT_0716500	163,02119	173,41535	246,0043668	278,0270617	168,2182663	262,0157142	1,557593715	0,639318967	0,0126425	0,0247763	cysteine repeat modular protein 2
PFIT_1422200	13781,328	18791,48	10827,60887	7193,059109	16286,40384	9010,333988	0,553242697	-0,854015592	0,0126524	0,0247811	40S ribosomal protein S25, putative
PFIT_0208000.2	552,46069	600,20978	422,5352782	363,5738499	576,3352344	393,054564	0,681989475	-0,55217862	0,0126537	0,0247811	conserved Plasmodium protein, unknown function
PFIT_1439700	3052,1189	2419,1441	1977,146207	1874,009329	2735,631478	1925,577768	0,703887853	-0,506582505	0,0126611	0,0247869	cleavage and polyadenylation specifity factor, putative
PFIT_0319300	245,53808	245,67174	169,6974567	144,3602051	245,6049118	157,0288309	0,639355417	-0,645309947	0,0127016	0,0248319	ubiquitin conjugating enzyme, putative
PFIT_0528600	7596,586	9060,9519	6379,0299	4275,557186	8328,768933	5327,293543	0,639625566	-0,64470049	0,0126965	0,0248319	cytosolic preribosomal GTP-binding protein, putative
PFIT_1246100	489,06356	562,63646	400,8960052	317,2360063	525,8500079	359,0660057	0,682829705	-0,550402274	0,0126962	0,0248319	conserved Plasmodium protein, unknown function
PFIT_1450500	4442,8305	4952,935	3724,232775	3011,959835	4697,882726	3368,096305	0,716939205	-0,480077309	0,0126995	0,0248319	transcription factor IIIb subunit, putative
PFIT_1416200	519,25267	456,66041	273,3381853	367,1382994	487,9565396	320,2382424	0,656284354	-0,607607056	0,0127239	0,0248668	DNA-directed RNA polymerase I/III subunit, putative
PFIT_1109200	5340,4533	6078,2079	4526,026785	3671,382994	5709,330597	4098,70389	0,717895701	-0,478153837	0,0127288	0,0248678	casein kinase 2, alpha subunit
PFIT_1368400	1716,754	1749,5682	1142,325833	1266,270688	1733,161067	1204,29826	0,694856516	-0,525212995	0,0127716	0,0249348	conserved Plasmodium protein, unknown function
PFIT_0726700	4068,4855	3990,4798	2741,354217	2926,413047	4029,482667	2833,883632	0,703287212	-0,507814109	0,0127719	0,0249348	cation transporting ATPase, putative
PFIT_0903300	888,56609	684,99062	525,0370977	536,4496511	786,7783566	530,7433744	0,674578005	-0,567942815	0,0127998	0,0249807	retrieval receptor for endoplasmic reticulum membrane proteins, putative
PFIT_1038100	22,13868	30,829395	63,77890991	51,68451788	26,48403726	57,73171389	2,179868323	1,12424099	0,0128517	0,0250645	phospholipase, putative
PFIT_0500700	5,0315181	3,8536744	15,94472748	21,38669705	4,44259628	18,66571227	4,201532462	2,070915631	0,0128508	0,0250645	stevor
PFIT_1134400	21089,105	15116,038	6982,651726	10961,57335	18102,57131	8972,112539	0,495626416	-1,012675012	0,0128787	0,025105	conserved Plasmodium protein, unknown function
PFIT_1108800	6014,6767	6105,1836	4481,60733	4146,345891	6059,93017	4313,976611	0,711885532	-0,490282815	0,0128813	0,025105	IWS1-like protein, putative
PFIT_0404000	401,51514	403,67239	578,5658256	606,8475289	402,5937667	592,7066772	1,472220204	0,557993475	0,01297	0,0252605	liver specific protein 2, putative (LISP2)
PFIT_1233400	163,02119	125,24442	202,7258208	270,8981627	144,1328015	236,8119917	1,643012481	0,716343439	0,0129693	0,0252605	homocysteine S-methyltransferase, putative
PFIT_0517300	7107,5224	6663,9664	4828,974608	4918,940322	6885,744425	4873,957465	0,707833048	-0,498518973	0,012993	0,0252966	conserved Plasmodium protein, unknown function
PFIT_1360100	21884,085	23001,619	17328,50204	14925,24121	22442,85183	16126,87162	0,718574972	-0,476789408	0,0130945	0,0254852	Sec24 subunit a
PFIT_1448700	76,479075	68,40272	38,72290959	34,75338271	72,44089748	36,73814615	0,50714648	-0,979525592	0,0131202	0,0255265	conserved Plasmodium protein, unknown function
PFIT_1036200	630,95237	711,00292	904,2938298	1025,670346	670,9776445	964,982088	1,438173233	0,524237463	0,013151	0,0255777	DNA polymerase zeta catalytic subunit, putative
PFIT_0310500.1	1111,9655	1260,1515	909,9883753	537,3407635	1186,058508	723,6645694	0,610142387	-0,712782135	0,0133164	0,0258818	SAC3/GNAP family-related protein, putative
PFIT_1120300	671,20451	599,24636	382,6734595	469,6162228	635,2254385	426,1448411	0,67085607	-0,575924821	0,0133165	0,0258818	clathrin coat assembly protein, putative
PFIT_1012900	637,99649	726,41762	1095,63056	902,6968381	682,2070559	999,1636988	1,464604756	0,550511386	0,0133301	0,0258993	conserved Plasmodium protein, unknown function
PFIT_1251200	73,460164	102,12237	52,38981886	42,77339411	87,79126741	47,58160648	0,541985643	-0,88367346	0,0133558	0,0259402	conserved Plasmodium protein, unknown function
PFIT_0629200	655,10365	944,15022	1441,858928	1168,248327	799,6269376	1305,053627	1,632078118	0,706710112	0,0133759	0,0259704	DNA polymerase epsilon, catalytic subunit a, putative
PFIT_0936800	268,68307	315,03788	202,7258208	178,2224754	291,8604727	190,4741481	0,65262057	-0,615683634	0,0133981	0,0260046	Plasmodium exported protein (hyp11), unknown function
PFIT_0807400	2003,5505	2268,8508	1589,917111	1438,255377	2136,200643	1514,086244	0,708775296	-0,496599774	0,0135083	0,0262094	U2 snRNA/tRNA pseudouridine synthase, putative
PFIT_1250800	3059,163	2982,744	2155,954937	2116,391896	3020,953479	2136,173416	0,707118938	-0,499975197	0,0135398	0,0262616	eukaryotic translation initiation factor 2b, subunit 2, putative
PFIT_0315100	4875,541	4408,6035	3519,229136	3108,199972	4642,072252	3313,714554	0,713843812	-0,486319645	0,0136037	0,0263764	translation initiation factor 4E
PFIT_0706400	4712,5198	4549,2626	7187,655365	5979,364051	4630,891216	6583,509708	1,421650693	0,507567031	0,0137061	0,0265658	conserved Plasmodium protein, unknown function
PFIT_1442900	12,075643	20,23179	46,69527333	34,75338271	16,15371692	40,72432802	2,521049999	1,334024731	0,0137478	0,0266375	metalloprotease, putative
PFIT_0421800	4106,7251	4019,3824	2953,191311	2823,044011	4063,053714	2888,117661	0,710824386	-0,492434919	0,0137943	0,0267185	conserved Plasmodium protein, unknown function
PFIT_1431000	14345,864	14889,634	10556,5485	10326,21023	14617,74935	10441,37936	0,714294596	-0,485408889	0,0138322	0,0267827	coatomer beta subunit, putative
PFIT_1237500	186,16617	143,54937	101,3629104	98,02236149	164,8577697	99,69263594	0,604719062	-0,725663037	0,013861	0,0268201	ubiquitin-like protein, putative
PFIT_1323100	2342,6748	3029,9515	1925,895297	1327,757442	2686,313147	1626,82637	0,605598186	-0,72356721	0,0138599	0,0268201	conserved Plasmodium protein, unknown function
PFIT_0111700	7328,9092	9087,9276	6158,081534	4272,883849	8208,418412	5215,482691	0,635382169	-0,654303492	0,0138814	0,026832	Plasmodium exported protein (hyp1), unknown function
PFIT_1021000	2023,6766	1902,7517	1462,359292	1316,172981	1963,214147	1389,266136	0,707648801	-0,498894553	0,0138766	0,026832	RNA helicase, putative
Pf_M7661102200	16,100858	21,195209	50,11200064	100,6956986	18,64803345	75,40384963	4,043528227	2,015614683	0,0138785	0,026832	unspecified product
PFIT_0823800	3215,1401	3144,5983	4029,460415	4823,591298	3179,869171	4426,525857	1,392046534	0,477207439	0,0140427	0,0271344	conserved Plasmodium protein, unknown function
PFIT_0826200	5982,475	6048,3419	4205,991327	4339,717277	6015,408463	4272,854302	0,710318232	-0,49346258	0,0141111	0,0272573	histone acetyltransferase GCN5
PFIT_1433800	970,07669	1065,541	695,8734635	716,4543513	1017,808825	706,1639074	0,693808002	-0,527391615	0,0141286	0,0272819	conserved Plasmodium protein, unknown function
PFIT_1366100	2824,6943	2127,2283	1601,306202	1629,844538	2475,961253	1615,57537	0,652504302	-0,61594068	0,0142109	0,0274313	U1 small nuclear ribonucleoprotein, putative
PFIT_0307200	86,542111	131,02493	178,8087296	177,3313631	108,7835198	178,0700463	1,636921168	0,710984845	0,0143146	0,0276221	EB1 homolog, putative
PFIT_0601700	903,66065	955,71124	740,2909186	570,3119214	929,6859459	655,30142	0,70486321	-0,504584788	0,0144092	0,0277952	JmjC domain containing protein
PFIT_1017200	925,79933	798,67401	1091,074923	1342,01524	862,2366704	1216,545082	1,410917818	0,496633957	0,0144154	0,0277976	conserved Plasmodium protein, unknown function
PFIT_0729000	88,554718	66,475883	112,7520014	155,944666	77,51530059	134,3483337	1,733184709	0,793425414	0,0144534	0,0278613	tRNA pseudouridine synthase D, putative
PFIT_1332900	6511,7907	4738,0926	2769,826945	3595,638442	5624,941672	3182,732693	0,565825013	-0,821572141	0,014594	0,0281132	U3 snoRNA-associated small subunit rRNA processing associated protein, putative
PFIT_1456400.1	11,06934	8,6707673	22,77818211	33,86227033	9,87005356	28,32022622	2,869308262	1,520702971	0,01459	0,0281132	methyltransferase, putative
PFIT_0923200	62,390824	79,000325	43,27854601	30,29782083	70,69557441	36,78818342	0,520374631	-0,942377466	0,0146723	0,0282478	OTU-like cysteine protease, putative
PFIT_1441700	201,26072	228,33021	143,5025473	129,2112947	214,7954649	136,356921	0,63482216	-0,655575605	0,0146739	0,0282478	allantoicase, putative
PFIT_0904800	4797,0493	5063,7281	3807,37314	3284,640222	4930,388732	3546,006681	0,719214422	-0,475506145	0,0147199	0,0283267	ubiquitin specific protease, putative
PFIT_1210000	2457,3934	2446,1198	1970,312753	1552,317761	2451,756619	1761,315257	0,71838911	-0,477162613	0,0147586	0,0283917	general transcription factor 3C polypeptide 5, putative
PFIT_0406100	275,72719	208,09842	159,4472748	149,7068794	241,9128035	154,5770771	0,638978487	-0,646160736	0,014785	0,0284326	mitochondrial carrier protein, putative
PFIT_0926800	909,69847	968,23568	616,1498261	680,8098562	938,9670776	648,4798411	0,690631074	-0,534012847	0,0147908	0,0284342	NAD synthase, putative
PFIT_1447600	252,58221	345,86727	173,114184	192,4802735	299,2247413	182,7972288	0,610902788	-0,71098527	0,0148056	0,028453	pyruvate dehydrogenase E1 beta subunit
PFIT_1203400	37967,835	46437,74	32501,04914	23645,66693	42202,78752	28073,35804	0,665201511	-0,588136649	0,0148147	0,0284608	nucleosome assembly protein
PFIT_1311500	11,06934	6,7439301	29,61163674	24,95114656	8,906634968	27,28119165	3,063041401	1,614964867	0,0148292	0,0284789	mitochondrial ribosomal protein S35 precursor, putative
PFIT_0309600	1448,0709	1560,7381	1089,936014	1033,690358	1504,404512	1061,813186	0,705802979	-0,502662576	0,0149101	0,0286245	SECIS-binding protein 2, putative
PFIT_1405500	1481,2789	1776,5439	1283,550562	955,2724684	1628,911404	1119,411515	0,687214487	-0,541167645	0,0149204	0,0286346	U3 small nucleolar ribonucleoprotein, U3 snoRNP, putative
PFIT_0518600	4185,2167	3141,708	2471,945734	2019,046377	3663,462387	2245,615799	0,612976346	-0,706096067	0,0149456	0,0286732	ATP-dependent RNA helicase DDX23, putative
PFIT_0712500	173,08422	187,86663	116,1687288	98,02236149	180,4754238	113,3595452	0,628116243	-0,670896517	0,0149608	0,0286839	calmodulin, putative
PFIT_0317800	375,35125	366,09906	235,7541848	253,9670275	370,7251571	244,8606062	0,660490936	-0,598389331	0,0149613	0,0286839	proteasome regulatory protein, putative
PFIT_0212900	360,2567	358,39172	232,3374575	242,3825666	359,3242056	237,3600121	0,6605734	-0,59820922	0,0150154	0,0287778	conserved Plasmodium protein, unknown function
PFIT_1013600	24,151287	14,451279	45,55636422	43,66450648	19,30128285	44,61043535	2,311267893	1,208684489	0,0150617	0,0288568	conserved Plasmodium protein, unknown function
PFIT_0904300	1720,792	1464,3963	982,8785581	1226,170631	1592,587723	1104,524595	0,69354082	-0,527947297	0,015097	0,0289063	adapter-related protein, putative
PF_apicoplast_genon	82,516897	84,780836	116,1687288	164,8557898	83,64886636	140,5122593	1,679786773	0,748278113	0,0150978	0,0289063	RNA polymerase beta subunit, putative
PFIT_0413100	413,59079	515,42895	348,5061863	286,9381855	464,5098667	317,7221859	0,683994482	-0,547934408	0,015109	0,0289197	RNA binding protein, putative
PFIT_1345900	31,195412	25,048883	54,66763707	61,48675403	28,12214777	58,07719555	2,065176388	1,046265008	0,0152557	0,0291888	transmission blocking target antigen precursor
PFIT_1302400.2	8,0504289	13,48786	28,47272764	32,08004558	10,76914461	30,27638661	2,811401248	1,491289372	0,0153174	0,029297	SNARE protein, putative
PFIT_1216800.2	96,605147	98,268696	58,08436438	50,7934055	97,43692183	54,43888494	0,558708998	-0,83983104	0,0153408	0,0293218	type IIB DNA topoisomerase, putative
PFIT_0827100	91,573629	71,292976	118,446547	167,5291269	81,4333025	142,9878369	1,755888961	0,812201615	0,0153371	0,0293218	conserved Plasmodium protein, unknown function
PFIT_0727900	531,32831	580,94141	453,285824	321,6915682	556,1348605	387,4886961	0,696753114	-0,521280569	0,0153496	0,0293289	signal recognition particle SRP9
PFIT_0808300	219,37419	216,76918	127,5578198	146,1424299	218,0716859	136,8501248	0,627546507	-0,672205714	0,0153838	0,0293842	acetyltransferase, putative
PFIT_1108500	5254,9175	7035,846	4275,464782	3068,991027	6145,381734	3672,227905	0,597558958	-0,742847032	0,0153942	0,0293873	pescadillo-like protein
PFIT_1111300	462,89966	455,69699	589,9549167	728,9299246	459,298329	659,4424206	1,435760548	0,521815161	0,0153958	0,0293873	vacuolar sorting protein 35, putative
PFIT_1214100	69,43495	105,01263	50,11200064	32,97115796	87,22378807	41,5415793	0,476264334	-1,070165582	0,0154388	0,0294593	conserved Plasmodium protein, unknown function
PFIT_0607900	20694,634	21193,282	15009,6831	15049,10583	20943,95804	15029,39446	0,717600486	-0,478747228	0,0154878	0,0295428	conserved Plasmodium protein, unknown function
PFIT_0101700	2242,0445	2124,338	1507,915656	1576,377795	2183,191228	1542,146726	0,70637272	-0,501498468	0,0154984	0,0295532	nucleoside transporter 4 (NT4)
PFIT_1233900	584,6624	536,62416	757,3745552	847,4478707	560,6432787	802,411213	1,431233091	0,517258649	0,0156294	0,0297929	conserved Plasmodium protein, unknown function, pseudogene
PFIT_1024800	293,84066	279,39139	407,7294598	440,2095143	286,616024	423,9694871	1,479224648	0,564841169	0,0157603	0,0300323	conserved Plasmodium protein, unknown function
PFIT_1234700	29,182805	32,756232	142,3636382	75,74455206	30,96951852	109,0540951	3,521336474	1,816123087	0,01578	0,0300597	PIH1 domain-containing protein, putative
PFIT_1405400	39,245841	27,939139	71,75127365	62,3778664	33,59249012	67,06457003	1,996415561	0,997412054	0,0158209	0,0301275	mitochondrial ribosomal protein L16 precursor, putative
PFIT_1224000	123479,49	181103,43	93147,95902	64693,86747	152291,4593	78920,91324	0,518222845	-0,94835548	0,0158266	0,0301281	polyadenylate-binding protein, putative
PFIT_0204100	181,13465	201,35449	102,5018195	132,7757442	191,2445684	117,6387819	0,615122211	-0,701055024	0,0158591	0,0301798	3'-5' exonuclease, putative
PFIT_1206300	10011,715	10482,958	8042,976553	6817,200798	10247,33619	7430,088451	0,725109268	-0,463172968	0,0159243	0,0302932	eukaryotic translation initiation factor 5, putative
PFIT_1458600	6839,371	9561,8564	7238,906275	6110,357571	9201,613669	6674,631923	0,72537624	-0,463198605	0,0159514	0,0303351	conserved Plasmodium protein, unknown function
PFIT_1104500	2543,9355	2915,3047	1968,034934	1928,367184	2729,620102	1948,201059	0,713726081	-0,486557602	0,0159732	0,0303587	casein kinase II beta chain
PFIT_1029400	2808,5934	2698,5355	2017,008026	1914,109386	2753,564436	1965,558706	0,713823392	-0,486360916	0,0159746	0,0303587	transcription factor, putative
PFIT_1477900	1789,2078	1833,3856	1396,302563	1194,090585	1811,296706	1295,196574	0,715065936	-0,483851817	0,0160462	0,0304846	lysophospholipase, putative
PFIT_1315800	4255,658	5177,4115	9709,200125	6939,092081	4716,534756	8324,146103	1,764885988	0,819574988	0,0161165	0,0306012	choline-phosphate cytidylyltransferase
PFIT_1457800	9,0567326	10,597605	30,75054585	26,73337132	9,827168535	28,74195858	2,924744649	1,548310672	0,0161184	0,0306012	mitochondrial ribosomal protein L28 precursor, putative
PFIT_1121300	3817,9159	3658,1004	2815,383309	2554,819185	3738,008159	2685,101247	0,718324073	-0,477293229	0,016195	0,0307363	tRNA nucleotidyltransferase, putative
PFIT_bin07650	82,516897	46,244092	112,7520014	110,4979348	64,38049452	111,6249681	1,733832101	0,7939642	0,0162711	0,0308703	erythrocyte membrane protein 1, PfEMP1
PFIT_1353000	3057,1504	2994,305	2367,79203	1993,418388	3025,727687	2180,605209	0,720687859	-0,472553553	0,0162945	0,0309043	proteasome subunit, putative
PFIT_0909300	115,72492	74,183232	140,08582	173,7669136	94,9540738	156,9263668	1,652655442	0,724785972	0,016309	0,0309214	conserved Plasmodium membrane protein, unknown function
PFIT_0930000	0	5,7805116	21,63927301	11,5844609	2,890255716	16,61186695	5,74754217	2,522945146	0,0163164	0,0309251	conserved Plasmodium protein, unknown function
PFIT_0834400	10928,457	11701,682	9449,528849	7110,185658	11315,06975	8279,857253	0,731754857	-0,450567678	0,0163424	0,0309639	Plasmodium exported protein (PHIST), unknown function
PFIT_1224000	123,77534	139,6957	187,9200024	224,5603191	131,7355204	206,2401607	1,565563273	0,646680065	0,0164437	0,0311443	DNA polymerase epsilon subunit b, putative
PFIT_1358600	467,93118	728,34446	332,5614588	226,3425438	598,1378188	279,4520013	0,467203364	-1,097877432	0,0164533	0,0311532	conserved Plasmodium protein, unknown function
PFIT_1367900	898,62913	962,45517	747,1243732	574,7674833	930,5421519	660,9459283	0,710280482	-0,493539253	0,0164709	0,031176	conserved Plasmodium protein, unknown function
PFIT_1144800	70,441253	62,622208	110,4741832	118,5179462	66,53173085	114,4960647	1,720924185	0,783183541	0,0165185	0,0312556	mitochondrial large subunit ribosomal protein, putative
PFIT_1459700	400,50884	320,81839	471,5083697	570,3119214	360,6636154	520,9101456	1,444310219	0,530380648	0,0165547	0,0313137	conserved Plasmodium protein, unknown function
PFIT_0606100	35,220627	32,756232	70,61236454	64,16009116	33,98842937	67,38622785	1,98262259	0,987410073	0,0165633	0,0313196	RING zinc finger protein, putative
PFIT_0622700	50534,555	52001,482	40622,60998	34090,3951	51268,01849	37356,50254	0,728651187	-0,456699748	0,0165962	0,0313712	protein kinase, putative
PFIT_1235500	86,542111	77,073487	119,5854561	150,5979917	81,80779923	135,0917239	1,651330621	0,723628998	0,0166198	0,0314054	conserved Plasmodium protein, unknown function
PFIT_1215600	4565,9995	4293,9567	3171,861859	3170,577838	4429,778088	3171,219849	0,715886842	-0,482196531	0,0166688	0,0314873	serpentine receptor, putative (SR10)
PFIT_1235200	776,86639	628,14892	463,536006	501,6962684	702,5076573	482,6161372	0,686990572	-0,541637795	0,0166803	0,031488	mRNA methyltransferase, putative
PFIT_0607700	922,78042	744,72257	1277,856016	1940,842758	833,7514944	1609,349387	1,930250678	0,948788219	0,016754	0,031488	ornithine aminotransferase
PFIT_1472300	520,25897	396,92846	894,0436479	1594,200043	458,5937151	1244,121845	2,712906445	1,439839297	0,0167319	0,0315749	inorganic anion exchanger, inorganic anion antiporter
PFIT_0904000	166,0401	197,50081	120,7243652	107,8245976	181,7704541	114,2744814	0,628676476	-0,669614444	0,0168096	0,0317004	LCCL domain-containing protein
PFIT_1215800	33,208019	30,829395	66,36218259	66,83342829	32,01870716	66,59780544	1,986270249	0,990061927	0,016806	0,0317004	conserved Plasmodium membrane protein, unknown function
PFIT_1476700	3727,3486	4492,4209	3212,862587	2744,626122	4109,884747	2978,744354	0,724775642	-0,464393625	0,0168386	0,0317444	bromodomain protein, putative

[illegible]	[illegible]	494,2865518	536,4496511	352,9318352	515,3681014	1,460248269	0,546213675	0,0169795	0,0319994	TPR domain containing protein
[illegible]	[illegible]	3917,847323	4416,352941	5866,299472	4167,100132	0,710345619	-0,493406955	0,0170018	0,0320308	conserved Plasmodium protein, unknown function
[illegible]	[illegible]	82,0014556	84,65567584	139,5401077	83,32856572	0,597165697	-0,743796799	0,0170294	0,0320677	transcription factor with AP2 domain(s), putative
[illegible]	[illegible]	42,13963691	75,74455206	21,18523501	58,94209448	2,782225189	1,476239194	0,0170327	0,0320677	conserved Plasmodium protein, unknown function
[illegible]	[illegible]	324,5890951	496,3495941	207,7957191	410,4693446	1,975350341	0,982108547	0,0170483	0,0320862	conserved Plasmodium membrane protein, unknown function
[illegible]	[illegible]	44,41745512	58,8134169	94,76109118	51,61543601	0,544690182	-0,876492232	0,0170929	0,0321594	Plasmodium exported protein (PHISTb), unknown function
[illegible]	[illegible]	3096,693858	3765,840906	2484,295437	3431,267382	1,38118305	0,4659048	0,0171449	0,0322466	unspecified product
[illegible]	[illegible]	2359,819667	2726,803874	3586,043837	2543,31177	0,709224953	-0,4956848	0,0171891	0,0323189	translation initiation factor eIF-1A, putative
[illegible]	[illegible]	2105,842936	2054,905142	2897,866796	2080,374039	0,717898435	-0,478148341	0,0172962	0,0325095	conserved Plasmodium protein, unknown function
[illegible]	[illegible]	14811,51292	14340,67149	20154,62403	14576,0922	0,723213302	-0,467506881	0,0173348	0,0325712	calcium-transporting ATPase
[illegible]	[illegible]	211,8370936	154,1624413	348,9809223	182,9997674	0,524383299	-0,931306356	0,0173612	0,0326099	conserved Plasmodium protein, unknown function
[illegible]	[illegible]	3486,200772	3033,346532	4496,053516	3259,773652	0,725029994	-0,463887416	0,0175316	0,0329191	conserved Plasmodium protein, unknown function
[illegible]	[illegible]	44,41745512	41,88228173	80,55565396	43,14986842	0,535652885	-0,900629691	0,017552	0,0329465	erythrocyte membrane protein 1, PfEMP1
[illegible]	[illegible]	4688,888788	3502,071642	5888,366384	4095,480215	0,695520616	-0,523834816	0,0175756	0,0329579	conserved Plasmodium membrane protein, unknown function
[illegible]	[illegible]	831,403647	928,5390971	621,0414438	879,9713721	1,416928582	0,502767043	0,0175678	0,0329579	peptidase family C50, putative
[illegible]	[illegible]	985,1563763	799,3278024	595,8594219	892,2420893	1,497403677	0,582463202	0,0175743	0,0329579	MORN repeat protein, putative
[illegible]	[illegible]	1251,661107	805,565589	1658,359999	1028,613348	0,620259382	-0,689056443	0,0176135	0,033018	conserved Plasmodium protein, unknown function
[illegible]	[illegible]	51,25090975	64,16009116	28,47520199	57,70550045	2,026517687	1,019002767	0,0176876	0,033146	malonyl CoA-acyl carrier protein transacylase precursor
[illegible]	[illegible]	19864,85262	13715,1106	29404,71444	16789,98161	0,570996248	-0,80844683	0,0177062	0,0331697	conserved Plasmodium protein, unknown function
[illegible]	[illegible]	1756,197841	1479,246546	2240,008493	1617,722193	0,72219467	-0,469540323	0,0177288	0,0332011	conserved Plasmodium protein, unknown function
[illegible]	[illegible]	4253,825509	4059,016878	5760,109521	4156,421194	0,721587181	-0,470754385	0,0177637	0,0332555	C-13 antigen
[illegible]	[illegible]	7891,501192	6409,771329	9772,218262	7150,636261	0,731731125	-0,450614468	0,0179232	0,033543	gametocyte associated protein, pseudogene (GAP)
[illegible]	[illegible]	2501,044396	2052,231805	3130,552821	2276,6381	0,727231972	-0,459512468	0,0180124	0,0336987	ES2 protein, putative
[illegible]	[illegible]	153,7527292	168,4202393	100,2957611	161,0864843	1,606114581	0,683574819	0,018045	0,0337485	conserved Plasmodium protein, unknown function
[illegible]	[illegible]	7537,300461	7747,331007	10617,35521	7642,315734	0,719794674	-0,474342667	0,0180556	0,0337572	conserved Plasmodium membrane protein, unknown function
[illegible]	[illegible]	289,2829128	297,631534	433,3174436	293,4572234	0,67723381	-0,562274094	0,018075	0,0337822	conserved Plasmodium protein, unknown function
[illegible]	[illegible]	807,4865558	567,6385843	1298,016527	687,5625701	0,529702478	-0,916745838	0,0180831	0,0337862	conserved Plasmodium protein, unknown function
[illegible]	[illegible]	5358,567342	5024,982695	7158,142569	5191,775018	0,725296398	-0,463357412	0,0181865	0,0339682	GTPase activator, putative
[illegible]	[illegible]	1175,354197	927,6479847	637,3078639	1051,501091	1,649910742	0,722387979	0,0182161	0,0340122	conserved Plasmodium protein, unknown function
[illegible]	[illegible]	1308,606562	1203,892822	1749,394576	1256,249692	0,7181054	-0,477732484	0,018325	0,0342043	conserved Plasmodium protein, unknown function
[illegible]	[illegible]	243,7265486	250,402578	161,1856095	247,0645633	1,532795416	0,616165151	0,0184205	0,0343712	mitochondrial carrier protein, putative
[illegible]	[illegible]	1463,498201	1320,628543	1933,510744	1392,063372	0,719966711	-0,473997892	0,0184428	0,0344014	conserved Plasmodium protein, unknown function
[illegible]	[illegible]	708,4014637	533,7763139	870,7029866	621,0888888	0,713318891	-0,487380914	0,018458	0,0344183	exosome complex component RRP42, putative
[illegible]	[illegible]	15999,39511	9959,96304	23630,01648	12979,67908	0,549287771	-0,864365922	0,018688	0,0348357	60S ribosomal protein L19, putative
[illegible]	[illegible]	15179,38056	12651,12242	18991,42727	13915,25149	0,732712254	-0,44868135	0,0187024	0,0348511	chromatin remodeling protein
[illegible]	[illegible]	13,66690927	12,47557328	33,39950751	13,07124127	0,3913603	-1,353430681	0,0187614	0,0349379	Plasmodium exported protein, unknown function
[illegible]	[illegible]	961,2392851	1025,670346	1662,357273	993,4548156	0,597618112	-0,742704221	0,0187558	0,0349379	conserved Plasmodium protein, unknown function
[illegible]	[illegible]	879,2378295	807,3478138	1184,283263	843,2928216	0,712070202	-0,489908614	0,0187752	0,0349522	ubiquitin fusion degradation protein UFD1, putative
[illegible]	[illegible]	8580,541201	7315,141505	10860,00957	7947,841353	0,731844784	-0,450390394	0,0190249	0,0354054	acetyl-CoA synthetase, putative
[illegible]	[illegible]	201,5869117	185,3513745	122,4658572	193,4691431	1,579780255	0,659723895	0,0190348	0,0354122	conserved Plasmodium protein, unknown function
[illegible]	[illegible]	3072,776767	1974,705028	4119,184332	2523,740897	0,612679767	-0,706794886	0,0191173	0,0355538	conserved Plasmodium protein, unknown function
[illegible]	[illegible]	2,277818211	2,673337132	13,78805547	2,475577671	0,179545091	-2,477581888	0,0191358	0,0355767	stevor
[illegible]	[illegible]	105,9185468	61,48675403	23,81818063	83,70265042	3,51423359	1,81321009	0,0191613	0,0356122	unspecified product
[illegible]	[illegible]	1551,194202	1182,506125	2125,983026	1366,850163	0,642926188	-0,637274978	0,0191724	0,0356212	conserved Plasmodium protein, unknown function, putative
[illegible]	[illegible]	507,9534611	547,1429996	364,718752	527,5482303	1,44645217	0,532518619	0,019182	0,0356273	conserved Plasmodium protein, unknown function
[illegible]	[illegible]	6218,443716	6633,440536	8912,41405	6425,942126	0,721010277	-0,471908271	0,0192648	0,0357693	conserved Plasmodium membrane protein, unknown function
[illegible]	[illegible]	8222,923742	8136,747116	11300,45038	8179,835429	0,723850391	-0,466236549	0,0193451	0,0359066	kelch protein, putative
[illegible]	[illegible]	1154,853833	1348,253027	1768,780622	1251,55343	0,707579795	-0,499035243	0,0195132	0,0362067	phosphatidylglycerophosphate synthase
[illegible]	[illegible]	1537,527292	1522,911053	2132,620231	1530,219173	0,717530084	-0,47888775	0,0196289	0,0364095	RNA polymerase II-associated protein 1, putative
[illegible]	[illegible]	810,9032831	591,6986185	415,9200609	701,3009508	1,686143605	0,753727412	0,0196428	0,0364233	erythrocyte membrane protein 1, PfEMP1
[illegible]	[illegible]	164,0029112	196,9358354	115,3145193	180,4693733	1,56501865	0,646179849	0,0197349	0,0365822	25 kDa ookinete surface antigen precursor
[illegible]	[illegible]	18036,9035	17368,67134	24350,94309	17702,78742	0,726985701	-0,460001107	0,0197597	0,036616	conserved Plasmodium protein, unknown function
[illegible]	[illegible]	4513,496785	5124,787281	6718,44169	4819,142033	0,717300567	-0,479350324	0,0198788	0,0368237	conserved Plasmodium protein, unknown function
[illegible]	[illegible]	1986,25748	1939,060533	2724,534718	1962,659006	0,720364836	-0,473200335	0,0198848	0,0368237	RIO-like serine/threonine kinase (RIO2)
[illegible]	[illegible]	786,9861919	639,8186868	994,6753103	713,4024394	0,717221421	-0,479509518	0,0199867	0,0370003	peptide deformylase
[illegible]	[illegible]	85,41818292	106,0423729	55,17366438	95,7302779	1,735071958	0,794995497	0,020202	0,0373865	translation initiation factor IF-2, putative
[illegible]	[illegible]	198,1701844	210,302521	306,7620444	204,2363527	0,665781039	-0,58688031	0,0203529	0,0376536	conserved Plasmodium protein, unknown function
[illegible]	[illegible]	1034,129468	861,7056688	1312,966961	947,9175683	0,721966048	-0,469997102	0,0205225	0,0379548	conserved Plasmodium protein, unknown function
[illegible]	[illegible]	2002,202208	2651,950435	3505,451276	2327,076321	0,663845005	-0,591081655	0,020559	0,0379947	U3 small nucleolar RNA-associated protein 6, putative
[illegible]	[illegible]	22574,31738	19739,92138	28800,72318	21157,11938	0,734603755	-0,444961825	0,0205642	0,0379947	protein transport protein Sec23
[illegible]	[illegible]	528,453825	667,4431705	422,6983965	597,9484978	1,414598453	0,500392588	0,0205574	0,0379947	GPI-anchored wall transfer protein 1, putative (GWT1)
[illegible]	[illegible]	2488,516396	2739,279448	3637,030721	2613,897922	0,718690086	-0,47655831	0,02079	0,0383994	conserved Plasmodium protein, unknown function
[illegible]	[illegible]	589,9549167	595,263068	414,1034252	592,6089923	1,431065179	0,517089382	0,0208606	0,038517	ubiquitin specific protease, putative
[illegible]	[illegible]	20176,91371	12252,79519	33127,17334	16214,85445	0,489472926	-1,030699033	0,0208971	0,0385718	60S ribosomal protein L7-3, putative
[illegible]	[illegible]	4,555636422	8,911123772	22,69469043	6,733380097	0,296694071	-1,752951997	0,0209505	0,0386579	rifin
[illegible]	[illegible]	1716,336022	1269,835138	2194,614629	1493,08558	0,680340667	-0,555670767	0,0209624	0,0386672	conserved Plasmodium protein, unknown function
[illegible]	[illegible]	3661,592774	3768,514243	5125,820081	3715,053509	0,724772514	-0,46439985	0,0210167	0,0387546	conserved Plasmodium membrane protein, unknown function
[illegible]	[illegible]	53,52872796	65,94231591	30,48780922	59,73552194	1,95932484	0,970356605	0,021035	0,0387758	conserved Plasmodium protein, unknown function
[illegible]	[illegible]	800,6531012	1147,752742	1634,811084	974,2029215	0,59591162	-0,746829715	0,0210962	0,038876	U2 snRNP auxiliary factor, putative
[illegible]	[illegible]	5221,898249	4773,689005	6827,413741	4997,793627	0,732018568	-0,450047852	0,0212103	0,0390735	splicing factor 3A subunit 1, putative
[illegible]	[illegible]	168,5585476	149,7068794	98,20735781	159,1327135	1,620374655	0,696327424	0,021224	0,0390858	conserved Plasmodium protein, unknown function
[illegible]	[illegible]	6389,280082	6830,376371	9123,650545	6609,828227	0,724471876	-0,464998409	0,0212955	0,0391821	conserved Plasmodium protein, unknown function
[illegible]	[illegible]	843,9316472	668,3342829	523,9660557	756,1329651	1,443095324	0,529166601	0,021297	0,0391821	sedlin, putative
[illegible]	[illegible]	22,77818211	41,88228173	11,33662396	32,33023192	2,851839492	1,511892786	0,0212921	0,0391821	conserved Plasmodium protein, unknown function
[illegible]	[illegible]	18,22254569	22,27780943	5,88724116	20,25017756	3,43938968	1,782152581	0,0213167	0,0392056	conserved Plasmodium protein, unknown function
[illegible]	[illegible]	417,9796417	409,0205811	706,6201363	413,5001114	0,585180198	-0,773047144	0,0213277	0,039213	erythrocyte membrane protein 1, PfEMP1 (IT4_var30)
[illegible]	[illegible]	[illegible]	[illegible]	[illegible]	[illegible]	[illegible]	[illegible]	[illegible]	[illegible]	[illegible]

PFIT_1204200	295,85326	401,74555	269,921458	222,7780943	348,7994082	246,3497762	0,706279226	-0,501689432	0,066307	0,1070997	conserved Plasmodium protein, unknown function
PFIT_0812600	40,252145	60,695371	27,3381853	29,40670845	50,473758	28,37026349	0,562079477	-0,831153956	0,0665552	0,1074698	RuvB-like protein 1 (RUVB1)
PFIT_0614700	1034,4801	1229,3221	974,9061943	781,5055548	1131,901121	878,2058746	0,775868014	-0,366116845	0,0668308	0,107884	RNA and export factor binding protein, putative
PFIT_1140800	375,35125	413,30658	547,8152798	511,4985045	394,3289126	529,6568921	1,343185537	0,425658601	0,0669581	0,1080586	vacuolar ATP synthase subunit f, putative
PFIT_0916800	38924,83	39535,809	33046,58661	28592,23174	39230,31949	30819,40917	0,785601789	-0,34812988	0,0670355	0,108151	RNA-binding protein musashi, putative
PFIT_1106400	37671,982	36031,855	31538,67095	26436,63089	36851,91878	28987,65092	0,786598144	-0,346301312	0,0670536	0,108151	translocon component PTEX88
PFIT_1453900	341,13693	352,6112	448,7301876	479,4184589	346,8740655	464,0743233	1,337875527	0,419943897	0,0671265	0,1082376	U1 snRNA associated protein, putative
PFIT_0406800	517,24006	616,5879	784,7083737	722,6921379	566,9139791	753,7002558	1,329479045	0,410861038	0,0673965	0,1086419	conserved Plasmodium protein, unknown function
PFIT_0722700	323,02346	299,62318	220,9483665	231,6892181	311,3233217	226,3187923	0,726957399	-0,460057272	0,0677498	0,1091804	conserved Plasmodium protein, unknown function
PFIT_0723800	17782,391	9379,8434	7353,936095	4344,172839	13581,11732	5849,054467	0,430675498	-1,215326846	0,0678512	0,1093127	18S ribosomal RNA
PFIT_1212200.1	196,22921	188,83004	95,66836487	160,4002279	192,5296247	128,0342964	0,665010886	-0,588550137	0,0681796	0,1098103	conserved protein, unknown function
PFIT_1125800	3855,1492	6287,2697	3374,58768	2007,676186	5071,209445	2691,131933	0,530668662	-0,914116739	0,0682379	0,1098729	60S ribosomal protein L35, putative
PFIT_0106000	57346,224	56819,538	49448,01664	40623,13994	57082,88127	45035,57829	0,788950685	-0,34199297	0,0683215	0,1099762	lipid/sterol:H symporter
PFIT_0825600	405,54036	376,69667	266,5047307	306,5426578	391,1185137	286,5236942	0,732575126	-0,44895138	0,0683657	0,1100159	conserved Plasmodium protein, unknown function
PFIT_0900100	95,598844	74,183232	104,7796377	142,5779808	84,89103762	123,678809	1,456912443	0,542914178	0,068408	0,1100528	B_ITvar41 - erythrocyte membrane protein 1, PfEMP1, putative
PFIT_1020000	310,94782	310,22079	233,4763666	221,8869819	310,5843022	227,6816743	0,73307528	-0,447966738	0,0691195	0,1111657	conserved Plasmodium protein, unknown function
PFIT_0728900	13,081947	9,6341859	23,91709122	26,73337132	11,35806647	25,32523127	2,229713246	1,156858183	0,0693915	0,1115714	conserved Plasmodium protein, unknown function
PFIT_1475500	500,1329	411,37974	556,9265526	804,6744766	455,7563183	680,8005146	1,493781846	0,578969471	0,0694792	0,1116807	conserved Plasmodium protein, unknown function
PFIT_1459600	201,26072	182,08611	141,2247291	130,1024071	191,6734187	135,6635681	0,707784987	-0,498616935	0,0695189	0,1117126	aminotransferase, putative
PFIT_1118600	1233,7282	1248,5905	938,461103	963,2924798	1241,159365	950,8767914	0,766119822	-0,384358047	0,0695683	0,1117602	DNA mismatch repair protein MLH, putative
PFIT_1334800	498,12029	332,37941	268,7825489	259,3137018	415,2498524	264,0481253	0,63587771	-0,653178757	0,069641	0,1118387	conserved Plasmodium protein, unknown function
PFIT_1114900.2	688,31167	700,40532	944,1556485	883,9834782	694,3584954	914,0695633	1,316423101	0,396623248	0,0696567	0,1118387	rhomboid protease ROM1
PFIT_0903700	8335,2129	10611,092	7848,222646	5161,322889	9473,152618	6504,772768	0,686653433	-0,542345967	0,0698086	0,1120507	conserved Plasmodium protein, unknown function
PFIT_0517600	566,54894	548,18518	380,3956413	449,1206381	557,3670577	414,7581397	0,744138237	-0,426357442	0,0699006	0,1121665	UTP--glucose-1-phosphate uridylyltransferase, putative
PFIT_0531300	2307,4542	2083,8744	1600,167293	1771,531406	2195,664305	1685,84935	0,767808333	-0,381181877	0,0701761	0,1125766	ubiquitin-conjugating enzyme E2 N, putative
PFIT_0209000	9470,3233	6915,4187	5391,595706	6072,039738	8192,870999	5731,817722	0,699610396	-0,515376368	0,0705608	0,1131617	RING zinc finger protein, putative
PFIT_1221300	34,214323	31,792814	51,25090975	60,59564165	33,00356826	55,9232757	1,694461497	0,760826855	0,070691	0,1133383	conserved Plasmodium protein, unknown function
PFIT_1466000	7150,7935	6703,4666	5613,682981	5222,809643	6927,130034	5418,246312	0,782177653	-0,354431776	0,0710068	0,1138123	ATP-dependent protease la, putative
PFIT_0622300	13,081947	20,23179	31,88945496	34,75338271	16,65686873	33,32141883	2,000461154	1,000332614	0,0711453	0,114002	RNA-binding protein mei2 homologue, putative
PFIT_1409400	1767,0692	1828,5685	2460,043668	2211,74092	1797,81882	2335,892294	1,29929238	0,377726118	0,0714484	0,1144552	DNA-repair helicase, putative
PFIT_1403400	80,504289	76,110069	33,02836406	61,48675403	78,30717908	47,25755904	0,603489483	-0,728599465	0,0717405	0,1148905	small nucleolar RNA snoR22
PFIT_1233000.1	630,95237	586,72192	767,6247371	822,4967242	608,8371453	795,0607307	1,305867647	0,385008684	0,0717891	0,1149083	conserved Plasmodium protein, unknown function
PFIT_1468900	27,170198	36,609906	55,80654617	54,35785501	31,89005208	55,08220059	1,727253391	0,788479744	0,0717923	0,1149083	conserved Plasmodium protein, unknown function
PFIT_0726800	8205,3997	8030,094	6450,781174	6241,35109	8117,746831	6346,066132	0,781752162	-0,355216792	0,0720567	0,1152989	conserved Plasmodium protein, unknown function
PFIT_0220300	27,170198	16,378116	39,86181869	40,99116935	21,77415687	40,42649402	1,856627298	0,892684235	0,0721157	0,1153607	Plasmodium exported protein, unknown function, pseudogene
PFIT_1010100	4550,505	3548,2707	2550,017487	3241,866828	4049,387816	2895,942158	0,715155547	-0,483671032	0,0721841	0,1154373	eukaryotic translation initiation factor 2 beta subunit, putative
PFIT_1454600	2785,4484	3353,6601	2554,573124	2261,643213	3069,554266	2408,108169	0,784513959	-0,350128978	0,0722961	0,1155837	conserved Plasmodium protein, unknown function
PFIT_0723900	17947,425	9413,5631	7435,93755	4497,444168	13680,49404	5966,690859	0,436145862	-1,197117395	0,0723183	0,1155865	18S ribosomal RNA
PFIT_0701500	168,0527	169,56167	112,7520014	122,0823957	168,8071882	117,4171986	0,695569898	-0,523732597	0,0723458	0,1155978	chitinase precursor fragment, truncated
PFIT_1458200	5094,9152	5302,6559	4210,546963	3932,478921	5198,785573	4071,512942	0,783166162	-0,352609663	0,0726003	0,1159717	signal peptide peptidase
PFIT_0812900	526,29679	333,34283	191,3367297	276,2448369	429,8198124	233,7907833	0,543927424	-0,878513929	0,0727766	0,1161875	acyl-CoA binding protein, putative
PFIT_0621900	316,98564	307,33053	424,8130964	413,476143	312,1580852	419,1446197	1,342731903	0,425171277	0,0727699	0,1161875	chorismate synthase
PFIT_1351000.2	1194,4824	1257,2613	1047,796377	862,5967811	1225,871828	955,1965791	0,779197757	-0,35993857	0,0729068	0,1163626	conserved Plasmodium protein, unknown function
PFIT_0708100	231,44983	226,40337	150,3360019	175,5491383	228,9266006	162,9425701	0,711767744	-0,49052154	0,07305	0,1165253	glutaredoxin-like protein
PFIT_1212800	275,72719	253,37909	177,6698205	203,173622	264,5531404	190,4217212	0,719786282	-0,474359489	0,0730384	0,1165253	conserved Plasmodium protein, unknown function
PFIT_0601400	410,57188	456,66041	338,2560043	313,6715568	433,6161443	325,9637806	0,751733497	-0,411706803	0,0736338	0,1174233	geranylgeranyltransferase, putative
PFIT_0523500	1749,962	2384,461	1634,334566	1209,239496	2067,211503	1421,787031	0,687780147	-0,539980623	0,0737151	0,1175199	conserved Plasmodium protein, unknown function, putative
PFIT_0628000	1964,3047	1781,361	1468,053837	1434,690927	1872,832819	1451,372382	0,774960994	-0,367804398	0,0738071	0,1176333	nicotinate phosphoribosyltransferase, putative
PFIT_0827300	462,89966	430,64811	544,3985525	625,5608888	446,7738873	584,9797206	1,309341788	0,388841745	0,0739378	0,1178085	nucleoside transporter 2
PFIT_0536500	16,100858	12,524442	1,138909106	7,128899018	14,31264979	4,133904062	0,288828702	-1,791713979	0,0744918	0,1186577	rifin
PFIT_0621400	241,51287	231,22046	209,5592754	129,2112947	236,3666651	169,3852851	0,716620869	-0,480718037	0,0746048	0,1188042	RNA methyltransferase, putative
PFIT_1147100	22,13868	17,341535	36,44509138	38,31783222	19,74010712	37,3814618	1,893680798	0,921193168	0,0748362	0,119139	leucine-rich repeat protein
PFIT_1225700	119,75013	113,68339	166,2807294	166,6380145	116,7167622	166,459372	1,426182229	0,512158332	0,074968	0,1193153	conserved Plasmodium protein, unknown function
PFIT_1459200	9637,3697	10481,994	9105,578299	6484,624769	10059,68201	7795,101534	0,774885481	-0,367944982	0,0750075	0,1193446	cysteine proteinase falcipain 1
PFIT_0301400	5,0315181	4,817093	9,111272844	19,6044723	4,924305524	14,35787257	2,915715222	1,543849818	0,0751067	0,1194687	Plasmodium exported protein, unknown function
PFIT_1312200	67,422342	50,097767	85,41818292	94,45791198	58,76005458	89,93804745	1,530598433	0,614095828	0,0751916	0,1195701	ubiquitin-like protein nedd8 homologue, putative
PFIT_1131500	433,71686	374,76983	460,1192786	594,3719556	404,2433457	527,2456171	1,304277789	0,383251172	0,0753011	0,1197105	structural maintenance of chromosome protein, putative
PFIT_0626300	117,73752	99,232115	59,22327349	81,9823387	108,4848191	70,6028061	0,650808165	-0,619695743	0,0753788	0,1197667	mitochondrial import inner membrane translocase subunit TIM22, putative (TIM22)
PFIT_1251100	241,51287	311,18421	212,9760027	194,2624982	276,3485367	203,6192505	0,73682044	-0,440615011	0,0753642	0,1197667	conserved Plasmodium protein, unknown function
PFIT_0219300	2413,1161	1962,4837	1747,086568	1656,577909	2187,799873	1701,832239	0,777873817	-0,362391948	0,0754678	0,1198744	Plasmodium exported protein, unknown function
PFIT_0806600	3185,9573	3492,3924	2816,522218	2442,539026	3339,174824	2629,530622	0,787479171	-0,344686331	0,075694	0,1201999	20S proteasome beta subunit
PFIT_0510800	217,36158	252,41567	158,3083657	179,1135878	234,8886262	168,7109767	0,718259455	-0,477423015	0,0758031	0,1203392	conserved Plasmodium protein, unknown function
PFIT_1420700	7524,1321	5438,498	3335,86477	4831,611309	6481,31505	4083,73804	0,63007862	-0,666396239	0,0761294	0,120808	conserved Plasmodium protein, unknown function
PFIT_1006000	128,80686	113,68339	174,2530931	170,202464	121,2451284	172,2277786	1,42049236	0,506391072	0,0761411	0,120808	conserved Plasmodium protein, unknown function
PFIT_0616100	11557,397	13044,688	10466,57468	9021,621707	12301,04239	9744,098193	0,792135974	-0,336179998	0,0763444	0,1210965	alpha adaptin-like protein, putative
PFIT_0831100	747,68359	779,40564	937,3221939	1036,363695	763,5446143	986,8429443	1,292449617	0,370108042	0,0768079	0,1217976	leucine--tRNA ligase, putative
PFIT_0903200	25,15759	17,341535	34,16727317	43,66450648	21,24956255	38,91588982	1,831373693	0,872926204	0,0769271	0,1219524	conserved protein, unknown function
PFIT_1339000	0	0	2,277818211	6,23778664	0	4,257802426	#DIV/0!	#DIV/0!	0,0769522	0,1219579	mitotic-spindle organizing protein 1, putative
PFIT_0921600	20,126072	29,865976	45,55636422	44,55561886	24,99602435	45,05599154	1,80252631	0,850020318	0,0769887	0,1219815	conserved Plasmodium protein, unknown function
PFIT_1146100	7615,7058	8320,083	11022,36232	9501,931278	7967,894369	10262,1468	1,287937104	0,365062142	0,0770431	0,1220335	dynamin-like protein
PFIT_1423300	612,8389	529,88023	443,0356421	424,1694916	571,3595643	433,6025668	0,758896138	-0,398025642	0,077124	0,1221273	DnaJ protein, putative
PFIT_0302600	37,233234	21,195209	43,27854601	56,14007976	29,21422143	49,70931289	1,701545016	0,76684532	0,0772087	0,1222273	CDGSH iron-sulfur domain-containing protein, putative
PFIT_0417600	29,182805	34,683069	56,94545528	52,57563026	31,93293711	54,76054277	1,714860822	0,778091492	0,0772333	0,122232	conserved Plasmodium protein, unknown function
PFIT_1353300	186,16617	150,2933	111,6130923	122,9735081	168,2297348	117,2933002	0,697220978	-0,520312117	0,0773603	0,1223987	conserved Plasmodium protein, unknown function
PFIT_1475900	2628,465	2734,182	2149,121482	2046,88513	2681,323506	2098,003306	0,782450645	-0,353928342	0,0774291	0,1224733	proteasome subunit alpha type 1, putative
PFIT_0506300	149,93924	133,91518	177,6698205	214,7580829	141,9272116	196,2139517	1,382497052	0,467276404	0,0774515	0,1224744	conserved Plasmodium protein, unknown function
PFIT_0216800	262,64524	258,19618	380,3956413	327,0382424	260,4207134	353,7169418	1,35825195	0,441751118	0,0774925	0,122505	conserved Plasmodium protein, unknown function
PFIT_1134200	94,59254	124,281	222,0872756	147,0335422	109,4367692	184,5604089	1,686457031	0,753995561	0,0777023	0,1228022	conserved Plasmodium protein, unknown function
PFIT_0321100	1975,374	2014,5083	1645,723658	1479,246546	1994,941138	1562,485102	0,783223661	-0,352503746	0,077824	0,1229257	conserved Plasmodium protein, unknown function
PFIT_1229600.1	212,33006	236,03756	302,9498221	305,6515454	224,1838092	304,3006837	1,357371368	0,440815487	0,0778061	0,1229257	conserved Plasmodium protein, unknown function
PFIT_1117400	585,66871	506,75818	634,3723718	775,2677682	546,2134424	704,82007	1,290374816	0,367790187	0,0785009	0,1239604	oligosaccharyl transferase STT3 subunit, putative
PFIT_1445600	634,97758	529,88023	365,5898229	491,0029198	582,4289041	428,2963714	0,735362494	-0,443472499	0,0786879	0,1241862	conserved Plasmodium protein, unknown function
PFIT_1136000	580,63719	633,92943	484,0363699	445,5561886	607,2833104	464,7962792	0,765369756	-0,385771201	0,0786676	0,1241862	conserved Plasmodium membrane protein, unknown function
PFIT_1449300	255,60112	237,96439	205,003639	159,5091155	246,7827555	182,2563773	0,738529631	-0,43727229	0,0788518	0,1244019	conserved Plasmodium protein, unknown function
PFIT_0812200	925,79933	1082,8825	1449,831291	1180,7239	1004,340913	1315,277596	1,309592768	0,38911826	0,0788686	0,1244019	unspecified product
PFIT_0419000	132,83208	142,58595	207,2814572	181,786925	137,7090146	194,5341911	1,412646744	0,498400741	0,079002	0,1245775	ribosome recycling factor, putative
PFIT_1242100	3210,1085	4166,7854	3047,720766	2447,8857	3688,446975	2747,803233	0,744975664	-0,424734797	0,079257	0,1248872	Tetratricopeptide repeat protein, putative
PFIT_1148500	1138,1294	1100,224	875,8211022	855,4678821	1119,176712	865,6444921	0,773465426	-0,37059129	0,0792544	0,1248872	conserved Plasmodium protein, unknown function
PFIT_1210200	4603,839	4950,0447	4116,017507	3457,516024	4776,941888	3786,766766	0,792717779	-0,335120761	0,0792647	0,1248872	conserved Plasmodium protein, unknown function
PFIT_1245300	491,07617	491,34348	622,9832807	660,3142715	491,2098236	641,6487761	1,306262101	0,385444402	0,0794189	0,1250953	kinesin-13, putative
PFIT_0833200	338,11802	563,59988	288,1440037	216,5403077	450,8589459	252,3421557	0,559692023	-0,837294908	0,0794915	0,1251747	sporozoite invasion-associated protein 2 (SIAP-2)
PFIT_0724400	1802,2898	820,83264	322,3112769	549,8163367	1311,56121	436,0638068	0,332476901	-1,588673982	0,0796277	0,1253543	unspecified product
PFIT_1409900	542,39765	409,4529	365,5898229	350,2071642	475,9252757	357,8984936	0,752005644	-0,411184605	0,0796739	0,1253799	tRNA-dihydrouridine synthase, putative
PFIT_0922500	243,52548	263,01328	354,2007318	330,6026919	253,2693755	342,4017119	1,351927019	0,435017273	0,0796884	0,1253799	para-aminobenzoic acid synthetase
PFIT_0500500	42,264752	28,902558	22,77818211	15,14891041	35,58365485	18,96354626	0,532928569	-0,90798592	0,0797384	0,1253888	rifin (RIF)
PFIT_1124900	3012,873	2632,0596	2196,955665	2207,285358	2822,466312	2202,120511	0,780211442	-0,358062939	0,0797376	0,1253888	endoplasmic reticulum oxidoreductin, putative
PFIT_1407100	1855,6239	1943,2153	1573,972384	1407,066444	1899,419585	1490,519414	0,78472362	-0,349743468	0,0799753	0,1257263	conserved Plasmodium protein, unknown function
PFIT_0626400	15742,614	28209,86	14072,36091	6154,022077	21976,23679	10113,19149	0,460187592	-1,11970601	0,0804277	0,1264024	4-methyl-5(B-hydroxyethyl)-thiazol monophosphate biosynthesis enzyme
PFIT_0221200	185,15987	266,86695	170,8363658	125,6468452	226,0134078	148,2416055	0,655897395	-0,608457949	0,0805128	0,126441	hypothetical protein
PFIT_1446700	1235,7408	1105,0411	939,6000121	877,7456916	1170,390984	908,6728518	0,776384016	-0,365157679	0,0805194	0,126441	conserved Plasmodium protein, unknown function
PFIT_1436100	12,075643	19,268372	27,3381853	34,75338271	15,67200762	31,04360062	1,980831133	0,986105895	0,0804927	0,126441	conserved Plasmodium protein, unknown function
PFIT_1242600	25,15759	26,012302	35,30618227	53,46674263	25,58494621	44,38646245	1,734866358	0,794824532	0,0806757	0,1266512	conserved Plasmodium protein, unknown function
PFIT_1223100	32129,262	27517,162	22203,03301	24435,1925	29823,21186	23319,11275	0,781911515	-0,354922742	0,0811953	0,1274314	phospholipid-transporting ATPase, putative
PFIT_1364800	1697,6342	1964,4105	2460,043668	2257,187651	1831,022356	2358,61566	1,288141378	0,365290942	0,0813536	0,1276446	conserved Plasmodium protein, unknown function
PFIT_1338800	350,19366	314,07446	429,3687328	448,2295257	332,1340599	438,7991293	1,32115065	0,401794986	0,0817677	0,1282586	conserved Plasmodium protein, unknown function
PFIT_1214300	83,5232	79,963743	44,41745512	58,8134169	81,74347169	51,61543601	0,631431904	-0,663300937	0,0822389	0,1289619	conserved Plasmodium protein, unknown function
PFIT_1102200	24,151287	34,683069	14,80581837	14,25779804	29,41717807	14,5318082	0,493990558	-1,017444628	0,0827283	0,1296933	erythrocyte membrane protein 1 (PfEMP1), exon2,, putative
PFIT_0508400	195,2229	210,98867	267,6436398	282,4826236	203,1057867	275,0631317	1,354285056	0,437531436	0,0829916	0,13007	transcription factor IIb, putative
PFIT_1424600	90,567326	88,63451	140,08582	122,0823957	89,60091802	131,0841078	1,462977286	0,54890737	0,083055	0,1300986	serine/threonine protein kinase, putative
PFIT_1478700	67,422342	65,512464	80,86254649	114,9534967	66,46740331	97,90802158	1,473023117	0,558780071	0,0830559	0,1300986	surface-associated interspersed protein 14.1 (SURFIN 14.1), pseudogene (SURF14.1)
PFIT_0111200	368,30712	403,67239	291,560731	288,7204102	385,989757	290,1405706	0,751679456	-0,41181052	0,083273	0,1304026	Plasmodium exported protein (hyp11), unknown function
PFIT_1405600.2	25,15759	22,158628	38,72290959	45,44673124	23,65810903	42,08482041	1,778875073	0,830965196	0,083393	0,1305544	adenylyl cyclase alpha
PFIT_1354000	312,96043	299,62318	215,2538209	236,14478	306,2918036	225,6993005	0,736876723	-0,440504814	0,0834767	0,130613	metacaspase 1
PFIT_1303100	10,063036	9,6341859	20,5003639	24,06003418	9,848611047	22,28019904	2,262268145	1,177769941	0,0834762	0,130613	conserved Plasmodium protein, unknown function
PFIT_1134600	2971,6146	2179,2529	1431,608746	1971,140578	2575,433719	1701,374662	0,660616753	-0,598114538	0,0837901	0,1310624	conserved Plasmodium protein, unknown function
PFIT_1475300	1091,8394	977,86987	1183,326561	1621,824527	1034,854648	1402,575544	1,355335792	0,438650331	0,0838103	0,1310624	conserved Plasmodium membrane protein, unknown function
PFIT_0829100	62,390824	54,91486	79,72363739	97,13124912	58,65284201	88,42744325	1,507641236	0,592293161	0,0839947	0,1313144	alpha/beta hydrolase, putative
PFIT_0620900	154,97076	105,01263	61,5010917	91,78457485	129,9916918	76,64283328	0,589597937	-0,762196619	0,0843458	0,1318268	conserved Plasmodium protein, unknown function
PFIT_0212600	40,252145	53,951441	64,91781902	81,9823387	47,10179293	73,45007886	1,559390297	0,640982063	0,0844358	0,1319309	conserved Plasmodium protein, unknown function
PFIT_0509600	822,15006	1061,6873	767,6247371	698,6321037	941,9186719	733,1284204	0,77833516	-0,361536565	0,0844778	0,13196	asparagine-tRNA ligase, putative
PFIT_1341700	104,65558	137,76886	230,0596393	157,7268908	121,2122174	193,893265	1,599618167	0,677727571	0,0846684	0,1322212	sporozoite protein essential for cell traversal
PFIT_0701300	6246,1266	12602,479	5340,344796	2396,201182	9424,302578	3868,272989	0,410457215	-1,284696248	0,0850353	0,1327575	conserved Plasmodium protein, unknown function
PFIT_0921400	429,69164	439,31888	330,2836406	327,0382424	434,5052613	328,6609415	0,756402674	-0,402773631	0,0850902	0,1328065	conserved Plasmodium protein, unknown function
PFIT_1000700.1	812,08702	644,52704	540,9818251	572,9852585	728,3070287	556,9835418	0,764764749	-0,38691207	0,0854846	0,1333852	acyl-CoA binding protein, isoform 1, ACBP1
PFIT_1357200	4290,8786	3199,5131	1736,836386	2870,272967	3745,195884	2303,554676	0,615069211	-0,701179336	0,0856328	0,1335796	small subunit rRNA processing factor, putative
PFIT_0501200.1	11336,01	10842,313	9317,415392	8309,622917	11089,16154	8813,519155	0,794786794	-0,331360193	0,0857112	0,1336281	heat shock protein 40, type II
PFIT_0533900	118,74383	132,95177	198,1701844	159,5091155	125,8477963	178,8396499	1,421078916	0,506986673	0,085701	0,1336281	conserved Plasmodium protein, unknown function
PFIT_0617300	20,126072	18,304953	42,13963691	31,1889332	19,2155128	36,66428505	1,90805655	0,93210393	0,0859725	0,1339985	conserved Plasmodium membrane protein, unknown function
PFIT_1116300	971,08299	1241,8466	931,6276483	600,6097422	1106,464778	766,1186953	0,692402244	-0,530317694	0,0860676	0,1341097	transcription factor with AP2 domain(s), putative
PFIT_0517500	4337,1686	3018,3904	1988,535298	2686,703817	3677,77952	2337,619558	0,635606225	-0,65379484	0,0861248	0,1341249	FACT complex subunit SPT16, putative
PFIT_1227600	982,15233	1088,663	864,4320111	757,4455206	1035,40767	810,9387659	0,783207223	-0,352534025	0,0861197	0,1341249	RNA pseudouridylate synthase, putative
PFIT_0418400	1703,672	1763,056	1347,329472	1363,401937	1733,364024	1355,365704	0,781927908	-0,354892495	0,0863997	0,1345159	60S ribosomal protein L7ae/L30e, putative
PFIT_0515300	10,063036	4,817093	17,08363658	19,6044723	7,440064567	18,34405444	2,465577318	1,301925495	0,0866272	0,134833	conserved Plasmodium protein, unknown function
PFIT_1445000	1141,1483	1318,9201	1002,240013	925,8657599	1230,034177	964,0528864	0,783761057	-0,351514205	0,0867219	0,1349431	zinc finger protein, putative
PFIT_0918700	380,38277	366,09906	444,1745512	526,6474149	373,2409162	485,410983	1,300529931	0,379099603	0,0867482	0,134947	DNA helicase, putative
PFIT_1319100	163,02119	212,91551	153,7527292	121,1912833	187,9683474	137,4720063	0,731357211	-0,451351873	0,0868674	0,1350554	conserved Plasmodium protein, unknown function
PFIT_1109000	333,0865	314,07446	371,2843684	471,3984475	323,5804792	421,341408	1,302122455	0,38086513	0,0868622	0,1350554	D-tyrosyl-tRNA(Tyr) deacylase
PFIT_0204600	532,33461	445,09939	648,0392811	625,5608888	488,7170016	636,8000849	1,303003748	0,381841234	0,0868896	0,1350554	conserved Plasmodium protein, unknown function
PFIT_1201800.2	382,39537	385,36744	252,8378214	317,2360063	383,8814057	285,0369139	0,742512947	-0,429511913	0,0869305	0,1350818	conserved protein, unknown function
PFIT_1141900	10,063036	5,7805116	13,66690927	30,29782083	7,921773863	21,98236505	2,774929634	1,472451188	0,0871522	0,135389	conserved Plasmodium protein, unknown function
PFIT_1018000	1144,1672	1416,2253	1108,15856	839,4278593	1280,196272	973,7932095	0,760659308	-0,394677667	0,087274	0,1355409	PHF5-like protein, putative
PFIT_0825900	703,40623	678,24669	530,7316432	531,9940892	690,8264587	531,3628662	0,769169825	-0,378625929	0,0874427	0,1357656	protein kinase, putative
PFIT_1470600	243,52548	220,62286	167,4196385	171,0935764	232,0741665	169,2566075	0,729321191	-0,455373782	0,0875929	0,1359616	conserved Plasmodium protein, unknown function
PFIT_0717500	3688,1028	2206,2286	1323,412381	1823,215924	2947,165667	1573,314152	0,53383974	-0,90552139	0,0876822	0,1360628	actin-related protein, putative (ARP6)
PFIT_1468800	58,36561	29,865976	15,94472748	21,38669705	44,11579308	18,66571227	0,423107258	-1,240904659	0,0879156	0,1363874	conserved Plasmodium protein, unknown function
PFIT_0537300	21,132376	11,561023	4,555636422	7,128899018	16,34669954	5,84226772	0,357397388	-1,484399003	0,0879772	0,136408	rifin (RIF)
PFIT_1129900	27574,732	30791,822	26584,41634	19450,30986	29183,27667	23017,3631	0,788717571	-0,342419311	0,0879607	0,136408	parasitophorous vacuolar protein 1
PFIT_1436700	1882,7941	2258,2532	1818,837842	1265,379576	2070,523624	1542,108709	0,744791651	-0,425091194	0,0881068	0,1365581	conserved Plasmodium protein, unknown function
PFIT_0508300	629,94606	572,27064	424,8130964	488,3295827	601,1083541	456,5713395	0,75954915	-0,396784771	0,0881223	0,1365581	triose phosphate transporter
PFIT_1004000	728,56382	572,27064	797,2363739	1211,912833	650,4172313	1004,574603	1,544507979	0,627147324	0,088413	0,1369711	RNA binding protein, putative
PFIT_0203100	41,258448	40,463581	69,47345544	62,3778664	40,86101459	65,92566092	1,613412236	0,690115103	0,0884949	0,1370604	conserved Plasmodium protein, unknown function
PFIT_1351600	2023,6766	1890,2273	1585,361475	1494,395457	1956,951926	1539,878466	0,786875981	-0,345791824	0,0886613	0,1372804	conserved Plasmodium protein, unknown function
PFIT_1359600	596,73805	660,90515	769,9025553	847,4478707	628,8215996	808,675213	1,286016914	0,362909617	0,0889203	0,1376438	guanylyl cyclase beta
PFIT_0809400	5184,4762	5756,4261	8212,67356	6315,313417	5470,451162	7263,993489	1,327860038	0,409103089	0,0890686	0,1378355	DnaJ protein, putative
PFIT_1031000	497,11399	463,40434	356,47855	372,4849737	480,2591649	364,4817619	0,758927239	-0,397966519	0,0891637	0,1379384	conserved Plasmodium protein, unknown function
PFIT_0112800	70,441253	75,14665	109,3352741	106,0423729	72,7939517	107,6888235	1,479364988	0,564978038	0,0891839	0,1379384	Pfmc-2TM Maurer's cleft two transmembrane protein
PFIT_bin08800	13,081947	19,268372	44,41745512	24,95114656	16,17515943	34,68430084	2,144294217	1,100502871	0,0896136	0,1385651	rifin
PF_apicoplast_genon	9,0567326	2,8902558	13,66690927	39,2089446	5,973494166	26,43792693	4,425873064	2,145962075	0,0896429	0,1385724	apicoplast ribosomal protein L14
PFIT_0111800	72,45386	65,512464	54,66763707	34,75338271	68,98316236	44,71050989	0,648136565	-0,625630268	0,0896733	0,1385815	Plasmodium exported protein, unknown function
PFIT_0810700	7653,9453	8032,0208	6845,982633	5714,703675	7842,983058	6280,343154	0,800759495	-0,320559095	0,090003	0,139053	proteasome subunit alpha type 5, putative
PFIT_0920400	3,0189109	1,9268372	6,833454633	12,47557328	2,472874018	9,654513957	3,904167332	1,965014888	0,0901047	0,1391721	conserved Plasmodium protein, unknown function
PFIT_1313500	70,441253	82,853999	97,94618308	123,8646204	76,64762607	110,9054018	1,446951555	0,533016621	0,0907187	0,1400822	conserved Plasmodium protein, unknown function
PFIT_0318600	4112,7629	3612,8197	3152,500404	2964,730879	3862,791302	3058,615642	0,791814883	-0,336764911	0,0909403	0,140386	cleavage and polyadenylation specifity factor, putative
PFIT_0913900	1229,703	1780,3976	6425,725173	2789,181741	1505,050289	4607,453457	3,061328575	1,614157899	0,0910722	0,1405512	conserved Plasmodium protein, unknown function
PFIT_1223300	1774,1133	1699,4704	1289,245107	1420,433129	1736,791837	1354,839118	0,780081464	-0,358303303	0,0911421	0,1406207	conserved Plasmodium protein, unknown function
PFIT_1008000	3719,2982	4178,3464	5349,456069	4721,113374	3948,822302	5035,284722	1,275135809	0,350650911	0,0912367	0,1407282	conserved Plasmodium membrane protein, unknown function
PFIT_1249900	100,63036	102,12237	159,4472748	131,8846318	101,3763662	145,6659533	1,436882764	0,522942356	0,0914033	0,1409467	conserved Plasmodium protein, unknown function
PFIT_0310500.2	812,08702	1109,8582	779,0138282	429,5161658	960,9726186	604,264997	0,628805634	-0,66931395	0,0916369	0,1412684	SAC3/GNAP family-related protein, putative
PFIT_1017500	531,32831	516,39237	392,9236414	407,2383564	523,8603377	400,0809989	0,763716911	-0,388890125	0,0916622	0,1412689	tRNA pseudouridine synthase, putative
PFIT_1310100	358,24409	424,8676	346,2283681	258,4225894	391,5558434	302,3254787	0,772113311	-0,37311551	0,0917985	0,1414017	mitochondrial ribosomal protein S15 precursor, putative
PFIT_0535000	110,6934	126,20784	187,9200024	148,815767	118,4506167	168,3678847	1,421418388	0,507331268	0,0917817	0,1414017	CPW-WPC family protein
PFIT_0412800.2	4,0252145	0	5,694545528	16,04002279	2,012607235	10,86728416	5,399605035	2,432853882	0,0921171	0,1418538	calmodulin-like protein
PFIT_0215400	259,62633	310,22079	388,368005	368,9205242	284,92356	378,6442646	1,328932801	0,410268155	0,0926049	0,1425661	DEAD/DEAH box helicase, putative
PFIT_0810200	2994,7596	3581,9903	2856,384037	2406,003418	3288,374945	2631,193728	0,800150157	-0,321657332	0,0926641	0,1426184	Rab GTPase 18
PFIT_0921500	9,0567326	10,597605	26,19490943	18,71335992	9,827168535	22,45413467	2,284903795	1,192133422	0,0927	0,1426349	Fe-S-cluster redox enzyme, putative
PFIT_1225600	111,6997	103,08579	132,1134562	166,6380145	107,3927454	149,3757354	1,390929478	0,476049275	0,0927375	0,1426537	conserved Plasmodium protein, unknown function
PFIT_1472700	131,82577	76,110069	138,9469109	183,5691497	103,9679213	161,2580303	1,551036399	0,633232543	0,0927633	0,1426546	conserved Plasmodium protein, unknown function
PFIT_1251600	356,23148	416,19683	312,0610949	278,0270617	386,2141562	295,0440783	0,763939057	-0,388470542	0,0930771	0,1430982	conserved Plasmodium protein, unknown function
PFIT_0323300	164,02749	180,15928	218,6705483	245,9470161	172,0933832	232,3087822	1,34989956	0,432852067	0,0939329	0,1443746	conserved Plasmodium protein, unknown function
PFIT_1329700.1	4009,1136	4887,4225	3805,095322	3319,393605	4448,268065	3562,244463	0,800816051	-0,320457205	0,0943283	0,1449428	ER lumen protein retaining receptor 1, putative
PFIT_0622400	817,11854	938,36971	750,5411006	632,6897878	877,744123	691,6154442	0,787946539	-0,343830346	0,0944821	0,1451321	superoxide dismutase
PFIT_1218100	4329,1182	3796,8327	3588,702592	2921,066372	4062,975417	3254,884482	0,801108584	-0,319930293	0,0945029	0,1451321	triose or hexose phosphate/phosphate translocator, putative
PFIT_0109000	1320,2703	1392,1399	1075,130196	1056,859279	1356,205106	1065,994738	0,786012922	-0,347375065	0,0947963	0,1455382	bromodomain protein, putative
PFIT_0407800	5139,1926	5110,9356	4715,083697	3556,429497	5125,064102	4135,756597	0,806966804	-0,309418769	0,0948445	0,1455382	40S ribosomal processing protein, putative
PFIT_0530300	36,22693	26,975721	47,83418243	56,14007976	31,6013254	51,9871311	1,645093376	0,718169474	0,0948279	0,1455382	conserved Plasmodium membrane protein, unknown function
PFIT_0105200	9853,725	7684,2267	5676,322982	7190,385772	8768,975856	6433,354377	0,733649457	-0,446837197	0,0952435	0,1461107	small ribosomal subunit assembling AARP2 protein
PFIT_0624300	7728,4118	6682,2714	6110,247351	5405,48768	7205,341568	5757,867516	0,799110974	-0,323532229	0,0953833	0,1462855	conserved Plasmodium protein, unknown function
PFIT_0418100	790,95464	661,86857	548,9541889	572,9852585	726,411608	560,9697237	0,772247742	-0,372864348	0,0957694	0,1468378	conserved Plasmodium membrane protein, unknown function
PFIT_1106000	49,308877	33,719651	72,89018275	59,70452927	41,51426399	66,29735601	1,596977753	0,675344215	0,0959157	0,1470222	conserved Plasmodium protein, unknown function
PFIT_0613200	1289,0749	1145,5047	1026,157104	896,4590515	1217,28982	961,3080778	0,789711753	-0,340601933	0,0960711	0,1472204	conserved Plasmodium protein, unknown function
PFIT_1210300	100,63036	66,475883	119,5854561	120,3001709	83,5531223	119,9428135	1,435527605	0,521581073	0,0961865	0,1473573	conserved Plasmodium protein, unknown function
PFIT_1119400	2487,5825	1701,3972	1313,162199	1495,286569	2094,489888	1404,224384	0,670437414	-0,576825434	0,0965648	0,1478967	histone acetyltransferase, putative
PFIT_1355100	360,2567	424,8676	306,3665494	293,1759721	392,5621471	299,7712607	0,763627525	-0,389058989	0,096814	0,1482383	conserved Plasmodium protein, unknown function
PFIT_0720900	26,163894	17,341535	29,61163674	51,68451788	21,75271435	40,64807731	1,868643915	0,901991678	0,0969038	0,1483355	conserved Plasmodium protein, unknown function
PFIT_0537400	28,176501	24,085465	41,0007278	48,12006837	26,13098304	44,56039808	1,705270636	0,770000722	0,0969719	0,1483996	rifin
PFIT_1300700	271,70198	153,18356	304,0887312	314,5626692	212,4427664	309,3257002	1,456042516	0,542052483	0,0970446	0,1484706	Plasmodium exported protein (PHISTa), unknown function
PFIT_1250700	246,54439	229,29362	288,1440037	337,731591	237,9190056	312,9377973	1,315312312	0,395405398	0,0971945	0,1486597	adaptor-related protein complex 3, sigma 2 subunit, putative
PFIT_1422300	125,78795	133,91518	148,0581837	210,302521	129,8515682	179,1803524	1,379885933	0,464549013	0,0972953	0,1487737	conserved Plasmodium protein, unknown function
PFIT_1004300	73,460164	88,63451	99,08509218	155,944666	81,04733727	127,5148791	1,573338291	0,653828905	0,0974504	0,1489705	conserved Plasmodium protein, unknown function
PFIT_0205300	5687,628	5255,4484	7158,043728	6645,916109	5471,538233	6901,979919	1,261433189	0,335063796	0,0975965	0,1491534	DNA repair endonuclease, putative
PFIT_1010400	355,22518	361,28197	297,2552765	251,2936904	358,2535745	274,2744835	0,76558757	-0,385360689	0,0977472	0,1493433	conserved Plasmodium protein, unknown function
PFIT_0526900	9,0567326	7,7073487	12,52800016	25,84225894	8,382040647	19,18512955	2,288837571	1,194615084	0,0978235	0,1494195	iron-sulfur assembly protein, putative
PFIT_0525600	86,542111	92,488185	133,2523653	123,8646204	89,51514797	128,5584929	1,436164669	0,522221177	0,097872	0,1494532	conserved Plasmodium protein, unknown function, putative
PFIT_0727000	2688,8433	3665,8077	4867,697517	3893,269976	3177,325504	4380,483747	1,378670124	0,463277303	0,0983443	0,1501339	conserved Plasmodium membrane protein, unknown function
PFIT_1145600	1328,3208	1249,5539	1657,112749	1613,804515	1288,937344	1635,458632	1,268842616	0,343513132	0,0983914	0,1501652	Rab GTPase 6
PFIT_1420500	32,201716	35,646488	59,22327349	52,57563026	33,92410183	55,89945187	1,647779863	0,720523517	0,0984305	0,1501843	conserved Plasmodium protein, unknown function
PFIT_0917200	464,91227	372,843	308,6443676	327,0382424	418,8776332	317,841305	0,758792735	-0,398222229	0,0985311	0,1502972	N-glycosylase/DNA lyase, putative
PFIT_0730700	1973,3614	2111,8136	1780,114932	1488,15767	2042,587474	1634,136301	0,80003247	-0,321869541	0,098626	0,1503607	alpha/beta hydrolase, putative
PFIT_0301700	5994,5506	5724,6333	5287,954977	4168,623701	5859,591961	4728,289339	0,806931501	-0,309481883	0,0986151	0,1503607	Plasmodium exported protein, unknown function
PFIT_1437700	61,384521	66,475883	79,72363739	106,9334853	63,93020176	93,32856133	1,459850881	0,54582101	0,0990122	0,1509088	GTP-binding protein, putative
PFIT_1369100	6,0378217	9,6341859	0	2,673337132	7,836003812	1,336668566	0,170580387	-2,551476314	0,0990482	0,1509229	tRNA Cysteine
PFIT_0502700	369,31343	323,70865	238,0320031	280,7003988	346,5110373	259,3662009	0,748507762	-0,417910818	0,0992838	0,1512412	conserved Plasmodium protein, unknown function
PFIT_0501200.2	8260,7464	7505,9943	6557,83863	6055,108603	7883,370323	6306,473616	0,799971758	-0,321979026	0,0996544	0,1517648	heat shock protein 40, type II
PFIT_0826700	2190,723	2138,7893	1747,086568	1688,657955	2164,756125	1717,872261	0,793563876	-0,333581741	0,1004461	0,1529293	DnaJ protein, putative
PFIT_bin06300	107,67449	76,110069	62,64000081	60,59564165	91,89227792	61,61782123	0,670544061	-0,576595961	0,1005872	0,1531028	B_ITvar19 - erythrocyte membrane protein 1, PfEMP1, putative
PFIT_1430000.2	210,31746	184,97637	153,7527292	137,2313061	197,6469129	145,4920177	0,736120871	-0,441985418	0,1013409	0,1541972	transcription initiation TFIID-like, putative
PFIT_1033500	4,0252145	2,8902558	7,972363739	14,25779804	3,457735123	11,11508089	3,214555335	1,684619185	0,1013608	0,1541972	conserved Plasmodium protein, unknown function
PFIT_1137000	9228,8105	10380,835	8203,562287	7549,50406	9804,822902	7876,533173	0,803332528	-0,3159308	0,101416	0,1542396	tudor staphylococcal nuclease
PFIT_1336200	288,80914	232,18388	173,114184	210,302521	260,4965094	191,7083525	0,735934439	-0,442350846	0,1015271	0,1543671	endopeptidase, putative
PFIT_1121100	503,15181	447,02623	572,8712801	641,6009116	475,0890177	607,2360958	1,278152248	0,354059694	0,1020959	0,1551902	metal ion channel - Mg2 , Co2 and Ni2
PFIT_0510500	1993,4875	1773,6536	1417,941836	1546,079974	1883,570547	1482,010905	0,786809343	-0,345914005	0,1021398	0,1552152	topoisomerase I
PFIT_1032400	505,16442	423,90418	338,2560043	370,7027489	464,5342982	354,4793766	0,763085477	-0,390083425	0,1023765	0,155533	conserved Plasmodium protein, unknown function
PFIT_1137300	7012,9299	4853,7029	3081,88804	4415,461829	5933,316388	3748,674934	0,631800951	-0,662457987	0,1024601	0,1556182	conserved Plasmodium protein, unknown function
PFIT_1232100	26,163894	9,6341859	28,47272764	37,42671984	17,89903999	32,94972374	1,840865419	0,880384159	0,1025949	0,1557811	cytochrome b5, putative
PFIT_1321100	2992,747	3393,1603	5479,291707	3834,456559	3192,95362	4656,874133	1,458484741	0,544470292	0,1035653	0,1572122	conserved Plasmodium protein, unknown function
PFIT_1403300	747,68359	644,52704	537,5650978	545,3607749	696,1053129	541,4629363	0,777846292	-0,362442999	0,1041144	0,1579716	U1 small nuclear ribonucleoprotein C, putative
PFIT_1428000	557,4922	519,28262	613,8720079	749,4255092	538,3874126	681,6487586	1,266093416	0,340383855	0,1041215	0,1579716	conserved Plasmodium protein, unknown function
PFIT_0311900	16,100858	5,7805116	26,19490943	20,49558468	10,94068472	23,34524705	2,13380128	1,093425825	0,1042581	0,1581365	conserved Plasmodium protein, unknown function
PFIT_0110300	266,67046	316,0013	387,2290959	377,8316479	291,3358784	382,5303719	1,313021843	0,392890916	0,1044159	0,1583333	conserved Plasmodium protein, unknown function
PFIT_1134500	2427,2043	2814,1457	2216,317119	1988,071714	2620,675016	2102,194416	0,802157613	-0,31804236	0,1045379	0,1584364	RNA (uracil-5-)methyltransferase, putative
PFIT_1427300	120,75643	71,292976	127,5578198	149,7068794	96,02470495	138,6323496	1,443715445	0,529786416	0,10454	0,1584364	dynein-associated protein, putative
PFIT_0936100	18629,699	19115,188	17618,92386	13155,49202	18872,44358	15387,20794	0,815326742	-0,294549759	0,1045825	0,1584584	gametocytogenesis-implicated protein
PFIT_0701100	6502,734	5850,8411	5687,712073	4337,04394	6176,787543	5012,378006	0,811486225	-0,301361489	0,1047487	0,1586676	lysophospholipase, putative
PFIT_1430200	245,53808	344,90386	236,893094	170,202464	295,2209693	203,547779	0,68947602	-0,536427719	0,1048744	0,1588154	conserved protein, unknown function
PFIT_1211100	283,77762	264,94011	223,2261847	192,4802735	274,3588665	207,8532291	0,75759618	-0,400499039	0,1049528	0,1588915	mitochondrial ribosomal protein S18 precursor, putative
PFIT_1345500	17067,916	12025,391	8464,372472	11016,82232	14546,65326	9740,597396	0,669610887	-0,57860511	0,105048	0,1589931	DNA/RNA-binding protein Alba 2 (ALBA2)
PFIT_1461400	106,66818	77,073487	144,6414564	117,6268338	91,8708354	131,1341451	1,427375124	0,513364535	0,1056533	0,1598664	conserved Plasmodium protein, unknown function
PFIT_1472800	19,119769	19,268372	31,88945496	37,42671984	19,19407029	34,6580874	1,805666379	0,852531361	0,1056914	0,1598813	conserved Plasmodium protein, unknown function
PFIT_1118800	268,68307	308,29395	231,1985484	207,6291839	288,4885077	219,4138662	0,760563628	-0,394859147	0,1063856	0,1608706	conserved Plasmodium protein, unknown function
PFIT_1359000	12781,062	13231,591	10958,58341	10007,192	13006,32659	10482,8877	0,805983736	-0,311177367	0,1064024	0,1608706	conserved Plasmodium membrane protein, unknown function
PFIT_0805100	3837,0357	2410,4733	1570,555657	2116,391896	3123,754505	1843,473776	0,590146816	-0,760854183	0,1065674	0,161077	rRNA processing WD-repeat protein, putative
PFIT_1230700	487,05095	497,12399	640,0669173	624,6697764	492,0874722	632,3683469	1,285073046	0,361850368	0,1068428	0,1614379	conserved Plasmodium protein, unknown function
PFIT_1131300	597,74435	765,91778	1100,186196	821,6056118	681,8310647	960,8959039	1,409287364	0,494965818	0,1068633	0,1614379	conserved Plasmodium protein, unknown function
PFIT_1221100	29,182805	18,304953	39,86181869	41,88228173	23,74387908	40,87205021	1,721372067	0,783558963	0,1071781	0,1618702	inner membrane complex protein 1h, putative (IMC1h)
PFIT_0404300	431,70425	428,72127	296,1163674	358,2271756	430,2127627	327,1717715	0,760488298	-0,395002047	0,10723	0,1618867	conserved Plasmodium membrane protein, unknown function
PFIT_bin00600	2,0126072	1,9268372	10,25018195	7,128899018	1,969722209	8,689540484	4,411556331	2,141287707	0,1072464	0,1618867	stevor
PFIT_0107300	426,67273	576,12432	413,4240053	321,6915682	501,3985259	367,5577867	0,733065152	-0,447986669	0,1073728	0,1620342	conserved Plasmodium protein, unknown function
PFIT_1446000	22183,963	22715,484	36175,16992	26066,81926	22449,7234	31120,99459	1,386252918	0,471190498	0,1076015	0,1623361	fructose-bisphosphate aldolase
PFIT_1214400	31,195412	21,195209	14,80581837	11,5844609	26,19531058	13,19513964	0,503721443	-0,989301949	0,1078611	0,1626842	conserved Plasmodium protein, unknown function
PFIT_1356300	383,40168	373,80641	506,814552	474,9628971	378,604046	490,8887245	1,296575485	0,3747062	0,1084124	0,1634721	glutamate--tRNA ligase, putative
PFIT_1203700	13,081947	26,012302	35,30618227	35,64449509	19,54712451	35,47533868	1,814862266	0,859860063	0,1090096	0,1643287	conserved Plasmodium protein, unknown function
PFIT_0400500	5351,5226	6569,5514	7790,138282	7184,147985	5960,537008	7487,143134	1,25611889	0,32897302	0,1090641	0,1643671	Plasmodium exported protein (PHISTb), unknown function
PFIT_1218200	17748,177	15941,687	15023,35001	12324,08418	16844,93217	13673,71709	0,811740704	-0,300909138	0,1091899	0,164469	conserved Plasmodium protein, unknown function
PFIT_0533400	50,315181	42,390418	67,19563723	73,96232731	46,35279946	70,57898227	1,522647674	0,606582154	0,1091833	0,164469	sugar transporter, putative
PFIT_1146200	121,76274	124,281	168,5585476	169,3113517	123,021868	168,9349496	1,373210733	0,457553039	0,1094633	0,1648369	transporter, putative
PFIT_1023000	8,0504289	17,341535	20,5003639	44,55561886	12,6959818	32,52799138	2,562069787	1,357309773	0,1097473	0,1652204	conserved Plasmodium protein, unknown function
PFIT_1106700	121,76274	97,305278	78,58472828	73,96232731	109,5340078	76,2735278	0,696345631	-0,522124529	0,1098503	0,1653315	conserved Plasmodium membrane protein, unknown function
PFIT_1010600	550,44808	486,52639	628,6778263	687,9387552	518,4872339	658,3082907	1,269671166	0,3444549	0,1100622	0,1656062	conserved Plasmodium protein, unknown function
PFIT_0403100	1314,2325	2124,338	1226,605107	874,181242	1719,28526	1050,393174	0,610947583	-0,710879487	0,110183	0,1657387	6-cysteine protein
PFIT_0113100	31,195412	34,683069	42,13963691	62,3778664	32,93924073	52,25875165	1,586519619	0,665865362	0,1102089	0,1657387	Plasmodium exported protein, unknown function, pseudogene
PFIT_1253400	276,73349	252,41567	187,9200024	207,6291839	264,574583	197,7745932	0,747519247	-0,419817368	0,1103159	0,1658554	Plasmodium exported protein, unknown function
PFIT_1459300	266,67046	202,3179	128,6967289	188,915824	234,4941815	158,8062764	0,67722907	-0,562284193	0,1103627	0,1658817	conserved Plasmodium protein, unknown function
PFIT_0910400	5791,2773	5669,7184	4377,966602	4762,104544	5730,497866	4570,035573	0,797493635	-0,326455091	0,1105167	0,166069	conserved Plasmodium protein, unknown function
PFIT_0932100	5,0315181	5,7805116	13,66690927	15,14891041	5,40601482	14,40790984	2,665162846	1,414223687	0,1108295	0,1664947	conserved Plasmodium protein, unknown function
PFIT_1250400	10898,268	11579,328	15779,58566	12586,07122	11238,79812	14182,82844	1,261952416	0,335657512	0,1114762	0,1674218	conserved Plasmodium membrane protein, unknown function
PFIT_1140500	2239,0255	2652,2914	2089,898209	1853,513745	2445,658466	1971,705977	0,806206592	-0,310778516	0,1116934	0,1677034	conserved Plasmodium protein, unknown function
PFIT_0919700	24,151287	11,561023	26,19490943	49,01118075	17,85615496	37,60304509	2,105887027	1,074428043	0,1119032	0,1679737	dolichyl-diphosphooligosaccharide-protein glycosyltransferase, putative
PFIT_1202300	6523,8664	5875,89	5020,311337	4932,307008	6199,878172	4976,309173	0,802646283	-0,317163746	0,1119571	0,1680101	conserved protein, unknown function
PFIT_1102100	13,081947	9,6341859	1,138909106	5,346674263	11,35806647	3,242791684	0,285505609	-1,808409007	0,1120254	0,1680596	RESA-like protein
PFIT_1253600	9,0567326	7,7073487	23,91709122	15,14891041	8,382040647	19,53300081	2,330339548	1,220540182	0,1120497	0,1680596	acyl-CoA binding protein, putative
PFIT_1235700	193,21029	228,33021	289,2829128	270,8981627	210,7702504	280,0905377	1,328890283	0,410221996	0,1125173	0,1687162	conserved Plasmodium protein, unknown function
PFIT_0518500	74,466468	58,768534	45,55636422	40,99116935	66,6175009	43,27376679	0,649585562	-0,622408527	0,1128255	0,1691335	cyclin
PFIT_1012800	575,60567	577,08774	444,1745512	454,4673124	576,3467029	449,3209318	0,779601808	-0,359190658	0,1131306	0,1695458	conserved Plasmodium protein, unknown function
PFIT_0525800	13,081947	19,268372	3,416727317	8,911123772	16,17515943	6,163925544	0,381073557	-1,391858592	0,1132322	0,1696532	conserved Plasmodium protein, unknown function
PFIT_1306900	6070,0234	6431,7825	8737,710658	7021,965532	6250,90297	7879,838095	1,260591971	0,334101379	0,1142299	0,1711027	conserved Plasmodium protein, unknown function
PFIT_1403700	4065,4666	4790,1172	4015,793506	3207,113446	4427,791927	3611,453476	0,815633059	-0,294007843	0,114649	0,1716848	conserved Plasmodium protein, unknown function
PFIT_0313200	57,359306	45,280674	25,05600032	36,53560747	51,31999001	30,79580389	0,600074238	-0,736787101	0,1155505	0,1729889	apicoplast conserved ycf19 protein precursor, unknown function
PFIT_1116200	11504,063	10674,678	9339,054665	8604,581114	11089,37048	8971,81789	0,809046637	-0,305705227	0,1158209	0,1733478	cysteine proteinase falcipain 3
PFIT_0831000	453,84293	549,1486	633,2334627	646,0564735	501,4957645	639,6449681	1,275474318	0,35103385	0,1158718	0,1733781	conserved Plasmodium protein, unknown function
PFIT_bin08600	20,126072	30,829395	1,138909106	16,93113517	25,47773365	9,035022136	0,354624248	-1,495636909	0,1163388	0,1740308	erythrocyte membrane protein 1 (PfEMP1), exon1, pseudogene
PFIT_1202000	724,5386	749,53966	939,6000121	924,0835352	737,0391346	931,8417736	1,264304336	0,338343782	0,1170314	0,1750205	dynein heavy chain, putative
PFIT_1408700	248,55699	438,35546	218,6705483	173,7669136	343,4562264	196,2187309	0,571306373	-0,807663472	0,1171091	0,1750903	conserved Plasmodium protein, unknown function
PFIT_0835300	3,0189109	1,9268372	5,694545528	18,71335992	2,472874018	12,20395272	4,93512918	2,303087848	0,1171401	0,1750904	Plasmodium exported protein (PHISTa-like), unknown function
PFIT_1452100	323,02346	511,57527	261,9490943	277,1359493	417,2993668	269,5425218	0,645921234	-0,630569848	0,1173912	0,1753828	acetyl-CoA acetyltransferase, putative
PFIT_1229600.2	181,13465	218,69602	274,4770944	256,6403646	199,9153358	265,5587295	1,328355969	0,409641808	0,1174185	0,1753828	conserved Plasmodium protein, unknown function
PFIT_1445900	111,6997	98,268696	138,9469109	149,7068794	104,984199	144,3268951	1,374748739	0,459167963	0,1174289	0,1753828	conserved Plasmodium protein, unknown function
PFIT_1145100	599,75696	705,22241	876,9600113	787,7433415	652,4896827	832,3516764	1,275654924	0,35123812	0,1175785	0,175524	conserved Plasmodium protein, unknown function
PFIT_1023100	54,340395	64,549046	86,55709202	88,22012534	59,4447205	87,38860868	1,470081917	0,555896548	0,1175856	0,175524	conserved Plasmodium protein, unknown function
PFIT_0602600	20,126072	24,085465	30,75054585	44,55561886	22,10576857	37,65308236	1,703314781	0,768345077	0,1178486	0,1758702	phenylalanyl-tRNA synthetase subunit, putative
PFIT_1249500	1792,2267	1620,4701	1269,883653	1434,690927	1706,348407	1352,28729	0,79250362	-0,335510569	0,1179407	0,1759612	protein phosphatase, putative
PFIT_0913500	85,535807	107,90288	77,44581918	58,8134169	96,71934489	68,12961804	0,704405288	-0,505522354	0,1180587	0,1760908	conserved Plasmodium protein, unknown function
PFIT_0311400	940,89388	623,33183	466,9527333	560,5096853	782,1128557	513,7312093	0,656850486	-0,606363077	0,1181941	0,1762461	conserved Plasmodium protein, unknown function
PFIT_1310300	244,53178	341,05018	233,4763666	196,9358354	292,7909803	215,206101	0,735016156	-0,444152133	0,118664	0,1769002	conserved Plasmodium protein, unknown function
PFIT_1345600	20156,261	17968,72	13825,21763	16596,07691	19062,49081	15210,64727	0,797935979	-0,325655095	0,1191903	0,1776379	conserved Plasmodium protein, unknown function
PFIT_0627900	36,22693	37,573325	63,77890991	53,46674263	36,90012766	58,62282627	1,588688983	0,667836716	0,1192913	0,1777415	conserved protein, unknown function
PFIT_0621000	964,03887	1009,6627	888,3491023	702,1965532	986,850775	795,2728278	0,805869386	-0,311382067	0,1193575	0,1777933	conserved Plasmodium protein, unknown function
PFIT_1009400	1425,9322	1289,0541	1122,964378	1049,73038	1357,493151	1086,347379	0,800259934	-0,321459414	0,119393	0,1777992	conserved Plasmodium protein, unknown function
PFIT_1145800	5933,1661	5678,3892	4709,389151	4655,171059	5805,777655	4682,280105	0,806486294	-0,310278079	0,1197909	0,1783447	coatamer gamma subunit, putative
PFIT_0204300	95,598844	91,524766	108,196365	147,9246546	93,56180495	128,0605098	1,368726372	0,45283406	0,1199602	0,1785499	conserved Plasmodium protein, unknown function
PFIT_0415900	2641,547	3996,2603	2573,934579	1758,16472	3318,903658	2166,049649	0,65264011	-0,615640439	0,1203899	0,1791421	LETM1-like protein, putative
PFIT_1350600	11,06934	5,7805116	1,138909106	2,673337132	8,424925672	1,906123119	0,226248064	-2,144022651	0,1206658	0,1794583	small nucleolar RNA snoR21
PFIT_0701900	377,36386	309,25737	397,4792778	474,0717847	343,3106123	435,7755313	1,269333122	0,344070738	0,1206396	0,1794583	conserved Plasmodium protein, unknown function
PFIT_1033200	12,075643	9,6341859	17,08363658	26,73337132	10,85491466	21,90850395	2,018302734	1,013142587	0,1207911	0,1795973	plasmepsin VII
PFIT_1315900	98,617755	103,08579	205,003639	126,5379576	100,8517719	165,7707983	1,643707345	0,716953456	0,1211199	0,1800388	conserved Plasmodium protein, unknown function
PFIT_1037900	747,68359	1483,6646	668,539645	429,5161658	1115,67411	549,0279054	0,492104191	-1,022964291	0,1211829	0,1800851	Plasmodium exported protein, unknown function
PFIT_1104900	1698,6405	1438,384	1209,52147	1286,766273	1568,512232	1248,143871	0,795750168	-0,329612539	0,1213721	0,1802019	syntaxin, Qa-SNARE family
PFIT_1323900	1300,1443	1407,5546	1133,21456	1042,601481	1353,849418	1087,908021	0,803566487	-0,315510698	0,1213631	0,1802019	SNARE protein, putative
PFIT_0812800	4929,8814	4444,25	3895,069141	3682,076343	4687,065693	3788,572742	0,808303743	-0,307030567	0,1213891	0,1802019	JmjC domain containing protein
PFIT_0409700	43,271056	38,536744	70,61236454	57,03119214	40,90389962	63,82177834	1,560285913	0,641810418	0,1213817	0,1802019	conserved Plasmodium protein, unknown function
PFIT_0102200	553,46699	852,62545	539,842916	356,4449509	703,0462218	448,1439335	0,637431679	-0,649657375	0,1215534	0,1802564	L-seryl-tRNA(Sec) kinase, putative (PSTK)
PFIT_1201600	654,09735	559,7462	707,2625545	811,8033756	606,9217766	759,5329651	1,251451166	0,323601995	0,1215433	0,1802564	conserved protein, unknown function
PFIT_1313200	78,491682	58,768534	89,97381934	105,1512605	68,63010814	97,56253992	1,421570541	0,50748569	0,1215015	0,1802564	conserved Plasmodium protein, unknown function
PFIT_1218400	19,119769	23,122046	27,3381853	44,55561886	21,12090747	35,9447187	1,701854844	0,767107991	0,121527	0,1802564	conserved Plasmodium protein, unknown function
PFIT_1465700	21325,586	18269,307	25537,75887	23549,42679	19797,44651	24543,59283	1,239735277	0,310032092	0,1215914	0,1802653	phosphatase, putative
PFIT_1104200	429,69164	484,59955	580,8436438	583,6786071	457,1455982	582,2611255	1,273688575	0,349012572	0,122236	0,1811736	iron-sulfur assembly protein, sufD, putative
PFIT_0315200	459,88075	348,75753	310,9221858	314,5626692	404,3191417	312,7424275	0,773503886	-0,370519554	0,1232284	0,1825964	circumsporozoite- and TRAP-related protein
PFIT_1118100	881,52197	812,16187	715,2349183	637,1453497	846,841921	676,190134	0,798484484	-0,324663722	0,1233423	0,1827173	conserved Plasmodium protein, unknown function
PFIT_0821200	207,29855	208,09842	297,2552765	253,0759151	207,6984805	275,1655958	1,324832012	0,405809438	0,1234057	0,1827634	dynactin subunit 6, putative
PFIT_1364200	43,271056	17,341535	7,972363739	11,5844609	30,3062951	9,778412321	0,322652845	-1,631945351	0,1237368	0,1832056	conserved Plasmodium protein, unknown function
PFIT_1349800	135,85099	98,268696	150,3360019	165,7469022	117,0598424	158,041452	1,350091106	0,433056766	0,1242515	0,1839195	conserved Plasmodium protein, unknown function
PFIT_0525100	299,87848	339,12334	220,9483665	265,5514884	319,5009112	243,2499274	0,761343454	-0,393380671	0,1243627	0,1840359	zinc finger protein, putative
PFIT_0928500	141,88881	140,65911	99,08509218	105,1512605	141,2739622	102,1181763	0,722837915	-0,468255913	0,1244325	0,184091	conserved Plasmodium protein, unknown function
PFIT_0604500	341,13693	323,70865	251,6989123	259,3137018	332,4227866	255,506307	0,76861851	-0,379660374	0,1245175	0,1841686	nucleoside diphosphate kinase, putative
PFIT_0618000	299,87848	297,69634	201,5869117	250,402578	298,7874115	225,9947448	0,756373047	-0,40283014	0,124812	0,1845558	conserved Plasmodium protein, unknown function
PFIT_0620400	234,46874	225,43995	152,6138201	189,8069363	229,9543467	171,2103782	0,744540735	-0,425577312	0,1248887	0,1846208	conserved Plasmodium protein, unknown function
PFIT_0835200.1	58,36561	75,14665	43,27854601	44,55561886	66,75612999	43,91708244	0,657873403	-0,604118107	0,1250102	0,1847523	Plasmodium exported protein (PHISTa-like), unknown function
PFIT_0916300	317,99194	234,11072	320,0334587	406,347244	276,0513305	363,1903513	1,315662383	0,395789321	0,1253075	0,1851432	conserved Plasmodium protein, unknown function
PFIT_1419800	45262,53	46082,238	43025,70819	29761,37117	45672,38425	36393,53968	0,796839059	-0,327639729	0,1257052	0,1856823	signal recognition particle RNA
PFIT_0601800	1652,3505	1663,8239	2120,648755	2013,913972	1658,087224	2067,281364	1,24678686	0,318214855	0,1257447	0,1856921	conserved Plasmodium protein, unknown function
PFIT_0713200	17,107161	18,304953	30,75054585	32,97115796	17,70605737	31,8608519	1,79943232	0,847541841	0,1259341	0,1859231	conserved Plasmodium protein, unknown function
PFIT_1354100	933,84976	825,64973	701,568009	696,849879	879,7497452	699,208944	0,794781639	-0,331369552	0,1261759	0,1862314	conserved Plasmodium protein, unknown function
PFIT_0834700	735,60794	1482,7012	658,289463	434,8628401	1109,154579	546,5761515	0,492786273	-1,020966025	0,1276915	0,18837	Plasmodium exported protein (PHISTa), unknown function, pseudogene
PFIT_1211900	367,30082	453,77016	506,814552	536,4496511	410,5354886	521,6321015	1,270613908	0,345525716	0,1276649	0,18837	conserved Plasmodium protein, unknown function
PFIT_1364700	3462,6907	3377,7456	4383,661147	4100,008048	3420,218166	4241,834597	1,240223399	0,310600013	0,1282262	0,1891094	conserved Plasmodium membrane protein, unknown function
PFIT_0809000	564,53633	579,01457	457,8414604	445,5561886	571,7754516	451,6988245	0,789993385	-0,340087522	0,128464	0,1894107	conserved Plasmodium protein, unknown function
PFIT_1366000	1009,3225	1377,6886	1007,934558	697,7409914	1193,505557	852,8377749	0,714565399	-0,484862038	0,1287338	0,1897589	suppressor of kinetochore protein 1, putative
PFIT_1230800	2018,6451	2319,912	3044,304039	2422,043441	2169,278513	2733,17374	1,259945979	0,333361879	0,1288483	0,1898781	Rab GTPase 2
PFIT_1237000	39,245841	22,158628	53,52872796	45,44673124	30,70223435	49,4877296	1,611860851	0,688727204	0,1291831	0,1903219	conserved Plasmodium protein, unknown function
PFIT_0109700	4,0252145	0,9634186	9,111272844	8,911123772	2,494316531	9,011198308	3,612692374	1,853074412	0,129247	0,1903664	conserved Plasmodium protein, unknown function
PFIT_0623400	137,8636	172,45193	176,5309114	245,9470161	155,1577618	211,2389637	1,36144632	0,4451401	0,1293454	0,1904616	anaphase promoting complex subunit, putative
PFIT_0512600	732,58903	752,42992	569,4545528	605,9564165	742,5094769	587,7054846	0,791512436	-0,337316078	0,129529	0,1906824	conserved Plasmodium protein, unknown function
PFIT_1002400	79,497986	88,63451	133,2523653	105,1512605	84,06624812	119,2018129	1,417950909	0,503807586	0,1305283	0,1921033	DnaJ protein, putative
PFIT_0612100	328,05498	203,28132	342,8116408	378,7227603	265,6681511	360,7672005	1,3579618	0,441442896	0,1307907	0,1924394	conserved Plasmodium protein, unknown function
PFIT_1103300	144,90772	122,35416	176,5309114	180,8958126	133,630941	178,713362	1,337365138	0,419393416	0,1310722	0,1928034	Plasmodium exported protein (PHISTb), unknown function
PFIT_1444300	932,84345	677,28327	933,9054665	1223,497294	805,0633618	1078,70138	1,339896251	0,422121296	0,1312615	0,1930317	conserved Plasmodium protein, unknown function
PFIT_0305600	1380,6486	1212,944	1006,795649	1065,770403	1296,796285	1036,283026	0,799110113	-0,323533782	0,1313771	0,1931513	ubiquitin conjugating enzyme E2, putative
PFIT_0934000	789,94834	930,66236	1055,768741	1091,612662	860,3053498	1073,690701	1,248034435	0,319657741	0,1314826	0,1932562	gamma-tubulin complex component, putative
PFIT_0911700.1	13,081947	10,597605	21,63927301	24,95114656	11,83977577	23,29520978	1,967538088	0,976391564	0,1316807	0,1934969	conserved Plasmodium protein, unknown function
PFIT_1400200	49,308877	40,463581	55,80654617	105,1512605	44,88622906	80,47890334	1,792953096	0,842337747	0,1319833	0,1938912	erythrocyte membrane protein 1, PfEMP1
PFIT_0316700	1042,5305	1070,3581	841,653829	850,1212079	1056,444302	845,8875184	0,800692963	-0,320678968	0,1321168	0,1940369	HVA22/TB2/DP1 family protein, putative
PFIT_1137500	1856,6302	2152,2771	1772,142568	1502,415468	2004,453654	1637,279018	0,816820591	-0,29190886	0,132292	0,1942426	DnaJ protein, putative
PFIT_1227400	4563,5869	3963,5041	5138,757884	5325,287566	4263,545496	5232,022725	1,227153018	0,295315155	0,1323256	0,1942426	conserved Plasmodium protein, unknown function
PFIT_1474800	275,72719	232,18388	150,3360019	220,9958695	253,9555359	185,6659357	0,731096233	-0,451866776	0,1324671	0,1943998	conserved Plasmodium protein, unknown function
PFIT_0628900	3,0189109	0,9634186	9,111272844	7,128899018	1,991164722	8,120085931	4,078058355	2,02788242	0,1327521	0,1947675	CPW-WPC family protein
PFIT_1460300	273,71458	269,75721	233,4763666	188,915824	271,7358949	211,1960953	0,777210885	-0,363621989	0,1331671	0,1953256	GTP-binding protein, putative
PFIT_1022800	18,113465	13,48786	20,5003639	40,10005697	15,8006627	30,30021044	1,917654405	0,939342745	0,1339054	0,1963575	conserved Plasmodium protein, unknown function
PFIT_0935500	1508,4491	2217,7896	1510,193474	984,6791768	1863,119361	1247,436325	0,669541819	-0,578753927	0,1341246	0,1966278	small nuclear ribonucleoprotein (snRNP), putative
PFIT_0821100	31685,482	34169,567	28985,23674	25199,76692	32927,5246	27092,50183	0,822791939	-0,281400435	0,1347225	0,1974532	14-3-3 protein (14-3-3I)
PFIT_bin01800	31,195412	17,341535	7,972363739	15,14891041	24,2684734	11,56063708	0,476364413	-1,069862456	0,1357625	0,1989259	rifin
PFIT_0933900	26,163894	24,085465	45,55636422	38,31783222	25,12467943	41,93709822	1,669159535	0,739121851	0,1359941	0,1992135	conserved Plasmodium protein, unknown function
PFIT_1206500	146,92033	148,36646	214,1149118	181,786925	147,6433957	197,9509184	1,340736695	0,423025937	0,1361009	0,1993183	conserved Plasmodium protein, unknown function
PFIT_0502000	163,02119	161,85432	103,6407286	133,6668566	162,4377547	118,6537926	0,730456985	-0,453128776	0,1361595	0,1993191	conserved protein, unknown function
PFIT_1470500	278,7461	259,1596	207,2814572	205,8469591	268,9528516	206,5642082	0,7680313	-0,380762989	0,136172	0,1993191	nucleoside transporter 3, putative (NT3)
PFIT_1202400	2550,9797	4084,8948	2578,490215	1397,264207	3317,93725	1987,877211	0,599130442	-0,739057956	0,1365559	0,1998293	conserved Plasmodium protein, unknown function
[truncated: 293,028 more chars]
